# Annotated and illustrated world checklist of Microgastrinae parasitoid wasps (Hymenoptera, Braconidae)

**DOI:** 10.3897/zookeys.920.39128

**Published:** 2020-03-23

**Authors:** Jose Fernandez-Triana, Mark R. Shaw, Caroline Boudreault, Melanie Beaudin, Gavin R. Broad

**Affiliations:** 1 Canadian National Collection of Insects, Ottawa, Canada Canadian National Collection of Insects Ottawa Canada; 2 National Museums of Scotland, Edinburgh, UK National Museums of Scotland Edinburgh United Kingdom; 3 Department of Biology, Carleton University, Ottawa, Canada Carleton University Ottawa Canada; 4 Natural History Museum, London, UK Natural History Museum London United Kingdom

**Keywords:** Microgastrinae, world fauna, checklist, nomenclature changes, genus diagnosis, genus illustration, distribution, Lepidoptera

## Abstract

A checklist of world species of Microgastrinae parasitoid wasps (Hymenoptera: Braconidae) is provided. A total of 81 genera and 2,999 extant species are recognized as valid, including 36 nominal species that are currently considered as *species inquirendae*. Two genera are synonymized under *Apanteles*. Nine lectotypes are designated. A total of 318 new combinations, three new replacement names, three species name amendments, and seven species status revised are proposed. Additionally, three species names are treated as *nomina dubia*, and 52 species names are considered as unavailable names (including 14 as *nomina nuda*). A total of three extinct genera and 12 extinct species are also listed. Unlike in many previous treatments of the subfamily, tribal concepts are judged to be inadequate, so genera are listed alphabetically. Brief diagnoses of all Microgastrinae genera, as understood in this paper, are presented. Illustrations of all extant genera (at least one species per genus, usually more) are included to showcase morphological diversity. Primary types of Microgastrinae are deposited in 108 institutions worldwide, although 76% are concentrated in 17 collections. Localities of primary types, in 138 countries, are reported. Recorded species distributions are listed by biogeographical region and by country. Microgastrine wasps are recorded from all continents except Antarctica; specimens can be found in all major terrestrial ecosystems, from 82°N to 55°S, and from sea level up to at least 4,500 m a.s.l. The Oriental (46) and Neotropical (43) regions have the largest number of genera recorded, whereas the Palaearctic region (28) is the least diverse. Currently, the highest species richness is in the Palearctic region (827), due to more historical study there, followed by the Neotropical (768) and Oriental (752) regions, which are expected to be the most species rich. Based on ratios of Lepidoptera and Microgastrinae species from several areas, the actual world diversity of Microgastrinae is expected to be between 30,000–50,000 species; although these ratios were mostly based on data from temperate areas and thus must be treated with caution, the single tropical area included had a similar ratio to the temperate ones. Almost 45,000 specimens of Microgastrinae from 67 different genera (83% of microgastrine genera) have complete or partial DNA barcode sequences deposited in the Barcode of Life Data System; the DNA barcodes represent 3,545 putative species or Barcode Index Numbers (BINs), as estimated from the molecular data. Information on the number of sequences and BINs per genus are detailed in the checklist. Microgastrinae hosts are here considered to be restricted to Eulepidoptera, i.e., most of the Lepidoptera except for the four most basal superfamilies (Micropterigoidea, Eriocranioidea, Hepialoidea and Nepticuloidea), with all previous literature records of other insect orders and those primitive Lepidoptera lineages being considered incorrect. The following nomenclatural acts are proposed: 1) Two genera are synonymyzed under *Apanteles*: *Cecidobracon* Kieffer & Jörgensen, 1910, **new synonym** and *Holcapanteles* Cameron, 1905, **new synonym**; 2) Nine **lectotype designations** are made for *Alphomelondisputabile* (Ashmead, 1900), *Alphomelonnigriceps* (Ashmead, 1900), *Cotesiasalebrosa* (Marshall, 1885), *Diolcogasterxanthaspis* (Ashmead, 1900), *Dolichogenideaononidis* (Marshall, 1889), *Glyptapantelesacraeae* (Wilkinson, 1932), *Glyptapantelesguyanensis* (Cameron, 1911), *Glyptapantelesmilitaris* (Walsh, 1861), and *Pseudapantelesannulicornis* Ashmead, 1900; 3) Three **new replacement names** are a) *Diolcogasteraurangabadensis* Fernandez-Triana, replacing *Diolcogasterindicus* (Rao & Chalikwar, 1970) [nec *Diolcogasterindicus* (Wilkinson, 1927)], b) *Dolichogenideaincystatae* Fernandez-Triana, replacing *Dolichogenidealobesia* Liu & Chen, 2019 [nec *Dolichogenidealobesia* Fagan-Jeffries & Austin, 2019], and c) *Microplitisvitobiasi* Fernandez-Triana, replacing *Microplitisvariicolor* Tobias, 1964 [nec *Microplitisvaricolor* Viereck, 1917]; 4) Three **names amended** are *Apantelesirenecarrilloae* Fernandez-Triana, 2014, *Cotesiaayerzai* (Brèthes, 1920), and *Cotesiariverai* (Porter, 1916); 5) Seven species have their **status revised**: *Cotesiaarctica* (Thomson, 1895), *Cotesiaokamotoi* (Watanabe, 1921), *Cotesiaukrainica* (Tobias, 1986), *Dolichogenideaappellator* (Telenga, 1949), *Dolichogenideamurinanae* (Capek & Zwölfer, 1957), *Hypomicrogasteracarnas* Nixon, 1965, and *Nyererianigricoxis* (Wilkinson, 1932); 6) **New combinations** are given for 318 species: *Alloplitiscongensis*, *Alloplitisdetractus*, *Apantelesasphondyliae*, *Apantelesbraziliensis*, *Apantelessulciscutis*, *Choerasaper*, *Choerasapollion*, *Choerasdaphne*, *Choerasfomes*, *Choerasgerontius*, *Choerashelle*, *Choerasirates*, *Choeraslibanius*, *Choeraslongiterebrus*, *Choerasloretta*, *Choerasrecusans*, *Choerassordidus*, *Choerasstenoterga*, *Choerassuperbus*, *Choerassylleptae*, *Choerasvacillatrix*, *Choerasvacillatropsis*, *Choerasvenilia*, *Cotesiaasavari*, *Cotesiabactriana*, *Cotesiabambeytripla*, *Cotesiaberberidis*, *Cotesiabhairavi*, *Cotesiabiezankoi*, *Cotesiabifida*, *Cotesiacaligophagus*, *Cotesiacheesmanae*, *Cotesiacompressithorax*, *Cotesiadelphinensis*, *Cotesiaeffrena*, *Cotesiaeuphobetri*, *Cotesiaelaeodes*, *Cotesiaendii*, *Cotesiaeuthaliae*, *Cotesiaexelastisae*, *Cotesiahiberniae*, *Cotesiahyperion*, *Cotesiahypopygialis*, *Cotesiahypsipylae*, *Cotesiajujubae*, *Cotesialesbiae*, *Cotesialevigaster*, *Cotesializeri*, *Cotesiamalevola*, *Cotesiamalshri*, *Cotesiamenezesi*, *Cotesiamuzaffarensis*, *Cotesianeptisis*, *Cotesianycteus*, *Cotesiaoeceticola*, *Cotesiaoppidicola*, *Cotesiaopsiphanis*, *Cotesiapachkuriae*, *Cotesiapaludicolae*, *Cotesiaparbhanii*, *Cotesiaparvicornis*, *Cotesiapratapae*, *Cotesiaprozorovi*, *Cotesiapterophoriphagus*, *Cotesiaradiarytensis*, *Cotesiarangii*, *Cotesiariverai*, *Cotesiaruficoxis*, *Cotesiasenegalensis*, *Cotesiaseyali*, *Cotesiasphenarchi*, *Cotesiasphingivora*, *Cotesiatransuta*, *Cotesiaturkestanica*, *Diolcogasterabengouroui*, *Diolcogasteragama*, *Diolcogasterambositrensis*, *Diolcogasteranandra*, *Diolcogasterannulata*, *Diolcogasterbambeyi*, *Diolcogasterbicolorina*, *Diolcogastercariniger*, *Diolcogastercincticornis*, *Diolcogastercingulata*, *Diolcogastercoronata*, *Diolcogastercoxalis*, *Diolcogasterdipika*, *Diolcogasterearina*, *Diolcogasterepectina*, *Diolcogasterepectinopsis*, *Diolcogastergrangeri*, *Diolcogasterheterocera*, *Diolcogasterhomocera*, *Diolcogasterindica*, *Diolcogasterinsularis*, *Diolcogasterkivuana*, *Diolcogastermediosulcata*, *Diolcogastermegaulax*, *Diolcogasterneglecta*, *Diolcogasternigromacula*, *Diolcogasterpalpicolor*, *Diolcogasterpersimilis*, *Diolcogasterplecopterae*, *Diolcogasterplutocongoensis*, *Diolcogasterpsilocnema*, *Diolcogasterrufithorax*, *Diolcogastersemirufa*, *Diolcogasterseyrigi*, *Diolcogastersubtorquata*, *Diolcogastersulcata*, *Diolcogastertorquatiger*, *Diolcogastertristiculus*, *Diolcogasterturneri*, *Diolcogastervulcana*, *Diolcogasterwittei*, *Distatrixanthedon*, *Distatrixcerales*, *Distatrixcuspidalis*, *Distatrixeuproctidis*, *Distatrixflava*, *Distatrixgeometrivora*, *Distatrixmaia*, *Distatrixtookei*, *Distatrixtermina*, *Distatrixsimulissima*, *Dolichogenideaagamedes*, *Dolichogenideaaluella*, *Dolichogenideaargiope*, *Dolichogenideaatreus*, *Dolichogenideabakeri*, *Dolichogenideabasiflava*, *Dolichogenideabersa*, *Dolichogenideabiplagae*, *Dolichogenideabisulcata*, *Dolichogenideacatonix*, *Dolichogenideachrysis*, *Dolichogenideacoffea*, *Dolichogenideacoretas*, *Dolichogenideacyane*, *Dolichogenideadiaphantus*, *Dolichogenideadiparopsidis*, *Dolichogenideadryas*, *Dolichogenideaearterus*, *Dolichogenideaensiger*, *Dolichogenideaeros*, *Dolichogenideaevadne*, *Dolichogenideafalcator*, *Dolichogenideagelechiidivoris*, *Dolichogenideagobica*, *Dolichogenideahyalinis*, *Dolichogenideairiarte*, *Dolichogenidealakhaensis*, *Dolichogenidealampe*, *Dolichogenidealaspeyresiella*, *Dolichogenidealatistigma*, *Dolichogenidealebene*, *Dolichogenidealucidinervis*, *Dolichogenideamalacosomae*, *Dolichogenideamaro*, *Dolichogenideamendosae*, *Dolichogenideamonticola*, *Dolichogenideanigra*, *Dolichogenideaolivierellae*, *Dolichogenideaparallelis*, *Dolichogenideapelopea*, *Dolichogenideapelops*, *Dolichogenideaphaenna*, *Dolichogenideapisenor*, *Dolichogenidearoepkei*, *Dolichogenideascabra*, *Dolichogenideastatius*, *Dolichogenideastenotelas*, *Dolichogenideastriata*, *Dolichogenideawittei*, *Exoryzaasotae*, *Exoryzabelippicola*, *Exoryzahylas*, *Exoryzamegagaster*, *Exoryzaoryzae*, *Glyptapantelesaggestus*, *Glyptapantelesagynus*, *Glyptapantelesaithos*, *Glyptapantelesamenophis*, *Glyptapantelesantarctiae*, *Glyptapantelesanubis*, *Glyptapantelesarginae*, *Glyptapantelesargus*, *Glyptapantelesatylana*, *Glyptapantelesbadgleyi*, *Glyptapantelesbataviensis*, *Glyptapantelesbistonis*, *Glyptapantelesborocerae*, *Glyptapantelescacao*, *Glyptapantelescadei*, *Glyptapantelescinyras*, *Glyptapanteleseryphanidis*, *Glyptapanteleseuproctisiphagus*, *Glyptapanteleseutelus*, *Glyptapantelesfabiae*, *Glyptapantelesfulvigaster*, *Glyptapantelesfuscinervis*, *Glyptapantelesgahinga*, *Glyptapantelesglobatus*, *Glyptapantelesglyphodes*, *Glyptapantelesguierae*, *Glyptapanteleshorus*, *Glyptapantelesintricatus*, *Glyptapanteleslamprosemae*, *Glyptapanteleslefevrei*, *Glyptapantelesleucotretae*, *Glyptapanteleslissopleurus*, *Glyptapantelesmadecassus*, *Glyptapantelesmarquesi*, *Glyptapantelesmelanotus*, *Glyptapantelesmelissus*, *Glyptapantelesmerope*, *Glyptapantelesnaromae*, *Glyptapantelesnepitae*, *Glyptapantelesnigrescens*, *Glyptapantelesninus*, *Glyptapantelesnkuli*, *Glyptapantelesparasundanus*, *Glyptapantelespenelope*, *Glyptapantelespenthocratus*, *Glyptapantelesphilippinensis*, *Glyptapantelesphilocampus*, *Glyptapantelesphoebe*, *Glyptapantelesphytometraduplus*, *Glyptapantelespropylae*, *Glyptapantelespuera*, *Glyptapantelesseydeli*, *Glyptapantelessiderion*, *Glyptapantelessimus*, *Glyptapantelesspeciosissimus*, *Glyptapantelesspilosomae*, *Glyptapantelessubpunctatus*, *Glyptapantelesthespis*, *Glyptapantelesthoseae*, *Glyptapantelesvenustus*, *Glyptapanteleswilkinsoni*, *Hypomicrogastersamarshalli*, *Iconellacajani*, *Iconelladetrectans*, *Iconellajason*, *Iconellalynceus*, *Iconellapyrene*, *Iconellatedanius*, *Illidopsazamgarhensis*, *Illidopslamprosemae*, *Illidopstrabea*, *Keylimepiestriatus*, *Microplitisadisurae*, *Microplitismexicanus*, *Neoclarkinellaariadne*, *Neoclarkinellacurvinervus*, *Neoclarkinellasundana*, *Nyereriaituriensis*, *Nyererianioro*, *Nyereriaproagynus*, *Nyereriataoi*, *Nyereriavallatae*, *Parapantelesaethiopicus*, *Parapantelesalternatus*, *Parapantelesaso*, *Parapantelesatellae*, *Parapantelesbagicha*, *Parapantelescleo*, *Parapantelescyclorhaphus*, *Parapantelesdemades*, *Parapantelesendymion*, *Parapantelesepiplemicidus*, *Parapantelesexpulsus*, *Parapantelesfallax*, *Parapantelesfolia*, *Parapantelesfurax*, *Parapanteleshemitheae*, *Parapanteleshyposidrae*, *Parapantelesindicus*, *Parapantelesjavensis*, *Parapantelesjhaverii*, *Parapantelesmaculipalpis*, *Parapantelesmaynei*, *Parapantelesneocajani*, *Parapantelesneohyblaeae*, *Parapantelesnydia*, *Parapantelesprosper*, *Parapantelesprosymna*, *Parapantelespunctatissimus*, *Parapantelesregalis*, *Parapantelessarpedon*, *Parapantelessartamus*, *Parapantelesscultena*, *Parapantelestransvaalensis*, *Parapantelesturri*, *Parapantelesxanthopholis*, *Pholetesoracutus*, *Pholetesorbrevivalvatus*, *Pholetesorextentus*, *Pholetesoringenuoides*, *Pholetesorkuwayamai*, *Promicrogasterapidanus*, *Promicrogasterbriareus*, *Promicrogasterconopiae*, *Promicrogasteremesa*, *Promicrogastergrandicula*, *Promicrogasterorsedice*, *Promicrogasterrepleta*, *Promicrogastertyphon*, *Sathonbekilyensis*, *Sathonflavofacialis*, *Sathonlaurae*, *Sathonmikeno*, *Sathonruandanus*, *Sathonrufotestaceus*, *Venanidesastydamia*, *Venanidesdemeter*, *Venanidesparmula*, and *Venanidessymmysta*.

## Introduction

With almost 3,000 described species and estimates of up to 46,000+ worldwide (Rodriguez et al. 2013), the parasitoid wasp subfamily Microgastrinae (Hymenoptera: Ichneumonoidea, Braconidae) is an important and hyperdiverse group, which has long played a central role in our understanding of insect parasitism in the context of many areas of ecological, agricultural, and basic science (Whitfield et al. 2018). Because of their diversity, prevalence in most terrestrial habitats, and the fact that species are exclusively parasitoids of larval Lepidoptera across nearly the full range of families within the taxon (Eulepidoptera, *sensu*Aarvik et al. 2017), microgastrine wasps are one of the most important groups in the biological control of agricultural and forestry lepidopterous pests worldwide (Whitfield 1997).

A world checklist of Microgastrinae has never been published, although Shenefelt (1972, 1973) listed the species as part of his monumental work cataloguing the world species of Braconidae. Unfortunately, those papers are outdated, especially since Mason (1981) published a seminal study that changed the generic and tribal classifications. In addition to taxonomic changes (many nominal species had been placed in synonymy), the number of newly described species has increased dramatically since Shenefelt’s catalogue: 1,446 new species of Microgastrinae (48.2%) were described between 1974 and 2019. In the past six years alone (2014–2019), 720 new species have been described (an average of 120 new species/year), which represents, by far, the largest increase in species for any subfamily of Braconidae in that time span (data extracted from this paper and Yu et al. 2016).

The database Taxapad, originally produced as a CD (Yu et al. 2005), and later available as a USB drive (Yu et al. 2012, 2016) or, partially, as a web product (now offline), has been used as the de facto catalogue of Ichneumonoidea (and associated data comprising some 350,000 names) for almost fifteen years. It is important to understand that it is essentially a compilation of all published information, whether correct or not. Nevertheless, Taxapad is an extraordinary product that contains copious information about the taxonomy, distribution, hosts and associated host plants, morphology, etc., of Ichneumonoidea that is easy to collate and analyze. As a result, it is widely consulted by researchers worldwide, and it has been adopted and (unfortunately uncritically) used in many other databases, websites, and publications pertinent to Ichneumonoidea.

However, for Microgastrinae, Taxapad follows a classification based on van Achterberg (2003), which is far from being universally accepted. A different classification, based on an older, more comprehensive paper (Mason 1981), is the one preferred and used by most researchers worldwide (e.g., Papp 1988, Kotenko 2007a, Shaw 2012, Broad et al. 2016 in the Palearctic; Whitfield 1995a, Fernandez-Triana 2010 in the Nearctic; Whitfield 1997, Fernandez-Triana et al. 2014e in the Neotropical region; Rousse and Gupta 2013 in the Afrotropical region; Chen and Song 2004, Liu et al. 2017, 2018 in the Oriental region; Austin and Dangerfield 1992 in Australasia). Thus, the Microgastrinae arrangement in Taxapad conflicts with that used by most taxonomists working on the subfamily, a situation that becomes even more confusing for ecologists, biocontrol researchers and other non-taxonomist users of Taxapad.

To complicate matters further, neither Mason (1981) nor van Achterberg (2003) treated all world species, having left many nominal species without checking their generic placement, especially those described in older literature. As a result, many of those species have remained where they were originally described or as Nixon (1965) interpreted them, usually in one of the three traditional genera historically considered to constitute practically all Microgastrinae: *Apanteles* Foerster, *Microgaster* Latreille, and *Microplitis* Foerster; or they were placed as part of an expanded *Apanteles* and *Protapanteles* Ashmead (*sensu*van Achterberg 2003). Some exceptions fared slightly better, e.g., Papp (1988) assigned many European species to Mason’s (1981) genera, Whitfield (1995a) did the same for North America, and Austin and Dangerfield (1992) for Australasia.

In this paper we **a)** summarize general information about Microgastrinae, including a historical outline of the internal classification, estimates of specific and generic diversity, distribution at local and world levels, advances in regional taxonomic studies, and general trends in host use; **b)** characterize all 81 currently accepted genera of extant Microgastrinae, including brief morphological diagnostic features, colour illustrations, available DNA barcodes and general comments on known host families; **c)** revise, to the best of our knowledge, the generic placement of all described species of Microgastrinae; **d)** compile an updated checklist of the extant and fossil world species of Microgastrinae, including recorded geographical distribution and taxonomic notes; and **e)** provide all information as a supplementary Excel file, to facilitate future use of the data. As work on Microgastrinae advances, we hope to provide updates in future versions of this checklist.

## Materials and methods

We used the last two versions of Taxapad (Yu et al. 2012, 2016) as the starting point to compile a list of world genera and species of Microgastrinae and their recorded geographical distribution. Because the last version of Taxapad includes only information published up to the end of 2015, with some data from early 2016 (Yu, pers. comm.), we checked Zoological Record and Google Scholar for all papers published after 2015. The information presented in this paper has the cut off date of 31 December 2019.

We also compiled information from some of the world’s largest collections of Microgastrinae. All primary types (representing almost 500 species) of the Canadian National Collection of Insects (Ottawa, Canada) were studied, and unpublished information on the distribution of many species and genera was extracted from that collection, probably the largest depository of world Microgastrinae, with 120,000+ pinned specimens. We examined all primary types (representing almost 500 species of Microgastrinae) in The Natural History Museum (London, United Kingdom). Most of the primary types (representing almost 400 species of Microgastrinae) in the National Museum of Natural History (Washington, United States) were either examined or studied from images (available at http://www.usnmhymtypes.com/). Types and non-type material were extensively studied in the Finnish Museum of Natural History (Helsinki, Finland), the National Museums of Scotland (Edinburgh, United Kingdom), four major Japanese collections (Hokkaido University, Sappporo; Kobe University, Kobe; Meijo University, Nagoya; and the Osaka Museum of Natural History, Osaka), the New Zealand Arthropod Collection (Auckland, New Zealand), Naturalis (Leiden, the Netherlands), the Hungarian Natural History Museum (Budapest, Hungary), and the Austrian Natural History Museum (Vienna, Austria). Extensive non-type material, representing thousands of specimens worldwide, were borrowed for study from several institutions in Canada, Costa Rica, France, Sweden, Thailand, and the United States. Several online databases such as the Barcoding of Life Data Systems (http://v4.boldsystems.org/) and Area de Conservación Guanacaste (ACG), Costa Rica (http://janzen.sas.upenn.edu/caterpillars/database.lasso) were searched as well. The final data were input into an Excel file, which is provided here as a supplementary file to facilitate access to all information for personal use and editing (Suppl. material 1). We also provide an index of all available species names of Microgastrinae in strict alphabetical order; with the valid names in bold and italics, and the synonyms, homonyms, and nomina dubia just in italics (Suppl. material 2).

After the initial list was compiled, all species were assessed as comprehensively as possible, including: a) examination of primary types whenever possible (in a few cases we examined high quality illustrations of the primary types, which were sufficient to establish their generic placement unambiguously; in those cases we clearly indicate the source of the illustrations); b) study of secondary types and/or authenticated specimens (= specimens in collections identified by experts on the group; in those cases we mention the name of the expert identifying the species); and c) checking relevant literature, either the original description (including illustrations whenever available) or subsequent references where the species was treated (e.g., taxonomic revision, regional checklist, etc.). Throughout the checklist, “not examined but original description checked” or “not examined but subsequent treatment of the species checked” means that one of us checked those references. For every species, we detail how we assessed its status, as it is evident that the conclusion will be more reliable if the primary type was examined as opposed to secondary types, authenticated specimens, or the reading of a description. For species where we could neither examine specimens nor check for relevant literature we (explicitly) maintain the original generic combination.

For a few species, mostly in *Apanteles* and *Microgaster*, the available information (usually only the original description) was enough to suggest that they belonged to a different genus, but not enough to confidently place them in another genus (usually because several alternatives were possible, or none was clear). In those cases we considered the species as *species inquirendae* and add a question mark before the genus name it was originally described in (e.g., ? *Apanteles*) to indicate the questionable generic placement.

In the checklist, at the beginning of each genus we detail its author, year of publication and page (of the original description of the genus), gender of the genus name, type species, genus synonyms, and comments (if needed). As far as we know, the gender of every Microgastrinae genus has not been stated in a single publication before (e.g., Shenefelt (1972, 1973) did not address that; Mason (1981) only discussed the gender of some of the new genera described there; Yu et al. (2016) did not present that information either). For our checklist we follow the original publication (if the gender was stated there), or expert advice from an ICZN commissioner (Doug Yanega, pers. comm.).

For each species in the checklist we provide current name, original combination, synonyms, homonyms, and details of the primary type (including sex, holding institution, and country of the type locality), as well as details of the recorded geographical distribution of the species. Where necessary, additional comments are added at the end of the species’ treatment under “Notes”. We do not include full details on the combination history of the species name or further taxonomic details (other than the ones detailed above). For such details, Taxapad (Yu et al. 2012, 2016) and Shenefelt (1972, 1972) must be consulted.

The spelling of some author’s last names was found to vary in the literature: de Saeger/De Saeger, de Santis/De Santis, Fernandez-Triana/Fernandez-Triana, Foerster/Förster, van Achterberg/Van Achterberg. For the sake of consistency, in this paper we are using the first alternative in each of the above cases. The only exception is María Teresa Oltra Moscardó (Spain), as she has recorded her last name in several publications as either Oltra (referring to species authorship and also as paper authorship for most of her papers) or Oltra-Moscardó (only applying to one paper cited in our checklist: Oltra-Moscardó and Jiménez-Peydró 2005). In this case we use the appropriate alternative according to the corresponding reference cited, but for all eight species that she has described we refer to her as Oltra.

The availability of species names was assessed following the latest version of the International Commission on Zoological Nomenclature (ICZN); throughout the text any reference to ICZN articles follows the online version (https://www.iczn.org/the-code/the-international-code-of-zoological-nomenclature/the-code-online/).

Details on species distribution are first presented by biogeographical regions, and then by countries within biogeographical regions, in both cases arranged in alphabetical order. For biogeographical boundaries we follow the O’Hara et al. (2009) approach of combining the Australasian and Oceanian regions into one, with the name of the former. Throughout the text we use six regions (there are no Microgastrinae recorded from their Antarctic region), abbreviated as follows: **NEO** Neotropical (sometimes referred to as Neotropics), **NEA** Nearctic, **PAL** Palaearctic, **OTL** Oriental, **AFR** Afrotropical (sometimes referred to as Afrotropics), and **AUS** Australasian.

Occasionally, we use wider terms such as Holarctic (NEA and PAL), New World (NEA and NEO), Old World tropics (AFR, OTL and AUS), and pantropical (NEO, AFR, OTL, AUS). Some of these terms can be vague or hard to define precisely (e.g., some of the Australasian or southern Neotropical taxa are not really “tropical”, and the southern limits of the Holarctic region have a mix of temperate and subtropical taxa). However, they are used throughout the paper as a way to discuss trends in generic distribution and are not meant to be taken as strictly defined boundaries.

The list of countries follows the Standard ISO 3166 (codes for names of countries and their subdivisions: https://www.iso.org/obp/ui/#search). Throughout the text, we abbreviate United States of America as USA. For the six largest countries by area (Russia, Canada, China, USA, Brazil and Australia) we also present finer species distributions by country subdivisions (provinces, republics, states, territories, etc.). For Australian states and territories, we follow http://www.bda-online.org.au/help/bda-conventions/abbreviations-states/. For states of the USA and for Canadian provinces and territories, acronyms consisting of two capital letters are used, following Canada Post (http://www.canadapost.ca/tools/pg/manual/PGaddress-e.asp). We follow Standard ISO 3166 for China provinces (https://www.iso.org/obp/ui/#iso:code:3166:CN) and Brazil states (https://www.iso.org/obp/ui/#iso:code:3166:BR). For Russia subdivisions we mostly follow Standard ISO 3166 (https://www.iso.org/obp/ui/#iso:code:3166:RU), but see next paragraph for explanation on exceptions.

In most cases the information on species distribution per subdivisions was summarized from Yu et al. (2016), with updates from publications after that date. For Brazil we followed Shimbori et al. (2019). For Russia we mostly followed Yu et al. (2016), but we also added information from a recent update from Belokobylskij et al. (2019). However, Belokobylskij et al. (2019) combined several of the Russian subdivisions (according to the Standard ISO 3166, followed by Yu et al. 2016 and also by us in this paper) into broader categories, its “geoscheme for Russia” being different. As a result, some species recorded from Russia have its distribution detailed only to the level of those broader categories, as dealt with by Belokobylskij et al. (2019). The acronyms for those categories are as follow: **C** Centre, **E** East, **N** North, **NC** North Caucasus, **NW** North-West and **S** South, in the “European Part of Russia”; **IR** Irkutsk Province, in “Eastern Siberia”; **UR** Ural in the “Ural” (no province or territory detailed); **KA** Kamchatka Territory and **PR** Primorskii Territory, in the “Far East” (for more details see Belokobylskij et al. 2019: 9, fig. 1 on page 10).

Some countries have political units located in different biogeographical regions (or, in some cases, islands which are separate from the continent where the country is located), we considered those units as separate entities in our checklist (and the “country” in those cases is recorded as the separate entity and not the actual country it politically belongs to). Those cases are: Chile (Juan Fernández Islands), France (French Guiana, Guadeloupe, Marquesas Islands, Réunion, Society Islands), Japan (Ryukyu Islands), the Netherlands (Netherlands Antilles), Portugal (Azores, Madeira Islands, Selvagens Islands), Spain (Canary Islands), United Kingdom (British Virgin Islands, Saint Helena), and USA (American Samoa, Hawaiian Islands, and the USA Virgin Islands).

For all species historically recorded from the former Czechoslovakia we were able to separate the records that belong to either Czech Republic or Slovakia, based on Capek and Lukas (1989). However, for some species historically recorded from the former Yugoslavia (currently six or seven different countries, depending on the source) and also from the former Sudan (currently two countries: Sudan and South Sudan), the sources of the species records did not contain enough information to determine to which country they currently belong; therefore we annotate those records just as Yugoslavia and Sudan respectively.

Apart from some general comments on Microgastrinae hosts, we have not attempted to add host information for particular species; we intend to publish a critical assessment of Microgastrinae host records at a later date. We do, however, state general trends in host parasitization on a generic level. We follow the arrangement in Aarvik et al. (2017) when referring to families and superfamilies of Lepidoptera. Taxapad (Yu et al. 2016) gives almost complete information on published host records up to the end of 2015, but that source is inevitably very far from a reliable indication of true host associations. A complete and critical analysis of those records would require a huge effort, and in many cases it might be very difficult to determine unambiguously which ones are correct. In this respect, the amount of misinformation in the general literature is far larger than generally realised and can completely mask any real understanding of a parasitoid’s host range; Noyes (1994), Shaw (1994) and Shaw and Aeschlimann (1994) discuss this with examples.

For collection acronyms we mostly follow the website “Insect and Spider Collections of the World” (http://hbs.bishopmuseum.org/codens/codens-r-us.html). In cases where institutions were not listed there, we propose codens based on some abbreviation of the institution name. The complete list of institutions mentioned in this paper is:

**AEIC** American Entomological Institute, Utah State University, Logan, USA

**AMNH** American Museum of Natural History, New York, New York, USA

**AMUZ** Aligarh Muslim University, Zoological Museum, Aligarh, Uttar Pradesh, India

**ANIC** Australian National Insect Collection, CSIRO, Canberra City, Australia

**ANSP** Academy of Natural Sciences, Philadelphia, Pennsylvania, USA

**BAMU** Dr. Babasaheb Ambedkar Marathwada University, Aurangabad, India

**BGM** Beth Gordon Agriculture and Nature Study Institute, Deganya, Israel

**BPBM** Bernice P. Bishop Museum, Honolulu, Hawaii, USA

**CAS** California Academy of Sciences, San Francisco, California, USA

**CBGP** Centre de Biologie pour la Gestion des Populations, Montpellier, France

**CFRB** Chinese Academy of Forestry, Forest Research Institute, Beijing, China

**CNC** Canadian National Collection of Insects, Ottawa, Canada

**CUIC** Cornell University, Ithaca, New York, USA

**DCBU** Departamento de Ecologia e Biologia Evolutiva, Universidad Federal de São Carlos, São Carlos, Brazil

**DCMP** Universidade Federal do Paraná, Curitiba, Paraná, Brazil

**DPBA** Departamento de Patologia Vegetal, Buenos Aires, Argentina

**DPPZ** Department of Plant Protection, University of Zabol, Zabol, Iran

**DZCU** Department of Zoology, University of Calicut, Kerala, India

**DZUC** University of Ceylon, Department of Zoology, Colombo, Sri Lanka

**EBW** Deutsches Entomologisches Institut, Eberswalde, Germany

**EIHU** Hokkaido University, Sapporo, Hokkaido, Japan

**ESUW** University of Wyoming, Laramie, USA

**FAFU** Fujian Agriculture and Forestry University, Fuzhou, China

**FNIC** Fiji National Insect Collection, Suva, Fiji

**FSCA** Florida State Collection of Arthropods, Division of Plant Industry, Gainesville, USA

**GUGC** Guizhou University, Guiyang, China

**HNHM** Hungarian Natural History Museum, Budapest, Hungary

**HUNAU** Hunan Agricultural University, Changsha, China

**IAVH** Instituto Alexander von Humboldt, Bogotá, Colombia

**IEAS** Academia Sinica, Institute of Entomology, Shanghai, Shanghai, China

**IEBR** Institute of Ecology and Biological Resources, Hanoi, Vietnam

**IECA** Institute of Entomology, Ceské Budejovice, Czech Republic

**IFRI** Indian Forest Research Institute, Dehradun, Uttarakhand, India

**IIAF** Instituto de Investigaciones Agropecuarias y Forestales, Universidad Michoacana San Nicolás de Hidalgo, México

**INBio** Instituto Nacional de Biodiversidad, Santo Domingo de Heredia, Costa Rica

**INHS** Illinois Natural History Survey, Champaign, Illinois, USA

**INPC** National Pusa Collections, Indian Agricultural Research Institute, New Delhi, India

**KUEC** Kyushu University, Fukuoka, Japan

**LNKD** Landessammlung für Naturkunde, Karlsruhe, Germany

**LSUK** The Linnean Society of London, London, United Kingdom

**LUNZ** Lincoln University, Lincoln, New Zealand

**MACN** Museo Argentino de Ciencias Naturales, Buenos Aires, Argentina

**MCZ** Museum of Comparative Zoology, Harvard University, Cambridge, USA

**MHNG** Muséum d'Histoire Naturelle, Geneva, Switzerland

**MIUP** Museo de Invertebrados Graham Bell Fairchild, Universidad de Panamá, Panama

**MLP** Museo de La Plata, La Plata, Argentina

**MMBC** Moravske Muzeum [Moravian Museum], Brno, Czech Republic

**MNCN** Museo Nacional de Ciencias Naturales, Madrid, Spain

**MNHN** Muséum National d'Histoire Naturelle, Paris, France

**MNNC** Museo Nacional de Historia Natural, Santiago, Chile

**MUSM** Museo de Historia Natural, Universidad Nacional Mayor de San Marcos, Lima, Peru

**MVMMA** Museums Victoria, Melbourne Museum, Melbourne, Australia

**MZH** Finnish Museum of Natural History, Helsinki, Finland

**MZLU** Lund University, Lund, Sweden

**MZUSP** Museum of Zoology, University of São Paulo, Brazil

**NBAIR** National Bureau of Agricultural Insect Resources, Bangalore, India

**NHMO** Zoological Museum, University of Oslo, Oslo, Norway

**NHMUK** Natural History Museum, London, United Kingdom

**NHMW** Naturhistorisches Museum Wien, Vienna, Austria

**NHRS** Naturhistoriska Riksmuseet, Stockholm, Sweden

**NIAES** National Institute for Agro-Environmental Sciences, Tsukuba, Japan

**NMID** National Museum of Ireland, Dublin, Ireland

**NMKE** National Museum of Kenya, Nairobi, Kenya

**NZAC** New Zealand Arthropod Collection, Landcare Research, Auckland, New Zealand

**NZSI** National Zoological Collection, Zoological Survey of India, Kolkata, West Bengal, India

**OUMNH** Museum of Natural History, Oxford University, United Kingdom

**PCMAG** Plymouth City Museum and Art Gallery, Plymouth, United Kingdom

**PPRI** Plant Protection Research Institute, Pretoria, Gauteng, South Africa

**QM** Queensland Museum, South Brisbane, Queensland, Australia

**QSBG** Queen Sirikit Botanic Garden, Chaing Mai, Thailand

**QCAZ** Pontificia Universidad Católica del Ecuador, Quito, Ecuador

**RBINS** Royal Belgian Institute of Natural Sciences, Brussels, Belgium

**RMCA** Musée Royal de l'Afrique Centrale, Tervuren, Belgium

**RMNH** Naturalis Biodiversity Centre, Leiden, Netherlands

**RSME** National Museums of Scotland, Edinburgh, United Kingdom

**SAMA** South Australian Museum, Adelaide, South Australia, Australia

**SAMC** Iziko Museum of Capetown, Cape Town, South Africa

**SAUC** Shandong Agricultural University, Tai'an, China

**SCAC** South China Agricultural College, Guangzhou, Guangdong, China

**SEMC** Snow Entomological Museum, University of Kansas, Lawrence, Kansas, USA

**SIZK** Schmalhausen Institute of Zoology, Kiev, Ukraine

**SJCA** St. John's College, Agra, Uttar Pradesh, India

**SMF** Forschungsinstitut und Naturmuseum Senckenberg, Frankfurt-am-Main, Germany

**SUKI** Shivaji University, Kolhapur, India

**TARI** Taiwan Agricultural Research Institute, Taichung, Taiwan, China

**TFRI** Insect Museum, Tropical Forest Research Institute, Jabalpur, Madhya Pradesh, India

**TMAG** Tasmanian Museum and Art Gallery, Hobart, Tasmania, Australia

**TMSA** Ditsong National Museum of Natural History, Pretoria, Gauteng, South Africa

**TMUC** Department of Entomology, Tarbiat Modares University, Tehran, Iran

**TUDTG** Technische Universität Dresden, Department of Forest Science, Tharandt, Germany

**UCDC** R.M. Bohart Museum of Entomology, University of California, Davis, California, USA

**UFSM** Universidade Federal de Santa Maria, Rio Grande do Sul, Brazil

**UFVB** Universidade Federal de Viçosa, Museum of Entomology, Viçosa, Minas Gerais, Brazil

**UKM** Universiti Kebangsaan, Bangi, Selangor, Malaysia

**UKZMP** Universiti Kebangsaan, Bangi, Selangor, Malaysia

**ULQC** University of Laval, Quebec City, Canada

**USNM** National Museum of Natural History, Washington, USA

**UUZM** Uppsala University, Uppsala, Sweden

**UVS** University of Valencia, Valencia, Spain

**VNMN** Vietnam National Museum of Nature, Vietnam Academy of Science and Technology, Hanoi, Vietnam

**WAM** Western Australian Museum, Perth, Western Australia, Australia

**ZIN** Zoological Institute, Russian Academy of Sciences, St. Petersburg, Russia

**ZJUH** Parasitic Hymenoptera Collection, Zhejiang University, Hangzhou, China

**ZMHB** Museum für Naturkunde der Humboldt-Universität, Berlin, Germany

**ZMTU** Zoological Museum, Trakya University, Turkey

**ZMUC** Zoological Museum, University of Copenhagen, Copenhagen, Danmark

**ZMUK** Zoologisches Museum, Universität Kiel, Kiel, Germany

**ZSM** Zoologische Staatssammlung, Munich, Germany

The concept of DNA barcoding as a tool for species discovery and identification was proposed approximately 15 years ago (Hebert et al. 2003a, 2003b). A short DNA sequence, approximately 650 base pairs (bp) in the mitochondrial gene encoding cytochrome c oxidase subunit 1 (CO1), has been accepted as a practical and standardized DNA barcode for many groups of animals (e.g., Kress et al. 2015). The Barcode Index Number (BIN) System uses DNA barcodes to indicate possible species limits (see more details on the BIN concept in Ratnasingham and Hebert 2013), and it has been used in taxonomic studies of Microgastrinae (e.g., Fernandez-Triana and Boudreault 2018, Fagan-Jeffries et al. 2018b). In the checklist below we provide details of the number of DNA barcode sequences and BINs for every genus of Microgastrinae currently available in the Barcoding of Life Data Systems (BOLD, see also http://v4.boldsystems.org/index.php) as of 31 December 2019. Sequences were considered as “barcode compliant” if they fulfilled the requirements set in Ratnasingham and Hebert (2007), namely: the sequence has at least 500 nucleotides with fewer than 1% ambiguous base calls (Ns); it has a species name (assigned by an expert taxonomist) or a provisional name; it has a unique specimen identifier, information related to the voucher specimen (including the name of the institution storing the voucher), and a collection record (e.g., collector, collection date, collection location, geospatial coordinates); and it has the sequence of PCR primers used to generate the CO1 amplicon and the trace files (Santschi et al. 2013).

We provide brief morphological diagnostic features and colour illustrations for all 81 valid genera of Microgastrinae (at least one species per genus is illustrated, usually more). For morphological terms we follow several published references (Huber and Sharkey 1993, Sharkey and Wharton 1997, Karlsson and Ronquist 2012, Fernandez-Triana et al. 2014e) as well as the Hymenoptera Anatomy Ontology (HAO) website (http://portal.hymao.org/projects/32/public/ontology/). We use the abbreviations T1, T2, and T3 for metasomal mediotergites 1, 2, and 3; and the fore wing second submarginal cell is mentioned throughout the text as areolet for the sake of brevity.

Photographs were taken with either a Keyence VHX-1000 Digital Microscope or with a Leica camera on a Leica M165 C Microscope, using lenses with a range of 10–130 ×. Multiple images were taken of a structure through the focal plane and then combined to produce a single in-focus image using the software associated with the Keyence System or, for the images taken with the Leica camera, the Zerene Stacker program (http://zerenesystems.com/cms/stacker). Images were corrected using Adobe Photoshop CS4 and Gimp 2.10.12; the plates were prepared using Microsoft PowerPoint 2010 and later saved as .tiff files. For seven figures in our paper we used other sources, all of which are acknowledged in the corresponding figure caption and in the Acknowledgements section below.

In the Results section, we discuss several topics concerning Microgastrinae before providing the checklist of world species. These include a detailed explanation of the generic concepts used here, geographical patterns, general overview of host data in the subfamily, extinct taxa, and limitations of both Taxapad and our checklist. It is very important to understand the limitations, as the user must be aware of the areas where Taxapad and/or our list lack strong support, e.g., critical review of host data, and/or missing information, such as examination of primary types. Further, there will undoubtedly be some yet to be recognised synonymy. We hope future versions of our world checklist will address some of the shortcomings of the present one. We also hope to prepare an online version that is continuously updated, probably in the style of a similar effort currently outdated (http://microgastrinae.myspecies.info/).

## Results

### Overview of the present paper and its limitations

In the checklist below, a total of 81 genera and 2,999 extant species are recognized as valid, including 36 nominal species that are currently considered to be *species inquirendae*.

Two genera are synonymized under *Apanteles*: *Cecidobracon* Kieffer & Jörgensen, 1910 syn. nov., and *Holcapanteles* Cameron, 1905 syn. nov. Nine lectotypes are designated. A total of 318 new combinations, three new replacement names, three species name amendments, and seven species status revised are proposed. Additionally, three species names are treated as *nomina dubia*, and 52 species names are considered to be unavailable (including 14 as *nomina nuda*), listed at the end of the checklist.

Extinct taxa, only known as fossils (three genera and 12 species) are listed in a separate section below (Table 3).

**Figure 1.**
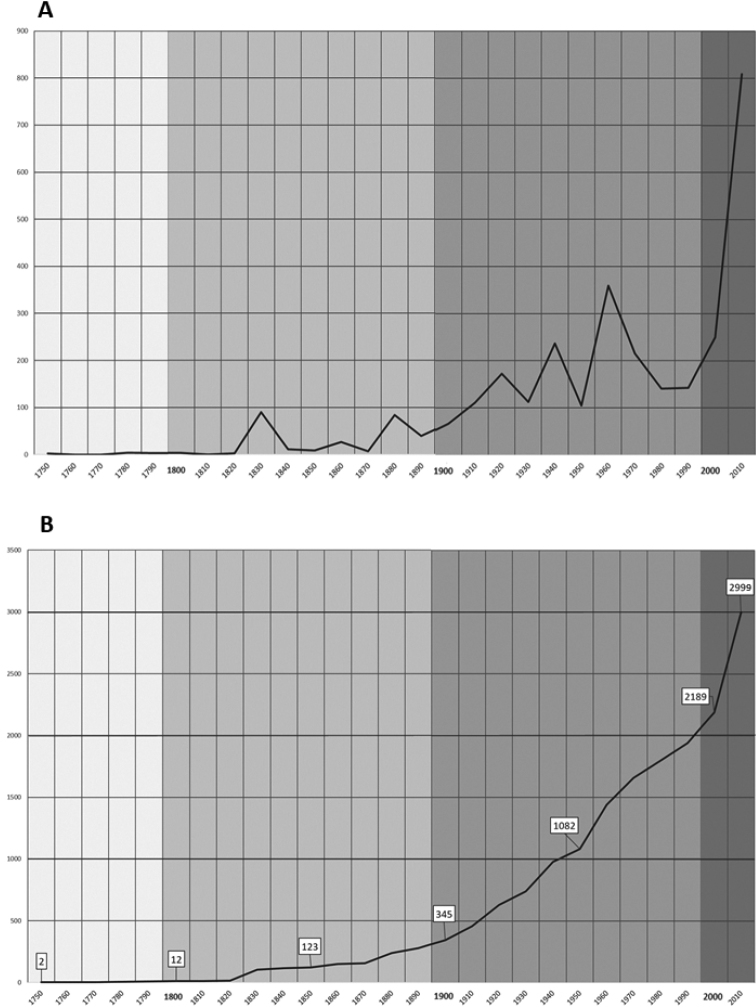
Microgastrinae species described since 1758 based on data in present paper **A** Total numbers per decade **B** Cumulative number (1758–2019).

The pace of species description in Microgastrinae has been steadily increasing since the first species was described in 1758 and has shown no signs of slowing down (Fig. 1). The total number of genera has also increased substantially, especially since 1965; the information is summarized in Whitfield et al. (2018), Fernandez-Triana and Boudreault (2018), and below.

Primary types of Microgastrinae are deposited in 108 institutions worldwide, although 76% of those types are concentrated in seventeen collections (Table 1), seven of which have more than 100 primary types each. Localities of primary types are reported from 138 different countries.

Microgastrine wasps have been recorded in most countries and all continents except Antarctica. Only 16 countries do not yet have any recorded species of Microgastrinae: Bahrain, Botswana, Bhutan, Cambodia, Djibouti, Equatorial Guinea, Gabon, Gambia, Guinea, Guinea-Bissau, Kuwait, Laos, Liberia, Mauritania, Qatar, and Swaziland. This is of course just an artifact of insufficient collecting and/or lack of studies in those countries; each is expected to harbour many species.

The current data (Table 2) show two countries with 400+ Microgastrinae species each (China with 448 and Costa Rica with 427), another two with 300+ species each (Russia with 388 and Hungary with 327) and five with 200+ species each (USA, Germany, India, United Kingdom, and Canada). Overall, 34 countries have more than 100 described species recorded, although those numbers can be misleading. For example, the reason Hungary ranks so high is because of extensive studies in that country, done over many years by Jenö Papp while working in the Hungarian Natural History Museum. A similar situation applies to both the United Kingdom and Germany, where a long European tradition of experts on the group coupled with extensive collecting have provided figures that are close to the actual diversity in those countries. While the microgastrine fauna of those three countries is relatively well known, the opposite occurs in large and/or mostly tropical countries, where more species are still undescribed. For example, in Costa Rica, DNA barcoding has already identified more than 1,200 species just in ACG (Janzen and Hallwachs 2016). And the figures for China and India (which are considered to be “megadiverse countries”, *sensu*Myers et al. 2000), are still very far from being complete, as both countries should easily reach more than 1,000 species each. Other megadiverse countries such as Australia, Brazil, Colombia, Democratic Republic of Congo, Indonesia, Madagascar, Mexico, Peru, Malaysia, Papua New Guinea and USA are all likely to have similar (in some cases higher) totals, but studies thus far have been insufficient, leading to most of those countries having “only” a hundred species or fewer recorded at present.

There are three main limitations in our paper that we want to point out. The first relates to the coverage of primary types in our study. We were able to examine primary types for 1,394 species (46.5%), and for another 1,568 species (52.3%) we studied authenticated specimens, checked original descriptions, or read subsequent revisions. However, for 37 species (1.2%) we could not check any source of information, or it was considered inadequate, and they are left in the genus in which they were originally described (or as *species inquirendae*), with explanatory annotations. In future versions, we aim to increase the number of species for which we have examined primary types, but for the present paper the reader must consider the relatively large number of species still needing to be thoroughly studied. It is especially important to keep in mind that for some of those species for which we could only study descriptions (which may not be detailed or clear enough), the generic placement made in this paper might be incorrect.

A second limitation is the coverage of references concerning Microgastrinae. In the References section we tried to list all papers where original descriptions of Microgastrinae were published (those references in turn are cited under the corresponding treatment of every species in the checklist below). However, our list is not complete and we are aware of some omissions; in that sense, the latest versions of Taxapad (Yu et al. 2012, 2016) have more comprehensive lists of references than our paper. Especially important and comprehensive is Yu et al. (2016), which lists 6,200+ references related to Microgastrinae.

A third limitation of our paper is that we do not treat host records in detail. We expect to present host data for microgastrine species with verified information in a subsequent version of the world checklist, although it is improbable that we will be able to comment with reliability on all published records. The latest versions of Taxapad (Yu et al. 2012, 2016) provide the best coverage of references on hosts of Microgastrinae; however, that is only an uncritical compilation of literature, and many of those references report incorrect data. The reader is strongly advised to double check host references and be very cautious in interpreting information from secondary sources.

**Table 1.** World collections with the largest numbers of primary types of Microgastrinae (data from valid species as recognized in the present paper).

**Collection code**	**Country**	**Number of primary types**
NHMUK	UK	491
CNC	Canada	476
USNM	USA	380
ZJUH	China	160
RMCA	Belgium	122
ZIN	Russia	113
HNHM	Hungary	108
MNHN	France	84
FAFU	China	63
ANIC	Australia	52
SIZK	Ukraine	44
ZMHB	Germany	40
MACN	Argentina	36
RMNH	The Netherlands	35
AEIC	USA	32
EIHU	Japan	29
HUNAU	China	29

**Table 2.** Alphabetic list of countries with described species of Microgastrinae (data based on this paper). Countries with political units located in different biogeographical regions (mostly islands) have species recorded from those entities listed separately below; those species are not added to the total for the country to which the entities belong politically.

Countries	No. of Species	Countries	No. of Species
Afghanistan	20	Lithuania	70
Albania	7	Luxembourg	1
Algeria	7	Macedonia	37
Andorra	2	Madagascar	67
Angola	1	Malawi	11
Argentina	68	Malaysia	70
Armenia	105	Mali	1
Australia	129	Malta	18
Austria	97	Mauritius	12
Azerbaijan	126	Mexico	54
Bahamas	1	Moldova	113
Bangladesh	11	Mongolia	161
Barbados	2	Montenegro	23
Belarus	23	Morocco	14
Belgium	61	Mozambique	7
Belize	7	Myanmar	9
Benin	3	Namibia	1
Bolivia	10	Nepal	6
Bosnia and Herzegovina	6	Netherlands	105
Brazil	120	Netherlands (Netherlands Antilles)	1
Brunei	1	New Zealand	27
Bulgaria	128	Nicaragua	5
Burkina Faso	1	Niger	1
Burundi	1	Nigeria	16
Cape Verde	32	Norway	15
Cameroon	13	Oman	1
Canada	213	Pakistan	20
Central African Republic	2	Panama	22
Chad	1	Papua New Guinea	47
Chile	21	Paraguay	10
Chile (Juan Fernández Islands)	2	Peru	39
China	448	Philippines	90
Colombia	31	Poland	170
Comoros	1	Portugal	7
Democratic Republic of Congo	135	Portugal (Azores)	3
Costa Rica	427	Portugal (Madeira Islands)	14
Croatia	70	Portugal (Selvagens Islands)	2
Cuba	20	Romania	174
Cyprus	11	Russia	388
Czech Republic	90	Rwanda	59
Denmark	20	Saint Kitts & Nevis	2
Dominica	3	Saint Lucia	2
Dominican Republic	5	Saint Vincent	18
Ecuador	101	Saudi Arabia	2
Egypt	12	Senegal	51
El Salvador	1	Serbia	95
Eritrea	3	Sierra Leone	3
Estonia	12	Singapore	11
Ethiopia	11	Slovakia	161
Fiji	29	Slovenia	18
Findland	162	Solomon Islands	5
France	122	Somalia	2
France (French Guiana)	6	South Africa	98
France (Guadeloupe)	2	Spain	103
France (Marquesas Islands)	1	Spain (Canary Islands)	18
France (Réunion)	34	Sri Lanka	37
France (Society Islands)	2	Sudan	8
Gambia	1	Suriname	5
Georgia	73	Sweden	121
Germany	248	Switzerland	166
Ghana	6	Syria	2
Greece	92	Tajikistan	42
Grenada	15	Tanzania	23
Guatemala	6	Thailand	30
Guyana	12	Togo	3
Haiti	2	Tonga	2
Honduras	8	Trinidad & Tobago	19
Hungary	327	Tunisia	40
Iceland	5	Turkey	173
India	245	Turkmenistan	63
Indonesia	63	Uganda	35
Iran	109	Ukraine	154
Iraq	2	United Arab Emirates	3
Ireland	81	United Kingdom	242
Israel	72	United Kingdom (British Virgin Islands)	1
Italy	149	United Kingdom (Saint Helena)	1
Ivory Coast	16	United States	299
Jamaica	6	United States (American Samoa)	3
Japan	96	United States (Hawaiian Islands)	14
Japan (Ryukyu Islands)	7	United States (USA Virgin Islands)	1
Jordan	10	Uruguay	11
Kazakhstan	121	Uzbekistan	72
Kenya	30	Vanuatu	8
Korea	130	Venezuela	21
Kyrgyzstan	18	Vietnam	137
Latvia	37	Western Samoa	10
Lebanon	2	Yemen	17
Lesotho	1	Zambia	3
Libya	2	Zimbabwe	7

### Fossil Microgastrinae taxa

Extinct genera and species of Microgastrinae have been found in Eocene and Oligocene deposits, from 37–44 million years ago (MYA). Many specimens from the Miocene (20–30 MYA) are known from Dominican and Chiapas ambers, but most appear to be undescribed representatives of extant genera (Murphy et al. 2008). Belokobylskij (2014) revised the taxonomic status of all previously known taxa of fossil Microgastrinae and described one new genus as well as two new species. The origin of Microgastrinae has been estimated at ~ 54 MYA (Murphy et al. 2008).

Unlike previous work (Mason 1981, Yu et al. 2005, 2012, 2016), we exclude fossil genera or species from our world checklist. Instead, we tabulate in this section the three genera and 12 species of fossil Microgastrinae currently described (Table 3).

**Table 3.** Extinct genera and species of Microgastrinae, compiled from Yu et al. (2012, 2016) and Belokobylskij (2014).

**Genera only known from fossils**	**Species only known from fossils**
*Dacnusites* Cockerell, 1921	*Apantelesconcinnus* Statz, 1938
*Eocardiochiles* Brues, 1933	*Apantelesmacrophthalmus* Statz, 1938
*Palaeomicrogaster* Belokobylskij, 2014	*Dacnusitesreductus* Cockerell, 1921
	*Dacnusitessepultus* Cockerell, 1921
	*Eocardiochilesfritschii* Brues, 1933
	*Microplitiselegans* Timon-David, 1944
	*Microplitisprimordialis* (Brues, 1906)
	*Microplitisvesperus* Brues, 1910
	*Semionisnixoni* Tobias, 1987
	*Semioniswightensis* Belokobylskij, 2014
	*Snelleniussuccinalis* Brues, 1933
	*Palaeomicrogasteroculatus* Belokobylskij, 2014

### Generic limits and taxonomic arrangement of the subfamily Microgastrinae

Microgastrinae was originally described at family rank, as ‘Microgasteroidae’, by Foerster (1863). At that time, it comprised only three genera: *Microgaster* Latreille, 1804 (the genus that provides the root for the subfamily name, meaning “small abdomen”, in reference to the relatively short length of the metasoma compared to other Braconidae), as well as two genera described by Foerster (1863): *Microplitis* (which means “small sword” or “small weapon”, referring to the generally short ovipositor in that genus) and *Apanteles* (meaning “incomplete”, in reference to the fore wing lacking the second intercubitus, leaving the second submarginal cell open or incomplete). *Fornicia*, although described by Brullé (1846) before Foerster’s work, was at the time considered to belong to other subfamilies in Braconidae (e.g., Dalla Torre (1898) placed the genus in Cheloninae; Ashmead (1900a) placed it in Sigalphinae; Granger (1949) placed it in Triaspidinae), and it was not recognized to be part of Microgastrinae until a century later (Baltazar 1962, Nixon 1965).

The high diversity of Microgastrinae quickly became evident, and so attempts to split the group into further genera started shortly after Foerster’s (1863) paper, e.g., by Reinhard (1880). Many additional genera (15 recognized in this paper) were described between 1882 and 1958, although some were not associated with Microgastrinae at the time, and others were not accepted as valid genera by some authors of the period, e.g., Muesebeck (1921) and Telenga (1955).

This view changed with two seminal works in 1965 and 1981. Nixon (1965) reclassified the subfamily limits and provided some structure to what was being recognized as a huge assemblage of parasitoids of Lepidoptera. He recognized 20 genera, eight of which were new, and reclassified the species within *Apantelessensu lato* into a more practical and useful array of 44 species groups to facilitate identification. Mason (1981) fundamentally changed the taxonomy of Microgastrinae by recognizing 50 genera (23 of which he described as new), including numerous taxa that mostly corresponded to particular species groups of Nixon (1965, 1973), and additionally proposing new combinations for some 350 species.

Since Mason (1981) 32 genera have been described. Whitfield et al. (2018: fig. 2) graphically showed the increase in description of new genera during the past 150 years. Nevertheless, there are still many more genera of Microgastrinae that remain to be described, e.g., Fernandez-Triana and Boudreault (2018). Additionally, several genera, as currently understood, are probably polyphyletic and need to be split, e.g., *Diolcogaster* and *Glyptapanteles*. A comprehensive phylogenetic analysis of the subfamily is needed before we can achieve a clearer picture. However, just based on the material we have seen in collections, we estimate that the Microgastrinae is likely to comprise close to one hundred genera.

**Figure 2.**
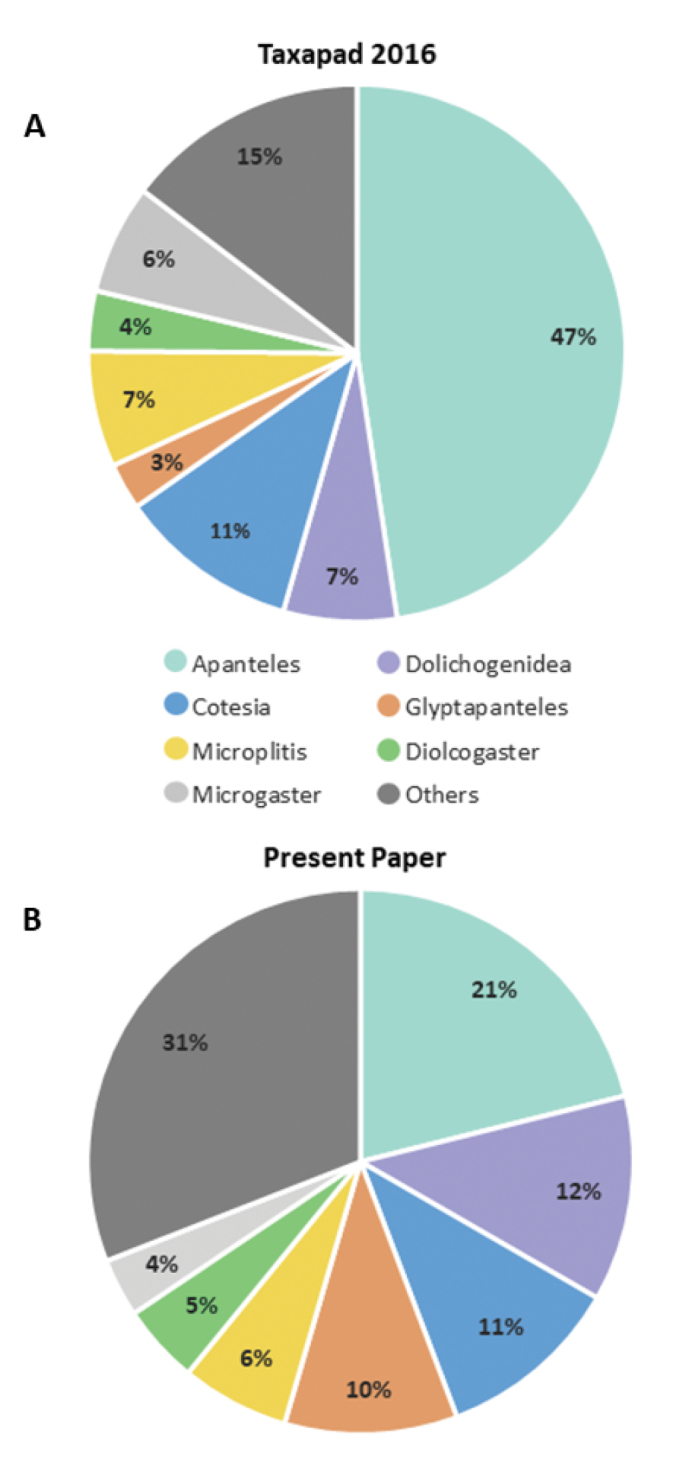
Number of extant species per larger genera of Microgastrinae**A** Data from Taxapad 2016, which is mostly an update, with slight modifications, of van Achterberg (2003), total number of species: 2,710 **B** Data from present paper, which is mostly based on Mason (1981) but extensively updated, total number of species: 2,999.

For the past few years the main problem with the generic concepts is that two different classifications of Microgastrinae have been proposed and are widely used: those based on Mason (1981) and on van Achterberg (2003). For a visual depiction of how the two classifications differ (based on the number of species assigned to each of the most speciose genera), see Figure 2.

The classification proposed by Mason (1981) had a narrower concept of *Apanteles* and *Protapanteles*, which resulted in a larger number of Microgastrinae genera treated as valid. Many of the new combinations resulting from that classification are in Mason (1981), although not all species have been properly transferred to the corresponding genus. Mason’s system has been followed by most researchers (see examples cited in the Introduction) and has remained largely stable for the past 38+ years, with a few exceptions: his genus *Teremys* was synonymized under *Pholetesor* (Whitfield 2006); and his arrangement of genera within tribes, largely based on phylogenetic grounds, has not been universally accepted (Austin 1990, Austin and Dangerfield 1992, Whitfield 1995a, Fernandez-Triana 2010; see also Walker et al. 1990 for further criticism of tribes within Microgastrinae). Mason (1981) based his paper on studies of the world fauna; however, a careful examination of the CNC collection (Mason’s base) and other material available to him at the time shows that specimens from the Afrotropical, Oriental, and Australasian regions were much more poorly represented than the remaining regions. Thus, most of the new genera from those regions described by Mason (1981) have later been found to have a wider distribution and greater morphological variation than originally thought, and some of those genera will need redefinition. Another consequence of the limited geographical coverage of the studied specimens is that the keys to tribes and genera in Mason (1981) work reasonably well for the temperate areas, but not as well for the tropical areas, especially the Old World tropics.

The classification proposed by van Achterberg (2003) reduced the number of genera by treating eleven genera recognised by Mason as synonyms or subgenera of *Apanteles* and *Protapanteles*. That system was later implemented in Taxapad (Yu et al. 2005, 2012, 2016) and other, mostly European, databases, e.g., Fauna Europaea (https://fauna-eu.org/) and Dyntaxa (https://www.dyntaxa.se/). Shortcomings of this approach have been pointed out by other authors, e.g., Broad et al. (2016) and Whitfield et al. (2018). The main issue with van Achterberg’s approach is that his classification was based mainly on the European species, a region with relatively little diversity in genera and species (see sections below), and is thus clearly insufficient to capture the rich fauna of Microgastrinae worldwide. Second, and more worrisome, van Achterberg’s generic concepts were applied in Taxapad to the entire world fauna, effectively producing numerous (perhaps hundreds) of new name combinations which have never been formally published, let alone critically assessed. The validity of those names may be questionable, but van Achterberg’s classification has been embraced uncritically by some users of Taxapad.

To complicate things further, generic concepts changed slightly in Taxapad from the 2012 to the 2016 version (Table 4). For example, Taxapad 2016 considers some taxa as subgenera that the 2012 version had listed as synonyms of *Apanteles* (*Dolichogenidea*, *Exoryza*, *Iconella*, *Illidops*, and *Pholetesor*) or as synonyms of *Protapanteles* (*Nyereria*, *Rasivalva*, *Sathon*, and *Venanides*). Other genera were treated differently, e.g., *Distatrix* is treated as a valid genus in the 2012 version but as a subgenus of *Protapanteles* in 2016, and *Glyptapanteles* is a synonym of *Protapanteles* in 2012 but a valid genus in 2016. Some of those decisions may have merit, but three are highly questionable:

a) *Rasivalva* should never have been considered to be part of *Protapanteles* as it has a complete areolet in the fore wing (a character not present in any *Protapanteles* or related genera);

b) *Ectadiophatnus* is listed as a genus of Microgastrinae in both the 2012 and 2016 versions, following Shenefelt (1973), despite having been published as belonging to the subfamily Blacinae since at least 1935 (Ferrière 1935, Mani 1938, Varshney 1976, Mason 1981) [van Achterberg (pers. comm.) has examined the type species and found that it is a new synonym of *Eubazus* Nees, in Brachistinae-Brachistini];

c) the species listed under *Lissogaster* have since 1988 been transferred back to *Microgaster* (see more details about that in Mason (1986) and in the checklist below, in the introductory comments to the genus *Microgaster*).

The rationale for the changes between versions of Taxapad is not always evident and, as far as we are aware, has never been explained in a published paper. As a result, it is difficult to follow the different arrangements of genera and subgenera, a problem which is further compounded by the use of tribes in the 2012 version, while the 2016 version added sub-tribes (Table 4).

We believe that the classification proposed by Mason (1981), although not entirely free from problems and shortcomings, provides the best framework currently available to deal with the world diversity of Microgastrinae and provides a solid and clear foundation from which to work towards future improvements. In this paper we largely follow that system, except for dividing the subfamily into tribes, as we do not think the tribes proposed by Mason properly reflect the phylogenetic relationships within the subfamily. We here classify the world species in 81 genera of Microgastrinae (Table 4 and checklist below).

**Table 4.** Microgastrinae arrangement (genera, subgenera, subtribes, and tribes) used in the 2012 and 2016 versions of Taxapad (Yu et al. 2012, 2016) and the present paper. Each column is independent of the others, so the lists must be read vertically only, as they are not comparable horizontally.

Taxapad 2012	Taxapad 2016	Present paper
**MICROGASTRINAE Foerster, 1862**	**MICROGASTRINAE Foerster, 1863**	**MICROGASTRINAE Foerster, 1863**
	**MICROGASTRINI Foerster, 1863**	**(No tribes)**
**APANTELINI Viereck, 1918**	**APANTELINA Viereck, 1918**	**(No subtribes)**
*Alphomelon* Mason, 1981	*Alphomelon* Mason, 1981	*Agupta* Fernandez-Triana, 2018
Apanteles (Apanteles) Foerster, 1862	Apanteles (Apanteles) Foerster, 1863	*Alloplitis* Nixon, 1965
*Dolichogenidea* Viereck, 1911	*Napamus* Papp, 1993	*Alphomelon* Mason, 1981
*Iconella* Mason, 1981	Apanteles (Choeras) Mason, 1981	*Apanteles* Foerster, 1863
*Illidops* Mason, 1981	Apanteles (Dolichogenidea) Viereck, 1911	*Austinicotesia* Fernandez-Triana, 2018
*Napamus* Papp, 1993	Apanteles (Exoryza) Mason, 1981	*Austrocotesia* Austin & Dangerfield, 1992
Apanteles (Choeras) Mason, 1981	Apanteles (Iconella) Mason, 1981	*Beyarslania* Koçak & Kemal, 2009
Apanteles (Exoryza) Mason, 1981	Apanteles (Illidops) Mason, 1981	*Billmasonius* Fernandez-Triana, 2018
*Austrocotesia* Austin & Dangerfield, 1992	Apanteles (Pholetesor) Mason, 1981	*Buluka* de Saeger, 1948
*Exulonyx* Mason, 1981	*Austrocotesia* Austin & Dangerfield, 1992	*Carlmuesebeckius* Fernandez-Triana, 2018
*Miropotes* Nixon, 1965	*Dasylagon* Muesebeck, 1958	*Chaoa* Luo & You, 2004
*Papanteles* Mason, 1981	*Exulonyx* Mason, 1981	*Choeras* Mason, 1981
*Parapanteles* Ashmead, 1900	*Miropotes* Nixon, 1965	*Clarkinella* Mason, 1981
*Pelicope* Mason, 1981	*Papanteles* Mason, 1981	*Cotesia* Cameron, 1891
*Pholetesor* Mason, 1981	*Parapanteles* Ashmead, 1900	*Cuneogaster* Choi & Whitfield, 2006
*Promicrogaster* Brues & Richardson, 1913	*Promicrogaster* Brues & Richardson, 1913	*Dasylagon* Muesebeck, 1958
*Sendaphne* Nixon, 1965	*Sendaphne* Nixon, 1965	*Deuterixys* Mason, 1981
*Xanthapanteles* Whitfield, 1995	*Xanthapanteles* Whitfield, 1995	*Diolcogaster* Ashmead, 1900
**COTESIINI Mason, 1981**	**COTESIINA Mason, 1981**	*Distatrix* Mason, 1981
*Buluka* de Saeger, 1948	*Buluka* de Saeger, 1948	*Dodogaster* Rousse, 2013
*Chaoa* Luo & You, 2004	*Chaoa* Luo & You, 2004	*Dolichogenidea* Viereck, 1911
*Cotesia* Cameron, 1891	*Cotesia* Cameron, 1891	*Eripnopelta* Xiong, van Achterberg & Chen, 2017
*Cuneogaster* Choi & Whitfield, 2006	*Cuneogaster* Choi & Whitfield, 2006	*Exix* Mason, 1981
*Deuterixys* Mason, 1981	*Deuterixys* Mason, 1981	*Exoryza* Mason, 1981
*Diolcogaster* Ashmead, 1900	*Diolcogaster* Ashmead, 1900	*Exulonyx* Mason, 1981
*Distatrix* Mason, 1981	*Exix* Mason, 1981	*Fornicia* Brullé, 1846
*Exix* Mason, 1981	*Glyptapanteles* Ashmead, 1904	*Gilbertnixonius* Fernandez-Triana, 2018
*Larissimus* Nixon, 1965	*Larissimus* Nixon, 1965	*Glyptapanteles* Ashmead, 1904
*Lathrapanteles* Williams, 1985	*Lathrapanteles* Williams, 1985	*Hygroplitis* Thomson, 1895
*Parenion* Nixon, 1965	*Nyereria* Mason, 1981	*Hypomicrogaster* Ashmead, 1898
Protapanteles (Protapanteles) Ashmead, 1898	*Parenion* Nixon, 1965	*Iconella* Mason, 1981
*Glyptapanteles* Ashmead, 1904	Protapanteles (Protapanteles) Ashmead, 1898	*Illidops* Mason, 1981
Protapanteles (Nyereria) Mason, 1981	Protapanteles (Distatrix) Mason, 1981	*Janhalacaste* Fernandez-Triana, 2018
Protapanteles (Rasivalva) Mason, 1981	Protapanteles (Rasivalva) Mason, 1981	*Jenopappius* Fernandez-Triana, 2018
Protapanteles (Sathon) Mason, 1981	Protapanteles (Sathon) Mason, 1981	*Jimwhitfieldius* Fernandez-Triana, 2018
Protapanteles (Venanides) Mason, 1981	Protapanteles (Venanides) Mason, 1981	*Keylimepie* Fernandez-Triana, 2016
*Protomicroplitis* Ashmead, 1898	*Protomicroplitis* Ashmead, 1898	*Kiwigaster* Fernandez-Triana, Ward & Whitfield, 2011
*Pseudovenanides* Xiao & You, 2002	*Pseudovenanides* Xiao & You, 2002	*Kotenkosius* Fernandez-Triana, 2018
*Venanus* Mason, 1981	*Venanus* Mason, 1981	*Larissimus* Nixon, 1965
*Wilkinsonellus* Mason, 1981	*Wilkinsonellus* Mason, 1981	*Lathrapanteles* Williams, 1985
**MICROGASTRINI Foerster, 1862**	**MICROGASTRINA Foerster, 1863**	*Mariapanteles* Whitfield & Fernandez-Triana, 2012
*Beyarslania* Koçak & Kemal, 2009	*Beyarslania* Koçak & Kemal, 2009	*Markshawius* Fernandez-Triana, 2018
*Cecidobracon* Kieffer & Jörgensen, 1910	*Cecidobracon* Kieffer & Jörgensen, 1910	*Microgaster* Latreille, 1804
*Clarkinella* Mason, 1981	*Clarkinella* Mason, 1981	*Microplitis* Foerster, 1863
*Dasylagon* Muesebeck, 1958	*Ectadiophatnus* Cameron, 1913	*Miropotes* Nixon, 1965
*Ectadiophatnus* Cameron, 1913	*Holcapanteles* Cameron, 1905	*Napamus* Papp, 1993
*Holcapanteles* Cameron, 1905	*Hygroplitis* Thomson, 1895	*Neoclarkinella* Rema & Narendran, 1996
*Hygroplitis* Thomson, 1895	*Hypomicrogaster* Ashmead, 1898	*Nyereria* Mason, 1981
*Hypomicrogaster* Ashmead, 1898	*Lissogaster* Bengtsson, 1926	*Ohenri* Fernandez-Triana, 2018
*Lissogaster* Bengtsson, 1926	*Mariapanteles* Whitfield & Fernandez-Triana, 2012	*Papanteles* Mason, 1981
*Microgaster* Latreille, 1804	*Microgaster* Latreille, 1804	*Parapanteles* Ashmead, 1900
*Neoclarkinella* Rema & Narendran, 1996	*Neoclarkinella* Rema & Narendran, 1996	*Parenion* Nixon, 1965
*Paroplitis* Mason, 1981	*Paroplitis* Mason, 1981	*Paroplitis* Mason, 1981
*Prasmodon* Nixon, 1965	*Prasmodon* Nixon, 1965	*Pelicope* Mason, 1981
*Pseudapanteles* Ashmead, 1898	*Pseudapanteles* Ashmead, 1898	*Philoplitis* Nixon, 1965
*Rhygoplitis* Mason, 1981	Rhygoplitis Mason, 1981	*Pholetesor* Mason, 1981
*Xanthomicrogaster* Cameron, 1911	*Shireplitis* Fernandez-Triana & Ward, 2013	*Prasmodon* Nixon, 1965
**MICROPLITINI Mason, 1981**	*Xanthomicrogaster* Cameron, 1911	*Promicrogaster* Brues & Richardson, 1913
*Alloplitis* Nixon, 1965	**MICROPLITINI Mason, 1981**	*Protapanteles* Ashmead, 1898
*Microplitis* Foerster, 1862	*Alloplitis* Nixon, 1965	*Protomicroplitis* Ashmead, 1898
*Philoplitis* Nixon, 1965	*Microplitis* Foerster, 1863	*Pseudapanteles* Ashmead, 1898
*Snellenius* Westwood, 1882	*Philoplitis* Nixon, 1965	*Pseudofornicia* van Achterberg, 2015
**FORNICIINI Mason, 1981**	*Snellenius* Westwood, 1882	*Pseudovenanides* Xiao & You, 2002
*Fornicia* Brullé, 1846	**FORNICIINI Mason, 1981**	*Qrocodiledundee* Fernandez-Triana, 2018
**SEMIONINI Tobias, 1987**	*Fornicia* Brullé, 1846	*Rasivalva* Mason, 1981
*Semionis* Nixon, 1965	*Pseudofornicia* van Achterberg, 2015	*Rhygoplitis* Mason, 1981
*Kiwigaster* Fernandez-Triana, Whitfield & Ward, 2011	**SEMIONINI Tobias, 1987**	*Sathon* Mason, 1981
	*Pelicope* Mason, 1981	*Semionis* Nixon, 1965
	*Semionis* Nixon, 1965	*Sendaphne* Nixon, 1965
	*Dodogaster* Rousse, 2013	*Shireplitis* Fernandez-Triana & Ward, 2013
	*Keylimepie* Fernandez-Triana, 2016	*Snellenius* Westwood, 1882
	*Kiwigaster* Fernandez-Triana, Whitfield & Ward, 2011	*Silvaspinosus* Fernandez-Triana, 2018
		*Tobleronius* Fernandez-Triana, 2018
		*Ungunicus* Fernandez-Triana, 2018
		*Venanides* Mason, 1981
		*Venanus* Mason, 1981
		*Wilkinsonellus* Mason, 1981
		*Xanthapanteles* Whitfield, 1995
		*Xanthomicrogaster* Cameron, 1911
		*Ypsilonigaster* Fernandez-Triana, 2018
		*Zachterbergius* Fernandez-Triana, 2018

### Brief diagnosis of all Microgastrinae genera as they are understood in this paper

The last two published keys to world genera of Microgastrinae were in Nixon (1965) and Mason (1981). Nixon (1965) recognized 19 genera in his key, whereas Mason (1981) included 50 genera (although Mason’s paper started with a key to tribes, and then genera within each tribe are keyed out and treated separately). Some regional generic keys have been published since, e.g., Tobias (1986) for the former Soviet Union, Austin and Dangerfield (1992) for the Australasian region, Whitfield (1997) for the New World, Chen and Song (2004) for China, and Kotenko (2007a) for the Russian Far East. However, with 81 genera considered in this paper, the information to recognize them in the aforementioned references is clearly outdated, and an updated key to world genera is badly needed.

Unfortunately, we still lack a robust phylogeny for the subfamily, which would be needed to provide a useful and comprehensive key. The limits of some genera at present are not well defined, and at times are contradictory; moreover, it is likely that future work will change many groups as currently understood. We anticipate that a few genera will end up as synonyms while several others, which are paraphyletic or polyphyletic as currently defined, will be split. This should likely result in an overall increase in the total number of genera as compared to present (e.g., see Fernandez-Triana and Boudreault 2018).

We divide the 81 genera recognized in this paper into four groups and characterize each group and singular genus with brief morphological diagnoses. We emphasize that these groups are not to be considered as monophyletic, and we caution that the discussion below is not to be taken as a new phylogeny for the subfamily, which is beyond the scope of the present paper. We do not present the information below as a surrogate key either; to key out Microgastrinae genera the reader is advised to initially consider the works mentioned at the beginning of this section. Our only intention here is to provide the reader with some basic information on the concepts we have followed when making decisions about generic placement of species, especially in the new combinations we propose in the checklist below. Besides comments on morphological diagnoses, we also provide illustrations for every Microgastrinae genus (at least one species per genus, usually more), the first time that has been done for the entire subfamily.

We separate Microgastrinae into four broadly defined groups:

**a) unplaced genera**, all of which have unique morphological characters that make them very distinctive, although they do not share any character in common per se, comprising 18 genera: *Austinicotesia*, *Austrocotesia*, *Beyarslania*, *Billmasonius*, *Clarkinella*, *Exulonyx*, *Fornicia*, *Janhalacaste*, *Kiwigaster*, *Mariapanteles*, *Miropotes*, *Neoclarkinella*, *Pelicope*, *Prasmodon*, *Qrocodiledundee*, *Semionis*, *Xanthomicrogaster*, and *Zachterbergius*;

**b) *Microplitis* group**, which includes the Microplitini (*sensu*Mason 1981) and four additional genera described by Fernandez-Triana and Boudreault (2018), for a total of eight genera: *Alloplitis*, *Gilbertnixonius*, *Jenopappius*, *Microplitis*, *Philoplitis*, *Silvaspinosus*, *Snellenius*, and *Tobleronius*;

**c) *Cotesia* group**, which includes most but not all of the Cotesiini (*sensu*Mason 1981), with 29 genera: *Buluka*, *Carlmuesebeckius*, *Chaoa*, *Cotesia*, *Cuneogaster*, *Deuterixys*, *Diolcogaster*, *Distatrix*, *Eripnopelta*, *Exix*, *Glyptapanteles*, *Jimwhitfieldius*, *Keylimepie*, *Larissimus*, *Lathrapanteles*, *Markshawius*, *Nyereria*, *Ohenri*, *Parenion*, *Protapanteles*, *Protomicroplitis*, *Pseudofornicia*, *Pseudovenanides*, *Rasivalva*, *Sathon*, *Ungunicus*, *Venanides*, *Venanus*, and *Wilkinsonellus*;

**d) *Apanteles* group**, which includes most but not all of the Apantelini + Microgastrini (*sensu*Mason 1981) with 26 genera: *Agupta*, *Alphomelon*, *Apanteles*, *Choeras*, *Dasylagon*, *Dodogaster*, *Dolichogenidea*, *Exoryza*, *Hygroplitis*, *Hypomicrogaster*, *Iconella*, *Illidops*, *Kotenkosius*, *Microgaster*, *Napamus*, *Papanteles*, *Parapanteles*, *Paroplitis*, *Pholetesor*, *Promicrogaster*, *Pseudapanteles*, *Rhygoplitis*, *Sendaphne*, *Shireplitis*, *Xanthapanteles*, and *Ypsilonigaster*.

**a) Unplaced genera**

*Kiwigaster* (Figs 136–137) is the only genus of Microgastrinae with sexual dimorphism in the number of antennal segments; females have 17 flagellomeres and males have 18 (Fernandez-Triana et al. 2011). All other known microgastrines have 16 flagellomeres in both sexes.

Only five genera of Microgastrinae, *Austinicotesia*, *Austrocotesia*, *Miropotes*, *Pelicope*, and *Semionis*, have hind wings without vein 2r-m (all other known Microgastrinae have that vein present, although often weakly pigmented).

*Pelicope* and *Semionis* can be recognized within this group because both have the fore wing areolet very large (while the other three genera are without an areolet or have a very small areolet). *Pelicope* (Fig. 181) has the propodeum unsculptured, notauli at least partially marked, and eyes in frontal view slightly divergent ventrally. *Semionis* (Figs 221, 222) has the propodeum with a partial transverse carina and many fine striations radiating from the nucha, the notauli not marked, and the eyes in frontal view are not divergent ventrally (Nixon 1965, Mason 1981).

*Miropotes* (Figs 157–159) differs from the other genera by the ovipositor sheaths and ovipositor with a unique shape, in most species strongly bent; eyes enlarged and strongly convergent with malar space totally or almost totally obliterated; metacoxa small and metatibial spurs very short (Fernandez-Triana et al. 2014d).

*Austinicotesia* (Figs 27, 28) and *Austrocotesia* (Figs 29–32) are similar to each other in several features (Austin and Dangerfield 1992, Fernandez-Triana and Boudreault 2018) but differ as follows: *Austinicotesia* has the fore wing without areolet (with areolet in *Austrocotesia*); fore wing with pterostigma relatively thin and long, 3.5 × as long as wide (pterostigma much less than 3.0 × as long as wide in *Austrocotesia*); fore wing vein 2RS much longer, ca. 1.5 ×, than vein r (fore wing vein 2RS much shorter, ca. 0.5 ×, than vein r in *Austrocotesia*); metafemur relatively thick and stout (of more normal proportions in *Austrocotesia*); T1 widening towards posterior margin and with strong hump followed by deeply excavated area and strong carinae (T1 more or less parallel-sided or narrowing towards posterior margin and without hump or excavate area in *Austrocotesia*); and T2 mostly smooth (usually mostly sculptured in *Austrocotesia*).

Only six genera of Microgastrinae have the propodeum mostly smooth except for complete longitudinal and transverse carinae: *Beyarslania*, *Clarkinella*, *Janhalacaste*, *Neoclarkinella*, *Mariapanteles*, and *Prasmodon*. We place them together because of the diagnostic value of that unique carination pattern, but it is clear that these genera do not constitute a monophyletic group.

*Prasmodon* (Figs 191–193) is the only genus in this subgroup with notauli strongly marked and fore wing areolet relatively large (Fernandez-Triana et al. 2014f).

*Clarkinella* and *Janhalacaste* also have a fore wing areolet (although very small, almost obliterated) and can be distinguished from each other as follows. *Clarkinella* (Figs 46, 47) has the scutellar disc with a smooth posteromedian band, T1 without a median longitudinal carina, and hypopygium mostly inflexible with only a sharp fold posteriorly (Mason 1981), whereas *Janhalacaste* (Figs 128, 129) has the scutellar disc with a coarse posteromedian band, T1 with a longitudinal sulcus on the anterior 0.6–0.7 of its length and posterior 0.3 with two short carinae centrally delimiting a slightly raised area, and hypopygium folded medially and with several pleats (Fernandez-Triana and Boudreault 2018).

*Neoclarkinella* (Figs 161–165), *Mariapanteles*, and *Beyarslania* all lack a fore wing areolet. *Neoclarkinella* can be recognized because it has a very distinctive T1 which sharply narrows towards posterior margin and has a wide depression on the anterior half, and a hypopygium with multiple pleats (Chen and Song 2004, Veena et al. 2014).

*Mariapanteles* and *Beyarslania* have the hypopygium mostly inflexible, with a posteromedian translucent fold where only a few or no pleats are visible; and T1 has a sharply defined median, longitudinal sulcus, at least on the anterior half. *Mariapanteles* (Figs 143, 144) has the ovipositor sheaths much longer (0.7 × as long as the metatibia length), and ovipositor mostly straight to slightly curved (Whitfield et al. 2012), whereas *Beyarslania* (Fig. 33) has the ovipositor sheaths relatively very short (less than 0.3 × metatibial length), and the ovipositor strongly downcurved (Mason 1981, at the time referring to the genus as *Xenogaster*). *Mariapanteles* is also the only genus in this group with the propodeum having some additional, small and short transverse carinae that radiate from the median carina (but, nevertheless, the propodeum still appears as if it is crossed by the median and transverse carinae, the defining trait of this group).

The remaining six genera in this group cannot easily be associated with any other genus and are discussed below in alphabetical order.

*Billmasonius* (Fig. 34) is recognized by T1 with a unique shape and desclerotization, with a relatively wide anterior 0.6 and very narrow posterior 0.4, so that widest part of tergite, near anterior margin, is around 4.0 × the narrowest width, along posterior 0.4, and with anterior 0.6 mostly desclerotized, only with lateral margins and narrow central strip sclerotized; T2 is also diagnostic, with a partially sclerotized area surrounding each spiracle on laterotergite 2 the same colour as T2, giving the impression of T2 having three peaks, the largest and central one being the actual T2, the two smaller lateral ones being the area surrounding the spiracles on each laterotergite (Fernandez-Triana and Boudreault 2018).

*Exulonyx* (Fig. 95) has a unique combination of features within Microgastrinae: propodeum with a partial median, longitudinal carina on anterior 0.6 and complete areola on posterior 0.4, hypopygium inflexible, ovipositor curving downwards on posterior 0.3, and T1 and T2 coarsely sculptured (Mason 1981).

*Fornicia* (Figs 96–98) is the only Microgastrinae genus with the epicnemial carina complete and the fore wing areolet absent; also, the head in lateral view is relatively small (compared to the mesosoma) (Austin and Dangerfield 1992), and T1–T3 form a carapace covering the entire dorsal surface of the metasoma. Only a few species in the *Microplitis* group (see below) have a partial to complete epicnemial carina, but all those genera have the fore wing with an areolet (usually relatively large), and the head of normal proportions.

*Qrocodiledundee* (Fig. 212) can easily be recognized by its propodeal apophysis, unique among Microgastrinae, as well as the flattened mesosoma, metafemur short and stout, pronotum dorsally enlarged, and the propodeum with a median carina and a partially defined areola (Fernandez-Triana and Boudreault 2018).

*Xanthomicrogaster* (Figs 246–249) is unique because of the following combination of features: hind wing with vein 1cu-a strongly sinuous and first submarginal cell tall (height at least 2 × its width), fore wing with a very small areolet, metacoxa very large (almost as large as the metasoma length), propodeum mostly smooth but with a strong and sharp median longitudinal carina, T1 very wide and with a strong median longitudinal sulcus, T2 rectangular and usually sculptured, hypopygium inflexible, and ovipositor sheaths relatively long (more than 0.5 × metatibia length) and with numerous setae. Some of these morphological features would suggest this genus could be placed within the *Cotesia* group, contrary to Mason’s (1981) opinion when he grouped it within his Microgastrini. However, *Xanthomicrogaster* has many other features that are so different to both our *Cotesia* group and Mason’s Microgastrini that we prefer to maintain it as an unplaced genus.

*Zachterbergius* (Figs 253, 254) has the longest and thinnest T2 among all known Microgastrinae, with T2 length 4.0 × its width at base and apex, 0.7–0.8 × as long as T1 length, and around 1.5× as long as T3 length. Also, the propodeum has a clearly defined median carina, partially defined transverse carina, and the posterior part of an areola; the antennal scape is very transverse, and the labial palpi are very long, extending to the mesopleuron (Fernandez-Triana and Boudreault 2018).

**b) *Microplitis* group**

This is one of the best-defined groups of genera within Microgastrinae (see Mason 1918), and most likely to be monophyletic. It is characterized by: tentorial pits relatively large, head mostly coarsely sculptured, stemmaticum usually very well defined and slightly to strongly raised from the surrounding areas, anteromesoscutum and scutellar disc usually coarsely sculptured, notauli almost always defined (often very clearly), propodeum always sculptured and with several strongly defined carinae, fore wing with areolet usually large, metacoxa relatively small, metatibial spurs short, T1 with median longitudinal sulcus, hypopygium inflexible and almost always relatively short, ovipositor sheaths with few setae that are mostly limited to the apex, and ovipositor almost always very short (much shorter than 0.5 × metatibia length).

*Philoplitis* (Figs 182, 183) has a unique combination of features including an enormous scutellum conically prolonged posteriorly over the propodeum (Mason 1981, Fernandez-Triana and Goulet 2009, Ranjith et al. 2019). It also has an occipital carina, and ocelli forming a very low triangle, to the point that the anterior ocellus seems almost on the same line as the posterior ones.

*Silvaspinosus* (Fig. 227) has the clypeus extremely long and thin, the malar line extremely short (almost absent), the mandible base separated from the rest of the head by a desclerotized area that looks almost like an opening, and mandibles relatively stout and large. The shape of the clypeus, and the separation of the mandible from the rest of the head by a desclerotized area are unique among Microgastrinae (Fernandez-Triana and Boudreault 2018). It also has the fore tarsus with a spine-like seta, and the scutellar disc with the posteromedian band smooth; both of which are unique and distinctive among the *Microplitis* group.

*Gilbertnixonius* (Fig. 99), is the only genus in this group that has the propodeum with both longitudinal and transverse carinae but without an areola (*Alloplitis* and *Tobleronius* have those carinae, although sometimes incomplete, but they also have a complete areola on the propodeum). *Gilbertnixonius* also has an epicnemial carina (otherwise only present in some species of *Snellenius* and in all species of the unrelated genus *Fornicia*) and an incomplete occipital carina (otherwise only present in *Alloplitis*, *Philoplitis*, and *Tobleronius*) (Fernandez-Triana and Boudreault 2018).

*Alloplitis* and *Tobleronius* are somewhat similar morphologically and distinguished from the other six genera in this group by the propodeum with a complete areola (in addition to partial longitudinal and transverse carinae). *Alloplitis* (Figs 7, 8) has T1 more or less parallel-sided or slightly widening towards the posterior margin, and T2 more or less rectangular; whereas *Tobleronius* (Fig. 233) has T1 strongly narrowing towards the posterior margin (width at posterior margin 0.3 × or less of width at anterior margin) and T2 very long and thin (although slightly widening towards the posterior margin) and with the area surrounding the spiracles on laterotergite 2 partially sclerotized and the same colour as T2 giving the impression of T2 having three peaks, the largest and central one being the actual T2, the two smaller lateral ones being the area surrounding the spiracle on each laterotergite (Fernandez-Triana and Boudreault 2018).

*Microplitis* (Figs 151–156) and *Snellenius* (Figs 228–232) are very similar and form one of the most morphologically distinct groups of Microgastrinae (Nixon 1965, Mason 1981, Walker et al. 1990, Shaw and Huddleston 1991, Austin and Dangerfield 1993, Fernandez-Triana et al. 2015b) with the following shared diagnostic features: propodeum with coarse sculpturing and a strong median carina and T2 and T3 with a poorly defined separation between them. Most species of *Snellenius* are easily distinguished by having the notauli and the scutellar disc strongly excavated and sculptured, and by having the scutoscutellar sulcus very wide and deep; both cases represent the most extreme examples within Microgastrinae. Additionally, the propodeum is divided into two distinct areas (faces) clearly marked by a strong angulation (observed in lateral view) and a transverse carina (observed in dorsal view). The main difficulty when trying to distinguish both genera is that those features appear to grade, from strongly excavated and sculptured notauli and scutellar disc (most *Snellenius*) to less excavated and less sculptured (a few *Snellenius*, most *Microplitis*), to basically smooth and unexcavated (some *Microplitis*). The only reliable feature to separate the two genera is the presence of an epicnemial carina in *Snellenius*, which is absent in *Microplitis* (Mason 1981, Austin and Dangerfield 1992, 1993, Fernandez-Triana et al. 2015b), although in practice it may be difficult to distinguish the epicnemial carina due to setae and/or sculpture on the epicnemium and mesopleuron.

*Jenopappius* (Figs 130–131) resembles *Microplitis* but with T2 strongly sculptured and rectangular, and T1 mostly sculptured and with a median depression anteriorly. Some *Alloplitis* may also have a somewhat similar sculpture on either T1 or T2 but the shape of those tergites is very different, and *Alloplitis* always has the propodeum with a complete areola, defined by strongly raised carinae. The combination of the sculptured propodeum without an areola, T1 with an anteromedian depression, and T1 and T2 with strong sculpture are very unusual and will separate *Jenopappius* from any other genus of Microgastrinae (Fernandez-Triana and Boudreault 2018).

**c) *Cotesia* group**

We place here genera with a completely inflexible hypopygium, ovipositor sheaths relatively short (less than 0.5 × metatibial length, usually much less) and mostly without setae (except apically in some cases). Most of the 29 genera considered here also have the propodeum without a complete areola (although some have it, and others have a complex arrangement of carinae and sculpture where a partial to complete areola can sometimes be defined). Although these features work well to recognize most members of the group, a few species of *Sathon*, *Lathrapanteles*, *Glyptapanteles*, and *Ohenri* have relatively long ovipositor sheaths, but in these cases the hypopygium is still always inflexible. Most or perhaps all the species within the *Cotesia* group posses a suite of characters indicative of parasitism of “macrolepidoptera” (*sensu*Mason 1981: 25), but the group is probably not monophyletic. From the Cotesiini (*sensu*Mason 1981) we exclude here *Parapanteles* and instead transfer it to the *Apanteles* group (see details under that group); the main reason being that this genus, as it had been understood, apparently includes two different sets of taxa: one that seems to be *Cotesia* species misidentified as *Parapanteles* (Valerio et al. 2009, Parks 2018, Freitas et al. 2019), and another (representing the majority of the genus, as currently understood, including the type species) that are more related to *Dolichogenidea* and *Apanteles* than to any genus in the *Cotesia* group. We also add here *Sathon*, which we consider to be closer to *Glyptapanteles* and related genera, unlike Mason (1981), who considered it to be part of his Microgastrini group.

The *Cotesia* group can be broadly split into two subgroups, based on whether the fore wing has an areolet (*Buluka*, *Cuneogaster*, *Diolcogaster*, *Eripnopelta*, *Exix*, *Jimwhitfieldius*, *Keylimepie*, *Larissimus*, *Markshawius*, *Parenion*, *Protomicroplitis*, *Rasivalva*, *Ungunicus*, *Venanus*) or does not have an areolet (*Carlmuesebeckius*, *Chaoa*, *Cotesia*, *Deuterixys*, *Distatrix*, *Glyptapanteles*, *Lathrapanteles*, *Nyereria*, *Ohenri*, *Protapanteles*, *Pseudofornicia*, *Pseudovenanides*, *Sathon*, *Venanides*, *Wilkinsonellus*).

Among the genera with a fore wing areolet, *Jimwhitfieldius* (Figs 132, 133) has the metatrochantellus with a unique shape (Fig. 133), the head with a strong depression behind the occiput, the metatibia with a very long and thick inner spur, and the ovipositor and ovipositor sheaths extremely short, probably the shortest in the entire subfamily (Fernandez-Triana and Boudreault 2018).

*Venanus* (Figs 237–240) is quite distinctive, and comprises small species, often with the body slightly depressed, face with a triangular flange between the antennal sockets, fore wing with a relatively large areolet, T2 with strongly defined lateral sulci, and ovipositor sheaths with very few and minute setae (Mason 1981).

The remaining genera in the subgroup seem to share one or several morphological features with *Diolcogaster* (whether those features are homoplastic or not). *Diolcogaster* (Figs 66–77), as currently understood, is most likely a polyphyletic genus that will need to be split into several genera. Until then, it is difficult to define unequivocally. Instead, we discuss the remaining genera in this subgroup in alphabetical order, with the features that distinguish them from *Diolcogaster*.

*Buluka* (Figs 35–37) has T1–T3 forming a carapace and occupying the entire dorsal surface of the metasoma, the fore wing has a complete areolet, and females have part of the ventral surface of the distal six or seven flagellomeres without longitudinal placodes, instead having an oblique groove bounded on one side by a row of bent-tipped sensilla (Austin 1989). The carapace is shared with *Fornicia* and very few species of other genera, e.g., *Deuterixys*, *Pholetesor*, none of which have a fore wing areolet. The *basimacula* species group of *Diolcogaster* (*sensu*Saeed et al. 1999) have both the carapace and areolet, but the antenna does not have the special groove and sensilla.

*Cuneogaster* (Figs 61, 62) resembles *Diolcogaster* but it has the glossa long and apically bilobed, T1 wedge-shaped, and the scutellar disc with the medioposterior band smooth (Choi and Whitfield 2006) whereas in *Diolcogaster* the glossa is not elongated, T1 is usually not wedge-shaped, and the scutellar disc has a medioposterior band of rugosity in most species.

*Eripnopelta* (Figs 87, 88) could be considered an atypical *Diolcogaster*, but the pronotal lateral surface does not have distinct furrows, the scutellar disc has a smooth and protruding medioposterior band, T1 does not have a distinct median groove on the basal half, and the fore wing areolet is very small, almost obliterated (Xiong et al. 2017).

*Exix* (Figs 89, 90) also seems morphologically related to *Diolcogaster*, but it is defined by T2 large and smooth, without submedian grooves, the hind wing has the vannal lobe concave and lacking setae, and the hind wing nervellus is externally concave (Mason 1981).

*Keylimepie* (Figs 134, 135) can be recognized by the reduced wings in females, relatively small eyes and long malar space. The shape and sculpture of the head, mesosoma sculpture, shape and sculpture of T2, and ovipositor are all similar to some *Diolcogaster*, but *Keylimepie* has a T1 without a median sulcus and instead it has the anterior 0.5 rather depressed and concave, and the posterior 0.5 with strong transversal striations (Fernandez-Triana and Boudreault 2016).

*Larissimus* (Figs 139, 140) is another genus related to *Diolcogaster* but it can be recognized by the greatly reduced vannal lobe in the hind wing with, almost entirely smooth body, and the only described species is the largest known species of Microgastrinae, with a body and fore wing length of 7–8 mm (Nixon 1965, Mason 1981).

*Markshawius* (Figs 145, 146) has a unique set of features (Fernandez-Triana and Boudreault 2018) which together are very distinctive (although some, but not all, are shared with other genera). The female head is elongated and strongly concave posteriorly, modified to be tightly appressed to the anterior margin of the pronotum (following its contour); the face has its upper margin produced dorsally between the antennal insertions into a triangular flange; the frons is very elongated, with ocelli clearly much higher than normal; the antenna is very short (much shorter than body length, usually shorter than the combined length of the head and mesosoma), with all flagellomeres except the first having a single row of placodes; the propodeum has a median carina (defined posteriorly) and transverse rugosity which includes a poorly and partially defined transverse carina; and T1 is either extremely long and thin, with length at least 6.0× its width centrally, or very thin on the anterior 0.3–0.4, then strongly widening posteriorly, its width at the posterior margin around 3.0 × its width centrally.

*Parenion* (Figs 176, 177) can only be confused with some *Diolcogaster*, but is distinguished by having T2 and T3 smooth and barely or not separated, scutellar disc with the medioposterior band smooth and very small lunules on its lateral surface (Mason 1981).

*Protomicroplitis* (Figs 201, 202) is closely related to *Diolcogaster*, both morphologically and molecularly, and some of the criteria used to define it may need revision. The genus is defined by some flagellomeres having three rows of placodes, relatively large fore wing areolet, and T1 very long and narrow (Mason 1981, Fernandez-Triana 2015), although the last two features are also present in a few *Diolcogaster* species.

*Rasivalva* (Figs 213, 214) is characterized by the ovipositor sheaths lacking setae, or with very few and minute setae (Mason 1981, Chen and Song 2004, Kotenko 2007b). This separates it from *Diolcogaster*, which has relatively long setae on the ovipositor sheaths, including a few strong and thickened setae in many species. Other distinguishing features that appear in some species are the scutellar disc with the medioposterior band smooth, body sculpture smoother overall than in *Diolcogaster*, and propodeum with a median, longitudinal carina that is sometimes reduced or absent.

*Ungunicus* (Fig. 234) has remarkable and very distinctive tarsal claws, with a very large basal tooth longer than the apex of the tarsal claw, and a median lobe with setae arising from its margin, which seems slightly bilobate. These claws are unique within Microgastrinae (Fernandez-Triana and Boudreault 2018).

Among the genera without the fore wing areolet, *Chaoa* (Fig. 39) was described from a single specimen (Luo et al. 2004), with little information provided. Based on the original description and illustrations of the holotype, this genus might just represent a species of *Glyptapanteles*, or perhaps *Nyereria* but without examining the type we cannot conclude and therefore retain it as a valid genus for the time being.

*Carlmuesebeckius* (Fig. 38) has the ovipositor and ovipositor sheaths relatively long, and the propodeum with a complete areola, unlike most other genera in this subgroup. Other unique features are T1 with a strong and raised median carina for most of its length, and the ovipositor bulging near apex and with two subapical serrate teeth on the lower (first) valvulae (Fernandez-Triana and Boudreault 2018).

*Cotesia* (Figs 48–60) is a relatively uniform genus morphologically, long considered the easiest group to recognize among all segregates from *Apantelessensu lato* (Mason 1981: 113). Defining characters are: fore wing without areolet; T1 and T2 usually mostly to entirely sculptured, T3 also often at least partially sculptured or, more rarely, completely sculptured; T1 either widening towards its posterior margin (very often), more or less parallel-sided or barrel-shaped (often), slightly widening towards the posterior 0.7–0.8 of the tergite length and from that point slightly narrowing towards the posterior margin which is more or less rounded (rarely), or medially constricted (extremely rare), but never completely narrowing towards the posterior margin; ovipositor and ovipositor sheaths are very short to short, very rarely moderately long. The propodeum varies considerably but has a well defined median longitudinal carina (very often), although the median carina may be difficult to distinguish on its own in species with the propodeum strongly sculptured with an irregular pattern of carinae (often), or the median carina may be partially absent (rarely), or the median carina may be combined with a partial to complete areola partially defined by a transverse carina (rarely), or the median carina is absent and/or the propodeal surface is shiny overall and almost without any sculpture (rarely). The only other genus that could be confused here would be *Protapanteles*, which may eventually be considered as just a species group within *Cotesia*, with smoother propodeum and T1–T3.

*Protapanteles* (Figs 198–200) usually has T1 either slightly widening towards the posterior 0.7–0.8 of the tergite length and then slightly narrowing towards the posterior margin which is more or less rounded (often), more or less parallel-sided or barrel-shaped (rarely), or slightly widening towards the posterior margin (rarely). The propodeum is variously sculptured, usually having a median longitudinal carina that may be partially or completely defined, and rarely lacking the median carina. A character commonly used to define this genus, a modified spine on the fore tarsus (Nixon 1965, 1972, 1973, 1976, Mason 1981), is present in some species of many related genera, e.g., *Cotesia*, *Glyptapanteles*, *Distatrix*, *Nyereria*, and even in some non-related genera such as *Silvaspinosus*, and thus does not have the same diagnostic value as expressed by Mason (1981). Some species may be considered as borderline between *Cotesia* and *Protapanteles*, and others may be considered as borderline between *Glyptapanteles* and *Protapanteles*; thus, it is difficult to clearly define these three genera. Differences between *Protapanteles* and *Cotesia* were given in the previous paragraph. Differences with *Glyptapanteles* are mostly related to the shape of T1. In *Glyptapanteles*, T1 is either parallel-sided anteriorly and then strongly narrowing posteriorly, or its sides are gradually to strongly converging posteriorly when compared to *Protapanteles* which has T1 parallel-sided throughout, except for a strongly rounded apex, and propodeum sculpture that is usually, but not always, more rugose and carinated than in *Glyptapanteles*. Additionally, *Protapanteles* larvae have mandibles with a row of 12 or fewer large teeth concentrated distally on the blade, and its species distribution is almost completely confined to the Holarctic region (Mason 1981). However, the morphological features mentioned above vary considerably among different species (Arias-Penna et al. 2019).

*Glyptapanteles* (Figs 100–110) is most likely a polyphyletic assemblage, and may eventually be split into several genera. As a result, it is difficult to define (Arias-Penna et al. 2019). Some of its species may be confused with *Protapanteles*, *Sathon*, *Lathrapanteles* and, to a lesser extent, also *Distatrix*, *Venanides*, and *Nyereria*. The main features defining *Glyptapanteles* are: fore wing without an areolet; propodeum that is either completely smooth (often) to more or less rugose (more rarely), with a median longitudinal carina that is entirely absent (often), partially defined posteriorly (often) to complete and strong (rarely), or no median carina but instead a series of very short carinae radiating from the nucha (rarely); T1 narrows towards the posterior margin, usually strongly (almost always), or more parallel-sided, or rounded at apex, as in some species of *Protapanteles* (rarely); T2 is almost always subtriangular or trapezoidal (rarely shaped differently); ovipositor and ovipositor sheaths are relatively short (usually) to moderately long (rarely); setae at apex of ovipositor sheaths relatively long (as long or longer than setae on hypopygium). The differences from *Protapanteles* were given in the previous paragraph. *Sathon* has the ovipositor sheaths longer and male specimens have enlarged external genitalia; however, a few *Glyptapanteles* species have females with longer ovipositor sheaths, and a very few other species have males with external genitalia similarly enlarged; whether those species should be transferred to *Sathon* requires further study. *Lathrapanteles* has similar characters to *Sathon* (see more about those two genera below) and can be separated in the same manner from *Glyptapanteles*. *Distatrix* has the pronotum with only one furrow laterally, eyes enlarged and ovipositor sheaths without setae or with very few minute setae, whereas *Glyptapanteles* has the pronotum with two furrows, eyes that are almost never enlarged (but see Fernandez-Triana 2018, for one exception) and the ovipositor sheaths have much longer setae. *Venanides* can in turn be separated from *Glyptapanteles* based on having similar ovipositor sheaths to *Distatrix* (Mason 1981).

*Distatrix* (Figs 78, 79) is similar to *Venanides*, but it has two rows of placodes in the flagellomeres in females, and T2 has a characteristic shape, with the lateral margins widely diverging (Mason 1981, Grinter et al. 2009).

*Venanides* (Figs 235, 236) can be differentiated from *Distatrix* because it has only a single row of placodes in the flagellomeres in females, and T2 has less diverging lateral margins (Mason 1981). Additionally, *Venanides* specimens tend to be smaller and have a dorsoventrally compressed body that is also generally mostly smooth and shiny.

*Sathon* (Figs 218–220) is distinguished mainly by the enlarged external genitalia in males and relatively long ovipositor sheaths in females; some species probably have the longest sheaths among the entire Cotesiini (*sensu*Mason 1981). However, these features are not unique: a few *Glyptapanteles* species have similarly enlarged male genitalia, and all described *Lathrapanteles* species (Figs 141, 142) are also very similar to *Sathon* (e.g., Williams 1985, 1988). The limits of *Lathrapanteles* and *Sathon* need revision and it is possible that one will eventually be placed in synonymy with the other.

*Deuterixys* (Figs 64, 65) is a very distinctive genus on account of its T1–T3 sculpture and shape (there appears to be a second constriction between T2 and T3), the propodeum being smooth and shiny and with a complete and strong median, longitudinal carina, and the relatively small body length (Mason 1981, Whitfield 1985, Zeng et al. 2011).

*Nyereria* (Figs 166–169) has T2 divided into three sections by two deep, usually crenulated, longitudinal grooves delimiting a raised, median area that is not wider than long (Mason 1981). This genus can only be confused with a few species of *Cotesia* and *Glyptapanteles* that have their T2 with a similar raised, median area, although in those cases T2 is never as strongly defined by grooves.

*Pseudovenanides* (Fig. 211) has very scarce information available, but from the original description (Xiao and You 2002) it is clear that it is related to *Glyptapanteles* and, to a lesser extent, to *Venanides*. Apparently, T1 with a strongly marked longitudinal sulcus on most of the tergite is the defining feature of this genus.

*Ohenri* (Fig. 170) has many unique features and is only tentatively considered to be part of this subgroup lacking the fore wing areolet. The pronotum is considerably enlarged dorsally, the ovipositor has its lower valvulae with four subapical teeth, the tarsal claws have large teeth, and the propodeum has a median carina with a partially defined areola (Fernandez-Triana and Boudreault 2018).

*Pseudofornicia* (Figs 208–210) superficially resembles the (probably) unrelated *Fornicia* because its metasoma mostly forms a dorsal carapace, but it differs in lacking the epicnemial carina, the fore wing does not have an areolet, and T1 is movably joined to T2, whereas *Fornicia* has an epicnemial carina, fore wing with an areolet, and T1 and T2 are immovably joined (van Achterberg et al. 2015).

*Wilkinsonellus* (Figs 241–244) is a very recognizable genus, with T1 very long and thin, propodeum with distinctive sculpture and carination pattern, and fore wing with veins r and 2RS strongly angled (Mason 1981, Long & van Achterberg 2011, Arias-Penna et al. 2013, 2014).

**d) *Apanteles* group**

Mason (1981) proposed the tribes Apantelini and Microgastrini to accommodate species with ovipositor sheaths mostly setose and relatively long (at least 0.5 × metatibial length), hypopygium with ventral margin usually flexible and either with one (rarely) or several (commonly) pleats. The latter is the most diagnostic feature for this group; however, there are exceptions (all *Alphomelon*, most *Hygroplitis*, and a few species of *Apanteles* and *Microgaster*) where the hypopygium is mostly to entirely inflexible. In this paper we combine most of the genera included in the two tribes into a single *Apanteles* group composed of 26 genera. The group is clearly not monophyletic. Most, if not all, of the species included here have the “microlepidoptera suite of characters” *sensu* Mason (see further discussion in Mason 1981, Walker et al. 1990). Here we separate the group into several subgroups that can be recognized on simple morphological features, although the genera included in each subgroup are not necessarily related.

The largest subgroup includes 13 genera that lack a fore wing areolet: *Alphomelon*, *Apanteles*, *Dolichogenidea*, *Exoryza*, *Iconella*, *Illidops*, *Napamus*, *Parapanteles*, *Pholetesor*, *Pseudapanteles*, *Rhygoplitis*, *Shireplitis*, and *Xanthapanteles*. Another two genera could be placed here, at least partially: some species of *Choeras* lack a fore wing areolet; however, most of the species have a complete or partial areolet so we consider *Choeras* to be better placed with the subgroup of genera with a complete or partial fore wing areolet; and a similar situation occurs with *Promicrogaster*, where smaller species tend to lack the areolet whereas the larger species have a complete areolet, and we similarly place that genus in the subgroup with an areolet. These two genera exemplify the challenges of delimiting precise groups in Microgastrinae (a frustration also shared by Mason 1981: 77).

Among the genera without a fore wing areolet, four have the propodeum either with a median longitudinal carina (*Iconella*, *Pseudapanteles*, *Rhygoplitis*) or with a complex pattern that includes full sculpturing and a series of short carinae radiating medially on the posterior 0.2–0.3 near the nucha (*Illidops*). A fifth genus, *Napamus*, could also be included in this subgroup, as one of its two described species has the propodeum with a median, longitudinal carina; however, the other species does not (Papp 1993: 170). Nevertheless, *Napamus* (Fig. 160) can be characterized by its mouth parts elongate, fore wing vein R1 very short (shorter than pterostigma length), inner metatibial spur much longer (1.3 ×) than the outer spur, body and legs black, and wings strongly infumate.

*Iconella* (Figs 122–124) was described by Mason (1981) as a new genus based on the hind wing with a sinuous vein cu-a as a plesiomorphic character that suggests its unique status among similar genera. Fernandez-Triana et al. (2013a, 2014e) also considered the presence of a median longitudinal carina on the propodeum as strong support for its generic status. However, some Oriental species (with large body size and large and bilobate glossae) currently assigned to *Iconella* may eventually be placed in a different genus.

*Illidops* (Figs 125–127) includes species that have the scutellar disc with a medioposterior band of rugosity, fore wing vein R1 shortened, and the propodeum with a series of short carinae medially on its posterior 0.2–0.3, near the nucha (Fernandez-Triana et al. 2014e). In some, but not all species the lower margins of the eyes converge, and T3–T7 are weakly sclerotized (Mason 1981).

*Pseudapanteles* (Figs 203–207) is characterized by the glossa elongate and strongly bilobed apically, propodeum with a strongly defined median longitudinal carina but no transverse carina (traces of a transverse carina are very rarely present in a few Neotropical species), and T1 with a sharp median sulcus (Mason 1981, Whitfield 1997, Fernandez-Triana et al. 2014a).

*Rhygoplitis* (Figs 215–217) is the only genus in this subgroup with notauli relatively well defined. It also has the propodeum coarsely sculptured (in addition to a median, longitudinal carina), and fore wing with very short vein R1 (Mason 1981, Whitfield 1997).

The other eight genera without a fore wing areolet have the propodeum with a complete to partial areola, although in large genera such as *Apanteles*, *Dolichogenidea*, and *Pholetesor*, some species have lost all carinae and the propodeum is mostly smooth.

*Shireplitis* (Figs 225, 226) has the propodeum entirely sculptured, without median or transverse carina, but with the areola defined on the posterior 0.5 by two lateral carinae, ovipositor sheaths relatively short (0.4–0.5 × metatibia length), and legs short and robust – with the metafemur usually less than 3.0 × as long as wide (Fernandez-Triana et al. 2013b).

*Alphomelon* (Figs 9–11) has the gena with a white/pale spot that is relatively large and very distinctive (Mason 1981, Deans et al. 2003). A few other Microgastrinae genera have some species with a similar pale spot, but it is usually much smaller. *Alphomelon* is distinguished from the other Microgastrinae with white/pale spot on gena by its ovipositor sheaths being relatively long (much shorter in *Cotesia*, *Glyptapanteles*, *Protapanteles*), mesoscutum anteriorly without strong notauli (strong notauli in *Prasmodon*), propodeum without a median, longitudinal carina (strong median, longitudinal carina in *Pseudapanteles*), and the hypopygium inflexible and unpleated (almost always flexible and with several pleats in *Apanteles*).

*Apanteles* (Figs 12–26) is currently the most speciose genus in Microgastrinae and has some morphological variability. It usually has the propodeum fully to partially areolated, rarely smooth and never with a median longitudinal carina; fore wing without an areolet; hind wing with the vannal lobe usually strongly concave or straight (see next paragraph for more details on that); ovipositor sheaths relatively long; and the hypopygium almost always flexible and pleated. This genus could only be confused with *Pholetesor* or *Dolichogenidea* (which seem to be related to one another, see below) and *Parapanteles*. Most *Apanteles* species can be distinguished from both *Pholetesor* and *Parapanteles* by the flexible, pleated hypopygium and relatively long ovipositor sheaths (usually at least 0.5 × length of metatibia). In contrast, *Parapanteles* and *Pholetesor* have the hypopygium either entirely inflexible or at most with a small, translucent area near the posterior margin (which may look like a pleat in a few species); and the ovipositor sheaths are relatively short (less than half the metatibia length, usually much less). However, a few species of *Apanteles* have relatively short ovipositor sheaths, and very few species may even have an inflexible hypopygium (e.g., Fernandez-Triana et al. 2014e); the generic placement of those species may be revisited, but at present those exceptions make for a more difficult separation of these three genera.

*Dolichogenidea* (Figs 81–86) is even more difficult to distinguish from *Apanteles*, as there is some overlap in some species groups of both genera (e.g., Mason 1981: 53, 54). The differences are frequently subtle and, at times it is very difficult to assign a species to one or other genus depending on the interpretation of morphological features alone. *Apanteles* has the hind wing with the vannal lobe usually strongly concave or, more rarely, straight to very slightly convex; the central part of the vannal lobe lacks any setae or has few, sparse setae that are often minute and not continuous. In contrast, *Dolichogenidea* has the vannal lobe convex to slightly straight; the central part of the vannal lobe is more or less entirely setose so that a continuous fringe of setae is almost always visible (although setae may be small in a few species). The fringe of setae (or lack of them) is the only morphological character that almost always seems to work in distinguishing these genera from each other; we are aware of very few species currently assigned to *Dolichogenidea* where the fringe is not complete and could lead to the species being placed within *Apanteles*, despite molecular data strongly suggesting the best generic placement is *Dolichogenidea*. Other features function only partially and seem to represent trends that are far from being universally present in one genus or the other. For example, the anteromesoscutum punctures (when present) tend to be partially or completely fused near the scutoscutellar sulcus in *Apanteles*, whereas in *Dolichogenidea*, which usually does not have punctures on the anteromesoscutum anteriorly and very rarely has them near scutoscutellar sulcus, the punctures never fuse. The scutoscutellar sulcus in many *Dolichogenidea* species tends to be very narrow and sometimes looks almost obliterated, whereas the sulcus in *Apanteles* is usually wider. Despite the rather subtle morphological differences, DNA barcodes tend to cluster both genera clearly apart (Smith et al. 2013, Fernandez-Triana et al. 2014e).

*Dolichogenidea* tends to cluster near *Pholetesor* (Figs 184–190) and these genera seem to be closer to each other than either is to *Apanteles*. *Dolichogenidea* has a flexible, pleated hypopygium and relatively long ovipositor sheaths (usually at least 0.5 × metatibia length) whereas *Pholetesor* has the hypopygium entirely inflexible or with a small, translucent area near the posterior margin that could look like a pleat in a few species, and the ovipositor sheaths are relatively short, less than half the metatibia length (Mason 1981, Whitfield 2006).

The status of *Exoryza* (Figs 92–94) as a valid genus has been questioned by many authors (Valerio et al. 2004, Rousse and Gupta 2013, Fernandez-Triana et al. 2014e, 2016c). Mason (1981) characterized it as having T1 and T2 heavily sculptured, and the propodeum coarsely rugose, with an areola present but obscured by heavy sculpture. However, the distinction between *Exoryza* and *Dolichogenidea* may be particularly difficult because many species of the latter genus have the propodeum sculptured, with or without an areola, and T1 is occasionally sculptured, although not as strongly as in *Exoryza* (Fernandez-Triana et al. 2014e, 2016c).

*Parapanteles* (Figs 172–175) is a very difficult genus to understand at present. Parks (2018) found it to be paraphyletic. Some species of “*Parapanteles*” with available DNA barcodes cluster within *Dolichogenidea* and could just be considered as species within that genus, with short ovipositor sheaths and an inflexible hypopygium (similar to *Pholetesor* and the few borderline species of *Apanteles* mentioned above). Another group of *Parapanteles* seems to represent misidentifications of *Cotesia* (e.g., Valerio et al. 2009, Freitas et al. 2019). Whether a group of species that could be considered true *Parapanteles* actually exists remains to be seen. For the present, the genus can be defined as having the propodeum completely to mostly areolated (usually with well defined carinae), ovipositor sheaths short, and an inflexible hypopygium.

*Xanthapanteles* (Fig. 245) is a very distinctive genus, on the basis of the propodeum fully areolated with strongly defined and raised carinae, T1 very large and wide, T1–T3 sculpture like a finely pebble-grained surface (unlike any other Microgastrinae), flagellomere placodes arranged irregularly and fore wing relatively slender and much longer than body length (Whitfield 1995b).

Another subgroup within the *Apanteles* group includes six genera, *Agupta*, *Dasylagon*, *Hypomicrogaster*, *Papanteles*, *Promicrogaster*, and *Sendaphne*, that can be recognized by the fore wing with a very small areolet, sometimes almost obliterated. They also share (except for *Agupta*, see below) having the scutellum with lunules relatively high, more than 0.5 × the height of its lateral face. These genera are separated from each other based on different propodeal carination patterns, and T1 and T2 shapes and sculptures. Some described species of *Choeras*, almost exclusively from the Oriental region, have a very small areolet and thus could be included in this group. However, these are exceptions and are very likely to be transferred elsewhere or classified separately. For now, we place *Choeras* (see below) within the subgroup with a large fore wing areolet.

*Agupta* (Figs 5, 6) does not have enlarged lunules; however, it can be recognized by several unusual features: in males (and sometimes in females) the antenna has the first few flagellomeres with placodes irregularly distributed in three rows or no row can be clearly defined; the propodeum has a strongly raised median carina with small radiating carinae across its length; T1 shape (narrowing for first half, then parallel-sided) and T1 sculpture (anterior half mostly smooth, strongly concave and with central sulcus, posterior half punctured and with a polished area on posterior margin) are distinctive; and the body length is among the largest in Microgastrinae (second only to the unrelated genus *Larissimus*) (Fernandez-Triana and Boudreault 2018). Some large specimens of *Choeras* in the Oriental region (see previous paragraph) might end being placed within *Agupta* when more studies are done in the future.

*Promicrogaster* and *Sendaphne* can be recognized by the following combination of features: glossa elongate and bilobate, metacoxa very long (0.8–1.0 × metafemur length and 0.6–0.8 × metatibia length), and ovipositor and ovipositor sheaths very long – among the longest in Microgastrinae usually 2.0 × as long as the metatibia or even longer. Most species have the body length longer than the fore wing length, usually by 0.2–0.4 mm (the majority of Microgastrinae species have the fore wing slightly longer than the body length). These two genera are very closely related and may eventually be treated as a single genus. *Promicrogaster* (Figs 194–197) has the ovipositor apically sinuate; propodeum sculptured and usually with some carination (which may include a complete or partial median longitudinal carina, or an indication of a partial areola posteriorly); T1 parallel-sided to slightly narrowing towards the posterior margin; and T2 transverse, its width at the posterior margin 3.0–4.5 × (rarely 2.0 ×) its length medially (Fernandez-Triana et al. 2016b, Fernandez-Triana 2019). *Sendaphne* (Figs 223, 224) has the ovipositor straight apically, propodeum mostly smooth and without carina (with the rare exception of having sparse punctures and a few rugae near the nucha), T1 strongly narrowing towards the posterior margin, and T2 subtriangular (Fernandez-Triana et al. 2014h).

*Dasylagon* (Fig. 63) has the propodeum fully areolated (defined by strong carinae), T1 comparatively very wide and large (in dorsal view more than 0.3 of entire metasoma), T2 very transverse, metasomal terga entirely smooth, hind wing with a sinuous vein cu-a, and ovipositor and ovipositor sheaths relatively very long (more than 1.5 × metatibia length) (Mason 1981).

*Hypomicrogaster* (Figs 113–121) has the propodeum with a complex carination pattern, which includes a median carina and a more or less complete areola, although some species have all carinae reduced, but still the propodeum would be mostly sculptured. The head is relatively transverse, i.e., wider than in most other genera of Microgastrinae, and T1 and T2 are mostly to entirely smooth (Mason 1981, Valerio and Whitfield 2015).

*Papanteles* (Fig. 171) has the propodeum fully areolated, T1 relatively long (ca. 2.0 × its width at posterior margin), T1 and T2 strongly sculptured, T2 and T3 comparatively narrow and not occupying the entire dorsal surface of the segment (dorsal width of T2 and T3 half the width of T5 and following terga), and the ovipositor sheaths are approximately the same length as the metatibia length (Mason 1981).

The remaining eight genera in the *Apanteles* group all have the fore wing areolet relatively large; even when some species may have a relatively smaller areolet, it never appears almost obliterated.

*Ypsilonigaster* (Figs 250–252) has a very characteristic T1, with a median sulcus shaped like an inverted Y, a unique feature to recognize the genus (Fernandez-Triana and Boudreault 2018).

*Hygroplitis* and *Microgaster* have the propodeum with a median carina, fore wing areolet relatively large, anteromesoscutum anteriorly mostly smooth, T1 and T2 heavily sculptured (also T3, partially or entirely), T1 relatively large and wide (width at posterior margin greater than width at anterior margin), and T2 mostly rectangular. The two genera are very closely related and DNA barcodes suggest *Hygroplitis* may eventually be synonymized under *Microgaster*. *Hygroplitis* (Figs 111, 112) has the body somewhat depressed dorsoventrally, notauli more strongly impressed, flagellomeres with three rows of placodes, and the hypopygium usually inflexible although in some cases it is weakly but distinctly pleated (Mason 1981); whereas *Microgaster* (Figs 147–150) does not have the body dorsoventrally depressed, the notauli are barely visible, flagellomeres are usually (but not always) with two rows of placodes, and the hypopygium is usually (but not always) flexible and pleated (Mason 1981).

*Paroplitis* (Figs 178–180) species are relatively small, with a body length of 2.5 mm or less; legs, especially the metafemur, short and robust; antenna short, with flagellomeres in females having only a single row of placodes; hypopygium almost entirely sclerotized but with a sharp fold medially; propodeum rarely entirely sculptured but almost always with a median longitudinal carina, at least on the anterior 0.5, and sometimes also with a complete or partial transverse carina; and T2 usually smooth, rarely sculptured (Mason 1981, Fernandez-Triana et al. 2013b).

*Kotenkosius* (Fig. 138) has a unique propodeal carination pattern that includes three complete longitudinal carinae, one medially, the other two sublaterally, and a complete transverse carina near posterior 0.6, with additional small striae radiating from the median and sublateral longitudinal carinae, and most carinae strongly defined and raised (Fernandez-Triana and Boudreault 2018).

*Choeras* (Figs 40–45), as presently understood, is clearly a polyphyletic assemblage of species, some of which may eventually be placed in different genera. It is one of the few Microgastrinae genera that has some species without a fore wing areolet (although the shape of the remaining veins r, 2RS, and 2M usually indicate a partially defined areolet), and other species with a complete areolet that can vary from very small in some species to large in others (van Achterberg 2002, Fagan-Jeffries and Austin 2018b). The propodeum also varies, from having a complete longitudinal median carina to having a partial one, to not having any visible median carina, or having just minute carinae radiating from the nucha. T1 is mostly rectangular (slightly narrowing towards the posterior margin in some species), but never much wider on the posterior margin than on the anterior margin, and T2 is mostly transverse. Many Oriental species of “*Choeras*” most likely represent different lineages from the temperate species and may warrant placement in different genera, e.g., some of the species may be better placed in *Agupta*.

*Dodogaster* (Fig. 80) has a unique set of features in the *Apanteles* group. The propodeum has a more or less complete areola and a partial median carina, the fore wing has a relatively large areolet, and T1–T3 are heavily sculptured and almost form a carapace (Rousse and Gupta 2013).

### Diversity and distribution of Microgastrinae genera at world and regional scales

Microgastrinae are present in all continents except Antarctica. Specimens can be found in all major terrestrial ecosystems, from 82°30'N (Canada, Nunavut, Ellesmere Island, Alert) to 55°S (Argentina and Chile, Tierra del Fuego) in the New World and 50°S (New Zealand, Auckland Islands) in the Old World, and from sea level up to at least 4,500 m (Fernandez-Triana 2018). The information currently available allows us to make preliminary comments on species diversity and distribution at the generic level (Table 5 and Fig. 2).

**Table 5.** World genera of Microgastrinae, based on the present paper. The column Species richness details the current number of described species and estimated total, for each genus, the two figures separate by a slash. The estimated total is very conservative and is based on specimens we have seen in collections. For many genera, more species are to be expected. World region keys: **NEO** Neotropical, **NEA** Nearctic, **PAL** Palaearctic, **OTL** Oriental, **AFR** Afrotropical, **AUS** Australasian (including Oceanian). **X** Genus present in specific region. X* New record for that region (based on undescribed species seen in collections). **X**- Introduced into that region, not native. **X**? Questionable record for a region. The column Host data tallies the genera that have at least one lepidopteran host recorded (although no critical assessment of how accurate those host records was made). The column DNA barcodes records all genera for which there is at least one DNA barcode available; **Yes**- denotes a genus with only partial sequence(s) available, without fulfilling the criteria for DNA-barcode compliant sequences (see Materials and methods for definition of a barcode-compliant sequence).

Genera	Species richness	NEO	NEA	PAL	OTL	AFR	AUS	Host data	DNA bar-codes
*Agupta*	4/30+				**X**		**X**	No	Yes
*Alloplitis*	8/30+				**X**	X*		No	Yes
*Alphomelon*	19/50+	**X**	**X**					Yes	Yes
*Apanteles*	633/3,000+	**X**	**X**	**X**	**X**	**X**	**X**	Yes	Yes
*Austinicotesia*	2/5						**X**	No	Yes
*Austrocotesia*	5/10	**X**?					**X**	No	Yes-
*Beyarslania*	1/2					**X**		No	Yes
*Billmasonius*	1/1				**X**			No	Yes
*Buluka*	11/20				**X**	**X**	**X**	Yes	Yes
*Carlmuesebeckius*	1/1					**X**		No	No
*Chaoa*	1/1				**X**			No	No
*Choeras*	80/100+	X*	**X**	**X**	**X**	**X**	**X**	Yes	Yes
*Clarkinella*	2/5+	**X**	**X**					No	Yes
*Cotesia*	328/1500+	**X**	**X**	**X**	**X**	**X**	**X**	Yes	Yes
*Cuneogaster*	1/5	**X**						No	No
*Dasylagon*	2/5	**X**						Yes	No
*Deuterixys*	18/20+	**X**	**X**	**X**	**X**		**X**	Yes	Yes
*Diolcogaster*	141/1,000+	**X**	**X**	**X**	**X**	**X**	**X**	Yes	Yes
*Distatrix*	32/40+	**X**	**X**	**X**	**X**	**X**		Yes	Yes
*Dodogaster*	1/1					**X**		No	No
*Dolichogenidea*	366/700+	**X**	**X**	**X**	**X**	**X**	**X**	Yes	Yes
*Eripnopelta*	1/1				**X**			No	No
*Exix*	7/10	**X**	**X**					No	Yes-
*Exoryza*	15/20+	**X**	**X**	**X**	**X**	**X**		Yes	Yes
*Exulonyx*	1/1					**X**		No	No
*Fornicia*	32/50+	**X**			**X**	**X**	**X**	Yes	Yes
*Gilbertnixonius*	1/1				**X**			No	Yes
*Glyptapanteles*	307/3,000+	**X**	**X**	**X**	**X**	**X**	**X**	Yes	Yes
*Hygroplitis*	9/10+		**X**	**X**	**X**			Yes	Yes
*Hypomicrogaster*	48/200+	**X**	**X**					Yes	Yes
*Iconella*	38/50+	**X**	**X**	**X**	**X**	**X**		Yes	Yes
*Illidops*	37/50+	**X**	**X**	**X**	**X**	**X**	**X**-	Yes	Yes
*Janhalacaste*	3/5	**X**						Yes	Yes
*Jenopappius*	3/5+					**X**		No	Yes
*Jimwhitfieldius*	2/5+				**X**			No	Yes
*Keylimepie*	4/10	X*	**X**			**X**		No	Yes-
*Kiwigaster*	1/1						**X**	No	Yes
*Kotenkosius*	1/2+				**X**			No	Yes
*Larissimus*	1/5+	**X**						Yes	Yes
*Lathrapanteles*	4/10+	**X**	**X**					Yes	Yes
*Mariapanteles*	2/10+	**X**						No	Yes
*Markshawius*	3/5				**X**			No	Yes
*Microgaster*	104/200+	**X**	**X**	**X**	**X**	**X**	**X**	Yes	Yes
*Microplitis*	192/500+	**X**	**X**	**X**	**X**	**X**	**X**	Yes	Yes
*Miropotes*	15/20				**X**	X*	**X**	Yes	Yes
*Napamus*	2/2			**X**				Yes	No
*Neoclarkinella*	7/50+			X*	**X**	X*		No	Yes
*Nyereria*	29/50+			**X**	**X**	**X**		Yes	Yes
*Ohenri*	1/1					**X**		No	No
*Papanteles*	2/5	**X**						Yes	Yes
*Parapanteles*	62/100+?	**X**	**X**	X*	**X**	**X**	**X**	Yes	Yes
*Parenion*	3/5+				**X**		**X**	No	Yes
*Paroplitis*	5/10		**X**	**X**	**X**			Yes	Yes
*Pelicope*	1/1		**X**					Yes	Yes
*Philoplitis*	9/10+			X*	**X**	**X**		No	Yes
*Pholetesor*	57/100+	**X**	**X**	**X**	**X**	X*	**X**	Yes	Yes
*Prasmodon*	18/30+	**X**						Yes	Yes
*Promicrogaster*	46/100+	**X**	X*	X*	X*	X*	X*	Yes	Yes
*Protapanteles*	25/30+		**X**	**X**	**X**			Yes	Yes
*Protomicroplitis*	3/5	**X**	**X**					Yes	Yes
*Pseudapanteles*	36/100+	**X**	**X**					Yes	Yes
*Pseudofornicia*	4/5+				**X**		**X**	No	No
*Pseudovenanides*	1/5+			X*	**X**			Yes	No
*Qrocodiledundee*	1/1						**X**	No	No
*Rasivalva*	12/20+	X*	**X**	**X**	**X**	**X**	X*	Yes	Yes
*Rhygoplitis*	4/10+	**X**	**X**					Yes	Yes
*Sathon*	23/30+	**X**	**X**	**X**	**X**	X*	**X**	Yes	Yes
*Semionis*	1/1					**X**		No	No
*Sendaphne*	11/20	**X**						No	Yes
*Shireplitis*	6/6						**X**	No	Yes
*Silvaspinosus*	1/2+					**X**		No	Yes
*Snellenius*	41/50+	**X**		**X**	**X**	X*	**X**	Yes	Yes
*Tobleronius*	1/2+				**X**			No	Yes
*Ungunicus*	1/1				**X**			No	Yes
*Venanides*	14/20+	**X**	**X**	X*	**X**	**X**	**X**	Yes	Yes
*Venanus*	11/15+	**X**	**X**					Yes	Yes
*Wilkinsonellus*	23/50+	**X**			**X**	**X**	**X**	Yes	Yes
*Xanthapanteles*	1/1	**X**						No	No
*Xanthomicrogaster*	6/30+	**X**						Yes	Yes
*Ypsilonigaster*	6/10+				**X**			No	Yes
*Zachterbergius*	1/1				**X**			No	Yes

The most species-rich genera are *Apanteles* (in its restricted sense) and *Glyptapanteles*. The latter is probably the largest, but it may eventually be split into several genera. In contrast, *Apanteles*, although also likely to have some species reclassified into other genera, is a much more cohesive group and might end up being the larger group if many species are removed from the current *Glyptapanteles*. Regardless, the diversity of both genera will likely comprise a few thousand species each.

*Apanteles* already contains more than 630 described species (see checklist below); just in ACG, Costa Rica, 186 new species were recently described (Fernandez-Triana et al. 2014e). The world fauna of *Apanteles* could number many more than 3,000 species. The genus is notably absent from New Zealand (although a few species have been introduced there), where it is replaced by *Dolichogenidea* and an undescribed genus. It also has not been found in the high Arctic (Fernandez-Triana et al. 2017b).

*Glyptapantes* contains more than 300 species, with hundreds of undescribed species from all biogeographical regions seen in collections; we estimate that the world total could be more than 3,000 species. However, the generic limits are controversial (see previous section) and it may eventually be restricted to a slightly smaller, although still substantial, number of species. Regardless, its status as one of the two largest genera of Microgastrinae is certain.

The following genera are also very speciose: *Cotesia*, *Diolcogaster*, *Dolichogenidea*, *Hypomicrogaster*, and *Microplitis*. Among these, *Diolcogaster* is clearly the largest, and it could attain more than 1,000 species. But it will almost certainly be split into several genera and thus it could potentially end up having just a few hundred species. *Cotesia*, already with more than 320 described species, will also attain more than 1,000 species (Mason (1981) estimated between 1,500–2,000 species), and is a more cohesive group, unlikely to be severely split. The other three genera will certainly surpass 500 species each, probably substantially (e.g., *Dolichogenidea* already has more than 360 described species). *Diolcogaster* and *Hypomicrogaster* are more speciose in tropical areas, whereas *Cotesia*, *Dolichogenidea* and *Microplitis* tend to be richer in temperate areas.

Other relatively large genera are *Microgaster*, *Choeras*, and *Pholetesor* in temperate areas, and *Parapanteles* and *Pseudapanteles* in tropical areas. All of them are likely to have more than one hundred (in most cases several hundred) species. A few other genera might be equally large, but the material in collections is not comprehensive enough to provide estimates.

In regional composition, the tropical areas have a larger representation than temperate areas (as expected) with the Oriental (46 genera) and Neotropical (43 genera) regions being of comparable diversity, and the Afrotropical (36 genera) and Australasian (28 genera) regions following. Furthermore, we have seen in collections several putative additional (undescribed) genera from all tropical regions. In temperate areas, the Nearctic region (33 genera, including several Neotropical genera having a few species entering North America) has the highest generic diversity and the Palaearctic region (28 genera, including some Oriental genera that have a few species entering the southernmost areas of the Palearctic) has the lowest diversity. Considered as a whole, the entire Holarctic region would have a relatively high diversity of 39 genera.

**Figure 3.**
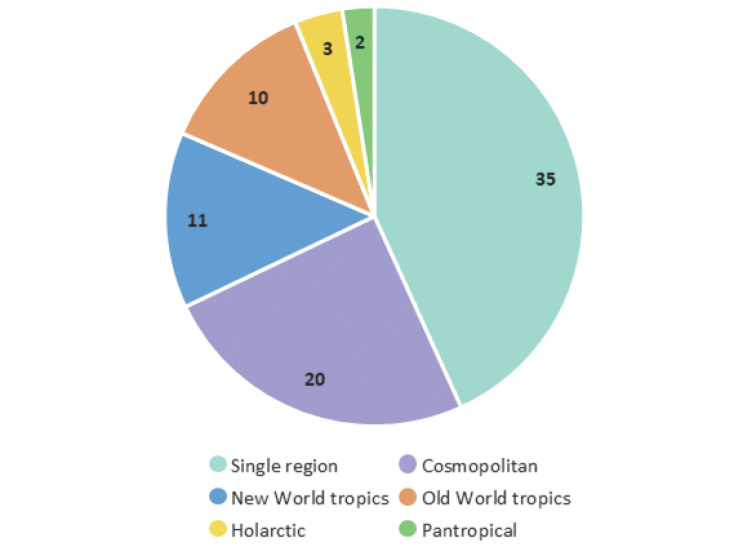
Biogeographical distribution of the 81 Microgastrinae genera currently known worldwide. Data from the present paper.

The distribution of individual genera worldwide (Fig. 3) shows that 20 genera (24.7%) are cosmopolitan or almost so: 15 are present in all biogeographical regions (*Apanteles*, *Choeras*, *Cotesia*, *Diolcogaster*, *Dolichogenidea*, *Glyptapanteles*, *Illidops*, *Microgaster*, *Microplitis*, *Parapanteles*, *Pholetesor*, *Promicrogaster*, *Rasivalva*, *Sathon*, and *Venanides*) while another five are present in five out of the six biogeographical regions (*Deuterixys*, *Distatrix*, *Exoryza*, *Iconella*, and *Snellenius*). A few additional genera may eventually be found to be cosmopolitan.

Eleven genera (13.6%) are restricted to the New World tropics (Neotropical region): *Cuneogaster*, *Dasylagon*, *Janhalacaste*, *Larissimus*, *Mariapanteles*, *Papanteles*, *Prasmodon*, *Sendaphne*, *Venanus*, *Xanthapanteles*, and *Xanthomicrogaster*. Another nine genera (*Alphomelon*, *Clarkinella*, *Exix*, *Hypomicrogaster*, *Lathrapanteles*, *Protomicroplitis*, *Pseudapanteles*, *Rhygoplitis*, and *Venanus*) are almost exclusively found in the Neotropics, with few species reaching the Nearctic. The only genus that can be considered as a Nearctic endemic is *Pelicope*.

Ten genera (12.3%) are relatively widespread in, but restricted to, the Old World tropics: *Agupta*, *Alloplitis*, *Buluka*, *Miropotes*, *Parenion*, and *Pseudofornicia*. We also consider here *Neoclarkinella*, *Nyereria*, *Philoplitis*, and *Pseudovenanides* as almost exclusively present in the Old World tropics, as only a few species reach the southernmost areas of the Palaearctic.

Only two genera (2.5%) (*Fornicia* and *Wilkinsonellus*) seem to be pantropical, and completely absent in the Holarctic. Because almost all undescribed genera of Microgastrinae in collections are from tropical areas, this proportion could increase. No genus has a strictly Holarctic distribution, but three genera almost fulfill that criterion, as just a few species of each reach the northern limits of either the Oriental region (*Hygroplitis* and *Paroplitis*) or the Neotropical region (*Rhygoplitis*).

A total of 35 genera (43.2%) are presently known only from a single biogeographical region, with the Neotropical and Oriental regions each having ten endemic genera, respectively, and the Afrotropical having eight (Table 5). However, some of those genera will almost certainly be found to have a wider distribution.

### DNA barcoding and Microgastrinae

**Figure 4.**
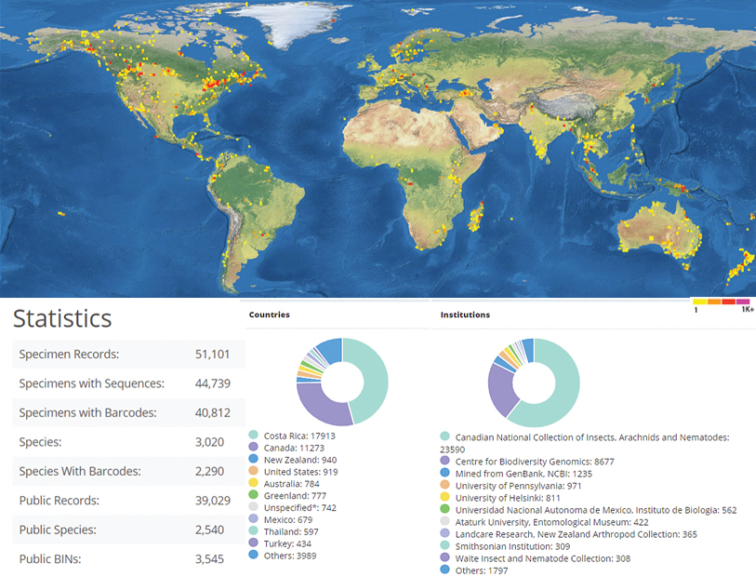
Overview of Microgastrinae data in the Barcoding of Life Data System (BOLD) as of 31 December 2019.

During the past 12+ years, an extensive library of DNA barcodes for Microgastrinae has been assembled (Smith et al. 2013), resulting in the subfamily comprising 37% of all DNA sequences of Braconidae and almost 5% of all Hymenoptera sequences currently available in BOLD. At present, 44,739 specimens of Microgastrinae have sequences deposited in BOLD; 40,812 of those specimens have DNA barcodes representing 3,545 public BINs (http://v4.boldsystems.org/index.php/Taxbrowser_Taxonpage?taxid=2099). The number of BINs will certainly increase, as most of the Microgastrinae specimens currently in BOLD come from just two countries: Canada and Costa Rica (Fig. 4).

BINs usually match well with putative species (as identified by an expert taxonomist), and thus could be used as a surrogate for analyses of species diversity, like other Operational Taxonomic Units (e.g., Ratnasingham and Hebert 2013, Fagan-Jeffries et al. 2018b). Based on our unpublished data, the correspondence between BINs and putative species in Microgastrinae may exceed 90%. For example, the number of Microgastrinae public BINs from Canada and Alaska (combined) currently found in BOLD is 551, very similar to the 550 species estimated for that area by Fernandez-Triana (2010; see also Fagan-Jeffries et al. 2018b). Even with the limited geographical coverage presently available, the total number of worldwide MicrogastrinaeBINs already surpasses the total of described species in our checklist by almost 200.

At the genus level, a significant proportion (67 genera or 83%) have some DNA data (Table 5). In most cases (64 genera or 79%) that includes at least one barcode compliant sequence, usually many more. Many of the 14 genera without molecular data in BOLD include taxa that are very rare in collections, i.e., only known from one or very few specimens, and/or the available specimens are very old (collected many decades ago) and did not yield any DNA. However, for at least a few of those genera it is expected that it will soon be possible to have DNA data.

### Estimating species richness in Microgastrinae

With 2,999 valid species of Microgastrinae recognized here, an interesting question is how many species remain undescribed, whether or not known from collections. The actual species richness of Microgastrinae worldwide has been variously estimated during the past 35 years. At the lower end, Dolphin and Quicke (2001) extrapolated species richness of Braconidae based on data from butterflies and (primarily) mammals, arriving at an estimated 3,617–4,178 species of Microgastrinae. Jones et al. (2009) obtained similar results by comparing taxonomic revision data, with their estimates ranging from 3,900–5,500 species. Mason (1981) thought that 5,000–10,000 species would be a reasonable estimate, based on museum specimens he had seen. At the higher end of the spectrum, Rodriguez et al. (2013) compared the number of Lepidoptera and Microgastrinae species in several areas to arrive at estimates ranging from 17,000 to 46,000+ species.

Obviously, these estimates vary considerably: if the lowest one (3,617) were accurate, then we would already know 82% of the Microgastrinae species; if the highest one (46,620) were accurate, then the described species would represent only 6% of the actual diversity worldwide. Which estimate is more likely to be correct?

While a definite answer cannot be provided, some refinement of the current estimates is possible. The lowest range (3,000–5,000 species) is clearly too low based on what is currently known (2,999 described, valid species are recognized in this paper). As mentioned in the previous section, and despite its limited geographical coverage, Microgastrinae public BINs already represent 3,545 putative species. But, even if DNA data is disregarded, we have certainly seen in collections a few thousand undescribed species, which are clearly distinct based on morphological features alone. In that sense, Mason’s estimate of 10,000 species seems very reasonable.

But could the figures from Rodriguez et al. (2013) also be considered reasonable, or are they way off the mark? Although this might be seen just as a numbers game, the implications are significant. If indeed there were 30-, 40- or even 50,000 species of Microgastrinae worldwide, that could extrapolate to the entire family Braconidae having at least 150–200,000 species, and the entire Hymenoptera having much more than one million species. Those values are an order of magnitude higher than the values presently known for subfamily, family, and order, although they agree with estimates of the entire Hymenoptera suggested by other authors (e.g., LaSalle and Gauld 1991, Hanson and Gauld 1995, Foottit and Adler 2017).

Rodriguez et al. (2013) based their estimates on what Fernandez-Triana (2010) had referred to as the Lepidoptera/Microgastrinae ratio (L/M). Briefly explained, the ratio between lepidopteran and microgastrine species (where sufficient data are available) seems to be surprisingly similar in different regions, regardless of the area and diversity of such regions. The initial calculations were limited and only included three separate areas in Canada (Table 2 in Fernandez-Triana 2010). Based on the average ratio calculated from those three areas (L/M = 12/1) it was concluded that the richness of Microgastrinae in Canada and Alaska would be approximately 550 species. Rodriguez et al. (2013: Table 1) expanded the dataset to eleven different regions, mostly from North America and Europe, but also including New Zealand and ACG in Costa Rica; the resulting L/M ratios were still remarkably close, mostly ranging from 10/1 to 20/1, with an average of 16.4/1.

But just a few years later, some of the numbers used by Fernandez-Triana (2010) and Rodriguez et al. (2013) are already outdated. For Microgastrinae, the species richness in Ottawa, based on Fernandez-Triana et al. (2016a) and subsequent unpublished data, is now approaching 180 species, which represents a 20% increase compared to the total published in 2010; ACG in Costa Rica has surpassed 1,200 species, a 50% increase (based on Janzen and Hallwachs 2016); the Canadian High Arctic now has 26 recorded species or 30% more than initially reported (based on Fernandez-Triana et al. 2017b); the New Zealand fauna will increase by more than 25% compared to previous estimates (Fernandez-Triana & Ward, unpublished); even for the UK, arguably the most thoroughly studied region, the microgastrine count increased by at least 15% (based on Broad et al. 2016). Those revised figures all share one element in common: the species richness of Microgastrinae in those areas was underestimated by both Fernandez-Triana (2010) and Rodriguez et al. (2013).

Thus, the updated L/M ratios calculated for the above regions decreased, from an average of 16/1 in Rodriguez et al. (2013) to around 10/1 at present (also including Finland, where comprehensive data have become available since the Rodriguez et al. paper was published). But the lower the L/M ratio the higher the actual species richness of Microgastrinae. For example, assuming an estimated world number of Lepidoptera between 300,000 (Kristensen et al. 2007) and 500,000 species (Foottit and Adler 2017), and a world average L/M ratio of 10/1, the estimated number of Microgastrinae would then range from 30,000–50,000 species. If anything, the current data still seem to support higher, rather than lower, estimates for the subfamily.

As far as we know, there is only one major caveat in using L/M ratios to extrapolate and calculate the world fauna of Microgastrinae: at present all known figures come from temperate areas, with the sole exception of ACG. There is no other tropical area in the world with sufficient data to allow for meaningful L/M ratios to be calculated. Thus, it may be argued that if different ratios were prevalent in temperate areas compared to the tropics, which harbour, by far, the highest richness of Microgastrinae, then the overall world estimates could not be as high as Rodriguez et al. (2013) suggested. Only more data will allow this to be answered in a definite way; however, for the present it is worth noting that the L/M ratio in ACG (10/1) is actually very similar to those of temperate areas.

### Overview of regional taxonomic studies on Microgastrinae

As with many insect groups, knowledge of Microgastrinae has been historically concentrated on the Northern Hemisphere temperate fauna. However, numerous recent studies are starting to shift focus to the tropics, with most new species in the past few years being described from the hitherto poorly worked Neotropical and Oriental regions, chiefly Costa Rica, China, and India.

In the Western Palearctic subregion, papers from the 1960s–1990s from Nixon and Papp treated most of the Microgastrinae species known up to that time, following careful work by Wilkinson from the 1920s–1940s aimed largely at interpreting poorly understood names (see papers of these three authors cited in the References section). Recent works have described a relatively small number of new species, although their papers sometimes included detailed accounts of species biology, and there is an ongoing concomitant deposition of DNA barcodes, etc. (Oltra and Michelena 1989, Oltra et al. 1995, 1996, Oltra-Moscardó & Jiménez-Peydró 2005, Shaw 1992, 2004, 2007, 2009, 2012b, van Achterberg 2002, Fernandez-Triana et al. 2014c). The Eastern Palearctic subregion is less well known, although progress has also been made (Tobias 1986, Kotenko 1981, 1986, 1992, 1993, 2004, 2007a, 2007b), and most of the new Palaearctic species to be discovered will probably come from the Eastern Palearctic. Some southern areas of the Palearctic, e.g., Iran, Turkey, and the Palearctic area of the Arabian Peninsula have also seen an increase in the number of publications in the last few years (Inanç 1992, 2002a, 2002b, Inanç and Çetin Erdogan 2004, Gadallah et al. 2015, Farahani et al. 2016, Ghahari and van Achterberg 2016, Fernandez-Triana and van Achterberg 2017, Ghafouri Moghaddam et al. 2018, 2019, Samin et al. 2018, Abdoli et al. 2019a, 2019b, Zargar et al. 2019); however, there have been few taxonomic revisions, with most of the work being biodiversity estimates, local checklists, or isolated species descriptions. With 827 described species of Microgastrinae, the Palearctic is currently the most speciose region, although it will almost certainly become the least when more studies in the other regions are undertaken.

In the Nearctic region progress has been slower than in the Palearctic. After two seminal papers from Muesebeck (1921, 1922), most of the new taxa have been described in isolated papers, mostly treating species of biocontrol relevance (Marsh 1975, 1978, 1979b, 1979c, Wharton 1983, Whitfield et al. 1999, Fernandez-Triana 2010, 2018; cf. other papers from Muesebeck cited in the References section), with some taxonomic revisions also produced (Whitfield 1985, 2006, Whitfield et al. 2011, Grinter et al. 2009, Valerio et al. 2009, Valerio and Whitfield 2015, Fernandez-Triana 2015, [Bibr B176][Bibr B179], Fernandez-Triana et al. 2013a). Hundreds of additional species from this region have been revealed by DNA barcoding, but the southernmost areas and west coast, which also happen to be the most species rich, have barely been studied (Fernandez-Triana 2018). It is expected that the actual numbers in the Nearctic will be several times higher than the current 350 described species.

The Neotropical region has been the focus of recent efforts, including the description of more than 400 new species and revision of many genera. However, most of those papers deal almost exclusively with the fauna of ACG, Costa Rica (Janzen et al. 2003, Valerio and Whitfield 2003, Valerio et al. 2005a, Fernandez-Triana 2015, Fernandez-Triana et al. 2013a, 2014a, 2014e, 2014f, 2014g, 2014h, 2015b, 2016b, 2016c), with only some marginal coverage of other countries (Austin and Dangerfield 1989, Penteado-Dias 1995, Penteado-Dias et al. 2000, 2002, 2011, Valerio and Whitfield 2005, 2015, Valerio et al. 2004, 2009, Choi and Whitfield 2006, Grinter et al. 2009, Arias-Penna et al. 2014, 2019, Salgado-Neto et al. 2018, 2019). Large collections have been amassed in South America, e.g., French Guiana, Colombia, Brazil, Ecuador, and Peru, but an impediment to assessing that material is the difficulty in exchanging specimens with colleagues from other countries. In general, most of the Neotropics are extremely understudied, with several thousand species awaiting description but only 768 species described so far. For Microgastrinae, this is likely to be the most speciose region of the world.

The Oriental region, with 752 described species, currently ranks third after the Palearctic and Neotropical regions. It also contains thousands of undescribed species and may rival the Neotropical region as the most speciose. Recent advances have mostly been made in China and India, but we are also aware of large collections of specimens from other countries such as Indonesia, Malaysia, Thailand and Vietnam, which have already resulted in several publications (Austin 1987, 1989, Chen et al. 1994, Long and van Achterberg 2003, 2008, 2011, 2013, 2014, 2015, Chen and Song 2004, Long 2007, 2010, 2015, Fernandez-Triana and Goulet 2009, Fernandez-Triana et al. 2014d, Zeng et al. 2011a, 2011b, Gupta 2013a, 2013b, Gupta and Kalesh 2012, Gupta and Fernandez-Triana 2014, 2015, Gupta et al. 2011, 2013a, 2013b, 2014a, 2014b, 2016a, 2016b, Liu et al. 2014, 205, 2016, 2018, Veena et al. 2014, van Achterberg et al. 2015, Xiong et al. 2017, Zhang et al. 2017, Ranjith et al. 2015a, 2015b, 2019; cf. papers from authors Chen, Sathe, Song, Xu, and You cited in the References section). The main problem (other than difficulties in exchanging material) is the lack of revisions covering the entire region; the available taxonomic keys and papers tend to cover single countries, with few efforts to coordinate work at a larger (regional) scale. There is also a number of species described from India in publications that do not comply with ICZN Article 16, and thus those names are unavailable (see section Unavailable names below).

No significant progress has been made in the Afrotropical region for the past half a century. The very few exceptions include recent papers on the fauna of Réunion (Rousse and Gupta 2013), the Afrotropical area of the Arabian Peninsula (Fernandez-Triana and van Achterberg 2017), and some new species of importance in biocontrol (Kaiser et al. 2017, Fiaboe et al. 2017), or more general papers not specifically devoted to the Afrotropics (Walker 1994, Valerio et al. 2009, Fernandez-Triana and Goulet 2009, Fernandez-Triana and Boudreault 2018). However, relatively large collections from Kenya, Madagascar, Republic of Congo, and South Africa have been amassed during the past few years (Fernandez-Triana and Boudreault 2018), and there is potential to add hundreds, if not thousands of new species. Although the current total of described species is just 429 it is estimated that this will be the third most species-rich region of the planet for Microgastrinae.

Since the 1990s, several papers have treated the Australasian species (Austin 1990, Austin and Dangerfield 1992, 1993, Walker 1996, Saeed et al. 1999, Fernandez-Triana et al. 2011, 2013b, Fagan-Jeffries and Austin 2018, Fagan-Jeffries et al. 2018a, 2018b, 2019), but progress has been comparatively slow. At present 222 species are described from this region. Work on Pacific islands is basically non-existent but, when done, may reveal many more new and interesting taxa.

### Hosts of Microgastrinae

The host range of a parasitoid is one of its most important features, linking its evolutionary past with its present autecology (Shaw 1994, Shaw and Aeschlimann 1994). Through knowledge of the host range it is possible to understand and to predict a parasitoid’s behaviour within current ecosystems (Shaw 2017b), and also gain some understanding of the speciation processes that brought them into existence (Shaw 2003).

Microgastrinae are the single most important group of parasitoids of Lepidoptera in the world, both in economic terms and in species richness (Whitfield 1995a, 1997). They are all koinobiont endoparasitoids and parasitize almost the entire taxonomic and biological spectrum of Lepidoptera (Shaw and Huddleston 1991, Whitfield 1997, Whitfield et al. 2018), with the probable exception of the four most basal superfamilies.

Adult female wasps typically oviposit into early instar larvae (with a few species known to oviposit into host eggs), within which the wasp eggs hatch and larval development takes place with the aid of venom and polydnavirus (PDV) effects on the host’s immune and endocrine system (summarized in Whitfield et al. 2018). All microgastrines fully depend on mutualistic PDVs to successfully parasitize hosts, the relationship between wasps and PDVs being the most remarkable known example of the evolution of a mutualistic endosymbiotic association between eukaryotes and viruses (Strand and Burke 2012, 2014).

Numerous literature records of non-Lepidoptera as hosts of Microgastrinae exist (Table 6), comprising at least 29 families within five orders of Insecta (data compiled from Yu et al. 2012). However, these records are wrong or at the very least highly questionable.

**Table 6.** Historical account of Microgastrinae hosts that are not Lepidoptera, based on the compilation of Yu et al. (2012).

**Order**	**Families**
Coleoptera	Anobiidae, Anthomyiidae, Attelabidae, Bostrichidae, Buprestidae, Cerambycidae, Chrysomelidae, Coccinellidae, Curculionidae, Melandryidae, Phalacridae, Scirtidae
Diptera	Agromyzidae, Cecidomyiidae, Chloropidae, Muscidae, Syrphidae, Tephritidae
Hymenoptera	Apidae, Argidae, Cimbicidae, Cynipidae, Diprionidae, Eurytomidae, Pteromalidae, Tenthredinidae, Vespidae
Mantodea	Mantidae
Trichoptera	Limnephilidae

For example, the record of Apidae (*Bombus* sp.) as “host” of Microgastrinae can be easily rejected. *Bombus* nests have associated case-bearing moth caterpillars (Tineidae) feeding within the nest and the three known species of Microgastrinae that emerge from those nests actually parasitize the caterpillars, not the bees (Whitfield and Cameron 1993, Whitfield et al. 2001).

Two other recent examples are equally illustrative. The record of *Enoicylapusilla* (Burmeister) (Trichoptera: Limnophilidae) as a host of the microgastrine *Choerasgielisi* (van Achterberg 2002) was at times considered to be a reliable example of a non-Lepidoptera host record; however, subsequent examination of the situation has called that record into doubt as it was reared from a substrate from which the host remains were not recovered (Shaw 2017a). Similarly, Kopelke (2011) reported two different species of *Dolichogenidea* (each from a single specimen), as part of his extensive rearing of inhabitants of 34,210 galls of nematine sawflies (Hymenoptera: Tenthredinidae) in Europe; he asserted those two cases to be accidental, but genuine (Kopelke 2011: 9). Unfortunately, it is not clear from the publication if host remains were available (in those two specific cases) to confirm host identity, and under such circumstances we consider it appropriate to regard those records as highly dubious. Sawfly galls are nutritious and frequently fed on by caterpillars. It is relatively easy for a small parasitized lepidopteran larva to enter such a structure to die and become practically entirely consumed by the parasitoid, leaving almost only the head capsule. This happens with most *Dolichogenidea* species, which have a final external feeding period that leaves the host remains easily overlooked or misinterpreted. Many similar deductions concerning other recorded supposed non-lepidopteran hosts are easily made.

Even if examples of parasitization of other insect orders by Microgastrinae are well founded, we consider such cases would be highly abnormal. Shaw (1994) provided a conceptual definition for the host range of a particular parasitoid species, which should include only those species of potential hosts that the parasitoid is usually able to attack successfully, following a pattern of searching behaviour enabling it to encounter them regularly. That rather loose definition implies that some perfectly correct rearing records should be excluded from the host range if they represent only freak events of no importance to the autecology of the parasitoid or the host, and lack phylogenetic significance. It also implies that some hosts within the host range may be intrinsically more important than others that are encountered less frequently, or attacked less enthusiastically, or with a less successful outcome.

We also consider that there is no convincing evidence that the four most basal superfamilies of Lepidoptera (*sensu*Aarvik et al. 2017) are parasitized by Microgastrinae. There is no published record of Microgastrinae parasitizing Micropterigoidea and Eriocranioidea, and the few literature records of hosts in Hepialoidea and Nepticuloidea are highly questionable; we discuss and reject them below.

*Sathonfalcatus* (Nees, 1834) was recorded in two broods (of 45 and 37 individuals) parasitizing *Hepialushumulis* (Linnaeus, 1758) (Hepialidae) in the United Kingdom (Hammond and Smith 1957). We have located those specimens in the NHMUK but, although the relevant cocoon masses are present, there are no host remains. *Sathonfalcatus* is a known parasitoid of the noctuid moth *Apameamonoglypha* (Hufnagel, 1766), whose larvae are superficially very similar to those of *Hepialushumuli*. Thus, we distrust the record strongly enough to refute it. It should also be noted that the rearings were not done by Hammond, who was the expert on Lepidoptera larvae.

The other known record is for *Cotesiaspuria* (Wesmael, 1837) parasitizing *Triodiasylvina* (Linnaeus, 1761) (Hepialidae), published by Telenga (1955) with no details whatsoever, i.e., no information was provided on who identified the host or the parasitoid, or where and when the sample was collected, nor the depository of specimens. *Cotesiaspuria* does have a wide host range, but confirmed hosts are all folivorous macrolepidoptera. Under these circumstances it is best to simply refute the record; although of course, if a rearing is repeated with appropriate credentials the refuted record could be recalled to stand as a possible previous instance.

The two published records of Nepticuloidea as hosts are also highly suspicious. Nixon’s (1976) record of *Fomoriaweaveri* (Stainton, 1855) (Nepticulidae) as a host of *Apantelescontaminatus* (Haliday, 1834) has recently been refuted by Shaw (2012b), who commented on the rearing. The inflated mines of *F.weaveri* are superficially similar to those *Epinotianemorivaga* (Tengström, 1848) (Tortricidae) from which *A.contaminatus* has been reliably reared; thus, in this case an error in host identification was almost certainly involved. Unfortunately, the specimen could not be found in the cited depository.

Gates et al. (2002: 221) recorded Stigmella?variella (Nepticulidae) being parasitized by *Dolichogenideatischeriae*Viereck (1912b) from a leaf mine on oak (*Quercusagrifolia* Née). However, that record is quoted as “parasitoids lot-reared from more than one leafmines from a single plant” (see caption of Table 2 on page 230 of Gates et al. 2002), and in that same Table other Lepidoptera families were recorded from that host plant, including several species of Gracillariidae and Tischeriidae, both of which had been reported as hosts of *D.tischeriae* in other papers and most likely represent the actual host(s). In that case, it is clear that the sample (leaves with mines) contained several lepidopteran species, and that *Stigmella* was wrongly assigned as a host of *D.tischeriae*.

Adeloidea and Tischerioidea are the most basal superfamilies of Lepidoptera (and the only non-Ditrysia groups) for which there is reasonably solid evidence supporting them as being hosts of Microgastrinae. There is reliable data showing that a few Microgastrinae indeed parasitize species of Adelidae (Shaw 2012b), Incurvariidae (Fernandez-Triana 2010), Prodoxidae (Nixon 1972, Shaw 2012b, Whitfield et al. 2005), Tischeriidae (Shaw 2012b) and even Heliozelidae (Fernandez-Triana et al., unpublished data).

Ditrysia (*sensu*Kristensen and Skalski 1999, Roe et al. 2009) constitutes the most derived clade of Lepidoptera, comprising more than 98% of all lepidopteran species, and representing by far the group most commonly parasitized by Microgastrinae. Eulepidoptera (*sensu*Aarvik et al. 2017) consists of Adeloidea + Tischerioidea + Ditrysia, which are the three groups for which we have solid evidence of parasitism by Microgastrinae. Thus, in this paper we propose that Microgastrinae hosts are restricted to Eulepidoptera, i.e., most of the Lepidoptera except for the four most basal superfamilies: Micropterigoidea, Eriocranioidea, Hepialoidea and Nepticuloidea. We consider all previous literature records of other insect orders and of the four early branching lineages of Lepidoptera as incorrect. Claims for hosts other than Eulepidoptera, which are made with conviction from time to time, are in our experience never supported by the recovery and preservation of associated host remains for careful assessment.

The published sources we compiled so far include Lepidoptera host data for 44 genera (54%) and around 1,250 species (42%) of Microgastrinae. Although the coverage is insufficient, those records include 3,200+ species of Lepidoptera and represent 5,500+ supposed host/parasitoid associations. In addition, there is a large amount of unpublished but databased host information (e.g., http://janzen.sas.upenn.edu/caterpillars/database.lasso; http://www.caterpillars.org/), with hundreds of additional host/parasitoid records from currently undescribed microgastrine species (e.g., Whitfield et al. 2009, 2018, Hrcek et al. 2013). Still, more than half of the described species of microgastrines lack any information about their hosts. Even worse, an unknown but probably very large proportion of the published associations are also almost certainly wrong. Clearly, there is much to be learned, and for the existing information to be a good basis for understanding host records there needs to be a critically examination of the data to (try to) prune out wrong host/parasitoid associations, an effort that would require years of work, and even then would leave much uncertainty. A better approach to secure real knowledge may be to ensure that higher standards of data collection and specimen deposition take place for the future: in fact, without that we cannot expect much improvement in our understanding.

From the data presently available, the top ten families of Lepidoptera (as per number of species recorded as host) which are parasitized by Microgastrinae are Noctuidae, Tortricidae, Pyralidae, Crambidae, Geometridae, Gracillariidae, Depressariidae, Hesperiidae, Gelechiidae, and Nymphalidae. Altogether those families account for two-thirds of all known host/microgastrine parasitoid associations, which is not surprising given that they are also among the most species- rich Lepidoptera families. That probably also reflects a bias in collecting effort: these families provide most of the economically important crop and forestry pests, which are accordingly the most intensely sampled taxa for their parasitoids. Further, in some of these families there are large and/or spectacular caterpillars that are the most often seen and reared by hobbyists. Other groups such as stem borers, leaf litter, and canopy caterpillars tend to be less commonly reared.

Earlier compilations for species within particular microgastrine genera are dominated by records from the northern temperate region which are unlikely to reflect the complete spectrum of host associations when the ongoing (but currently mostly unpublished) massive number of rearings from tropical surveys are taken into account, e.g., Whitfield et al. (2018). In addition, there is a need to recognise phenological aspects of host range, especially in temperate climates: many parasitoid species are plurivoltine yet use univoltine hosts, each available to only one generation of the parasitoid; sometimes it happens that the parasitoid is, at least locally, entirely dependent on a single host species at one time of year but able to use another host or a wider range of hosts at another (see Shaw and Aeshlimann 1994). Last but not least, a parasitoid’s realized host range may not be constant in either space or time unless, of course, the parasitoid is strictly monophagous, and thus the relative abundance of co-occurring hosts will also vary (Shaw 2006). Recognition of the realized host range at a point in space and time is often of more practical significance for population dynamics, conservation biology, or biological control (Shaw 1994, 2003, 2017).

Despite the constraints mentioned above and the relatively poor state of knowledge, some general comments can be made for some of the most speciose Microgastrinae genera. For example, most *Microgaster*, *Choeras*, *Apanteles*, and *Dolichogenidea* species parasitize more or less concealed host larvae, allowing the final instar larvae of these parasitoids to carry out their external feeding phase in a sheltered environment, and host Lepidoptera with this amenable larval biology overwhelmingly belong to the families of the so-called microlepidoptera. Other genera such as *Pholetesor* and *Deuterixys* specialize on leaf-miners and parasitize hosts that feed in at least moderate concealment, as is required by the final external tissue-feeding phase of their parasitoid larvae. This is correlated with their use of hosts primarily from microlepidopteran families, which tend to be small, resulting in most of the parasitoids of microlepidoptera being solitary. In contrast, genera such as *Microplitis*, *Cotesia*, *Distatrix*, *Diolcogaster*, *Protapanteles*, and *Glyptapanteles* are fully endophagous and well-suited to parasitize exposed Lepidoptera larvae, such as those of many macrolepidoptera, which tend to be large and are thus more suited to support gregariousness, which is much more expressed in these microgastrine genera. There are exceptions, but they can often be understood in autecological terms, e.g., the few *Microgaster* that parasitize macrolepidopterans have hosts that feed or rest in concealed sites (Shaw 2004); the few *Cotesia* that parasitize microlepidopterans are usually associated with semi-exposed hosts in webs which feed partly exposed (see Nixon 1974); the *flavipes* group of *Cotesia* parasitizes stem borers in the families Pyralidae and Crambidae (e.g., Fujie et al. 2018).

Whenever comprehensive data are available, be it in temperate (e.g., in Europe and especially the United Kingdom), or tropical areas (e.g., ACG), patterns emerge. Often, they show that many species within most genera of Microgastrinae appear to have a high host specificity, often having been recorded from only a single or very few taxonomically closely related species. An alternative is having ecologically similar hosts (Shaw 2003, Fernandez-Triana 2018). Earlier studies often did not differentiate these levels of host specificity clearly, partly due to the presence of many morphologically cryptic species in large genera of Microgastrinae but also because it is very much harder to discover all or most of the hosts of a particular parasitoid species than it is to discover all or most of the parasitoid species using a given host. Only recently have they been detected through integrative taxonomy that incorporates DNA barcoding and other molecular methods, as well as much greater levels of field ecological data (Shaw 2017b, Whitfield et al. 2018).

However, some species of Microgastrinae seem to be much less restricted. Examples include *Glyptapantelesvitripennis* (Curtis, 1830), an incredibly polyphagous species with an immense host range of mainly (but not entirely) exposed macrolepidoptera found on trees and bushes in Europe (Nixon 1973, Shaw unpublished data), or *Glyptapantelespseudotsugae* Fernandez-Triana, 2018, which parasitizes several lepidopteran species (Geometridae and Erebidae) feeding on Douglas fir across a range of 2,500 km in western North America (Fernandez-Triana 2018).

A few Microgastrinae genera seem to be restricted to only one host Lepidoptera family, e.g., *Alphomelon* (only reared from Hesperiidae), *Fornicia* (Limacodidae), *Janhalacaste* (Depressariidae), *Papanteles* and *Xanthomicrogaster* (Crambidae), and *Pelicope* (Prodoxidae). However, these microgastrine genera are not very species rich and it is difficult to know whether more data would extend their apparent associations.

For more speciose genera, the patterns are less clear or consistent, as the number of host families increases, in some cases dramatically. This may in part be a consequence of some Microgastrinae genera not being well defined, comprising at present an arrangement of different lineages that may be separated into different genera in future, e.g., *Choeras*, *Diolcogaster*, *Glyptapanteles*, and *Hypomicrogaster*. But some large and relatively well-defined genera, e.g., *Apanteles*, *Cotesia*, *Dolichogenidea*, *Microplitis*, and *Microgaster*, have large host ranges, including both early and more recently branching lepidopteran families, and ecological factors in their radiations have clearly been of importance.

There is no comprehensive account of the impact of Microgastrinae in biological control. Whitfield (1997) estimated that more than one hundred species had been studied and used in biocontrol programs against caterpillar pests worldwide, but he did not provide details or references to support that number. We have compiled the available information and have found that 800+ species of Lepidoptera considered as pests of some sort in agriculture and forestry are parasitized by Microgastrinae (Fernandez-Triana et al. unpublished data; host data for individual species of Microgastrinae is not presented in this paper, see next paragraph). That includes 110+ major pests, highlighting the importance of this group of parasitoid wasps in biological control programs anywhere.

In summary, Microgastrinae are the most abundant and diverse taxon of hymenopteran parasitoids reared from lepidopteran caterpillars worldwide. However, our current level of knowledge is still poor, as more than half of the wasp species have no host association records, and of the records that do exist, many of them are doubtful or plainly wrong. Considerable effort will be needed before we have a better and more accurate picture of the host/parasitoid associations of most species of Microgastrinae. Thus, in this paper we only provide general comments; details on individual host/parasitoid associations are intentionally omitted to avoid repeating and perpetuating inaccurate information.

### Checklist of world genera and species of Microgastrinae

[Genera, and species within each genus, are arranged in alphabetical order. At the end of the list we place the species we consider as *species inquirendae*, *nomina dubia*, and *nomina nuda*, also in alphabetical order. For a complete list of all Microgastrinae available names in strict alphabetical order see also Suppl. material 1, 2]

#### Genus *Agupta* Fernandez-Triana, 2018

***Agupta*** Fernandez-Triana, 2018: 28. Gender: neuter. Type species: *Aguptajeanphilippei* Fernandez-Triana & Boudreault, 2018, by original designation.

Four species are described from the Oriental region (Fernandez-Triana and Boudreault 2018); those authors stated that there are dozens of undescribed species, based on collection holdings and specimens with available DNA barcodes, from the Australasian and Oriental regions. No revision of the genus has yet been produced. No host data are currently available for this genus. There are dozens of DNA-barcode compliant sequences of *Agupta* in BOLD, representing more than 25 different BINs (but none of those sequences have been identified in BOLD as belonging to *Agupta*, see Fernandez-Triana and Boudreault (2018) for more details on that).

***Aguptadanyi* Fernandez-Triana & Boudreault, 2018**

*Aguptadanyi* Fernandez-Triana & Boudreault, 2018.

**Type information.** Holotype female, RMNH (examined). Country of type locality: Malaysia.

**Geographical distribution.**OTL.

**OTL**: Malaysia.

***Aguptajeanphilippei* Fernandez-Triana & Boudreault, 2018**

*Aguptajeanphilippei* Fernandez-Triana & Boudreault, 2018.

**Type information.** Holotype female, RMNH (examined). Country of type locality: Malaysia.

**Geographical distribution.**OTL.

**OTL**: Malaysia.

***Aguptaraymondi* Fernandez-Triana & Boudreault, 2018**

*Aguptaraymondi* Fernandez-Triana & Boudreault, 2018.

**Type information.** Holotype female, RMNH (examined). Country of type locality: Malaysia.

**Geographical distribution.**OTL.

**OTL**: Malaysia.

***Aguptasolangeae* Fernandez-Triana & Boudreault, 2018**

*Aguptasolangeae* Fernandez-Triana & Boudreault, 2018.

**Type information.** Holotype female, RMNH (examined). Country of type locality: Malaysia.

**Geographical distribution.**OTL.

**OTL**: Malaysia.

#### Genus Alloplitis Nixon, 1965

***Alloplitis*** Nixon, 1965: 268. Gender: masculine. Type species: *Alloplitisguapo* Nixon, 1965, by original designation.

Eight species are currently described from the Oriental and Afrotropical regions, but we have seen in collections (CNC, RMNH) numerous additional species from those regions. No revision of the genus has been produced, although a key to all four species known from Vietnam (Long & van Achterberg 2008) covers half of the described species. No host data are currently available for the genus. There are 20 DNA-barcode compliant sequences of *Alloplitis* in BOLD representing eight different BINs, most of them undescribed species from Thailand.

***Alloplitisalbiventris* Long & van Achterberg, 2008**

*Alloplitisalbiventris* Long & van Achterberg, 2008.

**Type information.** Holotype female, IEBR (not examined but original description checked). Country of type locality: Vietnam.

**Geographical distribution.**OTL.

**OTL**: Vietnam.

***Alloplitiscompletus* Mason, 1981**

*Alloplitiscompletus* Mason, 1981.

**Type information.** Holotype female, CNC (examined). Country of type locality: Malaysia.

**Geographical distribution.**OTL.

**OTL**: Malaysia.

***Alloplitiscongensis* (de Saeger, 1944), new combination**

*Microplitiscongensis* de Saeger, 1944.

**Type information.** Holotype male, RMCA (not examined but original description checked). Country of type locality: Democratic Republic of Congo.

**Geographical distribution.**AFR.

**AFR**: Democratic Republic of Congo.

**Notes.** Even in the original description (de Saeger 1944), this species was considered not likely to belong to *Microplitis*. Without examining the holotype (and only known specimen), the best generic placement at present would be *Alloplitis* based on the propodeal areola, T1 with an impression on the basal third and striae on lateral margins, T2 rectangular in shape, and T3 shorter than T2.

***Alloplitisdetractus* (Walker, 1860), new combination**

*Microgasterdetractus* Walker, 1860.

**Type information.** Holotype male, NHMUK (examined). Country of type locality: Sri Lanka.

**Geographical distribution.**OTL.

**OTL**: Sri Lanka.

**Notes.** From the original description and subsequent treatment of the species (Wilkinson 1927, 1929), it is clear that this species does not belong to *Microgaster*. After examining the holotype, we here transfer *detractus* to *Alloplitis* based on its short metatibial spurs, propodeum with a complete areola defined by strong carinae, T1 with a broad impression on anterior half, T2 broadly rectangular, and anteromesoscutum, scutellar disc, T1 and T2 heavily sculptured.

***Alloplitisguapo* Nixon, 1965**

*Alloplitisguapo* Nixon, 1965.

**Type information.** Holotype female, USNM (examined). Country of type locality: Philippines.

**Geographical distribution.**OTL.

**OTL**: Philippines, Vietnam.

***Alloplitislaevigaster* Long & van Achterberg, 2008**

*Alloplitislaevigaster* Long & van Achterberg, 2008.

**Type information.** Holotype male, IEBR (not examined but original description checked). Country of type locality: Vietnam.

**Geographical distribution.**OTL.

**OTL**: Vietnam.

***Alloplitistyphon* Nixon, 1965**

*Alloplitistyphon* Nixon, 1965.

**Type information.** Holotype female, USNM (examined). Country of type locality: Philippines.

**Geographical distribution.**OTL.

**OTL**: Philippines.

***Alloplitisvietnamicus* Long & van Achterberg, 2008**

*Alloplitisvietnamicus* Long & van Achterberg, 2008.

**Type information.** Holotype female, IEBR (not examined but original description checked). Country of type locality: Vietnam.

**Geographical distribution.**OTL.

**OTL**: Vietnam.

#### Genus Alphomelon Mason, 1981

***Alphomelon*** Mason, 1981: 54. Gender: neuter. Type species: *Urogasternigriceps* Ashmead, 1900, by original designation.

Known from 19 described species from the New World (mostly Neotropical, with a few extending north into the Nearctic). The revision by Deans et al. (2003) is outdated; we have seen in collections (CNC) dozens of additional species, and the genus will easily surpass 50 species with additional study of the Neotropical fauna. All data currently available suggest that *Alphomelon* species may exclusively be parasitoids of Hesperiidae. There are 1,200+ DNA-barcode compliant sequences of this genus in BOLD, representing 32 BINs, most of them undescribed species from Costa Rica.

***Alphomelonarecaphile* Deans, 2003**

*Alphomelonarecaphile* Deans, 2003.

**Type information.** Holotype female, USNM (not examined but paratype examined). Country of type locality: Costa Rica.

**Geographical distribution.**NEO.

**NEO**: Brazil (PA), Costa Rica.

***Alphomelonbrachymacher* Deans, 2003**

*Alphomelonbrachymacher* Deans, 2003.

**Type information.** Holotype female, USNM (not examined but authoritatively identified specimens examined). Country of type locality: Colombia.

**Geographical distribution.**NEO.

**NEO**: Brazil (ES, MT, PA, SC), Colombia, Costa Rica, Ecuador, Peru.

**Notes.** The specimens we studied were identified by the author of the species.

***Alphomelonbrasiliensis* Shimabukuro & Penteado-Dias, 2003**

*Alphomelonbrasiliensis* Shimabukuro & Penteado-Dias, 2003.

**Type information.** Holotype female, DCBU (not examined but original description checked). Country of type locality: Brazil.

**Geographical distribution.**NEO.

**NEO**: Brazil (MG, SP, RS).

***Alphomelonbromeliphile* Deans, 2003**

*Alphomelonbromeliphile* Deans, 2003.

**Type information.** Holotype female, USNM (not examined but paratype examined). Country of type locality: Costa Rica.

**Geographical distribution.**NEO.

**NEO**: Costa Rica, Mexico.

***Alphomeloncitroloma* Deans, 2003**

*Alphomeloncitroloma* Deans, 2003.

**Type information.** Holotype female, USNM (not examined but paratype examined). Country of type locality: Argentina.

**Geographical distribution.**NEO.

**NEO**: Argentina, Bolivia, Brazil (PE, RJ, RO), Costa Rica, Ecuador, Panama, Paraguay, Trinidad & Tobago, Venezuela.

***Alphomelonconforme* (Muesebeck, 1958)**

*Apantelesconformis* Muesebeck, 1958.

**Type information.** Holotype female, USNM (not examined but original description checked). Country of type locality: Venezuela.

**Geographical distribution.**NEO.

**NEO**: Brazil (RJ), Costa Rica, Venezuela.

**Notes.** This species was transferred from *Apanteles* to *Alphomelon* by Deans et al. (2003), although the new combination was not clearly formalized (but is implicit, see pages 1 and 18 of that paper). Deans et al. (2003) did not change the ending of the species name to agree in gender with the generic name (Article 34.2 of the ICZN). The genus *Alphomelon* was described by Mason (1981) as neuter, but *conformis* is a masculine adjective, and thus it must be changed to the neuter form conforme. Until now, no published paper had ever referred to this species as *Alphomelonconforme* although Taxapad (Yu et al. 2012, 2016) correctly did so.

***Alphomeloncrocostethus* Deans, 2003**

*Alphomeloncrocostethus* Deans, 2003.

**Type information.** Holotype female, USNM (not examined but paratype examined). Country of type locality: Jamaica.

**Geographical distribution.**NEO.

**NEO**: Bolivia, Brazil (ES, MG, RJ), Colombia, Jamaica, Puerto Rico.

***Alphomelondisputabile* (Ashmead, 1900), lectotype designation**

*Urogasterdisputabilis* Ashmead, 1900.

**Type information.** Lectotype male, NHMUK (examined). Country of type locality: Grenada.

**Geographical distribution.**NEA, NEO.

**NEA**: USA (KS, TX); **NEO**: Argentina, Belize, Bolivia, Brazil (ES, MT, PA, RJ, SC), Costa Rica, Cuba, Dominica, Ecuador, Grenada, Guatemala, Mexico, Panama, Paraguay, Puerto Rico, Saint Vincent, Trinidad & Tobago, Venezuela.

**Notes.**Ashmead (1900c: 286) did not designate a type in the original description of the species, which was based on 'several specimens'. Subsequent references to the species (e.g., Muesebeck 1921, Shenefelt 1972, Marsh et al. 1979) did not address that either. In the most complete nomenclatural account of the species (Shenefelt 1972: 494), it is implied that the type series, including both male and female specimens, was deposited in London (NHMUK), and could be from either Grenada or Saint Vincent. Much later Deans et al. (2003) mentioned that they had examined the holotype of the species, which they wrote was a male and was deposited in the USNM (with USNM type #6446). However, there cannot be a 'holotype' when Ashmead’s paper makes it clear that the species description was based on a series of specimens. From the Introduction section of the original paper (Ashmead 1900c: 207) it is also clear that the specimens studied were loaned to him from London (NHMUK). Thus, what likely happened was that, after studying the loaned material, Ashmead retained one specimen in Washington from the original type series and returned the rest to London. That means that the male specimen examined by Deans et al. (2003) in Washington is a syntype. The Washington specimen cannot be considered as the lectotype either, following ICZN Article 74.7 “Lectotype designation after 1999”, which clearly states that “To be valid, a lectotype designation made after 1999 must, 74.7.1. employ the term “lectotype” or an exact translation (e.g., “lectotypus” but not “the type”), 74.7.2. contain information sufficient to ensure recognition of the specimen designated, and 74.7.3. contain an express statement of deliberate designation (merely citing a specimen as “lectotype” is insufficient)”. For the sake of clarity, here we designate a male specimen as the lectotype [NHMUK, type number 3c.2395, specimen number 010636228, ‘St’ Vincent, | W.I. | H.H. Smith’, ‘W. Indies | 99-331.’]. There are an additional four paralectotype males in NHMUK, three from Grenada and one from St. Vincent, that from St. Vincent labelled by Ashmead as ‘Type male’ and with a yellow ‘co-type’ label. The specimen designated lectotype here is in better condition, albeit lacking its antennae.

***Alphomelonmelanoscelis* Deans, 2003**

*Alphomelonmelanoscelis* Deans, 2003.

**Type information.** Holotype female, ESUW (not examined but paratype examined). Country of type locality: Costa Rica.

**Geographical distribution.**NEO.

**NEO**: Belize, Brazil (AL, MT), Costa Rica, Mexico, Venezuela.

***Alphomelonnanosoma* Deans, 2003**

*Alphomelonnanosoma* Deans, 2003.

**Type information.** Holotype female, USNM (not examined but authoritatively identified specimens examined). Country of type locality: Costa Rica.

**Geographical distribution.**NEO.

**NEO**: Brazil (MT), Costa Rica, Ecuador, Mexico, Panama, Trinidad & Tobago.

**Notes.** The specimens we studied were identified by the author of the species.

***Alphomelonnigriceps* (Ashmead, 1900), lectotype designation**

*Urogasternigriceps* Ashmead, 1900.

**Type information.** Lectotype female, NHMUK (examined). Country of type locality: Saint Vincent.

**Geographical distribution.**NEA, NEO.

**NEA**: USA (FL, NC, TX); **NEO**: Argentina, Belize, Brazil (RO), Colombia, Cuba, Dominica, Grenada, Netherlands Antilles, Peru, Saint Lucia, Saint Vincent, Trinidad & Tobago, Venezuela.

**Notes.**Ashmead (1900c: 284) did not designate a type in the original description of the species, which was based on eight female specimens. Subsequent references to the species (e.g., Szépligeti 1904, Muesebeck 1921, Shenefelt 1972, Marsh et al. 1979) did not address that either. The most complete nomenclatural account of the species (Shenefelt 1972: 580) mentioned that the type series was in London (NHMUK), and a female specimen, with code 3c.1125 is referred to as the type. Much later, Deans et al. (2003) mentioned that they had examined the holotype of the species, which they wrote was a female and was deposited in the USNM (with USNM type #6443). Deans et al. (2003) probably overlooked Shenefelt’s account, but in any case, there cannot be a holotype when the original paper makes clear that it was a series of specimens. From the Introduction section of the original paper (Ashmead 1900c: 207) it is clear that the specimens studied were loaned to him from London (NHMUK). Thus, what likely happened was that, after studying the loaned material, Ashmead retained one specimen in Washington from the original type series and returned the rest to London. That means that the female specimen that Deans et al. (2003) saw in Washington is a syntype. We have seen in London the specimen referred to by Shenefelt (1972) with code 3c.1125. It is a female in good condition and, in addition to the standard type label from the NHMUK, it also has an additional, handwritten label that reads “*Urogasternigriceps*, ? type, Ash.” For the sake of clarity, here we designate that female specimen as the lectotype; the female specimen examined by Deans et al. (2003) and deposited in the USNM, as well as the rest of the female specimens deposited in NHMUK are thus to be considered as paralectotypes.

***Alphomelonpaurogenum* Deans, 2003**

*Alphomelonpaurogenum* Deans, 2003.

**Type information.** Holotype female, MCZ (not examined but original description checked). Country of type locality: Argentina.

**Geographical distribution.**NEO.

**NEO**: Argentina, Chile.

***Alphomelonpyrrhogluteum* Deans, 2003**

*Alphomelonpyrrhogluteum* Deans, 2003.

**Type information.** Holotype female, MCZ (not examined but original description checked). Country of type locality: Argentina.

**Geographical distribution.**NEO.

**NEO**: Argentina.

***Alphomelonrhyssocercus* Deans, 2003**

*Alphomelonrhyssocercus* Deans, 2003.

**Type information.** Holotype female, CNC (examined). Country of type locality: Ecuador.

**Geographical distribution.**NEO.

**NEO**: Argentina, Costa Rica, Ecuador, Panama, Peru, Trinidad & Tobago, Venezuela.

***Alphomelonrugosum* Shimabukuro & Penteado-Dias, 2003**

*Alphomelonrugosum* Shimabukuro & Penteado-Dias, 2003.

**Type information.** Holotype female, DCBU (not examined but original description checked). Country of type locality: Brazil.

**Geographical distribution.**NEO.

**NEO**: Brazil (DF, SP).

***Alphomelonsimpsonorum* Deans, 2003**

*Alphomelonsimpsonorum* Deans, 2003.

**Type information.** Holotype female, CNC (examined). Country of type locality: Brazil.

**Geographical distribution.**NEO.

**NEO**: Brazil (PR, SC), Costa Rica, Paraguay.

***Alphomelontalidicida* (Wilkinson, 1931)**

*Apantelestalidicida* Wilkinson, 1931.

**Type information.** Holotype female, NHMUK (examined). Country of type locality: Guyana.

**Geographical distribution.**NEO.

**NEO**: Belize, Brazil, Colombia, Costa Rica, Ecuador, Guyana, Mexico, Panama, Peru, Trinidad & Tobago, Venezuela.

***Alphomelonwinniewertzae* Deans, 2003**

*Alphomelonwinniewertzae* Deans, 2003.

**Type information.** Holotype female, USNM (not examined but authoritatively identified specimens examined). Country of type locality: USA.

**Geographical distribution.**NEA, NEO.

**NEA**: Canada (ON, QC), USA (AR, DC, FL, KS, MA, MI, NC, OH, TN, TX, VA); **NEO**: Costa Rica, Mexico.

**Notes.** The specimens we studied were identified by the author of the species.

***Alphomelonxestopyga* Deans, 2003**

*Alphomelonxestopyga* Deans, 2003.

**Type information.** Holotype female, USNM (not examined but paratype examined). Country of type locality: Costa Rica.

**Geographical distribution.**NEO.

**NEO**: Costa Rica.

#### Genus Apanteles Foerster, 1863

***Apanteles*** Foerster, 1863: 245. Gender: masculine. Type species: *Microgasterobscurus* Nees, 1834, by original designation and monotypy.

*Urogaster* Ashmead, 1898: 166. Type species: *Urogastervulgaris* Ashmead, 1898, by subsequent designation (Viereck 1914).

*Holcapanteles* Cameron, 1905: 44. Type species: *Holcapantelessulciscutis* Cameron, 1905, by monotypy. **New synonymy.**

*Xestapanteles* Cameron, 1910: 447. Type species: *Xestapanteleslatiannulatus* Cameron, 1910, by monotypy.

*Cecidobracon* Kieffer & Jörgensen, 1910: 436. Type species: *Cecidobraconasphondyliae* Kieffer & Jörgensen, 1910, by monotypy. **New synonymy**.

*Allapanteles* Brèthes, 1915: 404. Type species: *Allapantelescecidiptae* Brèthes, 1915, by monotypy.

The year of publication of Foerster’s paper, with the original description of *Apanteles*, was until recently almost universally cited as 1862 (e.g., Dalla Torre 1898, Szépligeti 1904, Shenefelt 1972, Marsh 1979a, Yu et al. 2012); however, it has been shown that the actual year of publication was 1863 (Foley et al. 2003), which has been followed by Yu et al. (2016) and it is also accepted here.

The type species of *Holcapanteles* is *H.sulciscutis* Cameron, 1905, from Indonesia. The holotype is apparently lost (Shenefelt 1973, van Achterberg 1980, Mason 1981). The type species of *Cecidobracon* is *C.asphondyliae* Kieffer & Jörgensen, 1910, from Argentina. Unfortunately, the type depository was never stated in the original description, and the specimen has not been located subsequently (Shenefelt 1973, Mason 1981). A second species, *Cecidobraconbraziliensis* Kieffer & Tavares, 1925, was described from Brazil a few years later, but the type depository is also unknown. Without seeing the type specimens it may never be possible to establish with certainty the validity of *Holcapanteles* and *Cecidobracon* as Microgastrinae genera; however, based on the original descriptions, Mason (1981: 26, 27) considered that both genera were likely to be synonyms of *Apanteles*, although he did not formally synonymize the names. After reading the three original descriptions (Cameron 1905a: 44, Kieffer and Jörgensen 1910: 436–437, Kieffer and Tavares 1925: 48), including the associated illustrations of the wings of the two *Cecidobracon* species, we concur with Mason’s opinion and thus formally synonymize both genera under *Apanteles* for the sake of clarity and stability. The three species are also formally transferred below.

Currently *Apanteles* is the largest genus of Microgastrinae with 633 described species from all biogeographical regions (although, interestingly, there are no native species in New Zealand and the genus has not been recorded from the high Arctic). Several regional revisions are available, but some are very outdated and the taxonomic coverage of world species is far from complete. We have seen a large number of undescribed species in collections, mostly from tropical areas, and the actual species richness may well attain several thousand species. The name *Apanteles* was traditionally applied to all species with the fore wing areolet open: subsequently *Apanteles* auctt. has been split into numerous genera starting as early as 1880 and resulting in more than two dozen new genera being proposed since (see Mason 1981, Whitfield et al. 2002b, and Fernandez-Triana et al. 2014e for summaries of the history of *Apanteles* and its different concepts). van Achterberg (2003) synonymised several of these genera under *Apanteles*, thus potentially increasing the number of described species to 1,290 (Fig. 2A; see also Yu et al. 2016); however, we do not follow that arrangement here (Fig. 2B; also, see above, under the section Brief diagnosis of all Microgastrinae genera as they are understood in this paper, a more detailed discussion on the generic limits of the subfamily). Even with the restricted generic concept that we use in this paper, *Apanteles* is still a huge and varied assemblage of species. Nixon (1965) proposed 44 species groups for the world fauna (although that was before Mason (1981) split the genus, meaning some of those groups are not currently in *Apanteles*); and Fernandez-Triana et al. (2014e) proposed 30 new species groups just for Mesoamerica. Many of the *Apanteles* species groups represent monophyletic or at least morphologically cohesive groups, but others are poorly defined, and some are just containers for species that do not fit into any other group. Many families of Lepidoptera have been recorded as hosts for *Apanteles*, but many records are likely to be incorrect and/or need further verification. In Costa Rica most of the known hosts belong to three families: Crambidae, Depresariidae, and Hesperiidae (Fernandez-Triana et al. 2014e; in that paper depressarids were treated as elachistids). There are 7,800+ DNA-barcode compliant sequences of *Apanteles* in BOLD representing almost 600 different BINs, mostly from Costa Rica and North America.

***Apantelesabdera* Nixon, 1965**

*Apantelesabdera* Nixon, 1965.

**Type information.** Holotype female, NHMUK (examined). Country of type locality: South Africa.

**Geographical distribution.**AFR.

**AFR**: Cape Verde, South Africa.

***Apantelesabditus* Muesebeck, 1957**

*Apantelesabditus* Muesebeck, 1957.

**Type information.** Holotype female, USNM (not examined but original description checked). Country of type locality: Brazil.

**Geographical distribution.**NEO.

**NEO**: Brazil (SP), Uruguay, Venezuela.

***Apantelesacoris* Nixon, 1965**

*Apantelesacoris* Nixon, 1965.

**Type information.** Holotype female, NHMUK (examined). Country of type locality: South Africa.

**Geographical distribution.**AFR.

**AFR**: South Africa.

***Apantelesacutissimus* Granger, 1949**

*Apantelesacutissimus* Granger, 1949.

**Type information.** Syntypes female and male, MNHN (not examined but original description checked). Country of type locality: Madagascar.

**Geographical distribution.**AFR.

**AFR**: Madagascar.

**Notes.** The original description mentions 15 female and 16 male specimens but does not explicitly designate a holotype, thus all are here considered to be syntypes.

***Apantelesadelinamoralesae* Fernandez-Triana, 2014**

*Apantelesadelinamoralesae* Fernandez-Triana, 2014.

**Type information.** Holotype female, CNC (examined). Country of type locality: Costa Rica.

**Geographical distribution.**NEO.

**NEO**: Costa Rica.

***Apantelesadoxophyesi* Minamikawa, 1954**

*Apantelesadoxophyesi* Minamikawa, 1954.

**Type information.** Holotype female, depository unknown (not examined but authoritatively identified specimens examined). Country of type locality: Japan.

**Geographical distribution.**OTL, PAL.

**OTL**: China (ZJ); **PAL**: China (AH, SD), Japan.

**Notes.** Our concept of *Apantelesadoxophyesi* is based on two female specimens we examined (EIHU), presumably identified by Watanabe. The digital collection of TARI also contains images of this species, although we could not confirm the accuracy of that identification (https://digiins.tari.gov.tw/tarie/treelist003E.php?id=Brac11122001&lev1=3&lev2=0/1/7/&lev3=01&page=5).

***Apantelesadreus* Nixon, 1965**

*Apantelesadreus* Nixon, 1965.

**Type information.** Holotype female, NHMUK (examined). Country of type locality: South Africa.

**Geographical distribution.**AFR.

**AFR**: South Africa.

***Apantelesadrianachavarriae* Fernandez-Triana, 2014**

*Apantelesadrianachavarriae* Fernandez-Triana, 2014.

**Type information.** Holotype female, CNC (examined). Country of type locality: Costa Rica.

**Geographical distribution.**NEO.

**NEO**: Costa Rica.

***Apantelesadrianaguilarae* Fernandez-Triana, 2014**

*Apantelesadrianaguilarae* Fernandez-Triana, 2014.

**Type information.** Holotype female, CNC (examined). Country of type locality: Costa Rica.

**Geographical distribution.**NEO.

**NEO**: Costa Rica.

***Apantelesadrianguadamuzi* Fernandez-Triana, 2014**

*Apantelesadrianguadamuzi* Fernandez-Triana, 2014.

**Type information.** Holotype female, CNC (examined). Country of type locality: Costa Rica.

**Geographical distribution.**NEO.

**NEO**: Costa Rica.

***Apantelesafer* Wilkinson, 1932**

*Apantelesafer* Wilkinson, 1932.

**Type information.** Holotype female, NHMUK (examined). Country of type locality: Uganda.

**Geographical distribution.**AFR.

**AFR**: Uganda.

***Apantelesagatillus* Nixon, 1965**

*Apantelesagatillus* Nixon, 1965.

**Type information.** Holotype female, NHMUK (examined). Country of type locality: South Africa.

**Geographical distribution.**AFR.

**AFR**: South Africa.

***Apantelesaglaope* Nixon, 1965**

*Apantelesaglaope* Nixon, 1965.

**Type information.** Holotype female, NHMUK (examined). Country of type locality: Indonesia.

**Geographical distribution.**OTL.

**OTL**: Indonesia.

***Apantelesaglaus* Nixon, 1965**

*Apantelesaglaus* Nixon, 1965.

**Type information.** Holotype female, NHMUK (examined). Country of type locality: Fiji.

**Geographical distribution.**AUS.

**AUS**: Fiji.

***Apantelesagrus* Nixon, 1965**

*Apantelesagrus* Nixon, 1965.

**Type information.** Holotype female, NHMUK (examined). Country of type locality: South Africa.

**Geographical distribution.**AFR.

**AFR**: South Africa.

***Apantelesaichagirardae* Fernandez-Triana, 2014**

*Apantelesaichagirardae* Fernandez-Triana, 2014.

**Type information.** Holotype female, CNC (examined). Country of type locality: Costa Rica.

**Geographical distribution.**NEO.

**NEO**: Costa Rica.

***Apantelesaidalopezae* Fernandez-Triana, 2014**

*Apantelesaidalopezae* Fernandez-Triana, 2014.

**Type information.** Holotype female, CNC (examined). Country of type locality: Costa Rica.

**Geographical distribution.**NEO.

**NEO**: Costa Rica.

***Apantelesalaspharus* Nixon, 1965**

*Apantelesalaspharus* Nixon, 1965.

**Type information.** Holotype female, NHMUK (examined). Country of type locality: South Africa.

**Geographical distribution.**AFR.

**AFR**: South Africa.

***Apantelesalastor* de Saeger, 1944**

*Apantelesalastor* de Saeger, 1944.

**Type information.** Holotype female, RMCA (not examined but original description checked). Country of type locality: Democratic Republic of Congo.

**Geographical distribution.**AFR.

**AFR**: Democratic Republic of Congo.

***Apantelesalazoni* Lozan, 2008**

*Apantelesalazoni* Lozan, 2008.

**Type information.** Holotype female, IECA (not examined but original description checked). Country of type locality: Canary Islands.

**Geographical distribution.**PAL.

**PAL**: Canary Islands.

***Apantelesalbanjimenezi* Fernandez-Triana, 2014**

*Apantelesalbanjimenezi* Fernandez-Triana, 2014.

**Type information.** Holotype female, CNC (examined). Country of type locality: Costa Rica.

**Geographical distribution.**NEO.

**NEO**: Costa Rica.

***Apantelesalbinervis* (Cameron, 1904)**

*Urogasteralbinervis* Cameron, 1904.

*Apantelesalbinervicam* Shenefelt, 1972.

**Type information.** Holotype male, NHMUK (examined). Country of type locality: Mexico.

**Geographical distribution.**NEO.

**NEO**: Mexico.

***Apantelesalejandromasisi* Fernandez-Triana, 2014**

*Apantelesalejandromasisi* Fernandez-Triana, 2014.

**Type information.** Holotype female, CNC (examined). Country of type locality: Costa Rica.

**Geographical distribution.**NEO.

**NEO**: Costa Rica.

***Apantelesalejandromorai* Fernandez-Triana, 2014**

*Apantelesalejandromorai* Fernandez-Triana, 2014.

**Type information.** Holotype female, CNC (examined). Country of type locality: Costa Rica.

**Geographical distribution.**NEO.

**NEO**: Costa Rica.

***Apantelesalexanderi* Brèthes, 1922**

*Apantelesalexanderi* Brèthes, 1922.

**Type information.** Lectotype female, MACN (not examined but subsequent treatment of the species checked). Country of type locality: Argentina.

**Geographical distribution.**NEO.

**NEO**: Argentina, Uruguay.

**Notes.** Our concept of *Apantelesalexanderi* is based on Martinez et al. (2012), who examined and designated the lectotype, and provided images and DNA barcodes of the species.

***Apantelesallofulvigaster* Long, 2007**

*Apantelesallofulvigaster* Long, 2007.

**Type information.** Holotype female, VNMN (not examined but original description checked). Country of type locality: Vietnam.

**Geographical distribution.**OTL.

**OTL**: Vietnam.

**Notes.** The holotype depository was not stated in the English version of the original description (Long 2007). That paper was written in two languages, the first part in Vietnamese, followed by a second part in English; based on the extent of both versions, we suspect that the English part is just a translation from the Vietnamese. However, we do not know if it is a literal translation or just a summarized (= shorter) version; thus, we do not know if the holotype depository is mentioned in the Vietnamese part of the paper. If the holotype was not stated in the Vietnamese version, then this species name would be unavailable (a subsequent paper (Long and Achterberg 2014) records the holotype depository; however, that alone does not comply with the ICZN requirements and would not make the name available). Although we have not been able to establish with certainty what is stated in the Vietnamese part of Long (2007), we provisionally consider here the species name as available.

***Apantelesalvarougaldei* Fernandez-Triana, 2014**

**Type information.** Holotype female, CNC (examined). Country of type locality: Costa Rica.

**Geographical distribution.**NEO.

**NEO**: Costa Rica.

***Apantelesanabellecordobae* Fernandez-Triana, 2014**

*Apantelesanabellecordobae* Fernandez-Triana, 2014.

**Type information.** Holotype female, CNC (examined). Country of type locality: Costa Rica.

**Geographical distribution.**NEO.

**NEO**: Costa Rica.

***Apantelesanamarencoae* Fernandez-Triana, 2014**

*Apantelesanamarencoae* Fernandez-Triana, 2014.

**Type information.** Holotype female, CNC (examined). Country of type locality: Costa Rica.

**Geographical distribution.**NEO.

**NEO**: Costa Rica.

***Apantelesanamartinezae* Fernandez-Triana, 2014**

*Apantelesanamartinezae* Fernandez-Triana, 2014.

*Apantelesanamartinesae* Fernandez-Triana, 2014 [incorrect original spelling].

**Type information.** Holotype female, CNC (examined). Country of type locality: Costa Rica.

**Geographical distribution.**NEO.

**NEO**: Costa Rica.

**Notes.** In the paper where this species was originally described, the name was spelled in two different ways: as *anamartinezae* (in the species list of Table 3, species description, references to ZooBank and caption of Figure 227) or as *anamartinesae* (in the Abstract, key to species, and caption to Figure 25). The correct spelling is obviously *anamartinezae*, as the species was named after Ana Martínez, and it is the one to be preserved, following Article 32 of the ICZN.

***Apantelesanariasae* Fernandez-Triana, 2014**

*Apantelesanariasae* Fernandez-Triana, 2014.

**Type information.** Holotype female, CNC (examined). Country of type locality: Costa Rica.

**Geographical distribution.**NEO.

**NEO**: Costa Rica.

***Apantelesanatole* Nixon, 1965**

*Apantelesanatole* Nixon, 1965.

**Type information.** Holotype female, NHMUK (examined). Country of type locality: South Africa.

**Geographical distribution.**AFR.

**AFR**: South Africa.

**Notes.** The holotype specimen has the vannal lobe with very few, very sparse setae across lobe length.

***Apantelesandreacalvoae* Fernandez-Triana, 2014**

*Apantelesandreacalvoae* Fernandez-Triana, 2014.

**Type information.** Holotype female, CNC (examined). Country of type locality: Costa Rica.

**Geographical distribution.**NEO.

**NEO**: Costa Rica.

***Apantelesangaleti* Muesebeck, 1956**

*Apantelesangaleti* Muesebeck, 1956.

**Type information.** Holotype female, USNM (examined). Country of type locality: India.

**Geographical distribution.**AFR, OTL, PAL.

**AFR**: Kenya; **OTL**: China (SN, ZJ), India, Indonesia, Pakistan, Vietnam; **PAL**: Iraq.

**Notes.** Introduced into Mexico and the USA (e.g., Mcgough and Noble 1957, Bartlett et al. 1978). In total more than 150,000 specimens were released but the species was never recovered in any of the USA states where it was released (Mcgough and Noble 1957), and a subsequent citation of the species from Mexico (Coronado-Blanco et al. 2004) is merely a repetition of the information cited in older references, not a confirmation of the species’ presence in the country. Thus, in this paper we do not consider *A.angaleti* as an established species in the Nearctic or Neotropical regions.

***Apantelesangelsolisi* Fernandez-Triana, 2014**

*Apantelesangelsolisi* Fernandez-Triana, 2014.

**Type information.** Holotype female, CNC (examined). Country of type locality: Costa Rica.

**Geographical distribution.**NEO.

**NEO**: Costa Rica.

***Apantelesangulatus* Granger, 1949**

*Apantelesangulatus* Granger, 1949.

**Type information.** Syntypes female and male, MNHN (not examined but original description checked). Country of type locality: Madagascar.

**Geographical distribution.**AFR.

**AFR**: Madagascar.

***Apantelesangustibasis* Gahan, 1925**

*Apantelesangustibasis* Gahan, 1925.

**Type information.** Holotype female, USNM (examined). Country of type locality: Philippines.

**Geographical distribution.**OTL.

**OTL**: China (HN), India, Malaysia, Pakistan, Philippines, Vietnam.

**Notes.** This species was transferred to *Cotesia* by Gupta and Pawar (1992), a non-taxonomic paper, in which it could be argued that those authors did not study the holotype. We have studied the holotype as well as illustrations of specimens from Malaysia identified by C. Watanabe that are deposited in EIHU. Both the holotype and the Malaysian specimens are clearly not *Cotesia* but *Apanteles*, and thus we restore the combination of this species here.

***Apantelesanodaphus* Nixon, 1965**

*Apantelesanodaphus* Nixon, 1965.

**Type information.** Holotype female, NHMUK (examined). Country of type locality: Papua New Guinea.

**Geographical distribution.**AUS.

**AUS**: Papua New Guinea.

***Apantelesansata* Song & Chen, 2004**

*Apantelesansata* Song & Chen, 2004.

**Type information.** Holotype female, FAFU (not examined but original description checked). Country of type locality: China.

**Geographical distribution.**OTL.

**OTL**: China (FJ).

***Apantelesanthozelae* de Saeger, 1941**

*Apantelesanthozelae* de Saeger, 1941.

**Type information.** Holotype female, RMCA (not examined but original description checked). Country of type locality: Democratic Republic of Congo.

**Geographical distribution.**AFR.

**AFR**: Democratic Republic of Congo.

***Apantelesanticlea* Nixon, 1965**

*Apantelesanticlea* Nixon, 1965

**Type information.** Holotype female, USNM (examined). Country of type locality: Malaysia.

**Geographical distribution.**OTL.

**OTL**: Malaysia.

***Apantelesantilla* Nixon, 1965**

*Apantelesantilla* Nixon, 1965.

**Type information.** Holotype female, NHMUK (examined). Country of type locality: South Africa.

**Geographical distribution.**AFR.

**AFR**: South Africa.

***Apantelesarachidis* Risbec, 1951**

*Apantelesarachidis* Risbec, 1951.

**Type information.** Holotype male, MNHN (not examined but original description checked). Country of type locality: Senegal.

**Geographical distribution.**AFR.

**AFR**: Senegal.

**Notes.** The original description is not clear enough to determine the correct generic placement of the species, thus is best kept in the genus it was originally described. Future study of the type specimen may change its current generic status.

***Apantelesaraeceri* Wilkinson, 1928**

*Apantelesaraeceri* Wilkinson, 1928.

**Type information.** Holotype female, NHMUK (examined). Country of type locality: Indonesia.

**Geographical distribution.**OTL.

**OTL**: India, Indonesia, Malaysia.

***Apantelesaragatzi* Tobias, 1976**

*Apantelesaragatzi* Tobias, 1976.

**Type information.** Holotype female, depository unknown (not examined but subsequent treatment of the species checked). Country of type locality: Armenia.

**Geographical distribution.**PAL.

**PAL**: Armenia, Russia (KDA), Sweden, Turkey.

**Notes.** Our concept of the species is based on the descriptions provided by Papp (1984a) and Tobias (1986).

***Apantelesarielopezi* Fernandez-Triana, 2014**

*Apantelesarielopezi* Fernandez-Triana, 2014.

**Type information.** Holotype female, CNC (examined). Country of type locality: Costa Rica.

**Geographical distribution.**NEO.

**NEO**: Costa Rica.

***Apantelesarion* Nixon, 1965**

*Apantelesarion* Nixon, 1965.

**Type information.** Holotype female, NHMUK (examined). Country of type locality: South Africa.

**Geographical distribution.**AFR.

**AFR**: South Africa.

***Apantelesariovistus* Nixon, 1965**

*Apantelesariovistus* Nixon, 1965.

**Type information.** Holotype female, NHMUK (examined). Country of type locality: Indonesia.

**Geographical distribution.**OTL.

**OTL**: Indonesia.

***Apantelesaristaeus* Nixon, 1965**

*Apantelesaristaeus* Nixon, 1965.

**Type information.** Holotype female, NHMUK (examined). Country of type locality: India.

**Geographical distribution.**OTL.

**OTL**: China (TW), India, Indonesia.

***Apantelesaristoteliae* Viereck, 1912**

*Apantelesaristoteliae* Viereck, 1912.

*Apantelesgelechiae* Viereck, 1912.

**Type information.** Holotype male, USNM (examined). Country of type locality: USA.

**Geographical distribution.**NEA.

**NEA**: Canada (NB, ON, QC), USA (AZ, CA, CO, CT, KS, LA, MI, NJ, NY, NC, OH, OR, PA, TX, UT, VT, WA).

***Apantelesarsanes* Nixon, 1965**

*Apantelesarsanes* Nixon, 1965.

**Type information.** Holotype female, NHMUK (examined). Country of type locality: Kenya.

**Geographical distribution.**AFR.

**AFR**: Kenya.

**Notes.** Despite its relatively short ovipositor sheaths, we are retaining this species in *Apanteles* because of its pleated hypopygium, strongly concave vannal lobe lacking setae, and anteromesoscutum punctures which are fusing near scutoscutellar disc.

***Apantelesarticas* Nixon, 1965**

*Apantelesarticas* Nixon, 1965.

**Type information.** Holotype female, NHMUK (examined). Country of type locality: Senegal.

**Geographical distribution.**AFR, PAL.

**AFR**: Senegal; **PAL**: Israel, Tunisia, Turkey.

***Apantelesartustigma* Liu & Chen, 2015**

*Apantelesartustigma* Liu & Chen, 2015.

**Type information.** Holotype female, ZJUH (not examined but original description checked). Country of type locality: China.

**Geographical distribution.**OTL.

**OTL**: China (GD, ZJ).

***Apantelesarundinariae* de Saeger, 1944**

*Apantelesarundinariae* de Saeger, 1944.

**Type information.** Holotype female, RMCA (not examined but original description checked). Country of type locality: Democratic Republic of Congo.

**Geographical distribution.**AFR.

**AFR**: Democratic Republic of Congo, Rwanda.

***Apantelesasphondyliae* (Kieffer & Jörgensen, 1910), new combination**

*Cecidobraconasphondyliae* Kieffer & Jörgensen, 1910.

**Type information.** Holotype male, lost (not examined but original description checked). Country of type locality: Argentina.

**Geographical distribution.**NEO.

**NEO**: Argentina.

**Notes.** The type depository was not stated in the original description, and the specimen has never been located (Shenefelt 1973, Mason 1981). See comments at the beginning of *Apanteles* for details on the decision to transfer this species to *Apanteles*.

***Apantelesassis* Nixon, 1965**

*Apantelesassis* Nixon, 1965.

**Type information.** Holotype female, USNM (examined). Country of type locality: Philippines.

**Geographical distribution.**OTL.

**OTL**: Philippines, Vietnam.

***Apantelesatrocephalus* Granger, 1949**

*Apantelesatrocephalus* Granger, 1949.

**Type information.** Holotype female, MNHN (not examined but original description checked). Country of type locality: Madagascar.

**Geographical distribution.**AFR.

**AFR**: Madagascar.

**Notes.** Based on some morphological features described by Granger (1949), e.g., the areolated propodeum, shape and sculpture of T1–T3, acute hypopygium, ovipositor sheaths half the metatibia length, we think that this species could potentially be placed in one of the following genera: *Apanteles*, *Parapanteles*, or *Cotesia*. Because the original description (the only source available, apart from the single known specimen, which we could not examine), is not sufficient to determine the correct generic placement, we maintain *atrocephalus* within the genus in which it was originally described.

***Apantelesattevae* Yousuf, Hassan & Singh, 2008**

*Apantelesattevae* Yousuf, Hassan & Singh, 2008.

**Type information.** Holotype female, TFRI (not examined but original description checked). Country of type locality: India.

**Geographical distribution.**OTL.

**OTL**: India.

***Apantelesaudens* Kotenko, 1986**

*Apantelesaudens* Kotenko, 1986.

**Type information.** Holotype female?, ZIN (not examined but original description checked). Country of type locality: Georgia.

**Geographical distribution.**PAL.

**PAL**: Georgia, Russia (NC).

**Notes.** The paper in which the original description is included does not clarify the sex of the type material, nor is it specified if there is a holotype (or syntypes) on which the species description was based (Tobias 1986: 805). Without examining the actual specimen(s) is impossible to determine its sex or type status; however, in the Foreword section of the paper (Tobias 1986: page numbered as ix) it is stated that, to comply with nomenclature rules, the type material is specified for all species. The author then explicitly says that the paper includes lectotype and paralectotype designations for species described from the USSR in the past. Such a statement allows the assumption that all new species descriptions must have been based on holotypes – and not a type series (syntypes) as was presumably done in the past. Thus, we are assuming that there is a holotype for *Apantelesaudens* Kotenko, 1986. Regarding the sex of the type, again only assumptions can be made until the specimen is examined, but the key is based on female specimens, including a brief original description that mentions the ovipositor sheaths. Thus, we consider here as very likely that the holotype is a female but add a question mark to clarify that it is only an educated guess.

***Apantelesaurangabadensis* Rao & Chalikwar, 1970**

*Apantelesaurangabadensis* Rao & Chalikwar, 1970.

**Type information.** Holotype male, NZSI (not examined but original description checked). Country of type locality: India.

**Geographical distribution.**OTL.

**OTL**: India.

***Apantelesazollae* Sumodan & Sevichan, 1989**

*Apantelesazollae* Sumodan & Sevichan, 1989.

**Type information.** Holotype female, RMNH (not examined but subsequent treatment of the species checked). Country of type locality: India.

**Geographical distribution.**OTL.

**OTL**: India.

**Notes.** See van Achterberg and Narendran (1997) for details about the type, and for the generic placement of the species. *Apantelesazollae* has been misspelled twice, as *azolae* and *azolla*, as previously noted by Yu et al. (2016).

***Apantelesbajariae* Papp, 1975**

*Apantelesbajariae* Papp, 1975.

**Type information.** Holotype female, HNHM (not examined but original description checked). Country of type locality: Hungary.

**Geographical distribution.**PAL.

**PAL**: Bulgaria, Canary Islands, Greece, Hungary, Montenegro, Turkey.

**Notes.** Based on the position this species occupies in the key of Papp (1984a), it is possible that *bajariae* would actually belong to *Dolichogenidea*. However, the details in both the original description and Papp (1984a) are not definite to conclude with certainty, thus it is here kept in the genus it was originally described.

***Apantelesbaldufi* Muesebeck, 1968**

*Apantelesbaldufi* Muesebeck, 1968.

**Type information.** Holotype female, USNM (not examined but original description checked). Country of type locality: USA.

**Geographical distribution.**NEA.

**NEA**: Canada (ON), USA (MI, MN).

***Apantelesbalteatae* Lal, 1942**

*Apantelesbalteatae* Lal, 1942.

**Type information.** Holotype male, INPC (not examined but original description checked). Country of type locality: India.

**Geographical distribution.**OTL.

**OTL**: India.

***Apantelesbalthazari* (Ashmead, 1900)**

*Urogasterbalthazari* Ashmead, 1900.

*Urogastermeridionalis* Ashmead, 1900.

*Apantelesmeridionalis* Ashmead, 1900.

**Type information.** Holotype female, NHMUK (examined). Country of type locality: Saint Vincent.

**Geographical distribution.**NEO.

**NEO**: Brazil (CE, PA, PB, PE, RN, SP), Cuba, Grenada, Saint Vincent.

**Notes.** The original description (Ashmead 1900c) does not match the holotype, as his description of the T1 shape, T2 sculpture and colouration of meso- and metafemora are completely different from the actual specimen examined (see Fernandez-Triana et al. 2014e).

***Apantelesbannaensis* Song, Chen & Yang, 2001**

*Apantelesbannaensis* Song, Chen & Yang, 2001.

**Type information.** Holotype female, FAFU (not examined but subsequent treatment of the species checked). Country of type locality: China.

**Geographical distribution.**OTL.

**OTL**: China (YN).

**Notes.** Our species concept is based on Chen and Song (2004).

***Apantelesbaoli* Risbec, 1951**

*Apantelesbaoli* Risbec, 1951.

**Type information.** Holotype male, depository unknown (not examined but original description checked). Country of type locality: Senegal.

**Geographical distribution.**AFR.

**AFR**: Senegal.

***Apantelesbasicavus* Liu & Chen, 2015**

*Apantelesbasicavus* Liu & Chen, 2015.

**Type information.** Holotype female, ZJUH (not examined but original description checked). Country of type locality: China.

**Geographical distribution.**PAL.

**PAL**: China (JL, LN).

***Apantelesbellatulus* de Saeger, 1944**

*Apantelesbellatulus* de Saeger, 1944.

**Type information.** Holotype female, RMCA (not examined but original description checked). Country of type locality: Democratic Republic of Congo.

**Geographical distribution.**AFR.

**AFR**: Democratic Republic of Congo.

***Apantelesbernardoespinozai* Fernandez-Triana, 2014**

*Apantelesbernardoespinozai* Fernandez-Triana, 2014.

**Type information.** Holotype female, CNC (examined). Country of type locality: Costa Rica.

**Geographical distribution.**NEO.

**NEO**: Costa Rica.

***Apantelesbernyapui* Fernandez-Triana, 2014**

*Apantelesbernyapui* Fernandez-Triana, 2014.

**Type information.** Holotype female, CNC (examined). Country of type locality: Costa Rica.

**Geographical distribution.**NEO.

**NEO**: Costa Rica.

***Apantelesbettymarchenae* Fernandez-Triana, 2014**

*Apantelesbettymarchenae* Fernandez-Triana, 2014.

**Type information.** Holotype female, CNC (examined). Country of type locality: Costa Rica.

**Geographical distribution.**NEO.

**NEO**: Costa Rica.

***Apantelesbienvenidachavarriae* Fernandez-Triana, 2014**

*Apantelesbienvenidachavarriae* Fernandez-Triana, 2014.

**Type information.** Holotype female, CNC (examined). Country of type locality: Costa Rica.

**Geographical distribution.**NEO.

**NEO**: Costa Rica.

***Apantelesbiroicus* Papp, 1973**

*Apantelesbiroicus* Papp, 1973.

**Type information.** Holotype female, HNHM (not examined but paratype examined). Country of type locality: Hungary.

**Geographical distribution.**PAL.

**PAL**: Hungary, Romania, Tunisia.

**Notes.** This species was transferred from *Apanteles* to *Illidops* by Papp (1988), but examination of two paratype specimens in the CNC revealed that those specimens do not have a median band of rugosity posteriorly on the scutellum, and the propodeum sculpture is also different from that found in *Illidops* (*sensu*Fernandez-Triana et al. 2014e). Thus, here we transfer the species back to *Apanteles*.

***Apantelesbitalensis* de Saeger, 1944**

*Apantelesbitalensis* de Saeger, 1944.

**Type information.** Syntypes female, RMCA (not examined but original description checked). Country of type locality: Rwanda.

**Geographical distribution.**AFR.

**AFR**: Democratic Republic of Congo, Rwanda.

***Apantelesbordagei* Giard, 1898**

*Apantelesbordagei* Giard, 1898.

**Type information.** Type lost (not examined but original description checked). Country of type locality: Réunion.

**Geographical distribution.**AFR.

**AFR**: Democratic Republic of Congo, Kenya, Réunion, Tanzania.

**Notes.** The year of description for this species has been incorrectly cited as 1902 by most authors (e.g., Granger 1949, Shenefelt 1972, Rousse and Gupta 2013, Yu et al. 2016), in all cases based on Giard (1902: 22). Having read that paper, it is clear that it only refers to the species as being described by the author in a previous work (Giard 1898: 202, which we have also read). This was correctly mentioned by de Saeger (1944: 316) and Wilkinson (1934: 150). Wilkinson comprehensively redescribed the species, based on specimens from Kenya and Tanzania, and he considered the type(s) to be lost based on his enquiry to a curator of the MNHN at the time, who could not find the specimen(s). Subsequent authors have provided shorter redescriptions, based on specimens from Democratic Republic of Congo (de Saeger 1944), Madagascar (Granger 1949), or Réunion (Rousse and Gupta 2013). Our species concept is based on Wilkinson (1934). We accept the following comments from Madl and van Achterberg (2014): “Known from the Afrotropical region. The record from Madagascar mentioned in Risbec (1960: 629) is doubtful. Brénière (1965b: 347) mentions *Apantelesbordagei* from Madagascar, citing Granger (1949: 359) as reference, but Granger recorded this species only from Réunion and Africa. The record from Madagascar in Appert et al. (1969: 568) is based on Brénière (1965b)”. Consequently, here we do not consider Madagascar as a country where this species is found.

***Apantelesbrachmiae* Bhatnagar, 1950**

*Apantelesbrachmiae* Bhatnagar, 1950.

**Type information.** Holotype female, INPC (not examined but original description checked). Country of type locality: India.

**Geographical distribution.**OTL.

**OTL**: India.

**Notes.** The year of publication of the Bhatnagar paper was until recently commonly cited as 1948 and/or 1950 (e.g., Chen and Song 2004, Yu et al. 2016), probably following Shenefelt (1972) who referred to this paper as “Bhatnagar (1948) 1950”. While the intended year for Volume X, Parts I & II of the Indian Journal of Entomology was 1948, the actual dates of publication were June 1950 (Part I) and October 1950 (Part II), as clearly shown on the cover page of the Volume, which we have checked. Because the dates of publication are the ones to be considered, and for the sake of clarity, we hereby revise the species year of description to 1950.

***Apantelesbraziliensis* (Kieffer & Tavares, 1925), new combination**

*Cecidobraconbraziliensis* Kieffer & Tavares, 1925.

**Type information.** Type and depository unknown (not examined but original description checked). Country of type locality: Brazil.

**Geographical distribution.**NEO.

**NEO**: Brazil (BA).

**Notes.** The type depository was not given in the original description, and the specimen has never been located (Shenefelt 1973, Mason 1981). See comments at the beginning of *Apanteles* for details on the decision to transfer this species to *Apanteles* (p 74, 75).

***Apantelesbredoi* de Saeger, 1941**

*Apantelesbredoi* de Saeger, 1941.

**Type information.** Holotype female, RMCA (not examined but original description checked). Country of type locality: Democratic Republic of Congo.

**Geographical distribution.**AFR.

**AFR**: Democratic Republic of Congo, Senegal.

***Apantelesbrethesi* Porter, 1917**

*Apantelesbrethesi* Porter, 1917.

**Type information.** Type and depository unknown (not examined). Country of type locality: Chile.

**Geographical distribution.**NEO.

**NEO**: Chile.

***Apantelesbrevicarinis* Song, 2002**

*Apantelesbrevicarinis* Song, 2002.

**Type information.** Holotype female, FAFU (not examined but subsequent treatment of the species checked). Country of type locality: China.

**Geographical distribution.**OTL.

**OTL**: China (HB).

**Notes.** Our concept of this species is based on Chen and Song (2004).

***Apantelesbrevimetacarpus* Hedqvist, 1965**

*Apantelesbrevimetacarpus* Hedqvist, 1965.

*Illidopsmetacarpus* Hedqvist, 1965 [subsequent misspelling (Papp 2003)].

**Type information.** Holotype female, MZH (examined). Country of type locality: Cape Verde.

**Geographical distribution.**AFR, PAL.

**AFR**: Cape Verde; **PAL**: Tunisia.

**Notes.**Papp (2003: 145) transferred this species to *Illidops* (although he misspelled the species name as *metacarpus*). A subsequent paper, also treating the species and reporting it for the first time from Tunisia, continued to place it within *Illidops* (Papp 2014). We examined the female holotype and a male paratype, and they clearly are not *Illidops*. The only feature that would suggest placement in that genus is the short vein R1 (metacarp), but that is known in several species of both *Apanteles* and *Dolichogenidea*. The posteromedian band of the scutellum is smooth. The propodeum, although without an areola, has a weak impression in its place, and its overall weak sculpture is not like that found in *Illidops*. Based on the hind wing, with a slightly concave vannal lobe lacking setae, the best generic placement for this species is *Apanteles*. This concurs with Forshage et al. (2016), although those authors were probably not aware of the two papers by Papp and were following the treatment of the original description. In any case, the statement by Forshage et al. (2016) that the holotype and paratype were missing is here updated, as in 2018 we found the specimens in the MZH.

***Apantelesbrevivena* Liu & Chen, 2015**

*Apantelesbrevivena* Liu & Chen, 2015.

**Type information.** Holotype female, ZJUH (not examined but original description checked). Country of type locality: China.

**Geographical distribution.**PAL.

**PAL**: China (XJ, LN, JL, NM, SD).

***Apantelesbruchi* Blanchard, 1941**

*Apantelesbruchi* Blanchard, 1941.

**Type information.** Type lost (not examined but subsequent treatment of the species checked). Country of type locality: Argentina.

**Geographical distribution.**NEO.

**NEO**: Argentina, Peru.

**Notes.** Our concept of this species is based on Aquino et al. (2010), including details on the fate of the type material.

***Apantelesbrunnistigma* Abdinbekova, 1969**

*Apantelesbrunnistigma* Abdinbekova, 1969.

*Apantelessotades* Nixon, 1976.

**Type information.** Holotype female, ZIN (not examined but authoritatively identified specimens examined). Country of type locality: Azerbaijan.

**Geographical distribution.**NEA, PAL.

**NEA**: Canada (MB, NL, NT, ON, YT); **PAL**: Azerbaijan, Canary Islands, Czech Republic, Finland, France, Germany, Hungary, Iran, Italy, Korea, Lithuania, Russia (ZAB, PRI, TOM), Sweden, Switzerland, Turkey, United Kingdom, Ukraine.

**Notes.** Our concept of this species is based on Fernandez-Triana et al. (2014c). We have also examined the type of *Apantelessotades* Nixon. New data from specimens with sequences in BOLD expand the species distribution within the Nearctic (northwestern Canada) as well as the Palearctic (Germany, Ukraine).

***Apantelesbrunnus* Rao & Chalikwar, 1976**

*Apantelesbrunnus* Rao & Chalikwar, 1976.

**Type information.** Holotype female, BAMU (not examined but original description checked). Country of type locality: India.

**Geographical distribution.**OTL.

**OTL**: India.

***Apantelesburunganus* de Saeger, 1944**

*Apantelesburunganus* de Saeger, 1944.

**Type information.** Holotype female, RMCA (not examined but original description checked). Country of type locality: Democratic Republic of Congo.

**Geographical distribution.**AFR.

**AFR**: Democratic Republic of Congo.

**Notes.** The original description does not provide enough detail to place this species in a genus unambiguously (it could be *Apanteles* but also *Dolichogenidea*). Until the type series is studied, we retain it in the genus in which it was originally described.

***Apantelescaesar* Wilkinson, 1938**

*Apantelescaesar* Wilkinson, 1938.

**Type information.** Holotype female, NHMUK (examined). Country of type locality: Namibia.

**Geographical distribution.**AFR.

**AFR**: Namibia, South Africa.

**Notes.** This species bears some resemblance to the two described species currently placed within *Napamus*. It shares with them the dark colour, infumate wings, elongate mouth parts (especially very long glossa and galea), and relatively short fore wing vein R1 (although not as short as in the two described *Napamus*). However, we retain *caesar* within *Apanteles* because it has some differences in propodeum sculpture (which is mostly smooth, having only small carinae near the nucha), metatibial spines (which are not as long as in *Napamus*) and the disparate geographic distribution of the known species.

***Apantelescalixtomoragai* Fernandez-Triana, 2014**

*Apantelescalixtomoragai* Fernandez-Triana, 2014.

**Type information.** Holotype female, CNC (examined). Country of type locality: Costa Rica.

**Geographical distribution.**NEO.

**NEO**: Costa Rica.

***Apantelescalycinae* Wilkinson, 1928**

*Apantelescalycinae* Wilkinson, 1928.

**Type information.** Holotype female, NHMUK (examined). Country of type locality: India.

**Geographical distribution.**OTL.

**OTL**: India, Vietnam.

***Apantelescamilla* Nixon, 1965**

*Apantelescamilla* Nixon, 1965.

**Type information.** Holotype female, NHMUK (examined). Country of type locality: India.

**Geographical distribution.**OTL.

**OTL**: India.

***Apantelescamirus* Nixon, 1965**

*Apantelescamirus* Nixon, 1965.

**Type information.** Holotype female, NHMUK (examined). Country of type locality: South Africa.

**Geographical distribution.**AFR.

**AFR**: South Africa.

***Apantelescanarsiae* Ashmead, 1898**

*Apantelescanarsiae* Ashmead, 1898.

*Apanteleshousatannuckorum* Viereck, 1917.

*Apantelesmaquinnai* Viereck, 1917.

**Type information.** Holotype female, USNM (examined). Country of type locality: USA.

**Geographical distribution.**NEA.

**NEA**: Canada (ON, QC), USA (AR, CT, DC, IL, IN, IA, KS, NY, VA).

**Notes.** We examined the holotype female of *housatannuckorum* and the holotype male of *maquinnai*, both currently considered as synonyms of *A.canarsiae*. All three holotypes are in the USNM and not in INHS as stated in Yu et al. (2016).

***Apantelescarloscastilloi* Fernandez-Triana, 2014**

*Apantelescarloscastilloi* Fernandez-Triana, 2014.

**Type information.** Holotype female, CNC (examined). Country of type locality: Costa Rica.

**Geographical distribution.**NEO.

**NEO**: Costa Rica.

***Apantelescarlosguadamuzi* Fernandez-Triana, 2014**

*Apantelescarlosguadamuzi* Fernandez-Triana, 2014.

**Type information.** Holotype female, CNC (examined). Country of type locality: Costa Rica.

**Geographical distribution.**NEO.

**NEO**: Costa Rica.

***Apantelescarlosrodriguezi* Fernandez-Triana, 2014**

*Apantelescarlosrodriguezi* Fernandez-Triana, 2014.

**Type information.** Holotype female, CNC (examined). Country of type locality: Costa Rica.

**Geographical distribution.**NEO.

**NEO**: Costa Rica.

***Apantelescarlosviquezi* Fernandez-Triana, 2014**

*Apantelescarlosviquezi* Fernandez-Triana, 2014.

**Type information.** Holotype female, CNC (examined). Country of type locality: Costa Rica.

**Geographical distribution.**NEO.

**NEO**: Costa Rica.

***Apantelescarloszunigai* Fernandez-Triana, 2014**

*Apantelescarloszunigai* Fernandez-Triana, 2014.

**Type information.** Holotype female, CNC (examined). Country of type locality: Costa Rica.

**Geographical distribution.**NEO.

**NEO**: Costa Rica.

***Apantelescarolinacanoae* Fernandez-Triana, 2014**

*Apantelescarolinacanoae* Fernandez-Triana, 2014.

**Type information.** Holotype female, CNC (examined). Country of type locality: Costa Rica.

**Geographical distribution.**NEO.

**NEO**: Costa Rica.

***Apantelescarpatus* (Say, 1836)**

*Microgastercarpata* Say, 1836.

*Urogastersolitarius* Ashmead, 1900.

*Protapanteleshawaiiensis* Ashmead, 1901.

*Urogasterfuscicornis* Cameron, 1910.

*Apantelespiceoventris* Muesebeck, 1921.

*Apantelesigae* Watanabe, 1932.

*Apantelessarcitorius* Telenga, 1955.

*Apantelesultericus* Telenga, 1955.

**Type information.** Holotype female, lost (not examined but subsequent treatment of the species checked). Country of type locality: USA.

**Geographical distribution.**AFR, AUS, NEA, NEO, OTL, PAL.

**AFR**: Democratic Republic of Congo, Ghana, Mozambique, South Africa, Tanzania; **AUS**: Australia (QLD), Fiji, Hawaiian Islands, New Zealand; **NEA**: Canada (AB, BC, NB, NL, ON, PE, QC, SK), USA (CO, CT, DE, IL, IN, MD, MA, MI, MO, NH, NJ, NY, SC, TX, VA); **NEO**: Argentina, Bermuda, Brazil (SP), Cuba, Grenada, Peru, Puerto Rico; **OTL**: China (SN, TW, ZJ), Malaysia, Vietnam; **PAL**: Armenia, Croatia, Finland, France, Germany, Greece, Hungary, Iran, Israel, Japan, Kazakhstan, Latvia, Lithuania, Malta, Moldova, Mongolia, Poland, Romania, Russia (AMU, AST, KHA, PRI, SAK), Serbia, Spain, Switzerland, Turkey, Turkmenistan, United Kingdom, Uzbekistan.

**Notes.** We examined the types of two of the seven currently accepted synonyms of *carpatus*: *hawaiiensis* (in USNM) and *solitarius* (in NHMUK). If *Apantelescarpatus* is ever going to be split into several species, the type of *hawaiiensis* would be a candidate to be considered as a different species, supported by morphological differences when compared to other *Apantelescarpatus* specimens and also through different host associations. We also examined one female (in EIHU, identified by Muesebeck) which also looks different to the traditional *carpatus* and could represent yet another species. Determining the limits of *A.carpatus* is beyond the scope of this paper and at present we leave all of the examined specimens as a single species.

***Apantelescassiae* Chalikwar & Rao, 1982**

*Apantelescassiae* Chalikwar & Rao, 1982.

**Type information.** Type and depository unknown (not examined). Country of type locality: India.

**Geographical distribution.**OTL.

**OTL**: India.

***Apantelescato* de Saeger, 1944**

*Apantelescato* de Saeger, 1944.

**Type information.** Holotype female, RMCA (not examined but original description checked). Country of type locality: Democratic Republic of Congo.

**Geographical distribution.**AFR.

**AFR**: Democratic Republic of Congo, Rwanda.

***Apantelescavatiptera* Chen & Song, 2004**

*Apantelescavatiptera* Chen & Song, 2004.

**Type information.** Holotype female, FAFU (not examined but original description checked). Country of type locality: China.

**Geographical distribution.**OTL.

**OTL**: China (FJ, YN).

***Apantelescavatithoracicus* Chen, 2001**

*Apantelescavatithoracica* Chen, 2001.

**Type information.** Holotype female, FAFU (not examined but subsequent treatment of the species checked). Country of type locality: China.

**Geographical distribution.**OTL.

**OTL**: China (FJ, HB).

**Notes.** For the generic placement of this species we follow Chen and Song (2004).

***Apantelescavifrons* Nixon, 1965**

*Apantelescavifrons* Nixon, 1965.

**Type information.** Holotype female, USNM (examined). Country of type locality: Philippines.

**Geographical distribution.**OTL.

**OTL**: Philippines.

***Apantelescebes* Nixon, 1965**

*Apantelescebes* Nixon, 1965.

**Type information.** Holotype female, USNM (examined). Country of type locality: Philippines.

**Geographical distribution.**OTL.

**OTL**: Philippines.

***Apantelescecidiptae* (Brèthes, 1916)**

*Allapantelescecidiptae* Brèthes, 1916.

**Type information.** Syntypes female and male, MACN (not examined). Country of type locality: Argentina.

**Geographical distribution.**NEO.

**NEO**: Argentina.

***Apantelescerberus* Nixon, 1965**

*Apantelescerberus* Nixon, 1965.

**Type information.** Holotype female, NHMUK (examined). Country of type locality: India.

**Geographical distribution.**OTL.

**OTL**: India.

***Apantelescestius* Nixon, 1965**

*Apantelescestius* Nixon, 1965.

**Type information.** Holotype female, USNM (examined). Country of type locality: Philippines.

**Geographical distribution.**OTL.

**OTL**: Philippines.

***Apanteleschalcomelas* Nixon, 1965**

*Apanteleschalcomelas* Nixon, 1965.

**Type information.** Holotype female, NHMUK (examined). Country of type locality: South Africa.

**Geographical distribution.**AFR.

**AFR**: South Africa.

***Apanteleschanghingensis* Chu, 1937**

*Apanteleschanghingensis* Chu, 1937.

**Type information.** Holotype female, depository unknown (not examined but subsequent treatment of the species checked). Country of type locality: China.

**Geographical distribution.**OTL.

**OTL**: China (FJ, ZJ).

**Notes.** For the generic placement of this species we follow Chen and Song (2004).

***Apantelescharacomae* Risbec, 1951**

*Apantelescharacomae* Risbec, 1951.

**Type information.** Holotype male, depository unknown (not examined but original description checked). Country of type locality: Ivory Coast.

**Geographical distribution.**AFR.

**AFR**: Ivory Coast.

***Apanteleschatterjeei* Sharma & Chatterjee, 1970**

*Apanteleschatterjeei* Sharma & Chatterjee, 1970.

**Type information.** Holotype female, IFRI (not examined but original description checked). Country of type locality: India.

**Geographical distribution.**OTL.

**OTL**: India.

***Apanteleschloris* Nixon, 1965**

*Apanteleschloris* Nixon, 1965.

**Type information.** Holotype female, USNM (examined). Country of type locality: Philippines.

**Geographical distribution.**OTL.

**OTL**: Philippines, Vietnam.

***Apanteleschristianzunigai* Fernandez-Triana, 2014**

*Apanteleschristianzunigai* Fernandez-Triana, 2014.

**Type information.** Holotype female, CNC (examined). Country of type locality: Costa Rica.

**Geographical distribution.**NEO.

**NEO**: Costa Rica.

***Apantelescingulicornis* Granger, 1949**

*Apantelescingulicornis* Granger, 1949.

**Type information.** Syntypes female, MNHN (not examined but original description checked). Country of type locality: Madagascar.

**Geographical distribution.**AFR.

**AFR**: Madagascar.

***Apantelescinthiabarrantesae* Fernandez-Triana, 2014**

*Apantelescinthiabarrantesae* Fernandez-Triana, 2014.

**Type information.** Holotype female, CNC (examined). Country of type locality: Costa Rica.

**Geographical distribution.**NEO.

**NEO**: Costa Rica.

***Apantelesciriloumanai* Fernandez-Triana, 2014**

*Apantelesciriloumanai* Fernandez-Triana, 2014.

**Type information.** Holotype female, CNC (examined). Country of type locality: Costa Rica.

**Geographical distribution.**NEO.

**NEO**: Costa Rica.

***Apantelesclita* Nixon, 1965**

*Apantelesclita* Nixon, 1965.

**Type information.** Holotype female, NHMUK (examined). Country of type locality: India.

**Geographical distribution.**OTL.

**OTL**: China (FJ), India, Vietnam.

***Apantelescockerelli* Muesebeck, 1921**

*Apantelescockerelli* Muesebeck, 1921.

**Type information.** Holotype female, USNM (not examined but original description checked). Country of type locality: USA.

**Geographical distribution.**NEA.

**NEA**: USA (CA, ID, IA, MI, MO, NE, NM, OR, SD, TX).

***Apantelescocotis* Wilkinson, 1934**

*Apantelescocotis* Wilkinson, 1934.

**Type information.** Holotype female, NHMUK (examined). Country of type locality: Indonesia.

**Geographical distribution.**OTL.

**OTL**: Indonesia, Vietnam.

***Apantelescoedicius* Nixon, 1965**

*Apantelescoedicius* Nixon, 1965.

**Type information.** Holotype female, USNM (examined). Country of type locality: Philippines.

**Geographical distribution.**OTL.

**OTL**: Philippines, Vietnam.

***Apantelescoffeellae* Muesebeck, 1958**

*Apantelescoffeellae* Muesebeck, 1958.

**Type information.** Holotype female, USNM (not examined but original description checked). Country of type locality: Guadeloupe.

**Geographical distribution.**NEO.

**NEO**: Guadeloupe, Puerto Rico.

***Apantelescoilus* Nixon, 1965**

*Apantelescoilus* Nixon, 1965.

**Type information.** Holotype female, NHMUK (examined). Country of type locality: South Africa.

**Geographical distribution.**AFR.

**AFR**: South Africa.

***Apantelesconanchetorum* Viereck, 1917**

*Apantelesconanchetorum* Viereck, 1917.

**Type information.** Holotype female, USNM (examined). Country of type locality: USA.

**Geographical distribution.**NEA.

**NEA**: Canada (NS, ON), USA (AR, CT, DC, IL, IA, KS, MI, MO, NY, OH, PA, SC, SD, WV, WI).

**Notes.** Specimens of *Apantelesconanchetorum* that have rendered DNA barcodes comprise two BINS: BOLD:AAC5506 (eastern North America) and BOLD:AAC5507 (principally western Canada, but some records from ON, PE). Whether they represent two different species or not has been mentioned in the past (Fernandez-Triana et al. 2014b), but for the time being all known specimens are kept as one species.

***Apantelesconcordalis* Cameron, 1911**

*Apantelesconcordalis* Cameron, 1911.

**Type information.** Holotype female, NHMUK (examined). Country of type locality: Guyana.

**Geographical distribution.**NEO.

**NEO**: Guyana, Peru.

**Notes.** Based on the carination and sculpture pattern of propodeum and fore wing venation, this species belongs to the *leucostigmus* group (*sensu*Fernandez-Triana et al. 2014e).

***Apantelesconon* Nixon, 1965**

*Apantelesconon* Nixon, 1965.

**Type information.** Holotype female, NHMUK (examined). Country of type locality: Indonesia.

**Geographical distribution.**OTL, PAL.

**OTL**: China (HB, TW), Indonesia, Philippines; **PAL**: Korea.

**Notes.** It is possible this is actually a *Dolichogenidea* species. The hind wing vannal lobes are not clearly visible (they are folded in both wings in the holotype) but what can be seen suggests they may be setose. Additionally, the anteromesoscutum punctures near the scutoscutellar sulcus do not fuse. However, because we cannot see the vannal lobe clearly, we refrain from transferring the species in this paper.

***Apantelesconspicabilis* de Saeger, 1944**

*Apantelesconspicabilis* de Saeger, 1944.

**Type information.** Holotype female, RMCA (not examined but original description checked). Country of type locality: Democratic Republic of Congo.

**Geographical distribution.**AFR.

**AFR**: Democratic Republic of Congo, Rwanda.

***Apantelescontactus* Papp, 1977**

*Apantelescontactus* Papp, 1977.

**Type information.** Holotype female, HNHM (not examined but original description checked). Country of type locality: Mongolia.

**Geographical distribution.**PAL.

**PAL**: Mongolia, Russia (ZAB).

***Apantelescontaminatus* (Haliday, 1834)**

*Microgastercontaminatus* Haliday, 1834.

**Type information.** Neotype female, NHMUK (examined). Country of type locality: United Kingdom.

**Geographical distribution.**PAL.

**PAL**: Ireland, Italy, Netherlands, United Kingdom.

***Apantelescontemptus* Nixon, 1965**

*Apantelescontemptus* Nixon, 1965.

**Type information.** Holotype female, USNM (examined). Country of type locality: Singapore.

**Geographical distribution.**OTL.

**OTL**: Singapore.

***Apantelescordoi* de Santis, 1980**

*Apantelescordoi* de Santis, 1980.

**Type information.** Holotype female, MLP (not examined but subsequent treatment of the species checked). Country of type locality: Argentina.

**Geographical distribution.**NEO.

**NEO**: Argentina.

**Notes.** Our concept of this species is based on Aquino et al. (2010).

***Apantelescornicula* Chen & Song, 2004**

*Apantelescornicula* Chen & Song, 2004.

**Type information.** Holotype female, FAFU (not examined but original description checked). Country of type locality: China.

**Geographical distribution.**OTL.

**OTL**: China (FJ).

***Apantelescosmopterygivorus* Liu & Chen, 2014**

*Apantelescosmopterygivorus* Liu & Chen, 2014.

**Type information.** Holotype female, ZJUH (not examined but original description checked). Country of type locality: China.

**Geographical distribution.**OTL.

**OTL**: China (ZJ).

***Apantelescoxalis* Szépligeti, 1911**

*Apantelescoxalis* Szépligeti, 1911.

**Type information.** Holotype female, ZMHB (not examined but subsequent treatment of the species checked). Country of type locality: Tanzania.

**Geographical distribution.**AFR.

**AFR**: Democratic Republic of Congo, Malawi, Senegal, Tanzania.

**Notes.** Our species concept is based on the redescription provided by Wilkinson (1932a), who was able to study the holotype.

***Apantelescrassicornis* (Provancher, 1886)**

*Microgastercrassicornis* Provancher, 1886.

*Microgastercrassicornis* Provancher [primary junior homonym of *Microgastercrassicornis* Ruthe, 1860].

**Type information.** Lectotype female, ULQC (examined). Country of type locality: Canada.

**Geographical distribution.**NEA.

**NEA**: Canada (AB, ON, QC, SK), USA (AZ, IL, IN, IA, KS, MD, MA, MI, MN, MO, NJ, NY, OH, PA).

***Apantelescrates* Nixon, 1965**

*Apantelescrates* Nixon, 1965.

**Type information.** Holotype female, USNM (examined). Country of type locality: Philippines.

**Geographical distribution.**OTL.

**OTL**: Philippines, Vietnam.

***Apantelescrispulae* Blanchard, 1943**

*Apantelescrispulae* Blanchard, 1943.

**Type information.** Holotype female, MACN (not examined but original description checked). Country of type locality: Argentina.

**Geographical distribution.**NEO.

**NEO**: Argentina.

***Apantelescristianalemani* Fernandez-Triana, 2014**

*Apantelescristianalemani* Fernandez-Triana, 2014.

**Type information.** Holotype female, CNC (examined). Country of type locality: Costa Rica.

**Geographical distribution.**NEO.

**NEO**: Costa Rica.

***Apantelescrius* Nixon, 1965**

*Apantelescrius* Nixon, 1965.

**Type information.** Holotype female, USNM (examined). Country of type locality: Philippines.

**Geographical distribution.**OTL.

**OTL**: Philippines.

**Notes.** The ovipositor sheaths are short, and the hypopygium has only one median fold (not pleated), similar to that of *Pholetesor*. However, the species otherwise resembles *Apanteles* and thus we have decided to maintain this species in the latter genus.

***Apantelescroceicornis* Muesebeck, 1958**

*Apantelescroceicornis* Muesebeck, 1958.

**Type information.** Holotype female, USNM (not examined but original description checked). Country of type locality: Peru.

**Geographical distribution.**NEO.

**NEO**: Peru.

***Apantelescrocidolomiae* Ahmad, 1945**

*Apantelescrocidolomiae* Ahmad, 1945.

**Type information.** Holotype female, INPC (not examined but original description checked). Country of type locality: India.

**Geographical distribution.**OTL.

**OTL**: India.

***Apantelescrouzeli* Blanchard, 1947**

*Apantelescrouzeli* Blanchard, 1947.

*Apantelescrouzelae* de Santis, 1967 [unjustified emendation].

**Type information.** Holotype female, MACN (not examined but original description checked). Country of type locality: Argentina.

**Geographical distribution.**NEO.

**NEO**: Argentina.

***Apantelescuneiformis* Song & Chen, 2004**

*Apantelescuneiformis* Song & Chen, 2004.

**Type information.** Holotype female, FAFU (not examined but original description checked). Country of type locality: China.

**Geographical distribution.**OTL.

**OTL**: China (FJ, YN).

***Apantelescurvicaudatus* Granger, 1949**

*Apantelescurvicaudatus* Granger, 1949.

**Type information.** Syntypes female and male, MNHN (not examined but original description checked). Country of type locality: Madagascar.

**Geographical distribution.**AFR.

**AFR**: Madagascar.

***Apantelescynthiacorderoae* Fernandez-Triana, 2014**

*Apantelescynthiacorderoae* Fernandez-Triana, 2014.

**Type information.** Holotype female, CNC (examined). Country of type locality: Costa Rica.

**Geographical distribution.**NEO.

**NEO**: Costa Rica.

***Apantelescyprioides* Nixon, 1965**

*Apantelescyprioides* Nixon, 1965.

**Type information.** Holotype female, USNM (examined). Country of type locality: Philippines.

**Geographical distribution.**AFR, OTL.

**AFR**: South Africa; **OTL**: China (FJ, HN), Philippines, Singapore.

***Apantelescypris* Nixon, 1965**

*Apantelescypris* Nixon, 1965.

**Type information.** Holotype female, USNM (examined). Country of type locality: Philippines.

**Geographical distribution.**OTL, PAL.

**OTL**: Bangladesh, China (FJ, GD, GX, GZ, HI, HK, HB, HN, JS, JX, SH, SN, TW, YN, ZJ), India, Indonesia, Malaysia, Nepal, Pakistan, Philippines, Singapore, Sri Lanka, Vietnam; **PAL**: China (AH, HA, SN, SD), Japan.

***Apantelesdaimenes* Nixon, 1965**

*Apantelesdaimenes* Nixon, 1965.

**Type information.** Holotype female, NHMUK (examined). Country of type locality: Fiji.

**Geographical distribution.**AUS.

**AUS**: Fiji.

***Apantelesdakotae* Muesebeck, 1921**

*Apantelesdakotae* Muesebeck, 1921.

**Type information.** Holotype female, USNM (not examined but original description checked). Country of type locality: USA.

**Geographical distribution.**NEA.

**NEA**: USA (ID, SD).

***Apantelesdecoloratus* Granger, 1949**

*Apantelesdecoloratus* Granger, 1949.

**Type information.** Syntypes female, MNHN (not examined but original description checked). Country of type locality: Madagascar.

**Geographical distribution.**AFR.

**AFR**: Madagascar.

***Apantelesdeifiliadavilae* Fernandez-Triana, 2014**

*Apantelesdeifiliadavilae* Fernandez-Triana, 2014.

**Type information.** Holotype female, CNC (examined). Country of type locality: Costa Rica.

**Geographical distribution.**NEO.

**NEO**: Costa Rica.

***Apantelesdelhiensis* Muesebeck & Subba Rao, 1958**

*Apantelesdelhiensis* Muesebeck & Subba Rao, 1958.

**Type information.** Holotype female, USNM (not examined but original description checked). Country of type locality: India.

**Geographical distribution.**OTL.

**OTL**: India.

***Apantelesdentatus* Muesebeck, 1958**

*Apantelesdentatus* Muesebeck, 1958.

**Type information.** Holotype female, USNM (not examined but original description checked). Country of type locality: Brazil.

**Geographical distribution.**NEO.

**NEO**: Brazil (SP).

***Apantelesdeplanatus* Muesebeck, 1957**

*Apantelesdeplanatus* Muesebeck, 1957.

**Type information.** Holotype female, USNM (not examined but subsequent treatment of the species checked). Country of type locality: Mexico.

**Geographical distribution.**NEO.

**NEO**: Mexico.

**Notes.** Our species concept is based on Austin and Dangerfield (1989) and Fernandez-Triana et al. (2014e).

***Apantelesdepressariae* Muesebeck, 1931**

*Apantelesdepressariae* Muesebeck, 1931.

**Type information.** Holotype female, USNM (not examined but original description checked). Country of type locality: USA.

**Geographical distribution.**NEA.

**NEA**: Canada (NS, ON, QC), USA (IA, ME, MA, VT).

***Apantelesderivatus* Long, 2010**

*Apantelesderivatus* Long, 2010.

**Type information.** Holotype female, IEBR (not examined but original description checked). Country of type locality: Vietnam.

**Geographical distribution.**OTL.

**OTL**: Vietnam.

***Apantelesdesantisi* Blanchard, 1947**

*Apantelesdesantisi* Blanchard, 1947.

**Type information.** Holotype female, MACN (not examined but original description checked). Country of type locality: Argentina.

**Geographical distribution.**NEO.

**NEO**: Argentina.

***Apantelesdespectus* Nixon, 1965**

*Apantelesdespectus* Nixon, 1965.

**Type information.** Holotype female, NHMUK (examined). Country of type locality: Thailand.

**Geographical distribution.**OTL.

**OTL**: Thailand.

***Apantelesdiatraeae* Muesebeck, 1921**

*Apantelesdiatraeae* Muesebeck, 1921.

**Type information.** Holotype female, USNM (not examined but subsequent treatment of the species checked). Country of type locality: Cuba.

**Geographical distribution.**NEA, NEO.

**NEA**: USA (AZ, FL); **NEO**: Colombia, Cuba, Dominican Republic, Grenada, Guatemala, Haiti, Honduras, Jamaica, Mexico, Nicaragua, Panama, Puerto Rico, Trinidad & Tobago, Venezuela.

**Notes.** Our species concept is based on Austin and Dangerfield (1989) and Fernandez-Triana et al. (2014e). Yu et al. (2016) recorded France as a country for the species, based on one reference (Paddock 1933). However, we have read that paper, and there is no mention of *A.diatraea* there. Because we have not found any other source citing or supporting the presence of this species for France, we consider that record to be incorrect. Other country records (for introductions of *diatraea*) can be found in Bartlett et al. (1978).

***Apantelesdickyui* Fernandez-Triana, 2014**

*Apantelesdickyui* Fernandez-Triana, 2014.

**Type information.** Holotype female, CNC (examined). Country of type locality: Costa Rica.

**Geographical distribution.**NEO.

**NEO**: Costa Rica.

***Apantelesdictys* Nixon, 1965**

*Apantelesdictys* Nixon, 1965.

**Type information.** Holotype female, NHMUK (examined). Country of type locality: Indonesia.

**Geographical distribution.**OTL.

**OTL**: Indonesia.

***Apantelesdidiguadamuzi* Fernandez-Triana, 2014**

*Apantelesdidiguadamuzi* Fernandez-Triana, 2014.

**Type information.** Holotype female, CNC (examined). Country of type locality: Costa Rica.

**Geographical distribution.**NEO.

**NEO**: Costa Rica.

***Apantelesdido* Nixon, 1965**

*Apantelesdido* Nixon, 1965.

**Type information.** Holotype female, NHMUK (examined). Country of type locality: South Africa.

**Geographical distribution.**AFR.

**AFR**: South Africa.

***Apantelesdiegoalpizari* Fernandez-Triana, 2014**

*Apantelesdiegoalpizari* Fernandez-Triana, 2014.

**Type information.** Holotype female, CNC (examined). Country of type locality: Costa Rica.

**Geographical distribution.**NEO.

**NEO**: Costa Rica.

***Apantelesdiegotorresi* Fernandez-Triana, 2014**

*Apantelesdiegotorresi* Fernandez-Triana, 2014.

**Type information.** Holotype female, CNC (examined). Country of type locality: Costa Rica.

**Geographical distribution.**NEO.

**NEO**: Costa Rica.

***Apantelesdiniamartinezae* Fernandez-Triana, 2014**

*Apantelesdiniamartinezae* Fernandez-Triana, 2014.

**Type information.** Holotype female, CNC (examined). Country of type locality: Costa Rica.

**Geographical distribution.**NEO.

**NEO**: Costa Rica.

***Apantelesdiocles* Nixon, 1965**

*Apantelesdiocles* Nixon, 1965.

**Type information.** Holotype female, NHMUK (examined). Country of type locality: India.

**Geographical distribution.**OTL.

**OTL**: China (HN), India, Indonesia, Philippines, Vietnam.

***Apantelesdiourbeli* Risbec, 1951**

*Apantelesdiourbeli* Risbec, 1951.

**Type information.** Holotype male, depository unknown (not examined but original description checked). Country of type locality: Senegal.

**Geographical distribution.**AFR.

**AFR**: Senegal.

***Apantelesdissimilis* Nixon, 1965**

*Apantelesdissimile* Nixon, 1965.

**Type information.** Holotype female, USNM (examined). Country of type locality: Philippines.

**Geographical distribution.**OTL, PAL.

**OTL**: China (FJ, HB), Philippines, Vietnam; **PAL**: China (JL).

**Notes.** Both versions of Taxapad (Yu et al. 2012, 2016) correctly spell the name of this species as *Apantelesdissimilis*. However, the original description of the species (Nixon 1965) and most of the subsequent, published references (e.g., Shenefelt 1972, Long et al. 2004, Chen and Song 2004) incorrectly refer to the species as *Apantelesdissimile*, which is the neuter rather than the masculine form of the adjective, and therefore violates ICZN Article 31.2. The Code-compliant spelling must be *dissimilis*, regardless of the original spelling (Doug Yanega, pers. comm.), and it is the one we follow here. The specimen is in poor condition, with detached metasoma and one pair of wings glued to a second card (underneath the point with the specimen). Because the wings were detached, the vannal lobe was torn and its shape and setation patterns can no longer be determined. But the punctures on the posterior margin of the anteromesoscutum are fused, thus we consider this species to belong to *Apanteles*.

***Apantelesdores* Nixon, 1965**

*Apantelesdores* Nixon, 1965.

**Type information.** Holotype female, USNM (examined). Country of type locality: Malaysia.

**Geographical distribution.**OTL.

**OTL**: Malaysia, Vietnam.

***Apantelesdotus* Nixon, 1965**

*Apantelesdotus* Nixon, 1965.

**Type information.** Holotype female, NHMUK (examined). Country of type locality: Sri Lanka.

**Geographical distribution.**OTL.

**OTL**: Philippines, Sri Lanka, Vietnam.

***Apantelesdrupes* Nixon, 1965**

*Apantelesdrupes* Nixon, 1965.

**Type information.** Holotype female, NHMUK (examined). Country of type locality: South Africa.

**Geographical distribution.**AFR.

**AFR**: South Africa.

***Apantelesdumosus* Liu & Chen, 2014**

*Apantelesdumosus* Liu & Chen, 2014.

**Type information.** Holotype female, ZJUH (not examined but original description checked). Country of type locality: China.

**Geographical distribution.**PAL.

**PAL**: China (JL, LN).

***Apantelesduniagarciae* Fernandez-Triana, 2014**

*Apantelesduniagarciae* Fernandez-Triana, 2014.

**Type information.** Holotype female, CNC (examined). Country of type locality: Costa Rica.

**Geographical distribution.**NEO.

**NEO**: Costa Rica.

***Apantelesduplicatus* Brèthes, 1922**

*Apantelesduplicatus* Brèthes, 1922.

**Type information.** Holotype female, MACN (not examined). Country of type locality: Argentina.

**Geographical distribution.**NEO.

**NEO**: Argentina.

***Apantelesduvalierbricenoi* Fernandez-Triana, 2014**

*Apantelesduvalierbricenoi* Fernandez-Triana, 2014.

**Type information.** Holotype female, CNC (examined). Country of type locality: Costa Rica.

**Geographical distribution.**NEO.

**NEO**: Costa Rica.

***Apantelesedgarjimenezi* Fernandez-Triana, 2014**

*Apantelesedgarjimenezi* Fernandez-Triana, 2014.

**Type information.** Holotype female, CNC (examined). Country of type locality: Costa Rica.

**Geographical distribution.**NEO.

**NEO**: Costa Rica.

***Apantelesedithlopezae* Fernandez-Triana, 2014**

*Apantelesedithlopezae* Fernandez-Triana, 2014.

**Type information.** Holotype female, CNC (examined). Country of type locality: Costa Rica.

**Geographical distribution.**NEO.

**NEO**: Costa Rica.

***Apanteleseduardoramirezi* Fernandez-Triana, 2014**

*Apanteleseduardoramirezi* Fernandez-Triana, 2014.

**Type information.** Holotype female, CNC (examined). Country of type locality: Costa Rica.

**Geographical distribution.**NEO.

**NEO**: Costa Rica.

***Apantelesedwardsii* Riley, 1889**

*Apantelesedwardsii* Riley, 1889.

**Type information.** Holotype female, USNM (examined). Country of type locality: USA.

**Geographical distribution.**NEA.

**NEA**: Canada (ON, QC), USA (CT, IL, IA, ME, MA, MI, MN, NY, OH).

***Apantelesedwinapuii* Fernandez-Triana, 2014**

*Apantelesedwinapuii* Fernandez-Triana, 2014.

**Type information.** Holotype female, CNC (examined). Country of type locality: Costa Rica.

**Geographical distribution.**NEO.

**NEO**: Costa Rica.

***Apanteleselagabalus* Nixon, 1965**

*Apanteleselagabalus* Nixon, 1965.

**Type information.** Holotype female, AEIC (not examined but original description checked). Country of type locality: Philippines.

**Geographical distribution.**OTL.

**OTL**: Philippines.

***Apanteleseldarayae* Fernandez-Triana, 2014**

*Apanteleseldarayae* Fernandez-Triana, 2014.

**Type information.** Holotype female, CNC (examined). Country of type locality: Costa Rica.

**Geographical distribution.**NEO.

**NEO**: Costa Rica.

***Apanteleseliethcantillanoae* Fernandez-Triana, 2014**

*Apanteleseliethcantillanoae* Fernandez-Triana, 2014.

**Type information.** Holotype female, CNC (examined). Country of type locality: Costa Rica.

**Geographical distribution.**NEO.

**NEO**: Costa Rica.

***Apantelesepiblemae* Muesebeck, 1935**

*Apantelesepiblemae* Muesebeck, 1935.

**Type information.** Holotype female, USNM (examined). Country of type locality: USA.

**Geographical distribution.**NEA.

**NEA**: USA (CA, DE, FL, GA, KS).

***Apantelesepijarbi* Rao, 1953**

*Apantelesepijarbi* Rao, 1953.

**Type information.** Holotype female, SJCA (not examined but original description checked). Country of type locality: India.

**Geographical distribution.**OTL.

**OTL**: India.

***Apantelesepinotiae* Viereck, 1912**

*Apantelesepinotiae* Viereck, 1912.

**Type information.** Holotype male, USNM (examined). Country of type locality: USA.

**Geographical distribution.**NEA.

**NEA**: Canada (ON), USA (CT, FL, IL, KS, KY, ME, MD, MA, MO, NE, NJ, NY, OH, OK, PA, SC, TX, VA, WV).

***Apanteleserickduartei* Fernandez-Triana, 2014**

*Apanteleserickduartei* Fernandez-Triana, 2014.

**Type information.** Holotype female, CNC (examined). Country of type locality: Costa Rica.

**Geographical distribution.**NEO.

**NEO**: Costa Rica.

***Apanteleseriphyle* Nixon, 1965**

*Apanteleseriphyle* Nixon, 1965.

**Type information.** Holotype female, USNM (examined). Country of type locality: Philippines.

**Geographical distribution.**OTL.

**OTL**: Philippines.

***Apanteleserse* Nixon, 1965**

*Apanteleserse* Nixon, 1965.

**Type information.** Holotype female, NHMUK (examined). Country of type locality: Indonesia.

**Geographical distribution.**OTL.

**OTL**: Indonesia, Malaysia.

***Apantelesesthercentenoae* Fernandez-Triana, 2014**

*Apantelesesthercentenoae* Fernandez-Triana, 2014.

**Type information.** Holotype female, CNC (examined). Country of type locality: Costa Rica.

**Geographical distribution.**NEO.

**NEO**: Costa Rica.

***Apanteleseublemmae* Nixon, 1965**

*Apanteleseublemmae* Nixon, 1965.

**Type information.** Holotype female, NHMUK (examined). Country of type locality: Tanzania.

**Geographical distribution.**AFR.

**AFR**: Kenya, South Africa, Tanzania.

***Apanteleseugeniaphilipsae* Fernandez-Triana, 2014**

*Apanteleseugeniaphilipsae* Fernandez-Triana, 2014.

**Type information.** Holotype female, CNC (examined). Country of type locality: Costa Rica.

**Geographical distribution.**NEO.

**NEO**: Costa Rica.

***Apanteleseulogiosequeirai* Fernandez-Triana, 2014**

*Apanteleseulogiosequeirai* Fernandez-Triana, 2014.

**Type information.** Holotype female, CNC (examined). Country of type locality: Costa Rica.

**Geographical distribution.**NEO.

**NEO**: Costa Rica.

***Apanteleseupolis* Nixon, 1965**

*Apanteleseupolis* Nixon, 1965.

**Type information.** Holotype female, NHMUK (examined). Country of type locality: South Africa.

**Geographical distribution.**AFR.

**AFR**: South Africa.

***Apanteleseurynome* Nixon, 1965**

*Apanteleseurynome* Nixon, 1965.

**Type information.** Holotype female, NHMUK (examined). Country of type locality: Fiji.

**Geographical distribution.**AUS.

**AUS**: Fiji.

***Apanteleseurytergis* de Saeger, 1941**

*Apanteleseurytergis* de Saeger, 1941.

**Type information.** Holotype female, RMCA (not examined but original description checked). Country of type locality: Democratic Republic of Congo.

**Geographical distribution.**AFR.

**AFR**: Cape Verde, Democratic Republic of Congo.

***Apantelesevadnix* Shenefelt, 1972**

*Apantelesevadnix* Shenefelt, 1972.

*Apantelesevadne* Nixon, 1965 [primary junior homonym of *Apantelesevadne* Nixon, 1955].

**Type information.** Holotype female, NHMUK (examined). Country of type locality: Uganda.

**Geographical distribution.**AFR.

**AFR**: Uganda.

***Apantelesevanidus* Papp, 1975**

*Apantelesevanidus* Papp, 1975.

*Apantelescalpurnia* Nixon, 1976.

**Type information.** Holotype female, HNHM (not examined but original description checked). Country of type locality: Hungary.

**Geographical distribution.**PAL.

**PAL**: Finland, Hungary, Moldova, Russia (S), Sweden, Turkey, Ukraine.

***Apantelesevansi* Nixon, 1971**

*Apantelesevansi* Nixon, 1971.

**Type information.** Holotype female, NHMUK (examined). Country of type locality: Kenya.

**Geographical distribution.**AFR.

**AFR**: Cape Verde, Kenya.

***Apantelesfaustina* Nixon, 1965**

*Apantelesfaustina* Nixon, 1965.

**Type information.** Holotype female, NHMUK (examined). Country of type locality: Mauritius.

**Geographical distribution.**AFR.

**AFR**: Mauritius.

***Apantelesfedericomatarritai* Fernandez-Triana, 2014**

*Apantelesfedericomatarritai* Fernandez-Triana, 2014.

**Type information.** Holotype female, CNC (examined). Country of type locality: Costa Rica.

**Geographical distribution.**NEO.

**NEO**: Costa Rica.

***Apantelesfelipechavarriai* Fernandez-Triana, 2014**

*Apantelesfelipechavarriai* Fernandez-Triana, 2014.

**Type information.** Holotype female, CNC (examined). Country of type locality: Costa Rica.

**Geographical distribution.**NEO.

**NEO**: Costa Rica.

***Apantelesfelixcarmonai* Fernandez-Triana, 2014**

*Apantelesfelixcarmonai* Fernandez-Triana, 2014.

**Type information.** Holotype female, CNC (examined). Country of type locality: Costa Rica.

**Geographical distribution.**NEO.

**NEO**: Costa Rica.

***Apantelesfeltiae* Viereck, 1912**

*Apantelesfeltiae* Viereck, 1912.

**Type information.** Holotype female, USNM (examined). Country of type locality: USA.

**Geographical distribution.**NEA.

**NEA**: Canada (SK), USA (AZ, CA, ID, IN, IA, MI, OH, SD, UT).

***Apantelesfernandochavarriai* Fernandez-Triana, 2014**

*Apantelesfernandochavarriai* Fernandez-Triana, 2014.

**Type information.** Holotype female, CNC (examined). Country of type locality: Costa Rica.

**Geographical distribution.**NEO.

**NEO**: Costa Rica.

***Apantelesfirmus* Telenga, 1949**

*Apantelesfirmus* Telenga, 1949.

*Apantelesfirmusrufipes* Telenga, 1955.

**Type information.** Holotype and depository unknown not examined but authoritatively identified specimens examined). Country of type locality: Tajikistan.

**Geographical distribution.**PAL.

**PAL**: Armenia, Azerbaijan, France, Hungary, Kazakhstan, Korea, Mongolia, Romania, Russia (YAR), Tajikistan, Ukraine, Yugoslavia.

**Notes.** We have examined a female paratype, donated to the CNC. That specimen looks like *Dolichogenidea* (the vannal lobes in both hind wings are broken but they appear to be setose, although it is not entirely clear). The descriptions and comments by Nixon (1973) and Papp (1984a) also suggest this species could be placed in *Dolichogenidea*. However, without examining more specimens we refrain to transfer the species in this paper and prefer to maintain it in *Apanteles* for the time being.

***Apantelesflavicapus* Liu & Chen, 2014**

*Apantelesflavicapus* Liu & Chen, 2014.

**Type information.** Holotype female, ZJUH (not examined but original description checked). Country of type locality: China.

**Geographical distribution.**OTL.

**OTL**: China (GD).

***Apantelesflavicentrus* Long, 2010**

*Apantelesflavicentrus* Long, 2010.

**Type information.** Holotype female, IEBR (not examined but original description checked). Country of type locality: Vietnam.

**Geographical distribution.**OTL.

**OTL**: Vietnam.

**Notes.** This species might not be *Apanteles*, but the original description does not provide enough details to determine its placement, so we retain it within *Apanteles*.

***Apantelesflavigaster* Long, 2010**

*Apantelesflavigaster* Long, 2010.

**Type information.** Holotype female, IEBR (not examined but original description checked). Country of type locality: Vietnam.

**Geographical distribution.**OTL.

**OTL**: Vietnam.

**Notes.** This species might not be *Apanteles*, but the original description does not provide enough details to determine its placement, so we retain it within *Apanteles*.

***Apantelesfloralis* Tobias, 1966**

*Apantelesfloralis* Tobias, 1966.

**Type information.** Holotype female, ZIN (not examined but subsequent treatment of the species checked). Country of type locality: Turkmenistan.

**Geographical distribution.**PAL.

**PAL**: Kazakhstan, Mongolia, Turkmenistan.

**Notes.** Our species concept is based on Papp (1984a) and Tobias (1986).

***Apantelesflormoralesae* Fernandez-Triana, 2014**

*Apantelesflormoralesae* Fernandez-Triana, 2014.

**Type information.** Holotype female, CNC (examined). Country of type locality: Costa Rica.

**Geographical distribution.**NEO.

**NEO**: Costa Rica.

***Apantelesflorus* Nixon, 1965**

*Apantelesflorus* Nixon, 1965.

**Type information.** Holotype female, NHMUK (examined). Country of type locality: China.

**Geographical distribution.**OTL.

**OTL**: China (GD, HN).

***Apantelesfluitantis* de Santis, 1980**

*Apantelesfluitantis* de Santis, 1980.

**Type information.** Holotype female, MLP (not examined but subsequent treatment of the species checked). Country of type locality: Argentina.

**Geographical distribution.**NEO.

**NEO**: Argentina.

**Notes.** Our concept of this species is based on Aquino et al. (2010).

***Apantelesfontinalis* de Saeger, 1944**

*Apantelesfontinalis* de Saeger, 1944.

**Type information.** Holotype female, RMCA (not examined but original description checked). Country of type locality: Rwanda.

**Geographical distribution.**AFR.

**AFR**: Democratic Republic of Congo, Réunion, Rwanda.

***Apantelesforbesi* Viereck, 1910**

*Apantelesforbesi* Viereck, 1910.

**Type information.** Holotype female, USNM (examined). Country of type locality: USA.

**Geographical distribution.**NEA.

**NEA**: Canada (MB, NS, ON), USA (AZ, CT, FL, IL, IN, IA, KS, KY, MD, MA, MO, NY, OR, SD).

***Apantelesfranciscopizarroi* Fernandez-Triana, 2014**

*Apantelesfranciscopizarroi* Fernandez-Triana, 2014.

**Type information.** Holotype female, CNC (examined). Country of type locality: Costa Rica.

**Geographical distribution.**NEO.

**NEO**: Costa Rica.

***Apantelesfranciscoramirezi* Fernandez-Triana, 2014**

*Apantelesfranciscoramirezi* Fernandez-Triana, 2014.

**Type information.** Holotype female, CNC (examined). Country of type locality: Costa Rica.

**Geographical distribution.**NEO.

**NEO**: Costa Rica.

***Apantelesfreddyquesadai* Fernandez-Triana, 2014**

*Apantelesfreddyquesadai* Fernandez-Triana, 2014.

**Type information.** Holotype female, CNC (examined). Country of type locality: Costa Rica.

**Geographical distribution.**NEO.

**NEO**: Costa Rica.

***Apantelesfreddysalazari* Fernandez-Triana, 2014**

*Apantelesfreddysalazari* Fernandez-Triana, 2014.

**Type information.** Holotype female, CNC (examined). Country of type locality: Costa Rica.

**Geographical distribution.**NEO.

**NEO**: Costa Rica.

***Apantelesfredi* Austin & Dangerfield, 1989**

*Apantelesfredi* Austin & Dangerfield, 1989.

**Type information.** Holotype female, NHMUK (not examined but original description checked). Country of type locality: Guatemala.

**Geographical distribution.**NEO.

**NEO**: Guatemala.

***Apantelesfrersi* (Brèthes, 1917)**

*Coelothoraxfrersi* Brèthes, 1917.

**Type information.** Holotype female, MACN (not examined). Country of type locality: Argentina.

**Geographical distribution.**NEO.

**NEO**: Argentina.

***Apantelesfumiferanae* Viereck, 1912**

*Apantelesfumiferanae* Viereck, 1912.

**Type information.** Holotype female, USNM (examined). Country of type locality: USA.

**Geographical distribution.**NEA, PAL.

**NEA**: Canada (BC, MB, NB, NL, NT, ON, QC), USA (AK, CO, ID, ME, MA, MI, MN, MT, NM, NY, OR, SC, SD, WA, WI); **PAL**: Poland.

**Notes.** We consider *A.fumiferanae* as a Nearctic species (Fernandez-Triana 2010, Fernandez-Triana and Huber 2010). The single record from the Palearctic is based on one publication compiling the Hymenoptera from Poland (Huflejt 1997), and it is very likely to be incorrect; however, we refrain to remove that record until more studies are done. The generic placement of this species is also somewhat controversial as the female holotype has the hind wings with a straight vannal lobe with small setae which are just slightly sparser than proximal and distal margins of lobe, and the (shallow and sparse) punctures on the anteromesoscutum are not fused near posterior margin. These two features are borderline with *Dolichogenidea* and more studies, combining morphology, biology, and molecular data, will be needed.

***Apantelesfundulus* Nixon, 1965**

*Apantelesfundulus* Nixon, 1965.

**Type information.** Holotype female, NHMUK (examined). Country of type locality: Australia.

**Geographical distribution.**AUS, OTL.

**AUS**: Australia (QLD); **OTL**: Vietnam.

***Apantelesgabrielagutierrezae* Fernandez-Triana, 2014**

*Apantelesgabrielagutierrezae* Fernandez-Triana, 2014.

**Type information.** Holotype female, CNC (examined). Country of type locality: Costa Rica.

**Geographical distribution.**NEO.

**NEO**: Costa Rica.

***Apantelesgalatea* Nixon, 1965**

*Apantelesgalatea* Nixon, 1965.

**Type information.** Holotype female, USNM (examined). Country of type locality: Philippines.

**Geographical distribution.**OTL.

**OTL**: Philippines.

***Apantelesgalleriae* Wilkinson, 1932**

*Apantelesgalleriae* Wilkinson, 1932.

**Type information.** Holotype female, NHMUK (examined). Country of type locality: France.

**Geographical distribution.**AFR, NEA, NEO, OTL, PAL.

**AUS**: Hawaiian Islands, New Zealand; **AFR**: Mauritius, Réunion; **NEA**: Canada (BC), USA (GA, NC, OH, SC); **NEO**: Argentina, Brazil (SP); **OTL**: China (GZ, HN, TW, ZJ), India, Pakistan; **PAL**: Armenia, Bulgaria, France, Greece, Hungary, Iran, Italy, Japan, Malta, Romania, Russia (PRI), Spain, Turkey, United Kingdom.

**Notes.** Distribution in Brazil based on de Santis (1964) and Shimbori (pers. comm.).

***Apantelesgandoensis* de Saeger, 1944**

*Apantelesgandoensis* de Saeger, 1944.

**Type information.** Holotype female, RMCA (not examined but original description checked). Country of type locality: Rwanda.

**Geographical distribution.**AFR.

**AFR**: Rwanda.

***Apantelesgarygibsoni* Fernandez-Triana, 2014**

*Apantelesgarygibsoni* Fernandez-Triana, 2014.

**Type information.** Holotype female, CNC (examined). Country of type locality: Costa Rica.

**Geographical distribution.**NEO.

**NEO**: Costa Rica.

***Apantelesgaytotini* Blanchard, 1959**

*Apantelesgaytotini* Blanchard, 1959.

**Type information.** Holotype female, MACN (not examined). Country of type locality: Argentina.

**Geographical distribution.**NEO.

**NEO**: Argentina.

***Apantelesgerardobandoi* Fernandez-Triana, 2014**

*Apantelesgerardobandoi* Fernandez-Triana, 2014.

**Type information.** Holotype female, CNC (examined). Country of type locality: Costa Rica.

**Geographical distribution.**NEO.

**NEO**: Costa Rica.

***Apantelesgerardosandovali* Fernandez-Triana, 2014**

*Apantelesgerardosandovali* Fernandez-Triana, 2014.

**Type information.** Holotype female, CNC (examined). Country of type locality: Costa Rica.

**Geographical distribution.**NEO.

**NEO**: Costa Rica.

***Apantelesghesquierei* de Saeger, 1941**

*Apantelesghesquierei* de Saeger, 1941.

**Type information.** Holotype female, RMCA (not examined but original description checked). Country of type locality: Democratic Republic of Congo.

**Geographical distribution.**AFR.

**AFR**: Democratic Republic of Congo, Senegal.

***Apantelesgialamensis* Long, 2007**

*Apantelesgialamensis* Long, 2007.

**Type information.** Holotype female, IEBR (not examined but original description checked). Country of type locality: Vietnam.

**Geographical distribution.**OTL.

**OTL**: Vietnam.

***Apantelesgitebe* de Saeger, 1944**

*Apantelesgitebe* de Saeger, 1944.

**Type information.** Holotype female, RMCA (not examined but original description checked). Country of type locality: Democratic Republic of Congo.

**Geographical distribution.**AFR.

**AFR**: Democratic Republic of Congo.

***Apantelesgladysrojasae* Fernandez-Triana, 2014**

*Apantelesgladysrojasae* Fernandez-Triana, 2014.

**Type information.** Holotype female, CNC (examined). Country of type locality: Costa Rica.

**Geographical distribution.**NEO.

**NEO**: Costa Rica.

***Apantelesglenriverai* Fernandez-Triana, 2014**

*Apantelesglenriverai* Fernandez-Triana, 2014.

**Type information.** Holotype female, CNC (examined). Country of type locality: Costa Rica.

**Geographical distribution.**NEO.

**NEO**: Costa Rica.

***Apantelesgloriasihezarae* Fernandez-Triana, 2014**

*Apantelesgloriasihezarae* Fernandez-Triana, 2014.

**Type information.** Holotype female, CNC (examined). Country of type locality: Costa Rica.

**Geographical distribution.**NEO.

**NEO**: Costa Rica.

***Apantelesgoron* Nixon, 1965**

*Apantelesgoron* Nixon, 1965.

**Type information.** Holotype female, NHMUK (examined). Country of type locality: Malaysia.

**Geographical distribution.**OTL.

**OTL**: Malaysia.

***Apantelesgracilicorne* Song & Chen, 2004**

*Apantelesgracilicorne* Song & Chen, 2004.

**Type information.** Holotype female, FAFU (not examined but original description checked). Country of type locality: China.

**Geographical distribution.**OTL.

**OTL**: China (FJ).

***Apantelesgracilipes* Song & Chen, 2004**

*Apantelesgracilipes* Song & Chen, 2004.

**Type information.** Holotype female, FAFU (not examined but original description checked). Country of type locality: China.

**Geographical distribution.**OTL.

**OTL**: China (FJ, GD, HI, HB, YN).

***Apantelesguadaluperodriguezae* Fernandez-Triana, 2014**

*Apantelesguadaluperodriguezae* Fernandez-Triana, 2014.

**Type information.** Holotype female, CNC (examined). Country of type locality: Costa Rica.

**Geographical distribution.**NEO.

**NEO**: Costa Rica.

***Apantelesguamensis* (Holmgren, 1868)**

*Microgasterguamensis* Holmgren, 1868.

**Type information.** Type and depository unknown (not examined but authoritatively identified specimens examined). Country of type locality: Guam.

**Geographical distribution.**AUS.

**AUS**: Guam.

**Notes.** The last two versions of Taxapad (Yu et al. 2012, 2016) have this species listed as *Microgaster*. However, we examined a female homotype in the CNC, previously studied by William Mason, and the species clearly belongs in *Apanteles*, which agrees with Shenefelt (1972) who had also transferred the species to that genus. For clarity we revise the combination of this species here. The type(s) details and depository are presently unknown but Shenefelt (1972: 527) recorded the female sex as part of the original description, although without elaborating.

***Apantelesguillermopereirai* Fernandez-Triana, 2014**

*Apantelesguillermopereirai* Fernandez-Triana, 2014.

**Type information.** Holotype female, CNC (examined). Country of type locality: Costa Rica.

**Geographical distribution.**NEO.

**NEO**: Costa Rica.

***Apanteleshainanensis* Liu & Chen, 2015**

*Apanteleshainanensis* Liu & Chen, 2015.

**Type information.** Holotype female, ZJUH (not examined but original description checked). Country of type locality: China.

**Geographical distribution.**OTL.

**OTL**: China (HI).

***Apanteleshalfordi* Ullyett, 1946**

*Apanteleshalfordi* Ullyett, 1946.

*Apanteleseriophyes* Nixon, 1965.

**Type information.** Holotype female, TMSA (not examined but original description checked). Country of type locality: South Africa.

**Geographical distribution.**AFR.

**AFR**: South Africa.

**Notes.** We examined the type of *Apanteleseriophyes* Nixon, 1965.

***Apanteleshapaliae* de Saeger, 1941**

*Apanteleshapaliae* de Saeger, 1941.

**Type information.** Holotype male, RMCA (not examined but original description checked). Country of type locality: Democratic Republic of Congo.

**Geographical distribution.**AFR.

**AFR**: Democratic Republic of Congo.

***Apantelesharryramirezi* Fernandez-Triana, 2014**

*Apantelesharryramirezi* Fernandez-Triana, 2014.

**Type information.** Holotype female, CNC (examined). Country of type locality: Costa Rica.

**Geographical distribution.**NEO.

**NEO**: Costa Rica.

***Apantelesharti* Viereck, 1910**

*Apantelesharti* Viereck, 1910.

**Type information.** Holotype female, USNM (examined). Country of type locality: USA.

**Geographical distribution.**NEA.

**NEA**: Canada (ON), USA (CT, DC, IL, IA, KS, MD, MI, MO, NJ, OH, TN).

***Apanteleshatinhensis* Long, 2010**

*Apanteleshatinhensis* Long, 2010.

**Type information.** Holotype female, IEBR (not examined but original description checked). Country of type locality: Vietnam.

**Geographical distribution.**OTL.

**OTL**: Vietnam.

***Apanteleshaywardi* Blanchard, 1947**

*Apanteleshaywardi* Blanchard, 1947.

**Type information.** Holotype female, MACN (not examined but original description checked). Country of type locality: Argentina.

**Geographical distribution.**NEO.

**NEO**: Argentina, Brazil (SP).

***Apanteleshazelcambroneroae* Fernandez-Triana, 2014**

*Apanteleshazelcambroneroae* Fernandez-Triana, 2014.

**Type information.** Holotype female, CNC (examined). Country of type locality: Costa Rica.

**Geographical distribution.**NEO.

**NEO**: Costa Rica.

***Apanteleshebrus* Nixon, 1965**

*Apanteleshebrus* Nixon, 1965.

**Type information.** Holotype female, NHMUK (examined). Country of type locality: South Africa.

**Geographical distribution.**AFR.

**AFR**: South Africa.

***Apanteleshectorsolisi* Fernandez-Triana, 2014**

*Apanteleshectorsolisi* Fernandez-Triana, 2014.

**Type information.** Holotype female, CNC (examined). Country of type locality: Costa Rica.

**Geographical distribution.**NEO.

**NEO**: Costa Rica.

***Apanteleshedwigi* Shenefelt, 1972**

*Apanteleshedwigi* Shenefelt, 1972.

*Apantelesareolaris* Hedwig, 1961 [primary junior homonym of *Apantelesareolaris* Blanchard, 1947].

**Type information.** Holotype female, LNKD (not examined but original description checked). Country of type locality: Afghanistan.

**Geographical distribution.**PAL.

**PAL**: Afghanistan.

**Notes.** The original description alone is not sufficient to unambiguously establish the generic placement for this species (it could be *Apanteles*, *Dolichogenidea*, *Pholetesor*, or perhaps even another genus). Until study of the only known specimen is done, we retain the species under *Apanteles*.

***Apantelesheichinensis* Sonan, 1942**

*Apantelesheichinensis* Sonan, 1942.

**Type information.** Holotype male, TARI (not examined but subsequent treatment of the species checked). Country of type locality: China.

**Geographical distribution.**OTL, PAL.

**OTL**: China (HN, TW, ZJ); **PAL**: China (AH).

**Notes.** For the generic placement of this species we follow Chen and Song (2004).

***Apanteleshellulae* Risbec, 1951**

*Apanteleshellulae* Risbec, 1951.

*Apanteleshellulaecrocidolomiae* Risbec, 1951 [primary junior homonym of *Apantelescrocidolomiae* Ahmad, 1945].

**Type information.** Syntypes female and male, depository unknown (not examined but original description checked). Country of type locality: Senegal.

**Geographical distribution.**AFR.

**AFR**: Senegal.

***Apanteleshemara* Nixon, 1965**

*Apanteleshemara* Nixon, 1965.

*Apantelescaboverdensis* Hedqvist, 1965.

*Apantelesproalastor* Hedqvist, 1965.

*Apantelesbulgaricus* Balevski & Tobias, 1980.

**Type information.** Holotype female, NHMUK (examined). Country of type locality: India.

**Geographical distribution.**AUS, AFR, OTL, PAL.

**AUS**: Australia (ACT); **AFR**: Cape Verde, Egypt, Kenya, Madagascar, Mauritius, Republic of the Congo, Senegal, South Africa, Yemen; **OTL**: China (HN), India, Pakistan, Vietnam; **PAL**: Bulgaria, Canary Islands, Cyprus, France, Greece, Iran, Israel, Italy, Madeira Islands, Oman, Russia (PRI), Spain, Saudi Arabia, Turkey, United Arab Emirates, Yugoslavia.

**Notes.** In Fernandez-Triana et al. (2017a: 3) this species is incorrectly listed as occurring in the Democratic Republic of Congo, when that record was actually from the Republic of Congo. Additional country distributions are also reported here, based on collections and DNA barcoding.

***Apanteleshemiaurantius* van Achterberg & Ng, 2009**

*Apanteleshemiaurantius* van Achterberg & Ng, 2009.

**Type information.** Holotype female, UKM (not examined but original description checked). Country of type locality: Malaysia.

**Geographical distribution.**OTL.

**OTL**: Malaysia.

***Apanteleshersilia* Nixon, 1965**

*Apanteleshersilia* Nixon, 1965.

**Type information.** Holotype female, NHMUK (examined). Country of type locality: South Africa.

**Geographical distribution.**AFR.

**AFR**: South Africa.

***Apantelesholmgreni* Shenefelt, 1972**

*Apantelesholmgreni* Shenefelt, 1972.

*Microgastercarbonarius* Holmgren, 1868 [primary junior homonym of *Microgastercarbonarius* Wesmael, 1837].

**Type information.** Holotype female, NHRS (not examined but subsequent treatment of the species checked). Country of type locality: Mauritius.

**Geographical distribution.**AFR.

**AFR**: Mauritius.

**Notes.** Our species concept is based on the comments that Wilkinson (1932a: 323) made on this species. However, examination of the type will be needed in the future to corroborate its generic placement.

***Apanteleshoraeus* Kotenko, 1986**

*Apanteleshoraeus* Kotenko, 1986.

**Type information.** Holotype female, SIZK (not examined but original description checked). Country of type locality: Ukraine.

**Geographical distribution.**PAL.

**PAL**: Russia (S), Ukraine.

***Apanteleshuberi* Fernandez-Triana, 2010**

*Apanteleshuberi* Fernandez-Triana, 2010.

**Type information.** Holotype female, CNC (examined). Country of type locality: Canada.

**Geographical distribution.**NEA.

**NEA**: Canada (BC).

***Apanteleshumbertolopezi* Fernandez-Triana, 2014**

*Apanteleshumbertolopezi* Fernandez-Triana, 2014.

**Type information.** Holotype female, CNC (examined). Country of type locality: Costa Rica.

**Geographical distribution.**NEO.

**NEO**: Costa Rica.

***Apanteleshyalinatus* Granger, 1949**

*Apanteleshyalinatus* Granger, 1949.

**Type information.** Syntypes female and male, MNHN (not examined but original description checked). Country of type locality: Madagascar.

**Geographical distribution.**AFR.

**AFR**: Madagascar.

***Apanteleshymeniae* Wilkinson, 1935**

*Apanteleshymeniae* Wilkinson, 1935.

**Type information.** Holotype female, NHMUK (examined). Country of type locality: Fiji.

**Geographical distribution.**AUS, OTL.

**AUS**: Fiji, Vietnam; **OTL**: Vietnam.

***Apantelesicarti* Blanchard, 1960**

*Apantelesicarti* Blanchard, 1960.

*Apantelesicartae* de Santis, 1967 [unjustified emendation].

**Type information.** Holotype female, DPBA (not examined). Country of type locality: Argentina.

**Geographical distribution.**NEO.

**NEO**: Argentina.

***Apantelesimitandus* Muesebeck, 1954**

*Apantelesimitandus* Muesebeck, 1954.

**Type information.** Holotype female, USNM (not examined but original description checked). Country of type locality: Brazil.

**Geographical distribution.**NEO.

**NEO**: Brazil (SP).

***Apantelesimpiger* Muesebeck, 1958**

*Apantelesimpiger* Muesebeck, 1958.

**Type information.** Holotype female, USNM (not examined but original description checked). Country of type locality: Puerto Rico.

**Geographical distribution.**NEO.

**NEO**: Cuba, Puerto Rico.

***Apantelesimportunus* Wilkinson, 1928**

*Apantelesimportunus* Wilkinson, 1928.

**Type information.** Holotype female, NHMUK (examined). Country of type locality: India.

**Geographical distribution.**OTL.

OTL: China (GX), India.

***Apantelesimpunctatus* Muesebeck, 1933**

*Apantelesimpunctatus* Muesebeck, 1933.

**Type information.** Holotype female, USNM (not examined but subsequent treatment of the species checked). Country of type locality: USA.

**Geographical distribution.**NEA.

**NEA**: USA (LA).

**Notes.** Our species concept is based on Austin and Dangerfield (1989).

***Apantelesinaron* Nixon, 1965**

*Apantelesinaron* Nixon, 1965.

**Type information.** Holotype female, NHMUK (examined). Country of type locality: South Africa.

**Geographical distribution.**AFR.

**AFR**: South Africa.

***Apantelesincurvus* Liu & Chen, 2014**

*Apantelesincurvus* Liu & Chen, 2014.

**Type information.** Holotype female, ZJUH (not examined but original description checked). Country of type locality: China.

**Geographical distribution.**PAL.

**PAL**: China (NX).

***Apantelesinesolisae* Fernandez-Triana, 2014**

*Apantelesinesolisae* Fernandez-Triana, 2014.

**Type information.** Holotype female, CNC (examined). Country of type locality: Costa Rica.

**Geographical distribution.**NEO.

**NEO**: Costa Rica.

***Apantelesinops* Nixon, 1965**

*Apantelesinops* Nixon, 1965.

**Type information.** Holotype female, USNM (examined). Country of type locality: Philippines.

**Geographical distribution.**OTL.

**OTL**: Philippines.

***Apantelesinsignicaudatus* Granger, 1949**

*Apantelesinsignicaudatus* Granger, 1949.

**Type information.** Syntypes female and male, MNHN (not examined but original description checked). Country of type locality: Madagascar.

**Geographical distribution.**AFR.

**AFR**: Madagascar.

***Apantelesinsularis* Muesebeck, 1921**

*Apantelesinsularis* Muesebeck, 1921.

*Urogastergrenadensis* Ashmead, 1900 [secondary homonym of *Cotesiagrenadensis* Ashmead, 1900].

**Type information.** Holotype female, NHMUK (examined). Country of type locality: Grenada.

**Geographical distribution.**NEO.

**NEO**: Grenada, Saint Vincent.

**Notes.** We also examined the type of *Urogastergrenadensis* in the NHMUK.

***Apantelesinunctus* Nixon, 1965**

*Apantelesinunctus* Nixon, 1965.

**Type information.** Holotype female, NHMUK (examined). Country of type locality: Malaysia.

**Geographical distribution.**OTL.

**OTL**: Malaysia.

***Apantelesione* Nixon, 1965**

*Apantelesione* Nixon, 1965.

**Type information.** Holotype female, NHMUK (examined). Country of type locality: South Africa.

**Geographical distribution.**AFR.

**AFR**: South Africa.

***Apantelesippeus* Nixon, 1965**

*Apantelesippeus* Nixon, 1965.

**Type information.** Holotype female, NHMUK (examined). Country of type locality: Australia.

**Geographical distribution.**AUS, OTL.

**AUS**: Australia (ACT, NSW, QLD); **OTL**: Vietnam.

***Apantelesirenecarrilloae* Fernandez-Triana, 2014, name amended**

*Apantelesirenecarrilloi* Fernandez-Triana, 2014 [incorrect original spelling].

**Type information.** Holotype female, CNC (examined). Country of type locality: Costa Rica.

**Geographical distribution.**NEO.

**NEO**: Costa Rica.

**Notes.** The original spelling of the species *Apantelesirenecarrilloi* is incorrect, as the species was named after Irene Carrillo, a woman, and thus its ending should be -*ae* instead of -*i*. The correct spelling is here amended to *Apantelesirenecarrilloae*.

***Apantelesisaacbermudezi* Fernandez-Triana, 2014**

*Apantelesisaacbermudezi* Fernandez-Triana, 2014.

**Type information.** Holotype female, CNC (examined). Country of type locality: Costa Rica.

**Geographical distribution.**NEO.

**NEO**: Costa Rica.

***Apantelesisander* Nixon, 1965**

*Apantelesisander* Nixon, 1965.

**Type information.** Holotype female, NHMUK (examined). Country of type locality: South Africa.

**Geographical distribution.**AFR, OTL.

**AFR**: South Africa; **OTL**: Vietnam.

***Apantelesisidrochaconi* Fernandez-Triana, 2014**

*Apantelesisidrochaconi* Fernandez-Triana, 2014.

**Type information.** Holotype female, CNC (examined). Country of type locality: Costa Rica.

**Geographical distribution.**NEO.

**NEO**: Costa Rica.

***Apantelesisidrovillegasi* Fernandez-Triana, 2014**

*Apantelesisidrovillegasi* Fernandez-Triana, 2014.

**Type information.** Holotype female, CNC (examined). Country of type locality: Costa Rica.

**Geographical distribution.**NEO.

**NEO**: Costa Rica.

***Apantelesivondroensis* Granger, 1949**

*Apantelesivondroensis* Granger, 1949.

**Type information.** Holotype female, MNHN (not examined but original description checked). Country of type locality: Madagascar.

**Geographical distribution.**AFR.

**AFR**: Madagascar.

***Apantelesivonnetranae* Fernandez-Triana, 2014**

*Apantelesivonnetranae* Fernandez-Triana, 2014.

**Type information.** Holotype female, CNC (examined). Country of type locality: Costa Rica.

**Geographical distribution.**NEO.

**NEO**: Costa Rica.

***Apantelesjairomoyai* Fernandez-Triana, 2014**

*Apantelesjairomoyai* Fernandez-Triana, 2014.

**Type information.** Holotype female, CNC (examined). Country of type locality: Costa Rica.

**Geographical distribution.**NEO.

**NEO**: Costa Rica.

***Apantelesjaviercontrerasi* Fernandez-Triana, 2014**

*Apantelesjaviercontrerasi* Fernandez-Triana, 2014.

**Type information.** Holotype female, CNC (examined). Country of type locality: Costa Rica.

**Geographical distribution.**NEO.

**NEO**: Costa Rica.

***Apantelesjavierobandoi* Fernandez-Triana, 2014**

*Apantelesjavierobandoi* Fernandez-Triana, 2014.

**Type information.** Holotype female, CNC (examined). Country of type locality: Costa Rica.

**Geographical distribution.**NEO.

**NEO**: Costa Rica.

***Apantelesjaviersihezari* Fernandez-Triana, 2014**

*Apantelesjaviersihezari* Fernandez-Triana, 2014.

**Type information.** Holotype female, CNC (examined). Country of type locality: Costa Rica.

**Geographical distribution.**NEO.

**NEO**: Costa Rica.

***Apantelesjenniferae* Fernandez-Triana, 2010**

*Apantelesjenniferae* Fernandez-Triana, 2010.

**Type information.** Holotype female, CNC (examined). Country of type locality: Canada.

**Geographical distribution.**NEA.

**NEA**: Canada (NB, ON, QC).

***Apantelesjesusbrenesi* Fernandez-Triana, 2014**

*Apantelesjesusbrenesi* Fernandez-Triana, 2014.

**Type information.** Holotype female, CNC (examined). Country of type locality: Costa Rica.

**Geographical distribution.**NEO.

**NEO**: Costa Rica.

***Apantelesjesusugaldei* Fernandez-Triana, 2014**

*Apantelesjesusugaldei* Fernandez-Triana, 2014.

**Type information.** Holotype female, CNC (examined). Country of type locality: Costa Rica.

**Geographical distribution.**NEO.

**NEO**: Costa Rica.

***Apantelesjimmychevezi* Fernandez-Triana, 2014**

*Apantelesjimmychevezi* Fernandez-Triana, 2014.

**Type information.** Holotype female, CNC (examined). Country of type locality: Costa Rica.

**Geographical distribution.**NEO.

**NEO**: Costa Rica.

***Apantelesjohanvargasi* Fernandez-Triana, 2014**

*Apantelesjohanvargasi* Fernandez-Triana, 2014.

**Type information.** Holotype female, CNC (examined). Country of type locality: Costa Rica.

**Geographical distribution.**NEO.

**NEO**: Costa Rica.

***Apantelesjorgecortesi* Fernandez-Triana, 2014**

*Apantelesjorgecortesi* Fernandez-Triana, 2014.

**Type information.** Holotype female, CNC (examined). Country of type locality: Costa Rica.

**Geographical distribution.**NEO.

**NEO**: Costa Rica.

***Apantelesjorgehernandezi* Fernandez-Triana, 2014**

*Apantelesjorgehernandezi* Fernandez-Triana, 2014.

**Type information.** Holotype female, CNC (examined). Country of type locality: Costa Rica.

**Geographical distribution.**NEO.

**NEO**: Costa Rica.

***Apantelesjosecalvoi* Fernandez-Triana, 2014**

*Apantelesjosecalvoi* Fernandez-Triana, 2014.

**Type information.** Holotype female, CNC (examined). Country of type locality: Costa Rica.

**Geographical distribution.**NEO.

**NEO**: Costa Rica.

***Apantelesjosecortezi* Fernandez-Triana, 2014**

*Apantelesjosecortezi* Fernandez-Triana, 2014.

**Type information.** Holotype female, CNC (examined). Country of type locality: Costa Rica.

**Geographical distribution.**NEO.

**NEO**: Costa Rica.

***Apantelesjosediazi* Fernandez-Triana, 2014**

*Apantelesjosediazi* Fernandez-Triana, 2014.

**Type information.** Holotype female, CNC (examined). Country of type locality: Costa Rica.

**Geographical distribution.**NEO.

**NEO**: Costa Rica.

***Apantelesjosejaramilloi* Fernandez-Triana, 2014**

*Apantelesjosejaramilloi* Fernandez-Triana, 2014.

**Type information.** Holotype female, CNC (examined). Country of type locality: Costa Rica.

**Geographical distribution.**NEO.

**NEO**: Costa Rica.

***Apantelesjosemonteroi* Fernandez-Triana, 2014**

*Apantelesjosemonteroi* Fernandez-Triana, 2014.

**Type information.** Holotype female, CNC (examined). Country of type locality: Costa Rica.

**Geographical distribution.**NEO.

**NEO**: Costa Rica.

***Apantelesjoseperezi* Fernandez-Triana, 2014**

*Apantelesjoseperezi* Fernandez-Triana, 2014.

**Type information.** Holotype female, CNC (examined). Country of type locality: Costa Rica.

**Geographical distribution.**NEO.

**NEO**: Costa Rica.

***Apantelesjoserasi* Fernandez-Triana, 2014**

*Apantelesjoserasi* Fernandez-Triana, 2014.

**Type information.** Holotype female, CNC (examined). Country of type locality: Costa Rica.

**Geographical distribution.**NEO.

**NEO**: Costa Rica.

***Apantelesjuanapuii* Fernandez-Triana, 2014**

*Apantelesjuanapuii* Fernandez-Triana, 2014.

**Type information.** Holotype female, CNC (examined). Country of type locality: Costa Rica.

**Geographical distribution.**NEO.

**NEO**: Costa Rica.

***Apantelesjuancarrilloi* Fernandez-Triana, 2014**

*Apantelesjuancarrilloi* Fernandez-Triana, 2014.

**Type information.** Holotype female, CNC (examined). Country of type locality: Costa Rica.

**Geographical distribution.**NEO.

**NEO**: Costa Rica.

***Apantelesjuangazoi* Fernandez-Triana, 2014**

*Apantelesjuangazoi* Fernandez-Triana, 2014.

**Type information.** Holotype female, CNC (examined). Country of type locality: Costa Rica.

**Geographical distribution.**NEO.

**NEO**: Costa Rica.

***Apantelesjuanhernandezi* Fernandez-Triana, 2014**

*Apantelesjuanhernandezi* Fernandez-Triana, 2014.

**Type information.** Holotype female, CNC (examined). Country of type locality: Costa Rica.

**Geographical distribution.**NEO.

**NEO**: Costa Rica.

***Apantelesjuanlopezi* Fernandez-Triana, 2014**

*Apantelesjuanlopezi* Fernandez-Triana, 2014.

**Type information.** Holotype female, CNC (examined). Country of type locality: Costa Rica.

**Geographical distribution.**NEO.

**NEO**: Costa Rica.

***Apantelesjuanmatai* Fernandez-Triana, 2014**

*Apantelesjuanmatai* Fernandez-Triana, 2014.

**Type information.** Holotype female, CNC (examined). Country of type locality: Costa Rica.

**Geographical distribution.**NEO.

**NEO**: Costa Rica.

***Apantelesjuanvictori* Fernandez-Triana, 2014**

*Apantelesjuanvictori* Fernandez-Triana, 2014.

**Type information.** Holotype female, CNC (examined). Country of type locality: Costa Rica.

**Geographical distribution.**NEO.

**NEO**: Costa Rica.

***Apantelesjubmeli* Hedqvist, 1972**

*Apantelesjubmeli* Hedqvist, 1972.

**Type information.** Holotype female, NHRS (examined). Country of type locality: Sweden.

**Geographical distribution.**PAL.

**PAL**: Sweden.

***Apantelesjuliodiazi* Fernandez-Triana, 2014**

*Apantelesjuliodiazi* Fernandez-Triana, 2014.

**Type information.** Holotype female, CNC (examined). Country of type locality: Costa Rica.

**Geographical distribution.**NEO.

**NEO**: Costa Rica.

***Apantelesjuniorlopezi* Fernandez-Triana, 2014**

*Apantelesjuniorlopezi* Fernandez-Triana, 2014.

**Type information.** Holotype female, CNC (examined). Country of type locality: Costa Rica.

**Geographical distribution.**NEO.

**NEO**: Costa Rica.

***Apanteleskeineraragoni* Fernandez-Triana, 2014**

*Apanteleskeineraragoni* Fernandez-Triana, 2014.

**Type information.** Holotype female, CNC (examined). Country of type locality: Costa Rica.

**Geographical distribution.**NEO.

**NEO**: Costa Rica.

***Apanteleskivuensis* de Saeger, 1941**

*Apanteleskivuensis* de Saeger, 1941.

**Type information.** Holotype female, RMCA (not examined but original description checked). Country of type locality: Democratic Republic of Congo.

**Geographical distribution.**AFR.

**AFR**: Democratic Republic of Congo.

***Apanteleskubensis* Abdinbekova, 1969**

*Apanteleskubensis* Abdinbekova, 1969.

**Type information.** Holotype female, ZIN (not examined but subsequent treatment of the species checked). Country of type locality: Azerbaijan.

**Geographical distribution.**PAL.

**PAL**: Azerbaijan, Hungary, Korea, Moldova, Mongolia, Russia (NC, S), Turkey.

**Notes.** Our species concept is based on Papp (1980a) and Tobias (1986).

***Apanteleslacteus* (Nees, 1834)**

*Microgasterlacteus* Nees, 1834.

**Type information.** Holotype female, lost (not examined but subsequent treatment of the species checked). Country of type locality: Germany.

**Geographical distribution.**PAL.

**PAL**: Armenia, Azerbaijan, Finland, Germany, Greece, Iran, Israel, Italy, Kazakhstan, Moldova, Poland, Romania, Russia (ORE, ROS, RYA, TAM), Slovakia, Sweden, Tajikistan, Tunisia, Turkey, Ukraine, United Kingdom, Uzbekistan.

**Notes.** This species was first transferred to *Dolichogenidea* (as *D.lacteus*) by Halperin (1986), following Papp’s identification of those specimens. Since then it has been variously treated as *Apanteles* or *Dolichogenidea* (e.g., Papp 1988, Shaw 2012b, Belokobylskij et al. 2003, Yu et al. 2012, 2016, Liu et al. 2015, Broad et al. 2016). For the sake of clarity, we revise the combination of this species here. The specimens we have examined all have a strongly concave hind wing vannal lobe, being clearly *Apanteles*.

***Apanteleslaevicoxis* Muesebeck, 1921**

*Apanteleslaevicoxis* Muesebeck, 1921.

**Type information.** Holotype female, USNM (examined). Country of type locality: USA.

**Geographical distribution.**NEA.

**NEA**: USA (MS).

***Apanteleslanassa* Nixon, 1965**

*Apanteleslanassa* Nixon, 1965.

**Type information.** Holotype female, NHMUK (examined). County of type locality: Malaysia.

**Geographical distribution.**OTL.

**OTL**: Malaysia.

***Apanteleslangenburgensis* Szépligeti, 1911**

*Apanteleslangenburgensis* Szépligeti, 1911.

**Type information.** Syntypes female and male, ZMHB (not examined but subsequent treatment of the species checked). Country of type locality: Tanzania.

**Geographical distribution.**AFR.

**AFR**: Democratic Republic of Congo, Ivory Coast, Malawi, Rwanda, Senegal, Tanzania.

**Notes.** Information about type specimens taken from Shenefelt (1972). Our concept of this species is based on Wilkinson (1932a), de Saeger (1944) and Risbec (1951).

***Apanteleslaricellae* Mason, 1959**

*Apanteleslaricellae* Mason, 1959.

**Type information.** Holotype female, CNC (examined). Country of type locality: Canada.

**Geographical distribution.**NEA.

**NEA**: Canada (NB, ON, QC), USA (WI).

***Apanteleslatericarinatus* Song & Chen, 2001**

*Apanteleslatericarinatus* Song & Chen, 2001.

**Type information.** Holotype female, FAFU (not examined but original description checked). Country of type locality: China.

**Geographical distribution.**OTL.

**OTL**: China (FJ, YN).

***Apanteleslatisulca* Chen & Song, 2004**

*Apanteleslatisulca* Chen & Song, 2004.

**Type information.** Holotype female, FAFU (not examined but original description checked). Country of type locality: China.

**Geographical distribution.**OTL.

**OTL**: China (FJ).

***Apanteleslaurahuberae* Fernandez-Triana, 2014**

*Apanteleslaurahuberae* Fernandez-Triana, 2014.

**Type information.** Holotype female, CNC (examined). Country of type locality: Costa Rica.

**Geographical distribution.**NEO.

**NEO**: Costa Rica.

***Apanteleslaurenmoralesae* Fernandez-Triana, 2014**

*Apanteleslaurenmoralesae* Fernandez-Triana, 2014.

**Type information.** Holotype female, CNC (examined). Country of type locality: Costa Rica.

**Geographical distribution.**NEO.

**NEO**: Costa Rica.

***Apanteleslavignei* Blanchard, 1959**

*Apanteleslavignei* Blanchard, 1959.

**Type information.** Holotype female, MACN (not examined). Country of type locality: Brazil.

**Geographical distribution.**NEO.

**NEO**: Brazil (BA).

**Notes.** The holotype was part of the Blanchard collection, which we assume is now deposited in the MACN.

***Apanteleslaxus* de Saeger, 1944**

*Apanteleslaxus* de Saeger, 1944.

**Type information.** Holotype female, RMCA (not examined but original description checked). Country of type locality: Democratic Republic of Congo.

**Geographical distribution.**AFR.

**AFR**: Democratic Republic of Congo.

***Apanteleslectus* Tobias, 1964**

*Apanteleslectus* Tobias, 1964.

**Type information.** Holotype female, ZIN (not examined but subsequent treatment of the species checked). Country of type locality: Kazakhstan.

**Geographical distribution.**PAL.

**PAL**: Kazakhstan, Lithuania, Macedonia, Mongolia, Russia (C, S), Yugoslavia.

**Notes.** Our species concept is based on Nixon (1976).

***Apanteleslenea* Nixon, 1976**

*Apanteleslenea* Nixon, 1976.

**Type information.** Holotype female, NHMUK (examined). Country of type locality: United Kingdom.

**Geographical distribution.**PAL.

**PAL**: Austria, Bulgaria, Czech Republic, France, Germany, Hungary, Ireland, Italy, Korea, Romania, Russia (ZAB, PRI, SAK), Serbia, Slovakia, Spain, Sweden, Switzerland, Turkey, United Kingdom.

***Apantelesleninguadamuzi* Fernandez-Triana, 2014**

*Apantelesleninguadamuzi* Fernandez-Triana, 2014.

**Type information.** Holotype female, CNC (examined). Country of type locality: Costa Rica.

**Geographical distribution.**NEO.

**NEO**: Costa Rica.

***Apantelesleonelgarayi* Fernandez-Triana, 2014**

*Apantelesleonelgarayi* Fernandez-Triana, 2014.

**Type information.** Holotype female, CNC (examined). Country of type locality: Costa Rica.

**Geographical distribution.**NEO.

**NEO**: Costa Rica.

***Apantelesleptothecus* (Cameron, 1907)**

*Pseudapantelesleptothecus* Cameron, 1907.

**Type information.** Holotype female, NHMUK (examined). Country of type locality: India.

**Geographical distribution.**OTL.

**OTL**: India.

**Notes.** The holotype is in very poor condition, missing the entire metasoma, the head badly smashed, and with the micropin (which was pinned through the mesosoma) corroding. However, the propodeum is clearly visible, as well as the vannal lobe of one hind wing. Based on that, it is still possible to corroborate the placement of this species within *Apanteles*, although probably the type would be mostly useless for a better characterization of the species.

***Apantelesleptoura* Cameron, 1909**

*Apantelesleptoura* Cameron, 1909.

**Type information.** Holotype female, NHMUK (examined). Country of type locality: Sri Lanka.

**Geographical distribution.**OTL.

**OTL**: China (HB, HN), Malaysia, Sri Lanka.

***Apantelesleucochiloneae* Cameron, 1911**

*Apantelesleucochiloneae* Cameron, 1911.

**Type information.** Syntypes female and male, NHMUK (examined). Country of type locality: Guyana.

**Geographical distribution.**NEO.

**NEO**: Guyana.

**Notes.**Yu et al. (2016) recorded the type as being female; however, we examined one female and one male specimens, both glued on the same card that has a type label, and thus are to be considered as syntypes, as correctly implied by Shenefelt (1972: 553).

***Apantelesleucopus* (Ashmead, 1900)**

*Urogasterleucopus* Ashmead, 1900.

**Type information.** Holotype female, NHMUK (examined). Country of type locality: Grenada.

**Geographical distribution.**NEO.

**NEO**: Grenada, Saint Vincent.

***Apantelesleucostigmus* (Ashmead, 1900)**

*Urogasterleucostigmus* Ashmead, 1900.

**Type information.** Holotype female, NHMUK (examined). Country of type locality: Saint Vincent.

**Geographical distribution.**NEO, NEA.

**NEA**: USA (FL); **NEO**: Cuba, Grenada, Puerto Rico, Saint Vincent.

***Apanteleslilliammenae* Fernandez-Triana, 2014**

*Apanteleslilliammenae* Fernandez-Triana, 2014.

**Type information.** Holotype female, CNC (examined). Country of type locality: Costa Rica.

**Geographical distribution.**NEO.

**NEO**: Costa Rica.

***Apanteleslineodos* Cameron, 1911**

*Apanteleslineodos* Cameron, 1911.

**Type information.** Holotype male, NHMUK (examined). Country of type locality: Guyana.

**Geographical distribution.**NEO.

**NEO**: Guyana.

**Notes.** Based on the carination and sculpture pattern of the propodeum and fore wing venation, this species belongs to the *leucostigmus* group (*sensu*Fernandez-Triana et al. 2014e).

***Apanteleslinus* Nixon, 1965**

*Apanteleslinus* Nixon, 1965.

**Type information.** Holotype female, NHMUK (examined). Country of type locality: South Africa.

**Geographical distribution.**AFR.

**AFR**: South Africa.

***Apantelesliopleuris* Szépligeti, 1914**

*Apantelesliopleuris* Szépligeti, 1914.

**Type information.** Holotype male, MNHN (not examined but original description checked). Country of type locality: Tanzania.

**Geographical distribution.**AFR.

**AFR**: Tanzania.

**Notes.** Only known from the male holotype (Wilkinson 1932a: 324). Without examining the type, it is not possible to conclude on the generic placement of this species.

***Apanteleslisabearssae* Fernandez-Triana, 2014**

*Apanteleslisabearssae* Fernandez-Triana, 2014.

**Type information.** Holotype female, CNC (examined). Country of type locality: Costa Rica.

**Geographical distribution.**NEO.

**NEO**: Costa Rica.

***Apanteleslongiantenna* Chen & Song, 2004**

*Apanteleslongiantenna* Chen & Song, 2004.

**Type information.** Holotype female, FAFU (not examined but original description checked). Country of type locality: China.

**Geographical distribution.**OTL.

**OTL**: China (FJ).

***Apanteleslongicaudatus* You & Zhou, 1991**

*Apanteleslongicaudatus* You & Zhou, 1991.

**Type information.** Holotype female, HUNAU (not examined but subsequent treatment of the species checked). Country of type locality: China.

**Geographical distribution.**OTL.

**OTL**: China (FJ, JX, ZJ).

**Notes.** The depository acronym was chosen from the English website of the Hunan Agricultural College (now Hunan Agricultural University). Our species concept is based on Liu et al. (2014).

***Apanteleslongirostris* Chen & Song, 2004**

*Apanteleslongirostris* Chen & Song, 2004.

**Type information.** Holotype female, FAFU (not examined but original description checked). Country of type locality: China.

**Geographical distribution.**OTL.

**OTL**: China (FJ, YN).

***Apanteleslongistylus* de Saeger, 1944**

*Apanteleslongistylus* de Saeger, 1944.

**Type information.** Holotype female, RMCA (not examined but original description checked). Country of type locality: Democratic Republic of Congo.

**Geographical distribution.**AFR.

**AFR**: Democratic Republic of Congo, Rwanda.

**Notes.** Based on the illustration of the ovipositor and ovipositor sheaths (de Saeger 1944), this could be either an *Apanteles* or *Dolichogenidea*. Until the vannal lobe of specimens are examined, it is not possible to conclude, thus we retain the species in the genus in which it was originally described.

***Apanteleslongitergiae* Rao & Kurian, 1950**

*Apanteleslongitergiae* Rao & Kurian, 1950.

**Type information.** Holotype female, NZSI (not examined but subsequent treatment of the species checked). Country of type locality: India.

**Geographical distribution.**OTL.

**OTL**: India.

**Notes.** Our species concept is based on Rao and Kurian (1950), Rao (1961), and Rao and Chalikwar (1971). The original description mentions “ovipositor sheaths short, exserted” which may suggest this species belongs to *Parapanteles*; however, because no other details are clear to conclude, we prefer to retain the species in *Apanteles* until specimens can be examined.

***Apantelesluciariosae* Fernandez-Triana, 2014**

*Apantelesluciariosae* Fernandez-Triana, 2014.

**Type information.** Holotype female, CNC (examined). Country of type locality: Costa Rica.

**Geographical distribution.**NEO.

**NEO**: Costa Rica.

***Apantelesluisbrizuelai* Fernandez-Triana, 2014**

*Apantelesluisbrizuelai* Fernandez-Triana, 2014.

**Type information.** Holotype female, CNC (examined). Country of type locality: Costa Rica.

**Geographical distribution.**NEO.

**NEO**: Costa Rica.

***Apantelesluiscanalesi* Fernandez-Triana, 2014**

*Apantelesluiscanalesi* Fernandez-Triana, 2014.

**Type information.** Holotype female, CNC (examined). Country of type locality: Costa Rica.

**Geographical distribution.**NEO.

**NEO**: Costa Rica.

***Apantelesluiscantillanoi* Fernandez-Triana, 2014**

*Apantelesluiscantillanoi* Fernandez-Triana, 2014.

**Type information.** Holotype female, CNC (examined). Country of type locality: Costa Rica.

**Geographical distribution.**NEO.

**NEO**: Costa Rica.

***Apantelesluisgarciai* Fernandez-Triana, 2014**

*Apantelesluisgarciai* Fernandez-Triana, 2014.

**Type information.** Holotype female, CNC (examined). Country of type locality: Costa Rica.

**Geographical distribution.**NEO.

**NEO**: Costa Rica.

***Apantelesluisgaritai* Fernandez-Triana, 2014**

*Apantelesluisgaritai* Fernandez-Triana, 2014.

**Type information.** Holotype female, CNC (examined). Country of type locality: Costa Rica.

**Geographical distribution.**NEO.

**NEO**: Costa Rica.

***Apantelesluishernandezi* Fernandez-Triana, 2014**

*Apantelesluishernandezi* Fernandez-Triana, 2014.

**Type information.** Holotype female, CNC (examined). Country of type locality: Costa Rica.

**Geographical distribution.**NEO.

**NEO**: Costa Rica.

***Apantelesluislopezi* Fernandez-Triana, 2014**

*Apantelesluislopezi* Fernandez-Triana, 2014.

**Type information.** Holotype female, CNC (examined). Country of type locality: Costa Rica.

**Geographical distribution.**NEO.

**NEO**: Costa Rica.

***Apantelesluisvargasi* Fernandez-Triana, 2014**

*Apantelesluisvargasi* Fernandez-Triana, 2014.

**Type information.** Holotype female, CNC (examined). Country of type locality: Costa Rica.

**Geographical distribution.**NEO.

**NEO**: Costa Rica.

***Apanteleslunata* Song & Chen, 2004**

*Apanteleslunata* Song & Chen, 2004.

**Type information.** Holotype female, FAFU (not examined but original description checked). Country of type locality: China.

**Geographical distribution.**OTL, PAL.

**OTL**: China (FJ, HB); **PAL**: China (JL).

***Apantelesluteocinctus* de Saeger, 1941**

*Apantelesluteocinctus* de Saeger, 1941.

**Type information.** Holotype female, RMCA (not examined but original description checked). Country of type locality: Democratic Republic of Congo.

**Geographical distribution.**AFR.

**AFR**: Democratic Republic of Congo, Rwanda.

***Apantelesluzmariaromeroae* Fernandez-Triana, 2014**

*Apantelesluzmariaromeroae* Fernandez-Triana, 2014.

**Type information.** Holotype female, CNC (examined). Country of type locality: Costa Rica.

**Geographical distribution.**NEO.

**NEO**: Costa Rica.

***Apanteleslycidas* Nixon, 1965**

*Apanteleslycidas* Nixon, 1965.

**Type information.** Holotype female, NHMUK (examined). Country of type locality: South Africa.

**Geographical distribution.**AFR.

**AFR**: South Africa.

***Apanteleslyridice* Nixon, 1965**

*Apanteleslyridice* Nixon, 1965.

**Type information.** Holotype female, AEIC (not examined but original description checked). Country of type locality: Philippines.

**Geographical distribution.**OTL.

**OTL**: Philippines, Vietnam.

***Apantelesmachaeralis* Wilkinson, 1928**

*Apantelesmachaeralis* Wilkinson, 1928.

**Type information.** Holotype female, NHMUK (examined). Country of type locality: India.

**Geographical distribution.**OTL.

**OTL**: China (GD), India, Myanmar, Vietnam.

***Apantelesmacromphaliae* Silva Figueroa, 1917**

*Apantelesmacromphaliae* Silva Figueroa, 1917.

**Type information.** Syntypes female, MNNC (not examined but subsequent treatment of the species checked). Country of type locality: Chile.

**Geographical distribution.**NEO.

**NEO**: Argentina, Chile.

**Notes.**Shenefelt (1972: 563) stated that the original description of the species mentioned female and male specimens, which would mean that they were syntypes; however, Yu et al. (2016) recorded the type of this species as a female; neither publications specified the depository of the specimens. We have found a local reference in Spanish that clearly states that the species was described based on 13 female syntypes, deposited in the MNNC (Camousseight 1975: 5), and we are following this source here. The generic placement of this species is impossible to define until the original material is examined.

***Apantelesmagnioculus* Liu & Chen, 2015**

*Apantelesmagnioculus* Liu & Chen, 2015.

**Type information.** Holotype female, ZJUH (not examined but original description checked). Country of type locality: China.

**Geographical distribution.**OTL.

**OTL**: China (GZ).

***Apantelesmalleus* Liu & Chen, 2014**

*Apantelesmalleus* Liu & Chen, 2014.

**Type information.** Holotype female, ZJUH (not examined but original description checked). Country of type locality: China.

**Geographical distribution.**PAL.

**PAL**: China (HE).

***Apantelesmamitus* Nixon, 1965**

*Apantelesmamitus* Nixon, 1965.

**Type information.** Holotype female, AEIC (not examined but original description checked). Country of type locality: Philippines.

**Geographical distribution.**OTL.

**OTL**: China (FJ, JX, TW), India, Philippines, Vietnam.

***Apantelesmanuelarayai* Fernandez-Triana, 2014**

*Apantelesmanuelarayai* Fernandez-Triana, 2014.

**Type information.** Holotype female, CNC (examined). Country of type locality: Costa Rica.

**Geographical distribution.**NEO.

**NEO**: Costa Rica.

***Apantelesmanuelpereirai* Fernandez-Triana, 2014**

*Apantelesmanuelpereirai* Fernandez-Triana, 2014.

**Type information.** Holotype female, CNC (examined). Country of type locality: Costa Rica.

**Geographical distribution.**NEO.

**NEO**: Costa Rica.

***Apantelesmanuelriosi* Fernandez-Triana, 2014**

*Apantelesmanuelriosi* Fernandez-Triana, 2014.

**Type information.** Holotype female, CNC (examined). Country of type locality: Costa Rica.

**Geographical distribution.**NEO.

**NEO**: Costa Rica.

***Apantelesmanuelzumbadoi* Fernandez-Triana, 2014**

*Apantelesmanuelzumbadoi* Fernandez-Triana, 2014.

**Type information.** Holotype female, CNC (examined). Country of type locality: Costa Rica.

**Geographical distribution.**NEO.

**NEO**: Costa Rica.

***Apantelesmarcobustosi* Fernandez-Triana, 2014**

*Apantelesmarcobustosi* Fernandez-Triana, 2014.

**Type information.** Holotype female, CNC (examined). Country of type locality: Costa Rica.

**Geographical distribution.**NEO.

**NEO**: Costa Rica.

***Apantelesmarcogonzalezi* Fernandez-Triana, 2014**

*Apantelesmarcogonzalezi* Fernandez-Triana, 2014.

**Type information.** Holotype female, CNC (examined). Country of type locality: Costa Rica.

**Geographical distribution.**NEO.

**NEO**: Costa Rica.

***Apantelesmarcovenicioi* Fernandez-Triana, 2014**

*Apantelesmarcovenicioi* Fernandez-Triana, 2014.

**Type information.** Holotype female, CNC (examined). Country of type locality: Costa Rica.

**Geographical distribution.**NEO.

**NEO**: Costa Rica.

***Apantelesmariachavarriae* Fernandez-Triana, 2014**

*Apantelesmariachavarriae* Fernandez-Triana, 2014.

**Type information.** Holotype female, CNC (examined). Country of type locality: Costa Rica.

**Geographical distribution.**NEO.

**NEO**: Costa Rica.

***Apantelesmariaguevarae* Fernandez-Triana, 2014**

*Apantelesmariaguevarae* Fernandez-Triana, 2014.

**Type information.** Holotype female, CNC (examined). Country of type locality: Costa Rica.

**Geographical distribution.**NEO.

**NEO**: Costa Rica.

***Apantelesmarialuisariasae* Fernandez-Triana, 2014**

*Apantelesmarialuisariasae* Fernandez-Triana, 2014.

**Type information.** Holotype female, CNC (examined). Country of type locality: Costa Rica.

**Geographical distribution.**NEO.

**NEO**: Costa Rica.

***Apantelesmariamendezae* Fernandez-Triana, 2014**

*Apantelesmariamendezae* Fernandez-Triana, 2014.

**Type information.** Holotype female, CNC (examined). Country of type locality: Costa Rica.

**Geographical distribution.**NEO.

**NEO**: Costa Rica.

***Apantelesmarianopereirai* Fernandez-Triana, 2014**

*Apantelesmarianopereirai* Fernandez-Triana, 2014.

**Type information.** Holotype female, CNC (examined). Country of type locality: Costa Rica.

**Geographical distribution.**NEO.

**NEO**: Costa Rica.

***Apantelesmariatorrentesae* Fernandez-Triana, 2014**

*Apantelesmariatorrentesae* Fernandez-Triana, 2014.

**Type information.** Holotype female, CNC (examined). Country of type locality: Costa Rica.

**Geographical distribution.**NEO.

**NEO**: Costa Rica.

***Apantelesmarisolarroyoae* Fernandez-Triana, 2014**

*Apantelesmarisolarroyoae* Fernandez-Triana, 2014.

**Type information.** Holotype female, CNC (examined). Country of type locality: Costa Rica.

**Geographical distribution.**NEO.

**NEO**: Costa Rica.

***Apantelesmarisolnavarroae* Fernandez-Triana, 2014**

*Apantelesmarisolnavarroae* Fernandez-Triana, 2014.

**Type information.** Holotype female, CNC (examined). Country of type locality: Costa Rica.

**Geographical distribution.**NEO.

**NEO**: Costa Rica.

***Apantelesmarvinmendozai* Fernandez-Triana, 2014**

*Apantelesmarvinmendozai* Fernandez-Triana, 2014.

**Type information.** Holotype female, CNC (examined). Country of type locality: Costa Rica.

**Geographical distribution.**NEO.

**NEO**: Costa Rica.

***Apantelesmasoni* Chen & Song, 2004**

*Apantelesmasoni* Chen & Song, 2004.

**Type information.** Holotype female, FAFU (not examined but original description checked). Country of type locality: China.

**Geographical distribution.**OTL.

**OTL**: China (FJ, HI, YN).

***Apantelesmauriciogurdiani* Fernandez-Triana, 2014**

*Apantelesmauriciogurdiani* Fernandez-Triana, 2014.

**Type information.** Holotype female, CNC (examined). Country of type locality: Costa Rica.

**Geographical distribution.**NEO.

**NEO**: Costa Rica.

***Apantelesmedioexcavatus* Granger, 1949**

*Apantelesmedioexcavatus* Granger, 1949.

**Type information.** Syntypes female and male, MNHN (not examined but original description checked). Country of type locality: Madagascar.

**Geographical distribution.**AFR.

**AFR**: Madagascar.

***Apantelesmedioimpressus* Granger, 1949**

*Apantelesmedioimpressus* Granger, 1949.

**Type information.** Syntypes female and male, MNHN (not examined but original description checked). Country of type locality: Madagascar.

**Geographical distribution.**AFR.

**AFR**: Madagascar.

***Apantelesmedon* Nixon, 1965**

*Apantelesmedon* Nixon, 1965.

**Type information.** Holotype female, NHMUK (examined). Country of type locality: Malaysia.

**Geographical distribution.**OTL.

**OTL**: Malaysia, Vietnam.

***Apantelesmegastidis* Muesebeck, 1958**

*Apantelesmegastidis* Muesebeck, 1958.

**Type information.** Holotype female, USNM (examined). Country of type locality: Trinidad & Tobago.

**Geographical distribution.**NEO.

**NEO**: Trinidad & Tobago.

***Apantelesmegathymi* Riley, 1881**

*Apantelesmegathymi* Riley, 1881.

**Type information.** Syntypes male, USNM (examined). Country of type locality: USA.

**Geographical distribution.**NEA, NEO.

**NEA**: USA (AZ, CA, NC, SC); **NEO**: Mexico.

***Apantelesmehdialii* Rao & Chalikwar, 1970**

*Apantelesmehdialii* Rao & Chalikwar, 1970.

**Type information.** Holotype female, depository unknown (not examined). Country of type locality: India.

**Geographical distribution.**OTL.

**OTL**: India.

***Apantelesmelpomene* Nixon, 1965**

*Apantelesmelpomene* Nixon, 1965.

**Type information.** Holotype female, NHMUK (examined). Country of type locality: Malaysia.

**Geographical distribution.**OTL.

**OTL**: Malaysia.

**Notes.** The propodeum sculpture is somewhat atypical for *Apanteles*, as noted by Nixon (1965); nevertheless, we think at present this is still the best generic placement for the species.

***Apantelesmenes* Nixon, 1965**

*Apantelesmenes* Nixon, 1965.

**Type information.** Holotype female, NHMUK (examined). Country of type locality: South Africa.

**Geographical distribution.**AFR.

**AFR**: South Africa.

***Apantelesmeriones* Nixon, 1965**

*Apantelesmeriones* Nixon, 1965.

**Type information.** Holotype female, NHMUK (examined). Country of type locality: South Africa.

**Geographical distribution.**AFR.

**AFR**: South Africa.

***Apantelesmetacarpalis* (Thomson, 1895)**

*Microgastermetacarpalis* Thomson, 1895.

**Type information.** Lectotype female, MZLU (not examined but subsequent treatment of the species checked). Country of type locality: Sweden.

**Geographical distribution.**PAL.

**PAL**: Azerbaijan, China (SN), Czech Republic, Finland, France, Germany, Greece, Hungary, Ireland, Italy, Korea, Malta, Moldova, Mongolia, Romania, Russia (PRI), Serbia, Spain, Sweden, Tajikistan, Tunisia, United Kingdom, Ukraine, Uzbekistan.

**Notes.** Our species concept is based on Nixon (1965, 1973) and Liu et al. (2014).

***Apantelesmetacarpellatus* Blanchard, 1963**

*Apantelesmetacarpellatus* Blanchard, 1963.

**Type information.** Holotype female, MACN (not examined but original description checked). Country of type locality: Argentina.

**Geographical distribution.**NEO.

**NEO**: Argentina.

***Apantelesmetagenes* Nixon, 1965**

*Apantelesmetagenes* Nixon, 1965.

**Type information.** Holotype female, NHMUK (examined). Country of type locality: India.

**Geographical distribution.**OTL.

**OTL**: India.

***Apantelesmetellus* Nixon, 1965**

*Apantelesmetellus* Nixon, 1965.

**Type information.** Holotype female, NHMUK (examined). Country of type locality: South Africa.

**Geographical distribution.**AFR.

**AFR**: South Africa.

***Apantelesmilenagutierrezae* Fernandez-Triana, 2014**

*Apantelesmilenagutierrezae* Fernandez-Triana, 2014.

**Type information.** Holotype female, CNC (examined). Country of type locality: Costa Rica.

**Geographical distribution.**NEO.

**NEO**: Costa Rica.

***Apantelesmilleri* Mason, 1974**

*Apantelesmilleri* Mason, 1974.

**Type information.** Holotype female, CNC (examined). Country of type locality: Canada.

**Geographical distribution.**NEA.

**NEA**: Canada (BC, NB, NT, ON, QC), USA (MT).

***Apantelesmimoristae* Muesebeck, 1922**

*Apantelesmimoristae* Muesebeck, 1922.

**Type information.** Holotype female, USNM (not examined but original description checked). Country of type locality: USA.

**Geographical distribution.**NEA.

**NEA**: USA (FL, TX).

***Apantelesminatchy* Rousse & Gupta, 2013**

*Apantelesminatchy* Rousse & Gupta, 2013.

**Type information.** Holotype female, MNHN (not examined but original description checked). Country of type locality: Réunion.

**Geographical distribution.**AFR.

**AFR**: Réunion.

***Apantelesminator* Muesebeck, 1957**

*Apantelesminator* Muesebeck, 1957.

**Type information.** Holotype female, USNM (examined). Country of type locality: Argentina.

**Geographical distribution.**NEA, NEO.

**NEA**: USA (TX); **NEO**: Argentina, Bolivia, Peru.

***Apantelesminor* Fahringer, 1938**

*Apantelesminor* Fahringer, 1938.

**Type information.** Holotype male, depository unknown (not examined but original description checked). Country of type locality: China.

**Geographical distribution.**PAL.

**PAL**: China (JS).

**Notes.**Williams (1988: 562) considered *Apantelesfalcatusminor* Fahringer, 1938, originally described as a subspecies of *Sathonfalcatus* (Nees, 1834), to be a different species. Williams elevated it to species rank and placed it in *Apanteles*; however, he also wrote that the *Apantelesminor* type had “a sparsely setose vannal lobe of the hind wing”. While this might indicate that the species is better placed in *Dolichogenidea* instead of *Apanteles*, for the time being we prefer to follow Williams (1988), as we have not been able to study the type of *minor*.

***Apantelesminorcarmonai* Fernandez-Triana, 2014**

*Apantelesminorcarmonai* Fernandez-Triana, 2014.

**Type information.** Holotype female, CNC (examined). Country of type locality: Costa Rica.

**Geographical distribution.**NEO.

**NEO**: Costa Rica.

***Apantelesminornavarroi* Fernandez-Triana, 2014**

*Apantelesminornavarroi* Fernandez-Triana, 2014.

**Type information.** Holotype female, CNC (examined). Country of type locality: Costa Rica.

**Geographical distribution.**NEO.

**NEO**: Costa Rica.

***Apantelesmiramis* Nixon, 1976**

*Apantelesmiramis* Nixon, 1976.

**Type information.** Holotype female, NHMUK (examined). Country of type locality: United Kingdom.

**Geographical distribution.**PAL.

**PAL**: Finland, United Kingdom.

***Apantelesmohandasi* Sumodan & Narendran, 1990**

*Apantelesmohandasi* Sumodan & Narendran, 1990.

**Type information.** Holotype female, RMNH (not examined but subsequent treatment of the species checked). Country of type locality: India.

**Geographical distribution.**OTL.

**OTL**: India.

**Notes.**van Achterberg and Narendran (1997) transferred the species to *Dolichogenidea*. Later, Gupta et al. (2011) transferred it back to *Apanteles* based on the hind wing vannal lobe being concave and without setae.

***Apantelesmonicachavarriae* Fernandez-Triana, 2014**

*Apantelesmonicachavarriae* Fernandez-Triana, 2014.

**Type information.** Holotype female, CNC (examined). Country of type locality: Costa Rica.

**Geographical distribution.**NEO.

**NEO**: Costa Rica.

***Apantelesmontezumae* Sánchez, Figueroa & Whitfield, 2015**

*Apantelesmontezumae* Sánchez, Figueroa & Whitfield, 2015.

**Type information.** Holotype female, IIAF (not examined but original description checked). Country of type locality: Mexico.

**Geographical distribution.**NEO.

**NEO**: Mexico.

**Notes.** The last name of the first author of the paper is Sánchez-García, as spelled on the title page (Sánchez-García et al. 2015: 10). However, for the species description in the Systematics section, only Sánchez was used (Sánchez-García et al. 2015: 11); thus, the authors of the species must be considered to be Sánchez, Figueroa and Whitfield.

***Apantelesmorrisi* Mason, 1974**

*Apantelesmorrisi* Mason, 1974.

**Type information.** Holotype female, CNC (examined). Country of type locality: Canada.

**Geographical distribution.**NEA, PAL.

**NEA**: Canada (BC, MB, NB, ON, QC), USA (MI, WI) **PAL**: Germany.

***Apantelesmorroensis* Nixon, 1955**

*Apantelesmorroensis* Nixon, 1955.

**Type information.** Holotype female, NHMUK (examined). Country of type locality: Juan Fernández Islands.

**Geographical distribution.**NEO.

**NEO**: Juan Fernández Islands.

**Notes.** Both the original description and references afterwards (e.g., Shenefelt 1972, Yu et al. 2016) refer to the type to be deposited in “the University of Santiago, Chile”. However, we have examined the type which is in NHMUK.

***Apantelesmujtabai* Bhatnagar, 1950**

*Apantelesmujtabai* Bhatnagar, 1950.

**Type information.** Holotype male, INPC (not examined but original description checked). Country of type locality: India.

**Geographical distribution.**OTL.

**OTL**: India.

**Notes.** The original description suggests that this species may not belong to *Apanteles*, based on the relatively short ovipositor sheaths; however, there are not enough details in the rest of the description to come to a conclusion, so in this paper we retain *mujtabai* in the genus it was originally described until specimens can be studied. The year of publication of the Bhatnagar paper was until recently commonly cited as 1948 and/or 1950 (e.g., Chen and Song 2004, Yu et al. 2016), probably following Shenefelt (1972) who referred to this paper as “Bhatnagar (1948) 1950”. While the intended year for Volume X, Parts I & II of the Indian Journal of Entomology was 1948, the actual dates of publication were June 1950 (Part I) and October 1950 (Part II), as clearly shown on the cover page of the Volume, which we have checked. Because the dates of publication are the ones to be considered, and for the sake of clarity, we hereby revise the year of description for this species to 1950.

***Apantelesmunnarensis* Sumodan & Narendran, 1990**

*Apantelesmunnarensis* Sumodan & Narendran, 1990.

**Type information.** Holotype female, RMNH (not examined but subsequent treatment of the species checked). Country of type locality: India.

**Geographical distribution.**OTL.

**OTL**: India.

**Notes.** Our species concept is based on van Achterberg and Narendran (1997).

***Apantelesmurcia* Nixon, 1965**

*Apantelesmurcia* Nixon, 1965.

**Type information.** Holotype female, USNM (examined). Country of type locality: Singapore.

**Geographical distribution.**OTL.

**OTL**: Singapore.

***Apantelesmuticiculus* Liu & Chen, 2014**

*Apantelesmuticiculus* Liu & Chen, 2014.

**Type information.** Holotype female, ZJUH (not examined but original description checked). Country of type locality: China.

**Geographical distribution.**OTL.

**OTL**: China (FJ).

***Apantelesmutilia* Nixon, 1965**

*Apantelesmutilia* Nixon, 1965.

**Type information.** Holotype female, NHMUK (examined). Country of type locality: Sudan.

**Geographical distribution.**AFR.

**AFR**: Sudan.

***Apantelesmycerinus* Nixon, 1965**

*Apantelesmycerinus* Nixon, 1965.

**Type information.** Holotype female, NHMUK (examined). Country of type locality: South Africa.

**Geographical distribution.**AFR, OTL.

**AFR**: South Africa; **OTL**: Vietnam.

**Notes.** Before 2014, this species was only known from three specimens from South Africa (Nixon 1965: 57). A recent record of this species from Vietnam (Long and van Achterberg 2014) should be considered suspicious because that paper does not claim to be the first record of the species for Vietnam (but no other published reference can be found), the authors did not see the type material of the species, and the geographical distribution of the specimens is disparate.

***Apantelesmycetophilus* Wilkinson, 1931**

*Apantelesmycetophilus* Wilkinson, 1931.

**Type information.** Holotype female, NHMUK (examined). Country of type locality: India.

**Geographical distribution.**OTL.

**OTL**: India.

***Apantelesmyrsus* Nixon, 1965**

*Apantelesmyrsus* Nixon, 1965.

**Type information.** Holotype female, USNM (examined). Country of type locality: Philippines.

**Geographical distribution.**OTL.

**OTL**: Philippines.

***Apantelesnamkumensis* Gupta, 1957**

*Apantelesnamkumensis* Gupta, 1957.

**Type information.** Holotype female, FSCA? (not examined but original description checked). Country of type locality: India.

**Geographical distribution.**OTL.

**OTL**: India.

**Notes.** The original description refers to the Gupta collection, which we assume to be currently deposited in FSCA.

***Apantelesnatras* Nixon, 1965**

*Apantelesnatras* Nixon, 1965.

**Type information.** Holotype female, AEIC (not examined but original description checked). Country of type locality: Philippines.

**Geographical distribution.**OTL.

**OTL**: Philippines.

***Apantelesnavius* Nixon, 1965**

*Apantelesnavius* Nixon, 1965.

**Type information.** Holotype female, NHMUK (examined). Country of type locality: South Africa.

**Geographical distribution.**AFR.

**AFR**: South Africa.

***Apantelesnemesis* Nixon, 1965**

*Apantelesnemesis* Nixon, 1965.

**Type information.** Holotype female, NHMUK (examined). Country of type locality: South Africa.

**Geographical distribution.**AFR.

**AFR**: South Africa.

***Apantelesneotaeniaticornis* Yousuf & Ray, 2010**

*Apantelesneotaeniaticornis* Yousuf & Ray, 2010.

**Type information.** Holotype female, IFRI (not examined but original description checked). Country of type locality: India.

**Geographical distribution.**OTL.

**OTL**: India.

***Apantelesnepe* Nixon, 1965**

*Apantelesnepe* Nixon, 1965.

**Type information.** Holotype female, NHMUK (examined). Country of type locality: South Africa.

**Geographical distribution.**AFR.

**AFR**: South Africa.

**Notes.** The holotype (only known specimen) has all the wings glued together, so it is not possible to see the vannal lobe in the hind wings with clarity.

***Apantelesnephereus* Nixon, 1965**

*Apantelesnephereus* Nixon, 1965.

**Type information.** Holotype female, USNM (examined). Country of type locality: Philippines.

**Geographical distribution.**OTL.

**OTL**: Philippines.

***Apantelesnephoptericis* (Packard, 1864)**

*Microgasternephoptericis* Packard, 1864.

*Apantelesephestiae* Baker, 1895.

**Type information.** Holotype sex unknown, MCZ (not examined but subsequent treatment of the species checked). Country of type locality: USA.

**Geographical distribution.**NEA.

**NEA**: Canada (ON), USA (AR, CA, CO, FL, IL, IN, IA, KS, NV, NJ, NY, OH, OR).

**Notes.** Our species concept is based on Whitfield et al. (2001). Shenefelt (1972: 578) stated that the type consisted of only one fore wing. Thus, it is not possible to determine the sex of the type.

***Apantelesnephus* Papp, 1974**

*Apantelesnephus* Papp, 1974.

**Type information.** Holotype female, HNHM (not examined but original description checked). Country of type locality: Hungary.

**Geographical distribution.**PAL.

**PAL**: Hungary, Russia (PRI), Ukraine.

***Apantelesniceppe* Nixon, 1965**

*Apantelesniceppe* Nixon, 1965.

**Type information.** Holotype female, AEIC (not examined but original description checked). Country of type locality: Philippines.

**Geographical distribution.**OTL.

**OTL**: Philippines, Vietnam.

***Apantelesnidophilus* Whitfield & Cameron, 2001**

*Apantelesnidophilus* Whitfield & Cameron, 2001.

**Type information.** Holotype female, USNM (examined). Country of type locality: Ecuador.

**Geographical distribution.**NEO.

**NEO**: Brazil (AM, SP), Colombia, Ecuador, Peru.

**Notes.** The holotype is missing the head and part of one of the hind legs.

***Apantelesnigrofemoratus* Granger, 1949**

*Apantelesnigrofemoratus* Granger, 1949.

**Type information.** Syntypes female and male, MNHN (not examined but original description checked). Country of type locality: Madagascar.

**Geographical distribution.**AFR.

**AFR**: Madagascar, Réunion.

***Apantelesninigretorum* Viereck, 1917**

*Apantelesninigretorum* Viereck, 1917.

**Type information.** Type lost (not examined but subsequent treatment of the species checked). Country of type locality: USA.

**Geographical distribution.**NEA.

**NEA**: USA (CT).

**Notes.** Our species concept is based on Muesebeck (1921).

***Apantelesnitidus* de Saeger, 1944**

*Apantelesnitidus* de Saeger, 1944.

**Type information.** Holotype female, RMCA (not examined but original description checked). Country of type locality: Rwanda.

**Geographical distribution.**AFR.

**AFR**: Rwanda.

**Notes.***Dolichogenidea* cannot be discarded as a potential generic placement for this species; however, until the hind wing vannal lobe of the holotype can be examined, we prefer to retain it in *Apanteles*.

***Apantelesnivellus* Nixon, 1965**

*Apantelesnivellus* Nixon, 1965.

**Type information.** Holotype female, NHMUK (examined). Country of type locality: Ghana.

**Geographical distribution.**AFR.

**AFR**: Ghana.

***Apantelesnixoni* Song, 2002**

*Apantelesnixoni* Song, 2002.

*Apantelesnixoni* Song, 2002 [primary junior homonym of *Apantelesnixoni* Papp, 1971].

**Type information.** Holotype female, FAFU (not examined but subsequent treatment of the species checked). Country of type locality: China.

**Geographical distribution.**OTL, PAL.

**OTL**: China (FJ, HB); **PAL**: China (JL).

**Notes.** Our species concept is based on Chen and Song (2004).

***Apantelesnoronhai* de Santis, 1975**

*Apantelesnoronhai* de Santis, 1975.

**Type information.** Holotype female, MLP (not examined but subsequent treatment of the species checked). Country of type locality: Brazil.

**Geographical distribution.**NEO.

**NEO**: Brazil (Fernando de Noronha Is, PE).

**Notes.** Our concept of this species is based on Aquino et al. (2010).

***Apantelesnovatus* Nixon, 1965**

*Apantelesnovatus* Nixon, 1965.

**Type information.** Holotype female, NHMUK (examined). Country of type locality: South Africa.

**Geographical distribution.**AFR.

**AFR**: South Africa.

***Apantelesnycon* Nixon, 1965**

*Apantelesnycon* Nixon, 1965.

**Type information.** Holotype female, NHMUK (examined). Country of type locality: South Africa.

**Geographical distribution.**AFR.

**AFR**: South Africa.

***Apantelesnymphis* Nixon, 1965**

*Apantelesnymphis* Nixon, 1965.

**Type information.** Holotype female, NHMUK (examined). Country of type locality: Indonesia.

**Geographical distribution.**OTL.

**OTL**: Indonesia.

***Apantelesoatmani* Marsh, 1979**

*Apantelesoatmani* Marsh, 1979.

**Type information.** Holotype female, USNM (not examined but original description checked). Country of type locality: Colombia.

**Geographical distribution.**NEO.

**NEO**: Colombia.

***Apantelesobscurus* (Nees, 1834)**

*Microgasterobscura* Nees, 1834.

*Microgasterarenarius* Haliday, 1834.

**Type information.** Lectotype female, ZMHB (not examined but subsequent treatment of the species checked). Country of type locality: Germany.

**Geographical distribution.**PAL.

**PAL**: Albania, Armenia, Azerbaijan, Belgium, Croatia, Denmark, Finland, France, Georgia, Germany, Greece, Hungary, Iran, Ireland, Israel, Italy, Kazakhstan, Lithuania, Macedonia, Moldova, Mongolia, Montenegro, Netherlands, Poland, Romania, Russia (KDA, KYA, PRI, SAK, SPE, YAR), Serbia, Slovakia, Slovenia, Spain, Sweden, Switzerland, Tunisia, Turkey, United Kingdom.

**Notes.** Our species concept is based on Wilkinson (1945), Nixon (1976), Papp (1980a), and Kotenko (2007a).

***Apantelesoculatus* Tobias, 1967**

*Apantelesoculatus* Tobias, 1967.

**Type information.** Holotype male, ZIN (not examined but subsequent treatment of the species checked). Country of type locality: Turkmenistan.

**Geographical distribution.**PAL.

**PAL**: Turkmenistan, Uzbekistan.

**Notes.** Our species concept is based on Papp (1984a).

***Apantelesodites* Nixon, 1965**

*Apantelesodites* Nixon, 1965.

**Type information.** Holotype female, USNM (examined). Country of type locality: Philippines.

**Geographical distribution.**OTL.

**OTL**: China (ZJ), Philippines.

***Apantelesoenone* Nixon, 1965**

*Apantelesoenone* Nixon, 1965.

**Type information.** Holotype female, NHMUK (examined). Country of type locality: Australia.

**Geographical distribution.**AUS, OTL.

**AUS**: Australia (NT, QLD, WA); **OTL**: Vietnam.

***Apantelesolorus* Nixon, 1965**

*Apantelesolorus* Nixon, 1965.

**Type information.** Holotype female, NHMUK (examined). Country of type locality: South Africa.

**Geographical distribution.**AFR.

**AFR**: South Africa.

***Apantelesopacus* (Ashmead, 1905)**

*Urogasteropacus* Ashmead, 1905.

*Apantelesderogatae* Watanabe, 1935.

**Type information.** Holotype female, USNM (examined). Country of type locality: Philippines.

**Geographical distribution.**AUS, OTL, PAL.

**AUS**: Hawaiian Islands; **OTL**: China (FJ, GX, HN, JS, SH, SN, ZJ), India, Indonesia, Malaysia, Philippines, Vietnam; **PAL**: China (SD), Japan.

**Notes.** The species was recorded from the Hawaiian Islands as an adventive species (Nishida 2002), but has been later found to be common (Howarth et al. 2012).

***Apantelesopuntiarum* Martínez & Berta, 2012**

*Apantelesopuntiarum* Martínez & Berta, 2012.

**Type information.** Holotype female, MACN (not examined but original description checked). Country of type locality: Argentina.

**Geographical distribution.**NEO.

**NEO**: Argentina.

***Apantelesorientalis* Szépligeti, 1913**

*Apantelesorientalis* Szépligeti, 1913.

**Type information.** Holotype male, HNHM (not examined but subsequent treatment of the species checked). Country of type locality: Tanzania.

**Geographical distribution.**AFR, OTL.

**AFR**: Tanzania; **OTL**: India.

**Notes.** Our species concept is based on Papp (2004).

***Apantelesoritias* Nixon, 1965**

*Apantelesoritias* Nixon, 1965.

**Type information.** Holotype female, NHMUK (examined). Country of type locality: India.

**Geographical distribution.**OTL.

**OTL**: China (ZJ), India.

***Apantelesoroetes* Nixon, 1965**

*Apantelesoroetes* Nixon, 1965.

**Type information.** Holotype female, NHMUK (examined). Country of type locality: South Africa.

**Geographical distribution.**AFR.

**AFR**: South Africa.

***Apantelesorphne* Nixon, 1965**

*Apantelesorphne* Nixon, 1965.

**Type information.** Holotype female, NHMUK (examined). Country of type locality: Fiji.

**Geographical distribution.**AUS.

**AUS**: Fiji.

***Apantelesortia* Nixon, 1965**

*Apantelesortia* Nixon, 1965.

**Type information.** Holotype female, NHMUK (examined). Country of type locality: Solomon Islands.

**Geographical distribution.**AUS.

**AUS**: Solomon Islands.

***Apantelesorus* Nixon, 1965**

*Apantelesorus* Nixon, 1965.

**Type information.** Holotype female, USNM (examined). Country of type locality: Philippines.

**Geographical distribution.**OTL.

**OTL**: Philippines.

***Apantelesoryzicola* Watanabe, 1967**

*Apantelesoryzicola* Watanabe, 1967.

**Type information.** Holotype female, KUEC (not examined but paratype examined). Country of type locality: Japan.

**Geographical distribution.**PAL.

**PAL**: Japan.

***Apantelesoscarchavezi* Fernandez-Triana, 2014**

*Apantelesoscarchavezi* Fernandez-Triana, 2014.

**Type information.** Holotype female, CNC (examined). Country of type locality: Costa Rica.

**Geographical distribution.**NEO.

**NEO**: Costa Rica.

***Apantelesoscus* Nixon, 1965**

*Apantelesoscus* Nixon, 1965.

**Type information.** Holotype female, NHMUK (examined). Country of type locality: South Africa.

**Geographical distribution.**AFR.

**AFR**: South Africa.

***Apantelesosvaldoespinozai* Fernandez-Triana, 2014**

*Apantelesosvaldoespinozai* Fernandez-Triana, 2014.

**Type information.** Holotype female, CNC (examined). Country of type locality: Costa Rica.

**Geographical distribution.**NEO.

**NEO**: Costa Rica.

***Apantelespablotranai* Fernandez-Triana, 2014**

*Apantelespablotranai* Fernandez-Triana, 2014.

**Type information.** Holotype female, CNC (examined). Country of type locality: Costa Rica.

**Geographical distribution.**NEO.

**NEO**: Costa Rica.

***Apantelespabloumanai* Fernandez-Triana, 2014**

*Apantelespabloumanai* Fernandez-Triana, 2014.

**Type information.** Holotype female, CNC (examined). Country of type locality: Costa Rica.

**Geographical distribution.**NEO.

**NEO**: Costa Rica.

***Apantelespablovasquezi* Fernandez-Triana, 2014**

*Apantelespablovasquezi* Fernandez-Triana, 2014.

**Type information.** Holotype female, CNC (examined). Country of type locality: Costa Rica.

**Geographical distribution.**NEO.

**NEO**: Costa Rica.

***Apantelespachycarinatus* Song & Chen, 2002**

*Apantelespachycarinatus* Song & Chen, 2002.

**Type information.** Holotype female, FAFU (not examined but original description checked). Country of type locality: China.

**Geographical distribution.**OTL.

**OTL**: China (FJ).

***Apantelespainei* Nixon, 1965**

*Apantelespainei* Nixon, 1965.

**Type information.** Holotype female, NHMUK (examined). Country of type locality: Papua New Guinea.

**Geographical distribution.**AUS.

**AUS**: Papua New Guinea.

***Apantelesparaglaope* Long, 2010**

*Apantelesparaglaope* Long, 2010.

**Type information.** Holotype female, IEBR (not examined but original description checked). Country of type locality: Vietnam.

**Geographical distribution.**OTL.

**OTL**: Vietnam.

***Apantelesparaguayensis* Brèthes, 1924**

*Apantelesparaguayensis* Brèthes, 1924.

**Type information.** Type unknown, MACN (not examined but original description checked). Country of type locality: Paraguay.

**Geographical distribution.**NEO.

**NEO**: Paraguay.

**Notes.** The original description is insufficient to conclude on the generic placement of this species.

***Apantelesparalus* Nixon, 1965**

*Apantelesparalus* Nixon, 1965.

**Type information.** Holotype female, NHMUK (examined). Country of type locality: South Africa.

**Geographical distribution.**AFR.

**AFR**: South Africa.

***Apantelesparanthrenidis* Muesebeck, 1921**

*Apantelesparanthrenidis* Muesebeck, 1921.

**Type information.** Holotype female, USNM (examined). Country of type locality: USA.

**Geographical distribution.**NEA, NEO.

**NEA**: USA (CA, DC, FL, MS, NY, OK, PA); **NEO**: Mexico.

***Apantelesparapholetesor* Liu & Chen, 2015**

*Apantelesparapholetesor* Liu & Chen, 2015.

**Type information.** Holotype female, ZJUH (not examined but original description checked). Country of type locality: China.

**Geographical distribution.**PAL.

**PAL**: China (LN).

***Apantelesparkeri* Muesebeck, 1954**

*Apantelesparkeri* Muesebeck, 1954.

**Type information.** Holotype female, USNM (examined). Country of type locality: Brazil.

**Geographical distribution.**NEO.

**NEO**: Brazil (MG).

***Apantelesparsodes* Nixon, 1965**

*Apantelesparsodes* Nixon, 1965.

**Type information.** Holotype female, NHMUK (examined). Country of type locality: South Africa.

**Geographical distribution.**AFR.

**AFR**: South Africa.

***Apantelesparvus* Liu & Chen, 2014**

*Apantelesparvus* Liu & Chen, 2014.

**Type information.** Holotype female, ZJUH (not examined but original description checked). Country of type locality: China.

**Geographical distribution.**OTL, PAL.

**OTL**: China (FJ, GD, ZJ); **PAL**: China (HA, SN).

***Apantelespashmina* Rousse, 2013**

*Apantelespashmina* Rousse, 2013.

**Type information.** Holotype female, MNHN (not examined but original description checked). Country of type locality: Réunion.

**Geographical distribution.**AFR.

**AFR**: Réunion.

***Apantelespastranai* Blanchard, 1960**

*Apantelespastranai* Blanchard, 1960.

**Type information.** Holotype female, MACN (not examined). Country of type locality: Argentina.

**Geographical distribution.**NEO.

**NEO**: Argentina.

***Apantelespatens* Nixon, 1965**

*Apantelespatens* Nixon, 1965.

**Type information.** Holotype female, NHMUK (examined). Country of type locality: South Africa.

**Geographical distribution.**AFR.

**AFR**: South Africa.

***Apantelespaulaixcamparijae* Fernandez-Triana, 2014**

*Apantelespaulaixcamparijae* Fernandez-Triana, 2014.

**Type information.** Holotype female, CNC (examined). Country of type locality: Costa Rica.

**Geographical distribution.**NEO.

**NEO**: Costa Rica.

***Apantelespeisonis* Fischer, 1965**

*Apantelespeisonis* Fischer, 1965.

*Apantelessubfirmus* Abdinbekova, 1969.

**Type information.** Holotype female, NHMW (not examined but subsequent treatment of the species checked). Country of type locality: Austria.

**Geographical distribution.**PAL.

**PAL**: Austria, Azerbaijan, Romania, Russia (NC).

**Notes.** Our species concept is based on Papp (1984a).

***Apantelespellucipterus* Song & Chen, 2001**

*Apantelespellucipterus* Song & Chen, 2001.

**Type information.** Holotype female, FAFU (not examined but original description checked). Country of type locality: China.

**Geographical distribution.**OTL.

**OTL**: China (FJ).

**Notes.** Our species concept is based on Song et al. (2001) and Chen and Song (2004).

***Apantelespentagonalis* Blanchard, 1963**

*Apantelespentagonalis* Blanchard, 1963.

**Type information.** Holotype male, MACN (not examined but original description checked). Country of type locality: Argentina.

**Geographical distribution.**NEO.

**NEO**: Argentina.

***Apantelespentagonius* de Saeger, 1944**

*Apantelespentagonius* de Saeger, 1944.

*Apanteleswilkinsoni* de Saeger, 1941 [homonym of *Apanteleswilkinsoni* Fahringer, 1936].

**Type information.** Syntypes female and male, RMCA (not examined but original description checked). Country of type locality: Democratic Republic of Congo.

**Geographical distribution.**AFR.

**AFR**: Democratic Republic of Congo.

***Apantelesperidoneus* Papp, 1974**

*Apantelesperidoneus* Papp, 1974.

**Type information.** Holotype female, HNHM (not examined but original description checked). Country of type locality: Hungary.

**Geographical distribution.**PAL.

**PAL**: Hungary.

***Apantelespersephone* Nixon, 1965**

*Apantelespersephone* Nixon, 1965.

**Type information.** Holotype female, NHMUK (examined). Country of type locality: Australia.

**Geographical distribution.**AUS.

**AUS**: Australia (WA).

***Apantelespertiades* Nixon, 1965**

*Apantelespertiades* Nixon, 1965.

**Type information.** Holotype female, NHMUK (examined). Country of type locality: Solomon Islands.

**Geographical distribution.**AUS.

**AUS**: Papua New Guinea, Solomon Islands.

***Apantelespetilicaudium* Chen, Song & Yang, 2002**

*Apantelespetilicaudium* Chen, Song & Yang, 2002.

**Type information.** Holotype female, FAFU (not examined but original description checked). Country of type locality: China.

**Geographical distribution.**OTL.

**OTL**: China (YN).

***Apantelespetronariosae* Fernandez-Triana, 2014**

*Apantelespetronariosae* Fernandez-Triana, 2014.

**Type information.** Holotype female, CNC (examined). Country of type locality: Costa Rica.

**Geographical distribution.**NEO.

**NEO**: Costa Rica.

***Apantelesphalis* Nixon, 1965**

*Apantelesphalis* Nixon, 1965.

**Type information.** Holotype female, USNM (examined). Country of type locality: Philippines.

**Geographical distribution.**OTL.

**OTL**: Philippines.

***Apantelesphtorimoeae* Risbec, 1951**

*Apantelesphtorimoeae* Risbec, 1951.

*Apantelesheliopae* Risbec, 1951.

**Type information.** Holotype female, depository unknown (not examined but original description checked). Country of type locality: Senegal.

**Geographical distribution.**AFR.

**AFR**: Senegal.

***Apantelesphycodis* Viereck, 1913**

*Apantelesphycodis* Viereck, 1913.

**Type information.** Holotype female, USNM (examined). Country of type locality: India.

**Geographical distribution.**OTL.

**OTL**: India, Vietnam.

***Apantelespiceotrichosus* Blanchard, 1947**

*Apantelespiceotrichosus* Blanchard, 1947.

**Type information.** Holotype female, MACN (not examined but original description checked). Country of type locality: Argentina.

**Geographical distribution.**NEO.

**NEO**: Argentina, Brazil (RS), Chile.

***Apantelespilosus* Telenga, 1955**

*Apantelespilosus* Telenga, 1955.

**Type information.** Lectotype female, ZIN (not examined but original description checked). Country of type locality: Turkmenistan.

**Geographical distribution.**PAL.

**PAL**: Kazakhstan, Turkmenistan, Uzbekistan.

**Notes.** Our species concept is based on the original description and Papp (1984a). We suspect that this species might belong to *Dolichogenidea* (or perhaps even *Pholetesor*), based on the description of the hypopygium, ovipositor sheaths, shapes of T1 and T2, and sculpture of anteromesoscutum; however, the hind wing vannal lobe is not described or illustrated in the sources we studied. Thus, we follow here Papp (1988) who kept the species in *Apanteles*.

***Apantelesplatyptiliophagus* Shenefelt, 1972**

*Apantelesplatyptiliophagus* Shenefelt, 1972.

*Apantelesplatyptiliae* Rao & Kurian, 1950 [homonym of *Apantelesplatyptiliae* Cameron, 1909].

**Type information.** Holotype male, NZSI (not examined but original description checked). Country of type locality: India.

**Geographical distribution.**OTL.

**OTL**: India.

***Apantelesplatyptiliovorus* Blanchard, 1965**

*Apantelesplatyptiliovorus* Blanchard, 1965.

**Type information.** Holotype female, MACN (not examined but original description checked). Country of type locality: Argentina.

**Geographical distribution.**NEO.

**NEO**: Argentina.

**Notes.** The original description suggests this species may belong to *Choeras* (based on the host species as well as the comparison the author made with *Choerasadjuntcus*). However, the details of the propodeum, T1–T3, and ovipositor cannot be interpreted unambiguously as being similar to *Choeras* (other genera could also be considered, including *Apanteles*). Thus, until specimens can be studied, we think is better to retain the species in the genus in which it was originally described.

***Apantelesplesius* Viereck, 1912**

*Apantelesplesius* Viereck, 1912.

**Type information.** Holotype female, USNM (examined). Country of type locality: USA.

**Geographical distribution.**NEA.

**NEA**: Canada (ON), USA (IL, MO, NJ, WI).

**Notes.** The holotype is currently missing the metasoma, three legs, and tips of antenna. We are following previous references to quote the holotype sex.

***Apantelespolychrosidis* Viereck, 1912**

*Apantelespolychrosidis* Viereck, 1912.

**Type information.** Holotype female, USNM (examined). Country of type locality: USA.

**Geographical distribution.**NEA.

**NEA**: Canada (BC, MB, NB, ON, QC), USA (AK, DC, FL, IL, KS, MI, MN, MO, NY, NC, OH, OR, PA, SD, WA, WI).

***Apantelespongamiae* Sumodan & Narendran, 1990**

*Apantelespongamiae* Sumodan & Narendran, 1990.

**Type information.** Holotype female, RMNH (not examined but subsequent treatment of the species checked). Country of type locality: India.

**Geographical distribution.**OTL.

**OTL**: India.

**Notes.** Our species concept is based on van Achterberg and Narendran (1997).

***Apantelesprinoptus* Papp, 1984**

*Apantelesprinoptus* Papp, 1984.

*Apantelesmetaclypealis* Tobias & Kotenko, 1986.

**Type information.** Holotype female, HNHM (not examined but original description checked). Country of type locality: Hungary.

**Geographical distribution.**PAL.

**PAL**: Germany, Hungary, Russia (S), Ukraine.

**Notes.** Our species concept is based on the original description and also comments from Kotenko (2006).

***Apantelesprocoxalis* Hedqvist, 1965**

*Apantelesprocoxalis* Hedqvist, 1965.

**Type information.** Holotype female, MZH (examined). Country of type locality: Cape Verde.

**Geographical distribution.**AFR.

**AFR**: Cape Verde.

**Notes.**Forshage et al. (2016) considered the type material to be lost; however, in 2017 it was found by the senior author of this paper in another section of the MZH collection.

***Apantelesprosopis* Risbec, 1951**

*Apantelesprosopis* Risbec, 1951.

**Type information.** Holotype female, depository unknown (not examined but original description checked). Country of type locality: Senegal.

**Geographical distribution.**AFR.

**AFR**: Senegal.

***Apantelesprusias* Nixon, 1965**

*Apantelesprusias* Nixon, 1965.

**Type information.** Holotype female, NHMUK (examined). Country of type locality: Sri Lanka.

**Geographical distribution.**OTL.

**OTL**: Sri Lanka.

**Notes.** The propodeum sculpture is somewhat atypical for *Apanteles*, as noted by Nixon (1965); nevertheless, we think at present this is still the best generic placement for the species.

***Apantelespsenes* Nixon, 1965**

*Apantelespsenes* Nixon, 1965.

**Type information.** Holotype female, NHMUK (examined). Country of type locality: Malaysia.

**Geographical distribution.**OTL.

**OTL**: Malaysia, Vietnam.

***Apantelespseudoglossae* Muesebeck, 1921**

*Apantelespseudoglossae* Muesebeck, 1921.

**Type information.** Holotype female, USNM (examined). Country of type locality: USA.

**Geographical distribution.**NEA.

**NEA**: Canada (QC), USA (IL, MD, MI, MN).

***Apantelespseudomacromphaliae* Havrylenko & Winterhalter, 1949**

*Apantelespseudomacromphaliae* Havrylenko & Winterhalter, 1949.

*Apantelesmacromphaliae* Blanchard, 1942 [*nomen nudum*].

**Type information.** Type and depository unknown (not examined). Country of type locality: Argentina.

**Geographical distribution.**NEO.

**NEO**: Argentina.

***Apantelespusaensis* Lal, 1942**

*Apantelespusaensis* Lal, 1942.

**Type information.** Holotype female, INPC (not examined but original description checked). Country of type locality: India.

**Geographical distribution.**OTL.

**OTL**: India.

***Apantelespycnos* Nixon, 1965**

*Apantelespycnos* Nixon, 1965.

**Type information.** Holotype female, AEIC (not examined but original description checked). Country of type locality: Philippines.

**Geographical distribution.**OTL.

**OTL**: Philippines.

***Apantelespyrodercetus* de Saeger, 1941**

*Apantelespyrodercetus* de Saeger, 1941.

**Type information.** Holotype female, RMCA (not examined but original description checked). Country of type locality: Democratic Republic of Congo.

**Geographical distribution.**AFR.

**AFR**: Democratic Republic of Congo.

***Apantelesquadratus* Anjum & Malik, 1978**

*Apantelesquadratus* Anjum & Malik, 1978.

**Type information.** Holotype female, UKZMP (not examined). Country of type locality: Pakistan.

**Geographical distribution.**PAL.

**PAL**: Pakistan.

**Notes.** The depository acronym (UKZMP) was selected based on the institution name: University of Karachi, Zoological Museum, Pakistan.

***Apantelesquadrifacies* Papp, 1984**

*Apantelesquadrifacies* Papp, 1984.

**Type information.** Holotype female, HNHM (not examined but subsequent treatment of the species checked). Country of type locality: Hungary.

**Geographical distribution.**PAL.

**PAL**: Hungary.

**Notes.** Our species concept is based on Papp (1984a).

***Apantelesquinquecarinis* Song & Chen, 2003**

*Apantelesquinquecarinis* Song & Chen, 2003.

**Type information.** Holotype female, FAFU (not examined but original description checked). Country of type locality: China.

**Geographical distribution.**OTL.

**OTL**: China (JX).

***Apantelesracilla* Nixon, 1965**

*Apantelesracilla* Nixon, 1965.

**Type information.** Holotype female, NHMUK (examined). Country of type locality: South Africa.

**Geographical distribution.**AFR.

**AFR**: South Africa.

***Apantelesraesus* Nixon, 1965**

*Apantelesraesus* Nixon, 1965.

**Type information.** Holotype female, NHMUK (examined). Country of type locality: South Africa.

**Geographical distribution.**AFR.

**AFR**: South Africa.

***Apantelesrandallgarciai* Fernandez-Triana, 2014**

*Apantelesrandallgarciai* Fernandez-Triana, 2014.

**Type information.** Holotype female, CNC (examined). Country of type locality: Costa Rica.

**Geographical distribution.**NEO.

**NEO**: Costa Rica.

***Apantelesrandallmartinezi* Fernandez-Triana, 2014**

*Apantelesrandallmartinezi* Fernandez-Triana, 2014.

**Type information.** Holotype female, CNC (examined). Country of type locality: Costa Rica.

**Geographical distribution.**NEO.

**NEO**: Costa Rica.

***Apantelesraulacevedoi* Fernandez-Triana, 2014**

*Apantelesraulacevedoi* Fernandez-Triana, 2014.

**Type information.** Holotype female, CNC (examined). Country of type locality: Costa Rica.

**Geographical distribution.**NEO.

**NEO**: Costa Rica.

***Apantelesraulsolorsanoi* Fernandez-Triana, 2014**

*Apantelesraulsolorsanoi* Fernandez-Triana, 2014.

**Type information.** Holotype female, CNC (examined). Country of type locality: Costa Rica.

**Geographical distribution.**NEO.

**NEO**: Costa Rica.

***Apantelesraviantenna* Chen & Song, 2004**

*Apantelesraviantenna* Chen & Song, 2004.

**Type information.** Holotype female, FAFU (not examined but original description checked). Country of type locality: China.

**Geographical distribution.**OTL, PAL.

**OTL**: China (FJ, HB); **PAL**: China (JL).

***Apantelesrhipheus* Nixon, 1965**

*Apantelesrhipheus* Nixon, 1965.

**Type information.** Holotype female, NHMUK (examined). Country of type locality: South Africa.

**Geographical distribution.**AFR.

**AFR**: South Africa.

***Apantelesrhomboidalis* (Ashmead, 1900)**

*Urogasterrhomboidalis* Ashmead, 1900.

**Type information.** Holotype female, NHMUK (examined). Country of type locality: Saint Vincent.

**Geographical distribution.**NEO.

**NEO**: Saint Vincent.

***Apantelesricardocaleroi* Fernandez-Triana, 2014**

*Apantelesricardocaleroi* Fernandez-Triana, 2014.

**Type information.** Holotype female, CNC (examined). Country of type locality: Costa Rica.

**Geographical distribution.**NEO.

**NEO**: Costa Rica.

***Apantelesricini* Bhatnagar, 1950**

*Apantelesricini* Bhatnagar, 1950.

**Type information.** Holotype male, INPC (not examined but original description checked). Country of type locality: India.

**Geographical distribution.**OTL.

**OTL**: India.

**Notes.** The year of publication of the Bhatnagar paper was until recently commonly cited as 1948 and/or 1950 (e.g., Chen and Song 2004, Yu et al. 2016), probably following Shenefelt (1972) who referred to this paper as “Bhatnagar (1948) 1950”. While the intended year for Volume X, Parts I & II of the Indian Journal of Entomology was 1948, the actual dates of publication were June 1950 (Part I) and October 1950 (Part II), as clearly shown on the cover page of the Volume, which we have checked. Because the dates of publication are the ones to be considered, and for the sake of clarity, we hereby revise the species year of description to 1950.

***Apantelesriograndensis* Brèthes, 1920**

*Apantelesriograndensis* Brèthes, 1920.

**Type information.** Holotype female, MACN (not examined). Country of type locality: Brazil.

**Geographical distribution.**NEO.

**NEO**: Brazil (RS).

**Notes.** The following information is based on Eduardo Shimbori (pers. comm.), and we use it as the most reliable source to conclude on the species distribution and type locality: a) there is no clear indication that *Apantelesriograndensis* Brèthes, 1920 is from Argentina, as far as we know, Taxapad (Yu et al. 2016) is the only source that states that, and it may be just an error based on the museum where the holotype is deposited; b) the title of the paper containing the original description is: “Insectos utiles y dañinos de Rio Grande do Sul y de la Plata” (Brèthes 1920), Rio Grande do Sul is certainly a Brazilian state (although there is a Rio Grande in Argentina, it is located in the Isla Grande de Tierra del Fuego, Patagonia, a place very far removed from La Plata); c) another paper (Ronna 1924) about insects of that same Brazilian state also mentions *A.riograndensis*; d) the catalogue on Hymenoptera Brasilenos (de Santis 1980) also cite *A.riograndensis* as from Rio Grande do Sul. Based on the information above we here consider *Apantelesriograndensis* Brèthes, 1920 as a Brazilian species, and not present in Argentina.

***Apantelesrisbeci* de Saeger, 1942**

*Apantelesrisbeci* de Saeger, 1942.

**Type information.** Holotype female?, RMCA (not examined but subsequent treatment of the species checked). Country of type locality: Senegal.

**Geographical distribution.**AFR.

**AFR**: Senegal.

**Notes.** We could not read the original description, but subsequent treatments of the species (de Saeger 1944, Risbec 1951) stated that the species was described based on the female sex (although it is not clear if one or more female specimens were studied). Thus, we have added a question mark after the holotype to denote the uncertainty.

***Apantelesrobertmontanoi* Fernandez-Triana, 2014**

*Apantelesrobertmontanoi* Fernandez-Triana, 2014.

**Type information.** Holotype female, CNC (examined). Country of type locality: Costa Rica.

**Geographical distribution.**NEO.

**NEO**: Costa Rica.

***Apantelesrobertoespinozai* Fernandez-Triana, 2014**

*Apantelesrobertoespinozai* Fernandez-Triana, 2014.

**Type information.** Holotype female, CNC (examined). Country of type locality: Costa Rica.

**Geographical distribution.**NEO.

**NEO**: Costa Rica.

***Apantelesrobertovargasi* Fernandez-Triana, 2014**

*Apantelesrobertovargasi* Fernandez-Triana, 2014.

**Type information.** Holotype female, CNC (examined). Country of type locality: Costa Rica.

**Geographical distribution.**NEO.

**NEO**: Costa Rica.

***Apantelesrobustus* Hedqvist, 1965**

*Apantelesrobustus* Hedqvist, 1965.

**Type information.** Holotype female, MZH (examined). Country of type locality: Cape Verde.

**Geographical distribution.**AFR.

**AFR**: Cape Verde.

**Notes.**Forshage et al. (2016) considered the type material to be lost; however, in 2017 it was found by the senior author of this paper in another section of the MZH collection.

***Apantelesrodrigogamezi* Fernandez-Triana, 2014**

*Apantelesrodrigogamezi* Fernandez-Triana, 2014.

**Type information.** Holotype female, CNC (examined). Country of type locality: Costa Rica.

**Geographical distribution.**NEO.

**NEO**: Costa Rica.

***Apantelesrogerblancoi* Fernandez-Triana, 2014**

*Apantelesrogerblancoi* Fernandez-Triana, 2014.

**Type information.** Holotype female, CNC (examined). Country of type locality: Costa Rica.

**Geographical distribution.**NEO.

**NEO**: Costa Rica.

***Apantelesrolandoramosi* Fernandez-Triana, 2014**

*Apantelesrolandoramosi* Fernandez-Triana, 2014.

**Type information.** Holotype female, CNC (examined). Country of type locality: Costa Rica.

**Geographical distribution.**NEO.

**NEO**: Costa Rica.

***Apantelesrolandovegai* Fernandez-Triana, 2014**

*Apantelesrolandovegai* Fernandez-Triana, 2014.

**Type information.** Holotype female, CNC (examined). Country of type locality: Costa Rica.

**Geographical distribution.**NEO.

**NEO**: Costa Rica.

***Apantelesromei* Rousse, 2013**

*Apantelesromei* Rousse, 2013.

**Type information.** Holotype female, MNHN (not examined but original description checked). Country of type locality: Réunion.

**Geographical distribution.**AFR.

**AFR**: Réunion.

***Apantelesronaldcastroi* Fernandez-Triana, 2014**

*Apantelesronaldcastroi* Fernandez-Triana, 2014.

**Type information.** Holotype female, CNC (examined). Country of type locality: Costa Rica.

**Geographical distribution.**NEO.

**NEO**: Costa Rica.

***Apantelesronaldgutierrezi* Fernandez-Triana, 2014**

*Apantelesronaldgutierrezi* Fernandez-Triana, 2014.

**Type information.** Holotype female, CNC (examined). Country of type locality: Costa Rica.

**Geographical distribution.**NEO.

**NEO**: Costa Rica.

***Apantelesronaldmurilloi* Fernandez-Triana, 2014**

*Apantelesronaldmurilloi* Fernandez-Triana, 2014.

**Type information.** Holotype female, CNC (examined). Country of type locality: Costa Rica.

**Geographical distribution.**NEO.

**NEO**: Costa Rica.

***Apantelesronaldnavarroi* Fernandez-Triana, 2014**

*Apantelesronaldnavarroi* Fernandez-Triana, 2014.

**Type information.** Holotype female, CNC (examined). Country of type locality: Costa Rica.

**Geographical distribution.**NEO.

**NEO**: Costa Rica.

***Apantelesronaldquirosi* Fernandez-Triana, 2014**

*Apantelesronaldquirosi* Fernandez-Triana, 2014.

**Type information.** Holotype female, CNC (examined). Country of type locality: Costa Rica.

**Geographical distribution.**NEO.

**NEO**: Costa Rica.

***Apantelesronaldzunigai* Fernandez-Triana, 2014**

*Apantelesronaldzunigai* Fernandez-Triana, 2014.

**Type information.** Holotype female, CNC (examined). Country of type locality: Costa Rica.

**Geographical distribution.**NEO.

**NEO**: Costa Rica.

***Apantelesrosaces* Nixon, 1965**

*Apantelesrosaces* Nixon, 1965.

**Type information.** Holotype female, NHMUK (examined). Country of type locality: South Africa.

**Geographical distribution.**AFR.

**AFR**: South Africa.

***Apantelesrosibelelizondoae* Fernandez-Triana, 2014**

*Apantelesrosibelelizondoae* Fernandez-Triana, 2014.

**Type information.** Holotype female, CNC (examined). Country of type locality: Costa Rica.

**Geographical distribution.**NEO.

**NEO**: Costa Rica.

***Apantelesrostermoragai* Fernandez-Triana, 2014**

*Apantelesrostermoragai* Fernandez-Triana, 2014.

**Type information.** Holotype female, CNC (examined). Country of type locality: Costa Rica.

**Geographical distribution.**NEO.

**NEO**: Costa Rica.

***Apantelesroughleyi* Fernandez-Triana, 2010**

*Apantelesroughleyi* Fernandez-Triana, 2010.

**Type information.** Holotype female, CNC (examined). Country of type locality: Canada.

**Geographical distribution.**NEA.

**NEA**: Canada (BC).

***Apantelesrufithorax* Hedqvist, 1965**

*Apantelesrufithorax* Hedqvist, 1965.

**Type information.** Holotype female, MZH (not examined but original description checked). Country of type locality: Cape Verde.

**Geographical distribution.**AFR.

**AFR**: Cape Verde.

**Notes.**Forshage et al. (2016) considered the type material to be lost; however, in 2017 it was found by the senior author in the MZH.

***Apantelesrugiceps* Wilkinson, 1934**

*Apantelesrugiceps* Wilkinson, 1934.

**Type information.** Holotype female, NHMUK (examined). Country of type locality: India.

**Geographical distribution.**OTL.

**OTL**: India.

***Apantelesruthfrancoae* Fernandez-Triana, 2014**

*Apantelesruthfrancoae* Fernandez-Triana, 2014.

**Type information.** Holotype female, CNC (examined). Country of type locality: Costa Rica.

**Geographical distribution.**NEO.

**NEO**: Costa Rica.

***Apantelesrutilans* Nixon, 1965**

*Apantelesrutilans* Nixon, 1965.

**Type information.** Holotype female, NHMUK (examined). Country of type locality: Kenya.

**Geographical distribution.**AFR, OTL.

**AFR**: Kenya; **OTL**: Vietnam.

***Apantelessaegeri* Risbec, 1951**

*Apantelessaegeri* Risbec, 1951.

*Apantelessaegeribambeyi* Risbec, 1951.

*Apantelessaegeriduplosenegalensis* Shenefelt, 1972 [new name for *Apantelessaegerisenegalensis* Risbec, 1951, a homonym of *Apantelessenegalensis* Risbec, 1951].

**Type information.** Syntypes female, depository unknown (not examined but original description checked). Country of type locality: Senegal.

**Geographical distribution.**AFR.

**AFR**: Senegal.

***Apantelessagax* Wilkinson, 1929**

*Apantelessagax* Wilkinson, 1929.

**Type information.** Holotype female, NHMUK (examined). Country of type locality: Tanzania.

**Geographical distribution.**AFR.

**AFR**: Cameroon, Democratic Republic of Congo, Ivory Coast, Nigeria, Senegal, Tanzania, Togo, Uganda.

***Apantelessalutifer* Wilkinson, 1931**

*Apantelessalutifer* Wilkinson, 1931.

**Type information.** Holotype female, NHMUK (examined). Country of type locality: Thailand.

**Geographical distribution.**OTL, PAL.

**OTL**: China (FJ, HB, YN), India, Myanmar, Thailand, Vietnam; **PAL**: Japan, Korea.

***Apantelessamedovi* Abdinbekova, 1969**

*Apantelessamedovi* Abdinbekova, 1969.

*Apanteleslencoranicus* Abdinbekova, 1969.

**Type information.** Holotype female, ZIN (not examined but subsequent treatment of the species checked). Country of type locality: Azerbaijan.

**Geographical distribution.**PAL.

**PAL**: Azerbaijan.

**Notes.** Our species concept is based on Papp (1980a) and Tobias (1986).

***Apantelessamoanus* Fullaway, 1940**

*Apantelessamoanus* Fullaway, 1940.

**Type information.** Holotype female, BPBM (not examined but subsequent treatment of the species checked). Country of type locality: American Samoa.

**Geographical distribution.**AUS.

**AUS**: American Samoa, Fiji.

**Notes.** Our species concept is based on Austin and Dangerfield (1992).

***Apantelessaravus* Nixon, 1965**

*Apantelessaravus* Nixon, 1965.

**Type information.** Holotype female, USNM (examined). Country of type locality: Philippines.

**Geographical distribution.**OTL.

**OTL**: Philippines.

***Apantelessauros* Nixon, 1965**

*Apantelessauros* Nixon, 1965.

**Type information.** Holotype female, NHMUK (examined). Country of type locality: India.

**Geographical distribution.**OTL.

**OTL**: India.

***Apantelesschneideri* Nixon, 1965**

*Apantelesschneideri* Nixon, 1965.

**Type information.** Holotype female, NHMUK (examined). Country of type locality: Indonesia.

**Geographical distribution.**OTL.

**OTL**: Indonesia.

***Apantelesschoutedeni* de Saeger, 1941**

*Apantelesschoutedeni* de Saeger, 1941.

**Type information.** Syntypes female and male, RMCA (not examined but original description checked). Country of type locality: Democratic Republic of Congo.

**Geographical distribution.**AFR.

**AFR**: Democratic Republic of Congo, Senegal.

***Apantelessergiocascantei* Fernandez-Triana, 2014**

*Apantelessergiocascantei* Fernandez-Triana, 2014.

**Type information.** Holotype female, CNC (examined). Country of type locality: Costa Rica.

**Geographical distribution.**NEO.

**NEO**: Costa Rica.

***Apantelessergioriosi* Fernandez-Triana, 2014**

*Apantelessergioriosi* Fernandez-Triana, 2014.

**Type information.** Holotype female, CNC (examined). Country of type locality: Costa Rica.

**Geographical distribution.**NEO.

**NEO**: Costa Rica.

***Apantelesseyrigi* Wilkinson, 1936**

*Apantelesseyrigi* Wilkinson, 1936.

**Type information.** Holotype female, MNHN (not examined but original description checked). Country of type locality: Madagascar.

**Geographical distribution.**AFR.

**AFR**: Madagascar.

***Apantelessigifredomarini* Fernandez-Triana, 2014**

*Apantelessigifredomarini* Fernandez-Triana, 2014.

**Type information.** Holotype female, CNC (examined). Country of type locality: Costa Rica.

**Geographical distribution.**NEO.

**NEO**: Costa Rica.

***Apantelessignificans* (Walker, 1860)**

*Microgastersignificans* Walker, 1860.

**Type information.** Holotype male, NHMUK (examined). Country of type locality: Sri Lanka.

**Geographical distribution.**OTL.

**OTL**: China (FJ), India, Pakistan, Philippines, Singapore, Sri Lanka, Vietnam.

***Apantelessingaporensis* Szépligeti, 1905**

*Apantelessingaporensis* Szépligeti, 1905.

**Type information.** Lectotype female, HNHM (not examined but subsequent treatment of the species checked). Country of type locality: Singapore.

**Geographical distribution.**OTL.

**OTL**: India, Singapore.

**Notes.** Our species concept is based on Wilkinson (1928b).

***Apantelessmerdis* Nixon, 1965**

*Apantelessmerdis* Nixon, 1965.

**Type information.** Holotype female, USNM (examined). Country of type locality: Philippines.

**Geographical distribution.**OTL.

**OTL**: Philippines.

***Apantelessodalis* (Haliday, 1834)**

*Microgastersodalis* Haliday, 1834.

*Microgastercarbonarius* Ratzeburg, 1848 [homonym of *Microgastercarbonarius* Wesmael, 1837].

*Microgasterater* Ratzeburg, 1852.

*Microgasterlugens* Ratzeburg, 1852.

*Apanteleslindbergi* Hedqvist, 1965.

**Type information.** Type lost (not examined but subsequent treatment of the species checked). Country of type locality: United Kingdom.

**Geographical distribution.**AFR, NEA, PAL, OTL.

**AFR**: Cape Verde; **NEA**: Canada (BC, NB, NL); **PAL**: Armenia, Azerbaijan, Bulgaria, China (SN), Czech Republic, France, Germany, Greece, Hungary, Ireland, Italy, Japan, Kazakhstan, Korea, Latvia, Lithuania, Moldova, Netherlands, Poland, Romania, Russia (KHA, KDA, MOS, PRI, SAK, SAM, SAR), Serbia, Slovakia, Sweden, Switzerland, Turkey, Ukraine, United Kingdom; **OTL**: China (GD, ZJ).

**Notes.** Our species concept is based on Fernandez-Triana and Huber (2010). The specimens of *Apantelessodalis* that have yielded DNA barcodes comprise two BINs, BOLD:AAM7223 (from Canada: BC, NL) and BOLD:AAN1859 (Canada: BC). Whether they represent two different species or not was mentioned by Fernandez-Triana et al. (2014b) but no further study has been conducted so in this paper all known specimens are kept as one species.

***Apantelessolox* Nixon, 1965**

*Apantelessolox* Nixon, 1965.

**Type information.** Holotype female, USNM (examined). Country of type locality: Singapore.

**Geographical distribution.**OTL.

**OTL**: Singapore.

***Apantelessosis* Nixon, 1965**

*Apantelessosis* Nixon, 1965.

**Type information.** Holotype female, NHMUK (examined). Country of type locality: South Africa.

**Geographical distribution.**AFR, OTL.

**AFR**: South Africa; **OTL**: Vietnam.

***Apantelessparsus* Liu & Chen, 2015**

*Apantelessparsus* Liu & Chen, 2015.

**Type information.** Holotype female, ZJUH (not examined but original description checked). Country of type locality: China.

**Geographical distribution.**OTL.

**OTL**: China (GD).

***Apantelesspicicula* Chen & Song, 2004**

*Apantelesspicicula* Chen & Song, 2004.

**Type information.** Holotype female, FAFU (not examined but original description checked). Country of type locality: China.

**Geographical distribution.**OTL.

**OTL**: China (FJ).

***Apantelesstagmatophorae* Gahan, 1919**

*Apantelesstagmatophorae* Gahan, 1919.

**Type information.** Holotype female, USNM (not examined but subsequent treatment of the species checked). Country of type locality: USA.

**Geographical distribution.**NEA.

**NEA**: USA (MD).

**Notes.** Our species concept is based on Muesebeck (1921) and Nixon (1972).

***Apantelesstarki* Mason, 1960**

*Apantelesstarki* Mason, 1960.

**Type information.** Holotype female, CNC (examined). Country of type locality: Canada.

**Geographical distribution.**NEA, OTL, PAL.

**NEA**: Canada (AB, BC), USA (ID, UT); **OTL**: China (HB); **PAL**: China (NX).

***Apantelesstegenodactylae* Cameron, 1909**

*Apantelesstegenodactylae* Cameron, 1909.

**Type information.** Holotype male, NHMUK (examined). Country of type locality: Sri Lanka.

**Geographical distribution.**OTL.

**OTL**: Sri Lanka.

**Notes.**Wilkinson (1928b: 137) considered that this species should probably be synonymized under *Apantelessubductus* Walker, 1860. The two species were described from a single male each, which were collected in the same island (Sri Lanka), and both are in relatively poor condition. However, after examining both specimens we do not think that is advisable to do what Wilkinson suggested. Although both specimens indeed share some resemblance morphologically, *subductus* lacks any biological information, the type locality is only stated as Ceylon (currently Sri Lanka), and the wings are partially shredded and glued together on the card, making it impossible to see any details on the hind wing. In contrast, *stegenodactylae* is slightly better preserved, has information about the type locality, and it also has preserved the wasp cocoon (and associated host information). Until more material from Sri Lanka is more comprehensively studied, we prefer to maintain both species as separate.

***Apantelesstennos* Nixon, 1965**

*Apantelesstennos* Nixon, 1965.

**Type information.** Holotype female, NHMUK (examined). Country of type locality: India.

**Geographical distribution.**OTL.

**OTL**: India.

***Apantelesstenomae* Muesebeck, 1958**

*Apantelesstenomae* Muesebeck, 1958.

**Type information.** Holotype female, USNM (examined). Country of type locality: Venezuela.

**Geographical distribution.**NEO.

**NEO**: Brazil (SP), Venezuela.

**Notes.** Distribution in Brazil from de Santis (1967b).

***Apantelesstictipes* Chen & Song, 2004**

*Apantelesstictipes* Chen & Song, 2004.

**Type information.** Holotype female, FAFU (not examined but original description checked). Country of type locality: China.

**Geographical distribution.**OTL.

**OTL**: China (FJ).

***Apantelesstriatopleurus* Hedqvist, 1965**

*Apantelesstriatopleurus* Hedqvist, 1965.

**Type information.** Holotype female, MZH (examined). Country of type locality: Cape Verde.

**Geographical distribution.**AFR.

**AFR**: Cape Verde.

***Apantelessubaltus* de Saeger, 1944**

*Apantelessubaltus* de Saeger, 1944.

**Type information.** Syntypes female and male, RMCA (not examined but original description checked). Country of type locality: Rwanda.

**Geographical distribution.**AFR.

**AFR**: Democratic Republic of Congo, Rwanda.

***Apantelessubandinus* Blanchard, 1947, restored combination**

*Apantelessubandinus* Blanchard, 1947.

**Type information.** Holotype female, MACN (not examined but original description checked). Country of type locality: Argentina.

**Geographical distribution.**AFR, AUS, NEO.

**AFR**: Réunion, South Africa; **AUS**: Australia (ACT, NSW, QLD, SA, TAS, VIC, WA), New Zealand; **NEO**: Argentina, Brazil (PR), Peru, Uruguay.

**Notes.** Under this species name there is likely a complex of species, some of them not even related. We have seen in the CNC two different species, one of them (from the USA, CA, and reared from the Gelechiidae moth *Phthorimaeaoperculella*) clearly belongs to *Apanteles*, and perhaps represents the true *Apantelessubandinus*. Another species (from Venezuela, reared from the same host) clearly belongs to *Dolichogenidea*, as it differs in the shape and fully setose vannal lobe of the hind wing, as well as the shape and sculpture of T1. Additionally, in BOLD, there are two BINs with the same name *Apantelessubandinus* but they are far apart from each other. BINBOLD:AAM4042 contains the Venezuelan specimens (as well as other specimens from Chile, also deposited in the CNC but with no associated host records); that BIN is close to species of *Dolichogenidea* and not *Apanteles*. The second BINBOLD:AAV2170 contains specimens from Colombia and New Zealand (with no host record available); that BIN is close to species of *Apanteles* and not *Dolichogenidea*. To complicate things further, Rousse and Gupta (2013: 534) considered the species as “unambiguously belonging to the genus *Glyptapanteles*” and thus transferred it to that genus; however, from their own figures in that paper (Rousse and Gupta 2013: fig. 15g-i) it is evident that the single female specimen they saw is not *Glyptapanteles* (e.g., see length and shape of the ovipositor sheaths and the hypopygium shown there in their fig. 15g). Solving the complexities of this species is beyond the scope of the present paper, but for now we transfer the species back to *Apanteles*, the best placement that it can be currently assigned to.

***Apantelessubcamilla* Long, 2007**

*Apantelessubcamilla* Long, 2007.

**Type information.** Holotype female, IEBR (not examined but original description checked). Country of type locality: Vietnam.

**Geographical distribution.**OTL.

**OTL**: Vietnam.

***Apantelessubcristatus* Blanchard, 1936**

*Apantelessubcristatus* Blanchard, 1936.

**Type information.** Holotype female, MACN (not examined but original description checked). Country of type locality: Argentina.

**Geographical distribution.**NEO.

**NEO**: Argentina, Chile, Uruguay.

***Apantelessubductus* (Walker, 1860)**

*Microgastersubductus* Walker, 1860.

**Type information.** Holotype male, NHMUK (examined). Country of type locality: Sri Lanka.

**Geographical distribution.**OTL.

**OTL**: Sri Lanka.

**Notes.** See comments above (under the species *Apantelesstegenodactylae* Cameron, 1909) for more comments on both species.

***Apantelessubrugosus* Granger, 1949**

*Apantelessubrugosus* Granger, 1949.

**Type information.** Syntypes female, MNHN (not examined but original description checked). Country of type locality: Madagascar.

**Geographical distribution.**AFR.

**AFR**: Madagascar.

***Apantelessulciscutis* (Cameron, 1905), new combination**

*Holcapantelessulciscutis* Cameron, 1905.

**Type information.** Type lost (not examined but original description checked). Country of type locality: Indonesia.

**Geographical distribution.**OTL.

**OTL**: Indonesia.

**Notes.** See comments at the beginning of *Apanteles* for more details on the decision to transfer this species to *Apanteles*.

***Apantelessyleptae* Ferrière, 1925**

*Apantelessyleptae* Ferrière, 1925.

**Type information.** Holotype female, MHNG (not examined but subsequent treatment of the species checked). Country of type locality: Sudan.

**Geographical distribution.**AFR.

**AFR**: Chad, Democratic Republic of Congo, Egypt, Kenya, Nigeria, Senegal, Sudan, Tanzania, Togo.

**Notes.** Our species concept is based on Nixon (1965). The species was recorded from India by Abraham et al. (1973); however, we consider that source as questionable (based on the previously known distribution of the species and different host species), pending further corroboration we prefer to exclude that record for the time being.

***Apantelessylvaticus* de Saeger, 1944**

*Apantelessylvaticus* de Saeger, 1944.

**Type information.** Holotype female, RMCA (not examined but original description checked). Country of type locality: Rwanda.

**Geographical distribution.**AFR.

**AFR**: Rwanda.

***Apantelessymithae* Bhatnagar, 1950**

*Apantelessymithae* Bhatnagar, 1950.

**Type information.** Holotype female, INPC (not examined but original description checked). Country of type locality: India.

**Geographical distribution.**OTL.

**OTL**: India.

**Notes.** The year of publication of the Bhatnagar paper was until recently commonly cited as 1948 and/or 1950 (e.g., Chen and Song 2004, Yu et al. 2016), probably following Shenefelt (1972) who referred to this paper as “Bhatnagar (1948) 1950”. While the intended year for Volume X, Parts I & II of the Indian Journal of Entomology was 1948, the actual dates of publication were June 1950 (Part I) and October 1950 (Part II), as clearly shown on the cover page of the Volume, which we have checked. Because the dates of publication are the ones to be considered, and for the sake of clarity, we hereby revise the species year of description to 1950.

***Apantelestachardiae* Cameron, 1913**

*Apantelestachardiae* Cameron, 1913.

**Type information.** Holotype female, NHMUK (examined). Country of type locality: India.

**Geographical distribution.**OTL.

**OTL**: China (HN), India.

***Apantelestaeniaticornis* Wilkinson, 1928**

*Apantelestaeniaticornis* Wilkinson, 1928.

**Type information.** Holotype female, NHMUK (examined). Country of type locality: Indonesia.

**Geographical distribution.**OTL.

**OTL**: Indonesia.

***Apantelestaiticus* (Holmgren, 1868)**

*Microgastertaiticus* Holmgren, 1868.

**Type information.** Holotype female, NHRS (not examined). Country of type locality: Society Islands.

**Geographical distribution.**AUS.

**AUS**: Society Islands.

***Apantelestalinum* Risbec, 1951**

*Apantelestalinum* Risbec, 1951.

**Type information.** Syntypes female and male, depository unknown (not examined but original description checked). Country of type locality: Senegal.

**Geographical distribution.**AFR.

**AFR**: Senegal.

***Apantelestapatapaoanus* Fullaway, 1946**

*Apantelestapatapaoanus* Fullaway, 1946.

*Apantelesbedelliae* Fullaway, 1941 [homonym of *Apantelesbedelliae* Viereck, 1911].

**Type information.** Holotype female, BPBM (not examined but subsequent treatment of the species checked). Country of type locality: Western Samoa.

**Geographical distribution.**AUS.

**AUS**: American Samoa, Western Samoa.

**Notes.** Our species concept is based on Austin and Dangerfield (1992).

***Apantelestaragamae* Viereck, 1912**

*Apantelestaragamae* Viereck, 1912.

*Apantelesplusiae* Viereck, 1913.

*Apanteleshomonae* Rohwer, 1922.

**Type information.** Holotype female, USNM (examined). Country of type locality: India.

**Geographical distribution.**AUS, OTL, PAL.

**AUS**: Papua New Guinea; **OTL**: China (FJ, GX, GZ, HB, HN, TW, ZJ), India, Indonesia, Sri Lanka, Thailand, Vietnam; **PAL**: Japan, Korea.

**Notes.** We examined the types of *Apantelesplusiaeplusiae* Viereck, 1913, and *Apanteleshomonae* Rohwer, 1922, both synonyms of *Apantelestaragamae*.

***Apantelestelon* Nixon, 1965**

*Apantelestelon* Nixon, 1965.

**Type information.** Holotype female, NHMUK (examined). Country of type locality: Pakistan.

**Geographical distribution.**PAL.

**PAL**: Pakistan.

***Apantelesthoracartus* Liu & Chen, 2015**

*Apantelesthoracartus* Liu & Chen, 2015.

**Type information.** Holotype female, ZJUH (not examined but original description checked). Country of type locality: China.

**Geographical distribution.**OTL.

**OTL**: China (GD).

***Apantelesthurberiae* Muesebeck, 1921**

*Apantelesthurberiae* Muesebeck, 1921.

**Type information.** Holotype female, USNM (examined). Country of type locality: USA.

**Geographical distribution.**NEA, NEO.

**NEA**: USA (AZ, TX); **NEO**: Colombia, Nicaragua, Trinidad & Tobago, Venezuela.

***Apantelestiapi* Risbec, 1952**

*Apantelestiapi* Risbec, 1952.

*Apanteleslongicornis* Risbec, 1951 [homonym of *Apanteleslongicornis* Provancher, 1886].

**Type information.** Holotype male, depository unknown (not examined but original description checked). Country of type locality: Senegal.

**Geographical distribution.**AFR.

**AFR**: Senegal.

**Notes.**Yu et al. (2016) listed the date of Risbec publication as 1951, but in fact that was for *Apanteleslongicornis*; the replacement name, *tiapi*, was proposed a year later (Risbec 1952: 701).

***Apantelestiboshartae* Fernandez-Triana, 2014**

*Apantelestiboshartae* Fernandez-Triana, 2014.

**Type information.** Holotype female, CNC (examined). Country of type locality: Costa Rica.

**Geographical distribution.**NEO.

**NEO**: Costa Rica.

***Apantelestigasis* Nixon, 1965**

*Apantelestigasis* Nixon, 1965.

**Type information.** Holotype female, NHMUK (examined). Country of type locality: Indonesia.

**Geographical distribution.**OTL.

**OTL**: Indonesia.

***Apantelestirathabae* Wilkinson, 1928**

*Apantelestirathabae* Wilkinson, 1928.

**Type information.** Holotype female, NHMUK (examined). Country of type locality: Malaysia.

**Geographical distribution.**AUS, OTL.

**AUS**: Fiji, Solomon Islands; **OTL**: Indonesia, Malaysia, Philippines, Vietnam.

***Apantelestownesi* Nixon, 1965**

*Apantelestownesi* Nixon, 1965.

**Type information.** Holotype female, AEIC (not examined but original description checked). Country of type locality: Philippines.

**Geographical distribution.**OTL.

**OTL**: Philippines.

***Apantelestranstergum* Liu & Chen, 2014**

*Apantelestranstergum* Liu & Chen, 2014.

**Type information.** Holotype female, ZJUH (not examined but original description checked). Country of type locality: China.

**Geographical distribution.**PAL.

**PAL**: China (HE).

***Apantelestriareus* Nixon, 1965**

*Apantelestriareus* Nixon, 1965.

**Type information.** Holotype female, NHMUK (examined). Country of type locality: South Africa.

**Geographical distribution.**AFR.

**AFR**: South Africa.

***Apantelestricoloripes* Granger, 1949**

*Apantelestricoloripes* Granger, 1949.

**Type information.** Syntypes female, MNHN (not examined but original description checked). Country of type locality: Madagascar.

**Geographical distribution.**AFR.

**AFR**: Madagascar.

***Apantelestrifasciatus* Muesebeck, 1946**

*Apantelestrifasciatus* Muesebeck, 1946.

**Type information.** Holotype female, USNM (examined). Country of type locality: Hawaiian Islands.

**Geographical distribution.**AUS.

**AUS**: Fiji, Hawaiian Islands.

***Apantelestrochanteratus* Szépligeti, 1911**

*Apantelestrochanteratus* Szépligeti, 1911.

**Type information.** Holotype female, ZMHB (not examined but subsequent treatment of the species checked). Country of type locality: Tanzania.

**Geographical distribution.**AFR.

**AFR**: Senegal, Tanzania.

**Notes.** Our species concept is based on Wilkinson (1932a).

***Apantelestulis* Nixon, 1965**

*Apantelestulis* Nixon, 1965.

**Type information.** Holotype female, USNM (examined). Country of type locality: Philippines.

**Geographical distribution.**OTL.

**OTL**: Philippines.

**Notes.** The holotype (only known specimen) is missing the metasoma, hind legs, and wings (one fore wing is glued to the card, and a pair of wings is loose in the unit tray). The species was described as “not very distinctive” (Nixon 1965: 78), and no illustration is available. The only remnants of this species are thus the holotype head (antenna missing apical flagellomeres) and the mesosoma.

***Apantelesuchidai* Watanabe, 1934**

*Apantelesuchidai* Watanabe, 1934.

**Type information.** Holotype female, EIHU (not examined but authoritatively identified specimens examined). Country of type locality: Japan.

**Geographical distribution.**PAL.

**PAL**: Japan.

**Notes.** In EIHU there is a specimen with a label that reads “?Type *Apantelesuchida* Watanabe”. However, that label is white, unlike all other labels of primary types in EIHU which are red, and the writing on it is not from Watanabe; thus, it was presumably added later by someone else, and perhaps indicates that the actual type is lost (or at least not clearly marked). In any case, the specimen is in very poor condition, as the only remains are the mesosoma (with a micropin through it, so some characters are not visible), two metacoxae, one metafemur, one fore wing, and the two hind wings.

***Apantelesunguifortis* Song & Chen, 2004**

*Apantelesunguifortis* Song & Chen, 2004.

**Type information.** Holotype female, FAFU (not examined but original description checked). Country of type locality: China.

**Geographical distribution.**OTL.

**OTL**: China (HB).

***Apantelesupis* Nixon, 1965**

*Apantelesupis* Nixon, 1965.

**Type information.** Holotype female, USNM (examined). Country of type locality: Philippines.

**Geographical distribution.**OTL.

**OTL**: Philippines.

***Apantelesuroxys* de Saeger, 1941**

*Apantelesuroxys* de Saeger, 1941.

**Type information.** Holotype female, RMCA (not examined but original description checked). Country of type locality: Democratic Republic of Congo.

**Geographical distribution.**AFR.

**AFR**: Democratic Republic of Congo, Rwanda, Senegal.

***Apantelesusipetes* Nixon, 1965**

*Apantelesusipetes* Nixon, 1965.

**Type information.** Holotype female, USNM (examined). Country of type locality: Malaysia.

**Geographical distribution.**OTL.

**OTL**: Malaysia.

**Notes.** The holotype is missing the metasoma, the fore wings and the hind legs. The hind wing vannal lobe is more or less straight with some setae visible, especially on the left hind wing where they seem to occupy most of the central part of the lobe, although the poor condition of the specimen makes it difficult to conclude. The presence of setae in the vannal lobe (also mentioned in the key to the species group in the original description (Nixon 1965: 39), although that paper only referred to few, sparse setae), would suggest that the species is better placed in *Dolichogenidea*. However, due to the poor condition of the type (only known specimen), we prefer to retain the species in the genus it was originally described.

***Apantelesussuriensis* Telenga, 1955**

*Apantelesussuriensis* Telenga, 1955.

**Type information.** Type and depository unknown (not examined but original description checked). Country of type locality: Russia.

**Geographical distribution.**PAL.

**PAL**: Russia (PRI).

***Apantelesvacillans* Nixon, 1965**

*Apantelesvacillans* Nixon, 1965.

**Type information.** Holotype female, USNM (not examined but original description checked). Country of type locality: Philippines.

**Geographical distribution.**OTL.

**OTL**: Philippines.

***Apantelesvala* Nixon, 1965**

*Apantelesvala* Nixon, 1965.

**Type information.** Holotype female, NHMUK (examined). Country of type locality: Australia.

**Geographical distribution.**AUS.

**AUS**: Australia (QLD).

***Apantelesvalvatus* de Saeger, 1944**

*Apantelesvalvatus* de Saeger, 1944.

*Apantelesvalvatusrwindicus* de Saeger, 1944.

**Type information.** Holotype female, RMCA (not examined but original description checked). Country of type locality: Rwanda.

**Geographical distribution.**AFR.

**AFR**: Democratic Republic of Congo, Rwanda.

**Notes.** This species could also be considered to belong to *Dolichogenidea*, but the original description does not detail the vannal lobe of the hind wing. Thus, until the specimens are examined, it is not possible to conclude, and we prefer to retain the species in *Apanteles* for the time being.

***Apantelesvalvulae* Rao & Kurian, 1951**

*Apantelesvalvulae* Rao & Kurian, 1951.

**Type information.** Holotype female, NZSI (not examined but original description checked). Country of type locality: India.

**Geographical distribution.**OTL.

**OTL**: India.

***Apantelesvannesabrenesae* Fernandez-Triana, 2014**

*Apantelesvannesabrenesae* Fernandez-Triana, 2014.

**Type information.** Holotype female, CNC (examined). Country of type locality: Costa Rica.

**Geographical distribution.**NEO.

**NEO**: Costa Rica.

***Apantelesverticalis* Song & Chen, 2004**

*Apantelesverticalis* Song & Chen, 2004.

**Type information.** Holotype female, FAFU (not examined but original description checked). Country of type locality: China.

**Geographical distribution.**OTL.

**OTL**: China (FJ, HB).

***Apantelesvictorbarrantesi* Fernandez-Triana, 2014**

*Apantelesvictorbarrantesi* Fernandez-Triana, 2014.

**Type information.** Holotype female, CNC (examined). Country of type locality: Costa Rica.

**Geographical distribution.**NEO.

**NEO**: Costa Rica.

***Apantelesvivax* de Saeger, 1944**

*Apantelesvivax* de Saeger, 1944.

**Type information.** Holotype female, RMCA (not examined but original description checked). Country of type locality: Democratic Republic of Congo.

**Geographical distribution.**AFR.

**AFR**: Democratic Republic of Congo.

***Apantelesvulgaris* (Ashmead, 1900)**

*Urogastervulgaris* Ashmead, 1900.

*Urogasterxanthopus* Ashmead, 1900.

**Type information.** Holotype male, NHMUK (examined). Country of type locality: Saint Vincent.

**Geographical distribution.**NEO.

**NEO**: Argentina, Brazil (SP), Grenada, Puerto Rico, Saint Vincent, Uruguay.

***Apanteleswadyobandoi* Fernandez-Triana, 2014**

*Apanteleswadyobandoi* Fernandez-Triana, 2014.

**Type information.** Holotype female, CNC (examined). Country of type locality: Costa Rica.

**Geographical distribution.**NEO.

**NEO**: Costa Rica.

***Apanteleswaldymedinai* Fernandez-Triana, 2014**

*Apanteleswaldymedinai* Fernandez-Triana, 2014.

**Type information.** Holotype female, CNC (examined). Country of type locality: Costa Rica.

**Geographical distribution.**NEO.

**NEO**: Costa Rica.

***Apanteleswanei* Risbec, 1951**

*Apanteleswanei* Risbec, 1951.

**Type information.** Syntypes female and male, depository unknown (not examined but original description checked). Country of type locality: Senegal.

**Geographical distribution.**AFR.

**AFR**: Senegal.

***Apantelesweitenweberi* (Amerling, 1862)**

*Microgasterweitenweberi* Amerling, 1862.

**Type information.** Type and depository unknown (not examined but original description checked). Country of type locality: Czech Republic.

**Geographical distribution.**PAL.

**PAL**: Czech Republic, Italy.

**Notes.** The original description, which is very brief and does not detail much, states that the species is close to *Sathonfalcatus* (Nees, 1834). Thus, it is likely that *weitenweberi* does not belong to *Apanteles*; however, without examining specimens we cannot conclude and prefer to retain it in the genus it was described.

***Apanteleswilbertharayai* Fernandez-Triana, 2014**

*Apanteleswilbertharayai* Fernandez-Triana, 2014.

**Type information.** Holotype female, CNC (examined). Country of type locality: Costa Rica.

**Geographical distribution.**NEO.

**NEO**: Costa Rica.

***Apanteleswilliamcamposi* Fernandez-Triana, 2014**

*Apanteleswilliamcamposi* Fernandez-Triana, 2014.

**Type information.** Holotype female, CNC (examined). Country of type locality: Costa Rica.

**Geographical distribution.**NEO.

**NEO**: Costa Rica.

***Apanteleswuyiensis* Song & Chen, 2002**

*Apanteleswuyiensis* Song & Chen, 2002.

**Type information.** Holotype female, FAFU (not examined but subsequent treatment of the species checked). Country of type locality: China.

**Geographical distribution.**OTL.

**OTL**: China (FJ).

**Notes.** Our species concept is based on Chen and Song (2004).

***Apantelesxanthostigma* (Haliday, 1834)**

*Microgasterxanthostigma* Haliday, 1834.

*Microgasterochrostigma* Wesmael, 1837.

*Apantelesxanthocarpus* Szépligeti, 1901.

**Type information.** Neotype female, NHMUK (examined). Country of type locality: United Kingdom.

**Geographical distribution.**AFR, NEA, PAL.

**AFR**: Uganda; **NEA**: Canada (BC, MB, NL, SK); **PAL**: Armenia, Austria, Azerbaijan, Belarus, Belgium, Bulgaria, Czech Republic, Faroe Islands, Finland, France, Germany, Hungary, Ireland, Italy, Japan, Kazakhstan, Latvia, Lithuania, Madeira Islands, Moldova, Mongolia, Netherlands, Poland, Portugal, Romania, Russia (ALT, ZAB, IRK, KAM, KHA, KIR, KDA, MOS, NGR, OMS, PRI, ROS, SAK, SPE, STA, YAR), Slovakia, Spain, Sweden, Switzerland, Tajikistan, Tunisia, Turkey, Ukraine, United Kingdom.

**Notes.** See Fernandez-Triana et al. (2014c) for a recent discussion of this species and its rather broad range of hosts.

***Apantelesxerophila* Risbec, 1951**

*Apantelesxerophila* Risbec, 1951.

**Type information.** Holotype female, depository unknown (not examined but original description checked). Country of type locality: Senegal.

**Geographical distribution.**AFR.

**AFR**: Senegal.

***Apantelesyeissonchavesi* Fernandez-Triana, 2014**

*Apantelesyeissonchavesi* Fernandez-Triana, 2014.

**Type information.** Holotype female, CNC (examined). Country of type locality: Costa Rica.

**Geographical distribution.**NEO.

**NEO**: Costa Rica.

***Apantelesyilbertalvaradoi* Fernandez-Triana, 2014**

*Apantelesyilbertalvaradoi* Fernandez-Triana, 2014.

**Type information.** Holotype female, CNC (examined). Country of type locality: Costa Rica.

**Geographical distribution.**NEO.

**NEO**: Costa Rica.

***Apantelesyolandarojasae* Fernandez-Triana, 2014**

*Apantelesyolandarojasae* Fernandez-Triana, 2014.

**Type information.** Holotype female, CNC (examined). Country of type locality: Costa Rica.

**Geographical distribution.**NEO.

**NEO**: Costa Rica.

***Apanteleszeneidabolanosae* Fernandez-Triana, 2014**

*Apanteleszeneidabolanosae* Fernandez-Triana, 2014.

**Type information.** Holotype female, CNC (examined). Country of type locality: Costa Rica.

**Geographical distribution.**NEO.

**NEO**: Costa Rica.

***Apanteleszhangi* Song & Chen, 2003**

*Apanteleszhangi* Song & Chen, 2003.

**Type information.** Holotype female, FAFU (not examined but original description checked). Country of type locality: China.

**Geographical distribution.**OTL.

**OTL**: China (FJ).

***Apanteleszizaniae* Muesebeck, 1957**

*Apanteleszizaniae* Muesebeck, 1957.

**Type information.** Holotype female, USNM (examined). Country of type locality: USA.

**Geographical distribution.**NEA.

**NEA**: USA (DE, DC).

***Apantelesznoikoi* Tobias, 1976**

*Apantelesznoikoi* Tobias, 1976.

**Type information.** Holotype female, ZIN (not examined but subsequent treatment of the species checked). Country of type locality: Azerbaijan.

**Geographical distribution.**PAL.

**PAL**: Armenia, Azerbaijan.

**Notes.** Our species concept is based on Papp (1984a).

#### Genus Austinicotesia Fernandez-Triana, 2018

***Austinicotesia*** Fernandez-Triana, 2018: 43. Gender: neuter. Type species: *Austinicotesiaindonesiensis* Fernandez-Triana & Boudreault, 2018, by original designation.

Two species were recently described from the Australasian region (Fernandez-Triana and Boudreault 2018), and in that same paper it was mentioned that one or two additional species had been seen in collections (but not described because the material was not sufficient). No host data are currently available for this genus. There are seven DNA-barcode compliant sequences of *Austinicotesia* in BOLD, representing one BIN.

***Austinicotesiaindonesiensis* Fernandez-Triana & Boudreault, 2018**

*Austinicotesiaindonesiensis* Fernandez-Triana & Boudreault, 2018.

**Type information.** Holotype female, RMNH (examined). Country of type locality: Indonesia.

**Geographical distribution.**OTL.

**OTL**: Indonesia.

***Austinicotesiapapuanus* Fernandez-Triana & Boudreault, 2018**

*Austinicotesiapapuanus* Fernandez-Triana & Boudreault, 2018.

**Type information.** Holotype female, MNHN (examined). Country of type locality: Papua New Guinea.

**Geographical distribution.**AUS.

**AUS**: Papua New Guinea.

#### Genus Austrocotesia Austin & Dangerfield, 1992

***Austrocotesia*** Austin & Dangerfield, 1992: 11. Gender: feminine. Type species: *Austrocotesiaexigua* Austin & Dangerfield, 1992, by original designation.

Five species are currently described from the Australasian and Neotropical regions, which can be separated using the key by Valerio and Whitfield (2005). However, it is possible that the Australasian and South American specimens actually represent different genera. In the original description of the genus, Austin and Dangerfield (1992: 11–12 and their figures 19 & 20) considered the lack of vein 2r-m in the hind wing as one of the main characters defining *Austrocotesia* (and indeed that is an important feature, as it is present in very few genera of Microgastrinae). However, the two Neotropical species defined by Valerio and Whitfield (2005, see their figures 1F & 2F) actually have such a vein. Further study of specimens from both regions will be needed to draw firm conclusions. In any case, it does not seem that *Austrocotesia* is very species rich, although a few additional species remain in collections. No host data are currently available. There are no DNA-barcode compliant sequences of the genus in BOLD, but there are two shorter sequences (minibarcodes) from paratypes of *A.croizati*.

***Austrocotesiacroizati* Valerio & Whitfield, 2005**

*Austrocotesiacroizati* Valerio & Whitfield, 2005.

**Type information.** Holotype female, IAVH (not examined but original description checked). Country of type locality: Colombia.

**Geographical distribution.**NEO.

**NEO**: Colombia, Ecuador.

**Notes.** This species is likely to represent a different genus (see a detailed explanation above, on comments about *Austrocotesia*). However, pending further study of the Neotropical fauna, we prefer to maintain it here for the time being.

***Austrocotesiadelicata* Austin & Dangerfield, 1992**

*Austrocotesiadelicata* Austin & Dangerfield, 1992.

**Type information.** Holotype female, CNC (examined). Country of type locality: Papua New Guinea.

**Geographical distribution.**AUS.

**AUS**: Australia (QLD), Papua New Guinea.

***Austrocotesiaexigua* Austin & Dangerfield, 1992**

*Austrocotesiaexigua* Austin & Dangerfield, 1992.

**Type information.** Holotype female, CNC (examined). Country of type locality: Papua New Guinea.

**Geographical distribution.**AUS.

**AUS**: Papua New Guinea.

***Austrocotesiaparadoxa* Austin & Dangerfield, 1992**

*Austrocotesiaparadoxa* Austin & Dangerfield, 1992.

**Type information.** Holotype female, AEIC (not examined but original description checked). Country of type locality: Papua New Guinea.

**Geographical distribution.**AUS.

**AUS**: Papua New Guinea.

***Austrocotesiarenei* Valerio & Whitfield, 2005**

*Austrocotesiarenei* Valerio & Whitfield, 2005.

**Type information.** Holotype female, CNC (examined). Country of type locality: Ecuador.

**Geographical distribution.**NEO.

**NEO**: Ecuador.

**Notes.** This species is likely to represent a different genus (see a detailed explanation above, on comments about *Austrocotesia*). However, pending further study of the Neotropical fauna, we prefer to maintain it here for the time being.

#### Genus Beyarslania Koçak & Kemal, 2009

***Beyarslania*** Koçak & Kemal, 2009: 14. Gender: feminine. Type species: *Apantelesinsolens* Wilkinson, 1930 by original designation (Mason 1981: 71).

*Xenogaster* Mason, 1981, preoccupied by *Xenogaster* Wasmann, 1891.

Only known from one species in the Afrotropical region (but see comments under that species about the possibility of a second, potentially new species). The genus was originally described by Mason (1981) as *Xenogaster*, but the name was later found to be a junior homonym of *Xenogaster* Wasmann, 1891 (Coleoptera) and thus subsequently changed to its current name (Koçak and Kemal 2009). No host data are currently available for this genus. There is one DNA-barcode compliant sequence in BOLD, its corresponding BIN characterizing the genus and species.

***Beyarslaniainsolens* (Wilkinson, 1930)**

*Apantelesinsolens* Wilkinson, 1930.

**Type information.** Neotype female, NHMUK (examined). Country of type locality: South Africa.

**Geographical distribution.**AFR.

**AFR**: Rwanda, South Africa, Yemen.

**Notes.**Fernandez-Triana and van Achterberg (2017) recorded *B.insolens* from Yemen, based on a single female specimen. In spite of the relatively large geographical separation (the record from Fernandez-Triana and van Achterberg (2017) expanded 2,000 km northwards the distribution of *Beyarslania* in Africa), those authors still considered it to belong to the same species previously recorded from South Africa (Wilkinson 1930b) and Rwanda (de Saeger 1944). After that 2017 paper was published, we have been able to study the holotype of *insolens* in the NHMUK, and two female and two male specimens deposited in the USNM (three from the type locality in South Africa, Cape Province, Mossel Bay; and one specimen from another, close locality, George, also in Cape Province). The five South African specimens seem different (although mostly in colouration) to the female from Yemen (deposited in the RMNH), which in turn is similar to another female specimen from Rwanda (deposited in the CNC). After comparing those seven specimens, we now think that the most northernly records in Africa (Rwanda and Yemen) may represent a different species, distinct from the South African one. However, until that is studied further (and the potential new species is properly described) we consider here only one species for Africa.

#### Genus Billmasonius Fernandez-Triana, 2018

***Billmasonius*** Fernandez-Triana, 2018: 28. Gender: neuter. Type species: *Billmasoniuscienci* Fernandez-Triana & Boudreault, 2018, by original designation.

The only known species was recently described from the Oriental region (Fernandez-Triana and Boudreault 2018). No host data are currently available for this genus. There is one DNA-barcode compliant sequences of *Billmasonius* in BOLD, representing one BIN (although that sequence has not been identified in BOLD as belonging to *Billmasonius*, see Fernandez-Triana and Boudreault 2018 for that).

***Billmasoniuscienci* Fernandez-Triana & Boudreault, 2018**

*Billmasoniuscienci* Fernandez-Triana & Boudreault, 2018.

**Type information.** Holotype female, QSBG (examined). Country of type locality: Thailand.

**Geographical distribution.**OTL.

**OTL**: Thailand.

#### Genus Buluka de Saeger, 1948

***Buluka*** de Saeger, 1948: 64. Gender: neuter (see below). Type species: *Bulukastraeleni* de Saeger, 1948, by original designation.

Known from eleven described species, mostly from the Oriental region, with a couple of taxa reaching the Afrotropical and Australasian regions. The revision by Austin (1989) is outdated. We have seen a few undescribed species in collections (CNC, RMNH) but the genus does not seem to be very diverse. Two Lepidoptera have been recorded as hosts, *Immathyriditis* Meyrick, 1906 (Immidae) and *Psimadaquadripennis* Walker, 1858 (Noctuidae) (Austin 1989, Gupta & Fernandez-Triana 2014). There is one DNA-barcode compliant sequence of *Buluka* in BOLD, from one undescribed species from Thailand.

Neither the etymology nor the gender of this genus was stated in the original description (de Saeger 1948) and, as far as we know, has never been discussed. *Buluka* was described based on a single species from the Belgian Congo, currently the Democratic Republic of the Congo, in central Africa. There is a word “Buluka” in the Xitsonga or Tsonga (a Bantu language spoken by the Tsonga people in central Africa), meaning “Explode. Burst. Blast” in Xitsonga (https://www.xitsonga.org/grammar/past?_=buluka); although it is not clear to us if that was the source de Saeger used for the genus name. Because of that, we here propose to consider the gender of this Microgastrinae genus to be neuter.

***Bulukaachterbergi* Austin, 1989**

*Bulukaachterbergi* Austin, 1989.

**Type information.** Holotype female, AEIC (not examined but original description checked). Country of type locality: Malaysia.

**Geographical distribution.**OTL.

**OTL**: Malaysia.

***Bulukacollessi* Austin & Dangerfield, 1992**

*Bulukacollessi* Austin & Dangerfield, 1992.

**Type information.** Holotype male, ANIC (not examined but original description checked). Country of type locality: Australia.

**Geographical distribution.**AUS.

**AUS**: Australia (QLD).

***Bulukahorni* Gupta, 2013**

*Bulukahorni* Gupta, 2013.

**Type information.** Holotype female, NBAIR (not examined but original description checked). Country of type locality: India.

**Geographical distribution.**OTL.

**OTL**: India.

***Bulukahuddlestoni* Austin, 1989**

*Bulukahuddlestoni* Austin, 1989.

**Type information.** Holotype female, NHMUK (examined). Country of type locality: Solomon Islands.

**Geographical distribution.**AUS.

**AUS**: Solomon Islands.

***Bulukanoyesi* Austin, 1989**

*Bulukanoyesi* Austin, 1989.

**Type information.** Holotype female, NHMUK (examined). Country of type locality: India.

**Geographical distribution.**OTL.

**OTL**: India.

***Bulukaorientalis* Chou, 1985**

*Bulukaorientalis* Chou, 1985.

**Type information.** Holotype female, TARI (not examined but subsequent treatment of the species checked). Country of type locality: China.

**Geographical distribution.**OTL.

**OTL**: China (TW).

**Notes.** Our species concept is based on Austin (1989).

***Bulukaquickei* Ranjith, 2015**

*Bulukaquickei* Ranjith, 2015.

**Type information.** Holotype female, DZCU (not examined but original description checked). Country of type locality: India.

**Geographical distribution.**OTL.

**OTL**: India.

***Bulukastraeleni* de Saeger, 1948**

*Bulukastraeleni* de Saeger, 1948.

**Type information.** Holotype female, RMCA (not examined but subsequent treatment of the species checked). Country of type locality: Democratic Republic of Congo.

**Geographical distribution.**AFR.

**AFR**: Cameroon, Democratic Republic of Congo, South Africa.

**Notes.** Our species concept is based on Austin (1989).

***Bulukataiwanensis* Austin, 1989**

*Bulukataiwanensis* Austin, 1989.

**Type information.** Holotype male, TARI (not examined but original description checked). Country of type locality: China.

**Geographical distribution.**OTL.

**OTL**: China (TW).

***Bulukatownesi* Austin, 1989**

*Bulukatownesi* Austin, 1989.

**Type information.** Holotype female, AEIC (not examined but original description checked). Country of type locality: Malaysia.

**Geographical distribution.**OTL.

**OTL**: India, Malaysia.

***Bulukavuquangensis* Long, 2015**

*Bulukavuquangensis* Long, 2015.

**Type information.** Holotype female, VNMN (not examined but original description checked). Country of type locality: Vietnam.

**Geographical distribution.**OTL.

**OTL**: Vietnam.

#### Genus Carlmuesebeckius Fernandez-Triana, 2018

***Carlmuesebeckius*** Fernandez-Triana, 2018: 28. Gender: neuter. Type species: *Carlmuesebeckiussmithsonian* Fernandez-Triana & Boudreault, 2018, by original designation.

The only known species was recently described from the Afrotropical region (Fernandez-Triana and Boudreault 2018). No host data are currently available for this genus. There are no DNA barcodes of *Carlmuesebeckius* in BOLD.

***Carlmuesebeckiussmithsonian* Fernandez-Triana & Boudreault, 2018**

*Carlmuesebeckiussmithsonian* Fernandez-Triana & Boudreault, 2018.

**Type information.** Holotype female, CNC (examined). Country of type locality: Madagascar.

**Geographical distribution.**AFR.

**AFR**: Madagascar.

**Notes.** In the original description the holotype depository was stated to be the CAS (Fernandez-Triana and Boudreault 2018: 54); however, that is a mistake as the specimen belongs to and is deposited in the CNC.

#### Genus Chaoa Luo & You, 2004

***Chaoa*** Luo & You, 2004: 339. Gender: neuter. Type species: *Chaoaflavipes* Luo, You & Xiao, 2004, by original designation.

One known species from the Oriental region, described from a single female from China. No host data are currently available for this genus. There are no DNA barcodes of *Chaoa* in BOLD. The only reference available is the original description, which included three line drawings showing the species habitus dorsally, and some details of the metasoma. We suspect the validity of this genus, as it seems to us to be just a species of *Glyptapanteles*. The appearance of mediotergite II (divided into three sections by a pair of longitudinal grooves delimiting a smooth, medial are) was considered by Luo et al. (2004) to be unique to *Chaoa* but in fact it is quite similar to that found in all or some species of several Microgastrinae genera (e.g., *Cotesia*, *Diolcogaster*, *Distatrix*, *Glyptapanteles*, *Nyereria*, *Rasivalva*). However, pending a reassessment of *Glyptapanteles* (which, as currently understood, seems to be polyphyletic), and without having examined the single known specimen of *Chaoa*, we refrain from changing its generic status for the time being.

***Chaoaflavipes* Luo, You & Xiao, 2004**

*Chaoaflavipes* Luo, You & Xiao, 2004.

**Type information.** Holotype female, HUNAU (not examined but original description checked). Country of type locality: China.

**Geographical distribution.**OTL.

**OTL**: China (FJ).

**Notes.** The information on the type depository was confirmed to us by Kees van Achterberg (pers. comm.), who examined the specimen.

#### Genus Choeras Mason, 1981

***Choeras*** Mason, 1981. Gender: masculine. Type species: *Apantelesconsimilis* Viereck, 1911, by original designation (Mason 1981: 76).

Currently with 80 described species, the genus is known from all biogeographical regions. No revision of *Choeras* has ever been produced, but most of the European/Palearctic species can be separated using the keys from van Achterberg (2002), Kotenko (2007a), Song et al. (2014), and Abdoli et al. (2019b). This is one of the most variable genera of Microgastrinae and, as currently understood, is probably polyphyletic. Even in the original description of the genus it was acknowledged that its limits might be changed in the future (Mason 1981: 77). The Holarctic species are relatively distinctive and uniform, but even in that region the species richness is much larger than documented at present. In tropical areas *Choeras* includes a mix of several unrelated groups, some of which might better be placed within separate genera. Depending on the generic concept that is adopted following future phylogenetic studies of Microgastrinae, *Choeras* may end up having several hundred species or just a few dozen. The host data are also very variable, with approximately 15 different families of Lepidoptera recorded so far, but records must be suspected in many cases. There are 820+ DNA-barcode compliant sequences of this genus in BOLD, representing 113 BINs, most of them from Canada and Thailand.

***Choerasachterbergi* Narendran, 1998**

*Choerasachterbergi* Narendran, 1998.

**Type information.** Holotype female, RMNH (not examined but original description checked). Country of type locality: India.

**Geographical distribution.**OTL.

**OTL**: India.

***Choerasadjunctus* (Nees, 1834)**

*Microgasteradjunctus* Nees, 1834.

**Type information.** Neotype female ZMHB (examined). Country of type locality: Germany.

**Geographical distribution.**OTL, PAL.

**OTL**: China (SN); **PAL**: Denmark, Germany, Netherlands, Sweden, United Kingdom.

**Notes.** Transferred from *Microgaster* to *Apanteles* by Reinhard (1881), then from *Apanteles* to *Dolichogenidea* by Papp (1988), and then from *Dolichogenidea* to *Choeras* by Shaw (2012b). The type from the original description was from Germany, we have not been able to determine the country of the neotype locality.

***Choerasafrotropicalis* Fernandez-Triana & van Achterberg, 2017**

*Choerasafrotropicalis* Fernandez-Triana & van Achterberg, 2017.

**Type information.** Holotype female, RMNH (examined). Country of type locality: Yemen.

**Geographical distribution.**AFR.

**AFR**: Yemen.

***Choerasalmus* (Tobias & Kotenko, 1984)**

*Apantelesalmus* Tobias & Kotenko, 1984.

**Type information.** Holotype female, ZIN (not examined but subsequent treatment of the species checked). Country of type locality: Russia.

**Geographical distribution.**PAL.

**PAL**: Russia (PRI).

**Notes.** Our species concept is based on Kotenko (2007a).

***Choerasangustus* Song & Chen, 2014**

*Choerasangustus* Song & Chen, 2014.

**Type information.** Holotype female, ZJUH (not examined but original description checked). Country of type locality: China.

**Geographical distribution.**OTL.

**OTL**: China (FJ, HI, HN, ZJ).

***Choerasaper* (Nixon, 1965), new combination**

*Apantelesaper* Nixon, 1965.

**Type information.** Holotype female, NHMUK (examined). Country of type locality: Australia.

**Geographical distribution.**AUS.

**AUS**: Australia (QLD).

**Notes.** Even in the original description it was recognized that this species was not at all typical of *Apanteles* (Nixon 1965: 62). The holotype (only known specimen of the species) has a propodeum with a coarse and irregular pattern of carinae and sculpture. A median, longitudinal carina is visible for most of the propodeum length; two shorter carinae near the nucha are also distinguished (suggesting the posterior half of an areola, although there are more carinae across the propodeum). The metanotum is strongly retracted from the scutellum, exposing the phragma. The ovipositor and sheaths are withdrawn into the hypopygium, but it is evident that the hypopygium is flexible (and supposedly pleated). The above characters strongly suggest the species does not belong in *Apanteles*. The best generic placement we can propose at present would be in *Choeras* (another candidate genus, *Sathon*, has an inflexible hypopygium), but study of additional specimens (if more are ever found) may change that in the future. The specimen was collected in an important area of wet subtropical rainforest habitats, with many endemic and/or significant species found there.

***Choerasapo* (Wilkinson, 1929)**

*Microgasterapo* Wilkinson, 1929.

**Type information.** Syntypes female and male, ZMHB (not examined but original description checked). Country of type locality: Philippines.

**Geographical distribution.**OTL.

**OTL**: Philippines.

***Choerasapollion* (Nixon, 1965), new combination**

*Hypomicrogasterapollion* Nixon, 1965.

**Type information.** Holotype female, NHMUK (examined). Country of type locality: South Africa.

**Geographical distribution.**AFR.

**AFR**: South Africa.

**Notes.** This species is clearly not an *Hypomicrogaster*, the most obvious characters to exclude it from that genus would be the large areolet in the fore wing, and the shapes of T1 and T2. The best generic placement at present is in *Choeras*.

***Choerasarene* (Nixon, 1973)**

*Apantelesarene* Nixon, 1973.

**Type information.** Holotype female, NHMUK (examined). Country of type locality: United Kingdom.

**Geographical distribution.**PAL.

**PAL**: Germany, Hungary, Ireland, Russia (ZAB, SAK), Spain, United Kingdom.

***Choerasavus* (Tobias & Kotenko, 1984)**

*Apantelesavus* Tobias & Kotenko, 1984.

**Type information.** Holotype female, ZIN (not examined but subsequent treatment of the species checked). Country of type locality: Russia.

**Geographical distribution.**PAL.

**PAL**: Russia (ZAB, IRK, MAG, PRI).

**Notes.** Our species concept is based on Kotenko (2007a).

***Choerasbatrachedrae* (Kotenko, 1992)**

*Apantelesbatrachedrae* Kotenko, 1992.

**Type information.** Holotype female, ZIN (not examined but subsequent treatment of the species checked). Country of type locality: Russia.

**Geographical distribution.**PAL.

**PAL**: Russia (ZAB, PRI).

**Notes.** Our species concept is based on Kotenko (2007a).

***Choerasbotydis* (Wilkinson, 1930)**

*Microgsterbotydis* Wilkinson, 1930.

**Type information.** Holotype female, NHMUK (examined). Country of type locality: Indonesia.

**Geographical distribution.**OTL, PAL.

**OTL**: Indonesia; **PAL**: Japan, Russia (SAK).

***Choerasbrevinervus* Song & Chen, 2014**

*Choerasbrevinervus* Song & Chen, 2014.

**Type information.** Holotype female, ZJUH (not examined but original description checked). Country of type locality: China.

**Geographical distribution.**OTL, PAL.

**OTL**: China (ZJ); **PAL**: China (GS, XJ).

***Choerasbushblitz* Fagan-Jeffries & Austin, 2019**

*Choerasbushblitz* Fagan-Jeffries & Austin, 2019.

**Type information.** Holotype female, TMAG (not examined but original description checked). Country of type locality: Australia.

**Geographical distribution.**AUS.

**AUS**: Australia (TAS).

***Choerascalacte* (Nixon, 1965)**

*Promicrogastercalacte* Nixon, 1965.

**Type information.** Holotype female, NHMUK (examined). Country of type locality: Australia.

**Geographical distribution.**AUS.

**AUS**: Australia (ACT, VIC).

**Notes.** This species is morphologically different from the typical *Choeras* that are usually found in temperate areas, specially the shape of the fore wing areolet. However, pending further study on “*Choerassensu lato*”, it is best kept as *Choeras* for the time being.

***Choerasceto* (Nixon, 1965)**

*Hypomicrogasterceto* Nixon, 1965.

**Type information.** Holotype female, NHMUK (examined). Country of type locality: Australia.

**Geographical distribution.**AUS.

**AUS**: Australia (ACT).

**Notes.** The ending of the species name has been variously treated as *cetus* (e.g., Austin and Dangerfield 1992, Fagan-Jeffries and Austin 2018), or *ceto* (e.g., Yu et al. 2016). Because the name is to be considered as a noun under ICZN Article 31.2.1, it must retain its original spelling and remain as *ceto*.

***Choerasciscaucasicus* (Tobias, 1971)**

*Apantelesciscaucasicus* Tobias, 1971.

**Type information.** Holotype female, ZIN (not examined but subsequent treatment of the species checked). Country of type locality: Russia.

**Geographical distribution.**PAL.

**PAL**: Lithuania, Russia (AD, PRI).

**Notes.** Our species concept is based on Kotenko (2007a).

***Choerascompressifemur* Chen & Song, 2004**

*Choerascompressifemur* Chen & Song, 2004.

**Type information.** Holotype female, FAFU (not examined but original description checked). Country of type locality: China.

**Geographical distribution.**OTL.

**OTL**: China (FJ, HB).

***Choerasconsimilis* (Viereck, 1911)**

*Apantelesconsimilis* Viereck, 1911.

*Microgasterlateralis* Provancher, 1886 [homonym of *Microgasterlateralis* Haliday, 1834].

**Type information.** Holotype female, USNM (examined). Country of type locality: USA.

**Geographical distribution.**NEA, OTL.

**NEA**: Canada (MB, NB, ON, QC), USA (AR, MI, NY, OH, PA, VA); **OTL**: China (HB).

**Notes.** This species was treated as *Dolichogenideaconsimilis* by Yu et al. (2012, 2016), following the decision by Chen and Song (2004) of transferring the species to that genus. However, after examining the holotype, numerous specimens deposited in the CNC, DNA barcodes, and other references (e.g., Fernandez-Triana 2010), all available evidence clearly indicates that this species belongs to *Choeras*.

***Choerasdaphne* (Nixon, 1965), new combination**

*Apantelesdaphne* Nixon, 1965.

**Type information.** Holotype female, USNM (examined). Country of type locality: Philippines.

**Geographical distribution.**OTL.

**OTL**: Philippines.

**Notes.** The holotype is missing the head but is otherwise in good condition. We place this species within *Choeras* based on the complete median carina on the propodeum and the shapes and sculpture of T1 and T2, although the fore wing venation is not that of a typical *Choeras*. It might be that this species represents a different genus, but with only one specimen available we prefer to maintain it in *Choeras*, the best placement at present.

***Choerasdissors* (Nixon, 1965)**

*Promicrogasterdissors* Nixon, 1965.

**Type information.** Holotype female, NHMUK (examined). Country of type locality: Australia.

**Geographical distribution.**AUS.

**AUS**: Australia (ACT).

***Choerasdorsalis* (Spinola, 1808)**

*Microgasterdorsalis* Spinola, 1808.

*Microgastercruciatus* Ratzeburg, 1844.

*Microgastersuffolciensis* Morley, 1902.

**Type information.** Type and depository unknown (not examined but authoritatively identified specimens examined). Country of type locality: Italy.

**Geographical distribution.**PAL.

**PAL**: Armenia, Austria, Azerbaijan, Belgium, Bulgaria, Canary Islands, Cyprus, Egypt, France, Georgia, Germany, Greece, Hungary, Iran, Israel, Italy, Jordan, Lithuania, Madeira Islands, Malta, Moldova, Poland, Romania, Russia (DA), Slovakia, Spain, Switzerland, Tunisia, Turkey, Ukraine, United Kingdom, Uzbekistan.

**Notes.**Shenefelt (1973: 703) summarized very well the status of the species when he wrote: “Much confusion exists regarding the species to which literature refers. Conflicting statements occur and some authors have simply chosen to ignore certain of the older names. It appears best for the present merely to list the references as found and hope that intensive study and corrections may be made later to rectify erroneous citations”. Unfortunately, not much progress has been achieved since, and here we restrict ourselves to citing the information as recorded in Yu et al. (2016). We examined the type of *Microgastersuffolciensis* Morley (which is missing the metasoma and the hind legs except for the metacoxae); we found that the pterostigma is notably wide (i.e., pterostigma height ca. two thirds its length), a character that may be unique among other Holarctic described species of *Choeras*.

***Choerasepaphus* (Nixon, 1965)**

*Hypomicrogasterepaphus* Nixon, 1965.

**Type information.** Holotype female, NHMUK (examined). Country of type locality: Australia.

**Geographical distribution.**AUS.

**AUS**: Australia (ACT, QLD).

***Choerasflavicorpus* Song & Chen, 2014**

*Choerasflavicorpus* Song & Chen, 2014.

**Type information.** Holotype female, ZJUH (not examined but original description checked). Country of type locality: China.

**Geographical distribution.**OTL.

**OTL**: China (HI, YN).

***Choerasfomes* (Nixon, 1965), new combination**

*Hypomicrogasterfomes* Nixon, 1965.

**Type information.** Holotype female, NHMUK (examined). Country of type locality: South Africa.

**Geographical distribution.**AFR.

**AFR**: South Africa.

**Notes.** This species is clearly not an *Hypomicrogaster*, the most obvious characters to exclude it from that genus would be the large areolet in the fore wing, and the shapes of T1 and T2. The best generic placement at present is in *Choeras*.

***Choerasformosus* Abdoli & Fernandez-Triana, 2019**

*Choerasformosus* Abdoli & Fernandez-Triana, 2019.

**Type information.** Holotype female, TMUC (examined). Country of type locality: Iran.

**Geographical distribution.**PAL.

**PAL**: Iran.

***Choerasfujianensis* Song & Chen, 2014**

*Choerasfujianensis* Song & Chen, 2014.

**Type information.** Holotype female, ZJUH (not examined but original description checked). Country of type locality: China.

**Geographical distribution.**OTL.

**OTL**: China (FJ).

***Choerasfulviventris* Fernandez-Triana & Abdoli, 2019**

*Choerasfulviventris* Fernandez-Triana & Abdoli, 2019.

**Type information.** Holotype female, TMUC (examined). Country of type locality: Iran.

**Geographical distribution.**PAL.

**PAL**: Iran.

***Choerasgerontius* (Nixon, 1965), new combination**

*Hypomicrogastergerontius* Nixon, 1965.

**Type information.** Holotype female, NHMUK (examined). Country of type locality: South Africa.

**Geographical distribution.**AFR.

**AFR**: South Africa.

**Notes.** This species is clearly not an *Hypomicrogaster*, the most obvious characters to exclude it from that genus would be the large areolet in the fore wing, and the shapes of T1 and T2. The best generic placement at present is in *Choeras*.

***Choerasgielisi* van Achterberg, 2002**

*Choerasgielisi* van Achterberg, 2002.

**Type information.** Holotype female, RMNH (not examined but original description checked). Country of type locality: Netherlands.

**Geographical distribution.**PAL.

**PAL**: France, Netherlands.

***Choerasgnarus* (Tobias & Kotenko, 1984)**

*Apantelesgnarus* Tobias & Kotenko, 1984.

**Type information.** Holotype female, SIZK (not examined but subsequent treatment of the species checked). Country of type locality: Ukraine.

**Geographical distribution.**PAL.

**PAL**: Belarus, Russia (C, NC), Ukraine.

**Notes.** Our species concept is based on van Achterberg (2002).

***Choerasgrammatitergitus* Song & Chen, 2014**

*Choerasgrammatitergitus* Song & Chen, 2014.

**Type information.** Holotype female, ZJUH (not examined but original description checked). Country of type locality: China.

**Geographical distribution.**OTL, PAL.

**OTL**: China (SN); **PAL**: China (NX).

***Choerashelespas* Walker, 1996**

*Choerashelespas* Walker, 1996.

**Type information.** Holotype female, LUNZ (not examined but original description checked). Country of type locality: New Zealand.

**Geographical distribution.**AUS.

**AUS**: New Zealand.

***Choerashelle* (Nixon, 1965), new combination**

*Hypomicrogasterhelle* Nixon, 1965.

**Type information.** Holotype female, NHMUK (examined). Country of type locality: Sierra Leone.

**Geographical distribution.**AFR.

**AFR**: Nigeria, Sierra Leone.

**Notes.** This species is clearly not an *Hypomicrogaster*, the most obvious characters to exclude it from that genus would be the large areolet in the fore wing, and the shapes of T1 and T2. The best generic placement at present is in *Choeras*.

***Choerasinfirmicarinatus* Song & Chen, 2014**

*Choerasinfirmicarinatus* Song & Chen, 2014.

**Type information.** Holotype female, ZJUH (not examined but original description checked). Country of type locality: China.

**Geographical distribution.**OTL.

**OTL**: China (FJ, GD, ZJ).

***Choerasinsignis* (Muesebeck, 1938)**

*Apantelesinsignis* Muesebeck, 1938.

**Type information.** Holotype female, USNM (not examined but original description checked). Country of type locality: USA.

**Geographical distribution.**NEA.

**NEA**: Canada (BC), USA (CA).

***Choerasirates* (Nixon, 1965), new combination**

*Hypomicrogasterirates* Nixon, 1965.

**Type information.** Holotype female, NHMUK (examined). Country of type locality: India.

**Geographical distribution.**OTL.

**OTL**: India.

**Notes.** This species is clearly not an *Hypomicrogaster*, the most obvious characters to exclude it from that genus would be the large areolet in the fore wing, and the shapes of T1 and T2. The best generic placement at the time is in *Choeras* but it must be noted that this is one of the Oriental species of *Choeras* that probably will need a different, perhaps new, genus. Because that is beyond the scope of this paper, for the time being the species is transferred to *Choeras*.

***Choeraskoalascatocola* Fagan-Jeffries & Austin, 2017**

*Choeraskoalascatocola* Fagan-Jeffries & Austin, 2017.

**Type information.** Holotype female, QM (not examined but original description checked). Country of type locality: Australia.

**Geographical distribution.**AUS.

**AUS**: Australia (QLD).

***Choeraslibanius* (Nixon, 1965), new combination**

*Hypomicrogasterlibanius* Nixon, 1965.

**Type information.** Holotype female, USNM (examined). Country of type locality: Philippines.

**Geographical distribution.**OTL.

**OTL**: Philippines.

**Notes.** This species was synonymized under *Microgasterpsarae* Wilkinson, 1927 (now in *Choeras*) by Valerio and Whitfield (2015). Here we raise *libanius* from synonymy and consider it to be a valid species, different from *psarae* (see below under *psarae* for more details on morphological differences between them). Also, we transfer *libanius* to *Choeras*, based on its pleated hypopygium, relatively long ovipositor sheaths, and T1 without a longitudinal sulcus. This species clearly belongs to a group of Oriental *Choeras* such as *apo*, *nephta*, *psarae* and many undescribed species we have seen in collections, with very large body size; they may be transferred to a different genus in the future (see discussion below, under *C.nephta*).

***Choeraslongiterebrus* (Rao & Chalikwar, 1976), new combination**

*Protomicroplitislongiterebrus* Rao & Chalikwar, 1976.

**Type information.** Holotype female, BAMU (not examined but original description checked). Country of type locality: India.

**Geographical distribution.**OTL.

**OTL**: India.

**Notes.** This species was transferred to *Diolcogaster* by Zeng et al. (2009). Based on the original description and extensive illustrations there, this species is clearly not *Diolcogaster*, based on the following: T1 excavate anteriorly but without median, longitudinal sulcus; ovipositor longer than metatibia; ovipositor sheaths long and moderately setose, but without thick setae apically; scutellar disc lacking a posteromedian band of rugosity; other characters (size of fore wing areolet, mostly smooth propodeum, T1 and T2) are not commonly present in *Diolcogaster*. The best generic placement at present would be in *Choeras*, although future study of the Oriental species currently placed in that genus may change its status. Following Article 31.2.1 of the ICZN the name is to be considered as a noun phrase in apposition, and the original spelling *longiterebrus* must be retained.

***Choeraslongitergitus* Song & Chen, 2014**

*Choeraslongitergitus* Song & Chen, 2014.

**Type information.** Holotype female, ZJUH (not examined but original description checked). Country of type locality: China.

**Geographical distribution.**OTL.

**OTL**: China (FJ, GZ, HI, HN, GD, ZJ).

***Choeraslongus* Song & Chen, 2014**

*Choeraslongus* Song & Chen, 2014.

**Type information.** Holotype female, ZJUH (not examined but original description checked). Country of type locality: China.

**Geographical distribution.**OTL.

**OTL**: China (FJ, ZJ).

***Choerasloretta* (Nixon, 1965), new combination**

*Hypomicrogasterloretta* Nixon, 1965.

**Type information.** Holotype female, NHMUK (examined). Country of type locality: South Africa.

**Geographical distribution.**AFR.

**AFR**: South Africa.

**Notes.** This species is clearly not an *Hypomicrogaster*, the most obvious characters to exclude it from that genus would be the large areolet in the fore wing, and the shapes of T1 and T2. The best generic placement at present is in *Choeras*.

***Choerasmorialta* Fagan-Jeffries & Austin, 2017**

*Choerasmorialta* Fagan-Jeffries & Austin, 2017.

**Type information.** Holotype female, SAMA (not examined but original description checked). Country of type locality: Australia.

**Geographical distribution.**AUS.

**AUS**: Australia.

***Choerasnephta* (Nixon, 1965)**

*Hypomicrogasternephta* Nixon, 1965.

**Type information.** Holotype female, USNM (examined). Country of type locality: Philippines.

**Geographical distribution.**OTL.

**OTL**: Philippines.

**Notes.** A very large species (body length 5.6 mm, fore wing length 6.2 mm). It belongs to a group of large *Choeras* from the Oriental region (e.g., *apo*, *libanius*, *psarae*, and many undescribed species we have seen in collection) which in the future may be better placed in a different genus. For the time being they are all kept in *Choeras*, until a better phylogenetic understanding of this group is gained.

***Choeraspapua* (Wilkinson, 1936)**

*Microgasterpapua* Wilkinson, 1936.

**Type information.** Holotype female, NHMUK (examined). Country of type locality: Papua New Guinea.

**Geographical distribution.**AUS.

**AUS**: Indonesia, Papua New Guinea.

***Choerasparabolus* Kotenko, 2007**

*Choerasparabolus* Kotenko, 2007.

**Type information.** Holotype female, SIZK (not examined but original description checked). Country of type locality: Russia.

**Geographical distribution.**PAL.

**PAL**: Russia (PRI).

***Choerasparasitellae* (Bouché, 1834)**

*Microgasterparasitellae* Bouché, 1834.

*Microgasterflavilabris* Ratzeburg, 1844.

*Microgasterrufilabris* Ratzeburg, 1844.

*Apanteleslictorius* Reinhard, 1880.

*Apantelespolypori* Gautier & Bonnamour, 1930.

**Type information.** Holotype female, ZMHB (not examined but subsequent treatment of the species checked). Country of type locality: Germany.

**Geographical distribution.**NEA, PAL.

**NEA**: Canada (Ontario); **PAL**: Austria, Belgium, Czech Republic, Finland, France, Georgia, Germany, Hungary, Iran, Israel, Italy, Korea, Latvia, Moldova, Netherlands, Poland, Romania, Russia (DA, PRI, SAK, SPE, TOM, YAR), Serbia, Slovakia, Spain, Sweden, Switzerland, Turkey, Ukraine, United Kingdom, Uzbekistan.

**Notes.** Our species concept is based on van Achterberg (2002) and Fernandez-Triana et al. (2016a).

***Choerasparasonium* Kotenko, 2007**

*Choerasparasonium* Kotenko, 2007.

**Type information.** Holotype female, ZIN (not examined but original description checked). Country of type locality: Russia.

**Geographical distribution.**PAL.

**PAL**: Russia (KAM).

***Choerasparviocellus* Song & Chen, 2014**

*Choerasparviocellus* Song & Chen, 2014.

**Type information.** Holotype female, ZJUH (not examined but original description checked). Country of type locality: China.

**Geographical distribution.**OTL, PAL.

**OTL**: China (GZ, HI, SN, TW, YN); **PAL**: China (NM, NX).

***Choerasparvoculus* Fagan-Jeffries & Austin, 2019**

*Choerasparvoculus* Fagan-Jeffries & Austin, 2019.

**Type information.** Holotype female, TMAG (not examined but original description checked). Country of type locality: Australia.

**Geographical distribution.**AUS.

**AUS**: Australia (TAS).

***Choeraspsarae* (Wilkinson, 1927)**

*Microgasterpsarae* Wilkinson, 1927.

**Type information.** Holotype female, NHMUK (examined). Country of type locality: Malaysia.

**Geographical distribution.**OTL, PAL.

**OTL**: China (TW), India, Malaysia, Sri Lanka, Thailand; **PAL**: Korea.

**Notes.** We consider the record of *psarae* from Korea (Papp 1987: 439), which is also reported in Yu et al. (2012, 2016), as very suspicious, although we do not remove it from the present checklist. The short description of the single Korean specimen (as provided by Papp 1987) is different from the *psarae* type we have examined, and the known distribution in Korea (near Pyongyang) is fully Palearctic, whereas all other known records of this species are in the Oriental region. Also, a literature record from Taiwan (Papp 1987: 439) had been overlooked by recent authors but it is accepted here. Mason (1981) transferred *Microgasterpsarae* to *Choeras*, a decision followed by several other authors (see Yu et al. 2016 for list of references). Then Valerio and Whitfield (2015) transferred the species to *Diolcogaster* and synonymized *Hypomicrogasterlibanius* Nixon, 1965 under *psarae*. After examining the types and original description, we consider both decisions from Valerio and Whitfield (2015) incorrect. Here we transfer *psarae* back to *Choeras* based on its pleated hypopygium, relatively long ovipositor sheaths (around two thirds metatibia length), T1 smooth and without longitudinal sulcus, T2 smooth, transversely subtriangular and without median field, and propodeum mostly smooth, with a strong, median longitudinal carina with several small carinae radiating from it. This species clearly belongs to a group of Oriental *Choeras* such as *apo*, *libanius*, *nephta*, and many undescribed species we have seen in collections, with very large body size; they may be transferred to a different genus in the future (see discussion above, under *C.nephta*). We also remove *H.libanius* from synonym with *psarae* and consider it as a valid species, as the differences between them are significant: *psarae* has a yellow metasoma (except for T2 brown on posterior half and T3 with small brown), all coxae yellow (except for brown spot on posterior third ventrally), T1 comparatively narrower at posterior margin (as compared to anterior margin), and smaller body size (ca. 4.5 mm); whereas *libanius* has the metasoma mostly dark brown, all coxae dark brown to black, T1 comparatively wider at the posterior margin, and much larger body size (ca. 6.2 mm) (see Nixon 1965 for more differences).

***Choerasqazviniensis* Fernandez-Triana & Talebi, 2019**

*Choerasqazviniensis* Fernandez-Triana & Talebi, 2019.

**Type information.** Holotype female, TMUC (examined). Country of type locality: Iran.

**Geographical distribution.**PAL.

**PAL**: Iran.

***Choerasrecusans* (Walker, 1860), new combination**

*Microgasterrecusans* Walker, 1860.

**Type information.** Holotype female, NHMUK (examined). Country of type locality: Sri Lanka.

**Geographical distribution.**OTL.

**OTL**: India, Sri Lanka.

**Notes.** This species was transferred to *Apanteles* by Wilkinson (1927: 178) and treated under that genus by most authors (but see Yu et al. 2016 for a different treatment). We have examined the holotype and it has a strong median, longitudinal carina, which precludes the species from belonging in *Apanteles* or related genera. Based on the mesosoma sculpture, T1 wide and with a shallow excavation anteriorly and relatively long ovipositor sheaths (as long as the metatibia), this species is better placed in *Choeras*.

***Choerasruficornis* (Nees, 1834)**

*Microgasterruficornis* Nees, 1834.

*Apanteleshedymeles* Nixon, 1973.

**Type information.** Neotype female, RBINS (not examined but authoritatively identified specimens examined). Country of type locality: Germany.

**Geographical distribution.**PAL.

**PAL**: Belgium, Finland, France, Georgia, Germany, Hungary, Italy, Latvia, Netherlands, Norway, Poland, Romania, Russia (AMU, PRI, SAK), Slovakia, Sweden, Switzerland, United Kingdom.

**Notes.** We examined the type of *Apanteleshedymeles* Nixon.

***Choerasrugulosus* Song & Chen, 2014**

*Choerasrugulosus* Song & Chen, 2014.

**Type information.** Holotype female, ZJUH (not examined but original description checked). Country of type locality: China.

**Geographical distribution.**OTL, PAL.

**OTL**: China (GZ, YN, ZJ); **PAL**: China (HA).

***Choerassemele* (Nixon, 1965)**

*Hypomicrogastersemele* Nixon, 1965.

**Type information.** Holotype female, NHMUK (examined). Country of type locality: Morocco.

**Geographical distribution.**PAL.

**PAL**: Canary Islands, Greece, Israel, Italy, Malta, Morocco, Spain.

***Choerassemilunatus* Song & Chen, 2014**

*Choerassemilunatus* Song & Chen, 2014.

**Type information.** Holotype female, ZJUH (not examined but original description checked). Country of type locality: China.

**Geographical distribution.**OTL.

**OTL**: China (GD).

***Choerassemirugosus* Song & Chen, 2014**

*Choerassemirugosus* Song & Chen, 2014.

**Type information.** Holotype female, ZJUH (not examined but original description checked). Country of type locality: China.

**Geographical distribution.**OTL, PAL.

**OTL**: China (HI, HN, YN, ZJ); **PAL**: China (HE).

***Choerassordidus* (Ashmead, 1900), new combination**

*Apantelessordidus* Ashmead, 1900.

*Microplitiscarinata* Ashmead, 1900.

**Type information.** Holotype male, NHMUK (examined). Country of type locality: Saint Vincent.

**Geographical distribution.**NEO.

**NEO**: Saint Vincent.

**Notes.**Muesebeck (1958b: 427) transferred this species from *Apanteles* to *Microplitis*, a decision accepted by other authors (Shenefelt 1972, Fernandez-Triana et al. 2015b). However, after we examined the male holotype it became clear that is not *Microplitis*, as it has an enlarged metacoxa (at least two thirds as long as entire metasoma), T1 does not have a median sulcus (the anterior half is broadly hollowed whereas the posterior half is rugose), the scutellar disc does not have a posteromedian band of rugosity, and the head and mesosoma are almost totally unsculptured (including completely smooth propodeum which has only a median longitudinal carina). We examined one female and two males of the type series of *Microplitiscarinata* Ashmead, 1900 which are also in the NHMUK; those specimens are very similar and clearly conspecific with the *sordidus* holotype. The female has a relatively long ovipositor and a pleated hypopygium. Based on the morphological characters discussed above, we consider this species to belong to the genus *Choeras*.

***Choerasstenoterga* (de Saeger, 1944), new combination**

*Microgasterstenoterga* de Saeger, 1944.

**Type information.** Holotype female, RMCA (not examined but original description checked). Country of type locality: Democratic Republic of Congo.

**Geographical distribution.**AFR.

**AFR**: Democratic Republic of Congo.

**Notes.** Based on the original description (de Saeger 1944), the best generic placement at present would be in *Choeras*. However, study of the type specimen will be needed in the future.

***Choerassuperbus* (de Saeger, 1944), new combination**

*Microgastersuperba* de Saeger, 1944.

**Type information.** Holotype female, RMCA (not examined but original description checked). Country of type locality: Democratic Republic of Congo.

**Geographical distribution.**AFR.

**AFR**: Democratic Republic of Congo.

**Notes.** Based on the original description, the best generic placement at present would be in *Choeras*, based on the propodeum with median carina, pleated hypopygium, length of ovipositor sheaths, and shapes of T1 and T2 (as described and illustrated in de Saeger 1944: 103–105).

***Choerassylleptae* (de Saeger, 1942), new combination**

*Microgastersylleptae* de Saeger, 1942.

**Type information.** Holotype female, RMCA (not examined but original description checked). Country of type locality: Democratic Republic of Congo.

**Geographical distribution.**AFR.

**AFR**: Democratic Republic of Congo, Ivory Coast, Rwanda.

**Notes.** Based on the original description and illustrations provided there (de Saeger 1942), the best generic placement at present would be in *Choeras*, based on the propodeum with median carina, pleated hypopygium, and length of the ovipositor sheaths.

***Choerastaftanensis* Ghafouri Moghaddam & van Achterberg, 2018**

*Choerastaftanensis* Ghafouri Moghaddam & van Achterberg, 2018.

**Type information.** Holotype female, DPPZ (not examined but original description checked). Country of type locality: Iran.

**Geographical distribution.**PAL.

**PAL**: Iran.

***Choerastakeuchii* (Watanabe, 1937)**

*Microgastertakeuchii* Watanabe, 1937.

**Type information.** Holotype female, EIHU (examined). Country of type locality: Japan.

**Geographical distribution.**PAL.

**PAL**: Japan, Russia (PRI).

***Choerastarasi* Kotenko, 2007**

*Choerastarasi* Kotenko, 2007.

**Type information.** Holotype female, SIZK (not examined but original description checked). Country of type locality: Russia.

**Geographical distribution.**PAL.

**PAL**: Russia (SAK).

***Choerastedellae* (Nixon, 1961)**

*Apantelestedellae* Nixon, 1961.

*Apantelesepinotiae* Fischer, 1962 [homonym of *Apantelesepinotiae* Viereck, 1912].

*Apantelesepinoticida* Fischer, 1966.

**Type information.** Holotype female, MMBC (not examined but subsequent treatment of the species checked). Country of type locality: Czech Republic.

**Geographical distribution.**PAL.

**PAL**: Austria, Bulgaria, Croatia, Czech Republic, Denmark, Finland, Germany, Greece, Hungary, Iran, Israel, Korea, Madeira Islands, Moldova, Netherlands, Poland, Romania, Russia (ZAB, PRI, YAR), Slovakia, Sweden, Switzerland, United Kingdom.

**Notes.** Our species concept is based on van Achterberg (2002).

***Choerastegularis* (Szépligeti, 1905)**

*Microgastertegularis* Szépligeti, 1905.

**Type information.** Type lost (not examined but subsequent treatment of the species checked). Country of type locality: Australia.

**Geographical distribution.**AUS.

**AUS**: Australia (NSW, WA).

**Notes.** Our species concept is based on Austin and Dangerfield (1992), Papp (2004) and Fernandez-Triana (2015). The later author commented on what we know about the status of this species: “Because the male holotype is lost (Austin and Dangerfield 1993, Papp 2004) the correct identity of this species may never be established unambiguously. However, it is clear that this species is not *Protomicroplitis*, based on the illustrations of the fore wing in Nixon (1965: fig. 304), and propodeum and mediotergites 1–3 in Austin and Dangerfield (1992: fig. 27). We agree with Austin and Dangerfield (1992) that it is most likely to be *Choeras*”.

***Choerastenuialatus* Song & Chen, 2014**

*Choerastenuialatus* Song & Chen, 2014.

**Type information.** Holotype female, ZJUH (not examined but original description checked). Country of type locality: China.

**Geographical distribution.**OTL.

**OTL**: China (ZJ).

***Choerastiro* (Reinhard, 1880)**

*Microgastertiro* Reinhard, 1880.

**Type information.** Type and depository unknown (not examined but subsequent treatment of the species checked). Country of type locality: Germany.

**Geographical distribution.**NEA, PAL.

**NEA**: Canada (NL, NS, PE); **PAL**: Austria, Bulgaria, France, Germany, Greece, Hungary, Iran, Israel, Poland, Romania, Russia (SAK, SAR), Slovakia, Spain, Switzerland, United Kingdom.

**Notes.** Location of type doubtful (see Nixon 1965). Our species concept is based on van Achterberg (2002) and Abdoli et al. (2019b).

***Choerastumidus* Song & Chen, 2014**

*Choerastumidus* Song & Chen, 2014.

**Type information.** Holotype female, ZJUH (not examined but original description checked). Country of type locality: China.

**Geographical distribution.**OTL, PAL.

**OTL**: China (GZ, HB, SN); **PAL**: China (NX).

***Choerasvacillatrix* (Wilkinson, 1930), new combination**

*Microgastervacillatrix* Wilkinson, 1930.

**Type information.** Holotype female, NHMUK (examined). Country of type locality: Uganda.

**Geographical distribution.**AFR.

**AFR**: Democratic Republic of Congo, Uganda.

**Notes.** Transferred to *Choeras* based on the fore wing having an areolet (vein r-m transparent but clearly visible), propodeum with median, longitudinal carina, hypopygium flexible and with several pleats, ovipositor sheaths relatively long and entirely setose, and the shapes of T1 and T2 in agreement with many other species in this genus.

***Choerasvacillatropsis* (de Saeger, 1944), new combination**

*Microgastervacillatropsis* de Saeger, 1944.

**Type information.** Holotype female, RMCA (not examined but original description checked). Country of type locality: Democratic Republic of Congo.

**Geographical distribution.**AFR.

**AFR**: Democratic Republic of Congo.

**Notes.** Based on the original description (de Saeger 1944), the best generic placement at present would be *Choeras*. This species was considered by de Saeger (1944: 97) as morphologically similar to *Microgastervacillatrix* Wilkinson, 1930, which is also being transferred to *Choeras* in the present paper (see notes under that species above).

***Choerasvalidicarinatus* Song & Chen, 2014**

*Choerasvalidicarinatus* Song & Chen, 2014.

**Type information.** Holotype female, ZJUH (not examined but original description checked). Country of type locality: China.

**Geographical distribution.**PAL.

**PAL**: China (NX).

***Choerasvalidus* (Thomson, 1895)**

*Microgastervalidus* Thomson, 1895.

**Type information.** Neotype female, MZLU (not examined but subsequent treatment of the species checked). Country of type locality: Sweden.

**Geographical distribution.**PAL.

**PAL**: France, Hungary, Italy, Netherlands, Russia (SAK), Slovakia, Sweden, Switzerland, United Kingdom.

**Notes.** Our species concept is based on van Achterberg (2002).

***Choerasvaricolor* Song & Chen, 2014**

*Choerasvaricolor* Song & Chen, 2014.

**Type information.** Holotype female, ZJUH (not examined but original description checked). Country of type locality: China.

**Geographical distribution.**OTL.

**OTL**: China (FJ, GD, GX, GZ, HI, SN, YN, ZJ).

***Choerasvenilia* (Nixon, 1965), new combination**

*Apantelesvenilia* Nixon, 1965.

**Type information.** Holotype female, AEIC (not examined but original description checked). Country of type locality: Philippines.

**Geographical distribution.**OTL.

**OTL**: Philippines.

**Notes.** We place this species within *Choeras* based on the complete median carina on the propodeum, and the fact it was keyed out by Nixon (1965) in the same couplet as *Apantelesdaphne* Nixon (a species for which we were able to examine the holotype and are transferring in this paper to *Choeras*, see above under that species for more details). Both *venilia* and *daphne* may be part of a different genus related to *Choeras* but, pending a comprehensive study of this genus, we consider the best placement at present is the one we propose here.

***Choerasyunnanensis* Song & Chen, 2014**

*Choerasyunnanensis* Song & Chen, 2014.

**Type information.** Holotype female, ZJUH (not examined but original description checked). Country of type locality: China.

**Geographical distribution.**OTL.

**OTL**: China (YN).

***Choeraszerovae* Kotenko, 2007**

*Choeraszerovae* Kotenko, 2007.

**Type information.** Holotype female, SIZK (not examined but original description checked). Country of type locality: Russia.

**Geographical distribution.**PAL.

**PAL**: Russia (PRI).

***Choeraszygon* Fagan-Jeffries & Austin, 2019**

*Choeraszygon* Fagan-Jeffries & Austin, 2019.

**Type information.** Holotype female, QM (not examined but original description checked). Country of type locality: Australia.

**Geographical distribution.**AUS.

**AUS**: Australia (QLD).

#### Genus Clarkinella Mason, 1981

***Clarkinella*** Mason, 1981: 66. Gender: feminine. Type species: *Clarkinellacanadensis* Mason, 1981, by original designation.

This is a New World genus, with two species currently described from the Nearctic and Neotropical regions. We have seen a few additional species in collections (CNC) but *Clarkinella* does not seem to be very species rich. No host data are currently available. There are eight DNA-barcode compliant sequences of this genus in BOLD, representing five BINs.

***Clarkinellacanadensis* Mason, 1981**

*Clarkinellacanadensis* Mason, 1981.

**Type information.** Holotype female, CNC (examined). Country of type locality: Canada.

**Geographical distribution.**NEA.

**NEA**: Canada (ON).

***Clarkinellaedithae* Mason, 1981**

*Clarkinellaedithae* Mason, 1981.

**Type information.** Holotype female, CNC (examined). Country of type locality: Trinidad & Tobago.

**Geographical distribution.**NEO.

**NEO**: Brazil (MG. RJ), Trinidad & Tobago.

#### Genus Cotesia Cameron, 1891

***Cotesia*** Cameron, 1891: 185. Gender: feminine. Type species: *Cotesiaflavipes* Cameron, 1891, by monotypy.

*Cryptapanteles* Viereck, 1909: 209. Type species: *Cryptapantelesrileyanus* Viereck, 1909 (= *Apantelesemarginatus* Riley, not Nees), by original designation and monotypy.

A cosmopolitan genus, with 328 described species known from all biogeographical regions of the planet, and perhaps 1,500–2,000 species (Mason 1981). Many European species were revised by Nixon and Papp in several papers from the 1970s and 1980s, as well as more recently by Shaw (2007, 2009, 2012a, 2012b, 2017b, Shaw et al. 2009, 2015). The Chinese species are keyed out in Chen and Song (2004). Other revisions include the species from Greenland (van Achterberg 2006), Réunion (Rousse and Gupta 2013), stem-borer parasitoids in Africa (van Achterberg and Polaszek 1996, van Achterberg and Walker 1998), the *flavipes* species group worldwide (Fujie et al. 2018) but overall the taxonomic coverage of the world species is far from complete. We have seen hundreds of undescribed species in collections, from both temperate and tropical areas. This is also one of the most morphologically distinctive genera of Microgastrinae, with perhaps only a few species of *Protapanteles* that might be confused with part of the genus. More than 30 families of Lepidoptera have been recorded as hosts for *Cotesia*, but many records are likely to be incorrect and/or need further verification. From a biological control perspective this is probably the most significant and well-studied genus of Microgastrinae in the world. In Costa Rica (ACG) most of the known hosts belong to three families: Nymphalidae, Saturniidae, and Sphingidae (unpublished information extracted from BOLD and ACG databases). There are almost 5,000 DNA-barcode compliant sequences of this genus in BOLD, representing 320 BINs, mostly from Canada and Costa Rica.

***Cotesiaabdinbekovae* Papp, 2009**

*Cotesiaabdinbekovae* Papp, 2009.

*Apantelesrufiventris* Abdinbekova, 1969 [secondary homonym of *Apantelesrufiventris* Bingham, 1906].

**Type information.** Holotype female, ZIN (not examined but subsequent treatment of the species checked). Country of type locality: Azerbaijan.

**Geographical distribution.**PAL.

**PAL**: Azerbaijan, Croatia, Russia (S), Turkmenistan.

**Notes.** Our species concept is based on Papp (1987a, 2009).

***Cotesiaabjecta* (Marshall, 1885)**

*Apantelesabjectus* Marshall, 1885.

*Apantelescomplanatus* Lyle, 1916.

**Type information.** Holotype female, NHMUK (examined). Country of type locality: United Kingdom.

**Geographical distribution.**PAL.

**PAL**: Croatia, Finland, France, Germany, Hungary, Iran, Ireland, Israel, Italy, Mongolia, Poland, Romania, Russia (N), Slovakia, Switzerland, United Kingdom, Yugoslavia.

**Notes.**Shenefelt (1972: 431) did not provide details for the type location and was even doubtful of the type being in London (NHMUK). However, Nixon (1974: 484 and especially 485) referred to the type specimen both in the species description and in additional comments added at the end of the species treatment. We have examined the type of *abjectus* (a female with number 3c.29, which is exactly the same code mentioned by Shenefelt) and also the type series of *Apantelescomplanatus* (Lyle, 1916), which was synonymized by Nixon (1974: 484), a decision we agree with. The species distribution in Iran and Russia is based on Belokobylskij et al. (2019).

***Cotesiaacaudus* (Provancher, 1886)**

*Microgasteracaudus* Provancher, 1886.

*Apanteleshydriae* Muesebeck, 1921.

**Type information.** Lectotype female, ULQC (not examined but subsequent treatment of the species checked). Country of type locality: Canada.

**Geographical distribution.**NEA.

**NEA**: Canada (NS, ON, QC), USA (CT, MA, NJ, NY, PA, RI, VA, WV, WI).

**Notes.** Our species concept is based on Muesebeck (1921), Mason (1981), Papp (1987a) and Fernandez-Triana (2010). The ending of the species name has been variously treated; following Article 31.2.1 of the ICZN the name is a noun in apposition and the original spelling *acaudus* must be retained.

***Cotesiaacerbiae* Shaw & Vikberg, 2015**

*Cotesiaacerbiae* Shaw & Vikberg, 2015.

**Type information.** Holotype female, RSME (examined). Country of type locality: Russia.

**Geographical distribution.**PAL.

**PAL**: Russia (YAN).

***Cotesiaacronyctae* (Riley, 1870)**

*Microgasteracronyctae* Riley, 1870.

*Apantelesorgyiae* Ashmead, 1893.

**Type information.** Holotype male, USNM (examined). Country of type locality: USA.

**Geographical distribution.**NEA.

**NEA**: Canada (AB, ON, SK), USA (CA, CO, CT, IL, IN, IA, MD, MA, MO, NH, NJ, OH).

**Notes.** The male specimen (USNM type number 2770) has one fore wing and the head detached (but glued to a piece of wood on the pin). The sex of the holotype had not been detailed before (e.g., Shenefelt 1972: 433 listed it as “?”) so it is here recorded for the first time. This species was first mentioned in Riley (1870: 120) as *Microgasteracronyctae*. From a footnote in that same page it appears that Riley intended to describe the species in a different manuscript; however, the 1870 publication provides details of the wasp larvae and cocoons, as well as comments on its Lepidoptera host, thus making that paper the de facto original description of *acronyctae*. This has been accepted by subsequent authors when recording the author and year of the species (e.g., Shenefelt 1972, Marsh 1979, Whitfield 1995, Fernandez-Triana 2010; also by Yu et al. 2016, although those authors considered 1871 (not 1870) as the year of publication). The wasp species was described in a more comprehensive way, including details of the adult wasp, differences with other Microgastrinae species, and a repetition of the biological information presented in 1870, in Riley (1881: 312–313), this time with the name *Apantelesacronyctae*.

***Cotesiaacuminata* (Reinhard, 1880)**

*Apantelesacuminatus* Reinhard, 1880.

*Apantelescultrator* Marshall, 1885.

**Type information.** Syntypes female and male, ZMHB (not examined but authoritatively identified specimens examined). Country of type locality: Germany.

**Geographical distribution.**OTL, PAL.

**OTL**: China (FJ); **PAL**: Armenia, Austria, China (BJ), Czech Republic, Finland, France, Georgia, Germany, Hungary, Israel, Romania, Russia (BU, PRI), Slovakia, Spain, Sweden, Tajikistan, Ukraine, Uzbekistan.

**Notes.** We examined the type of *Apantelescultrator* (Marshall, 1885). The species distribution in Uzbekistan is based on Belokobylskij et al. (2019).

***Cotesiaacutula* (Tobias, 1973)**

*Apantelesacutulus* Tobias, 1973.

**Type information.** Holotype female, ZIN (examined). Country of type locality: Lithuania.

**Geographical distribution.**PAL.

**PAL**: Hungary, Lithuania, Russia (NW).

***Cotesiaadippevora* Shaw, 2009**

*Cotesiaadippevora* Shaw, 2009.

**Type information.** Holotype female, RSME (examined). Country of type locality: Italy.

**Geographical distribution.**PAL.

**PAL**: Finland, Italy.

***Cotesiaaffinis* (Nees, 1834)**

*Microgasteraffinis* Nees, 1834.

*Microgastereuphorbiae* Bouché, 1834.

*Microgastervinulae* Bouché, 1834.

*Apantelesharpyiae* Niezabitowski, 1910.

*Apantelesplanus* Watanabe, 1932.

**Type information.** Neotype female, ZMHB (not examined but subsequent treatment of the species checked). Country of type locality: Germany.

**Geographical distribution.**AFR, OTL, PAL.

**AFR**: Cape Verde; **OTL**: China (GZ, HN, ZJ); **PAL**: Armenia, Austria, China (HL, LN, NM, NX, SN), France, Germany, Hungary, Italy, Japan, Kazakhstan, Korea, Latvia, Poland, Romania, Russia (PRI, ROS, YAR), Serbia, Slovakia, Spain, Sweden, Switzerland, Ukraine, United Kingdom.

**Notes.** Our species concept is based on Nixon (1974), Papp (1987a), Chen and Song (2004), and Kotenko (2007a).

***Cotesiaagricola* (Viereck, 1917)**

*Apantelesagricola* Viereck, 1917.

**Type information.** Holotype female, USNM (examined). Country of type locality: USA.

**Geographical distribution.**NEA.

**NEA**: USA (CT).

***Cotesiaalgonquinorum* (Viereck, 1917)**

*Apantelesalgonquinorum* Viereck, 1917.

**Type information.** Holotype female, USNM (examined). Country of type locality: USA.

**Geographical distribution.**NEA.

**NEA**: USA (CT).

***Cotesiaalia* (Muesebeck, 1958)**

*Apantelesalius* Muesebeck, 1958.

**Type information.** Holotype female, USNM (examined). Country of type locality: Venezuela.

**Geographical distribution.**NEO.

**NEO**: Brazil (SP), Peru, Venezuela.

**Notes.** The species name must be treated as an adjective and not as a noun (Doug Yanega, pers. comm.) and thus it must match the gender of the genus name.

***Cotesiaalternicolor* (You & Zhou, 1988)**

*Apantelesalternicolor* You & Zhou, 1988.

**Type information.** Holotype female, HUNAU (not examined but subsequent treatment of the species checked). Country of type locality: China.

**Geographical distribution.**PAL.

**PAL**: China (SD).

**Notes.** Our species concept is based on Chen and Song (2004).

***Cotesiaalypiae* (Muesebeck, 1922)**

*Apantelesalypiae* Muesebeck, 1922.

**Type information.** Holotype female, USNM (not examined but original description checked). Country of type locality: USA.

**Geographical distribution.**NEA.

**NEA**: USA (CT).

***Cotesiaamericana* (Lepeletier, 1825)**

*Microgasteramericanus* Lepeletier, 1825.

*Microgasterflaviventris* Cresson, 1865.

*Apantelesmexicanus* Ashmead, 1895.

**Type information.** Syntypes female and male, depository unknown (not examined but subsequent treatment of the species checked). Country of type locality: Martinique.

**Geographical distribution.**NEA, NEO.

**NEA**: USA (AZ, FL, OK, TX); **NEO**: Cuba, Dominican Republic, Guyana, Haiti, Jamaica, Martinique, Mexico, Puerto Rico.

**Notes.** Our species concept is based on Muesebeck (1921) and Wilkinson (1930c).

***Cotesiaamesis* (Nixon, 1974)**

*Apantelesamesis* Nixon, 1974.

**Type information.** Holotype female, NHMUK (examined). Country of type locality: Switzerland.

**Geographical distribution.**PAL.

**PAL**: Poland, Slovakia, Switzerland.

***Cotesiaammalonis* (Muesebeck, 1926)**

*Apantelesammalonis* Muesebeck, 1926.

**Type information.** Holotype female, USNM (not examined but original description checked). Country of type locality: USA.

**Geographical distribution.**NEA.

**NEA**: USA (NJ, NY).

***Cotesiaamphipyrae* (Watanabe, 1934)**

*Apantelesamphipyrae* Watanabe, 1934.

**Type information.** Holotype female, EIHU (examined). Country of type locality: Japan.

**Geographical distribution.**PAL.

**PAL**: Japan.

***Cotesiaanalis* (Nees, 1834)**

*Microgasteranalis* Nees, 1834.

*Microgasterpraetextata* Haliday, 1834.

*Microgastermediana* Ratzeburg, 1852.

*Apantelesleucaniae* Wilkinson, 1937.

**Type information.** Neotype female, ZMHB (not examined but authoritatively identified specimens examined). Country of type locality: United Kingdom.

**Geographical distribution.**PAL.

**PAL**: Armenia, Belgium, Czech Republic, France, Georgia, Germany, Hungary, Ireland, Italy, Netherlands, Russia (IRK), Sweden, Switzerland, United Kingdom.

**Notes.** We examined the type of *A.leucaniae* (Wilkinson).

***Cotesiaancilla* (Nixon, 1974)**

*Apantelesancilla* Nixon, 1974.

**Type information.** Holotype female, NHMUK (examined). Country of type locality: Germany.

**Geographical distribution.**PAL.

**PAL**: Armenia, Austria, Bulgaria, Croatia, Germany, Greece, Hungary, Iran, Israel, Italy, Japan, Macedonia, Mongolia, Netherlands, Russia (PRI), Slovakia, Spain, Switzerland, Turkey, Yugoslavia.

**Notes.** The holotype (with code 3c.1790) is deposited in the NHMUK and not in Berlin (ZHMB), as stated in Yu et al. (2016). The species distribution in Armenia is based on Belokobylskij et al. (2019).

***Cotesiaanisotae* (Muesebeck, 1921)**

*Apantelesanisotae* Muesebeck, 1921.

**Type information.** Holotype female, USNM (not examined but original description checked). Country of type locality: USA.

**Geographical distribution.**NEA.

**NEA**: Canada (NB, ON), USA (AR, CT, FL, MD, MA, NJ, NY, RI, TX, VA).

***Cotesiaanomidis* (Watanabe, 1942)**

*Apantelesanomidis* Watanabe, 1942.

**Type information.** Holotype female, EIHU (examined). Country of type locality: China.

**Geographical distribution.**OTL, PAL.

**OTL**: China (HN, JS, ZJ), Vietnam; **PAL**: China (LN, SN).

***Cotesiaanthelae* (Wilkinson, 1928)**

*Apantelesanthelae* Wilkinson, 1928.

**Type information.** Holotype female, NHMUK (not examined but subsequent treatment of the species checked). Country of type locality: Australia.

**Geographical distribution.**AUS.

**AUS**: Australia (NSW, VIC).

**Notes.** Our concept of this species is based on Austin and Dangerfield (1992).

***Cotesiaaphae* (Watanabe, 1934)**

*Apantelesaphae* Watanabe, 1934.

**Type information.** Holotype female, EIHU (examined). Country of type locality: Japan.

**Geographical distribution.**PAL.

**PAL**: Japan.

***Cotesiaarctica* (Thomson, 1895), status revised**

*Microgasterarcticus* Thomson, 1895.

**Type information.** Holotype female, MZLU (not examined but subsequent treatment of the species checked). Country of type locality: Norway.

**Geographical distribution.**PAL.

**PAL**: Norway.

**Notes.** Because of the confusion surrounding the application of this name and despite its possible synonymy (summarised by Shaw 2007, see also Broad et al. 2016) it seems best to regard *arcticus* as a valid species for the time being, especially because Marshall (1899) redescribed it without reference to any similarity with his own species *astrarches*.

***Cotesiaargynnidis* (Riley, 1889)**

*Apantelesargynnidis* Riley, 1889.

**Type information.** Holotype female, USNM (examined). Country of type locality: USA.

**Geographical distribution.**NEA.

**NEA**: USA (CA, CT, DC, IL, KY, MA, NJ, NY, TX, WV).

***Cotesiaasavari* (Sathe, 1989), new combination**

*Apantelesasavari* Sathe, 1989.

**Type information.** Holotype female, SUKI (not examined but original description checked). Country of type locality: India.

**Geographical distribution.**OTL.

**OTL**: India.

**Notes.** The original description is the only reference available for this species. Even though is not clear or consistent (e.g., compare the drawing of the propodeum with its description) it is evident that the species is not an *Apanteles*. Based on the short ovipositor, the drawing of the propodeum, and the recorded host, the best placement at present will be in *Cotesia*.

***Cotesiaastrarches* (Marshall, 1889)**

*Apantelesastrarches* Marshall, 1889.

*Apantelesgenalis* Tobias, 1964.

**Type information.** Lectotype female, PCMAG (examined). Country of type locality: United Kingdom.

**Geographical distribution.**PAL.

**PAL**: Afghanistan, Azerbaijan, Croatia, Cyprus, Finland, France, Georgia, Germany, Greece, Hungary, Kazakhstan, Macedonia, Moldova, Norway, Poland, Russia (C, NC, S), Slovakia, Slovenia, Spain, United Kingdom, Yugoslavia.

**Note.** In this paper we are removing *Microgasterarcticus* Thomson, 1895 (currently in *Cotesia*) from synonymy with *astrarches* and considering it as a distinct, valid species (see more details under *Cotesiaarctica* above).

***Cotesiaatalantae* (Packard, 1881)**

*Microgasteratalantae* Packard, 1881.

**Type information.** Syntypes female and male, depository unknown (not examined but subsequent treatment of the species checked). Country of type locality: USA.

**Geographical distribution.**NEA.

**NEA**: Canada (AB, MB, ON, QC, SK), USA (CO, CT, MA, MI, NH, NJ, NY, PA, RI, VT, WV, WY).

**Notes.**Shenefelt (1972: 448) mentioned the need to designate a lectotype but, as far as we know, none has been designated yet. Our species concept is based on Muesebeck (1921) and Papp (1987a).

***Cotesiaaururus* (Telenga, 1955)**

*Apantelesaururus* Telenga, 1955.

**Type information.** Lectotype female, ZIN (not examined but subsequent treatment of the species checked). Country of type locality: Russia.

**Geographical distribution.**PAL.

**PAL**: Georgia, Russia (ORE).

**Notes.** Our species concept is based on Tobias (1986) and Papp (1987a). The ending of the species name has been variously treated; following Article 31.2.1 of the ICZN the name is a noun in apposition and the original spelling *aururus* must be retained.

***Cotesiaaustraliensis* (Ashmead, 1900)**

*Apantelesaustraliensis* Ashmead, 1900.

**Type information.** Holotype female, USNM (examined). Country of type locality: Australia.

**Geographical distribution.**AUS.

**AUS**: Australia (VIC).

**Notes.** The holotype has a rather smooth propodeum, with only a trace of a median carina on the posterior 0.3, T1 is parallel-sided and T2 looks more like *Protapanteles*. Overall, the specimen looks more like a *Protapanteles* species than *Cotesia*, but we refrain from transferring it here until future studies better resolve the relationships between the two genera (see above under the section Brief diagnosis of all Microgastrinae genera as they are understood in this paper, for a discussion of *Protapanteles* as just a potential species group of *Cotesia*).

***Cotesiaautographae* (Muesebeck, 1921)**

*Apantelesautographae* Muesebeck, 1921.

**Type information.** Holotype female, USNM (not examined but original description checked). Country of type locality: USA.

**Geographical distribution.**NEA.

**NEA**: Canada (MB, NL, QC), USA (FL, GA, LA, MD, MI, OR, SC, SD, TN, TX, VA).

***Cotesiaautumnatae* Shaw, 2013**

*Cotesiaautumnatae* Shaw, 2013.

**Type information.** Holotype female, RSME (examined). Country of type locality: Finland.

**Geographical distribution.**PAL.

**PAL**: Finland.

***Cotesiaayerzai* (Brèthes, 1920), name amended**

*Apantelesayerzai* Brèthes, 1920.

*Apanteleswilliamsoni* Blanchard, 1935.

*Apantelesayerza* Blanchard, 1920 [incorrect subsequent spelling].

**Type information.** Lectotype female, MACN (not examined but subsequent treatment of the species checked). Country of type locality: Argentina.

**Geographical distribution.**NEO.

**NEO**: Argentina.

**Notes.** The original name of the species *Apantelesayerzai* was meant to honor Dr. Abel Ayerza, a man, as clearly mentioned in the original description, and also mentioned in a subsequent treatment of the species (Blanchard 1947: 8). Thus, the correct specific epithet must end with an i. That spelling of the species name was followed by most of the Spanish-speaking authors and is the one used in the catalogue of parasitic Hymenoptera from Argentina (de Santis 1967a: 135). However, the name was incorrectly spelled subsequently as *ayerza* by English-speaking authors, e.g., the catalogue of world Braconidae (Shenefelt 1972: 450) and the lectotype designation (Sharkey et al. 2000). Because the scientific literature about the species contains examples of both uses of the name within the past 50 years, it cannot be considered that the incorrect subsequent spelling is in prevailing use (cf. Article 33.3.1 of the ICZN) and thus there is no need to preserve that subsequent spelling. For that reason, we amend here the species name to its original spelling *ayerzai*.

***Cotesiabactriana* (Telenga, 1955), new combination**

*Apantelesbactrianus* Telenga, 1955.

**Type information.** Lectotype female, ZIN (not examined but original description checked). Country of type locality: Uzbekistan.

**Geographical distribution.**PAL.

**PAL**: Uzbekistan.

**Notes.** The original description as well as subsequent papers (Tobias 1986, Papp 1987a) clearly indicate this species belongs to *Cotesia*.

***Cotesiaballi* Oltra & Michelena, 1989**

*Cotesiaballi* Oltra & Michelena, 1989.

**Type information.** Holotype female, UVS (not examined but original description checked). Country of type locality: Spain.

**Geographical distribution.**PAL.

**PAL**: Spain.

**Notes.** The type material is probably in the Instituto Cavanilles de Biodiversitat y Biología Evolutiva, University of Valencia, Spain. But we have not been able to verify that information yet.

***Cotesiabambeytripla* (Shenefelt, 1972), new combination**

*Apantelesbambeytriplus* Shenefelt, 1972.

*Apantelesdiacrisiae* Risbec, 1951 [primary junior homonym of *Apantelesdiacrisiae* Gahan, 1917].

*Apantelesbambeyi* Risbec, 1952 [primary junior homonym of *Apantelesbambeyi* Risbec, 1951].

**Type information.** Syntypes female and male, depository unknown (not examined but original description checked). Country of type locality: Senegal.

**Geographical distribution.**AFR.

**AFR**: Senegal.

**Notes.** From the original description, as well as a drawing of propodeum and T1–T2 included there, it is clear that this species belongs in *Cotesia*. From a nomenclatural point of view, this species has had a rather complicated story, including having different names throughout the years. For the sake of clarity we briefly outline it here. It was originally described as *Apantelesdiacrisiae* by Risbec (1951). One year later, to avoid homonymy with *Apantelesdiacrisiae* (Gahan, 1917), it was changed to *Apantelesbambeyi* by Risbec (1952). Surprisingly, Risbec overlooked his own *Apantelesbambeyi* Risbec, 1951 (described in the same paper in which he had originally described *Apantelesdiacrisiae*!). Thus his 1952 paper created another junior homonym. To correct that, Shenefelt (1972) proposed a replacement name, *Apantelesbambeytriplus*.

***Cotesiaberberidis* (Rudow, 1910), new combination**

*Microgasterberberidis* Rudow, 1910.

**Type information.** Type and depository unknown (not examined but original description checked). Country of type locality: Austria.

**Geographical distribution.**PAL.

**PAL**: Austria.

**Notes.** The original description provides only a brief description of the cocoon mass shape and colour, and the number of wasps emerged (Rudow 1910: 230). We here transfer *berberidis* to *Cotesia* based on the statement that the species is similar to *glomeratus*, which has long been placed in *Cotesia*. In the original description it is stated that the host is a sawfly, *Argeberberidis* Schrank, 1802 (Argidae), but we deem that record likely to be incorrect, as the author reared many lepidopteran larvae alongside (as stated in that paper and others authored by him). In fact, the cocoons of *berberidis* are described as a sulfur yellow mass, which matches the shape and colour of cocoons of *Cotesia* species on *Aporiacrataegi* (Linnaeus, 1758) (Pieridae) (e.g., see http://www.lepiforum.de/lepiwiki.pl?Aporia_Crataegi), a lepidopteran treated by Rudow in the previous paragraph of his paper (we suspect that is the actual host of *berberidis*). Examination of the specimens will be needed in the future.

***Cotesiaberberis* (Nixon, 1974)**

*Apantelesberberis* Nixon, 1974.

**Type information.** Holotype female, NHMUK (examined). Country of type locality: Switzerland.

**Geographical distribution.**PAL.

**PAL**: Finland, Netherlands, Switzerland.

***Cotesiabhairavi* (Sathe & Inamdar, 1991), new combination**

*Parenionbhairavi* Sathe & Inamdar, 1991.

**Type information.** Holotype female, SUKI (not examined but original description checked). Country of type locality: India.

**Geographical distribution.**OTL.

**OTL**: India.

**Notes.** Based on the original description and drawings included there, it is very clear that this species does not belong to *Parenion*. Without examining the type material is difficult to conclude, but the best generic placement at present would be *Cotesia*, based on the sculpture of propodeum, T1-T3 shape, and hypopygium and ovipositor (as described and illustrated by the authors).

***Cotesiabiezankoi* (Blanchard, 1960), new combination**

*Apantelesbiezankoi* Blanchard, 1960.

**Type information.** Type and depository unknown (not examined but original description checked). Country of type locality: Brazil.

**Geographical distribution.**NEO.

**NEO**: Brazil (RS).

**Notes.** The original reference to this species appears to be in Biezanko (1960), where the wasp is mentioned as described by Everard E. Blanchard (in a letter he sent to Biezanko after examining the specimens he had sent to Blanchard). The paper from Biezanko (1960: 9) transcribed part of the information received, where the species name and species author (Blanchard) are clearly stated, the specimens collecting place and date, and Lepidoptera host are provided, and a brief comparison of metasoma color to differentiate adults of the new species from three other previously described *Apanteles* (all of those species currently placed in *Cotesia*) is also presented. Although the details in Biezanko (1960) are relatively scarce, they nevertheless satisfy the provisions of Articles 11 and 13 of the ICZN for a species name (published after 1930 but before 1961) to be available, and thus we include this species in our checklist. The new combination here proposed is based on the host information (the only known Microgastrinae wasps parasitizing *Opsiphanes* are two other *Cotesia* species), as well as the comparisons made with three other species that have long been placed in *Cotesia* (see previous sentences).

***Cotesiabifida* (Sharma, 1973), new combination**

*Apantelesbifida* Sharma, 1973.

**Type information.** Holotype female, IFRI (not examined but original description checked). Country of type locality: India.

**Geographical distribution.**OTL.

**OTL**: India.

**Notes.** Based on the drawings that are part of the original description, this species does not belong to *Deuterixys* (as had been proposed by Zeng et al. 2009). At present we consider the best generic placement to be *Cotesia*, based on the drawing of the propodeum as well as associated host record. Future study of the type material will be needed to conclude (as there is also a possibility that it could be *Parapanteles*).

***Cotesiabignellii* (Marshall, 1885)**

*Apantelesbignellii* Marshall, 1885.

**Type information.** Lectotype female, NHMUK (examined). Country of type locality: United Kingdom.

**Geographical distribution.**PAL.

**PAL**: Finland, France, Germany, Greece, Hungary, Ireland, Italy, Romania, Russia (ROS), Spain, Sweden, United Arab Emirates, United Kingdom, Yugoslavia.

**Notes.**Wilkinson (1945: 91) designated a lectotype (at the time referred to only as "the type") among four specimens and a cocoon mass, all glued to the same card, which is numbered as 1603. He marked the lectotype with a cross, which corresponds to the specimens to the top right on that card.

***Cotesiabonariensis* (Brèthes, 1916)**

*Protapantelesbonariensis* Brèthes, 1916.

**Type information.** Syntypes female and male, MACN (not examined). Country of type locality: Argentina.

**Geographical distribution.**NEO.

**NEO**: Argentina.

***Cotesiabosei* (Bhatnagar, 1950)**

*Apantelesbosei* Bhatnagar, 1950.

**Type information.** Holotype female, INPC (not examined but subsequent treatment of the species checked). Country of type locality: India.

**Geographical distribution.**OTL.

**OTL**: China (FJ, HN, SN), India.

**Notes.** Our species concept is based on Chen and Song (2004). The year of publication of the Bhatnagar paper was until recently commonly cited as 1948 and/or 1950 (e.g., Chen and Song 2004, Yu et al. 2016), probably following Shenefelt (1972) who referred to this paper as “Bhatnagar (1948) 1950”. While the intended year for Volume X, Parts I & II of the Indian Journal of Entomology was 1948, the actual dates of publication were June 1950 (Part I) and October 1950 (Part II), as clearly shown on the cover page of the Volume, which we have checked. Because the dates of publication are the ones to be considered, and for the sake of clarity, we hereby revise the species year of description to 1950.

***Cotesiabrachycera* (Thomson, 1895)**

Microgaster (Microplitis) brachycera Thomson, 1895.

Microgaster (Apanteles) brachycerus Thomson, 1895 [primary junior homonym of Microgaster (Microplitis) brachycera Thomson, 1895].

**Type information.** Lectotype female, MZLU (not examined but subsequent treatment of the species checked). Country of type locality: Sweden.

**Geographical distribution.**PAL.

**PAL**: Sweden.

**Notes.**Thomson (1895: 2237–2238) considered a single genus *Microgaster* with four subgenera (*Microgaster*, *HygroplitisMicroplitis*, and *Apanteles*), and described six new species within that framework. He described Microgaster (Microplitis) brachycera (page 2252 of his paper) and and a different species Microgaster (Apanteles) brachycerus (page 2259); as first revisers we designate the latter as a primary junior homonym as it appears later in the publication. Our species concept is based on van Achterberg (1997: 68), who stated that the species *Apanteles*/*Cotesiapraepotens* (*sensu*Wilkinson 1940, Nixon 1974, Papp 1988) was a misidentification and actually corresponded to *Cotesiabrachycera* (Thomson, 1895), a decision we agree with and follow here. The names of several species of *Cotesia* (*brachycera*, *juniperatae*, *praepotens*, *sericea*, *sessilis*, *tetrica*) have a complicated and somewhat interrelated history, which we attempt to detail below. While van Achterberg (1997: 68) deemed *C.brachycera* (Thomson, 1895) to be a valid species, some authors (e.g., Papp 1988: 153–156, Kotenko 2007: 186) considered *brachycera* to be a synonym of *C.praepotens* (Haliday, 1834), whereas others (e.g., Belokobylskij et al. 2003: 387, Broad et al. 2016: 243) considered *brachycera* to be a synonym of *C.sericea* (Nees, 1834). The name *praepotens* itself has been interpreted in two different ways: a) as the Haliday species (e.g., Papp 1988, Belokobylskij et al. 2003, Kotenko 2007, Broad et al. 2016); b) as a misidentification of *brachycera* (Thomson, 1895) (e.g., Wilkinson 1940, Nixon 1974, Papp 1988). The name *sericea* also has been interpreted in different ways: Lyle (1916: 186, 206–208) stated that Nees, subsequent to describing *sericea*, said later that it was the same species as *C.juniperatae* (Bouché, 1834), but then Reinhard (1881: 34) and Marshall (1885: 184) subsequently misinterpreted that. Thus, there has also been confusion regarding the status of *juniperatae*:

a) Yu et al. (2016) listed *juniperatae* as a synonym of *Cotesiasessilis* (Geoffroy, 1785) and cited Papp (1988) as the author of that synonymy, when in fact the opposite occurred, as Papp (1988: 154, also see 155) actually placed *sessilis* as a synonym of *juniperatae*, albeit with a question mark;

b) Belokobylskij et al. (2003: 387) considered *juniperatae* as a valid species and *sessilis* as its synonym;

c) Broad et al. (2016) also regarded *juniperatae* as valid but did not refer to *sessilis* as its synonym. Similarly, the name *sessilis* has been interpreted in different ways: Yu et al. (2016) considered it a valid species, with both *juniperatae* and *C.tetrica* (Reinhard, 1880) as synonyms; Papp (1988) and Kotenko (2007) considered *tetrica* a valid species with *sessilis* as a synonym (with a question mark); and Belokobylskij et al. (2003) and Broad et al (2016) deemed *tetrica* to be a valid species, but not with *sessilis* as a synonym of *tetrica*. Lastly, ongoing studies involving DNA barcoding, biology and morphology (Shaw, Quicke and Fernandez-Triana, unpublished data) indicate that there is the potential for other species/names to be involved, e.g., some of the current synonyms of *praepotens* (as accepted in this paper, see below under that species) may represent additional species, related to the ones we have mentioned in this paragraph and/or even other Palearctic *Cotesia* species. Because that is beyond the scope of the present paper, we do not expand on that here, but the reader must be aware that the situation with all these species is far from being resolved. For the sake of clarity, we detail here the arrangement that we are following in this paper, where we consider valid species *brachycera*, *juniperatae*, *praepotens*, *sericea*, and *tetrica*, whereas *sessilis* is listed as a *nomen dubium*.

***Cotesiabrevicornis* (Wesmael, 1837)**

*Microgasterbrevicornis* Wesmael, 1837.

*Apantelescleoceridis* Marshall, 1889.

**Type information.** Holotype female, RBINS (not examined but subsequent treatment of the species checked). Country of type locality: Belgium.

**Geographical distribution.**NEA, PAL.

**NEA**: Canada (AB); **PAL**: Azerbaijan, Belgium, Croatia, Finland, Germany, Hungary, Iceland, Ireland, Korea, Lithuania, Poland, Romania, Russia (YAR), Slovakia, Sweden, Switzerland, Turkey, Ukraine, United Kingdom, Yugoslavia.

**Notes.** Our species concept is based on Nixon (1974) and Papp (1986).

***Cotesiacajae* (Bouché, 1834)**

*Microgastercajae* Bouché, 1834.

*Microgasterdifficilis* Nees, 1834.

? *Microgasterperspicuus* Nees, 1834.

**Type information.** Lectotype female, ZMHB (not examined but subsequent treatment of the species checked). Country of type locality: Germany.

**Geographical distribution.**PAL.

**PAL**: Azerbaijan, Belarus, Belgium, China (XJ), Croatia, Czech Republic, Finland, France, Georgia, Germany, Hungary, Italy, Japan, Kazakhstan, Latvia, Moldova, Netherlands, Poland, Romania, Russia (AD, AST, IRK, KGD, KDA, ORL, PRI, ROS, SAK, SAM, SPE, TA, VOR, YAR), Slovakia, Spain, Sweden, Switzerland, Tajikistan, Ukraine, United Kingdom, Uzbekistan, Yugoslavia.

**Notes.** The name *perspicuus* (Nees, 1834) has sometimes been regarded as a senior synonym of both *cajae* (Bouché, 1834) and *ofella* (Nixon, 1974) (e.g., Yu et al. 2012, 2016). However, Marshall (1885: 183) had listed *cajae* as the senior name, and Papp (1988: 153) also considered *cajae* as a valid species. There has been little consensus on the correct application of the Nees name, the type of which is lost (see also comments under *Cotesiaofella* below). The arrangement proposed by Papp (1988), i.e., maintaining both *cajae* and *ofella* as valid species (and not as synonyms of *perspicuus*) has been subsequently followed by Papp (2005) and Broad et al. (2016), and it is also followed here. The species distribution in Japan is based on Belokobylskij et al. (2019).

***Cotesiacaligophagus* (Blanchard, 1964), new combination**

*Apantelescaligophagus* Blanchard, 1964.

**Type information.** Holotype female, MACN (not examined but original description checked). Country of type locality: Argentina.

**Geographical distribution.**NEO.

**NEO**: Argentina.

**Notes.** The generic placement of the species within *Cotesia* is clear from the original description and the accompanying drawings. Following Article 31.2.1 of the ICZN the name is a noun in apposition and the original spelling *caligophagus* must be retained.

***Cotesiacallimone* (Nixon, 1974)**

*Apantelescallimone* Nixon, 1974.

*Apantelesscelerata* Tobias, 1986.

**Type information.** Holotype female, NHMUK (examined). Country of type locality: United Kingdom.

**Geographical distribution.**PAL.

**PAL**: Bulgaria, Finland, Hungary, Iran, Ireland, Mongolia, Russia (KIR), Serbia, Slovakia, Switzerland, Turkey, Ukraine, United Kingdom.

**Notes.** The species’ distribution in Iran is based on Belokobylskij et al. (2019).

***Cotesiacalodetta* (Nixon, 1974)**

*Apantelescalodetta* Nixon, 1974.

**Type information.** Holotype female, NHMUK (examined). Country of type locality: Sweden.

**Geographical distribution.**PAL.

**PAL**: Russia (ALT), Sweden, Turkey.

***Cotesiacapucinae* (Fischer, 1961)**

*Apantelescapucinae* Fischer, 1961.

**Type information.** Holotype female, NHMW (not examined but subsequent treatment of the species checked). Country of type locality: Macedonia.

**Geographical distribution.**PAL.

**PAL**: Macedonia, Netherlands, Serbia.

**Notes.** Our concept of this species is based on Papp (1988) and Aquino et al. (2010).

***Cotesiacarduicola* (Packard, 1881)**

*Microgastercarduicola* Packard, 1881.

**Type information.** Syntypes female and male, depository unknown (not examined but subsequent treatment of the species checked). Country of type locality: USA.

**Geographical distribution.**NEA.

**NEA**: Canada (ON), USA (CT, IL, MA, NJ, TX).

**Notes.**Shenefelt (1972: 464) mentioned the need to designate a lectotype but, as far as we know, none has been designated yet. Our species concept is based on Muesebeck (1921), Mason (1981) and Fernandez-Triana (2010).

***Cotesiacerurae* (Muesebeck, 1926)**

*Apantelescerurae* Muesebeck, 1926.

**Type information.** Holotype female, USNM (not examined but original description checked). Country of type locality: USA.

**Geographical distribution.**NEA.

**NEA**: Canada (ON, QC), USA (CT, MD, NJ).

***Cotesiacharadrae* (Muesebeck, 1921)**

*Apantelescharadrae* Muesebeck, 1921.

**Type information.** Holotype female, USNM (not examined but original description checked). Country of type locality: USA.

**Geographical distribution.**NEA.

**NEA**: USA (CT, DC, MA).

***Cotesiachares* (Nixon, 1965)**

*Apanteleschares* Nixon, 1965.

**Type information.** Holotype female, NHMUK (examined). Country of type locality: United Kingdom.

**Geographical distribution.**PAL.

**PAL**: Hungary, Mongolia, Slovakia, United Kingdom.

***Cotesiacheesmanae* (Wilkinson, 1928), new combination**

*Apantelescheesmanae* Wilkinson, 1928.

**Type information.** Holotype female, NHMUK (examined). Country of type locality: Society Islands.

**Geographical distribution.**AUS.

**AUS**: Society Islands.

**Notes.** This species belongs to *Cotesia*, based on the strong, median longitudinal carinae of propodeum, shape and sculpture of T1 and T2, inflexible hypopygium, and short length of the ovipositor sheaths.

***Cotesiachiloluteelli* (You, Xiong & Wang, 1985)**

*Apanteleschiloluteelli* You, Xiong & Wang, 1985.

*Apanteleschiloluteelli* You, Xiong & Wang, 1985 [incorrect original spelling].

**Type information.** Holotype female, HUNAU (not examined but subsequent treatment of the species checked). Country of type locality: China.

**Geographical distribution.**OTL.

**OTL**: China (HN).

**Notes.** Our species concept is based on Zeng (2012). The emendation of the incorrect original spelling was done by You (1986).

***Cotesiachiloniponellae* (You & Wang, 1990)**

*Apanteleschiloniponellae* You & Wang, 1990.

**Type information.** Holotype female, HUNAU (not examined but subsequent treatment of the species checked). Country of type locality: China.

**Geographical distribution.**OTL.

**OTL**: China (HB, HN).

**Notes.** Our species concept is based on Chen and Song (2004).

***Cotesiachilonis* (Munakata, 1912)**

*Apanteleschilonis* Munakata, 1912.

*Apanteleschilocida* Viereck, 1912.

**Type information.** Neotype female, EIHU (not examined but subsequent treatment of the species checked). Country of type locality: Japan.

**Geographical distribution.**OTL, PAL.

**OLT**: China (FJ, GZ, HB, HN, JS, JX, SN, ZJ), India, Indonesia, Myanmar; **PAL**: China (AH), Iran, Japan, Korea.

**Notes.** An account of the rather complicated history of the species name and its year of publication was provided by Fernandez-Triana et al. (2015a). We also examined the type specimen, a female, of *Apanteleschilocida* (Viereck, 1912).

***Cotesiachinensis* (Wilkinson, 1930)**

*Apanteleschinensis* Wilkinson, 1930.

**Type information.** Holotype female, NHMUK (examined). Country of type locality: China.

**Geographical distribution.**OTL.

**OTL**: China (FJ, HB, HN, ZJ).

***Cotesiachrysippi* (Viereck, 1911)**

*Apanteleschrysippi* Viereck, 1911.

**Type information.** Holotype female, USNM (examined). Country of type locality: Mozambique.

**Geographical distribution.**AFR.

**AFR**: Madagascar, Mozambique, Nigeria, South Africa.

***Cotesiacingiliae* (Muesebeck, 1931)**

*Apantelescingiliae* Muesebeck, 1931.

**Type information.** Holotype female, USNM (not examined but original description checked). Country of type locality: USA.

**Geographical distribution.**NEA.

**NEA**: Canada (AB, BC, NB, NS, ON, QC), USA (MA).

***Cotesiacirphicola* (Bhatnagar, 1950)**

*Apantelescirphicola* Bhatnagar, 1950.

**Type information.** Holotype female, INPC (not examined but subsequent treatment of the species checked). Country of type locality: India.

**Geographical distribution.**OTL.

**OTL**: China (HN), India, Vietnam.

**Notes.** Our concept of this species is based on Chen and Song (2004). The year of publication of the Bhatnagar paper was until recently commonly cited as 1948 and/or 1950 (e.g., Chen and Song 2004, Yu et al. 2016), probably following Shenefelt (1972) who referred to this paper as “Bhatnagar (1948) 1950”. While the intended year for Volume X, Parts I & II of the Indian Journal of Entomology was 1948, the actual dates of publication were June 1950 (Part I) and October 1950 (Part II), as clearly shown on the cover page of the Volume, which we have checked. Because the dates of publication are the ones to be considered, and for the sake of clarity, we hereby revise the species year of description to 1950.

***Cotesiacleora* (Nixon, 1974)**

*Apantelescleora* Nixon, 1974.

**Type information.** Holotype female, NHMUK (examined). Country of type locality: United Kingdom.

**Geographical distribution.**PAL.

**PAL**: Czech Republic, Hungary, United Kingdom.

***Cotesiaclepta* (Tobias, 1986)**

*Apantelesclepta* Tobias, 1986.

**Type information.** Holotype female, ZIN (not examined but original description checked). Country of type locality: Moldova.

**Geographical distribution.**PAL.

**PAL**: Hungary, Moldova, Serbia, Sweden.

***Cotesiaclethrogynae* Long, 2014**

*Cotesiaclethrogynae* Long, 2014.

**Type information.** Holotype female, IEBR (not examined but original description checked). Country of type locality: Vietnam.

**Geographical distribution.**OTL.

**OTL**: Vietnam.

***Cotesiaclisiocampae* (Ashmead, 1903)**

*Apantelesclisiocampae* Ashmead, 1903.

**Type information.** Holotype female, USNM (examined). Country of type locality: USA.

**Geographical distribution.**NEA.

**NEA**: Canada (ON), USA (CT, NH, NJ, NY).

***Cotesiacompressithorax* (Hedqvist, 1965), new combination**

*Apantelescompressithorax* Hedqvist, 1965.

**Type information.** Holotype female, MZH (examined). Country of type locality: Cape Verde.

**Geographical distribution.**AFR.

**AFR**: Cape Verde.

**Notes.** This species was considered a junior synonym of *Cotesiapistrinariae* (Wilkinson, 1929) by Koponen (1989) and Forshage et al. (2016). However, we have examined the types (and other specimens) from both species and they are different species. *Cotesiacompresithorax* has a shorter malar space (as long as pedicel in the wording of the original description); thorax compressed and flattened; propodeum nearly smooth all over (only posteriorly with punctures); T1 not narrowing medially; T2 much more transverse (its width at posterior margin more than twice its median length); and darker colouration (most of legs and sternites brown). *Cotesiapistrinariae* has a longer malar space (at least twice the length of pedicel and slightly longer than mandible base width); thorax of normal appearance; propodeum mostly sculptured, with transverse striation centrally and a partial median carina (defined on posterior half of propodeum); T1 strongly narrowing medially; T2 much less transverse (its width at posterior margin around 1.5 × its median length); and lighter coloration (most of legs and sternites are yellow to light yellow-brown). Thus, here we remove *compressithorax* from synonymy with *pistrinariae* and transfer it from *Apanteles* to *Cotesia*.

***Cotesiacongestiformis* (Viereck, 1923)**

*Apantelescongestiformis* Viereck, 1923.

**Type information.** Holotype female, USNM (examined). Country of type locality: USA.

**Geographical distribution.**NEA.

**NEA**: USA (AK).

***Cotesiacongregata* (Say, 1836)**

*Microgastercongregata* Say, 1836.

*Microgasterutilis* French, 1880.

*Apantelesaugustus* Viereck, 1917.

**Type information.** Neotype female, USNM (not examined but authoritatively identified specimens examined). Country of type locality: USA.

**Geographical distribution.**NEA, NEO.

**NEA**: Canada (MB, NB, ON, PE), USA (AL, CO, CT, DC, FL, GA, IL, IA, KS, KY, MD, MA, MI, MS, MO, NH, NJ, NY, NC, PA, RI, SC, TN, VT, VA, WV); **NEO**: Brazil (SP), Honduras, Jamaica, Nicaragua, Peru, Puerto Rico.

**Notes.** We examined the female type and a paratype male of *Apantelesaugustus* (Viereck, 1917), currently a synonym of *C.congregata*.

***Cotesiacorylicolus* (Tobias, 1986)**

*Apantelescorylicolus* Tobias, 1986.

**Type information.** Holotype female, ZIN (not examined but original description checked). Country of type locality: Azerbaijan.

**Geographical distribution.**PAL.

**PAL**: Azerbaijan, Hungary, Netherlands, Russia (NC), Serbia.

**Notes.** Following Article 31.2.2 of the ICZN, in the absence of an original statement that the epithet is adjectival, the name is to be treated as a noun in apposition and the original spelling *corylicolus* must be retained.

***Cotesiacoryphe* (Nixon, 1974)**

*Apantelescoryphe* Nixon, 1974.

**Type information.** Holotype female, NHMUK (examined). Country of type locality: United Kingdom.

**Geographical distribution.**PAL.

**PAL**: United Kingdom.

**Notes.**Papp (1987a) synonymized *C.coryphe* under *C.rubripes* (Haliday, 1834), which was refuted by Broad et al. (2016), in part based on the host data given by Nixon (1974), a decision we accept and follow here.

***Cotesiacrambi* (Weed, 1887)**

*Apantelescrambi* Weed, 1887.

**Type information.** Lectotype female, INHS (not examined but subsequent treatment of the species checked). Country of type locality: USA.

**Geographical distribution.**NEA.

**NEA**: Canada (ON, QC), USA (CT, IL, KS, KY, MD, MO, NJ, OH, SD, TN).

**Notes.** Our species concept is based on Muesebeck (1921), Papp (1986) and Fernandez-Triana et al. (2014c).

***Cotesiacrassifemorata* van Achterberg, 2006**

*Cotesiacrassifemorata* van Achterberg, 2006.

**Type information.** Holotype female, ZMUC (not examined but original description checked). Country of type locality: Greenland.

**Geographical distribution.**NEA.

**NEA**: Greenland.

***Cotesiacultellata* (Tobias, 1966)**

*Apantelescultellatus* Tobias, 1966.

**Type information.** Holotype female, ZIN (not examined but original description checked). Country of type locality: Uzbekistan.

**Geographical distribution.**PAL.

**PAL**: Uzbekistan.

***Cotesiacuprea* (Lyle, 1925)**

*Apantelescupreus* Lyle, 1925.

**Type information.** Syntypes female and male, NHMUK (examined). Country of type locality: United Kingdom.

**Geographical distribution.**PAL.

**PAL**: Azerbaijan, Bulgaria, Canary Islands, Finland, France, Germany, Greece, Hungary, Iran, Lithuania, Mongolia, Netherlands, Poland, Romania, Russia (NW), Slovakia, Spain, Switzerland, Turkey, United Kingdom.

**Notes.** The species distribution in Iran is based on Belokobylskij et al. (2019).

***Cotesiacyaniridis* (Riley, 1889)**

*Apantelescyaniridis* Riley, 1889.

**Type information.** Holotype female, USNM (examined). Country of type locality: USA.

**Geographical distribution.**NEA.

**NEA**: Canada (ON, QC), USA (AZ, CO, CT, IL, IA, NJ, NY, WV).

***Cotesiacynthiae* (Nixon, 1974)**

*Apantelescynthiae* Nixon, 1974.

**Type information.** Holotype female, NHMUK (examined). Country of type locality: Austria.

**Geographical distribution.**PAL.

**PAL**: Austria, Bulgaria, France, Hungary, Iran, Switzerland, Turkey.

***Cotesiadanaisae* (Hedqvist, 1965)**

*Apantelesdanaisae* Hedqvist, 1965.

**Type information.** Holotype female, MZH (not examined but subsequent treatment of the species checked). Country of type locality: Cape Verde.

**Geographical distribution.**AFR.

**AFR**: Cape Verde.

**Notes.**Forshage et al. (2016) considered the type material to be lost; however, in 2017 it was found by the senior author of this paper in another section of the MZH collection.

***Cotesiadecaryi* (Granger, 1949)**

*Apantelesdecaryi* Granger, 1949.

**Type information.** Syntypes female and male, MNHN (not examined but original description checked). Country of type locality: Madagascar.

**Geographical distribution.**AFR.

**AFR**: Madagascar.

**Notes.** According to a recent catalogue on Braconidae from Madagascar (Madl and van Achterberg 2014), this species was transferred to *Cotesia* back in 2002 (Kuklinski and Borgemeister 2002). While Kuklinski and Borgemeister mentioned the species as *Cotesia* in their paper, there is no formal transfer there, nor an explanation as to why. After reading the original description, we concur that the species indeed belongs in *Cotesia*, and for the sake of clarity we revise its combination here.

***Cotesiadeliadis* (Bingham, 1906)**

*Apantelesdeliadis* Bingham, 1906.

**Type information.** Syntypes female and male, OUMNH (not examined but subsequent treatment of the species checked). Country of type locality: Australia.

**Geographical distribution.**AUS.

**AUS**: Australia (QLD).

**Notes.** Our species concept is based on Wilkinson (1928a).

***Cotesiadelicata* (Howard, 1897)**

*Apantelesdelicatus* Howard, 1897.

**Type information.** Holotype female, USNM (not examined but original description checked). Country of type locality: USA.

**Geographical distribution.**NEA.

**NEA**: USA (CT, DC, MD, NJ, NY).

**Notes.** Apart from the original description, our species concept is based on Muesebeck (1921).

***Cotesiadelphinensis* (Granger, 1949), new combination**

*Apantelesdelphinensis* Granger, 1949.

**Type information.** Syntypes female and male, MNHN (not examined but original description checked). Country of type locality: Madagascar.

**Geographical distribution.**AFR.

**AFR**: Madagascar.

**Notes.** This species is clearly not an *Apanteles*; although the original description is not clear or detailed enough to conclude, we consider that the best generic placement at present would be in *Cotesia*. Further examination of the type series will be needed in the future.

***Cotesiadepressa* (Viereck, 1912)**

*Apantelesdepressus* Viereck, 1912.

**Type information.** Holotype female, USNM (examined). Country of type locality: USA.

**Geographical distribution.**NEA.

**NEA**: Canada (QC), USA (IN).

**Notes.** Most of the holotype specimen is missing (the point has remnants of one leg glued, and the pin has all associated labels, but the rest of the specimen is missing from the unit tray).

***Cotesiadepressithorax* (Tobias, 1964)**

*Apantelesdepressithorax* Tobias, 1964.

**Type information.** Holotype female, ZIN (not examined but original description checked). Country of type locality: Kazakhstan.

**Geographical distribution.**PAL.

**PAL**: Kazakhstan.

**Notes.** Apart from the original description, our species concept is also based on Tobias (1986) and Papp (1987a).

***Cotesiadiacrisiae* (Gahan, 1917)**

*Apantelesdiacrisiae* Gahan, 1917.

**Type information.** Holotype female, USNM (not examined but subsequent treatment of the species checked). Country of type locality: USA.

**Geographical distribution.**NEA.

**NEA**: Canada (ON, QC), USA (CO, DE, DC, IL, KS, LA, MD, MS, MO, NJ, OK, SC, VA, WV).

**Notes.** Our species concept is based on Muesebeck (1921), Papp (1987a), Whitfield (1995a), Fernandez-Triana (2010), and images of the holotype available at http://www.usnmhymtypes.com/.

***Cotesiadictyoplocae* (Watanabe, 1940)**

*Apantelesdictyoplocae* Watanabe, 1940.

**Type information.** Holotype female, EIHU (examined). Country of type locality: Japan.

**Geographical distribution.**OTL, PAL.

**OTL**: China (FJ, HN, YN, ZJ), India; **PAL**: China (LN), Japan, Korea.

***Cotesiadisparis* (Tobias, 1986)**

*Apantelesdisparis* Tobias, 1986.

**Type information.** Holotype female, ZIN (not examined but paratype examined). Country of type locality: Azerbaijan.

**Geographical distribution.**PAL.

**PAL**: Azerbaijan, Hungary.

**Notes.** We examined one female paratype from the same cocoon mass than the (not examined by us) holotype.

***Cotesiadiurnii* Rao & Nikam, 1984**

*Cotesiadiurnii* Rao & Nikam, 1984.

**Type information.** Holotype female, NZSI (not examined but original description checked). Country of type locality: India.

**Geographical distribution.**OTL.

**OTL**: India.

***Cotesiadiversa* (Muesebeck & Walkely, 1951)**

*Apantelesdiversa* Muesebeck & Walkely, 1951.

*Apantelescoxalis* Muesebeck, 1926 [homonym of *Apantelescoxalis* Szépligeti, 1911].

**Type information.** Holotype female, USNM (not examined but subsequent treatment of the species checked). Country of type locality: USA.

**Geographical distribution.**NEA.

**NEA**: Canada (MB), USA (CT).

**Notes.** Our species concept is based on Muesebeck and Walkley (1951), Mason (1981), Whitfield (1995a) and Fernandez-Triana (2010).

***Cotesiaeffrena* (Wilkinson, 1928), new combination**

*Apanteleseffrenus* Wilkinson, 1928.

**Type information.** Holotype female, NHMUK (examined). Country of type locality: India.

**Geographical distribution.**OTL.

**OTL**: India.

**Notes.** Examination of the holotype reveals that this species belongs in *Cotesia*, based on the propodeum with complete median, longitudinal carina, partial transverse carina, shape and sculpture of T1 and T2, inflexible hypopygium and short ovipositor sheaths.

***Cotesiaelaeodes* (de Saeger, 1944), new combination**

*Apanteleselaeodes* de Saeger, 1944.

**Type information.** Holotype female, RMCA (not examined but original description checked). Country of type locality: Democratic Republic of Congo.

**Geographical distribution.**AFR.

**AFR**: Democratic Republic of Congo.

**Notes.** Based on the original description and drawings (de Saeger 1944), the best generic placement is in *Cotesia*.

***Cotesiaelectrae* (Viereck, 1912)**

*Apanteleselectrae* Viereck, 1912.

**Type information.** Holotype female, USNM (examined). Country of type locality: USA.

**Geographical distribution.**NEA, NEO.

**NEA**: Canada (BC), USA (AZ, CA, CO, NM, TX, UT); **NEO**: Mexico.

**Notes.** According to Papp (1987a) this species looks similar to *C.euchaetis*. But, after examining the holotypes of both species we found that there are many differences to clearly separate them.

***Cotesiaeliniae* Papp, 1989**

*Cotesiaeliniae* Papp, 1989.

**Type information.** Holotype female, HNHM (not examined but subsequent treatment of the species checked). Country of type locality: Greenland.

**Geographical distribution.**NEA.

**NEA**: Canada (NT, NU), Greenland.

**Notes.** Our species concept is based on van Achterberg (2006) and Fernandez-Triana et al. (2017b).

***Cotesiaelongata* Zargar & Gupta, 2019**

*Cotesiaelongata* Zargar & Gupta, 2019.

**Type information.** Holotype female, NBAIR (not examined but original description checked). Country of type locality: Iran.

**Geographical distribution.**PAL.

**PAL**: Iran.

***Cotesiaempretiae* (Viereck, 1913)**

*Apantelesempretiae* Viereck, 1913.

*Apantelessibinidis* Rohwer, 1915.

**Type information.** Holotype female, USNM (examined). Country of type locality: USA.

**Geographical distribution.**NEA, NEO.

**NEA**: USA (AL, DE, DC, FL, IL, LA, MD, MA, MO, NJ, VA); **NEO**: Ecuador.

**Notes.** We also examined the type of *Apantelessibinidis* (Rohwer, 1915), a female specimen.

***Cotesiaendii* (Sathe & Ingawale, 1995), new combination**

*Apantelesendii* Sathe & Ingawale, 1995.

**Type information.** Holotype female, NZSI (not examined but original description checked). Country of type locality: India.

**Geographical distribution.**OTL.

**OTL**: India.

**Notes.** The original description of the species is problematic, and drawings are clearly wrong (e.g., the depiction of the fore wing) or are in contradiction with the written description (e.g., is not clear in fig. 1h of Sathe and Ingawale (1995) what are the anterior and posterior margins of T1, as the sculpture described in the text does not match the drawing, and neither does the drawn shape match the text description of T1 as barrel-shaped). What is very clear is that the species is not an *Apanteles* (based on the propodeum sculpture, unpleated hypopygium, and setose vannal lobe in hind wings). Based on the overall description and recorded host, the best generic placement that can be proposed for this species at present is within *Cotesia* (coincidentally, the authors stated that the species is similar to *Apantelescirphicola*, which has long been considered as belonging in *Cotesia*). Further study of the specimens will be needed to unambiguously confirm the generic identity of the species.

***Cotesiaenypiae* (Mason, 1959)**

*Apantelesenypiae* Mason, 1959.

**Type information.** Holotype female, CNC (examined). Country of type locality: Canada.

**Geographical distribution.**NEA.

**NEA**: Canada (BC).

***Cotesiaerionotae* (Wilkinson, 1928)**

*Apanteleserionotae* Wilkinson, 1928.

**Type information.** Holotype female, NHMUK (not examined but subsequent treatment of the species checked). Country of type locality: Malaysia.

**Geographical distribution.**AFR, AUS, OTL.

**AFR**: Mauritius; **AUS**: Guam, Hawaiian Islands, Papua New Guinea; **OTL**: China (TW), India, Indonesia, Malaysia, Thailand.

**Notes.** Our concept of this species is based on Austin and Dangerfield (1992). The only Afrotropical record so far is from an introduction for biological control purposes (from Malaysia (Sabah) to Mauritius, see Madl and van Achterberg 2014); as far as we know there is no published information confirming if the species was established or not. Additional comments on distribution and biology of the species can be found in Cock (2015).

***Cotesiaerrator* (Nixon, 1974)**

*Apanteleserrator* Nixon, 1974.

**Type information.** Holotype female, NHMUK (examined). Country of type locality: United Kingdom.

**Geographical distribution.**PAL.

**PAL**: Austria, Switzerland, Russia (NW), United Kingdom.

***Cotesiaeuchaetis* (Ashmead, 1898)**

*Apanteleseuchaetis* Ashmead, 1898.

**Type information.** Syntypes female and male, USNM (examined). Country of type locality: USA.

**Geographical distribution.**NEA.

**NEA**: USA (CT, IL, MA, NH, NJ, NY, PA, RI, TX, VA, WV).

**Notes.** By all accounts this species seems to better be placed in *Protapanteles* than *Cotesia*, but we refrain from transferring it here until future studies resolve better the relationships between those two genera (see above under the section Brief diagnosis of all Microgastrinae genera as they are understood in this paper, for a discussion of *Protapanteles* as just a potential species group of *Cotesia*; p 35, 36. The whole body of *euchaetis* is unusually smooth (although the female syntype has many body parts covered in glue, artificially increasing the shiny and smooth appearance); T1 is parallel-sided, and especially T2 is subtriangular (trapezoidal) and rather small and narrow (very much unlike *Cotesia* and more like *Protapanteles*); the propodeum has only a short, apically defined, median carina (discernible in the female only) without any other carinae visible on the propodeum (although perhaps the glue obscures the sculpture, if there is any carination, it would still be very faint). Interestingly, Michel-Salzat and Whitfield (2004) in a phylogenetic analysis of 25 species of *Cotesia* clearly recovered *euchaetis* as part of *Cotesia*, with strong support (those results might indirectly support the opinion that *Protapanteles* species represent just a species group within *Cotesia*). It must also be noted that Papp (1987a) provided a rather poor diagnosis of the species in his key, not likely to work properly as a diagnostic tool (and best avoided when studying that group of species).

***Cotesiaeulipis* (Nixon, 1974)**

*Apanteleseulipis* Nixon, 1974.

**Type information.** Holotype female, NHMUK (examined). Country of type locality: United Kingdom.

**Geographical distribution.**PAL.

**PAL**: Bulgaria, Finland, Germany, Greece, Hungary, Sweden, United Kingdom.

***Cotesiaeunomiae* Shaw, 2009**

*Cotesiaeunomiae* Shaw, 2009.

**Type information.** Holotype female, RSME (examined). Country of type locality: Belgium.

**Geographical distribution.**PAL.

**PAL**: Belgium.

***Cotesiaeuphobetri* (Blanchard, 1935), new combination**

*Apanteleseuphobetri* Blanchard, 1935.

**Type information.** Syntypes female and male, MACN (not examined but original description checked). Country of type locality: Argentina.

**Geographical distribution.**NEO.

**NEO**: Argentina.

**Notes.** This species was described based on specimens from the Blanchard collection, which we believe is currently deposited in the MACN. The original description and illustrations there (scutellar disc, propodeum, T1–T2, part of fore wing, tip of antenna), strongly suggest that this species belongs in *Cotesia*. The species has the propodeum with a median, longitudinal carina, in addition to a more or less complete transverse carina forking around the spiracles, T1 slightly widening posteriorly, T2 is sub-rectangular, and the ovipositor sheaths barely protrude.

***Cotesiaeuphydryidis* (Muesebeck, 1921)**

*Apanteleseuphydryidis* Muesebeck, 1921.

**Type information.** Holotype female, USNM (not examined but original description checked). Country of type locality: USA.

**Geographical distribution.**NEA.

**NEA**: USA (MD, NJ, NY, VA).

***Cotesiaeuprocti* Sathe, 2005**

*Cotesiaeuprocti* Sathe, 2005.

**Type information.** Holotype female, SUKI (not examined but original description checked). Country of type locality: India.

**Geographical distribution.**OTL.

**OTL**: India.

***Cotesiaeuryale* (Nixon, 1974)**

*Apanteleseuryale* Nixon, 1974.

**Type information.** Holotype female, NHMUK (examined). Country of type locality: France.

**Geographical distribution.**PAL.

**PAL**: Bulgaria, Czech Republic, France, Greece, Hungary, Iran, Macedonia, Mongolia, Netherlands, Russia (C, IR, NW, S), Switzerland, Yugoslavia.

**Notes.** The presence of this species in the United Kingdom was questioned by Broad et al. (2016), a decision we accept and follow here. The presence of the species in Iran is based in Belokobylskij et al. (2019).

***Cotesiaeuthaliae* (Bhatnagar, 1950), new combination**

*Apanteleseuthaliae* Bhatnagar, 1950.

**Type information.** Holotype female, INPC (not examined but original description checked). Country of type locality: India.

**Geographical distribution.**OTL.

**OTL**: India.

**Notes.** Transferred to *Cotesia* based on the propodeum having a strong, median longitudinal carina, T1 parallel-sided, T2 more or less rectangular and as long as T3, and ovipositor sheaths very short. The year of publication of the Bhatnagar paper was until recently commonly cited as 1948 and/or 1950 (e.g., Chen and Song 2004, Yu et al. 2016), probably following Shenefelt (1972) who referred to this paper as “Bhatnagar (1948) 1950”. While the intended year for Volume X, Parts I & II of the Indian Journal of Entomology was 1948, the actual dates of publication were June 1950 (Part I) and October 1950 (Part II), as clearly shown on the cover page of the Volume, which we have checked. Because the dates of publication are the ones to be considered, and for the sake of clarity, we hereby revise the species year of description to 1950.

***Cotesiaevagata* (Papp, 1973)**

*Apantelesevagatus* Papp, 1973.

**Type information.** Holotype female, HNHM (not examined but original description checked). Country of type locality: Turkmenistan.

**Geographical distribution.**PAL.

**PAL**: Jordan, Turkmenistan.

***Cotesiaexelastisae* (Bhatnagar, 1950), new combination**

*Apantelesexelastisae* Bhatnagar, 1950.

**Type information.** Holotype male, INPC (not examined but original description checked). Country of type locality: India.

**Geographical distribution.**OTL.

**OTL**: India.

**Notes.** Because the only known specimen is male, it is difficult to conclude with certainty, but the original description makes clear this species is not *Apanteles*. Here we transfer it to *Cotesia* based on the propodeum having a strong, median longitudinal carina, metatibial spurs of equal size and relatively short, and T1 mostly parallel-sided (although posterior 0.2 narrows towards posterior margin). Additional support to consider *exelastisae* in *Cotesia*comes from the original description, which considered it morphologically close to *Apanteleserionotae* Wilkinson (currently placed in *Cotesia*) and also the host species being Pterophoridae (Bhatnagar 1950). The year of publication of the Bhatnagar paper was until recently commonly cited as 1948 and/or 1950 (e.g., Chen and Song 2004, Yu et al. 2016), probably following Shenefelt (1972) who referred to this paper as “Bhatnagar (1948) 1950”. While the intended year for Volume X, Parts I & II of the Indian Journal of Entomology was 1948, the actual dates of publication were June 1950 (Part I) and October 1950 (Part II), as clearly shown on the cover page of the Volume, which we have checked. Because the dates of publication are the ones to be considered, and for the sake of clarity, we hereby revise the species year of description to 1950.

***Cotesiafascifemorata* van Achterberg, 2006**

*Cotesiafascifemorata* van Achterberg, 2006.

**Type information.** Holotype female, ZMUC (not examined but original description checked). Country of type locality: Greenland.

**Geographical distribution.**NEA.

**NEA**: Greenland.

***Cotesiaferruginea* (Marshall, 1885)**

*Apantelesferrugineus* Marshall, 1885.

**Type information.** Lectotype female, NHMUK (examined). Country of type locality: United Kingdom.

**Geographical distribution.**PAL.

**PAL**: Belgium, Germany, Hungary, Italy, Korea, Lithuania, Netherlands, Romania, Russia (PRI), Slovakia, Switzerland, Turkey, Ukraine, United Kingdom.

**Notes.**Wilkinson (1945) stated that the specimen was in the Essex Museum of Natural History, but it is currently in the NHMUK.

***Cotesiafiskei* (Viereck, 1910)**

*Apantelesfiskei* Viereck, 1910.

**Type information.** Holotype female, USNM (examined). Country of type locality: USA.

**Geographical distribution.**NEA.

**NEA**: Canada (AB, BC, MB, NB, NL, NS, ON, SK), USA (CT, KS, MA, MT, OR, WI).

***Cotesiaflagellator* (Wilkinson, 1930)**

*Apantelesflagellator* Wilkinson, 1930.

**Type information.** Holotype female, NHMUK (examined). Country of type locality: Uganda.

**Geographical distribution.**AFR.

**AFR**: Uganda.

***Cotesiaflagitata* (Papp, 1971)**

*Apantelesflagitatus* Papp, 1971.

*Apantelesjaicus* Tobias, 1986.

**Type information.** Holotype female, HNHM (not examined but subsequent treatment of the species checked). Country of type locality: Mongolia.

**Geographical distribution.**PAL.

**PAL**: Kazakhstan, Mongolia.

**Notes.** Our species concept is based on Papp (1987a).

***Cotesiaflaviconchae* (Riley, 1881)**

*Apantelesflaviconchae* Riley, 1881.

**Type information.** Lectotype female, USNM (not examined but subsequent treatment of the species checked). Country of type locality: USA.

**Geographical distribution.**NEA.

**NEA**: Canada (ON), USA (AR, CA, CT, DC, IL, IA, LA, MD, MA, MN, MO, OK, TX, UT, VA, WA, WV).

**Notes.** Our species concept is based on Muesebeck (1921), Mason (1981), Whitfield (1995a) and Fernandez-Triana (2010).

***Cotesiaflavicornis* (Riley, 1889)**

*Apantelesflavicornis* Riley, 1889.

**Type information.** Syntypes female and male, USNM (examined). Country of type locality: USA.

**Geographical distribution.**NEA.

**NEA**: Canada (MB, ON), USA (CT, MO, NJ, TX).

***Cotesiaflavipes* Cameron, 1891**

*Cotesiaflavipes* Cameron, 1891.

Apanteles (Stenopleura) nonagriae Viereck, 1913.

*Apantelessimplicis* Viereck, 1913.

*Apantelesflavatus* Ishida, 1915.

**Type information.** Holotype male, NHMUK (examined). Country of type locality: India.

**Geographical distribution.**AFR, AUS, NEA, NEO, OTL, PAL.

**AFR**: Ethiopia, Kenya, Madagascar, Mauritius, Mozambique, Réunion, Tanzania, Uganda; **AUS**: Australia (NSW, QLD), Papua New Guinea; **NEA**: USA (FL, TX); **NEO**: Barbados, Brazil, Costa Rica, Guadeloupe, Jamaica, Mexico, Peru, Saint Kitts & Nevis, Trinidad & Tobago, Venezuela; **OTL**: Bangladesh, China (FJ, GD, GX, GZ, HI, HK, HB, HN, JS, JX, SN, TW, YN, ZJ), India, Indonesia, Malaysia, Myanmar, Nepal, Pakistan, Philippines, Ryukyu Islands, Sri Lanka, Thailand, Vietnam); **PAL**: China (AH), Japan.

**Notes.** We examined the type of *A.simplicis* (Viereck, 1913), a female specimen from Taiwan considered to be a synonym of *C.flavipes* since at least Wilkinson (1928a). The female type of *A.simplicis* looks slightly different to other specimens of *C.flavipes* we have examined, and we caution that it might represent another species within the *flavipes* species-complex (see Fujie et al. 2018). We also examined the type of Apanteles (Stenopleura) nonagriae Viereck, 1913, another female specimen from Taiwan, which looks similar to *A.simplicis* (but is not related to *Apantelesnongriae* Olliff, 1893).

***Cotesiafluvialis* (Balevski, 1980)**

*Apantelesfluvialis* Balevski, 1980.

**Type information.** Holotype female, ZIN (not examined but subsequent treatment of the species checked). Country of type locality: Bulgaria.

**Geographical distribution.**PAL.

**PAL**: Bulgaria.

**Notes.** We follow Papp (1986: 232), who based his species concept on the original description only, as he could not see any specimen.

***Cotesiagabera* Papp, 1990**

*Cotesiagabera* Papp, 1990.

**Type information.** Holotype female, HNHM (not examined but original description checked). Country of type locality: Korea.

**Geographical distribution.**PAL.

**PAL**: Korea.

***Cotesiagades* (Nixon, 1974)**

*Apantelesgades* Nixon, 1974.

**Type information.** Holotype female, NHMUK (examined). Country of type locality: Germany.

**Geographical distribution.**PAL.

**PAL**: Germany, Hungary, Macedonia, United Kingdom, Yugoslavia.

***Cotesiagastropachae* (Bouché, 1834)**

*Microgastergastropachae* Bouché, 1834.

**Type information.** Holotype male, ZMHB (not examined but subsequent treatment of the species checked). Country of type locality: Germany.

**Geographical distribution.**OTL, PAL.

**OTL**: China (ZJ); **PAL**: Azerbaijan, Bulgaria, China (SD, SN), Czech Republic, Finland, France, Germany, Hungary, Israel, Japan, Kazakhstan, Lithuania, Moldova, Mongolia, Montenegro, Poland, Romania, Russia (ZAB, KHA, KIR, KYA, ORE, PRI, SAK, SPE, VOR), Slovakia, Turkey, United Kingdom, Uzbekistan.

**Notes.** Our species concept is based on Nixon (1974) and Papp (1990a).

***Cotesiageometricae* Austin, 2000**

*Cotesiageometricae* Austin, 2000.

**Type information.** Holotype female, ANIC (not examined but original description checked). Country of type locality: Australia.

**Geographical distribution.**AUS.

**AUS**: Australia (ACT, VIC).

***Cotesiageryonis* (Marshall, 1885)**

*Apantelesgeryonis* Marshall, 1885.

**Type information.** Lectotype female, NHMUK (examined). Country of type locality: United Kingdom.

**Geographical distribution.**PAL.

**PAL**: Bulgaria, Germany, Hungary, Iran, Italy, Korea, Mongolia, Poland, Romania, Russia (PRI), Slovakia, Spain, Switzerland, Turkey, Ukraine, United Kingdom.

**Notes.** The presence of the species in Iran is based in Belokobylskij et al. (2019).

***Cotesiagillettei* (Baker, 1895)**

*Apantelesgillettei* Baker, 1895.

**Type information.** Holotype female, USNM (not examined but subsequent treatment of the species checked). Country of type locality: USA.

**Geographical distribution.**NEA.

**NEA**: USA (AZ, CO, NJ).

**Notes.** Our species concept is based on Muesebeck (1921), Mason (1981) and Whitfield (1995a).

***Cotesiaglabrata* (Telenga, 1955)**

*Apantelesglabratus* Telenga, 1955.

**Type information.** Lectotype female, ZIN (examined). Country of type locality: Russia.

**Geographical distribution.**PAL.

**PAL**: Bulgaria, Georgia, Germany, Hungary, Iran, Israel, Kazakhstan, Russia (DA, KDA, RYA, SPE, YAR), Turkey, Turkmenistan, Ukraine.

**Notes.** Our species concept is based on Shaw et al. (2009). The species distribution in Turkmenistan is based on Belokobylskij et al. (2019).

***Cotesiaglomerata* (Linnaeus, 1758)**

*Ichneumonglomeratus* Linnaeus, 1758.

*Ichneumonglomerator* Thunberg, 1822.

*Microgasternigriventris* Nees, 1834.

*Microgasterrecondita* Nees, 1834.

*Microgasterstellatarum* Bouché, 1834.

*Microgastercrataegi* Ratzeburg, 1844.

*Microgasteroleracea* Taylor, 1860.

*Microgasterpieridis* Packard, 1881 [homonym of *Microgasterpieridis* Bouché, 1834].

*Microgasterpieridivora* Riley, 1882.

*Apantelesaporiae* Ivanov, 1899.

*Glyptapantelesnawaii* Ashmead, 1906.

*Apantelesaporiae* Matsumura, 1908).

*Apantelesheterotergis* Fahringer, 1936.

**Type information.** Type unknown, NHRS (not examined but subsequent treatment of the species checked). Country of type locality: unclear.

**Geographical distribution.**AUS, NEA, NEO, OTL, PAL.

**AUS**: Australia (ACT, NSW, QLD), Fiji, Hawaiian Islands, New Zealand; **NEA**: Canada (BC, NB, ON, QC), USA (CA, CO, CT, DC, FL, IL, IA, LA, MD, MA, MI, MN, NH, NJ, NY, OR, PA, SC, VT, VA, WA, WI); **NEO**: Barbados, Brazil (SP), Chile, Uruguay; **OTL**: China (GZ, HN, JS, SH, SN, TW, ZJ), India, Pakistan, Vietnam; **PAL**: Armenia, Azerbaijan, Azores, Belarus, Belgium, Bulgaria, Canary Islands, China (BJ, HE, HA, JL, LN, NM, NX, SN, XJ), Croatia, Cyprus, Czech Republic, Denmark, Egypt, Estonia, Finland, France, Georgia, Germany, Hungary, Iran, Ireland, Israel, Italy, Japan, Jordan, Kazakhstan, Korea, Latvia, Lithuania, Macedonia, Malta, Moldova, Mongolia, Morocco, Netherlands, Poland, Portugal, Romania, Russia (AD, AST, BU, KGD, KAM, KHA, KIR, KDA, KRS, MOS, PRI, ROS, SAK, SPE, SAR, TAM, VGG, VLG, YAR), Serbia, Slovakia, Spain, Sweden, Switzerland, Syria, Turkey, Ukraine, United Kingdom, Uzbekistan.

**Notes.** Our species concept is based on Shaw et al. (2009). Information about type(s) status for this species was discussed by Shenefelt (1972) and Fitton (1978). The species distribution in Armenia, Georgia, Jordan, Kazakhstan and Mongolia is based on Belokobylskij et al. (2019).

***Cotesiagonopterygis* (Marshall, 1898)**

*Apantelesgonopterygis* Marshall, 1898.

**Type information.** Holotype female, PCMAG (not examined but subsequent treatment of the species checked). Country of type locality: United Kingdom.

**Geographical distribution.**PAL.

**PAL**: Germany, Hungary, Japan, Romania, Russia (C, S), Slovakia, Switzerland, Turkey, United Kingdom.

**Notes.** Our species concept is based on Shaw et al. (2009).

***Cotesiagordii* (Muesebeck, 1926)**

*Apantelesgordii* Muesebeck, 1926.

**Type information.** Holotype female, USNM (not examined but original description checked). Country of type locality: USA.

**Geographical distribution.**NEA.

**NEA**: USA (CT).

***Cotesiagramini* Sathe & Rokade, 2005**

*Cotesiagramini* Sathe & Rokade, 2005.

**Type information.** Holotype female, depository unknown (not examined). Country of type locality: India.

**Geographical distribution.**OTL.

**OTL**: India.

**Notes.** This species may not be valid as we suspect that no type depository was specified. However, because we could not read the original description to confirm that, we retain it as valid species for the time being.

***Cotesiagregalis* Yang & Wei, 2002**

*Cotesiagregalis* Yang & Wei, 2002.

**Type information.** Holotype female, CFRB (not examined but subsequent treatment of the species checked). Country of type locality: China.

**Geographical distribution.**PAL.

**PAL**: China (HE, LN, SD, TJ).

**Notes.** Our species concept is based on Zeng (2012).

***Cotesiagriffini* (Viereck, 1911)**

*Apantelesgriffini* Viereck, 1911.

**Type information.** Holotype female, USNM (examined). Country of type locality: USA.

**Geographical distribution.**NEA.

**NEA**: Canada (AB, NB, QC), USA (AR, KS, MA, NY, OK, SC, SD, TX, WA).

***Cotesiahadenae* (Muesebeck, 1926)**

*Apanteleshadenae* Muesebeck, 1926.

**Type information.** Holotype female, USNM (not examined but original description checked). Country of type locality: USA.

**Geographical distribution.**NEA.

**NEA**: USA (MI, NJ).

***Cotesiahalisidotae* (Muesebeck, 1931)**

*Apanteleshalisidotae* Muesebeck, 1931.

**Type information.** Holotype female, USNM (not examined but original description checked). Country of type locality: USA.

**Geographical distribution.**NEA.

**NEA**: Canada (BC, MB, ON, QC), USA (MA, NH, NY, VT).

***Cotesiahallii* (Packard, 1877)**

*Microgasterhallii* Packard, 1877.

**Type information.** Holotype male, ANSP (not examined but subsequent treatment of the species checked). Country of type locality: Greenland.

**Geographical distribution.**NEA.

**NEA**: Canada (NT, NU), Greenland.

**Notes.** Our species concept is based on van Achterberg (2006) and Fernandez-Triana et al. (2017b). Shenefelt (1972: 528) recorded the type as male but with a question mark.

***Cotesiahanshouensis* (You & Xiong, 1983)**

*Apanteleshanshouensis* You & Xiong, 1983.

**Type information.** Holotype female, HUNAU (not examined but subsequent treatment of the species checked). Country of type locality: China.

**Geographical distribution.**OTL.

**OTL**: China (HB, HN, JS, JX).

**Notes.** Our species concept is based on Chen and Song (2004).

***Cotesiaharteni* Papp, 2003**

*Cotesiaharteni* Papp, 2003.

**Type information.** Holotype female, HNHM (not examined but original description checked). Country of type locality: Cape Verde.

**Geographical distribution.**AFR.

**AFR**: Cape Verde.

***Cotesiahemileucae* (Riley, 1881)**

*Apanteleshemileucae* Riley, 1881.

**Type information.** Syntypes female and male, USNM (examined). Country of type locality: USA.

**Geographical distribution.**NEA.

**NEA**: Canada (NB, ON), USA (CT, FL, KS, MA, MN, MO, NY, OR).

***Cotesiahesperidivora* (Viereck, 1912)**

*Apanteleshesperidivorus* Viereck, 1912.

**Type information.** Holotype female, USNM (examined). Country of type locality: USA.

**Geographical distribution.**NEA.

**NEA**: USA (AZ, CA, CT, FL, MA).

***Cotesiahiberniae* (Kurdjumov, 1912), new combination**

*Apanteleshiberniae* Kurdjumov, 1912.

**Type information.** Syntypes female and male, depository unknown (not examined but subsequent treatment of the species checked). Country of type locality: Ukraine.

**Geographical distribution.**PAL.

**PAL**: Ukraine.

**Notes.** Our concept of this species is based on Fahringer (1936) and Telenga (1955). We are placing this species in *Cotesia* based on the shape of T1-T3, short ovipositor, and the placement those authors gave to this species in their respective papers (Fahringer placed it in the Wilkinson group F, which comprises mostly species of what is currently *Cotesia*; Telenga’s key had *hiberniae* within a large group of *Cotesia* as well).

***Cotesiahispanica* (Oltra & Falco, 1996)**

*Protapanteleshispanica* Oltra & Falco, 1996.

**Type information.** Holotype female, UVS (not examined but original description checked). Country of type locality: Spain.

**Geographical distribution.**PAL.

**PAL**: Spain.

**Notes.** The type material is probably in the Instituto Cavanilles de Biodiversitat y Biología Evolutiva, University of Valencia, Spain, but we have not been able to verify that information yet. Although the species was originally described in *Protapanteles*, Yu et al. (2016) treated it as *Cotesia*, a generic placement with agree with.

***Cotesiahonora* Papp, 1990**

*Cotesiahonora* Papp, 1990.

**Type information.** Holotype female, HNHM (not examined but original description checked). Country of type locality: Korea.

**Geographical distribution.**PAL.

**PAL**: Korea.

***Cotesiahyperion* (de Saeger, 1944), new combination**

*Apanteleshyperion* de Saeger, 1944.

**Type information.** Holotype female, RMCA (not examined but original description checked). Country of type locality: Democratic Republic of Congo.

**Geographical distribution.**AFR.

**AFR**: Democratic Republic of Congo, Rwanda.

**Notes.** Based on the details from the original description (de Saeger 1944), the best generic placement would be in *Cotesia*.

***Cotesiahyphantriae* (Riley, 1887)**

*Apanteleshyphantriae* Riley, 1887.

**Type information.** Holotype female, USNM (examined). Country of type locality: USA.

**Geographical distribution.**NEA, NEO, PAL.

**NEA**: Canada (BC, MB, NB, NS, ON, QC), USA (AR, CO, CT, DE, DC, LA, MD, MA, MI, MO, NJ, NM, NY, SC, TZ, VA, WV); **NEO**: Mexico; **PAL**: Bulgaria, Czech Republic, Germany, Greece, Hungary, Iran, Japan, Korea, Moldova, Netherlands, Poland, Romania, Russia (KDA), Serbia, Slovakia, Switzerland, Turkey, Ukraine, United Kingdom.

***Cotesiahypopygialis* (Granger, 1949), new combination**

*Apanteleshypopygialis* Granger, 1949.

**Type information.** Syntypes female and male, MNHN (not examined but original description checked). Country of type locality: Madagascar.

**Geographical distribution.**AFR.

**AFR**: Madagascar.

**Notes.** The details provided in the original description clearly show this species belongs in *Cotesia*.

***Cotesiahypsipylae* (Wilkinson, 1928), new combination**

*Apanteleshypsipylae* Wilkinson, 1928.

**Type information.** Holotype female, NHMUK (examined). Country of type locality: India.

**Geographical distribution.**OTL.

**OTL**: India.

**Notes.** Examination of the holotype reveals the species belongs in *Cotesia*, based on the propodeum with a complete median, longitudinal carina, partial transverse carina, shapes and sculptures of T1 and T2, inflexible hypopygium, and short ovipositor sheaths.

***Cotesiaicipe* Fernandez-Triana & Fiaboe, 2017**

*Cotesiaicipe* Fernandez-Triana & Fiaboe, 2017.

**Type information.** Holotype female, NMK (examined). Country of type locality: Kenya.

**Geographical distribution.**AFR, PAL.

**AFR**: Kenya, Madagascar, South Africa, Yemen. **PAL**: Saudi Arabia.

**Notes.** In the original description of the species, its distribution was recorded as only present in the Afrotropical region. However, in this paper we follow O’Hara et al. (2009) for boundaries of regions, and Saudi Arabia is considered to belong to the Palearctic, so the distribution above reflects that.

***Cotesiaindica* Sathe & Rokade, 2005**

*Cotesiaindica* Sathe & Rokade, 2005.

**Type information.** Holotype female, depository unknown (not examined). Country of type locality: India.

**Geographical distribution.**OTL.

**OTL**: India.

**Notes.** This species may not be valid as we suspect that no type depository was specified. However, because we could not read original description to confirm that, we consider it as valid species for the time being.

***Cotesiainducta* (Papp, 1973)**

*Apantelesinductus* Papp, 1973.

*Apantelestenuivalvis* Tobias, 1986.

**Type information.** Holotype female, HNHM (not examined but subsequent treatment of the species checked). Country of type locality: Hungary.

**Geographical distribution.**PAL.

**PAL**: Bulgaria, Hungary, Ireland, Israel, Korea, Moldova, Russia (KDA, PRI), Slovakia, Spain, Turkey, Ukraine, United Kingdom, Uzbekistan.

**Notes.** Our species concept is based on Shaw (2007).

***Cotesiaintermixta* (Balevski, 1980)**

*Apantelesintermixtus* Balevski, 1980.

**Type information.** Holotype female, ZIN (not examined but subsequent treatment of the species checked). Country of type locality: Bulgaria.

**Geographical distribution.**PAL.

**PAL**: Bulgaria.

**Notes.** We follow Papp (1986: 227) who based his species concept on the original description only, as he could not examine any specimens.

***Cotesiainvirae* Salgado-Neto & Whitfield, 2019**

*Cotesiainvirae* Salgado-Neto & Whitfield, 2019.

**Type information.** Holotype female, UFSM (not examined but original description checked). Country of type locality: Brazil.

**Geographical distribution.**NEO.

**NEO**: Brazil (RS).

***Cotesiaishizawai* (Watanabe, 1939)**

*Apantelesishizawai* Watanabe, 1939.

**Type information.** Syntypes female and male?, EIHU (examined). Country of type locality: Japan.

**Geographical distribution.**PAL.

**PAL**: Japan.

**Notes.** In the EIHU collection there are two specimens, one female and one male, both with red labels marked as Type. That suggests they are actually syntypes, but we could not read the original description from Watanabe to corroborate if indeed several specimens were part of the original description with none being designated as a holotype (i.e., they are indeed syntypes) or if a holotype was designated. For the time being, and based on the specimens and labels we examined, we consider the specimens to be syntypes.

***Cotesiaisolde* (Nixon, 1974)**

*Apantelesisolde* Nixon, 1974.

**Type information.** Holotype female, NHMUK (examined). Country of type locality: United Kingdom.

**Geographical distribution.**PAL.

**PAL**: Finland, Germany, Hungary, Poland, Slovakia, Switzerland, United Kingdom.

***Cotesiaitororensis* Sousa-Lopes & Whitfield, 2019**

*Cotesiaitororensis* Sousa-Lopes & Whitfield, 2019.

**Type information.** Holotype female, MZUSP (not examined but original description checked). Country of type locality: Brazil.

**Geographical distribution. NEO**.

**NEO**: Brazil (MG).

***Cotesiajayanagarensis* (Bhatnagar, 1950)**

*Apantelesjayanagarensis* Bhatnagar, 1950.

**Type information.** Holotype female, INPC (not examined but subsequent treatment of the species checked). Country of type locality: India.

**Geographical distribution.**OTL.

**OTL**: China (HN, SN, ZJ), India.

**Notes.** Our species concept is based on Chen and Song (2004). The year of publication of the Bhatnagar paper was until recently commonly cited as 1948 and/or 1950 (e.g., Chen and Song 2004, Yu et al. 2016), probably following Shenefelt (1972) who referred to this paper as “Bhatnagar (1948) 1950”. While the intended year for Volume X, Parts I & II of the Indian Journal of Entomology was 1948, the actual dates of publication were June 1950 (Part I) and October 1950 (Part II), as clearly shown on the cover page of the Volume, which we have checked. Because the dates of publication are the ones to be considered, and for the sake of clarity, we hereby revise the species year of description to 1950.

***Cotesiajucunda* (Marshall, 1885)**

*Apantelesjucundus* Marshall, 1885.

*Microgasternigrinervis* Thomson, 1895.

**Type information.** Holotype female, NHMUK (examined). Country of type locality: United Kingdom.

**Geographical distribution.**PAL.

**PAL**: Armenia, Austria, Azerbaijan, Bulgaria, Croatia, Denmark, Estonia, Finland, France, Germany, Greece, Hungary, Iran, Ireland, Moldova, Mongolia, Poland, Romania, Russia (PRI), Serbia, Slovakia, Spain, Sweden, Switzerland, Turkey, United Kingdom.

**Notes.** The species distribution in Azerbaijan is based on Belokobylskij et al. (2019).

***Cotesiajudaica* (Papp, 1970)**

*Apantelesjudaica* Papp, 1970.

*Apantelesdzhanybeki* Tobias, 1986.

**Type information.** Holotype female, BGM (not examined but original description checked). Country of type locality: Israel.

**Geographical distribution.**PAL.

**PAL**: Hungary, Israel, Italy, Kazakhstan, Russia (S), Tunisia, Ukraine.

**Notes.** The depository of the holotype was stated to be the “Beth Gordon Agriculture and Nature Study Institute, Deganya, Israel”. The depository acronym we provide here (BGM) is based on the way the museum is referred to in its Hebrew website as Beit Gordon Museum.

***Cotesiajujubae* (Wilkinson, 1929), new combination**

*Apantelesjujubae* Wilkinson, 1929.

**Type information.** Holotype female, NHMUK (examined). Country of type locality: India.

**Geographical distribution.**OTL.

**OTL**: India.

**Notes.** The holotype has very short ovipositor sheaths, inflexible hypopygium, T1 coarsely sculptured and widening posteriorly, and T2 more or less rectangular in shape and with similar sculpture to T1. All those features clearly indicate that this species belongs in *Cotesia*. The carination pattern on the propodeum is rather unusual for the genus, with a median longitudinal carina that is weakly defined anteriorly, transverse carina present and an areola that is partially defined by carinae. However, that pattern is present in other *Cotesia* species (see Gupta et al. 2016b for a discussion of examples and illustrations of other species with similar carination patterns).

***Cotesiajuniperatae* (Bouché, 1834)**

*Microgasterjuniperatae* Bouché, 1834.

**Type information.** Lectotype male, ZSM (not examined but original description checked). Country of type locality: Germany.

**Geographical distribution.**PAL.

**PAL**: United Kingdom.

**Notes.** In this paper we follow Broad et al. (2016) decision to consider *juniperatae* as a valid species, but see notes under *Cotesiabrachycera* for more details on the history and use of related names and species.

***Cotesiajunoniae* (Riley, 1889)**

*Apantelesjunoniae* Riley, 1889.

**Type information.** Holotype male, USNM (examined). Country of type locality: USA.

**Geographical distribution.**NEA.

**NEA**: USA (CT, KS, MO, NJ).

***Cotesiakamiyai* (Watanabe, 1934)**

*Apanteleskamiyai* Watanabe, 1934.

**Type information.** Syntypes female and male, EIHU (examined). Country of type locality: Japan.

**Geographical distribution.**OTL, PAL.

**OTL**: China (GZ, ZJ); **PAL**: Japan.

**Notes.** Despite previous references (e.g., Shenefelt 1972, Yu et al. 2016), the original description did not designate a holotype, and instead is based on a series of six female and two male specimens (Watanabe 1934: 134–135). We examined a male from the syntype series, in the Hokkaido collection.

***Cotesiakariyai* (Watanabe, 1937)**

*Apanteleskariyai* Watanabe, 1937.

*Apantelespurgata* (Telenga, 1955).

**Type information.** Syntypes female and male, EIHU (not examined but subsequent treatment of the species checked). Country of type locality: China.

**Geographical distribution.**PAL, OTL.

**OTL**: China (FJ, GX, GZ, HB, HN, JS, JX, SN, TW, YN, ZJ), Vietnam; **PAL**: China (AH, BJ, HL, HA, JL, LN, NX, SD, SN), Japan, Korea, Russia (PRI).

**Notes.** Our species concept is based on Papp (1986) and Chen and Song (2004). We also examined specimens from the EIHU collection.

***Cotesiakarviri* Sathe & Rokade, 2005**

*Cotesiakarviri* Sathe & Rokade, 2005.

**Type information.** Holotype female, depository unknown (not examined). Country of type locality: India.

**Geographical distribution.**OTL.

**OTL**: India.

**Notes.** This species may not be valid as we suspect that no type depository was specified. However, because we could not read the original description to confirm that, we retain it as valid species for the time being.

***Cotesiakasparyani* (Tobias, 1976)**

*Apanteleskasparyani* Tobias, 1976.

**Type information.** Holotype female, ZIN (not examined but subsequent treatment of the species checked). Country of type locality: Russia.

**Geographical distribution.**PAL.

**PAL**: Russia (KHA, PRI).

**Notes.** Our species concept is based on Papp (1987a) and Kotenko (2007a).

***Cotesiakazak* (Telenga, 1949)**

*Apanteleskazak* Telenga, 1949.

**Type information.** Lectotype female, ZIN (examined). Country of type locality: Tajikistan.

**Geographical distribution.**AUS, OTL, PAL.

**AUS**: Australia (VIC, WA), New Zealand; **OTL**: India; **PAL**: Armenia, Azerbaijan, Bulgaria, Croatia, Greece, Iran, Israel, Kazakhstan, Mongolia, Morocco, Portugal, Russia (ROS), Spain, Tajikistan, Tunisia, Turkey, Turkmenistan, Uzbekistan.

**Notes.** Our species concept is based on Telenga (1955) and Papp (1986, 1987).

***Cotesiakhuzestanensis* Zargar & Gupta, 2019**

*Cotesiakhuzestanensis* Zargar & Gupta, 2019.

**Type information.** Holotype female, NBAIR (not examined but original description checked). Country of type locality: Iran.

**Geographical distribution.**PAL.

**PAL**: Iran.

***Cotesiakoebelei* (Riley, 1889)**

*Apanteleskoebelei* Riley, 1889.

**Type information.** Syntypes female, USNM (examined). Country of type locality: USA.

**Geographical distribution.**NEA.

**NEA**: Canada (BC), USA (CA, NE).

**Notes.** One of the syntypes is missing the head and metasoma, thus is impossible to know its sex with certainty. All other syntypes are female specimens.

***Cotesiakraussi* (Muesebeck, 1958)**

*Apanteleskraussi* Muesebeck, 1958.

**Type information.** Holotype female, USNM (not examined but original description checked). Country of type locality: Mexico.

**Geographical distribution.**NEO.

**NEO**: Mexico.

***Cotesiakurdjumovi* (Telenga, 1955)**

*Apanteleskurdjumovi* Telenga, 1955.

*Apanteleslaverna* Nixon, 1974.

**Type information.** Lectotype female, ZIN (not examined but authoritatively identified specimens examined). Country of type locality: Ukraine.

**Geographical distribution.**PAL.

**PAL**: Bulgaria, Germany, Hungary, Israel, Lithuania, Moldova, Mongolia, Montenegro, Russia (C, S), Spain, Turkey, Turkmenistan, Ukraine, United Kingdom.

**Notes.** We examined the type of *Apanteleslaverna* Nixon.

***Cotesialaeviceps* (Ashmead, 1890)**

*Apanteleslaeviceps* Ashmead, 1890.

*Apantelesleviceps* Dalla Torre, 1898 [unjustified emendation].

**Type information.** Holotype female, USNM (examined). Country of type locality: USA.

**Geographical distribution.**NEA.

**NEA**: Canada (AB, BC, MB, NB, ON, QC, SK), USA (CA, CO, CT, GA, IL, IA, MO, NM, NY, UT).

**Notes.** The holotype is in poor condition, the only wing remaining is the right hind wing, antennae are missing, and the hind legs are embedded in glue.

***Cotesialangei* (Muesebeck, 1938)**

*Apanteleslangei* Muesebeck, 1938.

**Type information.** Holotype female, USNM (not examined but original description checked). Country of type locality: USA.

**Geographical distribution.**NEA.

**NEA**: USA (CA).

***Cotesialepidopteri* Sathe & Rokade, 2005**

*Cotesialepidopteri* Sathe & Rokade, 2005.

**Type information.** Holotype female, depository unknown (not examined). Country of type locality: India.

**Geographical distribution.**OTL.

**OTL**: India.

**Notes.** This species may not be valid as we suspect that no type depository was specified. However, because we could not read the original description to confirm that, we retain it as valid species for the time being.

***Cotesialesbiae* (Blanchard, 1947), new combination**

*Apanteleslesbiae* Blanchard, 1947.

*Apantelesgrioti* Blanchard, 1943 [*nomen nudum*].

**Type information.** Holotype female, MACN (not examined but original description checked). Country of type locality: Argentina.

**Geographical distribution.**NEO.

**NEO**: Argentina.

**Notes.** The generic placement of the species within *Cotesia* is clear from the original description and the accompanying drawings.

***Cotesialevigaster* (Granger, 1949), new combination**

*Apanteleslevigaster* Granger, 1949.

**Type information.** Syntypes female and male, MNHN (not examined but original description checked). Country of type locality: Madagascar.

**Geographical distribution.**AFR.

**AFR**: Madagascar.

**Notes.** The original description (which includes a drawing of T1 and T2) strongly suggests that this species does not belong to *Apanteles*. Based on the described shape of T1-T2, hypopygium, length of ovipositor sheaths, recorded hosts, and (to a lesser extent) propodeum sculpture, the best generic placement at present would be *Cotesia*.

***Cotesialimbata* (Marshall, 1885)**

*Apanteleslimbatus* Marshall, 1885.

*Apanteleskawadai* Watanabe, 1934.

**Type information.** Syntypes females, NHMUK (examined). Country of type locality: United Kingdom.

**Geographical distribution.**PAL.

**PAL**: Bulgaria, Croatia, France, Germany, Hungary, Japan, Moldova, Mongolia, Poland, Romania, Russia (AD, MOS), Serbia, Slovakia, Switzerland, United Kingdom.

**Notes.**Shenefelt (1972: 554) recorded the syntype specimens as female only.

***Cotesialimenitidis* (Riley, 1871)**

*Microgasterlimenitidis* Riley, 1871.

**Type information.** Syntypes female and male, USNM (examined). Country of type locality: USA.

**Geographical distribution.**NEA.

**NEA**: Canada (NS, ON), USA (CT, IL, IA, MD, MA, MO, NJ, NY, OH, PA, UT).

***Cotesialineola* (Curtis, 1830)**

*Microgasterlineola* Curtis, 1830.

*Microgasterlineola* Curtis, 1829. [*nomen nudum*].

*Apantelesgabrielis* Gautier & Riel, 1919.

**Type information.** Syntypes female and male, MVMMA (not examined but subsequent treatment of the species checked). Country of type locality: United Kingdom.

**Geographical distribution.**PAL.

**PAL**: Armenia, Bulgaria, Czech Republic, Finland, France, Germany, Hungary, Latvia, Moldova, Romania, Russia (SPE), Spain, Turkey, United Kingdom.

**Notes.** Our species concept is based on Wilkinson (1945), Nixon (1974), and Papp (1987a).

***Cotesializeri* (Blanchard, 1947), new combination**

*Apanteleslizeri* Blanchard, 1947.

**Type information.** Holotype female, MACN (not examined but original description checked). Country of type locality: Argentina.

**Geographical distribution.**NEO.

**NEO**: Argentina.

**Notes.** From the original description and accompanying drawings, it is clear that this species is not an *Apanteles*. There is a small chance it could be placed within *Parapanteles* (examination of the specimens will be needed in the future to conclude); however, we consider that it is much more likely the species belongs in *Cotesia*, based on the short ovipositor sheaths, the propodeum carination and the available host data. An indirect support (but far from conclusive) for this species being placed in *Cotesia* is that all of its reported hosts are moths of the family Lasiocampidae (see Yu et al. 2016 for references). There is only one *Parapanteles* species recorded as a parasitoid of Lasiocampidae (Valerio et al. 2009) whereas there are numerous records of *Cotesia* parasitizing that family (Yu et al. 2016).

***Cotesialuminata* Chen & Song, 2004**

*Cotesialuminata* Chen & Song, 2004.

**Type information.** Holotype female, FAFU (not examined but original description checked). Country of type locality: China.

**Geographical distribution.**PAL.

**PAL**: China (XZ).

***Cotesialunata* (Packard, 1881)**

*Microgasterlunatus* Packard, 1881.

**Type information.** Neotype male, EIHU (not examined but subsequent treatment of the species checked). Country of type locality: USA.

**Geographical distribution.**NEA.

**NEA**: Canada (QC), USA (CA, CT, FL, GA, IL, IA, KS, MA, MO, NJ, NY, NC, TX, WA).

**Notes.** Our species concept is based on Muesebeck (1921), Mason (1981), Whitfield (1995a) and Fernandez-Triana (2010).

***Cotesialyciae* (Muesebeck, 1926)**

*Apanteleslyciae* Muesebeck, 1926.

**Type information.** Holotype female, USNM (not examined but original description checked). Country of type locality: USA.

**Geographical distribution.**NEA.

**NEA**: Canada (QC), USA.

***Cotesialycophron* (Nixon, 1974)**

*Apanteleslycophron* Nixon, 1974.

**Type information.** Holotype female, NHMUK (examined). Country of type locality: France.

**Geographical distribution.**PAL.

**PAL**: France, Hungary, Israel, Netherlands.

***Cotesiamahoniae* (Mason, 1975)**

*Apantelesmahoniae* Mason, 1975.

**Type information.** Holotype female, CNC (examined). Country of type locality: Canada.

**Geographical distribution.**NEA.

**NEA**: Canada (BC), USA (ID).

***Cotesiamalevola* (Wilkinson, 1929), new combination**

*Apantelesmalevolus* Wilkinson, 1929.

**Type information.** Holotype female, NHMUK (examined). Country of type locality: India.

**Geographical distribution.**OTL.

**OTL**: India, Myanmar.

**Notes.** This species belongs in *Cotesia*. It has very short ovipositor sheaths, inflexible hypopygium, strongly sculptured T1 and T2, and propodeum with both longitudinal and transverse carinae.

***Cotesiamalshri* (Sathe & Inamdar, 1991), new combination**

*Glyptapantelesmalshri* Sathe & Inamdar, 1991.

**Type information.** Holotype female, SUKI (not examined but original description checked). Country of type locality: India.

**Geographical distribution.**OTL.

**OTL**: India.

**Notes.** Based on the original description and drawings included there, it is very clear that this species does not belong to *Glyptapanteles*. Without examining the type material it is difficult to conclude, but the best generic placement at present would be *Cotesia*, based on the sculpture of the propodeum, T1–T3 shape and hypopygium and ovipositor.

***Cotesiamarginiventris* (Cresson, 1865)**

*Microgastermarginiventris* Cresson, 1865.

*Apantelesgrenadensis* Ashmead, 1900.

*Apanteleslaphygmae* Ashmead, 1901 [*nomen nudum*].

*Apantelesharnedi* Viereck, 1912.

**Type information.** Holotype male, ANSP (not examined but authoritatively identified specimens examined). Country of type locality: Cuba.

**Geographical distribution.**AFR, AUS, NEA, NEO, OTL, PAL.

**AFR**: Cape Verde; **AUS**: Fiji, Hawaiian Islands; **NEA**: USA (AL, AZ, AR, CA, DE, FL, GA, IL, IN, IA, KS, KY, LA, MD, MS, MO, NC, OH, OK, SC, TN, TX, VA, WI); **NEO**: Argentina, Bermuda, Brazil (PR, RS, SC), Chile, Cuba, Grenada, Honduras, Mexico, Nicaragua, Peru, Puerto Rico, Suriname, Uruguay, Venezuela; **OTL**: India; **PAL**: Spain.

**Notes.** We have examined the types of *Apantelesgrenadensis* (Ashmead, 1900) (in the NHMUK), and *Apantelesharnedi* (Viereck, 1912) (in the USNM), as well as numerous specimens from several collections. There is considerable morphological variation, as well as wide host data; we suspect that *Cotesiamarginiventris* is likely to be a complex of cryptic species.

***Cotesiamayaguezensis* (Viereck, 1913)**

*Apantelesmayaguezensis* Viereck, 1913.

**Type information.** Holotype female, USNM (examined). Country of type locality: Puerto Rico.

**Geographical distribution.**NEO.

**NEO**: Puerto Rico.

***Cotesiamedicaginis* (Muesebeck, 1947)**

*Apantelesmedicaginis* Muesebeck, 1947.

**Type information.** Holotype female, USNM (not examined but original description checked). Country of type locality: USA.

**Geographical distribution.**NEA.

**NEA**: USA (AZ, CA, ID, KS, MO, NV, NM, OK).

***Cotesiameghrangini* Dawale, Bhosale & Sathe, 1993**

*Cotesiameghrangini* Dawale, Bhosale & Sathe, 1993.

**Type information.** Holotype female, NZSI (not examined but original description checked). Country of type locality: India.

**Geographical distribution.**OTL.

**OTL**: India.

***Cotesiamelanoscelus* (Ratzeburg, 1844)**

*Microgastermelanoscelus* Ratzeburg, 1844.

*Microgastersolitarius* Ratzeburg, 1844.

*Apantelescreata* Balevski, 1980.

**Type information.** Type and depository unknown (not examined but subsequent treatment of the species checked). Country of type locality: Germany.

**Geographical distribution.**NEA, OTL, PAL.

**NEA**: Canada (BC, NB, NL, NS, ON, PE, QC), USA (CT, MD, MA, NH, NJ, NY, OR, PA, RI, VT, VA, WA, WV); **OTL**: China (FJ, HB, HN), India; **PAL**: Armenia, Azerbaijan, Austria, Belarus, Belgium, Bulgaria, China (BJ, HL, JL, LN), Czech Republic, Denmark, Finland, France, Germany, Hungary, Iran, Italy, Japan, Kazakhstan, Korea, Latvia, Lithuania, Moldova, Mongolia, Netherlands, Poland, Portugal, Romania, Russia (ALT, AMU, ZAB, KGD, KHA, KIR, KDA, PRI, YAR), Serbia, Slovakia, Spain, Sweden, Switzerland, Tajikistan, Turkey, Ukraine, United Kingdom.

**Notes.** Our species concept is based on Muesebeck (1921), Nixon (1974), Papp (1987a), and Chen and Song (2004). Following Article 31.2.1 of the ICZN the name is a noun in apposition and the original spelling *melanoscelus* must be retained. The species distribution in Armenia and Azerbaijan is based on Belokobylskij et al. (2019).

***Cotesiamelitaearum* (Wilkinson, 1937)**

*Apantelesmelitaearum* Wilkinson, 1937.

*Apantelesmelittaearum* Wilkinson, 1937 [subsequent misspelling (Nixon 1974)].

**Type information.** Holotype female, NHMUK (examined). Country of type locality: United Kingdom.

**Geographical distribution.**PAL.

**PAL**: Armenia, Azerbaijan, China (BJ), Estonia, Finland, France, Germany, Hungary, Ireland, Italy, Kazakhstan, Korea, Moldova, Poland, Romania, Russia (BU, ZAB, PRI), Slovakia, Spain, Sweden, Turkey, United Kingdom, Uzbekistan.

**Notes.** The species distribution in Armenia is based on Belokobylskij et al. (2019).

***Cotesiamendicae* (Tobias, 1986)**

*Apantelesmendicae* Tobias, 1986.

**Type information.** Holotype female, ZIN (not examined but paratype examined). Country of type locality: Russia.

**Geographical distribution.**PAL.

**PAL**: Kazakhstan, Russia (VOR).

**Notes.** We examined one female paratype from the same cocoon mass than the (unexamined by us) holotype.

***Cotesiamenezesi* (de Santis & Redolfi, 1976), new combination**

*Apantelesmenezesi* de Santis & Redolfi, 1976.

**Type information.** Holotype female, MLP (not examined but subsequent treatment of the species checked). Country of type locality: Brazil.

**Geographical distribution.**NEO.

**NEO**:Brazil (SP).

**Notes.** Transferred to *Cotesia* based on the following details, all mentioned in the original description: propodeum with median longitudinal carina; T1 barrel-shaped (described as wide but slightly narrowing at anterior and posterior margins); T2 twice as wide as long; short ovipositor sheaths; and species being considered as close to *Apantelesschini* Muesebeck, 1958 (currently placed in *Cotesia*). Yu et al. (2016) referred to the second author of the species as del Carmen Redolfi but the last name should be just Redolfi, as correctly stated by Aquino et al. (2010).

***Cotesiamicrosomus* (Tobias, 1986)**

*Apantelesmicrosomus* Tobias, 1986.

**Type information.** Holotype female, ZIN (not examined but subsequent treatment of the species checked). Country of type locality: Belarus.

**Geographical distribution.**PAL.

**PAL**: Belarus.

**Notes.** Our species concept is based on Tobias (1986) and Papp (1988, 1990). Following Article 31.2.1 of the ICZN the name is a noun phrase in apposition and the original spelling *microsomus* is retained.

***Cotesiamiyoshii* (Watanabe, 1932)**

*Apantelesmiyoshii* Watanabe, 1932.

*Apantelessmerinthii* Matsumura, 1925 [primary homonym of *Apantelessmerinthii* Riley, 1881].

**Type information.** Syntypes female and male, EIHU (examined). Country of type locality: Japan.

**Geographical distribution.**OTL, PAL.

**OTL**: China (HB, JS, SN, ZJ); **PAL**: China (BJ, LN, SD), Japan, Korea.

***Cotesiamurtfeldtae* (Ashmead, 1898)**

*Apantelesmurtfeldtae* Ashmead, 1898.

**Type information.** Holotype female, USNM (not examined but subsequent treatment of the species checked). Country of type locality: USA.

**Geographical distribution.**NEA.

**NEA**: Canada (MB, ON, QC), USA (CT, MD, MA, MO, NH, NY, NC, VT, VA, WV).

**Notes.** Our species concept is based on Muesebeck (1921) and Papp (1987a).

***Cotesiamuzaffarensis* (Lal, 1939), new combination**

*Apantelesmuzaffarensis* Lal, 1939.

**Type information.** Holotype male, INPC (not examined but original description checked). Country of type locality: India.

**Geographical distribution.**OTL.

**OTL**: India.

**Notes.** The original description, although very brief, provides enough information to unequivocally place this species in *Cotesia*. The propodeum has a complete median longitudinal carina, the ovipositor sheaths are short, and the shape and sculpture of T1–T2 (as illustrated in a dorsal habitus provided in the original description) are all typical of this genus. Additionally, Lal (1939: 53) mentioned that *muzaffarensis* is morphologically close to *Cotesiasalebrosa*.

***Cotesianemoriae* (Ashmead, 1898)**

*Apantelesnemoriae* Ashmead, 1898.

*Apanteleswinkleyi* Viereck, 1917.

**Type information.** Holotype female, USNM (not examined but subsequent treatment of the species checked). Country of type locality: USA.

**Geographical distribution.**NEA.

**NEA**: Canada (MB, NL, NS, ON, QC, SK), USA (CA, CT, DC, KS, MA, MO, NH, NY, TN).

**Notes.** Our species concept is based on Muesebeck (1921) and Fernandez-Triana (2010). We also examined the female type of *Apanteleswinkleyi* (Viereck, 1917).

***Cotesianeptisis* (Watanabe, 1934), new combination**

*Apantelesneptisis* Watanabe, 1934.

**Type information.** Lectotype female, EIHU (examined). Country of type locality: Japan.

**Geographical distribution.**PAL.

**PAL**: Japan.

**Notes.** From Shenefelt (1972: 579), it could be implied that a lectotype for this species was designated by him; however, no details were provided as to which specimen should be considered as the lectotype. After we examined the available specimens, we believe that the lectotype specimen is a female missing its head and two legs (one fore and one mid leg). We also examined one female and one male specimen from the same locality (Sapporo), as well as a cocoon mass with the same label as those specimens (which presumably belongs to this species). Kotenko (2007a) had transferred this species from *Apanteles* to *Protapanteles*, but we found that the propodeum sculpture rather clearly indicates that this species is better placed in *Cotesia* than *Protapanteles* (although T1 and the central part of T2 are rather smooth, they are in line with other *Cotesia* with relatively smooth sculpture). Also, Kotenko (2007a) mentioned the species from Russia but that seems to be a mistake; thus, here we consider the species to be restricted to Japan only.

***Cotesianeustriae* (Tobias, 1986)**

*Apantelesneustriae* Tobias, 1986.

**Type information.** Holotype female, ZIN (not examined but paratype examined). Country of type locality: Moldova.

**Geographical distribution.**PAL.

**PAL**: Kazakhstan, Moldova, Russia (KDA, RYA, SAR, VGG, VOR), Turkey, Ukraine.

**Notes.** Our species concept is based on examined female paratypes, with the same host and locality data than the holotype – except for the collecting date being the following year compared to the holotype.

***Cotesianigritibialis* (Tobias, 1986)**

*Apantelesnigritibialis* Tobias, 1986.

**Type information.** Holotype female, ZIN (not examined but original description checked). Country of type locality: Russia.

**Geographical distribution.**PAL.

**PAL**: Hungary, Korea, Russia (KDA).

**Notes.** Our species concept is based on Tobias (1986) and Papp (1988, 1990).

***Cotesianikami* Kurhade & Nikam, 1998**

*Cotesianikami* Kurhade & Nikam, 1998.

**Type information.** Holotype female, BAMU (not examined). Country of type locality: India.

**Geographical distribution.**OTL.

**OTL**: India.

***Cotesianitens* (Muesebeck, 1921)**

*Apantelesnitens* Muesebeck, 1921.

**Type information.** Holotype female, USNM (examined). Country of type locality: USA.

**Geographical distribution.**NEA.

**NEA**: USA (CA, CO, OR, UT, WY).

***Cotesianoctuidiphagus* (Muesebeck, 1926)**

*Apantelesnoctuidiphagus* Muesebeck, 1926.

**Type information.** Holotype female, USNM (examined). Country of type locality: USA.

**Geographical distribution.**NEA.

**NEA**: USA (CT).

**Notes.** Following Article 31.2.1 of the ICZN the name is a noun in apposition and the original spelling *noctuidiphagus* must be retained.

***Cotesianonagriae* (Olliff, 1893)**

*Apantelesnonagriae* Olliff, 1893.

Apanteles (Stenopleura) nonagriae Viereck, 1913 [primary homonym of *Apantelesnonagriae* Olliff, 1893].

*Cotesiaparthenayae* Kittel, 2016 [unnecessary replacement name for *Cotesianonagriae* (Viereck, 1913)].

**Type information.** Syntypes female and male, depository unknown (not examined but subsequent treatment of the species checked). Country of type locality: Australia.

**Geographical distribution.**AUS.

**AUS**: Australia (NSW, QLD).

**Notes.**Wilkinson (1928b: 136) mentioned that this species was described based on female and male specimens, which was also corroborated by Muirhead et al. (2008: 38–43). However, recent efforts by Muirhead et al. (2008) could not locate those specimens in Australian collections and it is likely that the syntypes are lost (or the depository remains unknown at present). In the literature there are two species named as *Apantelesnonagriae*, the oldest one (Olliff 1893) applies to this species, whereas the youngest one (Viereck 1913) has long being considered as a synonym of *Cotesiaflavipes*Cameron 1891. Because Viereck’s name is not related to Olliff’s the replacement name proposed by Kittel (2016) is unnecessary.

***Cotesianothus* (Marshall, 1885)**

*Apantelesnothus* Marshall, 1885.

**Type information.** Lectotype female, NHMUK (examined). Country of type locality: United Kingdom.

**Geographical distribution.**PAL.

**PAL**: Germany, Greece, Hungary, Iran, Italy, Korea, Mongolia, Romania, Russia (SPE), Slovakia, Turkey, United Kingdom.

**Notes.** Following Article 31.2.2 of the ICZN, in the absence of an original statement that the epithet is adjectival, the name is to be treated as a noun in apposition and the original spelling *nothus* must be retained (Doug Yanega, pers. comm.). The species distribution in Iran is based on Belokobylskij et al. (2019).

***Cotesianuellorum* Whitfield, 2018**

*Cotesianuellorum* Whitfield, 2018.

**Type information.** Holotype female, USNM (not examined but original description checked). Country of type locality: USA.

**Geographical distribution.**NEA.

**NEA**: TX.

***Cotesianumen* (Nixon, 1974)**

*Apantelesnumen* Nixon, 1974.

**Type information.** Holotype female, NHMUK (examined). Country of type locality: United Kingdom.

**Geographical distribution.**PAL.

**PAL**: Czech Republic, Denmark, France, Germany, Hungary, Mongolia, Slovakia, Turkey, United Kingdom.

***Cotesianycteus* (de Saeger, 1944), new combination**

*Apantelesnycteus* de Saeger, 1944.

**Type information.** Holotype female, RMCA (not examined but original description checked). Country of type locality: Democratic Republic of Congo.

**Geographical distribution.**AFR.

**AFR**: Democratic Republic of Congo, Rwanda.

**Notes.** Based on the details provided in the original description (de Saeger 1944), the best generic placement would be in *Cotesia*. Following Article 31.2.1 of the ICZN, the name is a noun in apposition and the original spelling *nycteus* must be retained.

***Cotesiaobscuricornis* (Viereck, 1917)**

*Apantelesobscuricornis* Viereck, 1917.

**Type information.** Holotype female, USNM (examined). Country of type locality: USA.

**Geographical distribution.**NEA.

**NEA**: USA (CT, MD).

**Notes.** This species is rather similar to *C.marginiventris* (and other partially yellowish *Cotesia*) and may be part of a species complex.

***Cotesiaocneriae* (Ivanov, 1899)**

*Apantelesocneriae* Ivanov, 1899.

**Type information.** Type and depository unknown (not examined but subsequent treatment of the species checked). Country of type locality: Ukraine.

**Geographical distribution.**PAL.

**PAL**: Austria, Hungary, Moldova, Poland, Romania, Russia (S), Serbia, Ukraine.

**Notes.** Our species concept is based on Telenga (1955), Tobias (1986) and Papp (1987a, 1990). The information about the type is taken from Shenefelt (1972: 585).

***Cotesiaoeceticola* (Blanchard, 1935), new combination**

*Apantelesoeceticola* Blanchard, 1935.

**Type information.** Holotype female, MACN (not examined but original description checked). Country of type locality: Argentina.

**Geographical distribution.**NEO.

**NEO**: Argentina.

**Notes.** This species was described based on specimens from the Blanchard collection, which we believe is currently deposited in the MACN. The original description and illustrations (scutellar disc, propodeum, T1–T2, part of fore wing, tip of antenna), strongly suggest that this species belongs in *Cotesia*. The species has the propodeum with a median, longitudinal carina in addition to a more or less complete transverse carina, T1 and T2 are sculptured, T1 slightly widens towards the posterior margin, T2 is sub-rectangular, and the ovipositor sheaths barely protrude.

***Cotesiaofella* (Nixon, 1974)**

*Apantelesofella* Nixon, 1974.

? *Microgasterperspicuus* Nees, 1834.

**Type information.** Holotype female, NHMUK (examined). Country of type locality: United Kingdom.

**Geographical distribution.**PAL.

**PAL**: Belgium, Finland, Germany, Hungary, Iran, Israel, Italy, Netherlands, Poland, Serbia, Slovakia, Spain, Switzerland, Turkey, Ukraine, United Kingdom.

**Notes.** The name *perspicuus* (Nees, 1834) has sometimes been regarded as a senior synonym of both *cajae* (Bouché, 1834) and *ofella* (Nixon, 1974) (e.g., Yu et al. 2012, 2016). However, Marshall (1885: 183) had listed *cajae* as the senior name; and Papp (1988: 155) had tentatively suggested that *perspicuus* might be a synonym of *ofella* (Nixon, 1974), though without elaborating further. There has been little consensus on the correct application of the Nees name, the type of which is lost (see also comments under *Cotesiacajae* above). The arrangement proposed by Papp (1988), i.e., maintaining both *cajae* and *ofella* as valid species (and not as synonyms of *perspicuus*) has been subsequently followed by Papp (2005) and Broad et al. (2016), and it is also followed here.

***Cotesiaogara* Papp, 1990**

*Cotesiaogara* Papp, 1990.

**Type information.** Holotype female, HNHM (not examined but original description checked). Country of type locality: Korea.

**Geographical distribution.**PAL.

**PAL**: Korea.

***Cotesiaokamotoi* (Watanabe, 1921), status revised**

*Apantelesokamotoi* Watanabe, 1921.

**Type information.** Holotype male, EIHU (examined). Country of type locality: Japan.

**Geographical distribution.**PAL.

**PAL**: Japan.

**Notes.** This species was synonymized under *Cotesiaaffinis* (Nees, 1834) by Papp (1987a), who wrote that synonymy was based on examination and comparison of authenticated specimens as well as original descriptions. However, one female of *okamotoi* (from the EIHU collection) that we have examined (which is placed together with the male holotype and another male, the three specimens collected in the same locality and with same label data) is clearly not *affinis*, and differs in practically all characters mentioned in Papp’s work, e.g., the length/width of T1 and T2 is different (T1 width at posterior margin is actually larger than T1 length, and T2 is 3.0 x as wide at posterior margin as long), T3 is not longer than T2 but both are of the same length (although in males T3 is longer), and vein r arises from the pterostigma closer to the end. The Japanese specimens have yellow T2–T6. Based on all of the above, we resurrect this species from synonymy with *C.affinis* and consider *okamotoi* to be a valid species. *Cotesiaokamotoi* looks similar to *C.miyoshii*, as stated in the comments for both species by Watanabe (1932); however, the differences in T1–T3 shape and sculpture are diagnostic, as well as different tegula colour, and in addition they parasitize different hosts and are found in different habitats.

***Cotesiaolenidis* (Muesebeck, 1922)**

*Apantelesolenidis* Muesebeck, 1922.

**Type information.** Holotype female, USNM (examined). Country of type locality: Canada.

**Geographical distribution.**NEA.

**NEA**: Canada (BC).

***Cotesiaonaspis* (Nixon, 1974)**

*Apantelesonaspis* Nixon, 1974.

*Apantelesavetyanae* Tobias, 1976.

**Type information.** Holotype female, NHMUK (examined). Country of type locality: United Kingdom.

**Geographical distribution.**PAL.

**PAL**: Armenia, Finland, Hungary, Slovakia, United Kingdom.

***Cotesiaoppidicola* (Granger, 1949), new combination**

*Apantelesoppidicola* Granger, 1949.

**Type information.** Syntypes female and male, MNHN (not examined but original description checked). Country of type locality: Madagascar.

**Geographical distribution.**AFR.

**AFR**: Madagascar.

**Notes.** Based on the details provided in the original description, we consider that the best generic placement at present would be in *Cotesia*, but further examination of the type series will be needed in the future.

***Cotesiaopsiphanis* (Schrottky, 1909), new combination**

*Apantelesopsiphanis* Schrottky, 1909.

**Type information.** Type and depository unknown (not examined but original description checked). Country of type locality: Paraguay.

**Geographical distribution.**NEO.

**NEO**: Ecuador, Paraguay.

**Notes.** Here we transfer the species to *Cotesia* based on the original description, which mentions a coarsely rugose propodeum, T1 and T2, as well as short ovipositor sheaths (Schrottky 1909: 211). Those morphological details, as well as images and details of the wasp cocoon mass (Waterston 1923) and the record of two Nymphalidae as hosts (Schrottky 1909, Waterston 1923, Malo and Willis 1961), strongly indicate that the best generic placement of the species at present is in *Cotesia*. However, examination of specimens will be needed in the future.

***Cotesiaordinaria* (Ratzeburg, 1844)**

*Microgasterordinarius* Ratzeburg, 1844.

*Apantelesdendrolimi* Matsumura, 1926.

*Apantelesdendrolimusi* Matsumura, 1926.

**Type information.** Syntypes female and male, depository unknown (not examined but subsequent treatment of the species checked). Country of type locality: Germany.

**Geographical distribution.**OTL, PAL.

**OTL**: China (GX, HN, JS, ZJ); **PAL**: China (HL, JL, LN, SN), Czech Republic, Germany, Hungary, Iran, Israel, Italy, Japan, Korea, Mongolia, Poland, Romania, Russia (AMU, IRK, KYA, PRI, SAK, TOM, TY, YAR), Turkey, Ukraine.

**Notes.** Our species concept is based on Wilkinson (1945), Nixon (1974) and Papp (1987a). Wilkinson (1945: 71-72) wrote about the type series, which he examined, at the time deposited in the Forestry College of Eberswalde (Forstlichen Hochschule Eberswalde). Unfortunately, that collection was mostly destroyed during the Second World War; however, five drawers with Hymenoptera specimens, among them type species of Ratzeburg were spared and are now safe at the Senckenberg Deutsches Entomologisches Institut (SDEI) in Müncheberg, Germany (Schulz et al. 2018: 285-286). We do not know if the syntype specimens of *Microgasterordinarius* Ratzeburg are at present in Müncheberg. The species distribution in Iran is based on Belokobylskij et al. (2019).

***Cotesiaorestes* (Nixon, 1974)**

*Apantelesorestes* Nixon, 1974.

**Type information.** Holotype female, NHMUK (examined). Country of type locality: United Kingdom.

**Geographical distribution.**PAL.

**PAL**: Finland, Germany, Hungary, Korea, Netherlands, Russia (TVE), Turkey, United Kingdom.

***Cotesiaorientalis* Chalikwar & Nikam, 1984**

*Cotesiaorientalis* Chalikwar & Nikam, 1984.

**Type information.** Holotype female, NZSI (not examined but original description checked). Country of type locality: India.

**Geographical distribution.**OTL.

**OTL**: India.

***Cotesiaornatricis* (Muesebeck, 1958)**

*Apantelesornatricis* Muesebeck, 1958.

**Type information.** Holotype female, USNM (examined). Country of type locality: Colombia.

**Geographical distribution.**NEO.

**NEO**: Brazil (SP), Colombia.

***Cotesiaorobenae* (Forbes, 1883)**

*Apantelesorobenae* Forbes, 1883.

**Type information.** Lectotype female, INHS (not examined but subsequent treatment of the species checked). Country of type locality: USA.

**Geographical distribution.**NEA.

**NEA**: USA (AZ, CA, DC, FL, IL, LA, SC, VA).

**Notes.** Our species concept is based on Muesebeck (1921) and Papp (1987a).

***Cotesiapachkuriae* (Bhatnagar, 1950), new combination**

*Apantelespachkuriae* Bhatnagar, 1950.

**Type information.** Holotype female, INPC (not examined but original description checked). Country of type locality: India.

**Geographical distribution.**OTL.

**OTL**: India.

**Notes.** Transferred to *Cotesia* based on the propodeum with strong, median longitudinal carina and a transverse carina (near anterior), T1 sculptured and widening towards posterior margin, T2 slightly sculptured and around same length as T3 (Bhatnagar 1950). The year of publication of the Bhatnagar paper was until recently commonly cited as 1948 and/or 1950 (e.g., Chen and Song 2004, Yu et al. 2016), probably following Shenefelt (1972) who referred to this paper as “Bhatnagar (1948) 1950”. While the intended year for Volume X, Parts I & II of the Indian Journal of Entomology was 1948, the actual dates of publication were June 1950 (Part I) and October 1950 (Part II), as clearly shown on the cover page of the Volume, which we have checked. Because the dates of publication are the ones to be considered, and for the sake of clarity, we hereby revise the species year of description to 1950.

***Cotesiapaludicolae* (Cameron, 1909), new combination**

*Apantelespaludicolae* Cameron, 1909.

*Apantelesplatyptiliae* Cameron, 1909.

**Type information.** Syntypes female, NHMUK (examined). Country of type locality: Sri Lanka.

**Geographical distribution.**AFR, OTL.

**AFR**: Sudan, Uganda; **OTL**: India, Sri Lanka.

**Notes.** This species belongs in *Cotesia* based on sculpture and carination of the propodeum, shapes of T1 and T2, inflexible hypopygium and relatively short ovipositor sheaths. Wilkinson (1928a, 1932a) correctly associated this species with the *glomeratus* group.

***Cotesiapaphi* (Schrottky, 1902)**

*Apantelespaphi* Schrottky, 1902.

**Type information.** Syntypes female and male, depository unknown (not examined). Country of type locality: Argentina.

**Geographical distribution.**NEO.

**NEO**: Argentina, Brazil (SP), Peru, Uruguay.

**Notes.** The depository is presumed to be the MACN, but we have not been able to corroborate that.

***Cotesiapappi* Inanç, 2002**

*Cotesiapappi* Inanç, 2002.

**Type information.** Holotype female, ZMTU (not examined but original description checked). Country of type locality: Turkey.

**Geographical distribution.**PAL.

**PAL**: Turkey.

***Cotesiaparastichtidis* (Muesebeck, 1921)**

*Apantelesparastichtidis* Muesebeck, 1921.

**Type information.** Holotype female, USNM (examined). Country of type locality: USA.

**Geographical distribution.**NEA.

**NEA**: Canada (AB, BC, MB, NB, NS, ON, YT), USA (AK, MI, NY, TN).

***Cotesiaparbhanii* (Rao, 1969), new combination**

*Apantelesparbhanii* Rao, 1969.

**Type information.** Holotype female, NZSI (not examined but original description checked). Country of type locality: India.

**Geographical distribution.**OTL.

**OTL**: India.

**Notes.** From the original description it is clear that this species belongs to *Cotesia*, based on propodeum with median longitudinal carina, shapes of T1 and T2, and length of ovipositor sheaths. Rao (1969: 222) mentioned that although the specimens were in his personal collection, they would be deposited in the NZSI, information that we tentatively follow here when recording the type depository.

***Cotesiaparijati* Sathe, 2003**

*Cotesiaparijati* Sathe, 2003.

**Type information.** Holotype female, SUKI (not examined but original description checked). Country of type locality: India.

**Geographical distribution.**OTL.

**OTL**: India.

***Cotesiaparvicornis* (de Saeger, 1944), new combination**

*Apantelesparvicornis* de Saeger, 1944.

**Type information.** Holotype female, RMCA (not examined but original description checked). Country of type locality: Rwanda.

**Geographical distribution.**AFR.

**AFR**: Democratic Republic of Congo, Rwanda, Senegal.

**Notes.** Based on the details of the original description (de Saeger 1944), as well as the known host record, the best generic placement at present would be in *Cotesia*; however, future examination of the holotype will be needed (as there is a small chance that the species might belong to *Glyptapanteles*).

***Cotesiapeltoneni* (Papp, 1987)**

*Apantelespeltoneni* Papp, 1987.

**Type information.** Holotype female, MZH (not examined but original description checked). Country of type locality: Finland.

**Geographical distribution.**PAL.

**PAL**: Finland.

***Cotesiaphiloeampus* (Cameron, 1911)**

*Apantelesphiloeampus* Cameron, 1911.

**Type information.** Lectotype female, NHMUK (not examined but subsequent treatment of the species checked). Country of type locality: Australia.

**Geographical distribution.**AUS.

**AUS**: Australia (NSW).

**Notes.** Our species concept is based on Austin and Dangerfield (1992). There is some confusion with this species name and for the sake of clarity we provide more details here. Cameron (1911a: 342) described an Australian species as *Apantelesphiloeampus*. In another paper published that same year, Cameron (1911b: 327) also described a different species from Guyana as *Apantelesphilocampus*. Wilkinson (1928a: 96) correctly pointed that out; however, the two names being so similar (and being published by the same author in the same year, albeit in two different publications) have brought unintentional confusion over the years when dealing with them. For example, Austin and Dangerfield (1992: 22), when transferring the Australian species from *Apanteles* to *Cotesia* mentioned that *Apantelesphilocampus* was being transferred when they really meant *Apantelesphiloeampus*. The species from Guyana is also transferred from *Apanteles* in the present paper (see below under *Glyptapantelesphilocampus*).

***Cotesiaphobetri* (Rohwer, 1915)**

*Apantelesphobetri* Rohwer, 1915.

**Type information.** Holotype female, USNM (examined). Country of type locality: USA.

**Geographical distribution.**NEA.

**NEA**: Canada (AB, NL, ON), USA (IL, IN, KS, KY, MD, MA, MO, NH, NJ, NY, NC, RI, VT, VA, WV).

***Cotesiapholisorae* (Riley, 1889)**

*Apantelespholisorae* Riley, 1889.

**Type information.** Syntypes female and male, USNM (examined). Country of type locality: USA.

**Geographical distribution.**NEA.

**NEA**: USA (CT, DC, IL, KS, MD, MO, SC, WV).

***Cotesiapieridis* (Bouché, 1834)**

*Microgasterpieridis* Bouché, 1834.

**Type information.** Type lost (not examined but subsequent treatment of the species checked). Country of type locality: Germany.

**Geographical distribution.**PAL.

**PAL**: Armenia, Azerbaijan, China (SD), Georgia, Germany, Hungary, Israel, Kazakhstan, Lithuania, Moldova, Mongolia, Romania, Russia (PRI, SAK, VLA, VOR), Slovakia, Tajikistan, Turkey, Uzbekistan.

**Notes.** Our species concept is based on Nixon (1974), Papp (1987a), and Chen and Song (2004). Nixon (1974: 493) considered the type to be lost, based on unpublished notes from Wilkinson, who had examined the Bouché collection. The species distribution in Israel is based on Belokobylskij et al. (2019).

***Cotesiapilicornis* (Thomson, 1895)**

*Microgasterpilicornis* Thomson, 1895.

*Apantelespiliflagellaris* Tobias, 1986.

**Type information.** Holotype female, MZLU (not examined but subsequent treatment of the species checked). Country of type locality: Sweden.

**Geographical distribution.**PAL.

**PAL**: Bulgaria, Croatia, Finland, Germany, Hungary, Italy, Moldova, Romania, Russia (C, E, NC, S), Slovakia, Sweden, Switzerland, Turkey, United Kingdom.

**Notes.** Our species concept is based on Nixon (1974) and Papp (1987a). Broad et al. (2016) commented on the morphological variation of specimens they had examined, which they considered as conspecific anyway.

***Cotesiapistrinariae* (Wilkinson, 1929)**

*Apantelespistrinariae* Wilkinson, 1929.

*Apantelespistrinariaenyasaensis* Wilkinson, 1929.

**Type information.** Holotype female, NHMUK (examined). Country of type locality: Nigeria.

**Geographical distribution.**AFR.

**AFR**: Democratic Republic of Congo, Eritrea, Ethiopia, Malawi, Nigeria, Rwanda, South Africa.

**Notes.** The record of this species from Cape Verde (e.g., Koponen 1989, Forshage et al. 2016, Gupta et al. 2016b) is based on specimens of *Cotesiacompressithorax* (which was considered a synonym of *pistrinariae* until this paper, where we consider both as valid species, see more comments about differences between these two species under the Notes for *compressithorax*; p 292, 293). Thus, here we remove that country from the geographical distribution of *pistrinariae*.

***Cotesiaplanula* Song & Chen, 2004**

*Cotesiaplanula* Song & Chen, 2004.

**Type information.** Holotype female, FAFU (not examined but original description checked). Country of type locality: China.

**Geographical distribution.**PAL.

**PAL**: China (NX).

***Cotesiaplathypenae* (Muesebeck, 1921)**

*Apantelesplathypenae* Muesebeck, 1921.

**Type information.** Holotype female, USNM (not examined but original description checked). Country of type locality: USA.

**Geographical distribution.**NEA.

**NEA**: Canada (BC, MB), USA (ID, IL, IN, IA, KS, MO, NY, OH, SD, WA).

***Cotesiapodunkorum* (Viereck, 1912)**

*Apantelespodunkorum* Viereck, 1912.

**Type information.** Holotype female, USNM (examined). Country of type locality: USA.

**Geographical distribution.**NEA.

**NEA**: USA (CT, OH, VA, WV).

***Cotesiapraepotens* (Haliday, 1834)**

*Microgasterpraepotens* Haliday, 1834.

*Microgasterplacidus* Haliday, 1834.

*Apantelesmemnon* Nixon, 1974.

*Apantelesacutivalvis* Balevski, 1980.

*Apantelesbeshtaui* Tobias, 1986.

**Type information.** Lectotype female, NMID (not examined but authoritatively identified specimens examined). Country of type locality: Ireland.

**Geographical distribution.**PAL.

**PAL**: Afghanistan, Armenia, Azerbaijan, Bulgaria, Croatia, Czech Republic, Finland, Germany, Greece, Hungary, Iran, Ireland, Italy, Kazakhstan, Lithuania, Macedonia, Moldova, Mongolia, Poland, Romania, Russia (ZAB, KDA, PRI, SAR, STA, VOR), Slovakia, Spain, Sweden, Switzerland, Tajikistan, Turkey, Turkmenistan, United Kingdom, Uzbekistan, Yugoslavia.

**Notes.** We examined the type of *Apantelesmemnon* Nixon. Our species concept is based on van Achterberg (1997), but see notes under *Cotesiabrachycera* for more details on the history and use of related names and species.

***Cotesiapratapae* (Ashmead, 1896), new combination**

*Apantelespratapae* Ashmead, 1896.

**Type information.** Holotype female, USNM (examined). Country of type locality: Sri Lanka.

**Geographical distribution.**OTL.

**OTL**: Sri Lanka.

**Notes.** Transferred to *Cotesia* based on the following: inflexible hypopygium, ovipositor sheaths short, propodeum reticulated (although without clear median carina), T1 mostly parallel-sided, but slightly narrowing medially, and T2 rectangular and rugulose. The shape of T1 in this species is similar to that of *C.trabalae* Gupta, 2016, a very rare feature in *Cotesia* (see Gupta et al. 2016b for more details).

***Cotesiaprenidis* (Muesebeck, 1921)**

*Apantelesprenidis* Muesebeck, 1921.

**Type information.** Holotype female, USNM (examined). Country of type locality: Puerto Rico.

**Geographical distribution.**NEO.

**NEO**: Puerto Rico.

***Cotesiaprogahinga* (Hedqvist, 1965)**

*Apantelesprogahinga* Hedqvist, 1965.

**Type information.** Holotype female, MZH (examined). Country of type locality: Cape Verde.

**Geographical distribution.**AFR.

**AFR**: Cape Verde.

**Notes.**Forshage et al. (2016) considered the type material to be lost; however, in 2017 the holotype was found by the senior author in another section of the MZH collection.

***Cotesiaprozorovi* (Telenga, 1955), new combination**

*Apantelesprozorovi* Telenga, 1955.

**Type information.** Syntypes female and male, ZIN? (not examined but original description checked). Country of type locality: Russia.

**Geographical distribution.**PAL.

**PAL**: Russia (IRK, PRI).

**Notes.** Our species concept is based on the original description and Papp (1987a). It is not clear that the type specimens are deposited in the ZIN, but it is an educated guess based on Tobias (1986).

***Cotesiapterophoriphagus* (Shenefelt, 1972), new combination**

*Apantelespterophoriphagus* Shenefelt, 1972.

*Apantelespterophori* Risbec, 1951 [homonym of *Apantelespterophori* Muesebeck, 1926].

**Type information.** Holotype female, depository unknown (not examined but original description checked). Country of type locality: Senegal.

**Geographical distribution.**AFR.

**AFR**: Senegal.

**Notes.** Transferred to *Cotesia* based on the propodeum with a strong median carina (in addition to lateral and transverse carinae), shape and sculpture of T1–T2, acute hypopygium and short ovipositor sheaths (Risbec 1951: 435–437, see also figure 13 in that paper). The original description also compares this species with *sphenarchi* (Risbec, 1951), also currently placed in *Cotesia*. Because the name is to be considered as a noun under ICZN Article 31.2.1, it must retain its original spelling and remain as *pterophoriphagus*.

***Cotesiapyralidis* (Muesebeck, 1921)**

*Apantelespyralidis* Muesebeck, 1921.

**Type information.** Holotype female, USNM (examined). Country of type locality: USA.

**Geographical distribution.**NEA.

**NEA**: USA (AR, DC, IL, MD, MO, NC, OH, WI).

***Cotesiapyraustae* (Viereck, 1912)**

*Apantelespyraustae* Viereck, 1912.

**Type information.** Holotype female, USNM (examined). Country of type locality: USA.

**Geographical distribution.**NEA.

**NEA**: Canada (ON), USA (CT, MO, NJ).

**Notes.** The holotype is missing the metasoma, two legs, one pair of wings and the apical half of the antennae.

***Cotesiapyrophilae* (Muesebeck, 1926)**

*Apantelespyrophilae* Muesebeck, 1926.

**Type information.** Holotype female, USNM (examined). Country of type locality: USA.

**Geographical distribution.**NEA.

**NEA**: Canada (ON), USA (CT, MA, RI).

***Cotesiaradiantis* (Wilkinson, 1929)**

*Apantelesradiantis* Wilkinson, 1929.

**Type information.** Holotype female, NHMUK (not examined but subsequent treatment of the species checked). Country of type locality: Australia.

**Geographical distribution.**AUS, OTL.

**AUS**: Australia (QLD); **OTL**: China (HN).

**Notes.** Our concept of this species is based on Austin and Dangerfield (1992).

***Cotesiaradiarytensis* (Shenefelt, 1972), new combination**

*Apantelesradiarytensis* Shenefelt, 1972.

*Apantelesradiatus* Niezabitowski, 1910 [homonym of *Apantelesradiatus* Ashmead, 1898].

**Type information.** Holotype female, depository unknown (not examined but original description checked). Country of type locality: Poland.

**Geographical distribution.**PAL.

**PAL**: Poland.

**Notes.** Our species concept is based on the original description, as well as the key and comments provided by Telenga (1955). From those two sources, it is clear that this species is not *Apanteles*, based on the very short ovipositor, and the position in Telenga’s key (near many species of *Cotesia* and far from all true *Apanteles* keyed out in that paper). Without examining an actual specimen (and we note that there is no information about the whereabouts of the holotype), this species cannot be unambiguously assigned to genus. However, *Cotesia* seems the most reasonable choice, and is the one we propose here (other alternatives based on elements from the original description, such as *Protapanteles*, *Glyptapanteles* and even *Nyereria*, are much less plausible).

***Cotesiarangii* (Bhatnagar, 1950), new combination**

*Apantelesrangii* Bhatnagar, 1950.

**Type information.** Holotype male, INPC (not examined but original description checked). Country of type locality: India.

**Geographical distribution.**OTL.

**OTL**: India.

**Notes.** Transferred to *Cotesia* based on the followings details provided in the original description (Bhatnagar 1950: 165–166, also figs 40, 84 in that paper): propodeum rugulose and with transverse and median longitudinal carinae (both strongly marked), as well as other carinae (around the median one) running upwards; T1 relatively broad (length less than 2.0 x its width) but mostly parallel-sided; T2 as long as T3 and with curved margins laterally. The median, longitudinal carina in the propodeum excludes this species from *Apanteles*, whereas the strong transverse carina and the shapes of T1 and T2 exclude it from *Glyptapanteles*. Bhatnagar (1950) considered *rangii* to come close to *Apantelessundanus* (Wilkinson, 1930) [which in this paper is placed within the genus *Neoclarkinella*, see notes under that species in its treatment below], probably due to the presence of both longitudinal and transverse carinae in both taxa. However, *rangii* cannot be placed in *Neoclarkinella* as that genus has T1 strongly narrowing towards the posterior margin (T1 width at anterior margin being several times that of width at posterior margin), and T2 is much smaller than T3; also, the veins r and 2RS in *Neoclarkinella* are curved in a very characteristic way (e.g., see Figs 161–165 in present paper) whereas the shape of those veins in *rangii* are very different (see fig. 40 in Bhatnagar 1950). Thus, we consider that the available evidence strongly indicates *Cotesia* as the best generic placement at present. The year of publication of the Bhatnagar paper was until recently commonly cited as 1948 and/or 1950 (e.g., Chen and Song 2004, Yu et al. 2016), probably following Shenefelt (1972) who referred to this paper as “Bhatnagar (1948) 1950”. While the intended year for Volume X, Parts I & II of the Indian Journal of Entomology was 1948, the actual dates of publication were June 1950 (Part I) and October 1950 (Part II), as clearly shown on the cover page of the Volume, which we have checked. Because the dates of publication are the ones to be considered, and for the sake of clarity, we hereby revise the species year of description to 1950.

***Cotesiarisilis* (Nixon, 1974)**

*Apantelesrisilis* Nixon, 1974.

**Type information.** Holotype female, NHMUK (examined). Country of type locality: United Kingdom.

**Geographical distribution.**PAL.

**PAL**: Greece, Hungary, Iran, Italy, Mongolia, Montenegro, Netherlands, Romania, Slovakia, Turkey, United Kingdom.

***Cotesiariverai* (Porter, 1916), name amended and new combination**

*Apantelesriverae* Porter, 1916 [incorrect original spelling].

**Type information.** Syntypes female and male, depository unknown (not examined but original description checked). Country of type locality: Chile.

**Geographical distribution.**NEO.

**NEO**: Chile.

**Notes.** The original spelling of the species *Apantelesriverae* is incorrect, as the species was named after M.J. Rivera, a man (Porter 1916: 96), and thus its ending should be i instead of ae. The correct spelling is here amended to *riverai*. After reading the original description, we consider that the evidence there strongly supports this species as belonging to *Cotesia*. Porter (1916: 96–98) mentioned a median longitudinal carina on the propodeum, a quadrate T1, a T2 with a median field (smoother than the rest of the tergite), and very short ovipositor sheaths that barely project beyond the metasoma. He also provided illustrations of the fore wing and hind leg. Additionally, the host of the type series (Erebidae) and the gregarious wasp cocoons are common, although not exclusive, features of *Cotesia*.

***Cotesiarubecula* (Marshall, 1885)**

*Apantelesrubecula* Marshall, 1885.

**Type information.** Holotype female, NHMUK (examined). Country of type locality: United Kingdom.

**Geographical distribution.**AUS, NEA, OTL, PAL.

**AUS**: New Zealand; **NEA**: Canada (BC, ON, QC), USA (MA, MI, MN, OR, VT, VA, WA); **OTL**: China (FJ, HB, ZJ); **PAL**: Austria, Bulgaria, China (BJ, HE, JL, LN, SN), France, Germany, Hungary, Iran, Macedonia, Moldova, Netherlands, Poland, Romania, Russia (IRK, KHA, KDA, MOS, PRI, ROS, RYA, SAK), Slovakia, Spain, Switzerland, Ukraine, United Kingdom, Yugoslavia.

***Cotesiarubripes* (Haliday, 1834)**

*Microgasterrubripes* Haliday, 1834.

**Type information.** Lectotype female, MVMMA (not examined but subsequent treatment of the species checked). Country of type locality: United Kingdom.

**Geographical distribution.**PAL.

**PAL**: Belarus, Bulgaria, Croatia, Czech Republic, France, Germany, Hungary, Israel, Italy, Japan, Kazakhstan, Korea, Lithuania, Mongolia, Morocco, Poland, Romania, Russia (KDA, KYA, MOS, PRI, TOM, VOR, YAR), Serbia, Switzerland, Turkey, Ukraine, United Kingdom, Uzbekistan, Yugoslavia.

**Notes.** Our species concept is based on Wilkinson (1945), Nixon (1974), Papp (1987a), Kotenko (2007a), and Broad et al. (2016). The species distribution in Japan and Turkey is based on Belokobylskij et al. (2019).

***Cotesiaruficoxis* (Hedwig, 1962), new combination**

*Apantelesruficoxis* Hedwig, 1962.

**Type information.** Holotype female, depository unknown (not examined but original description checked). Country of type locality: Greece.

**Geographical distribution.**PAL.

**PAL**: Greece.

**Notes.** Based on the original description, this species is clearly not *Apanteles*, due to a median longitudinal carina on the propodeum and the short ovipositor. Those characters, coupled with the shape and sculpture of T1 and T2 (also taken from the original description), strongly suggest the best generic placement at present to be in *Cotesia*. However, until the holotype (only known specimen) is found and studied, this decision must be considered as provisional.

***Cotesiaruficrus* (Haliday, 1834)**

*Microgasterruficrus* Haliday, 1834.

*Apantelesantipoda* Ashmead, 1900.

*Apantelesmanilae* Ashmead, 1904.

*Apantelessydneyensis* Cameron, 1911.

*Apantelesnarangae* Viereck, 1913.

*Microgastercontextus* Imhof & Labram, 1836.

*Apantelessesamiae* Risbec, 1956 [*nomen nudum*].

**Type information.** Lectotype female, MVMMA (not examined but subsequent treatment of the species checked). Country of type locality: unknown.

**Geographical distribution.**AFR, AUS, NEO, OTL, PAL.

**AFR**: Cameroon, Cape Verde, Ethiopia, Kenya, Ivory Coast, Madagascar, Mauritius, Nigeria, Réunion, Senegal, Somalia, South Africa, Sudan, Tanzania, Uganda, Yemen, Zimbabwe; **AUS**: Australia (NSW, QLD), Fiji, Hawaiian Islands, New Zealand; **NEO**: Trinidad & Tobago; **OTL**: Bangladesh, China (FJ, GD, GX, GZ, HI, HK, HB, HN, JS, JX, SH, SN, TW, YN, ZJ), India, Indonesia, Malaysia, Pakistan, Philippines, Ryukyu Islands, Sri Lanka, Thailand, Vietnam; **PAL**: Afghanistan, Algeria, Armenia, Azerbaijan, Belarus, Belgium, Bulgaria, China (AH, BJ, HE, HL, HA, JL, LN, SN, SD), Cyprus, Egypt, Finland, France, Georgia, Germany, Hungary, Iceland, Iran, Iraq, Ireland, Israel, Italy, Japan, Kazakhstan, Korea, Libya, Lithuania, Malta, Moldova, Mongolia, Nepal, Netherlands, Poland, Romania, Russia (AD, AST, ZAB, KAM, KDA, NIZ, PNZ, PRI, ROS, SAK, YAR), Slovakia, Slovenia, Spain, Sweden, Switzerland, Turkey, Turkmenistan, Ukraine, United Kingdom, Yugoslavia.

**Notes.** The country of the lectotype was not specified by van Achterberg (1997: 74), it could be either Ireland or the United Kingdom. We examined the female holotype of *Apantelesantipoda* (Ashmead, 1900), and indeed it looks like *C.ruficrus*. We also examined the type, a female specimen, of *Apantelesnarangae* (Viereck, 1913), another synonym of *ruficrus*. van Achterberg (2014) synonymized *Microgastercontextus* (Imhof & Labram, 1836) under *C.rufricus* based on the figure and biology detailed in the original description of *contextus*. The species distribution in Afghanistan is based on Belokobylskij et al. (2019).

***Cotesiarufiventris* (Bingham, 1906)**

*Apantelesrufiventris* Bingham, 1906.

**Type information.** Lectotype female, OUMNH (not examined but subsequent treatment of the species checked). Country of type locality: Australia.

**Geographical distribution.**AUS.

**AUS**: Australia (QLD).

**Notes.** Our species concept is based on Wilkinson (1928a, 1930a), Austin and Dangerfield (1992) and van Achterberg & O’Toole (1993).

***Cotesiarufocoxalis* (Riley, 1881)**

*Apantelesrufocoxalis* Riley, 1881.

**Type information.** Syntypes female and male, USNM (examined). Country of type locality: USA.

**Geographical distribution.**NEA.

**NEA**: Canada (NS), USA (AL, CT, DC, FL, LA, MO, NJ, NY, TN, TX, VA).

**Notes.** The type series is on a single pin, which has a piece of card containing 32+ specimens and the cocoon mass.

***Cotesiarugosa* (Szépligeti, 1914)**

*Apantelesrugosus* Szépligeti, 1914.

**Type information.** Holotype male, MNHN (not examined but subsequent treatment of the species checked). Country of type locality: Kenya.

**Geographical distribution.**AFR.

**AFR**: Kenya.

**Notes.** Our species concept is based on Wilkinson (1932a) and Papp (2008).

***Cotesiaruidus* (Wilkinson, 1928)**

*Apantelesruidus* Wilkinson, 1928.

**Type information.** Holotype female, NHMUK (examined). Country of type locality: India.

**Geographical distribution.**OTL.

**OTL**: India.

***Cotesiasalebrosa* (Marshall, 1885), lectotype designation**

*Apantelessalebrosus* Marshall, 1885.

*Apantelescallunae* Nixon, 1974.

**Type information.** Lectoype female, NHMUK (examined). Country of type locality: United Kingdom.

**Geographical distribution.**PAL.

**PAL**: Bulgaria, Finland, France, Germany, Hungary, Iran, Italy, Korea, Lithuania, Mongolia, Norway, Poland, Russia (YAR), Sweden, Switzerland, Turkey, Ukraine, United Kingdom.

**Notes.** We have examined what by all indications seem to be the type series from Marshall, which is in the NHMUK with number 3c.25. It contains two female and two male specimens and the code agrees with that reported for this species by Shenefelt (1972: 621), although Shenefelt reported the specimens to be all female. However, there is another label attached to those specimens from Nixon (with date of 1952) and that label states that those specimens are not the type of *salebrosus* Marshall but rather *A.solitarus* Ratz (a reference to *Microgastersolitarius* Ratzeburg, 1844, which is currently considered to be a synonym of *Cotesiamelanoscela* Ratzeburg, 1844). However, having examined other specimens of both *Cotesiamelanoscela* and *C.salebrosa* (e.g., see Ruohomäki et al. 2013), we think that the specimens from Marshall that are deposited in the NHMUK belong to the latter species, in that sense disagreeing with Nixon’s label from 1952. As far as we know no specimen from that card has ever been designated as the lectotype, thus we designate one here. The card has the two male specimens towards the left side, close to a single cocoon which is glued near them. The right side of the card contains the female specimens, with the bottom one having the metasoma and one hind leg detached (but glued nearby). The female at the top right of the card is the only specimen in the series that is in very good condition, and thus is the one we select as the lectotype. We also examined the type of *Apantelescallunae* Nixon. The species distribution in Iran is based on Belokobylskij et al. (2019).

***Cotesiasaltator* (Thunberg, 1822)**

*Ichneumonsaltator* Thunberg, 1822.

*Ichneumonsalsator* Thunberg, 1822 [incorrect original spelling].

*Ichneumonsaltator* Thunberg, 1824 [justified emendation and homonym of *Ichneumonsaltator* Müller, 1776].

**Type information.** Syntypes male, UUZM (not examined but subsequent treatment of the species checked). Country of type locality: Sweden.

**Geographical distribution.**PAL.

**PAL**: Armenia, Bulgaria, France, Germany, Hungary, Iran, Israel, Lebanon, Mongolia, Poland, Russia (SPE), Slovakia, Sweden, Turkey, Ukraine, Yugoslavia.

**Notes.** Our species concept is based on Shaw et al. (2009).

***Cotesiasaltatoria* (Balevski, 1980)**

*Apantelessaltatorius* Balevski, 1980.

**Type information.** Holotype female, ZIN (not examined but subsequent treatment of the species checked). Country of type locality: Bulgaria.

**Geographical distribution.**PAL.

**PAL**: Bulgaria, Croatia, France, Germany, Hungary, Macedonia, Mongolia, Serbia, Slovakia, Spain, Turkey, United Kingdom.

**Notes.** Our species concept is based on Shaw (2007).

***Cotesiasasakii* (Watanabe, 1932)**

*Apantelessasakii* Watanabe, 1932.

**Type information.** Holotype female, EIHU (examined). Country of type locality: Japan.

**Geographical distribution.**PAL.

**PAL**: Japan, Korea.

**Notes.** The female holotype is missing the metasoma, but we have examined six other female specimens from the same date and locality which are in better condition. The species is rather characteristic in having ovipositor sheaths with long setae, much longer than the sheath width.

***Cotesiasatunini* (Tobias, 1986)**

*Apantelessatunini* Tobias, 1986.

**Type information.** Holotype female, ZIN (not examined but subsequent treatment of the species checked). Country of type locality: Azerbaijan.

**Geographical distribution.**PAL.

**PAL**: Azerbaijan.

**Notes.** Our species concept is based on Tobias (1986) and Papp (1988, 1990).

***Cotesiascabricula* (Reinhard, 1880)**

*Apantelesscabriculus* Reinhard, 1880.

*Apanteleseguchii* Watanabe, 1935.

**Type information.** Holotype female, depository unknown (not examined but subsequent treatment of the species checked). Country of type locality: Austria.

**Geographical distribution.**OTL, PAL.

**OTL**: China (HB, HN, SN, ZJ); **PAL**: Armenia, Austria, China (HE, SD, SN), Germany, Hungary, Iran, Italy, Korea, Macedonia, Moldova, Mongolia, Romania, Russia (KDA), Serbia, Slovakia, Switzerland.

**Notes.** Our species concept is based on Nixon (1974) and Papp (1987a). The species distribution in Iran is based on Belokobylskij et al. (2019).

***Cotesiaschaeferi* (Marsh, 1979)**

*Apantelesschaeferi* Marsh, 1979.

**Type information.** Holotype female, USNM (not examined but original description checked). Country of type locality: Japan.

**Geographical distribution.**PAL.

**PAL**: China (BJ), Japan, Korea.

***Cotesiaschaffneri* (Muesebeck, 1931)**

*Apantelesschaffneri* Muesebeck, 1931.

**Type information.** Holotype female, USNM (examined). Country of type locality: USA.

**Geographical distribution.**NEA.

**NEA**: USA (DE, NJ, PA, TX, VA).

***Cotesiaschini* (Muesebeck, 1958)**

*Apantelesschini* Muesebeck, 1958.

**Type information.** Holotype female, USNM (examined). Country of type locality: Brazil.

**Geographical distribution.**NEO.

**NEO**: Brazil (SP).

***Cotesiaschizurae* (Ashmead, 1898)**

*Apantelesschizurae* Ashmead, 1898.

**Type information.** Holotype female, USNM (not examined but subsequent treatment of the species checked). Country of type locality: USA.

**Geographical distribution.**NEA.

**NEA**: Canada (ON), USA (AZ, AR, CA, CT, FL, IL, MD, MA, MS, MO, NH, NY, VA, WI).

**Notes.** Our species concept is based on Muesebeck (1921), Papp (1987a), Mason (1981), and Fernandez-Triana (2010).

***Cotesiascitula* (Riley, 1881)**

*Apantelesscitulus* Riley, 1881.

*Apantelesemarginata* Riley, 1889.

*Apantelesparorgyiae* Ashmead, 1898.

*Cryptapantelesrileyana* Viereck, 1910.

**Type information.** Syntypes female, USNM (examined). Country of type locality: USA.

**Geographical distribution.**NEA.

**NEA**: Canada (NS, ON), USA (CT, DC, FL, IL, KS, KY, LA, MD, MO, NE, NH, NJ, TN, TX, WI).

**Notes.** We have also examined the type of *A.rileyanus* (Viereck, 1910).

***Cotesiascotti* (Valerio & Whitfield, 2009)**

*Parapantelesscotti* Valerio & Whitfield, 2009.

**Type information.** Holotype female, INBio (not examined but original description checked). Country of type locality: Costa Rica.

**Geographical distribution.**NEO.

**NEO**: Costa Rica.

**Notes.** Our species concept is based on Freitas et al. (2019).

***Cotesiaselenevora* Shaw, 2009**

*Cotesiaselenevora* Shaw, 2009.

**Type information.** Holotype female, RSME (examined). Country of type locality: Belgium.

**Geographical distribution.**PAL.

**PAL**: Belgium, Finland, Sweden.

***Cotesiasenegalensis* (Risbec, 1951), new combination**

*Apantelessenegalensis* Risbec, 1951.

**Type information.** Holotype female, depository unknown (not examined but original description checked). Country of type locality: Senegal.

**Geographical distribution.**AFR.

**AFR**: Senegal.

**Notes.** From the details provided in the original description it is clear that this species belongs to *Cotesia*.

***Cotesiasericea* (Nees, 1834)**

*Microgastersericeus* Nees, 1834.

**Type information.** Type and depository unknown (not examined but subsequent treatment of the species checked). Country of type locality: Germany.

**Geographical distribution.**PAL.

**PAL**: Azerbaijan, Belgium, France, Georgia, Germany, Italy, Mongolia, Russia (KDA, NGR, SPE, SAR, YAR), Tajikistan, Turkmenistan, Ukraine, United Kingdom, Uzbekistan.

**Notes.** Our species concept is based on Belokobylskij et al. (2003), which was followed by Broad et al. (2016), but see Notes under *Cotesiabrachycera* for more details on the history and use of related names and species.

***Cotesiasesamiae* (Cameron, 1906)**

*Apantelessesamiae* Cameron, 1906.

**Type information.** Type and depository unknown (not examined but subsequent treatment of the species checked). Country of type locality: South Africa.

**Geographical distribution.**AFR, OTL.

**AFR**: Benin, Burkina Faso, Cameroon, Central African Republic, Comoros, Democratic Republic of Congo, Eritrea, Ethiopia, Ghana, Ivory Coast, Kenya, Lesotho, Madagascar, Malawi, Mauritius, Mozambique, Nigeria, Réunion, Senegal, South Africa, Sudan, Tanzania, Uganda, Zambia, Zimbabwe; **OTL**: India.

**Notes.** Our species concept is based on Kaiser et al. (2017). According to Madl & van Achterberg (2014), this species was successfully introduced to Comoros as a biological control agent.

***Cotesiasetebis* (Nixon, 1974)**

*Apantelessetebis* Nixon, 1974.

*Apanteleskhibinica* Tobias, 1986.

**Type information.** Holotype female, NHMUK (examined). Country of type locality: Sweden.

**Geographical distribution.**PAL.

**PAL**: Bulgaria, Czech Republic, Greece, Hungary, Iran, Mongolia, Russia (MUR, SVE), Slovakia, Sweden, Switzerland, Turkey.

***Cotesiaseyali* (Risbec, 1951), new combination**

*Apantelesseyali* Risbec, 1951.

**Type information.** Type and depository unknown (not examined but original description checked). Country of type locality: Senegal.

**Geographical distribution.**AFR.

**AFR**: Senegal.

**Notes.** Transferred to *Cotesia* based on the propodeum with a median carina (in addition to lateral and transverse carinae), shape and sculpture of T1–T2. In the original description *seyali* is presented as very similar (morphologically) with *sphenarchi* (Risbec, 1951), also described in that same paper and currently placed in *Cotesia*.

***Cotesiashemachaensis* (Tobias, 1976)**

*Apantelesshemachaensis* Tobias, 1976.

**Type information.** Holotype female, ZIN (not examined but subsequent treatment of the species checked). Country of type locality: Azerbaijan.

**Geographical distribution.**PAL.

**PAL**: Azerbaijan, Hungary, Kazakhstan.

**Notes.** Our species concept is based on Tobias (1986) and Papp (1987a).

***Cotesiashrii* Sathe, Ingawale & Bhosale, 1994**

*Cotesiashrii* Sathe, Ingawale & Bhosale, 1994.

**Type information.** Type and depository unknown (not examined). Country of type locality: India.

**Geographical distribution.**OTL.

**OTL**: India.

***Cotesiasibyllarum* (Wilkinson, 1936)**

*Apantelessibyllarum* Wilkinson, 1936.

*Apantelessibyllarumnipponensis* Watanabe, 1942.

**Type information.** Holotype female, NHMUK (examined). Country of type locality: United Kingdom.

**Geographical distribution.**NEA, PAL.

**NEA**: USA (MA); **PAL**: Czech Republic, Germany, Hungary, Japan, Slovakia, United Kingdom.

***Cotesiasimurae* (You & Zhou, 1989)**

*Apantelessimurae* You & Zhou, 1989.

**Type information.** Holotype female, HUNAU (not examined but subsequent treatment of the species checked). Country of type locality: China.

**Geographical distribution.**OTL.

**OTL**: China (YN).

**Notes.** Our species concept is based on Chen and Song (2004).

***Cotesiasmerinthi* (Riley, 1881)**

*Apantelessmerinthi* Riley, 1881.

**Type information.** Syntypes female, USNM (examined). Country of type locality: USA.

**Geographical distribution.**NEA.

**NEA**: Canada (BC, ON, QC), USA (CA, CO, DC, IN, MD, MA, MO, NH, NJ, TX).

***Cotesiasorghiellae* (Muesebeck, 1933)**

*Apantelessorghiellae* Muesebeck, 1933.

**Type information.** Holotype female, USNM (examined). Country of type locality: USA.

**Geographical distribution.**NEA.

**NEA**: USA (AR, MO, TX).

**Notes.** The female holotype has the head detached.

***Cotesiaspecularis* (Szépligeti, 1896)**

*Apantelesspecularis* Szépligeti, 1896.

*Apantelesbalcanica* Balevski, 1980.

**Type information.** Lectotype female, HNHM (not examined but subsequent treatment of the species checked). Country of type locality: Hungary.

**Geographical distribution.**PAL.

**PAL**: Bulgaria, Germany, Greece, Hungary, Iran, Israel, Jordan, Kyrgyzstan, Moldova, Romania, Russia (PRI), Spain, Tajikistan, Turkey, Uzbekistan.

**Notes.** Our species concept is based on Shaw et al. (2009). The species distribution in Israel and Tajikistan is based on Belokobylskij et al. (2019).

***Cotesiasphenarchi* (Risbec, 1951), new combination**

*Apantelessphenarchi* Risbec, 1951.

**Type information.** Syntypes female and male, depository unknown (not examined but original description checked). Country of type locality: Senegal.

**Geographical distribution.**AFR.

**AFR**: Senegal.

**Notes.** Transferred to *Cotesia* based on the propodeum with a median carina (in addition to lateral and transverse carinae), shape and sculpture of T1–T2 and short ovipositor sheaths (Risbec 1951: 433–435, fig. 11). The original description also compares this species with *ruficrus* (Haliday, 1834) and *nycteus* (de Saeger, 1944), both currently placed in *Cotesia*.

***Cotesiasphingivora* (Granger, 1949), new combination**

*Apantelessphingivorus* Granger, 1949.

**Type information.** Syntypes female and male, MNHN (not examined but original description checked). Country of type locality: Madagascar.

**Geographical distribution.**AFR.

**AFR**: Madagascar.

**Notes.** Transferred to *Cotesia* based on the original description mentioning the propodeum rugose with more or less defined areola and costulae, ovipositor sheaths short (0.7 × metatarsus length), and T1–T3 shape and sculpture, as illustrated and described in Granger (1949: 270–271, fig. 281). The host is reported to be Sphingidae, and the wasp cocoons form a dense, white mass, both features also common in (although not exclusive from) *Cotesia*. A record of this species from Réunion was later considered as doubtful (Madl and van Achterberg 2014), a decision we also follow here and thus we consider the species to be present only in Madagascar.

***Cotesiaspuria* (Wesmael, 1837)**

*Microgasterspurius* Wesmael, 1837.

*Microgasterinsidens* Ratzeburg, 1844.

**Type information.** Lectotype female, RBINS (examined). Country of type locality: Belgium.

**Geographical distribution.**PAL.

**PAL**: Afghanistan, Armenia, Austria, Azerbaijan, Belgium, Bulgaria, China (JL), Croatia, Finland, France, Germany, Greece, Hungary, Iran, Ireland, Israel, Italy, Japan, Kazakhstan, Korea, Latvia, Lithuania, Moldova, Poland, Romania, Russia (AD, KDA, NVS, PRI, ROS, RYA, SAM, VOR), Serbia, Slovakia, Slovenia, Sweden, Switzerland, Tajikistan, Turkey, Ukraine, United Kingdom, Uzbekistan.

**Notes.** The species distribution in Israel is based on Belokobylskij et al. (2019).

***Cotesiasubancilla* (Balevski, 1980)**

*Apantelessubancilla* Balevski, 1980.

**Type information.** Holotype female, ZIN (not examined but subsequent treatment of the species checked). Country of type locality: Bulgaria.

**Geographical distribution.**PAL.

**PAL**: Bulgaria, Greece, Hungary, Slovakia.

**Notes.** Our species concept is based on Tobias (1986) and Papp (1987a).

***Cotesiasubordinaria* (Tobias, 1976)**

*Apantelessubordinarius* Tobias, 1976.

**Type information.** Holotype female, ZIN (not examined but subsequent treatment of the species checked). Country of type locality: Georgia.

**Geographical distribution.**PAL.

**PAL**: Azerbaijan, Georgia, Netherlands, Russia (NC), United Kingdom.

**Notes.** Our species concept is based on Shaw (2012a, 2012b).

***Cotesiasuvernii* Sathe, Ingawale & Bhosale, 1994**

*Cotesiasuvernii* Sathe, Ingawale & Bhosale, 1994.

**Type information.** Type and depository unknown (not examined). Country of type locality: India.

**Geographical distribution.**OTL.

**OTL**: India.

***Cotesiasuzumei* (Watanabe, 1932)**

*Apantelessuzumei* Watanabe, 1932.

**Type information.** Holotype male, EIHU (examined). Country of type locality: Japan.

**Geographical distribution.**PAL.

**PAL**: Japan.

**Notes.** We examined the holotype and it is a male specimen, not a female as it had been considered until now (e.g., Shenefelt, 1972). We also examined another six specimens and the remnants of a parasitized lepidopteran larva with rather loose wasp cocoons.

***Cotesiataprobanae* (Cameron, 1897)**

*Apantelestaprobanae* Cameron, 1897.

*Apantelesstauropi* Viereck, 1912.

*Apantelesformosae* Viereck, 1913.

**Type information.** Lectotype female, OUMNH (not examined but authoritatively identified specimens examined). Country of type locality: Sri Lanka.

**Geographical distribution.**OTL.

**OTL**: China (FJ, GD, HI, TW, ZJ), India, Indonesia, Sri Lanka, Vietnam.

**Notes.** We examined the types of *Apantelesstauropi*, *A.formosae*, and the two female paralectotypes of *taprobanae* deposited in the NHMUK.

***Cotesiatatehae* (Watanabe, 1932)**

*Apantelestatehae* Watanabe, 1932.

**Type information.** Holotype female, EIHU (examined). Country of type locality: Japan.

**Geographical distribution.**PAL.

**PAL**: Japan.

***Cotesiategera* (Papp, 1977)**

*Apantelestegerus* Papp, 1977.

**Type information.** Holotype female, HNHM (not examined but subsequent treatment of the species checked). Country of type locality: Mongolia.

**Geographical distribution.**PAL.

**PAL**: Mongolia.

**Notes.** Our species concept is based on Papp (1986, 1990, 2009).

***Cotesiateleae* (Muesebeck, 1926)**

*Apantelesteleae* Muesebeck, 1926.

**Type information.** Holotype female, USNM (examined). Country of type locality: USA.

**Geographical distribution.**NEA.

**NEA**: Canada (AB, BC), USA (CT, MD, PA).

**Notes.** The holotype has a mostly smooth T1 (only the apical 0.3 with shallow punctures) and an almost entirely smooth T2. The propodeum is also mostly smooth, with a few short carinae near the nucha, but without a median longitudinal carina, although there are traces (laterally) of the transverse carina, which forks around the spiracles. Overall this is a relatively very smooth species of *Cotesia* which could be considered to be a *Protapanteles*. However, because *Protapanteles* may represent just a species group of Cotesia (see above under the section Brief diagnosis of all Microgastrinae genera as they are understood in this paper for a discussion) we retain *teleae* within *Cotesia* for the time being.

***Cotesiatelengai* (Tobias, 1972)**

*Apantelestelengai* Tobias, 1972.

*Apantelesamabilis* Nixon, 1974.

**Type information.** Holotype female, ZIN (not examined but authoritatively identified specimens examined). Country of type locality: Armenia.

**Geographical distribution.**PAL.

**PAL**: Afghanistan, Albania, Algeria, Armenia, Azerbaijan, Bosnia and Herzegovina, Bulgaria, Croatia, Georgia, Germany, Greece, Hungary, Iran, Israel, Italy, Kazakhstan, Moldova, Morocco, Netherlands, Poland, Russia (VLA), Slovakia, Spain, Switzerland, Tajikistan, Tunisia, Turkey, Turkmenistan, United Kingdom, Uzbekistan.

**Notes.** We examined the type of *Apantelesamabilis* Nixon. The species distribution in Israel is based on Belokobylskij et al. (2019), that paper also recorded India and New Zealand as country records for the wasp species; however, we have not been able to find any published source supporting that and thus those records are excluded from our checklist until further evidence is available.

***Cotesiatenebrosa* (Wesmael, 1837)**

*Microgastertenebrosus* Wesmael, 1837.

**Type information.** Type lost (not examined but subsequent treatment of the species checked). Country of type locality: Belgium.

**Geographical distribution.**PAL.

**PAL**: Andorra, Azerbaijan, Belgium, Croatia, Finland, France, Georgia, Germany, Greece, Hungary, Iran, Israel, Kazakhstan, Korea, Macedonia, Moldova, Mongolia, Poland, Russia (PRI), Serbia, Spain, Sweden, Switzerland, Tajikistan, Turkey, Ukraine, United Kingdom, Uzbekistan.

**Notes.** Our species concept is based on Shaw (2007). Additional comments on this species are in Broad et al. (2016). The species distribution in Azerbaijan, Georgia, Iran, Kazakhstan, Tajikistan and Uzbekistan is based on Belokobylskij et al. (2019).

***Cotesiatestacea* Fujie, Shimizu & Fernandez-Triana, 2018**

*Cotesiatestacea* Fujie, Shimizu & Fernandez-Triana, 2018.

**Type information.** Holotype female, NIAES (examined). Country of type locality: Japan.

**Geographical distribution.**PAL.

**PAL**: Japan, Korea.

**Notes.** The original description discussed the possibility that a previous record of *Cotesiaferruginea* from Russia Far East in Primorsky Krai (Kotenko 2007a) might actually represent a specimen of *C.testacea*, as *C.ferruginea* is restricted to the Western Palaearctic.

***Cotesiatetrica* (Reinhard, 1880)**

*Apantelestetricus* Reinhard, 1880.

*Microgasteropaculus* Thomson, 1895.

**Type information.** Holotype female, ZMHB (not examined but subsequent treatment of the species checked). Country of type locality: Germany.

**Geographical distribution.**PAL.

**PAL**: Montenegro, United Kingdom.

**Notes.** Our species concept is based on Nixon (1974), Belokobylskij et al. (2003), and Broad et al. (2016), but see notes under *Cotesiabrachycera* for more details on the history and use of related names and species.

***Cotesiathapinthotha* Papp, 1990**

*Cotesiathapinthotha* Papp, 1990.

**Type information.** Holotype female, HNHM (not examined but original description checked). Country of type locality: Korea.

**Geographical distribution.**PAL.

**PAL**: Korea.

***Cotesiatheae* (Sonan, 1942)**

*Apantelestheae* Sonan, 1942.

**Type information.** Syntypes female and male, TARI (not examined but original description checked). Country of type locality: China.

**Geographical distribution.**OTL.

**OTL**: China (TW).

***Cotesiatheclae* (Riley, 1881)**

*Apantelestheclae* Riley, 1881.

**Type information.** Syntypes female and male, USNM (examined). Country of type locality: USA.

**Geographical distribution.**NEA, NEO.

**NEA**: USA (AL, CA, CO, CT, DC, GA, ID, KS, MO, NJ, OK, TX); **NEO**: Mexico.

***Cotesiatibialis* (Curtis, 1830)**

*Microgastertibialis* Curtis, 1830.

*Microgasteratrator* Curtis, 1829 [*nomen nudum*].

*Microgastergracilis* Curtis, 1829 [*nomen nudum*].

*Microgastertibialis* Curtis, 1829 [*nomen nudum*].

*Microgastercongesta* Nees, 1834.

*Microgasterintricata* Haliday, 1834.

*Microgastergracilipes* Thomson, 1895.

*Apantelessimilis* Szépligeti, 1901.

*Microgasteratratrix* Schulz, 1906.

*Apantelesaranearum* Goureau, 1908 [*nomen nudum*].

*Apantelesmamestrae* Matsumura, 1908.

*Apantelesimulans* (Lyle, 1917).

*Apantelesclaustrata* (Gautier & Bonnamour, 1923).

**Type information.** Holotype male, MVMMA (not examined but subsequent treatment of the species checked). Country of type locality: United Kingdom.

**Geographical distribution.**PAL.

**PAL**: Afghanistan, Armenia, Austria, Azerbaijan, Belgium, Bulgaria, Canary Islands, China (SN, XJ), Croatia, Czech Republic, Estonia, Finland, France, Georgia, Germany, Greece, Hungary, Iran, Ireland, Israel, Italy, Japan, Kazakhstan, Kyrgyzstan, Latvia, Lithuania, Macedonia, Moldova, Mongolia, Montenegro, Netherlands, Poland, Romania, Russia (AD, ZAB, IRK, KGD, KDA, KYA, MOS, PRI, SPE, TA, VLA, VGG, YAR), Serbia, Slovakia, Spain, Sweden, Switzerland, Tajikistan, Turkey, Turkmenistan, Ukraine, United Kingdom, Uzbekistan.

**Notes.** Our species concept is based on Nixon (1974), Papp (1986) and Tobias (1986). The information about the type is taken from Nixon (1974: 496). The species distribution in Tajikistan is based on Belokobylskij et al. (2019).

***Cotesiatiracolae* (Ashmead, 1896)**

*Apantelestivacolae* Ashmead, 1896.

*Apantelestivacholae* Ashmead, 1896 [original misspelling].

*Apantelestiracholae* Wilkinson, 1928 [unjustified emendation].

*Apantelestiracolae* Thompson, 1953 [justified emendation].

**Type information.** Holotype female, USNM (not examined but subsequent treatment of the species checked). Country of type locality: Sri Lanka.

**Geographical distribution.**OTL.

**OTL**: Sri Lanka.

**Notes.** Our species concept is based on Gupta & Fernandez-Triana (2014).

***Cotesiatmetocerae* (Muesebeck, 1921)**

*Apantelestmetocerae* Muesebeck, 1921.

**Type information.** Holotype female, USNM (examined). Country of type locality: Canada.

**Geographical distribution.**NEA.

**NEA**: Canada (NS).

***Cotesiatrabalae* Gupta, 2016**

*Cotesiatrabalae* Gupta, 2016.

**Type information.** Holotype female, NBAIR (examined). Country of type locality: India.

**Geographical distribution.**OTL.

**OTL**: India.

***Cotesiatransuta* (de Saeger, 1944), new combination**

*Apantelestransutus* de Saeger, 1944.

**Type information.** Holotype female, RMCA (not examined but original description checked). Country of type locality: Democratic Republic of Congo.

**Geographical distribution.**AFR.

**AFR**: Democratic Republic of Congo, Rwanda.

**Notes.** Based on the details provided in the original description, the best generic placement would be in *Cotesia*.

***Cotesiatuita* Papp, 2009**

*Cotesiatuita* Papp, 2009.

**Type information.** Holotype female, HNHM (not examined but original description checked). Country of type locality: Mongolia.

**Geographical distribution.**PAL.

**PAL**: Mongolia.

***Cotesiaturkestanica* (Telenga, 1955), new combination**

*Apantelesturkestanicus* Telenga, 1955.

**Type information.** Lectotype female, ZIN (not examined but original description checked). Country of type locality: Uzbekistan.

**Geographical distribution.**PAL.

**PAL**: Uzbekistan.

**Notes.** The original description and the key to species there, as well as the works of Tobias (1986) and Papp (1987a), make clear that this species belongs in *Cotesia*. We transfer it here based on the propodeum with a distinct, median longitudinal carina, shape and sculpture of T1 and T2, small hypopygium and very short ovipositor sheaths.

***Cotesiatyphae* Fernandez-Triana, 2017**

*Cotesiatyphae* Fernandez-Triana, 2017.

**Type information.** Holotype female, CBGP (examined). Country of type locality: Kenya.

**Geographical distribution.**AFR.

**AFR**: Ethiopia, Kenya, Tanzania.

***Cotesiaukrainica* (Tobias, 1986), status revised**

*Apantelesukrainicus* Tobias, 1986.

**Type information.** Holotype female, ZIN (examined). Country of type locality: Ukraine.

**Geographical distribution.**PAL.

**PAL**: Ukraine.

**Notes.**Papp (1988, his footnote 24 on page 154) synonymized *ukrainicus* under *Cotesiamelitaearum* (Wilkinson, 1937). However, after examining the holotype we consider it not conspecific with *melitaearum* but actually more related to the *Cotesiavestalis* and *C.ruficrus* species complex. Thus, here we resurrect *ukrainicus* from synonymy with *melitaearum* and consider it a valid species.

***Cotesiaunicolor* (Curtis, 1835)**

*Microgasterunicolor* Curtis, 1835.

**Type information.** Holotype male, NHMUK? (not examined but original description checked). Country of type locality: Canada.

**Geographical distribution.**NEA.

NEA: Canada (NU).

**Notes.** The information about this species is sparse and probably in need of further revision. The holotype (and only known specimen) was considered by Muesebeck (1922: 43) to be probably deposited in the NHMUK. Muesebeck transferred that species from *Microgaster* to *Apanteles* and he even commented that the species could be related to *Cotesiayakutatensis* (at that time the generic concept of *Apanteles* included *Cotesia*). Later, Mason (1981) transferred the species to *Cotesia*. Over the years several authors have cited the species (e.g., Shenefelt 1972, Marsh 1979, Whitfield 1995a) for North America, but without providing details on where it was found, the only information available so far is Arctic North America. The species was described by Curtis (1835: 62, with Roman numeral lxii) in an Appendix on Natural History that was part of the book about J. C. Ross’s second voyage in search of a North-west Arctic passage. We have carefully read the original description and agree that the species very likely belongs to *Cotesia*. Not considered before now, the original description actually mentions some information about the actual host: “A male was bred from a cluster of cocoons, enveloped in a silky ball, resembling those containing the eggs of some spiders”. That very likely refers to the hibernacula built by the larvae of the arctic woolly bear moth (*Gynaephoragroenlandica*, Lepidoptera: Erebidae), which looks like a silky ball from a spider egg sac. Two *Cotesia* species have already been recorded (Fernandez-Triana et al. 2017b) parasitizing larvae of *Gynaephora* spp. in the High Arctic: *Cotesiahalli* and an undetermined species with provisional name of *Cotesia* sp. 1; it may well be that *Cotesiaunicolor* is actually one of those species, but study of the holotype will be needed to form a firm conclusion. As for the actual collecting locality of the type, no details are provided in the original description. However, the second voyage of Ross was spent in areas of what is today considered to be Canadian territory of Nunavut, thus we provide all that information for the sake of the checklist completion.

***Cotesiaurabae* Austin & Allen, 1989**

*Cotesiaurabae* Austin & Allen, 1989.

**Type information.** Holotype female, ANIC (not examined but original description checked). Country of type locality: Australia.

**Geographical distribution.**AUS.

**AUS**: Australia (SA, TAS), New Zealand.

***Cotesiavanessae* (Reinhard, 1880)**

*Apantelesvanessae* Reinhard, 1880.

**Type information.** Type and depository unknown (not examined but subsequent treatment of the species checked). Country of type locality: Germany.

**Geographical distribution.**AFR, NEA, PAL.

**AFR**: Ethiopia; **NEA**: Canada (AB, ON); **PAL**: Afghanistan, Armenia, Austria, Azerbaijan, Bulgaria, Canary Islands, China (XJ), Czech Republic, Finland, France, Georgia, Germany, Greece, Hungary, Iran, Ireland, Israel, Italy, Japan, Kazakhstan, Korea, Latvia, Moldova, Montenegro, Morocco, Netherlands, Poland, Romania, Russia (BEL, BU, IRK, OMS, PRI, SAK, TOM), Serbia, Spain, Tunisia, Turkey, Ukraine, United Kingdom, Uzbekistan.

**Notes.** Our species concept is based on Shaw et al. (2009). The species distribution in Azerbaijan, Georgia, Israel, Japan, and Korea is based on Belokobylskij et al. (2019).

***Cotesiavestalis* (Haliday, 1834)**

*Microgastervestalis* Haliday, 1834.

*Apantelesplutellae* Kurdjumov, 1912.

**Type information.** Lectotype female, NMID (examined). Country of type locality: Ireland.

**Geographical distribution.**AFR, AUS, NEA, NEO, OTL, PAL.

**AFR**: Benin, Cape Verde, Kenya, Mauritius, Réunion, Saint Helena, Senegal, South Africa, Tanzania, Zimbabwe; **AUS**: Hawaiian Islands, New Zealand, Papua New Guinea, Western Samoa; **NEA**: USA (TX); **NEO**: Argentina, Brazil (PE); **OTL**: Bangladesh, China (GD, HN, SN, TW, ZJ), India, Malaysia, Pakistan, Philippines, Ryukyu Islands, Singapore, Sri Lanka, Thailand, Vietnam; **PAL**: Afghanistan, Armenia, Azerbaijan, Azores, Belgium, Bulgaria, China (BJ), Czech Republic, Finland, France, Germany, Greece, Hungary, Iran, Ireland, Israel, Italy, Japan, Kazakhstan, Korea, Kyrgyzstan, Latvia, Libya, Macedonia, Malta, Moldova, Mongolia, Morocco, Netherlands, Poland, Romania, Russia (AMU, ZAB, KDA, MOS, PRI, ROS, SAK, SPE, STA, VGG, YAR), Serbia, Spain, Sweden, Switzerland, Tajikistan, Tunisia, Turkey, Turkmenistan, Ukraine, United Kingdom, Uzbekistan.

**Notes.** Besides of examining the lectotype, our species concept is based on Shaw et al. (2009). New country data from Kant (2019), Ngowi et al. (2019), and Sithole et al. (2019).

***Cotesiavillana* (Reinhard, 1880)**

*Apantelesvillanus* Reinhard, 1880.

*Apantelesfasciatae* Gautier & du Dresnay, 1926.

*Apantelesrubroides* Papp, 1971.

**Type information.** Holotype female, ZMHB (not examined but subsequent treatment of the species checked). Country of type locality: Germany.

**Geographical distribution.**PAL.

**PAL**: Croatia, Finland, France, Germany, Greece, Hungary, Iran, Mongolia, Poland, Romania, Russia (ZAB, PRI), Slovakia, Switzerland, Turkey, United Kingdom.

**Notes.** Our species concept is based on Wilkinson (1945), Nixon (1974), and Papp (1986).

***Cotesiaviridanae* (Tobias, 1986)**

*Apantelesviridanae* Tobias, 1986.

**Type information.** Holotype female, ZIN (not examined but subsequent treatment of the species checked). Country of type locality: Russia.

**Geographical distribution.**PAL.

**PAL**: Russia (VOR).

**Notes.** Our species concept is based on Tobias (1986) and Papp (1990a).

***Cotesiaxavieri* Rousse, 2013**

*Cotesiaxavieri* Rousse, 2013.

**Type information.** Holotype female, MNHN (not examined but original description checked). Country of type locality: Réunion.

**Geographical distribution.**AFR.

**AFR**: Réunion.

***Cotesiaxylina* (Say, 1836)**

*Microgasterxylina* Say, 1836.

*Apantelescushmani* Viereck, 1912.

*Apantelesoxyacanthoidis* Viereck, 1912.

*Apanteleslanifica* Viereck, 1917.

**Type information.** Type lost (not examined but authoritatively identified specimens examined). Country of type locality: USA.

**Geographical distribution.**NEA.

**NEA**: Canada (AB, MB, NS, ON, QC), USA (CO, CT, DC, FL, IL, IN, KS, MA, NH, NJ, UT, VA, WV).

**Notes.** We have also examined the types of *Apantelescushmani* (Viereck, 1912), a male specimen, *Apanteleslanifica* (Viereck, 1917), a male specimen, and *Apantelesoxyacanthoidis* (Viereck, 1912), a female specimen, all currently synonyms of *C.xylina*.

***Cotesiayakutatensis* (Ashmead, 1902)**

*Apantelesyakutatensis* Ashmead, 1902.

*Apanteleshyslopi* Viereck, 1910.

**Type information.** Holotype female, USNM (not examined but authoritatively identified specimens examined). Country of type locality: USA.

**Geographical distribution.**NEA.

**NEA**: Canada (BC, MB, QC, NU), Greenland, USA (AK, CA, ID, OR, UT, WA).

**Notes.** We have examined the type of *Apanteleshyslopi* (Viereck, 1910), a female specimen.

***Cotesiazagrosensis* Zargar & Gupta, 2019**

*Cotesiazagrosensis* Zargar & Gupta, 2019.

**Type information.** Holotype female, NBAIR (not examined but original description checked). Country of type locality: Iran.

**Geographical distribution.**PAL.

**PAL**: Iran.

***Cotesiazygaenarum* (Marshall, 1885)**

*Apanteleszygaenarum* Marshall, 1885.

**Type information.** Lectotype female, NHMUK (examined). Country of type locality: United Kingdom.

**Geographical distribution.**OTL, PAL.

**OTL**: China (HB); **PAL**: Albania, Armenia, Austria, Azerbaijan, Czech Republic, Finland, France, Germany, Greece, Hungary, Iran, Ireland, Israel, Italy, Japan, Kazakhstan, Korea, Macedonia, Moldova, Mongolia, Poland, Romania, Russia (DA, KDA, OMS, PRI, RYA, VOR), Serbia, Slovakia, Switzerland, Turkey, Tunisia, United Kingdom.

**Notes.** The species distribution in Israel and Kazakhstan is based on Belokobylskij et al. (2019).

#### Genus Cuneogaster Choi & Whitfield, 2006

***Cuneogaster*** Choi & Whitfield, 2006: 120. Gender: feminine. Type species: Cuneogasterinae Choi & Whitfield, 2006, by original designation.

Only known from a single species from the Neotropical region (Choi and Whitfield 2006). We have seen in collections (CNC) a few additional species from South America, but the genus does not seem to be species rich. *Cuneogaster* is part of a group of genera (some described and some as yet undescribed) related to *Diolcogaster*; future phylogenetic studies of Microgastrinae may change the status of, and relationships between, some of those taxa. No host data are currently available for *Cuneogaster*. There are no DNA barcodes of this genus in BOLD.

***Cuneogasterinae* Choi & Whitfield, 2006**

*Cuneogasterinae* Choi & Whitfield, 2006.

**Type information.** Holotype female, IAVH (not examined but original description checked). Country of type locality: Colombia.

**Geographical distribution.**NEO.

**NEO**: Colombia, Panama, Venezuela.

#### Genus Dasylagon Muesebeck, 1958

***Dasylagon*** Muesebeck, 1958: 424. Gender: feminine. Type species: *Dasylagonaegeriae* Muesebeck, 1958, by original designation.

Only known from two described species from the Neotropical region. We have seen in collections (CNC) a few specimens that might represent additional species from South America, but the genus does not seem to be very speciose. Two families of Lepidoptera, Sesiidae, and Thyrididae, have been recorded as hosts of *Dasylagon*. There are no DNA barcodes of this genus in BOLD.

***Dasylagonaegeriae* Muesebeck, 1958**

*Dasylagonaegeriae* Muesebeck, 1958.

**Type information.** Holotype female, USNM (examined). Country of type locality: Colombia.

**Geographical distribution.**NEO.

**NEO**: Colombia.

***Dasylagonsimulans* Muesebeck, 1958**

*Dasylagonsimulans* Muesebeck, 1958.

**Type information.** Holotype male, USNM (examined). Country of type locality: Honduras.

**Geographical distribution.**NEO.

**NEO**: Brazil (BA), Honduras.

#### Genus Deuterixys Mason, 1981

***Deuterixys*** Mason, 1981: 123. Gender: feminine. Type species: *Microgastercarbonarius* Wesmael, 1837, by original designation (Mason 1981: 123).

Known from 18 described species from all biogeographical regions except for Africa (the lack of species recorded from the Afrotropical region is probably due to insufficient collecting and study there). Several revisions of the genus are available for the Nearctic (Whitfield 1985), Neotropics (Whitfield et al. 2004), Russia (Kotenko 2007a), and China (Zeng et al. 2011a). We have seen a few additional species in collections but the genus does not seem to be very large. The vast majority of the known host records belong to the family Bucculatricidae, the few other families cited in older literature must be considered as likely to be wrong. There are 30 DNA-barcode compliant sequences of this genus in BOLD, representing seven BINs.

***Deuterixysanica* Austin & Dangerfield, 1992**

*Deuterixysanica* Austin & Dangerfield, 1992.

**Type information.** Holotype female, ANIC (not examined but original description checked). Country of type locality: Australia.

**Geographical distribution.**AUS.

**AUS**: Australia (NSW, QLD, VIC).

***Deuterixysbennetti* Whitfield, 1985**

*Deuterixysbennetti* Whitfield, 1985.

**Type information.** Holotype female, USNM (examined). Country of type locality: USA.

**Geographical distribution.**NEA, NEO.

**NEA**: USA (FL); **NEO**: Cuba, Dominican Republic, Jamaica.

***Deuterixysbifossalis* Zeng & Chen, 2011**

*Deuterixysbifossalis* Zeng & Chen, 2011.

**Type information.** Holotype female, ZJUH (not examined but original description checked). Country of type locality: China.

**Geographical distribution.**OTL.

**OTL**: China (HI, ZJ).

***Deuterixyscarbonaria* (Wesmael, 1837)**

*Microgastercarbonarius* Wesmael, 1837.

*Apantelesanomalus* Lyle, 1925.

**Type information.** Lectotype female, RBINS (not examined but subsequent treatment of the species checked). Country of type locality: Belgium.

**Geographical distribution.**PAL.

**PAL**: Austria, Belgium, Czech Republic, Finland, France, Germany, Hungary, Italy, Japan, Korea, Lithuania, Mongolia, Netherlands, Poland, Romania, Russia (MOS, PRI, SAK, YAR), Slovenia, United Kingdom, Yugoslavia.

**Notes.** Our species concept is based on Nixon (1965), Papp (1983a), and Zeng et al. (2011a). The species distribution in Japan is based on Belokobylskij et al. (2019).

***Deuterixyscolombiana* Whitfield & Oltra, 2005**

*Deuterixyscolombiana* Whitfield & Oltra, 2005.

**Type information.** Holotype female, CNC (examined). Country of type locality: Colombia.

**Geographical distribution.**NEO.

**NEO**: Colombia, Ecuador, Peru.

***Deuterixyscondarensis* (Tobias, 1960)**

*Apantelescondarensis* Tobias, 1960.

*Apantelesnixoni* Papp, 1971.

**Type information.** Holotype female, ZIN (not examined but subsequent treatment of the species checked). Country of type locality: Tajikistan.

**Geographical distribution.**PAL.

**PAL**: Japan, Korea, Mongolia, Russia (PRI), Tajikistan.

**Notes.** Our species concept is based on Papp (1983a), Kotenko (2007a), and Zeng et al. (2011a). The species distribution in Japan is based on Belokobylskij et al. (2019).

***Deuterixyscurticalcar* Zeng & Chen, 2011**

*Deuterixyscurticalcar* Zeng & Chen, 2011.

**Type information.** Holotype female, ZJUH (not examined but original description checked). Country of type locality: China.

**Geographical distribution.**OTL, PAL.

**OTL**: China (GD, GZ, HN, JX, YN); **PAL**: China (HA, NX).

***Deuterixyserythrocephala* Whitfield & Oltra, 2005**

*Deuterixyserythrocephala* Whitfield & Oltra, 2005.

**Type information.** Holotype female, CNC (examined). Country of type locality: Trinidad & Tobago.

**Geographical distribution.**NEO.

**NEO**: Argentina, Dominican Republic, Trinidad & Tobago.

***Deuterixyshansoni* Whitfield & Oltra, 2005**

*Deuterixyshansoni* Whitfield & Oltra, 2005.

**Type information.** Holotype female, ESUW (not examined but original description checked). Country of type locality: Costa Rica.

**Geographical distribution.**NEO.

**NEO**: Bolivia, Costa Rica.

***Deuterixyspacifica* Whitfield, 1985**

*Deuterixyspacifica* Whitfield, 1985.

**Type information.** Holotype female, USNM (examined). Country of type locality: USA.

**Geographical distribution.**NEA, NEO.

**NEA**: Canada (BC), USA (CA, NM, WY); **NEO**: Mexico.

***Deuterixyspatro* (Nixon, 1965)**

*Apantelespatro* Nixon, 1965.

**Type information.** Holotype female, USNM (examined). Country of type locality: Philippines.

**Geographical distribution.**OTL.

**OTL**: Philippines.

**Notes.** This species represents one of the smallest Microgastrinae so far described (body and fore wing lengths 1.6–1.7 mm).

***Deuterixysplugarui* (Tobias, 1975)**

*Apantelesplugarui* Tobias, 1975.

**Type information.** Holotype female, ZIN (not examined but subsequent treatment of the species checked). Country of type locality: Moldova.

**Geographical distribution.**PAL.

**PAL**: Hungary, Moldova, Russia (S), Ukraine, United Kingdom.

**Notes.** Our species concept is based on Papp (1983a), Kotenko (2007a), Zeng et al. (2011a), and Shaw (2012b).

***Deuterixysquercicola* Whitfield, 1985**

*Deuterixysquercicola* Whitfield, 1985.

**Type information.** Holotype female, USNM (examined). Country of type locality: USA.

**Geographical distribution.**NEA, NEO.

**NEA**: USA (CA); **NEO**: Mexico.

***Deuterixysrimulosa* (Niezabitowski, 1910)**

*Apantelesrimulosus* Niezabitowski, 1910.

*Apantelescomes* Wilkinson, 1940.

**Type information.** Syntypes female and male, depository unknown (not examined but authoritatively identified specimens examined). Country of type locality: Poland.

**Geographical distribution.**PAL.

**PAL**: Azerbaijan, Croatia, Germany, Greece, Hungary, Iran, Kazakhstan, Mongolia, Poland, Russia (VOR), Slovakia, Spain, Turkmenistan, United Kingdom, Uzbekistan.

**Notes.** We examined the type of *Apantelescomes* Wilkinson, 1940. The species distribution in Turkmenistan is based on Belokobylskij et al. (2019).

***Deuterixyssvetlanae* Kotenko, 2007**

*Deuterixyssvetlanae* Kotenko, 2007.

**Type information.** Holotype female, SIZK (not examined but original description checked). Country of type locality: Russia.

**Geographical distribution.**PAL.

**PAL**: Russia (PRI).

***Deuterixystehuantepeca* Whitfield & Oltra, 2005**

*Deuterixystehuantepeca* Whitfield & Oltra, 2005.

**Type information.** Holotype female, CNC (examined). Country of type locality: Guatemala.

**Geographical distribution.**NEO.

**NEO**: Guatemala, Mexico.

***Deuterixystenuiconvergens* Zargar & Gupta, 2019**

*Deuterixystenuiconvergens* Zargar & Gupta, 2019.

**Type information.** Holotype female, NBAIR (not examined but original description checked). Country of type locality: Iran.

**Geographical distribution.**PAL.

**PAL**: Iran.

***Deuterixysx-formis* Papp, 2012**

*Deuterixysx-formis* Papp, 2012.

**Type information.** Holotype female, RMNH (not examined but original description checked). Country of type locality: Cape Verde.

**Geographical distribution.**AFR.

**AFR**: Cape Verde.

#### Genus Diolcogaster Ashmead, 1900

***Diolcogaster*** Ashmead, 1900: 132. Gender: feminine. Type species: *Microgasterbrevicaudus* Provancher, 1886, by subsequent designation and monotypy (Viereck 1914: 46).

*Zadiolcogaster* Viereck, 1913: 366. Type species: *Zadiolcogasteranomus* Viereck, 1913, by original designation.

A cosmopolitan genus, with 141 described species known from all biogeographical regions of the planet. Relatively recent revisions of the genus are available for the Australasian region (Saeed et al. 1999), Russia (Kotenko 2007a), China (Zeng et al. 2011b), and India (Gupta & Fernandez-Triana 2015), but overall the taxonomic coverage of the world species is far from complete. We have seen hundreds of undescribed species in collections, mostly from tropical areas. This is one of the most variable genera of Microgastrinae and, as currently defined, it is certainly polyphyletic. Depending on the generic concept adopted following future phylogenetic studies of Microgastrinae, *Diolcogaster* may end up having several hundred species or even more than one thousand. Around 15 families of Lepidoptera have been recorded as hosts, but many records are likely to be incorrect and/or need further verification. There are almost 4,000 DNA-barcode compliant sequences of this genus in BOLD, representing 270 BINs, most of them from Costa Rica and Canada.

***Diolcogasterabdominalis* (Nees, 1834)**

*Microgasterabdominalis* Nees, 1834.

**Type information.** Holotype male, depository unknown (not examined but subsequent treatment of the species checked). Country of type locality: Germany.

**Geographical distribution.**PAL.

**PAL**: Azerbaijan, Belgium, France, Georgia, Germany, Hungary, Ireland, Israel, Italy, Kazakhstan, Korea, Macedonia, Moldova, Mongolia, Montenegro, Poland, Romania, Russia (BU, ZAB, PRI), Serbia, Slovakia, Spain, Switzerland, United Kingdom.

**Notes.** Our species concept is based on Nixon (1965), Kotenko (2007), Shaw et al. (2009), and Shaw (2012b). The species distribution in Israel is based on Belokobylskij et al. (2019).

***Diolcogasterabengouroui* (Risbec, 1951), new combination**

*Microgasterabengouroui* Risbec, 1951.

**Type information.** Syntypes male, depository unknown (not examined but original description checked). Country of type locality: Ivory Coast.

**Geographical distribution.**AFR.

**AFR**: Ivory Coast.

**Notes.** The original description includes a drawing of propodeum, T1, and T2, which clearly shows that this species belongs to *Diolcogaster*.

***Diolcogasteradiastola* Saeed, Austin & Dangerfield, 1999**

*Diolcogasteradiastola* Saeed, Austin & Dangerfield, 1999.

**Type information.** Holotype female, ANIC (not examined but original description checked). Country of type locality: Australia.

**Geographical distribution.**AUS.

**AUS**: Australia (ACT, NSW, QLD, TAS).

***Diolcogasteragama* (de Saeger, 1944), new combination**

*Microgasteragama* de Saeger, 1944.

**Type information.** Holotype female, RMCA (not examined but original description checked). Country of type locality: Democratic Republic of Congo.

**Geographical distribution.**AFR.

**AFR**: Democratic Republic of Congo.

**Notes.** The original description and key place this species in the *basimacula* group, defined by T1–T3 forming a carapace (e.g., Mason 1981, Saeed et al. 1999, Fernandez-Triana 2015).

***Diolcogasteralce* (Nixon, 1965)**

*Protomicroplitisalce* Nixon, 1965.

**Type information.** Holotype female, NHMUK (examined). Country of type locality: Brazil.

**Geographical distribution.**NEO.

**NEO**: Brazil (SC).

***Diolcogasteralkingara* Saeed, Austin & Dangerfield, 1999**

*Diolcogasteralkingara* Saeed, Austin & Dangerfield, 1999.

**Type information.** Holotype female, CNC (examined). Country of type locality: Papua New Guinea.

**Geographical distribution.**AUS.

**AUS**: Australia (QLD), Papua New Guinea.

***Diolcogasteralvearia* (Fabricius, 1798)**

*Ichneumonalvearius* Fabricius, 1798.

*Ichneumonaleuarius* Fabricius, 1798 [incorrect original spelling].

*Anomalonaphidum* Panzer, 1804.

*Ichneumonalveator* Thunberg, 1822.

*Microgasterbicolor* Curtis, 1830.

*Apantelesareolata* Szépligeti, 1896.

**Type information.** Syntypes sex undetermined, ZMUK (not examined but original description checked). Country of type locality: France.

**Geographical distribution.**PAL.

**PAL**: Austria, Bulgaria, China (GS), Croatia, France, Germany, Greece, Hungary, Iran, Israel, Italy, Moldova, Netherlands, Romania, Russia (KDA, MOS), Slovakia, Slovenia, Spain, Switzerland, Turkey, United Kingdom, Yugoslavia.

**Notes.** The original description mentions two specimens, but no details of their sex is provided. The original species name (*alvearius*, currently *alvearia*), was misspelled in the original description as *aleuarius*, and it was also subsequently misspelled in a variety of ways, e.g., *aluearius*, *alevarius*, and even *alveolaris* (see Yu et al. 2016 for a compilation of references on those misspellings). The species distribution in Israel is based on Belokobylskij et al. (2019).

***Diolcogasterambositrensis* (Granger, 1949), new combination**

*Microgasterambositrensis* Granger, 1949.

**Type information.** Holotype female, MNHN (not examined but original description checked). Country of type locality: Madagascar.

**Geographical distribution.**AFR.

**AFR**: Madagascar.

**Notes.** Transferred to *Diolcogaster* based on the illustrations of the fore wing and T1–T3 provided in the original description.

***Diolcogasteranandra* (de Saeger, 1944), new combination**

*Microgasteranandra* de Saeger, 1944.

**Type information.** Holotype female, RMCA (not examined but original description checked). Country of type locality: Democratic Republic of Congo.

**Geographical distribution.**AFR.

**AFR**: Democratic Republic of Congo.

**Notes.** Based on the details provided in the original description, the best generic placement would be in *Diolcogaster*.

***Diolcogasterandamanensis* Gupta & Fernandez-Triana, 2015**

*Diolcogasterandamanensis* Gupta & Fernandez-Triana, 2015.

**Type information.** Holotype female, NBAIR (examined). Country of type locality: India.

**Geographical distribution.**OTL.

**OTL**: India.

***Diolcogasterannulata* (Granger, 1949), new combination**

*Microgasterannulata* Granger, 1949.

**Type information.** Holotype female, MNHN (not examined but original description checked). Country of type locality: Madagascar.

**Geographical distribution.**AFR.

**AFR**: Madagascar.

**Notes.** Transferred to *Diolcogaster* based on the illustrations of the fore wing and T1–T3 provided in the original description.

***Diolcogasteranoma* (Viereck, 1913)**

*Zadiolcogasteranomus* Viereck, 1913.

**Type information.** Holotype female, ZMHB (not examined but subsequent treatment of the species checked). Country of type locality: Paraguay.

**Geographical distribution.**NEO.

**NEO**: Paraguay.

**Notes.** Our species concept is based on Mason (1981). The species name must be treated as an adjective and not as a noun (Doug Yanega, pers. comm.) and thus it must match the gender of the genus name.

***Diolcogasterashmeadi* Saeed, Austin & Dangerfield, 1999**

*Diolcogasterashmeadi* Saeed, Austin & Dangerfield, 1999.

**Type information.** Holotype female, ANIC (not examined but original description checked). Country of type locality: Australia.

**Geographical distribution.**AUS.

**AUS**: Australia (NSW, QLD, TAS, VIC).

***Diolcogasteraurangabadensis* Fernandez-Triana, 2019, new replacement name**

*Protomicroplitisindicus* Rao & Chalikwar, 1970.

*Diolcogasterindica* (Rao & Chalikwar, 1970) [secondary homonym of *Diolcogasterindica* (Wilkinson, 1927)].

**Type information.** Holotype female, NZSI (not examined but original description checked). Country of type locality: India.

**Geographical distribution.**OTL.

**OTL**: India.

**Notes.***Diolcogasterindica* (Rao & Chalikwar, 1970) is a secondary homonym of *Diolcogasterindica* (Wilkinson, 1927). The replacement name refers to the city where the holotype was collected.

***Diolcogasterauripes* (Provancher, 1886)**

*Microgasterauripes* Provancher, 1886.

**Type information.** Lectotype female, ULQC (not examined but subsequent treatment of the species checked). Country of type locality: Canada.

**Geographical distribution.**NEA.

**NEA**: Canada (NB, ON, QC), USA (IL, IA, KS, KY, MD, MI, MO, NE, NJ, NY, OH, VA).

**Notes.** Our species concept is based on Muesebeck (1922), Nixon (1965), and Fernandez-Triana (2010).

***Diolcogasteraustrina* (Wilkinson, 1929)**

*Microgasteraustrina* Wilkinson, 1929.

**Type information.** Holotype female, NHMUK (examined). Country of type locality: South Africa.

**Geographical distribution.**AFR.

**AFR**: Cameroon, Cape Verde, Democratic Republic of Congo, Ivory Coast, Réunion, Rwanda, South Africa, Uganda.

**Notes.** Madl & van Achterberg (2014) noted of this species: “Known from the Afrotropical Region. As the record from Réunion is probably a misidentification (Rousse and Gupta 2013: 519), the material should be checked”. However, the species has been found in other countries from the region (e.g., Wilkinson 1927, 1929, de Saeger 1944), information we accept and follow here.

***Diolcogasterbakeri* (Muesebeck, 1922)**

*Microgasterbakeri* Muesebeck, 1922.

**Type information.** Holotype female, USNM (not examined but subsequent treatment of the species checked). Country of type locality: USA.

**Geographical distribution.**NEA, NEO.

**NEA**: Canada (ON, QC, SK), USA (AR, FL, GA, IL, IA, KS, LA, TX); **NEO**: Peru.

**Notes.** Our species concept is based on Muesebeck (1922), Mason (1981), Whitfield (1995a), and Fernandez-Triana (2010).

***Diolcogasterbambeyi* (Risbec, 1951), new combination**

*Microgasterbambeyi* Risbec, 1951.

**Type information.** Syntypes female and male, depository unknown (not examined but original description checked). Country of type locality: Senegal.

**Geographical distribution.**AFR.

**AFR**: Senegal.

**Notes.** From the original description (and drawings there of propodeum, T1 and part of the fore wing) it is clear this species belongs to *Diolocogaster*.

***Diolcogasterbasimacula* (Cameron, 1905)**

*Apantelesbasimacula* Cameron, 1905.

**Type information.** Syntypes male, NHMUK (examined). Country of type locality: South Africa.

**Geographical distribution.**AFR.

**AFR**: Democratic Republic of Congo, Madagascar, South Africa.

**Notes.** We have examined the two male specimens (mentioned by Wilkinson 1929a: 103) that were both labelled as Type in Cameron's writing. Those specimens are mounted on separate cards but share the same code in the NHMUK: 3c.988, and are from the type locality (Grahamstown) which was also mentioned by Wilkinson. We consider those two specimens to be syntypes. Additionally, we examined a third male specimen, also in the type collection of the NHMUK, and with code 3c.986; that specimen is mentioned by Wilkinson to have an additional label (Stellenbosch) not written by Cameron, and most likely was not part of the type series in the original description.

***Diolcogasterbelokobylskiji* Kotenko, 2007**

*Diolcogasterbelokobylskiji* Kotenko, 2007.

**Type information.** Holotype female, ZIN (not examined but original description checked). Country of type locality: Russia.

**Geographical distribution.**PAL.

**PAL**: Russia (PRI).

***Diolcogasterbicolorina* (Shenefelt, 1973), new combination**

*Microgasterbicolorinus* Shenefelt, 1973.

*Microgasterbicolor* Szépligeti, 1911 [primary homonym of *Microgasterbicolor* Nees, 1834].

**Type information.** Holotype female, ZMHB (not examined but original description checked). Country of type locality: Kenya.

**Geographical distribution.**AFR.

**AFR**: Kenya, Tanzania.

**Notes.** Based on the sculpture pattern of T1 and T2, as well as the setae on the ovipositor sheaths, which are clearly depicted in the redescription of the species (Wilkinson 1929a), the species belongs in *Diolcogaster*.

***Diolcogasterbifurcifossa* Zeng & Chen, 2011**

*Diolcogasterbifurcifossa* Zeng & Chen, 2011.

**Type information.** Holotype female, ZJUH (not examined but original description checked). Country of type locality: China.

**Geographical distribution.**OTL.

**OTL**: China (FJ, GD, GX, HI, ZJ), Japan, Vietnam.

***Diolcogasterbrevicaudus* (Provancher, 1886)**

*Microgasterbrevicaudus* Provancher, 1886.

**Type information.** Lectotype female, ULQC (not examined but subsequent treatment of the species checked). Country of type locality: Canada.

**Geographical distribution.**NEA.

**NEA**: Canada (QC), USA (IL, NJ, NY, PA).

**Notes.** Our species concept is based on Muesebeck (1922), Mason (1981), Whitfield (1995a), and Fernandez-Triana (2010). Because the name is to be considered as a noun under ICZN Article 31.2.1, it must retain its original spelling and remain as *brevicaudus*.

***Diolcogasterbreviterebrus* (Rao & Chalikwar, 1970)**

*Protomicroplitisbreviterebrus* Rao & Chalikwar, 1970.

**Type information.** Holotype female, NZSI (not examined but subsequent treatment of the species checked). Country of type locality: India.

**Geographical distribution.**OTL.

**OTL**: India.

**Notes.** Our species concept is based on Fernandez-Triana (2015).

***Diolcogasterbrevivena* Zeng & Chen, 2011**

*Diolcogasterbrevivena* Zeng & Chen, 2011.

**Type information.** Holotype female, ZJUH (not examined but original description checked). Country of type locality: China.

**Geographical distribution.**OTL.

**OTL**: China (YN).

***Diolcogastercariniger* (Granger, 1949), new combination**

*Microgastercariniger* Granger, 1949.

**Type information.** Syntypes female and male, MNHN (not examined but original description checked). Country of type locality: Madagascar.

**Geographical distribution.**AFR.

**AFR**: Madagascar.

**Notes.** Transferred to *Diolcogaster* based on the illustrations of the fore wing and T1–T3 provided in the original description.

***Diolcogasterchaoi* (Luo & You, 2003)**

*Caracallatuschaoi* Luo & You, 2003.

**Type information.** Holotype female, HUNAU (not examined but original description checked). Country of type locality: China.

**Geographical distribution.**OTL.

**OTL**: China (FJ, GZ, HI YN), Vietnam.

***Diolcogastercincticornis* (de Saeger, 1944), new combination**

*Microgastercincticornis* de Saeger, 1944.

**Type information.** Holotype female, RMCA (not examined but original description checked). Country of type locality: Democratic Republic of Congo.

**Geographical distribution.**AFR.

**AFR**: Democratic Republic of Congo.

**Notes.** Based on the details provided in the original description, the best generic placement would be in *Diolcogaster*.

***Diolcogastercingulata* (Granger, 1949), new combination**

*Microgastercingulata* Granger, 1949.

**Type information.** Holotype female, MNHN (not examined but original description checked). Country of type locality: Madagascar.

**Geographical distribution.**AFR.

**AFR**: Madagascar.

**Notes.** Transferred to *Diolcogaster* based on the illustrations of the fore wing and T1–T3 provided in the original description.

***Diolcogasterclaritibia* (Papp, 1959)**

*Microgasterclaritibia* Papp, 1959.

*Protomicroplitisorontes* Nixon, 1965.

**Type information.** Holotype female, HNHM (examined). Country of type locality: Hungary.

**Geographical distribution.**NEA, PAL.

**NEA**: Canada (AB, MB, ON); **PAL**: Afghanistan, Armenia, Austria, Azerbaijan, Belarus, Finland, Georgia, Greece, Hungary, Iran, Kazakhstan, Lithuania, Macedonia, Moldova, Russia (ZAB, KDA), Spain, Tunisia, Turkey, Turkmenistan, Ukraine, Yugoslavia.

**Notes.** We have also examined the type of *Protomicroplitisorontes* Nixon, 1965. The species distribution in Iran and Turkmenistan is based on Belokobylskij et al. (2019).

***Diolcogastercoenonymphae* (Watanabe, 1937)**

*Microgastercoenonymphae* Watanabe, 1937.

**Type information.** Holotype female, EIHU (examined). Country of type locality: Japan.

**Geographical distribution.**PAL.

**PAL**: Japan.

**Notes.**Nixon (1965) transferred *Microgastercoenonymphae* to *Protomicroplitis*, then Fernandez-Triana (2015) transferred it to *Diolcogaster*. Although Watanabe (1937a: 102) mentioned in the original description that there was a holotype and seven paratypes (all females, reared from the same caterpillar), we have examined the material and found four females from the type series, all with a small, thin label that reads Co-type; thus is not clear which specimens is the actual holotype.

***Diolcogasterconnexa* (Nees, 1834)**

*Microgasterconnexus* Nees, 1834.

*Microgasterconsularis* Haliday, 1834.

*Microgasterdiluta* Ratzeburg, 1852.

**Type information.** Type lost (not examined but subsequent treatment of the species checked). Country of type locality: Germany.

**Geographical distribution.**PAL.

**PAL**: Austria, Finland, France, Germany, Hungary, Italy, Korea, Netherlands, Poland, Romania, Russia (PRI, TY), Ukraine, United Kingdom.

**Notes.** Our species concept is based on Nixon (1965) and Kotenko (2007a). The type is presumed lost (Nixon 1965: 248). The country of the type locality is presumed by us to be Germany.

***Diolcogastercoronata* (de Saeger, 1944), new combination**

*Microgastercoronata* de Saeger, 1944.

**Type information.** Holotype female, RMCA (not examined but original description checked). Country of type locality: Rwanda.

**Geographical distribution.**AFR.

**AFR**: Democratic Republic of Congo.

**Notes.** Based on the details provided in the original description (de Saeger 1944), this species belongs to *Diolcogaster* and the *basimacula* species group within that genus. In the original description no details were provided on the etymology of the species name; as first revisers we consider it as a noun in apposition and thus its gender to be neuter, following Article 31.2.2 of the ICZN.

***Diolcogastercoxalis* (de Saeger, 1944), new combination**

*Microgastercoxalis* de Saeger, 1944.

**Type information.** Holotype female, RMCA (not examined but original description checked). Country of type locality: Democratic Republic of Congo.

**Geographical distribution.**AFR.

**AFR**: Democratic Republic of Congo.

**Notes.** We transfer here *coxalis* to *Diolcogaster* based on the details from original description and an illustration of the fore wing (partially) also provided there. This species was regarded by de Saeger (1944) as morphologically similar to *palpicolor*, another species described in the same paper and also transferred here to *Diolcogaster*, see more details under Notes for that species below.

***Diolcogastercurticornis* (Granger, 1949)**

*Microgastercurticornis* Granger, 1949.

**Type information.** Syntypes female and male, MNHN (not examined but subsequent treatment of the species checked). Country of type locality: Madagascar.

**Geographical distribution.**AFR.

**AFR**: Madagascar, Mauritius, Réunion.

**Notes.** Our species concept is based on Rousse and Gupta (2013).

***Diolcogasterdichromus* Saeed, Austin & Dangerfield, 1999**

*Diolcogasterdichromus* Saeed, Austin & Dangerfield, 1999.

**Type information.** Holotype female, ANIC (not examined but original description checked). Country of type locality: Australia.

**Geographical distribution.**AUS.

**AUS**: Australia (QLD).

***Diolcogasterdipika* (Bhatnagar, 1950) , new combination**

*Apantelesdipika* Bhatnagar, 1950.

**Type information.** Holotype male, lost (not examined but original description checked). Country of type locality: India.

**Geographical distribution.**OTL.

**OTL**: India.

**Notes.** This species was transferred to *Microplitis* (Rao 1953, Shenefelt 1973) but a recent paper (Gupta 2013a: 451) considered it as *incertae sedis*. However, the original description is detailed enough to prove that this species actually belongs to *Diolcogaster*. The illustrations of T1–T3 and the fore wing in Bhatnagar (1950: 136, 156) clearly belong to *Diolcogaster*, also the metatibia spurs are described as relatively long (inner spur three quarters and outer spur half the length of the first metatarsus segment), which would exclude the species from *Microplitis*. The year of publication of the Bhatnagar paper was until recently commonly cited as 1948 and/or 1950 (e.g., Chen and Song 2004, Yu et al. 2016), probably following Shenefelt (1972) who referred to this paper as “Bhatnagar (1948) 1950”. While the intended year for Volume X, Parts I & II of the Indian Journal of Entomology was 1948, the actual dates of publication were June 1950 (Part I) and October 1950 (Part II), as clearly shown on the cover page of the Volume, which we have checked. Because the dates of publication are the ones to be considered, and for the sake of clarity, we hereby revise the species year of description to 1950.

***Diolcogasterduocolor* Gupta & Fernandez-Triana, 2015**

*Diolcogasterduocolor* Gupta & Fernandez-Triana, 2015.

**Type information.** Holotype female, NBAIR (examined). Country of type locality: India.

**Geographical distribution.**OTL.

**OTL**: India.

***Diolcogasterduris* (Nixon, 1965)**

*Protomicroplitisduris* Nixon, 1965.

**Type information.** Holotype female, NHMUK (examined). Country of type locality: Mexico.

**Geographical distribution.**NEO.

**NEO**: Mexico.

***Diolcogasterearina* (Wilkinson, 1929), new combination**

*Microgasterearina* Wilkinson, 1929.

**Type information.** Holotype female, NHMUK (examined). Country of type locality: Nigeria.

**Geographical distribution.**AFR.

**AFR**: Democratic Republic of Congo, Nigeria.

**Notes.** We transfer this species to *Diolcogaster* based on the inflexible hypopygium, short ovipositor sheaths, T1 with a strong longitudinal sulcus, large metacoxae and metatibial spurs.

***Diolcogastereclectes* (Nixon, 1965)**

*Protomicroplitiseclectes* Nixon, 1965.

*Protomicroplitiseclectesextentus* Papp, 1974.

**Type information.** Holotype female, USNM (examined). Country of type locality: Philippines.

**Geographical distribution.**AUS, OTL, PAL.

**AUS**: Australia (QLD), Papua New Guinea; **OTL**: Malaysia, Philippines, Singapore; **PAL**: Korea.

**Notes.** Belongs to the *basimacula* species group.

***Diolcogasterepectina* (de Saeger, 1944), new combination**

*Microgasterepectina* de Saeger, 1944.

**Type information.** Holotype female, RMCA (not examined but original description checked). Country of type locality: Democratic Republic of Congo.

**Geographical distribution.**AFR.

**AFR**: Democratic Republic of Congo.

**Notes.** Based on the details provided in the original description, the best generic placement would be in *Diolcogaster* and, within that genus, in the *basimacula* species group.

***Diolcogasterepectinopsis* (de Saeger, 1944), new combination**

*Microgasterepectinopsis* de Saeger, 1944.

**Type information.** Holotype female, RMCA (not examined but original description checked). Country of type locality: Democratic Republic of Congo.

**Geographical distribution.**AFR.

**AFR**: Democratic Republic of Congo.

**Notes.** Based on the details provided in the original description, the best generic placement would be in *Diolcogaster* and, within that genus, in the *basimacula* species group.

***Diolcogastererro* (Nixon, 1965)**

*Protomicroplitiserro* Nixon, 1965.

**Type information.** Holotype female, NHMUK (examined). Country of type locality: Brazil.

**Geographical distribution.**NEO.

**NEO**: Brazil (SC).

**Notes.** This species belongs to the *Diolcogasterbasimacula* species group (Fernandez-Triana 2015).

***Diolcogastereuterpe* (Nixon, 1965)**

*Protomicroplitiseuterpe* Nixon, 1965.

**Type information.** Holotype female, NHMUK (examined). Country of type locality: Indonesia.

**Geographical distribution.**AUS.

**AUS**: Indonesia, Papua New Guinea.

**Notes.**Saeed et al. (1999) pointed out some of the unique features of this species, which may be transferred to a different genus following future studies of *Diolcogastersensu lato*.

***Diolcogasterfacetosa* (Weed, 1888)**

*Microgasterfacetosus* Weed, 1888.

*Microgastersolidaginis* Viereck, 1917.

**Type information.** Syntypes female and male, ANSP (not examined but subsequent treatment of the species checked). Country of type locality: USA.

**Geographical distribution.**NEA, OTL.

**NEA**: Canada (AB, BC, ON, QC), USA (AR, CO, CT, DE, GA, IL, IA, KS, KY, MD, MA, MI, MO, NH, NJ, NY, OH, OK, PA, SC, TN, VT, VA, WA, WV); **OTL**: China (FJ).

**Notes.** Our species concept is based on Nixon (1965), Chen and Song (2004) and Fernandez-Triana (2010). The latest version of Taxapad (Yu et al. 2016) lists the type material as deposited in the USNM. However, those specimens should be in the ANSP (e.g., Shenefelt 1972: 777). The online database on Hymenoptera Holotypes of the Smithsonian Institution (http://www.usnmhymtypes.com/default.asp) also confirms that the type material for this species is not in the USNM. We have also examined the type of *Microgastersolidaginis* (Viereck, 1917), a synonym of *D.facetosa*.

***Diolcogasterfasciipennis* (Gahan, 1918)**

*Microgasterfasciipennis* Gahan, 1918.

**Type information.** Holotype female, USNM (not examined but subsequent treatment of the species checked). Country of type locality: Uganda.

**Geographical distribution.**AFR.

**AFR**: Democratic Republic of Congo, Nigeria, Uganda.

**Notes.** Our species concept is based on Wilkinson (1929a), de Saeger (1944), and Nixon (1965).

***Diolcogasterflammea* Salgado-Neto & Fernandez-Triana, 2018**

*Diolcogasterflammeus* Salgado-Neto & Fernandez-Triana, 2018.

**Type information.** Holotype female, UFVB (examined). Country of type locality: Brazil.

**Geographical distribution.**NEO.

**NEO**: Brazil (MG).

**Notes.** The species name must be treated as an adjective and not as a noun (Doug Yanega, pers. comm.) and thus it must match the gender of the genus name.

***Diolcogasterflavipes* (Haliday, 1834)**

*Microgasterflavipes* Haliday, 1834.

**Type information.** Lectotype female, NMID (not examined but subsequent treatment of the species checked). Country of type locality: Ireland.

**Geographical distribution.**PAL.

**PAL**: Armenia, Austria, Finland, Germany, Hungary, Ireland, Italy, Poland, Russia (AMU, BU, ZAB, PRI, SAK, TY), United Kingdom.

**Notes.** Our species concept is based on van Achterberg (1997) and Kotenko (2007a). The species distribution in Armenia is based on Belokobylskij et al. (2019).

***Diolcogastergalazia* Kotenko, 2007**

*Diolcogastergalazia* Kotenko, 2007.

**Type information.** Holotype male, SIZK (not examined but original description checked). Country of type locality: Russia.

**Geographical distribution.**PAL.

**PAL**: Russia (ZAB).

***Diolcogastergarmani* (Ashmead, 1900)**

*Protomicroplitisgarmani* Ashmead, 1900.

*Protomicroplitisgermani* Ashmead, 1900 [incorrect original spelling].

**Type information.** Holotype female, USNM (not examined but subsequent treatment of the species checked). Country of type locality: USA.

**Geographical distribution.**NEA.

**NEA**: Canada (ON), USA (DC, IL, KS, KY, LA, MD, NY, TX, VA).

**Notes.** Our species concept is based on Muesebeck (1922), Mason (1981), Whitfield (1995a), and Fernandez-Triana (2010, 2015).

***Diolcogastergefidra* Kotenko, 2007**

*Diolcogastergefidra* Kotenko, 2007.

**Type information.** Holotype male, SIZK (not examined but original description checked). Country of type locality: Russia.

**Geographical distribution.**PAL.

**PAL**: Russia (PRI).

***Diolcogasterglaphyra* (de Saeger, 1944)**

*Microgasterglaphyra* de Saeger, 1944.

**Type information.** Holotype female, RMCA (not examined but subsequent treatment of the species checked). Country of type locality: Democratic Republic of Congo.

**Geographical distribution.**AFR.

**AFR**: Democratic Republic of Congo, Ethiopia.

**Notes.**Nixon (1965) transferred *Microgasterglaphyra* to *Protomicroplitis*, then Fernandez-Triana (2015) transferred it to *Diolcogaster*. It belongs to the *Diolcogasterbasimacula* species group (Fernandez-Triana 2015).

***Diolcogastergrammata* Zeng & Chen, 2011**

*Diolcogastergrammata* Zeng & Chen, 2011.

**Type information.** Holotype female, ZJUH (not examined but original description checked). Country of type locality: China.

**Geographical distribution.**OTL.

**OTL**: China (GD, HI, HN), Vietnam.

***Diolcogastergrangeri* (Shenefelt, 1973), new combination**

*Microgastergrangeri* Shenefelt, 1973.

*Microgastercrenulatus* Granger, 1949 [primary homonym of *Microgastercrenulatus* Provancher, 1888].

**Type information.** Syntypes female and male, MNHN (not examined but original description checked). Country of type locality: Madagascar.

**Geographical distribution.**AFR.

**AFR**: Madagascar.

**Notes.** The best generic placement of this species at this time is within *Diolcogaster*, based on the original description of the scutoscutellar sulcus, propodeum sculpture, fore wing venation, shape and sculpture of T1, and length of the ovipositor. However, the description does not closely match that of a typical *Diolcogaster*, which is a very variable genus; thus, further study of the type series will be required.

***Diolcogasterhadrommata* Saeed, Austin & Dangerfield, 1999**

*Diolcogasterhadrommatus* Saeed, Austin & Dangerfield, 1999.

**Type information.** Holotype female, ANIC (not examined but original description checked). Country of type locality: Australia.

**Geographical distribution.**AUS.

**AUS**: Australia (NSW, NT, QLD, SA, WA).

**Notes.** The species name must be treated as an adjective and not as a noun (Doug Yanega, pers. comm.) and thus it must match the gender of the genus name.

***Diolcogasterharrisi* Saeed, Austin & Dangerfield, 1999**

*Diolcogasterharrisi* Saeed, Austin & Dangerfield, 1999.

**Type information.** Holotype female, ANIC (not examined but original description checked). Country of type locality: Australia.

**Geographical distribution.**AUS.

**AUS**: Australia (NSW, TAS, VIC).

***Diolcogasterheterocera* (de Saeger, 1944), new combination**

*Microgasterheterocera* de Saeger, 1944.

*Microgasterheterocera* de Saeger, 1944 [primary homonym of *Microgasterheterocerus* Ruthe, 1860].

**Type information.** Holotype female, RMCA (not examined but original description checked). Country of type locality: Democratic Republic of Congo.

**Geographical distribution.**AFR.

**AFR**: Democratic Republic of Congo, Rwanda.

**Notes.** Based on the original description (de Saeger 1944), the best generic placement would be in *Diolcogaster*.

***Diolcogasterhinzi* (Nixon, 1965)**

*Protomicroplitishinzi* Nixon, 1965.

**Type information.** Holotype female, NHMUK (examined). Country of type locality: Germany.

**Geographical distribution.**PAL.

**PAL**: Finland, Germany, Hungary, Kazakhstan, Russia (KAM, SAK), United Kingdom.

**Notes.**Yu et al. (2016) recorded the holotype to be in Munich (ZSM); however, we have examined a specimen in the NHMUK that clearly is the type, including a handwritten label by Nixon stating so (type number: 3c.2114).

***Diolcogasterhomocera* (de Saeger, 1944), new combination**

*Microgasterhomocera* de Saeger, 1944.

**Type information.** Holotype female, RMCA (not examined but original description checked). Country of type locality: Democratic Republic of Congo.

**Geographical distribution.**AFR.

**AFR**: Democratic Republic of Congo.

**Notes.** Based on the original description (de Saeger 1944), the best generic placement would be in *Diolcogaster*.

***Diolcogasterichiroi* Fernandez-Triana, 2018**

*Diolcogasterichiroi* Fernandez-Triana, 2018.

**Type information.** Holotype female, CNC (examined). Country of type locality: USA.

**Geographical distribution.**NEA.

**NEA**: USA (FL).

***Diolcogasterindica* (Wilkinson, 1927), new combination**

*Microgasterindica* Wilkinson, 1927.

**Type information.** Holotype female, NHMUK (examined). Country of type locality: India.

**Geographical distribution.**OTL.

**OTL**: India, Indonesia, Myanmar.

**Notes.** We transfer this species to *Diolcogaster* based on the inflexible hypopygium, very short ovipositor sheaths with a few thickened setae at the apex, T1 with a strong longitudinal sulcus, and T2 with the median field clearly defined.

***Diolcogasterineminens* Zeng & Chen, 2011**

*Diolcogasterineminens* Zeng & Chen, 2011.

**Type information.** Holotype female, ZJUH (not examined but original description checked). Country of type locality: China.

**Geographical distribution.**OTL.

**OTL**: China (FJ, GD, ZJ), Vietnam.

***Diolcogasterinsularis* (Hedqvist, 1965), new combination**

*Microgasterinsularis* Hedqvist, 1965.

**Type information.** Holotype female, MZH (examined). Country of type locality: Cape Verde.

**Geographical distribution.**AFR.

**AFR**: Cape Verde.

**Notes.** A recent treatment of the species (Forshage et al. 2016) reported that the female holotype and male paratype were apparently deposited in the Lindberg collection (not MZH) and were apparently missing. However, we found those two specimens in the MZH collection and were able to study them. The species belongs to the genus *Diolcogaster*.

***Diolcogasterintegra* (Wilkinson, 1929)**

*Microgasterintegra* Wilkinson, 1929.

**Type information.** Holotype male, NHMUK (examined). Country of type locality: Uganda.

**Geographical distribution.**AFR.

**AFR**: Uganda.

**Notes.**Nixon (1965) transferred *Microgasterintegra* to *Protomicroplitis*, then Fernandez-Triana (2015) transferred it to the *basimacula* species group in the genus *Diolcogaster*.

***Diolcogasterippis* (Nixon, 1965)**

*Protomicroplitisippis* Nixon, 1965.

**Type information.** Holotype male, NHMUK (examined). Country of type locality: Brazil.

**Geographical distribution.**NEO.

**NEO**: Brazil (SC).

***Diolcogasteriqbali* Saeed, Austin & Dangerfield, 1999**

*Diolcogasteriqbali* Saeed, Austin & Dangerfield, 1999.

**Type information.** Holotype female, ANIC (not examined but original description checked). Country of type locality: Australia.

**Geographical distribution.**AUS.

**AUS**: Australia (NSW, NT, QLD, SA, WA).

***Diolcogasteriridescens* (Cresson, 1865)**

*Microgasteriridescens* Cresson, 1865.

**Type information.** Holotype female, ANSP (not examined but subsequent treatment of the species checked). Country of type locality: Cuba.

**Geographical distribution.**NEA, NEO.

**NEA**: USA (FL); **NEO**: Cuba.

**Notes.** Our species concept is based on Muesebeck (1922) and Mason (1981). Shenefelt (1973: 715) considered the holotype to be female; however, the last version of Taxapad (Yu et al. 2016) records the type as a male specimen.

***Diolcogasterkasachstanica* (Tobias, 1964)**

*Hygroplitiskasachstanica* Tobias, 1964.

**Type information.** Holotype female, ZIN (not examined but subsequent treatment of the species checked). Country of type locality: Kazakhstan.

**Geographical distribution.**PAL.

**PAL**: Kazakhstan, Russia (ZAB).

**Notes.** Our species concept is based on Tobias (1986) and Kotenko (2007a).

***Diolcogasterkasparyani* Kotenko, 2007**

*Diolcogasterkasparyani* Kotenko, 2007.

**Type information.** Holotype female, ZIN (not examined but original description checked). Country of type locality: Russia.

**Geographical distribution.**PAL.

**PAL**: Russia (YEV).

***Diolcogasterkivuana* (de Saeger, 1944), new combination**

*Microgasterkivuana* de Saeger, 1944.

**Type information.** Holotype female, RMCA (not examined but original description checked). Country of type locality: Democratic Republic of Congo.

**Geographical distribution.**AFR.

**AFR**: Democratic Republic of Congo.

**Notes.** Based on the original description (de Saeger 1944), the best generic placement would be in *Diolcogaster*.

***Diolcogasterlaetimedia* Zeng & Chen, 2011**

*Diolcogasterlaetimedia* Zeng & Chen, 2011.

**Type information.** Holotype female, ZJUH (not examined but original description checked). Country of type locality: China.

**Geographical distribution.**OTL.

**OTL**: China (FJ, GD, HI, ZJ), Vietnam.

***Diolcogasterlelaps* (Nixon, 1965)**

*Protomicroplitislelaps* Nixon, 1965.

**Type information.** Holotype female, NHMUK (examined). Country of type locality: Mexico.

**Geographical distribution.**NEO.

**NEO**: Mexico.

***Diolcogasterlongistria* Gupta & Fernandez-Triana, 2015**

*Diolcogasterlongistria* Gupta & Fernandez-Triana, 2015.

**Type information.** Holotype female, NBAIR (examined). Country of type locality: India.

**Geographical distribution.**OTL.

**OTL**: India.

***Diolcogasterlucindae* Saeed, Austin & Dangerfield, 1999**

*Diolcogasterlucindae* Saeed, Austin & Dangerfield, 1999.

**Type information.** Holotype female, AEIC (not examined but original description checked). Country of type locality: Australia.

**Geographical distribution.**AUS.

**AUS**: Australia (QLD, TAS).

***Diolcogastermalabarensis* Narendran & Sheeba, 2003**

*Diolcogastermalabarensis* Narendran & Sheeba, 2003.

**Type information.** Holotype female, NZSI (not examined but subsequent treatment of the species checked). Country of type locality: India.

**Geographical distribution.**OTL.

**OTL**: India.

**Notes.** Gupta & Fernandez-Triana (2015) mentioned the publication date of the original description as 2005, and other references online also consider 2005 as the publication year. However, the original paper, which we have examined, is dated 2003; and the online contents of the Journal of Bio-Sciences (https://www.banglajol.info/index.php/JBS) also indicate 2003 as the date for Volume 11, where the species was originally described.

***Diolcogastermasoni* Saeed, Austin & Dangerfield, 1999**

*Diolcogastermasoni* Saeed, Austin & Dangerfield, 1999.

**Type information.** Holotype female, ANIC (not examined but original description checked). Country of type locality: Australia.

**Geographical distribution.**AUS.

**AUS**: Australia (NSW, QLD).

***Diolcogastermayae* (Shestakov, 1932)**

*Microgastermayae* Shestakov, 1932.

*Microgasteriranensis* Hedwig, 1957.

**Type information.** Holotype female, depository unknown (not examined but subsequent treatment of the species checked). Country of type locality: unknown.

**Geographical distribution.**AFR, PAL.

**AFR**: Yemen; **PAL**: Afghanistan, Algeria, Armenia, Azerbaijan, Iran, Israel, Kazakhstan, Mongolia, Romania, Russia (NC, S), Tajikistan, Turkey, Turkmenistan, Uzbekistan.

**Notes.** Our species concept is based on Tobias (1986) and Kotenko (2007a). Shenefelt (1973: 716) considered the type to be a female specimen; he also recorded the type locality as Chiva: Ravatt. We believe that probably refers to the Ravat village, near the city of Khiva, in the Xorazm region of Uzbekistan. However, at present there is no certainty about the type locality. The published record of this species from Russia (Ghafouri Moghaddam et al. 2019) did not specify the subdivisions from that country where the specimens were collected.

***Diolcogastermediosulcata* (Granger, 1949), new combination**

*Microgastermediosulcatus* Granger, 1949.

**Type information.** Syntype female and male, MNHN (not examined but original description checked). Country of type locality: Madagascar.

**Geographical distribution.**AFR.

**AFR**: Madagascar.

**Notes.** Transferred to *Diolcogaster* based on the illustrations of the fore wing and T1–T3 provided in the original description.

***Diolcogastermedon* (Nixon, 1965)**

*Protomicroplitismedon* Nixon, 1965.

**Type information.** Holotype female, USNM (examined). Country of type locality: Philippines.

**Geographical distribution.**OTL.

**OTL**: Philippines.

**Notes.** This species belongs to the *Diolcogasterxanthaspis* species group (Fernandez-Triana 2015).

***Diolcogastermegaulax* (de Saeger, 1944), new combination**

*Microgastermegaulax* de Saeger, 1944.

**Type information.** Holotype female, RMCA (not examined but original description checked). Country of type locality: Democratic Republic of Congo.

**Geographical distribution.**AFR.

**AFR**: Democratic Republic of Congo.

**Notes.** Based on the original description, the best generic placement would be in *Diolcogaster*.

***Diolcogastermellea* (Nixon, 1965)**

*Protomicroplitismelleus* Nixon, 1965.

**Type information.** Holotype female, USNM (examined). Country of type locality: Philippines.

**Geographical distribution.**OTL.

**OTL**: Philippines.

**Notes.** This species belongs to the *Diolcogasterxanthaspis* species group (Fernandez-Triana 2015). The fore wing areolet is not visible, as veins are thickened in the area that the areolet should be (see original description for more details and drawing of fore wing). A mostly honey-yellow coloured species. The species name must be treated as an adjective and not as a noun (Doug Yanega, pers. comm.) and thus it must match the gender of the genus name.

***Diolcogastermerata* Saeed, Austin & Dangerfield, 1999**

*Diolcogastermerata* Saeed, Austin & Dangerfield, 1999.

**Type information.** Holotype female, AEIC (not examined but original description checked). Country of type locality: Papua New Guinea.

**Geographical distribution.**AUS.

**AUS**: Papua New Guinea.

***Diolcogastermiamensis* Fernandez-Triana, 2018**

*Diolcogastermiamensis* Fernandez-Triana, 2018.

**Type information.** Holotype female, CNC (examined). Country of type locality: USA.

**Geographical distribution.**NEA.

**NEA**: USA (FL).

***Diolcogasterminuta* (Reinhard, 1880)**

*Microgasterminutus* Reinhard, 1880.

**Type information.** Syntypes female and male, depository unknown (not examined but subsequent treatment of the species checked). Country of type locality: unknown.

**Geographical distribution.**PAL.

**PAL**: Armenia, Czech Republic, Finland, Germany, Lithuania, Poland, Romania, Russia (ZAB, IRK, TY, YAR), Switzerland, Turkey, Turkmenistan, Ukraine, United Kingdom.

**Notes.** Our species concept is based on Nixon (1968) and Tobias (1986). The species distribution in Turkey is based on Belokobylskij et al. (2019).

***Diolcogastermuzaffari* Saeed, Austin & Dangerfield, 1999**

*Diolcogastermuzaffari* Saeed, Austin & Dangerfield, 1999.

**Type information.** Holotype female, AEIC (not examined but original description checked). Country of type locality: Papua New Guinea.

**Geographical distribution.**AUS.

**AUS**: Papua New Guinea.

***Diolcogasternarendrani* Rema & Sheeba, 2004**

*Diolcogasternarendrani* Rema & Sheeba, 2004.

**Type information.** Holotype female, depository unknown (not examined but subsequent treatment of the species checked). Country of type locality: India.

**Geographical distribution.**OTL.

**OTL**: India.

**Notes.** Our species concept is based on Gupta & Fernandez-Triana (2015).

***Diolcogasternaumanni* Saeed, Austin & Dangerfield, 1999**

*Diolcogasternaumanni* Saeed, Austin & Dangerfield, 1999.

**Type information.** Holotype female, ANIC (not examined but original description checked). Country of type locality: Australia.

**Geographical distribution.**AUS.

**AUS**: Australia (WA).

***Diolcogasterneglecta* (de Saeger, 1944), new combination**

*Microgasterneglecta* de Saeger, 1944.

**Type information.** Holotype female, RMCA (not examined but original description checked). Country of type locality: Rwanda.

**Geographical distribution.**AFR.

**AFR**: Democratic Republic of Congo, Rwanda.

**Notes.** Based on the details provided in the original description, the best generic placement would be in *Diolcogaster* and, within that genus, in the *basimacula* species group. In the original description, no details were provided on the etymology of the species name; as first revisers we consider it as a noun in apposition and thus its gender to be neuter, following Article 31.2.2 of the ICZN.

***Diolcogasternephele* (Nixon, 1965)**

*Protomicroplitisnephele* Nixon, 1965.

**Type information.** Holotype female, NHMUK (examined). Country of type locality: Brazil.

**Geographical distribution.**NEO.

**NEO**: Brazil (SC).

**Notes.** It belongs to the *Diolcogasterbasimacula* species group (Fernandez-Triana 2015).

***Diolcogasternewguineaensis* Saeed, Austin & Dangerfield, 1999**

*Diolcogasternewguineaensis* Saeed, Austin & Dangerfield, 1999.

**Type information.** Holotype female, AEIC (not examined but original description checked). Country of type locality: Papua New Guinea.

**Geographical distribution.**AUS.

**AUS**: Papua New Guinea.

***Diolcogasternigromacula* (de Saeger, 1944), new combination**

*Microgasternigromacula* de Saeger, 1944.

**Type information.** Holotype female, RMCA (not examined but original description checked). Country of type locality: Democratic Republic of Congo.

**Geographical distribution.**AFR.

**AFR**: Democratic Republic of Congo.

**Notes.** Based on the original description, this species belongs to the *Diolcogasterbasimacula* species group. In the original description, no details were provided on the etymology of the species name; as first revisers we consider it as a noun in apposition and thus its gender to be neuter, following Article 31.2.2 of the ICZN.

***Diolcogasternixoni* Saeed, Austin & Dangerfield, 1999**

*Diolcogasternixoni* Saeed, Austin & Dangerfield, 1999.

**Type information.** Holotype female, AEIC (not examined but original description checked). Country of type locality: Papua New Guinea.

**Geographical distribution.**AUS.

**AUS**: Australia (QLD), Papua New Guinea.

***Diolcogasternotopecktos* Saeed, Austin & Dangerfield, 1999**

*Diolcogasternotopecktos* Saeed, Austin & Dangerfield, 1999.

**Type information.** Holotype female, AEIC (not examined but original description checked). Country of type locality: Australia.

**Geographical distribution.**AUS.

**AUS**: Australia (QLD), Papua New Guinea.

***Diolcogasterorientalis* (Rao & Chalikwar, 1970)**

*Protomicroplitisorientalis* Rao & Chalikwar, 1970.

**Type information.** Holotype female, NZSI (not examined but original description checked). Country of type locality: India.

**Geographical distribution.**OTL.

**OTL**: India.

***Diolcogasterpalpicolor* (de Saeger, 1944), new combination**

*Microgasterpalpicolor* de Saeger, 1944.

**Type information.** Holotype female, RMCA (not examined but original description checked). Country of type locality: Democratic Republic of Congo.

**Geographical distribution.**AFR.

**AFR**: Democratic Republic of Congo.

**Notes.** Based on the original description and illustrations provided there of T1–T3, fore wing (partially), apical half of metasoma and details of the ovipositor sheaths, this species clearly belongs in *Diolcogaster*. The hypopygium is inflexible, the ovipositor is short and strongly curved downwards, the ovipositor sheaths have strong setae apically, the T1 has a strong median sulcus, and the shape of the areolet in the fore wing is typical of the genus.

***Diolcogasterperiander* (Nixon, 1965)**

*Protomicroplitisperiander* Nixon, 1965.

**Type information.** Holotype female, USNM (examined). Country of type locality: Philippines.

**Geographical distribution.**OTL.

**OTL**: Philippines.

***Diolcogasterperniciosa* (Wilkinson, 1929)**

*Microgasterperniciosa* Wilkinson, 1929.

**Type information.** Holotype female, NHMUK (examined). Country of type locality: Australia.

**Geographical distribution.**AUS, OTL.

**AUS**: Australia (ACT, NSW, QLD, SA, TAS, VIC, WA), New Zealand; **OTL**: China (FJ, GZ, YN, ZJ).

***Diolcogasterpersimilis* (Wilkinson, 1929), new combination**

*Microgasterpersimilis* Wilkinson, 1929.

**Type information.** Holotype female, NHMUK (examined). Country of type locality: Uganda.

**Geographical distribution.**AFR.

**AFR**: Uganda.

**Notes.** We transfer this species to *Diolcogaster* based on the inflexible hypopygium, short ovipositor sheaths, T1 with a strong longitudinal sulcus, and large metacoxae and metatibial spurs. The metasoma and one hind leg of the holotype are in a gelatin capsule adjacent to the pinned specimen.

***Diolcogasterplecopterae* (Wilkinson, 1929), new combination**

*Microgasterplecopterae* Wilkinson, 1929.

**Type information.** Holotype female, NHMUK (examined). Country of type locality: India.

**Geographical distribution.**OTL.

**OTL**: India.

**Notes.** Transferred to *Diolcogaster* here based on its inflexible hypopygium, short ovipositor sheaths with some thickened setae on apex, T1 with a strong longitudinal sulcus, T2 with median field (although weakly defined), very large metacoxae, and large metatibial spurs.

***Diolcogasterpluriminitida* Zeng & Chen, 2011**

*Diolcogasterpluriminitida* Zeng & Chen, 2011.

**Type information.** Holotype female, ZJUH (not examined but original description checked). Country of type locality: China.

**Geographical distribution.**OTL.

**OTL**: China (GD, GZ, HN, ZJ), Vietnam.

***Diolcogasterplutocongoensis* (Shenefelt, 1973), new combination**

*Microgasterplutocongoensis* Shenefelt, 1973.

*Microgasterpluto* de Saeger, 1944 [primary homonym of *Microgasterpluto* Morley, 1936].

**Type information.** Holotype female, RMCA (not examined but original description checked). Country of type locality: Democratic Republic of Congo.

**Geographical distribution.**AFR.

**AFR**: Democratic Republic of Congo, Rwanda.

**Notes.** Transferred to *Diolcogaster* based on the original description and illustrations provided there. T1 has a strong median sulcus, T2 has a median field defined by sulci that narrow towards posterior margin of tergite, the hypopygium is inflexible, the ovipositor sheaths are short and at least with one thick seta apically, and the ovipositor is strongly curved downwards.

***Diolcogasterpraritas* Zeng & Chen, 2011**

*Diolcogasterpraritas* Zeng & Chen, 2011.

**Type information.** Holotype female, ZJUH (not examined but original description checked). Country of type locality: China.

**Geographical distribution.**OTL.

**OTL**: China (YN, ZJ).

***Diolcogasterprocris* (Fischer, 1964)**

*Microgasterprocris* Fischer, 1964.

**Type information.** Holotype female, MHNG (not examined but subsequent treatment of the species checked). Country of type locality: Austria.

**Geographical distribution.**PAL.

**PAL**: Austria.

**Notes.** Our species concept is based on Papp (1991b) and Shaw (2012b).

***Diolcogasterpsilocnema* (de Saeger, 1944), new combination**

*Microgasterpsilocnema* de Saeger, 1944.

**Type information.** Holotype male, RMCA (not examined but original description checked). Country of type locality: Democratic Republic of Congo.

**Geographical distribution.**AFR.

**AFR**: Democratic Republic of Congo, Rwanda.

**Notes.** Based on the original description, the best generic placement would be in *Diolcogaster*, based on the shapes of T1 and T2. However, the description is based on a male specimen only and it is not clear enough to conclude with certainty.

***Diolcogasterpunctata* (Rao & Chalikwar, 1976)**

*Protomicroplitispunctata* Rao & Chalikwar, 1976.

**Type information.** Holotype male, BAMU (not examined but subsequent treatment of the species checked). Country of type locality: India.

**Geographical distribution.**OTL.

**OTL**: India.

**Notes.** Our species concept is based on Gupta & Fernandez-Triana (2015).

***Diolcogasterpunctatiscutum* Zeng & Chen, 2011**

*Diolcogasterpunctatiscutum* Zeng & Chen, 2011.

**Type information.** Holotype female, ZJUH (not examined but original description checked). Country of type locality: China.

**Geographical distribution.**OTL.

**OTL**: China (GD).

***Diolcogasterpyrene* (Nixon, 1965)**

*Protomicroplitispyrene* Nixon, 1965.

**Type information.** Holotype female, USNM (examined). Country of type locality: Philippines.

**Geographical distribution.**OTL.

**OTL**: Philippines.

**Notes.** This species belongs to the *Diolcogasterxanthaspis* species group (Fernandez-Triana 2015).

***Diolcogasterreales* (Nixon, 1965)**

*Protomicroplitisreales* Nixon, 1965.

**Type information.** Holotype female, NHMUK (examined). Country of type locality: South Africa.

**Geographical distribution.**AFR.

**AFR**: South Africa.

***Diolcogasterrixosa* (Wilkinson, 1929)**

*Microgasterrixosa* Wilkinson, 1929.

**Type information.** Holotype female, NHMUK (examined). Country of type locality: Australia.

**Geographical distribution.**AUS.

**AUS**: Australia (ACT, NSW, QLD, SA, VIC, WA).

***Diolcogasterrobertsi* Saeed, Austin & Dangerfield, 1999**

*Diolcogasterrobertsi* Saeed, Austin & Dangerfield, 1999.

**Type information.** Holotype female, ANIC (not examined but original description checked). Country of type locality: Australia.

**Geographical distribution.**AUS.

**AUS**: Australia (QLD).

***Diolcogasterrufithorax* (Granger, 1949), new combination**

*Microgasterrufithorax* Granger, 1949.

**Type information.** Syntypes female, MNHN (not examined but illustrations of the holotype examined). Country of type locality: Madagascar.

**Geographical distribution.**AFR.

**AFR**: Madagascar.

**Notes.** The generic placement of this species has been determined based on information from the original description and low-resolution images of the holotype (taken with a cell phone) which we have examined. Transferred to *Diolcogaster* based on fore wing areolet, propodeum with median carina, large metacoxa and metatibial spurs, T1 with median sulcus, ovipositor sheaths short, and hypopygium inflexible.

***Diolcogasterrufula* Papp, 1991**

*Diolcogasterrufula* Papp, 1991.

**Type information.** Holotype female, HNHM (not examined but original description checked). Country of type locality: Hungary.

**Geographical distribution.**PAL.

**PAL**: Hungary.

***Diolcogasterrugosicoxa* (Papp, 1959)**

*Microgasterrugosicoxa* Papp, 1959.

*Protomicroplitismeges* Nixon, 1965.

**Type information.** Holotype female, HNHM (examined). Country of type locality: Hungary.

**Geographical distribution.**PAL.

**PAL**: Austria, Hungary, Italy, Romania, Switzerland.

**Notes.** We also examined the type of *Protomicroplitismeges* Nixon.

***Diolcogasterrugulosa* (Rao & Chalikwar, 1970)**

*Protomicroplitisrugulosus* Rao & Chalikwar, 1970.

**Type information.** Holotype male, NZSI (not examined but original description checked). Country of type locality: India.

**Geographical distribution.**OTL.

**OTL**: India.

**Notes.** The species name must be treated as an adjective and not as a noun (Doug Yanega, pers. comm.) and thus it must match the gender of the genus name.

***Diolcogasterschizurae* (Muesebeck, 1922)**

*Microgasterschizurae* Muesebeck, 1922.

**Type information.** Holotype female, USNM (not examined but original description checked). Country of type locality: USA.

**Geographical distribution.**NEA.

**NEA**: Canada (BC, ON), USA (AR, DC, KS, MD, MA, MI, NJ, OH, VA, WV).

***Diolcogasterscotica* (Marshall, 1885)**

*Microgasterscoticus* Marshall, 1885.

**Type information.** Holotype male, NHMUK (examined). Country of type locality: United Kingdom.

**Geographical distribution.**NEA, PAL.

**NEA**: Canada (BC, QC), USA (MI); **PAL**: Finland, Germany, Hungary, Mongolia, Poland, Romania, Russia (ZAB, IRK), Slovakia, Switzerland, United Kingdom.

***Diolcogastersemirufa* (de Saeger, 1944), new combination**

*Microgastersemirufa* de Saeger, 1944.

**Type information.** Holotype female, RMCA (not examined but original description checked). Country of type locality: Democratic Republic of Congo.

**Geographical distribution.**AFR.

**AFR**: Democratic Republic of Congo.

**Notes.** Based on the original description and illustrations provided there of the fore wing (partially) and posterior half of metasoma (de Saeger 1944: 79–80), the species is transferred to *Diolcogaster*, due to its propodeum with strong, median longitudinal carina, inflexible hypopygium and ovipositor sheaths with at least one thick seta on apex.

***Diolcogasterseriphus* (Nixon, 1965)**

*Protomicroplitisseriphus* Nixon, 1965.

**Type information.** Holotype female, USNM (examined). Country of type locality: Philippines.

**Geographical distribution.**OTL.

**OTL**: Philippines.

**Notes.**Kotenko (2007a: 163) transferred *Protomicroplitisseriphus* to *Diolcogaster*, although it was done within a key to species, without explicitly stating the new combination, and without placing the author’s name between parentheses. Yu et al. (2012: Taxapad) revised that combination, transferring the species back to *Protomicroplitis*. Fernandez-Triana (2015) then transferred *seriphus* back to *Diolcogaster*, as a member of the *xanthaspis* species group.

***Diolcogasterseyrigi* (Granger, 1949), new combination**

*Microgasterseyrigi* Granger, 1949.

**Type information.** Syntypes female, MNHN (not examined but original description checked). Country of type locality: Madagascar.

**Geographical distribution.**AFR.

**AFR**: Madagascar.

**Notes.** Transferred to *Diolcogaster* based on T1 with median longitudinal carina, T2 with delimited median field, fore wing with areolet, and short ovipositor sheaths (as detailed in the original description and illustrations there of T1–T3 and part of the fore wing).

***Diolcogastersolitaria* Gupta & Fernandez-Triana, 2015**

*Diolcogastersolitarium* Gupta & Fernandez-Triana, 2015.

**Type information.** Holotype female, NBAIR (examined). Country of type locality: India.

**Geographical distribution.**OTL.

**OTL**: India.

***Diolcogastersons* (Wilkinson, 1932)**

*Microgastersons* Wilkinson, 1932.

**Type information.** Holotype male, NHMUK (examined). Country of type locality: Australia.

**Geographical distribution.**AUS, OTL.

**AUS**: Australia (ACT, QLD, TAS, WA), New Caledonia; **OTL**: Indonesia.

**Notes.** This species belongs to the *Diolcogasterbasimacula* species group.

***Diolcogasterspreta* (Marshall, 1885)**

*Microgasterspreta* Marshall, 1885.

**Type information.** Type lost (not examined but subsequent treatment of the species checked). Country of type locality: United Kingdom.

**Geographical distribution.**PAL.

**PAL**: Bulgaria, China (SN), Denmark, Germany, Greece, Hungary, Iran, Moldova, Romania, Russia (NC, S), Slovakia, Spain, Turkey, United Kingdom.

**Notes.** Our species concept is based on Nixon (1965), Zeng et al. (2011b), and Ghafouri Moghaddam et al. (2019).

***Diolcogasterstepposa* (Tobias, 1964)**

*Hygroplitisstepposa* Tobias, 1964.

**Type information.** Holotype female, ZIN (not examined but subsequent treatment of the species checked). Country of type locality: Kazakhstan.

**Geographical distribution.**PAL.

**PAL**: Kazakhstan.

**Notes.** Our species concept is based on Nixon (1968) and Tobias (1986).

***Diolcogastersubtorquata* (Granger, 1949), new combination**

*Microgastersubtorquata* Granger, 1949.

**Type information.** Syntype female and male, MNHN (not examined but original description checked). Country of type locality: Madagascar.

**Geographical distribution.**AFR.

**AFR**: Madagascar.

**Notes.** Transferred to *Diolcogaster* based on T2 with delimited median field, fore wing with areolet, and short ovipositor sheaths (as detailed in the original description and illustrations there of T1–T3 and part of the fore wing).

***Diolcogastersulcata* (de Saeger, 1944), new combination**

*Microgastersulcata* de Saeger, 1944.

**Type information.** Holotype female, RMCA (not examined but original description checked). Country of type locality: Democratic Republic of Congo.

**Geographical distribution.**AFR.

**AFR**: Democratic Republic of Congo.

**Notes.** Based on the original description, this species belongs to the *Diolcogasterbasimacula* species group. In the original description no details were provided on the etymology of the species name; as first revisers we consider it as a noun in apposition and thus its gender to be neuter, following Article 31.2.2 of the ICZN.

***Diolcogastertearae* (Wilkinson, 1929)**

*Microgastertearae* Wilkinson, 1929.

**Type information.** Lectotype female, NHMUK (examined). Country of type locality: Australia.

**Geographical distribution.**AUS.

**AUS**: Australia (NSW, SA, VIC, WA).

**Notes.**Shenefelt (1973: 732) designated a female specimen as lectotype, from a type series that included three female specimens (Wilkinson 1929a: 107-108). However, Saeed et al. (1999: 167 and especially 169) wrongly mentioned a holotype, which they even considered to be the only known specimen of the species, until their revision added more specimens and localities. The host is also recorded as two potentially different species by Saeed et al. (1999); it is not clear if there is a reason for that, as their paper does not mention any extra host record. Thus, the host species associated with *D.tearae* need further verification.

***Diolcogastertegularia* (Papp, 1959)**

*Microgastertegularius* Papp, 1959.

**Type information.** Holotype female, HNHM (not examined but original description checked). Country of type locality: Hungary.

**Geographical distribution.**PAL.

**PAL**: Hungary.

***Diolcogastertomentosae* (Wilkinson, 1930)**

*Microgastertomentosae* Wilkinson, 1930.

**Type information.** Holotype female, NHMUK (examined). Country of type locality: India.

**Geographical distribution.**OTL.

**OTL**: India.

**Notes.**Watanabe (1937a: 101) recorded *Diolcogastertomentosae* from Taiwan, although he also noticed that the specimens from that area were slightly different from the original description of the species (which was based on Indian specimens). We have examined the material from Watanabe (deposited in Hokkaido) and it is clear that they represent a different species (to be eventually described). Therefore, we consider *D.tomentosae* to be present only in India.

***Diolcogastertorquatiger* (Granger, 1949), new combination**

*Microgastertorquatiger* Granger, 1949.

**Type information.** Holotype female, MNHN (not examined but illustrations of the holotype examined). Country of type locality: Madagascar.

**Geographical distribution.**AFR.

**AFR**: Madagascar.

**Notes.** The generic placement of this species has been determined based on information from the original description and low-resolution images of the holotype (taken with a cell phone) which we have examined. Transferred to *Diolcogaster* here based on fore wing areolet, propodeum with median longitudinal carina, metacoxa large, metatibial spurs relatively long, T1 with median sulcus, T2 with partially defined median field, hypopygium inflexible and ovipositor sheaths short.

***Diolcogastertranslucida* Zeng & Chen, 2011**

*Diolcogastertranslucida* Zeng & Chen, 2011.

**Type information.** Holotype female, ZJUH (not examined but original description checked). Country of type locality: China.

**Geographical distribution.**OTL, PAL.

**OTL**: China (FJ, GD, HN, ZJ), Vietnam; **PAL**: China (HA).

***Diolcogastertristiculus* (Granger, 1949), new combination**

*Microgastertristiculus* Granger, 1949.

**Type information.** Holotype female, MNHN (not examined but original description checked). Country of type locality: Madagascar.

**Geographical distribution.**AFR.

**AFR**: Madagascar.

**Notes.** Based on the original description and illustrations provided there of the fore wing (partially) and T1–T3 (Granger 1949: 222, 225–227), the species is transferred to *Diolcogaster*, due to its fore wing with areolet, inflexible hypopygium and ovipositor sheaths with at least one thick seta on apex.

***Diolcogastertropicalus* Saeed, Austin & Dangerfield, 1999**

*Diolcogastertropicalus* Saeed, Austin & Dangerfield, 1999.

**Type information.** Holotype female, ANIC (not examined but original description checked). Country of type locality: Australia.

**Geographical distribution.**AUS.

**AUS**: Australia (QLD), Papua New Guinea.

***Diolcogasterturneri* (Wilkinson, 1929), new combination**

*Microgasterturneri* Wilkinson, 1929.

**Type information.** Holotype female, NHMUK (examined). Country of type locality: South Africa.

**Geographical distribution.**AFR.

**AFR**: South Africa.

**Notes.** Transferred to *Diolcogaster* here based on its inflexible hypopygium, short ovipositor sheaths with some thickened setae at the apex, T1 with a strong longitudinal sulcus, large metacoxae, and large metatibial spurs.

***Diolcogasterurios* (Nixon, 1965)**

*Protomicroplitisurios* Nixon, 1965.

**Type information.** Holotype female, USNM (examined). Country of type locality: Malaysia.

**Geographical distribution.**OTL.

**OTL**: Malaysia.

**Notes.** This species belongs to the *Diolcogasterxanthaspis* species group (Fernandez-Triana 2015).

***Diolcogastervulcana* (de Saeger, 1944), new combination**

*Microgastervulcana* de Saeger, 1944.

**Type information.** Holotype female, RMCA (not examined but original description checked). Country of type locality: Democratic Republic of Congo.

**Geographical distribution.**AFR.

**AFR**: Democratic Republic of Congo, Rwanda.

**Notes.** Based on the original description, the best generic placement would be in *Diolcogaster*.

***Diolcogastervulpina* (Wilkinson, 1929)**

*Microgastervulpina* Wilkinson, 1929.

**Type information.** Holotype female, NHMUK (examined). Country of type locality: Australia.

**Geographical distribution.**AUS.

**AUS**: Australia (NSW, NT, QLD, SA, VIC, WA).

***Diolcogasterwalkerae* Saeed, Austin & Dangerfield, 1999**

*Diolcogasterwalkerae* Saeed, Austin & Dangerfield, 1999.

**Type information.** Holotype female, MCZC (not examined but original description checked). Country of type locality: Australia.

**Geographical distribution.**AUS.

**AUS**: Australia (NSW, WA).

***Diolcogasterwittei* (de Saeger, 1944), new combination**

*Microgasterwittei* de Saeger, 1944.

**Type information.** Holotype male, RMCA (not examined but original description checked). Country of type locality: Democratic Republic of Congo.

**Geographical distribution.**AFR.

**AFR**: Democratic Republic of Congo, Rwanda.

**Notes.** Based on the original description, the best generic placement would be in *Diolcogaster*. However, the description mentions that the ovipositor sheaths lack “appendix” (from the original description: “dépourvues d'appendice”) which could be interpreted as lacking setae, at least on the apex of the sheaths. The genus *Rasivalva* looks like *Diolcogaster* except for the ovipositor sheaths lacking visible setae, thus there is a small chance that this species could actually belong to *Rasivalva*. Without examining specimens, it is impossible to conclude, but because at present there is only one *Rasivalva* species recorded from the Afrotropical region (versus dozens of *Diolcogaster*), the probability of the species belonging to *Rasivalva* is much smaller.

***Diolcogasterxanthaspis* (Ashmead, 1900), lectotype designation**

*Apantelesxanthaspis* Ashmead, 1900.

**Type information.** Lectotype female, NHMUK (examined). Country of type locality: Saint Vincent.

**Geographical distribution.**NEO.

**NEO**: Grenada, Saint Vincent.

**Notes.** The original description (Ashmead 1900c: 280) was based on a series of two female and four male specimens. Later, Shenefelt (1973: 782) referred to the type as a female, with BMNH code 3c.992; however, he did not designate it as the lectotype (although Shenefelt did that for many other species dealt with in his catalogue of world Braconidae). For the sake of completion, it is here formally designated. We have examined that female specimen, which is currently missing the head but otherwise is in reasonably good condition.

***Diolcogasteryousufi* Saeed, Austin & Dangerfield, 1999**

*Diolcogasteryousufi* Saeed, Austin & Dangerfield, 1999.

**Type information.** Holotype female, ANIC (not examined but original description checked). Country of type locality: Australia.

**Geographical distribution.**AUS.

**AUS**: Australia (ACT, NSW, QLD, SA, TAS, WA).

#### Genus Distatrix Mason, 1981

***Distatrix*** Mason, 1981: 93. Gender: feminine. Type species: *Apantelespapilionis* Viereck, 1912, by original designation.

A total of 32 described species from all biogeographical regions except for Australasia. The New World species were revised recently (Grinter et al. 2009), but the taxonomic coverage of the world species is far from complete. Based on additional specimens we have seen in collections, the genus may well exceed 50 species. Nine families of Lepidoptera, mainly Geometridae and Papilionidae, have been recorded as hosts of *Distatrix*; some of those families may prove to be wrong. There are 50 DNA-barcode compliant sequences of this genus in BOLD, representing 22 BINs.

***Distatrixanthedon* (Nixon, 1965), new combination**

*Apantelesanthedon* Nixon, 1965.

**Type information.** Holotype female, NHMUK (examined). Country of type locality: South Africa.

**Geographical distribution.**AFR.

**AFR**: South Africa.

**Notes.** Transferred to *Distatrix* based on the pronotum laterally with a single furrow, T2 lateral sulcus only marked on the anterior half of the tergite, fore wing venation, and ovipositor sheaths with only very minute setae near the very apex. In the original description, Nixon (1965) did not provide any details on the etymology of the species name; as first revisers we consider it as a noun in apposition and thus its gender to be neuter, following Article 31.2.2 of the ICZN.

***Distatrixantirrheae* Whitfield & Grinter, 2009**

*Distatrixantirrheae* Whitfield & Grinter, 2009.

**Type information.** Holotype female, USNM (not examined but original description checked). Country of type locality: Ecuador.

**Geographical distribution.**NEO.

**NEO**: Ecuador.

***Distatrixbelliger* (Wilkinson, 1929)**

*Apantelesbelliger* Wilkinson, 1929.

**Type information.** Holotype female, NHMUK (examined). Country of type locality: Mauritius.

**Geographical distribution.**AFR.

**AFR**: Madagascar, Mauritius, Réunion.

***Distatrixcarolinae* Fernandez-Triana, 2010**

*Distatrixcarolinae* Fernandez-Triana, 2010.

**Type information.** Holotype female, CNC (examined). Country of type locality: Canada.

**Geographical distribution.**NEA.

**NEA**: Canada (QC).

***Distatrixcerales* (Nixon, 1965), new combination**

*Apantelescerales* Nixon, 1965.

**Type information.** Holotype female, NHMUK (examined). Country of type locality: Uganda.

**Geographical distribution.**AFR.

**AFR**: Uganda.

**Notes.** Transferred to *Distatrix* based on the pronotum laterally with a single furrow, T2 lateral sulcus only marked on the anterior half of the tergite, fore wing venation, and ovipositor sheaths with only very minute setae near the very apex.

***Distatrixcuspidalis* (de Saeger, 1944), new combination**

*Apantelescuspidalis* de Saeger, 1944.

**Type information.** Holotype female, RMCA (not examined but original description checked). Country of type locality: Democratic Republic of Congo.

**Geographical distribution.**AFR.

**AFR**: Democratic Republic of Congo.

**Notes.** Based on the original description and drawings, the best generic placement is *Distatrix*. However, it could also be *Glyptapanteles* and examination of the specimens would be needed to conclude.

***Distatrixeuproctidis* (Ullyett, 1946), new combination**

*Apanteleseuproctidis* Ullyett, 1946.

**Type information.** Holotype female, SAMC (not examined but original description checked). Country of type locality: South Africa.

**Geographical distribution.**AFR.

**AFR**: South Africa.

**Notes.** The original description strongly suggest that this species belongs to *Distatrix*, based on the entirely smooth propodeum, shape and sculpture of T1 and T2, and shape of the hypopygium. Ullyett (1946) also placed *euproctidis* close to another *Distatrix* species (in a modified couplet of a key from Wilkinson (1932a) that Ullyett discussed in his paper). Another plausible alternative could be *Glyptapanteles*; however, the distinguishing characters between these two genera (number of sulci on the pronotum laterally and setation pattern on ovipositor sheaths) were not detailed by Ullyett (1946). Although only a study of the type series can establish this unambiguously, we still consider *Distatrix* to be the best generic placement, based on T2 having lateral margins (diverging sulci) not extending to the apex of the tergite, a strong character of *Distatrix*.

***Distatrixflava* (Fernandez-Triana & van Achterberg, 2017), new combination**

*Venanidesflavus* Fernandez-Triana & van Achterberg, 2017.

**Type information.** Holotype female, RMNH (examined). Country of type locality: Yemen.

**Geographical distribution.**AFR.

**AFR**: Yemen.

**Notes.** This species clearly belongs to *Distatrix*, but it was wrongly placed within *Venanides* in the original description. The species name must be treated as an adjective and not as a noun (Doug Yanega, pers. comm.) and thus it must match the gender of the genus name.

***Distatrixformosa* (Wesmael, 1837)**

*Microgasterformosus* Wesmael, 1837.

*Apantelesmarshallii* Bignell, 1901.

**Type information.** Holotype female, RBINS (not examined but subsequent treatment of the species checked). Country of type locality: Belgium.

**Geographical distribution.**PAL.

**PAL**: Armenia, Belgium, France, Germany, Hungary, Italy, Japan, Korea, Netherlands, Poland, Romania, Russia (KEM, PRI), Switzerland, Ukraine, United Kingdom.

**Notes.** Our species concept is based on Nixon (1965, 1974), Papp (1983a), and Kotenko (2007a). Recent references treat this species as *Distatrix* (e.g., Mason 1981, Kotenko 2007a, Broad et al. 2016). But Taxapad (Yu et al. 2012, 2016) and references following Taxapad treat it as *Protapanteles*. For the sake of clarity we revise its combination here. The species distribution in is based on Belokobylskij et al. (2019).

***Distatrixgeometrivora* (de Saeger, 1944), new combination**

*Apantelesgeometrivora* de Saeger, 1941.

**Type information.** Holotype female, RMCA (not examined but original description checked). Country of type locality: Democratic Republic of Congo.

**Geographical distribution.**AFR.

**AFR**: Democratic Republic of Congo, Uganda.

**Notes.** Based on the original description (de Saeger 1941a) and that of Nixon (1965), the best generic placement would be in *Distatrix*. The original name of the species was spelled as *geometrivora* (de Saeger 1941a) although in a following paper, the same author spelled it as *geometrivorus* (de Saeger 1944).

***Distatrixgratiosa* (Wilkinson, 1930)**

*Apantelesgratiosus* Wilkinson, 1930.

**Type information.** Holotype female, NHMUK (examined). Country of type locality: Uganda.

**Geographical distribution.**AFR.

**AFR**: Democratic Republic of Congo, Uganda.

***Distatrixiglesiasi* (Viereck, 1913)**

*Apantelesiglesiasi* Viereck, 1913.

**Type information.** Holotype female, USNM (examined). Country of type locality: Brazil.

**Geographical distribution.**NEO.

**NEO**: Brazil (SP).

***Distatrixiraklii* (Kotenko, 1986)**

*Apantelesiraklii* Kotenko, 1986.

**Type information.** Holotype female, SIZK (not examined but original description checked). Country of type locality: Georgia.

**Geographical distribution.**PAL.

**PAL**: Georgia.

**Notes.**Papp (1988) had placed this species in the genus *Distatrix*, a decision we agree with after reading the original description and key provided in Kotenko (1986: 658) where several characters that define this genus (especially the pronotum laterally with a single, ventral furrow) are clearly stated.

***Distatrixloretta* Grinter, 2009**

*Distatrixloretta* Grinter, 2009.

**Type information.** Holotype female, USNM (not examined but original description checked). Country of type locality: Costa Rica.

**Geographical distribution.**NEO.

**NEO**: Costa Rica.

***Distatrixmaia* (Nixon, 1965), new combination**

*Apantelesmaia* Nixon, 1965.

**Type information.** Holotype female, NHMUK (examined). Country of type locality: South Africa.

**Geographical distribution.**AFR.

**AFR**: South Africa.

**Notes.** Transferred to *Distatrix* based on the pronotum laterally with a single furrow, T2 lateral sulcus marked only on the anterior half of the tergite, fore wing venation, and ovipositor sheaths with only very minute setae near the very apex.

***Distatrixmalloi* (Blanchard, 1942)**

*Apantelesmalloi* Blanchard, 1942.

**Type information.** Syntypes female and male, MACN (not examined but subsequent treatment of the species checked). Country of type locality: Argentina.

**Geographical distribution.**NEO.

**NEO**: Argentina.

**Notes.** Our species concept is based on Mason (1981).

***Distatrixpallidocinctus* (Gahan, 1918)**

*Apantelespallidocinctus* Gahan, 1918.

**Type information.** Holotype female, USNM (not examined but subsequent treatment of the species checked). Country of type locality: Uganda.

**Geographical distribution.**AFR.

**AFR**: Democratic Republic of Congo, Kenya, Uganda, Zimbabwe.

**Notes.** Our species concept is based on Nixon (1965) and Mason (1981). Under ICZN Article 31.2.2, this species name must be treated as a noun and thus retain its original spelling (Doug Yanega, pers. comm.).

***Distatrixpandora* Grinter, 2019**

*Distatrixpandora* Grinter, 2019.

*Distatrixpandora* Grinter, 2009 [unavailable name].

**Type information.** Holotype female, MIUP (not examined but original description checked). Country of type locality: Panama.

**Geographical distribution.**NEO.

**NEO**: Costa Rica, Ecuador, Panama.

**Notes.** The original description (Grinter et al. 2009: 14) did not detail where the holotype was deposited. As such, under ICZN Article 16.4.2, the name *Distratrixpandora* Grinter, 2009 must be considered unavailable. However, the species name was validated in a subsequent paper (Grinter and Whitfield 2019).

***Distatrixpapilionis* (Viereck, 1912)**

*Apantelespapilionis* Viereck, 1912.

*Apantelesagamemnonis* Wilkinson, 1928.

**Type information.** Holotype female, USNM (examined). Country of type locality: India.

**Geographical distribution.**OTL.

**OTL**: China (YN), India, Indonesia, Malaysia, Myanmar.

***Distatrixpitillaensis* Grinter, 2009**

*Distatrixpitillaensis* Grinter, 2009.

**Type information.** Holotype female, USNM (not examined but original description checked). Country of type locality: Costa Rica.

**Geographical distribution.**NEO.

**NEO**: Costa Rica.

***Distatrixpompelon* (Nixon, 1965)**

*Apantelespompelon* Nixon, 1965.

**Type information.** Holotype female, NHMUK (examined). Country of type locality: Japan.

**Geographical distribution.**PAL.

**PAL**: Japan, Korea, Slovakia, Switzerland.

**Notes.** This species is considered by most authors to belong to *Distatrix* (e.g., Papp 1988, 1990, Capek and Lukas 1989, van Achterberg and Rezbanyai-Reser 2001, Kotenko 2007a), a decision we agree with, based on our study of the holotype. However, Yu et al. (2016) list it in *Protapanteles*, following van Achterberg (2003). For the sake of clarity, we revise the species combination here.

***Distatrixsancus* (Nixon, 1965)**

*Apantelessancus* Nixon, 1965.

**Type information.** Holotype female, NHMUK (examined). Country of type locality: France.

**Geographical distribution.**PAL.

**PAL**: Azerbaijan, Bulgaria, France, Germany, Hungary, Russia (NC, S), Spain, Ukraine.

**Notes.** This species is considered by most authors to belong to *Distatrix* (e.g., Papp 1988, 2005, Schurian et al. 1993, Jiménez et al. 1996), a decision we agree with, based on our study of the holotype. However, Yu et al. (2016) list it in *Protapanteles*, following van Achterberg (2003). For the sake of clarity, we revise the species combination here.

***Distatrixsimulissima* (de Saeger, 1944), new combination**

*Apantelessimulissimus* de Saeger, 1944.

**Type information.** Holotype female, RMCA (not examined but original description checked). Country of type locality: Democratic Republic of Congo.

**Geographical distribution.**AFR.

**AFR**: Democratic Republic of Congo, Ivory Coast.

**Notes.** Based on the original description and that of Nixon (1965), the best generic placement would be in *Distatrix*. However, it could also be *Glyptapanteles* but examination of specimens would be needed to conclude. The species name must be treated as an adjective and not as a noun (Doug Yanega, pers. comm.) and thus it must match the gender of the genus name.

***Distatrixsolanae* Whitfield, 1996**

*Distatrixsolanae* Whitfield, 1996.

**Type information.** Holotype female, UCDC (not examined but original description checked). Country of type locality: USA.

**Geographical distribution.**NEA.

**NEA**: USA (CA, OR).

***Distatrixteapae* (Nixon, 1965)**

*Apantelesteapae* Nixon, 1965.

**Type information.** Holotype female, NHMUK (examined). Country of type locality: Mexico.

**Geographical distribution.**NEO.

**NEO**: Mexico.

***Distatrixtookei* (Shenefelt, 1972), new combination**

*Apantelestookei* Shenefelt, 1972.

*Apantelesflaviventris* Ullyett, 1946 [secondary homonym of *Apantelesflaviventris* Cresson, 1865].

**Type information.** Holotype female, SAMC (not examined but original description checked). Country of type locality: South Africa.

**Geographical distribution.**AFR.

**AFR**: South Africa.

**Notes.** The original description strongly suggest that this species belongs to *Distatrix*, based on the entirely smooth propodeum, shape and sculpture of T1–T2, and shape of the hypopygium. Ullyett (1946) also mentioned two previously described species of *Distatrix* as the closest to the new taxon, and placed the new species in a key by Wilkinson (1932a) with other species of the genus. Another plausible alternative could be *Glyptapanteles*; however, the distinguishing characters between these two genera (number of sulci on the pronotum laterally and setation pattern on the ovipositor sheaths) were not detailed by Ullyett (1946). Although only study of the type series can establish this unambiguously, we still consider *Distatrix* to be the best generic placement, based on T2 having lateral margins (diverging sulci) not extending to the apex of the tergite, a strong character of *Distatrix*.

***Distatrixtormina* (Nixon, 1965), new combination**

*Apantelestormina* Nixon, 1965.

**Type information.** Holotype female, NHMUK (examined). Country of type locality: Democratic Republic of Congo.

**Geographical distribution.**AFR.

**AFR**: Democratic Republic of Congo.

**Notes.** Transferred to *Distatrix* based on the pronotum laterally with a single furrow, T2 lateral sulcus only marked on the anterior half of the tergite, fore wing venation, and ovipositor sheaths with only very minute setae near the very apex.

***Distatrixugandaensis* (Gahan, 1918)**

*Apantelesugandaensis* Gahan, 1918.

**Type information.** Holotype female, USNM (not examined but original description checked). Country of type locality: Uganda.

**Geographical distribution.**AFR.

**AFR**: Democratic Republic of Congo, Ivory Coast, Kenya, Rwanda, Uganda.

**Notes.** The type series, from Uganda, was reared from a pyralid on *Hibiscus* (Wilkinson 1934a: 146); however, that same paper cites a few dozen specimens from three collecting events, reared from the tortricid host *Choristoneuraoccidentalis*. That is the only reference to a tortricid as host for the entire genus *Distatrix* and should be considered as a record needing further verification.

***Distatrixvigilis* Grinter, 2009**

*Distatrixvigilis* Grinter, 2009.

**Type information.** Holotype female, USNM (not examined but original description checked). Country of type locality: Costa Rica.

**Geographical distribution.**NEO.

**NEO**: Costa Rica.

***Distatrixxanadon* Grinter, 2009**

*Distatrixxanadon* Grinter, 2009.

**Type information.** Holotype female, USNM (not examined but original description checked). Country of type locality: Costa Rica.

**Geographical distribution.**NEO.

**NEO**: Costa Rica.

***Distatrixyemenitica* van Achterberg & Fernandez-Triana, 2017**

*Distatrixyemeniticus* van Achterberg & Fernandez-Triana, 2017.

**Type information.** Holotype female, RMNH (examined). Country of type locality: Yemen.

**Geographical distribution.**AFR.

**AFR**: Yemen.

**Notes.** The species name must be treated as an adjective and not as a noun (Doug Yanega, pers. comm.) and thus it must match the gender of the genus name.

***Distatrixyunae* Rousse & Gupta, 2013**

*Distatrixyunae* Rousse & Gupta, 2013.

**Type information.** Holotype female, MNHN (not examined but original description checked). Country of type locality: Réunion.

**Geographical distribution.**AFR.

**AFR**: Réunion.

#### Genus Dodogaster Rousse, 2013

***Dodogaster*** Rousse, 2013: 522. Gender: feminine. Type species: *Dodogastergrangeri* Rousse, 2013, by original designation.

Only known from a single species from the Afrotropical region, described from two females from Réunion (Rousse and Gupta 2013). Its relationship within Microgastrinae is unclear. No host data are currently available for this genus. There are no DNA barcodes of *Dodogaster* in BOLD.

***Dodogastergrangeri* Rousse, 2013**

*Dodogastergrangeri* Rousse, 2013.

**Type information.** Holotype female, MNHN (not examined but original description checked). Country of type locality: Réunion.

**Geographical distribution.**AFR.

**AFR**: Réunion.

**Notes.** In addition to the original description, we also examined the female paratype (in the CNC).

#### Genus Dolichogenidea Viereck, 1911

***Dolichogenidea*** Viereck, 1911: 173. Gender: feminine. Type species: Apanteles (Dolichogenidea) banksi Viereck, 1911, by monotypy.

Originally described as a subgenus of *Apanteles*, but elevated to generic rank by Mason (1981). This is a cosmopolitan genus, with 366 described species known from all biogeographical regions. Many European species were revised by Wilkinson, Nixon and Papp in several papers from the middle part of the 20^th^ century, and a few more recent papers treat the fauna of China (Chen and Song 2004), the Russian Far East (Kotenko 2007a), and a species group in Costa Rica (Fernandez-Triana et al. 2019). Overall, the taxonomic coverage of the world species is far from complete; we have seen at least the same number of undescribed species in collections, from both tropical and temperate areas, and it is likely that the genus will approach one thousand species. The concept of *Dolichogenidea* and its separation from *Apanteles* has been controversial (e.g., Mason 1981, van Achterberg 2003, Fernandez-Triana et al. 2014e), but we consider it as a valid genus. More than 30 families of Lepidoptera have been recorded as hosts for *Dolichogenidea*, but many records are likely to be incorrect and/or need further verification. In Costa Rica (ACG) most of the known hosts belong to four families: Depressariidae, Thyrididae, Tortricidae, and Mimallonidae (unpublished information extracted from BOLD and ACG databases). There are almost 3.500 DNA-barcode compliant sequences of *Dolichogenidea* in BOLD representing 372 different BINs, mostly from Costa Rica and North America.

***Dolichogenideaaberrantenna* Liu & Chen, 2018**

*Dolichogenideaaberrantenna* Liu & Chen, 2018.

**Type information.** Holotype female, ZJUH (not examined but original description checked). Country of type locality: China.

**Geographical distribution.**OTL.

**OTL**: China (SC).

***Dolichogenideaabsona* (Muesebeck, 1965)**

*Apantelesabsonus* Muesebeck, 1965.

**Type information.** Holotype female, USNM (not examined but original description checked). Country of type locality: USA.

**Geographical distribution.**NEA.

**NEA**: Canada (AB, BC, MB, NB, NL, NS, ON, PE, QC), USA (CO, MT, NM, OR, WA, WI).

**Notes.** Our species concept is based on Muesebeck (1965), Nixon (1972), Mason (1974) and Fernandez-Triana and Huber (2010).

***Dolichogenideaacratos* (Nixon, 1967)**

*Apantelesacratos* Nixon, 1967.

**Type information.** Holotype female, NHMUK (examined). Country of type locality: Papua New Guinea.

**Geographical distribution.**AUS, OTL.

**AUS**: Papua New Guinea; **OTL**: Vietnam.

***Dolichogenideaacrobasidis* (Muesebeck, 1921)**

*Apantelesacrobasidis* Muesebeck, 1921.

**Type information.** Holotype female, USNM (not examined but original description checked). Country of type locality: USA.

**Geographical distribution.**NEA.

**NEA**: USA (FL, MD, MS).

***Dolichogenideaacron* (Nixon, 1967)**

*Apantelesacron* Nixon, 1967.

**Type information.** Holotype female, NHMUK (examined). Country of type locality: Thailand.

**Geographical distribution.**OTL.

**OTL**: China (ZJ), Thailand.

**Notes.** After examining the holotype we had decided to transfer this species to *Dolichogenidea* based on the entirely setose hind wing vannal lobe, and the anteromesoscutum with punctures that do not fuse near the posterior margin. However, the species was transferred right before our paper by Liu et al. (2019), who also recorded the species from China based on one female specimen, an information we incorporate here.

***Dolichogenideaaegeriphagous* Liu & Chen, 2018**

*Dolichogenideaaegeriphagous* Liu & Chen, 2018.

**Type information.** Holotype female, ZJUH (not examined but original description checked). Country of type locality: China.

**Geographical distribution.**PAL.

**PAL**: China (HL, HA, JL, LN, SD, XJ).

***Dolichogenideaagamedes* (Nixon, 1965), new combination**

*Apantelesagamedes* Nixon, 1965.

**Type information.** Holotype female, NHMUK (examined). Country of type locality: Mauritius.

**Geographical distribution.**AFR, OTL.

**AFR**: Mauritius; **OTL**: Vietnam.

**Notes.** After examining the holotype, we believe the best generic placement for this species is in *Dolichogenidea*, based on the relatively coarse punctures on the anteromesoscutum (which do not fuse near the scutoscutellar sulcus), and the entirely setose vannal lobe in the hind wing. Yu et al. (2016) listed this species’ distribution as Oceanic and Oriental. However, Mauritius is better considered as part of the Afrotropical region, and thus here we record the species distribution as only AFR and OTL.

***Dolichogenideaagilis* (Ashmead, 1905)**

*Pseudapantelesagilis* Ashmead, 1905.

*Apanteleshidaridis* Rohwer, 1922.

**Type information.** Holotype female, USNM (examined). Country of type locality: Philippines.

**Geographical distribution.**OTL.

**OTL**: China (GD), India, Indonesia, Philippines, Vietnam.

**Notes.** We also examined the type of *Apanteleshidaridis* Rohwer, 1922, a female specimen. The species distribution in China is based in Liu et al. (2019).

***Dolichogenideaagilla* (Nixon, 1972)**

*Apantelesagilla* Nixon, 1972.

*Apantelespiraticus* Papp, 1977.

**Type information.** Holotype female, MZH (not examined but paratype examined). Country of type locality: Finland.

**Geographical distribution.**PAL.

**PAL**: Finland, Greece, Hungary, Iran, Mongolia, Russia (ZAB, PRI), United Kingdom.

**Notes.** Both the holotype and paratype are from Finland. Nixon (1972) points out that the difference between the two specimens may be significant. The English specimen recorded by Shaw (2012b) agrees closely with the paratype.

***Dolichogenideaagonoxenae* (Fullaway, 1941)**

*Apantelesagonoxenae* Fullaway, 1941.

*Apantelesorelia* Nixon,1967.

**Type information.** Holotype female, BPBM (not examined but authoritatively identified specimens examined). Country of type locality: Western Samoa.

**Geographical distribution.**AUS, OTL.

**AUS**: Fiji, Tonga, Western Samoa; **OTL**: Vietnam.

**Notes.** We studied the female type of *Apantelesorelia* Nixon, which was synonymized under *agonoxenae* by Walker (1989), a reference we also read.

***Dolichogenideaalaria* (Kotenko, 1986)**

*Apantelesalarius* Kotenko, 1986.

**Type information.** Holotype female, SIZK (not examined but original description checked). Country of type locality: Azerbaijan.

**Geographical distribution.**PAL.

**PAL**: Azerbaijan.

***Dolichogenideaalbipennis* (Nees, 1834)**

*Microgasteralbipennis* Nees, 1834.

**Type information.** Type lost (not examined but subsequent treatment of the species checked). Country of type locality: unknown.

**Geographical distribution.**PAL.

**PAL**: Afghanistan, Albania, Azerbaijan, Belarus, Denmark, France, Georgia, Germany, Hungary, Italy, Kazakhstan, Kyrgyzstan, Lithuania, Moldova, Mongolia, Netherlands, Poland, Romania, Russia (DA, KDA, MOS, PRI, ROS, SPE, TA, VOR, YAR), Sweden, Turkey, Turkmenistan, Ukraine, United Kingdom.

**Notes.** Our species concept is based on Papp (1978a, 1988), Tobias (1986) and Kotenko (2007a).

***Dolichogenideaalejandromasisi* Fernandez-Triana & Boudreault, 2019**

*Dolichogenideaalejandromasisi* Fernandez-Triana & Boudreault, 2019.

**Type information.** Holotype female, CNC (examined). Country of type locality: Costa Rica.

**Geographical distribution.**NEO.

**NEO**: Costa Rica.

***Dolichogenideaalophogaster* Liu & Chen, 2019**

*Dolichogenideaalophogaster* Liu & Chen, 2019.

**Type information.** Holotype female, ZJUH (not examined but original description checked). Country of type locality: China.

**Geographical distribution.**OTL.

**OTL**: China (FJ).

***Dolichogenideaaltithoracica* Liu & Chen, 2019**

*Dolichogenideaaltithoracica* Liu & Chen, 2019.

**Type information.** Holotype female, ZJUH (not examined but original description checked). Country of type locality: China.

**Geographical distribution.**OTL.

**OTL**: China (FJ).

***Dolichogenideaaluella* (Nixon, 1967), new combination**

*Apantelesaluella* Nixon, 1967.

**Type information.** Holotype female, NHMUK (examined). Country of type locality: Indonesia.

**Geographical distribution.**OTL.

**OTL**: Indonesia, Malaysia, Philippines.

**Notes.** It is clear, from the original description (also, from Austin 1987) that this species belongs to *Dolichogenidea* based on its uniformly setose hind wing vannal lobe and the anteromesoscutum punctures that do not fuse near the scutoscutellar sulcus. However, it was never transferred to that genus – Austin (1987) mentioned that it belonged to *Dolichogenideasensu*Mason (1981) but he preferred to retain it under an *Apantelessensu lato* at the time. Thus, here we formally transfer the species.

***Dolichogenideaalutacea* (Balevski, 1980)**

*Apantelesalutaceus* Balevski, 1980.

**Type information.** Holotype female, ZIN (not examined but subsequent treatment of the species checked). Country of type locality: Bulgaria.

**Geographical distribution.**PAL.

**PAL**: Bulgaria.

***Dolichogenideaamaris* (Nixon, 1967)**

*Apantelesamaris* Nixon, 1967.

**Type information.** Holotype female, NHMUK (examined). Country of type locality: Thailand.

**Geographical distribution.**OTL.

**OTL**: China (YN), Thailand.

***Dolichogenideaanarsiae* (Faure & Alabouvette, 1924)**

*Apantelesanarsiae* Faure & Alabouvette, 1924.

**Type information.** Syntypes female and male, depository unknown (not examined but subsequent treatment of the species checked). Country of type locality: France.

**Geographical distribution.**PAL.

**PAL**: Azerbaijan, Bulgaria, France, Georgia, Hungary, Italy, Moldova, Romania, Russia (KDA, PRI), Switzerland, Turkey.

**Notes.** Our species concept is based on Wilkinson (1945), Nixon (1976), Papp (1981) and Tobias (1986).

***Dolichogenideaancylotergita* Liu & Chen, 2018**

*Dolichogenideaancylotergita* Liu & Chen, 2018.

**Type information.** Holotype female, ZJUH (not examined but original description checked). Country of type locality: China.

**Geographical distribution.**OTL, PAL.

**OTL**: China (SC); **PAL**: China (NX).

***Dolichogenideaangelagonzalezae* Fernandez-Triana & Boudreault, 2019**

*Dolichogenideaangelagonzalezae* Fernandez-Triana & Boudreault, 2019.

**Type information.** Holotype female, CNC (examined). Country of type locality: Costa Rica.

**Geographical distribution.**NEO.

**NEO**: Costa Rica.

***Dolichogenideaangularis* Song, Chen & Yang, 2006**

*Dolichogenideaangularis* Song, Chen & Yang, 2006.

**Type information.** Holotype female, FAFU (not examined but original description checked). Country of type locality: China.

**Geographical distribution.**OTL.

**OTL**: China (FJ).

***Dolichogenideaannularis* (Haliday, 1834)**

*Microgasterannularis* Haliday, 1834.

**Type information.** Lectotype female, NMID (not examined but subsequent treatment of the species checked). Country of type locality: Ireland.

**Geographical distribution.**PAL.

**PAL**: Germany, Hungary, Ireland, Italy, Poland, Russia (AMU, BU, SPE), Sweden, Switzerland, United Kingdom.

**Notes.** Our species concept is based on Nixon (1972), Papp (1980a) and Tobias (1986).

***Dolichogenideaanterocava* Liu & Chen, 2019**

*Dolichogenideaanterocava* Liu & Chen, 2019.

**Type information.** Holotype female, ZJUH (not examined but original description checked). Country of type locality: China.

**Geographical distribution.**OTL.

**OTL**: China (FJ, GD, HI).

***Dolichogenideaanteruga* Liu & Chen, 2018**

*Dolichogenideaanteruga* Liu & Chen, 2018.

**Type information.** Holotype female, ZJUH (not examined but original description checked). Country of type locality: China.

**Geographical distribution.**OTL, PAL.

**OTL**: China (SC, ZJ); **PAL**: China (HA).

***Dolichogenideaapicurvus* Liu & Chen, 2019**

*Dolichogenideaapicurvus* Liu & Chen, 2019.

**Type information.** Holotype female, ZJUH (not examined but original description checked). Country of type locality: China.

**Geographical distribution.**OTL.

**OTL**: China (FJ).

***Dolichogenideaappellator* (Telenga, 1949), status revised**

*Apantelesappellator* Telenga, 1949.

*Apantelessalverdensis* Hedqvist, 1965.

*Apanteleslitae* Nixon, 1972.

Apanteleslitaevar.operculellae Nixon, 1972.

**Type information.** Holotype female, depository unknown (not examined but subsequent treatment of the species checked). Country of type locality: Tajikistan.

**Geographical distribution.**AFR, PAL.

**AFR**: Cape Verde, Ghana, Senegal; **PAL**: Afghanistan, Armenia, Azerbaijan, Belarus, Bulgaria, China (NM, XJ), Croatia, Cyprus, Egypt, Germany, Hungary, Iran, Israel, Italy, Jordan, Kazakhstan, Malta, Moldova, Mongolia, Romania, Russia (KDA, MOS, PRI, ROS, VGG, YAR), Selvagens Islands, Spain, Switzerland, Tajikistan, Tunisia, Turkey, Turkmenistan, Ukraine, United Kingdom, Uzbekistan, Yugoslavia.

**Notes.**Shaw (2012b) synonymized *Dolichogenidealitae* within *D.appellator*, and provided comments on the rationale for that. Additional comments were also provided in Broad et al. (2016). Papp (2015) brought *Dolichogenidealitae* back from synonymy and considered it to be a valid species, based on subtle morphological differences as well as ecological traits, including the distribution (of *litae*) mainly in marine environments. However, we have obtained DNA barcodes of specimens attributed to both species and they do not differ. We have also examined the holotype and many paratypes of *D.litae*, and a rather long series of specimens in the NHMUK identified as *D.appellator* and we cannot find enough differences to justify separation as two species. Thus, here we revise the species status and sink *D.litae* back into synonymy with *D.appellator*. An additional problem that remains to be solved is the status of what Nixon (1972) referred to as Apanteleslitaevar.operculellae; we have examined those specimens and they might represent a different species. However, resolving that is beyond the scope of the present paper.

***Dolichogenideaargiope* (Nixon, 1965), new combination**

*Apantelesargiope* Nixon, 1965.

**Type information.** Holotype female, NHMUK (examined). Country of type locality: South Africa.

**Geographical distribution.**AFR, AUS, OTL, PAL.

**AFR**: South Africa; **AUS**: Australia (QLD) Fiji, Vanuatu; **OTL**: India, Indonesia, Malaysia, Philippines, Singapore; **PAL**: Korea.

**Notes.** The holotype has a uniformly setose vannal lobe (clearly visible on the right hind wing, less clear on the left hind wing) and the anteromesoscutum has coarse punctures that do not fuse to each other near the scutoscutellar sulcus. Both characters indicate this species is better placed in *Dolichogenidea*.

***Dolichogenideaartissima* (Papp, 1971)**

*Apantelesartissimus* Papp, 1971.

*Apantelesabila* Nixon, 1972.

**Type information.** Holotype female, HNHM (examined). Country of type locality: Hungary.

**Geographical distribution.**PAL.

**PAL**: Finland, Germany, Hungary, Mongolia, Russia (PRI), Sweden, United Kingdom.

**Notes.** Our species concept is based on Shaw (2012b). In addition to the type of *artissima*, we have examined the female type of its synonym, *A.abila* (Nixon 1972), deposited in the NHMUK, which resembles *Pholetesor*, based on its inflexible hypopygium, relatively short ovipositor sheaths, and the shape and coarse sculpture of T2. Other species currently placed in *Dolichogenidea* (e.g., *bres*, *coniferoides*, and *mycale*, see notes under those species as well) also resemble *Pholetesor* because of their relatively short ovipositor and inflexible hypopygium. The available host records for those microgastrines often include species whose early instars are leaf miners; thus, there might be some convergence in species that oviposit into leaf miners on important functional things, e.g., short length of ovipositor and the associated relative robustness (lack of multiple creases) in the hypopygium. Until more evidence is available, especially based on molecular studies, we refrain to transfer those species in this paper and retain them in *Dolichogenidea*.

***Dolichogenideaartusicarina* Song & Chen, 2004**

*Dolichogenideaartusicarina* Song & Chen, 2004.

**Type information.** Holotype female, FAFU (not examined but original description checked). Country of type locality: China.

**Geographical distribution.**OTL.

**OTL**: China (FJ).

***Dolichogenideaashoka* Rousse, 2013**

*Dolichogenideaashoka* Rousse, 2013.

**Type information.** Holotype female, MNHN (not examined but original description checked). Country of type locality: Réunion.

**Geographical distribution.**AFR.

**AFR**: Réunion.

***Dolichogenideaatarsi* Liu & Chen, 2019**

*Dolichogenideaatarsi* Liu & Chen, 2019.

**Type information.** Holotype female, ZJUH (not examined but original description checked). Country of type locality: China.

**Geographical distribution.**OTL.

**OTL**: China (GZ).

***Dolichogenideaate* (Nixon, 1973)**

*Apantelesate* Nixon, 1973.

**Type information.** Holotype female, NHMUK (examined). Country of type locality: Sweden.

**Geographical distribution.**PAL.

**PAL**: Hungary, Poland, Sweden, United Kingdom.

***Dolichogenideaatreus* (Nixon, 1973), new combination**

*Apantelesatreus* Nixon, 1973.

**Type information.** Holotype female, NHMUK (examined). Country of type locality: United Kingdom.

**Geographical distribution.**PAL.

**PAL**: Bulgaria, Czech Republic, Denmark, Germany, Greece, Hungary, Italy, Russia (NC), Turkey, United Kingdom.

**Notes.** Transferred to *Dolichogenidea* based on the entirely setose hind wing vannal lobe, and the anteromesoscutum with punctures not fusing near the scutoscutellar sulcus. The holotype has a mostly inflexible hypopygium and relatively short ovipositor sheaths (ca. half the length of the metatibia), which might indicate that *Pholetesor* would be a better generic placement. However, we have decided to transfer this species to *Dolichogenidea* as it belongs to a group of species which are all currently placed in that genus. Furthermore, all known hosts of this wasp belong to Momphidae, a Lepidoptera family which has only been recorded as a host for a few other *Dolichogenidea* species (e.g., see Yu et al. 2016), and one species in the unrelated genus *Microgaster* (Shaw 2012b).

***Dolichogenideaazovica* (Kotenko, 1986)**

*Apantelesazovicus* Kotenko, 1986.

**Type information.** Holotype female, SIZK (not examined but original description checked). Country of type locality: Ukraine.

**Geographical distribution.**PAL.

**PAL**: Poland, Russia (PRI), Ukraine.

***Dolichogenideabakeri* (Wilkinson, 1932), new combination**

*Apantelesbakeri* Wilkinson, 1932.

**Type information.** Holotype female, USNM (examined). Country of type locality: Philippines.

**Geographical distribution.**OTL.

**OTL**: China (TW), Philippines.

**Notes.** After examining the holotype, it is clear this species belongs to *Dolichogenidea* based on its slighly convex and uniformly setose vannal lobe.

***Dolichogenideabambusae* (Wilkinson, 1928)**

*Apantelesbambusae* Wilkinson, 1928.

**Type information.** Holotype female, NHMUK (examined). Country of type locality: India.

**Geographical distribution.**OTL.

**OTL**: China (HI), India, Vietnam.

**Notes.** The holotype has one hind wing left, but its vannal lobe is entirely setose, which indicates this species belongs in *Dolichogenidea*. The species was transferred to this genus right before our paper by Liu et al. (2019), who also recorded the species from China based several female specimens, an information we incorporate here.

***Dolichogenideabanksi* (Viereck, 1911)**

*Apantelesbanksi* Viereck, 1911.

**Type information.** Holotype female, USNM (examined). Country of type locality: USA.

**Geographical distribution.**NEA.

**NEA**: USA (DC, MD, MI, NY, PA, VA).

***Dolichogenideabaoris* (Wilkinson, 1930)**

*Apantelesbaoris* Wilkinson, 1930.

*Apantelesparnarae* Watanabe, 1935.

**Type information.** Holotype female, NHMUK (examined). Country of type locality: Malaysia.

**Geographical distribution.**OTL, PAL.

**OTL**: Bangladesh, China (FJ, GD, GX, GZ, HI, HK, HN, JX, SC, SH, SN, TW, YN, ZJ), India, Malaysia, Pakistan, Philippines, Ryukyu Islands, Sri Lanka, Thailand, Vietnam; **PAL**: China (AH, HA, HB, JL, JS, LN, SN, SD), Japan, Nepal.

**Notes.** The species distribution in China is based in Liu et al. (2019).

***Dolichogenideabasiflava* (Papp, 1974), new combination**

*Apantelesbasiflavus* Papp, 1974.

**Type information.** Holotype female, HNHM (not examined but original description checked). Country of type locality: Korea.

**Geographical distribution.**PAL.

**PAL**: Korea.

**Notes.** Both the original description and a subsequence treatment of the species (Papp 1978a) considered *Apantelesbasiflavus* within a group of species now placed in *Dolichogenidea*; the original description also provides enough detail to transfer this species to that genus.

***Dolichogenideabenevolens* (Papp, 1973)**

*Apantelesbenevolens* Papp, 1973.

**Type information.** Holotype female, HNHM (not examined but original description checked). Country of type locality: Italy.

**Geographical distribution.**PAL.

**PAL**: Italy.

***Dolichogenideabenkevitshi* (Kotenko, 1986)**

*Apantelesbenkevitshi* Kotenko, 1986.

**Type information.** Holotype female, SIZK (not examined but original description checked). Country of type locality: Ukraine.

**Geographical distribution.**PAL.

**PAL**: Russia (NC), Ukraine.

**Notes.** The original description (Kotenko 1986: 785) referred to the hind wing vannal lobe as straight and without a clear fringe of setae, which suggests this species might not belong in *Dolichogenidea*. However, all other characters mentioned in the original description agree with that genus, as does the position of the species in the key provided by Kotenko (where *D.benkevitshi* is keyed out in a section with several couplets that include many other *Dolichogenidea* species). It could well be that the setae are present but are small and difficult to see, as it is the case with other borderline species of *Dolichogenidea* such as *D.murinanae* and *D.petrovae* (see below for more comments on those species). Without seeing the type it is impossible to conclude, thus we prefer to maintain the species in *Dolichogenidea* for the time being, which is also where Papp (1988) placed it when he re-assigned all European *Apantelessensu lato* to the generic framework proposed by Mason (1981).

***Dolichogenideabersa* (Papp, 1976), new combination**

*Apantelesbersus* Papp, 1976.

**Type information.** Holotype female, HNHM (not examined but original description checked). Country of type locality: Mongolia.

**Geographical distribution.**PAL.

**PAL**: Mongolia, Russia (ZAB).

**Notes.** From the original description and Papp (1978a) it is clear this species belongs to *Dolichogenidea*. The spelling of the species name follows Belokobylskij et al. (2019).

***Dolichogenideabetheli* (Viereck, 1911)**

*Apantelesbetheli* Viereck, 1911.

**Type information.** Holotype female, USNM (examined). Country of type locality: USA.

**Geographical distribution.**NEA.

**NEA**: USA (CO).

**Notes.**Muesebeck (1921) mentioned that the type series had three specimens, all deposited in the USNM; however, we could only find the female holotype, and no other specimen was found in the regular collection. Also, in Muesebeck's key (1921) this species is described as having a strongly compressed metasoma, but the holotype does not have such a strongly compressed metasoma. The holotype has the ovipositor sinuous, not at the tip as in *Promicrogaster* species, but sinuous throughout its entire length.

***Dolichogenideabicolor* Song & Chen, 2004**

*Dolichogenideabicolor* Song & Chen, 2004.

**Type information.** Holotype female, FAFU (not examined but original description checked). Country of type locality: China.

**Geographical distribution.**PAL.

**PAL**: China (JL).

***Dolichogenideabiconcava* Liu & Chen, 2018**

*Dolichogenideabiconcava* Liu & Chen, 2018.

**Type information.** Holotype female, ZJUH (not examined but original description checked). Country of type locality: China.

**Geographical distribution.**OTL.

**OTL**: China (GD).

***Dolichogenideabilecikensis* Inanç & Cetin Erdogan, 2004**

*Dolichogenideabilecikensis* Inanç & Cetin Erdogan, 2004.

**Type information.** Holotype female, ZMTU (not examined but original description checked). Country of type locality: Turkey.

**Geographical distribution.**PAL.

**PAL**: Turkey.

***Dolichogenideabimacula* Song & Chen, 2004**

*Dolichogenideabimacula* Song & Chen, 2004.

**Type information.** Holotype female, FAFU (not examined but original description checked). Country of type locality: China.

**Geographical distribution.**OTL.

**OTL**: China (FJ).

***Dolichogenideabiplagae* (Fischer, 1968), new combination**

*Apantelesbiplagae* Fischer, 1968.

**Type information.** Holotype female, MHNG (not examined but original description checked). Country of type locality: Ivory Coast.

**Geographical distribution.**AFR.

**AFR**: Ivory Coast.

**Notes.** Transferred to *Dolichogenidea* based on the original description mentioning the hind wing with vannal lobe convex and setose.

***Dolichogenideabiroi* (Szépligeti, 1905)**

*Apantelesbiroi* Szépligeti, 1905.

**Type information.** Lectotype female, HNHM (not examined but authoritatively identified specimens examined). Country of type locality: Australia.

**Geographical distribution.**AUS.

**AUS**: Australia (NSW).

**Notes.** We studied a female and a male paralectotype deposited in the NHMUK, incorrectly labelled with the primary type code 3c.1055.

***Dolichogenideabisulcata* (Cameron, 1909), new combination**

*Apantelesbisulcatus* Cameron, 1909.

**Type information.** Holotype female, NHMUK (examined). Country of type locality: Sri Lanka.

**Geographical distribution.**OTL.

**OTL**: China (TW), India, Sri Lanka.

**Notes.** The hind wing vannal lobe of this species is fully setose. Although the lobe in the right wing is partially and slightly folded behind in the holotype, giving the impression of not having setae, careful examination at higher magnification and study of the left hind wing corroborates the setosity of the vannal lobe. Also, the anteromesoscutum punctures do not fuse.

***Dolichogenideabonbonensis* Fagan-Jeffries & Austin, 2019**

*Dolichogenideabonbonensis* Fagan-Jeffries & Austin, 2019.

**Type information.** Holotype female, SAMA (not examined but original description checked). Country of type locality: Australia.

**Geographical distribution.**AUS.

**AUS**: Australia (SA).

***Dolichogenideaborysthenica* (Kotenko, 1986)**

*Apantelesborysthenicus* Kotenko, 1986.

**Type information.** Holotype female, SIZK (not examined but original description checked). Country of type locality: Ukraine.

**Geographical distribution.**PAL.

**PAL**: Russia (S), Ukraine.

***Dolichogenideabrabyi* Fagan-Jeffries & Austin, 2019**

*Dolichogenideabrabyi* Fagan-Jeffries & Austin, 2019.

**Type information.** Holotype female, ANIC (not examined but original description checked). Country of type locality: Australia.

**Geographical distribution.**AUS.

**AUS**: Australia (ACT).

***Dolichogenideabres* (Nixon, 1973)**

*Apantelesbres* Nixon, 1973.

**Type information.** Holotype female, NHMUK (examined). Country of type locality: United Kingdom.

**Geographical distribution.**PAL.

**PAL**: United Kingdom.

**Notes.** The setae on the hind wing vannal lobe are very small and not easily visible, but otherwise this species mostly agrees with the *Dolichogenidea* concept we follow in this paper. Although the hypopygium is evenly sclerotized, which would suggest *Pholetesor* as a better generic placement than *Dolichogenidea*, we refrain to transfer those species in this paper until more evidence is available, especially based on molecular studies (see also additional comments under *D.artisima* above).

***Dolichogenideabreviattenuata* Liu & Chen, 2019**

*Dolichogenideabreviattenuata* Liu & Chen, 2019.

**Type information.** Holotype female, ZJUH (not examined but original description checked). Country of type locality: China.

**Geographical distribution.**OTL.

**OTL**: China (GD, YN).

***Dolichogenideabrevicarinata* Chen & Song, 2004**

*Dolichogenideabrevicarinata* Chen & Song, 2004.

**Type information.** Holotype female, FAFU (not examined but original description checked). Country of type locality: China.

**Geographical distribution.**OTL, PAL.

**OTL**: China (FJ, GZ, ZJ); **PAL**: China (HE, JL, LN, SD).

**Notes.** The species distribution in China is based in Liu et al. (2019).

***Dolichogenideabrevifacialis* Liu & Chen, 2018**

*Dolichogenideabrevifacialis* Liu & Chen, 2018.

**Type information.** Holotype female, ZJUH (not examined but original description checked). Country of type locality: China.

**Geographical distribution.**OTL.

**OTL**: China (GD, ZJ).

***Dolichogenideabreviventris* (Ratzeburg, 1848)**

*Microgasterbreviventris* Ratzeburg, 1848.

*Apantelesmesoxanthus* Ruschka, 1917.

*Apantelesnilae* Telenga, 1961.

**Type information.** Neotype female, EBW (not examined but original description checked). Country of type locality: Germany.

**Geographical distribution.**NEA, OTL, PAL.

**NEA**: Canada (NL); **OTL**: China (ZJ); **PAL**: Czech Republic, Egypt, Finland, Germany, Hungary, Ireland, Italy, Korea, Moldova, Netherlands, Poland, Romania, Russia (VOR), Serbia, Slovakia, Sweden, Switzerland, Turkey, United Kingdom.

***Dolichogenideabritannica* (Wilkinson, 1941)**

*Apantelesbritannicus* Wilkinson, 1941.

*Apantelesmasmithi* Fernandez-Triana, 2010.

**Type information.** Holotype female, NHMUK (examined). Country of type locality: United Kingdom.

**Geographical distribution.**NEA, PAL.

**NEA**: Canada (MB, NS, ON); **PAL**: Armenia, Greece, Hungary, Iran, Israel, Malta, Russia (PRI), Slovakia, Tajikistan, Ukraine, United Kingdom.

**Notes.** In the holotype the hind wing vannal lobe is more or less straight (at most very slightly convex) and the fringe of setae is not very visible (as setae are minute). The species distribution in Israel is based on Belokobylskij et al. (2019).

***Dolichogenideabroadi* Rousse, 2013**

*Dolichogenideabroadi* Rousse, 2013.

**Type information.** Holotype female, MNHN (not examined but original description checked). Country of type locality: Réunion.

**Geographical distribution.**AFR.

**AFR**: Réunion.

***Dolichogenideabushnelli* (Muesebeck, 1933)**

*Apantelesbushnelli* Muesebeck, 1933.

**Type information.** Holotype female, USNM (not examined but original description checked). Country of type locality: USA.

**Geographical distribution.**NEA.

**NEA**: USA (CA, FL, IA, NE).

***Dolichogenideacacoeciae* (Riley, 1881)**

*Apantelescacoeciae* Riley, 1881.

*Pseudapantelesgallaediploppi* Ashmead, 1899 [*Nomen nudum*].

**Type information.** Syntypes female and male, USNM (examined). Country of type locality: USA.

**Geographical distribution.**NEA.

**NEA**: Canada (ON, QC), USA (CO, IL, KY, MD, MI, MO, NJ, NY, PA, SC, SD).

**Notes.** The type series is on a single pin, which has a piece of card cut into eight points. Three of the eight syntypes are missing, and another syntype is missing the head and metasoma.

***Dolichogenideacalifornica* (Muesebeck, 1921)**

*Apantelescalifornicus* Muesebeck, 1921.

**Type information.** Holotype female, USNM (not examined but original description checked). Country of type locality: USA.

**Geographical distribution.**NEA.

**NEA**: Canada (AB, BC, ON, QC), USA (CA, ID, OR).

***Dolichogenideacameroonensis* Walker, 1994**

*Dolichogenideacameroonensis* Walker, 1994.

**Type information.** Holotype female, MNHN (not examined but original description checked). Country of type locality: Cameroon.

**Geographical distribution.**AFR.

**AFR**: Cameroon.

***Dolichogenideacandidata* (Haliday, 1834)**

*Microgastercandidatus* Haliday, 1834.

*Microgasterterebrator* Ratzeburg, 1852.

**Type information.** Lectotype female, NMID (not examined but subsequent treatment of the species checked). Country of type locality: Ireland.

**Geographical distribution.**AFR, PAL.

**AFR**: Cape Verde; **PAL**: Azerbaijan, Bulgaria, Germany, Greece, Hungary, Ireland, Macedonia, Mongolia, Romania, Russia (KHA, KDA, PRI, SAK), Serbia, Sweden, Tajikistan, Turkmenistan, United Kingdom, Uzbekistan.

**Notes.** Our species concept is based on Papp (2005, 2009a, 2009b), Kotenko (2007) and Shaw (2012b). Here we consider *Dolichogenidealongicauda* (Wesmael, 1837) as a separate, different species, following Fernandez-Triana and Huber (2010) and Fernandez-Triana et al. (2014b), although that decision has not been followed by most authors (see further notes under our treatment of *longicauda* below).

***Dolichogenideacaniae* (Wilkinson, 1928)**

*Apantelescaniae* Wilkinson, 1928.

**Type information.** Holotype female, NHMUK (examined). Country of type locality: Indonesia.

**Geographical distribution.**OTL.

**OTL**: China (FJ, GD, GX, GZ, YN, ZJ), India, Indonesia, Sri Lanka, Thailand.

**Notes.** The species distribution in China is based in Liu et al. (2019).

***Dolichogenideacarborugosa* Liu & Chen, 2019**

*Dolichogenideacarborugosa* Liu & Chen, 2019.

**Type information.** Holotype female, ZJUH (not examined but original description checked). Country of type locality: China.

**Geographical distribution.**OTL, PAL.

**OTL**: China (ZJ); **PAL**: China (HB).

***Dolichogenideacarlosmanuelrodriguezi* Fernandez-Triana & Boudreault, 2019**

*Dolichogenideacarlosmanuelrodriguezi* Fernandez-Triana & Boudreault, 2019.

**Type information.** Holotype female, CNC (examined). Country of type locality: Costa Rica.

**Geographical distribution.**NEO.

**NEO**: Costa Rica.

***Dolichogenideacarposinae* (Wilkinson, 1938)**

*Apantelescarposinae* Wilkinson, 1938.

**Type information.** Holotype female, NHMUK (examined). Country of type locality: New Zealand.

**Geographical distribution.**AUS.

**AUS**: New Zealand.

***Dolichogenideacatonix* (Shenefelt, 1972), new combination**

*Apantelescatonix* Shenefelt, 1972.

*Apantelescato* Nixon, 1967 [primary junior homonym of *Apantelescato* de Saeger, 1944].

**Type information.** Holotype female, NHMUK (examined). Country of type locality: Malaysia.

**Geographical distribution.**OTL.

**OTL**: Malaysia.

**Notes.** We transfer the species to *Dolichogenidea* based on the anteromesoscutum having punctures that do not fuse near the scutoscutellar sulcus, and the entirely setose hind wing vannal lobe.

***Dolichogenideacauda* Song & Chen, 2004**

*Dolichogenideacauda* Song & Chen, 2004.

**Type information.** Holotype female, FAFU (not examined but original description checked). Country of type locality: China.

**Geographical distribution.**PAL.

**PAL**: China (JL).

***Dolichogenideacelsa* (Papp, 1975)**

*Apantelescelsus* Papp, 1975.

**Type information.** Holotype female, HNHM (not examined but original description checked). Country of type locality: Hungary.

**Geographical distribution.**PAL.

**PAL**: Bosnia and Herzegovina, Hungary, Montenegro, Tunisia.

***Dolichogenideacerialis* (Nixon, 1976)**

*Apantelescerialis* Nixon, 1976.

*Apantelesareolaris* Balevski & Tobias, 1980 [primary junior homonym of *Apantelesareolaris* Blanchard, 1947].

**Type information.** Holotype female, ZSM (not examined but original description checked). Country of type locality: Italy.

**Geographical distribution.**PAL.

**PAL**: Bulgaria, Hungary, Israel, Italy, Kazakhstan, Russia (S), Spain.

**Notes.** The species distribution in Kazakhstan and Russia is based on Belokobylskij et al. (2019).

***Dolichogenideachangbaiensis* Liu & Chen, 2018**

*Dolichogenideachangbaiensis* Liu & Chen, 2018.

**Type information.** Holotype female, ZJUH (not examined but original description checked). Country of type locality: China.

**Geographical distribution.**PAL.

**PAL**: China (JL).

***Dolichogenideacheles* (Nixon, 1972)**

*Apantelescheles* Nixon, 1972.

**Type information.** Holotype female, NHMUK (not examined but original description checked). Country of type locality: Sweden.

**Geographical distribution.**PAL.

**PAL**: Finland, Hungary, Poland, Russia (NW), Sweden, Turkey.

**Notes.** We could not find the holotype in the NHMUK collection (its assigned number is 3c.1760). In the place where it is supposed to be, there is a note from Annette K. Walker, dated August 1998, stating that the type was not present in the collection at that time either.

***Dolichogenideachrysis* (Nixon, 1973), new combination**

*Apanteleschrysis* Nixon, 1973.

**Type information.** Holotype female, NHMUK (examined). Country of type locality: United Kingdom.

**Geographical distribution.**PAL.

**PAL**: United Kingdom.

**Notes.** Transferred to *Dolichogenidea* based on the vannal lobe entirely setose, and the anteromesoscutum with punctures not fusing near the scutoscutellar sulcus.

***Dolichogenideacinerosa* (Papp, 1971)**

*Apantelescinerosus* Papp, 1971.

**Type information.** Holotype female, HNHM (not examined but subsequent treatment of the species checked). Country of type locality: Mongolia.

**Geographical distribution.**PAL.

**PAL**: Belgium, Hungary, Mongolia, Russia (PRI), Serbia.

**Notes.** Our species concept is based on Papp (1978a) and Tobias (1986).

***Dolichogenideacinnarae* Gupta, Lokhande & Soman, 2013**

*Dolichogenideacinnarae* Gupta, Lokhande & Soman, 2013.

**Type information.** Holotype female, NBAIR (not examined but original description checked). Country of type locality: India.

**Geographical distribution.**OTL.

**OTL**: India.

***Dolichogenideaclaniae* (You & Zhou, 1990)**

*Apantelesclaniae* You & Zhou, 1990.

**Type information.** Holotype female, HUNAU (not examined but subsequent treatment of the species checked). Country of type locality: China.

**Geographical distribution.**OTL, PAL.

**OTL**: China (FJ, JX); **PAL**: China (JL).

**Notes.** Our species concept is based on Chen and Song (2004).

***Dolichogenideaclausa* Liu & Chen, 2019**

*Dolichogenideaclausa* Liu & Chen, 2019.

**Type information.** Holotype female, ZJUH (not examined but original description checked). Country of type locality: China.

**Geographical distribution.**OTL.

**OTL**: China (HI).

***Dolichogenideaclavata* (Provancher, 1881)**

*Microgasterclavatus* Provancher, 1881.

**Type information.** Lectotype female, ULQC (not examined but subsequent treatment of the species checked). Country of type locality: Canada.

**Geographical distribution.**NEA.

**NEA**: Canada (ON, QC), USA (AR, CA, CT, GA, KY, MO, NJ, OH, OR, SC, TN, WI).

**Notes.** Our species concept is based on Nixon (1972) and Papp (1978a).

***Dolichogenideacoequata* (Nixon, 1967)**

*Apantelescoequatus* Nixon, 1967.

**Type information.** Holotype female, NHMUK (examined). Country of type locality: Tonga.

**Geographical distribution.**AUS, OTL.

**AUS**: Tonga; **OTL**: Vietnam.

***Dolichogenideacoffea* (Wilkinson, 1930), new combination**

*Apantelescoffea* Wilkinson, 1930.

**Type information.** Holotype female, NHMUK (examined). Country of type locality: Uganda.

**Geographical distribution.**AFR.

**AFR**: Democratic Republic of Congo, Kenya, Uganda.

**Note.** This species has the hind wing with an entirely setose vannal lobe, indicating it belongs to *Dolichogenidea* and not *Apanteles*. The ovipositor is apically sinuate, something very rarely present in Microgastrinae (very few species outside of the unrelated genus *Promicrogaster*). Other notable features of this species are T1 and T2 almost entirely smooth and shiny, and yellow metasoma and legs (except for metacoxa).

***Dolichogenideacolchica* (Tobias, 1976)**

*Apantelescolchicus* Tobias, 1976.

**Type information.** Holotype female, ZIN (not examined but subsequent treatment of the species checked). Country of type locality: Georgia.

**Geographical distribution.**PAL.

**PAL**: Georgia.

**Notes.** Our species concept is based on Papp (1978a) and Tobias (1986).

***Dolichogenideacoleophorae* (Wilkinson, 1938)**

*Apantelescoleophorae* Wilkinson, 1938.

**Type information.** Holotype female, NHMUK (examined). Country of type locality: United Kingdom.

**Geographical distribution.**NEA, PAL.

**NEA**: Canada (NL); **PAL**: Azerbaijan, Finland, Hungary, Poland, Romania, Russia (KHA, VOR, YAR), Slovakia, Switzerland, Tajikistan, Tunisia, Turkey, United Kingdom, Uzbekistan.

***Dolichogenideaconcentrica* Liu & Chen, 2018**

*Dolichogenideaconcentricus* Liu & Chen, 2018.

**Type information.** Holotype female, ZJUH (not examined but original description checked). Country of type locality: China.

**Geographical distribution.**OTL.

**OTL**: China (ZJ).

**Notes.** The species name must be treated as an adjective and not as a noun (Doug Yanega, pers. comm.) and thus it must match the gender of the genus name.

***Dolichogenideaconiferae* (Haliday, 1834)**

*Microgasterconiferae* Haliday, 1834.

**Type information.** Lectotype female, NMID (not examined but subsequent treatment of the species checked). Country of type locality: Ireland.

**Geographical distribution.**AFR, PAL.

**AFR**: Cape Verde; **PAL**: Azerbaijan, Germany, Hungary, Mongolia, Romania, Russia (KDA), Sweden, Tajikistan, Turkmenistan, United Kingdom, Uzbekistan.

**Notes.** Our species concept is based on Shaw (2012b) and Broad et al. (2016).

***Dolichogenideaconiferoides* (Papp, 1972)**

*Apantelesconiferoides* Papp, 1972.

*Apantelestrogos* Nixon, 1973.

**Type information.** Holotype female, HNHM (not examined but subsequent treatment of the species checked). Country of type locality: Hungary.

**Geographical distribution.** PAL

**PAL**: Hungary, Sweden, Turkey.

**Notes.** We examined the type of *Apantelestrogos* Nixon. Our species concept is based on Shaw (2012b), but see also additional comments under *D.artisima* above.

***Dolichogenideaconpuncta* Liu & Chen, 2019**

*Dolichogenideaconpuncta* Liu & Chen, 2019.

**Type information.** Holotype female, ZJUH (not examined but original description checked). Country of type locality: China.

**Geographical distribution.**OTL.

**OTL**: China (GD, HI).

***Dolichogenideacontergita* Song & Chen, 2004**

*Dolichogenideacontergita* Song & Chen, 2004.

**Type information.** Holotype female, FAFU (not examined but original description checked). Country of type locality: China.

**Geographical distribution.**PAL.

**PAL**: China (JL).

***Dolichogenideacordiae* Ahmad, 2019**

*Dolichogenideacontergita* Ahmad & Pandey, 2019.

**Type information.** Holotype female, AMUZ (not examined but original description checked). Country of type locality: India.

**Geographical distribution.**OTL.

**OTL**: India.

**Notes.** The authorship of the species names was not made clear in the original description paper, thus here we follow Ahmad (pers. comm.) for that information.

***Dolichogenideacoretas* (Nixon, 1965), new combination**

*Apantelescoretas* Nixon, 1965.

**Type information.** Holotype female, NHMUK (examined). Country of type locality: Vanuatu.

**Geographical distribution.**AUS.

**AUS**: Vanuatu.

**Notes.** We transfer the species to *Dolichogenidea* based on the anteromesoscutum having punctures that do not fuse near the scutoscutellar sulcus, and the hind wing vannal lobe entirely setose.

***Dolichogenideacorvina* (Reinhard, 1880)**

*Apantelescorvinus* Reinhard, 1880.

*Apanteleslucidus* Szépligeti, 1896.

*Apantelesrasteratus* Fahringer, 1936.

*Apantelesaptus* Papp, 1977.

**Type information.** Lectotype female, ZMHB (not examined but original description checked). Country of type locality: Germany.

**Geographical distribution.**NEA, PAL.

**NEA**: Canada (NL); **PAL**: Azerbaijan, Bulgaria, China (NX), Czech Republic, Finland, Georgia, Germany, Greece, Hungary, Iran, Ireland, Japan, Kazakhstan, Lithuania, Moldova, Mongolia, Netherlands, Poland, Romania, Russia (KDA, ROS), Sweden, Tajikistan, Turkmenistan, Ukraine, United Kingdom, Uzbekistan.

**Notes.** This species has been almost always been treated as *Apantelescorvinus* in the literature, even by authors recognizing *Dolichogenidea* as a valid genus (e.g., Broad et al. 2016). The only reference we have found that consider this species to belong to *Dolichogenidea* is Evenhuis and Vlug (1983). We have examined numerous specimens of this species and found that the vannal lobe indeed corresponds to *Dolichogenidea*.

***Dolichogenideacrassa* Liu & Chen, 2019**

*Dolichogenideacrassa* Liu & Chen, 2019.

**Type information.** Holotype female, ZJUH (not examined but original description checked). Country of type locality: China.

**Geographical distribution.**PAL.

**PAL**: China (SD).

***Dolichogenideacredne* (Nixon, 1973)**

*Apantelescredne* Nixon, 1973.

**Type information.** Holotype female, NHMUK (examined). Country of type locality: United Kingdom.

**Geographical distribution.**PAL.

**PAL**: Germany, Poland, Russia (NW), Slovakia, United Kingdom.

***Dolichogenideacucurbita* Liu & Chen, 2019**

*Dolichogenideacucurbita* Liu & Chen, 2019.

**Type information.** Holotype female, ZJUH (not examined but original description checked). Country of type locality: China.

**Geographical distribution.**OTL.

**OTL**: China (FJ).

***Dolichogenideacultriformis* Song & Chen, 2004**

*Dolichogenideacultriformis* Song & Chen, 2004.

**Type information.** Holotype female, FAFU (not examined but original description checked). Country of type locality: China.

**Geographical distribution.**OTL, PAL.

**OTL**: China (FJ); **PAL**: China (JL).

***Dolichogenideacyamon* (Nixon, 1967)**

*Apantelescyamon* Nixon, 1967.

**Type information.** Holotype female, NHMUK (examined). Country of type locality: Vanuatu.

**Geographical distribution.**AUS.

**AUS**: Vanuatu.

***Dolichogenideacyane* (Nixon, 1965), new combination**

*Apantelescyane* Nixon, 1965.

**Type information.** Holotype female, NHMUK (examined). Country of type locality: Fiji.

**Geographical distribution.**AUS.

**AUS**: Fiji.

**Notes.** We transfer this species to *Dolichogenidea* based on the hind wing with the vannal lobe being convex and entirely setose, and the anteromesoscutum mostly smooth, with punctures that do not fuse near scutoscutellar sulcus.

***Dolichogenideacytherea* (Nixon, 1972)**

*Apantelescytherea* Nixon, 1972.

**Type information.** Holotype female, NHMUK (examined). Country of type locality: United Kingdom.

**Geographical distribution.**PAL.

**PAL**: Greece, Hungary, Mongolia, Poland, Russia (S), Serbia, Slovakia, Switzerland, Ukraine, United Kingdom.

***Dolichogenideadecora* (Haliday, 1834)**

*Microgasterdecorus* Haliday, 1834.

*Apanteleslineatus* Reinhard, 1880.

*Apantelessibiricus* Fahringer, 1938.

**Type information.** Lectotype female, NMID (not examined but subsequent treatment of the species checked). Country of type locality: Ireland.

**Geographical distribution.**OTL, PAL.

**OTL**: China (JS); **PAL**: Bulgaria, Czech Republic, Estonia, Finland, Georgia, Germany, Greece, Hungary, Iran, Ireland, Kazakhstan, Lithuania, Poland, Romania, Russia (DA, IRK, KHA, KDA, NGR), Slovakia, Spain, Sweden, Turkmenistan.

**Notes.** Our species concept is based on Papp (1979a) and Tobias (1986).

***Dolichogenideadiaphantus* (Nixon, 1965), new combination**

*Apantelesdiaphantus* Nixon, 1965.

**Type information.** Holotype female, NHMUK (examined). Country of type locality: India.

**Geographical distribution.**OTL.

**OTL**: India.

**Notes.** We transfer the species to *Dolichogenidea* based on the anteromesoscutum having punctures that do not fuse near the scutoscutellar sulcus, and the hind wing vannal lobe setose (although the setae are relatively smaller and sparser than in typical members of the genus, the vannal lobe is still uniformly setose).

***Dolichogenideadilecta* (Haliday, 1834)**

*Microgasterdilectus* Haliday, 1834.

*Microgasterfemoralis* Bouché, 1834.

**Type information.** Lectotype female, NMID (not examined but subsequent treatment of the species checked). Country of type locality: Ireland.

**Geographical distribution.**OTL, PAL.

**OTL**: China (FJ); **PAL**: Armenia, Austria, Bulgaria, Czech Republic, Finland, France, Germany, Hungary, Ireland, Italy, Japan, Korea, Moldova, Netherlands, Poland, Romania, Russia (PRI, SAK), Slovakia, Sweden, Switzerland, Turkey, Ukraine, United Kingdom.

**Notes.** Our species concept is based on Wilkinson (1945), Nixon (1972), Papp (1978a), Tobias (1986), and Chen and Song (2004).

***Dolichogenideadioryctriphagous* Liu & Chen, 2018**

*Dolichogenideadioryctriphagous* Liu & Chen, 2018.

**Type information.** Holotype female, ZJUH (not examined but original description checked). Country of type locality: China.

**Geographical distribution.**OTL, PAL.

**OTL**: China (HN, ZJ); **PAL**: China (AH, HA, JL).

***Dolichogenideadiparopsidis* (Lyle, 1927), new combination**

*Apantelesdiparopsidis* Lyle, 1927.

**Type information.** Holotype female, NHMUK (examined). Country of type locality: South Africa.

**Geographical distribution.**AFR.

**AFR**: Democratic Republic of Congo, Ivory Coast, Malawi, Nigeria, South Africa, Tanzania, Uganda, Zimbabwe.

**Notes.** Transferred to *Dolichogenidea* based on the hind wing with the vannal lobe entirely setose, and the anteromesoscutum with punctures not fusing near the scutoscutellar sulcus.

***Dolichogenideadiscreta* (Szépligeti, 1914)**

*Apantelesdiscretus* Szépligeti, 1914.

**Type information.** Holotype female, MNHN (not examined but subsequent treatment of the species checked). Country of type locality: Kenya.

**Geographical distribution.**AFR.

**AFR**: Kenya, Madagascar.

**Notes.** Our species concept is based on Wilkinson (1932a), Granger (1949), and Madl & van Achterberg (2014).

***Dolichogenideadolichocephalus* (Muesebeck, 1921)**

*Apantelesdolichocephalus* Muesebeck, 1921.

**Type information.** Holotype female, USNM (not examined but original description checked). Country of type locality: USA.

**Geographical distribution.**NEA.

**NEA**: USA (IL, MI, VA).

**Notes.** Following Article 31.2.1 of the ICZN the name is a noun phrase in apposition and the original spelling *dolichocephalus* must be retained.

***Dolichogenideadrusilla* (Nixon, 1972)**

*Apantelesdrusilla* Nixon, 1972.

**Type information.** Holotype female, NHMUK (examined). Country of type locality: United Kingdom.

**Geographical distribution.**PAL.

**PAL**: Bulgaria, Germany, Hungary, Italy, Mongolia, Russia (PRI), Slovakia, Sweden, Turkey, Ukraine, United Kingdom.

***Dolichogenideadryas* (Nixon, 1965), new combination**

*Apantelesdryas* Nixon, 1965.

**Type information.** Holotype female, USNM (examined). Country of type locality: China.

**Geographical distribution.**PAL.

**PAL**: China (BJ).

**Notes.** Transferred to *Dolichogenidea* based on the hind wing with the vannal lobe being more or less straight but setose, and the anteromesoscutum with coarse punctures that do not fuse.

***Dolichogenideaearterus* (Wilkinson, 1930), new combination**

*Apantelesearterus* Wilkinson, 1930.

**Type information.** Holotype female, NHMUK (examined). Country of type locality: Sudan.

**Geographical distribution.**AFR.

**AFR**: Democratic Republic of Congo, Senegal, Sudan.

**Notes.** The holotype has the hind wing vannal lobe entirely setose, indicating the species belongs to *Dolichogenidea*.

***Dolichogenideaeleagnellae* (Tobias, 1976)**

*Apanteleseleagnellae* Tobias, 1976.

**Type information.** Holotype female, ZIN (not examined but subsequent treatment of the species checked). Country of type locality: Armenia.

**Geographical distribution.**PAL.

**PAL**: Armenia, Russia (NC).

**Notes.** Our species concept is based on Papp (1979a) and Tobias (1986). The holotype is presumed to be in the ZIN based on Tobias (1986).

***Dolichogenideaemarginata* (Nees, 1834)**

*Microgasteremarginatus* Nees, 1834.

*Microgasterscapularis* Bouché, 1834.

**Type information.** Type lost (not examined but subsequent treatment of the species checked). Country of type locality: Germany.

**Geographical distribution.**PAL.

**PAL**: Armenia, Austria, Azerbaijan, Belgium, Bulgaria, France, Germany, Hungary, Iran, Ireland, Israel, Italy, Kazakhstan, Lithuania, Moldova, Poland, Romania, Russia (ALT, KHA, PRI, ROS, SPE, YAR), Slovakia, Sweden, Switzerland, Turkey, United Kingdom.

**Notes.** Our species concept is based on Nixon (1972), Papp (1978a), and Tobias (1986).

***Dolichogenideaensiformis* (Ratzeburg, 1844)**

*Microgasterensiformis* Ratzeburg, 1844.

**Type information.** Neotype female, SMF (not examined but subsequent treatment of the species checked). Country of type locality: Germany.

**Geographical distribution.**PAL.

**PAL**: Germany, Hungary, Italy, Latvia, Mongolia, Poland, Romania, Russia (NW), Slovakia, Spain, Tunisia.

**Notes.** Our species concept is based on Papp (1979a) and Tobias (1986).

***Dolichogenideaensiger* (Say, 1836), new combination**

*Microgasterensiger* Say, 1836.

*Microgasterfemurnigrum* Provancher, 1886.

*Apantelestrachynotus* Viereck, 1912.

*Apantelesnipmuckorum* Viereck, 1917.

**Type information.** Neotype female, USNM (not examined but authoritatively identified specimens examined). Country of type locality: USA.

**Geographical distribution.**NEA.

**NEA**: Canada (AB, MB, NB, NL, NS, NT, ON, PE, QC, SK), USA (AK, AL, CO, CT, DE, DC, FL, GA, IL, IN, IA, KS, LA, MA, MD, MO, MT, NH, NJ, NY, NC, TN).

**Notes.** We could not find the neotype of *ensiger* in the USNM collection; however, we have studied many authoritatively named specimens of this species available in the CNC and USNM collections: a) One male paratype of *ensiger* (with a red USNM Paratype label no. 14707), which has a hind wing vannal lobe typical of *Dolichogenidea*; b) The types of two other species currently considered as synonyms of *ensiger*, both in the USNM: *A.trachynotus* Viereck, 1912, a male specimen; and *A.nipmuckorum* Viereck, 1917, a female specimen; c) Non-type material from another synonym, *Microgasterfemurnigrum* Provancher, 1886 (in the USNM); d) Many specimens of *ensiger* (in the CNC and USNM), determined by Viereck, Muesebeck or Mason. All of those specimens clearly belong to *Dolichogenidea*. Apart from the morphological evidence (hind wing vannal lobe setose) of the specimens we examined, DNA barcoding also supports the same generic placement. More than 500 sequences of *Apantelesensiger* are currently available in BOLD, they comprise two BINS: BOLD:ACE6783 (Canada: ON, MB) and BOLD:AAA3764 (Canada: AB, ON, SK and some localities of southern USA), and both BINs cluster near many other species of *Dolichogenidea* and far apart from *Apanteles* sequences. Whether those BINs represent two different species or not has been mentioned in the past (Fernandez-Triana et al. 2014b) but no further study has been conducted so far, thus all known specimens are kept as one species for the time being.

***Dolichogenideaerasmi* (Nixon, 1972)**

*Apanteleserasmi* Nixon, 1972.

**Type information.** Holotype female, ZSM (not examined but original description checked). Country of type locality: Germany.

**Geographical distribution.**PAL.

**PAL**: Germany, Italy, Slovakia.

***Dolichogenideaerdoesi* (Papp, 1973)**

*Apanteleserdoesi* Papp, 1973.

*Apantelesnegativus* Tobias, 1976.

**Type information.** Holotype female, HNHM (not examined but original description checked). Country of type locality: Hungary.

**Geographical distribution.**PAL.

**PAL**: Azerbaijan, Hungary, Russia (NC), Ukraine.

**Notes.***Dolichogenideaerdoesi* is related to several species of *Dolichogenidea*, and it was transferred to that genus by Papp (1988). However, Kotenko (2007a) placed it within *Iconella*. After carefully reading the original description and subsequent treatment of the species, it is clear to us that it does not belong to *Iconella*, as it does not have a median, longitudinal carina on the propodeum. Thus, here we are revising the species combination back to *Dolichogenidea*.

***Dolichogenideaerevanica* (Tobias, 1976)**

*Apanteleserevanicus* Tobias, 1976.

**Type information.** Holotype female, ZIN (not examined but subsequent treatment of the species checked). Country of type locality: Armenia.

**Geographical distribution.**PAL.

**PAL**: Armenia, Bulgaria, Serbia.

**Notes.** Our species concept is based on Papp (1981) and Tobias (1986). The holotype is presumed to be in the ZIN based on Tobias (1986).

***Dolichogenideaeros* (Wilkinson, 1932), new combination**

*Apanteleseros* Wilkinson, 1932.

**Type information.** Holotype female, NHMUK (examined). Country of type locality: South Africa.

**Geographical distribution.**AFR.

**AFR**: South Africa.

**Notes.** The holotype has the hind wing vannal lobe entirely setose, indicating the species belongs to *Dolichogenidea*.

***Dolichogenideaeucalypti* Austin & Allen, 1989**

*Dolichogenideaeucalypti* Austin & Allen, 1989.

**Type information.** Holotype female, ANIC (not examined but original description checked). Country of type locality: Australia.

**Geographical distribution.**AUS.

**AUS**: Australia (SA).

***Dolichogenideaevadne* (Nixon, 1955), new combination**

*Apantelesevadne* Nixon, 1955.

**Type information.** Holotype female, NHMUK (examined). Country of type locality: Juan Fernández Islands.

**Geographical distribution.**NEO.

**NEO**: Juan Fernández Islands.

**Notes.** The holotype has the hind wing vannal lobe entirely setose, indicating the species belongs to *Dolichogenidea*. Yu et al. (2016) stated that the holotype is deposited in Chile, but we examined a female specimen with code 3c.1465 in the NHMUK, which has a label with Nixon’s handwriting stating that is the female holotype. We have based our assessment of this species based on that specimen.

***Dolichogenideaevonymellae* (Bouché, 1834)**

*Microgasterevonymellae* Bouché, 1834.

*Apantelesiarbas* Nixon, 1972.

**Type information.** Holotype female, ZMHB (not examined but authoritatively identified specimens examined). Country of type locality: Germany.

**Geographical distribution.**PAL.

**PAL**: Armenia, Azerbaijan, Belarus, Bulgaria, Czech Republic, Germany, Hungary, Italy, Lebanon, Netherlands, Portugal, Romania, Russia (C, NC, NW, S), Serbia, United Kingdom.

**Notes.** We studied the type of *Apantelesiarbas* Nixon (in the NHMUK). The species’ presence in UK and Russia is based on Žiki et al. (2013) and Belokobylskij et al. (2019) respectively.

***Dolichogenideaexcellentis* Liu & Chen, 2019**

*Dolichogenideaexcellentis* Liu & Chen, 2019.

**Type information.** Holotype female, ZJUH (not examined but original description checked). Country of type locality: China.

**Geographical distribution.**OTL, PAL.

**OTL**: China (GD, GZ, HI, SC, YN); **PAL**: China (HA, HE).

***Dolichogenideaexilis* (Haliday, 1834)**

*Microgasterexilis* Haliday, 1834.

**Type information.** Lectotype female, NMID (examined). Country of type locality: Ireland.

**Geographical distribution.**PAL.

**PAL**: Bulgaria, Germany, Hungary, Ireland, Sweden, United Kingdom.

**Notes.** This species was transferred from *Apanteles* to *Dolichogenidea* by Shaw (2012b).

***Dolichogenideafakhrulhajiae* (Mahdihassan, 1925)**

*Apantelesfakhrulhajiae* Mahdihassan, 1925.

*Apantelesrufulus* Wilkinson, 1930.

*Apantelesfakhrulhajiae* nagoliensis Mahdihassan, 1955.

**Type information.** Type and depository unknown (not examined but authoritatively identified specimens examined). Country of type locality: India.

**Geographical distribution.**OTL.

**OTL**: India, Vietnam.

**Note.** We have examined the type of *A.rufulus* Wilkinson, deposited in the NHMUK.

***Dolichogenideafalcator* (Ratzeburg, 1852), new combination**

*Microgasterfalcator* Ratzeburg, 1852.

**Type information.** Type lost (not examined but subsequent treatment of the species checked). Country of type locality: unknown.

**Geographical distribution.**PAL.

**PAL**: Germany, Italy, Netherlands, Poland.

**Notes.** Our species concept is based on Telenga (1955) and van Achterberg (1976). The species is transferred to *Dolichogenidea* based on the entirely setose hind wing vannal lobe (van Achterberg 1976: fig. 54).

***Dolichogenideafaucula* (Nixon, 1972)**

*Apantelesfaucula* Nixon, 1972.

**Type information.** Holotype female, NHMUK (examined). Country of type locality: United Kingdom.

**Geographical distribution.**PAL.

**PAL**: Hungary, Finland, Poland, Russia (C, PR), United Kingdom.

***Dolichogenideafernandeztrianai* Abdoli & Talebi, 2019**

*Dolichogenideafernandeztrianai* Abdoli & Talebi, 2019.

**Type information.** Holotype female, TMUC (not examined but original description checked). Country of type locality: Iran.

**Geographical distribution.**PAL.

**PAL**: Iran.

***Dolichogenideaficicola* Donaldson, 1991**

*Dolichogenideaficicola* Donaldson, 1991.

**Type information.** Holotype female, TMSA (not examined but original description checked). Country of type locality: South Africa.

**Geographical distribution.**AFR.

**AFR**: South Africa.

***Dolichogenideafinchi* Fagan-Jeffries & Austin, 2018**

*Dolichogenideafinchi* Fagan-Jeffries & Austin, 2018.

**Type information.** Holotype female, WAM (not examined but original description checked). Country of type locality: Australia.

**Geographical distribution.**AUS.

**AUS**: Australia (NSW, QLD, VIC, WA).

***Dolichogenideaflavigastrula* Chen & Song, 2004**

*Dolichogenideaflavigastrula* Chen & Song, 2004.

**Type information.** Holotype female, FAFU (not examined but original description checked). Country of type locality: China.

**Geographical distribution.**OTL.

**OTL**: China (FJ).

***Dolichogenideaflavostriata* (Papp, 1977)**

*Apantelesflavostriatus* Papp, 1977.

**Type information.** Holotype female, HNHM (not examined but subsequent treatment of the species checked). Country of type locality: Hungary.

**Geographical distribution.**PAL.

**PAL**: Greece, Hungary.

**Notes.** Our species concept is based on Papp (1977b, 1980), and Tobias (1986).

***Dolichogenideaflexisulcus* Liu & Chen, 2019**

*Dolichogenideaflexisulcus* Liu & Chen, 2019.

**Type information.** Holotype female, ZJUH (not examined but original description checked). Country of type locality: China.

**Geographical distribution.**OTL, PAL.

**OTL**: China (GD, ZJ); **PAL**: China (JS).

***Dolichogenideaflexitergita* Liu & Chen, 2019**

*Dolichogenideaflexitergita* Liu & Chen, 2019.

**Type information.** Holotype female, ZJUH (not examined but original description checked). Country of type locality: China.

**Geographical distribution.**OTL.

**OTL**: China (YN).

***Dolichogenideafluctisulcus* Liu & Chen, 2019**

*Dolichogenideafluctisulcus* Liu & Chen, 2019.

**Type information.** Holotype female, ZJUH (not examined but original description checked). Country of type locality: China.

**Geographical distribution.**OTL.

**OTL**: China (YN).

***Dolichogenideaforrestae* Fagan-Jeffries & Austin, 2019**

*Dolichogenideaforrestae* Fagan-Jeffries & Austin, 2019.

**Type information.** Holotype female, SAMA (not examined but original description checked). Country of type locality: Australia.

**Geographical distribution.**AUS.

**AUS**: Australia (SA).

***Dolichogenideafrustrata* (Papp, 1975)**

*Apantelesfrustratus* Papp, 1975.

**Type information.** Holotype female, HNHM (not examined but original description checked). Country of type locality: Mongolia.

**Geographical distribution.**PAL.

**PAL**: Mongolia.

***Dolichogenideafumea* Liu & Chen, 2018**

*Dolichogenideafumeus* Liu & Chen, 2018.

**Type information.** Holotype female, ZJUH (not examined but original description checked). Country of type locality: China.

**Geographical distribution.**OTL, PAL.

**OTL**: China (FJ, GZ, HB, SC, ZJ); **PAL**: China (AH, HE, HA, SD).

**Notes.** The species name must be treated as an adjective and not as a noun (Doug Yanega, pers. comm.) and thus it must match the gender of the genus name.

***Dolichogenideafunalicauda* Liu & Chen, 2018**

*Dolichogenideafunalicauda* Liu & Chen, 2018.

**Type information.** Holotype female, ZJUH (not examined but original description checked). Country of type locality: China.

**Geographical distribution.**OTL.

**OTL**: China (FJ, GD, GZ, YN).

***Dolichogenideafurtim* (Papp, 1977)**

*Apantelesfurtim* Papp, 1977.

**Type information.** Holotype female, HNHM (not examined but original description checked). Country of type locality: Hungary.

**Geographical distribution.**PAL.

**PAL**: Azerbaijan, Greece, Hungary, Russia (S).

***Dolichogenideafuscivora* Walker, 1994**

*Dolichogenideafuscivora* Walker, 1994.

**Type information.** Holotype female, NHMUK (examined). Country of type locality: Ethiopia.

**Geographical distribution.**AFR.

**AFR**: Cameroon, Ethiopia, Kenya.

***Dolichogenideagagates* (Nees, 1834)**

*Microgastergagates* Nees, 1834.

**Type information.** Neotype female, RBINS (not examined but subsequent treatment of the species checked). Country of type locality: Belgium.

**Geographical distribution.**PAL.

**PAL**: Austria, Belgium, Bulgaria, Estonia, Finland, France, Georgia, Germany, Hungary, Italy, Latvia, Lithuania, Poland, Romania, Russia (MOS, YAR), Spain, Sweden, Switzerland, United Kingdom.

**Notes.** Our species concept is based on Nixon (1972), Papp (1979a), and Shaw (2012b).

***Dolichogenideagallicola* (Giraud, 1869)**

*Microgastergallicolus* Giraud, 1869.

**Type information.** Holotype female, MNHN (not examined but subsequent treatment of the species checked). Country of type locality: France.

**Geographical distribution.**PAL.

**PAL**: Algeria, Tunisia.

**Notes.** Our species concept is based on Papp (1978a).

***Dolichogenideagansuensis* Liu & Chen, 2018**

*Dolichogenideagansuensis* Liu & Chen, 2018.

**Type information.** Holotype female, ZJUH (not examined but original description checked). Country of type locality: China.

**Geographical distribution.**PAL.

**PAL**: China (GS).

***Dolichogenideagarytaylori* Fagan-Jeffries & Austin, 2019**

*Dolichogenideagarytaylori* Fagan-Jeffries & Austin, 2019.

**Type information.** Holotype female, SAMA (not examined but original description checked). Country of type locality: Australia.

**Geographical distribution.**AUS.

**AUS**: Australia (SA).

***Dolichogenideagelechiidivoris* (Marsh, 1975), new combination**

*Apantelesgelechiidivoris* Marsh, 1975.

**Type information.** Holotype female, USNM (examined). Country of type locality: Colombia.

**Geographical distribution.**NEO.

**OTL**: Chile, Colombia, Peru.

**Notes.** Examination of the holotype and paratypes show that they have the hind wing vannal lobe entirely setose, clearly indicating they are best placed within *Dolichogenidea*.

***Dolichogenideagentilis* (Nixon, 1967)**

*Apantelesgentilis* Nixon, 1967.

**Type information.** Holotype female, NHMUK (examined). Country of type locality: Papua New Guinea.

**Geographical distribution.**AUS, OTL.

**AUS**: Papua New Guinea, Solomon Islands; **OTL**: Vietnam.

***Dolichogenideagenuarnunezi* Fernandez-Triana & Boudreault, 2019**

*Dolichogenideagenuarnunezi* Fernandez-Triana & Boudreault, 2019.

**Type information.** Holotype female, CNC (examined). Country of type locality: Costa Rica.

**Geographical distribution.**NEO.

**NEO**: Costa Rica.

***Dolichogenideaglabra* (Papp, 1978)**

*Apantelesglaber* Papp, 1978.

**Type information.** Holotype female, HNHM (not examined but subsequent treatment of the species checked). Country of type locality: Finland.

**Geographical distribution.**PAL.

**PAL**: Finland, United Kingdom.

**Notes.** Our species concept is based on Papp (1981), Tobias (1986) and Shaw (2012b).

***Dolichogenideagleditsia* Liu & Chen, 2019**

*Dolichogenideagleditsia* Liu & Chen, 2019.

**Type information.** Holotype female, ZJUH (not examined but original description checked). Country of type locality: China.

**Geographical distribution.**OTL, PAL.

**OTL**: China (HN, ZJ); **PAL**: China (LN).

***Dolichogenideagobica* (Papp, 1976), new combination**

*Apantelesgobicus* Papp, 1976.

**Type information.** Holotype female, HNHM (not examined but original description checked). Country of type locality: Mongolia.

**Geographical distribution.**PAL.

**PAL**: Mongolia.

**Notes.** Although neither the original description nor a subsequent treatment of the species (Papp 1976b, 1984a) described the vannal lobe in the hind wing, both papers placed *gobicus* as very similar morphologically and very close to *Apantelespelopea* Nixon (which has been transferred in this paper to *Dolichogenidea* after we examined its holotype, see below under that species). Furthermore, other species considered by Papp to be relatively close morphologically to *gobicus* (based on how they were keyed out in both papers from Papp) have all been transferred to *Dolichogenidea* as well. The rest of the *gobicus* description fits well with it being *Dolichogenidea* (although no character is as conclusive as describing the setation pattern on the hind wing vannal lobe). Based on all available information and secondary evidence, we here formally transfer the species to *Dolichogenidea*.

***Dolichogenideagobustanica* (Kotenko, 1986)**

*Apantelesgobustanicus* Kotenko, 1986.

**Type information.** Holotype female, SIZK (not examined but original description checked). Country of type locality: Azerbaijan.

**Geographical distribution.**PAL.

**PAL**: Azerbaijan.

***Dolichogenideagolovushkini* (Kotenko, 1992)**

*Apantelesgolovushkini* Kotenko, 1992.

**Type information.** Holotype female, SIZK (not examined but subsequent treatment of the species checked). Country of type locality: Russia.

**Geographical distribution.**PAL.

**PAL**: Russia (ZAB).

**Notes.** Our species concept is based on Kotenko (2006).

***Dolichogenideagracilariae* (Wilkinson, 1940)**

*Apantelesgracilariae* Wilkinson, 1940.

**Type information.** Holotype female, NHMUK (examined). Country of type locality: United Kingdom.

**Geographical distribution.**PAL.

**PAL**: Armenia, Austria, Azerbaijan, Bulgaria, Czech Republic, Germany, Hungary, Iran, Kazakhstan, Moldova, Poland, Romania, Russia (KDA), Serbia, Slovakia, Spain, Sweden, Switzerland, Turkey, United Kingdom, Uzbekistan.

**Notes.** The species distribution in Iran and Uzbekistan is based on Belokobylskij et al. (2019).

***Dolichogenideagracilituba* Song & Chen, 2004**

*Dolichogenideagracilituba* Song & Chen, 2004.

**Type information.** Holotype female, FAFU (not examined but original description checked). Country of type locality: China.

**Geographical distribution.**OTL.

**OTL**: China (FJ).

***Dolichogenideagrata* (Kotenko, 1986)**

*Apantelesgratus* Kotenko, 1986.

**Type information.** Holotype female, SIZK (not examined but original description checked). Country of type locality: Ukraine.

**Geographical distribution.**PAL.

**PAL**: Russia (NC), Ukraine.

**Notes.** The species name must be treated as an adjective and not as a noun (Doug Yanega, pers. comm.) and thus it must match the gender of the genus name.

***Dolichogenideahalidayi* (Marshall, 1872)**

*Apanteleshalidayi* Marshall, 1872.

*Apanteleshalidaii* Marshall, 1872 [incorrect original spelling].

*Microgasteralbipennis* Haliday, 1834 [primary junior homonym of *Microgasteralbipennis* Nees, 1834].

**Type information.** Neotype female, MZLU (not examined but subsequent treatment of the species checked). Country of type locality: Sweden.

**Geographical distribution.**PAL.

**PAL**: Armenia, Croatia, Finland, Germany, Greece, Hungary, Iran, Ireland, Macedonia, Madeira Islands, Romania, Russia (MOS, NGR), Sweden, Ukraine, United Kingdom, Yugoslavia.

**Notes.**Wilkinson (1941b: 73) considered the original type series to be lost, and thus designated a neotype from Ringsjön, Sweden, which is deposited in the MZLU, and he provided ample explanation on the rationale to do so. However, van Achterberg (1997: 13) designated a lectotype from the Haliday material, presumably from Ireland, which is deposited in the NMID.

***Dolichogenideahamakii* (Watanabe, 1932)**

*Apanteleshamakii* Watanabe, 1932.

**Type information.** Holotype female, EIHU (examined). Country of type locality: Japan.

**Geographical distribution.**PAL.

**PAL**: Japan.

**Notes.** A transfer of this species to the genus *Dolichogenidea* was proposed by Liu et al. (2018), but it was only stated in the abstract of that paper. We have examined the female holotype and concur with them as the hind wing vannal lobe is fully setose.

***Dolichogenideahanoii* (Tobias & Long, 1990)**

*Apanteleshanoii* Tobias & Long, 1990.

**Type information.** Holotype female, ZIN (not examined but original description checked). Country of type locality: Vietnam.

**Geographical distribution.**OTL.

**OTL**: Vietnam.

**Notes.** Our species concept is based on Tobias and Long (1990) and Long and Belokobylskij (2004).

***Dolichogenideahasorae* Wilkinson, 1928**

*Apanteleshasorae* Wilkinson, 1928.

**Type information.** Holotype female, NHMUK (examined). Country of type locality: Indonesia.

**Geographical distribution.**OTL.

**OTL**: India, Indonesia.

***Dolichogenideahedyleptae* (Muesebeck, 1958)**

*Apanteleshedyleptae* Muesebeck, 1958.

**Type information.** Holotype female, USNM (examined). Country of type locality: Puerto Rico.

**Geographical distribution.**NEO.

**NEO**: Puerto Rico, Trinidad & Tobago.

***Dolichogenideahelleni* (Nixon, 1972)**

*Apanteleshelleni* Nixon, 1972.

**Type information.** Holotype female, MZH (not examined but original description checked). Country of type locality: Russia.

**Geographical distribution.**PAL.

**PAL**: Bulgaria, Finland, Germany, Hungary, Russia (KR), Ukraine.

***Dolichogenideahemerobiellicida* (Fischer, 1966)**

*Apanteleshemerobiellicida* Fischer, 1966.

**Type information.** Holotype female, NHMW (not examined but subsequent treatment of the species checked). Country of type locality: Austria.

**Geographical distribution.**PAL.

**PAL**: Austria.

**Notes.** Our species concept is based on Papp (1980a) and Tobias (1986).

***Dolichogenideahemituba* Liu & Chen, 2019**

*Dolichogenideahemituba* Liu & Chen, 2019.

**Type information.** Holotype female, ZJUH (not examined but original description checked). Country of type locality: China.

**Geographical distribution.**OTL.

**OTL**: China (FJ, GD, ZJ).

***Dolichogenideaheterusiae* (Wilkinson, 1928)**

*Apantelesheterusiae* Wilkinson, 1928.

**Type information.** Holotype female, NHMUK (examined). Country of type locality: Sri Lanka.

**Geographical distribution.**AUS, OTL, PAL.

**AUS**: Fiji; **OTL**: China (FJ, GX, HN, TW, ZJ), India, Sri Lanka, Vietnam; **PAL**: China (AH, BJ, CQ, GS, HB, JL, JS, SD, SN).

***Dolichogenideahexagona* Liu & Chen, 2019**

*Dolichogenideahexagona* Liu & Chen, 2019.

**Type information.** Holotype female, ZJUH (not examined but original description checked). Country of type locality: China.

**Geographical distribution.**OTL, PAL.

**OTL**: China (YN, ZJ); **PAL**: China (SD).

***Dolichogenideahilaris* (Haliday, 1834)**

*Microgasterhilaris* Haliday, 1834.

**Type information.** Lectotype female, NMID (not examined but subsequent treatment of the species checked). Country of type locality: Ireland.

**Geographical distribution.**PAL.

**PAL**: Ireland.

**Notes.** Transferred to *Dolichogenidea* by Broad et al. (2016), although the revised combination was not clearly stated.

***Dolichogenideahomoeosomae* (Muesebeck, 1933)**

*Apanteleshomoeosomae* Muesebeck, 1933.

**Type information.** Holotype female, USNM (not examined but original description checked). Country of type locality: Cuba.

**Geographical distribution.**NEA, NEO.

**NEA**: Canada (SK), USA (CA, MS, MO, SD, TX, WA); **NEO**: Cuba.

***Dolichogenideahyalinis* (Hedqvist, 1965), new combination**

*Apanteleshyalinis* Hedqvist, 1965.

**Type information.** Holotype male, MZH (examined). Country of type locality: Cape Verde.

**Geographical distribution.**AFR.

**AFR**: Cape Verde.

**Notes.**Forshage et al. (2016) considered that the type was missing and not found in Helsinki (MZH), as they wrote for a number of other Hedqvist types of Microgastrinae. Although Forshage et al. (2016) were unable to find those types, they are all present in the MZH collection, but were placed in a separate section of the collection and only recently found by the senior author of the present paper in 2017. The male holotype of *hyalinis* is missing one fore and one hind wing, one antenna and flagellomeres 15–16 of the other, but it is otherwise in reasonably good condition. The hind wing vannal lobe is clearly that of *Dolichogenidea*, slightly concave and entirely setose and thus the species is transferred to this genus here.

***Dolichogenideahyblaeae* (Wilkinson, 1928)**

*Apanteleshyblaeae* Wilkinson, 1928.

**Type information.** Holotype female, NHMUK (examined). Country of type locality: Western Samoa.

**Geographical distribution.**AUS, OTL.

**AUS**: Fiji, Western Samoa; **OTL**: China (GD), India, Indonesia, Vietnam.

**Notes.** The holotype is missing the metasoma.

***Dolichogenideailione* (Nixon, 1967)**

*Apantelesilione* Nixon, 1967.

**Type information.** Holotype female, NHMUK (examined). Country of type locality: Fiji.

**Geographical distribution.**AUS.

**AUS**: Fiji.

***Dolichogenideaimmissa* (Papp, 1977)**

*Apantelesimmissus* Papp, 1977.

**Type information.** Holotype female, HNHM (not examined but original description checked). Country of type locality: Hungary.

**Geographical distribution.**PAL.

**PAL**: Germany, Hungary, Slovakia, Turkey.

***Dolichogenideaimperator* (Wilkinson, 1939)**

*Apantelesimperator* Wilkinson, 1939.

**Type information.** Holotype female, NHMUK (examined). Country of type locality: United Kingdom.

**Geographical distribution.**PAL.

**PAL**: Armenia, Austria, Azerbaijan, Czech Republic, Germany, Hungary, Italy, Kazakhstan, Lithuania, Moldova, Netherlands, Romania, Russia (C, E, NC, NW, S), Switzerland, Turkmenistan, Tajikistan, United Kingdom, Uzbekistan.

**Notes.** The species distribution in Turkmenistan, Tajikistan and Uzbekistan is based on Belokobylskij et al. (2019).

***Dolichogenideaimpura* (Nees, 1834)**

*Microgasterimpurus* Nees, 1834.

**Type information.** Type lost (not examined but subsequent treatment of the species checked). Country of type locality: Germany.

**Geographical distribution.**PAL.

**PAL**: Austria, Azerbaijan, Belgium, Bulgaria, Czech Republic, France, Germany, Greece, Hungary, Italy, Latvia, Lithuania, Mongolia, Poland, Russia (KGD, YAR), Sweden, Switzerland.

**Notes.** Our species concept is based on Papp (1978a) and Tobias (1986). Broad et al. (2016) stated that this species is not present in the United Kingdom or Ireland. Additionally, they considered the name as uncertainly interpreted, although they did not elaborate more on that, and thus the species is here retained as valid for the time being. The species distribution in Azerbaijan is based on Belokobylskij et al. (2019).

***Dolichogenideaincompleta* (Szépligeti, 1914)**

*Apantelesincompletus* Szépligeti, 1914.

**Type information.** Holotype female, MNHN (not examined but subsequent treatment of the species checked). Country of type locality: Kenya.

**Geographical distribution.**AFR.

**AFR**: Kenya, Tanzania.

**Notes.** Our species concept is based on Papp (2008).

***Dolichogenideaincystatae* Fernandez-Triana, 2019, new replacement name**

*Dolichogenidealobesia* Liu & Chen, 2019 [junior primary homonym of *Dolichogenidealobesia* Fagan-Jeffries & Austin, 2019].

**Type information.** Holotype female, ZJUH (not examined but original description checked). Country of type locality: China.

**Geographical distribution.**OTL.

**OTL**: China (YN).

**Notes.***Dolichogenidealobesia* Liu & Chen, 2019 is a junior primary homonym of *Dolichogenidealobesia* Fagan-Jeffries & Austin, 2019. Both names represent different wasp species, named after two different host caterpillars in the genus *Lobesia* (Tortricidae). The replacement name was selected after the specific epithet of the host, *Lobesiaincystata* Liu & Yang, 1987, as mentioned in the original description of the Chinese wasp (Liu et al. 2019).

***Dolichogenideaindicaphagous* Liu & Chen, 2018**

*Dolichogenideaindicaphagous* Liu & Chen, 2018.

**Type information.** Holotype female, ZJUH (not examined but original description checked). Country of type locality: China.

**Geographical distribution.**OTL.

**OTL**: China (FJ, GD, GX, HI, JX, ZJ).

***Dolichogenideainfima* (Haliday, 1834)**

*Microgasterinfimus* Haliday, 1834.

**Type information.** Holotype female, NHMUK (examined). Country of type locality: United Kingdom.

**Geographical distribution.**PAL.

**PAL**: Azerbaijan, Czech Republic, Finland, France, Georgia, Germany, Hungary, Ireland, Italy, Kazakhstan, Lithuania, Macedonia, Mongolia, Netherlands, Poland, Romania, Russia (ZAB, KDA, PRI, YAR), Sweden, Switzerland, Turkey, United Kingdom, Uzbekistan, Yugoslavia.

***Dolichogenideainfirmus* Liu & Chen, 2019**

*Dolichogenideainfirmus* Liu & Chen, 2019.

**Type information.** Holotype female, ZJUH (not examined but original description checked). Country of type locality: China.

**Geographical distribution.**OTL.

**OTL**: China (FJ, GD, GZ, HN, ZJ).

***Dolichogenideainquisitor* (Wilkinson, 1928)**

*Apantelesinquisitor* Wilkinson, 1928.

**Type information.** Holotype female, NHMUK (examined). Country of type locality: Malaysia.

**Geographical distribution.**AUS, OTL.

**AUS**: Fiji; **OTL**: China (TW), Malaysia, Vietnam.

***Dolichogenideainterpolata* (Papp, 1975)**

*Apantelesinterpolatus* Papp, 1975.

**Type information.** Holotype female, HNHM (not examined but original description checked). Country of type locality: Hungary.

**Geographical distribution.**PAL.

**PAL**: Hungary.

***Dolichogenideairanica* (Telenga, 1955)**

*Apantelesiranicus* Telenga, 1955.

**Type information.** Type and depository unknown (not examined but subsequent treatment of the species checked). Country of type locality: Iran.

**Geographical distribution.**PAL.

**PAL**: Iran, Kazakhstan, Mongolia.

**Notes.** Our species concept is based on Papp (1978a) and Tobias (1986).

***Dolichogenideairiarte* (Nixon, 1965), new combination**

*Apantelesiriarte* Nixon, 1965.

**Type information.** Holotype female, USNM (examined). Country of type locality: Philippines.

**Geographical distribution.**OTL.

**OTL**: Philippines.

**Notes.** Transferred to *Dolichogenidea* based on the hind wing vannal lobe being more or less straight and setose, and the posterior margin of anteromesoscutum with punctures that do not fuse with each other. The label of the type spells the name as *iriate*; however, the proper name, as spelled in the original description and accounts of this species after that, is *iriarte*.

***Dolichogenideaiulis* (Nixon, 1967)**

*Apantelesiulis* Nixon, 1967.

**Type information.** Holotype female, NHMUK (examined). Country of type locality: Papua New Guinea.

**Geographical distribution.**AUS, OTL.

**AUS**: Papua New Guinea; **OTL**: Vietnam.

***Dolichogenideajaroshevskyi* (Tobias, 1976)**

*Apantelesjaroshevskyi* Tobias, 1976.

**Type information.** Holotype female, ZIN (not examined but subsequent treatment of the species checked). Country of type locality: Ukraine.

**Geographical distribution.**PAL.

**PAL**: Armenia, Russia (S), Ukraine.

**Notes.** Our species concept is based on Papp (1981) and Tobias (1986). The holotype is presumed to be in the ZIN based on Tobias (1986).

***Dolichogenideajilinensis* Chen & Song, 2004**

*Dolichogenideajilinensis* Chen & Song, 2004.

**Type information.** Holotype female, FAFU (not examined but original description checked). Country of type locality: China.

**Geographical distribution.**PAL.

**PAL**: China (JL).

***Dolichogenideajosealfredohernandezi* Fernandez-Triana & Boudreault, 2019**

*Dolichogenideajosealfredohernandezi* Fernandez-Triana & Boudreault, 2019.

**Type information.** Holotype female, CNC (examined). Country of type locality: Costa Rica.

**Geographical distribution.**NEO.

**NEO**: Costa Rica.

***Dolichogenideakelleri* Fagan-Jeffries & Austin, 2019**

*Dolichogenideakelleri* Fagan-Jeffries & Austin, 2019.

**Type information.** Holotype female, SAMA (not examined but original description checked). Country of type locality: Australia.

**Geographical distribution.**AUS.

**AUS**: Australia (SA).

***Dolichogenideakunhi* Gupta & Kalesh, 2012**

*Dolichogenideakunhi* Gupta & Kalesh, 2012.

**Type information.** Holotype female, NBAIR (not examined but original description checked). Country of type locality: India.

**Geographical distribution.**OTL.

**OTL**: India.

***Dolichogenideakurosawai* (Watanabe, 1940)**

*Apanteleskurosawai* Watanabe, 1940.

**Type information.** Holotype female, EIHU (examined). Country of type locality: Japan.

**Geographical distribution.**OTL, PAL.

**OTL**: China (FJ, GD, ZJ); **PAL**: China (NM), Japan.

**Notes.** After examining the holotype and other specimens (in the Hokkaido collection) we had decided to transfer this species to *Dolichogenidea* based on the hind wing vannal lobe being entirely setose. However, the species was transferred right before our paper by Liu et al. (2019), who also provided additional distribution records from China, an information we incorporate here.

***Dolichogenidealabaris* (Nixon, 1967)**

*Apanteleslabaris* Nixon, 1967.

**Type information.** Holotype female, NHMUK (examined). Country of type locality: Fiji.

**Geographical distribution.**AUS.

**AUS**: Fiji.

***Dolichogenidealacteicolor* (Viereck, 1911)**

*Apanteleslacteicolor* Viereck, 1911.

*Apantelesconspersae* Fiske, 1911.

**Type information.** Holotype female, USNM (examined). Country of type locality: USA.

**Geographical distribution.**NEA, OTL, PAL.

**NEA**: Canada (NB, NS, QC), USA (CO, CT, MA, NH); **OTL**: China (FJ, GX, GZ, HN, TW, YN, ZJ); **PAL**: Afghanistan, Armenia, Austria, Azerbaijan, Bulgaria, China (HA, SX), Finland, France, Germany, Hungary, Iran, Israel, Italy, Japan, Kazakhstan, Korea, Lithuania, Moldova, Mongolia, Poland, Portugal, Romania, Russia (KIR, KDA, PRI, SAK, VOR, YAR), Slovakia, Spain, Switzerland, Tajikistan, Turkey, Ukraine, United Kingdom, Uzbekistan, Yugoslavia.

***Dolichogenidealacteipennis* (Curtis, 1830)**

*Microgasterlacteipennis* Curtis, 1830.

*Apanteleslissonotus* Tobias, 1964.

*Microgasterlacteipennis* Curtis, 1829 [*nomen nudum*].

**Type information.** Holotype male, MVMMA (not examined but subsequent treatment of the species checked). Country of type locality: United Kingdom.

**Geographical distribution.**PAL.

**PAL**: Czech Republic, Germany, Italy, Kazakhstan, Latvia, Mongolia, Poland, Russia (C, NW), Slovakia, United Kingdom.

**Notes.** Our species concept is based on Papp (1978a, 1988).

***Dolichogenidealaevigata* (Ratzeburg, 1848)**

*Microgasterlaevigatus* Ratzeburg, 1848.

*Microgasterhoplites* Ratzeburg, 1848.

*Apantelescalcaratus* Ivanov, 1899.

**Type information.** Holotype male, ZMHB (not examined but subsequent treatment of the species checked). Country of type locality: Germany.

**Geographical distribution.**OTL, PAL.

**OTL**: China (FJ); **PAL**: Armenia, Azerbaijan, Bulgaria, China (SN), Finland, France, Georgia, Germany, Hungary, Israel, Italy, Kazakhstan, Korea, Latvia, Lithuania, Moldova, Netherlands, Poland, Romania, Russia (ALT, MOS, PRI, ROS, SPE, VOR), Serbia, Slovakia, Spain, Sweden, Switzerland, Turkey, Ukraine, United Kingdom, Uzbekistan.

**Notes.** Our species concept is based on Wilkinson (1945), Nixon (1972), Papp (1978a) and Chen and Song (2004).

***Dolichogenidealaevigatoides* (Nixon, 1972)**

*Apanteleslaevigatoides* Nixon, 1972.

**Type information.** Holotype female, NHMUK (examined). Country of type locality: United Kingdom.

**Geographical distribution.**PAL.

**PAL**: Czech Republic, Germany, Hungary, Poland, Russia (C, S), Switzerland, United Kingdom.

***Dolichogenidealaevissima* (Ratzeburg, 1848)**

*Microgasterlaevissimus* Ratzeburg, 1848.

*Apanteleslevissimus* Dalla Torre, 1898 [unjustified emendation].

*Apantelestersus* Papp, 1973.

**Type information.** Holotype male, depository unknown (not examined but subsequent treatment of the species checked). Country of type locality: France.

**Geographical distribution.**PAL.

**PAL**: Czech Republic, France, Germany, Hungary, Romania, Ukraine, United Kingdom.

**Notes.** Our species concept is based on Wilkinson (1945), Nixon (1973), Papp (1984a) and Tobias (1986). The holotype specimen was deposited in the Forestry College of Eberswalde (Forstlichen Hochschule Eberswalde). Unfortunately, that collection was mostly destroyed during the Second World War; however, five drawers with Hymenoptera specimens, among them type species of Ratzeburg were spared and are now safe at the Senckenberg Deutsches Entomologisches Institut (SDEI) in Müncheberg, Germany [see a detailed story in Schulz et al. (2018: 285-286)]. We do not know if the holotype of this species is at present in Müncheberg.

***Dolichogenidealakhaensis* (Ray & Yousuf, 2009), new combination**

*Apanteleslakhaensis* Ray & Yousuf, 2009.

**Type information.** Holotype female, IFRI (not examined but original description checked). Country of type locality: India.

**Geographical distribution.**OTL.

**OTL**: India.

**Notes.** Based on the original description and drawings (which clearly show a setose vannal lobe in the hind wing), this species is better placed within *Dolichogenidea*. Additional characters and details provided in the original description, e.g., anteromesoscutum and scutellar disc sculpture, and its similarity with another species of *Dolichogenidea* (*D.hyblaea*), also support the generic placement that we propose here.

***Dolichogenidealampe* (Nixon, 1965), new combination**

*Apanteleslampe* Nixon, 1965.

**Type information.** Holotype female, USNM (examined). Country of type locality: Philippines.

**Geographical distribution.**OTL.

**OTL**: Philippines.

**Notes.** Transferred to *Dolichogenidea* based on the hind wing vannal lobe being more or less straight but uniformly setose, and the posterior margin of the anteromesoscutum with few punctures that do not fuse with each other.

***Dolichogenidealaspeyresiae* (Viereck, 1913)**

*Apanteleslaspeyresiae* Viereck, 1913.

**Type information.** Holotype female, USNM (examined). Country of type locality: USA.

**Geographical distribution.**NEA.

**NEA**: Canada (BC), USA (CA, ID, OR).

***Dolichogenidealaspeyresiella* (Papp, 1972), new combination**

*Apanteleslaspeyresiella* Papp, 1972.

**Type information.** Holotype female, HNHM (not examined but paratype examined). Country of type locality: Hungary.

**Geographical distribution.**PAL.

**PAL**: Austria, Azerbaijan, Belarus, Bulgaria, China (NX), Hungary, Iran, Romania, Russia (PRI), Turkey.

**Notes.** We have examined one female and one male paratypes deposited in the CNC, and we think that the best generic placement is in *Dolichogenidea*. The propodeum lacks the strongly defined median carina that characterizes *Iconella* (instead it has several rugae near the nucha, defining a partial areola posteriorly); and the hind wing vein cu-a is not sinuous but straight. The original description and subsequent treatment of the species (Papp 1972, Nixon 1976) also suggest this species belongs to *Dolichogenidea*. The species distribution in Azerbaijan is based on Belokobylskij et al. (2019).

***Dolichogenidealaticauda* Chen & Song, 2004**

*Dolichogenidealaticauda* Chen & Song, 2004.

**Type information.** Holotype female, FAFU (not examined but original description checked). Country of type locality: China.

**Geographical distribution.**OTL.

**OTL**: China (FJ).

***Dolichogenidealatistigma* (Papp, 1977), new combination**

*Apanteleslatistigma* Papp, 1977.

**Type information.** Holotype female, HNHM (not examined but original description checked). Country of type locality: Mongolia.

**Geographical distribution.**PAL.

**PAL**: Mongolia.

**Notes.** The original description and illustrations, as well as comparison with similar European species in the key by Papp (1978a), strongly support *latistigma* to be a species of *Dolichogenidea*.

***Dolichogenidealatitergita* Liu & Chen, 2019**

*Dolichogenidealatitergita* Liu & Chen, 2019.

**Type information.** Holotype female, ZJUH (not examined but original description checked). Country of type locality: China.

**Geographical distribution.**OTL, PAL.

**OTL**: China (ZJ); **PAL**: China (HL, JL, LN, SC, SD).

***Dolichogenidealebene* (Nixon, 1967), new combination**

*Apanteleslebene* Nixon, 1967.

**Type information.** Holotype female, NHMUK (examined). Country of type locality: India.

**Geographical distribution.**OTL.

**OTL**: India.

**Notes.** Transferred to *Dolichogenidea* based on the hind wing vannal lobe being entirely setose.

***Dolichogenidealemariei* (Nixon, 1961)**

*Apanteleslemariei* Nixon, 1961.

**Type information.** Holotype female, MMBC (not examined but original description checked). Country of type locality: Czech Republic.

**Geographical distribution.**PAL.

**PAL**: Czech Republic, Hungary, Poland, Russia (SPE), United Kingdom.

***Dolichogenidealevifida* (Kotenko, 1992)**

*Apanteleslevifidus* Kotenko, 1992.

**Type information.** Holotype female, SIZK (not examined but subsequent treatment of the species checked). Country of type locality: Russia.

**Geographical distribution.**PAL.

**PAL**: Russia (ZAB).

**Notes.** Our species concept is based on Kotenko (2006).

***Dolichogenidealincostulata* Liu & Chen, 2019**

*Dolichogenidealincostulata* Liu & Chen, 2019.

**Type information.** Holotype female, ZJUH (not examined but original description checked). Country of type locality: China.

**Geographical distribution.**OTL.

**OTL**: China (FJ).

**Notes.** Based on the short ovipositor sheaths and hypopygium, as well as the similarities that Liu et al. (2019) stated *lincostulata* had with *Apanteleshyposidrae* Wilkinson, 1928 (transferred to *Parapanteles* by us, see below under that species), it is likely that *lincostulata* ends placed in a different genus in the future. However, until more study is done, we prefer here to retain it in *Dolichogenidea*.

***Dolichogenidealineipes* (Wesmael, 1837)**

*Microgasterlineipes* Wesmael, 1837.

**Type information.** Holotype female, RBINS (not examined but subsequent treatment of the species checked). Country of type locality: Belgium.

**Geographical distribution.**PAL.

**PAL**: Austria, Belgium, Bulgaria, Croatia, Czech Republic, Finland, France, Germany, Hungary, Israel, Italy, Latvia, Mongolia, Poland, Romania, Russia (SPE), Slovakia, Switzerland, United Kingdom.

**Notes.** Our species concept is based on Nixon (1972), Papp (1980a) and Tobias (1986).

***Dolichogenidealipsis* (Nixon, 1967)**

*Apanteleslipsis* Nixon, 1967.

**Type information.** Holotype female, NHMUK (examined). Country of type locality: Australia.

**Geographical distribution.**AUS.

**AUS**: Australia (WA).

***Dolichogenidealissos* (Nixon, 1967)**

*Apanteleslissos* Nixon, 1967.

**Type information.** Holotype female, NHMUK (examined). Country of type locality: China.

**Geographical distribution.**OTL.

**OTL**: China (GD, HI, SC).

**Notes.** After examining the holotype we had decided to transfer this species to *Dolichogenidea* based on the hind wing vannal lobe being entirely setose and the punctures on the anteromesoscutum not fusing near scutoscutellar sulcus. However, the species was transferred right before our paper by Liu et al. (2019), who also provided additional distribution records from China, an information we incorporate here. Because the name is to be considered as a noun under ICZN Article 31.2.1, it must retain its original spelling and remain as *lissos*.

***Dolichogenidealobesiae* Fagan-Jeffries & Austin, 2019**

*Dolichogenidealobesiae* Fagan-Jeffries & Austin, 2019.

**Type information.** Holotype female, QM (not examined but original description checked). Country of type locality: Australia.

**Geographical distribution.**AUS.

**AUS**: Australia (QLD).

***Dolichogenidealocastrae* (You & Tong, 1987)**

*Apanteleslocastrae* You & Tong, 1987.

**Type information.** Holotype female, HUNAU (not examined but subsequent treatment of the species checked). Country of type locality: China.

**Geographical distribution.**OTL.

**OTL**: China (HN, ZJ).

**Notes.** Our species concept is based on Chen and Song (2004).

***Dolichogenidealongialba* Liu & Chen, 2019**

*Dolichogenidealongialba* Liu & Chen, 2019.

**Type information.** Holotype female, ZJUH (not examined but original description checked). Country of type locality: China.

**Geographical distribution.**PAL.

**PAL**: China (XJ).

***Dolichogenidealongicalcar* (Thomson, 1895)**

*Microgasterlongicalcar* Thomson, 1895.

**Type information.** Lectotype female, MZLU (not examined but subsequent treatment of the species checked). Country of type locality: Sweden.

**Geographical distribution.**PAL.

**PAL**: Czech Republic, Finland, Hungary, Korea, Russia (KR, PRI), Sweden, Switzerland, United Kingdom.

**Notes.** Our species concept is based on Nixon (1973), Papp (1980a) and Tobias (1986).

***Dolichogenidealongicauda* (Wesmael, 1837)**

*Microgasterlongicauda* Wesmael, 1837.

*Apanteleslongicaudis* Marshall, 1885 [unjustified emendation].

*Apanteleslongicauderra* Shenefelt, 1972.

**Type information.** Holotype female, RBINS (not examined but subsequent treatment of the species checked). Country of type locality: Belgium.

**Geographical distribution.**NEA, PAL.

**NEA**: Canada (BC), USA (WA); **PAL**: Afghanistan, Armenia, Austria, Azerbaijan, Belarus, Belgium, Bulgaria, Estonia, Finland, France, Georgia, Germany, Hungary, Italy, Korea, Latvia, Lithuania, Moldova, Mongolia, Netherlands, Poland, Romania, Russia (BU, KDA, SPE), Serbia, Slovakia, Slovenia, Spain, Switzerland, Turkey, Turkmenistan, Ukraine, United Kingdom.

**Notes.** This species was synonymized under *D.candidata* by van Achterberg (1997), a decision that has been subsequently followed by most authors (e.g., Belokobylskij et al. 2003, Kotenko 2007, Papp 2009a, Broad et al. (2016). However, Fernandez-Triana and Huber (2010) and Fernandez-Triana et al. (2014b) considered it a valid species, based on Mason (1981) and Whitfield (1995a). Here we are considering them as separate species until further study allow us to conclude on this.

***Dolichogenidealongimagna* Liu & Chen, 2019**

*Dolichogenidealongimagna* Liu & Chen, 2019.

**Type information.** Holotype female, ZJUH (not examined but original description checked). Country of type locality: China.

**Geographical distribution.**OTL.

**OTL**: China (SC).

***Dolichogenidealongipalpis* (Reinhard, 1880)**

*Apanteleslongipalpis* Reinhard, 1880.

*Apantelestadzhicus* Telenga, 1949.

**Type information.** Type and depository unknown (not examined but subsequent treatment of the species checked). Country of type locality: Germany.

**Geographical distribution.**OTL, PAL.

**OTL**: China (JS); **PAL**: Armenia, Finland, Germany, Greece, Hungary, Iran, Poland, Romania, Russia (S, NC), Slovakia, Tajikistan, Turkey, Ukraine, United Kingdom.

**Notes.** Our species concept is based on Nixon (1965), Papp (1981) and Shaw (2012b). Papp (1981) reported that the type series of *Apantelestadzhicus* includes two species (*lacteus* and *longipalpis*) but he did not select a lectotype; Belokobylskij et al. (2003) treated the name as a synonym of *longipalpis*. The species distribution in Greece and Iran are based on Belokobylskij et al. (2019) and Kavallieratos et al. (2019).

***Dolichogenidealongituba* Song & Chen, 2004**

*Dolichogenidealongituba* Song & Chen, 2004.

**Type information.** Holotype female, FAFU (not examined but original description checked). Country of type locality: China.

**Geographical distribution.**OTL.

**OTL**: China (FJ).

***Dolichogenidealongivena* Liu & Chen, 2018**

*Dolichogenidealongivena* Liu & Chen, 2018.

**Type information.** Holotype female, ZJUH (not examined but original description checked). Country of type locality: China.

**Geographical distribution.**OTL.

**OTL**: China (FJ, GD, GX, HI, SC, ZJ).

***Dolichogenidealucidinervis* (Wilkinson, 1928), new combination**

*Apanteleslucidinervis* Wilkinson, 1928.

*Urogasteralbinervis* Ashmead, 1905 [primary junior homonym of *Urogasteralbinervis* Cameron, 1904].

**Type information.** Holotype male, USNM (examined). Country of type locality: Philippines.

**Geographical distribution.**OTL.

**OTL**: Philippines.

**Notes.** We examined the holotype and it clearly belongs to *Dolichogenidea*, based on its convex, uniformly setose vannal lobe in the hind wing, and the anteromesoscutum with punctures that do not fuse near the scutoscutellar sulcus.

***Dolichogenidealuctifica* (Papp, 1971)**

*Apantelesluctificus* Papp, 1971.

*Apantelesanfitrion* Nixon, 1972.

**Type information.** Holotype female, HNHM (not examined but subsequent treatment of the species checked). Country of type locality: Mongolia.

**Geographical distribution.**PAL.

**PAL**: Finland, Hungary, Mongolia, Russia (PRI, SPE), Yugoslavia.

**Notes.** This species was described from Mongolia by Papp (1971) as *Apantelesluctificus*. A year later, Nixon (1972) described the species *Apantelesanfitrion* from Finland and Yugoslavia, the Finnish specimen being the holotype. Papp (1978a: 278) was able to examine the type material of those two species and synonymized *anfitrion* under *luctificus*. However, the specimen from Finland was collected on the island of Tytärsaari (currently Bolshoi Tyuters), which became part of Russia after 1940. Thus, the record of this species as part of the Finnish fauna is questionable at present.

***Dolichogenidealumba* Rousse & Gupta, 2013**

*Dolichogenidealumba* Rousse & Gupta, 2013.

**Type information.** Holotype female, MNHN (not examined but original description checked). Country of type locality: Réunion.

**Geographical distribution.**AFR.

**AFR**: Réunion.

***Dolichogenidealunata* Liu & Chen, 2019**

*Dolichogenidealunatus* Liu & Chen, 2019.

**Type information.** Holotype female, ZJUH (not examined but original description checked). Country of type locality: China.

**Geographical distribution.**OTL.

**OTL**: China (YN).

***Dolichogenideamaetoi* Fernandez-Triana & Shimizu, 2018**

*Dolichogenideamaetoi* Fernandez-Triana & Shimizu, 2018.

**Type information.** Holotype female, NIAES (examined). Country of type locality: Japan.

**Geographical distribution.**PAL.

**PAL**: Japan.

**Notes.** The paper describing this species incorrectly stated the holotype as deposited in the CNC (Fernandez-Triana et al. 2018), when it is in fact deposited in the NIAES. This is corrected here.

***Dolichogenideamalacosomae* (Pandey, Ahmad, Haider & Shujauddin, 2004), new combination**

*Apantelesmalacosomae* Pandey, Ahmad, Haider & Shujauddin, 2004.

**Type information.** Holotype female, AMUZ (not examined but original description checked). Country of type locality: India.

**Geographical distribution.**OTL.

**OTL**: India.

**Notes.** Transferred to *Dolichogenidea* based on the original description mentioning and illustrating an entirely setose hind wing vannal lobe (fig. 2 in Pandey et al. 2004).

***Dolichogenideamarica* (Nixon, 1972)**

*Apantelesmarica* Nixon, 1972.

**Type information.** Holotype female, NHMUK (examined). Country of type locality: United Kingdom.

**Geographical distribution.**PAL.

**PAL**: Hungary, Switzerland, United Kingdom.

***Dolichogenideamaro* (Nixon, 1967), new combination**

*Apantelesmaro* Nixon, 1967.

**Type information.** Holotype female, NHMUK (examined). Country of type locality: India.

**Geographical distribution.**OTL.

**OTL**: India.

**Notes.** Transferred to *Dolichogenidea* based on the hind wing with the vannal lobe entirely setose, and the anteromesoscutum with punctures that do not fuse near the posterior margin. In the original description, no details on the etymology of the species name were provided; as first revisers we consider it as a noun in apposition and thus its gender to be neuter, following Article 31.2.2 of the ICZN.

***Dolichogenideamarokkana* (Fahringer, 1936)**

*Apantelesmarokkanus* Fahringer, 1936.

**Type information.** Type and depository unknown (not examined but subsequent treatment of the species checked). Country of type locality: Morocco.

**Geographical distribution.**PAL.

**PAL**: Morocco.

**Notes.** Our species concept is based on Papp (1981) and Liu et al. (2015).

***Dolichogenideamasoni* Pandey, Ahmad, Haider & Shujauddin, 2005**

*Dolichogenideamasoni* Pandey, Ahmad, Haider & Shujauddin, 2005.

**Type information.** Holotype female, AMUZ (not examined but original description checked). Country of type locality: India.

**Geographical distribution.**OTL.

**OTL**: India.

***Dolichogenideamedicava* Liu & Chen, 2019**

*Dolichogenideamedicava* Liu & Chen, 2019.

**Type information.** Holotype female, ZJUH (not examined but original description checked). Country of type locality: China.

**Geographical distribution.**OTL.

**OTL**: China (FJ).

***Dolichogenideamediocaudata* Fagan-Jeffries & Austin, 2018**

*Dolichogenideamediocaudata* Fagan-Jeffries & Austin, 2018.

**Type information.** Holotype female, ANIC (not examined but original description checked). Country of type locality: Australia.

**Geographical distribution.**AUS.

**AUS**: Australia (NSW).

***Dolichogenideamelaniamunozae* Fernandez-Triana & Boudreault, 2019**

*Dolichogenideamelaniamunozae* Fernandez-Triana & Boudreault, 2019.

**Type information.** Holotype female, CNC (examined). Country of type locality: Costa Rica.

**Geographical distribution.**NEO.

**NEO**: Costa Rica.

***Dolichogenideamelanopus* (Viereck, 1917)**

*Apantelesmelanopus* Viereck, 1917.

**Type information.** Holotype female, USNM (examined). Country of type locality: USA.

**Geographical distribution.**NEA.

**NEA**: Canada (AB, BC, MB, NL, PE, QC, SK, YT), USA (AK, CT).

**Notes.** The holotype is in relatively poor condition, missing the left pair of wings and most of the antennae. Following Article 31.2.1 of the ICZN the name is a noun phrase in apposition and the original spelling *melanopus* must be retained.

***Dolichogenideamendosae* (Wilkinson, 1929), new combination**

*Apantelesmendosae* Wilkinson, 1929.

**Type information.** Holotype female, NHMUK (examined). Country of type locality: Malaysia.

**Geographical distribution.**OTL.

**OTL**: Malaysia.

**Notes.** The holotype has the hind wing vannal lobe entirely setose, indicating the species belongs in *Dolichogenidea*.

***Dolichogenideamesocanalis* Liu & Chen, 2018**

*Dolichogenideamesocanalis* Liu & Chen, 2018.

**Type information.** Holotype female, ZJUH (not examined but original description checked). Country of type locality: China.

**Geographical distribution.**OTL.

**OTL**: China (YN).

***Dolichogenideametesae* (Nixon, 1967)**

*Apantelesmetesae* Nixon, 1967.

**Type information.** Holotype female, NHMUK (examined). Country of type locality: Malaysia.

**Geographical distribution.**OTL, PAL.

**OTL**: China (GX, HI, ZJ), Malaysia, Vietnam; **PAL**: China (SH).

***Dolichogenideamiantonomoi* (Viereck, 1917)**

*Apantelesmiantonomoi* Viereck, 1917.

*Apantelespequodorum* Viereck, 1917.

**Type information.** Holotype female, USNM (examined). Country of type locality: USA.

**Geographical distribution.**NEA.

**NEA**: USA (CT, MI).

**Notes.** We also examined the type of *A.pequodorum* (synonym of *A.miantonomoi*).

***Dolichogenideamidas* (Nixon, 1972)**

*Apantelesmidas* Nixon, 1972.

**Type information.** Holotype female, MZH (not examined but original description checked). Country of type locality: Finland.

**Geographical distribution.**PAL.

**PAL**: Finland, Hungary, Mongolia, Russia (BA, PRI).

***Dolichogenideamimi* (Papp, 1974)**

*Apantelesmimi* Papp, 1974.

**Type information.** Holotype female, HNHM (not examined but original description checked). Country of type locality: Hungary.

**Geographical distribution.**PAL.

**PAL**: Hungary, Moldova, Ukraine.

***Dolichogenideaminuscula* Liu & Chen, 2019**

*Dolichogenideaminuscula* Liu & Chen, 2019.

**Type information.** Holotype female, ZJUH (not examined but original description checked). Country of type locality: China.

**Geographical distribution.**OTL.

**OTL**: China (FJ, ZJ).

***Dolichogenideamira* (Papp, 1977)**

*Apantelesmirus* Papp, 1977.

**Type information.** Holotype female, HNHM (not examined but original description checked). Country of type locality: Hungary.

**Geographical distribution.**PAL.

**PAL**: Hungary.

***Dolichogenideamiris* (Nixon, 1967)**

*Apantelesmiris* Nixon, 1967.

**Type information.** Holotype female, NHMUK (examined). Country of type locality: Australia.

**Geographical distribution.**AUS.

**AUS**: Australia (ACT).

***Dolichogenideamolestae* (Muesebeck, 1933)**

*Apantelesmolestae* Muesebeck, 1933.

**Type information.** Holotype female, USNM (examined). Country of type locality: Japan.

**Geographical distribution.**OTL, PAL.

**OTL**: China (TW), Ryukyu Islands; **PAL**: Japan, Korea.

**Notes.** After examining the holotype we had decided to transfer this species to *Dolichogenidea* based on the entirely setose hind wing vannal lobe (the specimen also has a very distinctive T1 with strong longitudinal striation). However, the species was transferred just before our paper by Liu et al. (2019).

***Dolichogenideamonticola* (Ashmead, 1890), new combination**

*Apantelesmonticola* Ashmead, 1890.

**Type information.** Holotype male, USNM (examined). Country of type locality: USA.

**Geographical distribution.**NEA.

**NEA**: USA (CO, CT, ID, NM).

**Notes.** Transferred to *Dolichogenidea* because of the convex and fully setose hind wing vannal lobe. The specimen is missing both antenna, one pair of wings and some legs.

***Dolichogenideamulticolor* Liu & Chen, 2019**

*Dolichogenideamulticolor* Liu & Chen, 2019.

**Type information.** Holotype female, ZJUH (not examined but original description checked). Country of type locality: China.

**Geographical distribution.**OTL.

**OTL**: China (GD).

***Dolichogenideamurinanae* (Capek & Zwölfer, 1957), status revised**

*Apantelesmurinanae* Capek & Zwölfer, 1957.

? *Apantelesdioryctriae* Wilkinson, 1938.

? *Apantelesmagnus* Telenga, 1955.

**Type information.** Holotype female, MMBC (not examined but subsequent treatment of the species checked). Country of type locality: Slovakia.

**Geographical distribution.**PAL.

**PAL**: Austria, Czech Republic, Finland, France, Germany, Italy, Lithuania, Mongolia, Morocco, Poland, Romania, Russia (SA), Slovakia, Switzerland, Turkey, United Kingdom.

**Notes.***Apantelesmurinanae* Capek & Zwölfer, altogether with *A.dioryctriae* Wilkinson and *A.magnus* Telenga, were all synonymized under *Apantelespetrovae* Walley, 1937 by Papp (1980a: 253), who based his decision on the study of one female and one male specimens of *petrovae* sent to him by Mason (CNC). However, Capek (1989) reexamined the situation in more detail, by studying host relations, geographical distribution, and larval taxonomy of the species involved, and he concluded that the synonyms were not warranted. After that, some authors (e.g., Shaw 2012b, Broad et al. 2016) have considered all involved species as junior synonyms of *petrovae*, while others have considered *murinanae* as a valid species (e.g., Mills and Kenis 1991, van Achterberg 2014, Yu et al. 2016). However, those references did not assess the species involved, but just followed either Papp (1980a) or Capek (1989). We have examined the holotype, paratypes and additional specimens of *Apantelespetrovae* (all deposited in the CNC), and have compared them versus *Apantelesmurinanae* specimens (also deposited in the CNC, some of that material collected by Zwölfer and apparently identified by Capek; the rest of the material coming from France and apparently part of the specimens studied by Mills and Kenis 1991). There are slight morphological differences among those two groups of specimens, but most importantly, there are also substantial differences in DNA barcodes. There are more than 15 *petrovae* specimens with sequences available, representing BINBOLD:AAA6374; whereas the only barcode compliant sequence of *murinanae* (there are two other specimens with sequences available for this species, they are just minibarcodes, with only 144 bp) represents BINBOLD:AAZ7315. Both BINs are the closest between each other in BOLD, but still have 6% bp difference, which suggest they represent two different species. It may even be possible that they are part of a complex of morphologically cryptic species, but study of more specimens from the range of *petrovaesensu lato* would be needed, including obtaining more DNA barcodes. In this paper we restrict the name *petrovae* to American specimens, while considering the Palearctic specimens to represent a different species, *murinanae*. If all European specimens would actually end up belonging to just one species, then the proper name should actually be *Apantelesdioryctriae* Wilkinson, 1938 (the oldest, senior synonym). However, because we have not been able to study more specimens, we prefer to use *Apantelesmurinanae* Capek & Zwölfer, 1957 for the time being, as it has been more widely used than *dioryctriae* or *magnus* (these last two names have been considered as junior synonyms since 1980). Because it is impossible to conclude on the status of *dioryctriae* and *magnus* with the evidence available at present (they could be synonyms of *murinanae* or *petrovae*, or even valid species on their own); here we provisionally include them as synonyms of both *murinanae* and *petrovae*, with question marks to indicate this matter will require further investigation. In addition to the nomenclatural changes discussed above, we have also assessed the best generic placement for both *murinanae* and *petrovae*, and have decided to maintain them within *Dolichogenidea*. In the specimens we have studied (which include the type of *dioryctriae* in the NHMUK, non-type material from *murinanae* in the CNC, type and non-type material of *petrovae* in the CNC) the vannal lobe is more or less straight and with very minute setae that are sparse but still look like a fringe. Both species could be considered borderline between *Apanteles* and *Dolichogenidea*, but we have based our decision not only on morphology but also on DNA barcodes, which cluster with dozens of other *Dolichogenidea* species in BOLD, far apart from *Apanteles*.

***Dolichogenideamycale* (Nixon, 1972)**

*Apantelesmycale* Nixon, 1972.

**Type information.** Holotype female, NHMUK (examined). Country of type locality: Sweden.

**Geographical distribution.**OTL, PAL.

**OTL**: China (GZ, SC); **PAL**: Bulgaria, China (JL, LN), Czech Republic, Finland, Hungary, Poland, Slovakia, Sweden, Tunisia, Turkey.

**Notes.** Our species concept is based on Shaw (2012b).

***Dolichogenideamyron* (Nixon, 1973)**

*Apantelesmyron* Nixon, 1973.

**Type information.** Holotype female, NHMUK (examined). Country of type locality: United Kingdom.

**Geographical distribution.**PAL.

**PAL**: Austria, Finland, Germany, Greece, Switzerland, Turkey, United Kingdom.

**Notes.**Broad et al. (2016: 227-228) wrote that this species was being “Transferred from *Apanteles* in anticipation of publication by Jose Fernandez-Triana”, although the new combination was not made explicit in that paper. The holotype has the hind wing vannal lobe setose.

***Dolichogenideanigra* (Muesebeck, 1921), new combination**

*Apantelesniger* Muesebeck, 1921.

**Type information.** Holotype female, USNM (examined). Country of type locality: USA.

**Geographical distribution.**NEA.

**NEA**: USA (DC, ID, KS, MI, MN, NY, SD, VA).

**Notes.** After examining the type and several other specimens (in the USNM and CNC) determined by Muesebeck, we consider this species to belong to *Dolichogenidea*. The vannal lobe on the hind wing is fully setose, and the anteromesoscutum has very few and shallow puncture which do not fuse near the scutoscutellar sulcus.

***Dolichogenideanixosiris* (Papp, 1976)**

*Apantelesnixosiris* Papp, 1976.

*Apantelesosiris*Nixon 1972 [primary junior homonym of *Apantelesosiris* de Saeger, 1944].

**Type information.** Holotype female, MZH (not examined but original description checked). Country of type locality: Russia.

**Geographical distribution.**PAL.

**PAL**: China (HE, HL, NM, XJ), Finland, Hungary, Mongolia, Russia (ZAB, KR, NVS, PRI), Turkmenistan.

***Dolichogenideanovoguineensis* (Szépligeti, 1905)**

*Apantelesnovo-guineensis* Szépligeti, 1905.

**Type information.** Lectotype female, HNHM (not examined but subsequent treatment of the species checked). Country of type locality: Papua New Guinea.

**Geographical distribution.**AUS.

**AUS**: Papua New Guinea.

**Notes.** Our species concept is based on Austin and Dangerfield (1992) and Papp (2004).

***Dolichogenideanumenes* (Nixon, 1967)**

*Apantelesnumenes* Nixon, 1967.

**Type information.** Holotype female, NHMUK (examined). Country of type locality: Indonesia.

**Geographical distribution.**OTL.

**OTL**: China (HI), Indonesia, Vietnam.

**Notes.** The species distribution in China is based on Liu et al. (2019).

***Dolichogenideaoblicarina* Chen & Song, 2004**

*Dolichogenideaoblicarina* Chen & Song, 2004.

**Type information.** Holotype female, FAFU (not examined but original description checked). Country of type locality: China.

**Geographical distribution.**PAL.

**PAL**: China (JL).

***Dolichogenideaobscurugosa* Liu & Chen, 2018**

*Dolichogenideaobscurugosus* Liu & Chen, 2018.

**Type information.** Holotype female, ZJUH (not examined but original description checked). Country of type locality: China.

**Geographical distribution.**OTL, PAL.

**OTL**: China (ZJ); **PAL**: China (NM).

**Notes.** The species name must be treated as an adjective and not as a noun (Doug Yanega, pers. comm.) and thus it must match the gender of the genus name.

***Dolichogenideaobsoleta* Liu & Chen, 2019**

*Dolichogenideaobsoleta* Liu & Chen, 2019.

**Type information.** Holotype female, ZJUH (not examined but original description checked). Country of type locality: China.

**Geographical distribution.**OTL.

**OTL**: China (ZJ).

***Dolichogenideaobstans* (Papp, 1971)**

*Apantelesobstans* Papp, 1971.

**Type information.** Holotype female, HNHM (not examined but subsequent treatment of the species checked). Country of type locality: Mongolia.

**Geographical distribution.**PAL.

**PAL**: Kazakhstan, Mongolia, Slovakia.

**Notes.** Our species concept is based on Papp (1978a) and Tobias (1986).

***Dolichogenideaoehlkei* (Papp, 1982)**

*Apantelesoehlkei* Papp, 1982.

**Type information.** Holotype female, EBW (not examined but subsequent treatment of the species checked). Country of type locality: Germany.

**Geographical distribution.**PAL.

**PAL**: Germany.

**Notes.** Our species concept is based on Papp (1982, 1988) and Tobias (1986).

***Dolichogenideaoidaematophori* (Muesebeck, 1929)**

*Apantelesoidaematophori* Muesebeck, 1929.

*Apantelesoidematophori* Muesebeck, 1929 [incorrect original spelling].

**Type information.** Holotype female, USNM (examined). Country of type locality: USA.

**Geographical distribution.**NEA.

**NEA**: USA (ID, WI).

***Dolichogenideaolivierellae* (Wilkinson, 1936), new combination**

*Apantelesolivierellae* Wilkinson, 1936.

**Type information.** Holotype female, NHMUK (examined). Country of type locality: Algeria.

**Geographical distribution.**PAL.

**PAL**: Algeria, Morocco, Israel.

**Notes.** Since its original description, this species has been recognized to be a “remarkable species of *Apanteles*, since it possesses more than one character not previously described in the genus” (Wilkinson 1936b: 85). After examining the holotype we agree that there are some unique features, some not or very rarely present in *Apanteles*: strongly emarginate clypeus; mandible base separated from the head by a desclerotized area that looks like an opening; anteromesoscutum mostly smooth and shiny, the few punctures that are discernible (mostly on the anterior half of the anteromesoscutum) are shallow and sparse, never fusing with each other; scutoscutellar sulcus very narrow and shallow, almost imperceptible; propodeum almost entirely smooth and shiny, only with very short carinae near the nucha; ovipositor sheaths relatively short (ca. half the metatibia length), and fully setose; hind wing vannal lobe slightly convex to straight, with small setae that do not form a full fringe, but nevertheless cover more or less the entire area of the vannal lobe; hypopygium with narrow translucent area (more evident on the posterior third of the hypopygium, but very narrowly present on the anterior two thirds as well), the translucent area with one or two pleats barely visible. Wilkinson (1936b) mentioned other important features such as spines at the base of the ovipositor sheaths (which we could not see in the holotype), as well as the short antennae, overall smooth and shiny body, head shape, and uniqueness of the host caterpillar. It is clear that this species does not belong to *Apanteles* and, after studying the holotype, we think the best generic placement for the species is in *Dolichogenidea*.

***Dolichogenideaononidis* (Marshall, 1889), lectotype designation**

*Apantelesononidis* Marshall, 1889.

**Type information.** Lectotype female, NHMUK (examined). Country of type locality: United Kingdom.

**Geographical distribution.**PAL.

**PAL**: Germany, United Kingdom.

**Notes.** The original description was based on female and male specimens. We have examined a female in the NHMUK with a type label and a code 3c.45, which we here designate as the lectotype. Marshall (1889) described the species from *Gracillariaononidis* (now *Parechtopaononidis* (Zeller, 1839) (Gracillariidae)), and *Coleophorasalinella* Stainton, 1859 (Coleophoridae). The lectotype lacks host data but agrees with specimens we examined in the RSME which were reared in UK from *P.ononidis*.

***Dolichogenideaopacifinis* Liu & Chen, 2019**

*Dolichogenideaopacifinis* Liu & Chen, 2019.

**Type information.** Holotype female, ZJUH (not examined but original description checked). Country of type locality: China.

**Geographical distribution.**OTL.

**OTL**: China (HB, ZJ).

***Dolichogenideaovata* Liu & Chen, 2019**

*Dolichogenideaovata* Liu & Chen, 2019.

**Type information.** Holotype female, ZJUH (not examined but original description checked). Country of type locality: China.

**Geographical distribution.**OTL.

**OTL**: China (FJ).

***Dolichogenideapallidalata* (Tobias, 1964)**

*Apantelespallidalatus* Tobias, 1964.

**Type information.** Holotype female, ZIN (not examined but subsequent treatment of the species checked). Country of type locality: Kazakhstan.

**Geographical distribution.**PAL.

**PAL**: Kazakhstan, Russia (S), Ukraine.

**Notes.** Our species concept is based on Papp (1981) and Tobias (1986).

***Dolichogenideapalpator* (Tobias, 1960)**

*Apantelespalpator* Tobias, 1960.

**Type information.** Holotype female, ZIN (not examined but subsequent treatment of the species checked). Country of type locality: Tajikistan.

**Geographical distribution.**PAL.

**PAL**: Tajikistan.

**Notes.** Our species concept is based on Papp (1978a) and Tobias (1986).

***Dolichogenideaparacostulae* Liu & Chen, 2018**

*Dolichogenideaparacostulae* Liu & Chen, 2018.

**Type information.** Holotype female, ZJUH (not examined but original description checked). Country of type locality: China.

**Geographical distribution.**OTL.

**OTL**: China (ZJ).

***Dolichogenideaparalechiae* (Muesebeck, 1932)**

*Apantelesparalechiae* Muesebeck, 1932.

**Type information.** Holotype female, USNM (examined). Country of type locality: USA.

**Geographical distribution.**NEA.

**NEA**: Canada (NB, ON, QC), USA (CA, MA, MI, NH, NY, OH, PA, TN, WI).

***Dolichogenideaparallelis* (Ashmead, 1900), new combination**

*Protapantelesparallelis* Ashmead, 1900.

**Type information.** Holotype female, NHMUK (examined). Country of type locality: Saint Vincent.

**Geographical distribution.**NEO.

**NEO**: Saint Vincent.

**Notes.** The information about this species has been very limited. For his revision of Nearctic *Apanteles*, Muesebeck could not examine the only known specimen (the holotype being in London) and instead had to key it out based just on the original description (Muesebeck 1921: 491 and especially 523, see also Ashmead 1900c: 281). The only other source of information for the species is Shenefelt (1972: 595), who just lists the previous two references. Taxapad (Yu et al. 2012, 2016) refers to this species as *Cotesiaparallelis*, probably because most of the *Apanteles* in the part of the Muesebeck key where *parallelis* is placed are currently considered to belong to *Cotesia*. We have examined the female holotype (NHMUK) and conclude that it belongs to *Dolichogenidea*, as it has a fully setose hind wing vannal lobe. The species has a propodeum that is relatively smooth, but with a short carina near the nucha which partially defines an areola posteriorly. T1 and T2 are relatively smooth but T1 has some shallow longitudinal striations near the lateral margins. The anteromesoscutum and scutellar disc are mostly shiny and smooth (sparse, very shallow punctures on the anteromesoscutum). The ovipositor sheaths are slightly longer than the metatibia length. The antennae (except for scapes) are missing in the holotype. The overall colouration is about as described by Ahsmead (1900).

***Dolichogenideaparallodorsum* Liu & Chen, 2019**

*Dolichogenideaparallodorsum* Liu & Chen, 2019.

**Type information.** Holotype female, ZJUH (not examined but original description checked). Country of type locality: China.

**Geographical distribution.**OTL.

**OTL**: China (FJ, GD, ZJ).

***Dolichogenideaparametacarp* Liu & Chen, 2018**

*Dolichogenideaparametacarp* Liu & Chen, 2018.

**Type information.** Holotype female, ZJUH (not examined but original description checked). Country of type locality: China.

**Geographical distribution.**OTL, PAL.

**OTL**: China (FJ, GD, HI, HN, YN, ZJ); **PAL**: China (HL, JL, LN).

***Dolichogenideaparanthrenea* (You & Dang, 1987)**

*Apantelesparanthreneus* You & Dang, 1987.

**Type information.** Holotype female, HUNAU (not examined but subsequent treatment of the species checked). Country of type locality: China.

**Geographical distribution.**OTL, PAL.

**OTL**: China (FJ), **PAL**: China (SN).

**Notes.** Our species concept is based on Chen and Song (2004).

***Dolichogenideaparasae* (Rohwer, 1922)**

*Apantelesparasae* Rohwer, 1922.

*Urogasterphilippinensis* Ashmead, 1904 [primary junior homonym of *Apantelesphilippinensis* Ashmead, 1904].

**Type information.** Holotype female, USNM (examined). Country of type locality: Indonesia.

**Geographical distribution.**OTL.

**OTL**: China (GD, HI, HN, JX, TW, ZJ), India, Indonesia, Malaysia, Philippines, Sri Lanka, Thailand.

***Dolichogenideapartergita* Liu & Chen, 2018**

*Dolichogenideapartergita* Liu & Chen, 2018.

**Type information.** Holotype female, ZJUH (not examined but original description checked). Country of type locality: China.

**Geographical distribution.**OTL, PAL.

**OTL**: China (GD, GZ, HI, TW, YN, ZJ); **PAL**: China (JL, LN, SD).

***Dolichogenideapelopea* (Nixon, 1973), new combination**

*Apantelespelopea* Nixon, 1973.

**Type information.** Holotype female, NHMUK (examined). Country of type locality: Italy.

**Geographical distribution.**PAL.

**PAL**: China (NM), Italy, Mongolia.

**Notes.** Transferred to *Dolichogenidea* based on the hind wing vannal lobe being entirely setose, and the anteromesoscutum with punctures not fusing near the scutoscutellar sulcus. Liu et al. (2014) had already recognized the entirely setose vannal lobe, but the species had never been transferred to *Dolichogenidea* until now.

***Dolichogenideapelops* (de Saeger, 1944), new combination**

*Apantelespelops* de Saeger, 1944.

*Apantelespelopsbambeyduplus* Shenefelt, 1972 [new name for *Apantelespelopsbambeyi* Risbec, 1951, a homonym of *Apantelesbambeyi* Risbec, 1951].

**Type information.** Holotype female, RMCA (not examined but original description checked). Country of type locality: Democratic Republic of Congo.

**Geographical distribution.**AFR.

**AFR**: Democratic Republic of Congo, Rwanda, Senegal.

**Notes.** Here transferred to *Dolichogenidea* based on the anteromesoscutum punctures not fusing near the scutoscutellar sulcus, as well as the shape of the hypopygium and the length and shape of the ovipositor sheaths. Also, the original description compares this species as close to *Apantelescaniae* Wilkinson (placed in *Dolichogenidea* by Chen and Song 2004), and *Apanteleswittei* de Saeger (similarly placed in *Dolichogenidea* by us, see below under that species).

***Dolichogenideapentgona* Liu & Chen, 2019**

*Dolichogenideapentgona* Liu & Chen, 2019.

**Type information.** Holotype female, ZJUH (not examined but original description checked). Country of type locality: China.

**Geographical distribution.**OTL.

**OTL**: China (HI).

***Dolichogenideapetrovae* (Walley, 1937)**

*Apantelespetrovae* Walley, 1937.

? *Apantelesdioryctriae* Wilkinson, 1938.

? *Apantelesmagnus* Telenga, 1955.

**Type information.** Holotype female, CNC (examined). Country of type locality: Canada.

**Geographical distribution.**NEA.

**NEA**: Canada (AB, BC, MB, NB, NL, ON, QC, SK), USA (CA, CO, MI, MN, SC, WI)

**Notes.** This species has been variously treated as *Apanteles* or *Dolichogenidea* (Mason 1974 & 1981, Papp 1988, Whitfield 1995a, van Achterberg 2003, Fernandez-Triana 2010, Shaw 2012b). Fernandez-Triana (2010) mentioned that the morphological and molecular evidence was controversial (mostly pointing towards the species belonging to *Dolichogenidea*); however, he decided to maintain the species within *Apanteles* based on the examination of the holotype vannal lobe. After re-examination of the available evidence, including holotype, paratypes and other specimens, as well as DNA barcodes, we now consider that the best generic placement would be in *Dolichogenidea* (see more details and comments under *D.murinanae* above; both species could be considered borderline between *Apanteles* and *Dolichogenidea*).

***Dolichogenideaphaenna* (Nixon, 1965), new combination**

*Apantelesphaenna* Nixon, 1965.

**Type information.** Holotype female, AEIC (not examined but paratype checked). Country of type locality: Philippines.

**Geographical distribution.**OTL.

**OTL**: Philippines.

**Notes.** In the CNC there is a female paratype and two male specimens identified as this species. They are all from the same locality as the holotype and were all collected on the same date (May 11, 1954). All three CNC specimens have the vannal lobe slightly convex and uniformly setose, which indicates the species belongs to *Dolichogenidea*. The paratype has a blue label indicating its status and another label with the species identification (made by Nixon himself). The two male specimens were identified by Mason and indeed seem to be the same species as the paratype female (although there are slight differences in colour of legs and apical sculpture of T1, but those differences are rather normal between male and female specimens in Microgastrinae). The three specimens differ from the original description of *A.phaenna* as the wings are not that dark; rather, they look very slightly infumate.

***Dolichogenideaphaloniae* (Wilkinson, 1940)**

*Apantelesphaloniae* Wilkinson, 1940.

**Type information.** Holotype female, NHMUK (examined). Country of type locality: United Kingdom.

**Geographical distribution.**PAL.

**PAL**: Azerbaijan, Finland, Georgia, Germany, Hungary, Ireland, Israel, Italy, Lithuania, Madeira Islands, Moldova, Poland, Romania, Russia (KDA, MOS), Slovakia, United Kingdom.

**Notes.** The species distribution in Azerbaijan is based on Belokobylskij et al. (2019).

***Dolichogenideaphaola* (Nixon, 1972)**

*Apantelesphaola* Nixon, 1972.

**Type information.** Holotype female, NHMUK (examined). Country of type locality: United Kingdom.

**Geographical distribution.**OTL, PAL.

**OTL**: China (FJ, HB); **PAL**: Hungary, Russia (PR), Sweden, United Kingdom.

**Notes.** The holotype is missing the metasoma, one antenna and one hind leg.

***Dolichogenideaphthorimaeae* (Muesebeck, 1921)**

*Apantelesphthorimaeae* Muesebeck, 1921.

**Type information.** Holotype female, USNM (not examined but subsequent treatment of the species checked). Country of type locality: USA.

**Geographical distribution.**NEA, NEO.

**NEA**: Canada (ON), USA (FL, LA); **NEO**: Honduras.

**Notes.** This species was transferred from *Apanteles* to *Dolichogenidea* by Mason (1981), also followed by other authors (e.g., Whitfield 1995a). However, You et al. (2002b) referred to it as *Alphomelon* in a phylogenetic analysis of the subfamily. Fernandez-Triana (2010) transferred the species back to *Dolichogenidea*, but he did not state that would represent a revised combination. For the sake of clarity, here we revise the combination of *phthorimaeae* and retain it within *Dolichogenidea*.

***Dolichogenideapiliventris* (Tobias, 1966)**

*Apantelespiliventris* Tobias, 1966.

**Type information.** Holotype female, ZIN (not examined but subsequent treatment of the species checked). Country of type locality: Turkmenistan.

**Geographical distribution.**PAL.

**PAL**: Turkmenistan.

**Notes.** Our species concept is based on Tobias (1986) and Papp (1988).

***Dolichogenideapisenor* (Nixon, 1965), new combination**

*Apantelespisenor* Nixon, 1965.

**Type information.** Holotype female, NHMUK (examined). Country of type locality: Vanuatu.

**Geographical distribution.**AUS, OTL.

**AUS**: Vanuatu; **OTL**: Vietnam.

**Notes.** Based on the hind wing vannal lobe entirely setose, and anteromesoscutum with relatively coarse and deep punctures that do not fuse near the scutoscutellar disc, this species clearly belongs to *Dolichogenidea*.

***Dolichogenideaplatyedrae* (Wilkinson, 1928)**

*Apantelesplatyedrae* Wilkinson, 1928.

**Type information.** Holotype female, NHMUK (examined). Country of type locality: Fiji.

**Geographical distribution.**AUS, OTL.

**AUS**: Fiji; **OTL**: Vietnam.

**Notes.** The holotype is missing its antennae, but otherwise is in good condition.

***Dolichogenideapolaszeki* Walker, 1994**

*Dolichogenideapolaszeki* Walker, 1994.

**Type information.** Holotype female, NHMUK (examined). Country of type locality: Nigeria.

**Geographical distribution.**AFR.

**AFR**: Benin, Cameroon, Ethiopia, Ghana, Kenya, Malawi, Mozambique, Nigeria, Tanzania, Uganda, Zambia.

***Dolichogenideapoliobrevis* Liu & Chen, 2018**

*Dolichogenideapoliobrevis* Liu & Chen, 2018.

**Type information.** Holotype female, ZJUH (not examined but original description checked). Country of type locality: China.

**Geographical distribution.**OTL, PAL.

**OTL**: China (FJ, ZJ); **PAL**: China (HL, LN, XJ, XZ).

***Dolichogenideapolitiventris* (Muesebeck, 1958)**

*Apantelespolitiventris* Muesebeck, 1958.

**Type information.** Holotype female, USNM (examined). Country of type locality: Puerto Rico.

**Geographical distribution.**NEO.

**NEO**: Puerto Rico.

***Dolichogenideapolystinelliphagous* Liu & Chen, 2018**

*Dolichogenideapolystinelliphagous* Liu & Chen, 2018.

**Type information.** Holotype female, ZJUH (not examined but original description checked). Country of type locality: China.

**Geographical distribution.**PAL.

**PAL**: China (JL, LN, SN, SX).

***Dolichogenideapraetor* (Marshall, 1885)**

*Apantelespraetor* Marshall, 1885.

**Type information.** Lectotype male, NHMUK (examined). Country of type locality: United Kingdom.

**Geographical distribution.**PAL.

**PAL**: Armenia, Finland, France, Hungary, Mongolia, Romania, Russia (YAR), Slovakia, Sweden, Switzerland, United Kingdom.

***Dolichogenideapraetoria* (Tobias, 1976)**

*Apantelespraetorius* Tobias, 1976.

**Type information.** Holotype female, ZIN (not examined but subsequent treatment of the species checked). Country of type locality: Russia.

**Geographical distribution.**PAL.

**PAL**: Russia (KDA).

**Notes.**Papp (1978a, 1988; also followed by Yu et al. 2016) considered *Apantelespraetorius* Tobias, 1976 to be a junior synonym of *Apantelespropinquus* Papp, 1975. However, Tobias (1986: 769) stated that both species were clearly different, based on examination of the *praetorius* holotype – whereas Papp (1978: 293) had only examined a paratype of that species. Tobias species concepts were followed by Belokobylskij et al. (2019) and are also accepted by us here, thus we treat both species as distinct in our checklist.

***Dolichogenideaprinceps* (Wilkinson, 1941)**

*Apantelesprinceps* Wilkinson, 1941.

**Type information.** Holotype female, NHMUK (examined). Country of type locality: United Kingdom.

**Geographical distribution.**PAL.

**PAL**: Azerbaijan, Hungary, Italy, Korea, Malta, Mongolia, Romania, Russia (PRI), Serbia, Slovakia, Spain, Tunisia, Turkey, Ukraine, United Kingdom.

**Notes.** The species distribution in Azerbaijan and Mongolia are based on Belokobylskij et al. (2019).

***Dolichogenideaprisca* (Nixon, 1967)**

*Apantelespriscus* Nixon, 1967.

*Dolichogenideaacutituba* Song, Chen & Yang, 2006.

**Type information.** Holotype female, NHMUK (examined). Country of type locality: India.

**Geographical distribution.**OTL, PAL.

**OTL**: China (FJ, GD, GX, GZ, HN, SC, YN, ZJ), India, Malaysia, Sri Lanka, Vietnam; **PAL**: China (HA, SH).

**Notes.** We follow Liu et al. (2019) for the synonymy of *acutituba* under *prisca*, and also for additional distribution of the species in China.

***Dolichogenideaprobata* (Papp, 1973)**

*Apantelesprobatus* Papp, 1973.

**Type information.** Holotype female, HNHM (not examined but original description checked). Country of type locality: Hungary.

**Geographical distribution.**PAL.

**PAL**: Hungary.

**Notes.** The species name must be treated as an adjective and not as a noun (Doug Yanega, pers. comm.) and thus it must match the gender of the genus name.

***Dolichogenideaprodeniae* (Viereck, 1912)**

*Apantelesprodeniae* Viereck, 1912.

**Type information.** Holotype male, USNM (examined). Country of type locality: India.

**Geographical distribution.**OTL.

**OTL**: China (GX), India, Thailand, Vietnam.

**Notes.** The USNM collection contains nine female and five male specimens. One of the male specimens is labeled as the type, whereas the other 13 specimens each have labels stating that they all are paratypes. All of those 14 specimens have the same USNM code (14310), and they seem to come from the same collecting event (as all have the same labels, reared from *Spodopteralittoralis*). Shenefelt’s (1972) catalogue also confirms that there is a male holotype. It is unfortunate that a male was chosen as the species name bearer. In the future it would be advisable to photograph and provide details of the female specimens, which should better characterize the species as compared to the chosen male.

***Dolichogenideapropinqua* (Papp, 1975)**

*Apantelespropinquus* Papp, 1975.

**Type information.** Holotype female, HNHM (not examined but original description checked). Country of type locality: Hungary.

**Geographical distribution.**PAL.

**PAL**: Greece, Hungary, Madeira Islands, Netherlands, Poland, Switzerland.

**Notes.** See notes under *Dolichogenideapraetoria* (Tobias, 1976) for an explanation on why we consider these two species to be different.

***Dolichogenideapterophori* (Muesebeck, 1926)**

*Apantelespterophori* Muesebeck, 1926.

**Type information.** Holotype female, USNM (examined). Country of type locality: USA.

**Geographical distribution.**NEA.

**NEA**: USA (MA).

***Dolichogenideapulchra* (Telenga, 1955)**

*Apantelespulcher* Telenga, 1955.

**Type information.** Type unknown, ZIN (not examined but subsequent treatment of the species checked). Country of type locality: Kazakhstan.

**Geographical distribution.**PAL.

**PAL**: Kazakhstan.

**Notes.** Details on a type specimen or specimens are not provided in the original description (Tobias 1986). However female and male characters are used in two couplets of the key (where the species is described), and thus it is reasonable to assume that the author studied both sexes for the species description. Additionally, in the introductory sections of that book (Tobias 1986: foreword) the intention to detail holotypes for all new species (and to designate lectotypes/paralectotypes from species previously described from the former USSR) is clearly stated, so we can also assume that a holotype for *D.pulchra* was designated, even if not clearly stated in the actual description. It is likely that the holotype is a female specimen, but until the specimens are examined is not possible to confirm.

***Dolichogenideapunctiger* (Wesmael, 1837)**

*Microgasterpunctiger* Wesmael, 1837.

*Apantelesitea* Nixon, 1972.

**Type information.** Holotype female, RBINS (not examined but authoritatively identified specimens examined). Country of type locality: Belgium.

**Geographical distribution.**PAL.

**PAL**: Belgium, Croatia, Czech Republic, Denmark, France, Germany, Hungary, Ireland, Italy, Netherlands, Poland, Russia (BEL, RYA), Slovakia, Sweden, Switzerland, Ukraine, United Kingdom.

**Notes.** We examined the type of *Apantelesitea* Nixon.

***Dolichogenideapunctipila* Liu & Chen, 2019**

*Dolichogenideapunctipila* Liu & Chen, 2019.

**Type information.** Holotype female, ZJUH (not examined but original description checked). Country of type locality: China.

**Geographical distribution.**OTL.

**OTL**: China (FJ, GD, GZ, ZJ).

***Dolichogenideapurdus* (Papp, 1977)**

*Apantelespurdus* Papp, 1977.

**Type information.** Holotype female, HNHM (not examined but subsequent treatment of the species checked). Country of type locality: Hungary.

**Geographical distribution.**OTL, PAL.

**OTL**: China (HB); **PAL**: China (JL, SN), Hungary, Turkey.

**Notes.** Our species concept is based on Papp (1979a, 1988). Yu et al. (2016) treated this species as *Dolichogenideapurda*. However, the original description did not give an etymology and it is neither Latin nor Greek, so following ICZN Article 31.2.3, it must be treated as a noun in apposition and the original spelling *purdus* is retained.

***Dolichogenidearectivena* Liu & Chen, 2019**

*Dolichogenidearectivena* Liu & Chen, 2019.

**Type information.** Holotype female, ZJUH (not examined but original description checked). Country of type locality: China.

**Geographical distribution.**OTL.

**OTL**: China (ZJ).

***Dolichogenideareicharti* (Papp, 1974)**

*Apantelesreicharti* Papp, 1974.

**Type information.** Holotype female, HNHM (not examined but original description checked). Country of type locality: Hungary.

**Geographical distribution.**PAL.

**PAL**: Hungary.

***Dolichogenidearenata* (Kotenko, 1986)**

*Apantelesrenatus* Kotenko, 1986.

**Type information.** Holotype female, SIZK (not examined but original description checked). Country of type locality: Tajikistan.

**Geographical distribution.**PAL.

**PAL**: Mongolia, Tajikistan.

***Dolichogenidearenaulti* (Mason, 1974)**

*Apantelesrenaulti* Mason, 1974.

**Type information.** Holotype female, CNC (examined). Country of type locality: Canada.

**Geographical distribution.**NEA.

**NEA**: Canada (NB, NS, ON, QC).

***Dolichogenidearoepkei* (Shenefelt, 1972), new combination**

*Apantelesroepkei* Shenefelt, 1972.

*Apantelesthoseae* Roepke, 1935 [homonym of *Apantelesthoseae* Wilkinson, 1934].

**Type information.** Syntypes female and male, depository unknown (not examined but subsequent treatment of the species checked). Country of type locality: Indonesia.

**Geographical distribution.**OTL.

**OTL**: Indonesia.

**Notes.** Our species concept is based on Austin (1987), who recognized the species belonged to *Dolichogenidea* (*sensu*Mason 1981) but stopped short of transferring the species. For the sake of clarity, we do that here.

***Dolichogenidearogerblancoi* Fernandez-Triana & Boudreault, 2019**

*Dolichogenidearogerblancoi* Fernandez-Triana & Boudreault, 2019.

**Type information.** Holotype female, CNC (examined). Country of type locality: Costa Rica.

**Geographical distribution.**NEO.

**NEO**: Costa Rica.

***Dolichogenidearufescentis* Chen & Song, 2004**

*Dolichogenidearufescentis* Chen & Song, 2004.

**Type information.** Holotype female, FAFU (not examined but original description checked). Country of type locality: China.

**Geographical distribution.**PAL.

**PAL**: China (JL).

***Dolichogenideasagus* (Kotenko, 1986)**

*Apantelessagus* Kotenko, 1986.

**Type information.** Holotype female, ZIN (not examined but original description checked). Country of type locality: Turkmenistan.

**Geographical distribution.**PAL.

**PAL**: Turkmenistan.

***Dolichogenideasandwico* Liu & Chen, 2018**

*Dolichogenideasandwico* Liu & Chen, 2018.

**Type information.** Holotype female, ZJUH (not examined but original description checked). Country of type locality: China.

**Geographical distribution.**OTL, PAL.

**OTL**: China (FJ, GD, HN, SC, ZJ); **PAL**: China (NX).

***Dolichogenideascabra* (Tobias, 1977), new combination**

*Apantelesscaber* Tobias, 1977.

**Type information.** Holotype female, ZIN (not examined but subsequent treatment of the species checked). Country of type locality: Russia.

**Geographical distribution.**PAL.

**PAL**: Russia (PRI).

**Notes.** Our species concept is based on Papp (1978a). Based on his description of the sculpture and shape of T2–T3, this species is unique among Holarctic species of *Dolichogenidea*. The species name must be treated as an adjective and not as a noun (Doug Yanega, pers. comm.) and thus it must match the gender of the genus name.

***Dolichogenideascabipuncta* Chen & Song, 2004**

*Dolichogenideascabipuncta* Chen & Song, 2004.

**Type information.** Holotype female, FAFU (not examined but original description checked). Country of type locality: China.

**Geographical distribution.**OTL, PAL.

**OTL**: China (FJ, JX, TW); **PAL**: China (JL).

***Dolichogenideaseriphia* (Nixon, 1972)**

*Apantelesseriphia* Nixon, 1972.

**Type information.** Holotype female, ZSM (not examined but original description checked). Country of type locality: Italy.

**Geographical distribution.**PAL.

**PAL**: Greece, Hungary, Iran, Italy, Montenegro, Poland, Russia (S), Slovakia, Spain, Tunisia, Turkey.

**Notes.** The species distribution in Iran and Russia are based on Belokobylskij et al. (2019).

***Dolichogenideasicaria* (Marshall, 1885)**

*Apantelessicarius* Marshall, 1885.

*Apanteleschrysostictus* Marshall, 1889.

*Apantelescrudelis* Papp, 1971.

**Type information.** Lectotype female, NHMUK (examined). Country of type locality: United Kingdom.

**Geographical distribution.**AUS, NEA, OTL, PAL.

**AUS**: New Zealand; **NEA**: Canada (NU), Greenland; **OTL**: China (SC, ZJ); **PAL**: Azerbaijan, Belarus, China (HE, LN, NM, SN, XJ, XZ, ZJ), Czech Republic, Estonia, Finland, France, Georgia, Germany, Greece, Hungary, Iran, Kazakhstan, Kyrgyzstan, Macedonia, Moldova, Mongolia, Montenegro, Morocco, Netherlands, Poland, Romania, Russia (AMU, ZAB, KAM, KHA, MOS, OMS, PRI, YAR), Serbia, Slovakia, Spain, Switzerland, Tunisia, Turkey, Ukraine, United Kingdom.

**Notes.** The species distribution in Iran and Kyrgyzstan are based on Belokobylskij et al. (2019).

***Dolichogenideasimulata* (Papp, 1974)**

*Apantelessimulatus* Papp, 1974.

**Type information.** Holotype female, HNHM (not examined but original description checked). Country of type locality: Korea.

**Geographical distribution.**PAL.

**PAL**: Korea, Russia (PRI).

***Dolichogenideasingularis* Yang & You, 2002**

*Dolichogenideasingularis* Yang & You, 2002.

**Type information.** Holotype female, CFRB (not examined but original description checked). Country of type locality: China.

**Geographical distribution.**OTL, PAL.

**OTL**: China (ZJ); **PAL**: China (HE, SD, SN, TJ).

***Dolichogenideasisenna* (Nixon, 1972)**

*Apantelessisenna* Nixon, 1972.

**Type information.** Holotype female, NHMUK (examined). Country of type locality: United Kingdom.

**Geographical distribution.**PAL.

**PAL**: United Kingdom.

***Dolichogenideasoikai* (Nixon, 1972)**

*Apantelessoikai* Nixon, 1972.

**Type information.** Holotype female, NHMUK (examined). Country of type locality: Italy.

**Geographical distribution.**PAL.

**PAL**: Bulgaria, Greece, Hungary, Italy, Russia (S), Switzerland, Tunisia, Turkey, United Kingdom.

***Dolichogenideasolenobiae* (Walley, 1935)**

*Apantelessolenobiae* Walley, 1935.

**Type information.** Holotype female, CNC (examined). Country of type locality: Canada.

**Geographical distribution.**NEA.

**NEA**: Canada (ON, QC), USA (PA).

***Dolichogenideasonani* (Watanabe, 1932)**

*Apantelessonani* Watanabe, 1932.

**Type information.** Holotype female, EIHU (not examined but subsequent treatment of the species checked). Country of type locality: China.

**Geographical distribution.**OTL.

**OTL**: China (GD, GX, GZ, SC, TW, ZJ).

**Notes.** Our species concept is based on Watanabe (1937a), Song and Chen (2004) and Liu et al. (2019).

***Dolichogenideasophiae* (Papp, 1972)**

*Apantelessophiae* Papp, 1972.

**Type information.** Holotype female, HNHM (not examined but subsequent treatment of the species checked). Country of type locality: Hungary.

**Geographical distribution.**OTL, PAL.

**OTL**: China (ZJ); **PAL**: Armenia, Georgia, Hungary, Moldova, Russia (ZAB), Slovakia, Turkey, Ukraine.

**Notes.** Our species concept is based on Papp (1972, 1978).

***Dolichogenideaspanis* Chen & Song, 2004**

*Dolichogenideaspanis* Chen & Song, 2004.

**Type information.** Holotype female, FAFU (not examined but original description checked). Country of type locality: China.

**Geographical distribution.**OTL.

**OTL**: China (FJ, HB).

***Dolichogenideaspinulicula* Liu & Chen, 2018**

*Dolichogenideaspinulicula* Liu & Chen, 2018.

**Type information.** Holotype female, ZJUH (not examined but original description checked). Country of type locality: China.

**Geographical distribution.**OTL, PAL.

**OTL**: China (GZ, ZJ); **PAL**: China (LN, NX).

***Dolichogenideastantoni* (Ashmead, 1904)**

*Urogasterstantoni* Ashmead, 1904.

*Apantelesfistulae* Wilkinson, 1928.

**Type information.** Holotype female, USNM (not examined but authoritatively identified specimens examined). Country of type locality: Philippines.

**Geographical distribution.**AUS, OTL.

**AUS**: Fiji, Papua New Guinea; **OTL**: China (FJ, GD, GX, GZ, TW, ZJ), India, Malaysia, Philippines, Vietnam.

**Notes.** We examined the type of *A.fistulae* Wilkinson, which is missing the head and metasoma.

***Dolichogenideastatius* (Nixon, 1965), new combination**

*Apantelesstatius* Nixon, 1965.

**Type information.** Holotype female, USNM (examined). Country of type locality: Philippines.

**Geographical distribution.**OTL.

**OTL**: Philippines.

**Notes.** We transfer this species to *Dolichogenidea* based on its uniformly setose vannal lobe and anteromesoscutum with punctures that do not fuse near the scutoscutellar sulcus.

***Dolichogenideastenosis* Song & Chen, 2004**

*Dolichogenideastenosis* Song & Chen, 2004.

**Type information.** Holotype female, FAFU (not examined but original description checked). Country of type locality: China.

**Geographical distribution.**OTL.

**OTL**: China (FJ).

***Dolichogenideastenotelas* (Nixon, 1965), new combination**

*Apantelesstenotelas* Nixon, 1965.

**Type information.** Holotype female, NHMUK (examined). Country of type locality: Vanuatu.

**Geographical distribution.**AUS, OTL.

**AUS**: Vanuatu, **OTL**: Vietnam.

**Notes.** After examining the holotype, we believe the best generic placement for this species is in *Dolichogenidea*, based on the relatively coarse punctures on the anteromesoscutum (which do not fuse near the scutoscutellar sulcus), and the entirely setose vannal lobe in the hind wing (although setae are rather small and a magnification more than 100 × is recommended to see the setation pattern).

***Dolichogenideastictoscutella* Liu & Chen, 2018**

*Dolichogenideastictoscutella* Liu & Chen, 2018.

**Type information.** Holotype female, ZJUH (not examined but original description checked). Country of type locality: China.

**Geographical distribution.**OTL.

**OTL**: China (GD, ZJ).

***Dolichogenideastriata* (van Achterberg & Ng, 2009), new combination**

*Apantelesstriatus* van Achterberg & Ng, 2009.

**Type information.** Holotype female, UKM (not examined but original description checked). Country of type locality: Malaysia.

**Geographical distribution.**OTL.

**OTL**: Malaysia.

**Notes.** A drawing from the original description clearly shows a setose vannal lobe in the hind wing, and thus the species is here transferred to *Dolichogenidea*. The species name must be treated as an adjective and not as a noun (Doug Yanega, pers. comm.) and thus it must match the gender of the genus name.

***Dolichogenideasubemarginata* (Abdinbekova, 1969)**

*Apantelessubemarginatus* Abdinbekova, 1969.

**Type information.** Holotype female, ZIN (not examined but subsequent treatment of the species checked). Country of type locality: Azerbaijan.

**Geographical distribution.**PAL.

**PAL**: Armenia, Azerbaijan, Hungary, Turkey.

**Notes.** Our species concept is based on Papp (1980a) and Tobias (1986).

***Dolichogenideasubgentilis* (Tobias & Long, 1990)**

*Apantelessubgentilis* Tobias & Long, 1990.

**Type information.** Holotype female, ZIN (not examined but original description checked). Country of type locality: Vietnam.

**Geographical distribution.**OTL.

**OTL**: Vietnam.

***Dolichogenideasublabene* (Tobias & Long, 1990)**

*Apantelessublabene* Tobias & Long, 1990.

**Type information.** Holotype female, ZIN (not examined but original description checked). Country of type locality: Vietnam.

**Geographical distribution.**OTL.

**OTL**: Vietnam.

***Dolichogenideasugae* (Watanabe, 1932)**

*Apantelessugae* Watanabe, 1932.

**Type information.** Holotype female, EIHU (examined). Country of type locality: Japan.

**Geographical distribution.**PAL.

**PAL**: Japan, Korea.

**Notes.** The sex of the holotype had never been detailed before (e.g., Shenefelt 1972), and thus it is clarified here. The female holotype is missing the metasoma, head and three legs. But other female and one male specimens (plus a pin with a lepidopteran larva and wasp cocoon mass) are associated with the holotype and help to recognize the species. The entirely setose and slightly convex vannal lobe indicates this species belongs to *Dolichogenidea*. The transfer of the species to *Dolichogenidea* was proposed by Liu et al. (2018) in the abstract but those authors did not provide further details nor explanation in their paper. For the sake of clarity, the species combination is revised here.

***Dolichogenideasyngramma* Ahmad, 2019**

*Dolichogenideasyngramma* Ahmad & Pandey, 2019.

**Type information.** Holotype female, AMUZ (not examined but original description checked). Country of type locality: India.

**Geographical distribution.**OTL.

**OTL**: India.

**Notes.** The authorship of the species names was not made clear in the original description paper, thus here we follow Ahmad (pers. comm.) for that information.

***Dolichogenideaszalayi* (Papp, 1977)**

*Apantelesszalayi* Papp, 1977.

**Type information.** Holotype female, HNHM (not examined but original description checked). Country of type locality: Hungary.

**Geographical distribution.**PAL.

**PAL**: Hungary.

***Dolichogenideaszelenyii* (Papp, 1972)**

*Apantelesszelenyii* Papp, 1972.

**Type information.** Holotype female, HNHM (not examined but original description checked). Country of type locality: Hungary.

**Geographical distribution.**PAL.

**PAL**: Hungary.

***Dolichogenideataiwanensis* (Sonan, 1942)**

*Apantelestaiwanensis* Sonan, 1942.

**Type information.** Syntypes female and male, TARI (not examined but subsequent treatment of the species checked). Country of type locality: China.

**Geographical distribution.**OTL.

**OTL**: China (HI, TW).

**Notes.** Our species concept is based on Chen and Song (2004). Type information from Shenefelt (1972), depository information from Yu et al. (2016).

***Dolichogenideatasmanica* (Cameron, 1912)**

*Apantelestasmanica* Cameron, 1912.

**Type information.** Syntypes female and male, NHMUK (examined). Country of type locality: Australia.

**Geographical distribution.**AUS.

**AUS**: Australia (ACT, QLD, SA, TAS, VIC), New Zealand.

**Notes.**Yu et al. (2016) recorded the type as being female; however, we have examined one female and one male specimens, which both have a type label, and are thus to be considered as syntypes, as correctly stated by Shenefelt (1972: 648).

***Dolichogenideatestacea* Liu & Chen, 2018**

*Dolichogenideatestacea* Liu & Chen, 2018.

**Type information.** Holotype female, ZJUH (not examined but original description checked). Country of type locality: China.

**Geographical distribution.**OTL.

**OTL**: China (ZJ).

***Dolichogenideathujae* (Muesebeck, 1935)**

*Apantelesthujae* Muesebeck, 1935.

**Type information.** Holotype female, USNM (examined). Country of type locality: USA.

**Geographical distribution.**NEA.

**NEA**: Canada (ON, QC).

**Notes.** The holotype is missing the head but it is otherwise in good condition.

***Dolichogenideatischeriae* Viereck, 1912**

*Dolichogenideatischeriae* Viereck, 1912.

**Type information.** Holotype female, USNM (examined). Country of type locality: USA.

**Geographical distribution.**NEA.

**NEA**: Canada (QC), USA (CA, CT, DE, DC, KS, MO, NY, OH, WI).

***Dolichogenideatobiasi* (Balevski, 1980)**

*Apantelestobiasi* Balevski, 1980.

**Type information.** Holotype female, ZIN (examined). Country of type locality: Bulgaria.

**Geographical distribution.**PAL.

**PAL**: Bulgaria, Russia (S), Ukraine.

**Notes.** Our species concept is based on Tobias (1986) and Papp (1988).

***Dolichogenideatrachalus* (Nixon, 1965)**

*Apantelestrachalus* Nixon, 1965.

*Apantelessevocatus* Papp, 1975.

**Type information.** Holotype female, NHMUK (examined). Country of type locality: United Kingdom.

**Geographical distribution.**PAL.

**PAL**: Hungary, Ireland, Syria, United Kingdom.

***Dolichogenideatranscarinata* Liu & Chen, 2019**

*Dolichogenideatranscarinata* Liu & Chen, 2019.

**Type information.** Holotype female, ZJUH (not examined but original description checked). Country of type locality: China.

**Geographical distribution.**OTL.

**OTL**: China (ZJ).

***Dolichogenideatuliemensis* (Tobias & Long, 1990)**

*Apantelestuliemensis* Tobias & Long, 1990.

**Type information.** Holotype female, ZIN (not examined but original description checked). Country of type locality: Vietnam.

**Geographical distribution.**OTL.

**OTL**: Vietnam.

***Dolichogenideaturcmenica* (Tobias, 1967)**

*Apantelesturcmenicus* Tobias, 1967.

**Type information.** Holotype female, ZIN (not examined but subsequent treatment of the species checked). Country of type locality: Turkmenistan.

**Geographical distribution.**PAL.

**PAL**: Mongolia, Turkmenistan, Uzbekistan.

**Notes.** Our species concept is based on Tobias (1986) and Papp (1978a, 1988).

***Dolichogenideaturionellae* (Nixon, 1971)**

*Apantelesturionellae* Nixon, 1971.

**Type information.** Holotype female, NHMUK (examined). Country of type locality: Austria.

**Geographical distribution.**PAL.

**PAL**: Austria, Ukraine.

***Dolichogenideaturkmenus* (Telenga, 1955)**

*Apantelesturkmenus* Telenga, 1955.

**Type information.** Syntypes female and male, ZIN (not examined but subsequent treatment of the species checked). Country of type locality: Turkmenistan.

**Geographical distribution.**PAL.

**PAL**: Armenia, China (XJ), Jordan, Kazakhstan, Turkey, Turkmenistan, Uzbekistan.

**Notes.** Our species concept is based on Telenga (1955), Tobias (1986) and Papp (1978a, 1988). Type information from Shenefelt (1972), depository information from Tobias (1986). Following Article 31.2.3 of the ICZN the name is neither Latin nor Greek and must be treated as a noun in apposition, so the original spelling *turkmenus* is retained (the suffix -us is not definitively adjectival, unlike -icus).

***Dolichogenideaultima* (Kotenko, 1986)**

*Apantelesultimus* Kotenko, 1986.

**Type information.** Holotype female, SIZK (not examined but original description checked). Country of type locality: Ukraine.

**Geographical distribution.**PAL.

**PAL**: China (JL), Russia (S), Ukraine.

***Dolichogenideaultor* (Reinhard, 1880)**

*Apantelesultor* Reinhard, 1880.

*Microgasterlactipennis* Ratzeburg, 1852 [primary homonym of *Microgasterlacteipennis* Curtis, 1830].

**Type information.** Lectotype female, depository unknown (not examined but subsequent treatment of the species checked). Country of type locality: Germany.

**Geographical distribution.**PAL.

**PAL**: Azerbaijan, Czech Republic, Georgia, Germany, Hungary, Italy, Poland, Romania, Russia (IN, DA, KDA, STA), Serbia, Slovakia, Slovenia, Switzerland, Ukraine, United Kingdom.

**Notes.** Our species concept is based on Wilkinson (1945), Nixon (1976), and Papp (1981). The type series was deposited in the Forestry College of Eberswalde (Forstlichen Hochschule Eberswalde). Unfortunately, that collection was mostly destroyed during the Second World War; however, five drawers with Hymenoptera specimens, among them type species of Ratzeburg were spared and are now safe at the Senckenberg Deutsches Entomologisches Institut (SDEI) in Müncheberg, Germany [See a detailed story of that in Schulz et al. (2018: 285-286)]. We do not know whether the lectotype of this species is at present in Müncheberg. The species distribution in Azerbaijan is based on Belokobylskij et al. (2019).

***Dolichogenideaunicarina* Liu & Chen, 2018**

*Dolichogenideaunicarina* Liu & Chen, 2018.

**Type information.** Holotype female, ZJUH (not examined but original description checked). Country of type locality: China.

**Geographical distribution.**OTL, PAL.

**OTL**: China (GZ, ZJ); **PAL**: China (NX, SN, SD).

***Dolichogenideaupoluensis* (Fullaway, 1941)**

*Apantelesupoluensis* Fullaway, 1941.

**Type information.** Holotype male, BPBM (not examined but subsequent treatment of the species checked). Country of type locality: Western Samoa.

**Geographical distribution.**AUS.

**AUS**: Western Samoa.

**Notes.** Our species concept is based on Austin and Dangerfield (1992).

***Dolichogenideauru* Rousse & Gupta, 2013**

*Dolichogenideauru* Rousse & Gupta, 2013.

**Type information.** Holotype female, MNHN (not examined but original description checked). Country of type locality: Réunion.

**Geographical distribution.**AFR.

**AFR**: Réunion.

***Dolichogenideavadosulcus* Liu & Chen, 2019**

*Dolichogenideavadosulcus* Liu & Chen, 2019.

**Type information.** Holotype female, ZJUH (not examined but original description checked). Country of type locality: China.

**Geographical distribution.**OTL.

**OTL**: China (FJ, GD, GX, HI, ZJ).

***Dolichogenideavarifemur* (Abdinbekova, 1969)**

*Apantelesvarifemur* Abdinbekova, 1969.

**Type information.** Holotype female, ZIN (not examined but subsequent treatment of the species checked). Country of type locality: Azerbaijan.

**Geographical distribution.**PAL.

**PAL**: Azerbaijan, Lithuania, Russia (NC).

**Notes.** Our species concept is based on Papp (1978a) and Tobias (1986).

***Dolichogenideavernaliter* (Wilkinson, 1932)**

*Apantelesvernaliter* Wilkinson, 1932.

**Type information.** Holotype female, NHMUK (examined). Country of type locality: Indonesia.

**Geographical distribution.**OTL.

**AUS**: Vanuatu; **OTL**: Indonesia, Vietnam.

***Dolichogenideavictor* (Wilkinson, 1941)**

*Apantelesvictor* Wilkinson, 1941.

**Type information.** Holotype female, NHMUK (examined). Country of type locality: United Kingdom.

**Geographical distribution.**PAL.

**PAL**: United Kingdom.

***Dolichogenideavictoria* Liu & Chen, 2019**

*Dolichogenideavictoria* Liu & Chen, 2019.

**Type information.** Holotype female, ZJUH (not examined but original description checked). Country of type locality: China.

**Geographical distribution.**OTL.

**OTL**: China (FJ, GD, GX, ZJ).

***Dolichogenideavictoriae* (Muesebeck, 1921)**

*Apantelesvictoriae* Muesebeck, 1921.

**Type information.** Holotype female, USNM (examined). Country of type locality: Canada.

**Geographical distribution.**NEA.

**NEA**: Canada (BC).

**Notes.** The only know specimen is the holotype, which is in good condition, except for one fore wing being detached from the body but glued to the same point.

***Dolichogenideavictoriata* (Kotenko, 1986)**

*Apantelesvictoriatus* Kotenko, 1986.

**Type information.** Holotype female, SIZK (not examined but original description checked). Country of type locality: Ukraine.

**Geographical distribution.**PAL.

**PAL**: Mongolia, Russia (S), Ukraine.

***Dolichogenideavillemantae* Rousse, 2013**

*Dolichogenideavillemantae* Rousse, 2013.

**Type information.** Holotype female, MNHN (not examined but original description checked). Country of type locality: Réunion.

**Geographical distribution.**AFR.

**AFR**: Réunion.

***Dolichogenideawangi* Liu & Chen, 2019**

*Dolichogenideawangi* Liu & Chen, 2019.

**Type information.** Holotype female, ZJUH (not examined but original description checked). Country of type locality: China.

**Geographical distribution.**OTL.

**OTL**: China (YN).

***Dolichogenideawittei* (de Saeger, 1944), new combination**

*Apanteleswittei* de Saeger, 1944.

**Type information.** Holotype female, RMCA (not examined but original description checked). Country of type locality: Democratic Republic of Congo.

**Geographical distribution.**AFR.

**AFR**: Democratic Republic of Congo, Rwanda.

**Notes.** Here transferred to *Dolichogenidea* based on the anteromesoscutum punctures not fusing near scutoscutellar sulcus, as well as the shape of the hypopygium and the length and shape of the ovipositor sheaths. Additionally, the original description compares this species as close to *Apantelesbaoris* Wilkinson (placed in *Dolichogenidea* by Chen and Song 2004, among other authors), as well as *Apantelesearterus* Wilkinson and *Apantelespelops* de Saeger (both species similarly placed in *Dolichogenidea* by us, see more details under those two species above).

***Dolichogenideaxenomorph* Fagan-Jeffries & Austin, 2018**

*Dolichogenideaxenomorph* Fagan-Jeffries & Austin, 2018.

**Type information.** Holotype female, ANIC (not examined but original description checked). Country of type locality: Australia.

**Geographical distribution.**AUS.

**AUS**: Australia (NSW, WA).

***Dolichogenideayamini* Sathe & Rokade, 2005**

*Dolichogenideayamini* Sathe & Rokade, 2005.

**Type information.** Holotype female, depository unknown (not examined). Country of type locality: India.

**Geographical distribution.**OTL.

**OTL**: India.

**Notes.** This species name may not be valid as we suspect that no type depository was specified. However, because we could not check original description to confirm that, we retain it as valid species for the time being.

***Dolichogenideayeimycedenoae* Fernandez-Triana & Boudreault, 2019**

*Dolichogenideayeimycedenoae* Fernandez-Triana & Boudreault, 2019.

**Type information.** Holotype female, CNC (examined). Country of type locality: Costa Rica.

**Geographical distribution.**NEO.

**NEO**: Costa Rica.

***Dolichogenideazerafai* Papp, 2015**

*Dolichogenideazerafai* Papp, 2015.

**Type information.** Holotype female, RSME (examined). Country of type locality: Malta.

**Geographical distribution.**PAL.

**PAL**: Malta.

***Dolichogenideazeris* Papp, 2012**

*Dolichogenideazeris* Papp, 2012.

**Type information.** Holotype female, HNHM (not examined but original description checked). Country of type locality: Cape Verde.

**Geographical distribution.**AFR.

**AFR**: Cape Verde.

#### Genus Eripnopelta Xiong, van Achterberg & Chen, 2017

***Eripnopelta*** Xiong, van Achterberg & Chen, 2017: 392. Gender: feminine. Type species: *Eripnopeltaithyvena* Xiong, van Achterberg and Chen 2017, by original designation.

The only known species was recently described from the Oriental region (Xiong et al. 2017). No host data are currently available for this genus. There are no DNA barcodes of *Eripnopelta* in BOLD.

***Eripnopeltaithyvena* Xiong, van Achterberg & Chen, 2017**

*Eripnopeltaithyvena* Xiong, van Achterberg & Chen, 2017.

**Type information.** Holotype female, ZJUH (not examined but original description checked). Country of type locality: China.

**Geographical distribution.**OTL.

**OTL**: China (ZJ, NX, GZ).

#### Genus Exix Mason, 1981

***Exix*** Mason, 1981: 116. Gender: feminine. Type species: *Exixmexicana* Mason, 1981, by original designation.

This is a New World genus, with seven species currently described from the Nearctic and Neotropical regions and revised by Mason (1981). A few species may remain undescribed, but the genus does not seem very speciose. No host data are currently available. There are no DNA-barcode compliant sequences of this genus in BOLD, but four specimens have mini-barcodes of 110–120 bp.

***Exixbahia* Mason, 1981**

*Exixbahia* Mason, 1981.

**Type information.** Holotype female, CNC (examined). Country of type locality: Brazil.

**Geographical distribution.**NEO.

**NEO**: Brazil (BA).

***Exixcolorados* Mason, 1981**

*Exixcolorados* Mason, 1981.

**Type information.** Holotype female, CNC (examined). Country of type locality: Ecuador.

**Geographical distribution.**NEO.

**NEO**: Ecuador.

***Exixcolumbica* Mason, 1981**

*Exixcolumbica* Mason, 1981.

**Type information.** Holotype female, CNC (examined). Country of type locality: Canada.

**Geographical distribution.**NEA.

**NEA**: Canada (BC).

***Exixitatiaia* Souza-Gessner, Bortoni & Penteado-Dias, 2016**

*Exixitatiaia* Souza-Gessner, Bortoni & Penteado-Dias, 2016.

**Type information.** Holotype female, DCBU (not examined but original description checked). Country of type locality: Brazil.

**Geographical distribution.**NEO.

**NEO**: Brazil (MG, RJ).

***Exixmexicana* Mason, 1981**

*Exixmexicana* Mason, 1981.

**Type information.** Holotype female, CNC (examined). Country of type locality: Mexico.

**Geographical distribution.**NEO.

**NEO**: Mexico.

***Exixschunkei* (Nixon, 1965)**

*Protomicroplitisschunkei* Nixon, 1965.

**Type information.** Holotype female, NHMUK (examined). Country of type locality: Peru.

**Geographical distribution.**NEO.

**NEO**: Peru.

***Exixtinalandica* Mason, 1981**

*Exixtinalandica* Mason, 1981.

**Type information.** Holotype female, CNC (examined). Country of type locality: Ecuador.

**Geographical distribution.**NEO.

**NEO**: Ecuador.

#### Genus Exoryza Mason, 1981

***Exoryza*** Mason, 1981: 40. Gender: feminine. Type species: *Apantelesschoenobii* Wilkinson, 1932, by original designation.

Known from 15 described species from all biogeographical regions except for Australasian (the lack of species recorded from Australasian is likely an artefact due to insufficient collecting there). All known species were dealt with in a recent revision (Fernandez-Triana et al. 2016c). The status of *Exoryza* as a valid genus separate from *Dolichogenidea* has been questioned by many authors (e.g., Valerio et al. 2004, Rousse and Gupta 2013, Fernandez-Triana et al. 2014e, 2016c), but until a comprehensive phylogenetic study of Microgastrinae is available we have decided to maintain its present status. Host data include four families of Lepidoptera: Choreutidae, Crambidae, Depressariidae and Gelechiidae; and at least one species is an important biocontrol agent of stem-boring Lepidoptera in rice fields in Asia (Fernandez-Triana et al. 2016c). There are 46 DNA-barcode compliant sequences of *Exoryza* in BOLD representing three different BINs, although one of those BINs actually contains three nominal species (see Fernandez-Triana et al. 2016c for more details).

***Exoryzaasotae* (Watanabe, 1932), new combination**

*Apantelesasotae* Watanabe, 1932.

**Type information.** Holotype female, EIHU (examined). Country of type locality: China.

**Geographical distribution.**OTL, PAL.

**OTL**: China (FJ, GD, TW, ZJ); **PAL**: China (HL, SC), Japan.

**Notes.** After examining the holotype and two paratypes (female and male) we transfer *asotae* to *Exoryza* based on the entirely setose vannal lobe and T2 with strong longitudinal striae. The species distribution in China is based in Liu et al. (2019).

***Exoryzabelippicola* (Liu & You, 1988), new combination**

*Apantelesbelippicola* Liu & You, 1988.

**Type information.** Holotype female, HUNAU (not examined but subsequent treatment of the species checked). Country of type locality: China.

**Geographical distribution.**OTL.

**OTL**: China (SN, ZJ).

**Notes.** Our species concept is based on Chen and Song (2004) and Liu et al. (2019). The species is transferred to *Exoryza* based on T1–T2 strongly rugose (cf. figure 11e in Liu et al. 2019).

***Exoryzahylas* (Wilkinson, 1932), new combination**

*Apanteleshylas* Wilkinson, 1932.

**Type information.** Holotype female, NHMUK (examined). Country of type locality: South Africa.

**Geographical distribution.**AFR.

**AFR**: South Africa.

**Notes.** Transferred to *Exoryza* based on the entirely setose vannal lobe and T2 with strong longitudinal striae.

***Exoryzamariabustosae* Fernandez-Triana, 2016**

*Exoryzamariabustosae* Fernandez-Triana, 2016.

**Type information.** Holotype female, CNC (examined). Country of type locality: Costa Rica.

**Geographical distribution.**NEO.

**NEO**: Costa Rica.

***Exoryzamegagaster* (de Saeger, 1944), new combination**

*Apantelesmegagaster* de Saeger, 1944.

**Type information.** Holotype female, RMCA (not examined but original description checked). Country of type locality: Democratic Republic of Congo.

**Geographical distribution.**AFR.

**AFR**: Democratic Republic of Congo, Rwanda.

**Notes.** Based on the original description, the best generic placement would be in *Exoryza*, based on the shape and sculpture of T2.

***Exoryzaminnesota* Mason, 1981**

*Exoryzaminnesota* Mason, 1981.

**Type information.** Holotype female, USNM (examined). Country of type locality: USA.

**Geographical distribution.**NEA.

**NEA**: Canada (ON), USA (MN).

***Exoryzamonocavus* Valerio & Whitfield, 2004**

*Exoryzamonocavus* Valerio & Whitfield, 2004.

**Type information.** Holotype female, INBio (examined). Country of type locality: Costa Rica.

**Geographical distribution.**NEO.

**NEO**: Costa Rica.

***Exoryzaoryzae* (Walker, 1994), new combination**

*Dolichogenideaoryzae* Walker, 1994.

**Type information.** Holotype female, NHMUK (examined). Country of type locality: Senegal.

**Geographical distribution.**AFR.

**AFR**: Gambia, Ivory Coast, Niger, Senegal.

**Notes.**Fernandez-Triana et al. (2016c) considered this species to belong to *Exoryza*, based on the available evidence (shape and sculpture of T2, as well as host data). However, they stopped short of transferring the species to that genus due to the possibility that future phylogenetic studies would find that *Exoryza* is just a synonym of *Dolichogenidea*. While that possibility still exists, in this paper we are considering *Exoryza* as a valid genus, and for the sake of consistency we are placing here all species which currently fit that genus concept.

***Exoryzareticarina* Song & Chen, 2003**

*Exoryzareticarina* Song & Chen, 2003.

**Type information.** Holotype female, FAFU (not examined but original description checked). Country of type locality: China.

**Geographical distribution.**OTL.

**OTL**: China (YN).

***Exoryzarichardashleyi* Fernandez-Triana, 2016**

*Exoryzarichardashleyi* Fernandez-Triana, 2016.

**Type information.** Holotype female, CNC (examined). Country of type locality: Costa Rica.

**Geographical distribution.**NEO.

**NEO**: Costa Rica.

***Exoryzaritaashleyae* Fernandez-Triana, 2016**

*Exoryzaritaashleyae* Fernandez-Triana, 2016.

**Type information.** Holotype female, CNC (examined). Country of type locality: Costa Rica.

**Geographical distribution.**NEO.

**NEO**: Costa Rica.

***Exoryzarosamatarritae* Fernandez-Triana, 2016**

*Exoryzarosamatarritae* Fernandez-Triana, 2016.

**Type information.** Holotype female, CNC (examined). Country of type locality: Costa Rica.

**Geographical distribution.**NEO.

**NEO**: Costa Rica.

***Exoryzasafranum* Rousse & Gupta, 2013**

*Exoryzasafranum* Rousse & Gupta, 2013.

**Type information.** Holotype female, MNHN (not examined but original description checked). Country of type locality: Réunion.

**Geographical distribution.**AFR.

**AFR**: Réunion.

***Exoryzaschoenobii* (Wilkinson, 1932)**

*Apantelesschoenobii* Wilkinson, 1932.

**Type information.** Holotype female, NHMUK (examined). Country of type locality: India.

**Geographical distribution.**OTL.

**OTL**: Bangladesh, China (FJ, GD, GX, GZ, HI, HB, HN, JS, JX, SN, TW, YN, ZJ), India, Malaysia, Philippines, Sri Lanka, Vietnam.

***Exoryzayeimycedenoae* Fernandez-Triana, 2016**

*Exoryzayeimycedenoae* Fernandez-Triana, 2016.

**Type information.** Holotype female, CNC (examined). Country of type locality: Costa Rica.

**Geographical distribution.**NEO.

**NEO**: Costa Rica.

#### Genus Exulonyx Mason, 1981

***Exulonyx*** Mason, 1981: 33. Gender: masculine. Type species: *Apantelescamma* Nixon, 1965, by original designation.

Only known from a single, very divergent species from the Afrotropical region (Nixon 1965, Mason 1981). No host data are currently available for this genus. There are no DNA barcodes of *Exulonyx* in BOLD.

***Exulonyxcamma* (Nixon, 1965)**

*Apantelescamma* Nixon, 1965.

**Type information.** Holotype female, NHMUK (examined). Country of type locality: South Africa.

**Geographical distribution.**AFR.

**AFR**: South Africa.

#### Genus Fornicia Brullé, 1846

***Fornicia*** Brullé, 1846: 511. Gender: feminine. Type species: *Forniciaclathrata* Brullé, 1846, by monotypy.

*Odontofornicia* Enderlein, 1912: 260. Type species: *Odontoforniciaarata* Enderlein, 1912, by monotypy and original designation.

This is a pantropical genus with 32 species recorded from all regions except for the Holarctic. It` is one of the most distinctive genera of Microgastrinae from a morphological perspective. We have seen in collections many more undescribed species. All known host records are from Limacodidae. There are 67 DNA-barcode compliant sequences of *Fornicia* in BOLD representing 19 different BINs.

***Forniciaachterbergi* Yang & Chen, 2006**

*Forniciaachterbergi* Yang & Chen, 2006.

**Type information.** Holotype female, FAFU (not examined but original description checked). Country of type locality: China.

**Geographical distribution.**OTL.

**OTL**: China (FJ).

***Forniciaafricana* Wilkinson, 1930**

*Forniciaafricana* Wilkinson, 1930.

**Type information.** Holotype female, NHMUK (examined). Country of type locality: Zimbabwe.

**Geographical distribution.**AFR.

**AFR**: Nigeria, Zimbabwe.

***Forniciaafrorum* de Saeger, 1942**

*Forniciaafrorum* de Saeger, 1942.

**Type information.** Holotype female, RMCA (not examined but subsequent treatment of the species checked). Country of type locality: Democratic Republic of Congo.

**Geographical distribution.**AFR.

**AFR**: Democratic Republic of Congo.

**Notes.** Our species concept is based on de Saeger (1948).

***Forniciaalbalata* Ma & Chen, 1994**

*Forniciaalbalata* Ma & Chen, 1994.

**Type information.** Holotype male, ZJUH (not examined but subsequent treatment of the species checked). Country of type locality: China.

**Geographical distribution.**OTL.

**OTL**: China (SN).

**Notes.** Our species concept is based on Chen and Song (2004).

***Forniciaandamanensis* Sharma, 1984**

*Forniciaandamanensis* Sharma, 1984.

**Type information.** Holotype female, depository unknown (not examined but original description checked). Country of type locality: India.

**Geographical distribution.**OTL.

**OTL**: India.

***Forniciaannulipes* Ashmead, 1905**

*Forniciaannulipes* Ashmead, 1905.

**Type information.** Holotype male, USNM (examined). Country of type locality: Philippines.

**Geographical distribution.**OTL.

**OTL**: Philippines.

**Notes.** Specimen with legs and one antenna broken but within the tray that contains the holotype.

***Forniciaarata* (Enderlein, 1912)**

*Odontoforniciaarata* Enderlein, 1912.

**Type information.** Holotype female, depository unknown (not examined but subsequent treatment of the species checked). Country of type locality: China.

**Geographical distribution.**OTL, PAL.

**OTL**: China (GZ, SN, TW, ZJ); **PAL**: China (AH).

**Notes.** Our species concept is based on Watanabe (1937a) and Mason (1981).

***Forniciaballoui* Muesebeck, 1958**

*Forniciaballoui* Muesebeck, 1958.

**Type information.** Holotype female, USNM (not examined but original description checked). Country of type locality: Venezuela.

**Geographical distribution.**NEO.

**NEO**: French Guiana, Suriname, Venezuela.

***Forniciaborneana* (Cushman, 1929)**

*Odontoforniciaborneanus* Cushman, 1929.

**Type information.** Holotype female, USNM (not examined but illustrations of the holotype examined). Country of type locality: Malaysia.

**Geographical distribution.**OTL.

**OTL**: Malaysia.

***Forniciabrachymetacarpa* Luo & You, 2006**

*Forniciabrachymetacarpa* Luo & You, 2006.

**Type information.** Holotype female, HUNAU (not examined). Country of type locality: China.

**Geographical distribution.**OTL.

**OTL**: China (HN).

***Forniciaceylonica* Wilkinson, 1928**

*Forniciaceylonica* Wilkinson, 1928.

**Type information.** Holotype female, NHMUK (examined). Country of type locality: Sri Lanka.

**Geographical distribution.**OTL.

**OTL**: China (TW), India, Indonesia, Philippines, Sri Lanka, Thailand.

***Forniciachalcoscelidis* Wilkinson, 1936**

*Forniciachalcoscelidis* Wilkinson, 1936.

**Type information.** Holotype female, NHMUK (examined). Country of type locality: Malaysia.

**Geographical distribution.**OTL.

**OTL**: Indonesia, Malaysia.

***Forniciaclathrata* Brullé, 1846**

*Forniciaclathrata* Brullé, 1846.

**Type information.** Type and depository unknown (not examined but subsequent treatment of the species checked). Country of type locality: Brazil.

**Geographical distribution.**NEO, OTL.

**NEO**: Brazil (BA, MG), Guyana, Peru, Venezuela; **OTL**: Indonesia.

**Notes.** Our species concept is based on Muesebeck (1958b) and Mason (1981).

***Forniciaghesquierei* de Saeger, 1942**

*Forniciaghesquierei* de Saeger, 1942.

**Type information.** Holotype male, RMCA (not examined but subsequent treatment of the species checked). Country of type locality: Democratic Republic of Congo.

**Geographical distribution.**AFR.

**AFR**: Democratic Republic of Congo.

**Notes.** Our species concept is based on de Saeger (1948).

***Forniciaimbecilla* Chen & He, 1994**

*Forniciaimbecilla* Chen & He, 1994.

**Type information.** Holotype female, ZJUH (not examined but subsequent treatment of the species checked). Country of type locality: China.

**Geographical distribution.**OTL.

**OTL**: China (ZJ).

**Notes.** Our species concept is based on Chen and Song (2004).

***Forniciajarmilae* Mason, 1981**

*Forniciajarmilae* Mason, 1981.

**Type information.** Holotype female, CNC (examined). Country of type locality: Ecuador.

**Geographical distribution.**NEO.

**NEO**: Ecuador.

***Fornicialongiantenna* Luo & You, 2008**

*Fornicialongiantenna* Luo & You, 2008.

**Type information.** Holotype female, GUGC (not examined but original description checked). Country of type locality: China.

**Geographical distribution.**OTL.

**OTL**: China (GZ).

***Forniciamacistigma* Luo & You, 2006**

*Forniciamacistigma* Luo & You, 2006.

**Type information.** Holotype female, HUNAU (not examined). Country of type locality: China.

**Geographical distribution.**OTL.

**OTL**: China (HN).

***Forniciamicrocephala* Granger, 1949**

*Forniciamicrocephala* Granger, 1949.

**Type information.** Holotype female, MNHN (not examined but original description checked). Country of type locality: Madagascar.

**Geographical distribution.**AFR.

**AFR**: Madagascar.

***Forniciaminis* He & Chen, 1994**

*Forniciaminis* He & Chen, 1994.

**Type information.** Holotype male, ZJUH (not examined but subsequent treatment of the species checked). Country of type locality: China.

**Geographical distribution.**OTL.

**OTL**: China (ZJ).

**Notes.** Our species concept is based on Chen and Song (2004).

***Forniciamoronis* (Cushman, 1929)**

*Odontoforniciamoronis* Cushman, 1929.

**Type information.** Holotype female, USNM (not examined but subsequent treatment of the species checked). Country of type locality: Philippines.

**Geographical distribution.**OTL.

**OTL**: Philippines.

**Notes.** Our species concept is based on Papp (1980c).

***Forniciamuluensis* Austin, 1987**

*Forniciamuluensis* Austin, 1987.

**Type information.** Holotype female, NHMUK (examined). Country of type locality: Malaysia.

**Geographical distribution.**OTL.

**OTL**: Brunei, Malaysia.

***Forniciaobscuripennis* Fahringer, 1934**

*Forniciaobscuripennis* Fahringer, 1934.

**Type information.** Holotype male, NHRS (not examined but subsequent treatment of the species checked). Country of type locality: China.

**Geographical distribution.**OTL.

**OTL**: China (FJ, GX, GZ, HN, JS, SN, TW, ZJ).

**Notes.** Our species concept is based on Papp (1980c).

***Forniciapenang* (Cushman, 1929)**

*Odontoforniciapenang* Cushman, 1929.

**Type information.** Holotype female, USNM (not examined but subsequent treatment of the species checked). Country of type locality: Malaysia.

**Geographical distribution.**OTL.

**OTL**: China (TW), Indonesia, Malaysia.

**Notes.** Our species concept is based on Papp (1980c). Type information from Shenefelt (1973).

***Forniciapilosa* Cushman, 1931**

*Forniciapilosa* Cushman, 1931.

**Type information.** Holotype female, USNM (not examined but subsequent treatment of the species checked). Country of type locality: Costa Rica.

**Geographical distribution.**NEO.

**NEO**: Brazil (PA), Costa Rica.

**Notes.** Our species concept is based on Papp (1980c).

***Forniciaprominentis* Chen & He, 1994**

*Forniciaprominentis* Chen & He, 1994.

**Type information.** Holotype female, ZJUH (not examined but subsequent treatment of the species checked). Country of type locality: China.

**Geographical distribution.**OTL.

**OTL**: China (GX).

**Notes.** Our species concept is based on Chen and Song (2004).

***Forniciarixata* Papp, 1980**

*Forniciarixata* Papp, 1980.

**Type information.** Holotype male, HNHM (not examined but original description checked). Country of type locality: China.

**Geographical distribution.**OTL.

**OTL**: China (TW).

***Forniciaseyrigi* Granger, 1949**

*Forniciaseyrigi* Granger, 1949.

**Type information.** Holotype female, MNHN (not examined but original description checked). Country of type locality: Madagascar.

**Geographical distribution.**AFR.

**AFR**: Madagascar.

***Forniciasurinamensis* Muesebeck, 1958**

*Forniciasurinamensis* Muesebeck, 1958.

**Type information.** Holotype female, USNM (examined). Country of type locality: Suriname.

**Geographical distribution.**NEO.

**NEO**: Suriname.

***Forniciatagalog* (Cushman, 1929)**

*Odontoforniciatagalog* Cushman, 1929.

**Type information.** Holotype female, USNM (not examined but subsequent treatment of the species checked). Country of type locality: Philippines.

**Geographical distribution.**OTL.

**OTL**: Philippines.

**Notes.** Our species concept is based on Papp (1980c).

***Forniciatergiversata* Papp, 1980**

*Forniciatergiversata* Papp, 1980.

**Type information.** Holotype female, HNHM (not examined but original description checked). Country of type locality: China.

**Geographical distribution.**OTL.

**OTL**: China (TW).

***Forniciathoseae* Wilkinson, 1930**

*Forniciathoseae* Wilkinson, 1930.

**Type information.** Holotype female, NHMUK (examined). Country of type locality: Indonesia.

**Geographical distribution.**OTL.

**OTL**: Indonesia.

#### Genus Gilbertnixonius Fernandez-Triana, 2018

***Gilbertnixonius*** Fernandez-Triana, 2018: 56. Gender: neuter. Type species: *Gilbertnixoniusbiem*Fernandez-Triana and Boudreault 2018, by original designation.

The only known species was recently described from the Oriental region (Fernandez-Triana and Boudreault 2018). No host data are currently available for this genus. There is one DNA-barcode compliant sequence of *Gilbertnixonius* in BOLD.

***Gilbertnixoniusbiem* Fernandez-Triana & Boudreault, 2018**

*Gilbertnixoniusbiem* Fernandez-Triana & Boudreault, 2018.

**Type information.** Holotype female, QSBG (examined). Country of type locality: Thailand.

**Geographical distribution.**OTL.

**OTL**: Thailand.

#### Genus Glyptapanteles Ashmead, 1904

***Glyptapanteles*** Ashmead, 1904: 147. Gender: masculine. Type species: (*Glyptapantelesmanilae* Ashmead, 1904) = *Apantelesashmeadi* Wilkinson, 1928, by monotypy.

Viereck (1914: 62) correctly noted that Ashmead (1900a: 131) was intending to refer to this genus in his 1900 key in the second half of couplet 10, where *Protapanteles* is separated from another genus, the name of which is (accidentally ?) omitted, but it is clear that it would have been *Glyptapanteles*. Thus, technically Ashmead’s (1900a) would be the first intention to mention the name *Glyptapanteles* in a published paper, but because the actual name never appeared there due to an omission, the first official reference to the genus must be considered Ashmead (1904b). In any case the 1900 paper did not designate any type species, so the 1904 paper is the one that matters for that purpose (as Viereck also correctly noted). *Glyptapanteles* is a cosmopolitan genus, with 307 described species known from all biogeographical regions. Many European species were revised by Nixon and Papp in several papers from the 1970s and 1980s, following earlier work by Wilkinson (1945); and a recent paper dealt with 136 Neotropical species (Arias-Penna et al. 2019), which represents almost half of all described species in the genus. Overall, the taxonomic coverage of the world species is far from complete; we have seen hundreds of undescribed species in collections, mostly from tropical areas, and it is likely that the actual richness will reach several thousand species. The concept of *Glyptapanteles* and its separation from *Protapanteles* has been controversial (e.g., Mason 1981, van Achterberg 2003, Broad et al. 2016), but we consider it as a valid genus, although future studies on Microgastrinae phylogeny may split the genus into several. More than 25 families of Lepidoptera have been recorded as hosts for *Glyptapanteles*, but many records are likely to be incorrect and/or need further verification. There are almost 5,000 DNA-barcode compliant sequences of this genus in BOLD, representing 504 BINs.

***Glyptapantelesacasta* (Nixon, 1973)**

*Apantelesacasta* Nixon, 1973.

**Type information.** Holotype female, NHMUK (examined). Country of type locality: United Kingdom.

**Geographical distribution.**PAL.

**PAL**: Bulgaria, Finland, Germany, Greece, Hungary, Poland, Russia (ALT, ZAB), Slovakia, Switzerland, Turkey, United Kingdom.

***Glyptapantelesacherontiae* (Cameron, 1907)**

*Apantelesacherontiae* Cameron, 1907.

*Apantelesacherontiae* Muesebeck, 1927 [homonym of *Apantelesacherontiae* Cameron, 1907].

**Type information.** Syntypes female, NHMUK (examined). Country of type locality: Sri Lanka.

**Geographical distribution.**OTL.

**OTL**: China (FJ, HN), India, Sri Lanka.

***Glyptapantelesacraeae* (Wilkinson, 1932), lectotype designation**

*Apantelesacraeae* Wilkinson, 1932.

**Type information.** Lectotype female, NHMUK (examined). Country of type locality: Uganda.

**Geographical distribution.**AFR.

**AFR**: South Africa, Uganda.

**Notes.** The species was described from female and male specimens. We have examined a female specimen with a type label and code 3c.1027 in the NHMUK and are designating it here as the lectotype.

***Glyptapantelesafiamaluanus* (Fullaway, 1941)**

*Apantelesafiamaluana* Fullaway, 1941.

**Type information.** Holotype female, BPBM (not examined but subsequent treatment of the species checked). Country of type locality: Western Samoa.

**Geographical distribution.**AUS.

**AUS**: Western Samoa.

**Notes.** Our species concept is based on Austin and Dangerfield (1992).

***Glyptapantelesafricanus* (Cameron, 1911)**

*Apantelesafricanus* Cameron, 1911.

*Apantelesbeneficus* Viereck, 1911.

*Apantelescameroni* Brues, 1924.

**Type information.** Holotype female, TMSA (not examined but subsequent treatment of the species checked). Country of type locality: South Africa.

**Geographical distribution.**AFR, OTL.

**AFR**: Ghana, Kenya, Malawi, Mali, Mozambique, Nigeria, South Africa, Uganda, Zimbabwe; **OTL**: India, Pakistan.

**Notes.** Our species concept is based on Wilkinson (1932a), van Achterberg and Polaszek (1996), and van Achterberg and Walker (1998). We examined the type, a female specimen, of *Apantelesbeneficus* (Viereck, 1911), currently a synonym of *G.africanus*.

***Glyptapantelesaggestus* (Granger, 1949), new combination**

*Glytapantelesaggestus* Granger, 1949.

**Type information.** Syntypes female, MNHN (not examined but original description checked). Country of type locality: Madagascar.

**Geographical distribution.**AFR.

**AFR**: Madagascar.

**Notes.** This species clearly is not an *Apanteles*. Based on the original description it is provisionally transferred to *Glyptapanteles* until examination of the syntype series allows a more definitive identification.

***Glyptapantelesagrotivorus* Whitfield, 2002**

*Glyptapantelesagrotivorus* Whitfield, 2002.

**Type information.** Holotype female, USNM (examined). Country of type locality: Ecuador.

**Geographical distribution.**NEO.

**NEO**: Ecuador.

**Notes.** The holotype is dirty and not in good condition.

***Glyptapantelesagynus* (de Saeger, 1944), new combination**

*Apantelesagynus* de Saeger, 1944.

**Type information.** Holotype male, RMCA (not examined but original description checked). Country of type locality: Democratic Republic of Congo.

**Geographical distribution.**AFR.

**AFR**: Democratic Republic of Congo.

**Notes.** Based on the original description, which is the only reference available for this species, the best generic placement at present would be in *Glyptapanteles*. However, the only known specimen is a male and the description is not clear enough to rule out the genus *Distatrix*. Examination of the specimen will be needed to conclude.

***Glyptapantelesaithos* (Sharma, 1973), new combination**

*Apantelesaithos* Sharma, 1973.

**Type information.** Holotype female, IFRI (not examined but original description checked). Country of type locality: India.

**Geographical distribution.**OTL.

**OTL**: India.

**Notes.** This species is clearly not an *Apanteles*. The original description does not provide enough information to determine the generic identity in a conclusive way but *Glyptapanteles* seems to be the best match (although *Distatrix* might be another possibility). Examination of the type series will be needed to conclude on its generic status.

***Glyptapantelesalejandrovalerioi* Arias-Penna, 2019**

*Glyptapantelesalejandrovalerioi* Arias-Penna, 2019.

**Type information.** Holotype female, CNC (examined). Country of type locality: Costa Rica.

**Geographical distribution.**NEO.

**NEO**: Costa Rica.

***Glyptapantelesaletta* (Nixon, 1973)**

*Apantelesaletta* Nixon, 1973.

**Type information.** Holotype female, MZH (not examined but original description checked). Country of type locality: Finland.

**Geographical distribution.**PAL.

**PAL**: Belarus, Finland, Hungary, Korea, Slovakia, Switzerland.

***Glyptapantelesalexborisenkoi* Arias-Penna, 2019**

*Glyptapantelesalexborisenkoi* Arias-Penna, 2019.

**Type information.** Holotype female, CNC (examined). Country of type locality: Costa Rica.

**Geographical distribution.**NEO.

**NEO**: Costa Rica.

***Glyptapantelesalexwildi* Arias-Penna, 2019**

*Glyptapantelesalexwildi* Arias-Penna, 2019.

**Type information.** Holotype male, QCAZ (examined). Country of type locality: Ecuador.

**Geographical distribution.**NEO.

**NEO**: Ecuador.

***Glyptapantelesaliphera* (Nixon, 1973)**

*Apantelesaliphera* Nixon, 1973.

*Apantelessublateralis* Tobias, 1976.

**Type information.** Holotype female, NHMUK (examined). Country of type locality: United Kingdom.

**Geographical distribution.**PAL.

**PAL**: Armenia, Azerbaijan, Finland, France, Georgia, Germany, Greece, Hungary, Iran, Israel, Netherlands, Poland, Romania, Russia (KDA), Slovakia, Sweden, Switzerland, United Kingdom.

***Glyptapantelesalticola* (Ashmead, 1902)**

*Protapantelesalticola* Ashmead, 1902.

**Type information.** Holotype male, USNM (examined). Country of type locality: USA.

**Geographical distribution.**NEA.

**NEA**: Canada (BC, NB), USA (AK, CA, CO, ID, NH, OR, UT).

**Notes.** The holotype is a male, not a female as stated by Shenefelt (1972) and Yu et al. (2012). The metasoma is separate from the head and mesosoma, but it is glued to the same point; only the right wings are present. Muesebeck (1921) mentioned a type series, which we have not seen, and also provided a brief description of the species as part of his key to ‘*Apantelessensu lato*’. According to that key, Muesebeck states that the species has metafemur ‘dark reddish testaceous, usually edged with blackish’ and also ‘stigma and veins of forewing dark brown’ (Muesebeck 1921: 493). However, the holotype has yellow metafemur and the pterostigma is very pale brown. Other than that, the holotype resembles many ‘*Glyptapanteles*’ from the northern Nearctic in colour, propodeum sculpture (which has a faint median carina on posterior 0.5), and shape and sculpture of T1–T3.

***Glyptapantelesalvarowillei* Arias-Penna, 2019**

*Glyptapantelesalvarowillei* Arias-Penna, 2019.

**Type information.** Holotype female, CNC (examined). Country of type locality: Costa Rica.

**Geographical distribution.**NEO.

**NEO**: Costa Rica.

***Glyptapantelesamenophis* (de Saeger, 1944), new combination**

*Apantelesamenophis* de Saeger, 1944.

**Type information.** Holotype female, RMCA (not examined but original description checked). Country of type locality: Democratic Republic of Congo.

**Geographical distribution.**AFR.

**AFR**: Democratic Republic of Congo.

**Notes.** From the original description it is clear that this species does not belong in *Apanteles* (due to the short ovipositor and shape of the hypopygium). When describing it, de Saeger (1944) stated that it would come close to *Apantelesparallelus* (Lyle, 1971), which is currently placed within *Protapanteles*. We consider that *amenophis* is better placed within *Glyptapanteles* for the time being, but future examination of the specimens may change that.

***Glyptapantelesandrewdebeveci* Arias-Penna, 2019**

*Glyptapantelesandrewdebeveci* Arias-Penna, 2019.

**Type information.** Holotype female, QCAZ (examined). Country of type locality: Ecuador.

**Geographical distribution.**NEO.

**NEO**: Ecuador.

***Glyptapantelesandybennetti* Arias-Penna, 2019**

*Glyptapantelesandybennetti* Arias-Penna, 2019.

**Type information.** Holotype female, CNC (examined). Country of type locality: Costa Rica.

**Geographical distribution.**NEO.

**NEO**: Costa Rica.

***Glyptapantelesandydeansi* Arias-Penna, 2019**

*Glyptapantelesandydeansi* Arias-Penna, 2019.

**Type information.** Holotype female, CNC (examined). Country of type locality: Costa Rica.

**Geographical distribution.**NEO.

**NEO**: Costa Rica.

***Glyptapantelesandysuarezi* Arias-Penna, 2019**

*Glyptapantelesandysuarezi* Arias-Penna, 2019.

**Type information.** Holotype female, QCAZ (examined). Country of type locality: Ecuador.

**Geographical distribution.**NEO.

**NEO**: Ecuador.

***Glyptapantelesandywarreni* Arias-Penna, 2019**

*Glyptapantelesandywarreni* Arias-Penna, 2019.

**Type information.** Holotype female, QCAZ (examined). Country of type locality: Ecuador.

**Geographical distribution.**NEO.

**NEO**: Ecuador.

***Glyptapantelesankitaguptae* Arias-Penna, 2019**

*Glyptapantelesankitaguptae* Arias-Penna, 2019.

**Type information.** Holotype male, QCAZ (examined). Country of type locality: Ecuador.

**Geographical distribution.**NEO.

**NEO**: Ecuador.

***Glyptapantelesannettewalkerae* Arias-Penna, 2019**

*Glyptapantelesannettewalkerae* Arias-Penna, 2019.

**Type information.** Holotype female, CNC (examined). Country of type locality: Costa Rica.

**Geographical distribution.**NEO.

**NEO**: Costa Rica.

***Glyptapantelesantarctiae* (Blanchard, 1935), new combination**

*Apantelesantarctiae* Blanchard, 1935.

Apantelesantarctiaevar.fusca Blanchard, 1935.

**Type information.** Syntypes male, MACN (not examined but original description checked). Country of type locality: Argentina.

**Geographical distribution.**NEO.

**NEO**: Argentina.

**Notes.** The type material belongs to the Blanchard collection, deposited in MACN. The descriptions and illustrations (scutellar disc, propodeum, T1–T2, part of fore wing, tip of antenna) provided in the original description and a following paper (Blanchard 1935, 1936), strongly suggest that this species belongs to *Glyptapanteles*. The species has the propodeum mostly smooth and without carinae (although the illustration for a male specimen shows a weakly defined median, longitudinal carina), T1 anterior half is parallel-sided while posterior half slightly narrows towards posterior margin, T2 is trapezoidal, and ovipositor sheaths are barely protruding.

***Glyptapantelesantinoe* (Nixon, 1973)**

*Apantelesantinoe* Nixon, 1973.

**Type information.** Holotype female, NHMW (not examined but original description checked). Country of type locality: Austria.

**Geographical distribution.**PAL.

**PAL**: Austria, Germany, Hungary, Turkey.

***Glyptapantelesantsirabensis* (Granger, 1949)**

*Apantelesantsirabensis* Granger, 1949.

**Type information.** Holotype female, MNHN (not examined but subsequent treatment of the species checked). Country of type locality: Madagascar.

**Geographical distribution.**AFR.

**AFR**: Madagascar, Réunion.

**Notes.** Our species concept is based on Rousse and Gupta (2013).

***Glyptapantelesanubis* (de Saeger, 1944), new combination**

*Apantelesanubis* de Saeger, 1944.

**Type information.** Holotype female, RMCA (not examined but original description checked). Country of type locality: Democratic Republic of Congo.

**Geographical distribution.**AFR.

**AFR**: Democratic Republic of Congo.

**Notes.** From the original description it is clear that this species does not belong to *Apanteles* (due to the short ovipositor and propodeum with median carina). When describing it, de Saeger (1944) stated that, in the Wilkinson key to African species, *anubis* would come close to *Apantelespallidocinctus* (Gahan, 1918), which is currently placed within the genus *Distarix*. However, *anubis* should not be placed in that genus, due to having a rugose and carinated propodeum. Examination of the holotype will eventually be needed to conclude but, based on all information available in the original description (the only published source of information for this species), the best generic placement at present would be within *Glyptapanteles*.

***Glyptapantelesarcuatus* (Telenga, 1955)**

*Apantelesarcuatus* Telenga, 1955.

**Type information.** Syntypes female and male, depository unknown (not examined but subsequent treatment of the species checked). Country of type locality: Russia.

**Geographical distribution.**PAL.

**PAL**: Germany, Russia (PRI).

**Notes.** Our species concept is based on Telenga (1955), Papp (1983a) and Kotenko (2007a).

***Glyptapantelesarginae* (Bhatnagar, 1950), new combination**

*Apantelesarginae* Bhatnagar, 1950.

**Type information.** Holotype female, INPC (not examined but original description checked). Country of type locality: India.

**Geographical distribution.**OTL.

**OTL**: India.

**Notes.** Based on the original description this species is clearly not an *Apanteles*. The description of the propodeum with a faint median longitudinal carina, short ovipositor sheaths, T1 smooth and narrowing towards apex, and T2 smooth and subtriangular shaped, all suggest that the best generic placement for this species at present would be within *Glyptapanteles*. However, examination of the holotype and paratypes may change that in the future. The year of publication of the Bhatnagar paper was until recently commonly cited as 1948 and/or 1950 (e.g., Chen and Song 2004, Yu et al. 2016), probably following Shenefelt (1972) who referred to this paper as “Bhatnagar (1948) 1950”. While the intended year for Volume X, Parts I & II of the Indian Journal of Entomology was 1948, the actual dates of publication were June 1950 (Part I) and October 1950 (Part II), as clearly shown on the cover page of the Volume, which we have checked. Because the dates of publication are the ones to be considered, and for the sake of clarity, we hereby revise the species year of description to 1950.

***Glyptapantelesargus* (de Saeger, 1944), new combination**

*Apantelesargus* de Saeger, 1944.

**Type information.** Holotype male, RMCA (not examined but original description checked). Country of type locality: Rwanda.

**Geographical distribution.**AFR.

**AFR**: Democratic Republic of Congo, Rwanda.

**Notes.** The original description states this species is very close to *A.intricatus* (de Saeger, 1944), which in turn was described as very close to several species currently placed within *Glyptapanteles*. The drawings of the original description of *A.intricatus* indeed confirm it belongs to *Glyptapanteles*, and thus *argus* is also placed in that genus.

***Glyptapantelesaristolochiae* (Wilkinson, 1928)**

*Apantelesaristolochiae* Wilkinson, 1928.

**Type information.** Holotype female, NHMUK (examined). Country of type locality: Sri Lanka.

**Geographical distribution.**OTL.

**OTL**: India, Sri Lanka.

***Glyptapantelesartonae* (Rohwer, 1926)**

*Apantelesartonae* Rohwer, 1926.

**Type information.** Holotype female, USNM (examined). Country of type locality: Malaysia.

**Geographical distribution.**AUS, OTL.

**AUS**: Fiji; **OTL**: Indonesia, Malaysia.

**Notes.** See Austin and Dangerfield (1992) for details questioning the distibution of this species in Fiji. Yu et al. (2016) have the NHMUK as the type depository, but we have found and examined the holotype in Washington (USNM).

***Glyptapantelesashmeadi* (Wilkinson, 1928)**

*Apantelesashmeadi* Wilkinson, 1928.

*Glyptapantelesmanilae* Ashmead, 1904 [secondary homonym of *Apantelesmanilae* Ashmead, 1904].

**Type information.** Holotype female, USNM (not examined but original description checked). Country of type locality: Philippines.

**Geographical distribution.**OTL.

**OTL**: Philippines.

***Glyptapantelesatylana* (Nixon, 1965), new combination**

*Apantelesatylana* Nixon, 1965.

**Type information.** Holotype female, NHMUK (examined). Country of type locality: Indonesia.

**Geographical distribution.**OTL.

**OTL**: Indonesia.

**Notes.** See comments on *G.siderion* (Nixon, 1965) below for more details on the rationale to provisionally place these two species in *Glyptapanteles*.

***Glyptapantelesaucklandensis* (Cameron, 1909)**

*Apantelesaucklandensis* Cameron, 1909.

**Type information.** Holotype male, NHMUK (examined). Country of type locality: New Zealand.

**Geographical distribution.**AUS.

**AUS**: New Zealand.

***Glyptapantelesbadgleyi* (Wilkinson, 1928), new combination**

*Apantelesbadgleyi* Wilkinson, 1928.

**Type information.** Holotype female, NHMUK (examined). Country of type locality: India.

**Geographical distribution.**OTL.

**OTL**: India.

**Notes.** This species is placed in *Glyptapanteles* based on very short ovipositor sheaths, inflexible hypopygium, T1 narrowing towards posterior margin, and T2 subtriangular (trapezoidal).

***Glyptapantelesbarneyburksi* Arias-Penna, 2019**

*Glyptapantelesbarneyburksi* Arias-Penna, 2019.

**Type information.** Holotype female, CNC (examined). Country of type locality: Costa Rica.

**Geographical distribution.**NEO.

**NEO**: Costa Rica.

***Glyptapantelesbataviensis* (Rohwer, 1919), new combination**

*Apantelesbataviensis* Rohwer, 1919.

**Type information.** Holotype female, USNM (examined). Country of type locality: Indonesia.

**Geographical distribution.**OTL.

**OTL**: India, Indonesia, Vietnam.

**Notes.** The species was transferred from *Apanteles* to *Cotesia* by Long et al. (2004). However, after examining the holotype, we find it clearly belongs to *Glyptapanteles* as it has an entirely smooth propodeum and T1 is strongly narrowing towards posterior margin.

***Glyptapantelesbetogarciai* Arias-Penna, 2019**

*Glyptapantelesbetogarciai* Arias-Penna, 2019.

**Type information.** Holotype female, QCAZ (examined). Country of type locality: Ecuador.

**Geographical distribution.**NEO.

**NEO**: Ecuador.

***Glyptapantelesbidentatus* (Sharma, 1972)**

*Apantelesbidentatus* Sharma, 1972.

**Type information.** Holotype male, IFRI (not examined but original description checked). Country of type locality: India.

**Geographical distribution.**OTL.

**OTL**: India.

***Glyptapantelesbillbrowni* Arias-Penna, 2019**

*Glyptapantelesbillbrowni* Arias-Penna, 2019.

**Type information.** Holotype female, CNC (examined). Country of type locality: Costa Rica.

**Geographical distribution.**NEO.

**NEO**: Costa Rica.

***Glyptapantelesbimus* Papp, 1990**

*Glyptapantelesbimus* Papp, 1990.

**Type information.** Holotype female, HNHM (not examined but original description checked). Country of type locality: Korea.

**Geographical distribution.**PAL.

**PAL**: Korea.

**Notes.** Our species concept is based on Papp (1990b) and Kotenko (2007a).

***Glyptapantelesbistonis* (Watanabe, 1934), new combination**

*Apantelesbistonis* Watanabe, 1934.

**Type information.** Holotype male, EIHU (examined). Country of type locality: Japan.

**Geographical distribution.**PAL.

**PAL**: Japan.

**Notes.** We examined the male holotype and another pin which carries a cocoon mass. This species is clearly *Glyptapanteles* (as Papp had recognized in a label he wrote in 1992, although he never published that new combination).

***Glyptapantelesbobhanneri* Arias-Penna, 2019**

*Glyptapantelesbobhanneri* Arias-Penna, 2019.

**Type information.** Holotype female, CNC (examined). Country of type locality: Costa Rica.

**Geographical distribution.**NEO.

**NEO**: Costa Rica.

***Glyptapantelesbobkulai* Arias-Penna, 2019**

*Glyptapantelesbobkulai* Arias-Penna, 2019.

**Type information.** Holotype female, CNC (examined). Country of type locality: Costa Rica.

**Geographical distribution.**NEO.

**NEO**: Costa Rica.

***Glyptapantelesbobwhartoni* Arias-Penna, 2019**

*Glyptapantelesbobwhartoni* Arias-Penna, 2019.

**Type information.** Holotype female, CNC (examined). Country of type locality: Costa Rica.

**Geographical distribution.**NEO.

**NEO**: Costa Rica.

***Glyptapantelesboharti* Arias-Penna, 2019**

*Glyptapantelesboharti* Arias-Penna, 2019.

**Type information.** Holotype female, CNC (examined). Country of type locality: Costa Rica.

**Geographical distribution.**NEO.

**NEO**: Costa Rica.

***Glyptapantelesborocerae* (Granger, 1949), new combination**

*Apantelesborocerae* Granger, 1949.

**Type information.** Syntypes female and male, MNHN (not examined but original description checked). Country of type locality: Madagascar.

**Geographical distribution.**AFR.

**AFR**: Madagascar.

**Notes.** This species is not *Apanteles*. Based on the original description as well as host information, the species is provisionally transferred to *Glyptapanteles* until examination of the syntype series allows a more definitive identification.

***Glyptapantelesbourquini* (Blanchard, 1936)**

*Apantelesbourquini* Blanchard, 1936.

*Apanteleselegans* Blanchard, 1936.

**Type information.** Holotype female, MACN (not examined but subsequent treatment of the species checked). Country of type locality: Argentina.

**Geographical distribution.**NEO.

**NEO**: Argentina, Brazil (RS) Chile, Ecuador, Peru, Uruguay.

**Notes.** The type belongs to the Blanchard collection, which we assume is deposited in the MACN. Our species concept is based on Whitfield et al. (2002a). The record from Brazil is based on Shimbori et al. (2019).

***Glyptapantelesbreviscuta* Song & Chen, 2004**

*Glyptapantelesbreviscuta* Song & Chen, 2004.

**Type information.** Holotype female, FAFU (not examined but original description checked). Country of type locality: China.

**Geographical distribution.**OTL.

**OTL**: China (FJ).

***Glyptapantelesbrianestjaquesae* Arias-Penna, 2019**

*Glyptapantelesbrianestjaquesae* Arias-Penna, 2019.

**Type information.** Holotype female, CNC (examined). Country of type locality: Costa Rica.

**Geographical distribution.**NEO.

**NEO**: Costa Rica.

***Glyptapantelescaberatae* (Muesebeck, 1956)**

*Apantelescaberatae* Muesebeck, 1956.

**Type information.** Holotype female, USNM (not examined but subsequent treatment of the species checked). Country of type locality: USA.

**Geographical distribution.**NEA.

**NEA**: USA (CA).

**Notes.** Our species concept is based on Whitfield (1995a).

***Glyptapantelescacao* (Wilkinson, 1934), new combination**

*Apantelescacao* Wilkinson, 1934.

**Type information.** Holotype female, NHMUK (examined). Country of type locality: Sri Lanka.

**Geographical distribution.**OTL.

**OTL**: Sri Lanka.

**Notes.** This species is placed in *Glyptapanteles* based on the short ovipositor sheaths, inflexible hypopygium, T1 narrowing towards posterior margin, and T2 subtriangular (trapezoidal).

***Glyptapantelescadei* (Risbec, 1951), new combination**

*Apantelescadei* Risbec, 1951.

**Type information.** Holotype male, depository unknown (not examined but original description checked). Country of type locality: Senegal.

**Geographical distribution.**AFR.

**AFR**: Senegal.

**Notes.** The original description (and the included drawings of the propodeum, T1 and T2) suggests this species does not belong to *Apanteles*, and it is better placed in *Glyptapanteles* for the time being. However, the information available is not enough to conclude with absolute certainty on the generic status of the species, and study of the single male specimen will be required to clarify its status in the future.

***Glyptapantelescaffreyi* (Muesebeck, 1921)**

*Apantelescaffreyi* Muesebeck, 1921.

**Type information.** Holotype female, USNM (not examined but original description checked). Country of type locality: USA.

**Geographical distribution.**NEA, NEO.

**NEA**: USA (AZ); **NEO**: Mexico, Peru.

***Glyptapantelescallidus* (Haliday, 1834)**

*Microgastercallidus* Haliday, 1834.

*Apantelesurolus* Papp, 1983.

**Type information.** Lectotype female, NMID (not examined but subsequent treatment of the species checked). Country of type locality: unknown.

**Geographical distribution.**PAL.

**PAL**: Armenia, Austria, Belgium, Bulgaria, Czech Republic, Finland, France, Georgia, Germany, Hungary, Ireland, Israel, Lithuania, Netherlands, Poland, Romania, Russia (AMU, SAK), Slovakia, Sweden, Switzerland, Turkey, Ukraine, United Kingdom.

**Notes.**van Achterberg (1997) reinterpreted this name and treated it only *sensu*Haliday (1834). The species called *callidus* by Nixon (1973) and Papp (1983a) are now considered to be *Glyptapantelesmajalis* (Wesmael, 1837) (e.g., van Achterberg 1997, Broad et al. 2016).

***Glyptapantelescapeki* (Györfi, 1955)**

*Apantelescapeki* Györfi, 1955.

**Type information.** Holotype female, depository unknown (not examined). Country of type locality: Slovakia.

**Geographical distribution.**PAL.

**PAL**: Slovakia.

***Glyptapantelescarinachicaizae* Arias-Penna, 2019**

*Glyptapantelescarinachicaizae* Arias-Penna, 2019.

**Type information.** Holotype female, QCAZ (examined). Country of type locality: Ecuador.

**Geographical distribution.**NEO.

**NEO**: Ecuador.

***Glyptapantelescarinatus* (Szépligeti, 1913)**

*Apantelescarinatus* Szépligeti, 1913.

**Type information.** Holotype male, HNHM (not examined but subsequent treatment of the species checked). Country of type locality: Tanzania.

**Geographical distribution.**AFR.

**AFR**: Tanzania.

**Notes.** Our species concept is based on Papp (2004).

***Glyptapantelescarlhuffakeri* Arias-Penna, 2019**

*Glyptapantelescarlhuffakeri* Arias-Penna, 2019.

**Type information.** Holotype female, CNC (examined). Country of type locality: Costa Rica.

**Geographical distribution.**NEO.

**NEO**: Costa Rica.

***Glyptapantelescarlossarmientoi* Arias-Penna, 2019**

*Glyptapantelescarlossarmientoi* Arias-Penna, 2019.

**Type information.** Holotype female, CNC (examined). Country of type locality: Costa Rica.

**Geographical distribution.**NEO.

**NEO**: Costa Rica.

***Glyptapantelescarlrettenmeyeri* Arias-Penna, 2019**

*Glyptapantelescarlrettenmeyeri* Arias-Penna, 2019.

**Type information.** Holotype female, CNC (examined). Country of type locality: Costa Rica.

**Geographical distribution.**NEO.

**NEO**: Costa Rica.

***Glyptapantelescassianus* (Riley, 1881)**

*Apantelescassianus* Riley, 1881.

**Type information.** Syntypes female and male, USNM (examined). Country of type locality: USA.

**Geographical distribution.**NEA.

**NEA**: USA (CO, CT, IL, IA, MO, NJ, TX).

***Glyptapantelescelsoazevedoi* Arias-Penna, 2019**

*Glyptapantelescelsoazevedoi* Arias-Penna, 2019.

**Type information.** Holotype male, QCAZ (examined). Country of type locality: Ecuador.

**Geographical distribution.**NEO.

**NEO**: Ecuador.

***Glyptapantelescharlesmicheneri* Arias-Penna, 2019**

*Glyptapantelescharlesmicheneri* Arias-Penna, 2019.

**Type information.** Holotype female, CNC (examined). Country of type locality: Costa Rica.

**Geographical distribution.**NEO.

**NEO**: Costa Rica.

***Glyptapantelescharlesporteri* Arias-Penna, 2019**

*Glyptapantelescharlesporteri* Arias-Penna, 2019.

**Type information.** Holotype female, CNC (examined). Country of type locality: Costa Rica.

**Geographical distribution.**NEO.

**NEO**: Costa Rica.

***Glyptapanteleschidra* Rousse & Gupta, 2013**

*Glyptapanteleschidra* Rousse & Gupta, 2013.

**Type information.** Holotype female, MNHN (not examined but original description checked). Country of type locality: Réunion.

**Geographical distribution.**AFR.

**AFR**: Réunion.

***Glyptapanteleschrisdarlingi* Arias-Penna, 2019**

*Glyptapanteleschrisdarlingi* Arias-Penna, 2019.

**Type information.** Holotype female, CNC (examined). Country of type locality: Costa Rica.

**Geographical distribution.**NEO.

**NEO**: Costa Rica.

***Glyptapanteleschrisgrinteri* Arias-Penna, 2019**

*Glyptapanteleschrisgrinteri* Arias-Penna, 2019.

**Type information.** Holotype female, CNC (examined). Country of type locality: Costa Rica.

**Geographical distribution.**NEO.

**NEO**: Costa Rica.

***Glyptapanteleschristerhanssoni* Arias-Penna, 2019**

*Glyptapanteleschristerhanssoni* Arias-Penna, 2019.

**Type information.** Holotype female, CNC (examined). Country of type locality: Costa Rica.

**Geographical distribution.**NEO.

**NEO**: Costa Rica.

***Glyptapantelescinyras* (de Saeger, 1944), new combination**

*Apantelescinyras* de Saeger, 1944.

**Type information.** Holotype female, RMCA (not examined but original description checked). Country of type locality: Rwanda.

**Geographical distribution.**AFR.

**AFR**: Rwanda.

**Notes.** Based on the original description, the best generic placement would be in *Glyptapanteles*.

***Glyptapantelesclanisae* Gupta, 2013**

*Glyptapantelesclanisae* Gupta, 2013.

**Type information.** Holotype female, NBAIR (not examined but original description checked). Country of type locality: India.

**Geographical distribution.**OTL.

**OTL**: India.

***Glyptapantelesclaudiamartinezae* Arias-Penna, 2019**

*Glyptapantelesclaudiamartinezae* Arias-Penna, 2019.

**Type information.** Holotype female, QCAZ (examined). Country of type locality: Ecuador.

**Geographical distribution.**NEO.

**NEO**: Ecuador.

***Glyptapantelescolemani* (Viereck, 1912)**

*Apantelescolemani* Viereck, 1912.

**Type information.** Holotype female, USNM (examined). Country of type locality: India.

**Geographical distribution.**OTL.

**OTL**: China (GX), India, Vietnam.

***Glyptapantelescompressiventris* (Muesebeck, 1921)**

*Apantelescompressiventris* Muesebeck, 1921.

**Type information.** Holotype female, USNM (not examined but original description checked). Country of type locality: USA.

**Geographical distribution.**NEA, PAL.

**NEA**: Canada (MB, NT, NU, QC), USA (NH); **PAL**: Armenia, Azerbaijan, Croatia, Czech Republic, Finland, Germany, Hungary, Italy, Kazakhstan, Lithuania, Macedonia, Moldova, Netherlands, Romania, Russia (KAM, PRI, SAK, SPE, VOR), Serbia, Slovakia, Spain, Switzerland, Turkey, United Kingdom.

***Glyptapantelescompressus* (Muesebeck, 1919)**

*Apantelescompressus* Muesebeck, 1919.

**Type information.** Holotype female, USNM (not examined but subsequent treatment of the species checked). Country of type locality: USA.

**Geographical distribution.**NEA.

**NEA**: USA (MA, NH, RI, VA, WV).

**Notes.** Our species concept is based on Muesebeck (1921), Mason (1981) and Whitfield (1995a).

***Glyptapantelesconcinnus* (Muesebeck, 1958)**

*Apantelesconcinnus* Muesebeck, 1958.

*Apantelesconcinnus* Muesebeck, 1958 [primary homonym of *Apantelesconcinnus* Statz, 1938].

**Type information.** Holotype female, USNM (not examined but original description checked). Country of type locality: Brazil.

**Geographical distribution.**NEO.

**NEO**: Brazil (SP).

***Glyptapantelescorbetti* (Wilkinson, 1928)**

*Apantelescorbetti* Wilkinson, 1928.

**Type information.** Holotype female, NHMUK (examined). Country of type locality: Malaysia.

**Geographical distribution.**OTL.

**OTL**: China (GX), Malaysia.

***Glyptapantelescorriemoreauae* Arias-Penna, 2019**

*Glyptapantelescorriemoreauae* Arias-Penna, 2019.

**Type information.** Holotype female, CNC (examined). Country of type locality: Costa Rica.

**Geographical distribution.**NEO.

**NEO**: Costa Rica.

***Glyptapantelescreatonoti* (Viereck, 1912)**

*Apantelescreatonoti* Viereck, 1912.

**Type information.** Holotype female, USNM (examined). Country of type locality: India.

**Geographical distribution.**OTL.

**OTL**: Bangladesh, India, Malaysia.

***Glyptapantelesdalosoma* de Santis, 1987**

*Glyptapantelesdalosoma* de Santis, 1987.

**Type information.** Holotype female, MLP (not examined but subsequent treatment of the species checked). Country of type locality: Brazil.

**Geographical distribution.**NEO.

**NEO**: Brazil (SP).

**Notes.** Our species concept is based on Aquino et al. (2010).

***Glyptapantelesdarjeelingensis* (Sharma & Chatterjee, 1970)**

*Apantelesdarjeelingensis* Sharma & Chatterjee, 1970.

**Type information.** Holotype male, FSCA? (not examined but original description checked). Country of type locality: India.

**Geographical distribution.**OTL.

**OTL**: India.

**Notes.** The original description refers to the Gupta collection, which we assume to be currently deposited in the FSCA (at least the Braconidae part); however, there is also the possibility that the type of this species is deposited elsewhere.

***Glyptapantelesdaveroubiki* Arias-Penna, 2019**

*Glyptapantelesdaveroubiki* Arias-Penna, 2019.

**Type information.** Holotype female, CNC (examined). Country of type locality: Costa Rica.

**Geographical distribution.**NEO.

**NEO**: Costa Rica.

***Glyptapantelesdaveschindeli* Arias-Penna, 2019**

*Glyptapantelesdaveschindeli* Arias-Penna, 2019.

**Type information.** Holotype female, CNC (examined). Country of type locality: Costa Rica.

**Geographical distribution.**NEO.

**NEO**: Costa Rica.

***Glyptapantelesdavesmithi* Arias-Penna, 2019**

*Glyptapantelesdavesmithi* Arias-Penna, 2019.

**Type information.** Holotype female, CNC (examined). Country of type locality: Costa Rica.

**Geographical distribution.**NEO.

**NEO**: Costa Rica.

***Glyptapantelesdavidwahli* Arias-Penna, 2019**

*Glyptapantelesdavidwahli* Arias-Penna, 2019.

**Type information.** Holotype female, CNC (examined). Country of type locality: Costa Rica.

**Geographical distribution.**NEO.

**NEO**: Costa Rica.

***Glyptapantelesdeliasa* Austin & Dangerfield, 1992**

*Glyptapantelesdeliasa* Austin & Dangerfield, 1992.

**Type information.** Holotype female, ANIC (not examined but original description checked). Country of type locality: Australia.

**Geographical distribution.**AUS.

**AUS**: Australia (SA).

***Glyptapantelesdiegocamposi* Arias-Penna, 2019**

*Glyptapantelesdiegocamposi* Arias-Penna, 2019.

**Type information.** Holotype female, QCAZ (examined). Country of type locality: Ecuador.

**Geographical distribution.**NEO.

**NEO**: Ecuador.

***Glyptapantelesdistatus* Papp, 1990**

*Glyptapantelesdistatus* Papp, 1990.

**Type information.** Holotype female, HNHM (not examined but original description checked). Country of type locality: Korea.

**Geographical distribution.**PAL.

**PAL**: Korea.

***Glyptapantelesdonquickei* Arias-Penna, 2019**

*Glyptapantelesdonquickei* Arias-Penna, 2019.

**Type information.** Holotype female, CNC (examined). Country of type locality: Costa Rica.

**Geographical distribution.**NEO.

**NEO**: Costa Rica.

***Glyptapantelesdorislagosae* Arias-Penna, 2019**

*Glyptapantelesdorislagosae* Arias-Penna, 2019.

**Type information.** Holotype male, QCAZ (examined). Country of type locality: Ecuador.

**Geographical distribution.**NEO.

**NEO**: Ecuador.

***Glyptapantelesecuadorius* Whitfield, 2002**

*Glyptapantelesecuadorius* Whitfield, 2002.

**Type information.** Holotype female, USNM (examined). Country of type locality: Ecuador.

**Geographical distribution.**NEO.

**NEO**: Ecuador.

**Notes.** Holotype specimen is relatively dirty and not in good condition.

***Glyptapantelesedgardpalacioi* Arias-Penna, 2019**

*Glyptapantelesedgardpalacioi* Arias-Penna, 2019.

**Type information.** Holotype female, QCAZ (examined). Country of type locality: Ecuador.

**Geographical distribution.**NEO.

**NEO**: Ecuador.

***Glyptapantelesedwinnarvaezi* Arias-Penna, 2019**

*Glyptapantelesedwinnarvaezi* Arias-Penna, 2019.

**Type information.** Holotype female, QCAZ (examined). Country of type locality: Ecuador.

**Geographical distribution.**NEO.

**NEO**: Ecuador.

***Glyptapanteleseowilsoni* Arias-Penna, 2019**

*Glyptapanteleseowilsoni* Arias-Penna, 2019.

**Type information.** Holotype female, CNC (examined). Country of type locality: Costa Rica.

**Geographical distribution.**NEO.

**NEO**: Costa Rica.

***Glyptapanteleserictepei* Arias-Penna, 2019**

*Glyptapanteleserictepei* Arias-Penna, 2019.

**Type information.** Holotype female, QCAZ (examined). Country of type locality: Ecuador.

**Geographical distribution.**NEO.

**NEO**: Ecuador.

***Glyptapanteleseryphanidis* (Whitfield, 2011), new combination**

*Protapanteleseryphanidis* Whitfield, 2011.

**Type information.** Holotype male, USNM (not examined but original description checked). Country of type locality: Ecuador.

**Geographical distribution.**NEO.

**NEO**: Ecuador.

**Notes.** The original description (Greeney et al. 2011) was based on a single male, usually the more difficult sex to work with in Microgastrinae (Whitfield 1997). However, the illustrations in the original description clearly show T1 and T2 smooth, with T1 narrowing towards the posterior margin and T2 subtriangular; and the propodeum lacks a median carina and only has a few short carinulae radiating from the nucha. All of those characters suggest that the species is better placed within *Glyptapanteles* instead of *Protapanteles*, a decision we make here. More evidence, if only weak, comes from biology, something that even the authors recognized and mentioned in the paper (Greeney et al. 2011: 1087) when they acknowledged that the host family (Nymphalidae) had never been recorded for *Protapanteles* (e.g., Mason 1981, Whitfield 1997, Whitfield et al. 1999).

***Glyptapanteleseucosmae* (Wilkinson, 1929)**

*Apanteleseucosmae* Wilkinson, 1929.

*Apantelessalensis* Hedqvist, 1965.

**Type information.** Holotype female, NHMUK (examined). Country of type locality: Nigeria.

**Geographical distribution.**AFR, PAL.

**AFR**: Cape Verde, Democratic Republic of Congo, Nigeria, Senegal, Uganda, Zambia; **PAL**: China (LN), Mongolia.

***Glyptapanteleseuproctisiphagus* (Ahmad, 1945), new combination**

*Apanteleseuproctisiphagus* Ahmad, 1945.

**Type information.** Holotype female, INPC (not examined but subsequent treatment of the species checked). Country of type locality: India.

**Geographical distribution.**OTL.

**OTL**: India.

**Notes.** Our species concept is based on Papp (1983a), who placed *euproctisiphagus* within a key comprising other *Glyptapanteles*, and also provided illustrations of the species.

***Glyptapanteleseutelus* (de Saeger, 1941), new combination**

*Apanteleseutelus* de Saeger, 1941.

**Type information.** Holotype male, RMCA (not examined but original description checked). Country of type locality: Democratic Republic of Congo.

**Geographical distribution.**AFR.

**AFR**: Democratic Republic of Congo, Ivory Coast, Rwanda, Senegal.

**Notes.** Based on the original description (de Saeger 1941b), the best generic placement would be in *Glyptapanteles*.

***Glyptapantelesfabiae* (Wilkinson, 1928), new combination**

*Apantelesfabiae* Wilkinson, 1928.

**Type information.** Holotype female, NHMUK (examined). Country of type locality: India.

**Geographical distribution.**OTL.

**OTL**: India.

**Notes.** This species is placed in *Glyptapanteles* based on the very short ovipositor sheaths, inflexible hypopygium, T1 narrowing towards posterior margin, and T2 subtriangular (trapezoidal).

***Glyptapantelesfelipesotoi* Arias-Penna, 2019**

*Glyptapantelesfelipesotoi* Arias-Penna, 2019.

**Type information.** Holotype female, QCAZ (examined). Country of type locality: Ecuador.

**Geographical distribution.**NEO.

**NEO**: Ecuador.

***Glyptapantelesfemoratus* Ashmead, 1906**

*Glyptapantelesfemoratus* Ashmead, 1906.

**Type information.** Lectotype male, USNM (not examined but subsequent treatment of the species checked). Country of type locality: Japan.

**Geographical distribution.**OTL, PAL.

**OTL**: China (HB, ZJ); **PAL**: Japan, Korea.

**Notes.** Our species concept is based on Watanabe (1932, 1937) and Chen and Song (2004).

***Glyptapantelesferfernandezi* Arias-Penna, 2019**

*Glyptapantelesferfernandezi* Arias-Penna, 2019.

**Type information.** Holotype female, QCAZ (examined). Country of type locality: Ecuador.

**Geographical distribution.**NEO.

**NEO**: Ecuador.

***Glyptapantelesficus* (Granger, 1949)**

*Apantelesficus* Granger, 1949.

**Type information.** Holotype female, MNHN (not examined but original description checked). Country of type locality: Madagascar.

**Geographical distribution.**AFR.

**AFR**: Madagascar, Réunion.

**Notes.** Our species concept is based on Granger (1949) and Rousse and Gupta (2013).

***Glyptapantelesflavicoxis* (Marsh, 1979)**

*Apantelesflavicoxis* Marsh, 1979.

**Type information.** Holotype female, USNM (not examined but original description checked). Country of type locality: India.

**Geographical distribution.**OTL.

**OTL**: India.

***Glyptapantelesflavovariatus* (Muesebeck, 1921)**

*Apantelesflavovariatus* Muesebeck, 1921.

**Type information.** Holotype female, USNM (not examined but original description checked). Country of type locality: USA.

**Geographical distribution.**NEA.

**NEA**: Canada (BC, ON), USA (MI, OR, SD).

***Glyptapantelesfloridanus* (Muesebeck, 1921)**

*Apantelesfloridanus* Muesebeck, 1921.

**Type information.** Holotype female, USNM (not examined but original description checked). Country of type locality: USA.

**Geographical distribution.**NEA.

**NEA**: USA (FL).

***Glyptapantelesfraternus* (Reinhard, 1880)**

*Apantelesfraternus* Reinhard, 1880.

**Type information.** Holotype female, ZMHB (not examined but subsequent treatment of the species checked). Country of type locality: Austria.

**Geographical distribution.**PAL.

**PAL**: Austria, Bosnia and Herzegovina, Croatia, Czech Republic, France, Germany, Hungary, Kazakhstan, Moldova, Mongolia, Poland, Romania, Russia (ZAB, TY), Slovakia, Switzerland, Turkmenistan, Ukraine, United Kingdom, Uzbekistan, Yugoslavia.

**Notes.** Our species concept is based on Wilkinson (1945), Nixon (1973), Papp (1983a), Tobias (1986) and Kotenko (2007a). The species distribution in Turkmenistan is based on Belokobylskij et al. (2019).

***Glyptapantelesfullawayi* Austin & Dangerfield, 1992**

*Glyptapantelesfullawayi* Austin & Dangerfield, 1992.

*Apantelespolitus* Fullaway, 1941 [primary homonym of *Apantelespolitus* Riley, 1881].

**Type information.** Holotype male, BPBM (not examined but original description checked). Country of type locality: Western Samoa.

**Geographical distribution.**AUS.

**AUS**: Western Samoa.

***Glyptapantelesfulvigaster* (Granger, 1949), new combination**

*Apantelesfulvigaster* Granger, 1949.

**Type information.** Syntypes female and male, MNHN (not examined but original description checked). Country of type locality: Madagascar.

**Geographical distribution.**AFR.

**AFR**: Madagascar.

**Notes.** This species is clearly not an *Apanteles*, based on the short ovipositor sheaths. In the original description it is considered to be related to *Apantelesbelliger* Wilkinson, which is now placed within *Distatrix*. However, Granger (1949) did not mention in his description that *fulvigaster* has ovipositor sheaths lacking setae (which could be argued to be a noticeable feature and would have indeed shown the species to belong to *Distatrix*). The original description does not mention any details on the lateral sulci on pronotum either, which would have helped to clarify the generic position of the species. Due to all of the above, we take the conservative approach of transferring *fulvigaster* to *Glyptapanteles*, which fits better with the available description. However, we caution that the species could be within *Distatrix* once the specimens from the type series can be examined further.

***Glyptapantelesfulvipes* (Haliday, 1834)**

*Microgasterfulvipes* Haliday, 1834.

**Type information.** Lectotype female, NMID (not examined but subsequent treatment of the species checked). Country of type locality: Ireland.

**Geographical distribution.**NEA, PAL.

**NEA**: Canada (AB, NT, NU, QC), Greenland; **PAL**: Armenia, Austria, Azerbaijan, Belarus, Belgium, Bulgaria, Canary Islands, Croatia, Czech Republic, Faroe Islands, Finland, France, Georgia, Germany, Hungary, Iceland, Ireland, Italy, Japan, Kazakhstan, Korea, Lithuania, Macedonia, Moldova, Mongolia, Netherlands, Poland, Romania, Russia (AMU, ZAB, DA, AL, KDA, MOS, PRI, SAK, SPE, YAR), Serbia, Slovakia, Slovenia, Spain, Sweden, Switzerland, Turkey, Ukraine, United Kingdom.

**Notes.** Our species concept is based on Nixon (1973), Papp (1983a), Tobias (1986), Kotenko (2007a, van Achterberg (2006) and Fernandez-Triana et al. (2017b).

***Glyptapantelesfuscinervis* (Cameron, 1911), new combination**

*Apantelesfuscinervis* Cameron, 1911.

**Type information.** Holotype male, TMSA (not examined but original description checked). Country of type locality: South Africa.

**Geographical distribution.**AFR.

**AFR**: Rwanda, South Africa.

**Notes.** Based on the description of Wilkinson (1932a) the best generic placement at present would be in *Glyptapanteles*.

***Glyptapantelesgahinga* (de Saeger, 1944), new combination**

*Apantelesgahinga* de Saeger, 1944.

**Type information.** Syntypes female and male, RMCA (not examined but original description checked). Country of type locality: Rwanda.

**Geographical distribution.**AFR.

**AFR**: Rwanda.

**Notes.** Based on the original description, the best generic placement would be in *Glyptapanteles*.

***Glyptapantelesgarygibsoni* Arias-Penna, 2019**

*Glyptapantelesgarygibsoni* Arias-Penna, 2019.

**Type information.** Holotype female, CNC (examined). Country of type locality: Costa Rica.

**Geographical distribution.**NEO.

**NEO**: Costa Rica.

***Glyptapantelesgavinbroadi* Arias-Penna, 2019**

*Glyptapantelesgavinbroadi* Arias-Penna, 2019.

**Type information.** Holotype female, CNC (examined). Country of type locality: Costa Rica.

**Geographical distribution.**NEO.

**NEO**: Costa Rica.

***Glyptapantelesgenorodriguezae* Arias-Penna, 2019**

*Glyptapantelesgenorodriguezae* Arias-Penna, 2019.

**Type information.** Holotype female, QCAZ (examined). Country of type locality: Ecuador.

**Geographical distribution.**NEO.

**NEO**: Ecuador.

***Glyptapantelesgerarddelvarei* Arias-Penna, 2019**

*Glyptapantelesgerarddelvarei* Arias-Penna, 2019.

**Type information.** Holotype female, CNC (examined). Country of type locality: Costa Rica.

**Geographical distribution.**NEO.

**NEO**: Costa Rica.

***Glyptapantelesglobatus* (Linnaeus, 1758), new combination**

*Ichneumonglobatus* Linnaeus, 1758.

**Type information.** Syntypes female and male, LSUK (not examined but illustrations of the type series examined). Country of type locality: Sweden.

**Geographical distribution.**PAL.

**PAL**: Sweden.

**Notes.** The use of the name *Ichneumonglobatus* Linnaeus, 1758 has been problematic for a long time, as it was mostly associated with the genus *Microgaster* in Europe (e.g., Yu et al. 2012, 2016, Broad et al. 2016; see also van Achterberg 2014 and Scaramozzino et al. 2017, for more details on the topic). Because the type series of *globatus* clearly does not belong to *Microgaster*, van Achterberg (2014) proposed to use the name *Microgasterrufipes* Nees, 1834 (the oldest available name) for the historical references to that *Microgaster* species in Europe, a decision we accept and follow here (see our rationale to do that in the Notes we provide in this paper under the species *Microgasterrufipes*). As for the type series of *globatus*, those specimens are deposited in The Linnean Society, and two photos of those syntypes are shown in their website (http://linnean-on1ine.org/16250/). After examining those images (at least four specimens are distinguishable in the two photos, one clearly being a female), we think that the best generic placement at present would be in *Glyptapanteles*, and propose this new combination here, based on the T1 narrowing towards posterior margin and T2 subtriangular (as evident from one the specimens photographed that are on the cocoon mass) and the short ovipositor sheaths (as evident on the female specimen also photographed on the cocoon mass, the specimen being the closest to the pin holding the mass). The name *Glyptapantelesglobatus* (Linnaeus, 1758), as we propose here, would be limited for the time being to the specimens from the Linnaeus series, which are supposedly from Sweden (e.g., see Linnaeus 1761: 411, specimen 1645). Future studies of those specimens will be needed to place this species within the larger context of European and Palearctic *Glyptapanteles*.

***Glyptapantelesglyphodes* (Wilkinson, 1932), new combination**

*Apantelesglyphodes* Wilkinson, 1932.

**Type information.** Holotype female, NHMUK (examined). Country of type locality: Uganda.

**Geographical distribution.**AFR.

**AFR**: Uganda.

**Notes.** This species is placed in *Glyptapanteles* based on the very short ovipositor sheaths, inflexible hypopygium, T1 narrowing towards posterior margin, and T2 subtriangular (trapezoidal).

***Glyptapantelesgowdeyi* (Gahan, 1918)**

*Apantelesgowdeyi* Gahan, 1918.

**Type information.** Holotype female, USNM (not examined but subsequent treatment of the species checked). Country of type locality: Uganda.

**Geographical distribution.**AFR.

**AFR**: Democratic Republic of Congo, Uganda.

**Notes.** Our species concept is based on Wilkinson (1932a), de Saeger (1944) and Mason (1981).

***Glyptapantelesgrantgentryi* Arias-Penna, 2019**

*Glyptapantelesgrantgentryi* Arias-Penna, 2019.

**Type information.** Holotype female, QCAZ (examined). Country of type locality: Ecuador.

**Geographical distribution.**NEO.

**NEO**: Ecuador.

***Glyptapantelesguierae* (Risbec, 1951), new combination**

*Apantelesguierae* Risbec, 1951.

**Type information.** Syntypes female and male, depository unknown (not examined but original description checked). Country of type locality: Senegal.

**Geographical distribution.**AFR.

**AFR**: Senegal.

**Notes.** From the original description is evident that this species is not *Apanteles*, based on the sculpture of propodeum and shapes of T1 and T2, the best generic placement at present would be in *Glyptapanteles*. That is also supported by the original description, where Risbec (1951: 423) considered the species to be related to *Apanteleseucosmae* (Wilkinson, 1929) which has long been placed within *Glyptapanteles* (e.g., Mason 1981).

***Glyptapantelesgunnarbrehmi* Arias-Penna, 2019**

*Glyptapantelesgunnarbrehmi* Arias-Penna, 2019.

**Type information.** Holotype female, QCAZ (examined). Country of type locality: Ecuador.

**Geographical distribution.**NEO.

**NEO**: Ecuador.

***Glyptapantelesguyanensis* (Cameron, 1911), lectotype designation**

*Apantelesguyanensis* Cameron, 1911.

**Type information.** Lectotype female, NHMUK (examined). Country of type locality: Guyana.

**Geographical distribution.**NEO.

**NEO**: Guyana.

**Notes.** The type series has four female specimens, all glued on the same card. Shenefelt (1972: 527) mentioned the need to designate a lectotype but did not formally propose it (as he did for many other species in that paper). For the sake of completion, here we designate the lectotype. It is the female placed at the extreme left of the card, which is not only the best-preserved specimen but also has an X below it, which works to clearly mark the lectotype specimen among the series. Taxapad (Yu et al. 2012, 2016) reported the species as occurring in Guyana and Australia, with the latter country being based on Wilkinson (1930c). However, Austin and Dangerfield (1992) considered the Australian specimens to be different from the type series (Guyana). Here we agree with Austin and Dangerfield (1992) and consider *Glyptapantelesguyanensis* as strictly Neotropical (Guyana). A recent paper (Gallardo-Covas 2005) mentioned the possibility of this species also being in Puerto Rico (the species being reported as “probably *guayanensis*”, the species name being misspelled throughout the manuscript), and even mentions *Pseudoplusiaincludens* (Noctuidae) as its host in the island. However, Gallardo-Covas (2005) did not mention how the specimens were identified and thus we consider here that the Puerto Rico record must be confirmed before being formally listed as part of the species distribution.

***Glyptapantelesharoldgreeneyi* Arias-Penna, 2019**

*Glyptapantelesharoldgreeneyi* Arias-Penna, 2019.

**Type information.** Holotype female, QCAZ (examined). Country of type locality: Ecuador.

**Geographical distribution.**NEO.

**NEO**: Ecuador.

***Glyptapantelesharrisinae* (Muesebeck, 1953)**

*Apantelesharrisinae* Muesebeck, 1953.

**Type information.** Holotype female, USNM (not examined but subsequent treatment of the species checked). Country of type locality: USA.

**Geographical distribution.**NEA, NEO.

**NEA**: USA (AZ, CA, CT, FL); **NEO**: Mexico.

**Notes.** Our species concept is based on Papp (1984a) and Whitfield (1995a).

***Glyptapanteleshelmuthaguirrei* Arias-Penna, 2019**

*Glyptapanteleshelmuthaguirrei* Arias-Penna, 2019.

**Type information.** Holotype female, QCAZ (examined). Country of type locality: Ecuador.

**Geographical distribution.**NEO.

**NEO**: Ecuador.

***Glyptapanteleshenryhespenheidei* Arias-Penna, 2019**

*Glyptapanteleshenryhespenheidei* Arias-Penna, 2019.

**Type information.** Holotype female, QCAZ (examined). Country of type locality: Ecuador.

**Geographical distribution.**NEO.

**NEO**: Ecuador.

***Glyptapanteleshenrytownesi* Arias-Penna, 2019**

*Glyptapanteleshenrytownesi* Arias-Penna, 2019.

**Type information.** Holotype female, CNC (examined). Country of type locality: Costa Rica.

**Geographical distribution.**NEO.

**NEO**: Costa Rica.

***Glyptapantelesherbertii* (Ashmead, 1900)**

*Apantelesherbertii* Ashmead, 1900.

**Type information.** Holotype female, NHMUK (examined). Country of type locality: Saint Vincent.

**Geographical distribution.**NEA, NEO.

**NEA**: USA (FL); **NEO**: Argentina, Belize, Colombia, Cuba, Ecuador, Grenada, Mexico, Nicaragua, Peru, Saint Vincent, Venezuela.

***Glyptapanteleshorus* (de Saeger, 1944), new combination**

*Apanteleshorus* de Saeger, 1944.

**Type information.** Holotype female, RMCA (not examined but original description checked). Country of type locality: Democratic Republic of Congo.

**Geographical distribution.**AFR.

**AFR**: Democratic Republic of Congo, Rwanda.

**Notes.** Based on the original description, the best generic placement would be in *Glyptapanteles*.

***Glyptapanteleshowelldalyi* Arias-Penna, 2019**

*Glyptapanteleshowelldalyi* Arias-Penna, 2019.

**Type information.** Holotype female, CNC (examined). Country of type locality: Costa Rica.

**Geographical distribution.**NEO.

**NEO**: Costa Rica.

***Glyptapanteleshugokonsi* Arias-Penna, 2019**

*Glyptapanteleshugokonsi* Arias-Penna, 2019.

**Type information.** Holotype female, CNC (examined). Country of type locality: Costa Rica.

**Geographical distribution.**NEO.

**NEO**: Costa Rica.

***Glyptapanteleshydroeciae* (You & Xiong, 1983)**

*Apanteleshydroeciae* You & Xiong, 1983.

**Type information.** Holotype female, HUNAU (not examined but subsequent treatment of the species checked). Country of type locality: China.

**Geographical distribution.**OTL.

**OTL**: China (SN).

**Notes.** Our species concept is based on Chen and Song (2004).

***Glyptapanteleshypermnestrae* Gupta & Pereira, 2012**

*Glyptapanteleshypermnestrae* Gupta & Pereira, 2012.

**Type information.** Holotype female, NBAIR (not examined but original description checked). Country of type locality: India.

**Geographical distribution.**OTL.

**OTL**: India.

***Glyptapantelesiangauldi* Arias-Penna, 2019**

*Glyptapantelesiangauldi* Arias-Penna, 2019.

**Type information.** Holotype female, CNC (examined). Country of type locality: Costa Rica.

**Geographical distribution.**NEO.

**NEO**: Costa Rica.

***Glyptapantelesianyarrowi* Arias-Penna, 2019**

*Glyptapantelesianyarrowi* Arias-Penna, 2019.

**Type information.** Holotype female, CNC (examined). Country of type locality: Costa Rica.

**Geographical distribution.**NEO.

**NEO**: Costa Rica.

***Glyptapantelesilarisaaksjarvi* Arias-Penna, 2019**

*Glyptapantelesilarisaaksjarvi* Arias-Penna, 2019.

**Type information.** Holotype female, CNC (examined). Country of type locality: Costa Rica.

**Geographical distribution.**NEO.

**NEO**: Costa Rica.

***Glyptapantelesinclusus* (Ratzeburg, 1844)**

*Microgasterinclusus* Ratzeburg, 1844.

*Microgastercurvulus* Thomson, 1895.

*Apantelesrectinervis* Telenga, 1955.

**Type information.** Lectotype female, ZMHB (not examined but subsequent treatment of the species checked). Country of type locality: unknown.

**Geographical distribution.**AFR, PAL.

**AFR**: Cape Verde; **PAL**: Austria, Azerbaijan, Bulgaria, China (SD, SN), Denmark, France, Germany, Ireland, Italy, Japan, Kazakhstan, Korea, Mongolia, Poland, Romania, Russia (ZAB, IRK, PRI, TY), Slovakia, Switzerland, Ukraine, United Kingdom.

**Notes.** Our species concept is based on Wilkinson (1945), Nixon (1973), Papp (1983a), Tobias (1986), and Chen and Song (2004). The species distribution in Japan and Mongolia is based on Belokobylskij et al. (2019).

***Glyptapantelesindiensis* (Marsh, 1979)**

*Apantelesindiensis* Marsh, 1979.

**Type information.** Holotype female, USNM (not examined but original description checked). Country of type locality: India.

**Geographical distribution.**NEA, OTL.

**NEA**: USA (PA), **OTL**: India.

***Glyptapantelesintermedius* (Balevski, 1980)**

*Apantelesintermedius* Balevski, 1980.

**Type information.** Holotype female, ZIN (not examined but subsequent treatment of the species checked). Country of type locality: Bulgaria.

**Geographical distribution.**PAL.

**PAL**: Bulgaria, Ukraine.

**Notes.** Our species concept is based on Tobias (1986) and Kotenko (2006).

***Glyptapantelesintricatus* (de Saeger, 1944), new combination**

*Apantelesintricatus* de Saeger, 1944.

**Type information.** Holotype female, RMCA (not examined but original description checked). Country of type locality: Rwanda.

**Geographical distribution.**AFR.

**AFR**: Democratic Republic of Congo, Rwanda.

**Notes.** The original description of *A.intricatus* contains drawings that show this species is better placed within *Glyptapanteles*. See also comments above under *Glyptapantelesargus* (de Saeger, 1944).

***Glyptapantelesjacklonginoi* Arias-Penna, 2019**

*Glyptapantelesjacklonginoi* Arias-Penna, 2019.

**Type information.** Holotype female, CNC (examined). Country of type locality: Costa Rica.

**Geographical distribution.**NEO.

**NEO**: Costa Rica.

***Glyptapantelesjamesrobertsoni* Arias-Penna, 2019**

*Glyptapantelesjamesrobertsoni* Arias-Penna, 2019.

**Type information.** Holotype female, CNC (examined). Country of type locality: Costa Rica.

**Geographical distribution.**NEO.

**NEO**: Costa Rica.

***Glyptapantelesjaquioconnorae* Arias-Penna, 2019**

*Glyptapantelesjaquioconnorae* Arias-Penna, 2019.

**Type information.** Holotype female, QCAZ (examined). Country of type locality: Ecuador.

**Geographical distribution.**NEO.

**NEO**: Ecuador.

***Glyptapantelesjeremydewaardi* Arias-Penna, 2019**

*Glyptapantelesjeremydewaardi* Arias-Penna, 2019.

**Type information.** Holotype female, CNC (examined). Country of type locality: Costa Rica.

**Geographical distribution.**NEO.

**NEO**: Costa Rica.

***Glyptapantelesjerrypowelli* Arias-Penna, 2019**

*Glyptapantelesjerrypowelli* Arias-Penna, 2019.

**Type information.** Holotype female, QCAZ (examined). Country of type locality: Ecuador.

**Geographical distribution.**NEO.

**NEO**: Ecuador.

***Glyptapantelesjesusugaldei* Arias-Penna, 2019**

*Glyptapantelesjesusugaldei* Arias-Penna, 2019.

**Type information.** Holotype female, CNC (examined). Country of type locality: Costa Rica.

**Geographical distribution.**NEO.

**NEO**: Costa Rica.

***Glyptapantelesjimmilleri* Arias-Penna, 2019**

*Glyptapantelesjimmilleri* Arias-Penna, 2019.

**Type information.** Holotype female, QCAZ (examined). Country of type locality: Ecuador.

**Geographical distribution.**NEO.

**NEO**: Ecuador.

***Glyptapantelesjjrodriguezae* Arias-Penna, 2019**

*Glyptapantelesjjrodriguezae* Arias-Penna, 2019.

**Type information.** Holotype female, CNC (examined). Country of type locality: Costa Rica.

**Geographical distribution.**NEO.

**NEO**: Costa Rica.

***Glyptapantelesjohnburnsi* Arias-Penna, 2019**

*Glyptapantelesjohnburnsi* Arias-Penna, 2019.

**Type information.** Holotype female, CNC (examined). Country of type locality: Costa Rica.

**Geographical distribution.**NEO.

**NEO**: Costa Rica.

***Glyptapantelesjohnheratyi* Arias-Penna, 2019**

*Glyptapantelesjohnheratyi* Arias-Penna, 2019.

**Type information.** Holotype female, CNC (examined). Country of type locality: Costa Rica.

**Geographical distribution.**NEO.

**NEO**: Costa Rica.

***Glyptapantelesjohnlasallei* Arias-Penna, 2019**

*Glyptapantelesjohnlasallei* Arias-Penna, 2019.

**Type information.** Holotype female, CNC (examined). Country of type locality: Costa Rica.

**Geographical distribution.**NEO.

**NEO**: Costa Rica.

***Glyptapantelesjohnnoyesi* Arias-Penna, 2019**

*Glyptapantelesjohnnoyesi* Arias-Penna, 2019.

**Type information.** Holotype female, CNC (examined). Country of type locality: Costa Rica.

**Geographical distribution.**NEO.

**NEO**: Costa Rica.

***Glyptapantelesjohnstiremani* Arias-Penna, 2019**

*Glyptapantelesjohnstiremani* Arias-Penna, 2019.

**Type information.** Holotype female, QCAZ (examined). Country of type locality: Ecuador.

**Geographical distribution.**NEO.

**NEO**: Ecuador.

***Glyptapantelesjosesimbanai* Arias-Penna, 2019**

*Glyptapantelesjosesimbanai* Arias-Penna, 2019.

**Type information.** Holotype male, QCAZ (examined). Country of type locality: Ecuador.

**Geographical distribution.**NEO.

**NEO**: Ecuador.

***Glyptapantelesjuanvargasi* Arias-Penna, 2019**

*Glyptapantelesjuanvargasi* Arias-Penna, 2019.

**Type information.** Holotype male, QCAZ (examined). Country of type locality: Ecuador.

**Geographical distribution.**NEO.

**NEO**: Ecuador.

***Glyptapantelesjumamuturii* Arias-Penna, 2019**

*Glyptapantelesjumamuturii* Arias-Penna, 2019.

**Type information.** Holotype female, QCAZ (examined). Country of type locality: Ecuador.

**Geographical distribution.**NEO.

**NEO**: Ecuador.

***Glyptapanteleskeithwillmotti* Arias-Penna, 2019**

*Glyptapanteleskeithwillmotti* Arias-Penna, 2019.

**Type information.** Holotype female, QCAZ (examined). Country of type locality: Ecuador.

**Geographical distribution.**NEO.

**NEO**: Ecuador.

***Glyptapanteleskevinjohnsoni* Arias-Penna, 2019**

*Glyptapanteleskevinjohnsoni* Arias-Penna, 2019.

**Type information.** Holotype female, QCAZ (examined). Country of type locality: Ecuador.

**Geographical distribution.**NEO.

**NEO**: Ecuador.

***Glyptapanteleskyleparksi* Arias-Penna, 2019**

*Glyptapanteleskyleparksi* Arias-Penna, 2019.

**Type information.** Holotype female, QCAZ (examined). Country of type locality: Ecuador.

**Geographical distribution.**NEO.

**NEO**: Ecuador.

***Glyptapanteleslamborni* (Wilkinson, 1928)**

*Apanteleslamborni* Wilkinson, 1928.

**Type information.** Holotype female, NHMUK (examined). Country of type locality: Malaysia.

**Geographical distribution.**OTL.

**OTL**: China (GZ, HN, TW, YN), Malaysia.

***Glyptapanteleslamprosemae* (Wilkinson, 1928), new combination**

*Apanteleslamprosemae* Wilkinson, 1928.

**Type information.** Holotype female, NHMUK (examined). Country of type locality: Malaysia.

**Geographical distribution.**OTL.

**OTL**: Malaysia.

**Notes.** This species is placed in *Glyptapanteles* based on the very short ovipositor sheaths, inflexible hypopygium, T1 narrowing towards posterior margin, and T2 subtriangular (trapezoidal).

***Glyptapanteleslaxatus* (Wilkinson, 1930)**

*Apanteleslaxatus* Wilkinson, 1930.

**Type information.** Holotype female, NHMUK (examined). Country of type locality: Uganda.

**Geographical distribution.**AFR.

**AFR**: Uganda.

***Glyptapanteleslefevrei* (de Saeger, 1941), new combination**

*Apanteleslefevrei* de Saeger, 1941.

**Type information.** Holotype female, RMCA (not examined but original description checked). Country of type locality: Rwanda.

**Geographical distribution.**AFR.

**AFR**: Burundi, Rwanda.

**Notes.** Here transferred to *Glyptapanteles* based on propodeum with median, longitudinal carina (defined on posterior half of propodeum), short ovipositor sheaths, and shape and sculpture of T1 and T2 (de Saeger 1941a: 333–335).

***Glyptapantelesleucotretae* (Ullyett, 1946), new combination**

*Apantelesleucotretae* Ullyett, 1946.

**Type information.** Holotype female, TMSA (not examined but original description checked). Country of type locality: South Africa.

**Geographical distribution.**AFR.

**AFR**: South Africa.

**Notes.** Based on the original description, the best generic placement is in *Glyptapanteles*, due to the propodeum having a partial median carina, the shapes of T1 and T2, acute hypopygium and length of ovipositor sheaths. Ullyett (1946) also mentions the species as being close to *Glyptapantelesfuscinervis* Cameron.

***Glyptapanteleslinghsiuae* Arias-Penna, 2019**

*Glyptapanteleslinghsiuae* Arias-Penna, 2019.

**Type information.** Holotype female, QCAZ (examined). Country of type locality: Ecuador.

**Geographical distribution.**NEO.

**NEO**: Ecuador.

***Glyptapantelesliparidis* (Bouché, 1834)**

*Microgasterliparidis* Bouché, 1834.

*Microgasternemorum* Hartig, 1838.

*Microgasterliparidis* Ratzeburg, 1844 [primary homonym of *Microgasterliparidis* Bouché, 1834].

*Glyptapantelesjaponicus* Ashmead, 1906.

*Glyptapantelespolitus* Ashmead, 1906.

*Apantelesposticae* Sonan, 1927.

*Apantelesawanomeigae* Watanabe, 1942.

**Type information.** Holotype female, ZMHB (not examined but authoritatively identified specimens examined). Country of type locality: Germany.

**Geographical distribution.**OTL, PAL.

**OTL**: China (HN, TW, ZJ), India; **PAL**: Austria, Belarus, Bulgaria, China (BJ, HL, JL, LN, NM, SN), Czech Republic, Finland, France, Germany, Hungary, Iran, Italy, Japan, Kazakhstan, Korea, Lithuania, Moldova, Mongolia, Poland, Romania, Russia (ZAB, IRK, KGD, KHA, KDA, NVS, PRI, SAK, SPE, SAR, TOM, VOR, YAR), Serbia, Slovakia, Spain, Sweden, Switzerland, Ukraine.

**Notes.** We examined the female type of *A.japonicus*Ashmead (1906) in the USNM and most of the specimens of *Apantelesawanomeigae* (Watanabe, 1942) which were seen and determined by Watanabe.

***Glyptapanteleslissopleurus* (de Saeger, 1944), new combination**

*Apanteleslissopleurus* de Saeger, 1944.

**Type information.** Holotype male, RMCA (not examined but original description checked). Country of type locality: Democratic Republic of Congo.

**Geographical distribution.**AFR.

**AFR**: Democratic Republic of Congo.

**Notes.** Based on the original description, which is the only reference available for this species, the best generic placement at present would be in *Glyptapanteles*. However, the only known specimen is a male and the description is not clear enough to rule out the genus *Distatrix*. Examination of the specimen will be needed to conclude.

***Glyptapanteleslongiantennatus* (You & Xiong, 1987)**

*Apanteleslongiantennatus* You & Xiong, 1987.

**Type information.** Holotype female, HUNAU (not examined but subsequent treatment of the species checked). Country of type locality: China.

**Geographical distribution.**OTL.

**OTL**: China (HN).

**Notes.** Our species concept is based on Kotenko (2007a).

***Glyptapanteleslongistigma* Chen & Song, 2004**

*Glyptapanteleslongistigma* Chen & Song, 2004.

**Type information.** Holotype female, FAFU (not examined but original description checked). Country of type locality: China.

**Geographical distribution.**PAL.

**OTL**: China (HB).

***Glyptapanteleslongivena* Chen & Song, 2004**

*Glyptapanteleslongivena* Chen & Song, 2004.

**Type information.** Holotype female, FAFU (not examined but original description checked). Country of type locality: China.

**Geographical distribution.**OTL.

**OTL**: China (FJ).

***Glyptapanteleslubomasneri* Arias-Penna, 2019**

*Glyptapanteleslubomasneri* Arias-Penna, 2019.

**Type information.** Holotype female, CNC (examined). Country of type locality: Costa Rica.

**Geographical distribution.**NEO.

**NEO**: Costa Rica.

***Glyptapantelesluchosalagajei* Arias-Penna, 2019**

*Glyptapantelesluchosalagajei* Arias-Penna, 2019.

**Type information.** Holotype female, QCAZ (examined). Country of type locality: Ecuador.

**Geographical distribution.**NEO.

**NEO**: Ecuador.

***Glyptapantelesluciana* (Nixon, 1973)**

*Apantelesluciana* Nixon, 1973.

**Type information.** Holotype female, NHMUK (examined). Country of type locality: United Kingdom.

**Geographical distribution.**PAL.

**PAL**: Armenia, Bulgaria, Finland, Germany, Greece, Hungary, Korea, Madeira Islands, Netherlands, Romania, Slovakia, Switzerland, United Kingdom.

***Glyptapanteleslucidus* (Sharma, 1972)**

*Apanteleslucidus* Sharma, 1972.

*Apanteleslucidus* Sharma, 1972 [primary junior homonym of *Apanteleslucidus* Szépligeti].

**Type information.** Holotype female, IFRI (not examined but original description checked). Country of type locality: India.

**Geographical distribution.**OTL.

**OTL**: India.

***Glyptapantelesluteipennis* (Muesebeck, 1921)**

*Apantelesluteipennis* Muesebeck, 1921.

**Type information.** Holotype female, USNM (examined). Country of type locality: USA.

**Geographical distribution.**NEA.

**NEA**: USA (VA).

**Notes.** After examining the holotype, we believe the specimen may be better placed in *Protapanteles*, because of the sculpture and carination of propodeum. However, the fore tarsus does not have a thick seta (usual for *Protapanteles*) and the ovipositor sheaths are hidden inside the hypopygium so it is not clear if they have setae or not. Because only the holotype is known, we refrain from transferring the species here and prefer to retain it in *Glyptapanteles*, as Mason (1981) suggested, although future studies may change that.

***Glyptapantelesmaculitarsis* (Cameron, 1905)**

*Apantelesmaculitarsis* Cameron, 1905.

*Apantelescapensis* Cameron, 1907.

*Apantelesafricanus* Viereck, 1911 [primary homonym of *Apantelesafricanus* Cameron, 1911].

*Apantelestestaceioventris* Cameron, 1911.

*Apantelestestaceolineatus* Cameron, 1911.

*Apantelestestaceiventris* Brues, 1926 [emendation].

**Type information.** Holotype female, depository unknown (not examined but authoritatively identified specimens examined). Country of type locality: South Africa.

**Geographical distribution.**AFR.

**AFR**: Ethiopia, Kenya, Malawi, Nigeria, Senegal, Sierra Leone, South Africa, Tanzania, Uganda.

**Notes.** We examined the type, a female specimen, of *Apantelesafricanus* (Viereck, 1911), currently a synonym of *G.maculitarsis*.

***Glyptapantelesmadecassus* (Granger, 1949), new combination**

*Glyptapantelesmadecassus* Granger, 1949.

**Type information.** Syntypes female and male, MNHN (not examined but original description checked). Country of type locality: Madagascar.

**Geographical distribution.**AFR.

**AFR**: Madagascar.

**Notes.** This species is not an *Apanteles*. Based on the original description (including an illustration of T1-T3), as well as host information, the species is provisionally transferred to *Glyptapanteles* until examination of the syntype series allows a more definitive identification.

***Glyptapantelesmajalis* (Wesmael, 1837)**

*Microgastermajalis* Wesmael, 1837.

*Microgastercallidus* Haliday, 1834 [misidentification].

**Type information.** Syntypes female and male, RBINS (not examined but subsequent treatment of the species checked). Country of type locality: Belgium.

**Geographical distribution.**PAL.

**PAL**: Belgium, Germany, United Kingdom.

**Notes.**Van Achterberg (1997) treated *majalis* as the valid name for the species called *callidus* by Nixon (1973) and Papp (1983a). We follow Broad et al. (2016) for the generic placement of this species.

***Glyptapantelesmalleyneae* Arias-Penna, 2019**

*Glyptapantelesmalleyneae* Arias-Penna, 2019.

**Type information.** Holotype male, QCAZ (examined). Country of type locality: Ecuador.

**Geographical distribution.**NEO.

**NEO**: Ecuador.

***Glyptapantelesmalloryvanwyngaardenae* Arias-Penna, 2019**

*Glyptapantelesmalloryvanwyngaardenae* Arias-Penna, 2019.

**Type information.** Holotype female, CNC (examined). Country of type locality: Costa Rica.

**Geographical distribution.**NEO.

**NEO**: Costa Rica.

***Glyptapantelesmalthacae* (Muesebeck, 1958)**

*Apantelesmalthacae* Muesebeck, 1958.

**Type information.** Holotype female, USNM (examined). Country of type locality: Mexico.

**Geographical distribution.**NEO.

**NEO**: Mexico.

***Glyptapantelesmamiae* Arias-Penna, 2019**

*Glyptapantelesmamiae* Arias-Penna, 2019.

**Type information.** Holotype female, QCAZ (examined). Country of type locality: Ecuador.

**Geographical distribution.**NEO.

**NEO**: Ecuador.

***Glyptapantelesmarcelotavaresi* Arias-Penna, 2019**

*Glyptapantelesmarcelotavaresi* Arias-Penna, 2019.

**Type information.** Holotype female, QCAZ (examined). Country of type locality: Ecuador.

**Geographical distribution.**NEO.

**NEO**: Ecuador.

***Glyptapantelesmarcepsteini* Arias-Penna, 2019**

*Glyptapantelesmarcepsteini* Arias-Penna, 2019.

**Type information.** Holotype female, QCAZ (examined). Country of type locality: Ecuador.

**Geographical distribution.**NEO.

**NEO**: Ecuador.

***Glyptapantelesmarcpolleti* Arias-Penna, 2019**

*Glyptapantelesmarcpolleti* Arias-Penna, 2019.

**Type information.** Holotype male, QCAZ (examined). Country of type locality: Ecuador.

**Geographical distribution.**NEO.

**NEO**: Ecuador.

***Glyptapantelesmarjorietownesae* Arias-Penna, 2019**

*Glyptapantelesmarjorietownesae* Arias-Penna, 2019.

**Type information.** Holotype female, CNC (examined). Country of type locality: Costa Rica.

**Geographical distribution.**NEO.

**NEO**: Costa Rica.

***Glyptapantelesmarkshawi* Arias-Penna, 2019**

*Glyptapantelesmarkshawi* Arias-Penna, 2019.

**Type information.** Holotype female, CNC (examined). Country of type locality: Costa Rica.

**Geographical distribution.**NEO.

**NEO**: Costa Rica.

***Glyptapantelesmarquesi* (Brèthes, 1924), new combination**

*Protapantelesmarquesi* Brèthes, 1924.

**Type information.** Holotype female, MACN (not examined but authoritatively identified specimens examined). Country of type locality: Argentina.

**Geographical distribution.**NEO.

**NEO**: Argentina, Brazil (SC).

**Notes.** Since its description within *Protapanteles*, this species has been variously treated as *Apanteles* (Shenefelt 1972) or as *Cotesia* (Yu et al. 2016). We have examined a relatively large series of 23 specimens from Brazil, which are deposited in the CNC and were identified to species by William Mason in 1978, after he compared them versus the type. Those specimens clearly belong to *Glyptapanteles*, based on the metasoma dorsally smooth, T1 narrowing towards posterior margin, T2 subtriangular, and propodeum mostly smooth and without carinae. Two of those specimens (with voucher codes CNCHYM 01307 and CNCHYM 01308 in BOLD) rendered partial DNA barcodes, which cluster near other species of Neotropical *Glyptapanteles*, corroborating the generic placement we propose here.

***Glyptapantelesmarshawheelerae* Arias-Penna, 2019**

*Glyptapantelesmarshawheelerae* Arias-Penna, 2019.

**Type information.** Holotype female, QCAZ (examined). Country of type locality: Ecuador.

**Geographical distribution.**NEO.

**NEO**: Ecuador.

***Glyptapantelesmayberenbaumae* Arias-Penna, 2019**

*Glyptapantelesmayberenbaumae* Arias-Penna, 2019.

**Type information.** Holotype female, QCAZ (examined). Country of type locality: Ecuador.

**Geographical distribution.**NEO.

**NEO**: Ecuador.

***Glyptapantelesmeganmiltonae* Arias-Penna, 2019**

*Glyptapantelesmeganmiltonae* Arias-Penna, 2019.

**Type information.** Holotype female, CNC (examined). Country of type locality: Costa Rica.

**Geographical distribution.**NEO.

**NEO**: Costa Rica.

***Glyptapantelesmegistusocellus* Song & Chen, 2004**

*Glyptapantelesmegistusocellus* Song & Chen, 2004.

**Type information.** Holotype female, FAFU (not examined but original description checked). Country of type locality: China.

**Geographical distribution.**PAL.

**PAL**: China (JL).

***Glyptapantelesmehrdadhajibabaei* Arias-Penna, 2019**

*Glyptapantelesmehrdadhajibabaei* Arias-Penna, 2019.

**Type information.** Holotype female, CNC (examined). Country of type locality: Costa Rica.

**Geographical distribution.**NEO.

**NEO**: Costa Rica.

***Glyptapantelesmelanotus* (de Saeger, 1944), new combination**

*Apantelesmelanotus* de Saeger, 1944.

**Type information.** Holotype female, RMCA (not examined but original description checked). Country of type locality: Democratic Republic of Congo.

**Geographical distribution.**AFR.

**AFR**: Democratic Republic of Congo.

**Notes.** Based on the original description, the best generic placement would be in *Glyptapanteles*.

***Glyptapantelesmelissus* (de Saeger, 1944), new combination**

*Apantelesmelissus* de Saeger, 1944.

**Type information.** Holotype male, RMCA (not examined but original description checked). Country of type locality: Rwanda.

**Geographical distribution.**AFR.

**AFR**: Rwanda.

**Notes.** Based on the original description, the best generic placement would be in *Glyptapanteles*.

***Glyptapantelesmenander* (Nixon, 1973)**

*Apantelesmenander* Nixon, 1973.

**Type information.** Holotype female, MZH (not examined but original description checked). Country of type locality: Finland.

**Geographical distribution.**PAL.

**PAL**: Finland, United Kingdom.

***Glyptapantelesmerope* (Nixon, 1965), new combination**

*Apantelesmerope* Nixon, 1965.

**Type information.** Holotype female, USNM (examined). Country of type locality: Malaysia.

**Geographical distribution.**OTL.

**OTL**: Malaysia.

**Notes.** This species is placed in *Glyptapanteles* based on the propodeum with strong and complete median carina, T1 narrowing towards posterior margin, T2 subtriangular, inflexible hypopygium and short ovipositor sheaths.

***Glyptapantelesmichelleduennesae* Arias-Penna, 2019**

*Glyptapantelesmichelleduennesae* Arias-Penna, 2019.

**Type information.** Holotype female, QCAZ (examined). Country of type locality: Ecuador.

**Geographical distribution.**NEO.

**NEO**: Ecuador.

***Glyptapantelesmikegatesi* Arias-Penna, 2019**

*Glyptapantelesmikegatesi* Arias-Penna, 2019.

**Type information.** Holotype female, CNC (examined). Country of type locality: Costa Rica.

**Geographical distribution.**NEO.

**NEO**: Costa Rica.

***Glyptapantelesmikepoguei* Arias-Penna, 2019**

*Glyptapantelesmikepoguei* Arias-Penna, 2019.

**Type information.** Holotype female, QCAZ (examined). Country of type locality: Ecuador.

**Geographical distribution.**NEO.

**NEO**: Ecuador.

***Glyptapantelesmikeschauffi* Arias-Penna, 2019**

*Glyptapantelesmikeschauffi* Arias-Penna, 2019.

**Type information.** Holotype female, CNC (examined). Country of type locality: Costa Rica.

**Geographical distribution.**NEO.

**NEO**: Costa Rica.

***Glyptapantelesmikesharkeyi* Arias-Penna, 2019**

*Glyptapantelesmikesharkeyi* Arias-Penna, 2019.

**Type information.** Holotype female, CNC (examined). Country of type locality: Costa Rica.

**Geographical distribution.**NEO.

**NEO**: Costa Rica.

***Glyptapantelesmilitaris* (Walsh, 1861), lectotype designation**

*Microgastermilitaris* Walsh, 1861.

**Type information.** Lectotype female, USNM (examined). Country of type locality: USA.

**Geographical distribution.**AUS, NEA, NEO, PAL.

**AUS**: Hawaiian Islands; **NEA**: Canada (MB, NB, ON, QC), USA (AZ, AR, CA, CT, DC, FL, IL, IN, IA, KS, LA, MD, MA, MI, MN, MO, NJ, NM, NY, OK, TN, TX, VA); **NEO**: Argentina, Honduras, Mexico, Puerto Rico; **PAL**: Azores, Madeira Islands.

**Notes.** There is a single card piece on the pin, with seven cuts where each syntype is glued. Four syntypes are in relatively poor condition: one has only three legs glued to the card, another has only some legs and metasoma left, a third is missing the head (there is one head loose in the unit tray where the specimens are placed), and a fourth is missing the metasoma. The remaining three syntypes are mostly in good condition (although only two specimens each have one complete antenna remaining). The fourth specimen, from left to right, is a female in relatively fair condition (with one antenna complete and another antenna broken before the middle) and here we designate it as the lectotype; it is placed between a complete specimen to its left and a specimen missing the metasoma to its right.

***Glyptapantelesminor* Ashmead, 1906**

*Glyptapantelesminor* Ashmead, 1906.

**Type information.** Lectotype female, USNM (examined). Country of type locality: Japan.

**Geographical distribution.**OTL, PAL.

**OTL**: China (GZ, TW, ZJ); **PAL**: Japan, Korea.

**Notes.**Yu et al. (2016) transferred the species to *Protapanteles* based on an unpublished PhD thesis on Chinese Cotesiini (Zeng 2012). However, after examining the lectotype in the USNM as well as six female and two male specimens in the EIHU collection, we found that they clearly belong to *Glyptapanteles* (based on smooth propodeum, T1 and T2, as well as shapes of T1 and T2), which is in agreement with other authors (e.g., Papp 1990b, Chen and Song 2004, Kotenko 2007a). Thus, for the sake of clarity the species combination is revised here.

***Glyptapantelesmnesampela* Austin, 2000**

*Glyptapantelesmnesampela* Austin, 2000.

**Type information.** Holotype female, ANIC (not examined but original description checked). Country of type locality: Australia.

**Geographical distribution.**AUS.

**AUS**: Australia (ACT).

***Glyptapantelesmontywoodi* Arias-Penna, 2019**

*Glyptapantelesmontywoodi* Arias-Penna, 2019.

**Type information.** Holotype male, QCAZ (examined). Country of type locality: Ecuador.

**Geographical distribution.**NEO.

**NEO**: Ecuador.

***Glyptapantelesmuesebecki* (Blanchard, 1947)**

*Apantelesmuesebecki* Blanchard, 1947.

**Type information.** Holotype female, MACN (not examined but subsequent treatment of the species checked). Country of type locality: Argentina.

**Geographical distribution.**NEO.

**NEO**: Argentina, Brazil (PR), Paraguay, Peru.

**Notes.** Our species concept is based on Blanchard (1947) and Whitfield et al. (2002a).

***Glyptapantelesmygdonia* (Nixon, 1973)**

*Apantelesmygdonia* Nixon, 1973.

**Type information.** Holotype female, NHMUK (examined). Country of type locality: United Kingdom.

**Geographical distribution.**PAL.

**PAL**: Bulgaria, Finland, France, Germany, Hungary, Iran, Ireland, Italy, Korea, Madeira Islands, Russia (KDA, PRI), Slovakia, Spain, Switzerland, Turkey, United Kingdom.

***Glyptapantelesnaromae* (Risbec, 1951), new combination**

*Apantelesnaromae* Risbec, 1951.

**Type information.** Syntypes female and male, depository unknown (not examined but original description checked). Country of type locality: Senegal.

**Geographical distribution.**AFR.

**AFR**: Senegal.

**Notes.** Based on the original description (including a drawing of propodeum and T1-T2), the best generic placement of this species is in *Glyptapanteles*.

***Glyptapantelesnataliaivanovae* Arias-Penna, 2019**

*Glyptapantelesnataliaivanovae* Arias-Penna, 2019.

**Type information.** Holotype female, CNC (examined). Country of type locality: Costa Rica.

**Geographical distribution.**NEO.

**NEO**: Costa Rica.

***Glyptapantelesnealweberi* Arias-Penna, 2019**

*Glyptapantelesnealweberi* Arias-Penna, 2019.

**Type information.** Holotype female, CNC (examined). Country of type locality: Costa Rica.

**Geographical distribution.**NEO.

**NEO**: Costa Rica.

***Glyptapantelesneoliparidis* Chen & Song, 2004**

*Glyptapantelesneoliparidis* Chen & Song, 2004.

**Type information.** Holotype female, FAFU (not examined but original description checked). Country of type locality: China.

**Geographical distribution.**OTL.

**OTL**: China (FJ).

***Glyptapantelesnepitae* (Wilkinson, 1934), new combination**

*Apantelesnepitae* Wilkinson, 1934.

**Type information.** Holotype female, NHMUK (examined). Country of type locality: Sri Lanka.

**Geographical distribution.**OTL.

**OTL**: Sri Lanka.

**Notes.** After examining the holotype, we place this species in *Glyptapanteles* based on the inflexible hypopygium, short ovipositor sheaths with a few setae, T1 mostly parallel-sided but narrowing towards posterior margin on apical third, and T2 subtriangular (trapezoidal) in shape. However, this species is not typical within the genus, as the propodeum has two short carinae near the nucha, which appear to represent a partial areola (but just very short). Most *Glyptapanteles* species, when they have some carination it is mostly a complete (or partial) median, longitudinal carina, or a few, very short carinae near nucha that do not appear to represent a partial areola. But, other than those carinae, the specimen fits well within *Glyptapanteles* and thus we transfer it to that genus here.

***Glyptapantelesnigerrimus* (Roman, 1924)**

*Apantelesnigerrimus* Roman, 1924.

**Type information.** Lectotype female, NHMO (not examined but subsequent treatment of the species checked). Country of type locality: Russia.

**Geographical distribution.**PAL.

**PAL**: Poland, Romania, Russia (ARK), Yugoslavia.

**Notes.**Shenefelt (1972: 579) recorded the type material for this species (female and male specimens) as being deposited in the NHRS in Stockholm, Sweden. On the other hand, Nixon (1973: 185) referred to the type of the species as being found in the NHMUK London among material previously borrowed by Wilkinson; and Nixon stated that the type was being returned to the NHMO in Oslo, Norway, where it had been originally borrowed from. We follow Nixon for the depository of this species type. However, the type cannot be a holotype, as it was part of a series in the original description (Roman 1924: 19), thus the specimen that Nixon is referring to as type would actually be the lectotype.

***Glyptapantelesnigrescens* (Cameron, 1906), new combination**

*Protapantelesnigrescens* Cameron, 1906.

**Type information.** Holotype male, NHMUK (examined). Country of type locality: Pakistan.

**Geographical distribution.**OTL.

**OTL**: Pakistan.

**Notes.** The holotype, with code 3c.1032, is a male specimen and not a female as previously stated. The confusion is likely due to the relatively small size of the specimen (2.1 mm body length) and the fact that one of the gonoforceps is slightly pulled outwards, more than the rest of the external genitalia, giving the impression of being a very short ovipositor sheath. That must have been very difficult to appreciate with older microscopes and also explains why Wilkinson (1928a: 92) considered the ovipositor sheaths to be shorter than even the metatibial spurs. We have re-examined the specimen (which is in relatively poor condition, covered by metallic rust from the micropin through the mesosoma), and it is evident that is not *Apanteles* but *Glyptapanteles* (which agrees with Wilkinson’s (1928a) assessment of *nigriscens* being related to *creatonoti*, another *Glyptapanteles* species). Also, the type locality (only known locality for the species) is currently in Pakistan, not India (as older references mentioned, and still reflected in Yu et al. 2016).

***Glyptapantelesnigricornis* (Muesebeck, 1921)**

*Apantelesnigricornis* Muesebeck, 1921.

**Type information.** Holotype female, USNM (examined). Country of type locality: USA.

**Geographical distribution.**NEA.

**NEA**: USA (CA, VT).

***Glyptapantelesninazitaniae* Arias-Penna, 2019**

*Glyptapantelesninazitaniae* Arias-Penna, 2019.

**Type information.** Holotype female, CNC (examined). Country of type locality: Costa Rica.

**Geographical distribution.**NEO.

**NEO**: Costa Rica.

***Glyptapantelesninus* (de Saeger, 1944), new combination**

*Apantelesninus* de Saeger, 1944.

**Type information.** Syntypes female and male, RMCA (not examined but original description checked). Country of type locality: Rwanda.

**Geographical distribution.**AFR.

**AFR**: Rwanda.

**Notes.** Based on the original description, the best generic placement would be in *Glyptapanteles*.

***Glyptapantelesnivalis* (Papp, 1983)**

*Apantelesnivalis* Papp, 1983.

**Type information.** Holotype female, HNHM (not examined but original description checked). Country of type locality: Switzerland.

**Geographical distribution.**PAL.

**PAL**: Italy, Switzerland.

***Glyptapantelesnkuli* (de Saeger, 1941), new combination**

*Apantelesnkuli* de Saeger, 1941.

**Type information.** Holotype female, RMCA (not examined but original description checked). Country of type locality: Rwanda.

**Geographical distribution.**AFR.

**AFR**: Rwanda.

**Notes.** Based on the original description (de Saeger 1941a), the best generic placement would be in *Glyptapanteles*.

***Glyptapantelesobliquae* (Wilkinson, 1928)**

*Apantelesobliquae* Wilkinson, 1928.

*Apantelesobliquaeniger* Wilkinson, 1928 [homonym of *Apantelesniger* Muesebeck, 1921)].

**Type information.** Holotype female, NHMUK (examined). Country of type locality: India.

**Geographical distribution.**OTL.

**OTL**: Bangladesh, China (GX), India, Nepal.

***Glyptapantelesoctonarius* (Ratzeburg, 1852)**

*Microgasteroctonarius* Ratzeburg, 1852.

*Apantelesstauropodis* Marshall, 1889 [*nomen nudum*].

*Apanteleslucifugus* Lyle, 1917.

**Type information.** Type lost (not examined but subsequent treatment of the species checked). Country of type locality: unknown.

**Geographical distribution.**PAL.

**PAL**: Azerbaijan, Croatia, Georgia, Germany, Hungary, Ireland, Italy, Lithuania, Netherlands, Poland, Romania, Russia (PRI, TAM), Slovakia, Ukraine, United Kingdom, Yugoslavia.

**Notes.** Our species concept is based on Wilkinson (1945), Nixon (1973), Papp (1983a) and Tobias (1986). We examined the type series of *Apanteleslucifugus* (Lyle, 1917). The species distribution in Azerbaijan is based on Belokobylskij et al. (2019).

***Glyptapantelesoperculinae* (Fullaway, 1941)**

*Apantelesoperculinae* Fullaway, 1941.

**Type information.** Holotype female, BPBM (not examined but subsequent treatment of the species checked). Country of type locality: Western Samoa.

**Geographical distribution.**AUS.

**AUS**: American Samoa, Western Samoa.

**Notes.** Our species concept is based on Austin and Dangerfield (1992).

***Glyptapantelespachopinasi* Arias-Penna, 2019**

*Glyptapantelespachopinasi* Arias-Penna, 2019.

**Type information.** Holotype male, QCAZ (examined). Country of type locality: Ecuador.

**Geographical distribution.**NEO.

**NEO**: Ecuador.

***Glyptapantelespalabundus* (Tobias, 1986)**

*Apantelespalabundus* Tobias, 1986.

**Type information.** Holotype female, ZIN (not examined but original description checked). Country of type locality: Ukraine.

**Geographical distribution.**PAL.

**PAL**: Ukraine.

***Glyptapantelespallipes* (Reinhard, 1880)**

*Apantelespallipes* Reinhard, 1880.

*Apantelespallidipes* Marshall, 1885.

*Microgasterlongicornis* Provancher, 1886.

*Apantelesradiatus* Ashmead, 1898.

*Apantelesreinhardi* Wilkinson, 1936.

**Type information.** Lectotype female, ZMHB (not examined but subsequent treatment of the species checked). Country of type locality: Austria.

**Geographical distribution.**NEA, PAL.

**NEA**: Canada (BC, NB, ON, QC), Greenland, USA (AK, CT, IL, MA, NH, NY, OH, VA); **OTL**: China (FJ, HN, SH), India; **PAL**: Armenia, Austria, Azerbaijan, Belgium, Bulgaria, China (GS, LN), Czech Republic, Denmark, Finland, France, Germany, Hungary, Ireland, Italy, Japan, Korea, Latvia, Lithuania, Macedonia, Mongolia, Poland, Romania, Russia (KGD, MOS, PRI, SAK, VLG, VOR), Spain, Switzerland, Ukraine, United Kingdom, Yugoslavia.

**Notes.** Our species concept is based on Nixon (1965, 1973), Papp (1983a), Tobias (1986), Chen and Song (2004), van Achterberg (2006) and Fernandez-Triana et al. (2017b).

***Glyptapantelespamitchellae* Arias-Penna, 2019**

*Glyptapantelespamitchellae* Arias-Penna, 2019.

**Type information.** Holotype female, CNC (examined). Country of type locality: Costa Rica.

**Geographical distribution.**NEO.

**NEO**: Costa Rica.

***Glyptapantelesparasundanus* (Bhatnagar, 1950), new combination**

*Apantelesparasundanus* Bhatnagar, 1950.

**Type information.** Holotype female, INPC (not examined but original description checked). Country of type locality: India.

**Geographical distribution.**OTL.

**OTL**: India.

**Notes.** The best generic placement for this species is *Glyptapanteles*, based on propodeum having weak, median longitudinal carina but lacking a tranverse carina; T1 parallel-sided on anterior 0.7 but then strongly narrowing towards posterior margin; T2 smooth, trapezoidal in shape and shorter than T3 length; and ovipositor sheaths short. The year of publication of the Bhatnagar paper was until recently commonly cited as 1948 and/or 1950 (e.g., Chen and Song 2004, Yu et al. 2016), probably following Shenefelt (1972) who referred to this paper as “Bhatnagar (1948) 1950”. While the intended year for Volume X, Parts I & II of the Indian Journal of Entomology was 1948, the actual dates of publication were June 1950 (Part I) and October 1950 (Part II), as clearly shown on the cover page of the Volume, which we have checked. Because the dates of publication are the ones to be considered, and for the sake of clarity, we hereby revise the species year of description to 1950.

***Glyptapantelespaulhansoni* Arias-Penna, 2019**

*Glyptapantelespaulhansoni* Arias-Penna, 2019.

**Type information.** Holotype female, CNC (examined). Country of type locality: Costa Rica.

**Geographical distribution.**NEO.

**NEO**: Costa Rica.

***Glyptapantelespaulheberti* Arias-Penna, 2019**

*Glyptapantelespaulheberti* Arias-Penna, 2019.

**Type information.** Holotype female, CNC (examined). Country of type locality: Costa Rica.

**Geographical distribution.**NEO.

**NEO**: Costa Rica.

***Glyptapantelespaulhurdi* Arias-Penna, 2019**

*Glyptapantelespaulhurdi* Arias-Penna, 2019.

**Type information.** Holotype female, CNC (examined). Country of type locality: Costa Rica.

**Geographical distribution.**NEO.

**NEO**: Costa Rica.

***Glyptapantelespenelope* (Nixon, 1965), new combination**

*Apantelespenelope* Nixon, 1965.

**Type information.** Holotype female, USNM (examined). Country of type locality: Malaysia.

**Geographical distribution.**OTL.

**OTL**: Malaysia.

**Notes.** At present, the best generic placement for this species is *Glyptapanteles*, based on its inflexible hypopygium and short ovipositor sheaths. In the holotype a median sulcus on T1 is partially visible, as well as traces of transverse carinae laterally on propodeum (near spiracles).

***Glyptapantelespenelopeus* (Tobias, 1986)**

*Apantelespenelopeus* Tobias, 1986.

**Type information.** Holotype female, ZIN (not examined but original description checked). Country of type locality: Moldova.

**Geographical distribution.**PAL.

**PAL**: Moldova.

***Glyptapantelespenthocratus* (Austin, 1987), new combination**

*Apantelespenthocratus* Austin, 1987.

**Type information.** Holotype female, NHMUK (examined). Country of type locality: Philippines.

**Geographical distribution.**OTL.

**OTL**: Philippines.

**Notes.** The original description makes clear that this species belongs to *Glyptapanteles*, and even a comment from the author explicitly says so (Austin 1987: 149). After examining the holotype we here formally transfer it to *Glyptapanteles*, based on inflexible hypopygium, shapes of T1 and T2, and very short ovipositor sheaths with only setae near apex.

***Glyptapantelespetermarzi* Arias-Penna, 2019**

*Glyptapantelespetermarzi* Arias-Penna, 2019.

**Type information.** Holotype female, QCAZ (examined). Country of type locality: Ecuador.

**Geographical distribution.**NEO.

**NEO**: Ecuador.

***Glyptapantelesphildevriesi* Arias-Penna, 2019**

*Glyptapantelesphildevriesi* Arias-Penna, 2019.

**Type information.** Holotype female, QCAZ (examined). Country of type locality: Ecuador.

**Geographical distribution.**NEO.

**NEO**: Ecuador.

***Glyptapantelesphilippinensis* (Ashmead, 1904), new combination**

*Apantelesphilippinensis* Ashmead, 1904.

**Type information.** Holotype female, USNM (examined). Country of type locality: Philippines.

**Geographical distribution.**OTL.

**OTL**: Philippines.

**Notes.** This species is clearly not *Apanteles*. The holotype has a mostly smooth propodeum, although a median carina is visible on posterior 0.4, as well as two lateral carinae (at both sides of the median carina) which seem to define a partial areola on posterior 0.3 of propodeum; T1 is smooth and mostly parallel-sided but narrowing on posterior 0.3; T2 is smooth and trapezoidal in shape; the ovipositor and ovipositor sheaths are very short (less than 0.2 metatibia length) and the sheaths are mostly without setae (but with a few setae near apex, those setae being as long as the setae on the hypopygium). Most of those features could be associated with *Glyptapanteles* (shapes of T1 and T2; mostly smooth propodeum, T1, and T2, ovipositor and sheaths), but what appears to be a partially defined areola on posterior 0.3 of the propodeum would be closer to *Cotesia* (and in fact, there are *Cotesia* species with similar shape and sculpture of T1 and T2 and mostly smooth propodeum, e.g., see Figure 53 in this paper, showing *Cotesiahispanica*). We prefer to transfer the species to *Glyptapanteles* because Wilkinson (1928a: 91), who was able to examine a female paratype of the species, considered it as very close to *Apantelesphytometrae* Wilkinson, which is now placed in *Glyptapanteles*.

***Glyptapantelesphilocampus* Cameron, 1911, new combination**

*Apantelesphilocampus* Cameron, 1911.

**Type information.** Syntypes female, NHMUK (examined). Country of type locality: Guyana.

**Geographical distribution.**NEO.

**NEO**: Guyana.

**Notes.** After examining the type series, it is evident that this species belongs to the genus *Glyptapanteles* (based on the sort ovipositor sheaths, inflexible hypopygium, subtriangular (trapezoidal) shape of T2 and propodeum mostly shiny and with only small carinae near nucha). Both the original description (Cameron 1911b: 327) and Shenefelt (1972: 599) mention that the type series was composed of female and male; however, after carefully examining it, we found that the five syntypes are female (the ovipositor and sheaths on the extreme left specimen are barely visible because of being covered by glue, which might have been overlooked by earlier authors).

***Glyptapantelesphilwardi* Arias-Penna, 2019**

*Glyptapantelesphilwardi* Arias-Penna, 2019.

**Type information.** Holotype female, CNC (examined). Country of type locality: Costa Rica.

**Geographical distribution.**NEO.

**NEO**: Costa Rica.

***Glyptapantelesphoebe* (Nixon, 1965), new combination**

*Apantelesphoebe* Nixon, 1965.

**Type information.** Holotype female, USNM (examined). Country of type locality: Malaysia.

**Geographical distribution.**OTL.

**OTL**: Malaysia, Philippines.

**Notes.** Transferred to *Glyptapanteles* based on subtriangular T2, inflexible hypopygium and short ovipositor sheaths.

***Glyptapantelesphragmataeciae* (You & Zhou, 1990)**

*Apantelesphragmataeciae* You & Zhou, 1990.

**Type information.** Holotype female, HUNAU (not examined but subsequent treatment of the species checked). Country of type locality: China.

**Geographical distribution.**OTL.

**OTL**: China (HN).

**Notes.** Our species concept is based on Chen and Song (2004).

***Glyptapantelesphytometraduplus* (Shenefelt, 1972), new combination**

*Apantelesphytometraduplus* Shenefelt, 1972.

*Apantelesphytometrae* Risbec, 1951 [homonym of *Apantelesphytometrae* Wilkinson, 1928].

**Type information.** Holotype female, depository unknown (not examined but original description checked). Country of type locality: Senegal.

**Geographical distribution.**AFR.

**AFR**: Senegal.

**Notes.** Based on the original description (and associated drawing of propodeum, T1, and T2) the species is best placed in *Glyptapanteles*. The original description (Risbec 1951) is based on the female, but it does not detail the number of specimens actually examined by the author. However, we make the assumption that only one specimen was seen, as other descriptions in that paper mention the total number of specimens when it is more than one.

***Glyptapantelesphytometrae* (Wilkinson, 1928)**

*Apantelesphytometrae* Wilkinson, 1928.

**Type information.** Holotype female, NHMUK (examined). Country of type locality: Western Samoa.

**Geographical distribution.**AUS, OTL.

**AUS**: Fiji, Western Samoa; **OTL**: Bangladesh, Indonesia.

***Glyptapantelespinicola* (Lyle, 1917)**

*Apantelespinicola* Lyle, 1917.

**Type information.** Lectotype female, NHMUK (examined). Country of type locality: United Kingdom.

**Geographical distribution.**PAL.

**PAL**: Hungary, Italy, Madeira Islands, Romania, Russia (KIR, KRS), Slovakia, Switzerland, United Kingdom.

***Glyptapantelespolitus* (Riley, 1881)**

*Apantelespolitus* Riley, 1881.

**Type information.** Syntypes female and male, USNM (examined). Country of type locality: USA.

**Geographical distribution.**NEA.

**NEA**: USA (FL, IL, MO, NJ).

***Glyptapantelespopovi* (Telenga, 1955)**

*Apantelespopovi* Telenga, 1955.

**Type information.** Lectotype female, depository unknown (not examined but subsequent treatment of the species checked). Country of type locality: Turkmenistan.

**Geographical distribution.**PAL.

**PAL**: Turkey, Turkmenistan.

**Notes.** Our species concept is based on Papp (1983a), Tobias (1986).

***Glyptapantelesporthetriae* (Muesebeck, 1928)**

*Apantelesporthetriae* Muesebeck, 1928.

**Type information.** Holotype female, USNM (examined). Country of type locality: Hungary.

**Geographical distribution.**OTL, PAL.

**OTL**: India; **PAL**: Armenia, Austria, Azerbaijan, Bulgaria, China (JL), Croatia, Finland, France, Georgia, Germany, Greece, Hungary, Iran, Israel, Italy, Korea, Moldova, Morocco, Poland, Portugal, Romania, Russia (ZAB, DA, MOS, PRI, VOR, YAR), Serbia, Slovakia, Spain, Switzerland, Turkey, Ukraine, United Kingdom.

**Notes.** The species distribution in Israel is based on Belokobylskij et al. (2019).

***Glyptapantelespraesens* (Muesebeck, 1947)**

*Apantelespraesens* Muesebeck, 1947.

**Type information.** Holotype female, USNM (examined). Country of type locality: USA.

**Geographical distribution.**AUS, NEA.

**AUS**: Hawaiian Islands; **NEA**: USA (CA).

***Glyptapantelespropylae* (de Saeger, 1941), new combination**

*Apantelespropylae* de Saeger, 1941.

**Type information.** Holotype female, RMCA (not examined but original description checked). Country of type locality: Democratic Republic of Congo.

**Geographical distribution.**AFR.

**AFR**: Democratic Republic of Congo.

**Notes.** Based on the original description (de Saeger 1941a), the best generic placement would be in *Glyptapanteles*.

***Glyptapantelespseudacraeae* Donaldson, 1991**

*Glyptapantelespseudacraeae* Donaldson, 1991.

**Type information.** Holotype female, TMSA (not examined but original description checked). Country of type locality: South Africa.

**Geographical distribution.**AFR.

**AFR**: South Africa.

***Glyptapantelespseudotsugae* Fernandez-Triana, 2018**

*Glyptapantelespseudotsugae* Fernandez-Triana, 2018.

**Type information.** Holotype female, CNC (examined). Country of type locality: USA.

**Geographical distribution.**NEA.

**NEA**: Canada (AB, BC), USA (AZ, CA, OR).

***Glyptapantelespuera* (Wilkinson, 1928), new combination**

*Apantelespuera* Wilkinson, 1928.

**Type information.** Holotype female, NHMUK (examined). Country of type locality: India.

**Geographical distribution.**OTL.

**OTL**: India, Myanmar.

**Notes.** This species is placed in *Glyptapanteles* based on the very short ovipositor sheaths, inflexible hypopygium, T1 narrowing towards posterior margin, and T2 subtriangular (trapezoidal).

***Glyptapantelesrafamanitioi* Arias-Penna, 2019**

*Glyptapantelesrafamanitioi* Arias-Penna, 2019.

**Type information.** Holotype female, QCAZ (examined). Country of type locality: Ecuador.

**Geographical distribution.**NEO.

**NEO**: Ecuador.

***Glyptapantelesripus* (Papp, 1983)**

*Apantelesripus* Papp, 1983.

**Type information.** Holotype female, ZMHB (not examined but original description checked). Country of type locality: Slovakia.

**Geographical distribution.**PAL.

**PAL**: Germany, Hungary, Korea, Macedonia, Poland, Russia (TVE), Slovakia, Spain, Yugoslavia.

***Glyptapantelesrobbinthorpi* Arias-Penna, 2019**

*Glyptapantelesrobbinthorpi* Arias-Penna, 2019.

**Type information.** Holotype female, CNC (examined). Country of type locality: Costa Rica.

**Geographical distribution.**NEO.

**NEO**: Costa Rica.

***Glyptapantelesronaldzunigai* Arias-Penna, 2019**

*Glyptapantelesronaldzunigai* Arias-Penna, 2019.

**Type information.** Holotype female, CNC (examined). Country of type locality: Costa Rica.

**Geographical distribution.**NEO.

**NEO**: Costa Rica.

***Glyptapantelesroysnellingi* Arias-Penna, 2019**

*Glyptapantelesroysnellingi* Arias-Penna, 2019.

**Type information.** Holotype female, CNC (examined). Country of type locality: Costa Rica.

**Geographical distribution.**NEO.

**NEO**: Costa Rica.

***Glyptapantelesrubens* (Reinhard, 1880)**

*Apantelesrubens* Reinhard, 1880.

**Type information.** Holotype male, ZMHB (not examined but subsequent treatment of the species checked). Country of type locality: Germany.

**Geographical distribution.**PAL.

**PAL**: Germany, Israel, Russia (MOS), Ukraine.

**Notes.** Our species concept is based on Papp (1983a), Tobias (1986). The species distribution in Israel is based on Belokobylskij et al. (2019).

***Glyptapantelessagmaria* (Nixon, 1965)**

*Apantelessagmaria* Nixon, 1965.

**Type information.** Holotype female, AEIC (not examined but original description checked). Country of type locality: Philippines.

**Geographical distribution.**OTL.

**OTL**: Philippines.

***Glyptapantelessalepus* (Papp, 1983)**

*Apantelessalepus* Papp, 1983.

**Type information.** Holotype female, RMNH (not examined but original description checked). Country of type locality: Netherlands.

**Geographical distribution.**PAL.

**PAL**: Greece, Netherlands, United Kingdom.

***Glyptapantelessarrothripae* (Weed, 1887)**

*Apantelessarrothripae* Weed, 1887.

**Type information.** Lectotype female, INHS (not examined but subsequent treatment of the species checked). Country of type locality: USA.

**Geographical distribution.**NEA.

**NEA**: Canada (BC, NS, ON), USA (CT, DC, IL, MD, MA, MI, MO, NJ, NY, OH, RI, VA).

**Notes.** Our species concept is based on Muesebeck (1921), Mason (1981), Papp (1983a), Whitfield (1995a) and Fernandez-Triana (2010).

***Glyptapantelesscottmilleri* Arias-Penna, 2019**

*Glyptapantelesscottmilleri* Arias-Penna, 2019.

**Type information.** Holotype female, CNC (examined). Country of type locality: Costa Rica.

**Geographical distribution.**NEO.

**NEO**: Costa Rica.

***Glyptapantelesscottshawi* Arias-Penna, 2019**

*Glyptapantelesscottshawi* Arias-Penna, 2019.

**Type information.** Holotype female, CNC (examined). Country of type locality: Costa Rica.

**Geographical distribution.**NEO.

**NEO**: Costa Rica.

***Glyptapantelesseydeli* (de Saeger, 1941), new combination**

*Apantelesseydeli* de Saeger, 1941.

**Type information.** Syntypes female and male, RMCA (not examined but original description checked). Country of type locality: Democratic Republic of Congo.

**Geographical distribution.**AFR.

**AFR**: Democratic Republic of Congo.

**Notes.** Based on the original description, the best generic placement at present is in *Glyptapanteles*, due to the sculpture and carination pattern of propodeum, shape and sculpture of T1–T2, and the short ovipositor sheaths.

***Glyptapantelesshelbystedenfeldae* Arias-Penna, 2019**

*Glyptapantelesshelbystedenfeldae* Arias-Penna, 2019.

**Type information.** Holotype male, CNC (examined). Country of type locality: Costa Rica.

**Geographical distribution.**NEO.

**NEO**: Costa Rica.

***Glyptapantelessibiricus* (Papp, 1983)**

*Apantelessibiricus* Papp, 1983.

*Apantelessibiricus* Papp, 1983 [homonym of *Apantelessibiricus* Fahringer, 1938].

**Type information.** Holotype female, ZMHB (not examined but original description checked). Country of type locality: Russia.

**Geographical distribution.**PAL.

**PAL**: Germany, Russia, Serbia.

**Notes.** The species distribution in Russia is only quoted as Siberia (Papp 1983a, Belokobylskij et al. 2019).

***Glyptapantelessiderion* (Nixon, 1965), new combination**

*Apantelessiderion* Nixon, 1965.

**Type information.** Holotype female, NHMUK (examined). Country of type locality: Indonesia.

**Geographical distribution.**OTL.

**OTL**: Indonesia.

**Notes.** This species is clearly not an *Apanteles*, based on the inflexible hypopygium and very short, mostly glabrous, ovipositor sheaths. The best generic placement at present would be in *Glyptapanteles*; however, the propodeum has a complete transverse carina (in addition to the median one), and T1 has a weakly defined longitudinal sulcus on the anterior 0.3 of tergite. It is likely that this species, together with *Apantelesatylana* Nixon (which is similar to *siderion*) and several undescribed species we have seen in collections from the Oriental region, will be placed in a different, new genus (related to the Cotesiini group of genera; see section above Brief diagnosis of all Microgastrinae genera as they are understood in this paper, for details of our current concepts on Microgastrinae groups) in the future. Pending the resolution of these species in a future paper, here we transfer *siderion* and *atylana* to *Glyptapanteles*.

***Glyptapantelessimus* (de Saeger, 1944), new combination**

*Apantelessimus* de Saeger, 1944.

**Type information.** Holotype female, RMCA (not examined but original description checked). Country of type locality: Rwanda.

**Geographical distribution.**AFR.

**AFR**: Democratic Republic of Congo, Rwanda.

**Notes.** Based on the original description, the best generic placement at present would be in *Glyptapanteles*. However, the ovipositor sheaths shown in the drawing and in part of the original description also look similar to those found in *Pholetesor*. Further study of the specimens will be needed to conclude.

***Glyptapantelessondrawardae* Arias-Penna, 2019**

*Glyptapantelessondrawardae* Arias-Penna, 2019.

**Type information.** Holotype female, CNC (examined). Country of type locality: Costa Rica.

**Geographical distribution.**NEO.

**NEO**: Costa Rica.

***Glyptapantelesspeciosissimus* (Granger, 1949), new combination**

*Apantelesspeciosissimus* Granger, 1949.

**Type information.** Syntypes female and male, MNHN (not examined but original description checked). Country of type locality: Madagascar.

**Geographical distribution.**AFR.

**AFR**: Madagascar.

**Notes.** Based on the propodeum sculpture, shapes of T1 and T2, and the short length of the ovipositor sheaths (all detailed in the original description), this species is better placed in *Glyptapanteles*.

***Glyptapantelesspilosomae* (de Saeger, 1941), new combination**

*Apantelesspilosomae* de Saeger, 1941.

**Type information.** Holotype female, RMCA (not examined but original description checked). Country of type locality: Angola.

**Geographical distribution.**AFR.

**AFR**: Angola, Democratic Republic of Congo.

**Notes.** Based on the original description (de Saeger 1941a), the best generic placement would be in *Glyptapanteles*.

***Glyptapantelesspodopterae* Ahmad, 2009**

*Glyptapantelesspodopterae* Ahmad, 2009.

**Type information.** Holotype female, AMUZ (not examined but subsequent treatment of the species checked). Country of type locality: India.

**Geographical distribution.**OTL.

**OTL**: India.

**Notes.** Our species concept is based on Gupta & Fernandez-Triana (2014).

***Glyptapantelesstackelbergi* (Telenga, 1955)**

*Apantelesstackelbergi* Telenga, 1955.

**Type information.** Lectotype female, depository unknown (not examined but subsequent treatment of the species checked). Country of type locality: Uzbekistan.

**Geographical distribution.**PAL.

**PAL**: Uzbekistan.

**Notes.** Our species concept is based on Telenga (1955), Papp (1983a) and Tobias (1986).

***Glyptapantelesstephaniecluttsae* Arias-Penna, 2019**

*Glyptapantelesstephaniecluttsae* Arias-Penna, 2019.

**Type information.** Holotype female, CNC (examined). Country of type locality: Costa Rica.

**Geographical distribution.**NEO.

**NEO**: Costa Rica.

***Glyptapantelesstephaniekirkae* Arias-Penna, 2019**

*Glyptapantelesstephaniekirkae* Arias-Penna, 2019.

**Type information.** Holotype female, CNC (examined). Country of type locality: Costa Rica.

**Geographical distribution.**NEO.

**NEO**: Costa Rica.

***Glyptapantelessubpunctatus* (Granger, 1949), new combination**

*Apantelessubpunctatus* Granger, 1949.

**Type information.** Syntypes female and male, MNHN (not examined but original description checked). Country of type locality: Madagascar.

**Geographical distribution.**AFR.

**AFR**: Madagascar.

**Notes.** Based on the propodeum sculpture, shapes of T1 and T2, and the short length of the ovipositor sheaths (all from the original description), the best generic placement for this species is in *Glyptapanteles*.

***Glyptapantelessujeevanratnasinghami* Arias-Penna, 2019**

*Glyptapantelessujeevanratnasinghami* Arias-Penna, 2019.

**Type information.** Holotype female, CNC (examined). Country of type locality: Costa Rica.

**Geographical distribution.**NEO.

**NEO**: Costa Rica.

***Glyptapantelessuniae* Arias-Penna, 2019**

*Glyptapantelessuniae* Arias-Penna, 2019.

**Type information.** Holotype female, QCAZ (examined). Country of type locality: Ecuador.

**Geographical distribution.**NEO.

**NEO**: Ecuador.

***Glyptapantelessureshnaiki* Arias-Penna, 2019**

*Glyptapantelessureshnaiki* Arias-Penna, 2019.

**Type information.** Holotype female, CNC (examined). Country of type locality: Costa Rica.

**Geographical distribution.**NEO.

**NEO**: Costa Rica.

***Glyptapantelessuzannegreenae* Arias-Penna, 2019**

*Glyptapantelessuzannegreenae* Arias-Penna, 2019.

**Type information.** Holotype female, QCAZ (examined). Country of type locality: Ecuador.

**Geographical distribution.**NEO.

**NEO**: Ecuador.

***Glyptapantelessydneycameronae* Arias-Penna, 2019**

*Glyptapantelessydneycameronae* Arias-Penna, 2019.

**Type information.** Holotype female, CNC (examined). Country of type locality: Costa Rica.

**Geographical distribution.**NEO.

**NEO**: Costa Rica.

***Glyptapantelestaniaariasae* Arias-Penna, 2019**

*Glyptapantelestaniaariasae* Arias-Penna, 2019.

**Type information.** Holotype female, QCAZ (examined). Country of type locality: Ecuador.

**Geographical distribution.**NEO.

**NEO**: Ecuador.

***Glyptapantelestanyadapkeyae* Arias-Penna, 2019**

*Glyptapantelestanyadapkeyae* Arias-Penna, 2019.

**Type information.** Holotype male, CNC (examined). Country of type locality: Costa Rica.

**Geographical distribution.**NEO.

**NEO**: Costa Rica.

***Glyptapantelestaylori* (Wilkinson, 1928)**

*Apantelestaylori* Wilkinson, 1928.

**Type information.** Holotype female, NHMUK (examined). Country of type locality: Indonesia.

**Geographical distribution.**OTL.

**OTL**: Indonesia.

***Glyptapantelestheivorae* (Shenefelt, 1972)**

*Apantelestheivorae* Shenefelt, 1972.

*Apantelesgracilariae* Sonan, 1942 [primary homonym of *Apantelesgracilariae* Wilkinson, 1940].

**Type information.** Type and depository unknown (not examined but subsequent treatment of the species checked). Country of type locality: China.

**Geographical distribution.**OTL.

**OTL**: China (GZ, HN, TW, YN, ZJ).

**Notes.** Our species concept is based on Sonan (1942) and Chen and Song (2004).

***Glyptapantelesthespis* (de Saeger, 1944), new combination**

*Apantelesthespis* de Saeger, 1944.

**Type information.** Holotype female, RMCA (not examined but original description checked). Country of type locality: Democratic Republic of Congo.

**Geographical distribution.**AFR.

**AFR**: Democratic Republic of Congo, Rwanda.

**Notes.** Even in the original description, de Saeger (1944) suspected that this species did not belong to *Apanteles*, based on the ovipositor sheaths. The median longitudinal carina on the propodeum, also clearly excludes the species from *Apanteles*. Without examining the specimens, it is impossible to conclude but we consider the best generic placement at present to be in *Glyptapanteles*.

***Glyptapantelesthibautdelsinnei* Arias-Penna, 2019**

*Glyptapantelesthibautdelsinnei* Arias-Penna, 2019.

**Type information.** Holotype male, QCAZ (examined). Country of type locality: Ecuador.

**Geographical distribution.**NEO.

**NEO**: Ecuador.

***Glyptapantelesthomaspapei* Arias-Penna, 2019**

*Glyptapantelesthomaspapei* Arias-Penna, 2019.

**Type information.** Holotype female, QCAZ (examined). Country of type locality: Ecuador.

**Geographical distribution.**NEO.

**NEO**: Ecuador.

***Glyptapantelesthompsoni* (Lyle, 1927)**

*Apantelesthompsoni* Lyle, 1927.

**Type information.** Holotype female, NHMUK (examined). Country of type locality: France.

**Geographical distribution.**AFR, OTL, PAL.

**AFR**: Cameroon; **OTL**: China (TW, ZJ); **PAL**: Belgium, France, Hungary, Iran, Japan, Korea, Moldova, Romania, Russia (NGR, PRI, SAK).

***Glyptapantelesthoseae* (Wilkinson, 1934), new combination**

*Apantelesthoseae* Wilkinson, 1934.

**Type information.** Syntypes female and male, NHMUK (examined). Country of type locality: Indonesia.

**Geographical distribution.**AFR.

**AFR**: Democratic Republic of Congo, Rwanda.

**Notes.** This species is placed in *Glyptapanteles* based on the short ovipositor sheaths, inflexible hypopygium, T1 narrowing towards posterior margin, and T2 subtriangular (= trapezoidal).

***Glyptapantelestoluagunbiadeae* Arias-Penna, 2019**

*Glyptapantelestoluagunbiadeae* Arias-Penna, 2019.

**Type information.** Holotype female, QCAZ (examined). Country of type locality: Ecuador.

**Geographical distribution.**NEO.

**NEO**: Ecuador.

***Glyptapantelestomwallai* Arias-Penna, 2019**

*Glyptapantelestomwallai* Arias-Penna, 2019.

**Type information.** Holotype female, QCAZ (examined). Country of type locality: Ecuador.

**Geographical distribution.**NEO.

**NEO**: Ecuador.

***Glyptapantelestrilochae* Gupta, 2013**

*Glyptapantelestrilochae* Gupta, 2013.

**Type information.** Holotype female, NBAIR (not examined but original description checked). Country of type locality: India.

**Geographical distribution.**OTL.

**OTL**: India.

***Glyptapantelesvafer* (Nixon, 1965)**

*Apantelesvafer* Nixon, 1965.

**Type information.** Holotype female, AEIC (not examined but original description checked). Country of type locality: Philippines.

**Geographical distribution.**OTL.

**OTL**: Philippines.

***Glyptapantelesvenustus* (de Saeger, 1944), new combination**

*Apantelesvenustus* de Saeger, 1944.

**Type information.** Holotype female, RMCA (not examined but original description checked). Country of type locality: Rwanda.

**Geographical distribution.**AFR.

**AFR**: Democratic Republic of Congo, Rwanda, Senegal.

**Notes.** Based on the original description (de Saeger 1944), the best generic placement at present would be in *Glyptapanteles*.

***Glyptapantelesvictoriapookae* Arias-Penna, 2019**

*Glyptapantelesvictoriapookae* Arias-Penna, 2019.

**Type information.** Holotype female, CNC (examined). Country of type locality: Costa Rica.

**Geographical distribution.**NEO.

**NEO**: Costa Rica.

***Glyptapantelesvitripennis* (Curtis, 1830)**

*Microgastervitripennis* Curtis, 1830.

*Microgastervitripennis* Curtis, 1829 [*nomen nudum*].

*Microgasterfulcriger* Wesmael, 1837.

*Apantelesimpavidus* Gautier & du Dresnay, 1926.

**Type information.** Lectotype female, MVMMA (not examined but subsequent treatment of the species checked). Country of type locality: United Kingdom.

**Geographical distribution.**OTL, PAL.

**OTL**: India, Pakistan; **PAL**: Azerbaijan, Belgium, Bulgaria, Czech Republic, Finland, France, Georgia, Germany, Greece, Hungary, Ireland, Italy, Kazakhstan, Kyrgyzstan, Latvia, Poland, Romania, Russia (IRK, MOS, PRI, SPE), Serbia, Slovakia, Spain, Sweden, Switzerland, Tajikistan, Turkey, Turkmenistan, Ukraine, United Kingdom, Uzbekistan.

**Notes.** Our species concept is based on Nixon (1973) and Tobias (1986). The species distribution in Turkmenistan is based on Belokobylskij et al. (2019).

***Glyptapanteleswebsteri* (Muesebeck, 1921)**

*Apanteleswebsteri* Muesebeck, 1921.

**Type information.** Holotype female, USNM (examined). Country of type locality: USA.

**Geographical distribution.**NEA.

**NEA**: Canada (AB, NB, QC), USA (AR, NC, OH).

***Glyptapanteleswilkinsoni* (Fahringer, 1936), new combination**

*Apanteleswilkinsoni* Fahringer, 1936.

*Apantelesplutellae* Wilkinson, 1931 [primary homonym of *Apantelesplutellae* Kurdjumov, 1912].

**Type information.** Holotype female, NHMUK (examined). Country of type locality: Indonesia.

**Geographical distribution.**OTL.

**OTL**: Indonesia.

**Notes.** This species is placed in *Glyptapanteles* based on the short ovipositor sheaths, inflexible hypopygium, T1 slightly narrowing towards posterior margin, and T2 subtriangular (trapezoidal).

***Glyptapanteleswilmersimbanai* Arias-Penna, 2019**

*Glyptapanteleswilmersimbanai* Arias-Penna, 2019.

**Type information.** Holotype female, QCAZ (examined). Country of type locality: Ecuador.

**Geographical distribution.**NEO.

**NEO**: Ecuador.

***Glyptapanteleswonyoungchoi* Arias-Penna, 2019**

*Glyptapanteleswonyoungchoi* Arias-Penna, 2019.

**Type information.** Holotype female, CNC (examined). Country of type locality: Costa Rica.

**Geographical distribution.**NEO.

**NEO**: Costa Rica.

***Glyptapantelesyalizhangae* Arias-Penna, 2019**

*Glyptapantelesyalizhangae* Arias-Penna, 2019.

**Type information.** Holotype female, QCAZ (examined). Country of type locality: Ecuador.

**Geographical distribution.**NEO.

**NEO**: Ecuador.

***Glyptapantelesyanayacuensis* Arias-Penna, 2019**

*Glyptapantelesyanayacuensis* Arias-Penna, 2019.

**Type information.** Holotype female, QCAZ (examined). Country of type locality: Ecuador.

**Geographical distribution.**NEO.

**NEO**: Ecuador.

#### Genus Hygroplitis Thomson, 1895

***Hygroplitis*** Thomson, 1895: 2244. Gender: masculine. Type species: *Microgasterrussatus* Haliday, 1834, by subsequent designation (Viereck 1914: 73).

Originally described as a subgenus of *Microgaster* but elevated to the generic rank by Viereck (1914). Known from nine described species, mostly from the Palaearctic region, with a few taxa reaching the Oriental and Nearctic regions. We have seen a few additional species in collections. Revisions are available for species of China (Xu and Han 2007) and Russia (Kotenko 2007a). The known host records are mostly from three families of Lepidoptera (Crambidae, Noctuidae and Tortricidae). There are 18 DNA-barcode compliant sequences of *Hygroplitis* in BOLD, representing two BINs; molecular data suggest that this genus might be just a group of *Microgaster*, but the evidence is not conclusive at present. The gender of *Hygroplitis* has been treated historically as feminine, but that is incorrect (Doug Yanega, pers. comm.), as the name is based on the Greek noun *?p??t??* (*oplitis*), which is masculine; accordingly, species names are changed below to match the gender of the genus.

***Hygroplitisbasarukini* Kotenko, 1993**

*Hygroplitisbasarukini* Kotenko, 1993.

**Type information.** Holotype female, SIZK (not examined but subsequent treatment of the species checked). Country of type locality: Russia.

**Geographical distribution.**PAL.

**PAL**: Russia (SAK).

**Notes.** Our species concept is based on Kotenko (2006, 2007).

***Hygroplitismelligaster* (Provancher, 1886)**

*Microgastermelligaster* Provancher, 1886.

*Microgasterrubricoxa* Provancher, 1888.

**Type information.** Lectotype female, ULQC (examined). Country of type locality: Canada.

**Geographical distribution.**NEA.

**NEA**: Canada (MB, NB, NS, ON, PE, QC), USA (IA, MA, MI, NJ, NY, VA).

***Hygroplitisnigritus* Luo & You, 2005**

*Hygroplitisnigrita* Luo & You, 2005.

**Type information.** Holotype female, GUGC (not examined but subsequent treatment of the species checked). Country of type locality: China.

**Geographical distribution.**OTL.

**OTL**: China (GZ).

**Notes.** Our species concept is based on Xu and Han (2007) and Kotenko (2007a).

***Hygroplitispseudorussatus* Shaw, 1992**

*Hygroplitispseudorussata* Shaw, 1992.

**Type information.** Holotype female, RSME (examined). Country of type locality: United Kingdom.

**Geographical distribution.**PAL.

**PAL**: Netherlands, United Kingdom.

***Hygroplitisrugulosus* (Nees, 1834)**

*Microgasterrugulosus* Nees, 1834.

*Microgasterinfumata* Haliday, 1834.

*Microgasteropaca* Ruthe, 1858.

**Type information.** Type and depository unknown (not examined but subsequent treatment of the species checked). Country of type locality: Germany.

**Geographical distribution.**PAL.

**PAL**: Czech Republic, Finland, Germany, Hungary, Ireland, Italy, Netherlands, Poland, Russia (C, NW), Sweden, Switzerland, Turkey, Ukraine, United Kingdom.

**Notes.** Our species concept is based on Shaw (2012b).

***Hygroplitisruinosus* Kotenko, 2007**

*Hygroplitisruinosa* Kotenko, 2007.

**Type information.** Holotype female, ZIN (not examined but original description checked). Country of type locality: Russia.

**Geographical distribution.**PAL.

**PAL**: Russia (PRI).

***Hygroplitisrussatus* (Haliday, 1834)**

*Microgasterrussatus* Haliday, 1834.

*Microgasterdimidiata* Wesmael, 1837.

*Microgasterbasalis* Stephens, 1846.

*Microgasteraomoriensis* Matsumura, 1910.

**Type information.** Lectotype male, NHMUK (examined). Country of type locality: unknown.

**Geographical distribution.**OTL, PAL.

**OTL**: China (FJ, GX, GZ, HB, HN, JS, JX, SN, TW, YN, ZJ), Vietnam; **PAL**: Belgium, China (AH, BJ, HA, LN, SN, SD), Finland, France, Germany, Hungary, Ireland, Japan, Korea, Moldova, Netherlands, Poland, Russia (ALT, SA), Sweden, Turkey, Ukraine, United Kingdom.

**Notes.** The lectotype specimen is missing its head (except for the antennae, which are glued to the card) and the anterior part of mesosoma.

***Hygroplitissinicus* (Xu & He, 2000)**

*Microgastersinicus* Xu & He, 2000.

**Type information.** Holotype female, ZJUH (not examined but subsequent treatment of the species checked). Country of type locality: China.

**Geographical distribution.**OTL. PAL.

**OTL**: China (FJ); **PAL**: China (JL).

**Notes.** Our species concept is based on Xu and Han (2007).

***Hygroplitistoritarsis* Song & Chen, 2004**

*Hygroplitistoritarsis* Song & Chen, 2004.

**Type information.** Holotype female, FAFU (not examined but original description checked). Country of type locality: China.

**Geographical distribution.**OTL.

**OTL**: China (FJ).

#### Genus Hypomicrogaster Ashmead, 1898

***Hypomicrogaster*** Ashmead, 1898: 166. Gender: feminine. Type species: *Microgasterzonaria* Say, 1836, by subsequent designation and monotypy (Ashmead 1900a: 132).

Known from 48 species, *Hypomicrogaster* may end up as just a New World genus, with the majority of species found in the Neotropical region. Species from the Old World tropics previously assigned to this genus seem to represent different lineages, and they are all assigned to different genera in this paper. A recent revision of the world species (Valerio and Whitfield 2015) has a number of inaccuracies and does not work well for all species. In addition to that, we have seen more than 100 undescribed species in collections. More than 15 families of Lepidoptera have been recorded as hosts for *Hypomicrogaster*, but many records are likely to be incorrect and/or need further verification. There are 2,100+ DNA-barcode compliant sequences of this genus in BOLD, representing 148 BINs. The gender of *Hypomicrogaster* has at times being treated as masculine; however, all genera ending in *gaster* are feminine, without exception (Doug Yanega, pers. comm., see also Article 30.1.2 of the ICZN). Accordingly, a large number of adjectival epithets in *Hypomicrogaster* are incorrect and are changed below.

***Hypomicrogasteracarnas* Nixon, 1965, status revised**

*Hypomicrogasteracarnas* Nixon, 1965.

**Type information.** Holotype female, NHMUK (examined). Country of type locality: Brazil.

**Geographical distribution.**NEO.

**NEO**: Brazil (SC).

**Notes.**Valerio and Whitfield (2015) synonymized *H.acarnas* under *H.tydeus*, at the same time misspelling the name *acarnas* as *arcanas*. After examining the types of both *tydeus* and *acarnas* (both in the NHMUK) we consider that there are sufficient morphological features to support both as different species, and thus here we remove *acarnas* from synonym with *tydeus* and treat both as separate species. Additionally, we provide some morphological details to separate them.

1) *H.acarnas*: T1 length 1.8 x its width at posterior margin; T1 almost entirely smooth (only very few, shallow, and scattered punctures near posterior margin); T2 width at posterior margin 2.1 × its length; propleuron, pronotum laterally and metacoxa entirely yellow; ovipositor sheaths 0.36 × metatibia length; body length 2.4 mm and fore wing length 2.5 mm.

2) *H.tydeus*: T1 length 1.3 × its width at posterior margin; posterior 0.3 of T1 with punctures; T2 width at posterior margin 3.1 × its length; propleuron, pronotum laterally and anterior half of metacoxa brown; ovipositor sheaths 0.62 × metatibia length; body length and fore wing length 2.8 mm.

***Hypomicrogasteraodoa* Valerio, 2015**

*Hypomicrogasteraodus* Valerio, 2015.

**Type information.** Holotype female, IAVH (not examined but original description checked). Country of type locality: Colombia.

**Geographical distribution.**NEO.

**NEO**: Colombia.

**Notes.**Valerio and Whitfield (2015) stated that the name was adjectival; it therefore must change spelling in compliance with ICZN Article 31.2.

***Hypomicrogasteraplebis* Valerio, 2015**

*Hypomicrogasteraplebis* Valerio, 2015.

**Type information.** Holotype female, MCZC (not examined but original description checked). Country of type locality: Brazil.

**Geographical distribution.**NEO.

**NEO**: Brazil (MT).

***Hypomicrogasterareolaris* (Blanchard, 1947)**

*Apantelesareolaris* Blanchard, 1947.

*Microgasterblanchardi* Muesebeck, 1958 [replacement name].

*Hypomicrogasterdiaphaniae* Muesebeck, 1958.

*Hypomicrogasteracontes* Nixon, 1965.

*Hypomicrogastermetris* Nixon, 1965.

*Hypomicrogastermoscus* Nixon, 1965.

*Hypomicrogastersolox* Nixon, 1965.

**Type information.** Holotype female, MACN (not examined but authoritatively identified specimens examined). Country of type locality: Argentina.

**Geographical distribution.**NEA, NEO.

**NEA**: USA (FL); **NEO**: Argentina, Brazil (DF, SC), Costa Rica, El Salvador, Mexico.

**Notes.** The original name, *Apantelesareolaris* Blanchard, 1947, was transferred to *Microgaster* and then became a secondary junior homonym of *Microgasterareolaris* Thomson, 1895; so Muesebeck (1958b) changed the name to *Microgasterblanchardi* Muesebeck, 1958. Then Valerio and Whitfield (2015) transferred the species to *Hypomicrogaster* as *H.areolaris* (Blanchard). Valerio and Whitfield (2015) also synonymized under '*areolaris*' four other species of *Hypomicrogaster* that had been considered as valid species until that moment (see synonyms above). The type belongs to the Blanchard collection, which we assume is deposited in the MACN. We have examined the types of *H.acontes* Nixon, *H.metris* Nixon (which is broken in pieces, glued to two points on the same pin), *H.moscus* Nixon and *H.solox* Nixon (all in the NHMUK), and we consider that at least some of the synonyms proposed by Valerio and Whitfield (2015) are not justified, i.e., we think some of those species should be considered as valid. However, pending a reassessment of *Hypomicrogaster* in the New World, we refrain from changing the status of those species names in this paper.

***Hypomicrogastercernus* Valerio, 2015**

*Hypomicrogastercernus* Valerio, 2015.

**Type information.** Holotype female, IAVH (not examined but original description checked). Country of type locality: Colombia.

**Geographical distribution.**NEO.

**NEO**: Colombia.

**Notes.**Valerio and Whitfield (2015) stated that the name was adjectival, but this is not an actual Latin adjective; it therefore must be treated as indeclinable under ICZN Article 31.2.3.

***Hypomicrogastercrocina* Valerio, 2015**

*Hypomicrogastercrocinus* Valerio, 2015.

**Type information.** Holotype female, CNC (examined). Country of type locality: Brazil.

**Geographical distribution.**NEO.

**NEO**: Brazil (PE).

**Notes.**Valerio and Whitfield (2015) stated that the name was adjectival; it therefore must change spelling in compliance with ICZN Article 31.2.

***Hypomicrogasterdaktulios* Valerio, 2015**

*Hypomicrogasterdaktulios* Valerio, 2015.

**Type information.** Holotype female, ESUW (not examined but original description checked). Country of type locality: Costa Rica.

**Geographical distribution.**NEO.

**NEO**: Costa Rica.

***Hypomicrogasterdeltis* Valerio, 2015**

*Hypomicrogasterdeltis* Valerio, 2015.

**Type information.** Holotype female, CNC (examined). Country of type locality: Brazil.

**Geographical distribution.**NEO.

**NEO**: Brazil (MT, RJ, RO).

***Hypomicrogasterduo* Valerio, 2015**

*Hypomicrogasterduo* Valerio, 2015.

**Type information.** Holotype female, USNM (not examined but original description checked). Country of type locality: Honduras.

**Geographical distribution.**NEO.

**NEO**: Honduras.

***Hypomicrogasterecus* Nixon, 1965**

*Hypomicrogasterecus* Nixon, 1965.

**Type information.** Holotype female, NHMUK (examined). Country of type locality: Brazil.

**Geographical distribution.**NEO.

**NEO**: Brazil (SC).

***Hypomicrogasterepipagis* Valerio, 2015**

*Hypomicrogasterepipagis* Valerio, 2015.

**Type information.** Holotype female, USNM (not examined but original description checked). Country of type locality: Uruguay.

**Geographical distribution.**NEO.

**NEO**: Bolivia, Uruguay.

***Hypomicrogasterespera* Valerio, 2015**

*Hypomicrogasterespera* Valerio, 2015.

**Type information.** Holotype female, ESUW (not examined but original description checked). Country of type locality: Costa Rica.

**Geographical distribution.**NEO.

**NEO**: Costa Rica.

***Hypomicrogasterevrys* Valerio, 2015**

*Hypomicrogasterevrys* Valerio, 2015.

**Type information.** Holotype female, INBio (not examined but original description checked). Country of type locality: Costa Rica.

**Geographical distribution.**NEO.

**NEO**: Costa Rica.

***Hypomicrogasterguille* Valerio, 2015**

*Hypomicrogasterguille* Valerio, 2015.

**Type information.** Holotype female, CNC (examined). Country of type locality: Ecuador.

**Geographical distribution.**NEO.

**NEO**: Ecuador.

***Hypomicrogasterhektos* Valerio, 2015**

*Hypomicrogasterhektos* Valerio, 2015.

**Type information.** Holotype female, CNC (examined). Country of type locality: Brazil.

**Geographical distribution.**NEO.

**NEO**: Brazil (RJ).

***Hypomicrogasterhupsos* Valerio, 2015**

*Hypomicrogasterhupsos* Valerio, 2015.

**Type information.** Holotype female, CNC (examined). Country of type locality: Ecuador.

**Geographical distribution.**NEO.

**NEO**: Ecuador.

***Hypomicrogasterimitator* (Ashmead, 1900)**

*Urogasterimitator* Ashmead, 1900.

**Type information.** Holotype female, NHMUK (examined). Country of type locality: Saint Vincent.

**Geographical distribution.**NEO.

**NEO**: Grenada, Saint Vincent.

***Hypomicrogasteringensis* Valerio, 2015**

*Hypomicrogasteringensis* Valerio, 2015.

**Type information.** Holotype female, CNC (examined). Country of type locality: Brazil.

**Geographical distribution.**NEO.

**NEO**: Brazil (RJ).

***Hypomicrogasterinsolita* Valerio, 2015**

*Hypomicrogasterinsolitus* Valerio, 2015.

**Type information.** Holotype female, CNC (examined). Country of type locality: Brazil.

**Geographical distribution.**NEO.

**NEO**: Brazil (MT).

**Notes.**Valerio and Whitfield (2015) stated that the name was adjectival; it therefore must change spelling in compliance with ICZN Article 31.2.

***Hypomicrogasterinversalis* Valerio, 2015**

*Hypomicrogasterinversalis* Valerio, 2015.

**Type information.** Holotype male, CNC (examined). Country of type locality: Argentina.

**Geographical distribution.**NEO.

**NEO**: Argentina.

***Hypomicrogasterkoinos* Valerio, 2015**

*Hypomicrogasterkoinos* Valerio, 2015.

**Type information.** Holotype female, AEIC (not examined but original description checked). Country of type locality: Costa Rica.

**Geographical distribution.**NEA, NEO.

**NEA**: USA (MI); **NEO**: Brazil (PA, RJ), Costa Rica, Colombia, Ecuador, Mexico, Trinidad & Tobago, Venezuela.

***Hypomicrogasterlarga* Valerio, 2015**

*Hypomicrogasterlargus* Valerio, 2015.

**Type information.** Holotype female, CNC (examined). Country of type locality: Ecuador.

**Geographical distribution.**NEA, NEO.

**NEA**: Canada (ON), USA (OH); **NEO**: Argentina, Belize, Brazil (MT, PR, SP), Cayman Islands, Colombia, Costa Rica, Dominican Republic, Ecuador, Guatemala, Mexico, Panama.

**Notes.**Valerio and Whitfield (2015) stated that the name was adjectival; it therefore must change spelling in compliance with ICZN Article 31.2.

***Hypomicrogasterlaxa* Valerio & Mason, 2015**

*Hypomicrogasterlaxus* Valerio & Mason, 2015.

**Type information.** Holotype female, AEIC (not examined but original description checked). Country of type locality: USA.

**Geographical distribution.**NEA.

**NEA**: Canada (ON), USA (KS, TX).

**Notes.**Valerio and Whitfield (2015) stated that the name was adjectival; it therefore must change spelling in compliance with ICZN Article 31.2.

***Hypomicrogasterlinearis* Valerio, 2015**

*Hypomicrogasterlinearis* Valerio, 2015.

**Type information.** Holotype female, INBio (not examined but original description checked). Country of type locality: Costa Rica.

**Geographical distribution.**NEO.

**NEO**: Costa Rica.

***Hypomicrogasterlineata* Valerio, 2015**

*Hypomicrogasterlineatus* Valerio, 2015.

**Type information.** Holotype female, USNM (not examined but original description checked). Country of type locality: USA.

**Geographical distribution.**NEA.

**NEA**: USA (NY, VA).

**Notes.**Valerio and Whitfield (2015) stated that the name was adjectival; it therefore must change spelling in compliance with ICZN Article 31.2.

***Hypomicrogasterluisi* Valerio, 2015**

*Hypomicrogasterluisi* Valerio, 2015.

**Type information.** Holotype female, MCZC (not examined but original description checked). Country of type locality: Argentina.

**Geographical distribution.**NEO.

**NEO**: Argentina, Brazil (MT), Colombia, Costa Rica, Ecuador, Mexico, Peru.

***Hypomicrogastermasoni* Valerio, 2015**

*Hypomicrogastermasoni* Valerio, 2015.

**Type information.** Holotype female, CNC (examined). Country of type locality: Brazil.

**Geographical distribution.**NEO.

**NEO**: Brazil (RJ, SP).

***Hypomicrogastermesos* Valerio, 2015**

*Hypomicrogastermesos* Valerio, 2015.

**Type information.** Holotype female, CNC (examined). Country of type locality: Brazil.

**Geographical distribution.**NEO.

**NEO**: Brazil (SC).

***Hypomicrogastermikrosus* Valerio, 2015**

*Hypomicrogastermikrosus* Valerio, 2015.

**Type information.** Holotype female, USNM (not examined but original description checked). Country of type locality: Argentina.

**Geographical distribution.**NEO.

**NEO**: Argentina.

**Notes.**Valerio and Whitfield (2015) stated that the name was adjectival, but this is not an actual Greek adjective; it therefore must be treated as indeclinable under ICZN Article 31.2.3.

***Hypomicrogastermulta* Valerio, 2015**

*Hypomicrogastermultus* Valerio, 2015.

**Type information.** Holotype female, CNC (examined). Country of type locality: Brazil.

**Geographical distribution.**NEO.

**NEO**: Argentina, Brazil (RJ), Venezuela.

**Notes.**Valerio and Whitfield (2015) stated that the name was adjectival; it therefore must change spelling in compliance with ICZN Article 31.2.

***Hypomicrogasterpablouzagai* (Fernandez-Triana & Boudreault, 2016)**

*Promicrogasterpablouzagai* Fernandez-Triana & Boudreault, 2016.

**Type information.** Holotype female, CNC (examined). Country of type locality: Costa Rica.

**Geographical distribution.**NEO.

**NEO**: Costa Rica.

***Hypomicrogasterpectinata* Valerio, 2015**

*Hypomicrogasterpectinatus* Valerio, 2015.

**Type information.** Holotype male, CNC (examined). Country of type locality: Bolivia.

**Geographical distribution.**NEO.

**NEO**: Bolivia.

**Notes.**Valerio and Whitfield (2015) stated that the name was adjectival; it therefore must change spelling in compliance with ICZN Article 31.2.

***Hypomicrogasterplagios* Valerio, 2015**

*Hypomicrogasterplagios* Valerio, 2015.

**Type information.** Holotype female, INBio (not examined but original description checked). Country of type locality: Costa Rica.

**Geographical distribution.**NEO.

**NEO**: Costa Rica.

***Hypomicrogasterpollex* Valerio, 2015**

*Hypomicrogasterpollex* Valerio, 2015.

**Type information.** Holotype female, IAVH (not examined but original description checked). Country of type locality: Colombia.

**Geographical distribution.**NEO.

**NEO**: Colombia, Ecuador.

***Hypomicrogasterrugosa* Valerio, 2015**

*Hypomicrogasterrugosus* Valerio, 2015.

**Type information.** Holotype female, INBio (not examined but original description checked). Country of type locality: Costa Rica.

**Geographical distribution.**NEO.

**NEO**: Bolivia, Brazil (RO), Colombia, Costa Rica, Ecuador, Mexico, Panama, Peru.

**Notes.**Valerio and Whitfield (2015) stated that the name was adjectival; it therefore must change spelling in compliance with ICZN Article 31.2.

***Hypomicrogastersamarshalli* (Fernandez-Triana, 2010), new combination**

*Apantelessamarshalli* Fernandez-Triana, 2010.

**Type information.** Holotype female, CNC (examined). Country of type locality: USA.

**Geographical distribution.**NEA, NEO.

**NEA**: Canada (ON), USA (FL); **NEO**: Costa Rica, Mexico.

**Notes.** A critical re-examination of the many available specimens (including the holotype), as well as the numerous DNA barcodes available, clearly indicates that this species is better placed within *Hypomicrogaster*. Among the main morphological characters that suggest so, the propodeum has an irregular pattern of carinae radiating from the nucha, as well as coarse sculpture (over most of propodeum), which have been observed in other species of *Hypomicrogaster*; also, the fore wing venation suggests a very small (basically obliterated) areolet, which would clearly exclude the species from *Apanteles*. The DNA barcodes cluster with many species of *Hypomicrogaster* and relatively far from other species of *Apanteles*, further supporting the decision to transfer the species.

***Hypomicrogasterscindus* Valerio, 2015**

*Hypomicrogasterscindus* Valerio, 2015.

**Type information.** Holotype female, CNC (examined). Country of type locality: Brazil.

**Geographical distribution.**NEO.

**NEO**: Brazil (RJ).

**Notes.**Valerio and Whitfield (2015) stated that the name was adjectival, but this is not an actual Latin adjective; it therefore must be treated as indeclinable under ICZN Article 31.2.3.

***Hypomicrogastersicingens* Valerio, 2015**

*Hypomicrogastersicingens* Valerio, 2015.

**Type information.** Holotype female, CNC (examined). Country of type locality: Brazil.

**Geographical distribution.**NEO.

**NEO**: Brazil (RJ).

***Hypomicrogastersicpollex* Valerio, 2015**

*Hypomicrogastersicpollex* Valerio, 2015.

**Type information.** Holotype female, CNC (examined). Country of type locality: Mexico.

**Geographical distribution.**NEO.

**NEO**: Mexico.

***Hypomicrogastersicscindus* Valerio, 2015**

*Hypomicrogastersicscindus* Valerio, 2015.

**Type information.** Holotype female, AEIC (not examined but original description checked). Country of type locality: Brazil.

**Geographical distribution.**NEO.

**NEO**: Brazil (RJ).

**Notes.**Valerio and Whitfield (2015) stated that the name was adjectival, but this is not an actual Latin adjective; it therefore must be treated as indeclinable under ICZN Article 31.2.3.

***Hypomicrogastersiderion* Valerio, 2015**

*Hypomicrogastersiderion* Valerio, 2015.

**Type information.** Holotype female, CNC (examined). Country of type locality: Ecuador.

**Geographical distribution.**NEO.

**NEO**: Ecuador.

***Hypomicrogasterspatulae* Valerio, 2015**

*Hypomicrogasterspatulae* Valerio, 2015.

**Type information.** Holotype female, CNC (examined). Country of type locality: Brazil.

**Geographical distribution.**NEO.

**NEO**: Brazil (AM, PE, RO), Ecuador.

***Hypomicrogasterspecialis* Valerio, 2015**

*Hypomicrogasterspecialis* Valerio, 2015.

**Type information.** Holotype female, MCZC (not examined but original description checked). Country of type locality: Brazil.

**Geographical distribution.**NEO.

**NEO**: Bolivia, Brazil (AM, DF), Colombia, Costa Rica, Ecuador, Panama, Paraguay.

***Hypomicrogastertantilla* Valerio, 2015**

*Hypomicrogastertantillus* Valerio, 2015.

**Type information.** Holotype female, CNC (examined). Country of type locality: Brazil.

**Geographical distribution.**NEO.

**NEO**: Argentina, Brazil (BA, RJ).

**Notes.**Valerio and Whitfield (2015) stated that the name was adjectival; it therefore must change spelling in compliance with ICZN Article 31.2.

***Hypomicrogastertetra* Valerio, 2015**

*Hypomicrogastertetra* Valerio, 2015.

**Type information.** Holotype female, IAVH (not examined but original description checked). Country of type locality: Colombia.

**Geographical distribution.**NEO.

**NEO**: Colombia.

***Hypomicrogastertydeus* Nixon, 1965**

*Hypomicrogastertydeus* Nixon, 1965.

**Type information.** Holotype female, NHMUK (examined). Country of type locality: Brazil.

**Geographical distribution.**NEO.

**NEO**: Brazil (SC).

**Notes.** See comments under *H.acarnas* for a justification to consider both species as separate, including morphological details.

***Hypomicrogasterzan* Valerio, 2015**

*Hypomicrogasterzan* Valerio, 2015.

**Type information.** Holotype female, INBio (not examined but original description checked). Country of type locality: Costa Rica.

**Geographical distribution.**NEO.

**NEO**: Brazil (SC), Costa Rica.

***Hypomicrogasterzonaria* (Say, 1836)**

*Microgasterzonaria* Say, 1836.

*Microgastercincta* Provancher, 1881.

*Protapantelesrecurvariae* Ashmead, 1903.

*Hypomicrogasterecdytolophae* Muesebeck, 1922.

*Hypomicrogasterjocarae* Muesebeck, 1958.

*Hypomicrogasterhypsipylae* de Santis, 1972.

**Type information.** Type lost (not examined but subsequent treatment of the species checked). Country of type locality: USA.

**Geographical distribution.**NEA, NEO.

**NEA**: Canada (NB, NS, ON, QC), USA (AR, CO, CT, DE, DC, FL, IL, IN, IA, KS, KY, LA, MD, MA, MO, NE, NH, NJ, NY, OH, OK, PA, TX, VA, WV, WI); **NEO**: Costa Rica, Cuba, Guatemala, Puerto Rico.

**Notes.**Valerio and Whitfield (2015: 31) mentioned the species names *Protapantelesrecurviriae* Ashmead, 1903 and *Microgasterrecurvitae* (Ashmead) Muesebeck, 1920 as associated names to *H.zonaria* but both are typographical errors of *recurvariae* (and the correct year for the Muesebeck citation is 1921). In any case, *H.zonaria* (*sensu*Valerio and Whitfield 2015) seems to comprise a large assemblage of species dumped altogether, but DNA and host records strongly suggest they may represent several distinct species. However, resolution of this is beyond the scope of this paper.

#### Genus Iconella Mason, 1981

***Iconella*** Mason, 1981: 74. Gender: feminine. Type species: *Apantelesetiellae* Viereck, 1911, by original designation.

A cosmopolitan genus, with 38 described species known from all biogeographical regions except Australasian. There are revisions available for China (Chen and Song 2004), the Palaearctic (Kotenko 2007b), and the New World (Fernandez-Triana et al. 2013a), but we have seen in collections additional species, mostly from tropical areas. The genus may be split into several following more studies on the phylogeny of Microgastrinae (especially the species from the Old World tropics). The concept of *Iconella* and its separation from *Apanteles* has been controversial (e.g., Mason 1981, van Achterberg 2003, Fernandez-Triana et al. 2014e), but we consider it as a valid genus. Host data include mostly Crambidae and Pyralidae, with a couple of records from Tortricidae. There are 49 DNA-barcode compliant sequences of *Iconella* in BOLD, representing 12 BINs.

***Iconellaaeolus* (Nixon, 1965)**

*Apantelesaeolus* Nixon, 1965.

**Type information.** Holotype female, NHMUK (examined). Country of type locality: Germany.

**Geographical distribution.**PAL.

**PAL**: Armenia, Germany, Russia (MOS), Turkey, Ukraine, United Kingdom.

**Notes.** Because the name is to be considered as a noun under ICZN Article 31.2.1, it must retain its original spelling and remain as *aeolus*.

***Iconellaalbinervis* (Tobias, 1964)**

*Apantelesalbinervis* Tobias, 1964.

**Type information.** Holotype female, ZIN (not examined but subsequent treatment of the species checked). Country of type locality: Kazakhstan.

**Geographical distribution.**PAL.

**PAL**: Azerbaijan, Hungary, Kazakhstan, Moldova, Russia (S), Turkey, Ukraine.

**Notes.** Our species concept is based on Papp (1982), Tobias (1986), Kotenko (2007b).

***Iconellaalfalfae* (Nixon, 1960)**

*Apantelesalfalfae* Nixon, 1960.

**Type information.** Holotype female, NHMUK (examined). Country of type locality: Australia.

**Geographical distribution.**AUS.

**AUS**: Australia (SA).

**Notes.** This species was transferred to *Iconella* by Mason (1981), and it was also considered to belong to that genus by Austin and Dangerfield (1992). However, Yu et al. (2016) treated it as an *Apanteles*. After examining the female holotype, we agree it belongs to *Iconella*, and for the sake of clarity we revise its combination here.

***Iconellaandydeansi* Fernandez-Triana, 2013**

*Iconellaandydeansi* Fernandez-Triana, 2013.

**Type information.** Holotype female, CNC (examined). Country of type locality: Costa Rica.

**Geographical distribution.**NEO.

**NEO**: Costa Rica.

***Iconellaargante* (Nixon, 1976)**

*Apantelesargante* Nixon, 1976.

**Type information.** Holotype female, MZH (not examined but original description checked). Country of type locality: Finland.

**Geographical distribution.**PAL.

**PAL**: Finland, Kazakhstan, Russia (PRI), Ukraine.

***Iconellaassabensis* (Shenefelt, 1972)**

*Apantelesassabensis* Shenefelt, 1972.

*Apanteleslacteipennis* Szépligeti, 1913 [secondary homonym of *Apanteleslacteipennis* Curtis, 1830].

**Type information.** Lectotype female, HNHM (not examined but subsequent treatment of the species checked). Country of type locality: Eritrea.

**Geographical distribution.**AFR.

**AFR**: Eritrea, Tanzania.

**Notes.** Our species concept is based on Papp (2004).

***Iconellacajani* (Wilkinson, 1928), new combination**

*Apantelescajani* Wilkinson, 1928.

**Type information.** Holotype female, NHMUK (examined). Country of type locality: India.

**Geographical distribution.**OTL.

**OTL**: India.

**Notes.** This species is placed in *Iconella* based on the propodeum having a complete median, longitudinal carina; the scutellar lunules maximum height being more than 0.7 x the maximum height of the lateral face of the scutellum, and the hind wing having a sinuous vein cu-a.

***Iconellacanadensis* Fernandez-Triana, 2013**

*Iconellacanadensis* Fernandez-Triana, 2013.

**Type information.** Holotype female, CNC (examined). Country of type locality: Canada.

**Geographical distribution.**NEA.

**NEA**: Canada (NB, ON, QC).

**Notes.**Fernandez-Triana et al. (2013a) considered that a record of *Iconella* from Virginia, USA (reported in Yu et al. 2012) probably belongs to *I.canadensis*, but specimen examination is needed to conclude.

***Iconellacompressiabdominis* (You & Tong, 1991)**

*Apantelescompressiabdominis* You & Tong, 1991.

**Type information.** Holotype female, HUNAU (not examined but subsequent treatment of the species checked). Country of type locality: China.

**Geographical distribution.**OTL.

**OTL**: China (HN).

**Notes.** Our species concept is based on Chen and Song (2004) and Kotenko (2007b).

***Iconelladetrectans* (Wilkinson, 1928), new combination**

*Apantelesdetrectans* Wilkinson, 1928.

**Type information.** Holotype female, NHMUK (examined). Country of type locality: India.

**Geographical distribution.**AFR, OTL.

**AFR**: Sudan; **OTL**: India.

**Notes.** This species is placed in *Iconella* based on the propodeum having a complete median, longitudinal carina; the scutellar lunules maximum height being more than 0.7 x the maximum height of the lateral face of the scutellum, and the hind wing having a sinuous vein cu-a.

***Iconellaetiellae* (Viereck, 1911)**

*Apantelesetiellae* Viereck, 1911.

*Apantelesiselyi* Cushman, 1919.

**Type information.** Holotype male, USNM (examined). Country of type locality: USA.

**Geographical distribution.**NEA, NEO.

**NEA**: USA (AZ, AR, CA, CO, IA, KS, NM, OK, TX, UT, VA, WA); **NEO**: Mexico.

**Notes.** The record of this species from Mexico (Muesebeck 1958, Coronado-Blanco et al. 2004) probably refers to a different species, but specimen examination is needed to conclude.

***Iconellafedtschenkoi* (Kotenko, 1986)**

*Apantelesfedtschenkoi* Kotenko, 1986.

**Type information.** Type and depository unknown (not examined but original description checked). Country of type locality: Uzbekistan.

**Geographical distribution.**PAL.

**PAL**: Uzbekistan.

***Iconellainula* Papp, 2012**

*Iconellainula* Papp, 2012.

**Type information.** Holotype female, HNHM (not examined but original description checked). Country of type locality: Cape Verde.

**Geographical distribution.**AFR.

**AFR**: Cape Verde.

***Iconellaisolata* (Muesebeck, 1955)**

*Apantelesetiellae* Muesebeck, 1955.

*Apantelesetiellaeisolatus* Muesebeck, 1955.

**Type information.** Holotype female, USNM (not examined but subsequent treatment of the species checked). Country of type locality: Trinidad & Tobago.

**Geographical distribution.**NEO.

**NEO**: British Virgin Islands, Cayman Islands, Dominica, Grenada, Guyana, Montserrat, Puerto Rico, Saint Kitts & Nevis, Trinidad & Tobago.

**Notes.** Our species concept and geographical distribution is based on Fernandez-Triana et al. (2013a).

***Iconellaisus* (Nixon, 1965)**

*Apantelesisus* Nixon, 1965.

**Type information.** Holotype female, NHMUK (examined). Country of type locality: Hungary.

**Geographical distribution.**PAL.

**PAL**: Armenia, Hungary, Iran, Israel, Kazakhstan, Russia (C, S), Serbia, Spain, Uzbekistan.

**Notes.** The species distribution in Iran, Israel, Kazakhstan and Russia is based on Belokobylskij et al. (2019).

***Iconellajason* (Nixon, 1965), new combination**

*Apantelesjason* Nixon, 1965.

**Type information.** Holotype female, NHMUK (examined). Country of type locality: Malaysia.

**Geographical distribution.**OTL.

**OTL**: Indonesia, Malaysia.

**Notes.** Transferred to *Iconella* based on the well defined, strong median carina.

***Iconellajayjayrodriguezae* Fernandez-Triana, 2013**

*Iconellajayjayrodriguezae* Fernandez-Triana, 2013.

**Type information.** Holotype female, USNM (examined). Country of type locality: Costa Rica.

**Geographical distribution.**NEO.

**NEO**: Costa Rica, Mexico.

***Iconellalacteoides* (Nixon, 1965)**

*Apanteleslacteoides* Nixon, 1965.

*Apantelesmemorabilis* Alexeev, 1971.

**Type information.** Holotype female, NHRS (not examined but original description checked). Country of type locality: Sweden.

**Geographical distribution.**PAL.

**PAL**: Armenia, Azerbaijan, Germany, Greece, Hungary, Italy, Kazakhstan, Mongolia, Poland, Russia (PRI, ROS), Slovakia, Sweden, Turkey, Turkmenistan, Ukraine, Uzbekistan.

***Iconellalynceus* (Nixon, 1965), new combination**

*Apanteleslynceus* Nixon, 1965.

**Type information.** Holotype female, NHMUK (examined). Country of type locality: South Africa.

**Geographical distribution.**AFR.

**AFR**: South Africa.

**Notes.** Transferred to *Iconella* based on the well defined, strong median carina, with some smaller striae radiating from it.

***Iconellamasallensis* (Abdinbekova, 1969)**

*Apantelesmasallensis* Abdinbekova, 1969.

**Type information.** Holotype female, ZIN (not examined but subsequent treatment of the species checked). Country of type locality: Azerbaijan.

**Geographical distribution.**PAL.

**PAL**: Azerbaijan, Tajikistan.

**Notes.** Our species concept is based on Kotenko (1981), Papp (1982) and Tobias (1986).

***Iconellamemorata* Kotenko, 2007**

*Iconellamemorata* Kotenko, 2007.

**Type information.** Holotype female, SIZK (not examined but original description checked). Country of type locality: Russia.

**Geographical distribution.**PAL.

**PAL**: Russia (PRI).

***Iconellamera* (Kotenko, 1992)**

*Apantelesmerus* Kotenko, 1992.

**Type information.** Holotype female, SIZK (not examined but original description checked). Country of type locality: Russia.

**Geographical distribution.**PAL.

**PAL**: Russia (ZAB).

**Notes.** Our species concept is based on Kotenko (1992, 2007). The species name must be treated as an adjective and not as a noun (Doug Yanega, pers. comm.) and thus it must match the gender of the genus name.

***Iconellamerata* (Kotenko, 1981)**

*Apantelesmeratus* Kotenko, 1981.

**Type information.** Holotype female, SIZK (not examined but original description checked). Country of type locality: Ukraine.

**Geographical distribution.**PAL.

**PAL**: Russia (S), Ukraine.

***Iconellamerula* (Reinhard, 1880)**

*Apantelesmerula* Reinhard, 1880.

**Type information.** Holotype female, ZMHB (not examined but subsequent treatment of the species checked). Country of type locality: Germany.

**Geographical distribution.**PAL.

**PAL**: Austria, Belgium, Bulgaria, Finland, Germany, Hungary, Israel, Poland, Romania, Russia (S), Slovakia, Turkey, Ukraine.

**Notes.** Our species concept is based on Nixon (1968, 1976), Kotenko (1981), Papp (1982), and Tobias (1986). The species distribution in Israel is based on Belokobylskij et al. (2019).

***Iconellameruloides* (Nixon, 1965)**

*Apantelesmeruloides* Nixon, 1965.

**Type information.** Holotype female, NHMUK (examined). Country of type locality: Turkey.

**Geographical distribution.**PAL.

**PAL**: Hungary, Iran, Israel, Jordan, Malta, Romania, Turkey.

***Iconellamongashtensis* Zargar & Gupta, 2019**

*Iconellamongashtensis* Zargar & Gupta, 2019.

**Type information.** Holotype female, TMUC (not examined but original description checked). Country of type locality: Iran.

**Geographical distribution.**PAL.

**PAL**: Iran.

***Iconellamyeloenta* (Wilkinson, 1937)**

*Apantelesmyeloenta* Wilkinson, 1937.

**Type information.** Holotype female, NHMUK (examined). Country of type locality: Cyprus.

**Geographical distribution.**PAL.

**PAL**: Cyprus, Greece, Iran, Israel, Moldova, Russia (NC, S), Spain, Tunisia, Turkey, Turkmenistan.

**Notes.** The holotype is missing its head, but otherwise is in good condition.

***Iconellanagyi* (Papp, 1975)**

*Apantelesnagyi* Papp, 1975.

**Type information.** Holotype female, HNHM (not examined but original description checked). Country of type locality: Romania.

**Geographical distribution.**PAL.

**PAL**: Romania.

**Notes.** We suspect this species does not belong to *Iconella*, as it does not have a median longitudinal carinae on the propodeum, one of the main defining characters of the genus. Examination of specimens will be needed to conclude on that.

***Iconellaoppugnator* (Papp, 1974)**

*Apantelesoppugnator* Papp, 1974.

**Type information.** Holotype female, HNHM (not examined but original description checked). Country of type locality: Korea.

**Geographical distribution.**PAL.

**PAL**: Korea.

***Iconellapyrene* (Nixon, 1965), new combination**

*Apantelespyrene* Nixon, 1965.

**Type information.** Holotype female, NHMUK (examined). Country of type locality: South Africa.

**Geographical distribution.**AFR.

**AFR**: South Africa.

**Notes.** Transferred to *Iconella* based on the well-defined, strong, median carina on the propodeum.

***Iconellarudolphae* (Kotenko, 1986)**

*Apantelesrudolphae* Kotenko, 1986.

**Type information.** Type and depository unknown (not examined but original description checked). Country of type locality: Kazakhstan.

**Geographical distribution.**PAL.

**PAL**: Kazakhstan, Russia (S).

***Iconellasimilus* Zargar & Gupta, 2019**

*Iconellasimilus* Zargar & Gupta, 2019.

**Type information.** Holotype female, TMUC (not examined but original description checked). Country of type locality: Iran.

**Geographical distribution.**PAL.

**PAL**: Iran.

***Iconellasubcamilla* (Tobias, 1976)**

*Apantelessubcamilla* Tobias, 1976.

**Type information.** Holotype female, ZIN (not examined but subsequent treatment of the species checked). Country of type locality: Azerbaijan.

**Geographical distribution.**PAL.

**PAL**: Azerbaijan, Cape Verde, Iran, Israel.

**Notes.** Our species concept is based on Tobias (1986) and Kotenko (1981, 2007).

***Iconellatedanius* (Nixon, 1965), new combination**

*Apantelestedanius* Nixon, 1965.

**Type information.** Holotype female, USNM (examined). Country of type locality: Philippines.

**Geographical distribution.**OTL.

**OTL**: Philippines.

**Notes.** Transferred to *Iconella* based on the propodeum with a complete median carina. Also, Nixon (1965) placed the species within the *merula* species group, which comprises other *Iconella* species.

***Iconellaturanica* (Telenga, 1955)**

*Apantelesturanicus* Telenga, 1955.

*Apantelessubtilis* Alexeev, 1971.

**Type information.** Holotype female, depository unknown (not examined but subsequent treatment of the species checked). Country of type locality: unknown.

**Geographical distribution.**PAL.

**PAL**: Tajikistan, Turkmenistan.

**Notes.** Our species concept is based on Telenga (1955), Papp (1982) and Tobias (1986).

***Iconellavaliko* Kotenko, 2007**

*Iconellavaliko* Kotenko, 2007.

**Type information.** Holotype female, SIZK (not examined but original description checked). Country of type locality: Kyrgyzstan.

**Geographical distribution.**PAL.

**PAL**: Kyrgyzstan.

***Iconellaverae* (Tobias, 1976)**

*Apantelesverae* Tobias, 1976.

**Type information.** Holotype female, ZIN (not examined but subsequent treatment of the species checked). Country of type locality: Armenia.

**Geographical distribution.**PAL.

**PAL**: Armenia.

**Notes.** Our species concept is based on Papp (1984a), Tobias (1986) and Kotenko (2007b).

***Iconellavindicius* (Nixon, 1965)**

*Apantelesvindicius* Nixon, 1965.

**Type information.** Holotype female, NHMUK (examined). Country of type locality: Italy.

**Geographical distribution.**PAL.

**PAL**: Bulgaria, Georgia, Hungary, Italy, Korea, Russia (ZAB, DA, PRI), Turkey, Ukraine.

**Notes.** Because the name is to be considered as a noun under ICZN Article 31.2.1, it must retain its original spelling and remain as *vindicius*.

#### Genus Illidops Mason, 1981

***Illidops*** Mason, 1981: 56. Gender: masculine. Type species: *Apantelesbutalidis* Marshall, 1889, by original designation.

A cosmopolitan genus, with 37 described species known from all biogeographical regions except Australasian (one species has been introduced to Hawaii). A few species from the Neotropical region, India, and Russia Far East have been keyed out (Penteado-Dias et al. 2000, Ahmad et al. 2005a, Kotenko 2007a), but we have seen in collections many additional species, from both temperate and tropical areas. The concept of *Illidops* and its separation from *Apanteles* has been controversial (e.g., Mason 1981, van Achterberg 2003, Fernandez-Triana et al. 2014e), but we consider it a valid genus. Host data include the families Gelechiidae and Scythrididae, but they may need verification. There are 112 DNA-barcode compliant sequences of this genus in BOLD, representing 12 BINs.

***Illidopsalbostigmalis* van Achterberg & Fernandez-Triana, 2017**

*Illidopsalbostigmalis* van Achterberg & Fernandez-Triana, 2017.

**Type information.** Holotype female, RMNH (examined). Country of type locality: Yemen.

**Geographical distribution.**AFR.

**AFR**: United Arab Emirates, Yemen.

***Illidopsaridus* Penteado-Dias & Scatolini, 2000**

*Illidopsaridus* Penteado-Dias & Scatolini, 2000.

**Type information.** Holotype female, DCBU (not examined but original description checked). Country of type locality: Brazil.

**Geographical distribution.**NEO.

**NEO**: Brazil (SP).

***Illidopsassimilis* (Papp, 1976)**

*Apantelesassimilis* Papp, 1976.

**Type information.** Holotype female, HNHM (not examined but original description checked). Country of type locality: Mongolia.

**Geographical distribution.**PAL.

**PAL**: Mongolia.

***Illidopsazamgarhensis* (Ahmad, 2005), new combination**

*Apantelesazamgarhensis* Ahmad, 2005.

**Type information.** Holotype female, AMUZ (not examined but original description checked). Country of type locality: India.

**Geographical distribution.**OTL.

**OTL**: India.

**Notes.** This species was described as Apanteles (Illidops) azamgarhensis, as the authors of the paper considered *Illidops* to be a subgenus within *Apanteles* (Ahmad et al. 2005: 229). As far as we know, no other publication has dealt with this species, except for Taxapad, which last two versions treated *Illidops* as a synonym (Yu et al. 2012) or as a subgenus of *Apanteles* (Yu et al. 2016). Thus, until now all available references had placed this species within *Apanteles*. In the original description, the presence or absence of a postero-median band of rugosity on the scutellar disc is not discussed, but the details of the propodeum sculpture, metasoma and fore wing venation seem to suggest that this species belongs to *Illidops*, thus the new combination is here proposed.

***Illidopsbarcinonensis* (Marshall, 1898)**

*Apantelesbarcinonensis* Marshall, 1898.

*Apantelesrhamphus* Marshall, 1898.

**Type information.** Lectotype female, MNCN (not examined but subsequent treatment of the species checked). Country of type locality: Spain.

**Geographical distribution.**PAL.

**PAL**: Spain.

**Notes.** Our species concept is based on Papp (1986, 1988).

***Illidopsbellicosus* (Papp, 1977)**

*Apantelesbellicosus* Papp, 1977.

**Type information.** Holotype female, HNHM (not examined but original description checked). Country of type locality: Mongolia.

**Geographical distribution.**PAL.

**PAL**: Mongolia.

***Illidopsblandus* (Tobias & Kotenko, 1986)**

*Apantelesblandus* Tobias & Kotenko, 1986.

**Type information.** Holotype female, SIZK (not examined but original description checked). Country of type locality: Tajikistan.

**Geographical distribution.**PAL.

**PAL**: Tajikistan.

***Illidopsbutalidis* (Marshall, 1889)**

*Apantelesbutalidis* Marshall, 1889.

**Type information.** Holotype female, PCMAG (not examined but subsequent treatment of the species checked). Country of type locality: United Kingdom.

**Geographical distribution.**PAL.

**PAL**: Bulgaria, Croatia, Germany, Hungary, Mongolia, Romania, Russia (ZAB, PRI), Serbia, Slovakia, Spain, Sweden, Tunisia, Turkey, Ukraine, United Kingdom.

**Notes.** Our concept of this species is based on Wilkinson (1945), Nixon (1965, 1976), Papp (1981) and Kotenko (2007a).

***Illidopsbuteonis* (Kotenko, 1986)**

*Apantelesbuteonis* Kotenko, 1986.

**Type information.** Holotype female, SIZK (not examined but original description checked). Country of type locality: Ukraine.

**Geographical distribution.**PAL.

**PAL**: Russia (S), Ukraine.

***Illidopscloelia* (Nixon, 1965)**

*Apantelescloelia* Nixon, 1965.

**Type information.** Holotype female, NHMUK (examined). Country of type locality: Switzerland.

**Geographical distribution.**PAL.

**PAL**: Austria, Hungary, Korea, Russia (E, NC), Slovakia, Switzerland, Tajikistan, Yugoslavia.

**Notes.** The distribution in Tajikistan is based in Belokobylskij et al. (2019).

***Illidopsdauricus* Kotenko, 2007**

*Illidopsdauricus* Kotenko, 2007.

**Type information.** Holotype female, SIZK (not examined but original description checked). Country of type locality: Russia.

**Geographical distribution.**PAL.

**PAL**: Russia (ZAB).

***Illidopselectilis* (Tobias, 1964)**

*Apanteleselectilis* Tobias, 1964.

**Type information.** Holotype female, ZIN (not examined but subsequent treatment of the species checked). Country of type locality: Kazakhstan.

**Geographical distribution.**PAL.

**PAL**: Croatia, Hungary, Kazakhstan, Russia (S), Serbia, Tunisia.

**Notes.** Our species concept is based on Nixon (1976), Papp (1981) and Tobias (1986).

***Illidopskeralensis* (Narendran & Sumodan, 1992)**

*Chelonuskeralensis* Narendran & Sumodan, 1992.

**Type information.** Holotype female, HNHM (not examined but subsequent treatment of the species checked). Country of type locality: India.

**Geographical distribution.**OTL.

**OTL**: India.

**Notes.** Our species concept is based on van Achterberg and Narendran (1997).

***Illidopskostjuki* (Kotenko, 1986)**

*Apanteleskostjuki* Kotenko, 1986.

**Type information.** Holotype female, SIZK (not examined but original description checked). Country of type locality: Russia.

**Geographical distribution.**PAL.

**PAL**: Russia (ALT).

***Illidopskostylevi* (Kotenko, 1986)**

*Apanteleskostylevi* Kotenko, 1986.

**Type information.** Holotype female, SIZK (not examined but original description checked). Country of type locality: Ukraine.

**Geographical distribution.**PAL.

**PAL**: Russia (ROS), Ukraine.

***Illidopslamprosemae* (Ahmad, 2005), new combination**

*Apanteleslamprosemae* Ahmad, 2005.

*Apanteleslamprosemae* Ahmad, 2005 [primary junior homonym of *Apanteleslamprosemae* Wilkinson, 1928].

**Type information.** Holotype female, AMUZ (not examined but original description checked). Country of type locality: India.

**Geographical distribution.**OTL.

**OTL**: India.

**Notes.** This species was described as Apanteles (Illidops) lamprosemae Ahmad, 2005, as the authors of the paper considered *Illidops* to be a subgenus within *Apanteles* (Ahmad et al. 2005: 229). As far as we know, no other publication has dealt with this species, except for Taxapad, which last two versions treated *Illidops* as a synonym (Yu et al. 2012) or as a subgenus of *Apanteles* (Yu et al. 2016). Thus, until now all available references had placed this species within *Apanteles*. In the original description, the presence or absence of a postero-median band of rugosity on the scutellar disc is not discussed, but the details of the propodeum sculpture, metasoma and fore wing venation seem to suggest that this species belongs to *Illidops*, thus the new combination is here proposed.

***Illidopsmutabilis* (Telenga, 1955)**

*Apantelesmutabilis* Telenga, 1955.

*Apantelesszaboi* Papp, 1972.

**Type information.** Syntypes female and male, depository unknown (not examined but subsequent treatment of the species checked). Country of type locality: Ukraine.

**Geographical distribution.**PAL.

**PAL**: Austria, Bulgaria, Georgia, Hungary, Kazakhstan, Mongolia, Romania, Russia (KDA), Serbia, Slovakia, Spain, Tunisia, Turkey, Ukraine.

**Notes.** Our species concept is based on Papp (1981), Tobias (1986) and Kotenko (2007a).

***Illidopsnaso* (Marshall, 1885)**

*Apantelesnaso* Marshall, 1885.

*Apantelescontortus* Tobias, 1964.

*Apantelescrantor* Nixon, 1965.

*Apantelesevander* Nixon, 1965.

*Apantelescoresia* Nixon, 1973.

**Type information.** Holotype male, NHMUK (examined). Country of type locality: United Kingdom.

**Geographical distribution.**PAL.

**PAL**: Afghanistan, Armenia, Azerbaijan, Bulgaria, Croatia, Finland, Georgia, Greece, Hungary, Iran, Kazakhstan, Korea, Kyrgyzstan, Macedonia, Moldova, Mongolia, Romania, Russia (KC, VOR), Serbia, Slovakia, Switzerland, Turkey, Turkmenistan, United Kingdom, Uzbekistan.

**Notes.** The distribution in Turkmenistan is based in Belokobylskij et al. (2019).

***Illidopsnigritegula* (Tobias & Kotenko, 1986)**

*Apantelesnigritegula* Tobias & Kotenko, 1986.

**Type information.** Type and depository unknown (not examined but original description checked). Country of type locality: Kazakhstan.

**Geographical distribution.**PAL.

**PAL**: Kazakhstan, Russia (S).

***Illidopsparanaensis* Penteado-Dias & Scatolini, 2000**

*Illidopsparanaensis* Penteado-Dias & Scatolini, 2000.

**Type information.** Holotype female, DCMP (not examined but original description checked). Country of type locality: Brazil.

**Geographical distribution.**NEO.

**NEO**: Brazil (PR).

***Illidopsperseveratus* (Papp, 1977)**

*Apantelesperseveratus* Papp, 1977.

**Type information.** Holotype female, HNHM (not examined but original description checked). Country of type locality: Mongolia.

**Geographical distribution.**PAL.

**PAL**: Mongolia.

***Illidopsplaniscapus* (Tobias, 1976)**

*Apantelesplaniscapus* Tobias, 1976.

**Type information.** Holotype female, ZIN (not examined but subsequent treatment of the species checked). Country of type locality: Russia.

**Geographical distribution.**PAL.

**PAL**: Russia (DA).

**Notes.** Our species concept is based on Papp (1988) and Tobias (1988). Type depository inferred from Tobias (1986).

***Illidopsrostratus* (Tobias, 1976)**

*Apantelesrostratus* Tobias, 1976.

**Type information.** Holotype female, ZIN (not examined but subsequent treatment of the species checked). Country of type locality: Russia.

**Geographical distribution.**PAL.

**PAL**: Armenia, Russia (KDA), Uzbekistan.

**Notes.** Our species concept is based on Papp (1988) and Tobias (1988). Type depository inferred from Tobias (1986).

***Illidopsscutellaris* (Muesebeck, 1921)**

*Apantelesscutellaris* Muesebeck, 1921.

**Type information.** Holotype female, USNM (examined). Country of type locality: USA.

**Geographical distribution.**AUS, NEA, NEO, PAL.

**AUS**: Hawaiian Islands; **NEA**: USA (AZ, CA, FL, TX); **NEO**: Mexico; **PAL**: Bulgaria, Cyprus, Greece, Hungary, Iran.

***Illidopssophrosine* (Nixon, 1976)**

*Apantelessophrosine* Nixon, 1976.

**Type information.** Holotype female, NHMUK (examined). Country of type locality: Italy.

**Geographical distribution.**PAL.

**PAL**: Bulgaria, Hungary, Italy, Russia (ZAB, PRI).

***Illidopssplendidus* (Papp, 1974)**

*Apantelessplendidus* Papp, 1974.

**Type information.** Holotype female, HNHM (not examined but original description checked). Country of type locality: Hungary.

**Geographical distribution.**PAL.

**PAL**: Hungary, Russia (C).

***Illidopssubversor* (Tobias & Kotenko, 1986)**

*Apantelessubversor* Tobias & Kotenko, 1986.

**Type information.** Holotype female, SIZK (not examined but original description checked). Country of type locality: Russia.

**Geographical distribution.**PAL.

**PAL**: Russia (NVS).

***Illidopssuevus* (Reinhard, 1880)**

*Apantelessuevus* Reinhard, 1880.

*Apantelesminutus* Szépligeti, 1896.

*Apantelespolonicus* Fahringer, 1936.

*Apantelesbrevisternis* Tobias, 1964.

*Apantelessuspicax* Tobias, 1964.

*Apantelesdion* Nixon, 1965.

*Apantelessesostris* Nixon, 1976.

**Type information.** Holotype female, ZMHB (not examined but authoritatively identified specimens examined). Country of type locality: Germany.

**Geographical distribution.**PAL.

**PAL**: Armenia, Austria, Bulgaria, Croatia, Czech Republic, France, Germany, Greece, Hungary, Iran, Kazakhstan, Korea, Macedonia, Malta, Moldova, Mongolia, Montenegro, Poland, Romania, Russia (IRK), Serbia, Slovakia, Switzerland, United Kingdom.

**Notes.** We examined the type of *Apantelessesostris* Nixon. The species distribution in Iran is based in Belokobylskij et al. (2019).

***Illidopssuffectus* (Tobias & Kotenko, 1986)**

*Apantelessuffectus* Tobias & Kotenko, 1986.

**Type information.** Holotype female, ZIN (not examined but original description checked). Country of type locality: Kazakhstan.

**Geographical distribution.**PAL.

**PAL**: Kazakhstan.

***Illidopsterrestris* Wharton, 1983**

*Illidopsterrestris* Wharton, 1983.

**Type information.** Holotype female, USNM (examined). Country of type locality: USA.

**Geographical distribution.**NEA.

**NEA**: USA (CA, FL, GA, TX).

***Illidopstigris* (Kotenko, 1986)**

*Apantelestigris* Kotenko, 1986.

**Type information.** Holotype female, SIZK (not examined but original description checked). Country of type locality: Tajikistan.

**Geographical distribution.**PAL.

**PAL**: Tajikistan, Turkmenistan.

***Illidopstoreicus* Kotenko, 2007**

*Illidopstoreicus* Kotenko, 2007.

**Type information.** Holotype female, SIZK (not examined but original description checked). Country of type locality: Russia.

**Geographical distribution.**PAL.

**PAL**: Russia (ZAB).

***Illidopstrabea* (Nixon, 1965), new combination**

*Apantelestrabea* Nixon, 1965.

**Type information.** Holotype female, NHMUK (examined). Country of type locality: South Africa.

**Geographical distribution.**AFR.

**AFR**: South Africa.

**Notes.** The holotype has its eyes converging ventrally, scutellar disc with posteromedian band of rugosity, propodeum entirely strongly rugulose, and short vein R1 in the fore wing.

***Illidopsurgens* Kotenko, 2004**

*Illidopsurgens* Kotenko, 2004.

**Type information.** Holotype female, SIZK (not examined but subsequent treatment of the species checked). Country of type locality: Kazakhstan.

**Geographical distribution.**PAL.

**PAL**: Kazakhstan, Russia (SAR).

**Notes.** Our species concept is based on Kotenko (2006).

***Illidopsurgo* (Nixon, 1965)**

*Apantelesurgo* Nixon, 1965.

**Type information.** Holotype female, HNHM (not examined but original description checked). Country of type locality: Greece.

**Geographical distribution.**PAL.

**PAL**: Armenia, Azerbaijan, Croatia, Greece, Hungary, Iran, Mongolia, Russia (S), Slovakia, Turkey.

**Notes.** The species distribution in Armenia and Russia are based in Belokobylskij et al. (2019).

***Illidopsuvidus* Penteado-Dias & Scatolini, 2000**

*Illidopsuvidus* Penteado-Dias & Scatolini, 2000.

**Type information.** Holotype female, DCBU (not examined but original description checked). Country of type locality: Brazil.

**Geographical distribution.**NEO.

**NEO**: Brazil (PR, SP).

***Illidopsvitobiasi* Kotenko, 2004**

*Illidopsvitobiasi* Kotenko, 2004.

**Type information.** Holotype female, SIZK (not examined but subsequent treatment of the species checked). Country of type locality: Turkmenistan.

**Geographical distribution.**PAL.

**PAL**: Turkmenistan.

**Notes.** Our species concept is based on Kotenko (2006).

#### Genus Janhalacaste Fernandez-Triana, 2018

***Janhalacaste*** Fernandez-Triana, 2018: 59. Gender: neuter. Type species: *Janhalacastewinnieae* Fernandez-Triana & Boudreault, 2018, by original designation.

Known from three species recently described from the Neotropical region (Fernandez-Triana and Boudreault 2018). We are aware of at least one additional species in collections. All known host records are from Depressariidae. There are 12 DNA-barcode compliant sequences of *Janhalacaste* in BOLD, representing three BINs.

***Janhalacastedanieli* Fernandez-Triana & Boudreault, 2018**

*Janhalacastedanieli* Fernandez-Triana & Boudreault, 2018.

**Type information.** Holotype male, CNC (examined). Country of type locality: Costa Rica.

**Geographical distribution.**NEO.

**NEO**: Costa Rica.

***Janhalacasteguanacastensis* Fernandez-Triana & Boudreault, 2018**

*Janhalacasteguanacastensis* Fernandez-Triana & Boudreault, 2018.

**Type information.** Holotype female, CNC (examined). Country of type locality: Costa Rica.

**Geographical distribution.**NEO.

**NEO**: Costa Rica.

***Janhalacastewinnieae* Fernandez-Triana & Boudreault, 2018**

*Janhalacastewinnieae* Fernandez-Triana & Boudreault, 2018.

**Type information.** Holotype female, CNC (examined). Country of type locality: Costa Rica.

**Geographical distribution.**NEO.

**NEO**: Costa Rica.

#### Genus Jenopappius Fernandez-Triana, 2018

***Jenopappius*** Fernandez-Triana, 2018: 67. Gender: neuter. Type species: *Jenopappiusmagyarmuzeum*Fernandez-Triana and Boudreault 2018, by original designation.

Known from three species from the Afrotropical region, which were recently revised (Fernandez-Triana and Boudreault 2018). We are aware of additional species in collections. No host data are currently available for this genus. There are eleven DNA-barcode compliant sequences of *Jenopappius* in BOLD, representing one BIN (although those sequences have not been identified in BOLD as belonging to *Jenopappius*, see Fernandez-Triana and Boudreault 2018 for that).

***Jenopappiusaethiopicus* (de Saeger, 1944)**

*Microplitisaethiopicus* de Saeger, 1944.

**Type information.** Holotype female, RMCA (not examined but original description checked). Country of type locality: Democratic Republic of Congo.

**Geographical distribution.**AFR.

**AFR**: Democratic Republic of Congo, Kenya, Rwanda.

***Jenopappiusmagyarmuzeum* Fernandez-Triana & Boudreault, 2018**

*Jenopappiusmagyarmuzeum* Fernandez-Triana & Boudreault, 2018.

**Type information.** Holotype female, CNC (examined). Country of type locality: Democratic Republic of Congo.

**Geographical distribution.**AFR.

**AFR**: Democratic Republic of the Congo.

***Jenopappiusniger* (de Saeger, 1944)**

*Microplitisniger* de Saeger, 1944.

**Type information.** Holotype female, RMCA (not examined but original description checked). Country of type locality: Democratic Republic of Congo.

**Geographical distribution.**AFR.

**AFR**: Democratic Republic of Congo.

#### Genus Jimwhitfieldius Fernandez-Triana, 2018

***Jimwhitfieldius*** Fernandez-Triana, 2018: 75. Gender: neuter. Type species: *Jimwhitfieldiusjamesi*Fernandez-Triana and Boudreault 2018, by original designation.

Known from two species from the Oriental region, which were recently revised (Fernandez-Triana and Boudreault 2018). We are aware of additional species in collections. No host data are currently available for this genus. There are 19 DNA-barcode compliant sequences of *Jimwhitfieldius* in BOLD, representing five BINs (although those sequences have not been identified in BOLD as belonging to *Jimwhitfieldius*, see Fernandez-Triana and Boudreault 2018 for that).

***Jimwhitfieldiusjamesi* Fernandez-Triana & Boudreault, 2018**

*Jimwhitfieldiusjamesi* Fernandez-Triana & Boudreault, 2018.

**Type information.** Holotype female, QSBG (examined). Country of type locality: Thailand.

**Geographical distribution.**OTL.

**OTL**: Thailand, Vietnam.

***Jimwhitfieldiussydneyae* Fernandez-Triana & Boudreault, 2018**

*Jimwhitfieldiussydneyae* Fernandez-Triana & Boudreault, 2018.

**Type information.** Holotype female, QSBG (examined). Country of type locality: Thailand.

**Geographical distribution.**OTL.

**OTL**: Thailand.

#### Genus Keylimepie Fernandez-Triana, 2016

***Keylimepie*** Fernandez-Triana, 2016: 96. Gender: neuter. Type species: *Keylimepiepeckorum* Fernandez-Triana, 2016, by original designation.

Four species from the Nearctic and Afrotropical regions (Fernandez-Triana 2016, Fernandez-Triana & van Achterberg 2017). We have seen a few additional species in collections, including from the Neotropics, but the genus does not seem to be very speciose. The known species were collected in relatively hot and dry environments. No host data are currently available for this genus. There are no DNA-barcode compliant sequences of *Keylimepie* in BOLD, but the two African species have mini-barcodes of 276–278 bp.

***Keylimepiehadhramautensis* van Achterberg & Fernandez-Triana, 2017**

*Keylimepiehadhramautensis* van Achterberg & Fernandez-Triana, 2017.

**Type information.** Holotype female, RMNH (examined). Country of type locality: Yemen.

**Geographical distribution.**AFR.

**AFR**: Yemen.

***Keylimepiepeckorum* Fernandez-Triana, 2016**

*Keylimepiepeckorum* Fernandez-Triana, 2016.

**Type information.** Holotype female, CNC (examined). Country of type locality: USA.

**Geographical distribution.**NEA.

**NEA**: USA (FL).

***Keylimepiesanaaensis* van Achterberg & Fernandez-Triana, 2017**

*Keylimepiesanaaensis* van Achterberg & Fernandez-Triana, 2017.

**Type information.** Holotype female, RMNH (examined). Country of type locality: Yemen.

**Geographical distribution.**AFR.

**AFR**: Yemen.

***Keylimepiestriatus* (Muesebeck, 1922), new combination**

*Microplitisstriatus* Muesebeck, 1922.

**Type information.** Holotype male, USNM (examined). Country of type locality: USA.

**Geographical distribution.**NEA.

**NEA**: USA (IL, MI, TX).

**Notes.** Here we place this species in *Keylimepie*. The male holotype is not in great condition but fits well within the current genus concept (including shape and sculpture of the head, large tentorial pits, fore wing areolet, and T1 and T2 shapes and sculptures). In the USNM collection there are two other males of the genus, both identified as *M.striatus* by Muesebeck, but clearly representing different, undescribed species (with different venation patterns and body colouration from *striatus*).

#### Genus Kiwigaster Fernandez-Triana, Ward & Whitfield, 2011

***Kiwigaster*** Fernandez-Triana, Ward & Whitfield, 2011: 25. Gender: feminine. Type species: *Kiwigastervariabilis* Fernandez-Triana & Ward, 2011, by original designation.

Only known from a single, very unique species from the Australasian region (Fernandez-Triana et al. 2011). No host data are currently available for this genus. There is one DNA-barcode compliant sequence in BOLD, that BIN characterizing the genus and species. In the original description of *Kiwigaster*, its gender was incorrectly stated to be masculine (Fernandez-Triana et al. 2011: 25); however all genera ending in *gaster* are feminine, without exception (Doug Yanega, pers. comm., see also Article 30.1.2 of the ICZN); thus here we correct that previous mistake.

***Kiwigastervariabilis* Fernandez-Triana & Ward, 2011**

*Kiwigastervariabilis* Fernandez-Triana & Ward, 2011.

**Type information.** Holotype female, NZAC (examined). Country of type locality: New Zealand.

**Geographical distribution.**AUS.

**AUS**: New Zealand.

#### Genus Kotenkosius Fernandez-Triana, 2018

***Kotenkosius*** Fernandez-Triana, 2018: 84. Gender: neuter. Type species: *Kotenkosiustricarinatus*Fernandez-Triana and Boudreault 2018, by original designation.

Known from one recently described species from the Oriental region (Fernandez-Triana and Boudreault 2018). We are aware of at least one additional species in collections. No host data are currently available for this genus. There are at least three DNA-barcode compliant sequences of *Kotenkosius* in BOLD, representing one BIN, with another potential, undescribed species, having a BIN (see Fernandez-Triana and Boudreault 2018 for more details).

***Kotenkosiustricarinatus* Fernandez-Triana & Boudreault, 2018**

*Kotenkosiustricarinatus* Fernandez-Triana & Boudreault, 2018.

**Type information.** Holotype female, RMNH (examined). Country of type locality: Vietnam.

**Geographical distribution.**OTL.

**OTL**: Bangladesh, Malaysia, Taiwan, Thailand, Vietnam.

#### Genus Larissimus Nixon, 1965

***Larissimus*** Nixon, 1965: 204. Gender: masculine. Type species: *Larissimuscassander* Nixon, 1965, by original designation.

One described species from the Neotropical region (Nixon 1965, Mason 1981). We have seen in collections (CNC) a few additional species from South America, but the genus does not seem to be very speciose. The described species has been reared from Erebidae (Arctiinae). There is one DNA-barcode compliant sequence of this genus in BOLD, representing one BIN, which corresponds to the described species; additionally, there are seven shorter sequences from specimens which represent at least one other species.

***Larissimuscassander* Nixon, 1965**

*Larissimuscassander* Nixon, 1965.

**Type information.** Holotype female, NHMUK (examined). Country of type locality: Brazil.

**Geographical distribution.**NEO.

**NEO**: Brazil (SC, SP).

#### Genus Lathrapanteles Williams, 1985

***Lathrapanteles*** Williams, 1985: 1963. Gender: masculine. Type species: *Apantelespapaipemae* Muesebeck, 1921, by original designation.

This is a New World genus, with four species currently described from the Nearctic and Neotropical regions, which were revised by Williams (1985). We have seen a few additional species in collections (CNC), mostly from tropical areas, but *Lathrapanteles* does not seem to be very speciose. Host data include the family Noctuidae, with one record from Pyralidae. There are 41 DNA-barcode compliant sequences of this genus in BOLD, representing six BINs.

***Lathrapantelesampyx* Williams, 1985**

*Lathrapantelesampyx* Williams, 1985.

**Type information.** Holotype female, USNM (examined). Country of type locality: Colombia.

**Geographical distribution.**NEO.

**NEO**: Colombia, Peru.

***Lathrapantelesfuscus* Williams, 1985**

*Lathrapantelesfuscus* Williams, 1985.

**Type information.** Holotype female, CNC (examined). Country of type locality: Canada.

**Geographical distribution.**NEA.

**NEA**: Canada (BC, MB, NT, NS, QC), USA (CO, MN).

***Lathrapantelesheleios* Williams, 1985**

*Lathrapantelesheleios* Williams, 1985.

**Type information.** Holotype female, CNC (examined). Country of type locality: Canada.

**Geographical distribution.**NEA.

**NEA**: Canada (ON, QC).

**Notes.** The Canadian record from Quebec (Aylmer) is from Fernandez-Triana et al. (2016a), a paper where that specimen was wrongly reported to be from Ontario.

***Lathrapantelespapaipemae* (Muesebeck, 1921)**

*Apantelespapaipemae* Muesebeck, 1921.

**Type information.** Holotype female, USNM (examined). Country of type locality: USA.

**Geographical distribution.**NEA.

**NEA**: Canada (NL, ON, QC), USA (IL, IN, IA, KS, MA, MI, MO, NY, OH, OR).

#### Genus Mariapanteles Whitfield & Fernandez-Triana, 2012

***Mariapanteles*** Whitfield & Fernandez-Triana, 2012: 66. Gender: masculine. Type species: *Mariapantelesfelipei* Whitfield, 2012, by original designation.

This is a Neotropical genus with two species currently described (Whitfield et al. 2012). We have seen a few additional species in collections (CNC), mostly from tropical areas, but *Mariapanteles* does not seem to be very speciose. No host data are currently available for this genus. There are four DNA-barcode compliant sequences of this genus in BOLD, representing two BINs.

***Mariapantelesdapkeyae* Fernandez-Triana, 2012**

*Mariapantelesdapkeyae* Fernandez-Triana, 2012.

**Type information.** Holotype female, CNC (examined). Country of type locality: Brazil.

**Geographical distribution.**NEO.

**NEO**: Brazil (GO, MT).

***Mariapantelesfelipei* Whitfield, 2012**

*Mariapantelesfelipei* Whitfield, 2012.

**Type information.** Holotype female, USNM (examined). Country of type locality: Costa Rica.

**Geographical distribution.**NEO.

**NEO**: Costa Rica.

#### Genus Markshawius Fernandez-Triana, 2018

***Markshawius*** Fernandez-Triana, 2018: 88. Gender: neuter. Type species: *Markshawiuserucidoctus*Fernandez-Triana and Boudreault 2018, by original designation.

Known from three recently described species from the Oriental region (Fernandez-Triana and Boudreault 2018). We are aware of at least one additional species in collections. No host data are currently available for this genus. There is one DNA-barcode compliant sequence of *Markshawius* in BOLD, representing one BIN (although that sequence has not been identified in BOLD as belonging to *Markshawius*, see Fernandez-Triana and Boudreault 2018 for that).

***Markshawiuserucidoctus* Fernandez-Triana & Boudreault, 2018**

*Markshawiuserucidoctus* Fernandez-Triana & Boudreault, 2018.

**Type information.** Holotype female, RMNH (examined). Country of type locality: Vietnam.

**Geographical distribution.**OTL.

**OTL**: Vietnam.

***Markshawiusfrancescae* Fernandez-Triana & Boudreault, 2018**

*Markshawiusfrancescae* Fernandez-Triana & Boudreault, 2018.

**Type information.** Holotype female, QSBG (examined). Country of type locality: Thailand.

**Geographical distribution.**OTL.

**OTL**: Nepal, Thailand, Vietnam.

***Markshawiusthailandensis* Fernandez-Triana & Boudreault, 2018**

*Markshawiusthailandensis* Fernandez-Triana & Boudreault, 2018.

**Type information.** Holotype female, QSBG (examined). Country of type locality: Thailand.

**Geographical distribution.**OTL.

**OTL**: Thailand.

#### Genus Microgaster Latreille, 1804

***Microgaster*** Latreille, 1804: 175. Gender: feminine. Type species: Microgasteraustralis Thomson, 1895, by subsequent designation (ICZN 1988).

Liganira Walker, 1860: 308 [Name mentioned as previous error and suppressed as Microgaster (Shenefelt 1973: 694), see also Mason (1981: 80)]. Type species: Microgaster detractus Walker, 1860.

Lissogaster Bengtsson, 1926: 64. Type species: *Microgasterpolita*, Marshall, 1885, by subsequente designation (Muesebeck and Walkley 1951).

This was the first genus of Microgastrinae to be described and is the basis for the subfamily name. Until relatively recently, there was some confusion with the application of the name *Microgaster* and its type species (e.g., see van Achterberg 1982, Papp 1984c, Mason 1986, Tobias 1986, Whitfield 1987, Yu et al. 2012, 2016), which had the potential to complicate and confuse the treatment of many species used in biological control. Following van Achterberg’s (1982) examination of the lectotype of *Ichneumondeprimator* Fabricius, designated as the type species of *Microgaster*, which turned out to be a species of *Microplitis*, the generic name *Microgaster* was applied to what had been called *Microplitis*, and the junior synonym *Lissogaster* was brought into play for *Microgaster* auctt. Mason (1986) applied to ICZN and it was reversed by a 1988 ICZN Opinion (1510) by setting aside previous designations (i.e., *deprimator*) and making *Microgasteraustralis* Thomson the type species of *Microgaster* (which returned *Lissogaster* to synonymy under *Microgaster* and restored the traditional use of *Microplitis*). But, for a short period of time (1982–1988), the name *Lissogaster* was in legitimate use for *Microgaster*, and *Microgaster* for *Microplitis* (e.g., Papp 1984c). As currently understood, *Microgaster* is a cosmopolitan genus, with 104 described species. We have seen many additional species in collections, mostly from temperate areas. There are some revisions available for certain regions and/or countries, but most are outdated and even the most recent revisions do not take into account the hidden diversity that is revealed by DNA barcoding and biological data. Approximately 25 families of Lepidoptera have been recorded as hosts for *Microgaster*, but many records are likely to be incorrect and/or need further verification. There are 1,000+ DNA-barcode compliant sequences of this genus in BOLD, representing 67 BINs.

***Microgasteracilius* Nixon, 1968**

*Microgasteracilius* Nixon, 1968.

**Type information.** Holotype female, NHMUK (examined). Country of type locality: United Kingdom.

**Geographical distribution.**PAL.

**PAL**: United Kingdom.

**Notes.** Reinstated as a valid species by Shaw (2012b), a decision we agree with and follow here.

***Microgasteralbomarginata* Fahringer, 1935**

*Microgasteralbomarginata* Fahringer, 1935.

**Type information.** Holotype female, depository unknown (not examined but subsequent treatment of the species checked). Country of type locality: China.

**Geographical distribution.**OTL.

**OTL**: China (GZ, SN).

**Notes.** Our species concept is based on Xu and Han (2007).

***Microgasteralebion* Nixon, 1968**

*Microgasteralebion* Nixon, 1968.

**Type information.** Holotype female, NHMUK (examined). Country of type locality: United Kingdom.

**Geographical distribution.**PAL.

**PAL**: Czech Republic, Finland, Germany, Hungary, Italy, Poland, Romania, Russia (KR), Serbia, Switzerland, Turkey, United Kingdom.

***Microgasterarchboldensis* Fernandez-Triana, 2018**

*Microgasterarchboldensis* Fernandez-Triana, 2018.

**Type information.** Holotype female, CNC (examined). Country of type locality: USA.

**Geographical distribution.**NEA.

**NEA**: USA (FL).

***Microgasterarctostaphylica* Shaw, 2012**

*Microgasterarctostaphylica* Shaw, 2012.

**Type information.** Holotype female, RSME (examined). Country of type locality: United Kingdom.

**Geographical distribution.**PAL.

**PAL**: United Kingdom.

***Microgasterareolaris* Thomson, 1895**

*Microgasterareolaris* Thomson, 1895.

**Type information.** Type unknowm, MZLU (not examined but subsequent treatment of the species checked). Country of type locality: Sweden.

**Geographical distribution.**PAL.

**PAL**: Armenia, Bosnia and Herzegovina, Finland, Germany, Hungary, Ireland, Mongolia, Montenegro, Norway, Poland, Romania, Russia (STA), Sweden, Switzerland, Ukraine, United Kingdom.

**Notes.** Our species concept is based on Nixon (1968) and Papp (1976c).

***Microgasterasramenes* Nixon, 1968**

*Microgasterasramenes* Nixon, 1968.

**Type information.** Holotype female, NHMUK (examined). Country of type locality: Turkey.

**Geographical distribution.**OTL, PAL.

**OTL**: China (ZJ); **PAL**: Georgia, Hungary, Italy, Korea, Poland, Romania, Russia (PRI), Turkey.

***Microgasteratropa* de Saeger, 1944**

*Microgasteratropa* de Saeger, 1944.

**Type information.** Holotype female, RMCA (not examined but original description checked). Country of type locality: Democratic Republic of Congo.

**Geographical distribution.**AFR.

**AFR**: Democratic Republic of Congo.

***Microgasterauriculata* (Fabricius, 1804)**

*Ichneumonauriculatus* Fabricius, 1804.

*Microgasterauriculatrix* Schulz, 1906 [unjustified emendation].

**Type information.** Type and depository unknown (not examined but subsequent treatment of the species checked). Country of type locality: Austria.

**Geographical distribution.**PAL.

**PAL**: Austria, Germany, Italy, Russia (NC, S).

**Notes.** Our species concept and geographical distribution is based on Nixon (1968) and Papp (1976c), but we exclude it from the UK based on Broad et al. (2016). The species is treated as a member of Ichneumonidae, as *Scolobatesauriculatus* (Fabricius, 1804) in Yu et al. (2016), but the status of this species (and the history of the name use) will require further clarification. The issue is currently under investigation for publication (Ghafouri Moghaddam, pers. comm.), and thus for the time being we present the basic information for this species as it concerns Microgastrinae.

***Microgasteraustralis* Thomson, 1895**

*Microgasteraustralis* Thomson, 1895.

**Type information.** Type and depository unknown (not examined but subsequent treatment of the species checked). Country of type locality: Italy.

**Geographical distribution.**PAL.

**PAL**: Georgia, Germany, Greece, Hungary, Iran, Italy, Kazakhstan, Latvia, Moldova, Mongolia, Montenegro, Poland, Russia (PRI), Slovenia, Spain, Turkey, Turkmenistan.

**Notes.** Our species concept is based on Shaw et al. (2009). The species distribution in Georgia, Turkey and Turkmenistan is based on Belokobylskij et al. (2019).

***Microgasterbalearica* Marshall, 1898**

*Microgasterbalearica* Marshall, 1898.

**Type information.** Syntypes female and male, depository unknown (not examined but original description checked). Country of type locality: Spain.

**Geographical distribution.**PAL.

**PAL**: Spain.

**Notes.** Our species concept is based on Marshall (1898) and Telenga (1955).

***Microgasterbiaca* Xu & He, 1998**

*Microgasterbiaca* Xu & He, 1998.

**Type information.** Holotype female, ZJUH (not examined but subsequent treatment of the species checked). Country of type locality: China.

**Geographical distribution.**OTL.

**OTL**: China (ZJ).

**Notes.** Our species concept is based on Xu and Han (2007).

***Microgasterbreviterebrae* Xu & He, 2003**

*Microgasterbreviterebrae* Xu & He, 2003.

**Type information.** Holotype female, ZJUH (not examined but subsequent treatment of the species checked). Country of type locality: China.

**Geographical distribution.**PAL.

**PAL**: China (HL, JL, LN).

**Notes.** Our species concept is based on Xu and Han (2007).

***Microgasterbrittoni* Viereck, 1917**

*Microgasterbrittoni* Viereck, 1917.

**Type information.** Holotype male, USNM (examined). Country of type locality: USA.

**Geographical distribution.**NEA.

**NEA**: Canada (ON), USA (CT, GA, IA, MA, MI, MN, NY, WI).

***Microgastercampestris* Tobias, 1964**

*Microgastercampestris* Tobias, 1964.

**Type information.** Holotype female, ZIN (not examined but subsequent treatment of the species checked). Country of type locality: Kazakhstan.

**Geographical distribution.**PAL.

**PAL**: Azerbaijan, China (HA, LN), Kazakhstan, Russia (S), Serbia, Uzbekistan.

**Notes.** Our species concept is based on Papp (1976c), Tobias (1986) and Xu and Han (2007).

***Microgastercanadensis* Muesebeck, 1922**

*Microgastercanadensis* Muesebeck, 1922.

**Type information.** Holotype female, USNM (not examined but original description checked). Country of type locality: Canada.

**Geographical distribution.**NEA.

**NEA**: Canada (AB, BC, MB, NB, NS, ON, PE, QC, SK), USA (AR, CO, MA, MI, NY, OR).

***Microgastercaris* Nixon, 1968**

*Microgastercaris* Nixon, 1968.

**Type information.** Holotype female, NHMW (not examined but original description checked). Country of type locality: Austria.

**Geographical distribution.**PAL.

**PAL**: Austria, China (JL), Czech Republic, Hungary, Russia (C, PR), Slovakia, Switzerland.

***Microgasterchrysosternis* (Tobias, 1986)**

*Lissogasterchrysosternis* Tobias, 1986.

**Type information.** Holotype female, ZIN (not examined but original description checked). Country of type locality: Moldova.

**Geographical distribution.**PAL.

**PAL**: Moldova.

***Microgastercongregatiformis* Viereck, 1917**

*Microgastercongregatiformis* Viereck, 1917.

**Type information.** Holotype male, USNM (examined). Country of type locality: USA.

**Geographical distribution.**NEA.

**NEA**: Canada (AB, MB, ON), USA (CA, CT, MA, MI, NJ, NY).

***Microgasterconsors* Nixon, 1968**

*Microgasterconsors* Nixon, 1968.

**Type information.** Holotype female, NHMUK (examined). Country of type locality: United Kingdom.

**Geographical distribution.**PAL.

**PAL**: Hungary, Slovakia, United Kingdom.

***Microgastercrassicornis* Ruthe, 1860**

*Microgastercrassicornis* Ruthe, 1860.

**Type information.** Holotype female, depository unknown (not examined but subsequent treatment of the species checked). Country of type locality: Germany.

**Geographical distribution.**PAL.

**PAL**: Finland, Germany, Hungary, Ireland, Poland, Romania, Russia (BEL, YAR), Serbia, Sweden, Switzerland, United Kingdom.

**Notes.** Our species concept is based on Nixon (1968) and Papp (1976c).

***Microgasterdebilitata* Papp, 1976**

*Microgasterdebilitata* Papp, 1976.

**Type information.** Holotype female, HNHM (not examined but original description checked). Country of type locality: Mongolia.

**Geographical distribution.**PAL.

**PAL**: Mongolia.

***Microgasterdeceptor* Nixon, 1968**

*Microgasterdeceptor* Nixon, 1968.

**Type information.** Holotype female, MZH (examined). Country of type locality: Finland.

**Geographical distribution.**PAL.

**PAL**: Finland, Slovenia.

***Microgasterdeductor* Nixon, 1968**

*Microgasterdeductor* Nixon, 1968.

**Type information.** Holotype female, MZH (not examined but original description checked). Country of type locality: Finland.

**Geographical distribution.**NEA, PAL.

**NEA**: Canada (MB, NT, YT), USA (AK); **PAL**: Finland, Poland, Sweden.

**Notes.** The record from Poland was questioned by Fernandez-Triana (2014) as a possible misidentification, but is still kept here until more evidence is found.

***Microgasterdiscoidus* Xu & He, 2000**

*Microgasterdiscoidus* Xu & He, 2000.

**Type information.** Holotype female, ZJUH (not examined but subsequent treatment of the species checked). Country of type locality: China.

**Geographical distribution.**OTL.

**OTL**: China (SN).

**Notes.** Our species concept is based on Chen and Song (2004) and Xu and Han (2007).

***Microgasterductilis* Nixon, 1968**

*Microgasterductilis* Nixon, 1968.

**Type information.** Holotype female, MZH (examined). Country of type locality: Finland.

**Geographical distribution.**PAL.

**PAL**: Finland, Georgia, Hungary, Korea, Mongolia, Russia (PRI), United Kingdom.

***Microgasterdudichi* Papp, 1961**

*Microgasterdudichi* Papp, 1961.

**Type information.** Holotype female, ZMHB (not examined but subsequent treatment of the species checked). Country of type locality: Germany.

**Geographical distribution.**PAL.

**PAL**: Germany.

**Notes.** Our species concept is based on Nixon (1968), Papp (1976c) and Tobias (1986).

***Microgasterelegans* Herrich-Schäffer, 1838**

*Microgasterelegans* Herrich-Schäffer, 1838.

**Type information.** Type and depository unknown (not examined but subsequent treatment of the species checked). Country of type locality: Germany.

**Geographical distribution.**PAL.

**PAL**: Germany, Netherlands.

**Notes.** Our species concept is based on Herrich-Schäffer (1838), Shenefelt (1973) and Belokobylskij et al. (2003). We have examined the colour plates from the original source (Herrich-Schäffer 1838) and there are three plates (numbered as 153.13, 153.14 and 153.15) which all correspond to Microgastrinae genera. Those plates are detailed enough to allow us to assign each to a genus with a high degree of certainty: plate 13 corresponds to *Microgaster*, 14 to *Glyptapanteles*, and 15 to either *Dolichogenidea* (most likely) or *Apanteles*. However, both catalogues of Szépligeti (1904: 150) and Shenefelt (1973: 705) record *M.elegans* as being described in plate 14. That is likely to be a mistake, as that plate is clearly not *Microgaster* (but the previous one definitely is).

***Microgasterepagoges* Gahan, 1917**

*Microgasterepagoges* Gahan, 1917.

**Type information.** Holotype female, USNM (not examined but subsequent treatment of the species checked). Country of type locality: USA.

**Geographical distribution.**NEA.

**NEA**: Canada (BC, ON, QC), USA (CO, IL, IN, IA, MA, MO, NY, OH, PA, SC, TN, VA).

**Notes.** Our species concept is based on Muesebeck (1922), Nixon (1968) and Fernandez-Triana and Huber (2010).

***Microgastererro* Nixon, 1968**

*Microgastererro* Nixon, 1968.

**Type information.** Holotype female, MZH (examined). Country of type locality: Finland.

**Geographical distribution.**PAL.

**PAL**: Finland, Germany, Hungary, Kazakhstan, Mongolia, Russia (KR, PRI), Serbia, Slovakia, Sweden, Switzerland.

**Notes.** The species distribution in Kazakhstan is based on Belokobylskij et al. (2019).

***Microgastereupolis* Nixon, 1968**

*Microgastereupolis* Nixon, 1968.

**Type information.** Holotype female, NHMW (not examined but original description checked). Country of type locality: Austria.

**Geographical distribution.**PAL.

**PAL**: Austria, Germany, Italy, Serbia, Switzerland.

***Microgasterfamula* Nixon, 1968**

*Microgasterfamula* Nixon, 1968.

**Type information.** Holotype female, NHMW (not examined but original description checked). Country of type locality: Austria.

**Geographical distribution.**PAL.

**PAL**: Austria, Croatia, Hungary, Moldova, Romania, Russia (C), Serbia, Slovakia, Switzerland, Turkey.

***Microgasterfemoralamericana* Shenefelt, 1973**

*Microgasterfemoralamericana* Shenefelt, 1973.

*Microgasterfemoralis* Muesebeck, 1922 [primary homonym of *Microgasterfemoralis* Bouché, 1834].

**Type information.** Holotype female, USNM (not examined but original description checked). Country of type locality: USA.

**Geographical distribution.**NEA.

**NEA**: USA (CA, ID, OR, WA).

**Notes.** Our species concept is based on Muesebeck (1922).

***Microgasterferruginea* Xu & He, 2000**

*Microgasterferruginea* Xu & He, 2000.

**Type information.** Holotype male, ZJUH (not examined but subsequent treatment of the species checked). Country of type locality: China.

**Geographical distribution.**OTL, PAL.

**OTL**: China (ZJ); **PAL**: China (SD).

**Notes.** Our species concept is based on Chen and Song (2004) and Xu and Han (2007).

***Microgasterfilizinancae* Koçak & Kemal, 2013**

*Microgasterfilizinancae* Koçak & Kemal, 2013.

*Microgastergracilis* Inanç, 1992 [primary homonym of *Microgastergracilis* Curtis, 1830].

**Type information.** Holotype female, ZMTU (not examined but subsequent treatment of the species checked). Country of type locality: Turkey.

**Geographical distribution.**PAL.

**PAL**: Turkey.

**Notes.**Koçak and Kemal (2013) proposed the name *Microgasterfilizinancae* as a replacement for *M.gracilis* Inanç, 1992, junior primary homonym of *Microgastergracilis* Curtis, 1830.

***Microgasterfischeri* Papp, 1960**

*Microgasterfischeri* Papp, 1960.

**Type information.** Holotype male, NHMW (not examined but subsequent treatment of the species checked). Country of type locality: Austria.

**Geographical distribution.**PAL.

**PAL**: Austria, Hungary, Moldova, Mongolia, Russia (KDA, PRI), Turkey.

**Notes.** Our species concept is based on Shaw (2012b). We also examined two male paratypes.

***Microgasterflaviventris* Xu & He, 2002**

*Microgasterflaviventris* Xu & He, 2002.

*Microgasterflaviventris* Xu & He, 2002 [primary homonym of *Microgasterflaviventris* Cresson, 1865].

**Type information.** Holotype female, ZJUH (not examined but subsequent treatment of the species checked). Country of type locality: China.

**Geographical distribution.**PAL.

**PAL**: China (HL).

**Notes.** Our species concept is based on Xu and Han (2007).

***Microgasterfulvicrus* Thomson, 1895**

*Microgasterfulvicrus* Thomson, 1895.

*Microgasterstriatoscutellaris* Kiss, 1927.

**Type information.** Syntypes female and male, MZLU (not examined but subsequent treatment of the species checked). Country of type locality: Sweden.

**Geographical distribution.**PAL.

**PAL**: Finland, Germany, Hungary, Ireland, Japan, Korea, Moldova, Montenegro, Romania, Russia (DA, PRI), Serbia, Slovakia, Sweden, Turkey, United Kingdom, Uzbekistan.

**Notes.** Our species concept is based on Nixon (1968), Papp (1976c), and Tobias (1986). The species distribution in Japan and Uzbekistan is based on Belokobylskij et al. (2019).

***Microgasterfusca* Papp, 1959**

*Microgasterfusca* Papp, 1959.

*Microgasterphryne* Nixon, 1968.

**Type information.** Holotype female, HNHM (not examined but paratype examined). Country of type locality: Hungary.

**Geographical distribution.**PAL.

**PAL**: Hungary, Macedonia, Moldova, Romania, Russia (C), Yugoslavia.

**Notes.** We also examined the type of *Microgasterphryne* Nixon.

***Microgastergelechiae* Riley, 1869**

*Microgastergelechiae* Riley, 1869.

*Microgastergelechia* Riley, 1869 [incorrect original spelling].

*Microgastergelechiaetrichotaphae* Walley, 1932.

**Type information.** Holotype male, USNM (examined). Country of type locality: USA.

**Geographical distribution.**NEA.

**NEA**: Canada (ON, QC), USA (CO, CT, DC, IL, LA, MD, MA, MO, NJ, NY, NC, ND, VA, WI).

***Microgasterglabritergites* Xu & He, 2000**

*Microgasterglabritergites* Xu & He, 2000.

**Type information.** Holotype female, ZJUH (not examined but subsequent treatment of the species checked). Country of type locality: China.

**Geographical distribution.**PAL.

**PAL**: China (HL).

**Notes.** Our species concept is based on Xu and Han (2007).

***Microgastergregaria* (Schrank, 1781)**

*Ichneumongregarius* Schrank, 1781.

**Type information.** Type and depository unknown (not examined but original description checked). Country of type locality: unknown.

**Geographical distribution.**PAL.

**PAL**: Austria.

***Microgasterharnedi* Muesebeck, 1922**

*Microgasterharnedi* Muesebeck, 1922.

**Type information.** Holotype female, USNM (not examined but original description checked). Country of type locality: USA.

**Geographical distribution.**NEA.

**NEA**: USA (IN, MA, MI, MS, SC, VA, WA).

***Microgasterhimalayensis* Cameron, 1910**

*Microgasterhimalayensis* Cameron, 1910.

**Type information.** Holotype female, depository unknown (not examined but original description checked). Country of type locality: India.

**Geographical distribution.**OTL.

**OTL**: India.

**Notes.** From the original description, as well as subsequent treatment of the species (Wilkinson 1927), it is not clear if this species actually belongs to *Microgaster*. We suspect it does not, but until further study of the type is done, it is not possible to establish with certainty the generic placement of the species, so we leave it in the genus in which it was originally described.

***Microgasterhospes* Marshall, 1885**

*Microgasterhospes* Marshall, 1885.

*Microgastercomptanae* Viereck, 1911.

**Type information.** Syntypes female and male, NHMUK (examined). Country of type locality: United Kingdom.

**Geographical distribution.**NEA, PAL.

**NEA**: Canada (ON, QC), USA (CO, IA, KS, MD, NJ, NY, OH, UT, VA); **PAL**: Bulgaria, Czech Republic, Finland, Georgia, Germany, Hungary, Ireland, Italy, Lithuania, Moldova, Mongolia, Netherlands, Poland, Romania, Russia (BU, KR, PRI), Slovakia, Switzerland, United Kingdom, Uzbekistan.

**Notes.** We also examined the type of *Microgastercomptanae* Viereck, 1911, a female specimen.

***Microgasterhungarica* Szépligeti, 1896**

*Microgasterhungarica* Szépligeti, 1896.

**Type information.** Lectotype male, HNHM (not examined but subsequent treatment of the species checked). Country of type locality: Hungary.

**Geographical distribution.**PAL.

**PAL**: Austria, Azerbaijan, Hungary, Kyrgyzstan, Moldova, Mongolia, Romania, Russia (KDA, KYA), Ukraine.

**Notes.** Our species concept is based on Papp (1976c, 2004). The species distribution in Azerbaijan and Kyrgyzstan is based on Belokobylskij et al. (2019).

***Microgasterhyalina* Cresson, 1865**

*Microgasterhyalina* Cresson, 1865.

**Type information.** Holotype female, ANSP (not examined but subsequent treatment of the species checked). Country of type locality: Cuba.

**Geographical distribution.**NEO.

**NEO**: Cuba.

**Notes.** Our species concept is based on Muesebeck (1921).

***Microgasterintercus* (Schrank, 1781)**

*Ichneumonintercus* Schrank, 1781.

**Type information.** Holotype female, depository unknown (not examined but original description checked). Country of type locality: Austria.

**Geographical distribution.**PAL.

**PAL**: Austria.

***Microgasterkuchingensis* Wilkinson, 1927**

*Microgasterkuchingensis* Wilkinson, 1927.

**Type information.** Holotype female, NHMUK (examined). Country of type locality: Malaysia.

**Geographical distribution.**AUS, OTL, PAL.

**AUS**: Papua New Guinea; **OTL**: China (FJ, TW, ZJ), India, Malaysia, Philippines; **PAL**: China (JL), Japan.

**Notes.** The holotype is missing the head, the metasoma is detached (but glued to a card) and the micropin is full of rust.

***Microgasterlatitergum* Song & Chen, 2004**

*Microgasterlatitergum* Song & Chen, 2004.

**Type information.** Holotype female, FAFU (not examined but subsequent treatment of the species checked). Country of type locality: China.

**Geographical distribution.**OTL, PAL.

**OTL**: China (FJ, HB); **PAL**: China (JL).

**Notes.** Our species concept is based on Xu and Han (2007).

***Microgasterleechi* Walley, 1935**

*Microgasterleechi* Walley, 1935.

**Type information.** Holotype female, CNC (examined). Country of type locality: Canada.

**Geographical distribution.**NEA.

**NEA**: Canada (BC, MB, ON, QC), USA (FL, MD, MA, MI, OH, OR, PA).

***Microgasterlongicalcar* Xu & He, 2003**

*Microgasterlongicalcar* Xu & He, 2003.

*Microgasterlongicalcar* Xu & He, 2003 [homonym of *Microgasterlongicalcar* Thomson, 1895].

**Type information.** Holotype female, ZJUH (not examined but subsequent treatment of the species checked). Country of type locality: China.

**Geographical distribution.**PAL.

**PAL**: China (HB).

**Notes.** Our species concept is based on Xu and Han (2007).

***Microgasterlongicaudata* Xu & He, 2000**

*Microgasterlongicaudata* Xu & He, 2000.

**Type information.** Holotype female, ZJUH (not examined but subsequent treatment of the species checked). Country of type locality: China.

**Geographical distribution.**OTL.

**OTL**: China (ZJ).

**Notes.** Our species concept is based on Xu and Han (2007).

***Microgasterlongiterebra* Xu & He, 2000**

*Microgasterlongiterebra* Xu & He, 2000.

**Type information.** Holotype female, ZJUH (not examined but subsequent treatment of the species checked). Country of type locality: China.

**Geographical distribution.**OTL.

**OTL**: China (YN).

**Notes.** Our species concept is based on Xu and Han (2007).

***Microgasterluctuosa* Haliday, 1834**

*Microgasterluctuosus* Haliday, 1834.

*Microgastercurvicrus* Thomson, 1895.

**Type information.** Holotype male, NMID (not examined but original description checked). Country of type locality: United Kingdom.

**Geographical distribution.**PAL.

**PAL**: Austria, Azerbaijan, Bulgaria, Croatia, Finland, Germany, Greece, Hungary, Ireland, Israel, Moldova, Mongolia, Poland, Romania, Russia (KR, KIR, PRI, SPE, YAR), Serbia, Sweden, Switzerland, Tunisia, Turkey, Turkmenistan, United Kingdom, Uzbekistan.

**Notes.** The original description (Haliday 1834: 239) is based on a single male specimen. Not only is that clearly stated, but the actual description, which we thoroughly checked, undoubtedly refers to a male specimen as there is no mention of an ovipositor (all previous and subsequent descriptions in that paper, when based on female specimens, mention the ovipositor as aculeus and provide details on its length, but that is missing in the description of *luctuosus*). Shenefelt (1973: 734) also refers to the type as male. However, van Achterberg (1997: 54–55) in his revision of Haliday collection of Braconidae mentions the type as female, which is also referred to by Taxapad (Yu et al. 2016). We follow here the original description in considering the holotype to be a male. The species distribution in Israel and Turkey is based on Belokobylskij et al. (2019).

***Microgastermagnifica* Wilkinson, 1929**

*Microgastermagnifica* Wilkinson, 1929.

**Type information.** Holotype female, NHMUK (examined). Country of type locality: Australia.

**Geographical distribution.**AUS.

**AUS**: Australia (QLD).

***Microgastermemorata* Papp, 1971**

*Microgastermemorata* Papp, 1971.

**Type information.** Holotype female, HNHM (not examined but original description checked). Country of type locality: Mongolia.

**Geographical distribution.**PAL.

**PAL**: Mongolia.

***Microgastermeridiana* Haliday, 1834**

*Microgastermeridiana* Haliday, 1834.

*Microgasterspinolae* Haliday, 1834 [primary homonym of *Microgasterspinolae* Nees, 1834 (?)].

*Microgasteralexis* Curtis, 1837.

*Microgastergrandis* Thomson, 1895.

*Microgastercontubernalis* Marshall, 1898.

**Type information.** Lectotype female, NMID (examined). Country of type locality: unknown.

**Geographical distribution.**PAL.

**PAL**: Bulgaria, Canary Islands, Czech Republic, Finland, Germany, Hungary, Ireland, Italy, Kazakhstan, Latvia, Lithuania, Moldova, Poland, Romania, Russia (IRK, KR, PRI, RYA, SPE, YAR), Slovakia, Spain, Sweden, Switzerland, Turkey, Ukraine, United Kingdom.

**Notes.** Our species concept is based on Shaw (2012b), who also removed *Microgasteracilia* Nixon, 1968 from synonym and reinstated it as a valid species.

***Microgastermessoria* Haliday, 1834**

*Microgastermessoria* Haliday, 1834.

*Microgastertibialis* Nees, 1834 [primary homonym of *Microgastertibialis* Curtis, 1830].

*Microgasterambigua* Ruthe, 1860.

*Microgastermaculata* Ruthe, 1860.

*Microgastervulgaris* Ruthe, 1860.

*Microgasterpluto* Morley, 1936.

**Type information.** Lectotype female, NMID (examined). Country of type locality: unknown.

**Geographical distribution.**NEA, PAL.

**NEA**: Canada (ON, QC); **PAL**: Armenia, Austria, Azerbaijan, Bulgaria, Canary Islands, China (JL, SN, XJ), Croatia, Czech Republic, Finland, France, Georgia, Germany, Hungary, Ireland, Italy, Japan, Kazakhstan, Latvia, Macedonia, Malta, Moldova, Montenegro, Netherlands, Poland, Romania, Russia (DA, KAM, KIR, KDA, MOS, ORE, PRI, SAK, SPE, VGG, YAR), Serbia, Spain, Sweden, Switzerland, Turkey, Turkmenistan, Ukraine, United Kingdom, Uzbekistan.

**Notes.** We also examined the type of *Microgasterpluto* Morley.

***Microgasternerione* Nixon, 1968**

*Microgasternerione* Nixon, 1968.

**Type information.** Holotype female, NHMUK (examined). Country of type locality: Mexico.

**Geographical distribution.**NEO.

**NEO**: Mexico.

***Microgasternigricans* Nees, 1834**

*Microgasternigricans*, Nees, 1834.

**Type information.** Holotype male, ZMHB (not examined but subsequent treatment of the species checked). Country of type locality: Sweden.

**Geographical distribution.**PAL.

**PAL**: Germany, Hungary, Mongolia, Russia (PRI), Sweden, United Kingdom.

**Notes.** Our species concept is based on Papp (2016). Broad et al. (2016) considered the species as of doubtful status in the United Kingdom, but nevertheless listed in their account, a decision we accept and follow here.

***Microgasternitidula* Wesmael, 1837**

*Microgasternitidula* Wesmael, 1837.

**Type information.** Lectotype female, RBINS (not examined but subsequent treatment of the species checked). Country of type locality: Belgium.

**Geographical distribution.**PAL.

**PAL**: Belgium, France, Germany, Poland, Romania, Russia (DA, SPE, YAR), Sweden.

**Notes.** Our species concept is based on Papp (1976c) and Tobias (1986).

***Microgasternixalebion* Shaw, 2004**

*Microgasternixalebion* Shaw, 2004.

**Type information.** Holotype female, RSME (examined). Country of type locality: United Kingdom.

**Geographical distribution.**PAL.

**PAL**: Belgium, France, Greece, United Kingdom.

***Microgasternixoni* Austin & Dangerfield, 1992**

*Microgasternixoni* Austin & Dangerfield, 1992.

**Type information.** Holotype female, ANIC (not examined but original description checked). Country of type locality: Australia.

**Geographical distribution.**AUS.

**AUS**: Australia (NSW, TAS).

***Microgasternobilis* Reinhard, 1880**

*Microgasternobilis* Reinhard, 1880.

*Microgasternobiliscompressifemur* Fahringer, 1937.

**Type information.** Holotype male, depository unknown (not examined but subsequent treatment of the species checked). Country of type locality: Germany.

**Geographical distribution.**PAL.

**PAL**: Armenia, Canary Islands, France, Germany, Hungary, Moldova, Romania, Russia (RYA), Switzerland, Ukraine.

**Notes.** Our species concept is based on Shaw et al. (2009). The species distribution in Armenia is based on Belokobylskij et al. (2019).

***Microgasternovicia* Marshall, 1885**

*Microgasternovicia* Marshall, 1885.

*Microgasterswammerdamiae* Muesebeck, 1922.

**Type information.** Syntypes female and male, NHMUK (examined). Country of type locality: United Kingdom.

**Geographical distribution.**NEA, OTL, PAL.

**NEA**: USA (CT); **OTL**: China (GD, ZJ); **PAL**: Finland, Germany, Hungary, Mongolia, Russia (NW), Serbia, Switzerland, United Kingdom.

**Notes.** We also examined the type of *Microgasterswammerdamiae* Muesebeck, 1922, a female specimen: it has a relatively short ovipositor and the hypopygium is not pleated but inflexible.

***Microgasternoxia* Papp, 1976**

*Microgasternoxia* Papp, 1976.

**Type information.** Holotype female, HNHM (not examined but original description checked). Country of type locality: Mongolia.

**Geographical distribution.**PAL.

**PAL**: Mongolia.

***Microgasterobscuripennata* You & Xia, 1992**

*Microgasterobscuripennata* You & Xia, 1992.

**Type information.** Holotype female, HUNAU (not examined but subsequent treatment of the species checked). Country of type locality: China.

**Geographical distribution.**OTL.

**OTL**: China (HN, ZJ).

**Notes.** Our species concept is based on Xu and Han (2007).

***Microgasteropheltes* Nixon, 1968**

*Microgasteropheltes* Nixon, 1968.

**Type information.** Holotype female, NHMUK (examined). Country of type locality: Macedonia.

**Geographical distribution.**PAL.

**PAL**: Ireland, Italy, Macedonia, Romania, Turkey, Yugoslavia.

***Microgasterostriniae* Xu & He, 2000**

*Microgasterostriniae* Xu & He, 2000.

**Type information.** Holotype female, ZJUH (not examined but subsequent treatment of the species checked). Country of type locality: China.

**Geographical distribution.**OTL, PAL.

**OTL**: China (HN, ZJ); **PAL**: China (LN, SD).

**Notes.** Our species concept is based on Xu and Han (2007).

***Microgasterpantographae* Muesebeck, 1922**

*Microgasterpantographae* Muesebeck, 1922.

**Type information.** Holotype female, USNM (not examined but original description checked). Country of type locality: USA.

**Geographical distribution.**NEA, PAL.

**NEA**: Canada (ON), USA (IL, IA, MA, MI, MO, NY, OH, PA, VA); **PAL**: United Kingdom.

***Microgasterparvistriga* Thomson, 1895**

*Microgasterparvistriga* Thomson, 1895.

*Microgasterparvistrigis* Marshall, 1897 [unjustified emendation].

**Type information.** Type unknown, MZLU (not examined but subsequent treatment of the species checked). Country of type locality: Sweden.

**Geographical distribution.**PAL.

**PAL**: Armenia, Bulgaria, Finland, Germany, Greece, Hungary, Iran, Korea, Mongolia, Poland, Romania, Russia (PRI), Slovakia, Sweden, Switzerland, United Kingdom.

**Notes.** Our species concept is based on Papp (1976c) and Shaw (2012b). The species distribution in Iran is based on Belokobylskij et al. (2019).

***Microgasterperoneae* Walley, 1935**

*Microgasterperoneae* Walley, 1935.

**Type information.** Holotype female, CNC (examined). Country of type locality: Canada.

**Geographical distribution.**NEA.

**NEA**: Canada (BC, NB, NL, NS, ON, QC), USA (AK, DC, MI, OH, WA).

***Microgasterphthorimaeae* Muesebeck, 1922**

*Microgasterphthorimaeae* Muesebeck, 1922.

**Type information.** Holotype female, USNM (not examined but original description checked). Country of type locality: USA.

**Geographical distribution.**NEA.

**NEA**: USA (CA, OR, WA).

***Microgasterplaniabdominalis* You, 2002**

*Microgasterplaniabdominalis* You, 2002.

**Type information.** Holotype female, IEAS (not examined but original description checked). Country of type locality: China.

**Geographical distribution.**PAL.

**PAL**: China (QH).

***Microgasterpolita* Marshall, 1885**

*Microgasterpolita* Marshall, 1885.

*Microgastercarinata* Bengtsson, 1926 [primary homonym of *Microgastercarinata* Packard, 1881].

*Microgasterbengtssoni* Fahringer, 1937.

**Type information.** Holotype male, NHMUK (examined). Country of type locality: United Kingdom.

**Geographical distribution.**PAL.

**PAL**: Armenia, Finland, Germany, Hungary, Ireland, Kazakhstan, Korea, Lithuania, Norway, Poland, Romania, Russia (KAM, PRI, SAK, SPE), Sweden, Switzerland, United Kingdom.

**Notes.** The species distribution in Kazakhstan is based on Belokobylskij et al. (2019).

***Microgasterpostica* Nees, 1834**

*Microgasterpostica* Nees, 1834.

*Microgastermarginella* Wesmael, 1837.

**Type information.** Holotype female, ZMHB (not examined but subsequent treatment of the species checked). Country of type locality: Germany.

**Geographical distribution.**PAL.

**PAL**: Belgium, Czech Republic, France, Germany, Hungary, Netherlands, Poland, Romania, Russia (PRI).

**Notes.** Our species concept is based on Papp (1976c) and Tobias (1986).

***Microgasterprocera* Ruthe, 1860**

*Microgasterprocerus* Ruthe, 1860.

*Microgasterintermedia* Ivanov, 1899.

**Type information.** Type and depository unknown (not examined but subsequent treatment of the species checked). Country of type locality: Germany.

**Geographical distribution.**PAL.

**PAL**: Austria, Finland, Germany, Hungary, Ireland, Mongolia, Netherlands, Poland, Romania, Russia (SPE), Spain, Ukraine.

**Notes.** Our species concept is based on Nixon (1968), Papp (1976c) and Shaw (2012b).

***Microgasterpseudotibialis* Fahringer, 1937**

*Microgasterpseudotibialis* Fahringer, 1937.

*Microgastertibialis* Brullé, 1832 [primary homonym of *Microgastertibialis* Curtis, 1830].

**Type information.** Type and depository unknown (not examined but original description checked). Country of type locality: unknown.

**Geographical distribution.**PAL.

**PAL**: Algeria, Greece.

***Microgasterpunctithorax* Xu & He, 2000**

*Microgasterpunctithorax* Xu & He, 2000.

**Type information.** Holotype male, ZJUH (not examined but subsequent treatment of the species checked). Country of type locality: China.

**Geographical distribution.**PAL.

**PAL**: China (LN).

**Notes.** Our species concept is based on Xu and Han (2007).

***Microgasterraschkiellae* Shaw, 2012**

*Microgasterraschkiellae* Shaw, 2012.

**Type information.** Holotype female, RSME (examined). Country of type locality: United Kingdom.

**Geographical distribution.**NEA, PAL.

**NEA**: Canada (MB); **PAL**: United Kingdom.

***Microgasterrava* You & Zhou, 1996**

*Microgasterravus* You & Zhou, 1996.

**Type information.** Holotype female, HUNAU (not examined but subsequent treatment of the species checked). Country of type locality: China.

**Geographical distribution.**OTL.

**OTL**: China (GX).

**Notes.** Our species concept is based on Chen and Song (2004), Xu and Han (2007), and Kotenko (2007a). The species name must be treated as an adjective and not as a noun (Doug Yanega, pers. comm.) and thus it must match the gender of the genus name.

***Microgasterreticulata* Shestakov, 1940**

*Microgasterreticulata* Shestakov, 1940.

**Type information.** Holotype female, NHRS (not examined but subsequent treatment of the species checked). Country of type locality: Russia.

**Geographical distribution.**PAL.

**PAL**: Russia (PRI).

**Notes.** Our species concept is based on Nixon (1968) and Kotenko (2007a).

***Microgasterrubricollis* Spinola, 1851**

*Microgasterrubricollis* Spinola, 1851.

**Type information.** Type and depository unknown (not examined but original description checked). Country of type locality: Chile.

**Geographical distribution.**NEO.

**NEO**: Chile.

***Microgasterrufipes* Nees, 1834**

*Microgasterrufipes* Nees, 1834. [= *Microgasterglobata* auctt., not Linnaeus, 1758].

*Ichneumongossypinus* Retzius, 1783.

*Ichneumonglobator* Thunberg, 1822.

*Microgasteranthomyiarum* Bouché, 1834.

*Microgasteramentorum* Ratzeburg, 1844.

*Microgastersubincompleta* Ratzeburg, 1852.

*Microgasterlaeviscuta* Thomson, 1895.

*Microgasterincurvata* Papp, 1976.

**Type information.** Type and depository unknown (not examined but subsequent treatment of the species checked). Country of type locality: unknown.

**Geographical distribution.**PAL.

**PAL**: Albania, Armenia, Austria, Azerbaijan, Belgium, Bulgaria, Canary Islands, China (JL), Croatia, Czech Republic, Finland, France, Georgia, Germany, Greece, Hungary, Iran, Ireland, Italy, Japan, Kazakhstan, Korea, Latvia, Lithuania, Moldova, Mongolia, Montenegro, Netherlands, Norway, Poland, Romania, Russia (IRK, KAM, KC, MOS, PRI, ROS, SAK, SPE, SAR, VGG, VOR, YAR), Serbia, Slovakia, Slovenia, Spain, Sweden, Switzerland, Turkey, Turkmenistan, Ukraine, United Kingdom.

**Notes.** We accept and follow here the decision made by van Achterberg (2014) to apply the name *Microgasterrufipes* Nees, 1834 to what historically has been considered *Microgasterglobata* Linnaeus, 1758. Because of its importance and future implications, we reproduce verbatim what van Achterberg (2014: 207) wrote:

“*Ichneumonglobatus* Linnaeus, 1758 has been for long a problematic species and was mostly associated with the genus *Microgaster* Latreille, 1804. This is contradicted by the biological data supplied in the original description and the specimens in the Linnean Society (http://linnean-on1ine.org/16250/). They may be from the original Linnean collection and are labelled (by Linnaeus?) as «globatus ?». It concerns specimens of a gregarious species with yellow hind coxae on a white cocoon-mass, probably belonging to the genus *Protapanteles* Ashmead, 1900. This disagrees with Linnaeus' (1761) statement that the host is *Papiliobrassicae* and therewith implying that this species is *Cotesiaglomerata* (Linnaeus, 1758), the common gregarious parasitoid of the cabbage white (*Pierisbrassicae* (Linnaeus, 1758)). The oldest available name for *Ichneumonglobatus* auctt. is *Microgasterrufipes* Nees, 1834”.

After we examined the two photos of the purportedly syntypes of *Ichneumonglobatus* Linnaeus, 1758, as depicted in the web link mentioned by van Achterberg (2014), we agree that the specimens shown there do not belong to *Microgaster*; we think that the best generic placement at present would be in *Glyptapanteles*. That implies that all historical references to *Microgasterglobata* Linnaeus, which were commonly associated to any *Microgaster* with reddish femora/legs (e.g., see comments on that by Scaramozzino et al. 2017) should now be referred to *Microgasterrufipes* Nees, 1834, which is the oldest available name, as van Achterberg (2014) correctly proposed [For more details on this, see the treatment of *globata* by older sources; e.g., *rufipes* was considered as one of three varieties of *globata* in the Hymenoptera Catalogue of Dalla Torre (1898: 153), with the two other names listed as varieties of *globata* being junior to *rufipes*)]. However, there remains a tangle of species that have been, in some cases almost certainly incorrectly, synonymized under *globata* (e.g., as discussed by Shaw 2012b and Broad et al. 2016), so it is too simple a solution to suggest that by accepting and following van Achterberg’s (2014) decision, all records from Europe that were previously cited as *globata* (i.e., all countries listed for *globata* in Yu et al. 2012, Yu et al. 2016, Broad et al. 2016) should just be transferred to *rufipes*. [One example of the problems that remain is the name *Microgasterlaeviscuta* Thomson, 1895. Papp (1976c) synonymized it under *Ichneumonglobatus* Linnaeus, 1758; however, Shaw (2012b) questioned this, based on material from the NMS he had seen, and instead suggested that *laeviscuta* was probably a different species but more study was required before both species were recognized as separate; subsequently, Yu et al. (2016) considered both as different species. Until more evidence is available, here we are following Shaw (2012b) and thus list *laeviscuta* as a questionable synonym of *rufipes* (= *globata* auctt.)]. For the morphological concept of *rufipes* we follow Papp (1976c), Tobias (1986), Kotenko (2007a), and Xu and Han (2007); we also read the original descriptions of the names involved (Linnaeus 1758, Nees 1834). In addition to this, we here propose that the specimens from Linnaeus be considered as a different species, for now restricted to Sweden (e.g., see Linnaeus 1761: 411, specimen 1645), and to be placed in the genus *Glyptapanteles* (see further comments and rationale for our decision under the Notes for the species *Glyptapantelesglobatus* (Linnaeus, 1758)).

***Microgasterruralis* Xu & He, 1998**

*Microgasterruralis* Xu & He, 1998.

**Type information.** Holotype female, ZJUH (not examined but original description checked). Country of type locality: China.

**Geographical distribution.**OTL.

**OTL**: China (FJ, HB, JL, LN).

***Microgasterscopelosomae* Muesebeck, 1926**

*Microgasterscopelosomae* Muesebeck, 1926.

**Type information.** Holotype female, USNM (examined). Country of type locality: USA.

**Geographical distribution.**NEA.

**NEA**: USA (MA).

***Microgastershennongjiaensis* Xu & He, 2001**

*Microgastershennongjiaensis* Xu & He, 2001.

**Type information.** Holotype female, ZJUH (not examined but original description checked). Country of type locality: China.

**Geographical distribution.**OTL.

**OTL**: China (HB).

***Microgasterstictica* Ruthe, 1858**

*Microgasterstictica* Ruthe, 1858.

*Microgasterconfusa* Papp, 1971.

**Type information.** Type and depository unknown (not examined but subsequent treatment of the species checked). Country of type locality: unknown.

**Geographical distribution.**PAL.

**PAL**: Bulgaria, Croatia, Czech Republic, Finland, Germany, Hungary, Ireland, Italy, Korea, Mongolia, Netherlands, Poland, Romania, Russia (PRI), Slovakia, Spain, Sweden, Switzerland, Turkey, United Kingdom, Yugoslavia.

**Notes.** Our species concept is based on Nixon (1968), Papp (1986) and Kotenko (2007a).

***Microgastersubcompleta* Nees, 1834**

*Microgastersubcompleta* Nees, 1834.

*Microgasterannulipes* Curtis, 1830 [*nomen nudum*].

*Microgastercarinata* Packard, 1881.

**Type information.** Type and depository unknown (not examined but subsequent treatment of the species checked). Country of type locality: Germany.

**Geographical distribution.**NEA, PAL.

**NEA**: USA (IL, MA, NH, NJ, NY); **OTL**: China (GX); **PAL**: Armenia, Austria, Azerbaijan, Belarus, Belgium, Bulgaria, China (HL, JL), Croatia, Czech Republic, France, Georgia, Germany, Hungary, Ireland, Italy, Japan, Korea, Lithuania, Macedonia, Moldova, Netherlands, Poland, Romania, Russia (BEL, KHA, KDA, MOS, NGR, PRI, RYA, SAK, SPE, VOR, YAR), Slovakia, Spain, Switzerland, Turkey, Ukraine, United Kingdom, Yugoslavia.

**Notes.** Our species concept is based on Nixon (1968), Papp (1976c), Kotenko (2007a), Xu and Han (2007) and Shaw et al. (2009).

***Microgastersubtilipunctata* Papp, 1959**

*Microgastersubtilipunctata* Papp, 1959.

*Microgasterobsepiens* Nixon, 1968.

**Type information.** Holotype female, HNHM (not examined but subsequent treatment of the species checked). Country of type locality: Hungary.

**Geographical distribution.**PAL.

**PAL**: Austria, Germany, Hungary, Moldova, Romania, Russia (NC, S), Switzerland, Turkey.

**Notes.** Our species concept is based on Nixon (1968) and Papp (1976c).

***Microgastersyntopic* Fernandez-Triana, 2018**

*Microgastersyntopic* Fernandez-Triana, 2018.

**Type information.** Holotype female, CNC (examined). Country of type locality: USA.

**Geographical distribution.**NEA.

**NEA**: USA (FL, GA).

***Microgasterszelenyii* Papp, 1974**

*Microgasterszelenyii* Papp, 1974.

**Type information.** Holotype female, HNHM (not examined but paratype examined). Country of type locality: Korea.

**Geographical distribution.**OTL, PAL.

**OTL**: China (GZ, ZJ); **PAL**: China (HA, JL, LN), Korea, Russia (PRI).

***Microgastertaishana* Xu, He & Chen, 1998**

*Microgastertaishana* Xu, He & Chen, 1998.

**Type information.** Holotype female, SAUC (not examined but subsequent treatment of the species checked). Country of type locality: China.

**Geographical distribution.**PAL.

**PAL**: China (SD).

**Notes.** Our species concept is based on Chen and Song (2004), Xu and Han (2007) and Kotenko (2007a). The depository acronym is based on the institution name, Shandong Agricultural University, China.

***Microgastertianmushana* Xu & He, 2001**

*Microgastertianmushana* Xu & He, 2001.

**Type information.** Holotype female, ZJUH (not examined but subsequent treatment of the species checked). Country of type locality: China.

**Geographical distribution.**OTL.

**OTL**: China (ZJ).

**Notes.** Our species concept is based on Xu and Han (2007).

***Microgastertjibodas* Wilkinson, 1927**

*Microgastertjibodas* Wilkinson, 1927.

**Type information.** Holotype female, NHMUK (examined). Country of type locality: Indonesia.

**Geographical distribution.**OTL.

**OTL**: Indonesia.

***Microgastertortricis* (Schrank, 1781)**

*Ichneumontortricis* Schrank, 1781.

**Type information.** Type and depository unknown (not examined but original description checked). Country of type locality: Austria.

**Geographical distribution.**PAL.

**PAL**: Austria.

***Microgastertremenda* Papp, 1971**

*Microgastertremenda* Papp, 1971.

**Type information.** Holotype female, HNHM (not examined but subsequent treatment of the species checked). Country of type locality: Mongolia.

**Geographical distribution.**PAL.

**PAL**: Mongolia.

**Notes.** Our species concept is based on Kotenko (2007a).

***Microgasteruliginosa* Thomson, 1895**

*Microgasteruliginosus* Thomson, 1895.

**Type information.** Holotype female, MZLU (not examined but subsequent treatment of the species checked). Country of type locality: Sweden.

**Geographical distribution.**PAL.

**PAL**: Finland, Netherlands, Poland, Romania, Russia (NW), Sweden.

**Notes.** Our species concept is based on Papp (1976c).

***Microgasterutibilis* Papp, 1976**

*Microgasterutibilis* Papp, 1976.

**Type information.** Holotype female, HNHM (not examined but original description checked). Country of type locality: Mongolia.

**Geographical distribution.**PAL.

**PAL**: Mongolia.

**Notes.** Our species concept is based on Papp (1976c) and Kotenko (2007a).

***Microgastervaricornis* Rondani, 1872**

*Microgastervaricornis* Rondani, 1872.

**Type information.** Type and depository unknown (not examined). Country of type locality: Italy.

**Geographical distribution.**PAL.

**PAL**: Italy.

***Microgasteryichunensis* Xu & Chen, 2002**

*Microgasteryichunensis* Xu & Chen, 2002.

**Type information.** Holotype female, ZJUH (not examined but subsequent treatment of the species checked). Country of type locality: China.

**Geographical distribution.**PAL.

**PAL**: China (HL).

**Notes.** Our species concept is based on Xu and Han (2007).

***Microgasteryunnanensis* Xu & He, 1999**

*Microgasteryunnanensis* Xu & He, 1999.

**Type information.** Holotype female, ZJUH (not examined but subsequent treatment of the species checked). Country of type locality: China.

**Geographical distribution.**OTL.

**OTL**: China (YN).

**Notes.** Our species concept is based on Xu and Han (2007).

***Microgasterzhaoi* Xu & He, 1997**

*Microgasterzhaoi* Xu & He, 1997.

**Type information.** Holotype female, ZJUH (not examined but subsequent treatment of the species checked). Country of type locality: China.

**Geographical distribution.**OTL.

**OTL**: China (FJ).

**Notes.** Our species concept is based on Xu and Han (2007).

#### Genus Microplitis Foerster, 1863

***Microplitis*** Foerster, 1863: 245. Gender: masculine. Type species: *Microgastersordipes* Nees, 1834, by original designation.

This is a cosmopolitan genus, with 192 described species, but we have seen many additional species in collections, mostly from temperate areas, and the actual number of species could be at least twice that currently known. There are some revisions available for certain regions and/or countries, but most are outdated and even the most recent ones do not take into account the hidden diversity that is revealed by DNA barcoding and biological data. Approximately 12 families of Lepidoptera have been recorded as hosts for *Microplitis*, but many records are likely to be incorrect and/or need further verification. There are almost 4,000 DNA-barcode compliant sequences of this genus in BOLD, representing 212 BINs.

***Microplitisabrs* Austin & Dangerfield, 1993**

*Microplitisabrs* Austin & Dangerfield, 1993.

**Type information.** Holotype female, ANIC (not examined but original description checked). Country of type locality: Australia.

**Geographical distribution.**AUS, OTL.

**AUS**: Australia (QLD); **OTL**: Vietnam.

***Microplitisadelaidensis* Austin & Dangerfield, 1993**

*Microplitisadelaidensis* Austin & Dangerfield, 1993.

**Type information.** Holotype female, ANIC (not examined but original description checked). Country of type locality: Australia.

**Geographical distribution.**AUS.

**AUS**: Australia (SA).

***Microplitisadisurae* (Subba Rao & Sharma, 1960), new combination**

*Microgasteradisurae* Subba Rao & Sharma, 1960.

**Type information.** Holotype female, INPC (not examined but original description checked). Country of type locality: India.

**Geographical distribution.**OTL.

**OTL**: India.

**Notes.** Transferred to *Microplitis* based on the original descriptions and illustrations provided, which show short ovipositor and ovipositor sheaths (the sheaths with only few setae concentrated at apex), T1 narrowing towards posterior margin and more than 2.5 × as long medially as its width at posterior margin, T2 subtriangular, metatibial spurs less than half length of first segment of metatarsus. All these characters exclude the species from being *Microgaster* and strongly indicate the best generic placement at present to be in *Microplitis*.

***Microplitisadrianguadamuzi* Fernandez-Triana & Whitfield, 2015**

*Microplitisadrianguadamuzi* Fernandez-Triana & Whitfield, 2015.

**Type information.** Holotype female, USNM (examined). Country of type locality: Costa Rica.

**Geographical distribution.**NEO.

**NEO**: Costa Rica.

***Microplitisaduncus* (Ruthe, 1860)**

*Microgasteraduncus* Ruthe, 1860.

*Microgasterbrachycerus* Thomson, 1895.

**Type information.** Holotype female, NHMUK (examined). Country of type locality: Germany.

**Geographical distribution.**PAL.

**PAL**: Bulgaria, Finland, Georgia, Germany, Hungary, Iran, Korea, Mongolia, Netherlands, Poland, Russia (KAM), Selvagens Islands, Serbia, Sweden, Switzerland, Tunisia, Turkmenistan, United Kingdom.

**Notes.** The holotype is in relatively poor condition, missing the antennae and with the hind wings glued over the body, obscuring or impeding the observation of features of part of the mesosoma and most of the metasoma. The species distribution in Georgia, Korea, and Turkmenistan is based on Belokobylskij et al. (2019).

***Microplitisajmerensis* Rao & Kurian, 1950**

*Microplitisajmerensis* Rao & Kurian, 1950.

**Type information.** Holotype female, NZSI (not examined but subsequent treatment of the species checked). Country of type locality: India.

**Geographical distribution.**OTL.

**OTL**: India.

**Notes.** Our species concept is based on Gupta (2013a) and Ranjith et al. (2015a).

***Microplitisalajensis* Telenga, 1955, restored combination**

*Microplitisalajensis* Telenga, 1955.

**Type information.** Lectotype female, depository unknown (not examined but original description checked). Country of type locality: Kyrgyzstan.

**Geographical distribution.**PAL.

**PAL**: Kyrgyzstan, Mongolia.

**Notes.** Our species concept is based on Telenga (1955), Papp (1984c) and Tobias (1986). This species was at times considered to belong to *Microgaster*, e.g., Papp (1984c) and Tobias (1986), as part of the confusion with the application and use of the *Microplitis* and *Microgaster* names, which was only solved after 1988 (see more details and comments under our introduction to the genus *Microgaster* above, p 717). The correct generic placement at present would be in *Microplitis*, which is corroborated by the original description and images, as well as the key and images provided by Papp (1984c). Because some of the more recent references (e.g., Yu et al. 2016) still refer to the species within *Microgaster*, for the sake of clarity we restore its status here.

***Microplitisalaskensis* Ashmead, 1902**

*Microplitisalaskensis* Ashmead, 1902.

**Type information.** Holotype female, USNM (examined). Country of type locality: USA.

**Geographical distribution.**NEA.

**NEA**: Canada (AB, BC, MB, NS, ON, QC), USA (AK, AZ, CA, CO, IL, KS, MA, MT, NY, OR, WA).

***Microplitisalbipennis* Abdinbekova, 1969**

*Microplitisalbipennis* Abdinbekova, 1969.

**Type information.** Holotype male, ZIN (not examined but subsequent treatment of the species checked). Country of type locality: Azerbaijan.

**Geographical distribution.**PAL.

**PAL**: Azerbaijan, Hungary, Mongolia, Poland, Russia (NC), Turkey.

**Notes.** Our species concept is based on Papp (1984c) and Tobias (1986).

***Microplitisalbotibialis* Telenga, 1955**

*Microplitisalbotibialis* Telenga, 1955.

**Type information.** Type and depository unknown (not examined but original description checked). Country of type locality: Russia.

**Geographical distribution.**OTL, PAL.

**OTL**: China (HN); **PAL**: China (HA, JL, LN), Hungary, Korea, Mongolia, Russia (ZAB, PRI).

***Microplitisalexanderrojasi* Fernandez-Triana & Whitfield, 2015**

*Microplitisalexanderrojasi* Fernandez-Triana & Whitfield, 2015.

**Type information.** Holotype female, USNM (examined). Country of type locality: Costa Rica.

**Geographical distribution.**NEO.

**NEO**: Costa Rica.

***Microplitisaltissimus* Fernandez-Triana, 2018**

*Microplitisaltissimus* Fernandez-Triana, 2018.

**Type information.** Holotype female, CNC (examined). Country of type locality: USA.

**Geographical distribution.**NEA.

**NEA**: USA (CO).

***Microplitisamplitergius* Xu & He, 2002**

*Microplitisamplitergius* Xu & He, 2002.

**Type information.** Holotype female, ZJUH (not examined but original description checked). Country of type locality: China.

**Geographical distribution.**OTL, PAL.

**OTL**: China (GZ, ZJ); **PAL**: China (LN, NX).

***Microplitisaprilae* Austin & Dangerfield, 1993**

*Microplitisaprilae* Austin & Dangerfield, 1993.

**Type information.** Holotype female, ANIC (not examined but original description checked). Country of type locality: Australia.

**Geographical distribution.**AUS, OTL.

**AUS**: Australia (NSW, NT, QLD); **OTL**: Vietnam.

***Microplitisareyongensis* Austin & Dangerfield, 1993**

*Microplitisareyongensis* Austin & Dangerfield, 1993.

**Type information.** Holotype female, AEIC (not examined but original description checked). Country of type locality: Australia.

**Geographical distribution.**AUS, OTL.

**AUS**: Australia (NT); **OTL**: India, Vietnam.

***Microplitisariatus* Papp, 1979**

*Microplitisariatus* Papp, 1979.

**Type information.** Holotype female, HNHM (not examined but original description checked). Country of type locality: Tunisia.

**Geographical distribution.**PAL.

**PAL**: Canary Islands, Tunisia.

***Microplitisatamiensis* Ashmead, 1906**

*Microplitisatamiensis* Ashmead, 1906.

**Type information.** Holotype male, USNM (examined). Country of type locality: Japan.

**Geographical distribution.**PAL.

**PAL**: Japan, Korea.

***Microplitisautographae* Muesebeck, 1922**

*Microplitisautographae* Muesebeck, 1922.

**Type information.** Holotype female, USNM (not examined but original description checked). Country of type locality: USA.

**Geographical distribution.**NEA.

**NEA**: Canada (AB, ON, QC), USA (AZ, ID, KS, NM).

***Microplitisbamagensis* Austin & Dangerfield, 1993**

*Microplitisbamagensis* Austin & Dangerfield, 1993.

**Type information.** Holotype female, AEIC (not examined but original description checked). Country of type locality: Australia.

**Geographical distribution.**AUS.

**AUS**: Australia (QLD).

***Microplitisbasalis* (Bingham, 1906)**

*Microgasterbasalis* Bingham, 1906.

*Microgasterbasalis* Bingham, 1906 [primary homonym of *Microgasterbasalis* Stephens, 1846].

**Type information.** Holotype female, OUMNH (not examined but subsequent treatment of the species checked). Country of type locality: Australia.

**Geographical distribution.**AUS.

**AUS**: Australia (QLD).

**Notes.** Our species concept is based on Austin and Dangerfield (1993).

***Microplitisbasipallescentis* Song & Chen, 2008**

*Microplitisbasipallescentis* Song & Chen, 2008.

**Type information.** Holotype female, HUNAU (not examined but original description checked). Country of type locality: China.

**Geographical distribution.**OTL.

**OTL**: China (HB).

***Microplitisbeyarslani* Inanç, 2002**

*Microplitisbeyarslani* Inanç, 2002.

**Type information.** Holotype female, ZMTU (not examined). Country of type locality: Turkey.

**Geographical distribution.**PAL.

**PAL**: Turkey.

**Notes.** The depository acronym was selected based on the institution name (Zoological Museum, Trakya University, Turkey).

***Microplitisbicoloratus* Xu & He, 2003**

*Microplitisbicoloratus* Xu & He, 2003.

*Microplitisbicoloratus* Chen, 2004 [primary homonym of *Microplitisbicoloratus* Xu & He, 2003].

**Type information.** Holotype female, FAFU (not examined but original description checked). Country of type locality: China.

**Geographical distribution.**OTL, PAL.

**OTL**: China (FJ, HB, ZJ), India; **PAL**: China (SD).

***Microplitisblascoi* Papp & Shaw, 2001**

*Microplitisblascoi* Papp & Shaw, 2001.

**Type information.** Holotype female, RSME (examined). Country of type locality: Spain.

**Geographical distribution.**PAL.

**PAL**: Spain.

***Microplitisbomiensis* Zhang, 2019**

*Microplitisbomiensis* Zhang, 2019.

**Type information.** Holotype female, FAFU (not examined but original description checked). Country of type locality: China.

**Geographical distribution.**PAL.

**PAL**: China (XZ).

***Microplitisborealis* Xu & He, 2000**

*Microplitisborealis* Xu & He, 2000.

*Microplitisborealis* Xu & He, 2000 [primary homonym of *Microplitisborealis* Marshall, 1885].

**Type information.** Holotype male, ZJUH (not examined but original description checked). Country of type locality: China.

**Geographical distribution.**PAL.

**PAL**: China (JL, LN, XJ).

***Microplitisbradleyi* Muesebeck, 1922**

*Microplitisbradleyi* Muesebeck, 1922.

**Type information.** Holotype female, CUIC (not examined but original description checked). Country of type locality: USA.

**Geographical distribution.**NEA.

**NEA**: Canada (AB, BC), USA (CA, OR, UT).

***Microplitisbrassicae* Muesebeck, 1922**

*Microplitisbrassicae* Muesebeck, 1922.

**Type information.** Holotype female, USNM (not examined but original description checked). Country of type locality: USA.

**Geographical distribution.**NEA.

**NEA**: USA (AZ, CA, CO, NE, NM, TX).

***Microplitisbrevispina* Song & Chen, 2008**

*Microplitisbrevispina* Song & Chen, 2008.

**Type information.** Holotype female, HUNAU (not examined but original description checked). Country of type locality: China.

**Geographical distribution.**OTL.

**OTL**: China (FJ).

***Microplitiscapeki* Nixon, 1970**

*Microplitiscapeki* Nixon, 1970.

**Type information.** Holotype female, MMBC (not examined but original description checked). Country of type locality: Czech Republic.

**Geographical distribution.**PAL.

**PAL**: Czech Republic, Germany, Hungary.

***Microplitiscarinatus* Song & Chen, 2008**

*Microplitiscarinata* Song & Chen, 2008.

*Microplitiscarinata* Song & Chen, 2008 [primary homonym of *Microplitiscarinata* Ahsmead, 1900].

**Type information.** Holotype female, HUNAU (not examined but original description checked). Country of type locality: China.

**Geographical distribution.**OTL.

**OTL**: China (HB).

***Microplitiscarinicollis* (Cameron, 1905)**

*Microgaster* (?) *carinicollis* Cameron, 1905.

**Type information.** Type and depository unknown (not examined but original description checked). Country of type locality: Sri Lanka.

**Geographical distribution.**OTL.

**OTL**: India, Sri Lanka.

***Microplitiscarteri* Walley, 1932**

*Microplitiscarteri* Walley, 1932.

**Type information.** Holotype male, CNC (examined). Country of type locality: Canada.

**Geographical distribution.**NEA.

**NEA**: Canada (AB).

***Microplitiscebes* Nixon, 1970**

*Microplitiscebes* Nixon, 1970.

**Type information.** Holotype female, NHMUK (examined). Country of type locality: Switzerland.

**Geographical distribution.**PAL.

**PAL**: Austria, Croatia, Germany, Greece, Hungary, Mongolia, Poland, Serbia, Slovakia, Spain, Switzerland, Turkey.

***Microplitisceratomiae* Riley, 1881**

*Microplitisceratomiae* Riley, 1881.

*Microplitiswaldeni* Viereck, 1917.

*Microplitisceratomiaeactuosus* Riley, 1881.

**Type information.** Syntypes female and male, USNM (examined). Country of type locality: USA.

**Geographical distribution.**NEA.

**NEA**: Canada (NB, NS, ON, QC, SK), USA (AR, CA, CO, CT, DC, IL, KS, MA, MI, MO, NJ, NM, NY, OR, RI, TX).

**Notes.**Janzen et al. (2003) stated that *ceratomiae* could actually represent a complex of morphologically similar but biologically distinct species. We have examined the type of *Microplitiswaldeni* Viereck, 1917, currently a synonym of *M.ceratomiae*, and found it to be different based on a) larger body size, b) darker colour, c) coarser sculpture of frons, clypeus, anteromesoscutum, and mesopleuron, and d) slight differences in the shape of T1 and the setae pattern on metasomal terga. However, we refrain here to reinstate *waldeni* as a valid species until more study of specimens allows for a better sorting of their distribution (as well as clarifying if other potential cryptic species exist under the *ceratomiae* name).

***Microplitischacoensis* (Cameron, 1908)**

*Microgasterchacoensis* Cameron, 1908.

*Microplitisayerzai* Brèthes, 1910.

**Type information.** Holotype female, NHMUK (examined). Country of type locality: United Kingdom.

**Geographical distribution.**NEO, PAL.

**NEO**: Argentina, Brazil, Paraguay, Trinidad & Tobago, Uruguay, Venezuela; **PAL**: United Kingdom.

***Microplitischangbaishanus* Song & Chen, 2008**

*Microplitischangbaishanus* Song & Chen, 2008.

**Type information.** Holotype female, HUNAU (not examined but original description checked). Country of type locality: China.

**Geographical distribution.**PAL.

**PAL**: China (JL).

***Microplitischivensis* Telenga, 1955, restored combination**

*Microplitischivensis* Telenga, 1955.

**Type information.** Lectotype male, ZIN (not examined but original description checked). Country of type locality: Uzbekistan.

**Geographical distribution.**PAL.

**PAL**: Uzbekistan.

**Notes.** Our species concept is based on Telenga (1955), Papp (1984c) and Tobias (1986). Transferred back to *Microplitis* because of the short ovipositor and metatibial spurs shorter than half the length of first segment of metatarsus. The reference to this species as *Microgaster* in papers after the original description (e.g., Papp 1984c, Tobias 1986, Yu et al. 2012, 2016) is only due to the confusion with the application of the *Microgaster* name and its type species (see details on that above, under the introduction to the genus *Microgaster*).

***Microplitischoui* Xu & He, 2000**

*Microplitischoui* Xu & He, 2000.

**Type information.** Holotype female, ZJUH (not examined but subsequent treatment of the species checked). Country of type locality: China.

**Geographical distribution.**PAL.

**PAL**: China (GS, SN).

**Notes.** Our species concept is based on Xu and He (2003b).

***Microplitischrysostigma* Tobias, 1964, restored combination**

*Microplitischrysostigma* Tobias, 1964.

**Type information.** Holotype female, ZIN (not examined but subsequent treatment of the species checked). Country of type locality: Kazakhstan.

**Geographical distribution.**PAL.

**PAL**: Kazakhstan.

**Notes.** Our species concept is based on Papp (1984c) and Tobias (1986); both authors dealt with this taxon in their keys to *Microplitis*, and the closest species (morphologically) in these papers are all *Microplitis*. The reference to this species as *Microgaster* in papers after the original description (e.g., Papp 1984c, Tobias 1986, Yu et al. 2012, 2016) is only due to the confusion with the application of the name *Microgaster* and its type species (see details above, under the introduction to the genus *Microgaster*; p 717).

***Microplitischui* Xu & He, 2002**

*Microplitischui* Xu & He, 2002.

**Type information.** Holotype female, ZJUH (not examined but subsequent treatment of the species checked). Country of type locality: China.

**Geographical distribution.**OTL.

**OTL**: China (ZJ).

**Notes.** Our species concept is based on Chen and Song (2004), Kotenko (2007a) and Ranjith et al. (2015a).

***Microplitiscoactus* (Lundbeck, 1896)**

*Microgastercoactus* Lundbeck, 1896.

**Type information.** Lectotype female, ZMUC (not examined but subsequent treatment of the species checked). Country of type locality: Greenland.

**Geographical distribution.**NEA, PAL.

**NEA**: Canada (NU), Greenland; **PAL**: Iceland.

**Notes.** Our species concept is based on Muesebeck (1922), Papp (1984c), van Achterberg (2006), and Fernandez-Triana et al. (2017b).

***Microplitiscombinatus* (Papp, 1984)**

*Microgastercombinata* Papp, 1984.

**Type information.** Holotype female, ZSM (not examined but original description checked). Country of type locality: Austria.

**Geographical distribution.**PAL.

**PAL**: Austria, Germany.

***Microplitisconfusus* Muesebeck, 1922**

*Microplitisconfusus* Muesebeck, 1922.

**Type information.** Holotype male, USNM (not examined but original description checked). Country of type locality: USA.

**Geographical distribution.**NEA.

**NEA**: Canada (NB, ON), USA (IN, MD, MI, NY, TX).

***Microplitiscrassiantenna* Song & Chen, 2008**

*Microplitiscrassiantenna* Song & Chen, 2008.

**Type information.** Holotype female, HUNAU (not examined but original description checked). Country of type locality: China.

**Geographical distribution.**PAL.

**PAL**: China (JL).

***Microplitiscrassifemoralis* Alexeev, 1971**

*Microplitiscrassifemoralis* Alexeev, 1971.

**Type information.** Holotype female, ZIN (not examined but original description checked). Country of type locality: Turkmenistan.

**Geographical distribution.**PAL.

**PAL**: Turkmenistan.

***Microplitiscrenulatus* (Provancher, 1888)**

*Microgastercrenulatus* Provancher, 1888.

**Type information.** Lectotype female, ULQC (examined). Country of type locality: Canada.

**Geographical distribution.**NEA.

**NEA**: Canada (QC), USA (MA, MI).

***Microplitiscroceipes* (Cresson, 1872)**

*Microgastercroceipes* Cresson, 1872.

*Microplitisnigripennis* Ashmead, 1905.

**Type information.** Holotype female, ANSP (not examined but subsequent treatment of the species checked). Country of type locality: USA.

**Geographical distribution.**AUS, NEA.

**AUS**: New Zealand; **NEA**: USA (AL, AZ, AR, CO, GA, IL, KS, MD, MS, MO, NJ, NM, NC, OK, OR, SC, TN, UT, VA).

**Notes.** Our species concept is based on Muesebeck (1922), Papp (1986) and Papp and Shaw (2001).

***Microplitiscubitellanus* Xu & He, 2000**

*Microplitiscubitellanus* Xu & He, 2000.

**Type information.** Holotype male, ZJUH (not examined but original description checked). Country of type locality: China.

**Geographical distribution.**PAL.

**PAL**: China (JL, XJ).

***Microplitisdaitojimensis* Sonan, 1940**

*Microplitisdaitojimensis* Sonan, 1940.

**Type information.** Type and depository unknown (not examined). Country of type locality: Ryukyu Islands.

**Geographical distribution.**OTL.

**OTL**: Ryukyu Islands.

***Microplitisdecens* Tobias, 1964**

*Microplitisdecens* Tobias, 1964.

**Type information.** Holotype female, ZIN (not examined but subsequent treatment of the species checked). Country of type locality: Kazakhstan.

**Geographical distribution.**PAL.

**PAL**: Finland, Germany, Hungary, Italy, Kazakhstan, Korea, Mongolia, Montenegro, Netherlands, Russia (S), Serbia, Spain, Switzerland, Turkey, United Kingdom.

**Notes.** The presence of this species in the United Kingdom has been questioned by Shaw (2012b).

***Microplitisdecipiens* Prell, 1925**

*Microplitisdecipiens* Prell, 1925.

**Type information.** Lectotype female, TUDTG (not examined but subsequent treatment of the species checked). Country of type locality: Germany.

**Geographical distribution.**PAL.

**PAL**: Azerbaijan, Germany, Hungary, Iran, Kazakhstan, Lithuania, Moldova, Poland, Russia (C, NW), Turkey.

**Notes.** Our species concept is based on Papp (1984c). The depository acronym (TUDTG) is based on the institution name: Technische Universität Dresden, Department of Forest Science in Tharandt, Germany.

***Microplitisdemolitor* Wilkinson, 1934**

*Microplitisdemolitor* Wilkinson, 1934.

**Type information.** Holotype female, NHMUK (examined). Country of type locality: Australia.

**Geographical distribution.**AUS, OTL.

**AUS**: Australia (NSW, NT, QLD, SA, WA); **OTL**: India, Pakistan, Vietnam.

***Microplitisdeprimator* (Fabricius, 1798)**

*Ichneumondeprimator* Fabricius, 1798.

*Microgasteringratus* Haliday, 1834.

*Microgasterdeprimatrix* Schulz, 1906.

**Type information.** Lectotype male, ZMUC (not examined but subsequent treatment of the species checked). Country of type locality: Germany.

**Geographical distribution.**PAL.

**PAL**: Armenia, Austria, Azerbaijan, Belgium, Bulgaria, Cyprus, Czech Republic, Finland, France, Georgia, Germany, Hungary, Iran, Ireland, Italy, Kazakhstan, Korea, Latvia, Moldova, Mongolia, Netherlands, Norway, Poland, Romania, Russia (DA, KYA, RYA, SPE, SAR), Serbia, Spain, Switzerland, Turkey, Ukraine, United Kingdom.

**Notes.** Our species concept is based on Papp (1984c) and Kotenko (2007a). The species distribution in Kazakhstan is based on Belokobylskij et al. (2019).

***Microplitisdesertorum* Telenga, 1955, restored combination**

*Microplitisdesertorum* Telenga, 1955.

**Type information.** Lectotype female, ZIN (not examined but original description checked). Country of type locality: Kazakhstan.

**Geographical distribution.**PAL.

**PAL**: Kazakhstan.

**Notes.** Our species concept is based on Telenga (1955), Papp (1984c) and Tobias (1986). Transferred back to *Microplitis* because of the short ovipositor and metatibial spurs shorter than half the length of first segment of metatarsus. The reference to this species as *Microgaster* in papers after the original description (e.g., Papp 1984c, Tobias 1986, Yu et al. 2012, 2016) is only due to the confusion with the application of the *Microgaster* name and its type species (see details above, under the introduction to the genus *Microgaster*; p 717).

***Microplitisdesertus* Alexeev, 1977**

*Microplitisdesertus* Alexeev, 1977.

**Type information.** Holotype female, ZIN (not examined but original description checked). Country of type locality: Turkmenistan.

**Geographical distribution.**PAL.

**PAL**: Turkmenistan.

***Microplitisdocilis* Nixon, 1970**

*Microplitisdocilis* Nixon, 1970.

**Type information.** Holotype female, MZH (not examined but original description checked). Country of type locality: Finland.

**Geographical distribution.**PAL.

**PAL**: Bulgaria, Croatia, Finland, Germany, Hungary, Russia (BU, PRI), Serbia, Sweden, Turkey.

***Microplitisdornator* (Papp, 1987)**

*Microgasterdornator* Papp, 1987.

**Type information.** Holotype female, HNHM (not examined but subsequent treatment of the species checked). Country of type locality: Korea.

**Geographical distribution.**PAL.

**PAL**: Korea, Russia (PRI).

**Notes.** Our species concept is based on Kotenko (2007a).

***Microplitiseminius* (Papp, 1987)**

*Microgastereminius* Papp, 1987.

**Type information.** Holotype female, HNHM (not examined but subsequent treatment of the species checked). Country of type locality: Korea.

**Geographical distribution.**PAL.

**PAL**: Korea.

**Notes.** Our species concept is based on Kotenko (2007a).

***Microplitiseremitus* Reinhard, 1880**

*Microplitiseremitus* Reinhard, 1880.

**Type information.** Lectotype male, ZMHB (not examined but subsequent treatment of the species checked). Country of type locality: Germany.

**Geographical distribution.**PAL.

**PAL**: Armenia, Austria, Azerbaijan, Croatia, Finland, France, Germany, Hungary, Kazakhstan, Korea, Lithuania, Mongolia, Netherlands, Poland, Russia (ZAB, IRK, PRI, SPE, VOR, YAR), Serbia, Spain, Sweden, Switzerland, Turkey, Ukraine, Uzbekistan.

**Notes.** Our species concept is based on Nixon (1970), Papp (1984c) and Kotenko (2007a).

***Microplitiserythrogaster* Abdinbekova, 1969**

*Microplitiserythrogaster* Abdinbekova, 1969.

**Type information.** Holotype female, ZIN (not examined but subsequent treatment of the species checked). Country of type locality: Azerbaijan.

**Geographical distribution.**PAL.

**PAL**: Azerbaijan, Denmark, Germany, Hungary, Russia (NC, S), Tajikistan, Turkmenistan.

**Notes.** Our species concept is based on Papp (1984c) and Tobias (1986).

***Microplitisespinachi* Walker, 2003**

*Microplitisespinachi* Walker, 2003.

**Type information.** Holotype female, NHMUK (examined). Country of type locality: Costa Rica.

**Geographical distribution.**NEO.

**NEO**: Costa Rica.

**Notes.**Janzen et al. (2003) indicated that the holotype and an unspecified number of paratypes for *M.espinachi* were deposited in the USNM. However, Fernandez-Triana et al. (2015b) could not locate any of the specimens in that collection, unit trays did not exist for any specimen of *M.espinachi* in the USNM, and there was no record of their existence in any USNM database. Because of that, Fernandez-Triana et al. (2015b) considered it unlikely that specimens of this species were ever deposited in the USNM and they speculated that the type might be misplaced or lost. The finding of the holotype for this species in London (NHMUK) is thus very important as it clarifies its situation. Also, it will allow for future studies about the limits between *M.espinachi* and *M.adrianguadamuzi* Fernandez-Triana & Whitfield, and the validity of the latter species, something that was not possible until the *M.figueresi* Walker type series was also found (see Fernandez-Triana et al. 2015b for more details about these three species).

***Microplitisexcisus* Telenga, 1955**

*Microplitisexcisus* Telenga, 1955.

**Type information.** Lectotype female, ZIN (not examined but original description checked). Country of type locality: Ukraine.

**Geographical distribution.**PAL.

**PAL**: Azerbaijan, Russia (NC, S), Ukraine.

***Microplitisfeltiae* Muesebeck, 1922**

*Microplitisfeltiae* Muesebeck, 1922.

**Type information.** Holotype male, USNM (not examined but original description checked). Country of type locality: USA.

**Geographical distribution.**NEA.

**NEA**: USA (AL, AZ, CA, CO, ID, IL, IN, KS, LA, MO, OK, TN, TX, WA).

***Microplitisfigueresi* Walker, 2003**

*Microplitisfigueresi* Walker, 2003.

**Type information.** Holotype female, NHMUK (examined). Country of type locality: Costa Rica.

**Geographical distribution.**NEO.

**NEO**: Costa Rica.

**Notes.** Until this paper, the type of *M.figueresi* was considered lost or misplaced (Fernandez-Triana et al. 2015b). The finding of the holotype for this species in London (NHMUK) is thus very important as it clarifies its situation, and will allow for future studies about the limits between *M.espinachi* Walker and *M.adrianguadamuzi* Fernandez-Triana & Whitfield, and the validity of the latter species, something that was not possible until the *M.figueresi* Walker type series was found. See Fernandez-Triana et al. (2015b) for more details about these three species, and also comments above, under *Microplitisespinachi*.

***Microplitisflavipalpis* (Brullé, 1832)**

*Microgasterflavipalpis* Brullé, 1832.

*Microplitisruricola* Lyle, 1918.

**Type information.** Lectotype male, MNHN (not examined but authoritatively identified specimens examined). Country of type locality: Greece.

**Geographical distribution.**PAL.

**PAL**: Algeria, Armenia, Bulgaria, Finland, France, Germany, Greece, Hungary, Israel, Kazakhstan, Korea, Lithuania, Moldova, Mongolia, Poland, Russia (ZAB, PRI), Serbia, Slovakia, Spain, Switzerland, Tunisia, Turkey, United Kingdom.

**Notes.** We examined the type of *Microplitisruricola* Lyle, 1918. The species distribution in Israel is based on Belokobylskij et al. (2019).

***Microplitisfordi* Nixon, 1970**

*Microplitisfordi* Nixon, 1970.

**Type information.** Holotype female, NHMUK (examined). Country of type locality: United Kingdom.

**Geographical distribution.**PAL.

**PAL**: Austria, Bulgaria, Croatia, Czech Republic, Germany, Greece, Hungary, Israel, Italy, Jordan, Macedonia, Mongolia, Russia (C), Switzerland, Tunisia, Turkey, United Kingdom, Yugoslavia.

**Notes.**Papp (1984c) suggested that this species might be a junior synonym of *Microplitissemicircularis* (Ratzeburg, 1834), but nevertheless retained the species as valid (also Broad et al. 2016), a decision we follow here.

***Microplitisfrancopupulini* Fernandez-Triana & Whitfield, 2015**

*Microplitisfrancopupulini* Fernandez-Triana & Whitfield, 2015.

**Type information.** Holotype female, USNM (examined). Country of type locality: Costa Rica.

**Geographical distribution.**NEO.

**NEO**: Costa Rica.

***Microplitisfraudulentus* (Papp, 1984)**

*Microgasterfraudulenta* Papp, 1984.

**Type information.** Holotype female, HNHM (not examined but original description checked). Country of type locality: Mongolia.

**Geographical distribution.**PAL.

**PAL**: Mongolia, Russia (ZAB).

***Microplitisfujianicus* Song & Zhang, 2017**

*Microplitisfujianica* Song & Zhang, 2017.

**Type information.** Holotype female, FAFU (not examined but original description checked). Country of type locality: China.

**Geographical distribution.**OTL.

**OTL**: China (FJ).

**Notes.** The species name must be treated as an adjective and not as a noun (Doug Yanega, pers. comm.) and thus it must match the gender of the genus name.

***Microplitisfulvicornis* (Wesmael, 1837)**

*Microgasterfulvicornis* Wesmael, 1837.

*Microplitispallidicornis* Marshall, 1898.

**Type information.** Lectotype male, RBINS (not examined but subsequent treatment of the species checked). Country of type locality: Belgium.

**Geographical distribution.**PAL.

**PAL**: Belgium, Croatia, Czech Republic, Finland, Germany, Hungary, Iran, Ireland, Netherlands, Poland, Romania, Russia (RYA, SAR), Serbia, Slovakia, Switzerland, Turkey, United Kingdom.

**Notes.** Our species concept is based on Papp (1984c) and Tobias (1986).

***Microplitisgalinarius* Kotenko, 2007**

*Microplitisgalinarius* Kotenko, 2007.

**Type information.** Holotype female, SIZK (not examined but original description checked). Country of type locality: Russia.

**Geographical distribution.**PAL.

**PAL**: Russia (ZAB).

***Microplitisgerulus* Papp, 1980, restored combination**

*Microplitisgerulus* Papp, 1980.

**Type information.** Holotype female, HNHM (not examined but subsequent treatment of the species checked). Country of type locality: Mongolia.

**Geographical distribution.**PAL.

**PAL**: Mongolia.

**Notes.** Our species concept is based on Papp (1984c) and Tobias (1986). This species was at times considered to belong to *Microgaster*, e.g., Papp (1984c) and Tobias (1986), as part of the confusion with the application and use of the *Microplitis* and *Microgaster* names (which was only solved after 1988, see more details and comments under our introduction to the genus *Microgaster* above). The correct generic placement at present would be in *Microplitis*, which is also corroborated by the description and images in Papp (1984c). Because some of the more recent references (e.g., Kotenko 2007a, Yu et al. 2016) still refer to the species within *Microgaster*, for the sake of clarity we restore its status here.

***Microplitisgidjus* Austin & Dangerfield, 1993**

*Microplitisgidjus* Austin & Dangerfield, 1993.

**Type information.** Holotype female, AEIC (not examined but original description checked). Country of type locality: Australia.

**Geographical distribution.**AUS, OTL.

**AUS**: Australia (NT); **OTL**: Vietnam.

***Microplitisglabrior* Alexeev, 1971, restored combination**

*Microplitisglabrior* Alexeev, 1971.

**Type information.** Holotype female, ZIN (not examined but original description checked). Country of type locality: Turkmenistan.

**Geographical distribution.**PAL.

**PAL**: Turkmenistan.

**Notes.** Our species concept is based on Alexeev (1971), Papp (1984c), and Tobias (1986). This species was at times considered to belong to *Microgaster*, e.g., Papp (1984c) and Tobias (1986), as part of the confusion with the application and use of the *Microplitis* and *Microgaster* names (which was only solved after 1988, see more details and comments under our introduction to the genus *Microgaster* above). The correct generic placement at present would be in *Microplitis*, which is also corroborated by the description and images in Alexeev (1971) and Papp (1984c). Because some of the more recent references (e.g., Yu et al. 2016) still refer to the species within *Microgaster*, for the sake of clarity we restore its status here.

***Microplitisgortynae* Riley, 1881**

*Microplitisgortynae* Riley, 1881.

**Type information.** Syntypes female and male, USNM (examined). Country of type locality: USA.

**Geographical distribution.**NEA.

**NEA**: Canada (ON), USA (CO, IL, IA, KS, MS, MO, NH, NJ, NY, OH, OR, PA, VA, WI).

***Microplitisgoughi* Austin & Dangerfield, 1993**

*Microplitisgoughi* Austin & Dangerfield, 1993.

**Type information.** Holotype female, ANIC (not examined but original description checked). Country of type locality: Australia.

**Geographical distribution.**AUS.

**AUS**: Australia (ACT, NSW, SA, TAS, VIC, WA).

***Microplitishebertbakeri* Fernandez-Triana & Whitfield, 2015**

*Microplitishebertbakeri* Fernandez-Triana & Whitfield, 2015.

**Type information.** Holotype female, USNM (examined). Country of type locality: Costa Rica.

**Geographical distribution.**NEO.

**NEO**: Costa Rica.

***Microplitishelicoverpae* Xu & He, 2000**

*Microplitishelicoverpae* Xu & He, 2000.

**Type information.** Holotype male, ZJUH (not examined but subsequent treatment of the species checked). Country of type locality: China.

**Geographical distribution.**OTL, PAL.

**OTL**: China (HB); **PAL**: China (HE).

**Notes.** Our species concept is based on Chen and Song (2004).

***Microplitisheterocerus* (Ruthe, 1860)**

*Microgasterheterocerus* Ruthe, 1860.

**Type information.** Holotype female, NHMUK (examined). Country of type locality: Germany.

**Geographical distribution.**PAL.

**PAL**: Croatia, Germany, Hungary, Israel, Italy, Korea, Poland, Romania, Russia (IN, ROS, VGG), Slovakia, Spain, Turkey, Ukraine, Yugoslavia.

**Notes.** The holotype is missing the metasoma.

***Microplitishirtifacialis* Song & You, 2008**

*Microplitishirtifacialis* Song & You, 2008.

**Type information.** Holotype female, FAFU (not examined but original description checked). Country of type locality: China.

**Geographical distribution.**OTL, PAL.

**OTL**: China (HB); **PAL**: China (JL).

**Notes.** The shape and sculpture of T2 is uncommon for *Microplitis*, but until specimens can be examined, we prefer to retain the species in this genus.

***Microplitishispalensis* Marshall, 1898**

*Microplitishispalensis* Marshall, 1898.

*Microgasterserotinus* Papp, 1984.

**Type information.** Lectotype female, MNCN (not examined but subsequent treatment of the species checked). Country of type locality: Spain.

**Geographical distribution.**PAL.

**PAL**: France, Spain.

**Notes.** Our species concept is based on Papp (1984c) and Shaw (2012b).

***Microplitishova* Granger, 1949**

*Microplitishova* Granger, 1949.

**Type information.** Syntypes female and male, MNHN (not examined but original description checked). Country of type locality: Madagascar.

**Geographical distribution.**AFR.

**AFR**: Madagascar.

***Microplitishyalinipennis* Alexeev, 1971**

*Microplitishyalinipennis* Alexeev, 1971.

**Type information.** Holotype male, ZIN (not examined but original description checked). Country of type locality: Turkmenistan.

**Geographical distribution.**PAL.

**PAL**: Turkmenistan.

***Microplitishyphantriae* Ashmead, 1898**

*Microplitishyphantiae* Ashmead, 1898.

*Microplitishyphantiae* Ashmead, 1898 [incorrect original spelling].

**Type information.** Holotype female, USNM (examined). Country of type locality: USA.

**Geographical distribution.**NEA.

**NEA**: Canada (AB, ON, QC), USA (AR, IL, IN, MD, MA, MI, MO, NH, NJ, NY, OH, TX, WV).

**Notes.**Yu et al. (2012, 2016) listed the holotype of this species as being in the INHS collection. However, we have examined it in the USNM, which should be recorded as the correct depository for the holotype.

***Microplitisidia* Nixon, 1970**

*Microplitisidia* Nixon, 1970.

**Type information.** Holotype female, NHMUK (examined). Country of type locality: Sweden.

**Geographical distribution.**PAL.

**PAL**: Germany, Hungary, Israel, Russia (NW), Sweden, Turkey.

***Microplitisimpressus* (Wesmael, 1837)**

*Microgasterimpressus* Wesmael, 1837.

*Microgastersispes* Nixon, 1970.

**Type information.** Lectotype male, RBINS (not examined but authoritatively identified specimens examined). Country of type locality: Belgium.

**Geographical distribution.**NEA, PAL.

**NEA**: Canada (MB, ON, QC); **PAL**: Belgium, France, Germany, Hungary, Poland, Slovakia, United Kingdom.

**Notes.** We have examined the type of *Microgastersispes* Nixon.

***Microplitisimprovisus* (Papp, 1984)**

*Microgasterimprovisa* Papp, 1984.

**Type information.** Holotype female, RMNH (not examined but paratype examined). Country of type locality: Netherlands.

**Geographical distribution.**PAL.

**PAL**: Netherlands.

**Notes.** We examined female and male paratypes.

***Microplitisincurvatus* Xu & He, 2002**

*Microplitisincurvata* Xu & He, 2002.

**Type information.** Holotype female, ZJUH (not examined but original description checked). Country of type locality: China.

**Geographical distribution.**PAL.

**PAL**: China (XJ).

***Microplitisindicus* Marsh, 1978**

*Microplitisindicus* Marsh, 1978.

**Type information.** Holotype female, USNM (not examined but original description checked). Country of type locality: India.

**Geographical distribution.**OTL.

**OTL**: India.

***Microplitisinfula* (Kotenko, 1994)**

*Microgasterinfula* Kotenko, 1994.

**Type information.** Holotype female, SIZK (not examined but subsequent treatment of the species checked). Country of type locality: Russia.

**Geographical distribution.**PAL.

**PAL**: Russia (ZAB, PRI).

**Notes.** Our species concept is based on Kotenko (2006, 2007).

***Microplitisjamesi* Austin & Dangerfield, 1993**

*Microplitisjamesi* Austin & Dangerfield, 1993.

**Type information.** Holotype female, ANIC (not examined but original description checked). Country of type locality: Australia.

**Geographical distribution.**AUS.

**AUS**: Australia (NSW).

***Microplitisjiangsuensis* Xu & He, 2000**

*Microplitisjiangsuensis* Xu & He, 2000.

**Type information.** Holotype male, ZJUH (not examined but original description checked). Country of type locality: China.

**Geographical distribution.**PAL.

**PAL**: China (JS).

***Microplitisjorgehernandezi* Fernandez-Triana & Whitfield, 2015**

*Microplitisjorgehernandezi* Fernandez-Triana & Whitfield, 2015.

**Type information.** Holotype female, USNM (examined). Country of type locality: Costa Rica.

**Geographical distribution.**NEO.

**NEO**: Costa Rica.

***Microplitisjorgeluisi* Fernandez-Triana, 2018**

*Microplitisjorgeluisi* Fernandez-Triana, 2018.

**Type information.** Holotype female, CNC (examined). Country of type locality: USA.

**Geographical distribution.**NEA.

**NEA**: USA (TX).

***Microplitisjuanmanueli* Fernandez-Triana, 2018**

*Microplitisjuanmanueli* Fernandez-Triana, 2018.

**Type information.** Holotype female, CNC (examined). Country of type locality: USA.

**Geographical distribution.**NEA.

**NEA**: Canada (BC), USA (CO).

***Microplitisjulioalbertoi* Fernandez-Triana, 2018**

*Microplitisjulioalbertoi* Fernandez-Triana, 2018.

**Type information.** Holotype female, CNC (examined). Country of type locality: USA.

**Geographical distribution.**NEA.

**NEA**: USA (CT, GA).

***Microplitiskarakurti* Rossikov, 1904**

*Microplitiskara-kurti* Rossikov, 1904.

**Type information.** Type and depository unknown (not examined). Country of type locality: unknown.

**Geographical distribution.**PAL.

**PAL**: Kazakhstan, Turkmenistan.

***Microplitiskaszabi* Papp, 1980**

*Microplitiskaszabi* Papp, 1980.

**Type information.** Holotype female, HNHM (not examined but subsequent treatment of the species checked). Country of type locality: Mongolia.

**Geographical distribution.**PAL.

**PAL**: Korea, Mongolia, Russia (PRI).

**Notes.** Our species concept is based on Papp (1984c) and Kotenko (2007a).

***Microplitiskewleyi* Muesebeck, 1922**

*Microplitiskewleyi* Muesebeck, 1922.

**Type information.** Holotype female, USNM (not examined but original description checked). Country of type locality: USA.

**Geographical distribution.**NEA.

**NEA**: Canada (AB, MB, NB, NL, NS, ON, PE, QC), USA (CA, DC, IA, MD, MI, NJ, NY, WI).

***Microplitiskurandensis* Austin & Dangerfield, 1993**

*Microplitiskurandensis* Austin & Dangerfield, 1993.

**Type information.** Holotype male, ANIC (not examined but original description checked). Country of type locality: Australia.

**Geographical distribution.**AUS.

**AUS**: Australia (QLD).

***Microplitislacteus* Austin & Dangerfield, 1993**

*Microplitislacteus* Austin & Dangerfield, 1993.

**Type information.** Holotype female, CNC (examined). Country of type locality: Papua New Guinea.

**Geographical distribution.**AUS.

**AUS**: Papua New Guinea.

***Microplitislaticinctus* Muesebeck, 1922**

*Microplitislaticinctus* Muesebeck, 1922.

**Type information.** Holotype female, USNM (not examined but original description checked). Country of type locality: USA.

**Geographical distribution.**NEA.

**NEA**: Canada (QC), USA (AL, DC, IL, IA, MA, NY, OH, VA).

***Microplitislatistigmus* Muesebeck, 1922**

*Microplitislatistigmus* Muesebeck, 1922.

**Type information.** Holotype female, USNM (not examined but original description checked). Country of type locality: USA.

**Geographical distribution.**NEA.

**NEA**: USA (MD).

***Microplitisleoniae* Niezabitowski, 1910**

*Microplitisleoniae* Niezabitowski, 1910.

**Type information.** Syntypes female and male, depository unknown (not examined but subsequent treatment of the species checked). Country of type locality: Poland.

**Geographical distribution.**PAL.

**PAL**: Georgia, Hungary, Korea, Poland, Russia (PRI).

**Notes.** Our species concept is based on Papp (1984c) and Kotenko (2007a). The species distribution in Korea is based on Belokobylskij et al. (2019).

***Microplitisleucaniae* Xu & He, 2002**

*Microplitisleucaniae* Xu & He, 2002.

**Type information.** Holotype female, ZJUH (not examined but subsequent treatment of the species checked). Country of type locality: China.

**Geographical distribution.**OTL, PAL.

**OTL**: China (FJ, GX, JS, ZJ); **PAL**: China (XJ).

**Notes.** Our species concept is based on Chen and Song (2004), Kotenko (2007a) and Ranjith et al. (2015a).

***Microplitislineatus* Austin & Dangerfield, 1993**

*Microplitislineatus* Austin & Dangerfield, 1993.

**Type information.** Holotype female, AEIC (not examined but original description checked). Country of type locality: Papua New Guinea.

**Geographical distribution.**AUS.

**AUS**: Papua New Guinea.

***Microplitislongicaudus* Muesebeck, 1922**

*Microplitislongicaudus* Muesebeck, 1922.

**Type information.** Holotype female, USNM (not examined but original description checked). Country of type locality: USA.

**Geographical distribution.**NEA.

**NEA**: USA (CA, CO, ID, NV, OR).

***Microplitislongiradiusis* Xu & He, 2003**

*Microplitislongiradiusis* Xu & He, 2003.

**Type information.** Holotype female, ZJUH (not examined but original description checked). Country of type locality: China.

**Geographical distribution.**PAL.

**PAL**: China (HL).

***Microplitislongwangshanus* Xu & He, 2000**

*Microplitislongwangshana* Xu & He, 2000.

**Type information.** Holotype female, ZJUH (not examined but subsequent treatment of the species checked). Country of type locality: China.

**Geographical distribution.**OTL.

**OTL**: China (FJ, HB, ZJ).

**Notes.** Our species concept is based on Chen and Song (2004) and Ranjith et al. (2015a).

***Microplitislugubris* (Ruthe, 1860)**

*Microgasterlugubris* Ruthe, 1860.

*Microplitisborealis* Marshall, 1885.

*Microgastercoracinus* Thomson, 1895.

*Microplitisrutheana* Fahringer, 1937.

**Type information.** Holotype female, NHMUK (examined). Country of type locality: Poland.

**Geographical distribution.**NEA, PAL.

**NEA**: Canada (MB, NU), Greenland; **PAL**: Armenia, Finland, Germany, Hungary, Ireland, Lithuania, Mongolia, Poland, Russia (TA, YAR), Serbia, Sweden, Switzerland, Turkey, United Kingdom.

**Notes.** We also examined the type of *M.borealis* Marshall. A new distribution record (from Canada, MB, Churchill, at ca. 59° N), which had been named *Microplitis* jft01 in previous papers (Fernandez-Triana 2010, Fernandez-Triana et al. 2011) expands considerably the southernmost distribution of the species within the Nearctic region.

***Microplitislugubroides* van Achterberg, 2006**

*Microplitislugubroides* van Achterberg, 2006.

**Type information.** Holotype female, ZMUC (not examined but original description checked). Country of type locality: Greenland.

**Geographical distribution.**NEA.

**NEA**: Greenland.

***Microplitismahunkai* (Papp, 1979)**

*Glabromicroplitismahunkai* Papp, 1979.

**Type information.** Holotype female, HNHM (not examined but original description checked). Country of type locality: Tunisia.

**Geographical distribution.**PAL.

**PAL**: Tunisia.

***Microplitismalimba* (Papp, 1984)**

*Microgastermalimba* Papp, 1984.

**Type information.** Holotype female, RMNH (not examined but paratype examined). Country of type locality: Netherlands.

**Geographical distribution.**PAL.

**PAL**: Netherlands, Russia (PRI), Ukraine, United Kingdom.

**Notes.** According to Shaw (2012b) and Broad et al. (2016), the interpretation by (Nixon 1970) of *Microplitistrochanterata* (not *tuberculifer*, of which *trochanterata* is a junior synonym, see below under that species in our checklist) is actually referable to *malimba*. Because the name is to be considered as a noun under ICZN Article 31.2.1, it must retain its original spelling and remain as *malimba*. We examined one female paratype.

***Microplitismamestrae* Weed, 1887**

*Microplitismamestrae* Weed, 1887.

**Type information.** Lectotype female, INHS (not examined but subsequent treatment of the species checked). Country of type locality: USA.

**Geographical distribution.**NEA.

**NEA**: Canada (BC), USA (CT, DC, IL, KS, MA, MI, NH, NJ, NM, NY, OH, UT, WI).

**Notes.** Our species concept is based on Muesebeck (1922) and Whitfield (1995a).

***Microplitismandibularis* (Thomson, 1895)**

*Microgastermandibularis* Thomson, 1895.

**Type information.** Lectotype female, MZLU (not examined but subsequent treatment of the species checked). Country of type locality: Sweden.

**Geographical distribution.**NEA, PAL.

**NEA**: Greenland; **PAL**: Armenia, Azerbaijan, Croatia, Finland, Georgia, Germany, Hungary, Macedonia, Mongolia, Netherlands, Russia (PRI, SAK), Serbia, Slovakia, Spain, Sweden, Switzerland, Tunisia, Turkey, United Kingdom.

**Notes.** Our species concept is based on van Achterberg (2006) and Kotenko (2007a). The species distribution in Armenia, Azerbaijan and Georgia is based on Belokobylskij et al. (2019).

***Microplitismanilae* Ashmead, 1904**

*Microplitismanilae* Ashmead, 1904.

**Type information.** Holotype female, USNM (not examined but subsequent treatment of the species checked). Country of type locality: Philippines.

**Geographical distribution.**AUS, OTL, PAL.

**AUS**: Australia (QLD), Papua New Guinea; **OTL**: China (GD, TW, ZJ), India, Malaysia, Philippines, Ryukyu Islands, Thailand, Vietnam; **PAL**: Korea.

**Notes.** Our species concept is based on Austin and Dangerfield (1993), Gupta (2013a) and Ranjith et al. (2015a).

***Microplitismariamargaritae* Fernandez-Triana, 2018**

*Microplitismariamargaritae* Fernandez-Triana, 2018.

**Type information.** Holotype female, CNC (examined). Country of type locality: USA.

**Geographical distribution.**NEA.

**NEA**: USA (CO).

***Microplitismarini* Whitfield, 2003**

*Microplitismarini* Whitfield, 2003.

**Type information.** Holotype female, USNM (examined). Country of type locality: Costa Rica.

**Geographical distribution.**NEA, NEO.

**NEA**: USA (AZ); **NEO**: Costa Rica.

**Notes.** The record of this species from Arizona (US) was questioned by Fernandez-Triana et al. (2015b) as the available information suggests it may represent a different (most likely undescribed) species. However, to conclude, examination of the US specimen would be needed; thus, for the time being they are listed under *marini*.

***Microplitismarshallii* Kokujev, 1898**

*Microplitismarshallii* Kokujev, 1898.

**Type information.** Lectotype female, ZIN (not examined but subsequent treatment of the species checked). Country of type locality: Georgia.

**Geographical distribution.**OTL, PAL.

**OTL**: China (FJ, HB, YN); **PAL**: Armenia, Azerbaijan, China (JL), Finland, Georgia, Hungary, Iran, Moldova, Romania, Russia (KEM, ROS, STA), Turkey.

**Notes.** Our species concept is based on Papp (1984c), Chen and Song (2004), Kotenko (2007a) and Ranjith et al. (2015a).

***Microplitismasneri* Austin & Dangerfield, 1993**

*Microplitismasneri* Austin & Dangerfield, 1993.

**Type information.** Holotype female, ANIC (not examined but original description checked). Country of type locality: Australia.

**Geographical distribution.**AUS.

**AUS**: Australia (QLD).

***Microplitismaturus* Weed, 1888**

*Microplitismaturus* Weed, 1888.

*Microplitiscincta* Ashmead, 1891.

*Microgastertuckeri* Viereck, 1905.

**Type information.** Holotype male, ANSP (not examined but subsequent treatment of the species checked). Country of type locality: USA.

**Geographical distribution.**NEA.

**NEA**: Canada (BC, ON, QC), USA (AR, CT, FL, GA, IL, IA, KS, LA, MD, MI, MO, NJ, NY, SD, VT).

**Notes.** The information about the holotype is taken from Shenefelt (1973: 750). We have also examined the holotype of *M.cincta* in the USNM, a male specimen.

***Microplitismediator* (Haliday, 1834)**

*Microgastermediator* Haliday, 1834.

*Microgastermedianus* Ruthe, 1860 [primary homonym of *Microgastermedianus* Ratzeburg, 1852].

*Microplitishalidayi* Fahringer, 1937.

*Microplitispseudomedianus* Fahringer, 1937.

**Type information.** Lectotype male, NMID (examined). Country of type locality: unknown.

**Geographical distribution.**NEA, NEO, OTL, PAL.

**NEA**: Greenland, **NEO**: Brazil (PR); **OTL**: China (HN, JS, ZJ), Pakistan; **PAL**: Albania, Armenia, Austria, Azerbaijan, Belgium, Bulgaria, China (HE, HL, HA, LN, NM, SD, SN, XJ), Croatia, Czech Republic, Finland, France, Georgia, Germany, Hungary, Iran, Ireland, Italy, Japan, Kazakhstan, Korea, Latvia, Lithuania, Moldova, Mongolia, Netherlands, Norway, Poland, Romania, Russia (AST, ZAB, KIR, KDA, MOS, ORE, PRI, RYA, SAK, SAR, STA, YAR), Serbia, Slovakia, Slovenia, Spain, Sweden, Switzerland, Turkey, Ukraine, United Kingdom, Uzbekistan.

**Notes.**van Achterberg (1997) revised the Haliday collection of Braconidae and designated a lectotype for *Microplitismediator*. Unfortunately, the type locality or country for the lectotype specimen are not clearly specified (van Achterberg 1997: 57). Based on the first few sections of van Achterberg’s paper (where he detailed the process he used to recognize Haliday’s type specimens, publication dates, and list of taxa described), Ireland seems to be the most likely country of the lectotype. We also examined the type of *Microgastermedianus* Ruthe. The species distribution in Armenia, Georgia, and Iran is based on Belokobylskij et al. (2019).

***Microplitismelianae* Viereck, 1911**

*Microplitismelianae* Viereck, 1911.

**Type information.** Holotype male, USNM (examined). Country of type locality: USA.

**Geographical distribution.**NEA.

**NEA**: Canada (AB, ON), USA (IL, IA, KS, NY, OH, OK, TN, TX).

***Microplitismencianus* Xu & He, 1999**

*Microplitismenciana* Xu & He, 1999.

**Type information.** Holotype female, ZJUH (not examined but original description checked). Country of type locality: China.

**Geographical distribution.**PAL.

**PAL**: China (HL).

**Notes.** The species name must be treated as an adjective and not as a noun (Doug Yanega, pers. comm.) and thus it must match the gender of the genus name.

***Microplitismexicanus* (Cameron, 1887), new combination**

*Microgastermexicana* Cameron, 1887.

**Type information.** Holotype female, NHMUK (examined). Country of type locality: Mexico.

**Geographical distribution.**NEO.

**NEO**: Mexico.

**Notes.** After the original description, the only taxonomist that has commented on this species was Muesebeck (1922: 42). He stated that he did not know that species but guessed that it did not belong to *Microgaster*, and then correctly guessed that it could be *Microplitis*, based on the description from Cameron. After examining the female holotype, we formally transfer the species here to *Microplitis*, based on its inflexible hypopygium, very short ovipositor sheaths, T1 very narrow and with polished knob at apex, T2 subtriangular, and metatibial spurs shorter than half the length of the first metatarsus segment.

***Microplitisminutus* Alexeev, 1977**

*Microplitisminutus* Alexeev, 1977.

**Type information.** Holotype female, ZIN (not examined). Country of type locality: Turkmenistan.

**Geographical distribution.**PAL.

**PAL**: Turkmenistan.

***Microplitismoestus* (Ratzeburg, 1852)**

*Microgaster möstus* Ratzeburg, 1852.

*Microplitismaestus* Dalla Torre, 1898 [unjustified emendation].

**Type information.** Type and depository unknown (not examined but subsequent treatment of the species checked). Country of type locality: Germany.

**Geographical distribution.**PAL.

**PAL**: Austria, Germany, Hungary, Netherlands, United Kingdom.

**Notes.** Our species concept is based on Papp (1984c).

***Microplitismongolicus* Papp, 1967**

*Microplitismongolicus* Papp, 1967.

**Type information.** Holotype male, HNHM (not examined but subsequent treatment of the species checked). Country of type locality: Mongolia.

**Geographical distribution.**PAL.

**PAL**: Hungary, Israel, Jordan, Mongolia, Russia (ZAB).

**Notes.** Our species concept is based on Papp (1984c).

***Microplitismontanus* Muesebeck, 1922**

*Microplitismontanus* Muesebeck, 1922.

**Type information.** Holotype female, USNM (not examined but original description checked). Country of type locality: USA.

**Geographical distribution.**NEA.

**NEA**: USA (CA, MO, NV).

***Microplitismurkyi* Gupta, 2013**

*Microplitismurkyi* Gupta, 2013.

**Type information.** Holotype female, NBAIR (not examined but original description checked). Country of type locality: India.

**Geographical distribution.**OTL.

**OTL**: India.

***Microplitismurrayi* Austin & Dangerfield, 1993**

*Microplitismurrayi* Austin & Dangerfield, 1993.

**Type information.** Holotype female, ANIC (not examined but original description checked). Country of type locality: Australia.

**Geographical distribution.**AUS.

**AUS**: Australia (ACT, NSW, QLD, TAS, VIC, WA).

***Microplitisnaenia* Nixon, 1970**

*Microplitisnaenia* Nixon, 1970.

**Type information.** Holotype female, NHMUK (examined). Country of type locality: United Kingdom.

**Geographical distribution.**PAL.

**PAL**: Hungary, Russia (C, NW), Slovakia, Turkey, United Kingdom.

***Microplitisnarendrani* Ranjith & Nasser, 2015**

*Microplitisnarendrani* Ranjith & Nasser.

**Type information.** Holotype female, DZUC (not examined but original description checked). Country of type locality: India.

**Geographical distribution.**OTL.

**OTL**: India.

**Notes.** The depository acronym (DZUC) was selected based on Ranjith et al. (2015a) and not the Insect and Spider Collections of the World website, which lists a different institution under that same acronym.

***Microplitisnecopinatus* (Papp, 1984)**

*Microgasternecopinata* Papp, 1984.

**Type information.** Holotype female, HNHM (not examined but original description checked). Country of type locality: Finland.

**Geographical distribution.**PAL.

**PAL**: Finland.

***Microplitisnewguineaensis* Austin & Dangerfield, 1993**

*Microplitisnewguineaensis* Austin & Dangerfield, 1993.

**Type information.** Holotype female, CNC (examined). Country of type locality: Papua New Guinea.

**Geographical distribution.**AUS.

**AUS**: Papua New Guinea.

***Microplitisnielseni* Austin & Dangerfield, 1993**

*Microplitisnielseni* Austin & Dangerfield, 1993.

**Type information.** Holotype female, ANIC (not examined but original description checked). Country of type locality: Australia.

**Geographical distribution.**AUS.

**AUS**: Australia (WA).

***Microplitisnigrifemur* Xu & He, 2006**

*Microplitisnigrifemur* Xu & He, 2006.

**Type information.** Holotype female, ZJUH (not examined but original description checked). Country of type locality: China.

**Geographical distribution.**PAL.

**PAL**: China (HE).

***Microplitisnigritus* Muesebeck, 1922**

*Microplitisnigritus* Muesebeck, 1922.

**Type information.** Holotype female, USNM (not examined but original description checked). Country of type locality: USA.

**Geographical distribution.**NEA.

**NEA**: USA (CO).

***Microplitisobscuripennatus* Xu & He, 1999**

*Microplitisobscuripennatus* Xu & He, 1999.

**Type information.** Holotype female, ZJUH (not examined but original description checked). Country of type locality: China.

**Geographical distribution.**OTL.

**OTL**: China (FJ).

**Notes.** The illustration of the mesosoma and metasoma in the original description suggest that this species might belong to *Snellenius*, based on the deep notauli, the deeply impressed scutellar disc, and the shape of T1. However, the English translation (Xu and He 1999: 67, 68) that follows the Chinese description makes no mention of an epicnemial carina, the most distinguishing character of *Snellenius*; thus, we retain the species under the genus in which it was originally described.

***Microplitisocellatae* (Bouché, 1834)**

*Microgasterocellatae* Bouché, 1834.

*Microgastercanaliculatus* Wesmael, 1837.

**Type information.** Type and depository unknown (not examined but subsequent treatment of the species checked). Country of type locality: Germany.

**Geographical distribution.**OTL, PAL.

**OTL**: China (JS); **PAL**: Belgium, China (LN), Croatia, Czech Republic, Finland, France, Germany, Hungary, Italy, Japan, Moldova, Netherlands, Poland, Romania, Russia (ZAB, SAK), Serbia, Ukraine, United Kingdom.

**Notes.** Our species concept is based on Nixon (1970), Papp (1984c) and Kotenko (2007a).

***Microplitisochraceus* Szépligeti, 1896**

*Microplitisochraceus* Szépligeti, 1896.

*Microplitisflaviventris* Ivanov, 1898.

**Type information.** Holotype female, HNHM (not examined but subsequent treatment of the species checked). Country of type locality: Hungary.

**Geographical distribution.**PAL.

**PAL**: Azerbaijan, Greece, Hungary, Iran, Kazakhstan, Moldova, Mongolia, Romania, Russia (KDA, ROS), Ukraine, Uzbekistan.

**Notes.** Our species concept is based on Papp (1984c) and Kotenko (2007a).

***Microplitispaizhensis* Zhang, 2019**

*Microplitispaizhensis* Zhang, 2019.

**Type information.** Holotype female, FAFU (not examined but original description checked). Country of type locality: China.

**Geographical distribution.**PAL.

**PAL**: China (XZ).

***Microplitispallidipennis* Tobias, 1964**

*Microplitispallidipennis* Tobias, 1964.

**Type information.** Holotype male, ZIN (not examined but subsequent treatment of the species checked). Country of type locality: Kazakhstan.

**Geographical distribution.**PAL.

**PAL**: Kazakhstan, Mongolia, Russia (S).

**Notes.** Our species concept is based on Papp (1984c), Tobias (1986), and Kotenko (2007a).

***Microplitispallidipes* Szépligeti, 1902**

*Microplitispallidipes* Szépligeti, 1902.

**Type information.** Holotype male, HNHM (not examined but subsequent treatment of the species checked). Country of type locality: Singapore.

**Geographical distribution.**OTL, PAL.

**OTL**: China (FJ, HN, TW, ZJ), Singapore, Vietnam; **PAL**: China (SD), Korea, Russia (PRI, SAK).

**Notes.** Our species concept is based on Wilkinson (1930a), Long and Belokobylski (2004), and Kotenko (2007a).

***Microplitispellucidus* Telenga, 1955**

*Microplitispellucidus* Telenga, 1955.

**Type information.** Lectotype female, ZIN (not examined but subsequent treatment of the species checked). Country of type locality: Russia.

**Geographical distribution.**PAL.

**PAL**: Denmark, Germany, Hungary, Korea, Netherlands, Russia (ALT, PRI), Serbia.

**Notes.** Our species concept is based on Telenga (1955), Papp (1984c), Tobias (1986) and Kotenko (2007a).

***Microplitispennatulae* Ranjith & Nasser, 2015**

*Microplitispennatulae* Ranjith & Nasser.

**Type information.** Holotype female, DZUC (not examined but original description checked). Country of type locality: India.

**Geographical distribution.**OTL.

**OTL**: India.

***Microplitisperelegans* (Bingham, 1906)**

*Microgasterperelegans* Bingham, 1906.

**Type information.** Holotype female, OUMNH (not examined but subsequent treatment of the species checked). Country of type locality: Australia.

**Geographical distribution.**AUS.

**AUS**: Australia (NT, QLD, WA).

**Notes.** Our species concept is based on Austin and Dangerfield (1992, 1993).

***Microplitispipus* Austin & Dangerfield, 1993**

*Microplitispipus* Austin & Dangerfield, 1993.

**Type information.** Holotype male, ANIC (not examined but original description checked). Country of type locality: Australia.

**Geographical distribution.**AUS.

**AUS**: Australia (QLD).

***Microplitisplutellae* Muesebeck, 1922**

*Microplitisplutellae* Muesebeck, 1922.

**Type information.** Holotype female, USNM (not examined but original description checked). Country of type locality: USA.

**Geographical distribution.**NEA, OTL, PAL.

**NEA**: Canada (AB, ON, PE, QC, SK), USA (CA, CO, ID, IA, MI, MN, NY, ND, OH, SC, TX, UT); **OTL**: China (TW); **PAL**: Egypt, Russia (MUR).

***Microplitisprodeniae* Rao & Kurian, 1950**

*Microplitisprodeniae* Rao & Kurian, 1950.

*Microplitisbicoloratus* Chen, 2004 [*M.bicoloratus* Chen, 2004 is also a primary homonym of *Microplitisbicoloratus* Xu & He, 2003].

*Microplitiskovalevskayae* Kittel, 2016 [unnecessary replacement name for *M.bicoloratus* Chen, 2004].

**Type information.** Holotype female, NZSI (not examined but subsequent treatment of the species checked). Country of type locality: India.

**Geographical distribution.**OTL.

**OTL**: China (GD, GX), India, Vietnam.

**Notes.** Our species concept is based on Gupta (2013a), Gupta & Fernandez-Triana (2014), and Ranjith et al. (2015a). The status of *Microplitisbicoloratus* (Chen, 2004) as a synonym of *M.prodenia* Rao & Kurian, 1950, and as a junior synonym of *M.bicoloratus* Xu & He, 2003 was established by Zhang et al. (2017). Thus, there is no need to replace the name *Microplitisbicoloratus* (Chen, 2004) with *Microplitiskovalevskayae* Kittel, 2016 as proposed by Kittel (2016).

***Microplitispseudomurinus* Abdinbekova, 1969**

*Microplitispseudomurina* Abdinbekova, 1969.

**Type information.** Holotype female, ZIN (not examined but subsequent treatment of the species checked). Country of type locality: Azerbaijan.

**Geographical distribution.**PAL.

**PAL**: Azerbaijan, Bulgaria, Georgia, Greece, Hungary, Kazakhstan, Moldova, Russia (ZAB, PRI), Turkey.

**Notes.** Our species concept is based on Papp (1984c), Tobias (1986), and Kotenko (2007a). The species name must be treated as an adjective and not as a noun (Doug Yanega, pers. comm.) and must match the gender of the genus name.

***Microplitispseudoochraceus* Alexeev, 1977**

*Microplitispseudoochraceus* Alexeev, 1977.

**Type information.** Holotype female, ZIN (not examined but original description checked). Country of type locality: Turkmenistan.

**Geographical distribution.**PAL.

**PAL**: Turkmenistan.

***Microplitisquadridentatus* (Provancher, 1886)**

*Microgaster 4-dentatus* Provancher, 1886.

*Microplitisterminatus* Weed, 1888.

**Type information.** Lectotype male, ULQC (examined). Country of type locality: Canada.

**Geographical distribution.**NEA.

**NEA**: Canada (ON), USA (IL, IN, MA, NH, NY, SD).

***Microplitisquintilis* Viereck, 1917**

*Microplitisquintilis* Viereck, 1917.

**Type information.** Holotype male, USNM (not examined but original description checked). Country of type locality: USA.

**Geographical distribution.**NEA.

**NEA**: USA (CT, MO).

***Microplitisratzeburgii* (Ruthe, 1858)**

*Microgasterratzeburgii* Ruthe, 1858.

*Microgasterspinolae* Ratzeburg, 1852 [homonym of *Microgasterspinolae* Nees, 1834].

*Microplitiscerurae* Matsumura, 1921.

**Type information.** Holotype female, NHMUK (examined). Country of type locality: Germany.

**Geographical distribution.**PAL.

**PAL**: Armenia, Bulgaria, Denmark, Finland, France, Germany, Israel, Italy, Japan, Poland, Russia (ZAB, PRI, SAK), Serbia, Ukraine.

**Notes.** Our species concept is based on Shenefelt (1973: 756-757). Nixon (1970) referred to this species as *ratzeburgi* but the original and correct spelling is *ratzeburgii* (Ruthe 1858: 6).

***Microplitisretentus* (Papp, 1984)**

*Microgasterretenta* Papp, 1984.

**Type information.** Holotype female, ZSM (not examined but subsequent treatment of the species checked). Country of type locality: France.

**Geographical distribution.**PAL.

**PAL**: France.

**Notes.** Our species concept is based on Papp (1984c) and Shaw et al. (2009).

***Microplitisrufipes* Dutu-Lacatusu, 1961**

*Microplitisrufipes* Dutu-Lacatusu, 1961.

**Type information.** Holotype female, depository unknown (not examined). Country of type locality: Romania.

**Geographical distribution.**PAL.

**PAL**: Romania.

**Notes.** The information about the holotype is taken from Shenefelt (1973: 757).

***Microplitisrufiventris* Kokujev, 1914**

*Microplitisrufiventris* Kokujev, 1914

**Type information**. Type and depository unknown (not examined but subsequent treatment of the species checked). Country of type locality: Uzbekistan.

**Geographical distribution.**OTL, PAL.

**OTL**: China (HN); **PAL**: Afghanistan, Cyprus, Egypt, Iran, Israel, Jordan, Romania, Russia (NC, S), Turkey, Turkmenistan, Uzbekistan.

**Notes.** Our species concept is based on Papp (1984c) and Tobias (1986).

***Microplitisschmidti* Austin & Dangerfield, 1993**

*Microplitisschmidti* Austin & Dangerfield, 1993.

**Type information.** Holotype female, AEIC (not examined but original description checked). Country of type locality: Australia.

**Geographical distribution.**AUS.

**AUS**: Australia (NT, South WA).

***Microplitisscrophulariae* Szépligeti, 1898**

*Microplitisscrophulariae* Szépligeti, 1898.

**Type information.** Lectotype female, HNHM (not examined but subsequent treatment of the species checked). Country of type locality: Hungary.

**Geographical distribution.**PAL.

**PAL**: Armenia, Azerbaijan, Bulgaria, Croatia, Czech Republic, France, Georgia, Greece, Hungary, Iran, Kazakhstan, Korea, Mongolia, Romania, Russia (ZAB, IRK, KEM, SPE, YAR), Serbia, Slovakia, Sweden, Turkey, United Kingdom.

**Notes.** Our species concept is based on Papp (2004).

***Microplitisscutellatus* Muesebeck, 1922**

*Microplitisscutellatus* Muesebeck, 1922.

**Type information.** Holotype female, USNM (examined). Country of type locality: USA.

**Geographical distribution.**NEA.

**NEA**: Canada (AB, ON), USA (ID, IA, KS, MI, MT, NY, OR, SD, WA).

**Notes.** The female holotype has the scutellar disc with deep impressions close to and around the margins (like *Snellenius* species that have that feature not so pronounced); however, there is no epicnemial carina nor deeply marked notauli, so we retain this species in *Microplitis*.

***Microplitissemicircularis* (Ratzeburg, 1844)**

*Microgastersemicircularis* Ratzeburg, 1844.

**Type information.** Type and depository unknown (not examined but original description checked). Country of type locality: Germany.

**Geographical distribution.**PAL.

**PAL**: Germany, Hungary.

**Notes.** Our species concept is based on the descriptions and comments from Ratzeburg (1844), Fahringer (1937) and Papp (1984c). See comments under the species *Microplitisfordi* Nixon above, and also Broad et al. (2016) for more details on these two species.

***Microplitissimilis* Lyle, 1921**

*Microplitissimilis* Lyle, 1921.

**Type information.** Holotype female, NHMUK (examined). Country of type locality: India.

**Geographical distribution.**OTL.

**OTL**: Bangladesh, India, Indonesia, Vietnam.

***Microplitissofron* Nixon, 1970**

*Microplitissofron* Nixon, 1970.

**Type information.** Holotype female, NHMUK (examined). Country of type locality: Sweden.

**Geographical distribution.**NEA, PAL.

**NEA**: Greenland; **PAL**: Armernia, Azerbaijan, Bulgaria, Denmark, Finland, France, Germany, Greece, Hungary, Iran, Ireland, Italy, Kazakhstan, Netherlands, Norway, Russia (ZAB, SPE), Serbia, Spain, Sweden, Switzerland, Turkey, Turkmenistan, United Kingdom.

**Notes.**Papp (1984c) suggested that this species might be a junior synonym of *Microplitisstigmaticus* (Ratzeburg, 1844), but nevertheless maintained the species as valid (also Broad et al. 2016), a decision we follow here. The species distribution in Armenia and Turkmenistan is based in Belokobylskij et al. (2019).

***Microplitissordipes* (Ziegler, 1834)**

*Microgastersordipes* Ziegler, 1834.

*Microgastertau* Ratzeburg, 1852.

**Type information.** Holotype male, lost (not examined but subsequent treatment of the species checked). Country of type locality: Austria.

**Geographical distribution.**PAL.

**PAL**: Albania, Armenia, Austria, Azerbaijan, Belgium, Czech Republic, Finland, France, Georgia, Germany, Hungary, Italy, Kazakhstan, Lithuania, Moldova, Mongolia, Poland, Romania, Russia (IRK, KL, KIR, KDA, RYA, SPE, VOR, YAR), Slovakia, Sweden, Switzerland, Turkey, Turkmenistan, Ukraine, United Kingdom, Uzbekistan, Yugoslavia.

**Notes.** Our species concept is based on Papp (2016), who also provided details and emendation of the species author name (the species author name was previously considered to be Nees, 1834 by most references, e.g., Yu et al. 2016).

***Microplitisspectabilis* (Haliday, 1834)**

*Microgasterspectabilis* Haliday, 1834.

*Microgasterfossulatus* Bouché, 1834.

*Microgasterparvulus* Ruthe, 1860.

*Microgasterseuratii* Marshall, 1898.

*Dapsilotomatestaceipes* Cameron, 1906.

**Type information.** Lectotype female, NMID (not examined but authoritatively identified specimens examined). Country of type locality: Ireland.

**Geographical distribution.**OTL, PAL.

**OTL**: Pakistan; **PAL**: Armenia, Austria, Azerbaijan, Belgium, Bulgaria, Croatia, Finland, France, Germany, Greece, Hungary, Iran, Ireland, Israel, Italy, Kazakhstan, Latvia, Lithuania, Madeira Islands, Malta, Moldova, Mongolia, Morocco, Poland, Romania, Russia (AD, ZAB, DA, IRK, KIR, KDA, PRI, RYA, SAK, VOR, YAR), Slovakia, Sweden, Switzerland, Tajikistan, Tunisia, Turkey, Turkmenistan, Ukraine, United Kingdom, Uzbekistan.

**Notes.** We examined the type of *Microgasterparvulus* Ruthe. The species distribution in Israel is based in Belokobylskij et al. (2019).

***Microplitisspinolae* (Nees, 1834)**

*Microgasterspinolae* Nees, 1834.

*Microplitissapporoensis* Ashmead, 1906.

*Microplitisradiorimatus* Telenga, 1955.

**Type information.** Neotype female, ZMHB (not examined but subsequent treatment of the species checked). Country of type locality: Germany.

**Geographical distribution.**PAL.

**PAL**: Armenia, Austria, Azerbaijan, Belgium, Bosnia and Herzegovina, Bulgaria, Croatia, Czech Republic, Finland, France, Georgia, Germany, Greece, Hungary, Iran, Ireland, Italy, Japan, Kazakhstan, Korea, Kyrgyzstan, Lithuania, Macedonia, Moldova, Netherlands, Poland, Romania, Russia (KAM, KDA, NGR, PRI, SAK, SPE, VOR, YAR, ZAB), Serbia, Slovakia, Sweden, Switzerland, Tajikistan, Turkey, Ukraine, United Kingdom, Uzbekistan.

**Notes.** Our species concept is based on Nixon (1970), Tobias (1986), Kotenko (2007a) and Shaw (2012b).

***Microplitisspodopterae* Rao & Kurian, 1950**

*Microplitisspodopterae* Rao & Kurian, 1950.

**Type information.** Holotype female, NZSI (not examined but subsequent treatment of the species checked). Country of type locality: India.

**Geographical distribution.**OTL.

**OTL**: India.

**Notes.** Our species concept is based on Gupta (2013a), Gupta & Fernandez-Triana (2014), and Ranjith et al. (2015a).

***Microplitissteinbergi* Tobias, 1964, restored combination**

*Microplitissteinbergi* Tobias, 1964.

**Type information.** Holotype female, ZIN (not examined but original description checked). Country of type locality: Kazakhstan.

**Geographical distribution.**PAL.

**PAL**: Kazakhstan, Russia (S).

**Notes.** Our species concept is based on Tobias (1964, 1986) and Papp (1984c). This species was at times considered to belong to *Microgaster*, e.g., Papp (1984c) and Tobias (1986), as part of the confusion with the application and use of the *Microplitis* and *Microgaster* names, which was only solved after 1988 (see more details and comments under our introduction to the genus *Microgaster* above, p 717). The correct generic placement at present would be in *Microplitis*, which is also corroborated by the description and images in Tobias (1964, 1986). Because some of the more recent references (e.g., Yu et al. 2016) still refer to the species within *Microgaster*, for the sake of clarity we restore its status here.

***Microplitisstigmaticus* (Ratzeburg, 1844)**

*Microgasterstigmaticus* Ratzeburg, 1844.

*Microplitisstigmaticus* Ratzeburg, 1844 [secondary homonym of *Microplitisstigmaticus* Muesebeck, 1922].

*Microplitisstigmativetus* Shenefelt, 1973.

**Type information.** Type and depository unknown (not examined but subsequent treatment of the species checked). Country of type locality: unknown.

**Geographical distribution.**PAL.

**PAL**: Armenia, Azerbaijan, Finland, Germany, Italy, Kazakhstan, Latvia, Poland, Romania, Russia (ALT, KDA, SPE, SAR), Serbia, Turkmenistan, Ukraine, Uzbekistan.

**Notes.** Our species concept is based on Telenga (1955) and Tobias (1986).

***Microplitisstoreyi* Austin & Dangerfield, 1993**

*Microplitisstoreyi* Austin & Dangerfield, 1993.

**Type information.** Holotype female, ANIC (not examined but original description checked). Country of type locality: Australia.

**Geographical distribution.**AUS.

**AUS**: Australia (QLD).

***Microplitisstrenuus* Reinhard, 1880**

*Microplitisstrenuus* Reinhard, 1880.

*Microgastergracilis* Ruthe, 1860 [primary homonym of *Microplitisgracilis* Curtis, 1830].

**Type information.** Holotype female, NHMUK (examined). Country of type locality: Germany.

**Geographical distribution.**PAL.

**PAL**: Afghanistan, Armenia, Azerbaijan, China (GS, SN), Croatia, Czech Republic, Germany, Hungary, Kazakhstan, Moldova, Mongolia, Netherlands, Poland, Russia (ZAB, KDA, PRI, YAR), Serbia, Sweden, Switzerland, Turkey, Ukraine, United Kingdom, Uzbekistan.

***Microplitissuavis* Alexeev, 1971**

*Microplitissuavis* Alexeev, 1971.

**Type information.** Holotype female, ZIN (not examined but original description checked). Country of type locality: Turkmenistan.

**Geographical distribution.**PAL.

**PAL**: Turkmenistan.

***Microplitissubsulcatus* Granger, 1949**

*Microplitissubsulcatus* Granger, 1949.

**Type information.** Holotype female, MNHN (not examined but subsequent treatment of the species checked). Country of type locality: Madagascar.

**Geographical distribution.**AFR.

**AFR**: Madagascar, Réunion.

**Notes.** Our species concept is based on Granger (1949) and Rousse and Gupta (2013).

***Microplitistadzhicus* Telenga, 1949**

*Microplitistadzhicus* Telenga, 1949.

*Microplitismurina* Telenga, 1955.

*Microplitisintermedius* Hedwig, 1961.

**Type information.** Type and depository unknown (not examined but subsequent treatment of the species checked). Country of type locality: Tajikistan.

**Geographical distribution.**OTL, PAL.

**OTL**: China (JS); **PAL**: Afghanistan, Azerbaijan, China (SD), France, Hungary, Kazakhstan, Korea, Russia (UR), Tajikistan, Turkmenistan, Uzbekistan.

**Notes.** Our species concept is based on Telenga (1955), Papp (1984c), Tobias (1986) and Chen and Song (2004).

***Microplitistaptor* (Papp, 1987)**

*Microgastertaptor* Papp, 1987.

**Type information.** Holotype female, HNHM (not examined but subsequent treatment of the species checked). Country of type locality: Korea.

**Geographical distribution.**PAL.

**PAL**: Korea, Russia (PRI).

**Notes.** Our species concept is based on Kotenko (2007a).

***Microplitistasmaniensis* Austin & Dangerfield, 1993**

*Microplitistasmaniensis* Austin & Dangerfield, 1993.

**Type information.** Holotype female, ANIC (not examined but original description checked). Country of type locality: Australia.

**Geographical distribution.**AUS.

**AUS**: Australia (TAS).

***Microplitistaylori* Austin & Dangerfield, 1993**

*Microplitistaylori* Austin & Dangerfield, 1993.

**Type information.** Holotype female, ANIC (not examined but original description checked). Country of type locality: Australia.

**Geographical distribution.**AUS.

**AUS**: Australia (ACT, NSW, QLD, VIC).

***Microplitisteba* (Kotenko, 1994)**

*Microgasterteba* Kotenko, 1994.

**Type information.** Holotype female, SIZK (not examined but subsequent treatment of the species checked). Country of type locality: Russia.

**Geographical distribution.**PAL.

**PAL**: Russia (ZAB).

**Notes.** Our species concept is based on Kotenko (2006, 2007).

***Microplitistestaceicornis* Niezabitowski, 1910**

*Microplitistestaceicornis* Niezabitowski, 1910.

**Type information.** Type and depository unknown (not examined but subsequent treatment of the species checked). Country of type locality: Poland.

**Geographical distribution.**PAL.

**PAL**: Poland.

**Notes.** Our species concept is based on Telenga (1955) and Papp (1984c).

***Microplitistobiasi* Kotenko, 2007**

*Microplitistobiasi* Kotenko, 2007.

**Type information.** Holotype female, SIZK (not examined but original description checked). Country of type locality: Russia.

**Geographical distribution.**PAL.

**PAL**: Russia (PRI).

***Microplitistristis* (Nees, 1834)**

*Microgastertristis* Nees, 1834.

*Microplitisdolens* Marshall, 1885

**Type information.** Type lost (not examined but authoritatively identified specimens examined). Country of type locality: unknown.

**Geographical distribution.**PAL.

**PAL**: Azerbaijan, Belgium, Croatia, France, Germany, Hungary, Kazakhstan, Kyrgyzstan, Lithuania, Moldova, Mongolia, Netherlands, Poland, Romania, Russia (PRI), Slovakia, Switzerland, Turkey, Ukraine, United Kingdom.

**Notes.** We have examined the type of *Microplitisdolens* Marshall, 1885, which is deposited in the NHMUK with code 3c.18. The species distribution in Azerbaijan and Kyrgyzstan is based in Belokobylskij et al. (2019).

***Microplitistuberculatus* (Bouché, 1834)**

*Microgastertuberculatus* Bouché, 1834.

*Microgasterfumipennis* Ratzeburg, 1852.

**Type information.** Holotype female, ZMHB (not examined but subsequent treatment of the species checked). Country of type locality: unknown.

**Geographical distribution.**PAL.

**PAL**: Armenia, Azerbaijan, Finland, Georgia, Germany, Hungary, Ireland, Israel, Italy, Moldova, Mongolia, Poland, Romania, Russia (IN, ZAB, KYA, ROS, RYA, VOR), Slovakia, Sweden, Switzerland, Ukraine, United Kingdom.

**Notes.** Our species concept is based on Papp (1984c), Tobias (1986) and Kotenko (2007a). The species distribution in Israel is based in Belokobylskij et al. (2019).

***Microplitistuberculifer* (Wesmael, 1837)**

*Microgastertuberculifer* Wesmael, 1837.

*Microgastercalcaratus* Thomson, 1895.

*Microgastertrochanteratus* Thomson, 1895.

*Microplitismanevali* Gautier & Bonnamour, 1939.

**Type information.** Lectotype female, RBINS (examined). Country of type locality: Belgium.

**Geographical distribution.**OTL, PAL.

**OTL**: China (FJ, GZ, HB, SN, TW, ZJ), India; **PAL**: Armenia, Austria, Azerbaijan, Belarus, Belgium, Bulgaria, China (BJ, HE, HL, HA, JL, LN, SD, XJ), Croatia, Czech Republic, Estonia, Finland, France, Georgia, Germany, Greece, Hungary, Iran, Ireland, Israel, Italy, Japan, Kazakhstan, Korea, Kyrgyzstan, Latvia, Lithuania, Moldova, Mongolia, Morocco, Netherlands, Poland, Romania, Russia (ARK, ZAB, KAM, KEM, KDA, MOS, NGR, PRI, RYA, SAK, SPE, STA, SA, YAR), Serbia, Slovakia, Spain, Sweden, Switzerland, Turkey, Ukraine, United Kingdom, Uzbekistan, Vietnam.

**Notes.** The species distribution in Iran and Israel is based in Belokobylskij et al. (2019).

***Microplitistunetensis* Marshall, 1901**

*Microplitistunetensis* Marshall, 1901.

**Type information.** Lectotype female, MNHN (not examined but subsequent treatment of the species checked). Country of type locality: Tunisia.

**Geographical distribution.**PAL.

**PAL**: Hungary, Tunisia.

**Notes.** Our species concept is based on Papp (1984c).

***Microplitisvaricolor* Viereck, 1917**

*Microplitisvaricolor* Viereck, 1917.

**Type information.** Holotype male, USNM (examined). Country of type locality: USA.

**Geographical distribution.**NEA.

**NEA**: Canada (MB, NB, ON, QC), USA (AL, CO, CT, DC, FL, IL, LA, MI, MO, NY, OK, PA, SC, TN, TX).

**Notes.**Choi and Kim (2018) reported this species from Korea, and also considered that the species was previously distributed in other countries of the Palearctic region (Bulgaria, China, Finland, Germany, Japan, Norway, and Russia), but without citing any references to support those claims. Because the illustrations of the paper clearly show a male specimen (and not a female, as referred to by the authors), there are no details on the expert identifying the specimens, and the previous distribution of the species in other Palearctic countries has no supporting evidence, we strongly suspect that Choi and Kim (2018) misidentified the species they collected and refute their claims that *varicolor* is a Palearctic species. Those specimens likely belong to *Microplitismediator*, a widespread Palearctic species which seems morphologically and molecularly (DNA barcodes) similar to *M.varicolor* (Fernandez-Triana, unpublished data).

***Microplitisvaripes* (Ruthe, 1860)**

*Microgastervaripes* Ruthe, 1860.

*Microplitisvariipes* Dalla Torre, 1898 [unjustified emendation].

**Type information.** Holotype female, NHMUK (examined). Country of type locality: Germany.

**Geographical distribution.**PAL.

**PAL**: Austria, Azerbaijan, China (QH, XJ), Finland, Georgia, Germany, Hungary, Italy, Kazakhstan, Malta, Moldova, Mongolia, Montenegro, Netherlands, Poland, Russia (ZAB, KDA, RYA, SPE, YAR), Serbia, Slovakia, Switzerland, Turkey, Ukraine.

**Notes.** The species distribution in Georgia is based in Belokobylskij et al. (2019).

***Microplitisviduus* (Ruthe, 1860)**

*Microgasterviduus* Ruthe, 1860.

**Type information.** Holotype female, NHMUK (examined). Country of type locality: Germany.

**Geographical distribution.**PAL.

**PAL**: Armenia, Azerbaijan, Croatia, Cyprus, Czech Republic, Finland, Georgia, Germany, Greece, Hungary, Iran, Israel, Italy, Kazakhstan, Macedonia, Moldova, Mongolia, Netherlands, Poland, Romania, Russia (ZAB, DA, PRI, SAR, YAR), Serbia, Switzerland, Turkey, Ukraine, United Kingdom, Uzbekistan.

**Notes.** The species distribution in Armenia, Georgia, Kyrgyzstan and Turkmenistan is based in Belokobylskij et al. (2019).

***Microplitisvitobiasi* Fernandez-Triana, 2019, new replacement name**

*Microplitisvariicolor* Tobias, 1964 [junior primary homonym of *Microplitisvaricolor* Viereck, 1917].

**Type information.** Holotype female, ZIN (not examined but subsequent treatment of the species checked). Country of type locality: Kazakhstan.

**Geographical distribution.**PAL.

**PAL**: Azerbaijan, Kazakhstan, Mongolia, Russia (S), Ukraine.

**Notes.** Our species concept is based on Papp (1984c) and Kotenko (2006). *Microplitisvariicolor* Tobias, 1964 is a junior primary homonym of *Microplitisvaricolor* Viereck, 1917 under ICZN Article 58.15 (they differ only in the presence or absence of a connecting -i before a suffix). The replacement name is a combination of the initials and last name of V.I. Tobias, the author originally describing the species.

***Microplitisxanthopus* (Ruthe, 1860)**

*Microgasterxanthopus* Ruthe, 1860.

*Microgastertenuipes* Thomson, 1895.

**Type information.** Holotype female, NHMUK (examined). Country of type locality: Germany.

**Geographical distribution.**PAL.

**PAL**: Belarus, Bulgaria, Croatia, Czech Republic, Finland, Georgia, Germany, Hungary, Iran, Ireland, Italy, Kazakhstan, Moldova, Poland, Romania, Russia (IRK, KDA, SAK, SPE, VGG, YAR), Serbia, Sweden, Switzerland, Ukraine, United Kingdom.

**Notes.** The species distribution in Iran is based in Belokobylskij et al. (2019).

***Microplitiszhaoi* Xu & He, 2000**

*Microplitiszhaoi* Xu & He, 2000.

**Type information.** Holotype female, ZJUH (not examined but subsequent treatment of the species checked). Country of type locality: China.

**Geographical distribution.**OTL.

**OTL**: China (FJ, ZJ), India.

**Notes.** Our species concept is based on Ranjith et al. (2015a).

#### Genus Miropotes Nixon, 1965

***Miropotes*** Nixon, 1965: 200. Gender: feminine. Type species: *Miropotescreon* Nixon, 1965, by original designation.

Known from 15 described species from the Oriental, Australasian and Afrotropical regions. We have seen additional species in collections but *Miropotes* does not seem to be very speciose. There are 34 DNA-barcode compliant sequences of this genus in BOLD, representing 12 BINs.

***Miropotesaustini* Fernandez-Triana & Whitfield, 2014**

*Miropotesaustini* Fernandez-Triana & Whitfield, 2014.

**Type information.** Holotype female, CNC (examined). Country of type locality: Australia.

**Geographical distribution.**AUS.

**AUS**: Australia (NSW).

***Miropotesboothis* Austin, 1990**

*Miropotesboothis* Austin, 1990.

**Type information.** Holotype female, ANIC (not examined but subsequent treatment of the species checked). Country of type locality: Australia.

**Geographical distribution.**AUS.

**AUS**: Australia (QLD).

**Notes.** Our species concept is based on Fernandez-Triana et al. (2014d).

***Miropotesburringbaris* Austin, 1990**

*Miropotesburringbaris* Austin, 1990.

**Type information.** Holotype female, ANIC (not examined but subsequent treatment of the species checked). Country of type locality: Australia.

**Geographical distribution.**AUS, OTL.

**AUS**: Australia (ACT, NSW, QLD, TAS, VIC), Papua New Guinea; **OTL**: Indonesia.

**Notes.** Our species concept is based on Fernandez-Triana et al. (2014d).

***Miropotescadgeis* Austin, 1990**

*Miropotescadgeis* Austin, 1990.

**Type information.** Holotype female, ANIC (not examined but subsequent treatment of the species checked). Country of type locality: Australia.

**Geographical distribution.**AUS.

**AUS**: Australia (NSW, QLD).

**Notes.** Our species concept is based on Fernandez-Triana et al. (2014d).

***Miropoteschookolis* Austin, 1990**

*Miropoteschookolis* Austin, 1990.

**Type information.** Holotype female, ANIC (not examined but subsequent treatment of the species checked). Country of type locality: Australia.

**Geographical distribution.**AUS.

**AUS**: Australia (QLD).

**Notes.** Our species concept is based on Fernandez-Triana et al. (2014d).

***Miropotescreon* Nixon, 1965**

*Miropotescreon* Nixon, 1965.

**Type information.** Holotype female, NHMUK (examined). Country of type locality: Australia.

**Geographical distribution.**AUS.

**AUS**: Australia (TAS).

***Miropotesgoobitis* Austin, 1990**

*Miropotesgoobitis* Austin, 1990.

**Type information.** Holotype female, ANIC (not examined but subsequent treatment of the species checked). Country of type locality: Australia.

**Geographical distribution.**AUS.

**AUS**: Australia (NT, QLD, WA).

**Notes.** Our species concept is based on Fernandez-Triana et al. (2014d).

***Miropotesinexpectatus* van Achterberg & Fernandez-Triana, 2017**

*Miropotesinexpectatus* van Achterberg & Fernandez-Triana, 2017.

**Type information.** Holotype female, RMNH (examined). Country of type locality: Yemen.

**Geographical distribution.**AFR.

**AFR**: Yemen.

***Miropoteskatois* Austin, 1990**

*Miropoteskatois* Austin, 1990.

**Type information.** Holotype female, ANIC (not examined but subsequent treatment of the species checked). Country of type locality: Australia.

**Geographical distribution.**AUS.

**AUS**: Australia (SA).

**Notes.** Our species concept is based on Fernandez-Triana et al. (2014d).

***Miropoteskilkulunis* Austin, 1990**

*Miropoteskilkulunis* Austin, 1990.

**Type information.** Holotype female, ANIC (not examined but subsequent treatment of the species checked). Country of type locality: Australia.

**Geographical distribution.**AUS.

**AUS**: Australia (NT).

**Notes.** Our species concept is based on Fernandez-Triana et al. (2014d).

***Miropoteslordhowensis* Fernandez-Triana & Whitfield, 2014**

*Miropoteslordhowensis* Fernandez-Triana & Whitfield, 2014.

**Type information.** Holotype female, CNC (examined). Country of type locality: Australia.

**Geographical distribution.**AUS.

**AUS**: Australia (NSW, TAS).

***Miropotesneglectus* Fernandez-Triana & Whitfield, 2014**

*Miropotesneglectus* Fernandez-Triana & Whitfield, 2014.

**Type information.** Holotype female, CNC (examined). Country of type locality: Papua New Guinea.

**Geographical distribution.**AUS.

**AUS**: Papua New Guinea.

***Miropotesorientalis* Fernandez-Triana & van Achterberg, 2014**

*Miropotesorientalis* Fernandez-Triana & van Achterberg, 2014.

**Type information.** Holotype female, RMNH (examined). Country of type locality: Vietnam.

**Geographical distribution.**OTL.

**OTL**: Thailand, Vietnam.

***Miropotespetiolaris* (Szépligeti, 1905)**

*Microgasterpetiolaris* Szépligeti, 1905.

**Type information.** Lectoype lost (not examined but subsequent treatment of the species checked). Country of type locality: Australia.

**Geographical distribution.**AUS.

**AUS**: Australia (ACT, NSW, NT, QLD, SA, WA).

**Notes.** Our species concept is based on Fernandez-Triana et al. (2014d). The female lectotype is considered to be lost (Austin and Dangerfield 1993: 1156).

***Miropotesthuraris* Austin, 1990**

*Miropotesthuraris* Austin, 1990.

**Type information.** Holotype female, ANIC (not examined but subsequent treatment of the species checked). Country of type locality: Australia.

**Geographical distribution.**AUS.

**AUS**: Australia (NSW, NT, QLD, SA, TAS, VIC, WA), New Caledonia, Papua New Guinea, Vanuatu.

**Notes.** Our species concept is based on Fernandez-Triana et al. (2014d).

#### Genus Napamus Papp, 1993

***Napamus*** Papp, 1993: 168. Gender: masculine. Type species: *Apantelesvipio* Reinhard, 1880, by original designation.

Known from two described species (Papp 1993), but the limits of this genus are not clear at present. One of the species has been reared from Scythrididae and Tineidae. There are no DNA barcode sequences of *Napamus* in BOLD.

***Napamusvipio* (Reinhard, 1880)**

*Apantelesvipio* Reinhard, 1880.

**Type information.** Syntypes female and male, depository unknown (not examined but subsequent treatment of the species checked). Country of type locality: unknown.

**Geographical distribution.**PAL.

**PAL**: Austria, Croatia, France, Germany, Hungary, Israel, Italy, Romania, Russia (C), Spain, Turkey.

**Notes.** Our species concept is based on Papp (1993). The species distribution in Israel is based in Belokobylskij et al. (2019).

***Napamuszomborii* Papp, 1993**

*Napamuszomborii* Papp, 1993.

**Type information.** Holotype female, HNHM (not examined but original description checked). Country of type locality: Armenia.

**Geographical distribution.**PAL.

**PAL**: Armenia.

#### Genus Neoclarkinella Rema & Narendran, 1996

***Neoclarkinella*** Rema & Narendran, 1996: 264. Gender: feminine. Type species: *Apantelesnilamburensis* Sumodan & Narendran, 1990, by original designation.

There are seven described species of *Neoclarkinella*, all from the Oriental region, but the genus has never been revised and we have seen many undescribed species in collections, including species from the Afrotropical, Oriental, and Palearctic regions. No host data are currently available for this genus. There are 130 DNA-barcode compliant sequences of this genus in BOLD, representing 32 BINs.

***Neoclarkinellaariadne* (Nixon, 1965), new combination**

*Apantelesariadne* Nixon, 1965.

**Type information.** Holotype female, NHMUK (examined). Country of type locality: Sri Lanka.

**Geographical distribution.**OTL.

**OTL**: China (GX), India, Sri Lanka.

**Notes.** This species was transferred to *Iconella* by Mason (1981) based on its strong median longitudinal carina on the propodeum. However, the propodeum also has a transverse carina (near the anterior margin), T1 has a wide depression in the anterior half (in addition to a median, longitudinal sulcus throughout the entire tergite), and the veins r and 2RS have the characteristic shape found in *Neoclarkinella* (e.g., Figs 161C, 162C, 163D, 164C, 165C). Based on these characters, we here transfer the species to that genus.

***Neoclarkinellacurvinervus* (Song & Chen, 2014), new combination**

*Choerascurvinervus* Song & Chen, 2014.

**Type information.** Holotype female, ZJUH (not examined but original description checked). Country of type locality: China.

**Geographical distribution.**OTL.

**OTL**: China (FJ, GD, GZ, HI, HN, SN, YN, ZJ).

**Notes.** Transferred to *Neoclarkinella* based on the curved veins r and 2RS in the fore wing, shape of T1, and propodeum carination.

***Neoclarkinellajanakikkadensis* Veena, 2014**

*Neoclarkinellajanakikkadensis* Veena, 2014.

**Type information.** Holotype female, DZUC (not examined but original description checked). Country of type locality: India.

**Geographical distribution.**OTL.

**OTL**: India.

***Neoclarkinellanarendrani* Veena, 2014**

*Neoclarkinellanarendrani* Veena, 2014.

**Type information.** Holotype female, DZUC (not examined but original description checked). Country of type locality: India.

**Geographical distribution.**OTL.

**OTL**: India.

***Neoclarkinellapunctata* Ahmad, Pandey, Haider & Shujauddin, 2005**

*Neoclarkinellapunctata* Ahmad, Pandey, Haider & Shujauddin, 2005.

**Type information.** Holotype female, AMUZ (not examined but original description checked). Country of type locality: India.

**Geographical distribution.**OTL.

**OTL**: India.

***Neoclarkinellasundana* (Wilkinson, 1930), new combination**

*Apantelessundanus* Wilkinson, 1930.

**Type information.** Holotype female, NHMUK (examined). Country of type locality: Indonesia.

**Geographical distribution.**OTL.

**OTL**: Indonesia.

**Notes.** This species was transferred to *Iconella* by Mason (1981), based on the median longitudinal carina on the propodeum. However, we have examined the holotype and there is also an almost complete transverse carina (only interrupted centrally), and the fore wing venation and shape of T1 clearly show this species is better placed in *Neoclarkinella*.

***Neoclarkinellavitellinipes* (You & Zhou, 1990)**

*Apantelesvitellinipes* You & Zhou, 1990.

*Apantelesnilamburensis* Sumodan & Narendran, 1990.

**Type information.** Holotype female, HUNAU (not examined but subsequent treatment of the species checked). Country of type locality: China.

**Geographical distribution.**OTL.

**OTL**: China (FJ, GX, HB, YN), India.

**Notes.** Our species concept is based on van Achterberg and Narendran (1997), Chen and Song (2004), Veena et al. (2014) and Gupta & Fernandez-Triana (2014).

#### Genus Nyereria Mason, 1981

***Nyereria*** Mason, 1981: 108. Gender: feminine. Type species: *Apantelesmlanje* Wilkinson, 1929, by original designation.

There are 29 described species of *Nyereria*, but the genus has never been revised and we have seen many undescribed species in collections, including species from the Afrotropical, Oriental and Palearctic regions. Five families of Lepidoptera have been recorded as hosts of *Nyereria*, but they require further verification. There are 26 DNA-barcode compliant sequences of this genus in BOLD, representing five BINs.

***Nyereriaachaeus* (de Saeger, 1944)**

*Apantelesachaeus* de Saeger, 1944.

**Type information.** Holotype female, RMCA (not examined but original description checked). Country of type locality: Rwanda.

**Geographical distribution.**AFR.

**AFR**: Rwanda.

**Notes.** Because the name is to be considered as a noun under ICZN Article 31.2.1, it must retain its original spelling and remain as *achaeus*.

***Nyereriaalbicentrus* (Long & van Achterberg, 2008)**

*Protapantelesalbicentrus* Long & van Achterberg, 2008.

**Type information.** Holotype female, IEBR (not examined but original description checked). Country of type locality: Vietnam.

**Geographical distribution.**OTL.

**OTL**: Vietnam.

***Nyereriaankaratrensis* (Granger, 1949)**

*Apantelesankaratrensis* Granger, 1949.

**Type information.** Holotype female, MNHN (not examined but original description checked). Country of type locality: Madagascar.

**Geographical distribution.**AFR.

**AFR**: Madagascar.

***Nyereriaareatus* (Granger, 1949)**

*Apantelesareatus* Granger, 1949.

**Type information.** Syntypes female and male, MNHN (not examined but original description checked). Country of type locality: Madagascar.

**Geographical distribution.**AFR.

**AFR**: Madagascar.

**Notes.** Because the name is to be considered as a noun under ICZN Article 31.2.1, it must retain its original spelling and remain as *areatus*.

***Nyereriabicolorata* Long & van Achterberg, 2015**

*Nyereriabicolorata* Long & van Achterberg, 2015.

**Type information.** Holotype female, VNMN (not examined but original description checked). Country of type locality: Vietnam.

**Geographical distribution.**OTL.

**OTL**: Vietnam.

***Nyereriabifissa* (de Saeger, 1944)**

*Apantelesbifissus* de Saeger, 1944.

**Type information.** Holotype female, RMCA (not examined but original description checked). Country of type locality: Democratic Republic of Congo.

**Geographical distribution.**AFR.

**AFR**: Democratic Republic of Congo.

***Nyereriacircinus* (de Saeger, 1944)**

*Apantelescircinus* de Saeger, 1944.

**Type information.** Holotype female, RMCA (not examined but original description checked). Country of type locality: Democratic Republic of Congo.

**Geographical distribution.**AFR.

**AFR**: Democratic Republic of Congo.

**Notes.** Because the name is to be considered as a noun under ICZN Article 31.2.1, it must retain its original spelling and remain as *circinus*.

***Nyereriaepaphus* (de Saeger, 1944)**

*Apantelesepaphus* de Saeger, 1944.

**Type information.** Holotype female, RMCA (not examined but original description checked). Country of type locality: Democratic Republic of Congo.

**Geographical distribution.**AFR.

**AFR**: Democratic Republic of Congo, Rwanda.

**Notes.** Because the name is to be considered as a noun under ICZN Article 31.2.1, it must retain its original spelling and remain as *epaphus*.

***Nyereriaflavotorquata* (Granger, 1949)**

*Apantelesflavotorquatus* Granger, 1949.

**Type information.** Syntypes female, MNHN (not examined but original description checked). Country of type locality: Madagascar.

**Geographical distribution.**AFR.

**AFR**: Madagascar.

***Nyereriaforensis* (Tobias, 1977)**

*Apantelesforensis* Tobias, 1977.

**Type information.** Holotype female, ZIN (not examined but subsequent treatment of the species checked). Country of type locality: Russia.

**Geographical distribution.**PAL.

**PAL**: Korea, Russia (KHA).

**Notes.** Our species concept is based on Kotenko (2007a). The species name was misspelled as *forensic* by Belokobylskij et al. (2019).

***Nyereriaganges* Rousse & Gupta, 2013**

*Nyereriaganges* Rousse & Gupta, 2013.

**Type information.** Holotype female, MNHN (not examined but original description checked). Country of type locality: Réunion.

**Geographical distribution.**AFR.

**AFR**: Réunion.

***Nyereriageometrae* (Granger, 1949)**

*Apantelesgeometrae* Granger, 1949.

**Type information.** Syntypes female and male, MNHN (not examined but original description checked). Country of type locality: Madagascar.

**Geographical distribution.**AFR.

**AFR**: Madagascar.

***Nyereriahiero* (de Saeger, 1944)**

*Apanteleshiero* de Saeger, 1944.

**Type information.** Holotype female, RMCA (not examined but original description checked). Country of type locality: Democratic Republic of Congo.

**Geographical distribution.**AFR.

**AFR**: Democratic Republic of Congo.

***Nyereriaituriensis* (de Saeger, 1941), new combination**

*Apantelesituriensis* de Saeger, 1941.

**Type information.** Holotype female, RMCA (not examined but original description checked). Country of type locality: Democratic Republic of Congo.

**Geographical distribution.**AFR.

**AFR**: Democratic Republic of Congo.

**Notes.** The original description and drawing of T1 and T2 are clear enough to allow us to place the species within the genus *Nyereria*.

***Nyereriamayurus* Rousse & Gupta, 2013**

*Nyereriamayurus* Rousse & Gupta, 2013.

**Type information.** Holotype female, MNHN (not examined but original description checked). Country of type locality: Réunion.

**Geographical distribution.**AFR.

**AFR**: Réunion.

***Nyereriamenuthias* (Wilkinson, 1935)**

*Apantelesmenuthias* Wilkinson, 1935.

**Type information.** Holotype female, MNHN (not examined but original description checked). Country of type locality: Madagascar.

**Geographical distribution.**AFR.

**AFR**: Madagascar.

***Nyereriamlanje* (Wilkinson, 1929)**

*Apantelesmlanje* Wilkinson, 1929.

*Apantelesmlanjeflaviventris* Risbec, 1951.

*Apantelesmlanjepallidus* Risbec, 1951.

**Type information.** Holotype female, NHMUK (examined). Country of type locality: Malawi.

**Geographical distribution.**AFR.

**AFR**: Democratic Republic of Congo, Malawi, Senegal.

**Notes.** This species has long been considered to be very variable. In the original description of *Apantelesmlanjenigricoxis*, Wilkinson (1932a) provided details on some of the differences, mostly in colour, between the new taxon (from Uganda) and the type series of *A.mlanje* (which was also described by Wilkinson in 1929, from Malawi), but for some reason he decided to retain *nigricoxis* as a subspecies of *mlanje*. Other authors working on the African fauna of Microgastrinae also found specimens related to (but morphologically different from) *mlanje*. De Saeger (1944) described three taxa from the Democratic Republic of Congo, which he considered the same as Wilkinson species (*mlanje*) but awarded them infraspecific status as “aberrations”; those three names were mentioned by Shenefelt (1972: 573-574) but treated as excluded names in his Braconidae catalogue. Similarly, Risbec (1951) mentioned at least two “groups” or “forms” from Senegal, which he called *Apantelesmlanjeflaviventris* and *Apantelesmlanjepallidus*; those two names were not referred to by Shenefelt (1972). Both de Saeger and Risbec found considerable variation within *mlanjesensu lato*, and they detailed differences beyond colouration, e.g., sculpture, fore wing venation, shape of T2; Risbec (1951: 431) even acknowledged that the range of variation in the species seemed to be considerably more than in other species of Microgastrinae. Regardless of that, until now these specimens have all been kept as one species. After examining the holotypes of *Apantelesmlanje* Wilkinson, 1929 (from Malawi) and *A.mlanjenigricoxis* Wilkinson, 1932 (from Uganda), both deposited in the NHMUK, we consider them to represent distinct species. The differences in colour are substantial, and the variation in shapes of T1 and T2 (especially the shape of the raised, central area of T2) are also significant. Thus, we elevate *nigricoxis* to species status (see below, under that species, for more details; p 822, 823). As for the other forms or subspecies proposed by de Saeger and Risbec, we suspect some may represent additional species (especially the specimens from Senegal, in Western Africa, which are far from all other specimens in Central Africa and seem to have lighter colouration). However, we cannot make any decisions based only on the original descriptions alone; until we have studied these specimens we prefer to leave them as *Nyereriamlanje*.

***Nyererianeavei* (Wilkinson, 1929)**

*Apantelesneavei* Wilkinson, 1929.

**Type information.** Holotype female, NHMUK (examined). Country of type locality: Malawi.

**Geographical distribution.**AFR, OTL.

**AFR**: Democratic Republic of Congo, Malawi; **OTL**: China (FJ, YN).

***Nyererianeleus* (de Saeger, 1944)**

*Apantelesneleus* de Saeger, 1944.

**Type information.** Holotype female, RMCA (not examined but original description checked). Country of type locality: Democratic Republic of Congo.

**Geographical distribution.**AFR.

**AFR**: Democratic Republic of Congo.

**Notes.** Because the name is to be considered as a noun under ICZN Article 31.2.1, it must retain its original spelling and remain as *neleus*.

***Nyererianigricoxis* (Wilkinson, 1932), status revised**

*Apantelesmlanjenigricoxis* Wilkinson, 1932.

**Type information.** Holotype female, NHMUK (examined). Country of type locality: Uganda.

**Geographical distribution.**AFR.

**AFR**: Uganda.

**Notes.** Until now this was considered a subspecies of *Nyereriamlanje* (Wilkinson, 1929). After comparing the holotypes of both taxa, we consider them to be distinct species (see more comments above under *mlanje*). *Nyererianigricoxis* has darker legs (especially metacoxa and metatibia), T1 narrower at the posterior margin, and T2 with a median raised area much thinner than in *mlanje*. The fore wing venation also differs, specially the proportional lengths of veins r and 2RS.

***Nyererianioro* (Risbec, 1951), new combination**

*Apantelesnioro* Risbec, 1951.

**Type information.** Syntypes female and male, depository unknown (not examined but original description checked). Country of type locality: Senegal.

**Geographical distribution.**AFR.

**AFR**: Senegal.

**Notes.** From the original description and drawings there, it is clear that this species is not an *Apanteles*. The best generic placement at present is in *Nyereria*, based on the shape and sculpture of T2, and also on comments made by Risbec (1951) on its closest relatives (which are also *Nyereria* species).

***Nyereriaosiris* (de Saeger, 1944)**

*Apantelesosiris* de Saeger, 1944.

**Type information.** Holotype female, RMCA (not examined but original description checked). Country of type locality: Democratic Republic of Congo.

**Geographical distribution.**AFR.

**AFR**: Cameroon, Democratic Republic of Congo, Rwanda.

***Nyereriaproagynus* (Hedqvist, 1965), new combination**

*Apantelesproagynus* Hedqvist, 1965.

**Type information.** Holotype male, MZH (examined). Country of type locality: Cape Verde.

**Geographical distribution.**AFR.

**AFR**: Cape Verde.

**Notes.**Forshage et al. (2016) considered the type material to be lost; however, it was found by the senior author of this paper in another section of the MZH collection. We examined the holotype and paratype, both male specimens in relatively good condition. They clearly belong to the genus *Nyereria* based on the carination pattern of propodeum and the median field in T2. Because the name is considered as a noun under ICZN Article 31.2.1, it must retain its original spelling and remain as *proagynus*.

***Nyereriarageshri* Sathe, 1988**

*Nyereriarageshri* Sathe, 1988.

**Type information.** Holotype female, NZSI (not examined). Country of type locality: India.

**Geographical distribution.**OTL.

**OTL**: India.

***Nyereriataoi* (Watanabe, 1935), new combination**

*Apantelestaoi* Watanabe, 1935.

**Type information.** Holotype female, EIHU (not examined but original description checked). Country of type locality: China.

**Geographical distribution.**OTL, PAL.

**OTL**: China (JX, ZJ); **PAL**: China (SD).

**Notes.** Here transferred to *Nyereria* based in the original description mentioning T2 having sulci enclosing a smooth median area, short ovipositor sheaths, acute hypopygium, and the author’s statement that *taoi* closely resembles *Apantelesmlanje* Wilkinson, a species long placed in *Nyereria*.

***Nyereriatereus* (de Saeger, 1944)**

*Apantelestereus* de Saeger, 1944.

**Type information.** Holotype female, RMCA (not examined but original description checked). Country of type locality: Rwanda.

**Geographical distribution.**AFR.

**AFR**: Rwanda.

**Notes.** Because the name is to be considered as a noun under ICZN Article 31.2.1, it must retain its original spelling and remain as *tereus*.

***Nyereriatriptolemus* (de Saeger, 1944)**

*Apantelestriptolemus* de Saeger, 1944.

**Type information.** Holotype female, RMCA (not examined but original description checked). Country of type locality: Democratic Republic of Congo.

**Geographical distribution.**AFR.

**AFR**: Democratic Republic of Congo, Ivory Coast, Rwanda.

**Notes.** Because the name is to be considered as a noun under ICZN Article 31.2.1, it must retain its original spelling and remain as *triptolemus*.

***Nyereriavallatae* (Watanabe, 1934), new combination**

*Apantelesvallatae* Watanabe, 1934.

**Type information.** Syntypes female and male, EIHU (examined). Country of type locality: Japan.

**Geographical distribution.**PAL.

**PAL**: Japan.

**Notes.** The original description (Watanabe 1934: 132–133) was based on five female specimens and did not designate a holotype. We have examined four of those specimens, in the EIHU collection, all with red labels that have the word Type written, and also a second, smaller, white label that reads Cotype. Thus, we consider that they are all syntypes (and that there is no holotype, as stated by other sources, e.g., Shenefelt 1972: 658; Yu et al. 2016). Furthermore, one of the specimens is a male, its relatively small genitalia might have been difficult to see clearly in 1934. There is also a fifth pin with the cocoon mass on a plant twig. One of the syntypes had lost its metasoma, but the other three have their metasomae intact; in two of those cases T2 is relatively narrow and delimited by strong, parallel sulci, clearly similar to other *Nyereria* species. That agrees with Watanabe’s statement, in his original description, that the species belongs to Wilkinson’s *mlanje* subgroup (which is currently considered to belong to the genus *Nyereria*). The third syntype has an intact metasoma has T2 with a slightly different shape (slightly widening towards posterior margin), but overall is very similar to the other two specimens.

***Nyereriayenthuyensis* (Long & van Achterberg, 2008)**

*Protapantelesyenthuyensis* Long & van Achterberg, 2008.

**Type information.** Holotype female, IEBR (not examined but original description checked). Country of type locality: Vietnam.

**Geographical distribution.**OTL.

**OTL**: Vietnam.

#### Genus Ohenri Fernandez-Triana, 2018

***Ohenri*** Fernandez-Triana, 2018: 98. Gender: neuter. Type species: *Ohenrigouletorum* Fernandez-Triana & Boudreault, 2018, by original designation.

Known from a single species from the Afrotropical region, which was recently described (Fernandez-Triana and Boudreault 2018). No host data are currently available for this genus. There are no DNA barcode sequences of *Ohenri* in BOLD.

***Ohenrigouletorum* Fernandez-Triana & Boudreault, 2018**

*Ohenrigouletorum* Fernandez-Triana & Boudreault, 2018.

**Type information.** Holotype female, CNC (examined). Country of type locality: Nigeria.

**Geographical distribution.**AFR.

**AFR**: Nigeria.

#### Genus Papanteles Mason, 1981

***Papanteles*** Mason, 1981: 47. Gender: masculine. Type species: *Papantelespeckorum* Mason, 1981, by original designation.

Known from two described species from the Neotropics; we have seen a few more in collections but the genus does not seem to be species rich. Although no host information has ever been published for *Papanteles*, the ACG caterpillar database records a few species of Crambidae as hosts. There are 56 DNA-barcode compliant sequences of this genus in BOLD, representing three BINs.

***Papantelespeckorum* Mason, 1981**

*Papantelespeckorum* Mason, 1981.

**Type information.** Holotype female, CNC (examined). Country of type locality: Ecuador.

**Geographical distribution.**NEO.

**NEO**: Belize, Brazil (RJ), Ecuador, Mexico, Panama, Trinidad & Tobago.

***Papantelesvirbius* (Nixon, 1965)**

*Hypomicrogastervirbius* Nixon, 1965.

**Type information.** Holotype female, NHMUK (examined). Country of type locality: Brazil.

**Geographical distribution.**NEO.

**NEO**: Brazil (SC).

#### Genus Parapanteles Ashmead, 1900

***Parapanteles*** Ashmead, 1900: 131. Gender: masculine. Type species: *Apantelesaletiae* Riley, 1881, by original designation and monotypy.

A recent revision of the genus (Valerio et al. 2009) is now considered to be outdated, as we recognize 62 described species of *Parapanteles* (including a relatively large number transferred in the present paper). However, the limits of this genus are highly controversial (see discussion above on section Brief diagnosis of all Microgastrinae genera as they are understood in this paper, for more details on p 41), and it is difficult to estimate the potential species richness. Regardless of that, we have seen many undescribed species in collections, from all regions. Approximately a dozen Lepidoptera host families have been recorded in the literature, but many of those records may be wrong. There are almost 1,000 DNA-barcode compliant sequences of this genus in BOLD, representing 97 BINs, but many of those sequences are likely to represent other genera.

***Parapantelesaethiopicus* (Wilkinson, 1931), new combination**

*Dolichogenideaaethiopicus* Wilkinson, 1931.

*Apantelesprocerae* Risbec, 1951.

**Type information.** Holotype female, NHMUK (examined). Country of type locality: Uganda.

**Geographical distribution.**AFR.

**AFR**: Cameroon, Democratic Republic of Congo, Egypt, Ethiopia, Ivory Coast, Kenya, Rwanda, Senegal, Sierra Leone, Somalia, South Africa, Sudan, Tanzania, Uganda.

**Notes.** Based on the relatively short ovipositor sheaths (approximately one third as long as metatibia length), inflexible hypopygium, and fully areolated propodeum, this species is placed in *Parapanteles*.

***Parapantelesaletiae* (Riley, 1881)**

*Apantelesaletiae* Riley, 1881.

**Type information.** Syntypes female, USNM (examined). Country of type locality: USA.

**Geographical distribution.**NEA, NEO.

**NEA**: USA (AL, FL); **NEO**: Cuba, Puerto Rico.

**Notes.**Valerio et al. (2009: 12) mentioned a female holotype and two paratypes of this species in the USNM. We have examined the same material and found that the three specimens (mounted on individual points) are all on the same pin, which also contains a fourth point with the three cocoons. The red label attached to that pin shows that it is USNM Type number 2771, which agrees with both Valerio et al. (2009) and Shenefelt's catalogue (1972). None of the available labels associated with those specimens (nor any other data or published papers that we are aware of) suggest that a lectotype was designated from among the three syntypes, so we consider them all to be syntypes; in any case, it is obvious that there cannot be a holotype for this species. At the time one of us (JFT) examined the syntypes, in October 2017, the first specimen (the top point) was almost entirely missing, with only parts of two legs glued to that point. The other two specimens were both missing the entire metasoma (and one of them was also missing one antenna). That leaves the entire type series as currently having only two syntypes with missing metasomae. Additionally, the drawing of the propodeum from Mason (1981), reproduced by Valerio et al. (2009), does not entirely reflect the two syntypes we examined, which have the areola wider at the posterior end, i.e., the carinae meet the nucha more separated from each other than is depicted by Mason or Valerio.

***Parapantelesalternatus* (Papp, 1973), new combination**

*Apantelesalternatus* Papp, 1973.

**Type information.** Holotype female, HNHM (not examined but original description checked). Country of type locality: India.

**Geographical distribution.**OTL.

**OTL**: India.

**Notes.** The original description and drawings provided there clearly show that this species belongs to *Parapanteles*.

***Parapantelesarka* Gupta, 2014**

*Parapantelesarka* Gupta, 2014.

**Type information.** Holotype female, NBAIR (not examined but original description checked). Country of type locality: India.

**Geographical distribution.**OTL.

**OTL**: India.

***Parapantelesaso* (Nixon, 1967), new combination**

*Apantelesaso* Nixon, 1967.

**Type information.** Holotype female, NHMUK (examined). Country of type locality: India.

**Geographical distribution.**OTL.

**OTL**: China (YN), India.

**Notes.** This species was transferred from *Apanteles* to *Dolichogenidea* by Chen and Song (2004). However, we have examined the holotype, which has an inflexible hypopygium and very short ovipositor sheaths (less than 0.3 × metatibia length). Those characters suggest this species is better placed in *Parapanteles*, as is the case with two related taxa (*Apanteleshyposidrae* Wilkinson, 1928 and *Apantelescleo* Nixon, 1967). These three species were keyed out together in the same section of the key to Indo-Australian species of the *ultor* group by Nixon (1967) and are all transferred to *Parapanteles* in the present paper.

***Parapantelesatellae* (Wilkinson, 1932), new combination**

*Apantelesatellae* Wilkinson, 1932.

**Type information.** Holotype female, NHMUK (examined). Country of type locality: Uganda.

**Geographical distribution.**AFR.

**AFR**: Uganda.

**Notes.** Based on the relatively short ovipositor sheaths (approximately one third as long as metatibia length), inflexible hypopygium and fully areolated propodeum, this species is placed in *Parapanteles*.

***Parapantelesathamasae* Gupta, Khot & Chorge, 2014**

*Parapantelesathamasae* Gupta, Khot & Chorge, 2014.

**Type information.** Holotype female, NBAIR (not examined but original description checked). Country of type locality: India.

**Geographical distribution.**OTL.

**OTL**: India.

***Parapantelesbagicha* (Narayanan & Subba Rao, 1961), new combination**

*Apantelesbagicha* Narayanan & Subba Rao, 1961.

**Type information.** Holotype female, INPC (not examined but original description checked). Country of type locality: India.

**Geographical distribution.**OTL.

**OTL**: India.

**Notes.** Based on the original description and drawings included there, this species is better placed within *Parapanteles*, based on the areolated propodeum but very short ovipositor sheaths.

***Parapantelescleo* (Nixon, 1967), new combination**

*Apantelescleo* Nixon, 1967.

**Type information.** Holotype female, NHMUK (examined). Country of type locality: India.

**Geographical distribution.**OTL.

**OTL**: India, Vietnam.

**Notes.** This species was transferred from *Apanteles* to *Dolichogenidea* by Long and Belokobylskij (1990). However, we have examined the holotype, which has an inflexible hypopygium and very short ovipositor sheaths (less than 0.3 × metatibia length). Those characters suggest this species is better placed in *Parapanteles*, as is the case with two related taxa (*Apanteleshyposidrae* Wilkinson, 1928 and *Apantelesaso* Nixon, 1967). These three species were keyed out together in the same section of the key to Indo-Australian species of the *ultor* group by Nixon (1967) and are all transferred to *Parapanteles* in the present paper.

***Parapantelescomplexus* Valerio & Janzen, 2009**

*Parapantelescomplexus* Valerio & Janzen, 2009.

**Type information.** Holotype male, INBio (not examined but original description checked). Country of type locality: Costa Rica.

**Geographical distribution.**NEO.

**NEO**: Costa Rica.

***Parapantelescontinuus* Valerio & Whitfield, 2009**

*Parapantelescontinua* Valerio & Whitfield, 2009.

**Type information.** Holotype female, INBio (not examined but original description checked). Country of type locality: Costa Rica.

**Geographical distribution.**NEO.

**NEO**: Costa Rica.

**Notes.** The species name must be treated as an adjective and not as a noun (Doug Yanega, pers. comm.) and thus it must match the gender of the genus name.

***Parapantelescovino* Rousse, 2013**

*Parapantelescovino* Rousse, 2013.

**Type information.** Holotype female, MNHN (not examined but original description checked). Country of type locality: Réunion.

**Geographical distribution.**AFR.

**AFR**: Réunion.

***Parapantelescyclorhaphus* (de Saeger, 1944), new combination**

*Apantelescyclorhaphus* de Saeger, 1944.

**Type information.** Syntypes female, RMCA (not examined but original description checked). Country of type locality: Democratic Republic of Congo.

**Geographical distribution.**AFR.

**AFR**: Democratic Republic of Congo.

**Notes.** Based on the original description, the best generic placement is in *Parapanteles*.

***Parapantelesdarignac* Rousse, 2013**

*Parapantelesdarignac* Rousse, 2013.

**Type information.** Holotype female, MNHN (not examined but original description checked). Country of type locality: Réunion.

**Geographical distribution.**AFR.

**AFR**: Réunion.

***Parapantelesdemades* (Nixon, 1965), new combination**

*Apantelesdemades* Nixon, 1965.

**Type information.** Holotype female, NHMUK (examined). Country of type locality: Malaysia.

**Geographical distribution.**OTL.

**OTL**: Malaysia, Vietnam.

**Notes.** Based on the propodeal areola, hypopygium mostly inflexible and unpleated (but with small area postero-ventrally slightly translucent) and short ovipositor sheaths, this species is better placed in the genus *Parapanteles*.

***Parapantelesecheriae* Gupta, Pereira & Churi, 2013**

*Parapantelesecheriae* Gupta, Pereira & Churi, 2013.

**Type information.** Holotype female, NBAIR (not examined but original description checked). Country of type locality: India.

**Geographical distribution.**OTL.

**OTL**: India.

***Parapantelesem* Valerio & Whitfield, 2009**

*Parapantelesem* Valerio & Whitfield, 2009.

**Type information.** Holotype female, INBio (not examined but original description checked). Country of type locality: Costa Rica.

**Geographical distribution.**NEO.

**NEO**: Costa Rica.

***Parapantelesendymion* (Wilkinson, 1932), new combination**

*Apantelesendymion* Wilkinson, 1932.

**Type information.** Holotype female, NHMUK (examined). Country of type locality: Uganda.

**Geographical distribution.**AFR.

**AFR**: Uganda.

**Notes.** Based on the relatively short ovipositor sheaths (approximately one third as long as metatibial lengths), inflexible hypopygium and fully areolated propodeum, this species is placed in *Parapanteles*.

***Parapantelesepiplemicidus* (de Saeger, 1941), new combination**

*Apantelesepiplemicidus* de Saeger, 1941.

**Type information.** Holotype female, RMCA (not examined but original description checked). Country of type locality: Democratic Republic of Congo.

**Geographical distribution.**AFR.

**AFR**: Democratic Republic of Congo.

**Notes.** Transferred to *Parapanteles* based on the propodeum with pentagonal areolet and very short ovipositor sheaths.

***Parapanteleseros* Gupta, 2014**

*Parapanteleseros* Gupta, 2014.

**Type information.** Holotype female, NBAIR (not examined but original description checked). Country of type locality: India.

**Geographical distribution.**OTL.

**OTL**: India.

***Parapantelesesha* Gupta, 2014**

*Parapantelesesha* Gupta, 2014.

**Type information.** Holotype female, NBAIR (not examined but original description checked). Country of type locality: India.

**Geographical distribution.**OTL.

**OTL**: India.

***Parapantelesexpulsus* (Turner, 1919), new combination**

*Apantelesexpulsus* Turner, 1919.

*Apantelesmendanae* Wilkinson, 1928.

**Type information.** Holotype female, NHMUK (examined). Country of type locality: Fiji.

**Geographical distribution.**AUS, OTL.

**AUS**: Fiji, Marquesas Islands, Western Samoa; **OTL**: China (FJ, GD, GX, HI, ZJ), Sri Lanka, Vietnam.

**Notes.** The holotype has an inflexible ovipositor, very short ovipositor sheaths (less than 0.3 x metatibial lengths), and the propodeum has a complete areola defined by strong carinae. All of this suggests this species is better placed in *Parapanteles*. We have also examined the type of *A.mendanae* Wilkinson, in the NHMUK. The species distribution in China is based in Liu et al. (2019).

***Parapantelesfallax* (de Saeger, 1944), new combination**

*Apantelesfallax* de Saeger, 1944.

**Type information.** Holotype female, RMCA (not examined but original description checked). Country of type locality: Democratic Republic of Congo.

**Geographical distribution.**AFR.

**AFR**: Democratic Republic of Congo.

**Notes.** Based on the original description, the best generic placement is in *Parapanteles*.

***Parapantelesfolia* (Nixon, 1965), new combination**

*Apantelesfolia* Nixon, 1965.

**Type information.** Holotype female, NHMUK (examined). Country of type locality: Malaysia.

**Geographical distribution.**AUS, OTL.

**AUS**: Australia (QLD), Papua New Guinea; **OTL**: China (GD, TW), India, Malaysia, Philippines.

**Notes.** The holotype is missing the antennae and the micropin is full of rust, but nevertheless most of the morphological features are visible. Based on the propodeal areola, hypopygium mostly inflexible and unpleated (but with small area postero-ventrally slightly translucent), and short ovipositor sheaths, this species is better placed in the genus *Parapanteles*. This species most likely contains a complex of species, also suggested by Nixon (1965).

***Parapantelesfurax* (de Saeger, 1944), new combination**

*Apantelesfurax* de Saeger, 1944.

**Type information.** Holotype female, RMCA (not examined but original description checked). Country of type locality: Democratic Republic of Congo.

**Geographical distribution.**AFR.

**AFR**: Democratic Republic of Congo, Rwanda.

**Notes.** Based on the original description, the best generic placement would be in *Parapanteles*.

***Parapantelesgerontogeae* Donaldson, 1991**

*Parapantelesgerontogeae* Donaldson, 1991.

**Type information.** Holotype female, TMSA (not examined but original description checked). Country of type locality: South Africa.

**Geographical distribution.**AFR.

**AFR**: South Africa.

***Parapanteleshemitheae* (Wilkinson, 1928), new combination**

*Apanteleshemitheae* Wilkinson, 1928.

**Type information.** Holotype female, NHMUK (examined). Country of type locality: Malaysia.

**Geographical distribution.**OTL, PAL.

**OTL**: China (FJ, GD, GX, TW, ZJ), Malaysia, Vietnam; **PAL**: China (JS).

**Notes.** This species was transferred to *Dolichogenidea* by Long and Belokobylskij (2004), as part of their listing of Braconidae from Vietnam. We have examined the holotype and consider it would be better placed in a different genus. The ovipositor sheaths are very short (shorter than 0.3 x metatibia length), the hypopygium is mostly inflexible (with only a small translucent area near the apex, where no pleat is discernible), and T1, T2 and the anterior half of T3 are strongly sculptured. Those characters are very unusual (if at all present) in *Dolichogenidea*. Although some features would suggest *Pholetesor*, the host caterpillar recorded by Wilkinson (1928b) for the type series is Geometridae, a Lepidoptera family that has never been reported as host for *Pholetesor*. Thus, we believe that the best generic placement at present would be in *Parapanteles*, based on the complete areola on the propodeum, inflexible hypopygium, short ovipositor sheaths and known host. More studies of this and other Oriental species of *Parapanteles* may change that in the future (a similar situation might also apply to the species *Parapantelesexclusus* and *P. hyposidrae*). The species distribution in China is based in Liu et al. (2019).

***Parapanteleshyposidrae* (Wilkinson, 1928), new combination**

*Apanteleshyposidrae* Wilkinson, 1928.

**Type information.** Holotype female, NHMUK (examined). Country of type locality: Indonesia.

**Geographical distribution.**AUS, OTL.

**AUS**: Australia (QLD), Papua New Guinea; **OTL**: China (FJ, GD, GX, HB, HN, TW, YN, ZJ), India, Indonesia, Malaysia, Myanmar, Vietnam.

**Notes.** This species was considered to belong to Dolichogenidea by Yu et al. (2016) and Liu et al. (2019). However, we have examined the holotype and it has an inflexible hypopygium, very short ovipositor sheaths (less than 0.2 x metatibia length), and the propodeum has a complete areola defined by strong carinae; these features suggest this species is better placed in *Parapanteles*.

***Parapantelesindicus* (Bhatnagar, 1950), new combination**

*Apantelesindica* Bhatnagar, 1950.

**Type information.** Holotype female, INPC (not examined but original description checked). Country of type locality: India.

**Geographical distribution.**OTL.

**OTL**: India.

**Notes.** Transferred to *Parapanteles* based on the propodeum with a quadrate areola and ovipositor sheaths very short (Bhatnagar, 1950: 178–179). The year of publication of the Bhatnagar paper was until recently commonly cited as 1948 and/or 1950 (e.g., Chen and Song 2004, Yu et al. 2016), probably following Shenefelt (1972) who referred to this paper as “Bhatnagar (1948) 1950”. While the intended year for Volume X, Parts I & II of the Indian Journal of Entomology was 1948, the actual dates of publication were June 1950 (Part I) and October 1950 (Part II), as clearly shown on the cover page of the Volume, which we have checked. Because the dates of publication are the ones to be considered, and for the sake of clarity, we hereby revise the species year of description to 1950.

***Parapantelesjavensis* (Rohwer, 1919), new combination**

*Apantelesjavensis* Rohwer, 1919.

**Type information.** Holotype female, USNM (examined). Country of type locality: Indonesia.

**Geographical distribution.**OTL, PAL.

**OTL**: China (FJ, GX, HB, SN), India, Indonesia, Sri Lanka, Thailand, Vietnam; **PAL**: Japan.

**Notes.** The holotype is more reddish, when compared to the paratype illustrated in Gupta & Fernandez-Triana (2014), which looks more black. The holotype also has transverse striation on the middle of the hypopygium (very unusual and nothing to do with the hypopygium pleats, as it is actually oriented perpendicular to the hypopygium margin). Based on the inflexible hypopygium lacking pleats, we transfer this species to *Parapanteles*.

***Parapantelesjhaverii* (Bhatnagar, 1950), new combination**

*Apantelesjhaverii* Bhatnagar, 1950.

**Type information.** Holotype female, INPC (not examined but original description checked). Country of type locality: India.

**Geographical distribution.**OTL.

**OTL**: India.

**Notes.** Transferred to *Parapanteles* based on the propodeum with an areola, T1 with longitudinal carina, and very short ovipositor sheaths (Bhatnagar, 1950: 172–174). The year of publication of the Bhatnagar paper was until recently commonly cited as 1948 and/or 1950 (e.g., Chen and Song 2004, Yu et al. 2016), probably following Shenefelt (1972) who referred to this paper as “Bhatnagar (1948) 1950”. While the intended year for Volume X, Parts I & II of the Indian Journal of Entomology was 1948, the actual dates of publication were June 1950 (Part I) and October 1950 (Part II), as clearly shown on the cover page of the Volume, which we have checked. Because the dates of publication are the ones to be considered, and for the sake of clarity, we hereby revise the species year of description to 1950.

***Parapanteleslincolnii* Valerio & Whitfield, 2009**

*Parapanteleslincolnii* Valerio & Whitfield, 2009.

**Type information.** Holotype male, INHS (not examined but original description checked). Country of type locality: USA.

**Geographical distribution.**NEA.

**NEA**: USA (MO).

***Parapantelesmaculipalpis* (de Saeger, 1941), new combination**

*Apantelesmaculipalpis* de Saeger, 1941.

**Type information.** Holotype female, RMCA (not examined but original description checked). Country of type locality: Democratic Republic of Congo.

**Geographical distribution.**AFR.

**AFR**: Democratic Republic of Congo.

**Notes.** Transferred to *Parapanteles* based on the relatively very short ovipositor sheaths, areolated propodeum, and also the comments by de Saeger (1941b: 261) about *maculipalpis* being very close to *Apantelesatellae* Wilkinson, a species that we have also transferred to *Parapanteles* in this paper, after examining its holotype.

***Parapantelesmariae* Valerio & Whitfield, 2009**

*Parapantelesmariae* Valerio & Whitfield, 2009.

**Type information.** Holotype female, INBio (not examined but original description checked). Country of type locality: Costa Rica.

**Geographical distribution.**NEO.

**NEO**: Costa Rica.

***Parapantelesmasoni* Austin & Dangerfield, 1992**

*Parapantelesmasoni* Austin & Dangerfield, 1992.

**Type information.** Holotype female, ANIC (not examined but original description checked). Country of type locality: Australia.

**Geographical distribution.**AUS.

**AUS**: Australia (NT).

***Parapantelesmaynei* (de Saeger, 1941), new combination**

*Apantelesmaynei* de Saeger, 1941.

**Type information.** Holotype male, RMCA (not examined but original description checked). Country of type locality: Democratic Republic of Congo.

**Geographical distribution.**AFR.

**AFR**: Democratic Republic of Congo, Senegal.

**Notes.** Transferred to *Parapanteles* based on the relatively short ovipositor sheaths, areolated propodeum, and also the comments by de Saeger (1941b: 256) about *maynei* being close to *Apantelesaethipicus* Wilkinson and *Apantelesprosper* Wilkinson, two species that we have also transferred to *Parapanteles* in this paper, after examining their holotypes.

***Parapantelesneocajani* (Yousuf & Ray, 2010), new combination**

*Apantelesneocajani* Yousuf & Ray, 2010.

**Type information.** Holotype female, IFRI (not examined but original description checked). Country of type locality: India.

**Geographical distribution.**OTL.

**OTL**: India.

**Notes.** The original description and drawings included there show a hind wing with vannal lobe fully setose, an inflexible hypopygium, and very short ovipositor sheaths (its length equal to the first segment of the metatarsus). Based on those characters, this species is clearly not an *Apanteles* but is better placed in *Parapanteles*.

***Parapantelesneohyblaeae* (Ray & Yousuf, 2009), new combination**

*Apantelesneohyblaeae* Ray & Yousuf, 2009.

**Type information.** Holotype female, IFRI (not examined but original description checked). Country of type locality: India.

**Geographical distribution.**OTL.

**OTL**: India.

**Notes.** The species was described as *Apanteles*, but the very small ovipositor and ovipositor sheaths indicate it does not belong to that genus. The original description does not provide any details about the propodeum, which would have helped considerably to assess the genus to which this species belongs. Without examining the specimens, the best generic placement at present is in *Parapanteles*.

***Parapantelesnephos* Valerio & Whitfield, 2009**

*Parapantelesnephos* Valerio & Whitfield, 2009.

**Type information.** Holotype female, USNM (examined). Country of type locality: Peru.

**Geographical distribution.**NEO.

**NEO**: Peru.

***Parapantelesnoae* Valerio & Whitfield, 2009**

*Parapantelesnoae* Valerio & Whitfield, 2009.

**Type information.** Holotype female, INBio (not examined but original description checked). Country of type locality: Costa Rica.

**Geographical distribution.**NEO.

**NEO**: Costa Rica.

***Parapantelesnydia* (Nixon, 1967), new combination**

*Apantelesnydia* Nixon, 1967.

**Type information.** Holotype female, NHMUK (examined). Country of type locality: India.

**Geographical distribution.**OTL.

**OTL**: India.

**Notes.** This species exemplifies the sometimes-blurred lines separating *Dolichogenidea* from *Parapanteles*. The holotype has the hind wing vannal lobe entirely setose, the anteromesoscutum punctures do not fuse near the scutoscutellar sulcus, the ovipositor sheaths are approximately half the length of the metatibia, and the hypopygium is mostly inflexible (although with a minor fold, seen as a translucent area ventro-posteriorly, but with no pleats marked). With the current understanding of both genera we think at present there is more support for the species to be transferred to *Parapanteles*, a decision we adopt here, but we note that future research on Microgastrinae may change that.

***Parapantelesparadoxus* (Muesebeck, 1958)**

*Apantelesparadoxus* Muesebeck, 1958.

**Type information.** Holotype female, USNM (examined). Country of type locality: Costa Rica.

**Geographical distribution.**NEO.

**NEO**: Costa Rica.

***Parapantelespolus* Valerio & Whitfield, 2009**

*Parapantelespolus* Valerio & Whitfield, 2009.

**Type information.** Holotype female, INBio (not examined but original description checked). Country of type locality: Costa Rica.

**Geographical distribution.**NEO.

**NEO**: Costa Rica.

***Parapantelesprosper* (Wilkinson, 1932), new combination**

*Apantelesprosper* Wilkinson, 1932.

**Type information.** Holotype female, NHMUK (examined). Country of type locality: Uganda.

**Geographical distribution.**AFR.

**AFR**: Uganda.

**Notes.** Based on the relatively very short ovipositor sheaths (less than 0.3 × metatibia length), inflexible hypopygium, and areolated propodeum (although the areola is poorly defined anteriorly), this species is placed in *Parapanteles*.

***Parapantelesprosymna* (Nixon, 1965), new combination**

*Apantelesprosymna* Nixon, 1965.

**Type information.** Holotype female, NHMUK (examined). Country of type locality: Malaysia.

**Geographical distribution.**OTL.

**OTL**: Malaysia.

**Notes.** Based on the propodeal areola, hypopygium mostly inflexible and short ovipositor sheaths, this species is better placed in the genus *Parapanteles*.

***Parapantelespunctatissimus* (Granger, 1949), new combination**

*Apantelespunctatissimus* Granger, 1949.

**Type information.** Syntypes female and male, MNHN (not examined but original description checked). Country of type locality: Madagascar.

**Geographical distribution.**AFR.

**AFR**: Madagascar.

**Notes.** Transferred to *Parapanteles* based on the original description mentioning the propodeum with a complete areola, ovipositor sheaths very short, and T1–T3 shape and sculpture, as illustrated and described in Granger (1949: 269–270, fig. 280).

***Parapantelesrarus* Valerio & Whitfield, 2009**

*Parapantelesrarus* Valerio & Whitfield, 2009.

**Type information.** Holotype female, INHS (not examined but original description checked). Country of type locality: Costa Rica.

**Geographical distribution.**NEO.

**NEO**: Costa Rica.

***Parapantelesregale* Gupta, 2014**

*Parapantelesregale* Gupta, 2014.

**Type information.** Holotype female, NBAIR (not examined but original description checked). Country of type locality: India.

**Geographical distribution.**OTL.

**OTL**: India.

***Parapantelesregalis* (de Saeger, 1941), new combination**

*Apantelesregalis* de Saeger, 1941.

**Type information.** Holotype female, RMCA (not examined but original description checked). Country of type locality: Democratic Republic of Congo.

**Geographical distribution.**AFR.

**AFR**: Democratic Republic of Congo.

**Notes.** Transferred to *Parapanteles* based on the original description mentioning the propodeum with a complete areola (in addition to a partially defined median carina), ovipositor sheaths very short, and T1–T3 shapes and sculptures as illustrated and described in de Saeger (1941: 218–220, fig. 7). The presence of a partial median carina would suggest *Cotesia* as another possible genus; however, the shapes of T1 (anterior 0.4 more or less parallel-sided, posterior 0.6 strongly narrowing towards posterior margin of tergite) and T2 (subtriangular) precludes the species to be considered in that genus, and *Parapanteles* is a much better generic placement. Future study of this species may be needed.

***Parapantelesrooibos* Valerio, Whitfield & Kole, 2005**

*Parapantelesrooibos* Valerio, Whitfield & Kole, 2005.

**Type information.** Holotype female, PPRI (not examined but original description checked). Country of type locality: South Africa.

**Geographical distribution.**AFR.

**AFR**: South Africa.

***Parapantelessarpedon* (de Saeger, 1944), new combination**

*Apantelessarpedon* de Saeger, 1944.

**Type information.** Holotype female, RMCA (not examined but original description checked). Country of type locality: Democratic Republic of Congo.

**Geographical distribution.**AFR.

**AFR**: Democratic Republic of Congo, Rwanda.

**Notes.** Based on the original description, the best generic placement would be in *Parapanteles* because of the inflexible hypopygium, relatively short ovipositor sheaths, and propodeum with areola.

***Parapantelessartamus* (Nixon, 1965), new combination**

*Apantelessartamus* Nixon, 1965.

**Type information.** Holotype female, USNM (examined). Country of type locality: Philippines.

**Geographical distribution.**OTL.

**OTL**: Philippines.

**Notes.** Here transferred to *Parapanteles*, based on the propodeal areola complete and the short ovipositor sheaths (Nixon 1965).

***Parapantelesscultena* (Nixon, 1965), new combination**

*Apantelesscultena* Nixon, 1965.

**Type information.** Holotype female, NHMUK (examined). Country of type locality: Malaysia.

**Geographical distribution.**OTL.

**OTL**: Malaysia.

**Notes.** We place this species in *Parapanteles*, based on the propodeal areola, short ovipositor sheaths and hypopygium inflexible and unfolded.

***Parapantelesshivranginii* Sathe & Ingawale, 1989**

*Parapantelesshivranginii* Sathe & Ingawale, 1989.

**Type information.** Holotype female, NZSI (not examined but original description checked). Country of type locality: India.

**Geographical distribution.**OTL.

**OTL**: India.

***Parapantelessicpolus* Valerio & Whitfield, 2009**

*Parapantelessicpolus* Valerio & Whitfield, 2009.

**Type information.** Holotype female, INBio (not examined but original description checked). Country of type locality: Costa Rica.

**Geographical distribution.**NEO.

**NEO**: Costa Rica.

***Parapantelessireeshaae* Ahmad & Akhtar, 2010**

*Parapantelessireeshaae* Ahmad & Akhtar, 2010.

**Type information.** Holotype female, INPC (not examined but original description checked). Country of type locality: India.

**Geographical distribution.**OTL.

**OTL**: India.

***Parapantelestessares* Valerio & Whitfield, 2009**

*Parapantelestessares* Valerio & Whitfield, 2009.

**Type information.** Holotype female, INBio (not examined but original description checked). Country of type locality: Costa Rica.

**Geographical distribution.**NEO.

**NEO**: Costa Rica.

***Parapantelesthrix* Valerio & Whitfield, 2009**

*Parapantelesthrix* Valerio & Whitfield, 2009.

**Type information.** Holotype female, INHS (not examined but original description checked). Country of type locality: USA.

**Geographical distribution.**NEA.

**NEA**: USA (MO).

***Parapantelestlinea* Valerio & Whitfield, 2009**

*Parapantelestlinea* Valerio & Whitfield, 2009.

**Type information.** Holotype female, INHS (not examined but original description checked). Country of type locality: Costa Rica.

**Geographical distribution.**NEO.

**NEO**: Costa Rica.

***Parapantelestransvaalensis* (Cameron, 1911), new combination**

*Apantelestransvaalensis* Cameron, 1911.

**Type information.** Holotype female, TMSA (not examined but subsequent treatment of the species checked). Country of type locality: South Africa.

**Geographical distribution.**AFR.

**AFR**: Malawi, South Africa.

**Notes.** Our species concept is based on Wilkinson (1932a: 320–321), who redescribed the species after examining the female holotype (the only specimen known). Transferred to *Parapanteles* based on the relatively very short ovipositor (shorter than the first segment of the metatarsus), truncate hypopygium, and fully areolated propodeum.

***Parapantelesturri* (Rao & Chalikwar, 1976), new combination**

*Apantelesturri* Rao & Chalikwar, 1976.

**Type information.** Holotype female, BAMU (not examined but original description checked). Country of type locality: India.

**Geographical distribution.**OTL.

**OTL**: India.

**Notes.** The drawings in the original description suggest that this species is better placed in *Parapanteles*, based on its short ovipositor sheaths and unpleated hypopygium. The authors even considered the species to have a “superficial resemblance with *Apantelesfolia* (Nixon, 1965)” (Rao and Chalikwar 1976a: 185), which is an indirect confirmation of the generic placement, since *Apantelesfolia* is also transferred to *Parapanteles* in the present paper.

***Parapantelesxanthopholis* (de Saeger, 1944), new combination**

*Apantelesxanthopholis* de Saeger, 1944.

**Type information.** Holotype female, RMCA (not examined but original description checked). Country of type locality: Democratic Republic of Congo.

**Geographical distribution.**AFR.

**AFR**: Democratic Republic of Congo, Rwanda.

**Notes.** Based on the original description (de Saeger 1944), the best generic placement would be in *Parapanteles*.

#### Genus Parenion Nixon, 1965

***Parenion*** Nixon, 1965: 208. Gender: feminine. Type species: *Microgasterkokodana* Wilkinson, 1936, by original designation.

Three described species are known from Australasia, but we have seen a few more in collections. No host data are currently available for this genus. There are four DNA-barcode compliant sequences of this genus in BOLD, representing one BIN. The gender of *Parenion* is not stated in the original description, but it is here assumed to be feminine based on the way Nixon (1965) treated the name of the only species known (at the time the genus was described).

***Parenionbeelaronga* Austin & Dangerfield, 1992**

*Parenionbeelaronga* Austin & Dangerfield, 1992.

**Type information.** Holotype female, ANIC (not examined but original description checked). Country of type locality: Australia.

**Geographical distribution.**AUS.

**AUS**: Australia (QLD).

***Parenionbootha* Austin & Dangerfield, 1992**

*Parenionbootha* Austin & Dangerfield, 1992.

**Type information.** Holotype female, ANIC (not examined but original description checked). Country of type locality: Australia.

**Geographical distribution.**AUS.

**AUS**: Australia (QLD).

***Parenionkokodana* (Wilkinson, 1936)**

*Microgasterkokodana* Wilkinson, 1936.

**Type information.** Holotype female, NHMUK (examined). Country of type locality: Papua New Guinea.

**Geographical distribution.**AUS.

**AUS**: Australia (QLD), Papua New Guinea.

#### Genus Paroplitis Mason, 1981

***Paroplitis*** Mason, 1981: 68. Gender: masculine. Type species: *Paroplitisberingianus* Mason, 1981, by original designation.

Five described species were recently revised (Fernandez-Triana et al. 2013b) but we have seen more species in collections. The genus is essentially Holarctic, but occasionally reaching the northern limits of the Oriental region. Host records representing four Lepidoptera families have been reported for one species of *Paroplitis*, but only Crambidae (Scopariinae) has been confirmed (Shaw 2012b). There are 32 DNA-barcode compliant sequences of this genus in BOLD, representing one BIN.

***Paroplitisberingianus* Mason, 1981**

*Paroplitisberingianus* Mason, 1981.

**Type information.** Holotype female, CNC (examined). Country of type locality: USA.

**Geographical distribution.**NEA.

**NEA**: Canada (BC), USA (AK).

***Paroplitisluzonicus* Mason, 1981**

*Paroplitisluzonicus* Mason, 1981.

**Type information.** Holotype female, AEIC (not examined but subsequent treatment of the species checked). Country of type locality: Philippines.

**Geographical distribution.**OTL.

**OTL**: Philippines.

**Notes.** Our species concept is based on Fernandez-Triana et al. (2013b).

***Paroplitisrugosus* Papp, 1991**

*Paroplitisrugosus* Papp, 1991.

**Type information.** Holotype female, HNHM (not examined but subsequent treatment of the species checked). Country of type locality: Austria.

**Geographical distribution.**PAL.

**PAL**: Austria.

**Notes.** Our species concept is based on Fernandez-Triana et al. (2013b).

***Paroplitisvietnamensis* van Achterberg & Fernandez-Triana, 2013**

*Paroplitisvietnamensis* van Achterberg & Fernandez-Triana, 2013.

**Type information.** Holotype female, RMNH (examined). Country of type locality: Vietnam.

**Geographical distribution.**OTL.

**OTL**: Vietnam.

**Notes.** A recent PhD thesis (Ahmed 2017) recorded this species from India (from two different districts in the state of Jammu and Kashmir). The accompanying images in that paper strongly indicate that this is a different, undescribed species, based on different sculpture of T1 and T2, and also the fact that the Indian localities are more than 3,500 km from the type locality in Vietnam. Thus, we here consider *P. vietnamensis* not to be present in India.

***Paroplitiswesmaeli* (Ruthe, 1860)**

*Microgasterwesmaeli* Ruthe, 1860.

*Microgasterpicipes* Wesmael, 1837 [primary homonym of *Microgasterpicipes* Bouché, 1834].

**Type information.** Holotype female, RBINS (not examined but subsequent treatment of the species checked). Country of type locality: Belgium.

**Geographical distribution.**PAL.

**PAL**: Azerbaijan, Belgium, Finland, France, Germany, Hungary, Poland, Romania, Russia (KDA), Sweden, Switzerland, Ukraine, United Kingdom.

**Notes.** Our species concept is based on Shaw (2012b) and Fernandez-Triana et al. (2013b).

#### Genus Pelicope Mason, 1981

***Pelicope*** Mason, 1981: 57. Gender: feminine. Type species: *Pelicopeyuccamica* Mason, 1981, by original designation.

Only known from one species in the Nearctic region. The parasitoid has been reared from Prodoxidae. There is one DNA-barcode compliant sequence of *Pelicope* in BOLD, representing one BIN.

***Pelicopeyuccamica* Mason, 1981**

*Pelicopeyuccamica* Mason, 1981.

**Type information.** Holotype female, USNM (not examined but original description checked). Country of type locality: USA.

**Geographical distribution.**NEA.

**NEA**: USA (CA).

#### Genus Philoplitis Nixon, 1965

***Philoplitis*** Nixon, 1965: 267. Gender: masculine. Type species: *Philoplitisconiferens* Nixon, 1965 by original designation and monotypy.

*Philoplitis* has been revised twice in the past ten years (Fernandez-Triana and Goulet 2009, Ranjith et al. 2019), with the latest paper recording nine species. We suspect a few more will be found when more collections are studied, but the genus does not seem to be species rich. *Philoplitis* species are mainly found in the Oriental region, but it also reaches the Afrotropics and one species marginally reaches the southernmost limits of the Palearctic region (Ranjith et al. 2019). No host data are currently available for this genus. There are seven DNA-barcode compliant sequences of this genus in BOLD, representing four BINs.

***Philoplitisadustipalpus* Ahmad, 2005**

*Philoplitisadustipalpus* Ahmad, 2005.

**Type information.** Holotype female, AMUZ (not examined but subsequent treatment of the species checked). Country of type locality: India.

**Geographical distribution.**OTL.

**OTL**: India.

**Notes.** Our species concept is based on Fernandez-Triana and Goulet (2009) and Ranjith et al. (2019).

***Philoplitisconiferens* Nixon, 1965**

*Philoplitisconiferens* Nixon, 1965.

**Type information.** Holotype female, USNM (not examined but subsequent treatment of the species checked). Country of type locality: Philippines.

**Geographical distribution.**OTL.

**OTL**: China (GD, GX), Philippines.

**Notes.** Our species concept is based on Fernandez-Triana and Goulet (2009) and Ranjith et al. (2019).

***Philoplitisdzangasangha* Fernandez-Triana & Ranjith, 2019**

*Philoplitisdzangasangha* Fernandez-Triana & Ranjith, 2019.

**Type information.** Holotype male, CNC (examined). Country of type locality: Central African Republic.

**Geographical distribution.**AFR.

**AFR**: Central African Republic.

***Philoplitiskeralensis* Ranjith & Fernandez-Triana, 2019**

*Philoplitiskeralensis* Ranjith & Fernandez-Triana, 2019.

**Type information.** Holotype female, DZUC (examined). Country of type locality: India.

**Geographical distribution.**OTL.

**OTL**: India.

***Philoplitismargalla* Fernandez-Triana & Ranjith, 2019**

*Philoplitismargalla* Fernandez-Triana & Ranjith, 2019.

**Type information.** Holotype female, CNC (examined). Country of type locality: Pakistan.

**Geographical distribution.**PAL.

**PAL**: Pakistan.

***Philoplitismasneri* Fernandez-Triana & Goulet, 2009**

*Philoplitismasneri* Fernandez-Triana & Goulet, 2009.

**Type information.** Holotype male, CNC (examined). Country of type locality: Kenya.

**Geographical distribution.**AFR.

**AFR**: Kenya.

***Philoplitispunctatus* Fernandez-Triana & Goulet, 2009**

*Philoplitispunctatus* Fernandez-Triana & Goulet, 2009.

**Type information.** Holotype male, CNC (examined). Country of type locality: Thailand.

**Geographical distribution.**OTL.

**OTL**: Thailand.

***Philoplitisstriatus* Fernandez-Triana & Goulet, 2009**

*Philoplitisstriatus* Fernandez-Triana & Goulet, 2009.

**Type information.** Holotype male, CNC (examined). Country of type locality: India.

**Geographical distribution.**OTL.

**OTL**: India, Sri Lanka.

***Philoplitistrifoveatus* Ranjith & Fernandez-Triana, 2019**

*Philoplitistrifoveatus* Ranjith & Fernandez-Triana, 2019.

**Type information.** Holotype female, DZUC (examined). Country of type locality: India.

**Geographical distribution.**OTL.

**OTL**: India.

#### Genus Pholetesor Mason, 1981

***Pholetesor*** Mason, 1981: 37. Gender: masculine. Type species: *Apantelesornigis* Weed, 1887, by original designation.

This is a cosmopolitan genus, with 57 described species, but we have seen many additional species in collections, mostly from temperate areas. There are some revisions available for the Nearctic (Whitfield 2006) and Palearctic regions (see works of Nixon and Papp in the References section below), but most can be considered as outdated because none of them take into account the hidden diversity that is revealed by DNA barcoding and biological data. Around two dozen families of Lepidoptera have been recorded as hosts for *Pholetesor*, but many records are likely to be incorrect and/or need further verification. There are 1,000+ DNA-barcode compliant sequences of this genus in BOLD, representing 50 BINs.

***Pholetesoracricauda* Liu & Chen, 2016**

*Pholetesoracricauda* Liu & Chen, 2016.

**Type information.** Holotype female, ZJUH (not examined but original description checked). Country of type locality: China.

**Geographical distribution.**OTL, PAL.

**OTL**: China (SN); **PAL**: China (HA).

***Pholetesoracutus* (Papp, 1971), new combination**

*Apantelesacutus* Papp, 1971.

**Type information.** Holotype female, HNHM (not examined but original description checked). Country of type locality: Mongolia.

**Geographical distribution.**PAL.

**PAL**: Mongolia.

**Notes.** From the original description it is evident that this species is not *Apanteles*. Here we transfer *acutus* to *Pholetesor* based on the short length of the ovipositor sheaths, the inflexible and unpleated hypopygium, the shapes of T1 and T2, the propodeum sculpture, and the fact that Papp (1971: 311) considered the species to be closely related to *Apantelesingenuus* (which is currently placed within *Pholetesor*). In a subsequent paper illustrating *Apantelesacutus* (Papp 1984a: 290, figure 21), the drawing seems to show much longer ovipositor sheaths, although that may be a mistake, as the key to species in that same paper places *acutus* with other species which have “Ovipositor sheath short, in lateral view at most as long as first joint of hind tarsus” (quoted from couplet 17 in Papp 1984a: 267). We suspect that many of the species placed within the *metacarpalis* group by Papp (1984a), which includes species mostly described by Papp and Tobias, belong to *Pholetesor*.

***Pholetesorambiguus* (Papp, 1977)**

*Apantelesambiguus* Papp, 1977.

**Type information.** Holotype female, HNHM (not examined but original description checked). Country of type locality: Mongolia.

**Geographical distribution.**PAL.

**PAL**: Mongolia.

***Pholetesorargyresthiae* Liu & Chen, 2016**

*Pholetesorargyresthiae* Liu & Chen, 2016.

**Type information.** Holotype female, ZJUH (not examined but original description checked). Country of type locality: China.

**Geographical distribution.**PAL.

**PAL**: China (GS).

***Pholetesorarisba* (Nixon, 1973)**

*Apantelesarisba* Nixon, 1973.

**Type information.** Holotype female, NHMUK (examined). Country of type locality: United Kingdom.

**Geographical distribution.**AUS, OTL, PAL.

**AUS**: New Zealand; **OTL**: China (FJ, GZ, ZJ); **PAL**: Austria, Bulgaria, China (NX), Czech Republic, Denmark, Egypt, Germany, Greece, Hungary, Israel, Italy, Netherlands, Norway, Russia (NC), Serbia, Spain, Ukraine, United Kingdom.

**Notes.** The species distribution in Israel is based in Belokobylskij et al. (2019).

***Pholetesorartusisulcus* Liu & Chen, 2016**

*Pholetesorartusisulcus* Liu & Chen, 2016.

**Type information.** Holotype female, ZJUH (not examined but original description checked). Country of type locality: China.

**Geographical distribution.**OTL, PAL.

**OTL**: China (ZJ); **PAL**: China (NX).

***Pholetesorbedelliae* (Viereck, 1911)**

*Apantelesbedelliae* Viereck, 1911.

**Type information.** Holotype female, USNM (examined). Country of type locality: USA.

**Geographical distribution.**AUS, NEA, NEO, PAL.

**AUS**: Hawaiian Islands; **NEA**: Canada (AB, BC, MB, NS, ON, QC, SK), USA (AK, AZ, AR, CA, CT, DC, FL, IL, IA, KA, LA, MO, NJ, NY, OR, VA); **NEO**: Bermuda, Peru; **PAL**: Finland.

**Notes.** This species was introduced to the Hawaiian Islands (Fullaway 1950). There is also a record from Peru (de Huiza 1995) which should be considered as suspicious, but we retain it here as we could not examine it in more detail. The species is probably Holarctic in distribution.

***Pholetesorbicolor* (Nees, 1834)**

*Microgasterbicolor* Nees, 1834.

*Microgasterardeaepenellae* Bouché, 1834.

*Apantelesschillei* Niezabitowski, 1910.

*Apanteleslongicauda* Fahringer, 1938.

*Apantelespedias* Nixon, 1973.

**Type information.** Neotype female, ZMHB (not examined but subsequent treatment of the species checked). Country of type locality: Germany.

**Geographical distribution.**AUS, NEA, OTL, PAL.

**AUS**: New Zealand; **NEA**: Canada (ON); **OTL**: China (JS); **PAL**: Belgium, Bulgaria, Canary Islands, China (NX), Croatia, Finland, France, Georgia, Germany, Greece, Hungary, Iran, Ireland, Israel, Italy, Japan, Kyrgyzstan, Lithuania, Moldova, Mongolia, Poland, Romania, Russia (KAM, MOS, PRI, SPE, YAR), Serbia, Slovakia, Spain, Switzerland, Tunisia, Turkmenistan, Ukraine, United Kingdom.

**Notes.** According to Papp (1983a: remark on pages 253–254, not page 267 as referenced in footnote on page 251), *Microgasterbicolor* Curtis, 1830 is a *nomen nudum*, so *Microgasterbicolor* Nees, 1834 is valid. That statement was accepted by Shaw (2012b) and Broad et al. (2016) and it is also followed here, where we consider *Pholetesorbicolor* (Nees, 1834) as a valid name and a valid species. However, Wilkinson (1938d) listed *bicolor* (= *pediassensu* Nixon) as a synonym of *circumscriptus*, a species he interpreted widely and considered very variable in some characters. Shaw (2012b) has added strong evidence (biological and morphological data) that support *bicolor* and *circumscriptus* being considered as different species. Thus, all the synonyms listed by Wilkinson (1938d) need to be re-apportioned between *bicolor* and *circumscriptus*. Here we consider as synonyms of *bicolor*: a) *Microgasterardeaepenellae* (Bouché, 1834), following Papp (1983a, 1988), who saw the type; b) *Apantelesschillei* (Niezabitowski, 1910) is tentatively placed (with a question mark) under *bicolor*, following Papp (1988: 148), who did not see the type of that species; c) *Apanteleslongicauda* (Fahringer, 1938), following the original description (Fahringer 1938: 10); and d) *Apantelespedias* Nixon, 1973, based on our study of the type. We consider synonyms of *circumscriptus*: e) *Microgasterexiguus* (Haliday, 1834), based on our study of the lectotype and also van Achterberg (1997); f) *Microgasterumbellatarum* (Haliday, 1834), following Papp (1988), who did not see the type of that species [but also note that van Achterberg (1997), who did not see the type either, placed *umbellatarum* as a synonym of *bicolor*]; g) *Microgasterblancardellae* (Bouché, 1834), following Papp (1983a, 1988) who saw the type; h) *Microgasterlividipes* (Wesmael, 1837), following Papp (1988), although it is not clear to us if he saw that type; i) *Microgasterflavolimbatus* (Ratzeburg, 1848), following Papp (1983a, 1988), it is not clear to us if he saw that type; j) *Apanteleslautellus* (Marshall, 1885), based on our study of the type. In addition to the above, material determined by Nixon as *exiguus* Haliday (see Nixon 1973) is a different species, the status of which is still unresolved; Shaw (2012b) thought that species (*exiguussensu* Nixon *nec* Haliday) was probably a northern form of *laetus* Marshall, partly on the basis of rearing experiments, but ongoing research involving DNA barcoding will be needed before a conclusion can be reached. Additionally, Shaw (2012b) rejected the statement by van Achterberg (1997) that *exiguussensu* Nixon is the same as *salalicus* Mason, a position we follow here. Because of the convoluted story of the use and application of the names *bicolor* and *circumscriptus* (and corresponding synonyms), it is very difficult to determine with certainty the actual distribution of the two species which, based on current data, seem to overlap for the most part (e.g., see Yu et al. 2016). Both species seem to be rather broadly distributed in the Palearctic region, also reaching into the northern part of the Oriental region (China); however, until comprehensive studies of the specimens mentioned in the historical literature are done it will not be possible to untangle the distributional information. Similarly, there are a few references to these two species in New Zealand and North America (e.g., Bartlett et al. 1978, Valentine and Walker 1991, Fernandez-Triana 2010), mostly as introductions for biological control. DNA barcodes are equally confusing at present, as among dozens of specimens in BOLD which are labelled as either *Pholetesorcircumscriptus*, *Pholetesorexiguuus*, or *Pholetesor* (with some interim names), there seems to be a complex of molecularly (DNA barcodes) related species. Solving these problems is beyond the scope of the present paper, and thus we are limited here to pointing out the difficulties and unknowns related to these species. The species distribution in Israel and Kyrgyzstan are based in Belokobylskij et al. (2019).

***Pholetesorbrevivalvatus* (Balevski & Tobias, 1980), new combination**

*Apantelesbrevivalvatus* Balevski & Tobias, 1980.

**Type information.** Holotype female, ZIN (not examined but subsequent treatment of the species checked). Country of type locality: Bulgaria.

**Geographical distribution.**PAL.

**PAL**: Bulgaria.

**Notes.** This species is clearly not an *Apanteles*, based on the short ovipositor sheaths (and, to a lesser extent, also based on the shapes of T1 and T2, which is not commonly found in *Apanteles*). Papp (1984a) considered this species to be related to *Pholetesoringenuus* (Tobias, 1964), based on a number of features; the available drawings for both species indeed look similar. Examination of the type specimen will be needed to conclude, but for the time being we follow Papp's suggestion and transfer the species from *Apanteles* to *Pholetesor*.

***Pholetesorbucculatricis* (Muesebeck, 1921)**

*Apantelesbucculatricis* Muesebeck, 1921.

**Type information.** Holotype female, USNM (not examined but original description checked). Country of type locality: USA.

**Geographical distribution.**NEA.

**NEA**: USA (CA).

***Pholetesorcaloptiliae* Whitfield, 2006**

*Pholetesorcaloptiliae* Whitfield, 2006.

**Type information.** Holotype female, CNC (examined). Country of type locality: Canada.

**Geographical distribution.**NEA.

**NEA**: Canada (ON), USA (CT, IN, NY, OH).

***Pholetesorchiricahuensis* Whitfield, 2006**

*Pholetesorchiricahuensis* Whitfield, 2006.

**Type information.** Holotype female, USNM (examined). Country of type locality: USA.

**Geographical distribution.**NEA.

**NEA**: USA (AZ, CA, CO, FL, NM).

***Pholetesorcircumlatus* Kotenko, 2007**

*Pholetesorcircumlatus* Kotenko, 2007.

**Type information.** Holotype female, SIZK (not examined but original description checked). Country of type locality: Russia.

**Geographical distribution.**PAL.

**PAL**: Russia (SAK).

***Pholetesorcircumscriptus* (Nees, 1834)**

*Microgastercircumscriptus* Nees, 1834.

*Microgasterexiguus* Haliday, 1834.

*Microgasterumbellatarum* Haliday, 1834.

*Microgasterblancardellae* Bouché, 1834.

*Microgasterlividipes* Wesmael, 1837.

*Microgasterflavolimbatus* Ratzeburg, 1848.

*Apanteleslautellus* Marshall, 1885.

**Type information.** Neotype female, ZMHB (not examined but subsequent treatment of the species checked). Country of type locality: Germany.

**Geographical distribution.**AUS, NEA, OTL, PAL.

**AUS**: New Zealand; **NEA**: USA (AK); **OTL**: China (SN, YN, ZJ); **PAL**: Armenia, Austria, Azerbaijan, Belgium, Bulgaria, China (SD), Croatia, Czech Republic, Finland, Georgia, Germany, Greece, Hungary, Iran, Ireland, Israel, Italy, Japan, Kazakhstan, Korea, Latvia, Lithuania, Madeira Islands, Malta, Moldova, Netherlands, Poland, Romania, Russia (IRK, KEM, KHA, KDA, MOS, PRI, ROS, SAK, SPE, VLA, VOR), Slovakia, Spain, Switzerland, Ukraine, United Kingdom, Yugoslavia.

**Notes.** See notes under *Pholetesorbicolor* above for detailed explanations on the history of names used for these two species, their synonyms, distribution and molecular data. The species distribution in Japan and Kazakhstan are based in Belokobylskij et al. (2019).

***Pholetesorconfusus* Liu & Chen, 2016**

*Pholetesorconfusus* Liu & Chen, 2016.

**Type information.** Holotype female, ZJUH (not examined but original description checked). Country of type locality: China.

**Geographical distribution.**PAL.

**PAL**: China (LN).

***Pholetesordixianus* Whitfield, 2006**

*Pholetesordixianus* Whitfield, 2006.

**Type information.** Holotype female, SEMC (not examined but original description checked). Country of type locality: USA.

**Geographical distribution.**NEA.

**NEA**: USA (NC, TX).

***Pholetesordmitriyi* Kotenko, 2007**

*Pholetesordmitriyi* Kotenko, 2007.

**Type information.** Holotype female, SIZK (not examined but original description checked). Country of type locality: Russia.

**Geographical distribution.**PAL.

**PAL**: Russia (PRI).

***Pholetesorelpis* (Nixon, 1973)**

*Apanteleselpis* Nixon, 1973.

*Apantelesgirkanus* Tobias, 1976.

**Type information.** Holotype female, NHMUK (examined). Country of type locality: United Kingdom.

**Geographical distribution.**PAL.

**PAL**: Austria, Azerbaijan, Bulgaria, Croatia, Finland, Germany, Greece, Hungary, Iran, Korea, Mongolia, Netherlands, Poland, Russia (MAG, PRI, SAK), Serbia, Slovakia, Ukraine, United Kingdom.

***Pholetesorerrans* (Nixon, 1973)**

*Pholetesorerrans* Nixon, 1973.

*Apantelesarenicola* Papp, 1973.

**Type information.** Holotype female, NHMUK (examined). Country of type locality: United Kingdom.

**Geographical distribution.**PAL.

**PAL**: Hungary, United Kingdom.

***Pholetesorextentus* (Papp, 1977), new combination**

*Apantelesextentus* Papp, 1977.

**Type information.** Holotype female, HNHM (not examined but original description checked). Country of type locality: Mongolia.

**Geographical distribution.**PAL.

**PAL**: Mongolia.

**Notes.** Based on the original description and illustrations provided there, this species is clearly not an *Apanteles*. The relatively short ovipositor sheaths and shapes of T1 and T2 strongly suggest the best generic placement would be *Pholetesor* (although future examination of the specimens in the HNHM may show *Dolichogenidea* as a possible alternative).

***Pholetesorflavigleba* Liu & Chen, 2016**

*Pholetesorflavigleba* Liu & Chen, 2016.

**Type information.** Holotype female, ZJUH (not examined but original description checked). Country of type locality: China.

**Geographical distribution.**OTL, PAL.

**OTL**: China (ZJ); **PAL**: China (HE, LN, SN).

***Pholetesorflaviparvus* Liu & Chen, 2016**

*Pholetesorflaviparvus* Liu & Chen, 2016.

**Type information.** Holotype female, ZJUH (not examined but original description checked). Country of type locality: China.

**Geographical distribution.**OTL.

**OTL**: China (JS).

***Pholetesorglacialis* (Ashmead, 1902)**

*Protapantelesglacialis* Ashmead, 1902.

**Type information.** Holotype male, USNM (examined). Country of type locality: USA.

**Geographical distribution.**NEA.

**NEA**: Canada (BC), USA (AK).

***Pholetesorhanniae* (Valerio & Whitfield, 2003)**

*Teremyshanniae* Valerio & Whitfield, 2003.

**Type information.** Holotype female, INBio (not examined but original description checked). Country of type locality: Costa Rica.

**Geographical distribution.**NEO.

**NEO**: Costa Rica.

***Pholetesorhayati* Akhtar, 2010**

*Pholetesorhayati* Akhtar, 2010.

**Type information.** Holotype female, INPC (not examined but original description checked). Country of type locality: India.

**Geographical distribution.**OTL.

**OTL**: India.

***Pholetesoringenuoides* (Papp, 1971), new combination**

*Apantelesingenuoides* Papp, 1971.

*Apantelesfrater* Tobias, 1976.

**Type information.** Holotype female, HNHM (not examined but original description checked). Country of type locality: Mongolia.

**Geographical distribution.**PAL.

**PAL**: Armenia, Bulgaria, Croatia, France, Germany, Greece, Hungary, Iran, Korea, Mongolia, Montenegro, Turkey.

**Notes.** Our concept for this species is based on Papp (1971, 1984). The descriptions and illustrations in those two papers strongly suggest this species is not an *Apanteles*. Until the type material can be examined, we consider that the best generic placement at present would be in *Pholetesor*, based on the shapes of T1 and T2, smooth propodeum, short hypopygium, and relatively short ovipositor sheaths. Another line of supporting evidence is that Papp (1971: 318) considered the species to be closely related to *ingenuus* (Tobias, 1964), which is currently placed within *Pholetesor*. However, *ingenuoides* could also be placed in *Dolichogenidea*; the two genera are closely related and unfortunately the papers we have consulted do not provide enough details to corroborate or refute that possibility.

***Pholetesoringenuus* (Tobias, 1964)**

*Apantelesingenuus* Tobias, 1964.

**Type information.** Holotype female, ZIN (not examined but subsequent treatment of the species checked). Country of type locality: Kazakhstan.

**Geographical distribution.**PAL.

**PAL**: Hungary, Kazakhstan, Mongolia.

**Notes.** Our species concept is based on Papp (1984a), Tobias (1986) and Kotenko (2007a).

***Pholetesorintercedens* (Tobias, 1977)**

*Apantelesintercedens* Tobias, 1977.

**Type information.** Holotype female, ZIN (not examined). Country of type locality: Russia.

**Geographical distribution.**PAL.

**PAL**: Russia (PRI).

***Pholetesorkuwayamai* (Watanabe, 1932), new combination**

*Apanteleskuwayamai* Watanabe, 1932.

**Type information.** Holotype female, EIHU (examined). Country of type locality: Japan.

**Geographical distribution.**PAL.

**PAL**: Japan, Korea.

**Notes.** This species is clearly not *Apanteles* but *Pholetesor*. Papp had recognized that (based on a label he wrote in 1992 and attached to the specimen, although that combination was never published). The female holotype is in poor condition, missing the metasoma and some legs, but two other females (supposedly paratypes, because they have the same labels) are in relatively good condition. In the same collection there is also a gelatin capsule with some other specimens and cocoons.

***Pholetesorlaetus* (Marshall, 1885)**

*Apanteleslaetus* Marshall, 1885.

*Apantelesmetallicus* Jakimavicius, 1972.

? *Microgasterexiguus* Haliday, 1834 [misidentification by Nixon (1973)].

? *Apantelessalalicus* Mason, 1959 [misidentification by van Achterberg (1997)].

**Type information.** Lectotype female, NHMUK (examined). Country of type locality: United Kingdom.

**Geographical distribution.**OTL, PAL.

**OTL**: China (FJ, GD, HI, HN, SN, YN, ZJ); **PAL**: Austria, Bulgaria, Germany, Hungary, Japan, Lithuania, Netherlands, Poland, Romania, Russia (ZAB, IRK, PRI, SAK), Slovenia, Switzerland, United Kingdom, Yugoslavia.

**Notes.**Wilkinson (1945: 155–156) designated a type for this species, which should be considered as the lectotype, but until now no reference to that specimen as the lectotype had been made. Originally, the specimen was stated to be in the Essex Museum of Natural History, but currently is in the NHMUK. For more details on *laetus* and the probable misidentifications of *exiguus* and *salalicus* see Shaw (2012b) and Broad et al. (2016).

***Pholetesorlithocolletis* Liu & Chen, 2016**

*Pholetesorlithocolletis* Liu & Chen, 2016.

**Type information.** Holotype female, ZJUH (not examined but original description checked). Country of type locality: China.

**Geographical distribution.**PAL.

**PAL**: China (SD).

***Pholetesorlongicoxis* Whitfield, 2006**

*Pholetesorlongicoxis* Whitfield, 2006.

**Type information.** Holotype female, CNC (examined). Country of type locality: Canada.

**Geographical distribution.**NEA.

**NEA**: Canada (QC), USA (MI).

***Pholetesorlyonetiae* Liu & Chen, 2016**

*Pholetesorlyonetiae* Liu & Chen, 2016.

**Type information.** Holotype female, ZJUH (not examined but original description checked). Country of type locality: China.

**Geographical distribution.**PAL.

**PAL**: China (SN).

***Pholetesormaritimus* (Wilkinson, 1941)**

*Apantelesmaritimus* Wilkinson, 1941.

**Type information.** Holotype female, NHMUK (examined). Country of type locality: United Kingdom.

**Geographical distribution.**OTL, PAL.

**OTL**: China (JS); **PAL**: China (AH, XJ), Denmark, France, Germany, Hungary, Kyrgyzstan, Poland, Russia (C, NW), Slovakia, United Kingdom.

**Notes.** The species distribution in Kyrgyzstan and Russia are based in Belokobylskij et al. (2019).

***Pholetesormasneri* (Mason, 1981)**

*Teremysmasneri* Mason, 1981.

**Type information.** Holotype female, CNC (examined). Country of type locality: Canada.

**Geographical distribution.**NEA.

**NEA**: Canada (ON), USA (CT).

***Pholetesormasoni* Whitfield, 2006**

*Pholetesormasoni* Whitfield, 2006.

**Type information.** Holotype female, USNM (examined). Country of type locality: USA.

**Geographical distribution.**NEA, NEO.

**NEA**: Canada (AB, BC, MB, NS, ON, QC, YT), USA (AZ, CA, IN, MD, MA, MI, MN, NY, NC, OH, WY); **NEO**: Mexico.

**Notes.** Because of its fully areolated propodeum, if this species indeed is to be placed in *Pholetesor*, then many *Parapanteles* that have similar propodeum areola could be considered to have the same generic placement. An alternative would be that *Pholetesormasoni* should be transferred to *Parapanteles*. More study on those species (including DNA and biological data) will be needed before a conclusion on the topic can be reached; for the time being we retain this species where it was originally described.

***Pholetesormoczari* Papp, 2014**

*Pholetesormoczari* Papp, 2014.

**Type information.** Holotype female, HNHM (not examined but original description checked). Country of type locality: Tunisia.

**Geographical distribution.**PAL.

**PAL**: Tunisia.

**Notes.** In the original description the generic position of this species and also that of *Pholetesorrufulus* (Tobias, 1964) are discussed; it is implied that both species could equally be placed in a different genus (*Glyptapanteles*) and that the “generic assignment depends mainly on the deliberation that which generic feature composition is considered more decisive to *Pholetesor* or to *Glyptapanteles*” (Papp 2014: 164). After reading the original description and studying the drawings that are provided there, we agree that the status of those two species is ambiguous at present. However, we refrain to transfer them to *Glyptapanteles* until specimens can be examined.

***Pholetesornanus* (Reinhard, 1880)**

*Apantelesnanus* Reinhard, 1880.

*Apantelesszoecsi* Papp, 1973.

**Type information.** Syntypes female and male, depository unknown (not examined but subsequent treatment of the species checked). Country of type locality: unknown.

**Geographical distribution.**NEA, PAL.

**NEA**: Canada (ON); **PAL**: Austria, Czech Republic, Finland, Germany, Hungary, Italy, Lithuania, Netherlands, Poland, Romania, Russia (C, IR, KA, NW), Serbia, Sweden, Switzerland, Ukraine, United Kingdom.

**Notes.** Our species concept is based on Nixon (1973), Papp (1983a), Kotenko (2007a), Shaw (2012b) and Fernandez-Triana et al (2016a).

***Pholetesorornigis* (Weed, 1887)**

*Apantelesornigis* Weed, 1887.

*Microgasterrobiniae* Fitch, 1859.

*Protapantelestortricis* Ashmead, 1898.

*Apantelesbraunae* Viereck, 1912.

*Apanteleslithocolletidis* Viereck, 1912.

**Type information.** Lectotype female, INHS (not examined but authoritatively identified specimens examined). Country of type locality: USA.

**Geographical distribution.**NEA.

**NEA**: Canada (MB, NB, NS, ON, QC), USA (AR, CA, CT, DC, IL, IN, KS, KY, MA, MI, MN, MO, NH, NY, NC, OR, PA, TX, UT, VT, VA, WV, WI).

**Notes.** We examined the types of *Apantelesbraunae* (Viereck, 1912), a male specimen, and *Apanteleslithocolletidis* (Viereck, 1912), a male specimen, currently synonyms of *P. ornigis* and both deposited in the USNM.

***Pholetesorphaetusa* (Nixon, 1973)**

*Apantelesphaetusa* Nixon, 1973.

**Type information.** Holotype female, NHMUK (examined). Country of type locality: United Kingdom.

**Geographical distribution.**PAL.

**PAL**: Bulgaria, Germany, Hungary, Korea, Mongolia, Netherlands, Romania, Russia (SAK), Ukraine, United Kingdom.

***Pholetesorpinifoliellae* Whitfield, 2006**

*Pholetesorpinifoliellae* Whitfield, 2006.

**Type information.** Holotype female, CNC (examined). Country of type locality: Canada.

**Geographical distribution.**NEA.

**NEA**: Canada (ON, QC), USA (CA, MD).

***Pholetesorpowelli* Whitfield, 2006**

*Pholetesorpowelli* Whitfield, 2006.

**Type information.** Holotype female, CAS (examined). Country of type locality: USA.

**Geographical distribution.**NEA.

**NEA**: USA (CA, OR).

**Notes.** The female holotype has the ovipositor sheaths ca. half as long as metatibia; the hypopygium has a translucent median fold, where one or two pleats are visible (second pleat not clearly defined); the hind wing vannal lobe is convex and entirely setose; the propodeum is rugose, without areola or median carina; and T1 and T2 are rugose. Most of those features could also be interpreted as being *Dolichogenidea*, especially the hypopygium pleats, but more study will be required, so the for the time being we prefer to retain this species in *Pholetesor*.

***Pholetesorpseudocircumscriptus* Abdoli, 2019**

*Pholetesorpseudocircumscriptus* Abdoli, 2019.

**Type information.** Holotype female, TMUC (not examined but original description checked). Country of type locality: Iran.

**Geographical distribution.**PAL.

**PAL**: Iran.

***Pholetesorrhygoplitoides* Whitfield, 2006**

*Pholetesorrhygoplitoides* Whitfield, 2006.

**Type information.** Holotype female, USNM (examined). Country of type locality: USA.

**Geographical distribution.**NEA.

**NEA**: Canada (AB, NL, ON, QC), USA (AZ, ID, MN).

***Pholetesorrohweri* (Muesebeck, 1921)**

*Apantelesrohweri* Muesebeck, 1921.

*Apantelesnigripes* Rohwer, 1913 [homonym of *Apantelesnigripes* Ratzeburg, 1844].

**Type information.** Holotype female, USNM (not examined but authoritatively identified specimens examined). Country of type locality: USA.

**Geographical distribution.**NEA.

**NEA**: Canada (NB, ON), USA (NY, PA, VA).

**Notes.** We examined the type of *Apantelesnigripes* (Rohwer, 1913), a female specimen deposited in the USNM.

***Pholetesorrufulus* (Tobias, 1964)**

*Apantelesrufulus* Tobias, 1964.

*Apantelesrufulus* Tobias, 1964 [homonym of *Apantelesrufulus* Wilkinson, 1930].

**Type information.** Holotype female, ZIN (not examined but subsequent treatment of the species checked). Country of type locality: Kazakhstan.

**Geographical distribution.**PAL.

**PAL**: Azerbaijan, Hungary, Kazakhstan, Turkey, Uzbekistan.

**Notes.** Our species concept is based on Papp (1983a) and Tobias (1986).

***Pholetesorsalalicus* (Mason, 1959)**

*Apantelessalalicus* Mason, 1959.

**Type information.** Holotype female, USNM (not examined but subsequent treatment of the species checked). Country of type locality: USA.

**Geographical distribution.**NEA, OTL, PAL.

**NEA**: Canada (BC, QC), USA (CA, OR); **OTL**: China (GZ, JS, ZJ); **PAL**: Finland, Netherlands, Norway, United Kingdom.

**Notes.** Our species concept is based on Whitfield (2006).

***Pholetesorsalicifoliellae* (Mason, 1959)**

*Apantelessalicifoliellae* Mason, 1959.

**Type information.** Holotype female, CNC (examined). Country of type locality: Canada.

**Geographical distribution.**NEA.

**NEA**: Canada (BC, MB, NB, NS, NT, ON, QC, YT), USA (AK, CA, NY, OR, UT).

***Pholetesorspinadensus* Liu & Chen, 2016**

*Pholetesorspinadensus* Liu & Chen, 2016.

**Type information.** Holotype female, ZJUH (not examined but original description checked). Country of type locality: China.

**Geographical distribution.**OTL.

**OTL**: China (GZ, SN).

***Pholetesortaiwanensis* Liu & Chen, 2016**

*Pholetesortaiwanensis* Liu & Chen, 2016.

**Type information.** Holotype female, ZJUH (not examined but original description checked). Country of type locality: China.

**Geographical distribution.**OTL.

**OTL**: China (TW).

***Pholetesorteresitergum* Liu & Chen, 2016**

*Pholetesorteresitergum* Liu & Chen, 2016.

**Type information.** Holotype female, ZJUH (not examined but original description checked). Country of type locality: China.

**Geographical distribution.**OTL, PAL.

**OTL**: China (ZJ); **PAL**: China (XJ).

***Pholetesorterneicus* Kotenko, 2007**

*Pholetesorterneicus* Kotenko, 2007.

**Type information.** Holotype female, ZIN (not examined but original description checked). Country of type locality: Russia.

**Geographical distribution.**PAL.

**PAL**: Russia (PRI).

***Pholetesorthuiellae* Whitfield, 2006**

*Pholetesorthuiellae* Whitfield, 2006.

**Type information.** Holotype female, CNC (examined). Country of type locality: Canada.

**Geographical distribution.**NEA.

**NEA**: Canada (NB, ON, QC), USA (CT, NY).

***Pholetesorvariabilis* Whitfield, 2006**

*Pholetesorvariabilis* Whitfield, 2006.

**Type information.** Holotype female, USNM (examined). Country of type locality: USA.

**Geographical distribution.**NEA.

**NEA**: Canada (AB, BC, ON, SK), USA (CA, CO, ID, MI, NV, OR, UT).

***Pholetesorviminetorum* (Wesmael, 1837)**

*Microgasterviminetorum* Wesmael, 1837.

*Microgasterfuliginosus* Wesmael, 1837.

**Type information.** Neotype female, ZMHB (not examined but subsequent treatment of the species checked). Country of type locality: Belgium.

**Geographical distribution.**NEA, OTL, PAL.

**NEA**: Canada (AB, BC, MB, NB, NL, NS, NT, ON, SK, YT), USA (AK, CO, ID, MI, MN, NH, SD, UT, WA, WY); **OTL**: China (SN); **PAL**: Azerbaijan, Belarus, Belgium, Croatia, Czech Republic, Estonia, Finland, France, Georgia, Germany, Hungary, Iran, Ireland, Italy, Japan, Kazakhstan, Kyrgyzstan, Moldova, Netherlands, Poland, Romania, Russia (AD, IRK, KEM, KDA, MOS, ORE, ROS, SPE, SMO, STA, VOR, YAR), Slovenia, Spain, Sweden, Switzerland, Ukraine, United Kingdom, Uzbekistan, Yugoslavia.

**Notes.** Our species concept is based on Whitfield (2006). The species distribution in Japan and Kyrgyzstan is based in Belokobylskij et al. (2019).

***Pholetesorzelleriae* Whitfield, 2006**

*Pholetesorzelleriae* Whitfield, 2006.

**Type information.** Holotype female, CNC (examined). Country of type locality: Canada.

**Geographical distribution.**NEA.

**NEA**: Canada (MB, ON, QC), USA (MI).

***Pholetesorzherikhini* Kotenko, 2007**

*Pholetesorzherikhini* Kotenko, 2007.

**Type information.** Holotype female, ZIN (not examined but original description checked). Country of type locality: Russia.

**Geographical distribution.**PAL.

**PAL**: Russia (PRI).

#### Genus Prasmodon Nixon, 1965

***Prasmodon*** Nixon, 1965: 205. Gender: masculine. Type species: *Prasmodoneminens* Nixon, 1965, by original designation.

This Neotropical genus was recently revised (Fernandez-Triana et al. 2014f) and at present comprises 18 described species, but we have seen a few more in collections. Most of the host records include the family Crambidae, with other two families recorded (Fernandez-Triana et al. 2014f, and ACG data available online) which require further verification. There are 204 DNA-barcode compliant sequences of this genus in BOLD, representing 15 BINs.

***Prasmodonalmasolisae* Fernandez-Triana & Whitfield, 2014**

*Prasmodonalmasolisae* Fernandez-Triana & Whitfield, 2014.

**Type information.** Holotype female, CNC (examined). Country of type locality: Costa Rica.

**Geographical distribution.**NEO.

**NEO**: Costa Rica.

***Prasmodonaureus* Fernandez-Triana & Whitfield, 2014**

*Prasmodonaureus* Fernandez-Triana & Whitfield, 2014.

**Type information.** Holotype female, CNC (examined). Country of type locality: Ecuador.

**Geographical distribution.**NEO.

**NEO**: Ecuador.

***Prasmodonbobpoolei* Fernandez-Triana & Whitfield, 2014**

*Prasmodonbobpoolei* Fernandez-Triana & Whitfield, 2014.

**Type information.** Holotype female, CNC (examined). Country of type locality: Costa Rica.

**Geographical distribution.**NEO.

**NEO**: Costa Rica.

***Prasmodonbobrobbinsi* Fernandez-Triana & Whitfield, 2014**

*Prasmodonbobrobbinsi* Fernandez-Triana & Whitfield, 2014.

**Type information.** Holotype female, CNC (examined). Country of type locality: Costa Rica.

**Geographical distribution.**NEO.

**NEO**: Costa Rica.

***Prasmodondondavisi* Fernandez-Triana & Whitfield, 2014**

*Prasmodondondavisi* Fernandez-Triana & Whitfield, 2014.

**Type information.** Holotype female, CNC (examined). Country of type locality: Costa Rica.

**Geographical distribution.**NEO.

**NEO**: Costa Rica.

***Prasmodoneminens* Nixon, 1965**

*Prasmodoneminens* Nixon, 1965.

**Type information.** Holotype male, NHMUK (examined). Country of type locality: Peru.

**Geographical distribution.**NEO.

**NEO**: Brazil (AM), Costa Rica, Ecuador, Peru.

***Prasmodonerenadupontae* Braet & Fernandez-Triana, 2014**

*Prasmodonerenadupontae* Braet & Fernandez-Triana, 2014.

**Type information.** Holotype female, MNHN (examined). Country of type locality: French Guiana.

**Geographical distribution.**NEO.

**NEO**: Brazil (MT), French Guiana.

***Prasmodonjohnbrowni* Fernandez-Triana & Whitfield, 2014**

*Prasmodonjohnbrowni* Fernandez-Triana & Whitfield, 2014.

**Type information.** Holotype female, CNC (examined). Country of type locality: Costa Rica.

**Geographical distribution.**NEO.

**NEO**: Costa Rica.

***Prasmodonmasoni* Fernandez-Triana & Whitfield, 2014**

*Prasmodonmasoni* Fernandez-Triana & Whitfield, 2014.

**Type information.** Holotype female, CNC (examined). Country of type locality: Brazil.

**Geographical distribution.**NEO.

**NEO**: Brazil (AM, MT).

***Prasmodonmikepoguei* Fernandez-Triana & Whitfield, 2014**

*Prasmodonmikepoguei* Fernandez-Triana & Whitfield, 2014.

**Type information.** Holotype male, CNC (examined). Country of type locality: Costa Rica.

**Geographical distribution.**NEO.

**NEO**: Costa Rica.

***Prasmodonnixoni* Fernandez-Triana & Whitfield, 2014**

*Prasmodonnixoni* Fernandez-Triana & Whitfield, 2014.

**Type information.** Holotype female, CNC (examined). Country of type locality: Peru.

**Geographical distribution.**NEO.

**NEO**: French Guiana, Peru.

***Prasmodonpaulgoldsteini* Fernandez-Triana & Whitfield, 2014**

*Prasmodonpaulgoldsteini* Fernandez-Triana & Whitfield, 2014.

**Type information.** Holotype male, CNC (examined). Country of type locality: Costa Rica.

**Geographical distribution.**NEO.

**NEO**: Costa Rica.

***Prasmodonscottmilleri* Fernandez-Triana & Whitfield, 2014**

*Prasmodonscottmilleri* Fernandez-Triana & Whitfield, 2014.

**Type information.** Holotype female, CNC (examined). Country of type locality: Costa Rica.

**Geographical distribution.**NEO.

**NEO**: Costa Rica.

***Prasmodonsilvatlanticus* Fernandez-Triana & Whitfield, 2014**

*Prasmodonsilvatlanticus* Fernandez-Triana & Whitfield, 2014.

**Type information.** Holotype female, CNC (examined). Country of type locality: Brazil.

**Geographical distribution.**NEO.

**NEO**: Brazil (RJ).

***Prasmodonsubfuscus* Fernandez-Triana & Whitfield, 2014**

*Prasmodonsubfuscus* Fernandez-Triana & Whitfield, 2014.

**Type information.** Holotype male, CNC (examined). Country of type locality: Brazil.

**Geographical distribution.**NEO.

**NEO**: Brazil (RJ).

***Prasmodontijucaensis* Fernandez-Triana & Whitfield, 2014**

*Prasmodontijucaensis* Fernandez-Triana & Whitfield, 2014.

**Type information.** Holotype female, CNC (examined). Country of type locality: Brazil.

**Geographical distribution.**NEO.

**NEO**: Brazil (RJ).

***Prasmodonverhoogdenokus* Braet & Fernandez-Triana, 2014**

*Prasmodonverhoogdenokus* Braet & Fernandez-Triana, 2014.

**Type information.** Holotype female, CNC (examined). Country of type locality: Brazil.

**Geographical distribution.**NEO.

**NEO**: Brazil (MT), Colombia, Ecuador, French Guiana, Peru, Suriname.

***Prasmodonzlotnicki* Valerio & Rodriguez, 2005**

*Prasmodonzlotnicki* Valerio & Rodriguez, 2005.

**Type information.** Holotype female, INBio (not examined but subsequent treatment of the species checked). Country of type locality: Costa Rica.

**Geographical distribution.**NEO.

**NEO**: Costa Rica.

**Notes.** Our species concept is based on Fernandez-Triana et al. (2014f).

#### Genus Promicrogaster Brues & Richardson, 1913

***Promicrogaster*** Brues & Richardson, 1913: 499. Gender: feminine. Type species: *Promicrogasterterebrator* Brues & Richardson, 1913, by original designation.

Until very recently (e.g., Fernandez-Triana et al. 2016b), this genus was considered restricted to the New World. However, during the preparation of this paper we found evidence than this taxon is cosmopolitan, reported below. Currently, there are 46 described species of *Promicrogaster*, with recent reviews of the Mesoamerican (Fernandez-Triana et al. 2016b) and North American species (Fernandez-Triana 2019). We have seen many more species in collections, mostly from the Neotropical region. Known hosts are from the families Sessidae and Tineidae. There are 134 DNA-barcode compliant sequences of this genus in BOLD, representing 37 BINs.

***Promicrogasteralexmartinezi* Fernandez-Triana & Boudreault, 2016**

*Promicrogasteralexmartinezi* Fernandez-Triana & Boudreault, 2016.

**Type information.** Holotype female, CNC (examined). Country of type locality: Costa Rica.

**Geographical distribution.**NEO.

**NEO**: Costa Rica.

***Promicrogasterandreyvallejosi* Fernandez-Triana & Boudreault, 2016**

*Promicrogasterandreyvallejosi* Fernandez-Triana & Boudreault, 2016.

**Type information.** Holotype female, CNC (examined). Country of type locality: Costa Rica.

**Geographical distribution.**NEO.

**NEO**: Costa Rica.

***Promicrogasterapharea* Nixon, 1965**

*Promicrogasterapharea* Nixon, 1965.

**Type information.** Holotype female, NHMUK (examined). Country of type locality: Mexico.

**Geographical distribution.**NEO.

**NEO**: Brazil (SC), Mexico.

***Promicrogasterapidanus* (Nixon, 1965), new combination**

*Apantelesapidanus* Nixon, 1965.

**Type information.** Holotype female, NHMUK (examined). Country of type locality: South Africa.

**Geographical distribution.**AFR.

**AFR**: South Africa.

**Notes.** Although the well defined, strong median carina on the propodeum, and the species group to which Nixon (1965) assigned this species might suggest it belongs to *Iconella*, other characters indicate a different genus. The ovipositor tip is sinuate (versus straight in *Iconella*); the ovipositor and sheaths are relatively very long (versus ca. twice metatibia length, much longer than in described species of *Iconella*); and the hind wing vein cu-a is straight (versus sinuate in *Iconella*). We consider that the available evidence provides more support for this species to be placed in *Promicrogaster*.

***Promicrogasterbrandondinartei* Fernandez-Triana & Boudreault, 2016**

*Promicrogasterbrandondinartei* Fernandez-Triana & Boudreault, 2016.

**Type information.** Holotype female, CNC (examined). Country of type locality: Costa Rica.

**Geographical distribution.**NEO.

**NEO**: Costa Rica, Panama.

***Promicrogasterbriareus* (Nixon, 1965), new combination**

*Apantelesbriareus* Nixon, 1965.

**Type information.** Holotype female, NHMUK (examined). Country of type locality: Australia.

**Geographical distribution.**AUS.

**AUS**: Australia (QLD), Vanuatu.

**Notes.**Austin and Dangerfield (1992) transferred this species from *Apanteles* to *Iconella*. However, *briareus* lacks the two main characters defining *Iconella*: the hind wing does not have the vein cu-a sinuate; and its propodeum does not have a median, longitudinal carina but instead has a few, shorter carinae radiating from the nucha which seem to partially define an areola (Austin and Dangerfield (1992: 36) referred to that as “more diffuse posterior striae”). After examining the holotype, we found that the ovipositor tip is sinuate, the ovipositor length is almost twice that of the metatibia, and the polished area of the lateral face of the scutellar disc occupies most of the face. All those characters suggest this species is better placed in *Promicrogaster*, a genus that was recently considered to be found only in the New World (e.g., Fernandez-Triana et al. 2016b). The report in this paper of species from the Afrotropical and Australasian regions indicate a much wider distribution of *Promicrogaster* worldwide.

***Promicrogastercara* Nixon, 1965**

*Promicrogastercarus* Nixon, 1965.

**Type information.** Holotype female, NHMUK (examined). Country of type locality: Brazil.

**Geographical distribution.**NEO.

**NEO**: Brazil (BA).

**Notes.** The species name must be treated as an adjective and not as a noun (Doug Yanega, pers. comm.) and thus it must match the gender of the genus name.

***Promicrogasterconopiae* (Watanabe, 1934), new combination**

*Apantelesconopiae* Watanabe, 1934.

**Type information.** Holotype female, EIHU (examined). Country of type locality: Japan.

**Geographical distribution.**OTL, PAL.

**OTL**: Malaysia; **PAL**: China (QH), Japan, Korea.

**Notes.** We have examined the holotype and several more specimens in the EIHU collection. They look similar to the described species from the New World, based on the sinuate ovipositor tip, shapes of T1 andT2, large metacoxa and relatively high polished area of the lateral face of the scutellum. The only differences we observed were that the Japanese specimens (which are relatively large, at least by *Promicrogaster* standards) do not have a bilobate glossa and the fore wing lacks an areolet; in the New World, the currently described species all have an elongate and bilobate glossa, and all large species have a small areolet in the fore wing (with only a few small species lacking the areolet in the fore wing). These differences are minor and thus we consider that the best generic placement for this species is in *Promicrogaster*. The known host data for this species (Sesiidae) also agree with the very few host records known from the New World (Fernandez-Triana et al. 2016b).

***Promicrogasterdaniellopezi* Fernandez-Triana & Boudreault, 2016**

*Promicrogasterdaniellopezi* Fernandez-Triana & Boudreault, 2016.

**Type information.** Holotype female, CNC (examined). Country of type locality: Costa Rica.

**Geographical distribution.**NEO.

**NEO**: Costa Rica.

***Promicrogasterdaretrizoi* Fernandez-Triana & Boudreault, 2016**

*Promicrogasterdaretrizoi* Fernandez-Triana & Boudreault, 2016.

**Type information.** Holotype female, CNC (examined). Country of type locality: Costa Rica.

**Geographical distribution.**NEO.

**NEO**: Costa Rica.

***Promicrogastereddycastroi* Fernandez-Triana & Boudreault, 2016**

*Promicrogastereddycastroi* Fernandez-Triana & Boudreault, 2016.

**Type information.** Holotype female, CNC (examined). Country of type locality: Costa Rica.

**Geographical distribution.**NEO.

**NEO**: Costa Rica.

***Promicrogastereimyobandoae* Fernandez-Triana & Boudreault, 2016**

*Promicrogastereimyobandoae* Fernandez-Triana & Boudreault, 2016.

**Type information.** Holotype female, CNC (examined). Country of type locality: Costa Rica.

**Geographical distribution.**NEO.

**NEO**: Costa Rica.

***Promicrogasteremesa* (Nixon, 1965), new combination**

*Apantelesemesa* Nixon, 1965.

**Type information.** Holotype female, NHMUK (examined). Country of type locality: South Africa.

**Geographical distribution.**AFR.

**AFR**: South Africa.

**Notes.** We transfer this species to *Promicrogaster* based on the ovipositor length being more than twice the metatibia length, sinuate ovipositor tip, propodeum with irregular carinae radiating from the nucha, a large polished area of the lateral face of scutellum, and relatively large metacoxae.

***Promicrogastererigone* Nixon, 1965**

*Promicrogastererigone* Nixon, 1965.

**Type information.** Holotype female, NHMUK (examined). Country of type locality: Brazil.

**Geographical distribution.**NEO.

**NEO**: Brazil (SC).

***Promicrogasterfabiancastroi* Fernandez-Triana & Boudreault, 2016**

*Promicrogasterfabiancastroi* Fernandez-Triana & Boudreault, 2016.

**Type information.** Holotype female, CNC (examined). Country of type locality: Costa Rica.

**Geographical distribution.**NEO.

**NEO**: Costa Rica.

***Promicrogasterfabriciocambroneroi* Fernandez-Triana & Boudreault, 2016**

*Promicrogasterfabriciocambroneroi* Fernandez-Triana & Boudreault, 2016.

**Type information.** Holotype female, CNC (examined). Country of type locality: Costa Rica.

**Geographical distribution.**NEO.

**NEO**: Costa Rica.

***Promicrogasterfloridakeys* Fernandez-Triana, 2019**

*Promicrogasterfloridakeys* Fernandez-Triana, 2019.

**Type information.** Holotype female, CNC (examined). Country of type locality: USA.

**Geographical distribution.**NEA.

**NEA**: USA (FL).

***Promicrogastergainesvillensis* Fernandez-Triana, 2019**

*Promicrogastergainesvillensis* Fernandez-Triana, 2019.

**Type information.** Holotype female, CNC (examined). Country of type locality: USA.

**Geographical distribution.**NEA.

**NEA**: USA (FL).

***Promicrogastergrandicula* (Wilkinson, 1929), new combination**

*Apantelesgrandiculus* Wilkinson, 1929.

**Type information.** Holotype female, NHMUK (examined). Country of type locality: India.

**Geographical distribution.**OTL.

**OTL**: China (FJ), India, Vietnam.

**Notes.** We transfer this species to *Promicrogaster* based on the ovipositor length ca. twice the metatibia length, sinuate ovipositor tip, propodeum with irregular carinae radiating from the nucha, a large polished area of the lateral face of the scutellum, and relatively large metacoxae. The species name must be treated as an adjective and not as a noun (Doug Yanega, pers. comm.) and thus it must match the gender of the genus name.

***Promicrogasterhillaryvillafuerteae* Fernandez-Triana & Boudreault, 2016**

*Promicrogasterhillaryvillafuerteae* Fernandez-Triana & Boudreault, 2016.

**Type information.** Holotype female, CNC (examined). Country of type locality: Costa Rica.

**Geographical distribution.**NEO.

**NEO**: Costa Rica.

***Promicrogasterhuachuca* Fernandez-Triana, 2019**

*Promicrogasterhuachuca* Fernandez-Triana, 2019.

**Type information.** Holotype female, CNC (examined). Country of type locality: USA.

**Geographical distribution.**NEA.

**NEA**: USA (AZ).

***Promicrogasterjaymeae* Fernandez-Triana, 2019**

*Promicrogasterjaymeae* Fernandez-Triana, 2019.

**Type information.** Holotype female, CNC (examined). Country of type locality: Canada.

**Geographical distribution.**NEA.

**NEA**: Canada (ON), USA (MA).

***Promicrogasterkevinmartinezi* Fernandez-Triana & Boudreault, 2016**

*Promicrogasterkevinmartinezi* Fernandez-Triana & Boudreault, 2016.

**Type information.** Holotype female, CNC (examined). Country of type locality: Costa Rica.

**Geographical distribution.**NEO.

**NEO**: Costa Rica.

***Promicrogasterkiralycastilloae* Fernandez-Triana & Boudreault, 2016**

*Promicrogasterkiralycastilloae* Fernandez-Triana & Boudreault, 2016.

**Type information.** Holotype female, CNC (examined). Country of type locality: Costa Rica.

**Geographical distribution.**NEO.

**NEO**: Costa Rica.

***Promicrogasterleilycastilloae* Fernandez-Triana & Boudreault, 2016**

*Promicrogasterleilycastilloae* Fernandez-Triana & Boudreault, 2016.

**Type information.** Holotype female, CNC (examined). Country of type locality: Costa Rica.

**Geographical distribution.**NEO.

**NEO**: Costa Rica.

***Promicrogasterliagrantae* Fernandez-Triana & Boudreault, 2016**

*Promicrogasterliagrantae* Fernandez-Triana & Boudreault, 2016.

**Type information.** Holotype female, CNC (examined). Country of type locality: Costa Rica.

**Geographical distribution.**NEO.

**NEO**: Costa Rica.

***Promicrogasterluismendezi* Fernandez-Triana & Boudreault, 2016**

*Promicrogasterluismendezi* Fernandez-Triana & Boudreault, 2016.

**Type information.** Holotype female, CNC (examined). Country of type locality: Costa Rica.

**Geographical distribution.**NEO.

**NEO**: Costa Rica.

***Promicrogastermadreanensis* Fernandez-Triana, 2019**

*Promicrogastermadreanensis* Fernandez-Triana, 2019.

**Type information.** Holotype female, CNC (examined). Country of type locality: USA.

**Geographical distribution.**NEA.

**NEA**: USA (AZ).

***Promicrogastermerella* Nixon, 1965**

*Promicrogastermerella* Nixon, 1965.

**Type information.** Holotype female, NHMUK (examined). Country of type locality: Brazil.

**Geographical distribution.**NEO.

**NEO**: Brazil (SC).

***Promicrogastermiranda* Muesebeck, 1958**

*Promicrogastermiranda* Muesebeck, 1958.

**Type information.** Holotype female, USNM (not examined but subsequent treatment of the species checked). Country of type locality: Panama.

**Geographical distribution.**NEO.

**NEO**: Panama, Trinidad & Tobago.

**Notes.** Our species concept is based on Fernandez-Triana et al. (2016b).

***Promicrogastermonteverdensis* Fernandez-Triana & Boudreault, 2016**

*Promicrogastermonteverdensis* Fernandez-Triana & Boudreault, 2016.

**Type information.** Holotype female, CNC (examined). Country of type locality: Costa Rica.

**Geographical distribution.**NEO.

**NEO**: Costa Rica.

***Promicrogastermunda* Muesebeck, 1958**

*Promicrogastermunda* Muesebeck, 1958.

**Type information.** Holotype female, USNM (not examined but subsequent treatment of the species checked). Country of type locality: Honduras.

**Geographical distribution.**NEO.

**NEO**: Costa Rica, Honduras, Mexico, Panama.

**Notes.** Our species concept is based on Fernandez-Triana et al. (2016b). According to those authors, *P. munda* may actually represent a species complex.

***Promicrogasternaomiduarteae* Fernandez-Triana & Boudreault, 2016**

*Promicrogasternaomiduarteae* Fernandez-Triana & Boudreault, 2016.

**Type information.** Holotype female, CNC (examined). Country of type locality: Costa Rica.

**Geographical distribution.**NEO.

**NEO**: Costa Rica.

***Promicrogasterorsedice* (Nixon, 1965), new combination**

*Apantelesorsedice* Nixon, 1965.

**Type information.** Holotype female, NHMUK (examined). Country of type locality: Papua New Guinea.

**Geographical distribution.**AUS, OTL.

**AUS**: Papua New Guinea; **OTL**: Vietnam.

**Notes.** We transfer this species to *Promicrogaster* based on the ovipositor length more than twice the metatibia length, sinuate ovipositor tip, propodeum with irregular carinae radiating from the nucha, a large polished area of the lateral face of scutellum, and relatively large metacoxae.

***Promicrogasterpolyporicola* Muesebeck, 1958**

*Promicrogasterpolyporicola* Muesebeck, 1958.

**Type information.** Holotype female, USNM (examined). Country of type locality: Panama.

**Geographical distribution.**NEO.

**NEO**: Panama.

***Promicrogasterprater* Nixon, 1965**

*Promicrogasterprater* Nixon, 1965.

**Type information.** Holotype female, NHMUK (examined). Country of type locality: Brazil.

**Geographical distribution.**NEO.

**NEO**: Brazil (SC).

***Promicrogasterrepleta* (Papp, 1990), new combination**

*Iconellarepleta* Papp, 1990.

**Type information.** Holotype female, HNHM (not examined but original description checked). Country of type locality: Korea.

**Geographical distribution.**PAL.

**PAL**: Korea.

**Notes.** Transferred to *Promicrogaster* based on the the sinuate ovipositor tip, shape of T1–T2, large metacoxa and relatively high polished area on the lateral face of scutellum (Papp 1990b).

***Promicrogasterrondeau* Fernandez-Triana, 2019**

*Promicrogasterrondeau* Fernandez-Triana, 2019.

**Type information.** Holotype female, CNC (examined). Country of type locality: Canada.

**Geographical distribution.**NEA.

**NEA**: Canada (ON).

***Promicrogasterronycastilloi* Fernandez-Triana & Boudreault, 2016**

*Promicrogasterronycastilloi* Fernandez-Triana & Boudreault, 2016.

**Type information.** Holotype female, CNC (examined). Country of type locality: Costa Rica.

**Geographical distribution.**NEO.

**NEO**: Costa Rica.

***Promicrogastersebastiancambroneroi* Fernandez-Triana & Boudreault, 2016**

*Promicrogastersebastiancambroneroi* Fernandez-Triana & Boudreault, 2016.

**Type information.** Holotype female, CNC (examined). Country of type locality: Costa Rica.

**Geographical distribution.**NEO.

**NEO**: Costa Rica.

***Promicrogasterspilopterus* Nixon, 1965**

*Promicrogasterspilopterus* Nixon, 1965.

**Type information.** Holotype female, NHMUK (examined). Country of type locality: Brazil.

**Geographical distribution.**NEO.

**NEO**: Brazil (SC).

***Promicrogastersterope* Nixon, 1965**

*Promicrogastersterope* Nixon, 1965.

**Type information.** Holotype female, NHMUK (examined). Country of type locality: Brazil.

**Geographical distribution.**NEO.

**NEO**: Brazil (SC).

***Promicrogasterterebrator* Brues & Richardson, 1913**

*Promicrogasterterebrator* Brues & Richardson, 1913.

**Type information.** Holotype female, AMNH (examined). Country of type locality: Guyana.

**Geographical distribution.**NEO.

**NEO**: Guyana.

**Notes.** Our species concept is based on Brues and Richardson (1913), Muesebeck (1958b) and Mason (1981).

***Promicrogastertracyvindasae* Fernandez-Triana & Boudreault, 2016**

*Promicrogastertracyvindasae* Fernandez-Triana & Boudreault, 2016.

**Type information.** Holotype female, CNC (examined). Country of type locality: Costa Rica.

**Geographical distribution.**NEO.

**NEO**: Costa Rica.

***Promicrogastertyphon* (Nixon, 1965), new combination**

*Apantelestyphon* Nixon, 1965.

**Type information.** Holotype female, NHMUK (examined). Country of type locality: Togo.

**Geographical distribution.**AFR.

**AFR**: South Africa, Togo.

**Notes.** The ovipositor tip is sinuate, the ovipositor length is almost twice that of metatibia, the propodeum has a series of short carinae radiating from the nucha, and the polished area of the lateral face of the scutellar disc occupies most of the face. These characters indicate that this species is better placed in *Promicrogaster*.

***Promicrogastervirginiana* Fernandez-Triana, 2019**

*Promicrogastervirginianus* Fernandez-Triana, 2019.

**Type information.** Holotype female, CNC (examined). Country of type locality: USA.

**Geographical distribution.**NEA.

**NEA**: USA (VA).

**Notes.** The species name must be treated as an adjective and not as a noun (Doug Yanega, pers. comm.) and thus it must match the gender of the genus name.

#### Genus Protapanteles Ashmead, 1898

***Protapanteles*** Ashmead, 1898: 166. Gender: masculine. Type species: (*Protapantelesephyrae* Ashmead, 1898) = *Apantelespaleacritae* Riley, 1881, by subsequent designation (Viereck 1914: 123).

We record 25 described species, although the limits of this genus are controversial (see discussion above in section Brief diagnosis of all Microgastrinae genera as they are understood in this paper for more details, p 35, 36), and it is difficult to even estimate the potential species richness. As considered in this paper, *Protapanteles* is essentially Holarctic, occasionally reaching the Oriental region. Many Lepidoptera families have been recorded as hosts but, as the limits of this genus have varied considerably (e.g., Mason, 1981, Yu et al. 2016, present paper), all records need to be verified. There are 481 DNA-barcode compliant sequences of this genus in BOLD, representing 26 BINs, but some of those sequences are likely to represent other genera.

***Protapantelesalaskensis* Ashmead, 1902, restored combination**

*Protapantelesalaskensis* Ashmead, 1902.

**Type information.** Holotype male, USNM (examined). Country of type locality: USA.

**Geographical distribution.**NEA.

**NEA**: Canada (BC, MB, ON, QC, NL), USA (AK, CA, MI).

**Notes.** Both Shenefelt (1972: 437) and Yu et al. (2016) referred to the holotype of this species as a female specimen, and Shenefelt even recorded the type number (5704). However, examination of the holotype (which indeed has the same type number that Shenefelt mentioned) clearly shows that it is a male specimen, and thus we are correcting that information here. Yu et al. (2016) listed this species as belonging to the genus *Cotesia*, without any valid (published) paper to support that change. After studying the holotype and other specimens, we think this species is better placed in *Protapanteles*, based on the propodeum mostly smooth, with a few short striae near the nucha, T1 smooth and mostly parallel-sided (but slightly narrowing on posterior 0.2), and T2 subtriangular to trapezoidal in shape and with a smooth, poorly defined median area. For the sake of clarity, we restore the species combination here.

***Protapantelesanchisiades* (Nixon, 1973)**

*Apantelesanchisiades* Nixon, 1973.

**Type information.** Holotype female, NHMUK (examined). Country of type locality: United Kingdom.

**Geographical distribution.**PAL.

**PAL**: Bulgaria, Czech Republic, Finland, Germany, Hungary, Italy, Korea, Mongolia, Netherlands, Norway, Poland, Russia (KAM, PRI, SAK), Slovakia, Sweden, Switzerland, Turkey, Ukraine, United Kingdom.

***Protapantelesandromica* (Nixon, 1976)**

*Apantelesandromica* Nixon, 1976.

**Type information.** Holotype female, ZSM (not examined but original description checked). Country of type locality: Germany.

**Geographical distribution.**PAL.

**PAL**: Germany, Hungary, Poland, Russia (C, S), Slovakia.

***Protapantelesarmeniacus* (Tobias, 1976)**

*Apantelesarmeniacus* Tobias, 1976.

**Type information.** Holotype female, depository unknown (not examined but subsequent treatment of the species checked). Country of type locality: Armenia.

**Geographical distribution.**PAL.

**PAL**: Armenia.

**Notes.** Our species concept is based on Papp (1984a) and Tobias (1986).

***Protapantelesbuzurae* (You, Xiong & Zhou, 1987)**

*Apantelesbuzurae* You, Xiong & Zhou, 1987.

**Type information.** Holotype female, HUNAU (not examined but subsequent treatment of the species checked). Country of type locality: China.

**Geographical distribution.**OTL.

**OTL**: China (YN).

**Notes.** Our species concept is based on Chen and Song (2004) and Kotenko (2007a).

***Protapantelesdelitutus* (Papp, 1984)**

*Apantelesdelitutus* Papp, 1984.

**Type information.** Holotype female, RBINS (not examined but original description checked). Country of type locality: Belgium.

**Geographical distribution.**PAL.

**PAL**: Belgium, Germany, Hungary, Netherlands, Slovakia.

***Protapantelesendemus* (Nixon, 1965)**

*Apantelesendemus* Nixon, 1965.

**Type information.** Holotype female, NHMUK (examined). Country of type locality: United Kingdom.

**Geographical distribution.**PAL.

**PAL**: France, Hungary, Kazakhstan, Russia (ZAB, SPE), Switzerland, Ukraine, United Kingdom.

**Notes.** Until the limits of *Protapanteles* are clearly established (the validity of this genus is questionable), we prefer not to transfer species to other genera. But it is likely that *endemus* will be placed in *Cotesia* because it has a propodeum with a transverse carina (in addition to other sculpture), and the shapes of T1 and T2 are closer to typical *Cotesia* than to *Protapanteles*. The holotype does not have a specialized seta on the protarsus.

***Protapantelesenephes* (Nixon, 1965)**

*Apantelesenephes* Nixon, 1965.

**Type information.** Holotype female, NHMUK (examined). Country of type locality: United Kingdom.

**Geographical distribution.**PAL.

**PAL**: Germany, Hungary, Korea, Russia (AMU, PRI, SAK), Slovakia, Sweden, Switzerland, Turkmenistan, Ukraine, United Kingdom.

**Notes.** Until the limits of *Protapanteles* are clearly established (the validity of this genus is questionable), we prefer not to transfer species to other genera. But it is likely that *enephes* will be placed in *Cotesia*. This species was recently recorded from Brazil (Penteado-Dias et al. 2011), which would represent a significant range expansion, as *Protapantelesenephes* was only known from the Palearctic region (e.g., Yu et al. 2016). We have examined the holotype as well as many European specimens (deposited in the CNC) versus the illustrations and description in Penteado-Dias et al. (2011), and it is clear that the Brazilian specimens, although sharing with *enephes* the relatively unusual pale spot on the gena, actually represent a completely different species. The Brazilian species remains undescribed at present, and we are not even sure of its generic status (as the images of propodeum, T1, and T2 suggest it could be a species of *Cotesia* or perhaps even *Nyereria*).

***Protapanteleshirtariae* (Kotenko & Tobias, 1986)**

*Apanteleshirtariae* Kotenko & Tobias, 1986.

**Type information.** Holotype female, SIZK (not examined but subsequent treatment of the species checked). Country of type locality: Russia.

**Geographical distribution.**PAL.

**PAL**: Russia (VGG), United Kingdom.

**Notes.** Our species concept is based on Tobias (1986), Papp (1988), Kotenko (2006) and Shaw (2012b).

***Protapantelesiapetus* (Nixon, 1976)**

*Apantelesiapetus* Nixon, 1976.

**Type information.** Holotype female, ZSM (not examined but original description checked). Country of type locality: Germany.

**Geographical distribution.**PAL.

**PAL**: Germany.

***Protapantelesimmunis* (Haliday, 1834)**

*Microgasterimmunis* Haliday, 1834.

**Type information.** Lectotype female, NMID (not examined but subsequent treatment of the species checked). Country of type locality: Ireland.

**Geographical distribution.**NEA, PAL.

**NEA**: Greenland; **PAL**: Armenia, Austria, Bulgaria, Croatia, Estonia, Finland, Germany, Hungary, Ireland, Italy, Kazakhstan, Korea, Lithuania, Moldova, Netherlands, Norway, Poland, Romania, Russia (ZAB, NVS, PRI, SAK, TOM, VOR), Serbia, Slovakia, Sweden, Switzerland, Tunisia, Ukraine, United Kingdom.

**Notes.** Our species concept is based on Nixon (1976), Papp (1984a), Tobias (1986), van Achterberg (2006) and Kotenko (2007a).

***Protapantelesincertus* (Ruthe, 1859)**

*Microgasterincertus* Ruthe, 1859.

*Apantelescaberae* Marshall, 1885.

*Apantelesjugosus* Lyle, 1916.

*Apantelesmihalyii* Papp, 1973.

**Type information.** Holotype male, NHMW (not examined but authoritatively identified specimens examined). Country of type locality: Iceland.

**Geographical distribution.**PAL.

**PAL**: Austria, Azerbaijan, Georgia, Germany, Hungary, Iceland, Italy, Mongolia, Poland, Romania, Russia (VOR), Slovakia, Sweden, Switzerland, Ukraine, United Kingdom, Yugoslavia.

**Notes.** We examined the female type of *Apantelescaberae* (Marshall, 1885) and the type series (syntypes) of *Apantelesjugosus* (Lyle, 1916), which are deposited in the NHMUK. The species distribution in Azerbaijan and Georgia is based on Belokobylskij et al. (2019).

***Protapanteleslymantriae* (Marsh, 1979)**

*Apanteleslymantriae* Marsh, 1979.

**Type information**. Holotype female, USNM (examined). Country of type locality: Japan.

**Geographical distribution.**PAL.

**PAL**: Japan.

**Notes.** This species was described as *Apanteles*, as it predated the paper by Mason (1981) where *Apanteles* was split into many genera. After the original description, Maeto (1996) referred to the species as *Protapanteles*, although he did not specify that as a new combination. Yu et al. (2016) considered the species to belong to *Cotesia*, although there is no published reference that we are aware of for the treatment of *lymantriae* in that genus. We have examined the holotype and the best generic placement at present is in *Protapanteles*, based on the sculpture of propodeum, T1, and T2, the shapes of T1 and T2, and presence of a spine on the fore tarsus. For the sake of clarity, we revise the species combination here.

***Protapantelesmandanis* (Nixon, 1965)**

*Apantelesmandanis* Nixon, 1965.

**Type information.** Holotype female, NHMUK (examined). Country of type locality: Germany.

**Geographical distribution.**PAL.

**PAL**: Finland, Germany, Hungary, Switzerland.

**Notes.** Until the limits of *Protapanteles* are clearly established (the validity of this genus is questionable), we prefer not to transfer species to other genera. But it is very likely that *mandanis* will be placed in *Glyptapanteles* because it has a propodeum without any strong carinae or sculpture (at most a few short carinae radiating from the nucha), and the shapes of T1 and T2 are closer to typical *Glyptapanteles* than to *Protapanteles*. Additionally, the holotype does not have a specialized seta on the protarsus.

***Protapantelesneparallelus* Kotenko, 2007**

*Protapantelesneparallelus* Kotenko, 2007.

**Type information.** Holotype female, SIZK (not examined but original description checked). Country of type locality: Russia.

**Geographical distribution.**PAL.

**PAL**: Russia (ZAB).

***Protapantelespaleacritae* (Riley, 1881)**

*Apantelespaleacritae* Riley, 1881.

*Protapantelesephyrae* Ashmead, 1898.

**Type information.** Lectotype male, USNM (examined). Country of type locality: USA.

**Geographical distribution.**NEA.

**NEA**: Canada (BC, MB, NL, NS, ON), USA (AR, DE, GA, IL, KS, MD, MA, MO, NH, NJ, NY, VT, VA, WV).

**Notes.**Shenefelt (1972: 591) designated a lectotype from the four available syntypes and stated that it was “The specimen on point no. 2 directly ahead of the cocoon on the card”. We have examined the series, and the lectotype is a male specimen, a correction reflected in the type details we present here. Of the three remaining specimens (paralectotypes) one is entirely missing (except for two legs glued to the card) and the other two are missing the metasoma. Apart from the type material of *P. paleacritae*, we also examined the male holotype of *P. ephyrae* Ashmead, 1898 (currently a synonym of *P. paleacritae*), which has the metasoma detached but glued to another card.

***Protapantelesparallelus* (Lyle, 1917)**

*Apantelesparallelus* Lyle, 1917.

*Apantelesparallelus* Lyle, 1917 [secondary homonym of *Cotesiaparallelis* (Ashmead, 1900)].

*Apanteleslylei* Shenefelt, 1972 [new name for secondary homonym].

**Type information.** Holotype female, NHMUK (examined). Country of type locality: United Kingdom.

**Geographical distribution.**PAL.

**PAL**: Germany, Hungary, Russia (ZAB), United Kingdom.

***Protapantelesphigaliae* (Muesebeck, 1919)**

*Apantelesphigaliae* Muesebeck, 1919.

**Type information.** Holotype female, USNM (examined). Country of type locality: USA.

**Geographical distribution.**NEA.

**NEA**: Canada (NB, ON), USA (MA, NJ).

**Notes.** This species was transferred to *Protapanteles* by Mason (1981), also followed by Whitfield (1995a) and Fernandez-Triana (2010). However, Yu et al. (2016) considered the species to belong to *Cotesia*, although there is no published reference that we are aware of for this treatment of *phigaliae*. We have examined the holotype and the best generic placement at present is in *Protapanteles*, based on the mostly smooth propodeum, T1 and T2, as well as shape of T1 (mostly parallel-sided, but narrowing towards apex on posterior 0.3), and shape of T2 (subtriangular). For the sake of clarity, we revise the species combination here.

***Protapantelesphlyctaeniae* (Muesebeck, 1929)**

*Apantelesphlyctaeniae* Muesebeck, 1929.

**Type information.** Holotype female, USNM (examined). Country of type locality: USA.

**Geographical distribution.**NEA.

**NEA**: Canada (ON), USA (IL, KS, OH).

**Notes.** This species was transferred to *Protapanteles* by Mason (1981), also followed by Whitfield (1995a) and Fernandez-Triana (2010). However, Yu et al. (2016) considered the species to belong to *Cotesia*, although there is no published reference that we are aware of for the treatment of *phlyctaeniae* in that genus. We have examined the holotype and the best generic placement at present is in *Protapanteles*, based on the shape of T1 (mostly parallel-sided, but narrowing towards apex on posterior 0.3) and the fore tarsus with a spine. Available DNA barcodes (with sequence lengths 164–422 bp, from three authenticated specimens) also place *phlyctaeniae* close to other *Protapanteles* species and not *Cotesia*. For the sake of clarity, we revise the species combination here.

***Protapantelespopularis* (Haliday, 1834)**

*Microgasterpopularis* Haliday, 1834.

**Type information.** Neotype female, NHMUK (examined). Country of type locality: United Kingdom.

**Geographical distribution.**OTL, PAL.

**OTL**: China (JS); **PAL**: Finland, France, Germany, Hungary, Ireland, Mongolia, Netherlands, Romania, Russia (YAR), Slovakia, Turkmenistan, United Kingdom.

***Protapantelespraecipuus* Papp, 1993**

*Protapantelespraecipuus* Papp, 1993.

**Type information.** Holotype female, HNHM (not examined but original description checked). Country of type locality: Italy.

**Geographical distribution.**PAL.

**PAL**: Italy.

***Protapantelesquerceus* (Tobias, 1986)**

*Apantelesquerceus* Tobias, 1986.

**Type information.** Holotype female, ZIN (not examined but original description checked). Country of type locality: Ukraine.

**Geographical distribution.**PAL.

**PAL**: Russia (S), Ukraine.

***Protapantelessantolinae* Oltra, 1995**

*Protapantelessantolinae* Oltra, 1995.

**Type information.** Holotype female, UVS (not examined but original description checked). Country of type locality: Spain.

**Geographical distribution.**PAL.

**PAL**: Spain.

***Protapantelestriangulator* (Wesmael, 1837)**

*Microgastertriangulator* Wesmael, 1837.

**Type information.** Syntypes female and male, RBINS (not examined but subsequent treatment of the species checked). Country of type locality: Belgium.

**Geographical distribution.**PAL.

**PAL**: Belgium, Czech Republic, France, Germany, Hungary, Ireland, Italy, Poland, Romania, Russia (YAR), Serbia, Slovakia, Sweden, Ukraine, United Kingdom.

**Notes.** We follow Broad et al. (2016) for the generic placement of this species.

***Protapantelesyunnanensis* (You & Xiong, 1987)**

*Apantelesyunnanensis* You & Xiong, 1987.

**Type information.** Holotype female, HUNAU (not examined but subsequent treatment of the species checked). Country of type locality: China.

**Geographical distribution.**OTL, PAL.

**OTL**: China (YN); **PAL**: Korea, Russia (PRI).

**Notes.** Our species concept is based on Chen and Song (2004) and Kotenko (2007a). The species distribution in Korea is based on Belokobylskij et al. (2019).

#### Genus Protomicroplitis Ashmead, 1898

***Protomicroplitis*** Ashmead, 1898: 167. Gender: masculine. Type species: *Microgastermediatus* Cresson, 1865, by subsequent designation and monotypy (Viereck 1914: 124).

A recent review (Fernandez-Triana 2015) restricted the genus to three species in the New World. We have seen at least one other undescribed species in collections. The only known host records are from the family Noctuidae. There are six DNA-barcode compliant sequences of this genus in BOLD, representing three BINs.

***Protomicroplitiscalliptera* (Say, 1836)**

*Microgastercalliptera* Say, 1836.

*Microgastermaculipennis* Cresson, 1872.

**Type information.** Type lost (not examined but subsequent treatment of the species checked). Country of type locality: USA.

**Geographical distribution.**NEA, NEO.

**NEA**: Canada (ON), USA (AL, AR, CO, FL, GA, IN, IA, KS, LA, MD, MS, NE, NJ, NY, NC, SC, SD, TN, TX); **NEO**: Mexico.

**Notes.** Our species concept is based on Fernandez-Triana (2015).

***Protomicroplitiscentroamericanus* Fernandez-Triana 2015**

*Protomicroplitiscentroamericanus*Fernandez-Triana 2015.

**Type information.** Holotype female, CNC (examined). Country of type locality: Mexico.

**Geographical distribution.**NEO.

**NEO**: Costa Rica, Mexico.

***Protomicroplitismediatus* (Cresson, 1865)**

*Microgastermediatus* Cresson, 1865.

**Type information.** Holotype male, ANSP (not examined but subsequent treatment of the species checked). Country of type locality: Cuba.

**Geographical distribution.**NEA, NEO.

**NEA**: USA (FL); **NEO**: Bahamas, Cuba.

**Notes.** Our species concept is based on Fernandez-Triana (2015). The specimens recorded from Mexico in the older literature (Muesebeck 1958a, Nixon 1965) actually belong to *P. calliptera* (Fernandez-Triana 2015). We found two males from the Bahamas (San Salvador island, 12-14.vi.1978, coll. N. Elliot) in the USNM collection, which we record in this paper because they represent the first Microgastrinae ever recorded from that country. [In the USNM there are also specimens from the Florida Keys and Miami (USA, FL) and several localities in Cuba, all of them representing new records of the species, but those details will be published elsewhere.]

#### Genus Pseudapanteles Ashmead, 1898

***Pseudapanteles*** Ashmead, 1898: 166. Gender: masculine. Type species: *Pseudapantelesannulicornis* Ashmead, 1900, by subsequent designation (Viereck 1911b: 177).

This genus is widely distributed in the New World, with most of the species found in the Neotropics and just a few extending north into the Nearctic Region. A recent paper provided a key to all 36 known species (Fernandez-Triana et al. 2014a), but we have seen dozens of undescribed species in collections, and the genus is likely to surpass one hundred species. Six Lepidoptera families have been recorded as hosts. There are 676 DNA-barcode compliant sequences of this genus in BOLD, representing 55 BINs.

***Pseudapantelesabantidas* (Nixon, 1965)**

*Apantelesabantidas* Nixon, 1965.

**Type information.** Holotype female, NHMUK (examined). Country of type locality: Brazil.

**Geographical distribution.**NEO.

**NEO**: Brazil (SC).

***Pseudapantelesalfiopivai* Fernandez-Triana & Whitfield, 2014**

*Pseudapantelesalfiopivai* Fernandez-Triana & Whitfield, 2014.

**Type information.** Holotype female, CNC (examined). Country of type locality: Costa Rica.

**Geographical distribution.**NEO.

**NEO**: Costa Rica.

***Pseudapantelesalvaroumanai* Fernandez-Triana & Whitfield, 2014**

*Pseudapantelesalvaroumanai* Fernandez-Triana & Whitfield, 2014.

**Type information.** Holotype female, CNC (examined). Country of type locality: Costa Rica.

**Geographical distribution.**NEO.

**NEO**: Costa Rica.

***Pseudapantelesanalorenaguevarae* Fernandez-Triana & Whitfield, 2014**

*Pseudapantelesanalorenaguevarae* Fernandez-Triana & Whitfield, 2014.

**Type information.** Holotype female, CNC (examined). Country of type locality: Costa Rica.

**Geographical distribution.**NEO.

**NEO**: Costa Rica.

***Pseudapantelesannulicornis* Ashmead, 1900, lectotype designation**

*Pseudapantelesannulicornis* Ashmead, 1900.

**Type information.** Lectotype female, NHMUK (examined). Country of type locality: Saint Vincent.

**Geographical distribution.**NEO.

**NEO**: Panama, Saint Vincent.

**Notes.**Fernandez-Triana et al. (2014a: 19) mentioned that they had examined the female holotype. That is incorrect, as the original description was based on four specimens, female and male (Ashmead 1900c: 292). Thus, Fernandez-Triana et al. (2014a) only examined a female syntype from the original type series, but because that specimen was fully illustrated (Fernandez-Triana et al. 2014: 48, figs 24–31), is in good condition, and perfectly matches the original description we are here designating it as the lectotype. It has the code 3c.1077.

***Pseudapantelesbrunneus* Ashmead, 1900**

*Pseudapantelesbrunneus* Ashmead, 1900.

**Type information.** Holotype male, NHMUK (examined). Country of type locality: Saint Vincent.

**Geographical distribution.**NEO.

**NEO**: Saint Vincent.

***Pseudapantelescarlosespinachi* Fernandez-Triana & Whitfield, 2014**

*Pseudapantelescarlosespinachi* Fernandez-Triana & Whitfield, 2014.

**Type information.** Holotype male, CNC (examined). Country of type locality: Costa Rica.

**Geographical distribution.**NEO.

**NEO**: Costa Rica.

***Pseudapantelescarlosrodriguezi* Fernandez-Triana & Whitfield, 2014**

*Pseudapantelescarlosrodriguezi* Fernandez-Triana & Whitfield, 2014.

**Type information.** Holotype female, CNC (examined). Country of type locality: Costa Rica.

**Geographical distribution.**NEO.

**NEO**: Costa Rica.

***Pseudapanteleschristianafigueresae* Fernandez-Triana & Whitfield, 2014**

*Pseudapanteleschristianafigueresae* Fernandez-Triana & Whitfield, 2014.

**Type information.** Holotype female, CNC (examined). Country of type locality: Costa Rica.

**Geographical distribution.**NEO.

**NEO**: Costa Rica.

***Pseudapantelesdignus* (Muesebeck, 1938)**

*Apantelesdignus* Muesebeck, 1938.

**Type information.** Holotype female, USNM (examined). Country of type locality: USA.

**Geographical distribution.**AUS, NEA, NEO.

**AUS**: Hawaiian Islands; **NEA**: USA (CA, FL); **NEO**: Argentina, Bermuda, Cuba, Mexico, Puerto Rico, US Virgin Islands.

***Pseudapantelesgouleti* Fernandez-Triana, 2010**

*Pseudapantelesgouleti* Fernandez-Triana, 2010.

**Type information.** Holotype female, CNC (examined). Country of type locality: Canada.

**Geographical distribution.**NEA.

**NEA**: Canada (ON, QC).

***Pseudapanteleshernanbravoi* Fernandez-Triana & Whitfield, 2014**

*Pseudapanteleshernanbravoi* Fernandez-Triana & Whitfield, 2014.

**Type information.** Holotype female, CNC (examined). Country of type locality: Costa Rica.

**Geographical distribution.**NEO.

**NEO**: Costa Rica.

***Pseudapantelesjorgerodriguezi* Fernandez-Triana & Whitfield, 2014**

*Pseudapantelesjorgerodriguezi* Fernandez-Triana & Whitfield, 2014.

**Type information.** Holotype female, CNC (examined). Country of type locality: Costa Rica.

**Geographical distribution.**NEO.

**NEO**: Costa Rica.

***Pseudapantelesjosefigueresi* Fernandez-Triana & Whitfield, 2014**

*Pseudapantelesjosefigueresi* Fernandez-Triana & Whitfield, 2014.

**Type information.** Holotype female, CNC (examined). Country of type locality: Costa Rica.

**Geographical distribution.**NEO.

**NEO**: Costa Rica.

***Pseudapanteleslaurachinchillae* Fernandez-Triana & Whitfield, 2014**

*Pseudapanteleslaurachinchillae* Fernandez-Triana & Whitfield, 2014.

**Type information.** Holotype female, CNC (examined). Country of type locality: Costa Rica.

**Geographical distribution.**NEO.

**NEO**: Costa Rica.

***Pseudapanteleslipomeringis* (Muesebeck, 1958)**

*Apanteleslipomeringis* Muesebeck, 1958.

**Type information.** Holotype female, USNM (examined). Country of type locality: Panama.

**Geographical distribution.**NEO.

**NEO**: Panama.

***Pseudapantelesluisguillermosolisi* Fernandez-Triana & Whitfield, 2014**

*Pseudapantelesluisguillermosolisi* Fernandez-Triana & Whitfield, 2014.

**Type information.** Holotype female, CNC (examined). Country of type locality: Costa Rica.

**Geographical distribution.**NEO.

**NEO**: Costa Rica.

***Pseudapantelesmargaritapenonae* Fernandez-Triana & Whitfield, 2014**

*Pseudapantelesmargaritapenonae* Fernandez-Triana & Whitfield, 2014.

**Type information.** Holotype female, CNC (examined). Country of type locality: Costa Rica.

**Geographical distribution.**NEO.

**NEO**: Costa Rica.

***Pseudapantelesmariobozai* Fernandez-Triana & Whitfield, 2014**

*Pseudapantelesmariobozai* Fernandez-Triana & Whitfield, 2014.

**Type information.** Holotype male, CNC (examined). Country of type locality: Costa Rica.

**Geographical distribution.**NEO.

**NEO**: Costa Rica.

***Pseudapantelesmariocarvajali* Fernandez-Triana & Whitfield, 2014**

*Pseudapantelesmariocarvajali* Fernandez-Triana & Whitfield, 2014.

**Type information.** Holotype female, CNC (examined). Country of type locality: Costa Rica.

**Geographical distribution.**NEO.

**NEO**: Costa Rica.

***Pseudapantelesmaureenballesteroae* Fernandez-Triana & Whitfield, 2014**

*Pseudapantelesmaureenballesteroae* Fernandez-Triana & Whitfield, 2014.

**Type information.** Holotype male, CNC (examined). Country of type locality: Costa Rica.

**Geographical distribution.**NEO.

**NEO**: Costa Rica.

***Pseudapantelesmoerens* (Nixon, 1965)**

*Apantelesmoerens* Nixon, 1965.

**Type information.** Holotype female, NHMUK (examined). Country of type locality: Brazil.

**Geographical distribution.**NEO.

**NEO**: Brazil (SC).

***Pseudapantelesmunifigueresae* Fernandez-Triana & Whitfield, 2014**

*Pseudapantelesmunifigueresae* Fernandez-Triana & Whitfield, 2014.

**Type information.** Holotype female, CNC (examined). Country of type locality: Costa Rica.

**Geographical distribution.**NEO.

**NEO**: Costa Rica.

***Pseudapantelesnerion* (Nixon, 1965)**

*Apantelesnerion* Nixon, 1965.

**Type information.** Holotype female, NHMUK (examined). Country of type locality: Brazil.

**Geographical distribution.**NEO.

**NEO**: Brazil (SC).

***Pseudapantelesnigrovariatus* (Muesebeck, 1921)**

*Apantelesnigrovariatus* Muesebeck, 1921.

**Type information.** Holotype female, USNM (examined). Country of type locality: USA.

**Geographical distribution.**NEA.

**NEA**: USA (GA, PA).

**Notes.** The specimen photographed by Fernandez-Triana et al. (2014a) was not the holotype. We examined the actual holotype in 2016, and it has dark orange metanotum and propodeum, unlike the specimen studied for the 2014 paper, which had those areas black. This slightly modifies the species concept and key presented by Fernandez-Triana et al. (2014a) but, other than that, the holotype looks mostly similar.

***Pseudapantelesoscarariasi* Fernandez-Triana & Whitfield, 2014**

*Pseudapantelesoscarariasi* Fernandez-Triana & Whitfield, 2014.

**Type information.** Holotype female, CNC (examined). Country of type locality: Costa Rica.

**Geographical distribution.**NEO.

**NEO**: Costa Rica.

***Pseudapantelesottonsolisi* Fernandez-Triana & Whitfield, 2014**

*Pseudapantelesottonsolisi* Fernandez-Triana & Whitfield, 2014.

**Type information.** Holotype female, CNC (examined). Country of type locality: Costa Rica.

**Geographical distribution.**NEO.

**NEO**: Costa Rica.

***Pseudapantelespedroleoni* Fernandez-Triana & Whitfield, 2014**

*Pseudapantelespedroleoni* Fernandez-Triana & Whitfield, 2014.

**Type information.** Holotype female, CNC (examined). Country of type locality: Costa Rica.

**Geographical distribution.**NEO.

**NEO**: Costa Rica.

***Pseudapantelesraulsolorzanoi* Fernandez-Triana & Whitfield, 2014**

*Pseudapantelesraulsolorzanoi* Fernandez-Triana & Whitfield, 2014.

**Type information.** Holotype female, CNC (examined). Country of type locality: Costa Rica.

**Geographical distribution.**NEO.

**NEO**: Costa Rica.

***Pseudapantelesrenecastroi* Fernandez-Triana & Whitfield, 2014**

*Pseudapantelesrenecastroi* Fernandez-Triana & Whitfield, 2014.

**Type information.** Holotype female, CNC (examined). Country of type locality: Costa Rica.

**Geographical distribution.**NEO.

**NEO**: Costa Rica.

***Pseudapantelesrodrigogamezi* Fernandez-Triana & Whitfield, 2014**

*Pseudapantelesrodrigogamezi* Fernandez-Triana & Whitfield, 2014.

**Type information.** Holotype female, CNC (examined). Country of type locality: Costa Rica.

**Geographical distribution.**NEO.

**NEO**: Costa Rica.

***Pseudapantelesrosemarykarpinskiae* Fernandez-Triana & Whitfield, 2014**

*Pseudapantelesrosemarykarpinskiae* Fernandez-Triana & Whitfield, 2014.

**Type information.** Holotype female, CNC (examined). Country of type locality: Costa Rica.

**Geographical distribution.**NEO.

**NEO**: Costa Rica.

***Pseudapantelesruficollis* (Cameron, 1911)**

*Xanthomicrogasterruficollis* Cameron, 1911.

**Type information.** Lectotype female, NHMUK (examined). Country of type locality: Guyana.

**Geographical distribution.**NEO.

**NEO**: Costa Rica, Cuba, Guyana.

***Pseudapantelessesiae* (Viereck, 1912)**

*Apantelessesiae* Viereck, 1912.

**Type information.** Holotype female, USNM (examined). Country of type locality: USA.

**Geographical distribution.**NEA.

**NEA**: Canada (ON), USA (DC, FL, IN, NJ, TX, VA).

***Pseudapantelessoniapicadoae* Fernandez-Triana & Whitfield, 2014**

*Pseudapantelessoniapicadoae* Fernandez-Triana & Whitfield, 2014.

**Type information.** Holotype female, CNC (examined). Country of type locality: Costa Rica.

**Geographical distribution.**NEO.

**NEO**: Costa Rica.

***Pseudapantelesteofilodelatorrei* Fernandez-Triana & Whitfield, 2014**

*Pseudapantelesteofilodelatorrei* Fernandez-Triana & Whitfield, 2014.

**Type information.** Holotype female, CNC (examined). Country of type locality: Costa Rica.

**Geographical distribution.**NEO.

**NEO**: Costa Rica.

#### Genus Pseudofornicia van Achterberg, 2015

***Pseudofornicia*** van Achterberg, 2015: 91. Gender: feminine. Type species: *Pseudofornicianigrisoma* van Achterberg & Long, 2015, by original designation.

The four described species, from the Oriental and Australasian regions, were recently revised (van Achterberg et al. 2015), but we have seen at least one additional, undescribed, species in collections. No host data are currently available for this genus. There are no DNA barcodes of *Pseudofornicia* in BOLD.

***Pseudoforniciacommoni* (Austin & Dangerfield, 1992)**

*Forniciacommoni* Austin & Dangerfield, 1992.

**Type information.** Holotype female, ANIC (not examined but subsequent treatment of the species checked). Country of type locality: Australia.

**Geographical distribution.**AUS.

**AUS**: Australia (QLD).

**Notes.** Our species concept is based on van Achterberg et al. (2015).

***Pseudoforniciaflavoabdominis* (He & Chen, 1994)**

*Forniciaflavoabdominis* He & Chen, 1994.

**Type information.** Holotype female, ZJUH (not examined but subsequent treatment of the species checked). Country of type locality: China.

**Geographical distribution.**OTL.

**OTL**: China (ZJ).

**Notes.** Our species concept is based on van Achterberg et al. (2015).

***Pseudofornicianigrisoma* van Achterberg & Long, 2015**

*Pseudofornicianigrisoma* van Achterberg & Long, 2015.

**Type information.** Holotype female, IEBR (not examined but original description checked). Country of type locality: Vietnam.

**Geographical distribution.**OTL.

**OTL**: Vietnam.

***Pseudoforniciavanachterbergi* Long, 2015**

*Pseudoforniciavanachterbergi* Long, 2015.

*Forniciaachterbergi* Long, 2007 [primary homonym of *Forniciaachterbergi* Yang & Chen, 2006].

**Type information.** Holotype female, IEBR (not examined but subsequent treatment of the species checked). Country of type locality: Vietnam.

**Geographical distribution.**OTL.

**OTL**: Vietnam.

**Notes.** Our species concept is based on van Achterberg et al. (2015).

#### Genus Pseudovenanides Xiao & You, 2002

***Pseudovenanides*** Xiao & You, 2002: 616. Gender: masculine. Type species: *Pseudovenanideshunanus* Xiao & You, 2002, by original designation and monotypy.

Apart from the single known species, we have seen a few more in collections, from the Oriental and Palearctic regions. The described species was reared from Gelechiidae. There are no DNA barcodes of *Pseudovenanides* in BOLD.

***Pseudovenanideshunanus* Xiao & You, 2002**

*Pseudovenanideshunanus* Xiao & You, 2002.

**Type information.** Holotype female, HUNAU (not examined but original description checked). Country of type locality: China.

**Geographical distribution.**OTL.

**OTL**: China (GX, HN).

#### Genus Qrocodiledundee Fernandez-Triana, 2018

***Qrocodiledundee*** Fernandez-Triana, 2018: 101. Gender: neuter. Type species: *Qrocodiledundeeoutbackense* Fernandez-Triana & Boudreault, 2018, by original designation.

Only known from a single described species in the Australasian region. No host data are currently available for this genus. There are no DNA barcodes of *Qrocodiledundee* in BOLD.

***Qrocodiledundeeoutbackense* Fernandez-Triana & Boudreault, 2018**

*Qrocodiledundeeoutbackense* Fernandez-Triana & Boudreault, 2018.

**Type information.** Holotype male, CNC (examined). Country of type locality: Australia.

**Geographical distribution.**AUS.

**AUS**: Australia (QLD).

#### Genus Rasivalva Mason, 1981

***Rasivalva*** Mason, 1981: 91. Gender: feminine. Type species: *Microplitisstigmaticus* Muesebeck, 1922, by original designation.

Twelve species are recognized here, but many undescribed ones remain in collections. The genus is essentially Holarctic, occasionally reaching the Afrotropical and Oriental regions. The only known host records are all from Geometridae, but future studies may change that. There are 68 DNA-barcode compliant sequences of this genus in BOLD, representing 12 BINs, although several species and BINs are currently misidentified in BOLD as *Diolcogaster* and may actually represent *Rasivalva*.

***Rasivalvacalceata* (Haliday, 1834)**

*Microgastercalceatus* Haliday, 1834.

*Microgasterpubescens* Ratzeburg, 1844.

**Type information.** Type lost (not examined but subsequent treatment of the species checked). Country of type locality: Ireland.

**Geographical distribution.**PAL.

**PAL**: Germany, Hungary, Ireland, Italy, Netherlands, Poland, Romania, Russia (C), Slovakia, Sweden, Switzerland, United Kingdom.

**Notes.** Our species concept is based on Nixon (1965), Tobias (1986), Oltra-Moscardó & Jiménez-Peydró (2005), and Kotenko (2007a).

***Rasivalvacircumvecta* (Lyle, 1918)**

*Diolcogastercircumvectus* Lyle, 1918.

**Type information.** Lectotype female, NHMUK (examined). Country of type locality: United Kingdom.

**Geographical distribution.**PAL.

**PAL**: Finland, Russia (C), United Kingdom.

**Notes.** The species was originally described based on four specimens (mounted on individual cards), which are all deposited in the NHMUK, and share the same type number 3c.31. There is a fifth pin with a label that has written: “Type to be selected from the above 4 specimens”. That fifth label was presumably added by Nixon, because when he dealt with that species he mentioned “Type in British Museum” (Nixon 1965: 256). In fact, one of the female specimens (the specimen occupying the top left corner in the unit tray at the NHMUK) has a Holotype round label added. Article 74.5 of the ICZN “Lectotype designations before 2000” stipulates that: “the term lectotype, or an exact translation or equivalent expression (e.g., the type), must have been used”, and also states that “a subsequent use of the term holotype does not constitute a valid lectotype designation unless the author, when wrongly using that term, explicitly indicated that he or she was selecting from the type series that particular specimen to serve as the name-bearing type.” Both situations clearly apply to Nixon (1965), as he referred to a type which he also unambiguously selected among the available specimens (even adding to that specimen an extra label marked as holotype). Thus, Nixon’s designation is to be considered valid, although that specimen should be considered as the lectotype and not the holotype, and the remaining three specimens are paralectotypes.

***Rasivalvadesueta* Papp, 1989**

*Rasivalvadesueta* Papp, 1989.

**Type information.** Holotype female, HNHM (examined). Country of type locality: Hungary.

**Geographical distribution.**PAL.

**PAL**: Switzerland.

***Rasivalvakaradagi* Tobias, 1986**

*Rasivalvakaradagi* Tobias, 1986.

**Type information.** Holotype female, ZIN (not examined but subsequent treatment of the species checked). Country of type locality: Russia.

**Geographical distribution.**PAL.

**PAL**: Russia (NC), Ukraine.

**Notes.** Our species concept is based on Oltra-Moscardó & Jiménez-Peydró (2005).

***Rasivalvaleleji* Kotenko, 2007**

*Rasivalvaleleji* Kotenko, 2007.

**Type information.** Holotype female, SIZK (not examined but original description checked). Country of type locality: Ukraine.

**Geographical distribution.**PAL.

**PAL**: Russia (SAK).

***Rasivalvalepelleyi* (Wilkinson, 1934)**

*Microgasterlepelleyi* Wilkinson, 1934.

**Type information.** Holotype female, NHMUK (examined). Country of type locality: Kenya.

**Geographical distribution.**AFR.

**AFR**: Kenya.

***Rasivalvalongivena* Song & Chen, 2004**

*Rasivalvalongivena* Song & Chen, 2004.

**Type information.** Holotype female, FAFU (not examined but original description checked). Country of type locality: China.

**Geographical distribution.**PAL.

**PAL**: China (HB).

***Rasivalvamarginata* (Nees, 1834)**

*Microgastermarginatus* Nees, 1834.

**Type information.** Holotype female, depository unknown (not examined but subsequent treatment of the species checked). Country of type locality: Germany.

**Geographical distribution.**OTL, PAL.

**OTL**: Philippines; **PAL**: Austria, Finland, Germany, Hungary, Poland, Russia (KR, PRI, RYA, SPE, YAR), Slovenia, Sweden, Switzerland, United Kingdom, Yugoslavia.

**Notes.** Our species concept is based on Oltra-Moscardó & Jiménez-Peydró (2005).

***Rasivalvaperplexa* (Muesebeck, 1922)**

*Microplitisperplexus* Muesebeck, 1922.

**Type information.** Holotype female, USNM (not examined but original description checked). Country of type locality: USA.

**Geographical distribution.**NEA.

**NEA**: Canada (BC, NB, ON), USA (IN, MI).

***Rasivalvapyrenaica* Oltra & Jiménez, 2005**

*Rasivalvapyrenaica* Oltra & Jiménez, 2005.

**Type information.** Holotype female, UVS (not examined but original description checked). Country of type locality: Andorra.

**Geographical distribution.**PAL.

**PAL**: Andorra.

***Rasivalvarugosa* (Muesebeck, 1922)**

*Microplitisrugosus* Muesebeck, 1922.

*Microplitiscoloradensis* Muesebeck, 1922.

**Type information.** Holotype male, MCZC (not examined but original description checked). Country of type locality: USA.

**Geographical distribution.**NEA.

**NEA**: Canada (ON, QC), USA (CO, MI, MN, NJ).

***Rasivalvastigmatica* (Muesebeck, 1922)**

*Microplitisstigmaticus* Muesebeck, 1922.

*Microplitismuesebecki* Marsh, 1974 [replacement name for *Microplitisstigmaticus* Muesebeck, 1922].

**Type information.** Holotype female, MCZC (not examined but original description checked). Country of type locality: USA.

**Geographical distribution.**NEA.

**NEA**: Canada (AB, BC, QC), USA (CA, CO, ID, NH, WA).

#### Genus Rhygoplitis Mason, 1981

***Rhygoplitis*** Mason, 1981: 81. Gender: masculine. Type species: Apanteles (Pseudapanteles) terminalis Gahan, 1912, by original designation.

This genus is distributed in the New World, with four described species but several more remain in collections undescribed, mostly from the Neotropical region. Known hosts are mostly Crambidae, but more studies are needed. There are 294 DNA-barcode compliant sequences of this genus in BOLD, representing 13 BINs.

***Rhygoplitisaciculatus* (Ashmead, 1900)**

*Urogasteraciculatus* Ashmead, 1900.

*Pseudapantelessancti-vincenti* Ashmead, 1900.

*Apantelesthoracicus* Muesebeck, 1921.

**Type information.** Holotype male, NHMUK (examined). Country of type locality: Grenada.

**Geographical distribution.**NEA, NEO.

**NEA**: USA (KS, TX); **NEO**: Costa Rica, Grenada, Panama, Saint Vincent.

**Notes.** We have examined the female type of *P. sancti-vincenti* and indeed it is the same species as the male type of *U.aciculatus*. Thus, although the name bearer for this species is the male specimen (following the rules of the ICZN), the female specimen of *P. sancti-vincenti* should be considered as useful, if not more useful, in any further study of the genus, as most of Microgastrinae taxonomy is based on female specimens.

***Rhygoplitischoreuti* (Viereck, 1912)**

*Apanteleschoreuti* Viereck, 1912.

**Type information.** Holotype female, USNM (not examined but subsequent treatment of the species checked). Country of type locality: USA.

**Geographical distribution.**NEA.

**NEA**: USA (FL, IA, NJ, SC, TX, VA).

**Notes.** Our species concept is based on Mason (1981) and Whitfield (1995a).

***Rhygoplitissanctivincenti* (Ashmead, 1900)**

*Apantelessanctivincenti* Ashmead, 1900.

**Type information.** Type lost (not examined but subsequent treatment of the species checked). Country of type locality: Saint Vincent.

**Geographical distribution.**NEO.

**NEO**: Saint Vincent.

**Notes.** Our species concept is based on Fernandez-Triana et al. (2014e).

***Rhygoplitisterminalis* (Gahan, 1912)**

*Apantelesterminalis* Gahan, 1912.

**Type information.** Holotype male, USNM (examined). Country of type locality: USA.

**Geographical distribution.**NEA.

**NEA**: USA (AR, CO, FL, GA, IL, IA, KY, MD, NY, TX).

#### Genus Sathon Mason, 1981

***Sathon*** Mason, 1981: 78. Gender: masculine. Type species: *Apantelesneomexicanus* Muesebeck, 1921, by original designation.

This genus is distributed in all biogeographical regions, with 23 described species, although is not highly species rich anywhere. We have seen additional species in collections, but it is difficult to estimate the actual diversity due to some species being similar to other genera (e.g., *Glyptapanteles* and *Lathrapanteles*). Williams’ (1988) revision is the most up to date and comprehensive work for this genus but is now outdated. Five families of Lepidoptera have been reported as hosts, but in most cases those records need further verification. There are 266 DNA-barcode compliant sequences of *Sathon* in BOLD, representing 27 BINs.

***Sathonaggeris* Williams, 1988**

*Sathonaggeris* Williams, 1988.

**Type information.** Holotype female, CNC (examined). Country of type locality: Ecuador.

**Geographical distribution.**NEO.

**NEO**: Ecuador.

***Sathonalbicoxus* Austin & Dangerfield, 1992**

*Sathonalbicoxus* Austin & Dangerfield, 1992.

**Type information.** Holotype female, ANIC (not examined but original description checked). Country of type locality: Australia.

**Geographical distribution.**AUS.

**AUS**: Australia (NSW, TAS, VIC).

***Sathonbekilyensis* (Granger, 1949), new combination**

*Microgasterbekilyensis* Granger, 1949.

**Type information.** Holotype female, MNHN (not examined but illustrations of the holotype examined). Country of type locality: Madagascar.

**Geographical distribution.**AFR.

**AFR**: Madagascar.

**Notes.** The generic placement of this species has been determined based on information from the original description and low-resolution images of the holotype (taken with a cell phone) which we have examined. The species is clearly not *Microgaster*, and we are transferring it to *Sathon* here based on propodeum with a median longitudinal carina, T2 subtriangular or trapezoidal and with lateral margins well defined by sulcus, ovipositor sheaths moderately long (almost half metatibia length), and hypopygium inflexible. The type also has antenna with some central flagellomeres white-yellow, and the body is mostly yellow. This species seems to be related to *Microgasterrufotestacea* Granger, also transferred in this paper to *Sathon*. We have seen in collections several undescribed species from Africa which share similar morphological features to these two species, and the best generic placement at present would be in *Sathon*. However, future study of this group of species may change that, and we suspect that they may represent an undescribed genus.

***Sathonbelippae* (Rohwer, 1919)**

*Apantelesbelippae* Rohwer, 1919.

**Type information.** Holotype female, USNM (examined). Country of type locality: Indonesia.

**Geographical distribution.**AUS, OTL.

**AUS**: Fiji; **OTL**: India, Indonesia.

***Sathoncinctiformis* (Viereck, 1911)**

*Apantelescinctiformis* Viereck, 1911.

**Type information.** Holotype female, USNM (examined). Country of type locality: USA.

**Geographical distribution.**NEA.

**NEA**: Canada (ON, QC), USA (IN, IA, MD, MI, NJ, NY, NC, OH, PA, RI, VT, VA, WI).

***Sathoncircumflexus* Williams, 1988**

*Sathoncircumflexus* Williams, 1988.

**Type information.** Holotype female, CNC (examined). Country of type locality: USA.

**Geographical distribution.**NEA.

**NEA**: USA (AZ, CO, NM).

***Sathoneugeni* (Papp, 1972)**

*Apanteleseugeni* Papp, 1972.

*Apantelesmagnicoxis* Jakimavicius, 1972.

**Type information.** Holotype female, HNHM (examined). Country of type locality: Hungary.

**Geographical distribution.**PAL.

**PAL**: Austria, Bulgaria, Finland, Germany, Hungary, Italy, Latvia, Lithuania, Netherlands, Russia (C, NW), Slovakia, Sweden, Switzerland, Turkey, United Kingdom.

**Notes.** Our species concept is based on Williams (1988), which considered it part of the *lateralis* group (see more comments about this species group in the Notes provided under *S.lateralis*). The species distribution in Russia is based on Belokobylskij et al. (2019).

***Sathonfalcatus* (Nees, 1834)**

*Microgasterfalcatus* Nees, 1834.

*Microgasterequestris* Haliday, 1834.

*Apantelesgladiator* Szépligeti, 1901.

*Apantelespriapus* Gautier & Cleu, 1927.

**Type information.** Neotype female, ZMHB (not examined but authoritatively identified specimens examined). Country of type locality: unknown.

**Geographical distribution.**OTL, PAL.

**OTL**: Indonesia; **PAL**: Afghanistan, Austria, Armenia, Azerbaijan, Belarus, Bosnia and Herzegovina, China, Croatia, Czech Republic, Denmark, Egypt, Estonia, Finland, France, Georgia, Germany, Hungary, Ireland, Italy, Japan, Kazakhstan, Kyrgyzstan, Korea, Latvia, Lithuania, Luxembourg, Macedonia, Mongolia, Montenegro, Netherlands, Poland, Romania, Russia (ZAB, DA, IRK, KAM, KR, KIR, KYA, SAK, SPE, VLG, YAR), Serbia, Slovenia, Spain, Sweden, Switzerland, Tajikistan, Turkey, United Kingdom, Uzbekistan.

**Notes.** We examined the type of *M.equestris* (Haliday) which is in the NHMUK, as well as numerous authenticated specimens in the CNC, MZH and RSME. *Apantelespriapus* Gautier & Cleu was considered by Telenga (1955: 54) to be a valid species, not a synonym of *falcatus*, based on differences in body size and sculpture, as well as ovipositor size; however, Telenga did not examine specimens of the *priapus* type series (from France), his species concept was only based on the original description. Yu et al. (2016), following Telenga, also considered *priapus* to be a valid species. However, Wilkinson (1945: 133–137) actually examined two cotypes of the *priapus* series and considered them to be the same species than *falcatus*; Wilkinson also designated a neotype for *falcatus* and was able to study a large number of specimens from different localities and collections. Thus, we consider Wilkinson (1945) a more accurate account and here we accept his decision to synonymize *priapus* under *falcatus*, which has also been accepted and followed by most authors (e.g., Shenefelt 1972, Papp 1982, 1988, Williams 1998, Kotenko 2007). The species distribution in Afghanistan, Armenia, Azerbaijan, China, Georgia, Kyrgyzstan, and Tajikistan is based on Belokobylskij et al. (2019).

***Sathonfausta* (Nixon, 1973)**

*Apantelesfausta* Nixon, 1973.

**Type information.** Holotype female, NHMUK (examined). Country of type locality: United Kingdom.

**Geographical distribution.**PAL.

**PAL**: Austria, Finland, Germany, Slovakia, Sweden, Switzerland, United Kingdom.

**Notes.** This species was placed in *Sathon* by Mason (1981) when he described the genus. The only comprehensive revision ever done of *Sathon* (Williams 1988) also treated *fausta* within the genus (although as a synonym of *S.eugeni* Papp, 1972). Since then, *fausta* has been variously treated as *Protapanteles* (van Achterberg 2003), *Glyptapanteles* (Papp 1988, Belokobylskij et al. 2003, Shaw 2012, Broad et al. 2016), or *Sathon* (Capek and Hofmann 1997). Here we follow Williams (1988) study and consider at present the best generic placement for *fausta* to be in *Sathon* (as part of *lateralis* group of species, see more comments about this species group in the Notes provided under *S.lateralis*). The status of *fausta* as a valid species has varied during the years (e.g., Papp 1983a, 1998), and we suspect it is only a synonym of *eugeni* (e.g., Shaw 2012, Broad et al. 2016), but because that will require further investigation, for the time being we retain it as a valid species in this paper.

***Sathonflavofacialis* (Granger, 1949), new combination**

*Microgasterflavofacialis* Granger, 1949.

**Type information.** Syntypes female, MNHN (not examined but illustrations of the holotype examined). Country of type locality: Madagascar.

**Geographical distribution.**AFR.

**AFR**: Madagascar.

**Notes.** The generic placement of this species has been determined based on information from the original description and low-resolution images of the holotype (taken with a cell phone) which we have examined. The species is clearly not *Microgaster*, and we are transferring it to *Sathon* here based on propodeum with median longitudinal carina, T2 subtriangular or trapezoidal and with lateral margins well defined by sulcus, ovipositor sheaths moderately long (almost half metatibia length), and hypopygium inflexible. The type also has antenna with some central flagellomeres white-yellow, and the head, propleuron, most of legs, and sternites are orange-yellow. We have seen in collections several undescribed species from Africa which share similar morphological features to this species, and the best generic placement at present would be in *Sathon*. However, future study of this group of species may change that and we suspect that they may represent an undescribed genus.

***Sathonlaevidorsum* Williams, 1988**

*Sathonlaevidorsum* Williams, 1988.

**Type information.** Holotype female, CNC (examined). Country of type locality: Mexico.

**Geographical distribution.**NEO.

**NEO**: Mexico.

***Sathonlateralis* (Haliday, 1834)**

*Microgasterlateralis* Haliday, 1834.

**Type information.** Lectotype female, NHMUK (examined). Country of type locality: unknown.

**Geographical distribution.**PAL.

**PAL**: Armenia, Azerbaijan, Belgium, Finland, France, Georgia, Germany, Hungary, Ireland, Italy, Kazakhstan, Lithuania, Madeira Islands, Moldova, Netherlands, Romania, Russia (C, NC, S), Serbia, Slovakia, Spain, Sweden, Switzerland, Turkey, Ukraine, United Kingdom.

**Notes.** This species was placed in *Sathon* by Mason (1981) when he described the genus. The only comprehensive revision ever done of the genus (Williams 1988) also considered *lateralis* to be in *Sathon*, as part of a newly created *lateralis* group of species, which also comprises three other species: *eugeni* Papp, 1972, *fausta* (Nixon, 1973), and *papilionae* Williams, 1988. These four species were differentiated from the more derived *falcatus* group, which comprises the rest of *Sathon* (*sensu*Williams 1988), based on the propodeum sculpture, shape and sculpture of T2, hypopygium shape, straight ovipositor sheaths, males without enlarged genitalia and host data. The *lateralis* group resembles *Glyptapanteles* with relatively long ovipositor sheaths (longer than the majority of the described species in that genus), which has likely influenced the decision of many subsequent authors to treat some of the species in the *lateralis* group as *Glyptapanteles* (e.g., Papp 1988, Belokobylskij et al. 2003, Shaw 2012, Broad et al. 2016) or *Protapantelessensu lato* (van Achterberg 2003); although other authors continued to treat those species as *Sathon* (Oltra and Michelena 1988, Maetô 1996, Capek and Hofmann 1997). Until a more robust phylogenetic framework for Microgastrinae is available, we prefer to maintain the *lateralis* group in *Sathon*, as we consider Williams (1988) the most detailed study currently available. It should also be noted that in large neighbour-joining trees with thousands of Microgastrinae DNA barcodes (e.g., Smith et al. 2013), the described species of *Sathon*, from both *lateralis* and *falcatus* groups, cluster together.

***Sathonlaurae* (de Saeger, 1944), new combination**

*Microgasterlaurae* de Saeger, 1944.

**Type information.** Holotype female, RMCA (not examined but original description checked). Country of type locality: Democratic Republic of Congo.

**Geographical distribution.**AFR.

**AFR**: Democratic Republic of Congo, South Africa.

**Notes.** Based on the original description (de Saeger 1944), the best generic placement at present is in *Sathon*, based on the propodeum with a strong median carina, ovipositor and ovipositor sheaths relatively long, and hypopygium supposedly unpleated. We consider the hypopygium of this species as lacking pleats because it is depicted as such in figure 22 of the paper (de Saeger 1944: 62), although that detail was not mentioned in the written part of the description. However, we deem this a fair assumption because in the same paper, illustrations of other species with pleated hypopygium were clearly drawn as such, and often also explicitly mentioned in the written part of the descriptions.

***Sathonmasoni* Williams, 1988**

*Sathonmasoni* Williams, 1988.

**Type information.** Holotype female, CNC (examined). Country of type locality: USA.

**Geographical distribution.**NEA.

**NEA**: Canada (NT, NU), USA (AK, ID, MN).

***Sathonmikeno* (de Saeger, 1944), new combination**

*Microgastermikeno* de Saeger, 1944.

**Type information.** Holotype female, RMCA (not examined but original description checked). Country of type locality: Democratic Republic of Congo.

**Geographical distribution.**AFR.

**AFR**: Democratic Republic of Congo.

**Notes.** Based on the original description, this species is clearly not *Microgaster*. The best generic placement at present would be in *Sathon*, as many of the characters described would correspond to that genus, e.g., ovipositor and ovipositor sheaths relatively long (as long as metatibia), inflexible hypopygium, and propodeum with strong median carina. A few characters differ from previously described species of *Sathon*: a) the male specimens from the type series studied by de Saeger (1944) were not described as having large external genitalia, one of the most distinctive traits of *Sathon*, although that trait is not always present (see Williams 1988 for a discussion of that character); b) the head (described by de Saeger as more transverse and globose than normal, and with face rugose and prominent) is not like in typical species of *Sathon*, but is similar to some species of several genera (e.g., *Cotesia*, *Diolcogaster*, *Keylimepie*, *Venanides*), where it seems to be related to some specialized activity; c) T2, as illustrated in de Saeger (1944: fig. 78) is not as in typical *Sathon*, in the sense of being more transverse. However, we do not consider these differences as sufficient to invalidate our decision to transfer *mikeno* to *Sathon*.

***Sathonmorata* (Wilkinson, 1929)**

*Microgastermorata* Wilkinson, 1929.

**Type information.** Holotype female, NHMUK (examined). Country of type locality: Australia.

**Geographical distribution.**AUS.

**AUS**: Australia (ACT, SA, VIC, WA).

***Sathonnaryciae* Austin & Dangerfield, 1992**

*Sathonnaryciae* Austin & Dangerfield, 1992.

**Type information.** Holotype female, ANIC (not examined but original description checked). Country of type locality: Australia.

**Geographical distribution.**AUS.

**AUS**: Australia (VIC).

***Sathonneomexicanus* (Muesebeck, 1921)**

*Apantelesneomexicanus* Muesebeck, 1921.

*Apantelescaudatus* Muesebeck, 1922.

**Type information.** Holotype female, USNM (examined). Country of type locality: USA.

**Geographical distribution.**NEA.

**NEA**: Canada (AB, BC, MB, NL, NT, ON, PE), USA (AK, AZ, CA, CO, ID, MI, MN, MT, NV, NM, OR, SD, UT, WA, WI, WY).

***Sathonoreo* Fagan-Jeffries & Austin, 2019**

*Sathonoreo* Fagan-Jeffries & Austin, 2019.

**Type information.** Holotype female, SAMA (not examined but original description checked). Country of type locality: Australia.

**Geographical distribution.**AUS.

**AUS**: Australia (SA).

***Sathonpapilionae* Williams, 1988**

*Sathonpapilionae* Williams, 1988.

**Type information.** Holotype female, CNC (examined). Country of type locality: Canada.

**Geographical distribution.**NEA.

**NEA**: Canada (BC), USA (AK).

**Notes.** Our species concept is based on Williams (1988), which considered it part of the *lateralis* group (see more comments about this species group in the Notes provided under *S.lateralis*).

***Sathonresplendens* (Wilkinson, 1929)**

*Microgasterresplendens* Wilkinson, 1929.

**Type information.** Holotype female, NHMUK (examined). Country of type locality: Australia.

**Geographical distribution.**AUS.

**AUS**: Australia (TAS).

**Notes.** The holotype is in relatively poor condition, broken into several pieces all glued together, with the consequence that some morphological details are lost.

***Sathonruandanus* (de Saeger, 1944), new combination**

*Microgasterruandana* de Saeger, 1944.

**Type information.** Holotype female, RMCA (not examined but original description checked). Country of type locality: Democratic Republic of Congo.

**Geographical distribution.**AFR.

**AFR**: Rwanda.

**Notes.** Based on the original description, the best generic placement at present would be in *Sathon*, as many of the characters described would correspond to that genus, e.g., ovipositor and ovipositor sheaths relatively long (as long as metatibia), inflexible hypopygium, and propodeum with median carina.

***Sathonrufotestaceus* (Granger, 1949), new combination**

*Microgasterrufotestacea* Granger, 1949.

**Type information.** Holotype female, MNHN (not examined but original description checked). Country of type locality: Madagascar.

**Geographical distribution.**AFR.

**AFR**: Madagascar.

**Notes.** The generic placement of this species has been determined based on information from the original description. The species is clearly not *Microgaster*, and we are transferring it to *Sathon* based on the propodeum being mostly smooth but with a strong median longitudinal carina, T1 and T2 shapes and sculptures (as detailed in Granger 1949: 225, fig. 218), and ovipositor sheaths moderately long (as long as metafemur). This species seems to be related to *Microgasterbekilyensis* Granger, also transferred to *Sathon* in this paper. We have seen in collections several undescribed species from Africa which share similar morphological features to these two species, and the best generic placement at present would be in *Sathon*. However, future study of this group of species may change that; we suspect that they may represent an undescribed genus.

#### Genus Semionis Nixon, 1965

***Semionis*** Nixon, 1965: 206. Gender: masculine. Type species: *Semionisrarus* Nixon, 1965, by original designation and monotypy.

There is only one extant species (Nixon 1965, Mason 1981), which is very distinctive, with a fossil species also recently described (Belokobylskij 2014). No host data are currently available for this genus. There are no DNA barcodes of *Semionis* in BOLD.

***Semionisrarus* Nixon, 1965**

*Semionisrarus* Nixon, 1965.

**Type information.** Holotype male, NHMUK (examined). Country of type locality: South Africa.

**Geographical distribution.**AFR.

**AFR**: South Africa.

#### Genus Sendaphne Nixon, 1965

***Sendaphne*** Nixon, 1965: 203. Gender: feminine. Type species: *Sendaphneolearus* Nixon, 1965, by original designation.

This is a strictly Neotropical genus, recently revised (Fernandez-Triana et al. 2014h) with eleven species recorded, but also several additional, undescribed species in collections. No host data are currently available for *Sendaphne*. There are seven DNA-barcode compliant sequences of this genus in BOLD (with 24 additional, shorter sequences ranging from 102 to 420 bp.), representing two BINs.

***Sendaphneanitae* Fernandez-Triana & Whitfield, 2014**

*Sendaphneanitae* Fernandez-Triana & Whitfield, 2014.

**Type information.** Holotype female, CNC (examined). Country of type locality: Ecuador.

**Geographical distribution.**NEO.

**NEO**: Ecuador.

***Sendaphnebennetti* Fernandez-Triana & Whitfield, 2014**

*Sendaphnebennetti* Fernandez-Triana & Whitfield, 2014.

**Type information.** Holotype male, CNC (examined). Country of type locality: Mexico.

**Geographical distribution.**NEO.

**NEO**: Mexico.

***Sendaphnebrasilianus* Penteado-Dias, 1995**

*Sendaphnebrasilianus* Penteado-Dias, 1995.

**Type information.** Holotype female, DCBU (not examined but subsequent treatment of the species checked). Country of type locality: Brazil.

**Geographical distribution.**NEO.

**NEO**: Brazil (DF).

**Notes.** Our species concept is based on Fernandez-Triana et al. (2014h).

***Sendaphnebroadi* Fernandez-Triana & Whitfield, 2014**

*Sendaphnebroadi* Fernandez-Triana & Whitfield, 2014.

**Type information.** Holotype male, CNC (examined). Country of type locality: Ecuador.

**Geographical distribution.**NEO.

**NEO**: Ecuador.

***Sendaphnedianariaspennae* Fernandez-Triana & Whitfield, 2014**

*Sendaphnedianariaspennae* Fernandez-Triana & Whitfield, 2014.

**Type information.** Holotype female, CNC (examined). Country of type locality: Brazil.

**Geographical distribution.**NEO.

**NEO**: Brazil (PE, RJ, SC), Colombia.

***Sendaphnejatai* Penteado-Dias, 1995**

*Sendaphnejatai* Penteado-Dias, 1995.

**Type information.** Holotype female, DCBU (not examined but subsequent treatment of the species checked). Country of type locality: Brazil.

**Geographical distribution.**NEO.

**NEO**: Brazil (MT, SP), Ecuador, French Guiana.

**Notes.** Our species concept is based on Fernandez-Triana et al. (2014h).

***Sendaphneolearus* Nixon, 1965**

*Sendaphneolearus* Nixon, 1965.

**Type information.** Holotype female, NHMUK (examined). Country of type locality: Brazil.

**Geographical distribution.**NEO.

**NEO**: Brazil (SC), French Guiana, Peru.

***Sendaphneparanaensis* Scatolini & Penteado-Dias, 1999**

*Sendaphneparanaensis* Scatolini & Penteado-Dias, 1999.

**Type information.** Holotype female, DCMP (not examined but subsequent treatment of the species checked). Country of type locality: Brazil.

**Geographical distribution.**NEO.

**NEO**: Brazil (ES, PR, RJ), Paraguay.

**Notes.** Our species concept is based on Fernandez-Triana et al. (2014h).

***Sendaphnepenteadodiasae* Fernandez-Triana & Whitfield, 2014**

*Sendaphnepenteadodiasae* Fernandez-Triana & Whitfield, 2014.

**Type information.** Holotype female, CNC (examined). Country of type locality: Brazil.

**Geographical distribution.**NEO.

**NEO**: Brazil (PR).

***Sendaphnerogerblancoi* Fernandez-Triana & Whitfield, 2014**

*Sendaphnerogerblancoi* Fernandez-Triana & Whitfield, 2014.

**Type information.** Holotype female, CNC (examined). Country of type locality: Costa Rica.

**Geographical distribution.**NEO.

**NEO**: Costa Rica.

***Sendaphnesulmo* Nixon, 1965**

*Sendaphnesulmo* Nixon, 1965.

**Type information.** Holotype female, NHMUK (examined). Country of type locality: Mexico.

**Geographical distribution.**NEO.

**NEO**: Mexico.

#### Genus Shireplitis Fernandez-Triana & Ward, 2013

***Shireplitis*** Fernandez-Triana & Ward, 2013: 556. Gender: masculine. Type species: *Shireplitisbilboi* Fernandez-Triana & Ward, 2013, by original designation.

The genus was recently described, to include six species limited to New Zealand (Fernandez-Triana et al. 2013b). We are not aware of any undescribed species in collections. No host data are currently available for *Shireplitis*. There are three DNA-barcode compliant sequences of this genus in BOLD, representing one BIN.

***Shireplitisbilboi* Fernandez-Triana & Ward, 2013**

*Shireplitisbilboi* Fernandez-Triana & Ward, 2013.

**Type information.** Holotype female, NZAC (examined). Country of type locality: New Zealand.

**Geographical distribution.**AUS.

**AUS**: New Zealand.

***Shireplitisfrodoi* Fernandez-Triana & Ward, 2013**

*Shireplitisfrodoi* Fernandez-Triana & Ward, 2013.

**Type information.** Holotype female, NZAC (examined). Country of type locality: New Zealand.

**Geographical distribution.**AUS.

**AUS**: New Zealand.

***Shireplitismeriadoci* Fernandez-Triana & Ward, 2013**

*Shireplitismeriadoci* Fernandez-Triana & Ward, 2013.

**Type information.** Holotype female, NZAC (examined). Country of type locality: New Zealand.

**Geographical distribution.**AUS.

**AUS**: New Zealand.

***Shireplitisperegrini* Fernandez-Triana & Ward, 2013**

*Shireplitisperegrini* Fernandez-Triana & Ward, 2013.

**Type information.** Holotype female, NZAC (examined). Country of type locality: New Zealand.

**Geographical distribution.**AUS.

**AUS**: New Zealand.

***Shireplitissamwisei* Fernandez-Triana & Ward, 2013**

*Shireplitissamwisei* Fernandez-Triana & Ward, 2013.

**Type information.** Holotype female, NZAC (examined). Country of type locality: New Zealand.

**Geographical distribution.**AUS.

**AUS**: New Zealand.

***Shireplitistolkieni* Fernandez-Triana & Ward, 2013**

*Shireplitistolkieni* Fernandez-Triana & Ward, 2013.

**Type information.** Holotype female, NZAC (examined). Country of type locality: New Zealand.

**Geographical distribution.**AUS.

**AUS**: New Zealand.

#### Genus Silvaspinosus Fernandez-Triana, 2018

***Silvaspinosus*** Fernandez-Triana, 2018: 102. Gender: neuter. Type species: *Silvaspinosusvespa*Fernandez-Triana and Boudreault 2018, by original designation.

A single species from the Afrotropical region was recently described, but in collections there is at least one additional species (Fernandez-Triana & Boudreault, 2018). No host data are currently available for this genus. There are no full DNA barcodes of *Silvaspinosus* in BOLD, but two short sequences.

***Silvaspinosusvespa* Fernandez-Triana & Boudreault, 2018**

*Silvaspinosusvespa* Fernandez-Triana & Boudreault, 2018.

**Type information.** Holotype female, CAS (examined). Country of type locality: Madagascar.

**Geographical distribution.**AFR.

**AFR**: Madagascar.

#### Genus Snellenius Westwood, 1882

*Snellenius* Westwood, 1882: 19. Gender: masculine. Type species: *Snelleniusvollenhovii* Westwood, 1882, by original designation and monotypy.

The 41 described species of this genus are distributed in all regions except the Nearctic (although that might be due to relatively little collecting effort and fewer studies of Microgastrinae in southwestern North America, where a few species may be found). Although some revisions are available (e.g., Austin and Dangerfield 1993, Long & van Achterberg 2013, Fernandez-Triana et al. 2015b, Perez and Berta 2017) there are many undescribed species in collections and the genus is far from being completely understood from a taxonomic perspective. All known host records are from three Lepidoptera families (Erebidae, Noctuidae, Sphingidae). There are 185 DNA-barcode compliant sequences of this genus in BOLD, representing 25 BINs.

***Snelleniusatratus* Shenefelt, 1968**

*Snelleniusatratus* Shenefelt, 1968.

**Type information.** Holotype female, depository unknown (not examined but original description checked). Country of type locality: Peru.

**Geographical distribution.**NEO.

**NEO**: Peru.

**Notes.** The original description (Shenefelt 1968) mentions the holotype as deposited in the collection of the author. We do not know where that collection is stored at present but suspect it might be in the USNM.

***Snelleniusbasalis* (Walker, 1874)**

*Proteropsbasalis* Walker, 1874.

**Type information.** Holotype female, NHMUK (examined). Country of type locality: Japan.

**Geographical distribution.**PAL.

**PAL**: Japan.

**Notes.** Van Achterbeg & de Chenon (2009) transferred the species to *Snellenius*, after the authors were able to examine the holotype; at that time the authors included a synonym within that species, *Snelleniustheretrae* (Watanabe, 1937). Subsequently, Long & van Achterberg (2013) revised this and removed *S.theretrae* from synonymy, to be considered as a valid species, a decision we follow here.

***Snelleniusbicolor* Shenefelt, 1968**

*Snelleniusbicolor* Shenefelt, 1968.

**Type information.** Holotype female, depository unknown (not examined but original description checked). Country of type locality: Peru.

**Geographical distribution.**NEO.

**NEO**: Argentina, Bolivia, Peru.

**Notes.** The original description (Shenefelt 1968) mentions the holotype as deposited in the collection of the author. We do not know where that collection is stored at present but suspect it might be in the USNM.

***Snelleniusbillburgeri* Fernandez-Triana & Whitfield, 2015**

*Snelleniusbillburgeri* Fernandez-Triana & Whitfield, 2015.

**Type information.** Holotype male, CNC (examined). Country of type locality: Costa Rica.

**Geographical distribution.**NEO.

**NEO**: Costa Rica.

***Snelleniusbobdressleri* Fernandez-Triana & Whitfield, 2015**

*Snelleniusbobdressleri* Fernandez-Triana & Whitfield, 2015.

**Type information.** Holotype female, USNM (examined). Country of type locality: Costa Rica.

**Geographical distribution.**NEO.

**NEO**: Costa Rica.

***Snelleniusclavitergum* Austin & Dangerfield, 1993**

*Snelleniusclavitergum* Austin & Dangerfield, 1993.

**Type information.** Holotype female, AEIC (not examined but original description checked). Country of type locality: Papua New Guinea.

**Geographical distribution.**AUS.

**AUS**: Papua New Guinea.

***Snelleniusdonstonei* Fernandez-Triana & Whitfield, 2015**

*Snelleniusdonstonei* Fernandez-Triana & Whitfield, 2015.

**Type information.** Holotype female, USNM (examined). Country of type locality: Costa Rica.

**Geographical distribution.**NEO.

**NEO**: Costa Rica.

***Snelleniusfelipechavarriai* Fernandez-Triana & Whitfield, 2015**

*Snelleniusfelipechavarriai* Fernandez-Triana & Whitfield, 2015.

**Type information.** Holotype female, CNC (examined). Country of type locality: Costa Rica.

**Geographical distribution.**NEO.

**NEO**: Costa Rica.

***Snelleniusgelleus* Nixon, 1965**

*Snelleniusgelleus* Nixon, 1965.

**Type information.** Holotype female, NHMUK (examined). Country of type locality: China.

**Geographical distribution.**OTL.

**OTL**: China (FJ).

***Snelleniusgerardoherrerai* Fernandez-Triana & Whitfield, 2015**

*Snelleniusgerardoherrerai* Fernandez-Triana & Whitfield, 2015.

**Type information.** Holotype female, USNM (examined). Country of type locality: Costa Rica.

**Geographical distribution.**NEO.

**NEO**: Costa Rica.

***Snelleniusguizhouensis* Luo & You, 2005**

*Snelleniusguizhouensis* Luo & You, 2005.

**Type information.** Holotype female, GUGC (not examined but subsequent treatment of the species checked). Country of type locality: China.

**Geographical distribution.**OTL.

**OTL**: China (GZ).

**Notes.** Our species concept is based on Long and van Achterberg (2013).

***Snelleniushippotionus* Austin & Dangerfield, 1993**

*Snelleniushippotionus* Austin & Dangerfield, 1993.

**Type information.** Holotype male, ANIC (not examined but original description checked). Country of type locality: Papua New Guinea.

**Geographical distribution.**AUS.

**AUS**: Papua New Guinea.

***Snelleniusirenebakerae* Fernandez-Triana & Whitfield, 2015**

*Snelleniusirenebakerae* Fernandez-Triana & Whitfield, 2015.

**Type information.** Holotype female, USNM (examined). Country of type locality: Costa Rica.

**Geographical distribution.**NEO.

**NEO**: Costa Rica.

***Snelleniusisidrochaconi* Fernandez-Triana & Whitfield, 2015**

*Snelleniusisidrochaconi* Fernandez-Triana & Whitfield, 2015.

**Type information.** Holotype male, CNC (examined). Country of type locality: Costa Rica.

**Geographical distribution.**NEO.

**NEO**: Costa Rica, Panama.

***Snelleniusjohnkressi* Fernandez-Triana & Whitfield, 2015**

*Snelleniusjohnkressi* Fernandez-Triana & Whitfield, 2015.

**Type information.** Holotype female, USNM (examined). Country of type locality: Costa Rica.

**Geographical distribution.**NEO.

**NEO**: Costa Rica.

***Snelleniusjorgecampabadali* Fernandez-Triana & Whitfield, 2015**

*Snelleniusjorgecampabadali* Fernandez-Triana & Whitfield, 2015.

**Type information.** Holotype female, INBio (examined). Country of type locality: Costa Rica.

**Geographical distribution.**NEO.

**NEO**: Costa Rica.

***Snelleniusjorgegomezlauritoi* Fernandez-Triana & Whitfield, 2015**

*Snelleniusjorgegomezlauritoi* Fernandez-Triana & Whitfield, 2015.

**Type information.** Holotype female, CNC (examined). Country of type locality: Costa Rica.

**Geographical distribution.**NEO.

**NEO**: Costa Rica.

***Snelleniusjosesarukhani* Fernandez-Triana & Whitfield, 2015**

*Snelleniusjosesarukhani* Fernandez-Triana & Whitfield, 2015.

**Type information.** Holotype male, CNC (examined). Country of type locality: Costa Rica.

**Geographical distribution.**NEO.

**NEO**: Costa Rica.

***Snelleniuskerrydresslerae* Fernandez-Triana & Whitfield, 2015**

*Snelleniuskerrydresslerae* Fernandez-Triana & Whitfield, 2015.

**Type information.** Holotype female, USNM (examined). Country of type locality: Costa Rica.

**Geographical distribution.**NEO.

**NEO**: Costa Rica.

***Snelleniuslatigenus* Luo & You, 2005**

*Snelleniuslatigenus* Luo & You, 2005.

**Type information.** Holotype female, GUGC (not examined but subsequent treatment of the species checked). Country of type locality: China.

**Geographical distribution.**OTL.

**OTL**: China (GZ).

**Notes.** Our species concept is based on Long and van Achterberg (2013).

***Snelleniuslucindamcdadeae* Fernandez-Triana & Whitfield, 2015**

*Snelleniuslucindamcdadeae* Fernandez-Triana & Whitfield, 2015.

**Type information.** Holotype female, USNM (examined). Country of type locality: Costa Rica.

**Geographical distribution.**NEO.

**NEO**: Costa Rica.

***Snelleniusluisdiegogomezi* Fernandez-Triana & Whitfield, 2015**

*Snelleniusluisdiegogomezi* Fernandez-Triana & Whitfield, 2015.

**Type information.** Holotype female, CNC (examined). Country of type locality: Costa Rica.

**Geographical distribution.**NEO.

**NEO**: Costa Rica, Panama.

***Snelleniusmaculipennis* (Szépligeti, 1900)**

*Microplitismaculipennis* Szépligeti, 1900.

*Microplitiseusirus* Lyle, 1921.

*Microplitisophiusae* Ramakrishna Ayyar, 1921.

**Type information.** Type lost (not examined but authoritatively identified specimens examined). Country of type locality: Papua New Guinea.

**Geographical distribution.**AUS, OTL.

**AUS**: Australia (QLD), Papua New Guinea; **OTL**: India, Thailand, Vietnam.

**Notes.** The female holotype is considered to be lost (see details in Austin and Dangerfield 1993: 1156). Although several authors have placed this species in *Snellenius*, Gupta (2013a) transferred the species to *Microplitis*, despite the illustrations of her paper clearly showing the presence of an epicnemial carina in that species, which would place it within *Snellenius*. Ranjith et al. (2015a) also followed Gupta (2013a). We have examined the type of *Microplitiseusirus* Lyle, and it also has an epicnemial carina, in addition to having the scutellar disc strongly impressed, as is typical of species of *Snellenius*. Thus, we revise the species combination here back to *Snellenius*.

***Snelleniusmariakuzminae* Fernandez-Triana & Whitfield, 2015**

*Snelleniusmariakuzminae* Fernandez-Triana & Whitfield, 2015.

**Type information.** Holotype male, CNC (examined). Country of type locality: Costa Rica.

**Geographical distribution.**NEO.

**NEO**: Costa Rica.

***Snelleniusmariamartachavarriae* Fernandez-Triana & Whitfield, 2015**

*Snelleniusmariamartachavarriae* Fernandez-Triana & Whitfield, 2015.

**Type information.** Holotype male, CNC (examined). Country of type locality: Costa Rica.

**Geographical distribution.**NEO.

**NEO**: Costa Rica.

***Snelleniusnigellus* Long & van Achterberg, 2013**

*Snelleniusnigellus* Long & van Achterberg, 2013.

**Type information.** Holotype male, VNMN (not examined but original description checked). Country of type locality: Vietnam.

**Geographical distribution.**OTL.

**OTL**: Vietnam.

***Snelleniusperuensis* Shenefelt, 1968**

*Snelleniusperuensis* Shenefelt, 1968.

**Type information.** Holotype female, depository unknown (not examined but original description checked). Country of type locality: Peru.

**Geographical distribution.**NEO.

**NEO**: Peru.

**Notes.** The original description (Shenefelt 1968) mentions the holotype as deposited in the collection of the author. We do not know where that collection is stored at present, but suspect it might be in the USNM.

***Snelleniusphildevriesi* Fernandez-Triana & Whitfield, 2015**

*Snelleniusphildevriesi* Fernandez-Triana & Whitfield, 2015.

**Type information.** Holotype female, USNM (examined). Country of type locality: Costa Rica.

**Geographical distribution.**NEO.

**NEO**: Costa Rica.

***Snelleniusphilippinensis* (Ashmead, 1904)**

*Microplitisphilippinensis* Ashmead, 1904.

*Microplitisbimaculatus* Cameron, 1909.

**Type information.** Holotype male, USNM (not examined but subsequent treatment of the species checked). Country of type locality: Philippines.

**Geographical distribution.**OTL.

**OTL**: Indonesia, Malaysia, Philippines, Vietnam.

**Notes.** Our species concept is based on Long and van Achterberg (2013).

***Snelleniusquiricojimenezi* Fernandez-Triana & Whitfield, 2015**

*Snelleniusquiricojimenezi* Fernandez-Triana & Whitfield, 2015.

**Type information.** Holotype female, CNC (examined). Country of type locality: Costa Rica.

**Geographical distribution.**NEO.

**NEO**: Costa Rica.

***Snelleniusradicalis* (Wilkinson, 1929)**

*Microplitisradicalis* Wilkinson, 1929.

**Type information.** Syntypes female and male, ZMHB (not examined but subsequent treatment of the species checked). Country of type locality: China.

**Geographical distribution.**OTL.

**OTL**: China (TW).

**Notes.** The information about type specimens and depository we follow here is from the original description (Wilkinson 1945: 206-207); however, Nixon (1965: 270) mentioned that the type was deposited in the NHMUK.

***Snelleniusrobertoespinozai* Fernandez-Triana & Whitfield, 2015**

*Snelleniusrobertoespinozai* Fernandez-Triana & Whitfield, 2015.

**Type information.** Holotype male, CNC (examined). Country of type locality: Costa Rica.

**Geographical distribution.**NEO.

**NEO**: Costa Rica.

***Snelleniussandyknappae* Fernandez-Triana & Whitfield, 2015**

*Snelleniussandyknappae* Fernandez-Triana & Whitfield, 2015.

**Type information.** Holotype female, USNM (examined). Country of type locality: Costa Rica.

**Geographical distribution.**NEO.

**NEO**: Costa Rica.

***Snelleniussedlaceki* Austin & Dangerfield, 1993**

*Snelleniussedlaceki* Austin & Dangerfield, 1993.

**Type information.** Holotype female, AEIC (not examined but original description checked). Country of type locality: Papua New Guinea.

**Geographical distribution.**AUS.

**AUS**: Papua New Guinea.

***Snelleniussimilis* Long & van Achterberg, 2013**

*Snelleniussimilis* Long & van Achterberg, 2013.

**Type information.** Holotype female, VNMN (not examined but original description checked). Country of type locality: Vietnam.

**Geographical distribution.**OTL.

**OTL**: Vietnam.

***Snelleniustheretrae* (Watanabe, 1937)**

*Microplitistheretrae* Watanabe, 1937.

**Type information.** Holotype female, EIHU (not examined but subsequent treatment of the species checked). Country of type locality: Japan.

**Geographical distribution.**PAL.

**PAL**: Japan, Korea.

**Notes.** The status of this species was revised by Long & van Achterberg (2013), who removed the species from synonymy with *Snelleniusbasalis* (Walker, 1874), a decision we accept and follow.

***Snelleniustricolor* Shenefelt, 1968**

*Snelleniustricolor* Shenefelt, 1968.

**Type information.** Holotype female, USNM (examined). Country of type locality: Argentina.

**Geographical distribution.**NEO.

**NEO**: Argentina.

***Snelleniusvelvaruddae* Fernandez-Triana & Whitfield, 2015**

*Snelleniusvelvaruddae* Fernandez-Triana & Whitfield, 2015.

**Type information.** Holotype female, CNC (examined). Country of type locality: Costa Rica.

**Geographical distribution.**NEO.

**NEO**: Costa Rica.

***Snelleniusvickifunkae* Fernandez-Triana & Whitfield, 2015**

*Snelleniusvickifunkae* Fernandez-Triana & Whitfield, 2015.

**Type information.** Holotype female, USNM (examined). Country of type locality: Costa Rica.

**Geographical distribution.**NEO.

**NEO**: Costa Rica.

***Snelleniusvollenhovii* Westwood, 1882**

*Snelleniusvollenhovii* Westwood, 1882.

**Type information.** Holotype male, OUMNH (not examined but subsequent treatment of the species checked). Country of type locality: Papua New Guinea.

**Geographical distribution.**AUS.

**AUS**: Papua New Guinea.

**Notes.** Our species concept is based on Nixon (1965), Austin and Dangerfield (1993), and Long and van Achterberg (2013).

***Snelleniuswarrenwagneri* Fernandez-Triana & Whitfield, 2015**

*Snelleniuswarrenwagneri* Fernandez-Triana & Whitfield, 2015.

**Type information.** Holotype female, USNM (examined). Country of type locality: Costa Rica.

**Geographical distribution.**NEO.

**NEO**: Costa Rica.

#### Genus Tobleronius Fernandez-Triana, 2018

***Tobleronius*** Fernandez-Triana, 2018: 108. Gender: neuter. Type species: *Tobleroniusorientalis*Fernandez-Triana and Boudreault 2018, by original designation.

One species was recently described from the Oriental region (Fernandez-Triana & Boudreault, 2018), but at least another one may exist in collections. No host data are currently available for this genus. There are two DNA-barcode compliant sequences of *Tobleronius* in BOLD, representing two BINs (although those sequences have not been identified in BOLD as belonging to *Tobleronius*; see Fernandez-Triana and Boudreault 2018).

***Tobleroniusorientalis* Fernandez-Triana & Boudreault, 2018**

*Tobleroniusorientalis* Fernandez-Triana & Boudreault, 2018.

**Type information.** Holotype male, RMNH (examined). Country of type locality: Vietnam.

**Geographical distribution.**OTL.

**OTL**: Thailand, Vietnam.

#### Genus Ungunicus Fernandez-Triana, 2018

***Ungunicus*** Fernandez-Triana, 2018: 113. Gender: neuter. Type species: *Ungunicusvietnamensis* Fernandez-Triana & Boudreault, 2018, by original designation.

One species was recently described from the Oriental region (Fernandez-Triana and Boudreault 2018); we are not aware of additional species in collections. No host data are currently available for this genus. There is one DNA-barcode compliant sequence of *Ungunicus* in BOLD, representing one BIN (although that sequence has not been identified in BOLD as belonging to *Ungunicus*, see Fernandez-Triana and Boudreault 2018 for that).

***Ungunicusvietnamensis* Fernandez-Triana & Boudreault, 2018**

*Ungunicusvietnamensis* Fernandez-Triana & Boudreault, 2018.

**Type information.** Holotype female, RMNH (examined). Country of type locality: Vietnam.

**Geographical distribution.**OTL.

**OTL**: Vietnam.

#### Genus Venanides Mason, 1981

***Venanides*** Mason, 1981: 99. Gender: masculine. Type species: *Venanidesxeste* Mason, 1981, by original designation.

A cosmopolitan genus, with 14 described species and several more undescribed and found in collections. No revision of this genus is currently available. Host records are from six families of Lepidoptera, but some are questionable. There are 89 DNA-barcode compliant sequences of this genus in BOLD, representing five BINs.

***Venanidesastydamia* (Nixon, 1965), new combination**

*Apantelesastydamia* Nixon, 1965.

**Type information.** Holotype female, NHMUK (examined). Country of type locality: South Africa.

**Geographical distribution.**AFR.

**AFR**: South Africa.

**Notes.** Transferred to *Venanides* based on pronotum laterally with a single furrow, venation of fore wing, smooth propodeum, T1 and T2 shapes, and ovipositor sheaths without setae. In the original description, Nixon (1965) did not provide any detail on the etymology of the species name. As first revisers, we thus consider its gender to be neuter.

***Venanidescaspius* Abdoli, Fernandez-Triana & Talebi, 2019**

*Venanidescaspius* Abdoli, Fernandez-Triana & Talebi, 2019.

**Type information.** Holotype female, TMUC (examined). Country of type locality: Iran.

**Geographical distribution.**PAL.

**PAL**: Iran.

***Venanidescongoensis* (de Saeger, 1941)**

*Apantelescongoensis* de Saeger, 1941.

**Type information.** Holotype female, RMCA (not examined but original description checked). Country of type locality: Democratic Republic of Congo.

**Geographical distribution.**AFR.

**AFR**: Cameroon, Democratic Republic of Congo, Uganda.

***Venanidescurticornis* (Granger, 1949)**

*Apantelescurticornis* Granger, 1949.

**Type information.** Holotype female, MNHN (not examined but original description checked). Country of type locality: Madagascar.

**Geographical distribution.**AFR.

**AFR**: Madagascar, Réunion.

***Venanidesdemeter* (Wilkinson, 1934), new combination**

*Apantelesdemeter* Wilkinson, 1934.

**Type information.** Holotype female, NHMUK (examined). Country of type locality: New Zealand.

**Geographical distribution.**AUS.

**AUS**: New Zealand.

**Notes.** This species had been transferred to *Glyptapanteles* by Mason (1981), but it is placed in *Venanides* in this work because the pronotum laterally has only one ventral sulcus (two sulci in *Glyptapanteles*) and the propodeum is mostly sculptured, including numerous, relatively long carinae radiating from nucha (propodeum not like that in *Glyptapanteles*). DNA barcodes obtained from this species suggest that it might even belong to a different genus on its own but solving that will require further study beyond the scope of this paper; for the time being the best generic placement is *Venanides*.

***Venanideslongifrons* Fernandez-Triana & van Achterberg, 2017**

*Venanideslongifrons* Fernandez-Triana & van Achterberg, 2017.

**Type information.** Holotype female, RMNH (examined). Country of type locality: Yemen.

**Geographical distribution.**AFR.

**AFR**: Yemen.

***Venanidesparmula* (Nixon, 1965), new combination**

*Apantelesparmula* Nixon, 1965.

**Type information.** Holotype female, NHMUK (examined). Country of type locality: South Africa.

**Geographical distribution.**AFR.

**AFR**: South Africa.

**Notes.** Transferred to *Venanides* based on pronotum laterally with a single furrow, venation of fore wing, smooth propodeum, T1 and T2 shapes, and ovipositor sheaths without setae.

***Venanidesplancina* (Nixon, 1965)**

*Apantelesplancina* Nixon, 1965.

**Type information.** Holotype female, NHMUK (examined). Country of type locality: India.

**Geographical distribution.**OTL.

**OTL**: China (HN), India.

***Venanidespyrogrammae* (Nixon, 1965)**

*Apantelespyrogrammae* Nixon, 1965.

**Type information.** Holotype female, NHMUK (examined). Country of type locality: Papua New Guinea.

**Geographical distribution.**AUS.

**AUS**: Australia (QLD), Papua New Guinea.

***Venanidessupracompressus* Fernandez-Triana & van Achterberg, 2017**

*Venanidessupracompressus* Fernandez-Triana & van Achterberg, 2017.

**Type information.** Holotype female, RMNH (examined). Country of type locality: Yemen.

**Geographical distribution.**AFR.

**AFR**: Yemen.

***Venanidessymmysta* (Nixon, 1965), new combination**

*Apantelessymmysta* Nixon, 1965.

**Type information.** Holotype female, NHMUK (examined). Country of type locality: South Africa.

**Geographical distribution.**AFR.

**AFR**: South Africa.

**Notes.** Transferred to *Venanides* based on pronotum laterally with a single furrow, venation of fore wing, smooth propodeum, T1 and T2 shapes, and ovipositor sheaths without setae.

***Venanidestenuitergitus* Fernandez-Triana & van Achterberg, 2017**

*Venanidestenuitergitus* Fernandez-Triana & van Achterberg, 2017.

**Type information.** Holotype female, RMNH (examined). Country of type locality: Yemen.

**Geographical distribution.**AFR.

**AFR**: Yemen.

***Venanidesvanharteni* Fernandez-Triana & van Achterberg, 2017**

*Venanidesvanharteni* Fernandez-Triana & van Achterberg, 2017.

**Type information.** Holotype female, RMNH (examined). Country of type locality: Yemen.

**Geographical distribution.**AFR.

**AFR**: Yemen.

***Venanidesxeste* Mason, 1981**

*Venanidesxeste* Mason, 1981.

**Type information.** Holotype female, CNC (examined). Country of type locality: Canada.

**Geographical distribution.**NEA, NEO.

**NEA**: Canada (MB, ON), USA (AZ, AR, CT, IA, MI, MN, MO, NY, NC, TX); **NEO**: Brazil (SC), Saint Lucia.

#### Genus Venanus Mason, 1981

***Venanus*** Mason, 1981: 94. Gender: masculine. Type species: *Venanuspinicola* Mason, 1981, by original designation.

This genus seems to be restricted to the New World, with most of the eleven described species being found in the Neotropical region. A recent revision of *Venanus* (Whitfield et al. 2011) covered most of the known species, but we have seen in collections a few additional ones. Known host records include the families Gelechiidae and Gracillariidae. There are 71 DNA-barcode compliant sequences of this genus in BOLD, representing five BINs.

***Venanuschilensis* Mason, 1981**

*Venanuschilensis* Mason, 1981.

**Type information.** Holotype male, CNC (examined). Country of type locality: Chile.

**Geographical distribution.**NEO.

**NEO**: Chile.

***Venanusgreeneyi* Whitfield & Arias-Penna, 2011**

*Venanusgreeneyi* Whitfield & Arias-Penna, 2011.

**Type information.** Holotype female, USNM (not examined but original description checked). Country of type locality: Ecuador.

**Geographical distribution.**NEO.

**NEO**: Ecuador.

***Venanusheberti* Fernandez-Triana, 2010**

*Venanusheberti* Fernandez-Triana, 2010.

**Type information.** Holotype male, CNC (examined). Country of type locality: Canada.

**Geographical distribution.**NEA.

**NEA**: Canada (NS, PE, QC).

**Notes.**Mason (1981) described the new genus *Venanus*, with the type species being *Venanuspinicola*, a species widespread in Canada, and also Idaho, USA. Mason (1981: 97) reported *V.pinicola* as parasitizing “*Gracillariaasplenifoliella*” (Gracillaridae). Fernandez-Triana (2010) considered *V.pinicola* specimens (*sensu* Mason) to actually comprise two different species, *pinicola* (restricted to western Canada and Idaho) and a new species he described as *Venanusheberti* (from eastern Canada). Some of the *pinicola* specimens (*sensu* Mason) were transferred to *heberti*, and when doing so, Fernandez-Triana (2010) spelled the host name as “*Caloptiliaasplenifoliella*”; and Fernandez-Triana (2014) repeated that same information. The actual name of the host is *Caloptiliaasplenifoliatella* (Darlington, 1949), thus, both Mason (1981) and Fernandez-Triana (2010, 2014) spelled the specific name incorrectly. Summarizing, the correct identity of the known hosts for both species of *Venanus* are: *Coleotechnitesmilleri* (Busck, 1914) and *Coleotechnitesstarki* (Freeman, 1957) (both Gelechiidae) for *Venanuspinicola*; and *Caloptiliaasplenifoliatella* (Darlington, 1949) for *Venanusheberti*.

***Venanushelavai* Mason, 1981**

*Venanushelavai* Mason, 1981.

**Type information.** Holotype male, CNC (examined). Country of type locality: Colombia.

**Geographical distribution.**NEO.

**NEO**: Colombia, Ecuador.

***Venanusjohnnyrosalesi* Fernandez-Triana & Whitfield, 2014**

*Venanusjohnnyrosalesi* Fernandez-Triana & Whitfield, 2014.

**Type information.** Holotype female, CNC (examined). Country of type locality: Costa Rica.

**Geographical distribution.**NEO.

**NEO**: Costa Rica.

***Venanuskusikuyllurae* Rasmussen & Whitfield, 2011**

*Venanuskusikuyllurae* Rasmussen & Whitfield, 2011.

**Type information.** Holotype female, MUSM (not examined but original description checked). Country of type locality: Peru.

**Geographical distribution.**NEO.

**NEO**: Peru.

***Venanusminutalis* (Muesebeck, 1958)**

*Microplitisminutalis* Muesebeck, 1958.

**Type information.** Holotype female, USNM (examined). Country of type locality: Chile.

**Geographical distribution.**NEO.

**NEO**: Chile.

***Venanusperuensis* Mason, 1981**

*Venanusperuensis* Mason, 1981.

**Type information.** Holotype female, CNC (examined). Country of type locality: Peru.

**Geographical distribution.**NEO.

**NEO**: Peru.

***Venanuspinicola* Mason, 1981**

*Venanuspinicola* Mason, 1981.

**Type information.** Holotype female, CNC (examined). Country of type locality: Canada.

**Geographical distribution.**NEA.

**NEA**: Canada (AB, BC, NS, QC, YT), USA (ID).

**Notes.** See notes on *Venanusheberti* above for details on the correct identity of the hosts for these two species of *Venanus*.

***Venanusrandallgarciai* Fernandez-Triana & Whitfield, 2014**

*Venanusrandallgarciai* Fernandez-Triana & Whitfield, 2014.

**Type information.** Holotype female, CNC (examined). Country of type locality: Costa Rica.

**Geographical distribution.**NEO.

**NEO**: Costa Rica.

***Venanusyanayacuensis* Arias-Penna & Whitfield, 2011**

*Venanusyanayacuensis* Arias-Penna & Whitfield, 2011.

**Type information.** Holotype female, USNM (not examined but original description checked). Country of type locality: Ecuador.

**Geographical distribution.**NEO.

**NEO**: Ecuador.

#### Genus Wilkinsonellus Mason, 1981

***Wilkinsonellus*** Mason, 1981: 122. Gender: masculine. Type species: *Apantelesiphitus* Nixon, 1965, by original designation.

Several recent papers on this genus have increased the total of described species to 23, but there are still many undescribed species in collections. The genus seems to be pantropical. A single host record is known, from Crambidae. There are 55 DNA-barcode compliant sequences of this genus in BOLD, representing 14 BINs.

***Wilkinsonellusalexsmithi* Arias-Penna & Whitfield, 2013**

*Wilkinsonellusalexsmithi* Arias-Penna & Whitfield, 2013.

**Type information.** Holotype female, CNC (not examined but original description checked). Country of type locality: Costa Rica.

**Geographical distribution.**NEO.

**NEO**: Costa Rica.

***Wilkinsonellusamplus* Austin & Dangerfield, 1992**

*Wilkinsonellusamplus* Austin & Dangerfield, 1992.

**Type information.** Holotype female, ANIC (not examined but original description checked). Country of type locality: Australia.

**Geographical distribution.**AUS.

**AUS**: Australia (NT, QLD).

***Wilkinsonellusarabicus* van Achterberg & Fernandez-Triana, 2017**

*Wilkinsonellusarabicus* van Achterberg & Fernandez-Triana, 2017.

**Type information.** Holotype female, RMNH (examined). Country of type locality: Yemen.

**Geographical distribution.**AFR.

**AFR**: Yemen.

***Wilkinsonelluscorpustriacolor* Arias-Penna, Zhang & Whitfield, 2014**

*Wilkinsonelluscorpustriacolor* Arias-Penna, Zhang & Whitfield, 2014.

**Type information.** Holotype female, FNIC (not examined but original description checked). Country of type locality: Fiji.

**Geographical distribution.**AUS.

**AUS**: Fiji.

***Wilkinsonellusdaira* (Nixon, 1965)**

*Apantelesdaira* Nixon, 1965.

**Type information.** Holotype male, NHMUK (examined). Country of type locality: Papua New Guinea.

**Geographical distribution.**AUS.

**AUS**: Papua New Guinea.

***Wilkinsonellusfijiensis* Arias-Penna, Zhang & Whitfield, 2014**

*Wilkinsonellusfijiensis* Arias-Penna, Zhang & Whitfield, 2014.

*Wilkinsonellusfijienis* Arias-Penna, Zhang & Whitfield, 2014 [original misspelling].

**Type information.** Holotype female, FNIC (not examined but original description checked). Country of type locality: Fiji.

**Geographical distribution.**AUS.

**AUS**: Fiji.

***Wilkinsonellusflavicrus* Long & van Achterberg, 2011**

*Wilkinsonellusflavicrus* Long & van Achterberg, 2011.

**Type information.** Holotype female, IEBR (not examined but original description checked). Country of type locality: Vietnam.

**Geographical distribution.**OTL.

**OTL**: Vietnam.

***Wilkinsonellusgranulatus* Ahmad, Pandey, Haider & Shujauddin, 2005**

*Wilkinsonellusgranulatus* Ahmad, Pandey, Haider & Shujauddin, 2005.

**Type information.** Holotype female, AMUZ (not examined but original description checked). Country of type locality: India.

**Geographical distribution.**OTL.

**OTL**: India.

***Wilkinsonellushenicopus* (de Saeger, 1944)**

*Apanteleshenicopus* de Saeger, 1944.

**Type information.** Holotype female, RMCA (not examined but original description checked). Country of type locality: Democratic Republic of Congo.

**Geographical distribution.**AFR.

**AFR**: Democratic Republic of Congo, Kenya.

***Wilkinsonellusiphitus* (Nixon, 1965)**

*Apantelesiphitus* Nixon, 1965.

**Type information.** Holotype female, USNM (examined). Country of type locality: Philippines.

**Geographical distribution.**OTL.

**OTL**: China (HI, TW), Philippines.

***Wilkinsonelluskogui* Arias-Penna & Whitfield, 2013**

*Wilkinsonelluskogui* Arias-Penna & Whitfield, 2013.

**Type information.** Holotype male, IAVH (not examined but original description checked). Country of type locality: Colombia.

**Geographical distribution.**NEO.

**NEO**: Colombia.

***Wilkinsonelluslongicentrus* Long & van Achterberg, 2003**

*Wilkinsonelluslongicentrus* Long & van Achterberg, 2003.

**Type information.** Holotype female, IEBR (not examined but original description checked). Country of type locality: Vietnam.

**Geographical distribution.**OTL.

**OTL**: Vietnam.

***Wilkinsonellusmasoni* Long & van Achterberg, 2011**

*Wilkinsonellusmasoni* Long & van Achterberg, 2011.

**Type information.** Holotype female, IEBR (not examined but original description checked). Country of type locality: Vietnam.

**Geographical distribution.**OTL.

**OTL**: Vietnam.

***Wilkinsonellusnarangahus* Rousse & Gupta, 2013**

*Wilkinsonellusnarangahus* Rousse & Gupta, 2013.

**Type information.** Holotype female, MNHN (not examined but original description checked). Country of type locality: Réunion.

**Geographical distribution.**AFR.

**AFR**: Réunion.

***Wilkinsonellusnescalptura* Arias-Penna, Zhang & Whitfield, 2014**

*Wilkinsonellusnescalptura* Arias-Penna, Zhang & Whitfield, 2014.

**Type information.** Holotype female, FNIC (not examined but original description checked). Country of type locality: Fiji.

**Geographical distribution.**AUS.

**AUS**: Fiji.

***Wilkinsonellusnigratus* Long & van Achterberg, 2011**

*Wilkinsonellusnigratus* Long & van Achterberg, 2011.

**Type information.** Holotype male, IEBR (not examined but original description checked). Country of type locality: Vietnam.

**Geographical distribution.**OTL.

**OTL**: Vietnam.

***Wilkinsonellusnigrocentrus* Long & van Achterberg, 2011**

*Wilkinsonellusnigrocentrus* Long & van Achterberg, 2011.

**Type information.** Holotype female, IEBR (not examined but original description checked). Country of type locality: Vietnam.

**Geographical distribution.**OTL.

**OTL**: Vietnam.

***Wilkinsonelluspanamaensis* Arias-Penna & Whitfield, 2013**

*Wilkinsonelluspanamaensis* Arias-Penna & Whitfield, 2013.

**Type information.** Holotype female, CNC (not examined but original description checked). Country of type locality: Panama.

**Geographical distribution.**NEO.

**NEO**: Panama.

***Wilkinsonellusparamplus* Long & van Achterberg, 2003**

*Wilkinsonellusparamplus* Long & van Achterberg, 2003.

**Type information.** Holotype female, IEBR (not examined but original description checked). Country of type locality: Vietnam.

**Geographical distribution.**OTL.

**OTL**: China (GD, GX), Vietnam.

***Wilkinsonellusstriatus* Austin & Dangerfield, 1992**

*Wilkinsonellusstriatus* Austin & Dangerfield, 1992.

**Type information.** Holotype female, ANIC (not examined but original description checked). Country of type locality: Australia.

**Geographical distribution.**AUS.

**AUS**: Australia (QLD), Papua New Guinea.

***Wilkinsonellusthyone* (Nixon, 1965)**

*Apantelesthyone* Nixon, 1965.

**Type information.** Holotype female, USNM (examined). Country of type locality: Philippines.

**Geographical distribution.**OTL.

**OTL**: Philippines.

***Wilkinsonellustobiasi* Long, 2007**

*Wilkinsonellustobiasi* Long, 2007.

**Type information.** Holotype female, IEBR (not examined but original description checked). Country of type locality: Vietnam.

**Geographical distribution.**OTL.

**OTL**: Vietnam.

***Wilkinsonellustomi* Austin & Dangerfield, 1992**

*Wilkinsonellustomi* Austin & Dangerfield, 1992.

**Type information.** Holotype female, AEIC (not examined but original description checked). Country of type locality: Papua New Guinea.

**Geographical distribution.**AUS.

**AUS**: Australia (QLD), Papua New Guinea.

#### Genus Xanthapanteles Whitfield, 1995

***Xanthapanteles*** Whitfield, 1995: 879. Gender: masculine. Type species: *Xanthapantelescameronae* Whitfield, 1995, by original designation and monotypy.

Only one species is known, from the Neotropics (Whitfield 1995b). No host data are currently available for this genus. There are no DNA barcodes of *Xanthapanteles* in BOLD.

***Xanthapantelescameronae* Whitfield, 1995**

*Xanthapantelescameronae* Whitfield, 1995.

**Type information.** Holotype female, MCZC (not examined but original description checked). Country of type locality: Argentina.

**Geographical distribution.**NEO.

**NEO**: Argentina.

#### Genus Xanthomicrogaster Cameron, 1911

***Xanthomicrogaster*** Cameron, 1911: 324. Gender: feminine. Type species: *Xanthomicrogasterfortipes* Cameron, 1911, by subsequent designation (Viereck 1914).

This genus seems to be restricted to the Neotropical region. Apart from the six described species, there are many more undescribed in collections. No host data are currently available for this genus. There are 112 DNA-barcode compliant sequences of this genus in BOLD, representing 23 BINs.

***Xanthomicrogasterfortipes* Cameron, 1911**

*Xanthomicrogasterfortipes* Cameron, 1911.

**Type information.** Holotype female, NHMUK (examined). Country of type locality: Guyana.

**Geographical distribution.**NEO.

**NEO**: Brazil (MG, PA, SP), Guyana, Suriname.

***Xanthomicrogastermaculata* Penteado-Dias, Shimabukuro & van Achterberg, 2002**

*Xanthomicrogastermaculatus* Penteado-Dias, Shimabukuro & van Achterberg, 2002.

**Type information.** Holotype female, DCBU (not examined but original description checked). Country of type locality: Brazil.

**Geographical distribution.**NEO.

**NEO**: Brazil (MG, SP).

**Notes.** The species name must be treated as an adjective and not as a noun (Doug Yanega, pers. comm.) and thus it must match the gender of the genus name.

***Xanthomicrogasterotamendi* Martínez, 2018**

*Xanthomicrogasterotamendi* Martínez, 2018.

**Type information.** Holotype female, MACN (not examined but original description checked). Country of type locality: Argentina.

**Geographical distribution.**NEO.

**NEO**: Argentina.

***Xanthomicrogasterpelides* Nixon, 1965**

*Xanthomicrogasterpelides* Nixon, 1965.

**Type information.** Holotype female, NHMUK (examined). Country of type locality: Brazil.

**Geographical distribution.**NEO.

**NEO**: Brazil (SC).

***Xanthomicrogastersayjuhu* Martínez, 2018**

*Xanthomicrogastersayjuhu* Martínez, 2018.

**Type information.** Holotype female, MACN (not examined but original description checked). Country of type locality: Argentina.

**Geographical distribution.**NEO.

**NEO**: Argentina.

***Xanthomicrogasterseres* Nixon, 1965**

*Xanthomicrogasterseres* Nixon, 1965.

**Type information.** Holotype male, NHMUK (examined). Country of type locality: Mexico.

**Geographical distribution.**NEO.

**NEO**: Mexico.

#### Genus Ypsilonigaster Fernandez-Triana, 2018

***Ypsilonigaster*** Fernandez-Triana, 2018: 116. Gender: feminine. Type species: *Ypsilonigastertiger* Fernandez-Triana & Boudreault, 2018, by original designation.

Six species were recently described (Fernandez-Triana and Boudreault 2018), but we have seen a few undescribed ones in collections. No host data are currently available for this genus. There are two DNA-barcode compliant sequences of this genus in BOLD, representing two BINs (although those sequences have not been identified in BOLD as belonging to *Ypsilonigaster*, see Fernandez-Triana and Boudreault 2018 for that). In the original description of *Ypsilonigaster*, its gender was incorrectly stated to be neuter (Fernandez-Triana & Boudreault, 2018: 117); however, all genera ending in *gaster* are feminine, without exception (Doug Yanega, pers. comm., see also Article 30.1.2 of the ICZN), so here we correct that previous mistake.

***Ypsilonigasterbumbana* (de Saeger, 1942)**

*Microgasterbumbana* de Saeger, 1942.

**Type information.** Holotype female, RMCA (not examined but original description checked). Country of type locality: Democratic Republic of Congo.

**Geographical distribution.**AFR.

**AFR**: Democratic Republic of Congo.

***Ypsilonigasternaturalis* Fernandez-Triana & Boudreault, 2018**

*Ypsilonigasternaturalis* Fernandez-Triana & Boudreault, 2018.

**Type information.** Holotype female, RMNH (examined). Country of type locality: Malaysia.

**Geographical distribution.**OTL.

**OTL**: Malaysia.

***Ypsilonigasterpteroloba* (de Saeger, 1944)**

*Microgasterpteroloba* de Saeger, 1944.

**Type information.** Holotype female, RMCA (not examined but original description checked). Country of type locality: Democratic Republic of Congo.

**Geographical distribution.**AFR.

**AFR**: Democratic Republic of Congo.

***Ypsilonigastersharkeyi* Fernandez-Triana & Boudreault, 2018**

*Ypsilonigastersharkeyi* Fernandez-Triana & Boudreault, 2018.

**Type information.** Holotype male, CNC (examined). Country of type locality: Democratic Republic of Congo.

**Geographical distribution.**AFR.

**AFR**: Democratic Republic of the Congo.

***Ypsilonigastertiger* Fernandez-Triana & Boudreault, 2018**

*Ypsilonigastertiger* Fernandez-Triana & Boudreault, 2018.

**Type information.** Holotype female, QSBG (examined). Country of type locality: Thailand.

**Geographical distribution.**OTL.

**OTL**: Thailand.

***Ypsilonigasterzuparkoi* Fernandez-Triana & Boudreault, 2018**

*Ypsilonigasterzuparkoi* Fernandez-Triana & Boudreault, 2018.

**Type information.** Holotype male, CAS (examined). Country of type locality: Madagascar.

**Geographical distribution.**AFR.

**AFR**: Madagascar.

#### Genus Zachterbergius Fernandez-Triana, 2018

***Zachterbergius*** Fernandez-Triana, 2018: 129. Gender: neuter. Type species: *Zachterbergiustenuitergum* Fernandez-Triana & Boudreault, 2018, by original designation.

Only one species is known, from the Oriental region. No host data are currently available for this genus. There is a single DNA-barcode compliant sequence of this genus in BOLD, representing one BIN (although that sequence has not been identified in BOLD as belonging to *Zachterbergius*, see Fernandez-Triana and Boudreault 2018 for that).

***Zachterbergiustenuitergum* Fernandez-Triana & Boudreault, 2018**

*Zachterbergiustenuitergum* Fernandez-Triana & Boudreault, 2018.

**Type information.** Holotype male, QSBG (examined). Country of type locality: Thailand.

**Geographical distribution.**OTL.

**OTL**: Thailand.

#### Species inquirendae

Below we treat 36 species for most of which we could not examine the types or any other specimens; the original descriptions, if available to us, were insufficient to determine a correct generic placement (in the case of *Apantelessanctivicenti* Ashmead, 1900, the type was in poor condition; and in the case of *Apantelesanapiedrae* Fernandez-Triana, 2014 more studies on the holotype and paratypes are required). Until types of those species can be examined and/or more studies are done, we consider them here as *species inquirendae* – they can almost certainly be placed with further research. Additionally, Gupta (2013a: 451) had proposed two Indian species of *Microplitis* to be *incertae sedis*; however, in this paper we consider that one of those species actually belongs to *Diolcogaster* (see *D.dipika* above, p 398, 399), whereas the other should be listed as an unavailable name (see *Microplitisbageshri* in the section Other unavailable names below, p 1033).

? ***Apantelesacaciae* Risbec, 1951**

*Apantelesacaciae* Risbec, 1951.

**Type information.** Holotype male, depository unknown (not examined but original description checked). Country of type locality: Senegal.

**Geographical distribution.**AFR.

**AFR**: Senegal.

**Notes.** This species is likely not an *Apanteles*, but the original description, based on a single male specimen, is not clear enough to determine the correct generic placement.

? ***Apantelesahmednagarensis* Kurhade & Nikam, 1997**

*Apantelesahmednagarensis* Kurhade & Nikam, 1997.

**Type information.** Holotype female, BAMU (not examined but original description checked). Country of type locality: India.

**Geographical distribution.**OTL.

**OTL**: India.

**Notes.** The original description does not provide enough details to confirm the species placement. The low-quality figures provided seem to indicate that the propodeum and T1 are not as in *Apanteles*.

? ***Apantelesanapiedrae* Fernandez-Triana, 2014**

*Apantelesanapiedrae* Fernandez-Triana, 2014.

**Type information.** Holotype female, CNC (examined). Country of type locality: Costa Rica.

**Geographical distribution.**NEO.

**NEO**: Costa Rica.

**Notes.** This species is likely not an *Apanteles*, as stated even in the original description (Fernandez-Triana et al. 2014e). We have re-examined the holotype and many paratypes in the CNC; in addition to the inflexible hypopygium and relatively short ovipositor and sheaths (noted in the original description), we have also found that the vannal lobe is mostly setose, which would exclude this species from *Apanteles*. However, we cannot conclude on which genus would be the best placement at the moment, as other morphological traits are variable between *Dolichogenidea*, *Pholetesor* or even *Parapanteles*; and molecular data (DNA barcodes) is not conclusive either.

? ***Apantelesautomeridis* Brèthes, 1926**

*Apantelesautomeridis* Brèthes, 1926.

**Type information.** Type and depository unknown (not examined). Country of type locality: Colombia.

**Geographical distribution.**NEO.

**NEO**: Colombia.

**Notes.**Shenefelt (1972: 450) could not find the original description or the type material and neither could we, so we cannot confirm the generic placement of this species. Here we consider the type and depository of this species as unknown.

? ***Apantelesbarrosi* Porter, 1926**

*Apantelesbarrosi* Porter, 1926.

**Type information.** Holotype female, depository unknown (not examined but original description checked). Country of type locality: Chile.

**Geographical distribution.**NEO.

**NEO**: Chile.

**Notes.** The short original description is insufficient to determine the correct generic placement, but it is clear that this species does not belong to *Apanteles*. Porter (1926: 143) stated that the species has a very short ovipositor and it is morphologically related to *Apantelesriverae* Porter (a species transferred to *Cotesia* in our paper, see under that species, p 351, 352).

? ***Apantelesbauhiniae* Risbec, 1951**

*Apantelesbauhiniae* Risbec, 1951.

**Type information.** Holotype male, depository unknown (not examined but original description checked). Country of type locality: Senegal.

**Geographical distribution.**AFR.

**AFR**: Senegal.

**Notes.** The original description is not detailed enough to determine the correct generic placement. The species does not seem to belong to *Apanteles*, based on the illustration of the propodeum with a complete median carina bisecting a complete areola (Risbec 1951: 460).

? ***Apantelescamachoi* Silva Figueroa, 1917**

*Apantelescamachoi* Silva Figueroa, 1917.

**Type information.** Holotype female, MNNC (not examined but original description checked). Country of type locality: Chile.

**Geographical distribution.**NEO.

**NEO**: Chile.

**Notes.** The original description is not detailed enough to determine the correct generic placement of this species.

? ***Apantelesdeepica* Rao & Chalikwar, 1971**

*Apantelesdeepica* Rao & Chalikwar, 1971.

**Type information.** Holotype male, BAMU (not examined but original description checked). Country of type locality: India.

**Geographical distribution.**OTL.

**OTL**: India.

**Notes.** The original description (based on three male specimens) is not detailed enough to determine the correct generic placement, but this species does not belong to *Apanteles*; it could belong either to *Glyptapanteles* or *Sathon*.

? ***Apantelesdirphiae* Silva Figueroa, 1917**

*Apantelesdirphiae* Silva Figueroa, 1917.

**Type information.** Syntypes female and male, MNNC (not examined but original description checked). Country of type locality: Chile.

**Geographical distribution.**NEO.

**NEO**: Chile.

**Notes.** The original description is not detailed enough to determine the correct generic placement of this species.

? ***Apantelesespinosai* Porter, 1920**

*Apantelesespinosai* Porter, 1920.

**Type information.** Type and depository unknown (not examined but original description checked). Country of type locality: Chile.

**Geographical distribution.**NEO.

**NEO**: Chile.

**Notes.** The original description is not detailed enough to determine the correct generic placement of this species, but it is evident that is not *Apanteles*, because it mentions a median longitudinal carina on the propodeum. The only known specimen, not even clear if it is a female or male, was deposited in Porter’s personal collection, but we are not aware of the current depository of that collection or if the specimen still exists.

? ***Apanteleshoffmanni* Silva Figueroa, 1917**

*Apanteleshoffmanni* Silva Figueroa, 1917.

**Type information.** Holotype female, MNNC (not examined but original description checked). Country of type locality: Chile.

**Geographical distribution.**NEO.

**NEO**: Chile.

**Notes.** The original description is not detailed enough to determine the correct generic placement of this species.

? ***Apanteleslaorae* Porter, 1921**

*Apanteleslaorae* Porter, 1921.

**Type information.** Syntypes female and male, depository unknown (not examined but original description checked). Country of type locality: Chile.

**Geographical distribution.**NEO.

**NEO**: Chile.

**Notes.** The original description is not detailed enough to determine the correct generic placement of this species. There are no details of the number of specimens studied or the depository, but Porter mentions many specimens from the host larva, so we infer that the type series must have included both females and males.

? ***Apanteleslatiannulatus* (Cameron, 1910)**

*Xestapanteleslatiannulatus* Cameron, 1910.

**Type information.** Holotype female, ZMHB (not examined but subsequent treatment of the species checked). Country of type locality: Mozambique.

**Geographical distribution.**AFR.

**AFR**: Mozambique.

**Notes.**Wilkinson (1932a: 324) explained why it may never be possible to establish the status of this species, due to the very poor condition of the two known specimens.

? ***Apantelesmontanus* de Saeger, 1944**

*Apantelesmontanus* de Saeger, 1944.

**Type information.** Holotype female, RMCA (not examined but original description checked). Country of type locality: Democratic Republic of Congo.

**Geographical distribution.**AFR.

**AFR**: Democratic Republic of Congo.

**Notes.** The original description is not detailed enough (or characters are uninformative) to determine the correct generic placement of this species. It does not belong to *Apanteles* because a median longitudinal carina on the propodeum is mentioned. Other features such as a pleated hypopygium and relatively long ovipositor sheaths, would suggest *Choeras* as the likely genus, but the shape of T2 would be very unusual (as compared to other known species in that genus).

? ***Apantelesnecator* (Bechstein & Scharfenberg, 1805)**

*Ichneumonnecator* Bechstein & Scharfenberg, 1805.

*Microgasternecatrix* Schulz, 1906.

*Ichneumonnecator* Bechstein & Scharfenberg, 1805 [junior primary homonym of *Ichneumonnecator* Fabricius, 1777].

**Type information.** Holotype male, ZMUC (not examined but subsequent treatment of the species checked). Country of type locality: Germany.

**Geographical distribution.**PAL.

**PAL**: Germany.

**Notes.** Our species concept is based on descriptions provided in the historical literature (Haliday 1834, Fahringer 1837). Both authors mentioned the ovipositor as not visible (or hidden), an indication that the ovipositor and sheaths are very short, which excludes *necator* from *Apanteles*. The rest of the information provided in those papers is too general, e.g., the description of antenna length, colour of body, wings and veins, to help determine the correct generic placement. Based on the general colour of the body, host data (Pterophoridae) and the number of wasp cocoons (forming a cocoon mass), this species could be placed either in *Cotesia* (most likely) or *Glyptapanteles*.

? ***Apantelesnigripes* (Ratzeburg, 1844)**

*Microgasternigripes* Ratzeburg, 1844.

**Type information.** Type and depository unknown (not examined but subsequent treatment of the species checked). Country of type locality: Germany.

**Geographical distribution.**PAL.

**PAL**: Bulgaria, Germany, Latvia.

**Notes.** Like many of Ratzeburg’s types, the *nigripes* type is lost or presumed destroyed. Shenefelt (1972) treated this species as *Apanteles* (*sensu lato*), but the actual generic placement of this species is unknown. Besides Germany, supposedly the country of the type locality, the other countries cited for this species, Bulgaria and Latvia (see Yu et al. 2016 for details) should be considered as suspicious. Broad et al. (2016) excluded the species from UK, based on Papp (1988) and van Achterberg (2003), a decision we accept and follow here.

? ***Apantelesreedi* Porter, 1920**

*Apantelesreedi* Porter, 1920.

**Type information.** Type and depository unknown (not examined but original description checked). Country of type locality: Argentina.

**Geographical distribution.**NEO.

**NEO**: Argentina, Chile.

**Notes.** This species is not an *Apanteles*, based on the very short ovipositor. The original description is not detailed enough to allow us to determine the correct generic placement.

? ***Apantelessanctivicenti* Ashmead, 1900**

*Apantelessanctivicenti* Ashmead, 1900.

*Rhygoplitissanctivincenti* Ashmead, 1900. See Fernandez-Triana et al. (2014e).

**Type information.** Holotype male, NHMUK (examined). Country of type locality: Saint Vincent.

**Geographical distribution.**NEO.

**NEO**: Saint Vincent.

**Notes.**Fernandez-Triana et al. (2014e) discussed in detail problems with this species name and tentatively classified it in *Rhygoplitis*. At the time, the type was thought to be lost and all of the assumptions were based on the original description. However, we recently found the type specimen in NHMUK and have been able to examine it in detail. Unfortunately a large drop of glue covers most of the propodeum and T1 and thus it is not possible to determine with certainty its generic status. But it is now evident that the species does not belong to *Rhygoplitis* as it does not have visible notauli, T2 is smooth, and vein R1 in the fore wing is relatively very large (longer than pterostigma length and several times longer than the distance between its end and the end of vein RS). Those features are unlike any known species of *Rhygoplitis* (a genus characterized, among other things, by strong notauli, T2 strongly sculptured, and relatively short vein R1 in fore wing). Based on the mostly smooth anteromesoscutum, thin scutoscutellar sulcus, T1 shape and hind wing with vannal lobe apparently fully setose (but vannal lobe not totally clear because of glue obscuring its view) this species could be placed within *Dolichogenidea* (but it could also be *Apanteles* if the hind wing vannal lobe is interpreted differently). The main problem in placing this species in *Dolichogenidea* (or *Apanteles* for that matter) is that the original description mentions a median longitudinal carina in the propodeum, which would exclude it from either genus. But it is possible that Ashmead (1900) misinterpreted the presence of a median longitudinal carina (indeed, if he examined the specimen after it was glued, it would have not been possible to see it, especially with the microscope available at that time). Another possibility would be *Pseudapanteles* (a genus with a median longitudinal carina), but what can be seen from propodeum and T1 (both relatively sculptured) does not match well with our current concept of *Pseudapanteles*. Because of that, it is not possible to establish with certainty the generic identity of *sanctivincenti* until the type is unglued from the pin for re-examination and/or DNA is extracted.

? ***Apantelesshrii* Sathe & Ingawale, 1995**

*Apantelesshrii* Sathe & Ingawale, 1995.

**Type information.** Holotype female, NZSI (not examined but original description checked). Country of type locality: India.

**Geographical distribution.**OTL.

**OTL**: India.

**Notes.** The original description and illustrations are very deficient and the elements provided therein do not allow to establish with any accuracy to which genus the species belongs, but it is very clear that this species does not belong to *Apanteles*. The described sculpture of propodeum, unpleated hypopygium, and length of ovipositor sheaths all suggest it could be *Parapanteles*; but the authors also stated that the antennal flagellomeres are three segmented and that the ovipositor sheaths have no setae, both features not known in any described species of *Parapanteles*. The authors mentioned that the new taxon is similar to two previously described Microgastrinae species, one of which belongs to *Cotesia* and the other to *Dolichogenidea* (two unrelated genera with many different morphological features). The illustrations provided are somewhat inaccurate, e.g., the venation of the hind wing, and the proportions of the metacoxa and metafemur are different in the drawing as compared to what is detailed in the written description. The specimens on which the species description was based (45 female and 20 male specimens) were all reared from *Eariasvittella* (Fabricius, 1794) (Nolidae). That host record cannot be attributed unequivocally to any specific genus of Microgastrinae (four genera: *Apanteles*, *Cotesia*, *Diolcogaster*, and *Dolichogenidea* all have species previously recorded as parasitizing the genus *Earias*). All of the above evidence indicates a rather poorly characterized species with insufficient information to establish its generic placement, other than not belonging in *Apanteles*.

? ***Apantelestineaephagus* Bhatnagar, 1950**

*Apantelestineaephagus* Bhatnagar, 1950.

**Type information.** Holotype female, INPC (not examined but original description checked). Country of type locality: India.

**Geographical distribution.**OTL.

**OTL**: India.

**Notes.** The original description is not detailed enough to determine the correct generic placement; we consider that it could be either *Parapanteles*, *Apanteles* or *Dolichogenidea*. The year of publication of the Bhatnagar paper was until recently commonly cited as 1948 and/or 1950 (e.g., Chen and Song 2004, Yu et al. 2016), probably following Shenefelt (1972) who referred to this paper as “Bhatnagar (1948) 1950”. While the intended year for Volume X, Parts I & II of the Indian Journal of Entomology was 1948, the actual dates of publication were June 1950 (Part I) and October 1950 (Part II), as clearly shown on the cover page of the Volume, which we have checked. Because the dates of publication are the ones to be considered, and for the sake of clarity, we hereby revise the species year of description to 1950.

? ***Choeraspappi* Narendran, 1998**

*Choeraspappi* Narendran, 1998.

**Type information.** Holotype female, RMNH (not examined but original description checked). Country of type locality: India.

**Geographical distribution.**OTL.

**OTL**: India.

**Notes.** The original description is not detailed enough to determine the correct generic placement. But it mentions a very short ovipositor (half metacoxal length), which indicates that this species does not belong in *Choeras*. This is also corroborated by the illustration of veins r and 2RS in the fore wing (Narendran 1998: fig. 5), which do not look like those of any other described species of *Choeras*.

? ***Cotesiapicipes* (Bouché, 1834)**

*Microgasterpicipes* Bouché, 1834.

**Type information.** Type and depository unknown (not examined but subsequent treatment of the species checked). Country of type locality: unknown.

**Geographical distribution.**PAL.

**PAL**: Azerbaijan, France, Germany, Hungary, Italy, Russia (SPE, VLA), Tajikistan, Uzbekistan.

**Notes.** The type of *picipes* is presumed to be lost. Broad et al (2016), based on van Achterberg (2003) not citing *picipes* for the Western Palearctic, excluded the species from their United Kingdom list. Broad et al (2016) considered that *picipes* might be a synonym or a *nomen dubium*. However, at least two sources (Belokobylskij et al. 2003, Papp 2005) considered the species as valid, and actually belonging to *Cotesia*, in contrast with Papp (1987a), who provisionally considered *picipes* to be a synonym of *Apantelesxanthostigmus*.

? ***Glyptapantelesconopomorphae* Tsang & You, 2007**

*Glyptapantelesconopomorphae* Tsang & You, 2007.

**Type information.** Holotype female, SCAC (not examined but original description checked). Country of type locality: China.

**Geographical distribution.**OTL.

**OTL**: China (GD).

**Notes.** Based on the illustrations from the original description, this species is not likely to be *Glyptapanteles* (the ovipositor sheaths are relatively long, the propodeum shows a partial areola defined apically, and T2 is relatively transverse, unlike most described *Glyptapanteles*). We believe this species could be better placed in *Dolichogenidea*; however, the vannal lobe in the hind wing and the hypopygium are not clearly visible to help determine the correct generic placement.

? ***Microgasteralvearifex* (Schrank, 1781)**

*Ichneumonalvearifex* Schrank, 1781.

*Ichneumonalveariformis* Geoffroy, 1785.

**Type information.** Type and depository unknown (not examined but subsequent treatment of the species checked). Country of type locality: unknown.

**Geographical distribution.**PAL.

**PAL**: Austria, Germany, Italy.

**Notes.** We read the very short comments and/or descriptions in Gmelin (1790: 2712), Haliday (1834: 257) and Fahringer (1837: 368), mostly reproducing what Schrank (1781) wrote about the species. The species was considered to be part of “*Microgastersensu lato*, including *Apanteles* and *Microplitis*” (see Fahringer (1937), which would include most of the Microgastrinae at the time. The available details, about the cocoons (white and forming a mass like a honeycomb) as well as general colour of the adult wasp (body black, legs reddish), are not sufficient to place this species correctly to genus but seem to indicate that is probably not *Microgaster*. For example, the shape of the cocoon mass is stated to be similar to those of *Ichneumonalvearius* Fabricius, 1798 (a species currently in *Diolcogaster*) and we are aware of a similar cocoon mass made by *Sathonfalcatus* (Nees, 1834).

? ***Microgasterannulipesiduo* Shenefelt, 1973**

*Microgasterannulipesiduo* Shenefelt, 1973.

*Microgasterannulipes* Motschoulsky, 1863 [primary homonym of *Microgasterannulipes* Curtis, 1830].

**Type information.** Holotype male, depository unknown (not examined but subsequent treatment of the species checked). Country of type locality: Sri Lanka.

**Geographical distribution.**OTL.

**OTL**: Sri Lanka.

**Notes.**Wilkinson (1927: 173) thought this species did not belong in *Microgaster*, a statement we agree with.

? ***Microgasterduvauae* Brèthes, 1916**

*Microgasterduvauae* Brèthes, 1916.

**Type information.** Holotype female, MACN (not examined but original description checked). Country of type locality: Argentina.

**Geographical distribution.**NEO.

**NEO**: Argentina.

**Notes.** The original description is not clear enough to conclude, but it seems that *duvauae* does not belong to *Microgaster*, based on details of T1 and T2, body size, wing length, and presumed host. Until further study of the type is done, it is not possible to establish with certainty the generic placement of the species.

? ***Microgastereurygaster* (Cameron, 1911)**

*Apanteleseurygaster* Cameron, 1911.

**Type information.** Holotype male, TMSA (not examined but subsequent treatment of the species checked). Country of type locality: South Africa.

**Geographical distribution.**AFR.

**AFR**: South Africa.

**Notes.** Based on the original description of the holotype male (only known specimen) and the subsequent treatment (Cameron 1911c, Wilkinson 1929a, de Saeger 1944) this species is clearly not a *Microgaster*. However, it is not possible to place it in any genus with any degree of certainty, as the description is not conclusive.

? ***Microgastermortuorum* (Rossi, 1792)**

*Ichneumonmortuorum* Rossi, 1792.

**Type information.** Type and depository unknown (not examined). Country of type locality: unknown.

**Geographical distribution.**PAL.

**PAL**: Italy.

**Notes.**Yu et al. (2012) list this species with a question mark regarding its status and generic placement. Other than the original description, which we have not been able to see, very few references, all of them catalogues, treat this species.

? ***Microgasternigricornis* Motschoulsky, 1863**

*Microgasternigricornis* Motschoulsky, 1863.

**Type information.** Type and depository unknown (not examined but subsequent treatment of the species checked). Country of type locality: Sri Lanka.

**Geographical distribution.**OTL.

**OTL**: Sri Lanka.

**Notes.**Wilkinson (1927: 173) thought this species did not possibly belong in *Microgaster*, a statement we agree with. Shenefelt (1973: 718) added a male sign when referring to the original description of the species, but it is not clear if that means a single specimen (which would then be considered as the holotype), or a series of male specimens (which would then be syntypes); thus, we consider the type status as unknown for the time being. Based on the key by Wilkinson (1927), we suspect that this species does not belong to *Microgaster*, but until further study(ies) of the type(s), it is not possible to establish with certainty the generic placement of the species.

? ***Microgasterpictipes* Marshall, 1898**

*Microgasterpictipes* Marshall, 1898.

**Type information.** Holotype male, depository unknown (not examined but original description checked). Country of type locality: Spain.

**Geographical distribution.**PAL.

**PAL**: France, Spain.

**Notes.** Based on the original description, this species is not *Microgaster*, as T2 is relatively very short (half the length of T3) and T2 has oblique divergent grooves delimiting a triangular area. The original description, and a similar translation by Telenga (1955) are not clear enough to determine if this species should be better placed in *Microplitis* or *Diolcogaster* (or even *Rasivalva*).

? ***Microgasterpinos* Cresson, 1865**

*Microgasterpinos* Cresson, 1865.

**Type information.** Holotype female, ANSP (not examined but original description checked). Country of type locality: Cuba.

**Geographical distribution.**NEO.

**NEO**: Cuba.

**Notes.** The original description is not clear enough to determine the correct generic placement. Cresson (1865: 67) considered the fore wing venation of *pinos* to be similar to that of *Microgastermarginiventris* Cresson (i.e., without an areolet), a species currently classified in *Cotesia*. This strongly suggest that *pinos* does not belong in *Microgaster* (in the modern sense), as all *Microgaster* have a large areolet whereas all *Cotesia* lack an areolet. Muesebeck (1921: 11), even though he did not see the type of *pinos*, placed it within *Apanteles* (which, at the time included *Cotesia*, but not *Microgaster*). After analyzing all available evidence, we conclude that *pinos* very likely belongs to one of the *Apantelessensu lato* genera.

? ***Microgasterruficoxis* Ruthe, 1858**

*Microgasterruficoxis* Ruthe, 1858.

**Type information.** Type and depository unknown (not examined but subsequent treatment of the species checked). Country of type locality: Germany.

**Geographical distribution.**PAL.

**PAL**: Germany.

**Notes.** The original description is not sufficiently detailed to determine the correct generic placement. Our species concept is based on Telenga (1955). This species is very unlikely to belong to *Microgaster*, as the metacoxa is described as very long, half the metasoma length. It could likely belong to *Diolcogaster*.

? ***Microplitisbambusanus* de Saeger, 1944**

*Microplitisbambusanus* de Saeger, 1944.

**Type information.** Holotype female, RMCA (not examined but original description checked). Country of type locality: Democratic Republic of Congo.

**Geographical distribution.**AFR.

**AFR**: Democratic Republic of Congo, Rwanda.

**Notes.** This species almost certainly does not belong in *Microplitis*. The original description provides some features that suggest it could belong to *Jenopappius*, but other characters are slightly different.

? ***Microplitisisis* de Saeger, 1944**

*Microplitisisis* de Saeger, 1944.

**Type information.** Holotype male, RMCA (not examined but original description checked). Country of type locality: Democratic Republic of Congo.

**Geographical distribution.**AFR.

**AFR**: Democratic Republic of Congo.

**Notes.** This species almost certainly does not belong in *Microplitis*. The original description provides some features that suggest it could belong to *Alloplitis*, but other characters are slightly different.

? ***Promicrogastersaraswatii* Sathe & Bhoje, 1998**

*Promicrogastersaraswatii* Sathe & Bhoje, 1998.

**Type information.** Holotype female, depository unknown (not examined but original description checked). Country of type locality: India.

**Geographical distribution.**OTL.

**OTL**: India.

**Notes.**Fernandez-Triana et al. (2016b) provided several reasons to treat this species as *incertae sedis*; however, here we think it is more appropriate to consider it as a *species inquirenda*. The host record associated with this species in the original description is highly suspicious.

? ***Venanidesmoldavicus* (Tobias, 1975)**

*Apantelesmoldavicus* Tobias, 1975.

**Type information.** Holotype female, ZIN (not examined but subsequent treatment of the species checked). Country of type locality: Moldova.

**Geographical distribution.**PAL.

**PAL**: Armenia, Korea, Moldova, Russia (VOR), Slovakia, Ukraine, United Kingdom.

**Notes.** The drawings in Tobias (1975, 1986) suggest that this species belongs to *Venanides*, the same generic placement reported by Capek and Lukas (1989). However, Papp (1988, 1990b), Belokobylskij et al. (2019), Shaw (2012b), and Broad et al. (2016) placed it in *Pholetesor* although the latter two papers considered that as a provisional or even questionable generic placement. Specimens of *moldavicus* we examined seem to fit better within the Cotesini group (*sensu*Mason 1981), which contains genera such as *Venanides* and *Glyptapanteles* (two genera that we consider are the best candidates for *moldavicus*). Morphological evidence is not sufficient to determine the correct generic placement but ongoing molecular studies of those specimens should help determine this.

#### Nomina dubia

***Apantelesanomalon* (Curtis, 1830)**

*Microgasteranomalon* Curtis, 1830.

**Type information.** Type unknown, MVMMA (not examined but subsequent treatment of the species checked). Country of type locality: United Kingdom.

**Geographical distribution.**PAL.

**PAL**: United Kingdom.

**Notes.**Shenefelt (1972: 443) gave England as the country where this species is found, information accepted by most researchers afterwards (see Yu et al. 2016 for complete list of historical references). However, Broad et al (2016) did not consider this species to be present in the United Kingdom, adding the following: “This name appeared in Huddleston (1978) but is not listed by Papp (1988) or van Achterberg (2003c) and remains uninterpreted”. Because this species had been only recorded from the United Kingdom, its status will require further investigation.

***Cotesiasessilis* (Geoffroy, 1785)**

*Evaniasessilis* Geoffroy, 1785.

*Evaniasessilis* (Fabricius, 1793).

*Apantelestetrica* (Reinhard, 1880).

*Microgasteropacula* (Thomson, 1895).

**Type information.** Type and depository unknown (not examined but subsequent treatment of the species checked). Country of type locality: unknown.

**Geographical distribution.**PAL.

**PAL**: Armenia, Austria, Azerbaijan, Belarus, Belgium, Croatia, Czech Republic, Estonia, Finland, France, Germany, Greece, Hungary, Iran, Ireland, Italy, Kazakhstan, Latvia, Lithuania, Moldova, Norway, Poland, Romania, Russia (ALT, DA, KGD, KDA, MOS, PRI, RYA, SPE, SAR, VLA, VOR, YAR), Serbia, Sweden, Switzerland, Tajikistan, Turkey, Uzbekistan.

**Notes.** The name *sessilis* has been interpreted in different ways: a) Yu et al. (2016) considered it a valid species, with both *C.juniperatae* (Bouché, 1834) and *C.tetrica* (Reinhard, 1880) as its synonyms; b) Papp (1988) and Kotenko (2007) considered *tetrica* as a valid species, with *sessilis* as its synonym, with a question mark; c) Belokobylskij et al. (2003) deemed *juniperatae* as a valid species, with *sessilis* as its synonym; and d) Broad et al (2016) deemed *juniperatae* and *tetrica* to be a valid species, but did not list *sessilis* as a synonym of either. With the type and depository unknown, and the evidence available to us being contradictory (for more details see our Notes under *Cotesiabrachycera* in the checklist above, p 285–287) we consider it impossible to conclude on the status of the *sessilis* name for the time being and thus consider it as a *nomen dubium*. The distribution of *sessilis* detailed above is taken from Yu et al. (2016), which is a compilation of historical references; however, that is very likely to be inaccurate, due to the many potential species linked to this name over the years.

***Microgastersubcutanea* (Linnaeus, 1758)**

*Ichneumonsubcutaneus* Linnaeus, 1758.

**Type information.** Type and depository unknown (not examined but subsequent treatment of the species checked). Country of type locality: unknown.

**Geographical distribution.**PAL.

**PAL**: Finland, Norway.

**Notes.** We have studied a) the original description (Linnaeus 1758: 568); b) a lateral habitus of the species illustrated in DeGeer (1752, plate 30, figure 21), a paper that predates Linnaeus work, but which is supposed to be the source used by Linnaeus to describe the species (see Fitton 1978: 379 for a discussion on that topic); and c) Zetterstedt’s (1838: 404–405) redescription of the species. The illustration from DeGeer (1752; fig. 21) indeed seems to represent a braconid wasp (as recognized by Fitton 1978), but it does not look like a Microgastrinae, as there appears to be a closed marginal cell in the fore wing (defined by a complete vein RS) and the vein M is also very long, almost reaching the apex of the wing. That same illustration also shows what appears to be an elongate glossa (not common but present in a few species of several genera in Microgastrinae), and the overall appearance of the metasoma looks somewhat different from a typical microgastrine wasp; however, we are hesitant to make a decision based just on an old drawing which may not be accurate enough to be meaningful. The other source we read, the description from Zetterstedt (1838), is actually more in line with the Microgastrinae concept of that time (where all species were considered to belong to the genus *Microgaster*), and it seems to support the idea of the species belonging to that subfamily. With the evidence available to us being contradictory and relatively very old (a drawing from 1752, a description from 1838) we consider it impossible to conclude on the status of this species for the time being, thus we are here following Fitton (1978) who considered it as a *nomen dubium*. It is also worth mentioning that, according to Fitton (1978), material from the species can still exist in the NHRS in Stockholm and its future study may clarify the status of this name.

#### Nomina nuda

***Apantelesargentinensis* Blanchard, 1937**

*Apantelesargentinensis* Blanchard, 1937.

**Notes.** This species name is mentioned in Bourquin (1937) as a manuscript name and must be considered as *nomen nudum* (see also de Santis 1967a).

***Apantelesdeltinea* Blanchard, 1961**

*Apantelesdeltinea* Blanchard, 1961.

**Notes.** This species name is mentioned in Bourquin (1961) as a manuscript name and must be considered as *nomen nudum* (see also de Santis 1967a).

***Apantelesgeometraephagus* Blanchard, 1939**

*Apantelesgeometraephagus* Blanchard, 1939.

**Notes.** This species name is mentioned in Blanchard (1939) as a manuscript name and must be considered as *nomen nudum* (see also de Santis 1967a).

***Apanteleskoehleri* Blanchard, 1942**

*Apantelesgeometraephagus* Blanchard, 1942.

**Notes.** This species name is mentioned in Blanchard (1942b) as a manuscript name and must be considered as *nomen nudum* (see also de Santis 1967a).

***Apantelesmysticus* Blanchard, 1961**

*Apantelesmysticus* Blanchard, 1961.

**Notes.** This species name is mentioned in Bourquin (1961) as a manuscript name and must be considered as *nomen nudum* (see also de Santis 1967a).

***Apantelesplatystigma* Blanchard, 1938**

*Apantelesplatystigma* Blanchard, 1938.

**Notes.** This species name is mentioned in Blanchard (1938) as a manuscript name and must be considered as *nomen nudum* (see also de Santis 1967a).

***Apantelessericeoneesi* Papp, 1974**

*Apantelessericeoneesi* Papp 1974.

**Notes.** This name was mentioned in two papers by Papp (1974c, 1976a). In both cases the name is only cited in the caption of figure 54, a drawing of T1–T3. No other details, description, or depository are available, and thus the species name is to be treated as a *nomen nudum*. In the CNC there is a female specimen donated by Papp with the same species name, and the specimen metasoma agrees with the drawing in Papp (1974c, 1976a). The CNC specimen was sampled for DNA barcoding, and the resulting, partial sequence (144 base pairs) is deposited in BOLD (voucher code CNCHYM 00707, sequence code: HYCNE518-11).

***Apantelesspeocropiae* Blanchard, 1941**

*Apantelesspeocropiae* Blanchard, 1941.

**Notes.** This species name is mentioned in de Santis (1941) as a manuscript name and must be considered as *nomen nudum* (see also de Santis 1967a).

***Apantelesveronesi* Blanchard, 1940**

*Apantelesveronesi* Blanchard, 1940

**Notes.** This species name is mentioned in Blanchard (1940) as a manuscript name and must be considered as *nomen nudum* (see also de Santis 1967a).

***Cotesiaferventis* Kotenko, 2007**

*Cotesiaferventis* Kotenko, 2007.

**Notes.**Kotenko (2007a: 185) mentioned this name as part of his treatment of Microgastrinae of the Russian Far East. However, the name is not accompanied by any description or any other detail.

***Glyptapantelesobvius* Kotenko, 2007**

*Glyptapantelesobvius* Kotenko, 2007.

**Notes.**Kotenko (2007a: 185) mentioned this name as part of his treatment of Microgastrinae of the Russian Far East. However, the name is not accompanied by any description or any other detail.

***Glyptapantelesurios* Kotenko, 2007**

*Glyptapantelesurios* Kotenko, 2007.

**Notes.**Kotenko (2007a: 185) mentioned this name as part of his treatment of Microgastrinae of the Russian Far East. However, the name is not accompanied by any description or any other detail.

***Microgastereuchthoniae* Blanchard, 1939**

*Microgastereuchthoniae* Blanchard, 1939.

**Notes.** This species name is mentioned in Bourquin (1939) as a manuscript name and must be considered as *nomen nudum* (see also de Santis 1967a).

***Microplitisparapsidalis* Blanchard, 1950**

*Microplitisparapsidalis* Blanchard, 1950.

**Notes.** This species name is mentioned in Ratkovich (1950) as a manuscript name and must be considered as *nomen nudum* (see also de Santis 1967a).

#### Other unavailable names

Below we list 38 species names that were described in post 1999 publications that did not state the type depository – thus they do not fulfill the requirements of ICZN Article 16.4.2 and must be considered as unavailable names. Additionally, in BOLD (http://v4.boldsystems.org/), there are some Microgastrinae sequences with associated names that have never been described in a publication and do not fulfill most of the requirements of ICZN Article 16 to be considered as available names (most of those cases are in the genus *Glyptapanteles*). However, we do not list those names here because they have never been published (BOLD, being an online database, is not considered to be a publication, *sensu*ICZN Article 8 “What constitutes published work”), but we caution against using those names in future publications, as currently they cannot be considered as available.

***Apantelesindica* Chougale, 2016.**

**Notes.** The type and depository are not specified in the original publication.

***Apantelesmultani* Sathe, Inamdar & Dawale, 2003.**

**Notes.** The depository of the type is not specified in the original publication.

***Cotesiaanari* Sathe & Bhoje, 2000.**

**Notes.** The depository of the type is not specified in the original publication.

***Cotesiaarachi* Sathe & Bhoje, 2000.**

**Notes.** The depository of the type is not specified in the original publication.

***Cotesiabazari* Sathe & Bhoje, 2000.**

**Notes.** The depository of the type is not specified in the original publication

***Cotesiachiloi* Sathe & Bhoje, 2000 .**

**Notes.** The depository of the type is not specified in the original publication.

***Cotesiahandhwani* Sathe, Inamdar & Dawale, 2003.**

**Notes.** The depository of the type is not specified in the original publication.

***Cotesiajanati* Sathe & Bhoje, 2000.**

**Notes.** The depository of the type is not specified in the original publication.

***Cotesiamangiferi* Sathe & Bhoje, 2000.**

**Notes.** The depository of the type is not specified in the original publication.

***Cotesiaparnari* Sathe & Bhoje, 2000.**

**Notes.** The depository of the type is not specified in the original publication.

***Cotesiasunflowari* Sathe & Bhoje, 2000.**

**Notes.** The depository of the type is not specified in the original publication.

***Cotesiatuski* Sathe & Bhoje, 2000.**

**Notes.** The depository of the type is not specified in the original publication.

***Dolichogenideabageshri* Sathe, Inamdar & Dawale, 2003.**

**Notes.** The depository of the type is not specified in the original publication.

***Dolichogenideadarbari* Sathe, Inamdar & Dawale, 2003.**

**Notes.** The depository of the type is not specified in the original publication.

***Dolichogenideaexiguvi* Sathe & Bhoje, 2000.**

**Notes.** The depository of the type is not specified in the original publication.

***Dolichogenidealycopersi* Sathe & Bhoje, 2000.**

**Notes.** The depository of the type is not specified in the original publication.

***Dolichogenideamythimna* Sathe & Bhoje, 2000.**

**Notes.** The depository of the type is not specified in the original publication.

***Dolichogenideaoryzae* Bhoje & Sathe, 2002.**

**Notes.** The depository of the type is not specified in the original publication. Additionally, this species name is a secondary homonym of *Dolichogenideaoryzae* Walker, 1994.

***Dolichogenideaparijatki* Sathe & Rokade, 2005.**

**Notes.** The depository of the type is not specified in the original publication.

***Dolichogenidearevatl* Sathe & Rokade, 2005.**

**Notes.** The depository of the type is not specified in the original publication.

***Dolichogenideasathei* Sathe & Rokade, 2005.**

**Notes.** The depository of the type is not specified in the original publication.

***Dolichogenideasushili* Bhoje & Sathe, 2002.**

**Notes.** The depository of the type is not specified in the original publication.

***Dolichogenideasunflowari* Sathe & Bhoje, 2000.**

**Notes.** The depository of the type is not specified in the original publication.

***Dolichogenideatarvadi* Sathe & Rokade, 2005.**

**Notes.** The depository of the type is not specified in the original publication.

***Dolichogenideaujlai* Sathe & Rokade, 2005.**

**Notes.** The depository of the type is not specified in the original publication.

***Glyptapantelesbhupali* Sathe, Inamdar & Dawale, 2003.**

**Notes.** The depository of the type is not specified in the original publication.

***Glyptapantelesmalshri* Sathe, Inamdar & Dawale, 2003.**

**Notes.** This same species was described previously by two of the authors (as *Glyptapantelesmalshri* Sathe & Inamdar, 1991; that species is valid and is treated in this paper – see notes under *Cotesiamalshri* above for more details on that species, p 328). In any case, the name *Glyptapantelesmalshri* Sathe, Inamdar & Dawale, 2003 must be considered as an unavailable name because the depository of the type is not specified in the original (2003) publication.

***Glyptapantelesmelentis* Sathe & Bhoje, 2000.**

**Notes.** The depository of the type is not specified in the original publication.

***Hypomicrogasterminari* Sathe & Bhoje, 2000.**

**Notes.** The depository of the type is not specified in the original publication.

***Microplitisbageshri* Sathe, Inamdar & Dawale, 2003.**

**Notes.** The depository of the type is not specified in the original publication.

***Microplitisvitellipedis* Li, Tan & Song, 2009.**

**Notes.** The depository of the type is not specified in the original description. Ranjith et al. (2015a) mentioned that the holotype of this species is deposited in the HUNAU collection, but that was only an assumption (Ranjith, pers. comm.). They also stated that “the type specimen of this species could not be examined” and that instead they based their species description and illustration on specimens from India which they actually examined. Ranjith et al. (2015a) does not fulfill ICZN Article 16.1, and thus it does not make the species name available.

***Parenionbhairavi* Sathe, Inamdar & Dawale, 2003.**

**Notes.** This same species was described previously by two of the authors (as *Parenionbhairavi* Sathe & Inamdar, 1991; that species is valid and is treated in this paper – see notes under *Cotesiabhairavi* above for more details, p 283, 284). In any case, the name *Parenionbhairavi* Sathe, Inamdar & Dawale, 2003 must be considered as an unavailable name because the depository of the type is not specified in the original (2003) publication.

***Pholetesorrangini* Sathe, Inamdar & Dawale, 2003.**

**Notes.** The depository of the type is not specified in the original publication.

***Promicrogastervachaspati* Sathe, Inamdar & Dawale, 2003.**

**Notes.** The depository of the type is not specified in the original publication.

***Protomicroplitisindus* Ahmed & Usmani, 2016.**

**Notes.** The depository of the type is not specified in the original publication.

***Protomicroplitisshivrangini* Sathe, Inamdar & Dawale, 2003.**

**Notes.** The depository of the type is not specified in the original publication.

***Rhygoplitispahadi* Sathe, Inamdar & Dawale, 2003.**

**Notes.** The depository of the type is not specified in the original publication.

***Semionismadhuvanti* Sathe, Inamdar & Dawale, 2003.**

**Notes.** The depository of the type is not specified in the original publication.

**Figure 5.**
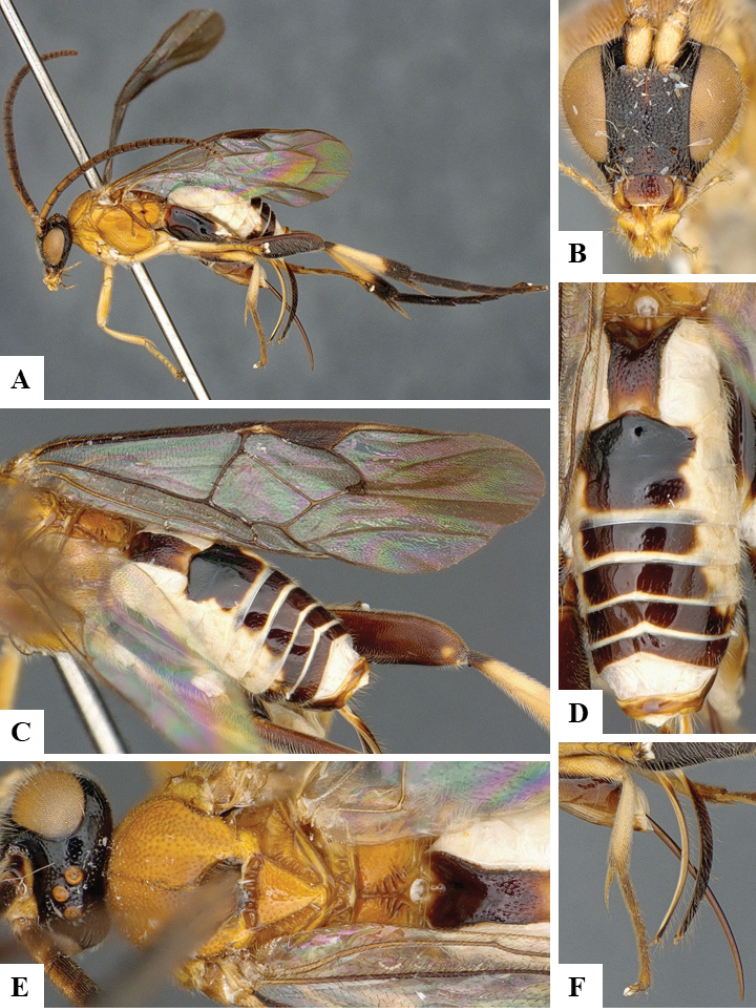
*Aguptadanyi* female holotype **A** Habitus, lateral **B** Head, frontal **C** Fore wing **D** Metasoma, dorsal **E** Head and mesosoma, dorsal **F** Ovipositor and ovipositor sheaths.

**Figure 6.**
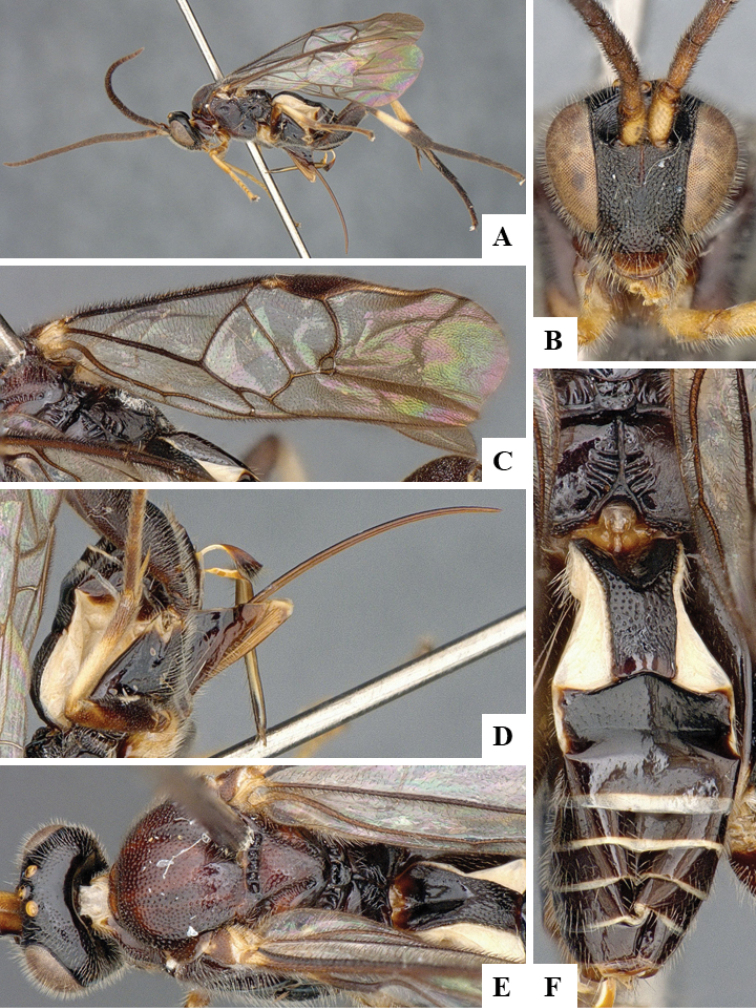
*Aguptajeanphilippei* female holotype **A** Habitus, lateral **B** Head, frontal **C** Fore wing **D** vipositor and ovipositor sheaths **E** Head and mesosoma, dorsal **F** Propodeum and metasoma, dorsal.

**Figure 7.**
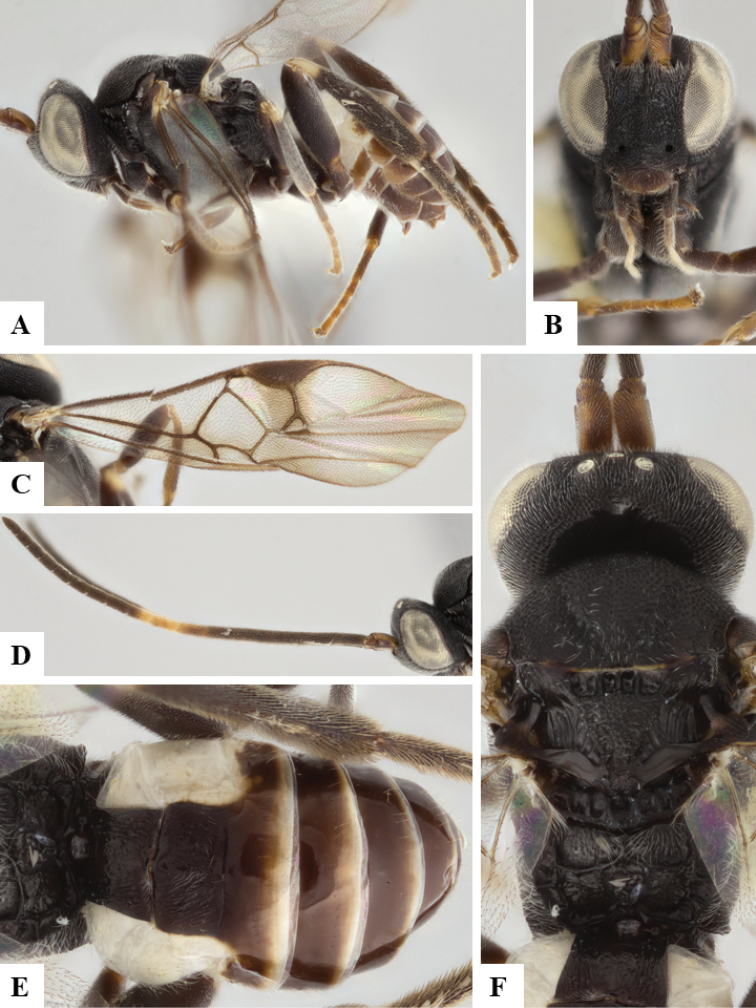
*Alloplitiscompletus* female holotype **A** Habitus, lateral **B** Head, frontal **C** Fore wing **D** Antenna and head, lateral **E** Metasoma, dorsal **F** Head, mesosoma and propodeum, dorsal.

**Figure 8.**
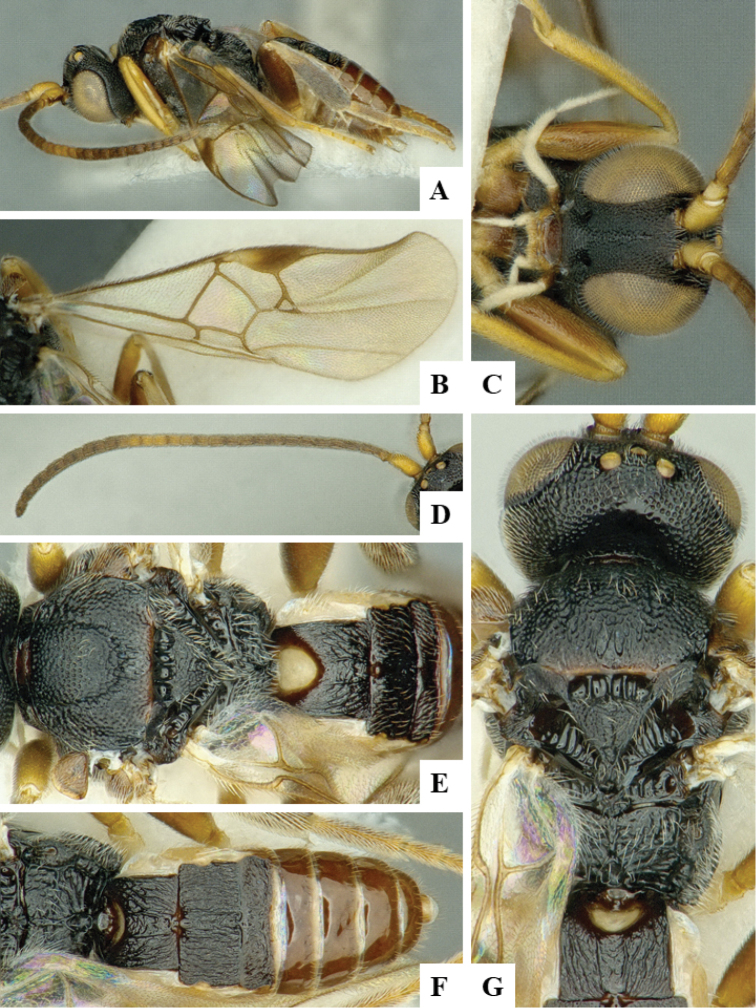
*Alloplitis* sp. female CNC1065631 **A** Habitus, lateral **B** Fore wing **C** Head, frontal **D** Antenna **E** Mesosoma and tergite 1, dorsal **F** Metasoma, dorsal **G** Head and mesosoma, dorsal.

**Figure 9.**
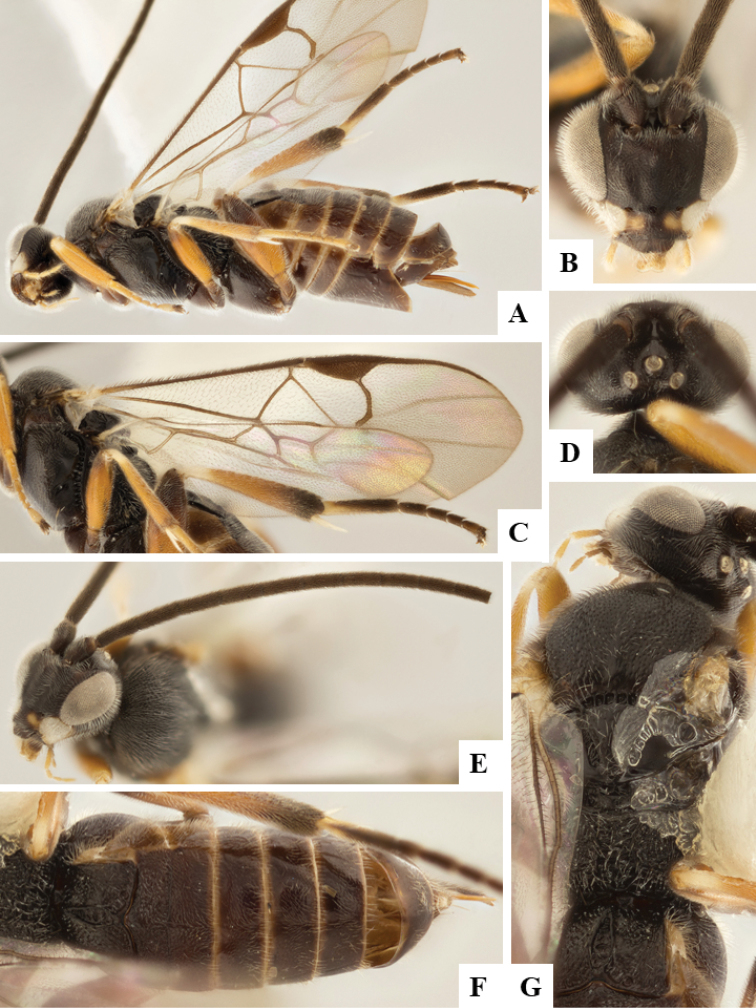
*Alphomelonrhyssocerus* female holotype **A** Habitus, lateral **B** Head, frontal **C** Fore wing **D** Head, dorsal **E** Antenna and head, frontolateral **F** Metasoma, dorsal **G** Mesosoma, dorsal.

**Figure 10.**
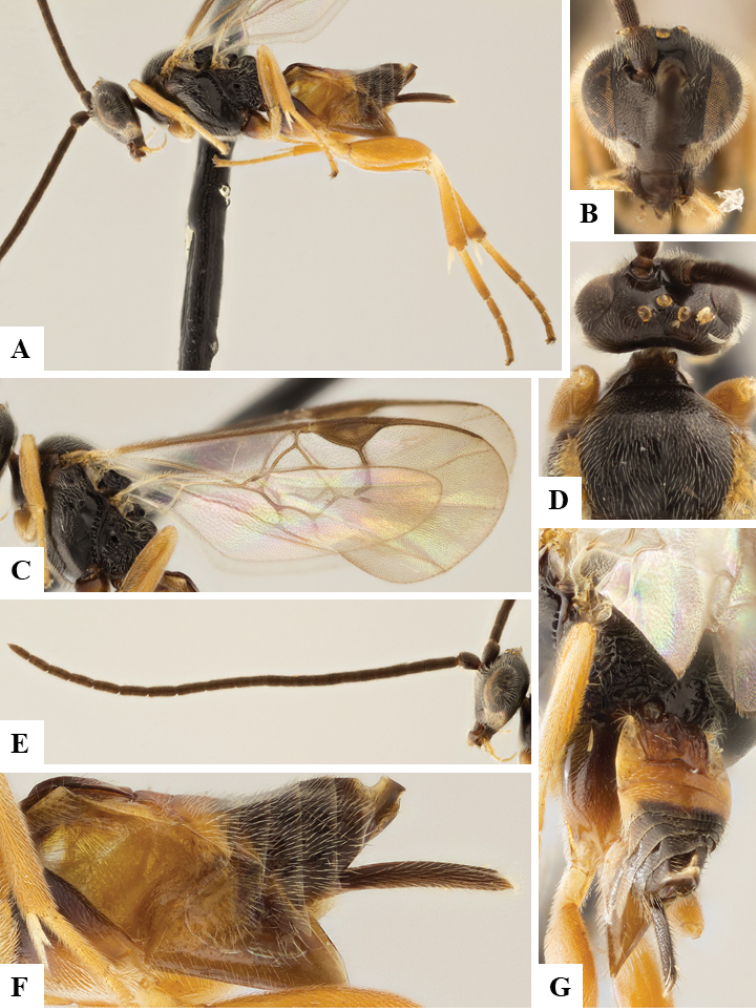
*Alphomelonsimpsonorum* female holotype **A** Habitus, lateral **B** Head, frontal **C** Fore wing **D** Head, dorsal **E** Antenna and head, lateral, **F**- Metasoma and ovipositor sheaths, lateral **G** Propodeum and metasoma, dorsal.

**Figure 11.**
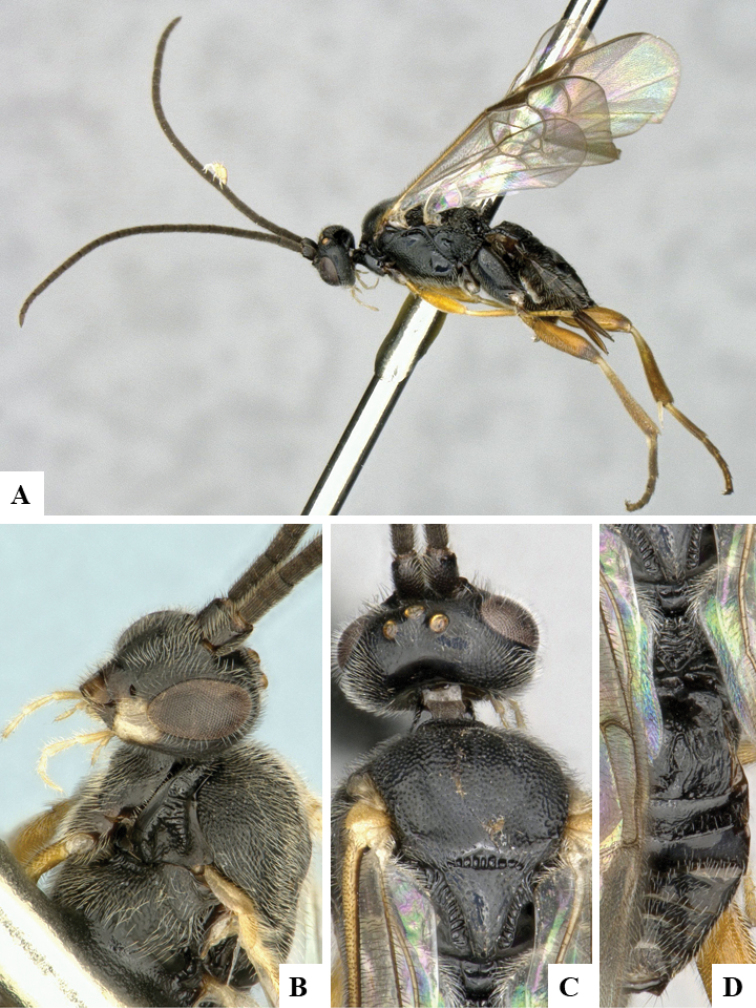
*Alphomelonwinniewertzae* female CNCHYM00025 **A** Habitus, lateral **B** Head and mesosoma, frontolateral **C** Head and mesosoma, dorsal **D** Propodeum and metasoma, dorsal.

**Figure 12.**
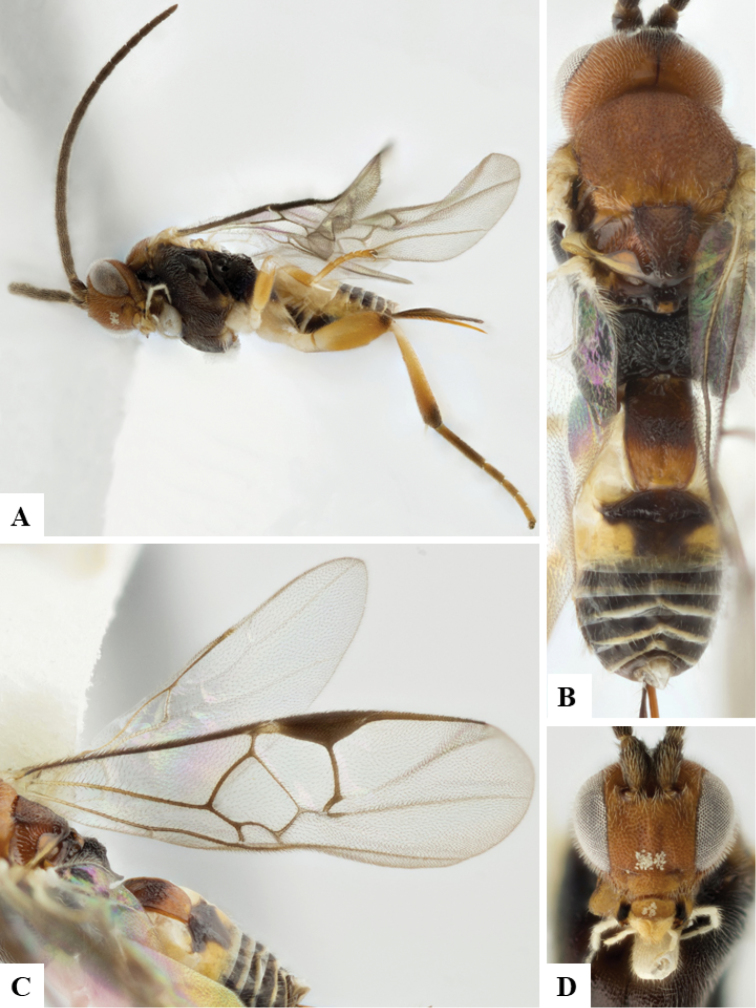
*Apantelesalejandromasisi* female holotype **A** Habitus, lateral **B** Mesosoma and metasoma, dorsal **C** Fore wing and hind wing **D** Head, frontal.

**Figure 13.**
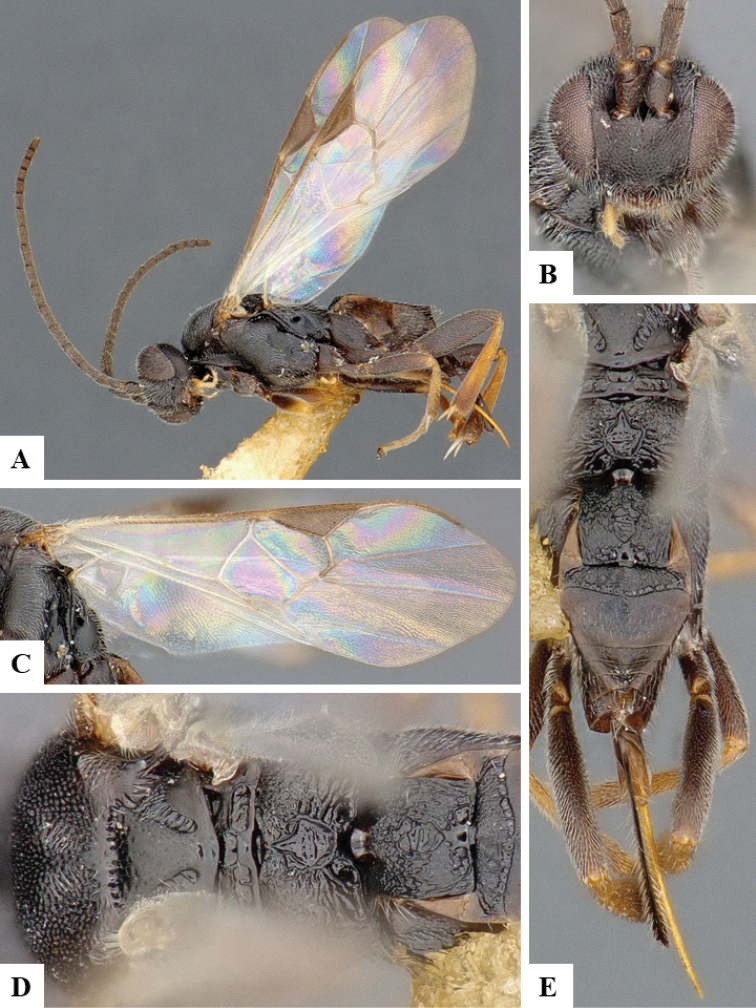
*Apantelesaristoteliae* female CNCHYM00068 **A** Habitus, lateral **B** Head, frontal **C** Fore wing **D** Mesosoma, propodeum and tergite 1, dorsal **E** Metasoma, dorsal.

**Figure 14.**
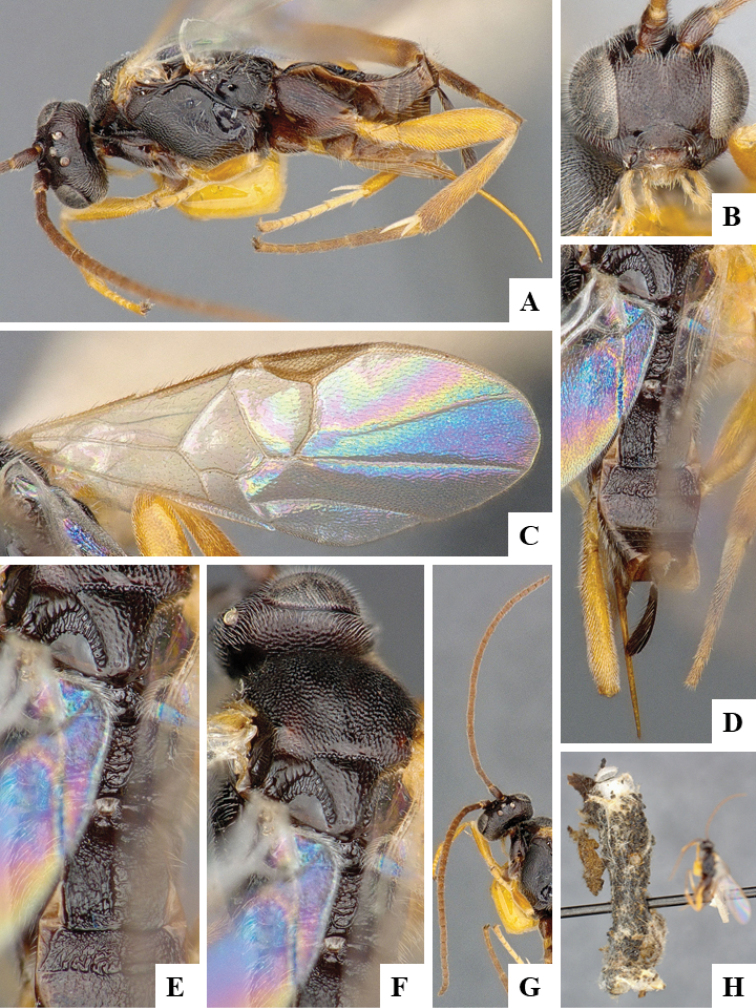
*Apantelesbaldufi* female MIC000024 **A** Habitus, lateral **B** Head, frontal **C** Fore wing **D** Metasoma, ovipositor and ovipositor sheaths, dorsal **E** Propodeum and tergite 1, dorsal **F** Mesosoma, dorsolateral **G** Antenna and head, dorsal **H** Cocoon.

**Figure 15.**
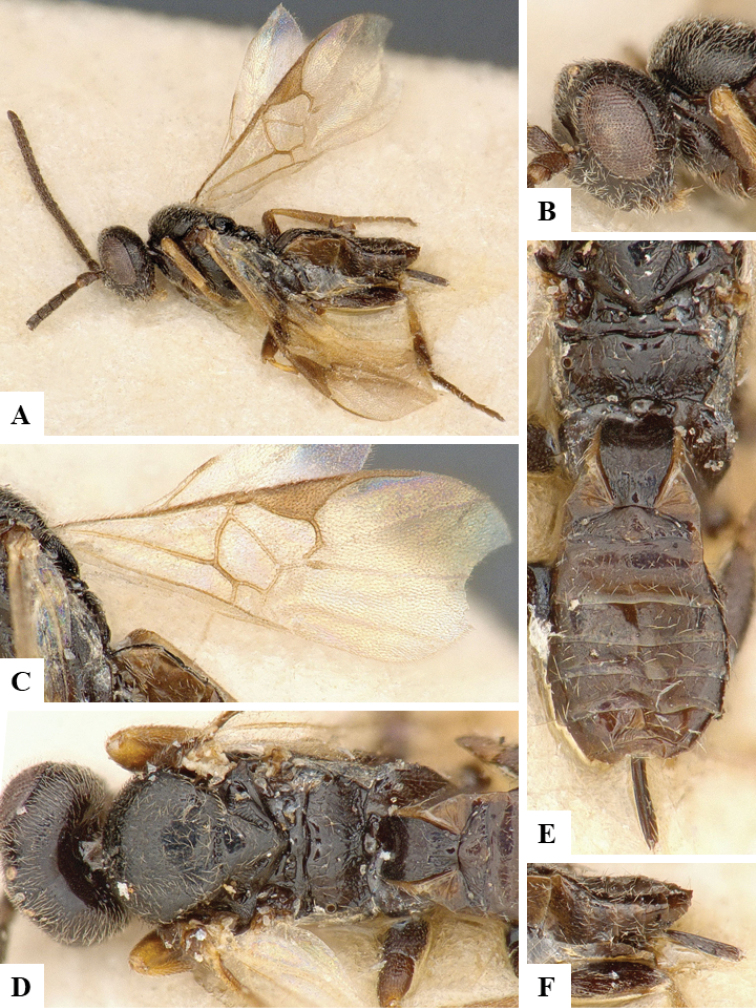
*Apantelesbiroicus* female CNC280546 **A** Habitus, lateral **B** Head, lateral **C** Fore wing **D** Mesosoma and tergite 1, dorsal **E** Propodeum and metasoma, dorsal **F** Metasoma and ovipositor sheaths, lateral.

**Figure 16.**
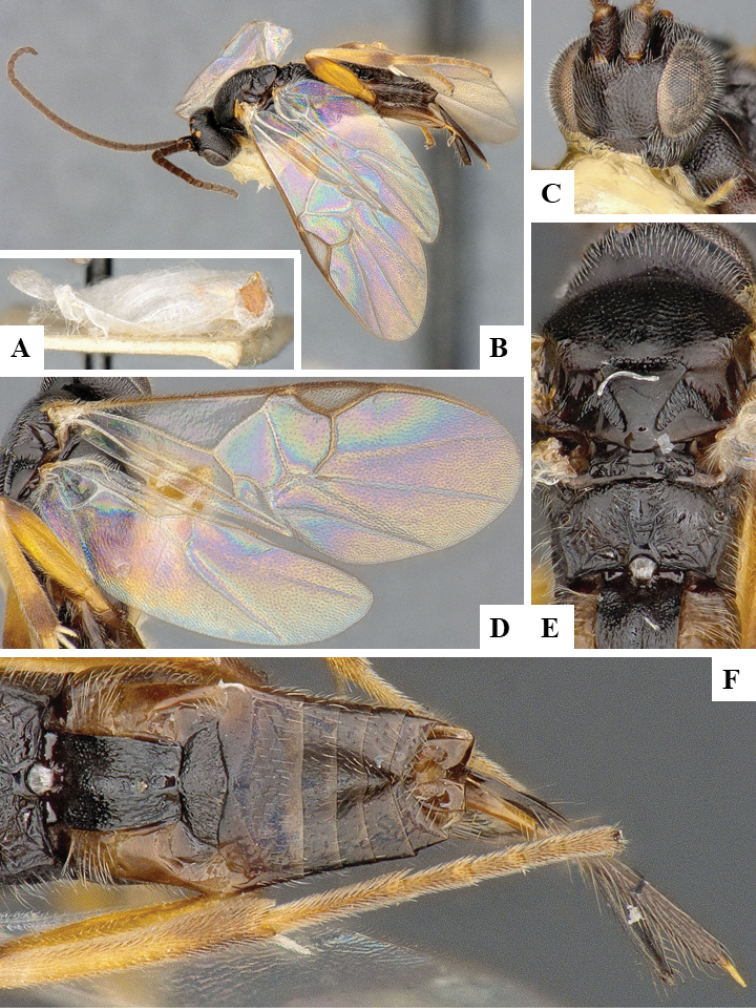
*Apantelescanarsiae* female MIC000030 **A** Cocoon **B** Habitus, lateral **C** Head, frontolateral **D** Fore wing and hind wing **E** Mesosoma and propodeum, dorsal **F** Propodeum and metasoma, dorsal.

**Figure 17.**
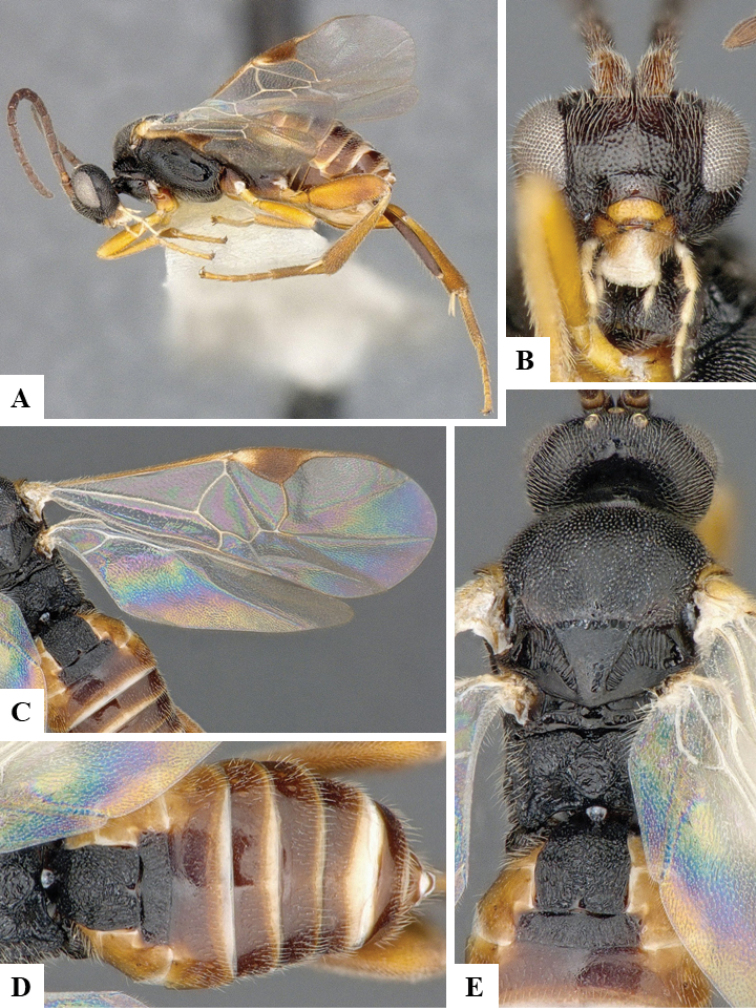
*Apantelescarpatus* female MIC000036 **A** Habitus, lateral **B** Head, frontoventral **C** Fore wing and hind wing **D** Metasoma, dorsal **E** Mesosoma and propodeum, dorsal.

**Figure 18.**
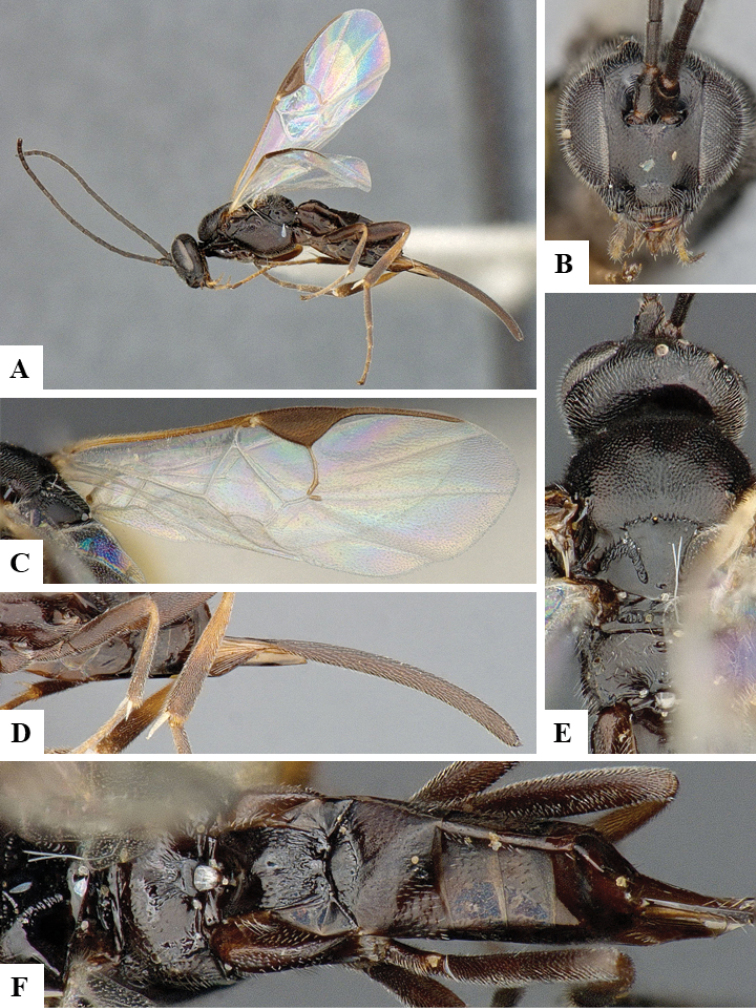
*Apantelescockerelli* female MIC000043 **A** Habitus, lateral **B** Head, frontal **C** Fore wing **D** Hypopygium and ovipositor sheaths, lateral **E** Mesosoma, dorsal **F** Propodeum and metasoma, dorsal.

**Figure 19.**
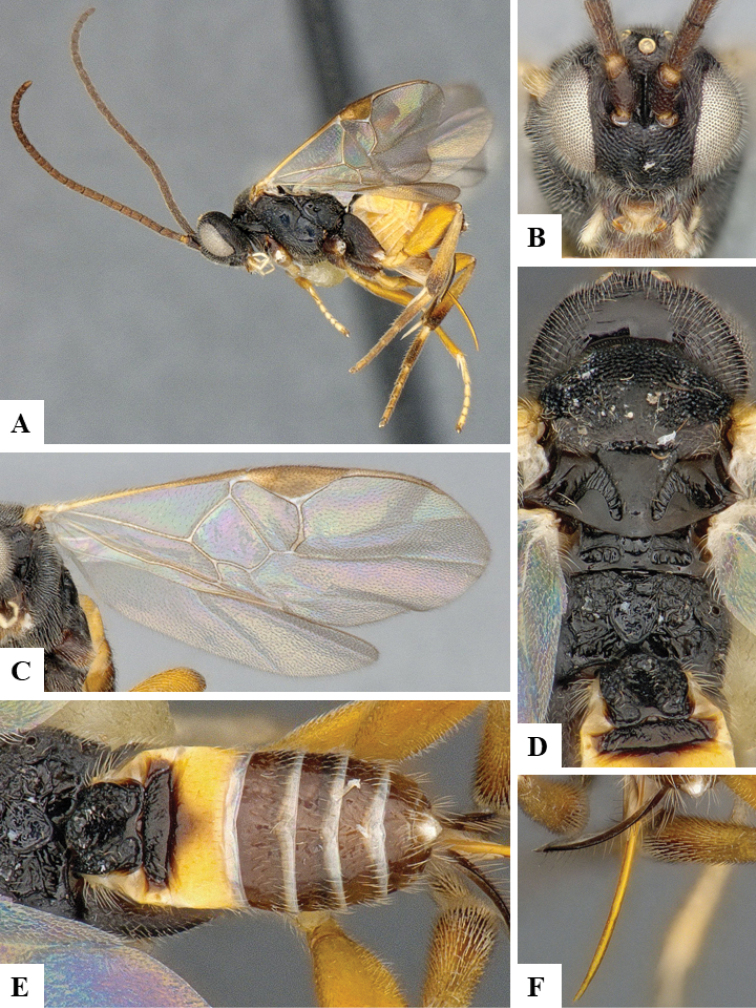
*Apantelesconanchetorum* female MIC000060 **A** Habitus, lateral **B** Head, frontal **C** Fore wing and hind wing **D** Mesosoma and propodeum, dorsal **E** Metasoma, dorsal **F** Ovipositor and ovipositor sheaths.

**Figure 20.**
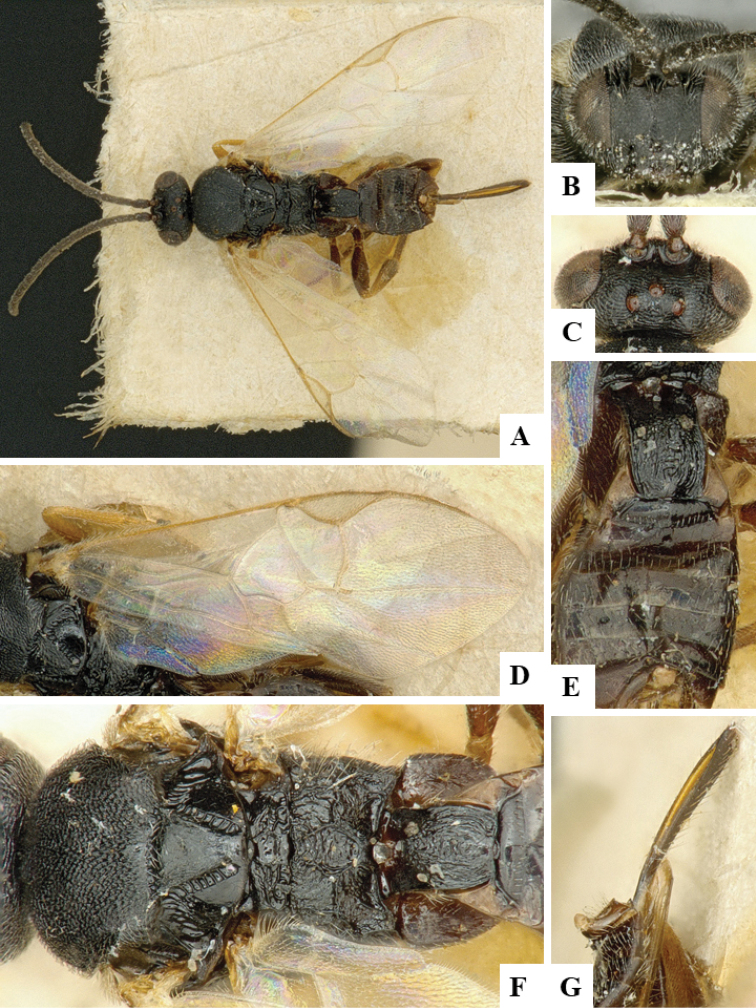
*Apantelesconcordalis* female holotype **A** Habitus, dorsal **B** Head, frontal **C** Head, dorsal **D** Fore wing **E** Metasoma, dorsal **F** Mesosoma and propodeum, dorsal **G** Ovipositor and ovipositor sheaths.

**Figure 21.**
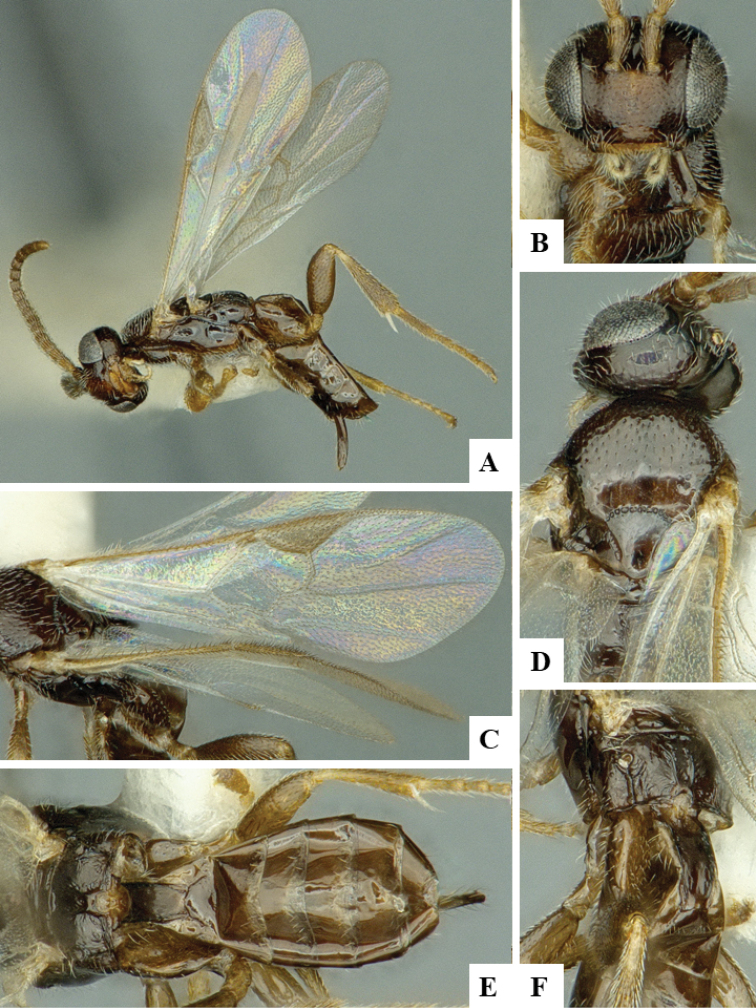
*Apantelesdeplanatus* female CNC280564 **A** Habitus, lateral **B** Head, frontal **C** Fore wing **D** Mesosoma, dorsal **E** Metasoma, dorsal **F** Propodeum, dorsolateral.

**Figure 22.**
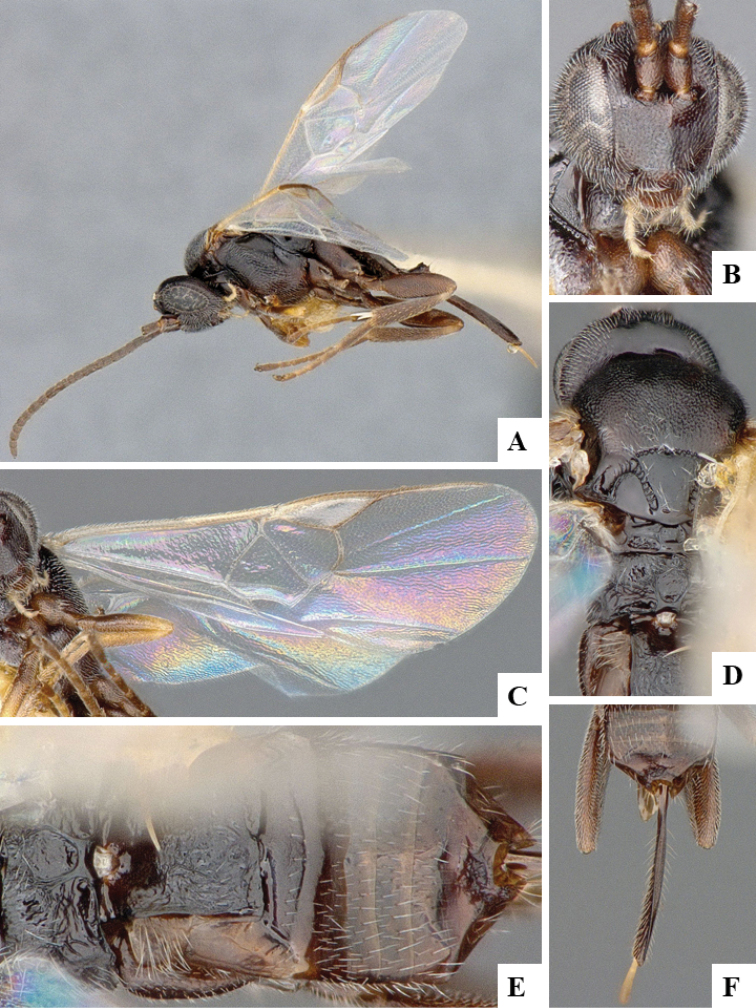
*Apantelesepinotiae* female CNC280581 **A** Habitus, lateral **B** Head, frontal **C** Fore wing and hind wing **D** Mesosoma, dorsal **E** Propodeum and metasoma, dorsal **F** Ovipositor and ovipositor sheaths.

**Figure 23.**
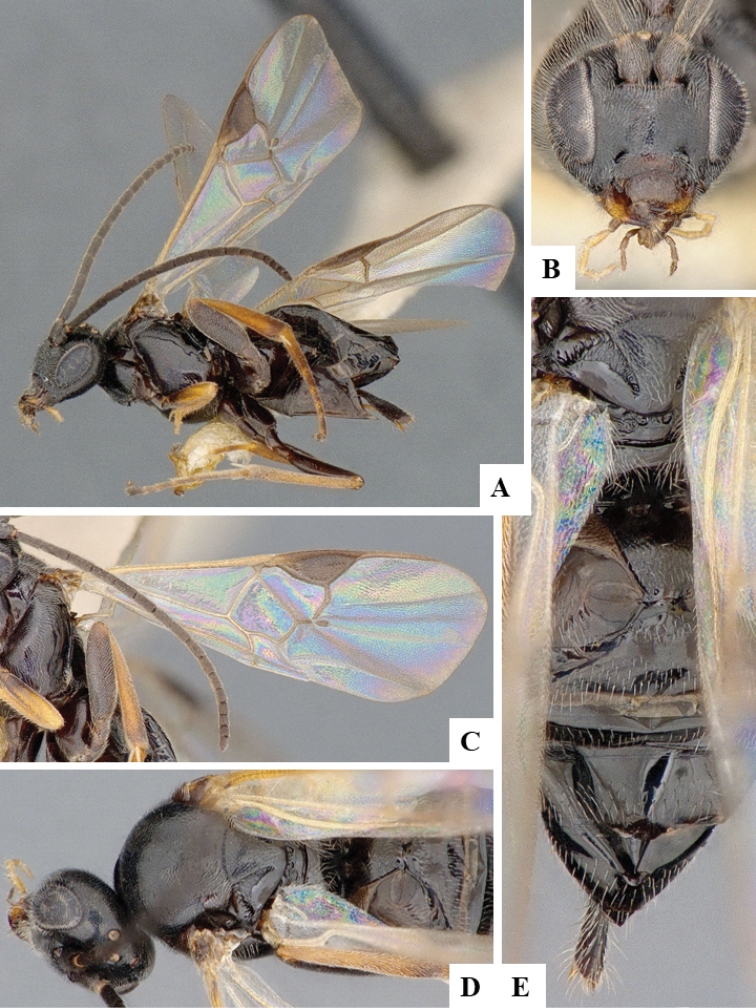
*Apantelesfeltiae* female MIC000097 **A** Habitus, lateral **B** Head, frontal **C** Fore wing **D** Mesosoma, dorsal **E** Metasoma, dorsal.

**Figure 24.**
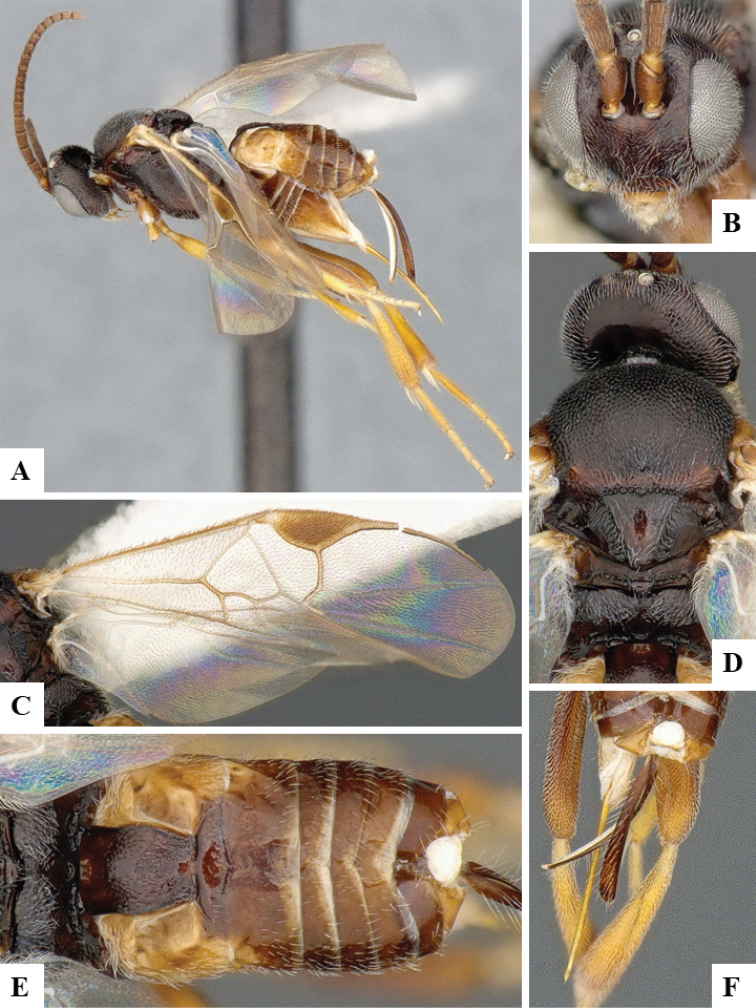
*Apantelesgalleriae* female MIC000116 **A** Habitus, lateral **B** Head, frontal **C** Fore wing and hind wing **D** Mesosoma, dorsal **E** Metasoma, dorsal **F** Ovipositor and ovipositor sheaths.

**Figure 25.**
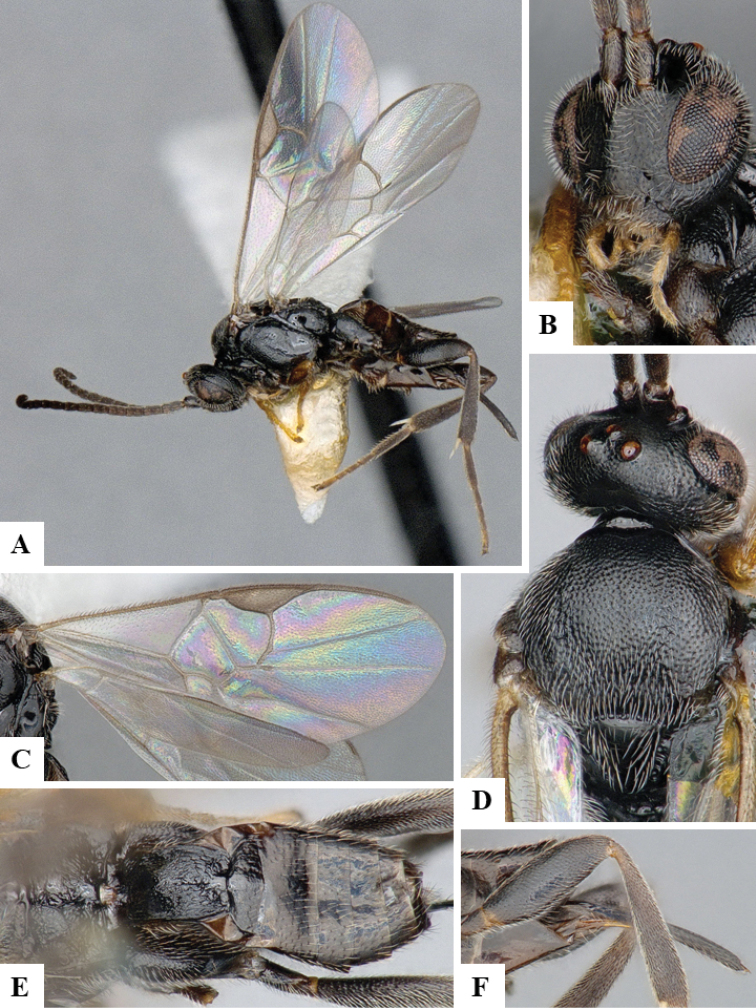
*Apantelessodalis* female MIC000013 **A** Habitus, lateral **B** Head, frontolateral **C** Fore wing and hind wing **D** Mesosoma, dorsal **E** Metasoma, dorsal **F** Ovipositor and ovipositor sheaths.

**Figure 26.**
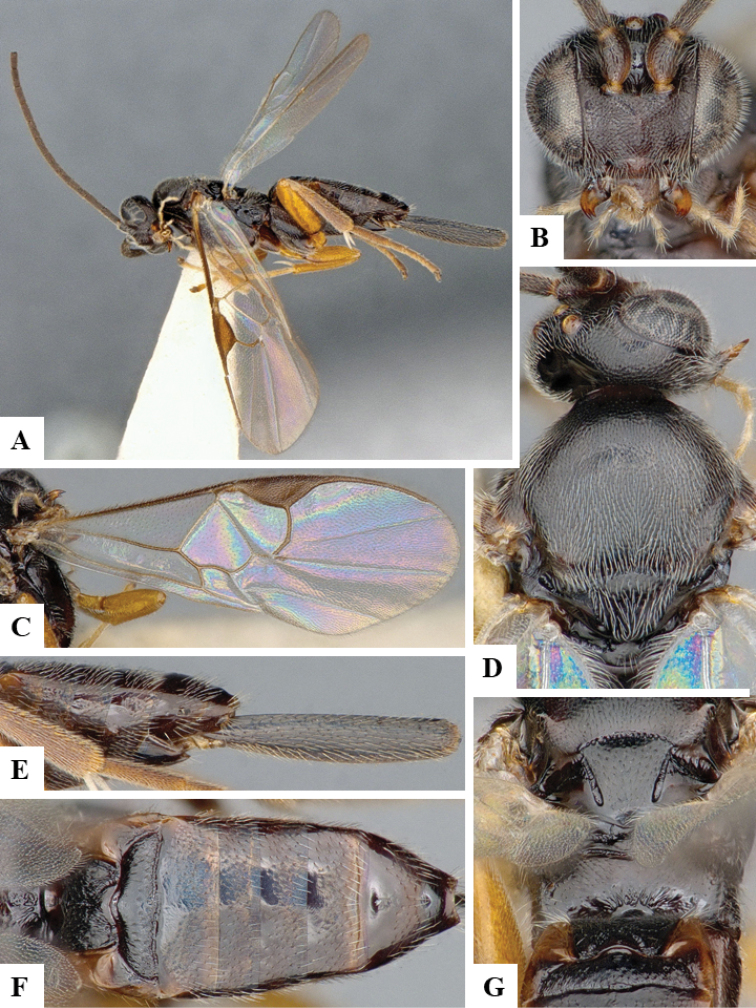
*Apantelesstagmatophorae* female CNCHYM00217 **A** Habitus, lateral **B** Head, frontal **C** Fore wing **D** Mesosoma, dorsal **E** Ovipositor sheaths **F** Metasoma, dorsal **G** Propodeum, dorsal.

**Figure 27.**
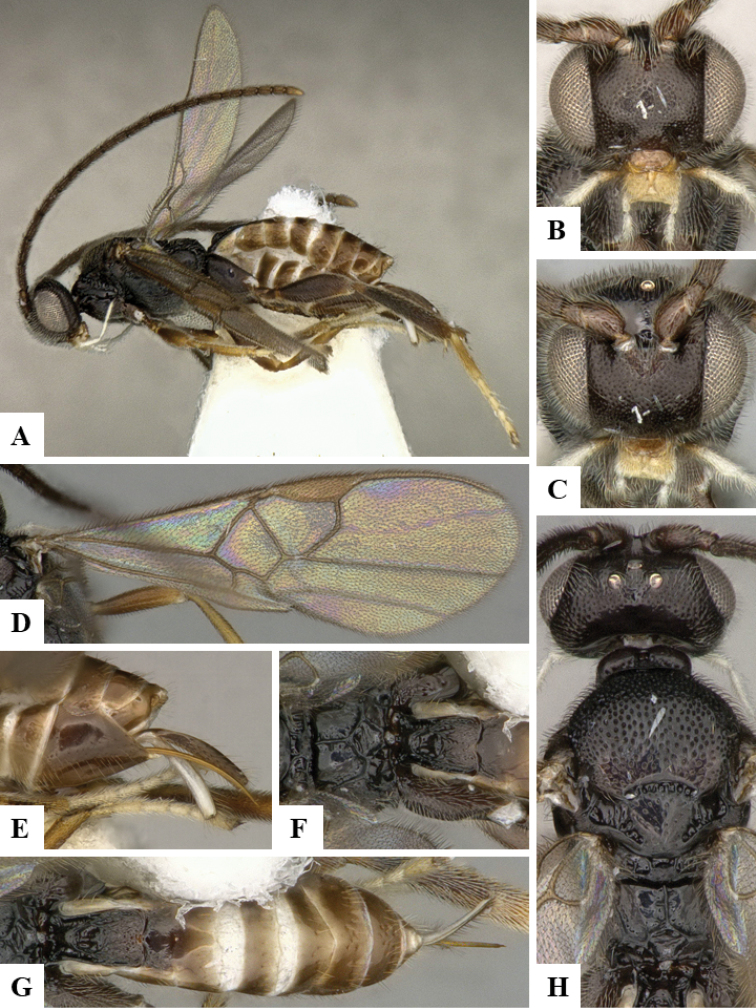
*Austinicotesiaindonesiensis* female holotype **A** Habitus, lateral **B** Head, frontoventral **C** Head, frontal **D** Fore wing **E** Ovipositor and ovipositor sheaths **F** Propodeum and tergite 1, dorsal **G** Metasoma, dorsal; Head and mesosoma, dorsal.

**Figure 28.**
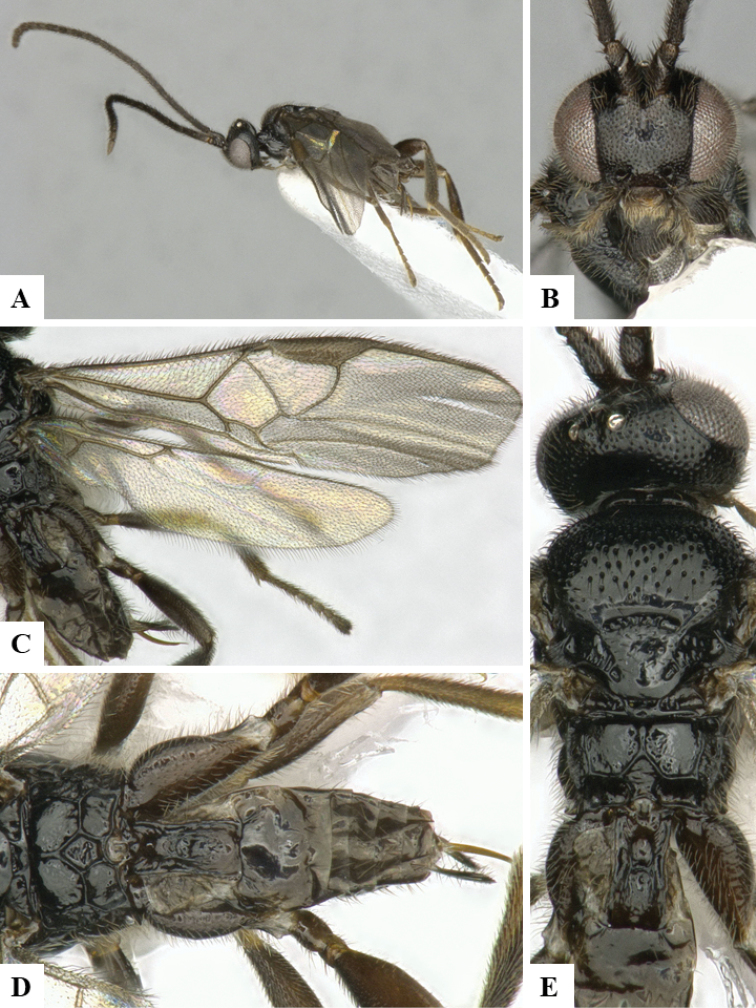
*Austinicotesiapapuanus* female holotype **A** Habitus, lateral **B** Head, frontal **C** Fore wing and hind wing **D** Propodeum and metasoma, dorsal **E** Mesosoma, dorsal.

**Figure 29.**
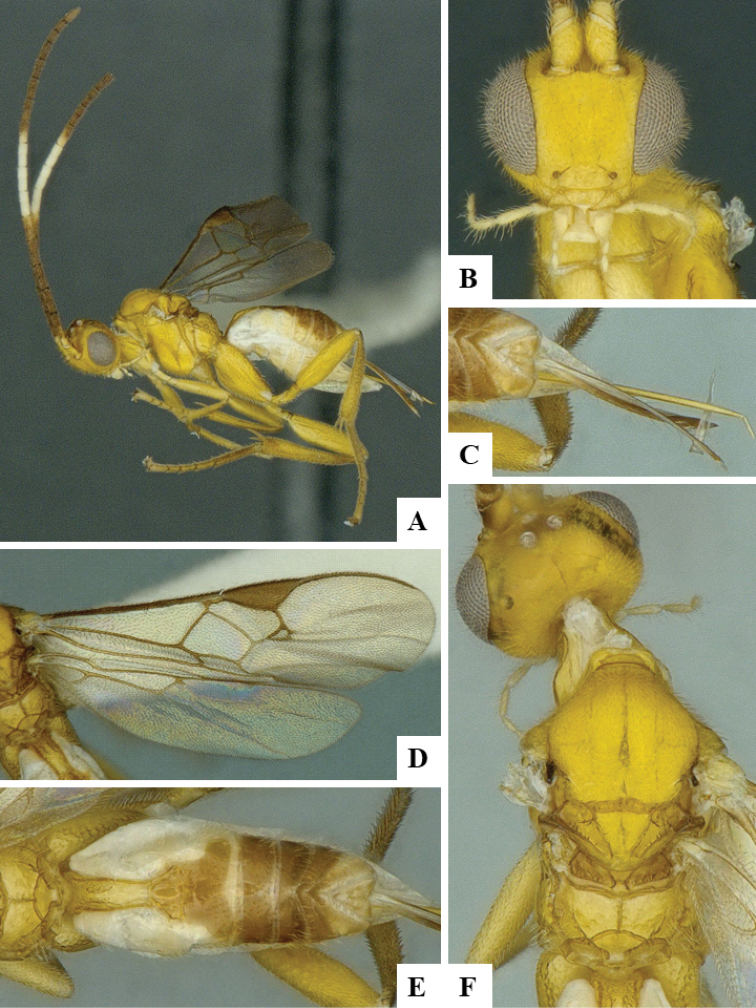
*Austrocotesiacroizati* female CNC280750 **A** Habitus, lateral **B** Head, frontal **C** Ovipositor and ovipositor sheaths **D** Fore wing and hind wing **E** Metasoma, dorsal **F** Head and mesosoma, dorsal.

**Figure 30.**
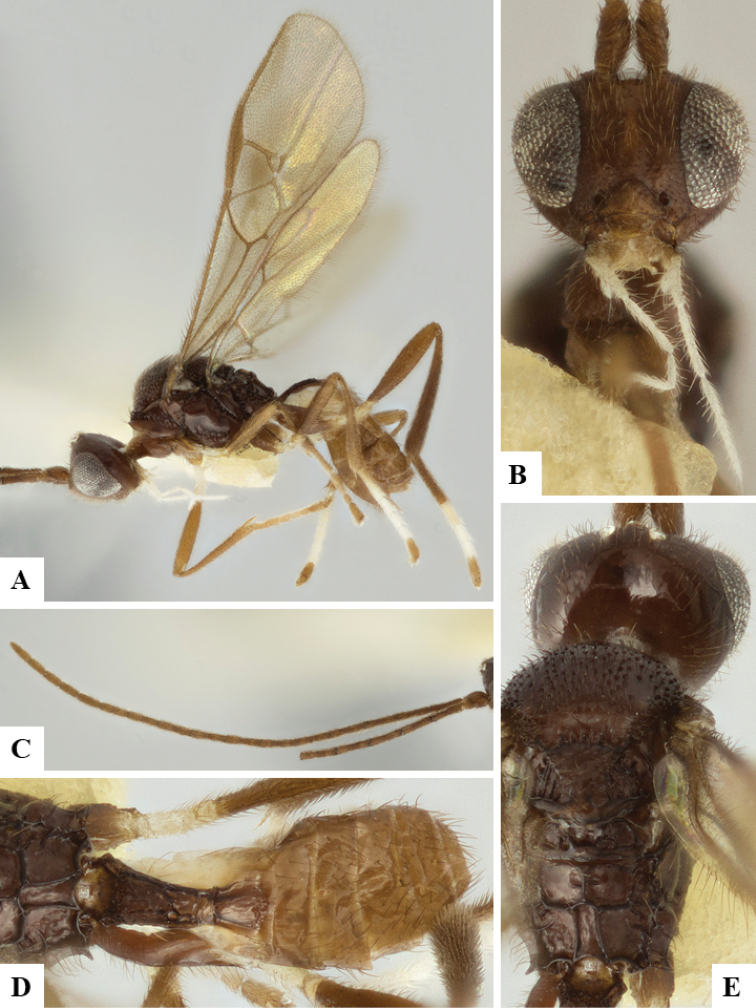
*Austrocotesiadelicata* female holotype **A** Habitus, lateral **B** Head, frontal **C** Antenna **D** Metasoma, dorsal **E** Mesosoma, dorsal.

**Figure 31.**
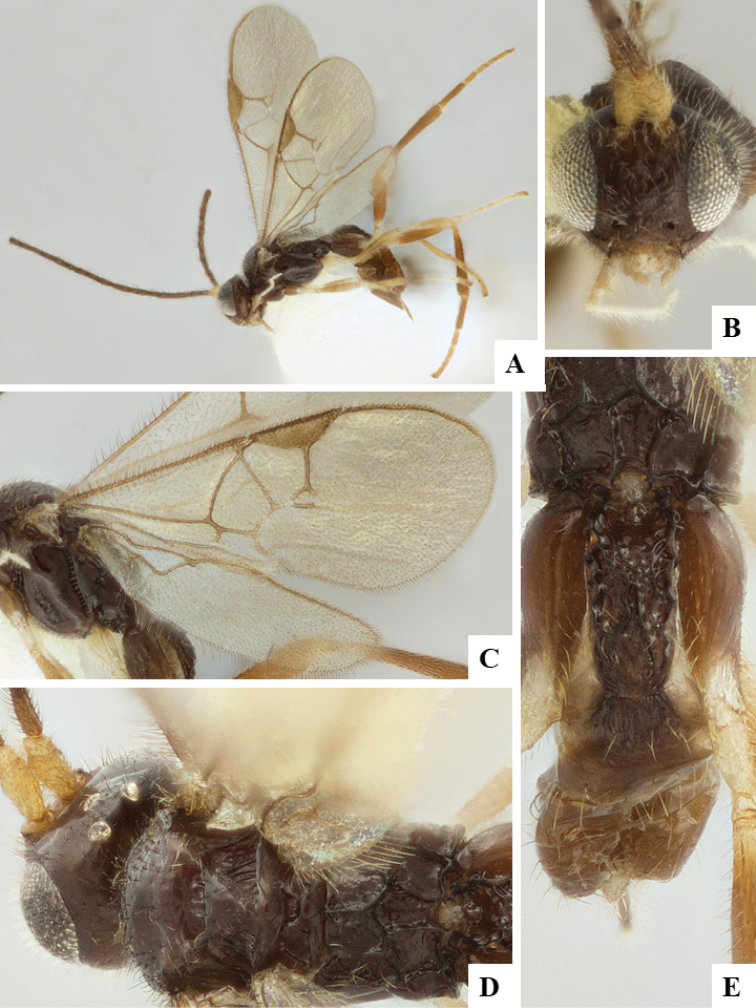
*Austrocotesiaexigua* female holotype **A** Habitus, lateral **B** Head, frontal **C** Fore wing and hind wing **D** Mesosoma, dorsal **E** Metasoma, dorsal.

**Figure 32.**
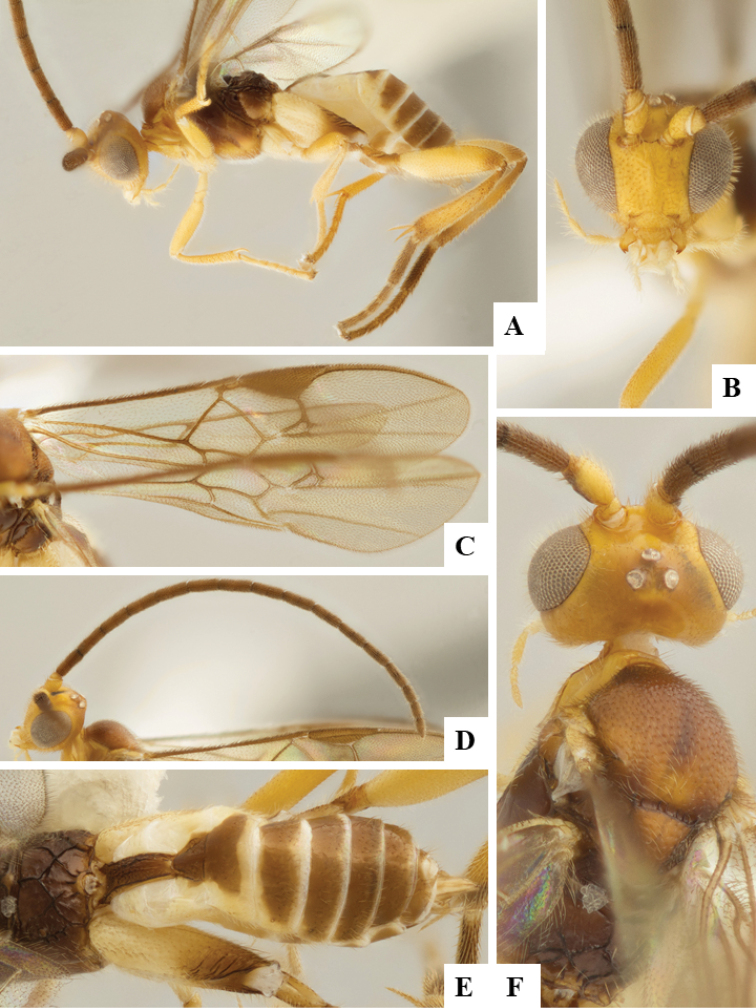
*Austrocotesiarenei* female holotype **A** Habitus, lateral **B** Head, frontal **C** Fore wing **D** Antenna **E** Propodeum and metasoma, dorsal **F** Mesosoma, dorsolateral.

**Figure 33.**
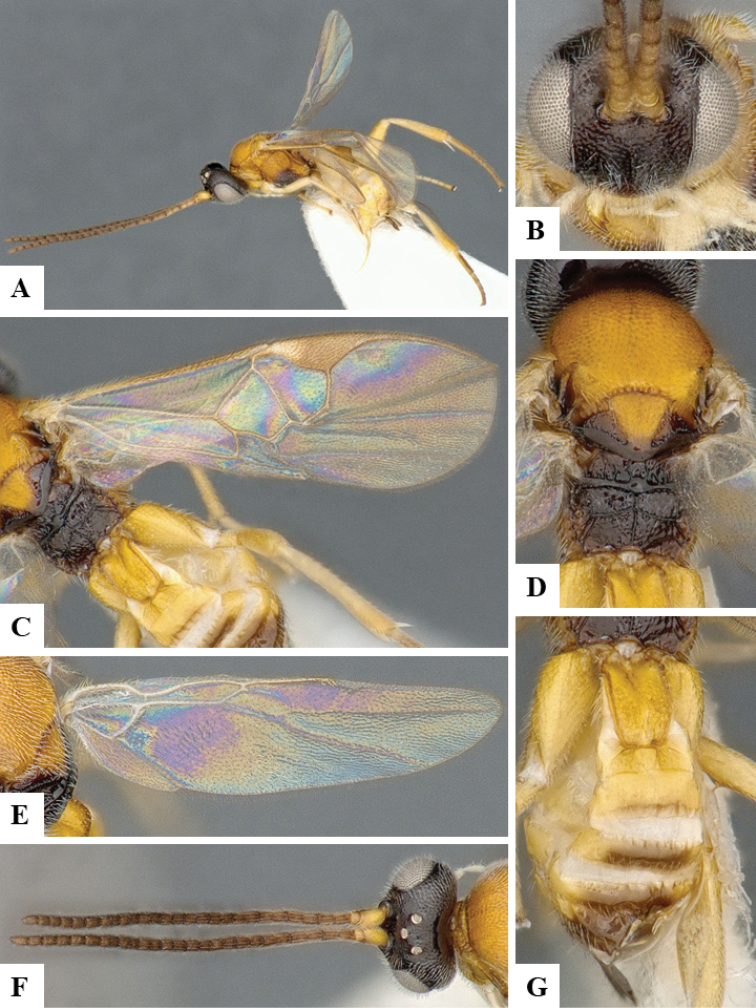
*Beyarslaniainsolens* female WAM 0141 **A** Habitus, lateral **B** Head, frontal **C** Fore wing **D** Mesosoma, dorsal **E** Hind wing **F** Antenna and head, dorsal **G** Mesosoma, dorsal.

**Figure 34.**
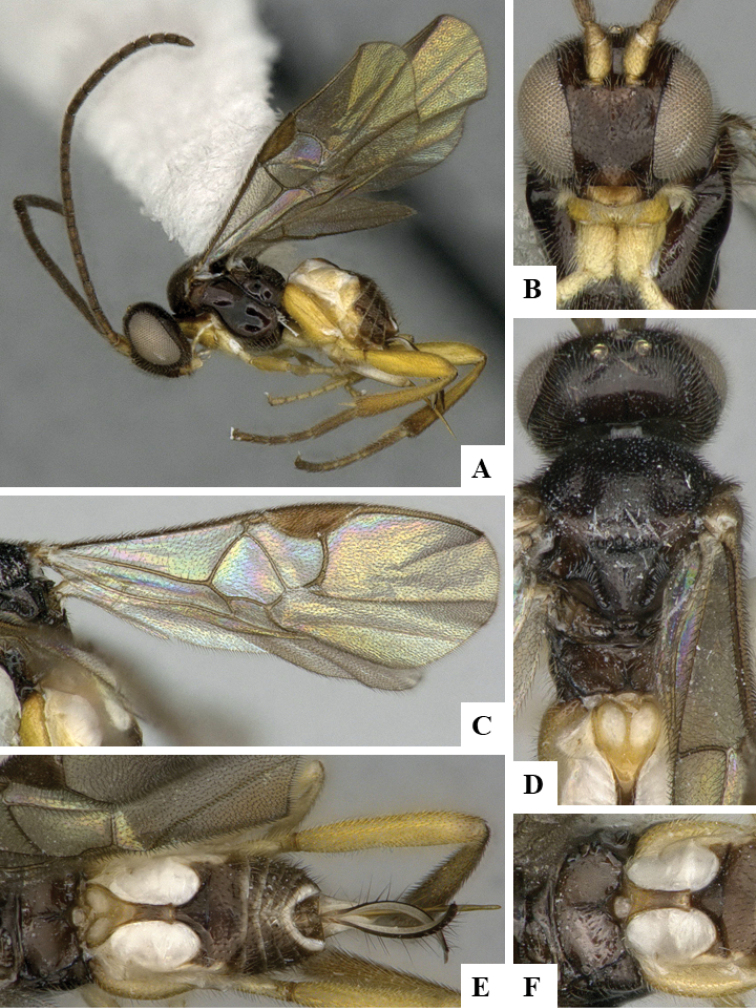
*Billmasoniuscienci* female holotype **A** Habitus, lateral **B** Head, frontal **C** Fore wing **D** Mesosoma, dorsal **E** Metasoma, dorsal **F** Propodeum and tergite 1, dorsal.

**Figure 35.**
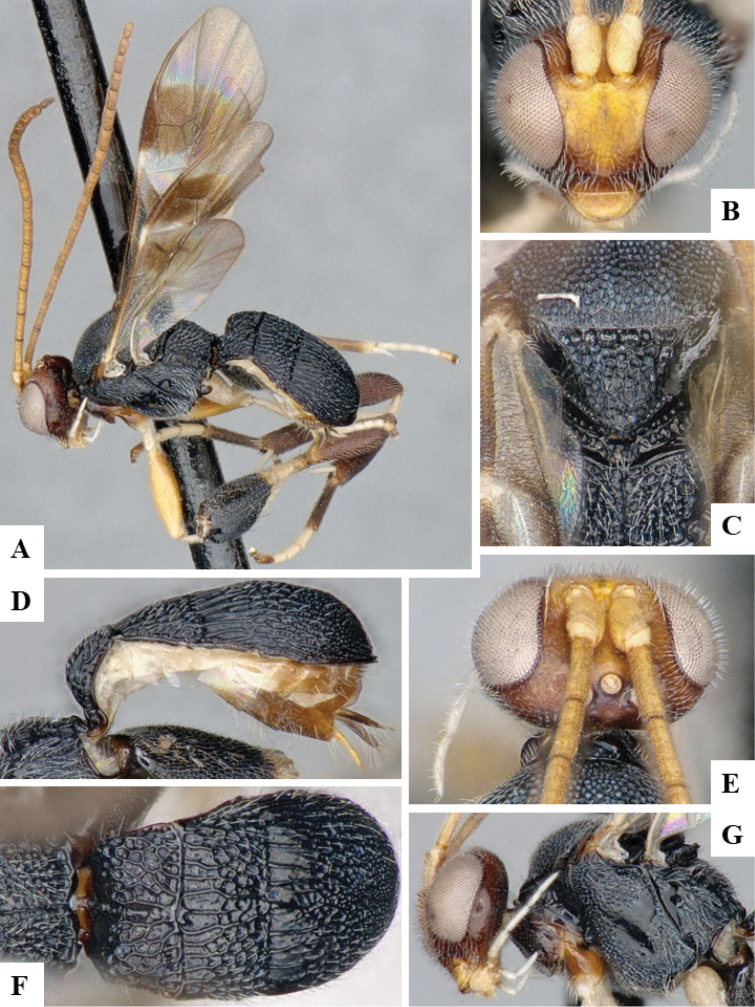
*Bulukaachterbergi* female paratype CNCHYM00244 **A** Habitus, lateral **B** Head, frontal **C** Mesosoma, dorsal **D** Metasoma, lateral **E** Head, dorsal **F** Metasoma, dorsal **G** Mesosoma, lateral.

**Figure 36.**
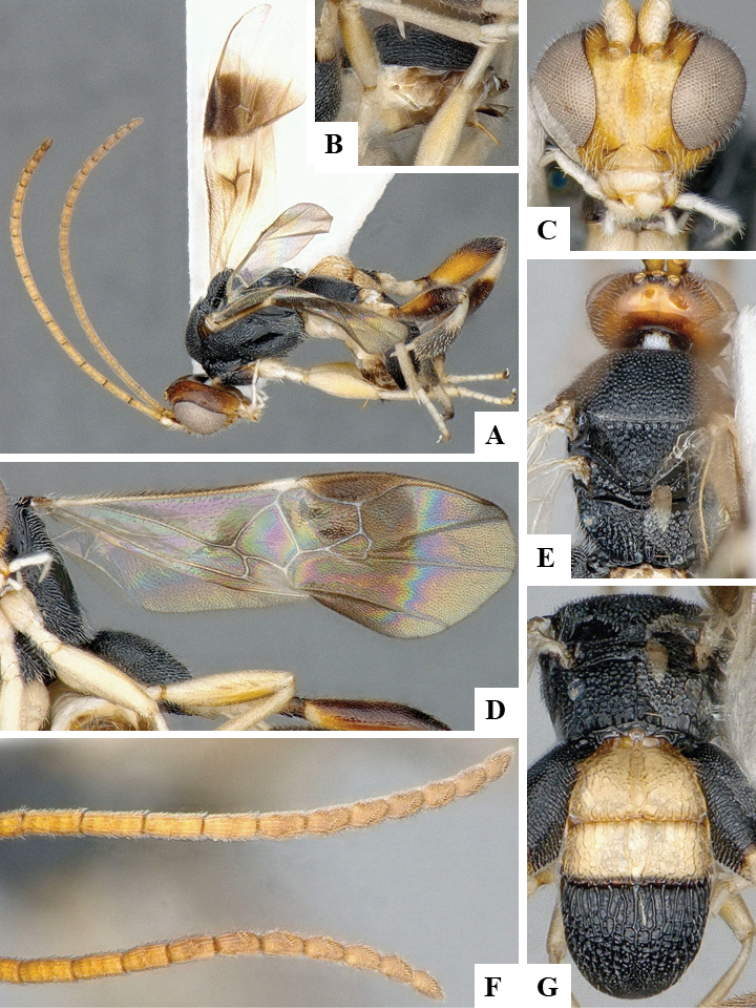
*Buluka* sp. female CNC281638 **A** Habitus, lateral **B** Ovipositor and ovipositor sheaths **C** Head, frontal **D** Fore wing **E** Mesosoma, dorsal **F** Apex of antennae **F** Metasoma and propodeum, dorsal.

**Figure 37.**
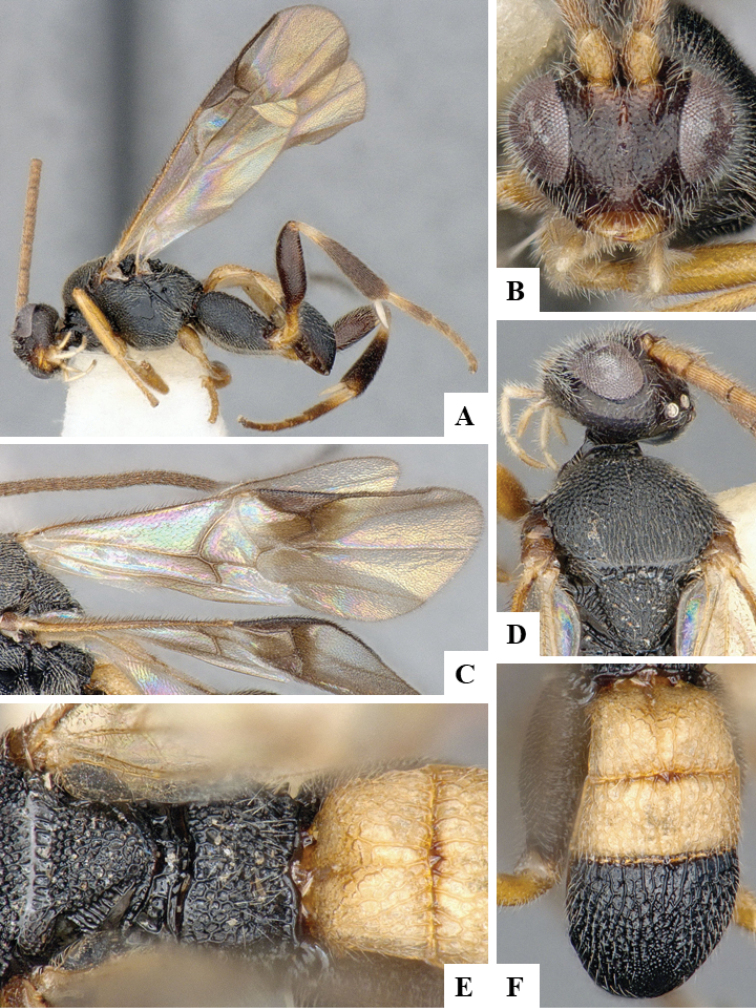
*Bulukastraeleni* male CNCHYM00245 **A** Habitus, lateral **B** Head, frontal **C** Fore wing **D** Mesosoma, dorsal **E** Propodeum and tergite 1, dorsal **F** Metasoma, dorsal.

**Figure 38.**
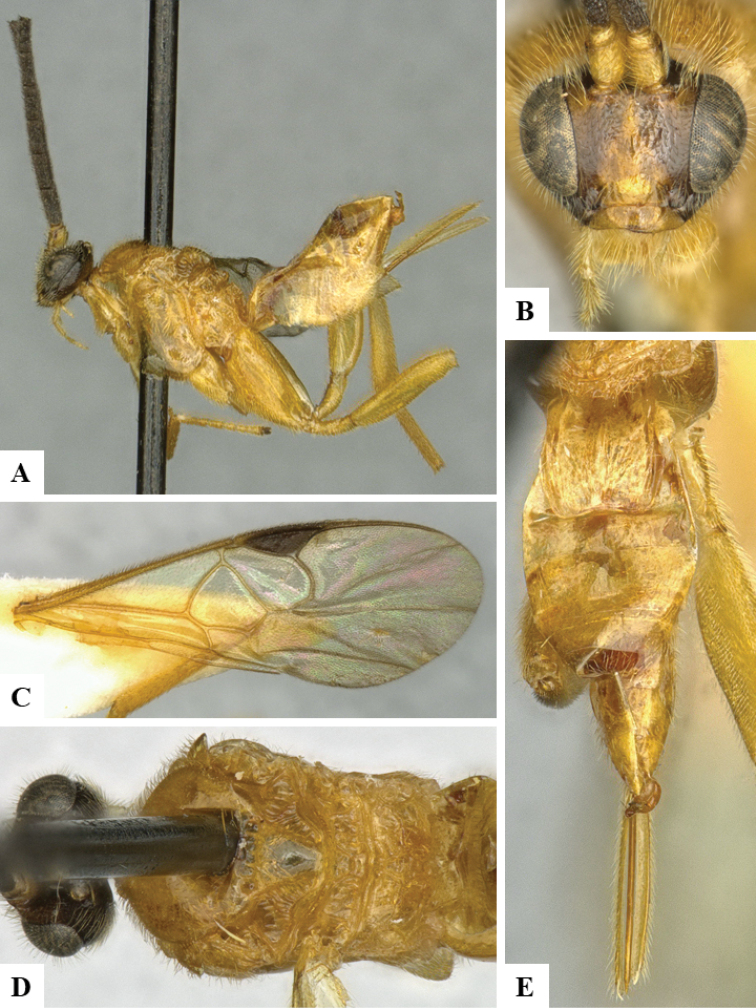
*Carlmuesebeckiussmithsonian* female holotype **A** Habitus, lateral **B** Head, frontal **C** Fore wing **D** Head and mesosoma, dorsal **E** Metasoma, dorsal.

**Figure 39.**
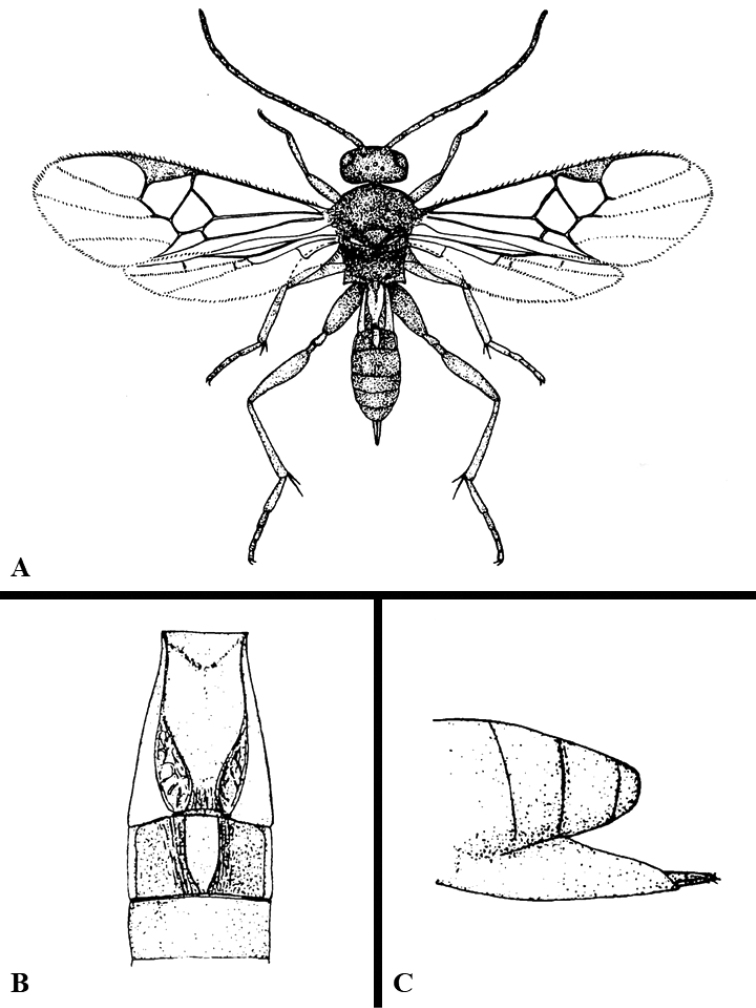
*Chaoaflavipes* female holotype based on modified drawings from the original descriptions of the species (Luo, You & Xiao, 2004) **A** Habitus, dorsal **B** Tergites 1–3, dorsal **C** Apex of metasoma, lateral.

**Figure 40.**
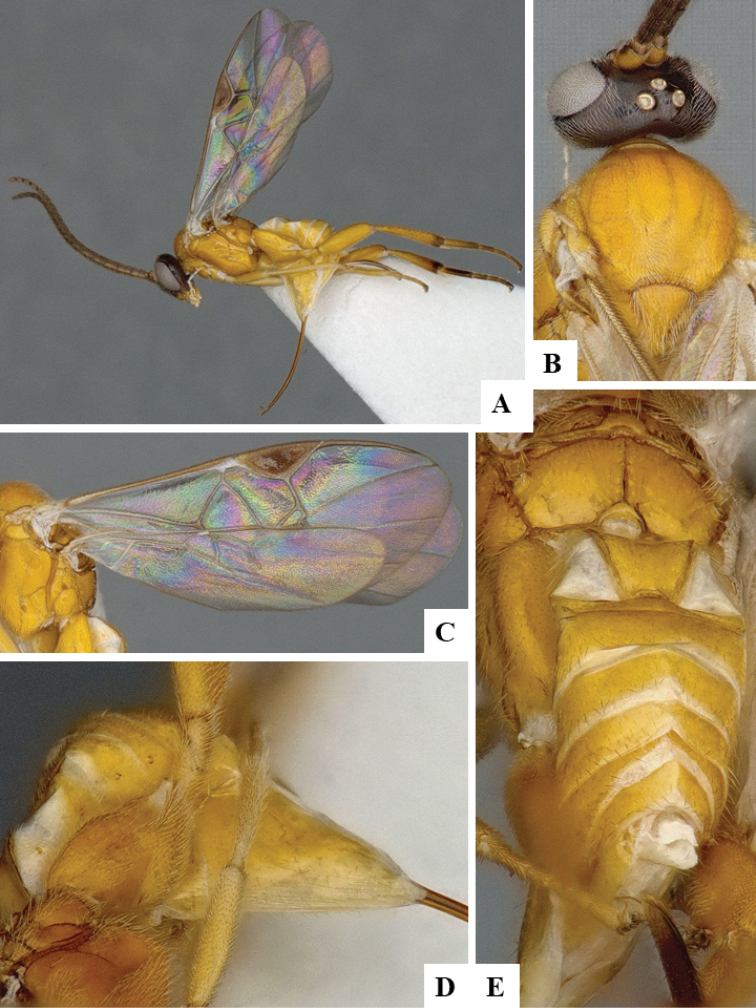
*Choerasafrotropicalis* female holotype **A** Habitus, lateral **B** Head and mesosoma, dorsal **C** Fore wing and hind wing **D** Metasoma, lateral **E** Propodeum and metasoma, dorsolateral.

**Figure 41.**
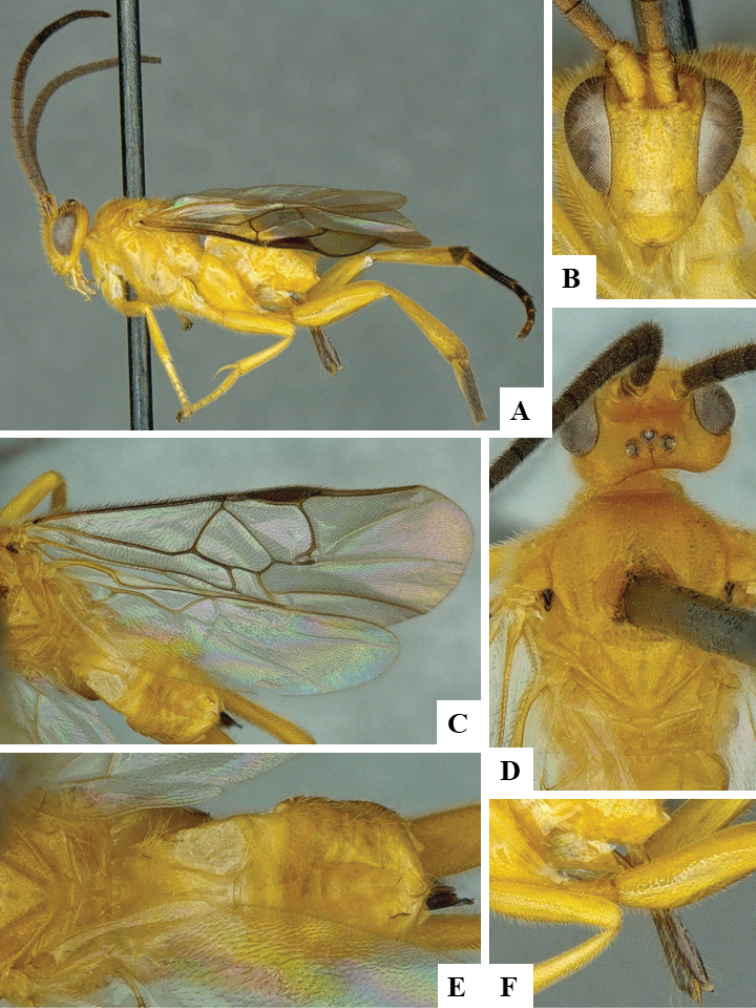
*Choerasapo* female CNC280754 **A** Habitus, lateral **B** Head, frontal **C** Fore wing and hind wing **D** Head and mesosoma, dorsal **E** Metasoma, dorsal **F** Ovipositor and ovipositor sheaths.

**Figure 42.**
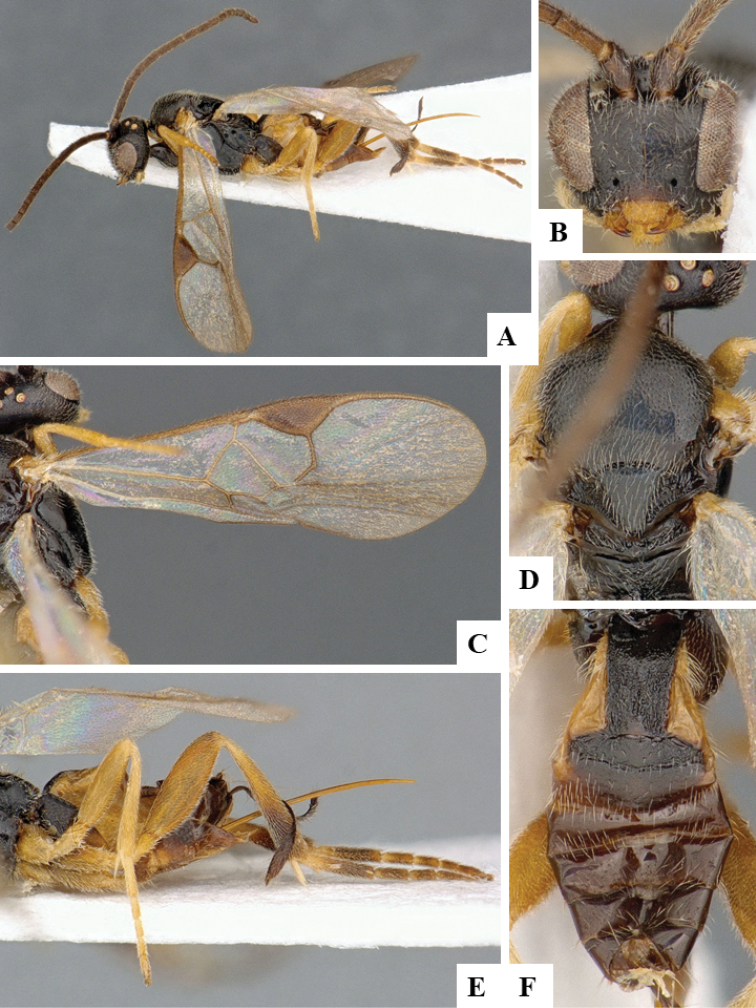
*Choerasparasitellae* female CNC474678 **A** Habitus, lateral **B** Head, frontal **C** Fore wing **D** Mesosoma, dorsal **E** Metasoma, lateral **F** Metasoma, dorsal.

**Figure 43.**
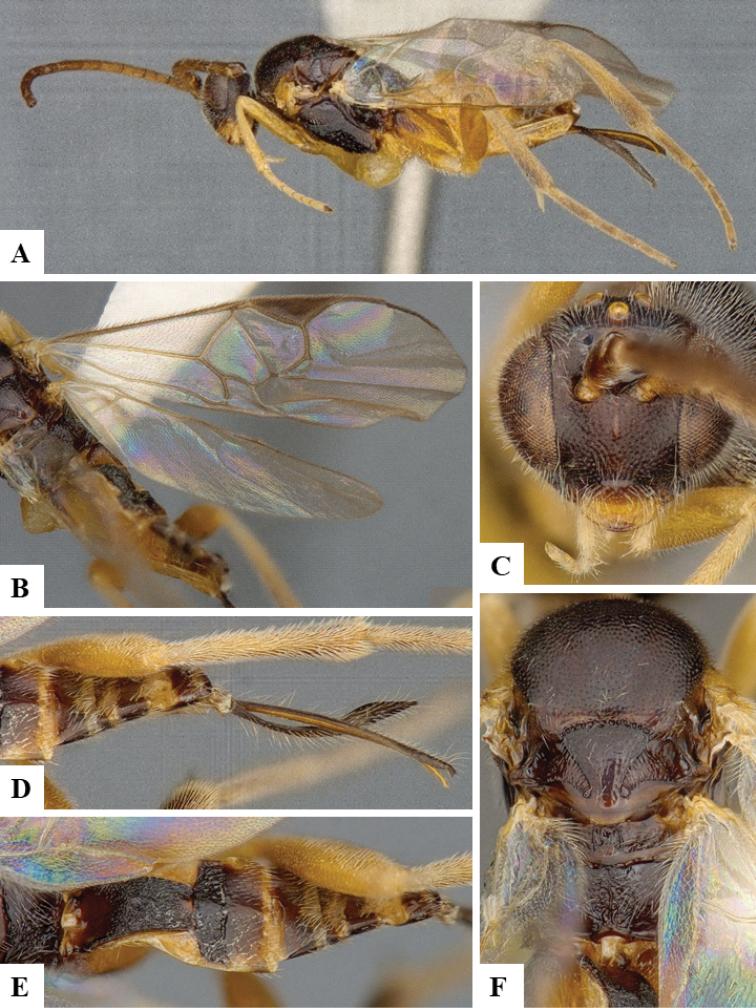
*Choerasruficornis* female CNC280764 **A** Habitus, lateral **B** Fore wing and hind wing **C** Head, frontal **D** Ovipositor and ovipositor sheaths **E** Metasoma, dorsal **F** Mesosoma, dorsal.

**Figure 44.**
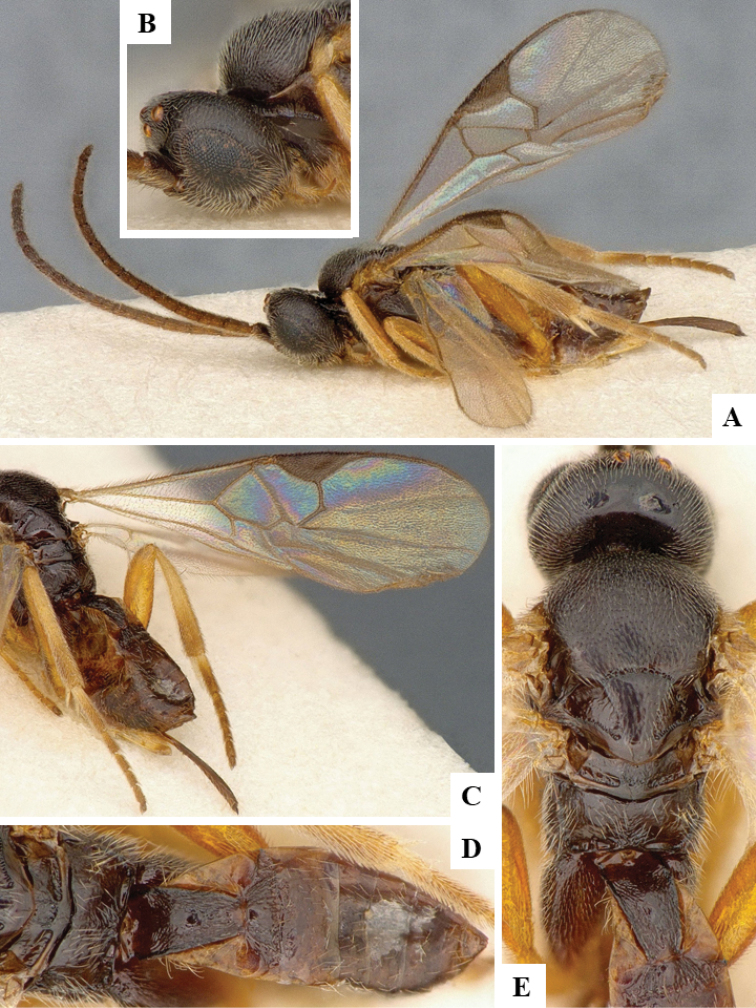
*Choerastedellae* female CNC474677 **A** Habitus, lateral **B** Head, lateral **C** Fore wing **D** Propodeum and metasoma, dorsal **E** Head and mesosoma, dorsal.

**Figure 45.**
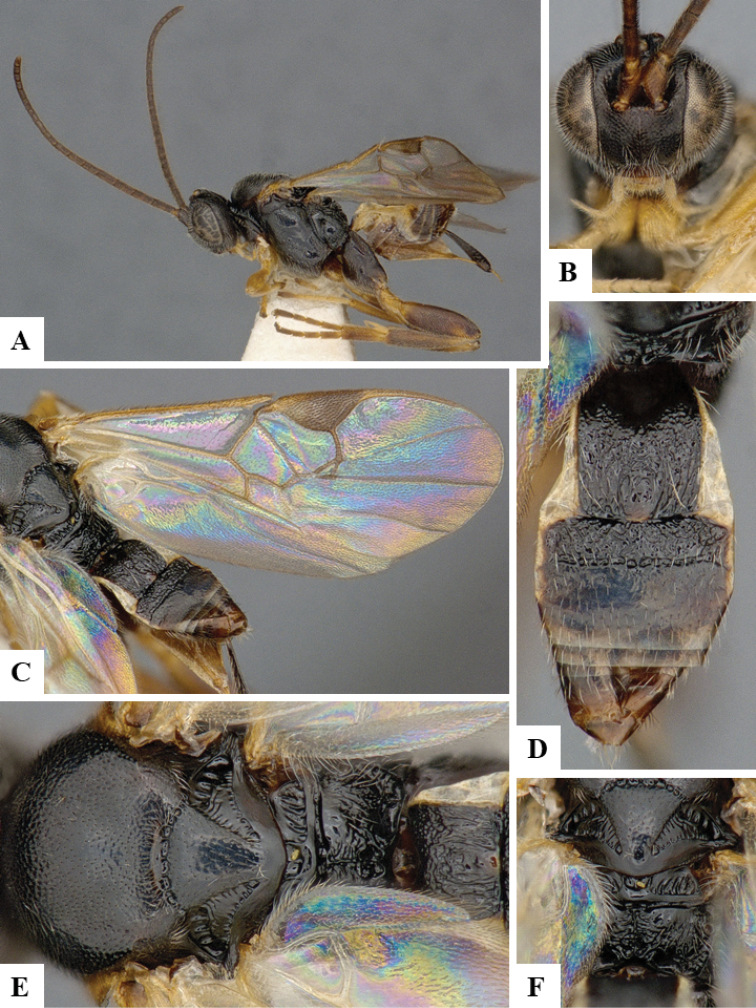
*Choerastiro* female CNC474677 **A** Habitus, lateral **B** Head, frontal **C** Fore wing and hind wing **D** Metasoma, dorsal **E** Mesosoma, dorsal **F** Propodeum, dorsal.

**Figure 46.**
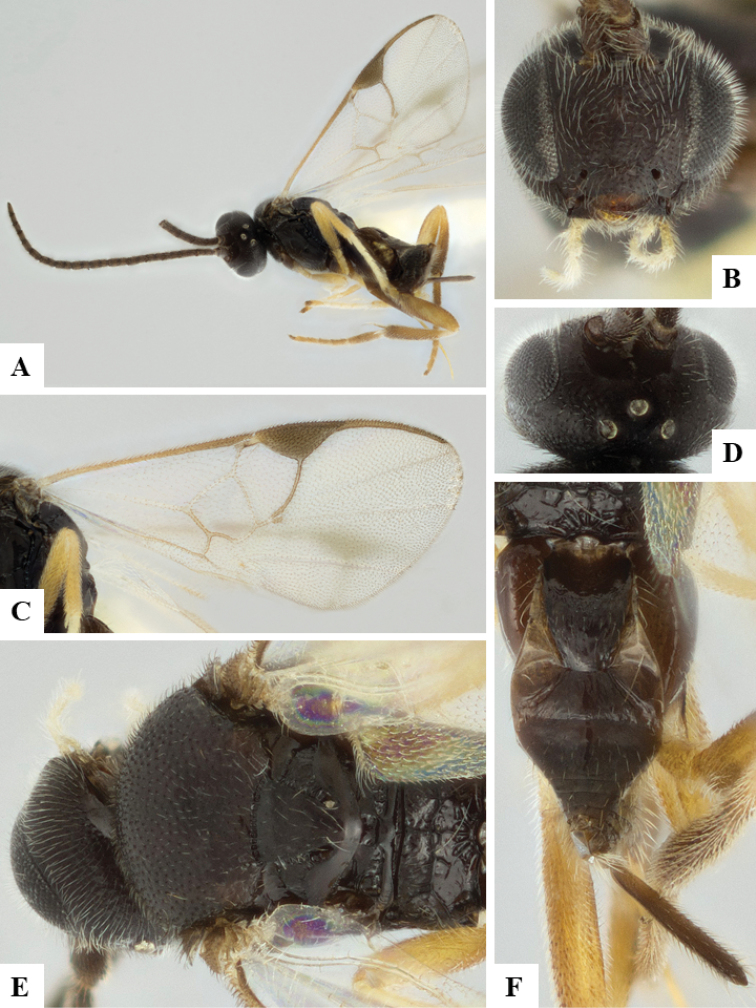
*Clarkinellacanadensis* female holotype **A** Habitus, lateral **B** Head, frontal **C** Fore wing **D** Head, dorsal **E** Mesosoma, dorsal **F** Propodeum and metasoma, dorsal.

**Figure 47.**
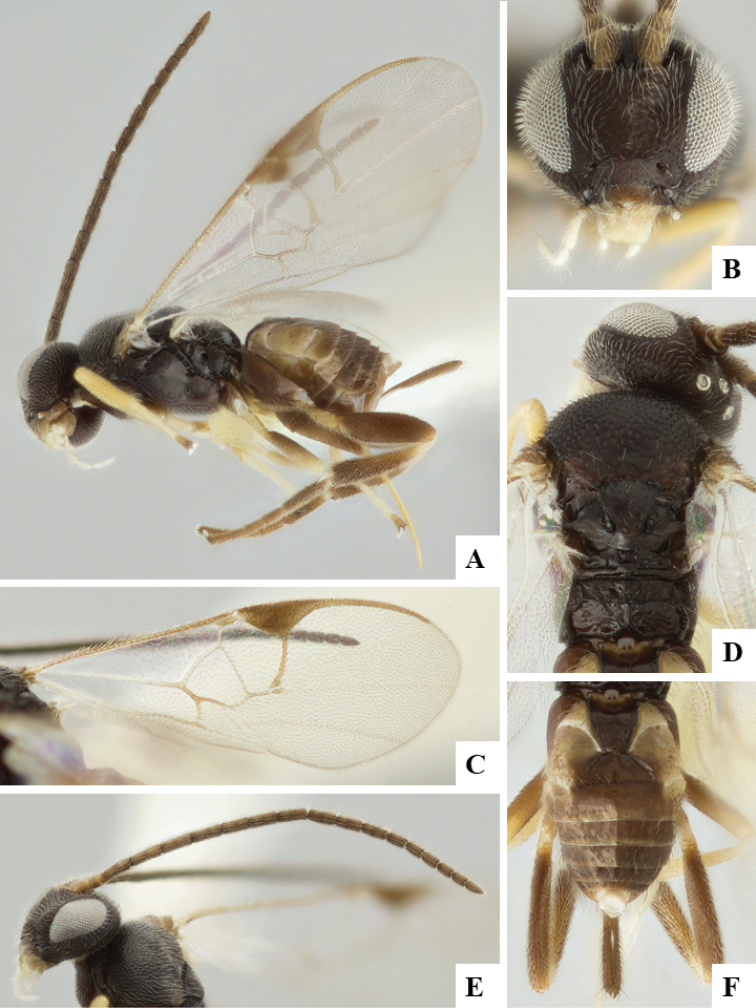
*Clarkinellaedithae* female holotype **A** Habitus, lateral **B** Head, frontal **C** Fore wing **D** Mesosoma, dorsal **E** Head and antenna, lateral **F** Metasoma, dorsal.

**Figure 48.**
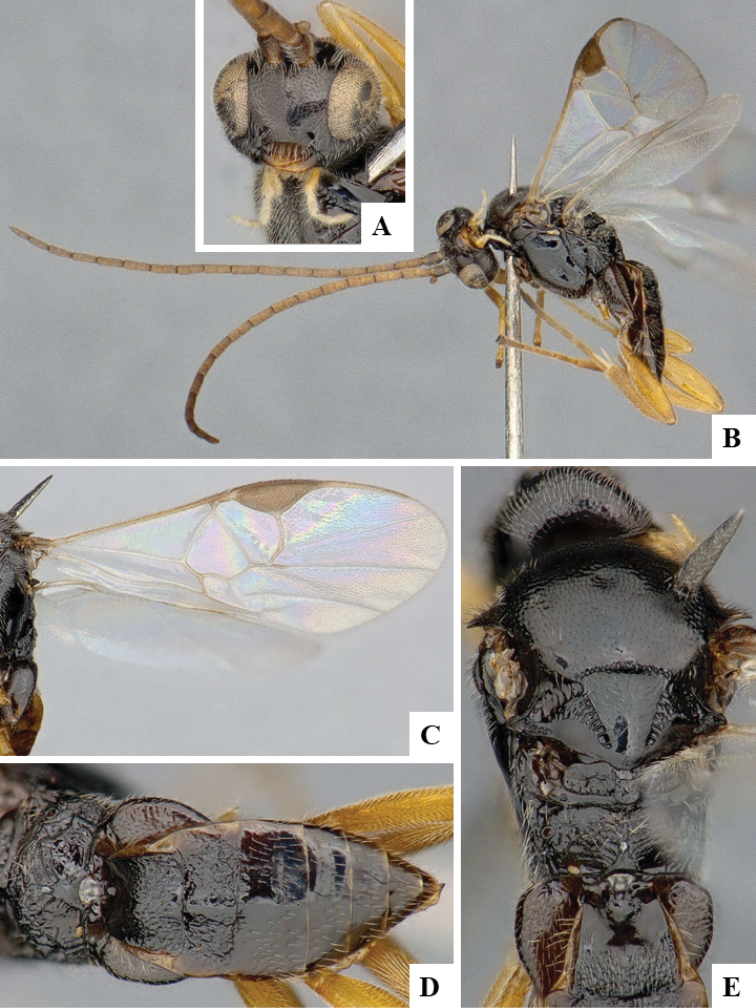
*Cotesiaaffinis* male CNCHYM00340 **A** Head frontal **B** Habitus, lateral **C** Fore wing **D** Metasoma, dorsal **E** Mesosoma and tergite 1, dorsal.

**Figure 49.**
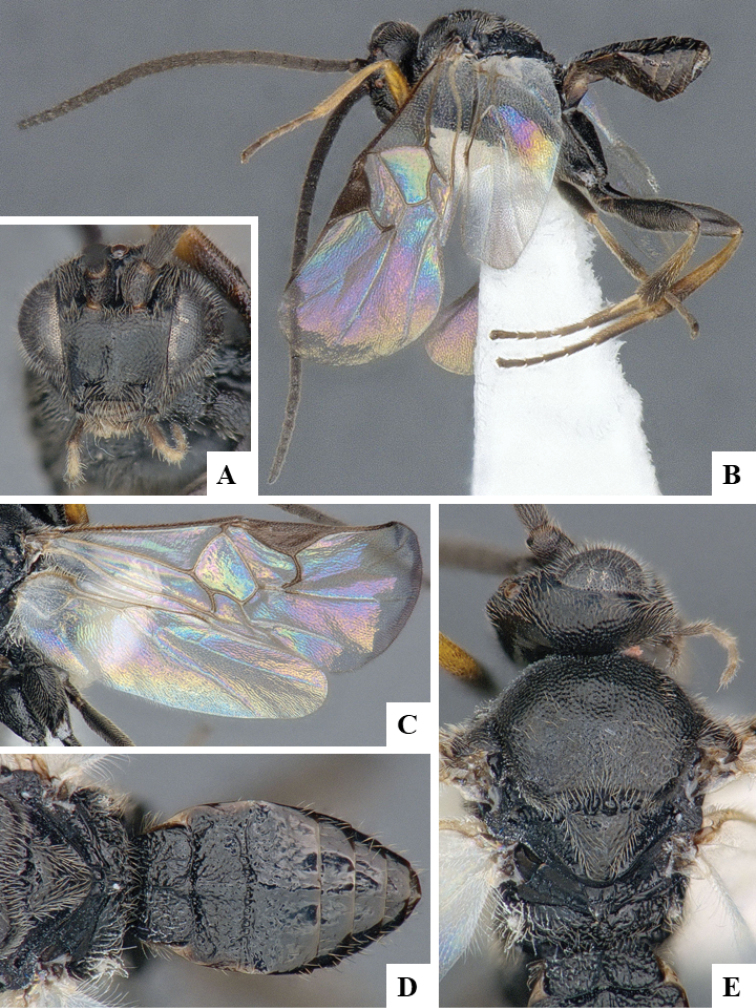
*Cotesiaancilla* male CNC735671 **A** Head, frontal **B** Habitus, lateral **C** Fore wing and hind wing **D** Metasoma, dorsal **E** Mesosoma, dorsal.

**Figure 50.**
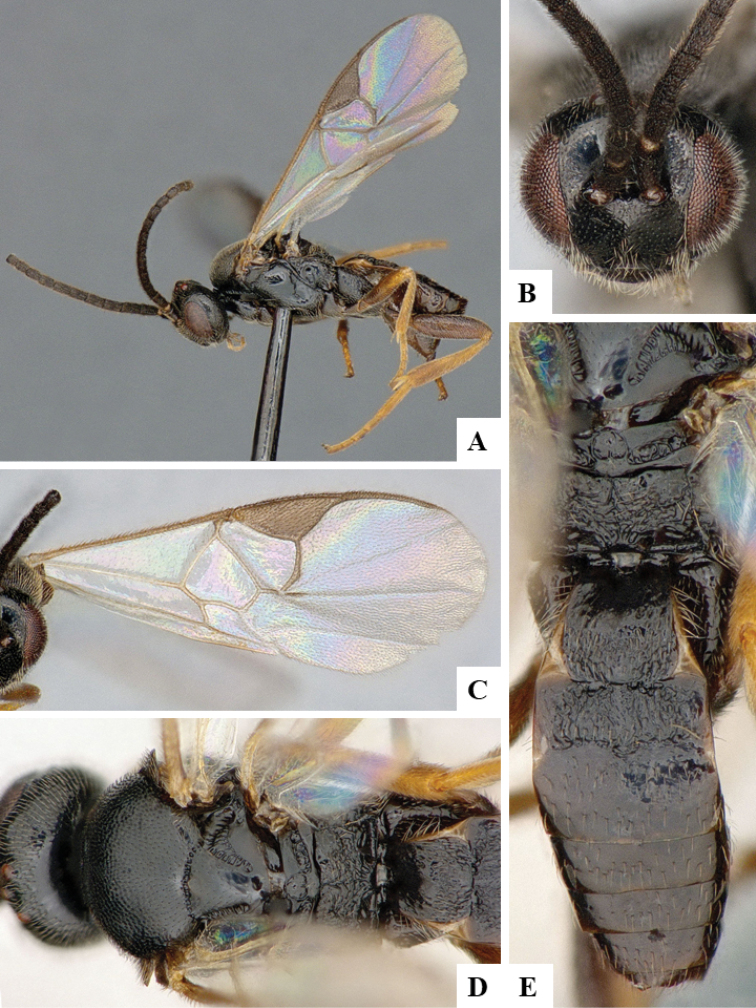
*Cotesiaeulipis* female CNC474686 **A** Habitus, lateral **B** Head, frontal **C** Fore wing **D** Mesosoma, dorsal **E** Propodeum and metasoma, dorsal.

**Figure 51.**
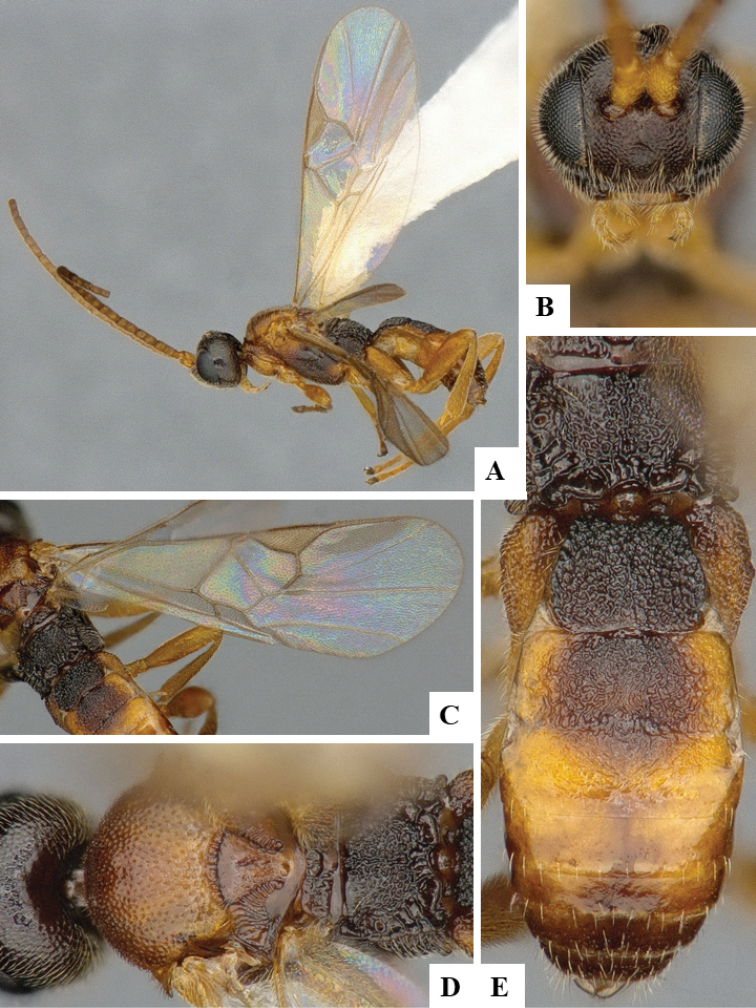
*Cotesiaferruginea* male CNCHYM00455 **A** Habitus, lateral **B** Head, frontal **C** Fore wing **D** Mesosoma, dorsal **E** Propodeum and metasoma, dorsal.

**Figure 52.**
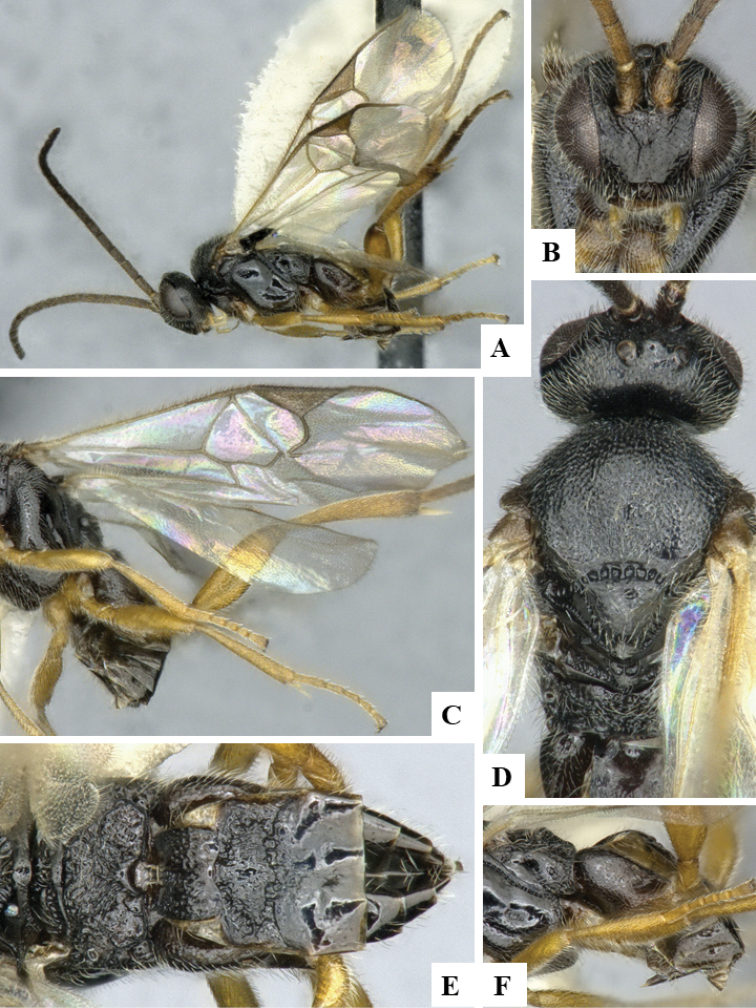
*Cotesiaglomerata* female CNC722382 **A** Habitus, lateral **B** Head, frontal **C** Fore wing and hind wing **D** Head and mesosoma, dorsal **E** Propodeum and metasoma, dorsal **F** Ovipositor and ovipositor sheaths.

**Figure 53.**
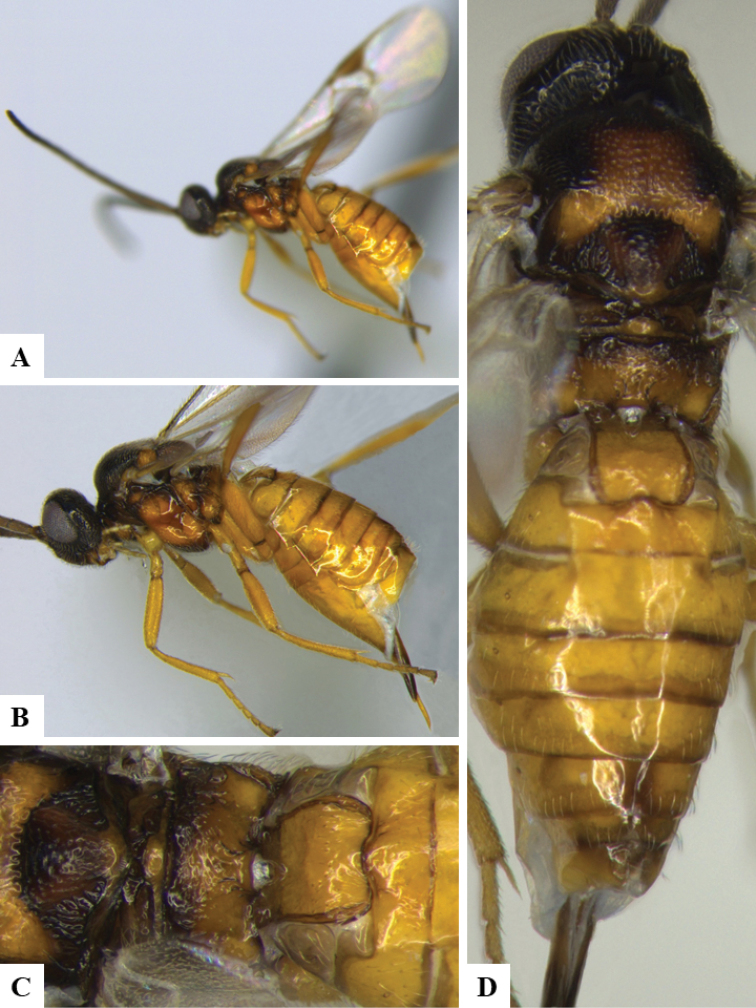
*Cotesiahispanica* female, specimen without voucher code **A** Habitus, lateral **B** Habitus, magnified **C** Propodeum and tergite 1–2, dorsal **D** Mesosoma and metasoma, dorsal.

**Figure 54.**
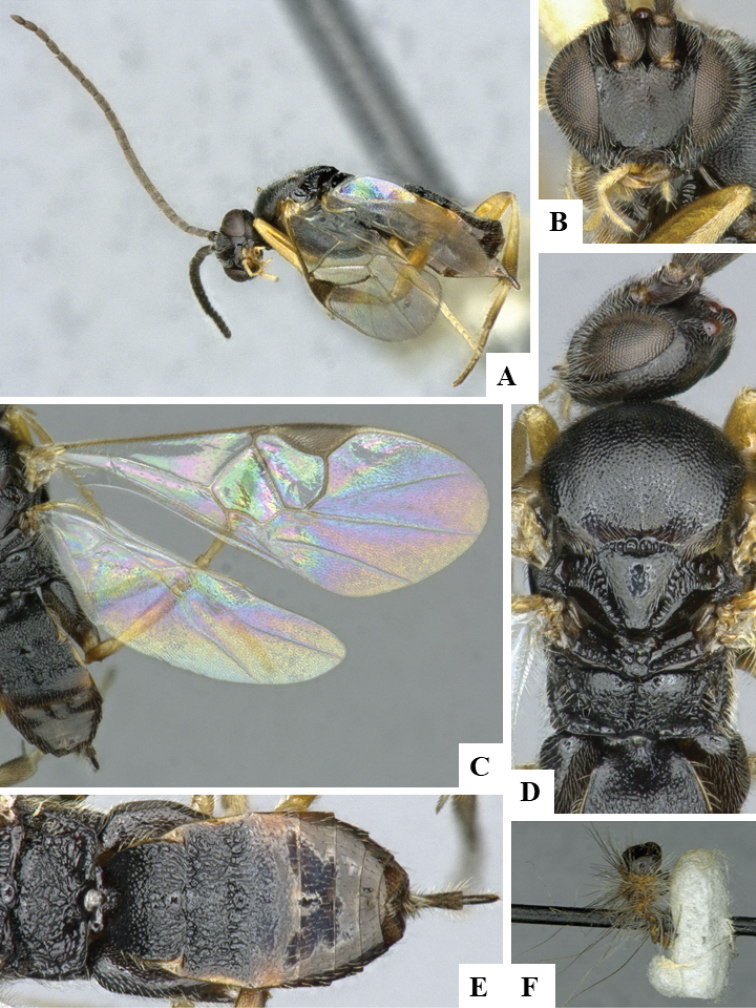
*Cotesiahyphantriae* female CNC721970 **A** Habitus, lateral **B** Head, frontal **C** Fore wing and hind wing **D** Mesosoma, dorsal **E** Propodeum and metasoma, dorsal **F** Cocoon and host larvae.

**Figure 55.**
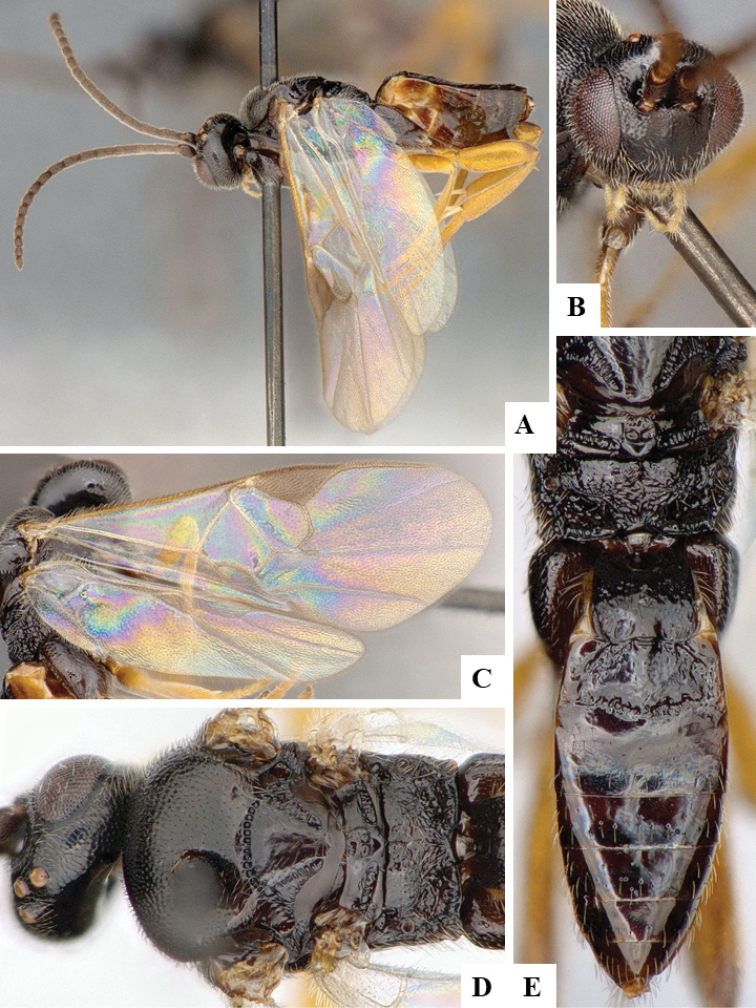
*Cotesiaisolde* female CNC475164 **A** Habitus, lateral **B** Head, frontal **C** Fore wing and hind wing **D** Mesosoma, dorsal **E** Propodeum and metasoma, dorsal.

**Figure 56.**
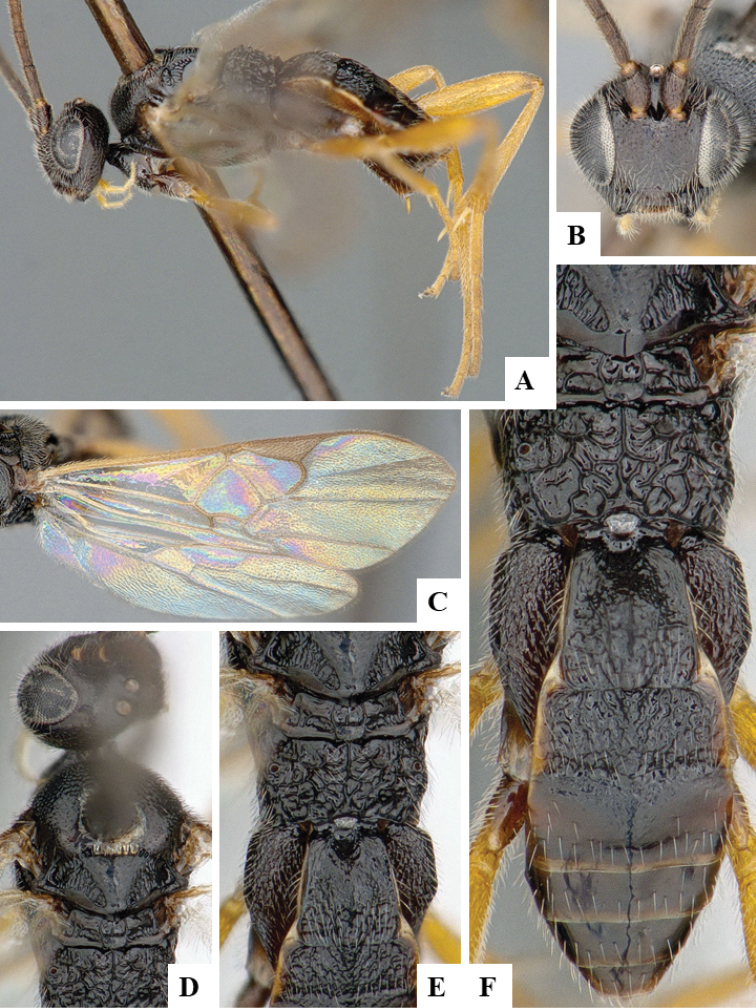
*Cotesiaofella* female CNC474690 **A** Habitus, lateral **B** Head, frontal **C** Fore wing and hind wing **D** Mesosoma, dorsal **E** Propodeum and tergites 1–2, dorsal **F** Propodeum and metasoma, dorsal.

**Figure 57.**
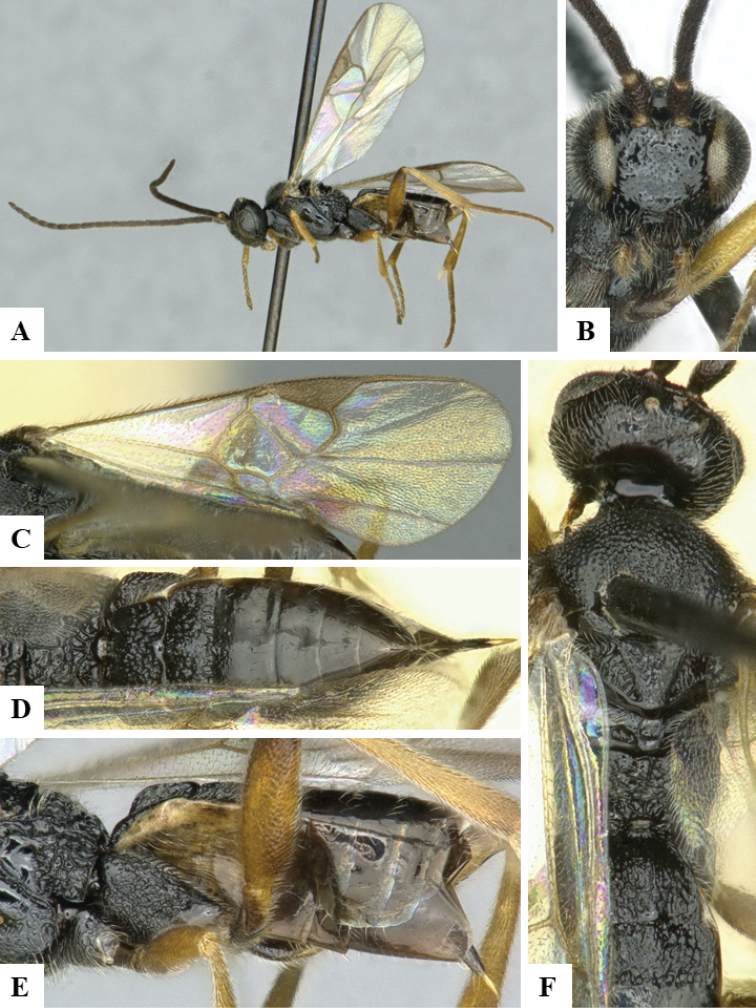
*Cotesiaordinaria* female CNC280830 **A** Habitus, lateral **B** Head, frontal **C** Fore wing **D** Propodeum and metasoma, dorsal **E** Metasoma, lateral **F** Head and mesosoma, dorsal.

**Figure 58.**
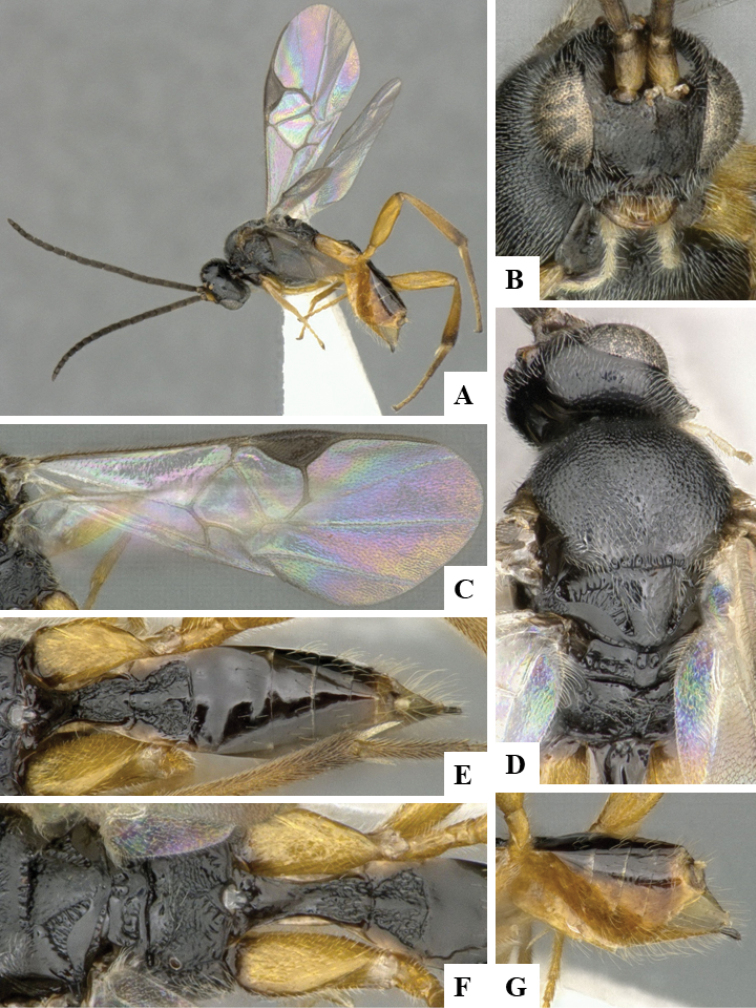
*Cotesiapistrinariae* female CNC841352 **A** Habitus, lateral **B** Head, frontal **C** Fore wing **D** Mesosoma, dorsal **E** Metasoma, dorsal **F** Propodeum and tergites 1–2, dorsal **G** Ovipositor and ovipositor sheaths.

**Figure 59.**
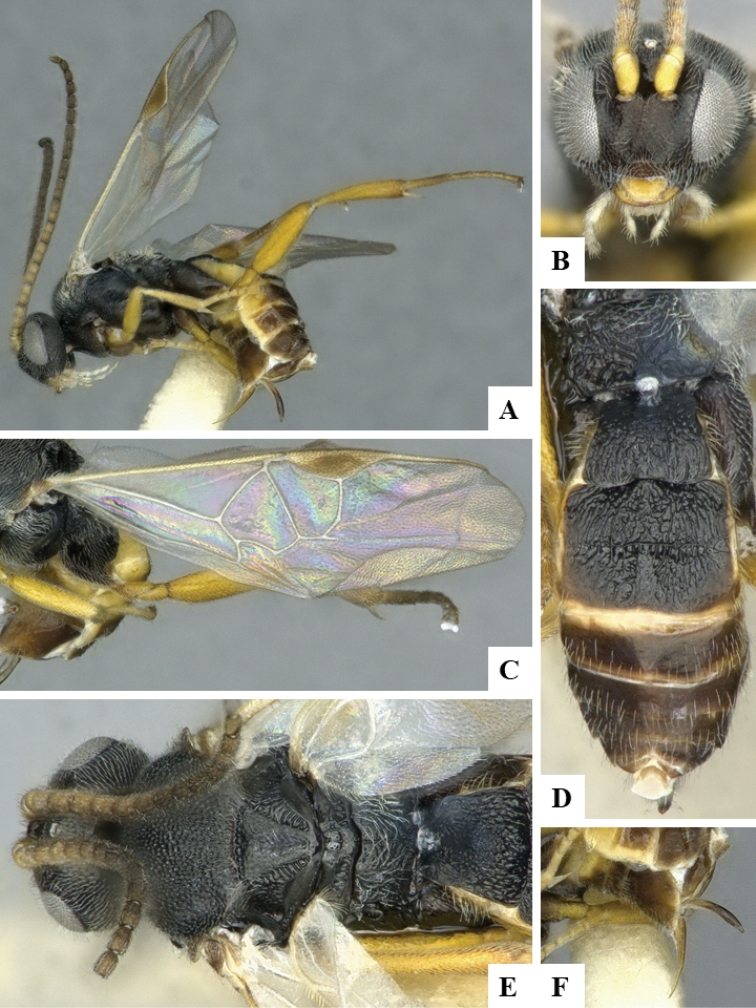
*Cotesiaschaeferi* female CNC280847 **A** Habitus, lateral **B** Head, frontal **C** Fore wing **D** Metasoma, dorsal **E** Head and mesosoma, dorsal **F** Ovipositor and ovipositor sheaths.

**Figure 60.**
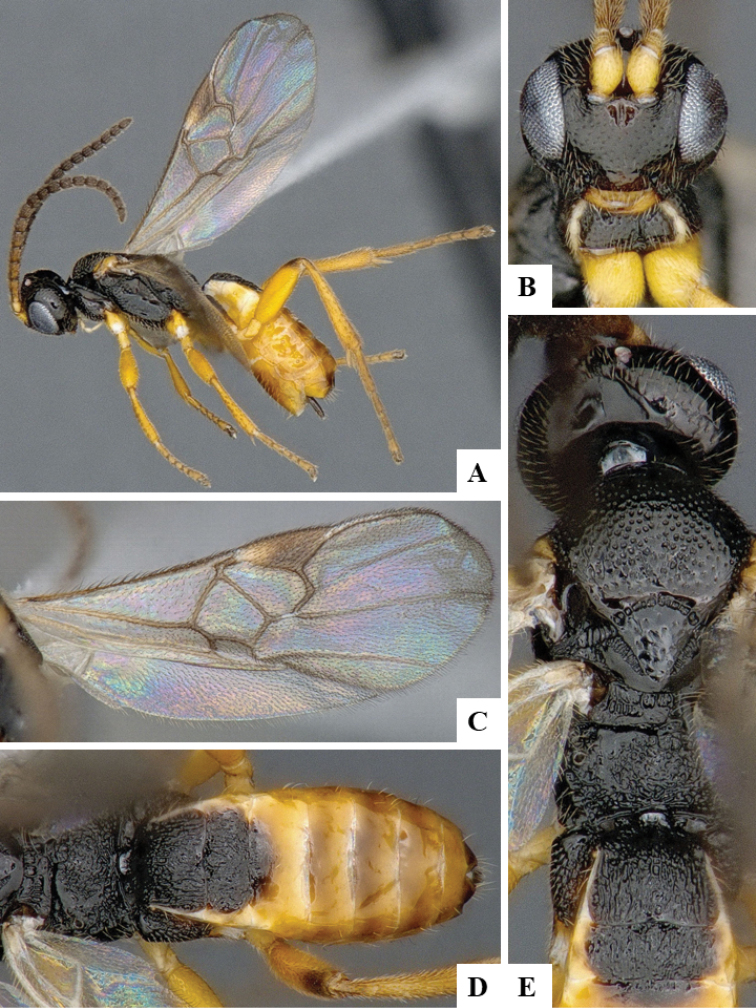
*Cotesiatyphae* female CNC634434 **A** Habitus, lateral **B** Head, frontal **C** Fore wing **D** Propodeum and metasoma, dorsal **E** Mesosoma, tergites 1–2, dorsal.

**Figure 61.**
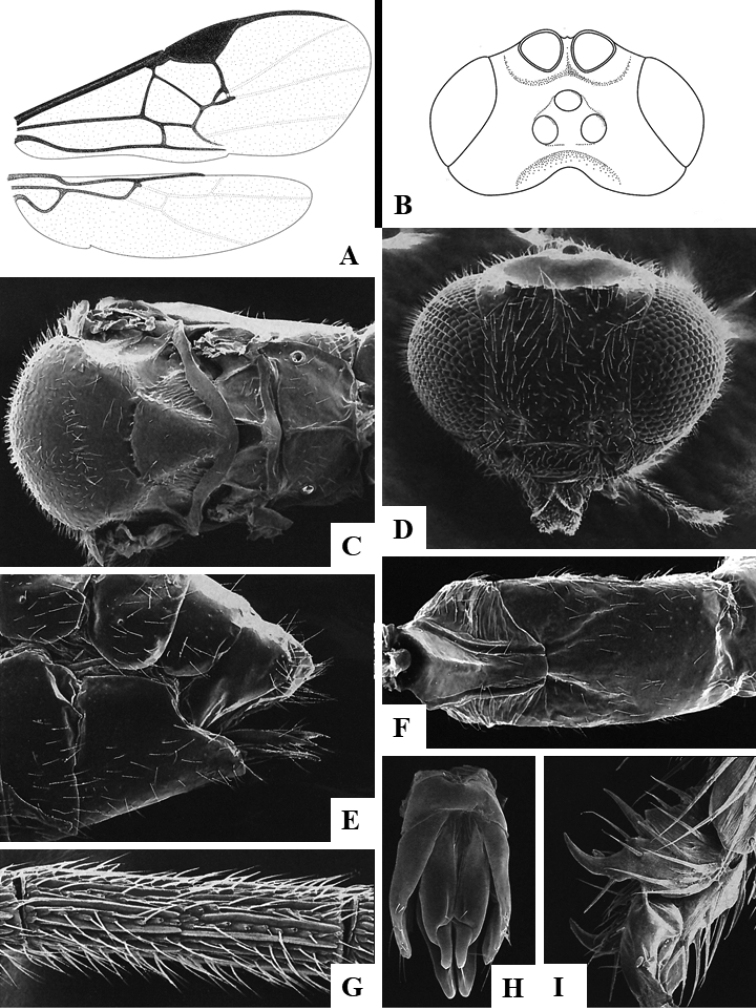
*Cuneogasterinae* female holotype (except H that is a paratype image) based on modified drawings and SEM images from the original descriptions of the species (Choi and Whitfield 2006) **A** Fore wing and hind wing **B** Head, dorsal **C** Mesosoma, dorsal **D** Head, frontal **E** Hypopygium and ovipositor sheaths **F** Tergites 1–3 **G** Fifth antennal segment, dorsal **H** Male genitalia, dorsal **I** Hind tarsal claw, lateral.

**Figure 62.**
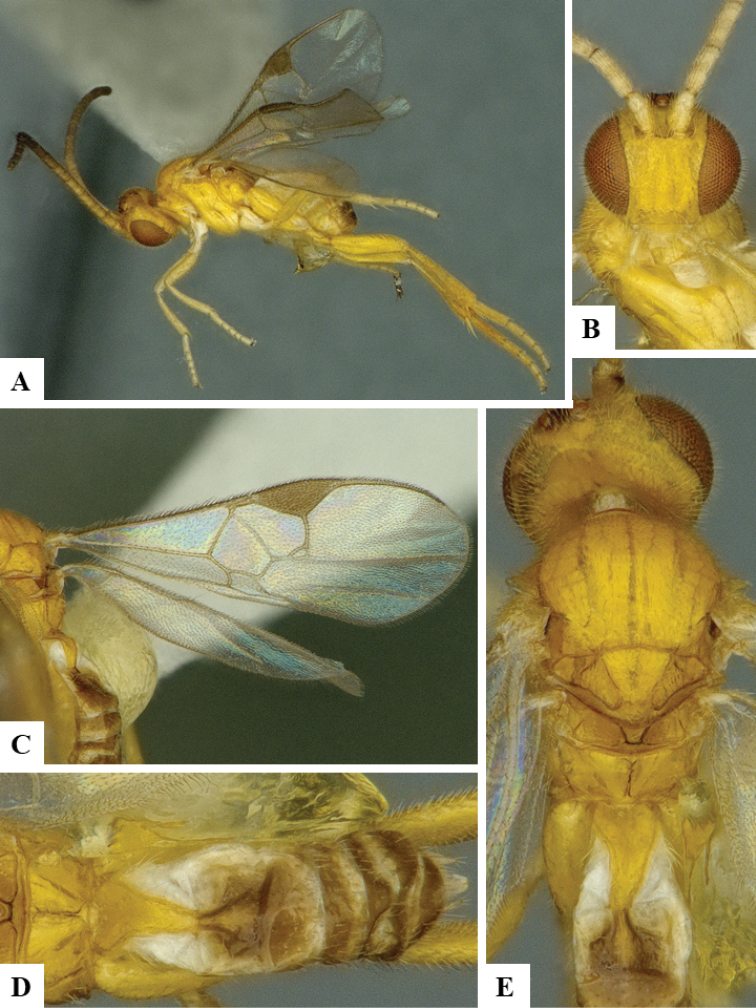
*Cuneogaster* sp. male CNC1065632 **A** Habitus, lateral **B** Head, frontal **C** Fore wing and hind wing **D** Propodeum and metasoma, dorsal **E** Mesosoma, dorsal.

**Figure 63.**
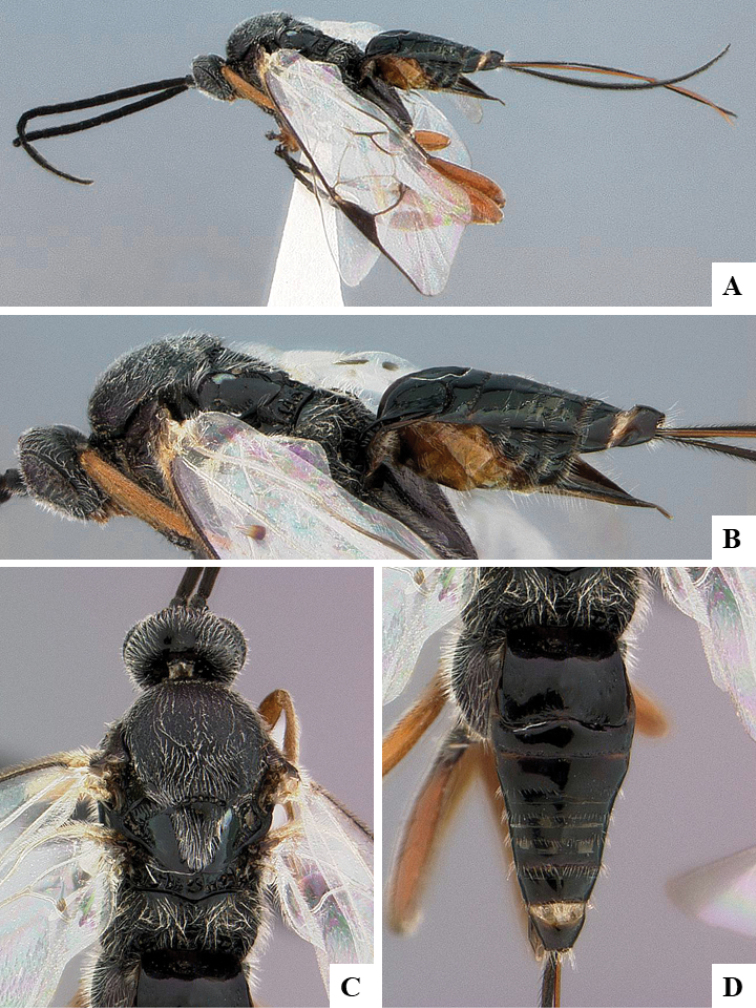
*Dasylagonaegeriae* female holotype **A** Habitus, lateral **B** Habitus, magnified **C** Mesosoma, dorsal **D** Metasoma, dorsal.

**Figure 64.**
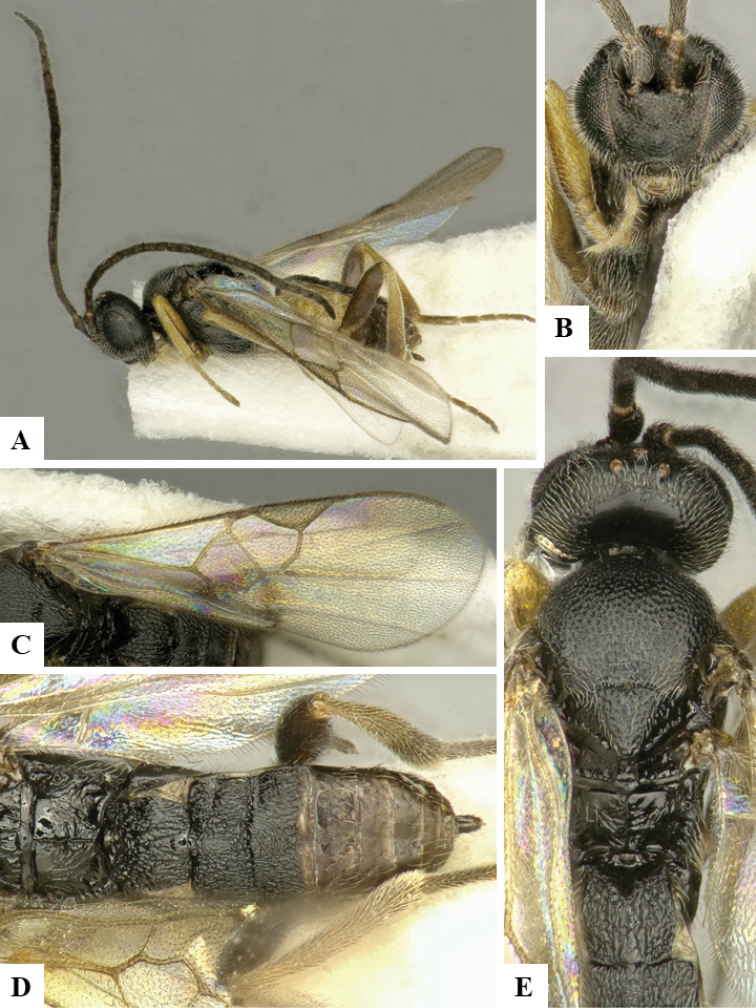
*Deuterixyscarbonaria* female CNC878801 **A** Habitus, lateral **B** Head, frontal **C** Fore wing **D** Propodeum and metasoma, dorsal **E** Head and mesosoma, dorsal.

**Figure 65.**
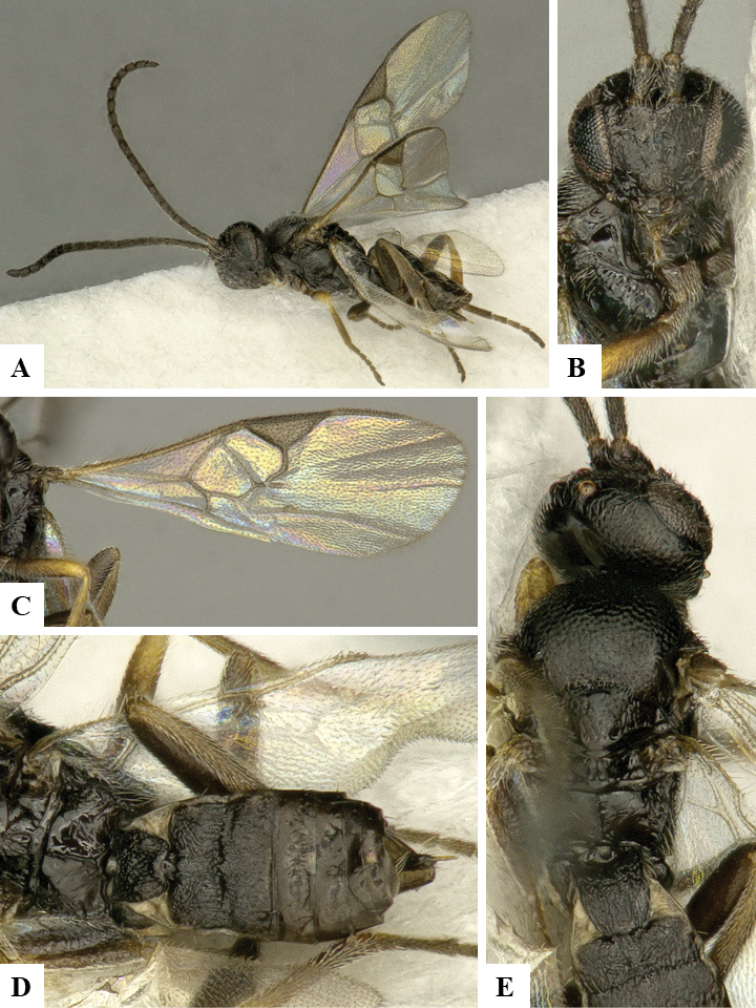
*Deuterixysrimulosa* female CNC638336 **A** Habitus, lateral **B** Head, frontal **C** Fore wing **D** Propodeum and metasoma, dorsal **E** Mesosoma, dorsal.

**Figure 66.**
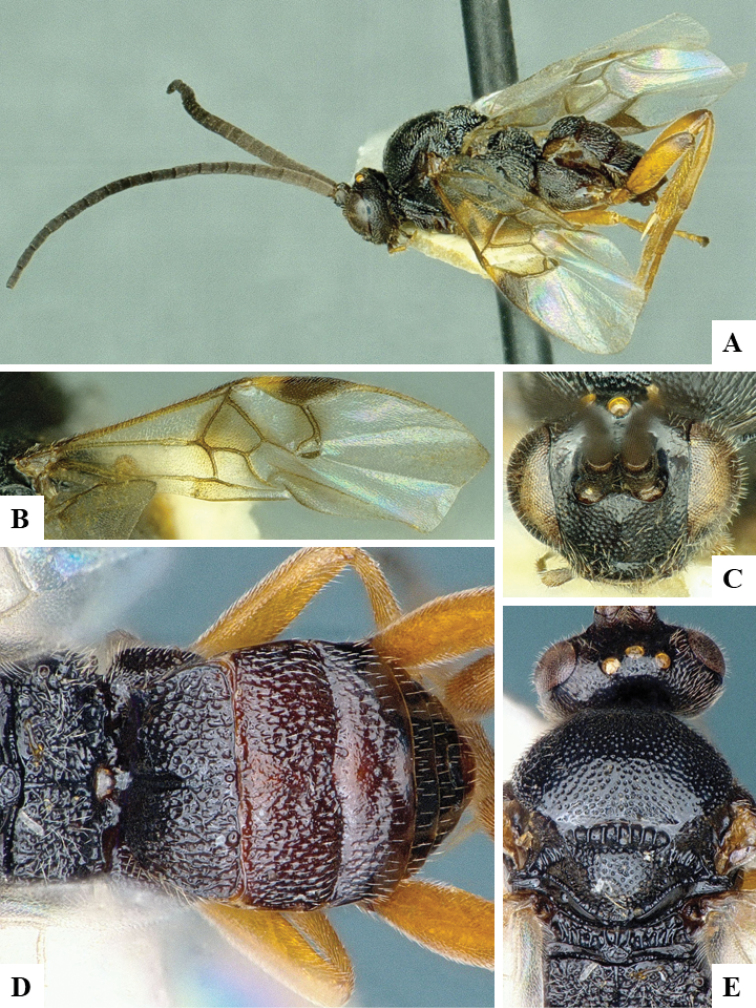
*Diolcogasterabdominalis* male CNCHYM00768 **A** Habitus, lateral **B** Fore wing **C** Head, frontal **D** Propodeum and metasoma, dorsal **E** Head and mesosoma, dorsal.

**Figure 67.**
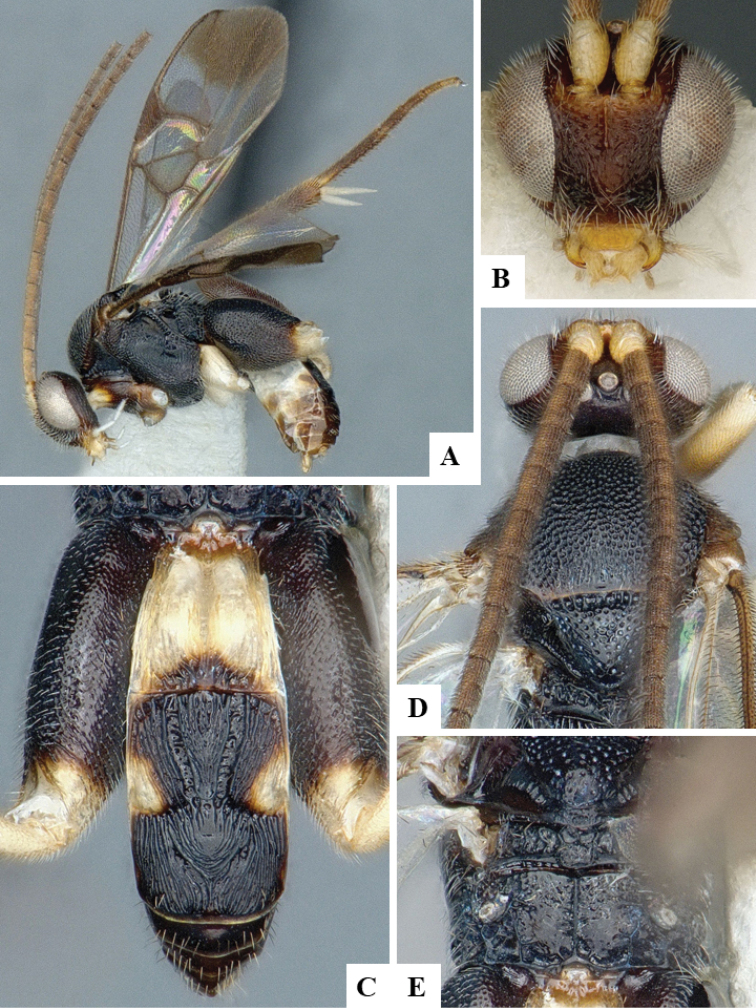
*Diolcogasteralkingara* male CNCHYM00775 **A** Habitus, lateral **B** Head, frontal **C** Metasoma, dorsal **D** Head and mesosoma, dorsal **E** Propodeum, dorsal.

**Figure 68.**
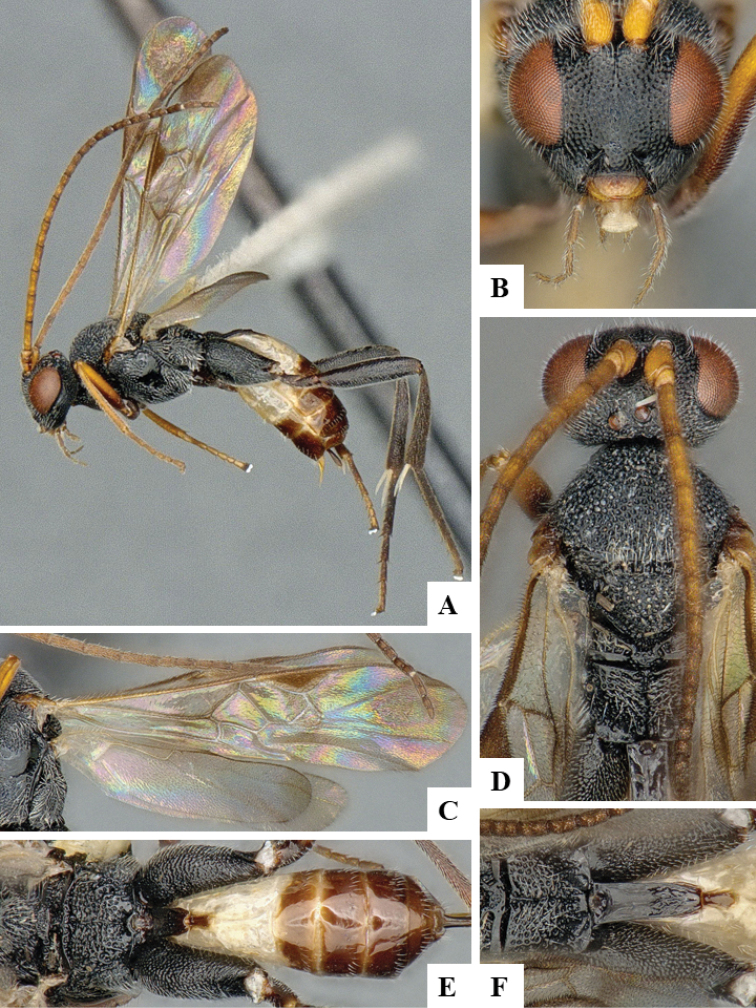
*Diolcogasterashmeadi* female CNCHYM00785 **A** Habitus, lateral **B** Head, frontal **C** Fore wing and hind wing **D** Head and mesosoma, dorsal **E** Propodeum and metasoma, dorsal **F** Propodeum and tergites 1–2, dorsal.

**Figure 69.**
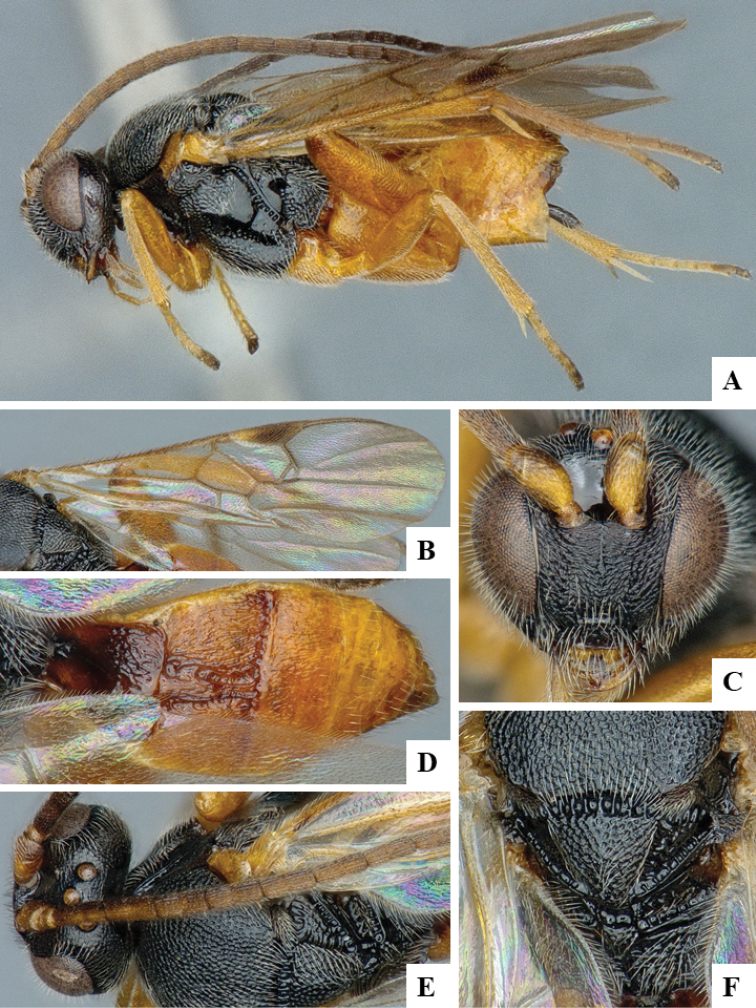
*Diolcogasterauripes* female CNC475093 **A** Habitus, lateral **B** Fore wing **C** Head, frontal **D** Metasoma, dorsal **E** Head, dorsal **F** Mesosoma, dorsal.

**Figure 70.**
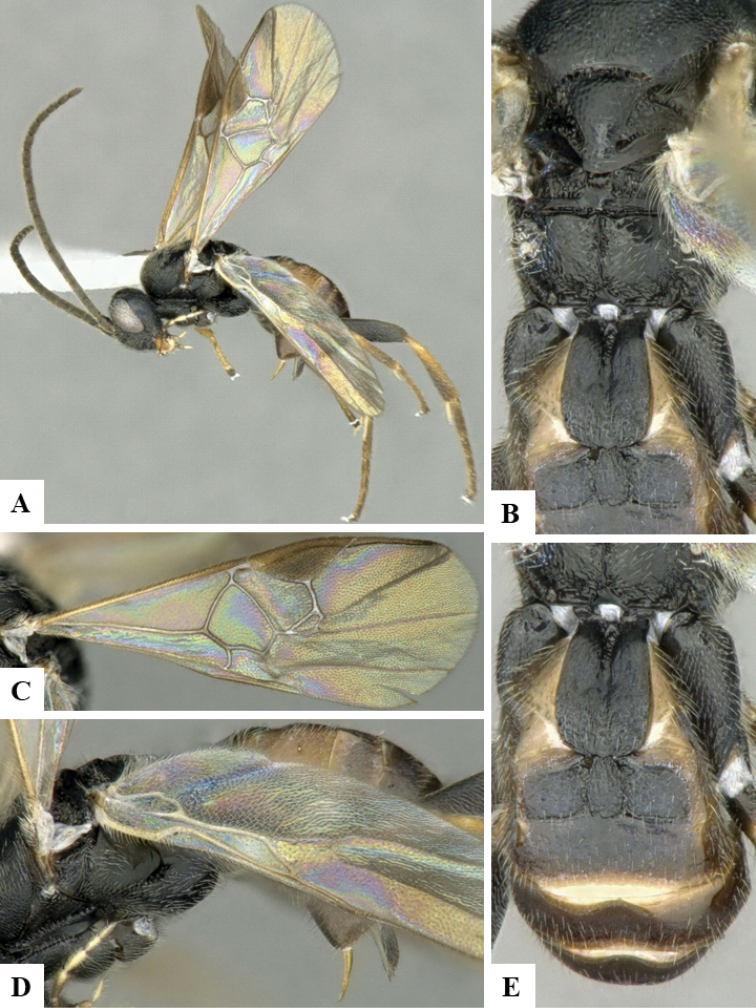
*Diolcogasterclaritibia* female HYM00000437 **A** Habitus, lateral **B** Mesosoma and tergites 1–2, dorsal **C** Fore wing **D** Mesosoma and metasoma, lateral **E** Metasoma, dorsal.

**Figure 71.**
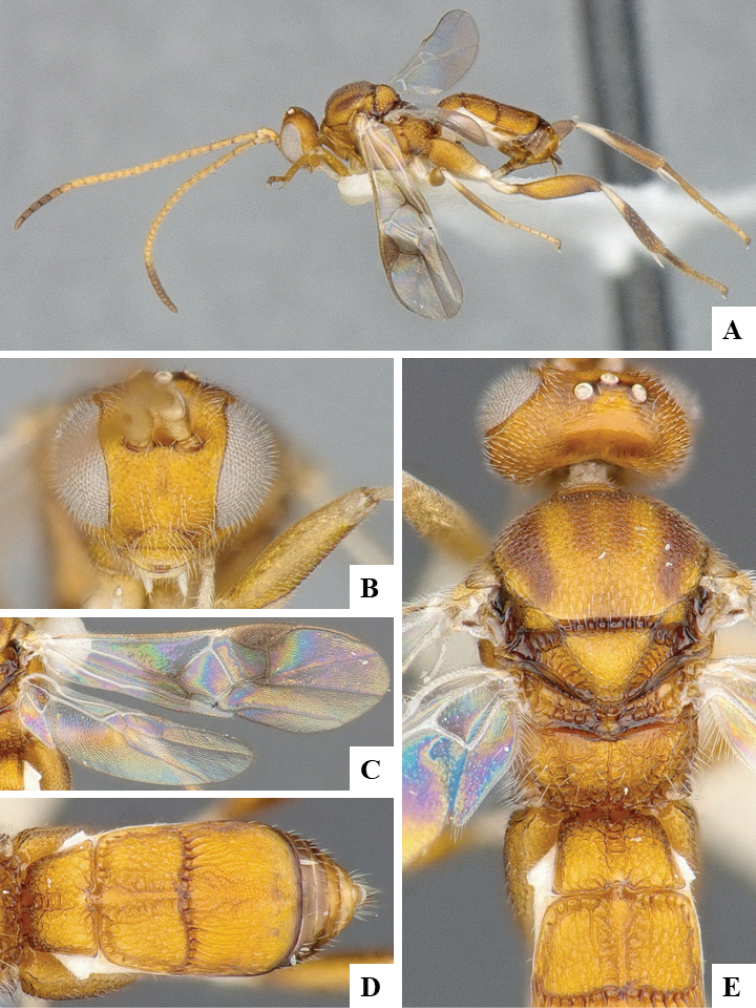
*Diolcogasterichiroi* female holotype **A** Habitus, lateral **B** Head, frontal **C** Fore wing and hind wing **D** Metasoma, dorsal **E** Head and mesosoma, dorsal.

**Figure 72.**
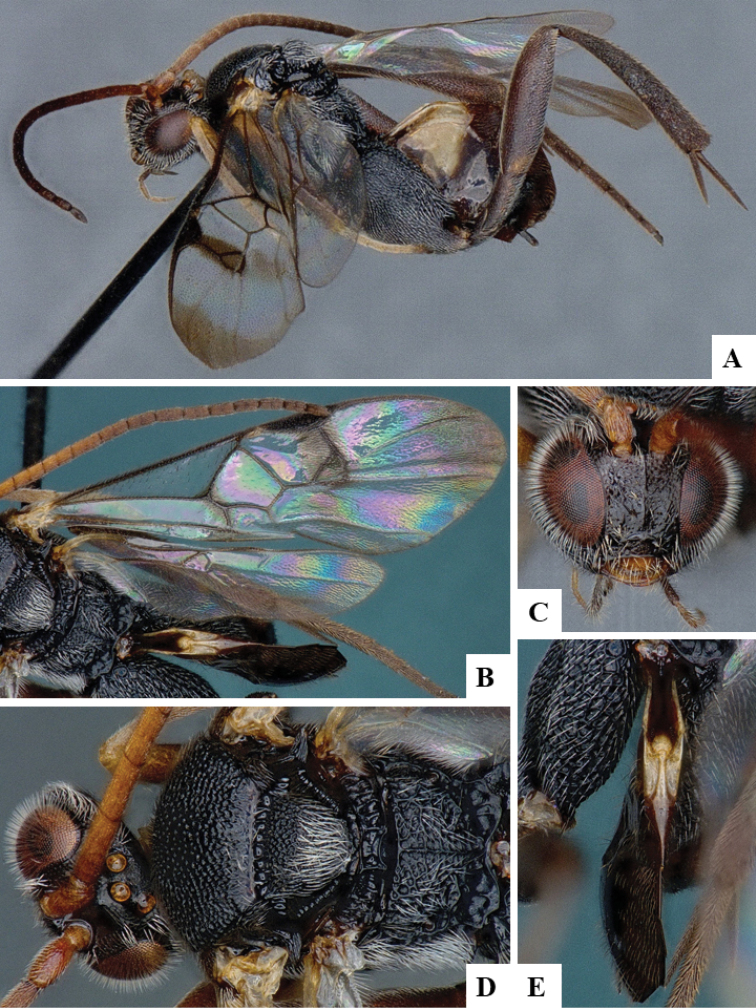
*Diolcogasterippis* female CNCHYM00835 **A** Habitus, lateral **B** Fore wing and hind wing **C** Head, frontal **D** Head and mesosoma; dorsal **E** Metasoma, dorsal.

**Figure 73.**
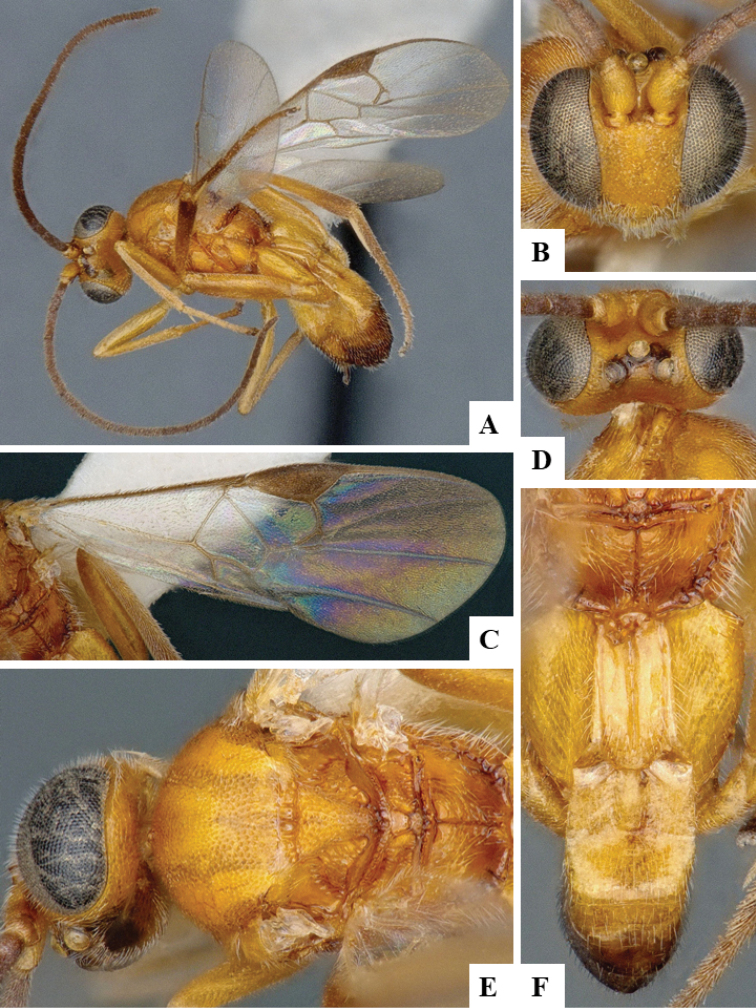
*Diolcogasteriqbali* female CNCHYM00833 **A** Habitus, lateral **B** Head, frontal **C** Fore wing **D** Head, dorsal **E** Mesosoma, dorsal **F** Propodeum and metasoma, dorsal.

**Figure 74.**
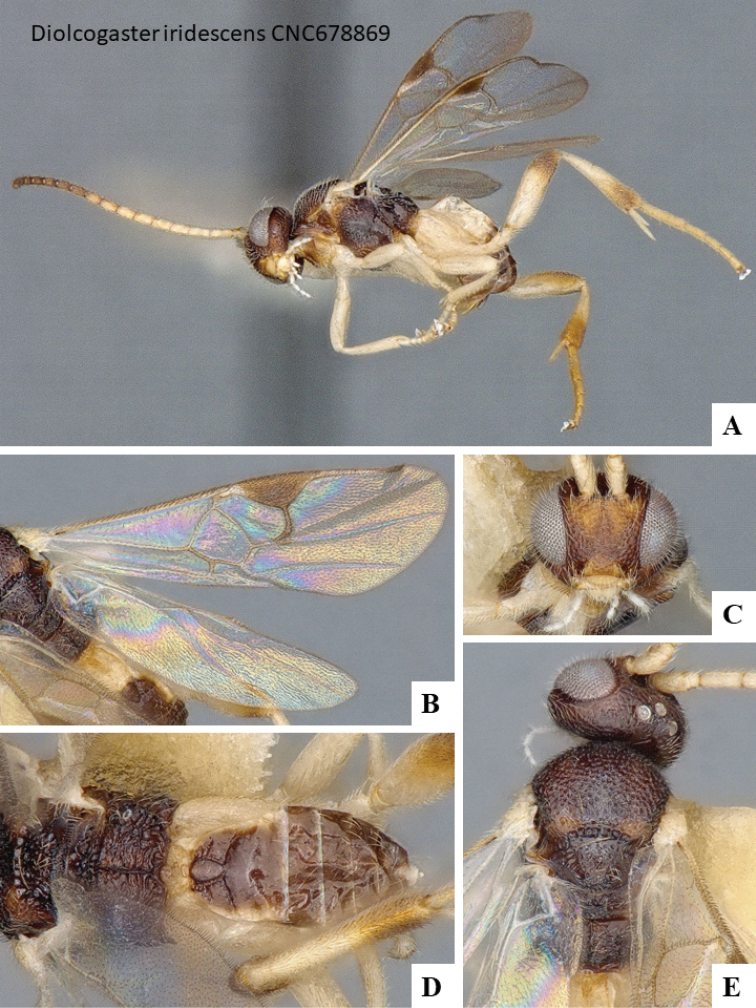
*Diolcogasteriridescens* female CNC678869 **A** Habitus, lateral **B** Fore wing and hind wing **C** Head, frontal **D** Propodeum and metasoma, dorsal **E** Head and mesosoma, dorsal.

**Figure 75.**
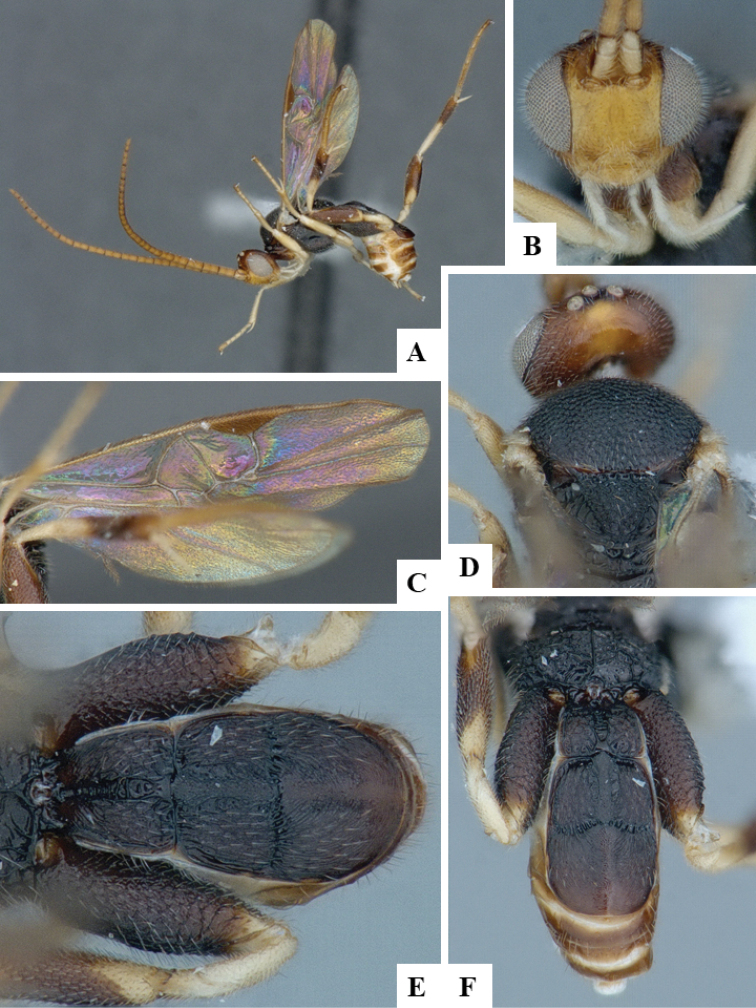
*Diolcogastermiamensis* male paratype CNC489838 **A** Habitus, lateral **B** Head, frontal **C** Fore wing **D** Mesosoma, dorsal **E** Metasoma, dorsal **F** Propodeum and metasoma, dorsal.

**Figure 76.**
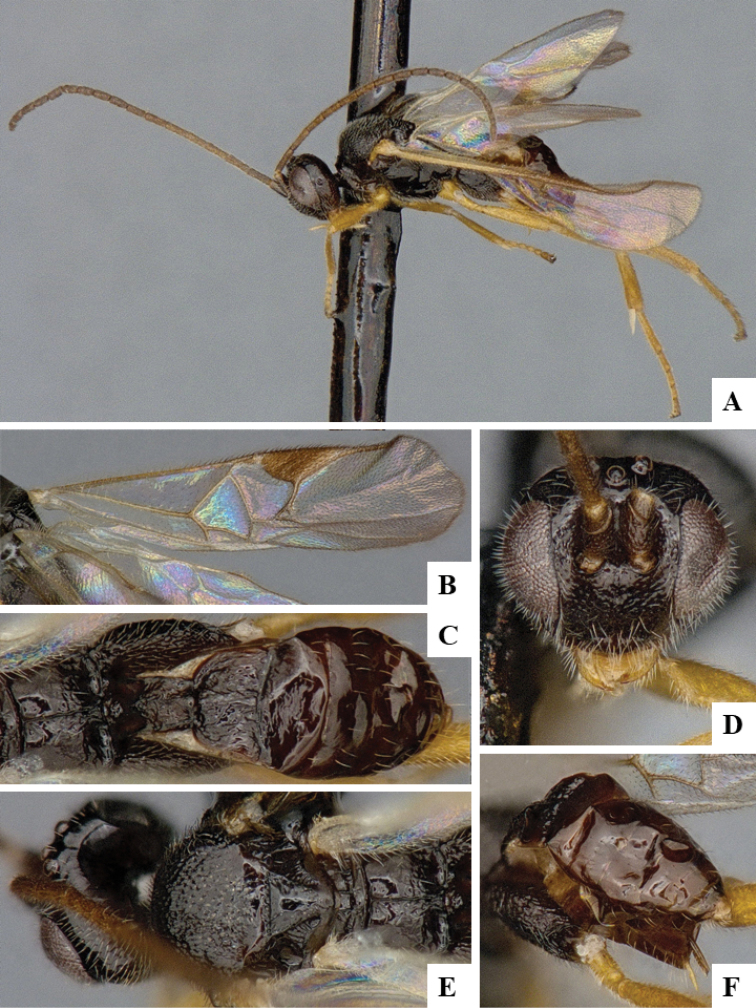
*Diolcogasterminuta* female CNC280884 **A** Habitus, lateral **B** Fore wing **C** Propodeum and metasoma, dorsal **D** Head, frontal **E** Mesosoma, dorsal **F** Ovipositor and ovipositor sheaths.

**Figure 77.**
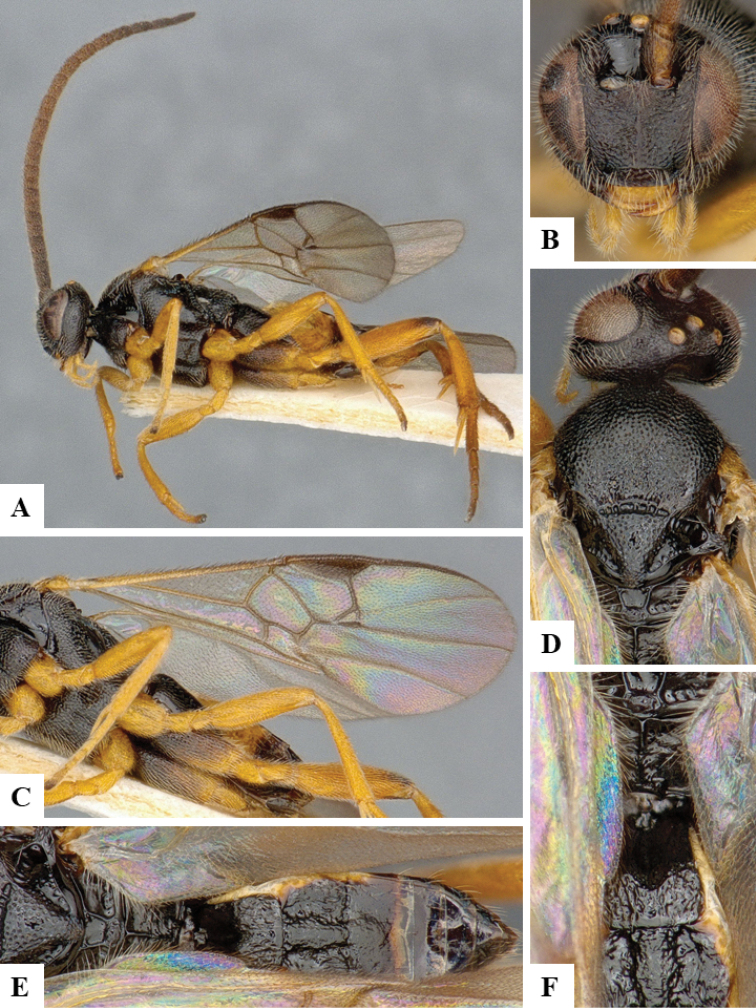
*Diolcogasterscotica* male CNC474705 **A** Habitus, lateral **B** Head, frontal **C** Fore wing **D** Head and mesosoma, dorsal **E** Propodeum and metasoma, dorsal **F** Propodeum and tergites 1–2, dorsal.

**Figure 78.**
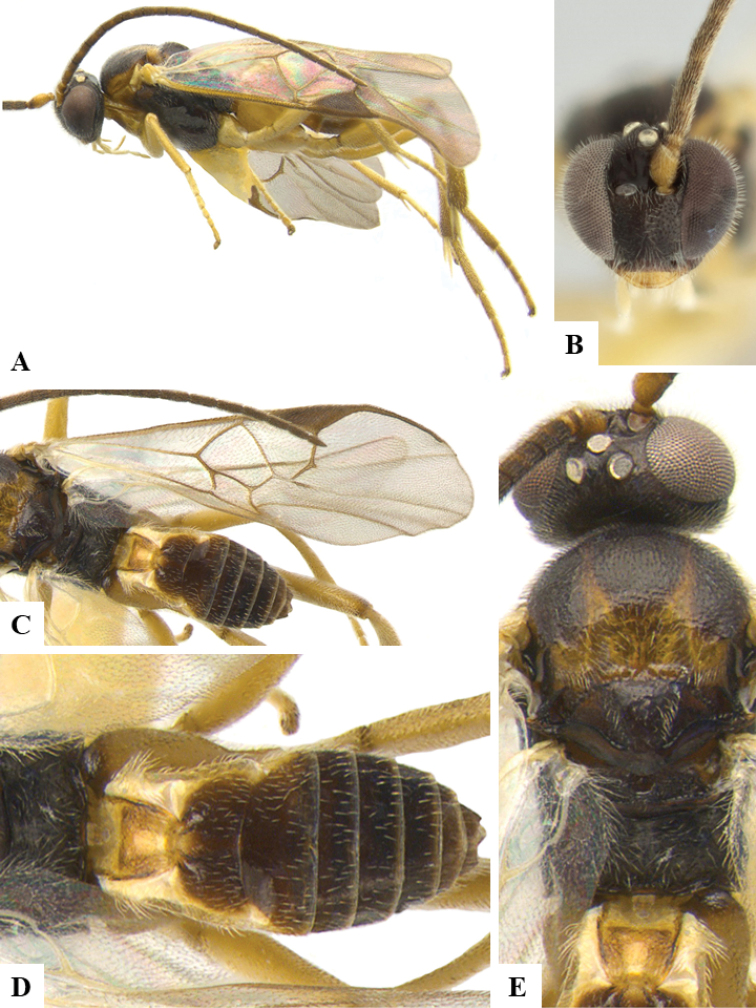
*Distatrixcarolinae* female holotype **A** Habitus, lateral **B** Head, frontal **C** Fore wing **D** Metasoma, dorsal **E** Head and mesosoma, dorsal.

**Figure 79.**
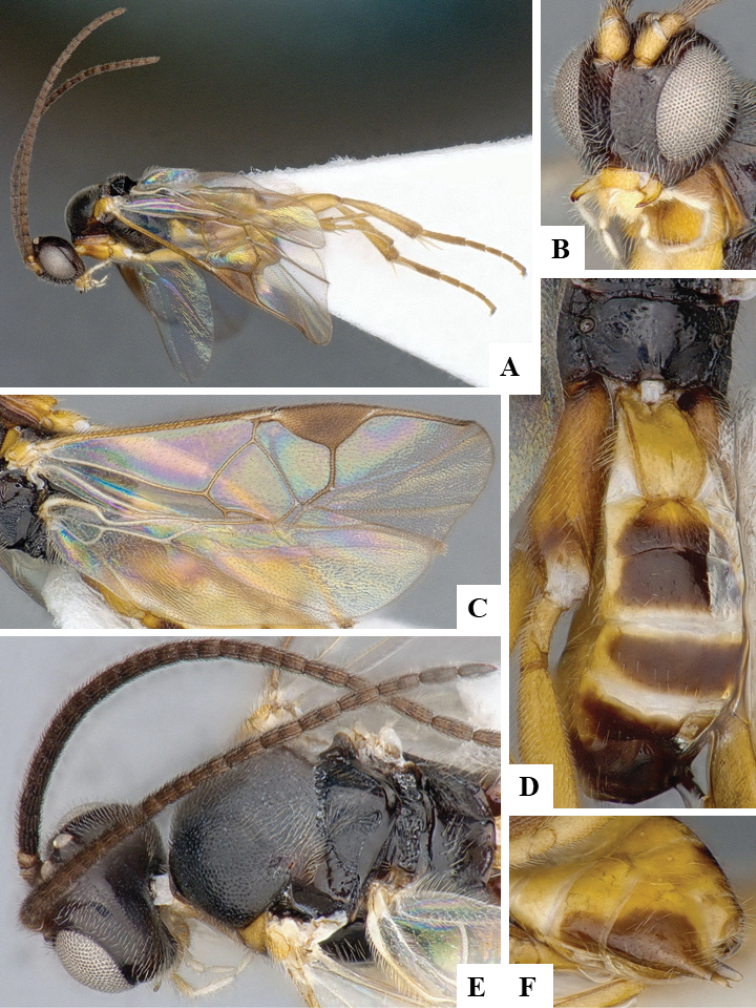
*Distatrixyemeniticus* female paratype WAM 0138 **A** Habitus, lateral **B** Head, frontolateral **C** Fore wing and hind wing **D** Propodeum and metasoma, dorsal **E** Mesosoma, dorsal **F** Ovipositor and ovipositor sheaths.

**Figure 80.**
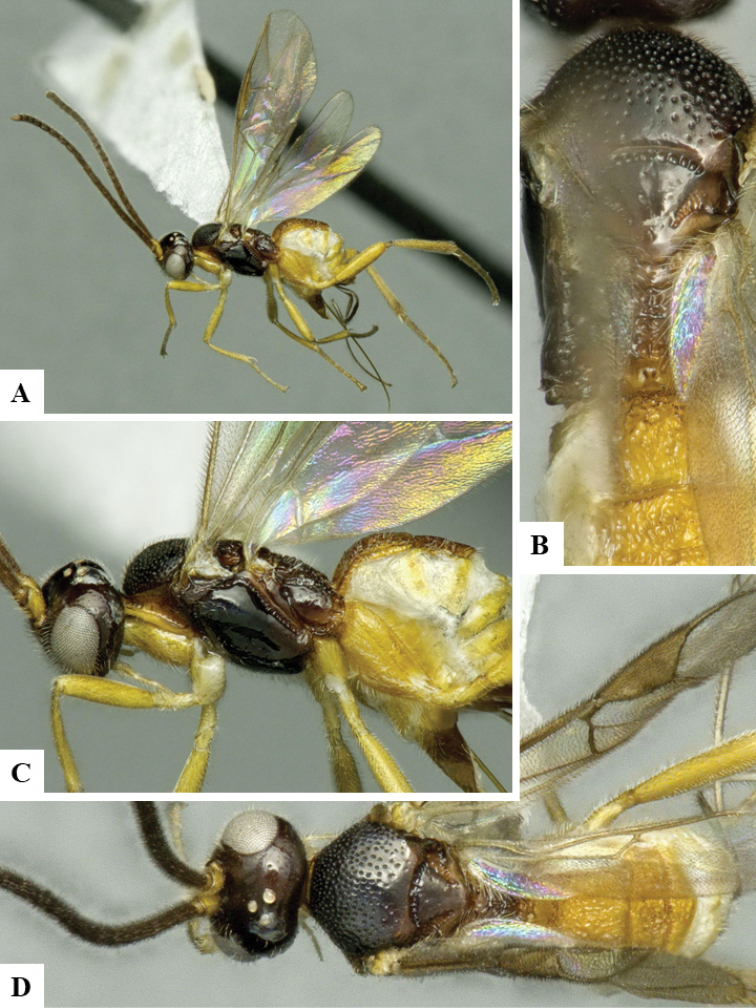
*Dodogastergrangeri* female paratype (CNC841340) **A** Habitus, lateral **B** Mesosoma, dorsal **C** Mesosoma, lateral **D** Habitus, dorsal.

**Figure 81.**
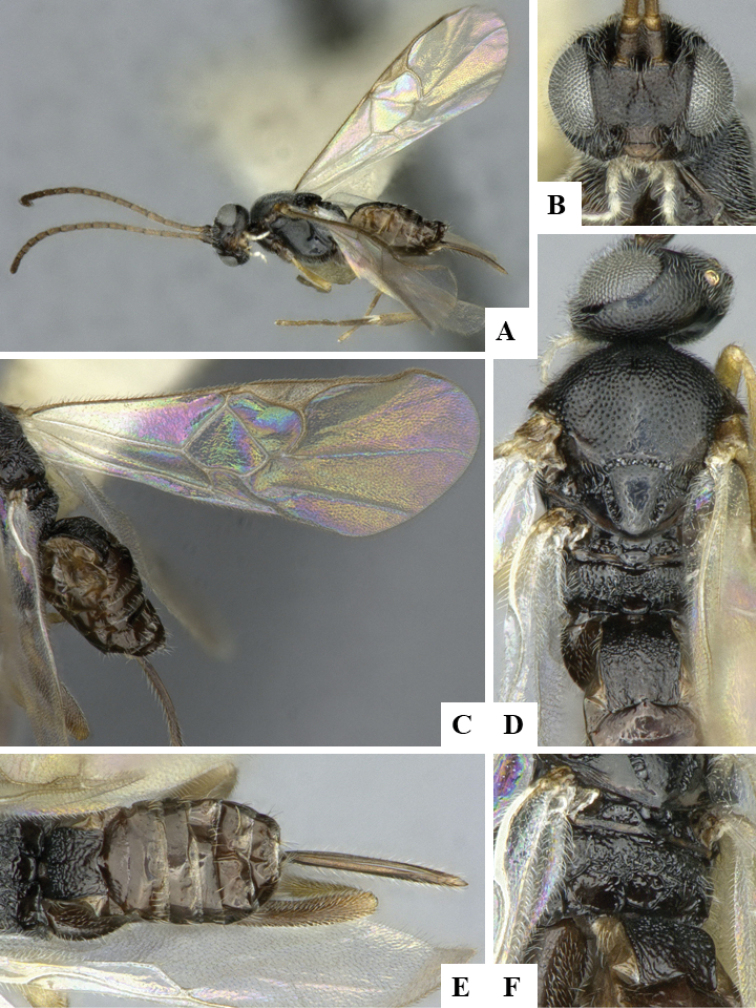
*Dolichogenideabaoris* female CNCHYM00980 **A** Habitus, lateral **B** Head, frontal **C** Fore wing **D** Mesosoma, dorsal **E** Metasoma, dorsal **F** Propodeum, dorsolateral.

**Figure 82.**
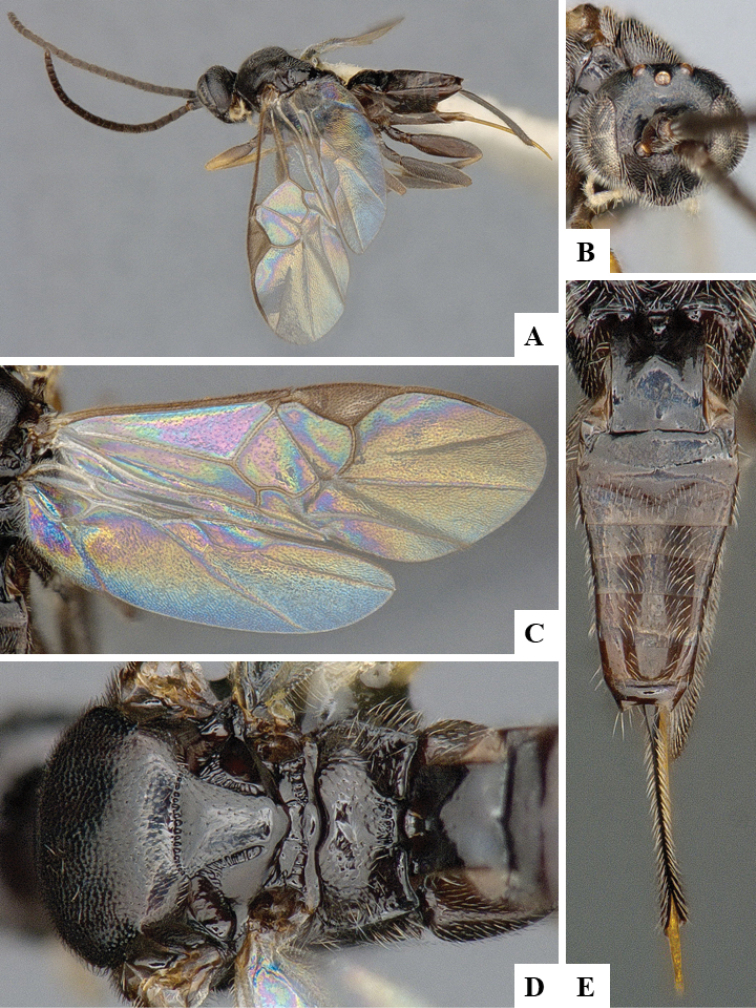
*Dolichogenideacandidata* female CNC677699 **A** Habitus, lateral **B** Head, frontal **C** Fore wing and hind wing **D** Mesosoma, dorsal **E** Metasoma, dorsal.

**Figure 83.**
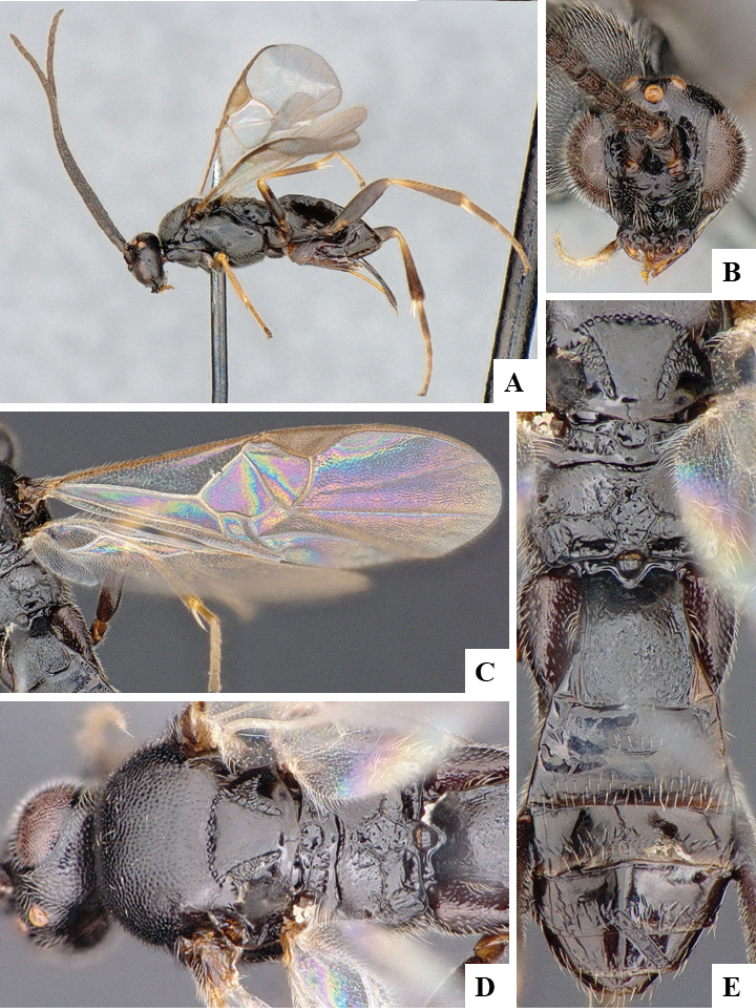
*Dolichogenideacoleophorae* female CNC475168 **A** Habitus, lateral **B** Head, frontal **C** Fore wing **D** Mesosoma, dorsal **E** Propodeum and metasoma, dorsal.

**Figure 84.**
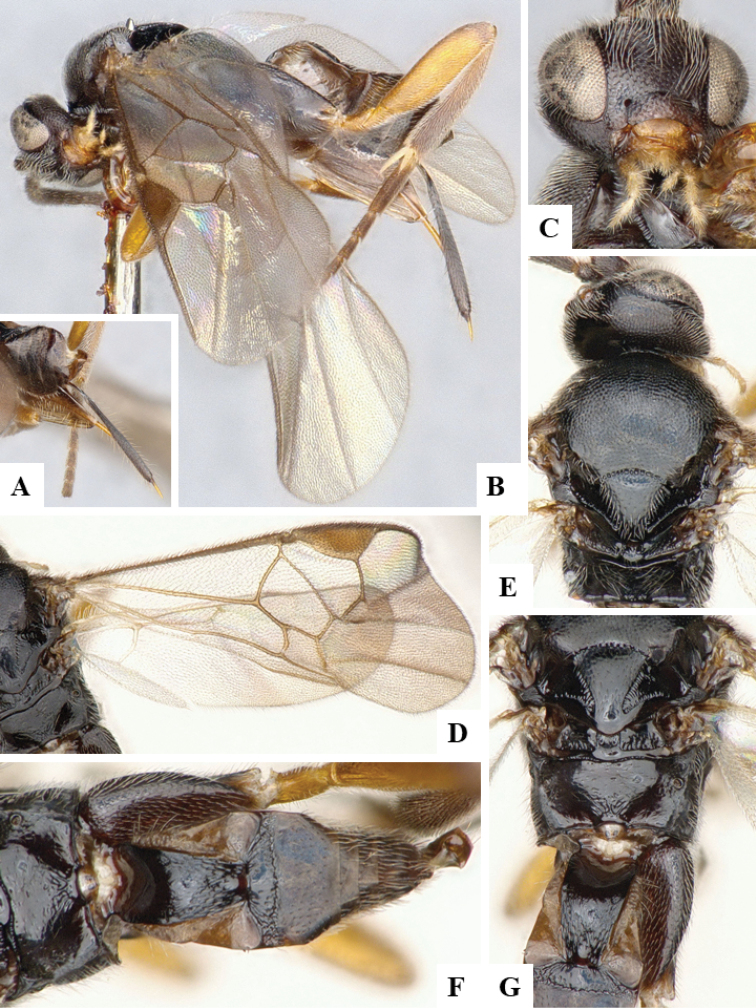
*Dolichogenideadilecta* female CNCHYM01030 **A** Ovipositor and ovipositor sheaths **B** Habitus, lateral **C** Head, frontal **D** Fore wing and hind wing **E** Mesosoma, dorsal **F** Propodeum and metasoma, dorsal **G** Propodeum and tergites 1–2, dorsal.

**Figure 85.**
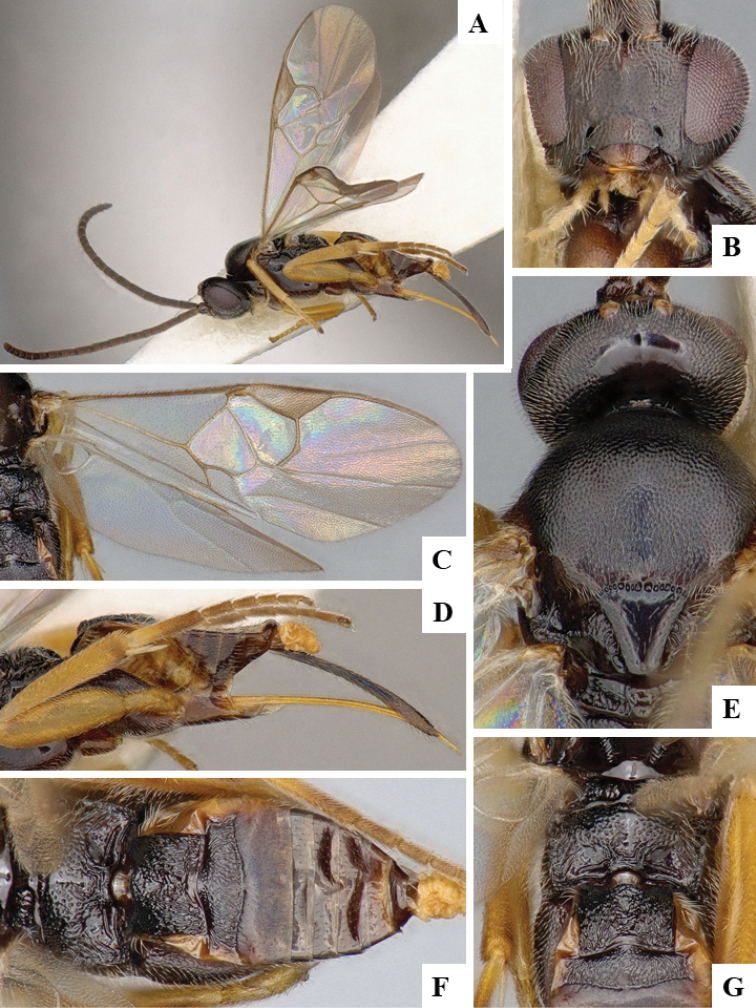
*Dolichogenideasolenobiae* female CNCHYM01140 **A** Habitus, lateral **B** Head, frontal **C** Fore wing and hind wing **D** Metasoma, lateral **E** Head and mesosoma, dorsal **F** Propodeum and metasoma, dorsal **G** Propodeum and tergites 1–2, dorsal.

**Figure 86.**
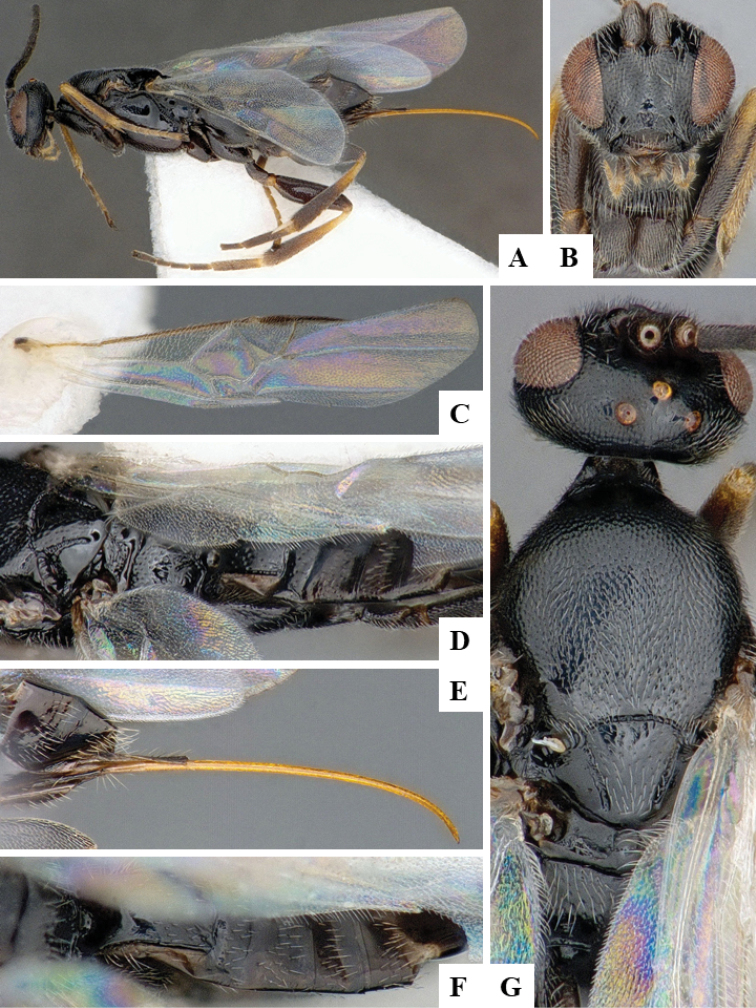
*Dolichogenideavictoriata* female CNCHYM01172 **A** Habitus, lateral **B** Head, frontal **C** Fore wing **D** Propodeum, dorsal **E** Ovipositor **F** Metasoma, dorsal **G** Head and mesosoma, dorsal.

**Figure 87.**
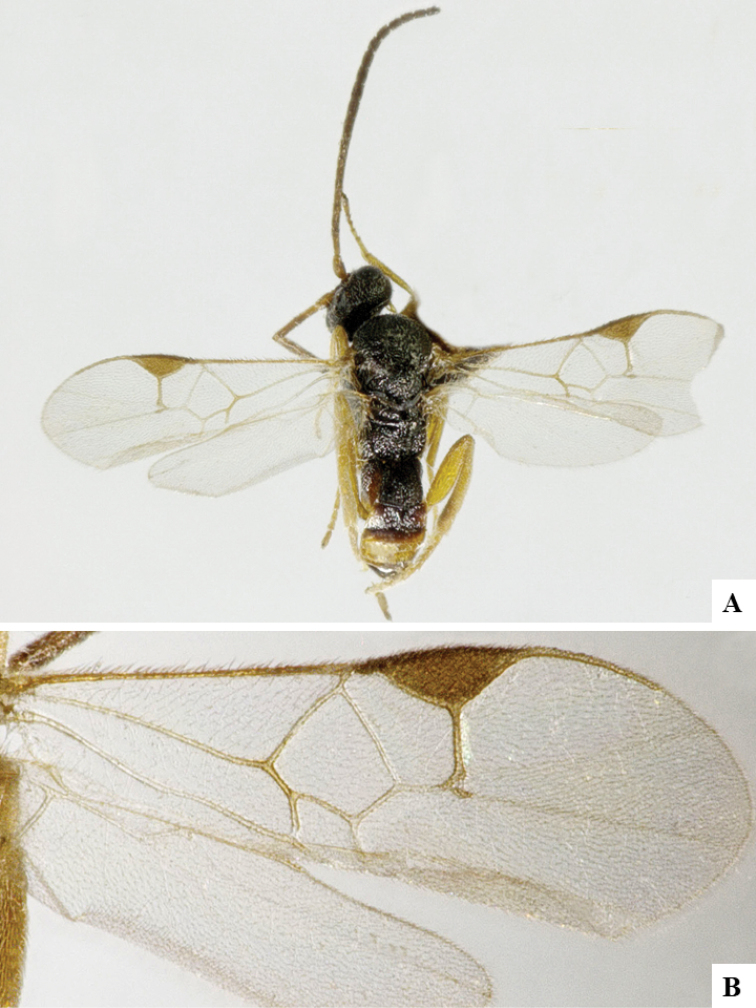
*Eripnopeltaithyvena* female holotype based on modified images from the original descriptions of the species (Xiong et al. 2017) **A** Habitus, dorsal **B** Fore wing and hind wing.

**Figure 88.**
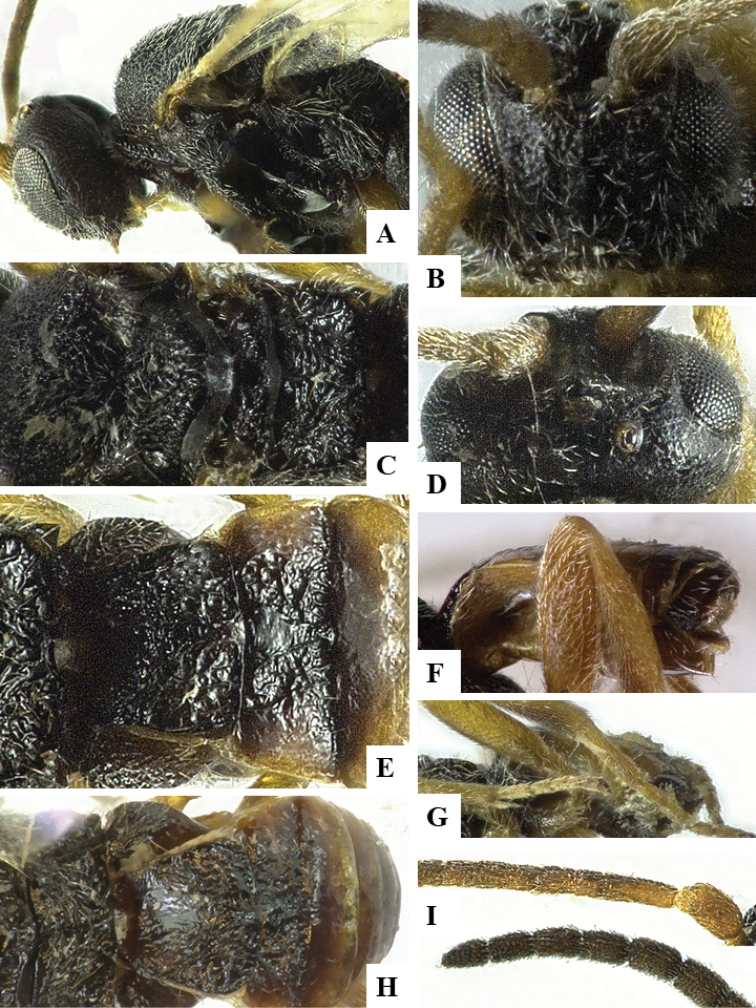
*Eripnopeltaithyvena* female holotype (except F and H that are male paratype images) based on modified images from the original descriptions of the species (Xiong et al. 2017) **A** Head and mesosoma, lateral **B** Head, frontal **C** Mesosoma, dorsal **D** Head, dorsal **E** Propodeum and tergites 1–2, dorsal **F** Metasoma of paratype, lateral **G** Hind leg **H** Propodeum and basal part of metasoma of paratype, dorsal **I** Antenna, basal segments and apical segments.

**Figure 89.**
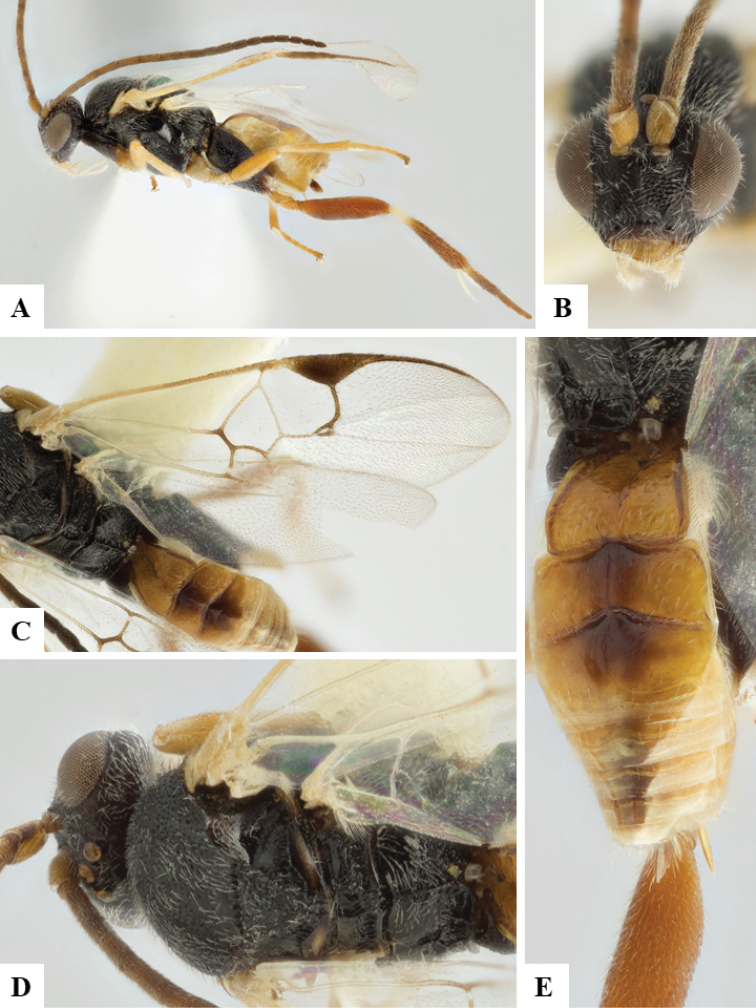
*Exixbahia* female holotype **A** Habitus, lateral **B** Head, frontal **C** Fore wing and hind wing **D** Mesosoma, laterodorsal **E** Metasoma, laterodorsal.

**Figure 90.**
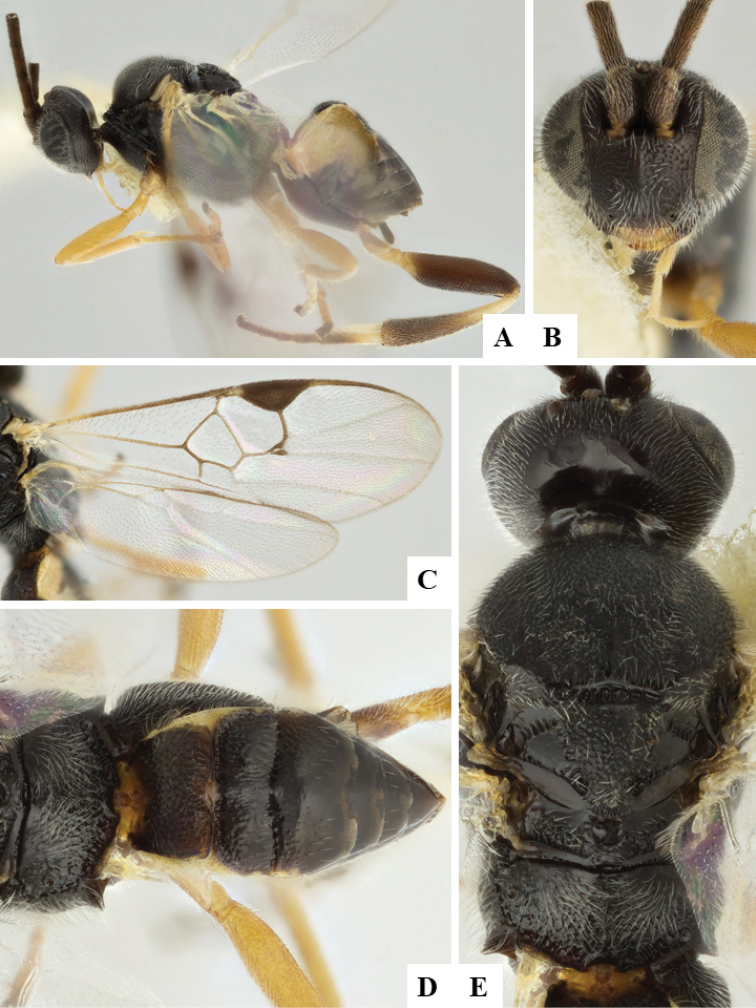
*Exixcolumbica* female holotype **A** Habitus, lateral **B** Head, frontal **C** Fore wing and hind wing **D** Propodeum and metasoma, dorsal **E** Head and mesosoma, dorsal.

**Figure 91.**
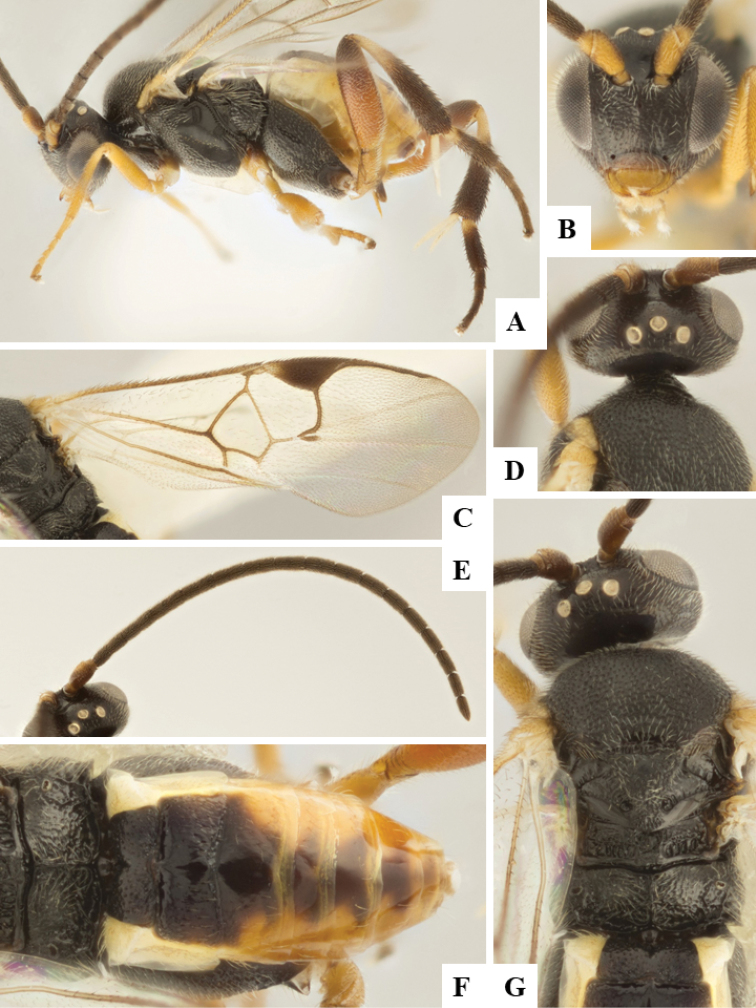
*Exixtinalandica* female holotype **A** Habitus, lateral **B** Head, frontal **C** Fore wing **D** Head, dorsal **E** Antenna **F** Propodeum and metasoma, dorsal **G** Mesosoma, dorsal.

**Figure 92.**
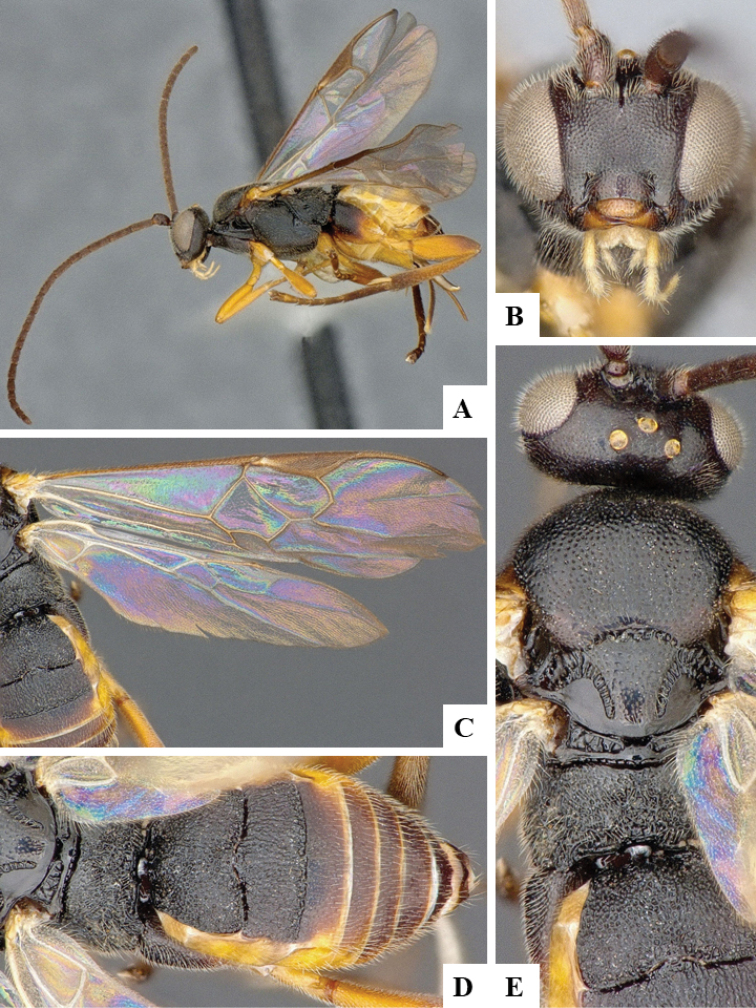
*Exoryzaoryzae* female CNCHYM01202 **A** Habitus, lateral **B** Head, frontal **C** Fore wing and hind wing **D** Propodeum and metasoma, dorsal **E** Head and mesosoma, dorsal.

**Figure 93.**
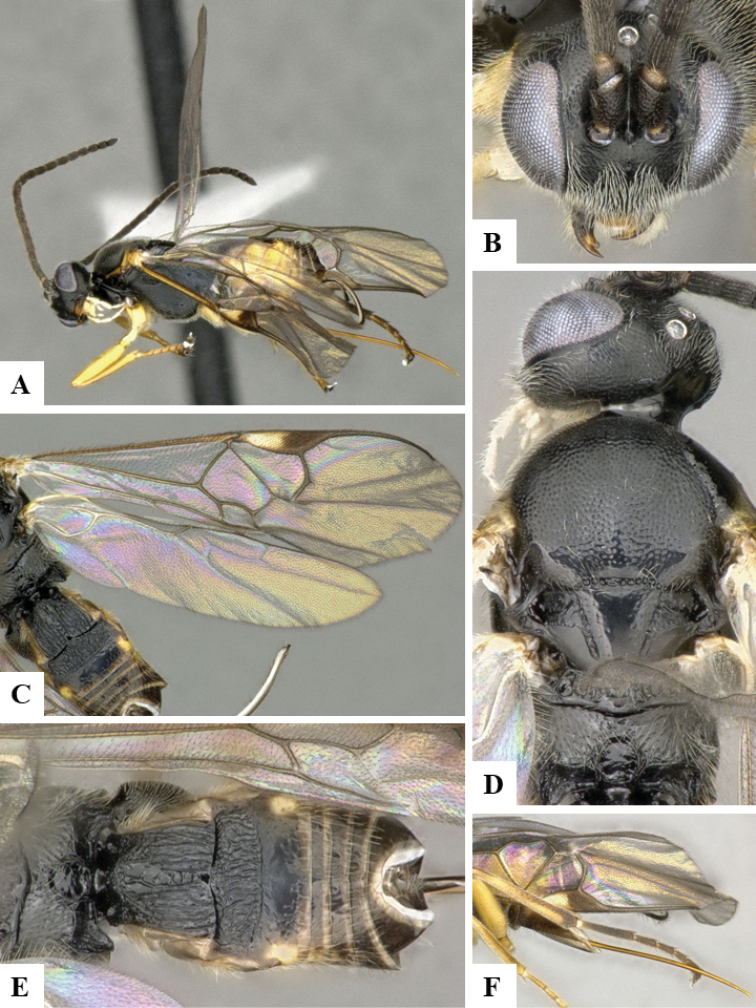
*Exoryzarichardashleyi* female DHJPAR0031507 **A** Habitus, lateral **B** Head, frontal **C** Fore wing and hind wing **D** Mesosoma, dorsal **E** Propodeum and metasoma, dorsal **F** Ovipositor.

**Figure 94.**
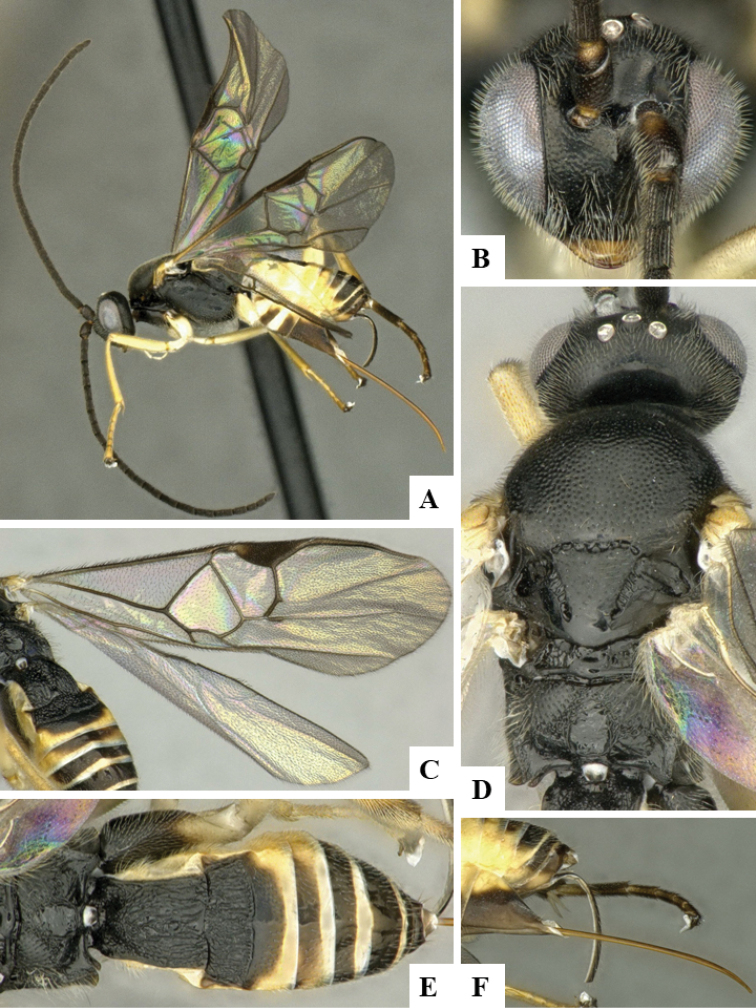
*Exoryzaritaashleyae* female DHJPAR0031500 **A** Habitus, lateral **B** Head, frontal **C** Fore wing and hind wing **D** Head and mesosoma, dorsal **E** Propodeum and metasoma, dorsal **F** Ovipositor and ovipositor sheaths.

**Figure 95.**
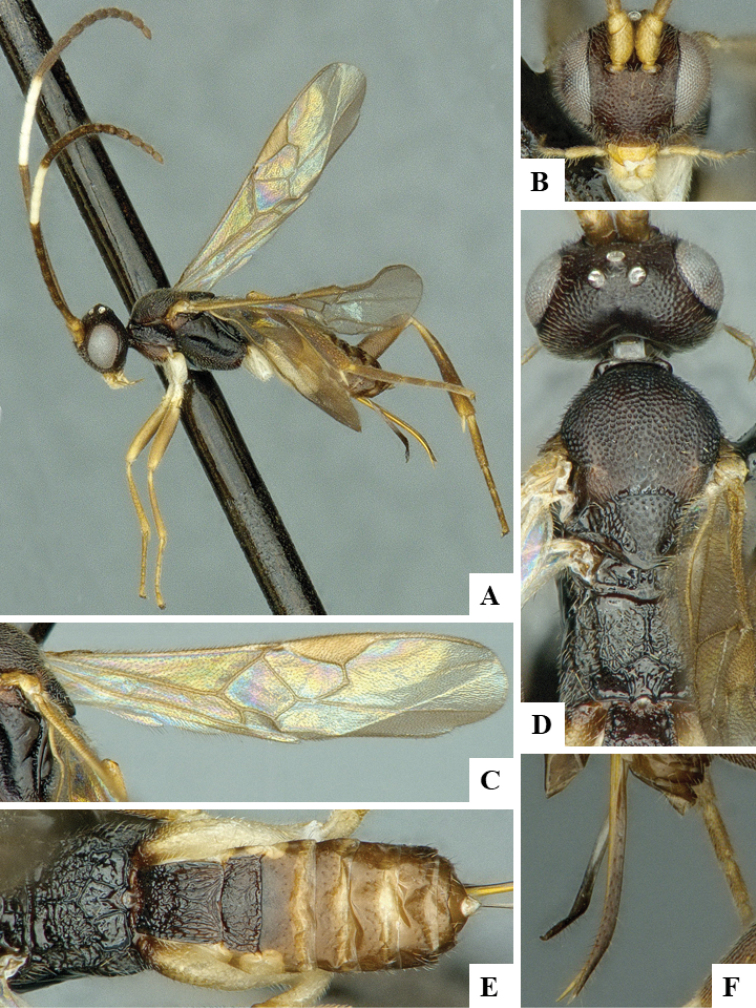
*Exulonyxcamma* female CNCHYM01205 **A** Habitus, lateral **B** Head, frontal **C** Fore wing **D** Head and mesosoma, dorsal **E** Propodeum and metasoma, dorsal **F** Ovipositor and ovipositor sheaths.

**Figure 96.**
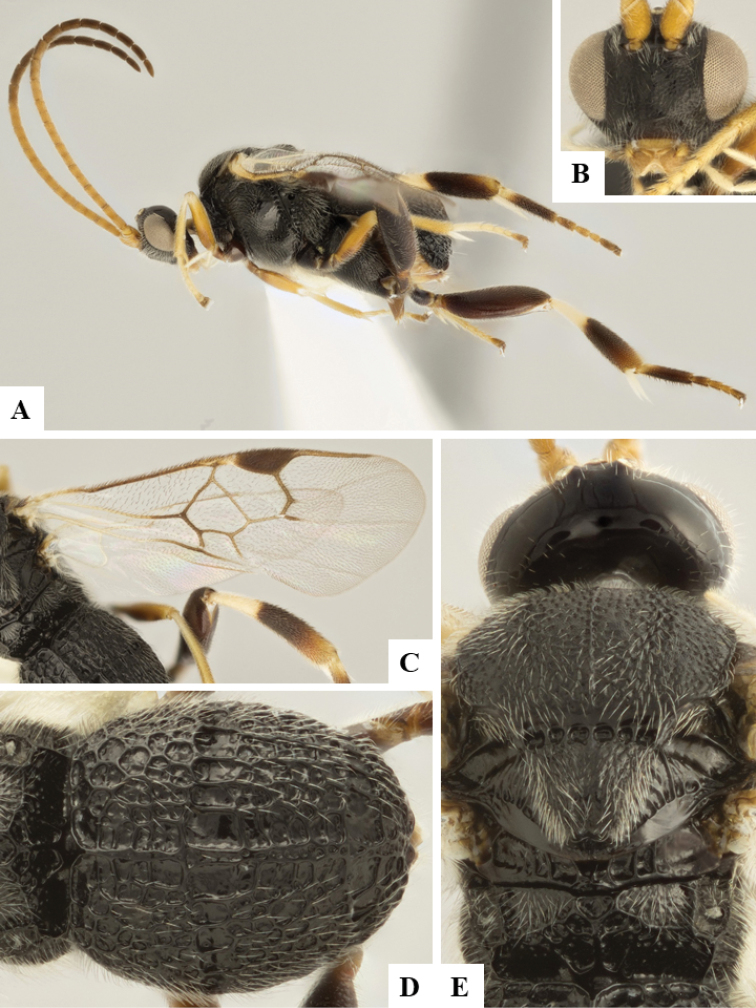
*Forniciajarmilae* female holotype **A** Habitus, lateral **B** Head, frontal **C** Fore wing **D** Metasoma, dorsal **E** Mesosoma, dorsal.

**Figure 97.**
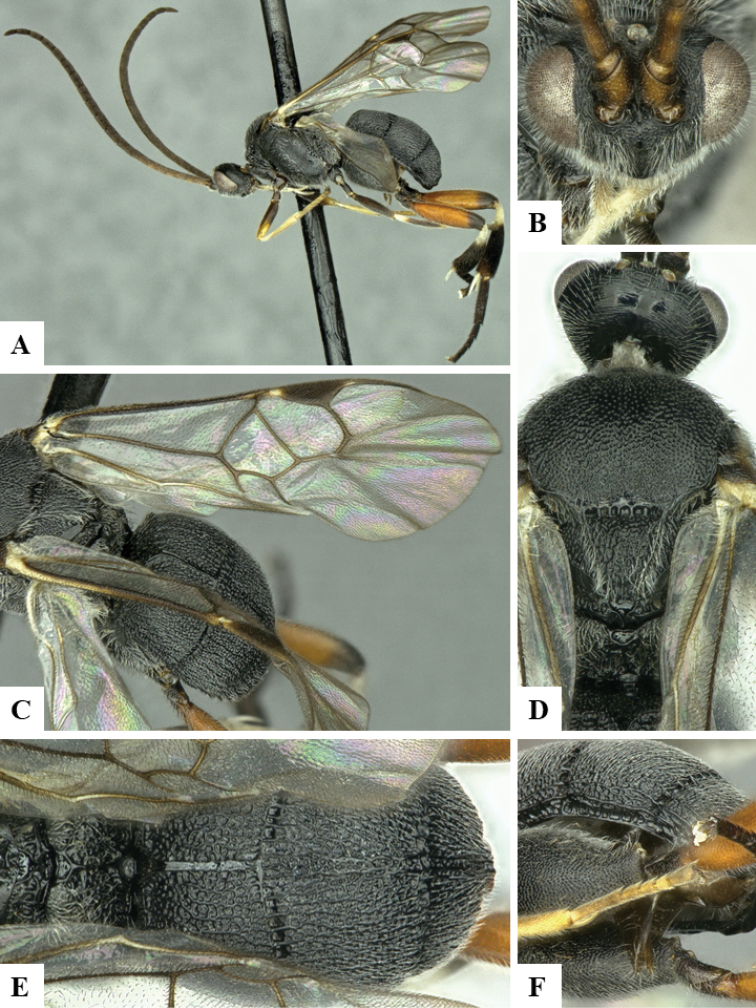
*Fornicia* sp. female CNCHYM01223 **A** Habitus, lateral **B** Head, frontal **C** Fore wing **D** Mesosoma, dorsal **E** Propodeum and metasoma, dorsal **F** Ovipositor and ovipositor sheaths.

**Figure 98.**
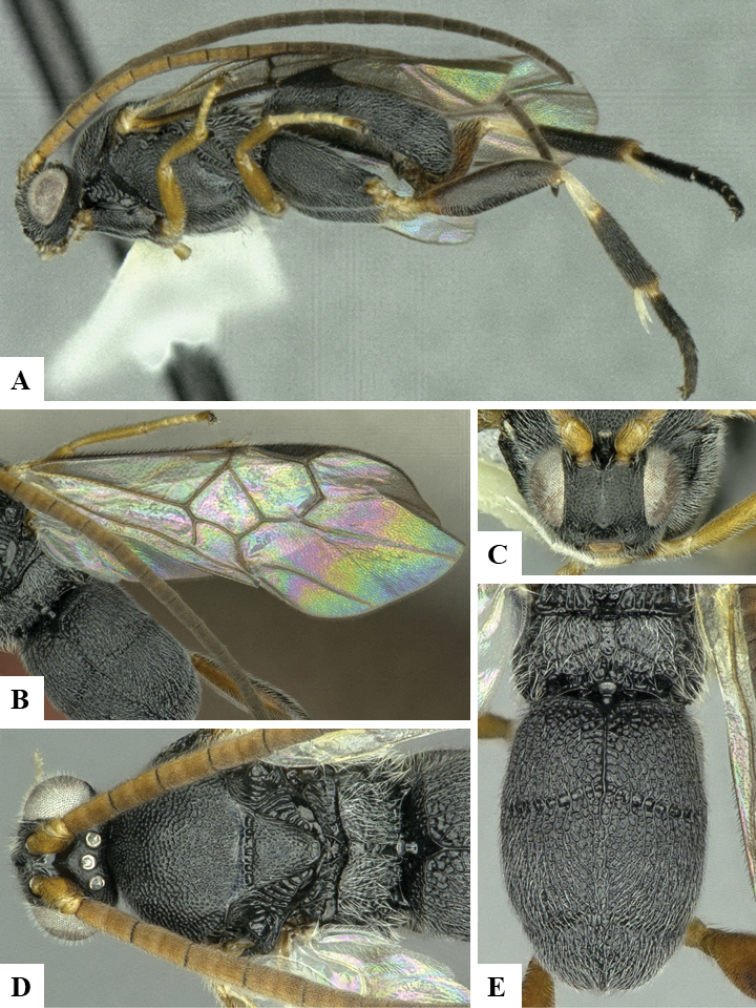
*Fornicia* sp. male JMIC 0049 **A** Habitus, lateral **B** Fore wing **C** Head, frontal **D** Head and mesosoma, dorsal **E** Propodeum and metasoma, dorsal.

**Figure 99.**
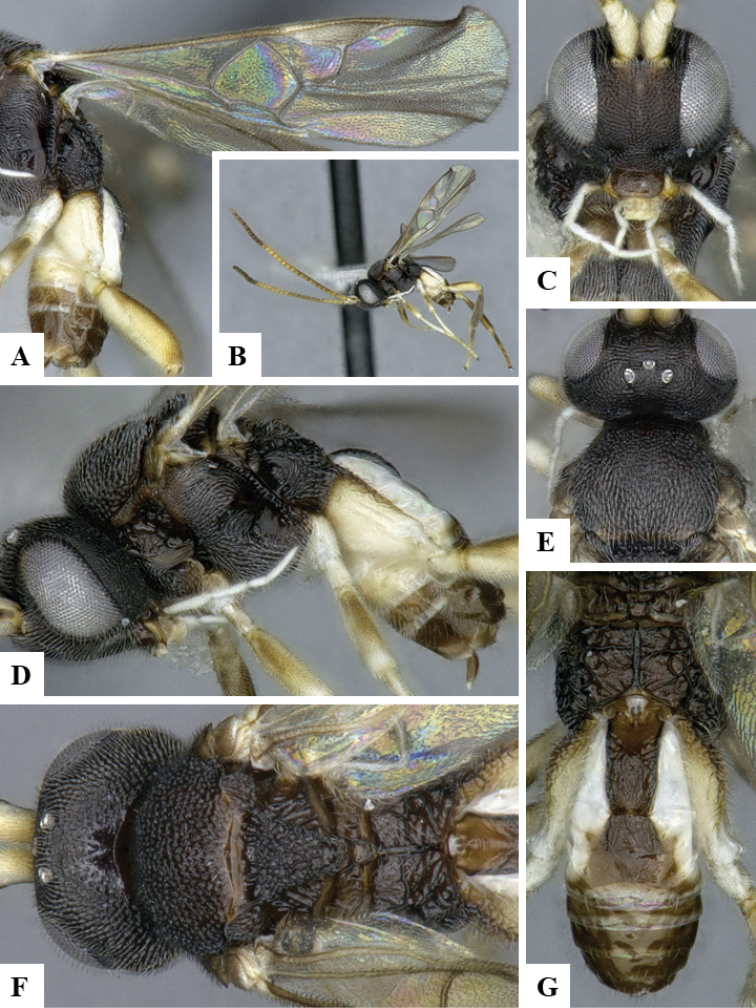
*Gilbertnixoniusbiem* female holotype **A** Fore wing **B** Habitus, lateral **C** Head, frontal **D** Head, mesosoma and metasoma, lateral **E** Head and mesosoma, dorsal **F** Mesosoma, dorsal **G** Propodeum and metasoma, dorsal.

**Figure 100.**
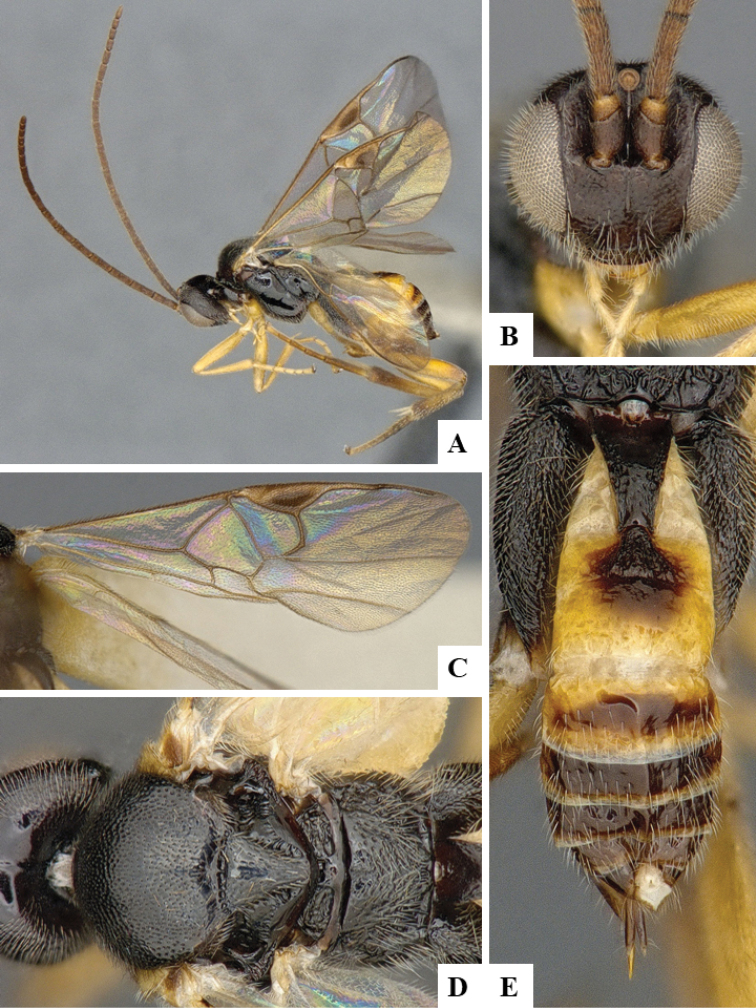
*Glyptapantelesaliphera* female CNCHYM01229 **A** Habitus, lateral **B** Head, frontal **C** Fore wing **D** Mesosoma, dorsal **E** Metasoma, dorsal.

**Figure 101.**
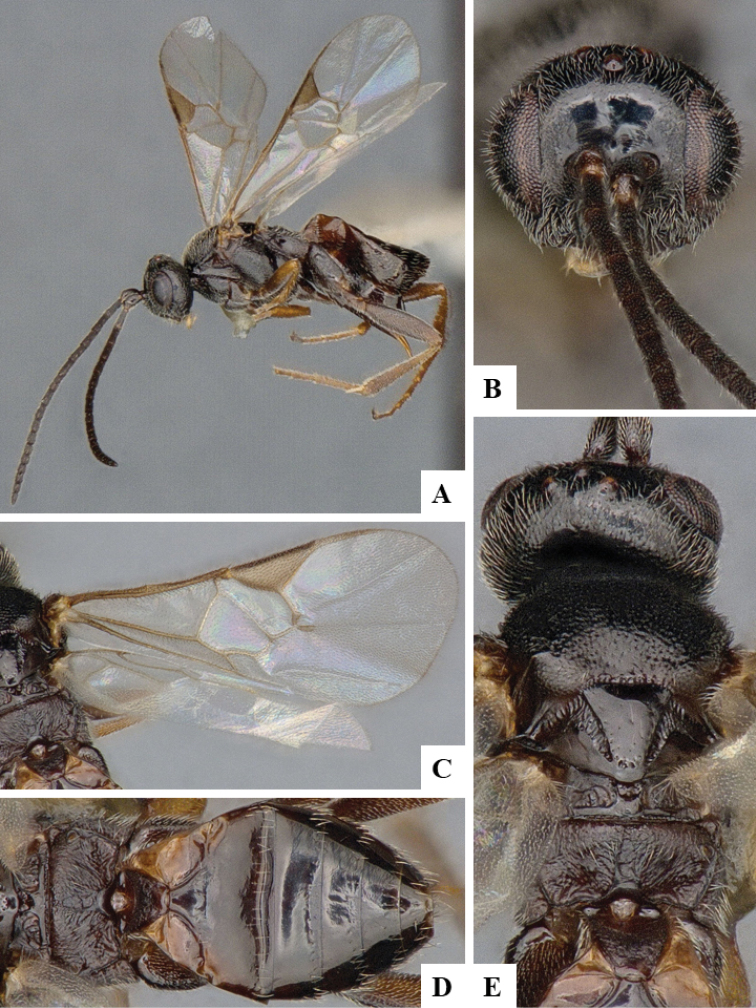
*Glyptapantelesbourquini* female CNCHYM01239 **A** Habitus, lateral **B** Head, frontodorsal **C** Fore wing **D** Propodeum and metasoma, dorsal **E** Mesosoma, dorsal.

**Figure 102.**
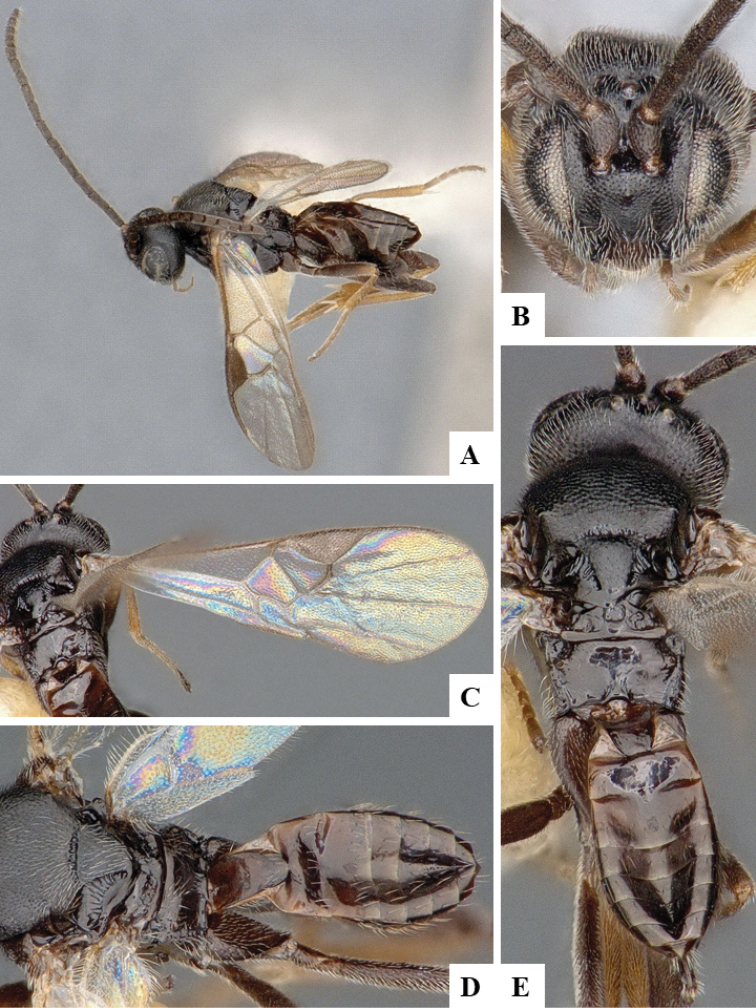
*Glyptapantelesfraternus* female CNC497049 **A** Habitus, lateral **B** Head, frontal **C** Fore wing **D** Mesosoma and metasoma, dorsal **E** Mesosoma and metasoma, dorsal.

**Figure 103.**
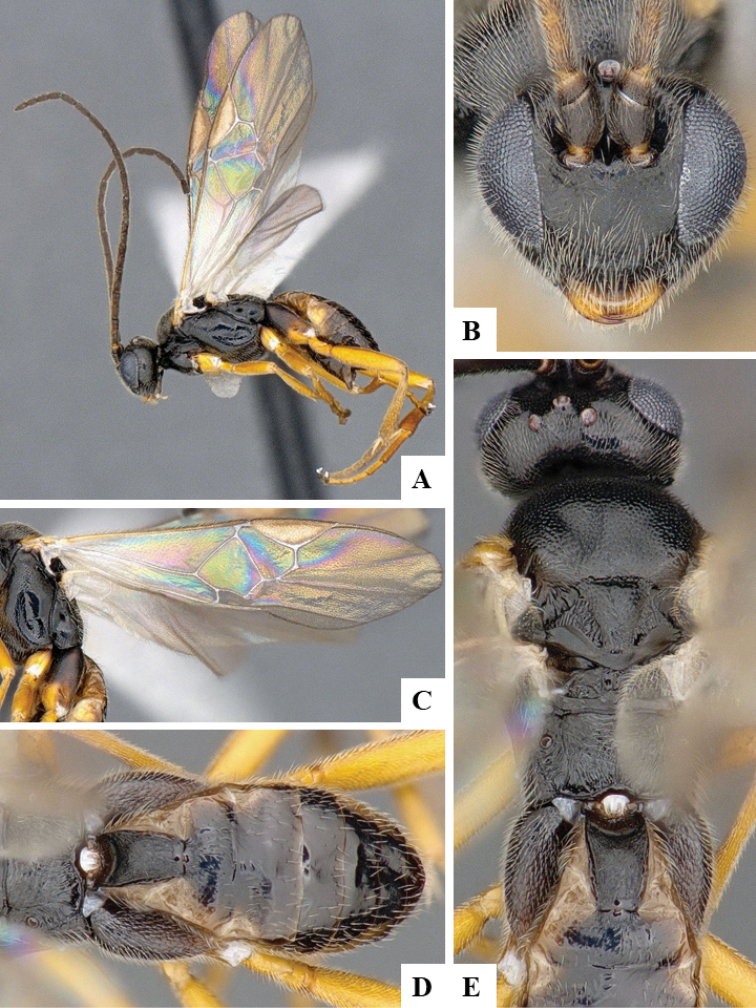
*Glyptapantelesfulvipes* female MRSJFT0427 **A** Habitus, lateral **B** Head, frontal **C** Fore wing **D** Metasoma, dorsal **E** Head, mesosoma and tergites 1–2, dorsal.

**Figure 104.**
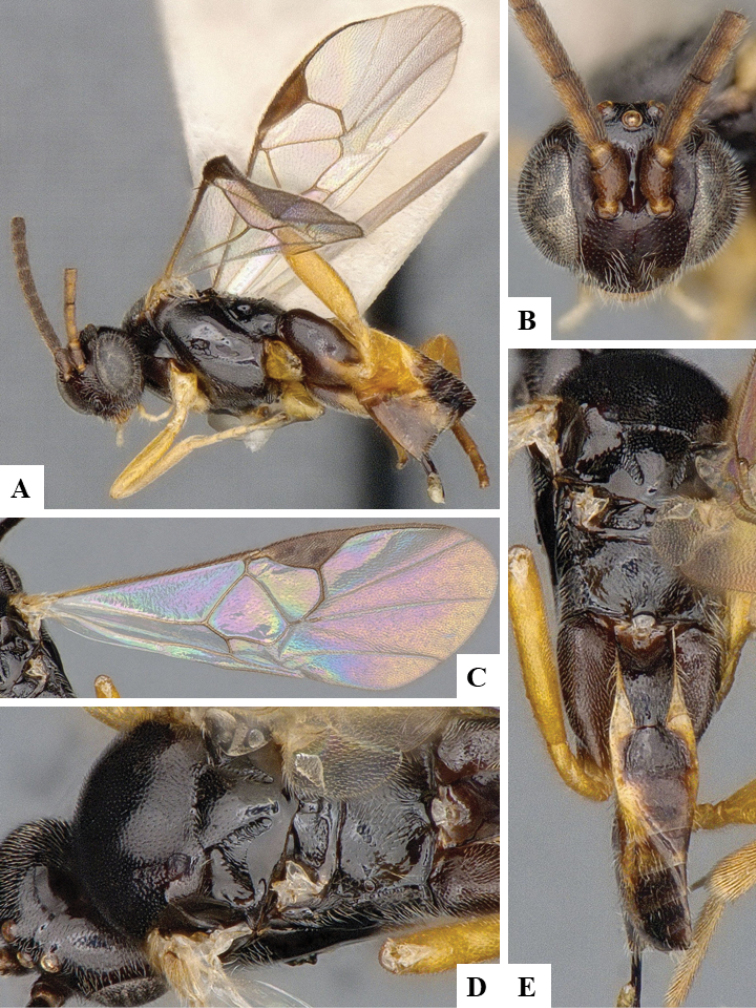
*Glyptapantelesindiensis* female paratype CNCHYM03231 **A** Habitus, lateral **B** Head, frontal **C** Fore wing **D** Mesosoma, dorsolateral **E** Propodeum and metasoma, dorsal.

**Figure 105.**
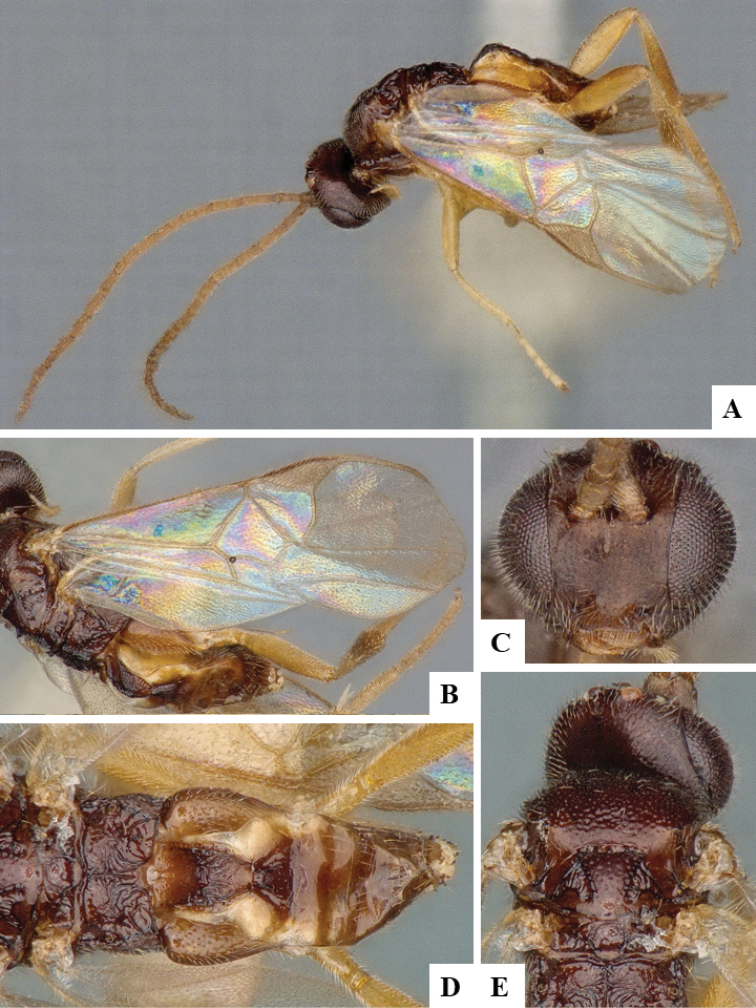
*Glyptapantelesluteipennis* female paratype CNC679221 **A** Habitus, lateral **B** Fore wing **C** Head, frontal **D** Propodeum and metasoma, dorsal **E** Mesosoma, dorsal.

**Figure 106.**
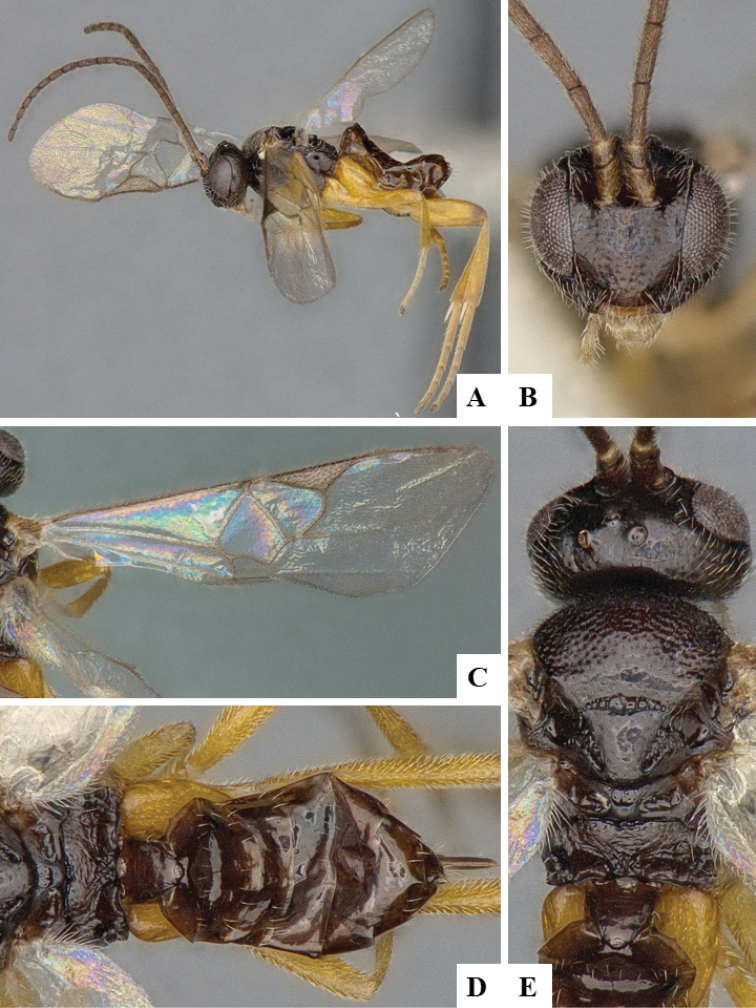
*Glyptapantelesmilitaris* female CNC679219 **A** Habitus, lateral **B** Head, frontal **C** Fore wing **D** Propodeum and metasoma, dorsal **E** Head and mesosoma, dorsal.

**Figure 107.**
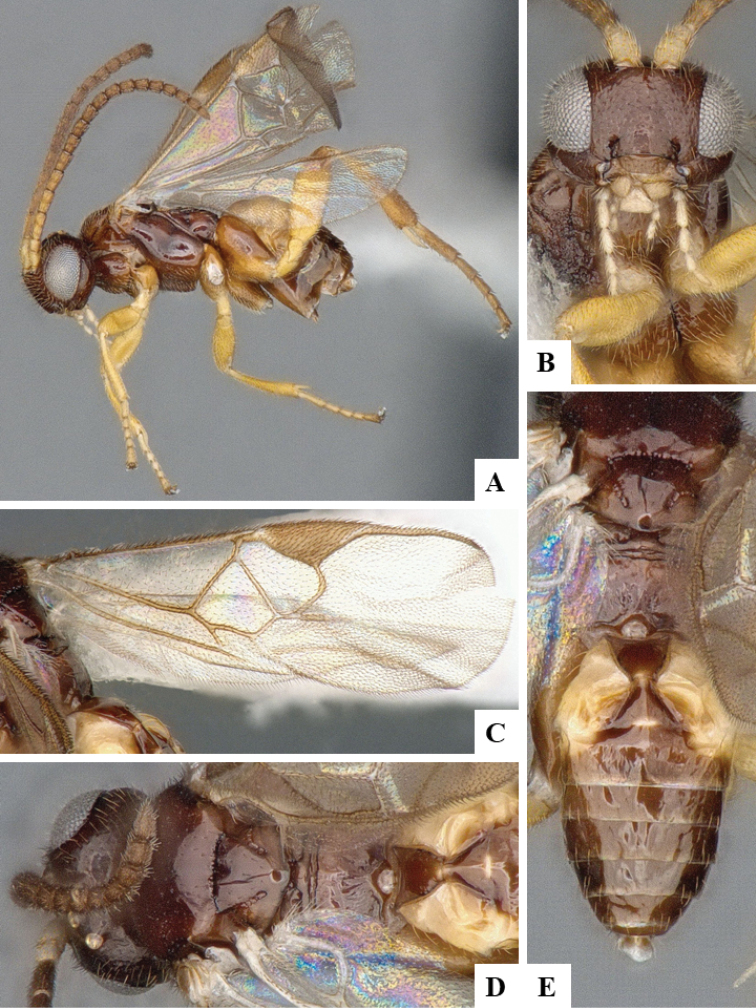
*Glyptapantelespolitus* female CNCH1334 **A** Habitus, lateral **B** Head, frontal **C** Fore wing **D** Mesosoma and tergites 1–2, dorsal **E** Propodeum and metasoma, dorsal.

**Figure 108.**
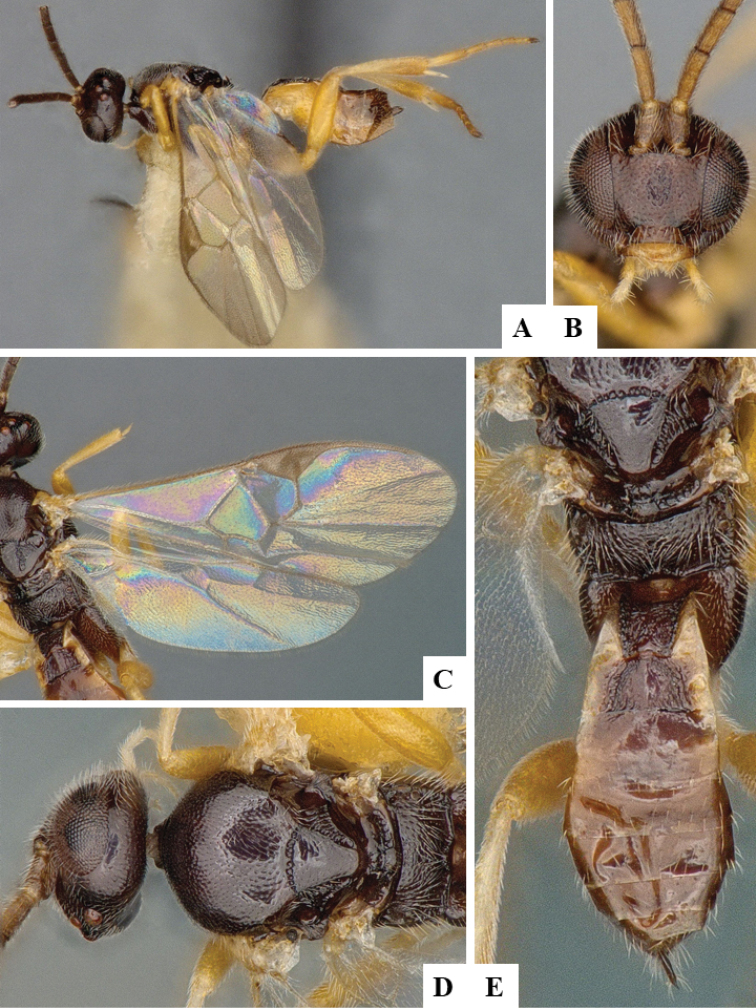
*Glyptapantelessarrothripae* female CNC679326 **A** Habitus, lateral **B** Head, frontal **C** Fore wing and hind wing **D** Mesosoma, dorsal **E** Propodeum and metasoma, dorsal.

**Figure 109.**
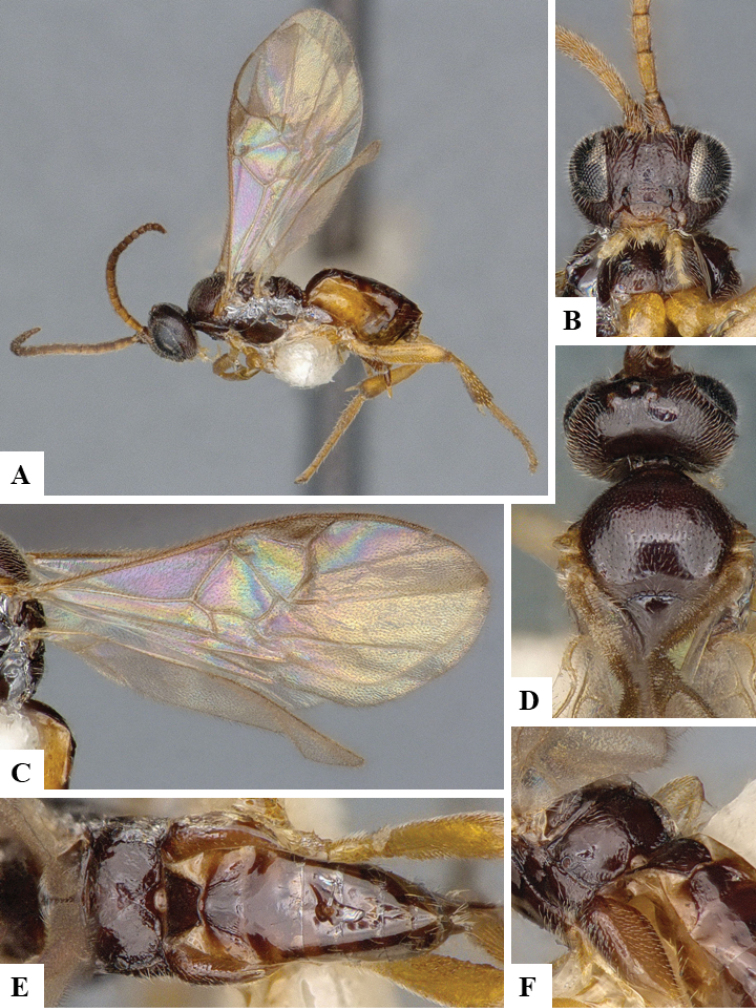
*Glyptapantelesthompsoni* female CNCHYM01350 **A** Habitus, lateral **B** Head, frontal **C** Fore wing **D** Head and mesosoma, dorsal **E** Propodeum and metasoma, dorsal **F** Propodeum, dorsolateral.

**Figure 110.**
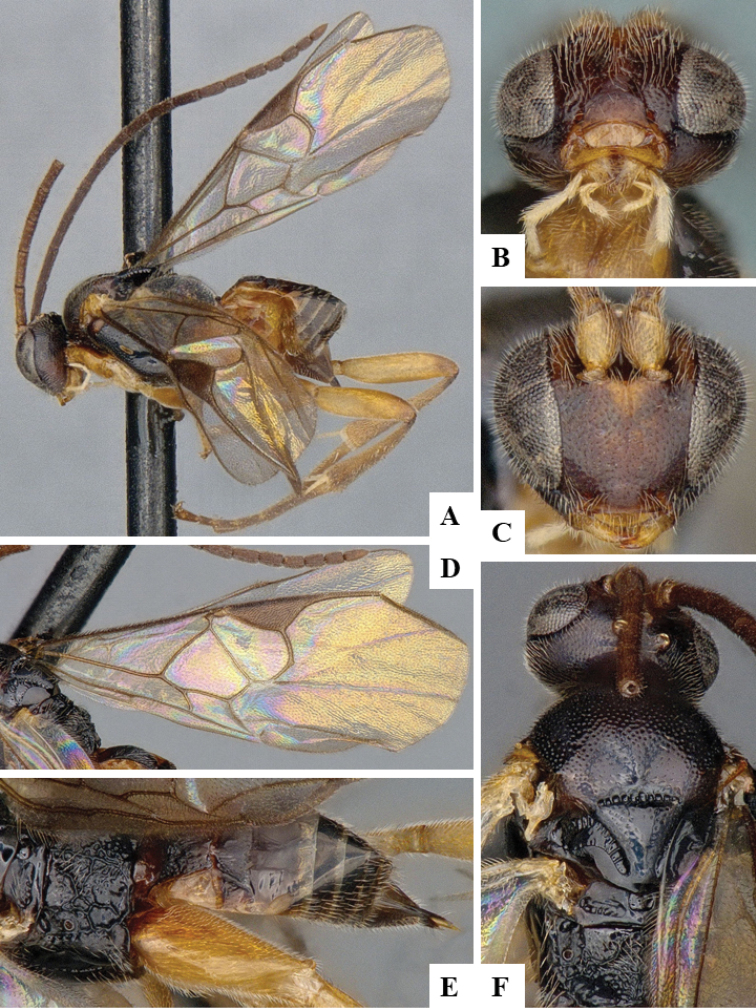
*Glyptapantelesvafer* female CNCHYM03251 **A** Habitus, lateral **B** Head, frontoventral **C** Head, frontal **D** Fore wing **E** Propodeum and metasoma, laterodorsal **F** Head and mesosoma, dorsal.

**Figure 111.**
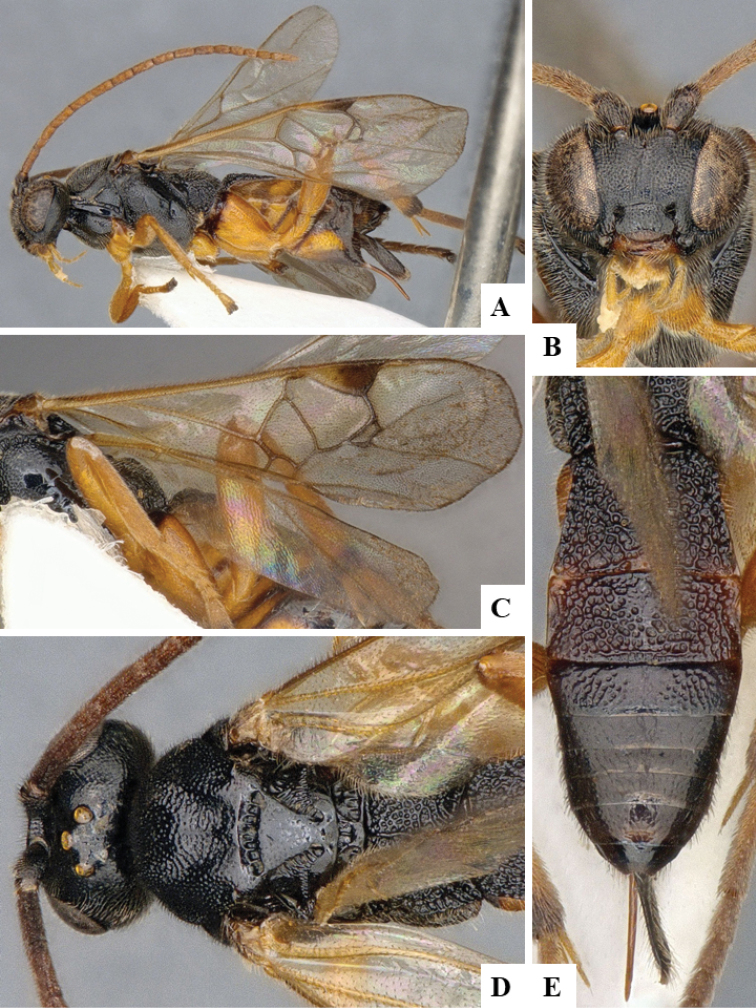
*Hygroplitisbasarukini* female paratype CNCHYM01362 **A** Habitus, lateral **B** Head, frontal **C** Fore wing and hind wing **D** Head and mesosoma, dorsal **E** Metasoma, dorsal.

**Figure 112.**
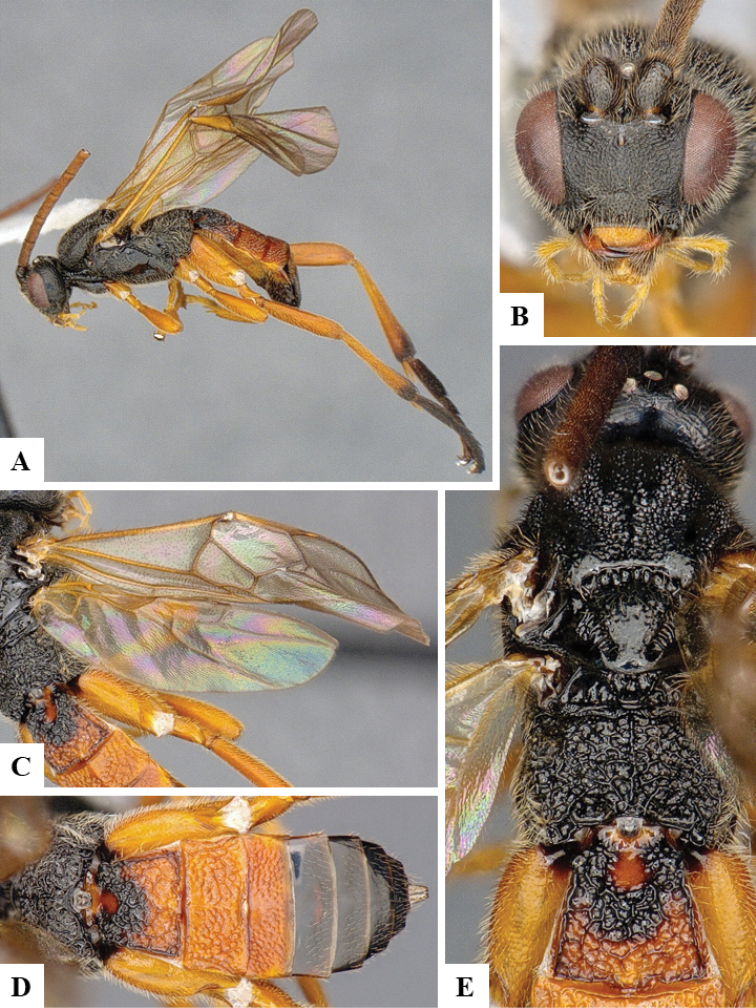
*Hygroplitispseudorussata* male MRSJFT0057 **A** Habitus, lateral **B** Head, frontal **C** Fore wing and hind wing **D** Metasoma, dorsal **E** Mesosoma and tergite 1, dorsal.

**Figure 113.**
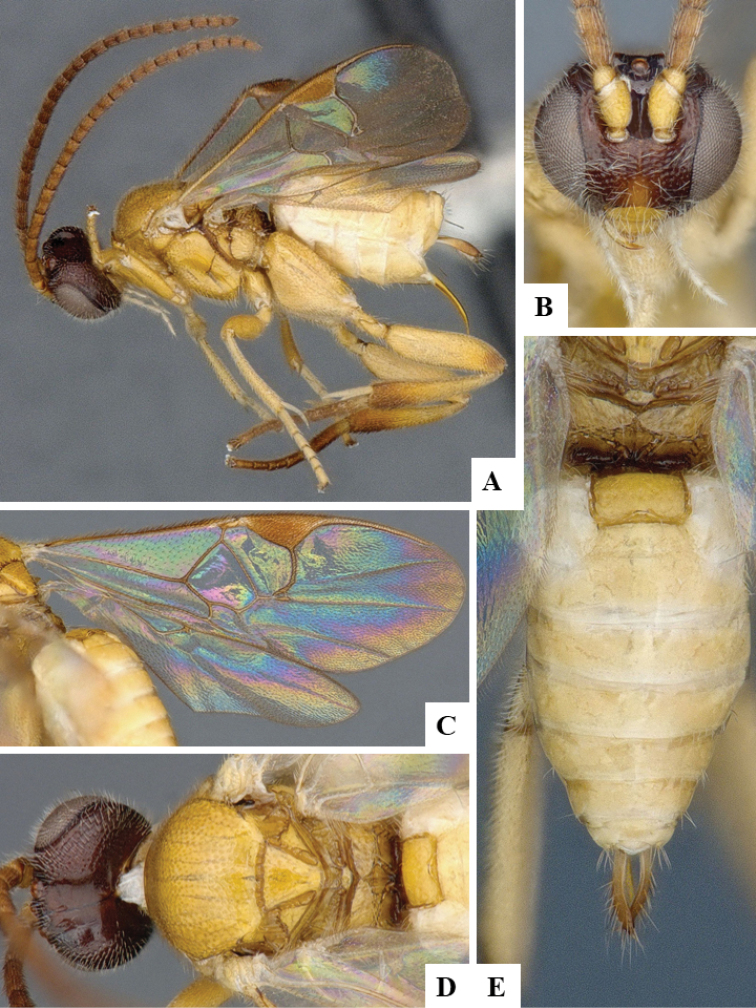
*Hypomicrogastercrocinus* female holotype **A** Habitus, lateral **B** Head, frontal **C** Fore wing and hind wing **D** Head and mesosoma, dorsal **E** Metasoma, dorsal.

**Figure 114.**
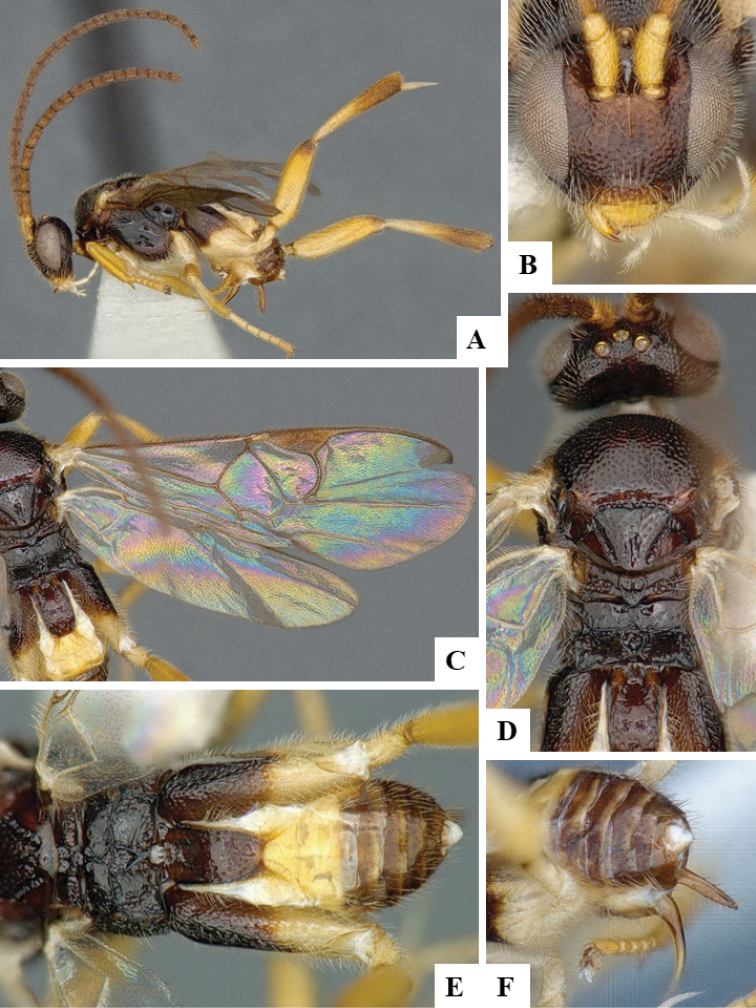
*Hypomicrogasterdeltis* female holotype **A** Habitus, lateral **B** Head, frontal **C** Fore wing and hind wing **D** Head and mesosoma, dorsal **E** Propodeum and metasoma, dorsal **F** Ovipositor and ovipositor sheaths.

**Figure 115.**
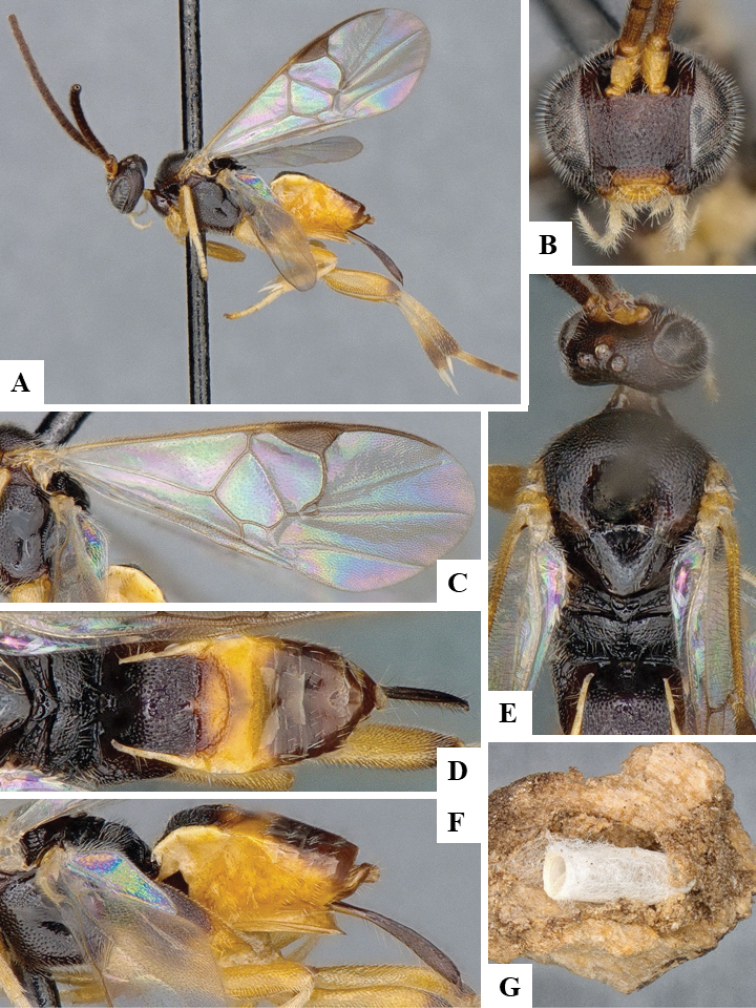
*Hypomicrogasterecdytolophae* female CNC482258 **A** Habitus, lateral **B** Head, frontal **C** Fore wing **D** Propodeum and metasoma, dorsal **E** Mesosoma, dorsal **F** Mesosoma, metasoma and ovipositor sheaths, lateral **G** Cocoon.

**Figure 116.**
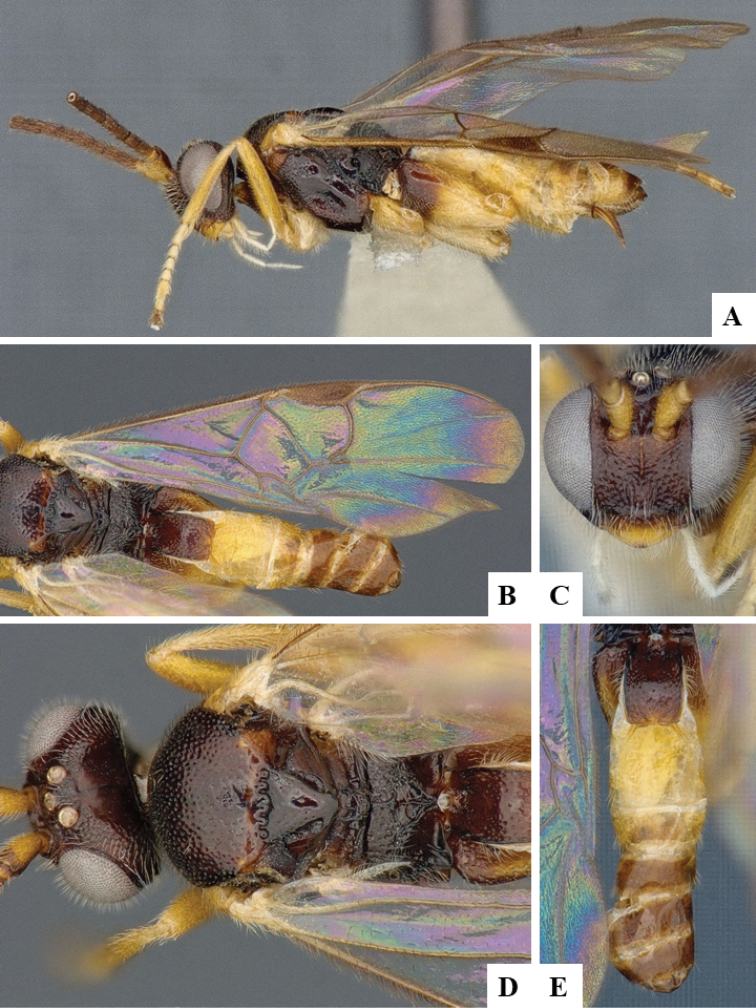
*Hypomicrogasterguille* female holotype **A** Habitus, lateral **B** Fore wing **C** Head, frontal **D** Head and mesosoma, dorsal **E** Metasoma, dorsal.

**Figure 117.**
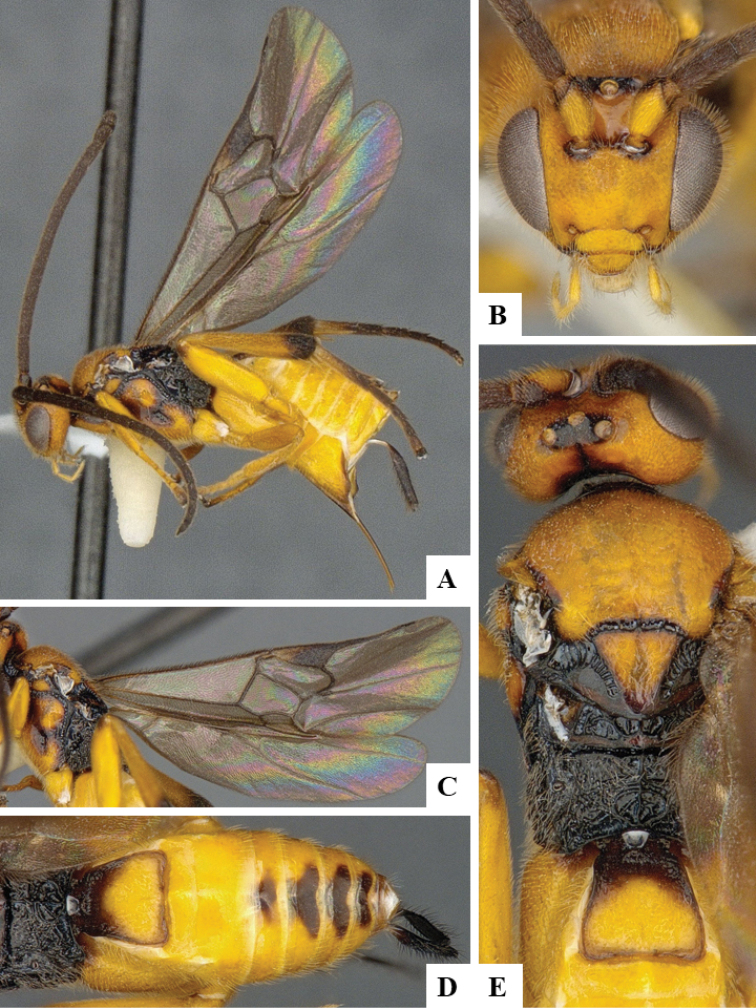
*Hypomicrogasterhektos* female holotype **A** Habitus, lateral **B** Head, frontal **C** Fore wing and hind wing **D** Metasoma, dorsal **E** Head, mesosoma and tergite 1, dorsal.

**Figure 118.**
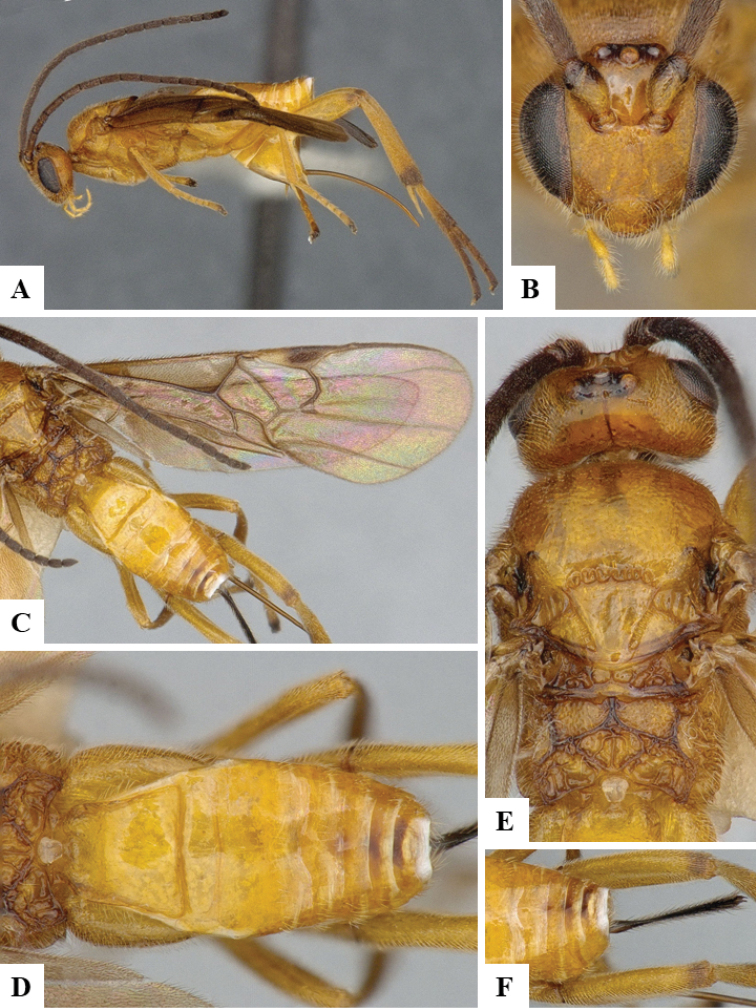
*Hypomicrogastermultus* female holotype **A** Habitus, lateral **B** Head, frontal **C** Fore wing **D** Metasoma, dorsal **E** Head and mesosoma, dorsal **F** Ovipositor sheaths.

**Figure 119.**
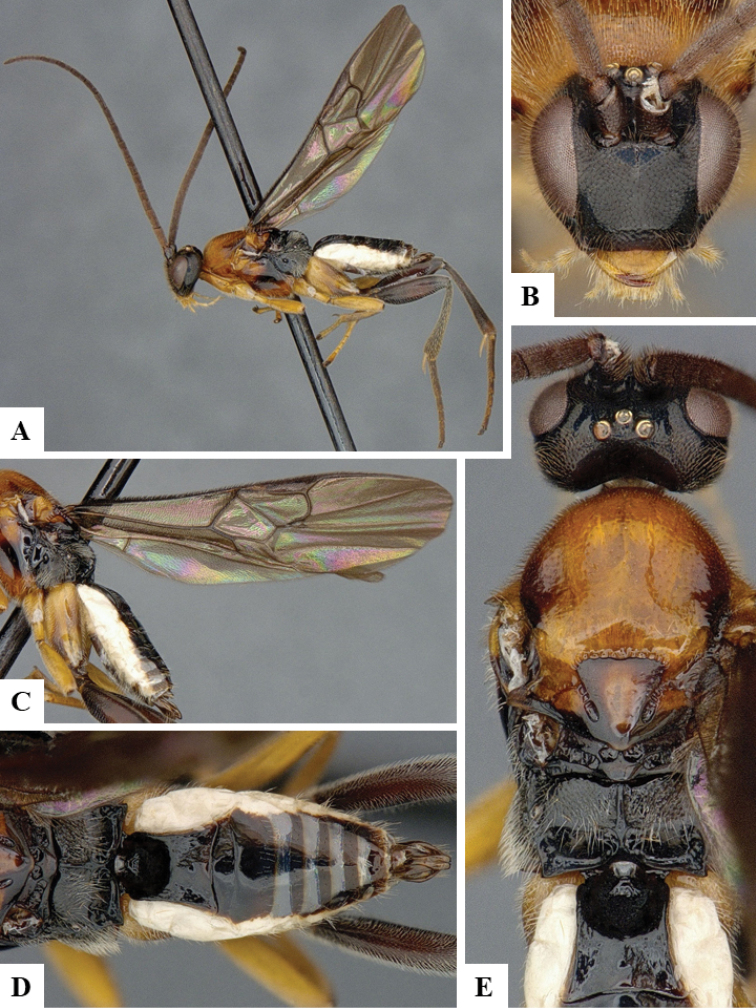
*Hypomicrogasterpectinatus* male holotype **A** Habitus, lateral **B** Head, frontal **C** Fore wing **D** Propodeum and metasoma, dorsal **E** Head and mesosoma, dorsal.

**Figure 120.**
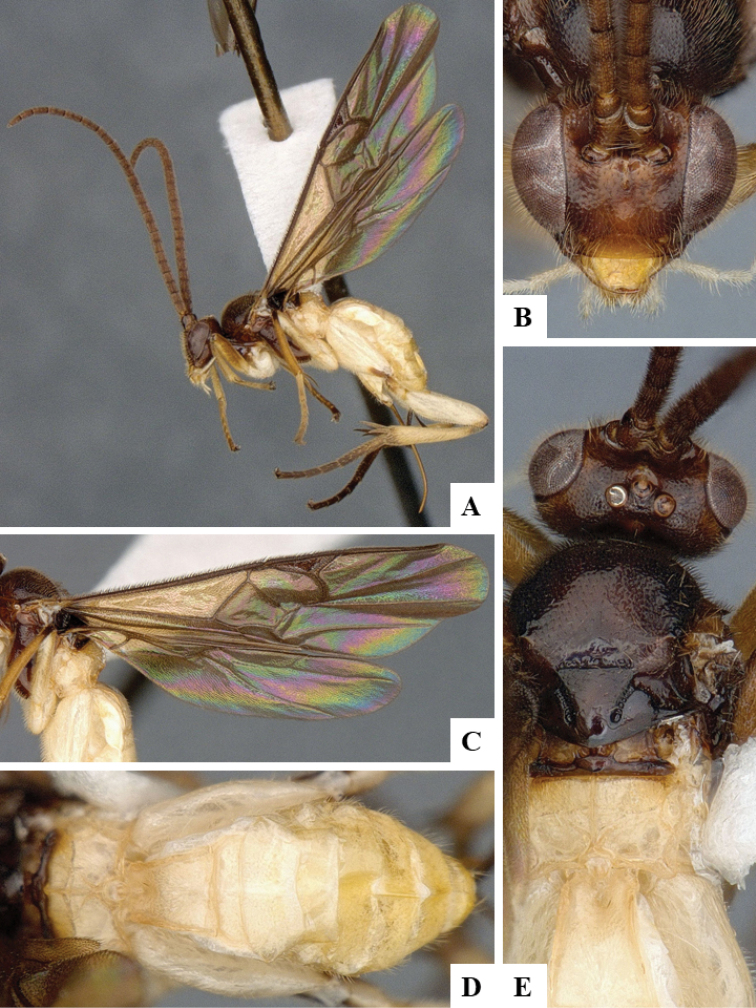
*Hypomicrogastersiderion* female holotype **A** Habitus, lateral **B** Head, frontal **C** Fore wing and hind wing **D** Propodeum and metasoma, dorsal **E** Head and mesosoma, dorsal.

**Figure 121.**
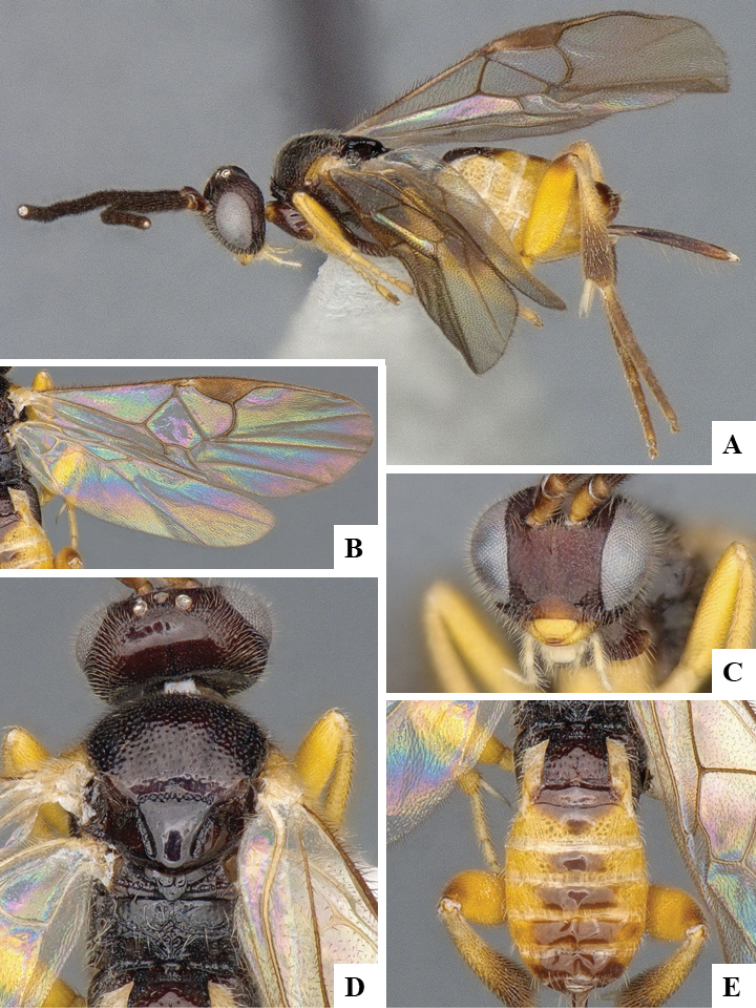
*Hypomicrogasterzonaria* female CNCHYM01436 **A** Habitus, lateral **B** Fore wing and hind wing **C** Head, frontal **D** Head and mesosoma, dorsal **E** Metasoma, dorsal.

**Figure 122.**
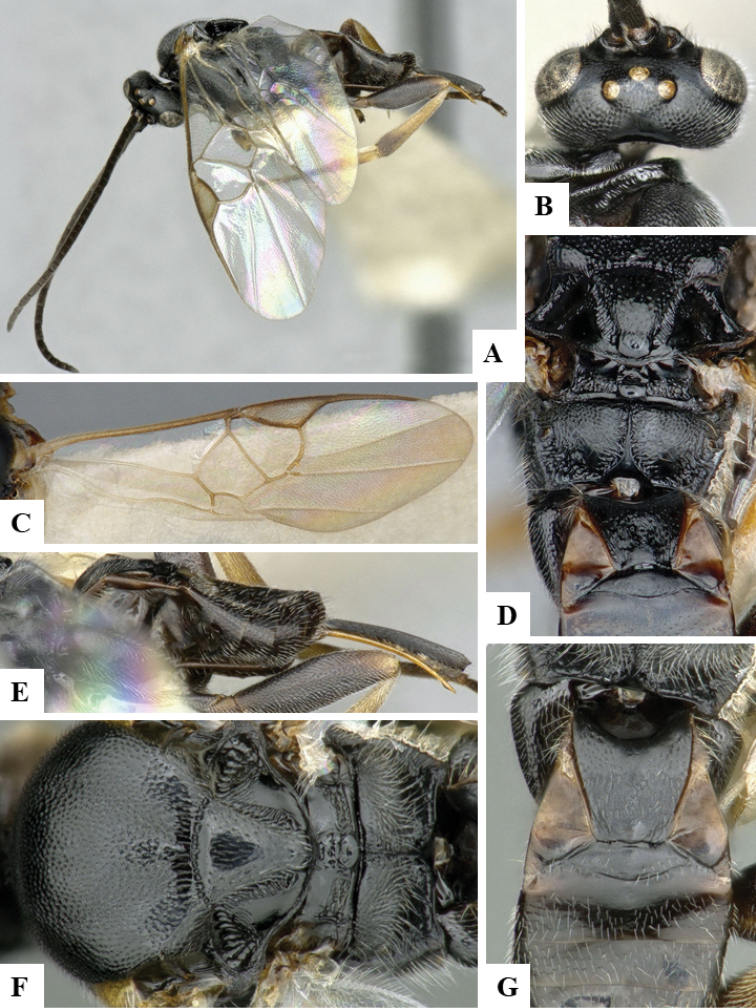
*Iconellacanadensis* female holotype **A** Habitus, lateral **B** Head, dorsal **C** Fore wing **D** Propodeum and tergites 1 to 2, dorsal **E** Metasoma, lateral **F** Mesosoma, dorsal **G** Tergites 1–4, dorsal.

**Figure 123.**
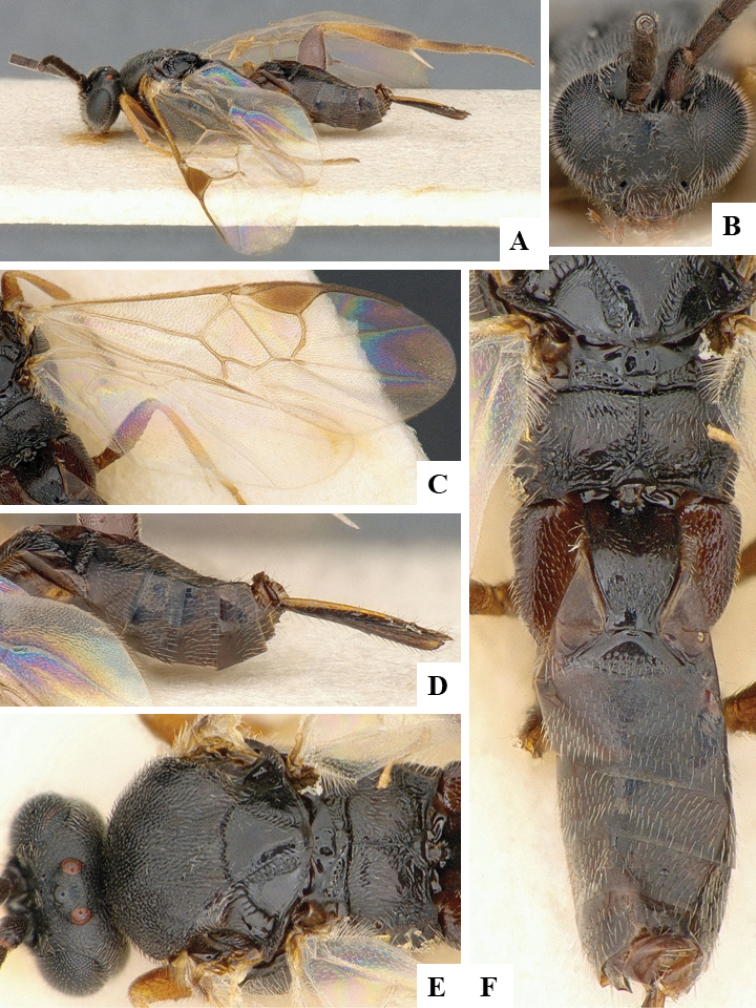
*Iconellamerula* female CNC474671 **A** Habitus, lateral **B** Head, frontal **C** Fore wing and hind wing **D** Metasoma, lateral **E** Head and mesosoma, dorsal **F** Propodeum and metasoma, dorsal.

**Figure 124.**
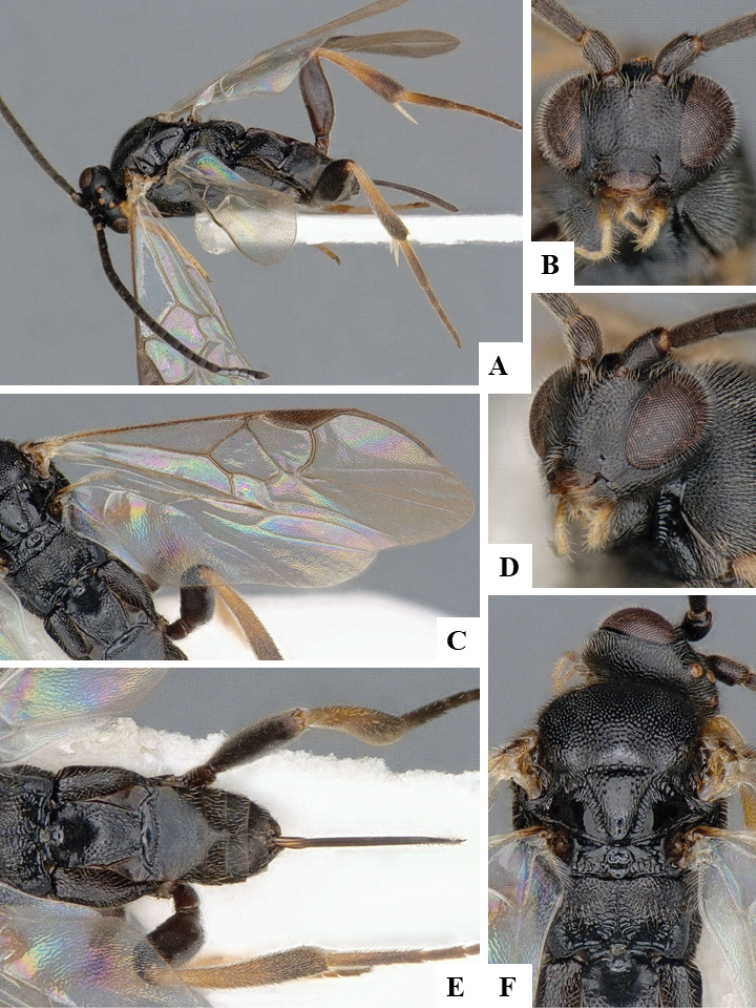
*Iconellavindicia* female CNCHYM01472 **A** Habitus, dorsolateral **B** Head, frontal **C** Fore wing and hind wing **D** Head, frontolateral **E** Metasoma, dorsal **F** Mesosoma, dorsal.

**Figure 125.**
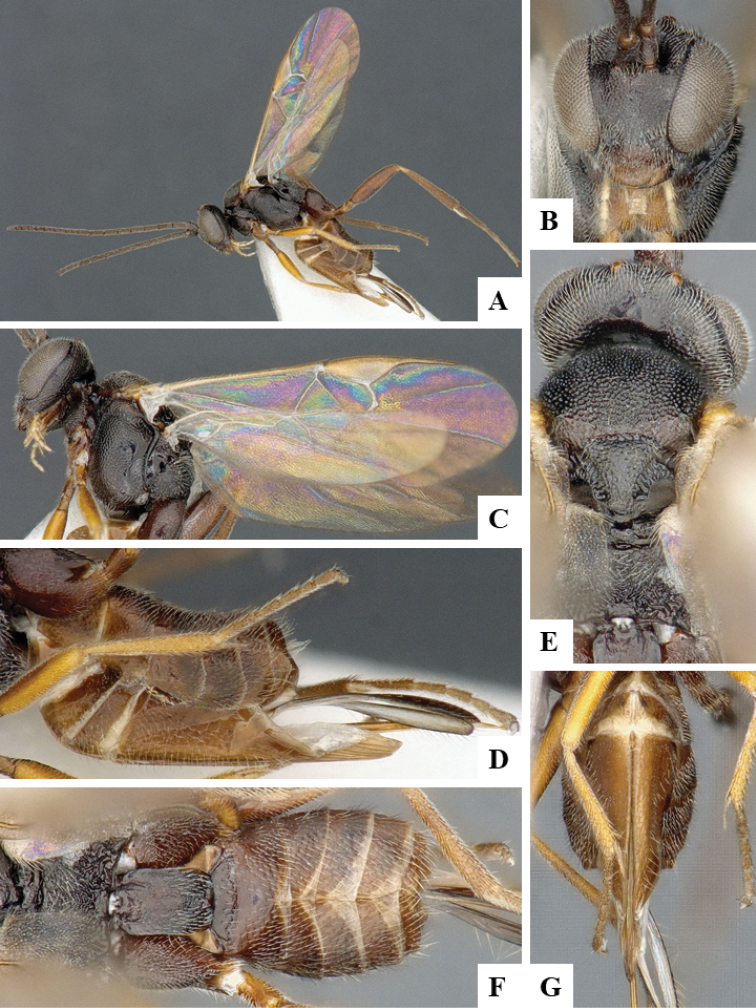
*Illidopsalbostigmalis* female holotype **A** Habitus, lateral **B** Head, frontal **C** Fore wing and hind wing **D** Metasoma, lateral **E** Mesosoma, dorsal **F** Propodeum and metasoma, dorsal **G** Hypopygium, ventral.

**Figure 126.**
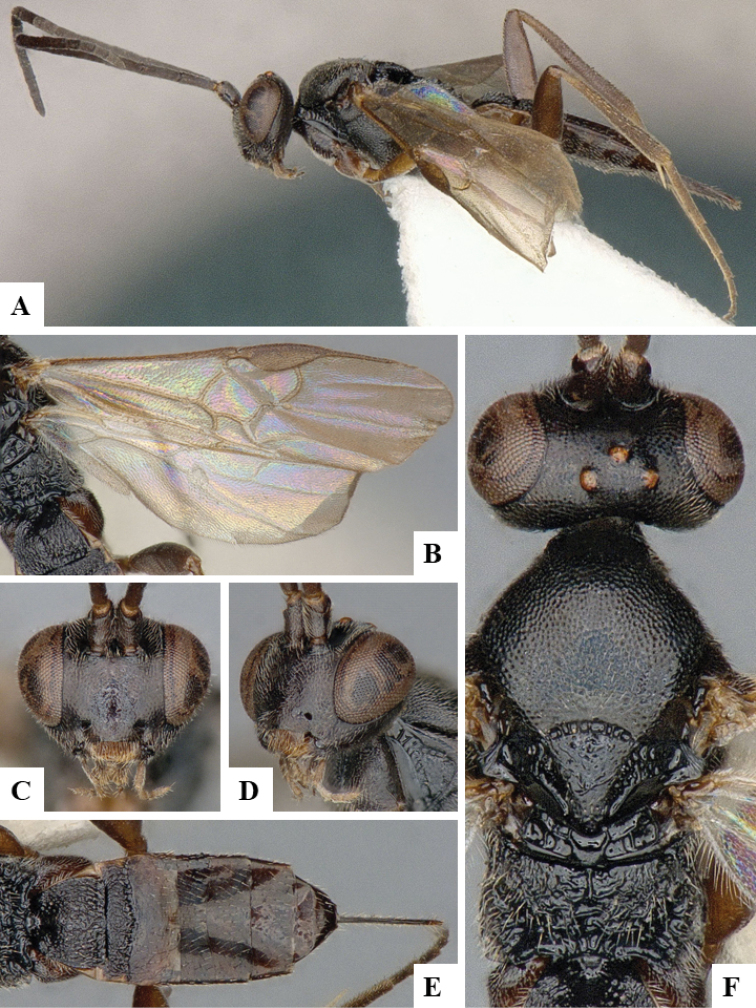
*Illidopssuevus* female CNCHYM01526 **A** Habitus, lateral **B** Fore wing and hind wing **C** Head, frontal **D** Head, frontolateral **E** Metasoma, dorsal **F** Head and mesosoma, dorsal.

**Figure 127.**
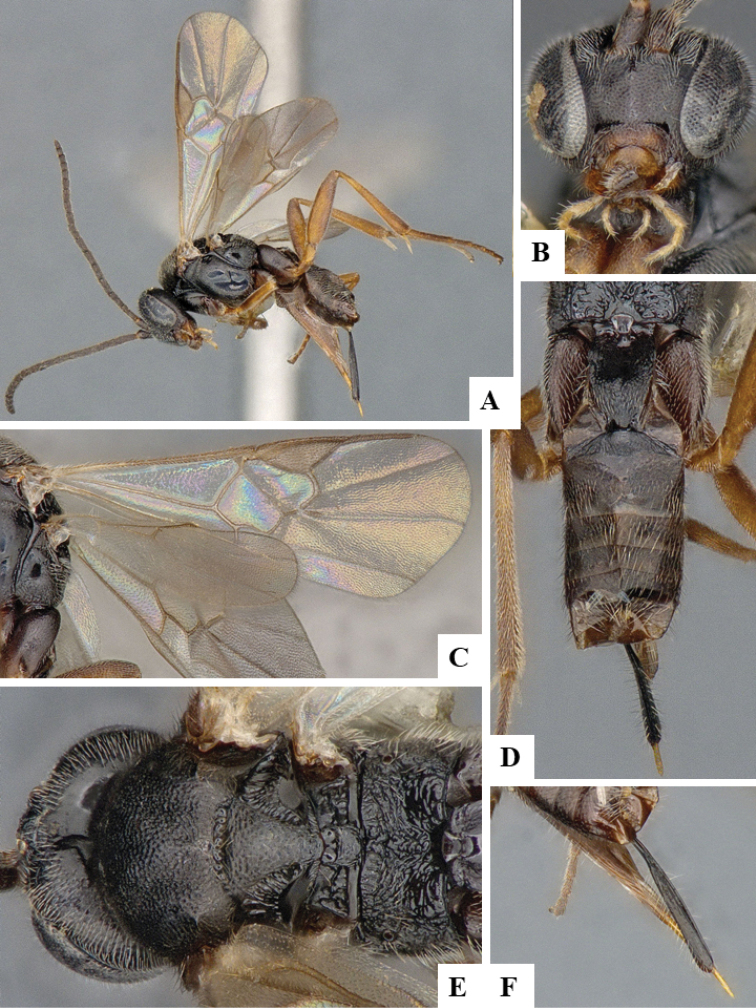
*Illidopsterrestris* female paratype CNCHYM01522 **A** Habitus, lateral **B** Head, frontal **C** Fore wing **D** Metasoma, dorsal **E** Mesosoma, dorsal **F** Ovipositor and ovipositor sheaths.

**Figure 128.**
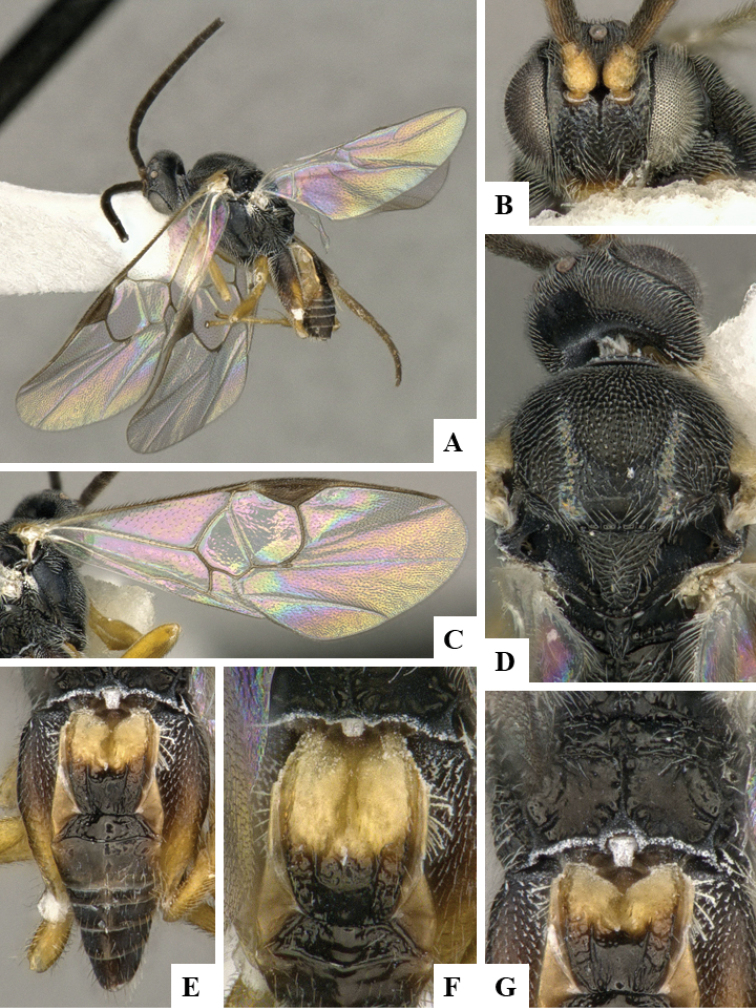
*Janhalacastedanieli* male holotype **A** Habitus, lateral **B** Head, frontal **C** Fore wing **D** Mesosoma, dorsal **E** Metasoma, dorsal **F** Tergites 1–2, dorsal **G** Propodeum and tergite 1, dorsal.

**Figure 129.**
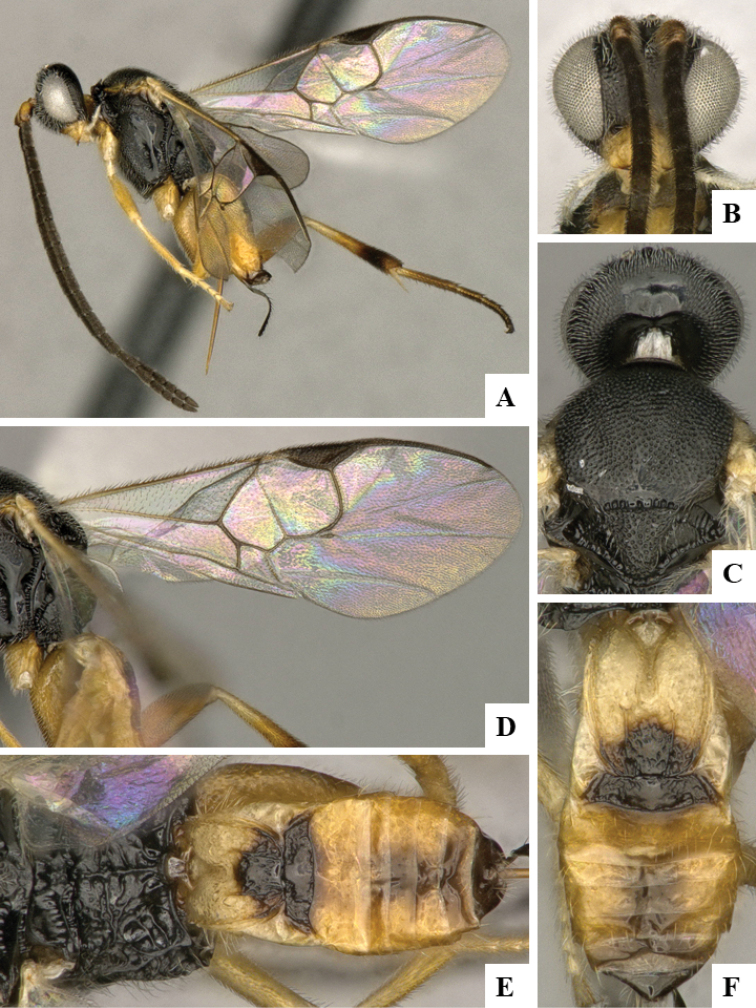
*Janhalacastewinnieae* female holotype **A** Habitus, lateral **B** Head, frontal **C** Mesosoma, dorsal **D** Fore wing **E** Propodeum and metasoma, dorsal **F** Metasoma, dorsal.

**Figure 130.**
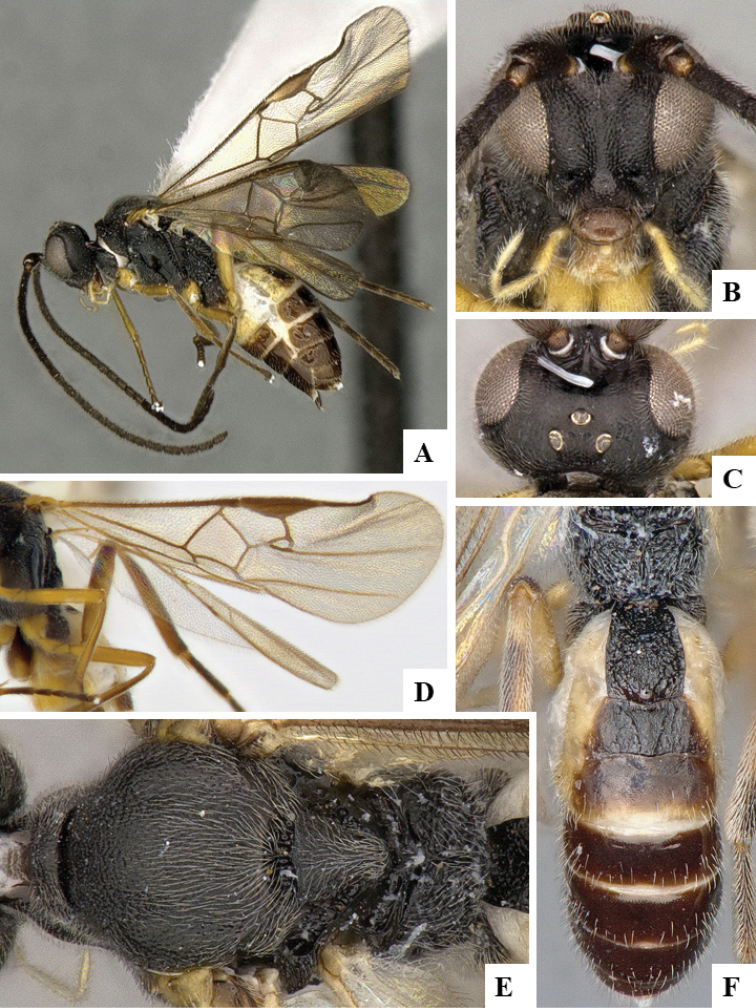
*Jenopappiusaethiopica* female CNC878534 **A** Habitus, lateral **B** Head, frontal **C** Head, dorsal **D** Fore wing **E** Mesosoma, dorsal **F** Metasoma, dorsal.

**Figure 131.**
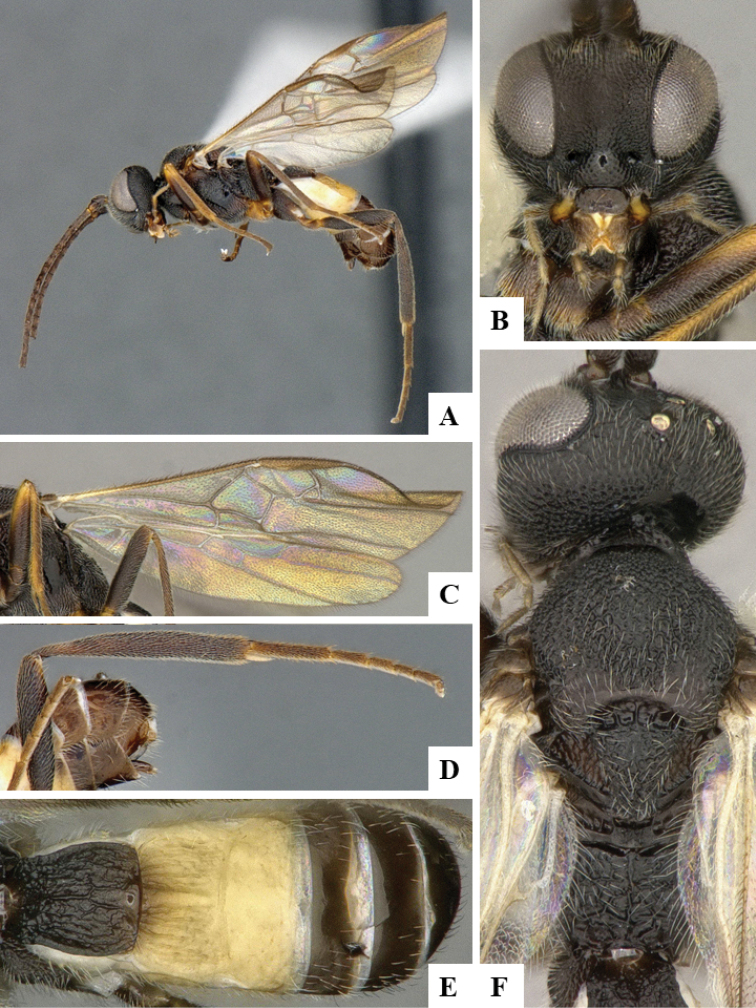
*Jenopappiusmagyarmuzeum* female holotype **A** Habitus, lateral **B** Head, frontal **C** Fore wing and hind wing **D** Hind leg and apex of metasoma, lateral **E** Metasoma, dorsal **F** Mesosoma, dorsal.

**Figure 132.**
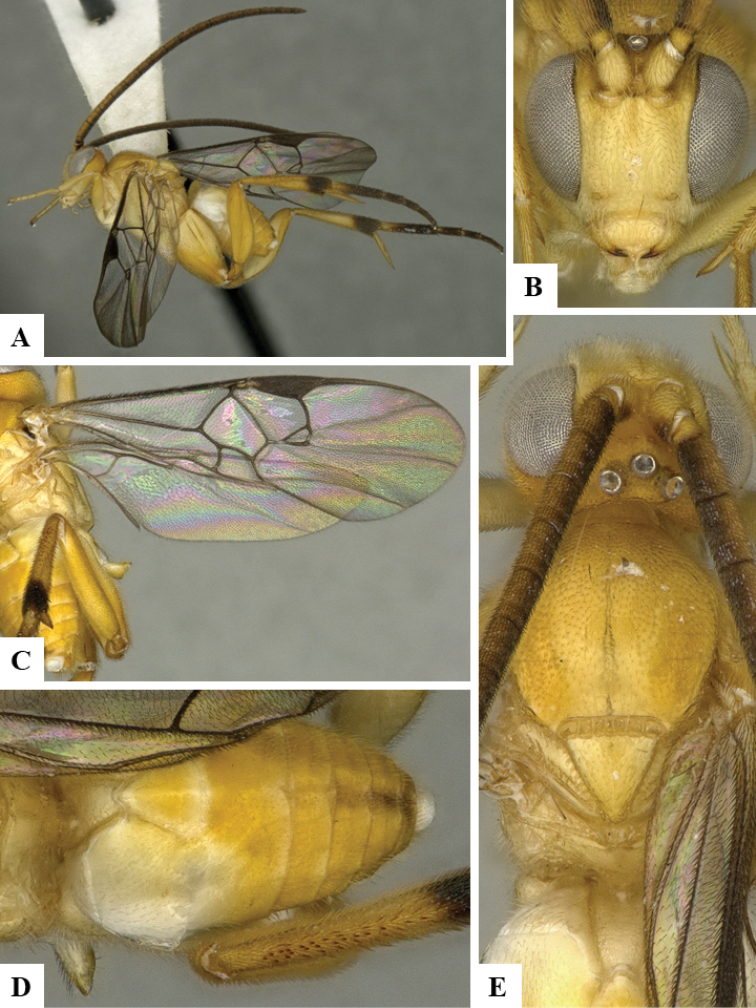
*Jimwhitfieldiusjamesi* female holotype **A** Habitus, lateral **B** Head, frontal **C** Fore wing and hind wing **D** Metasoma, dorsal **E** Head and mesosoma, dorsal.

**Figure 133.**
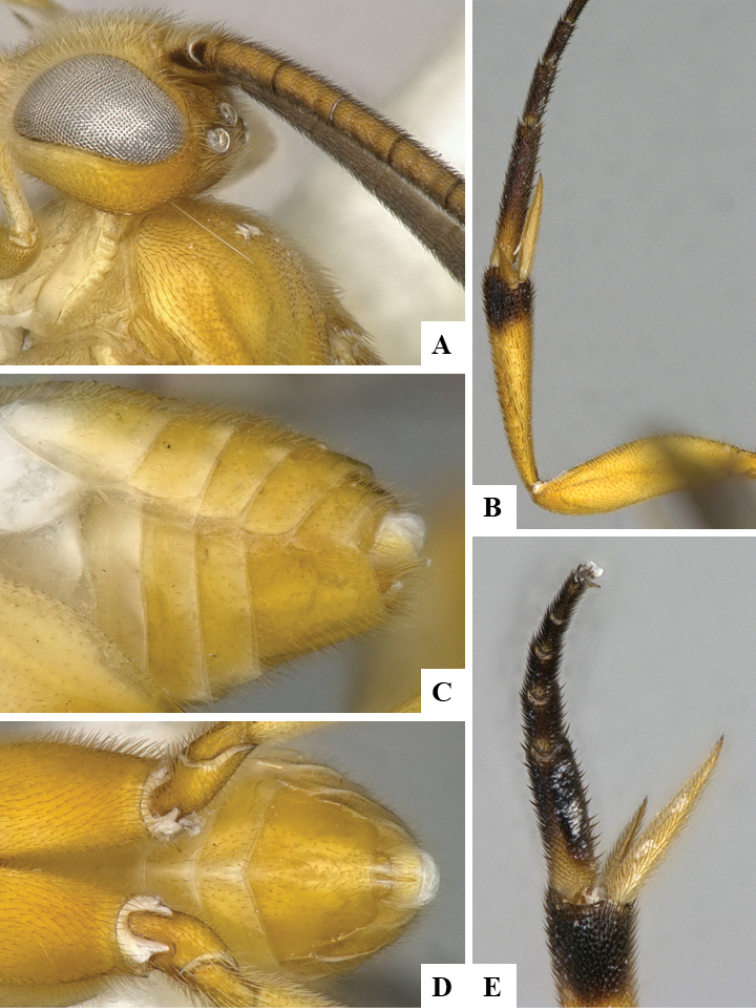
*Jimwhitfieldiusjamesi* female holotype **A** Antennal flagellomeres 1–4 **B** Hind leg **C** Hypopygium and ovipositor, lateral **D** Hypopygium and ovipositor, ventral **E** Inner and outer spines of metatibia.

**Figure 134.**
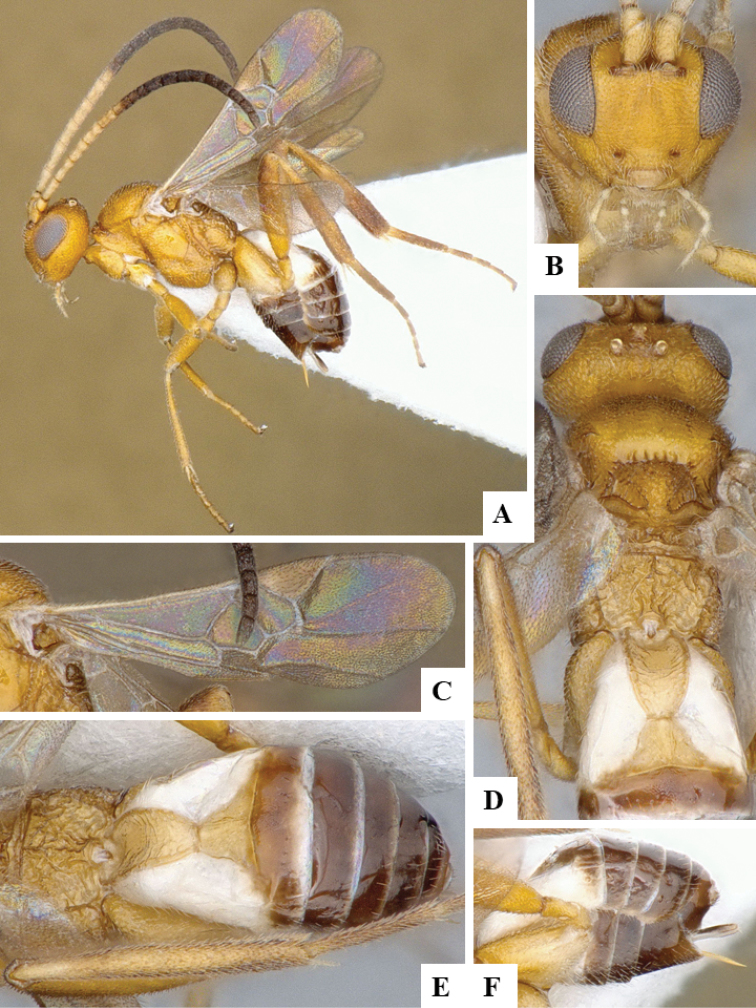
*Keylimepiehadhramautensis* female holotype **A** Habitus, lateral **B** Head, frontal **C** Fore wing **D** Head, mesosoma and tergites 1–3, dorsal **E** Propodeum and metasoma, dorsal **F** Metasoma, lateral.

**Figure 135.**
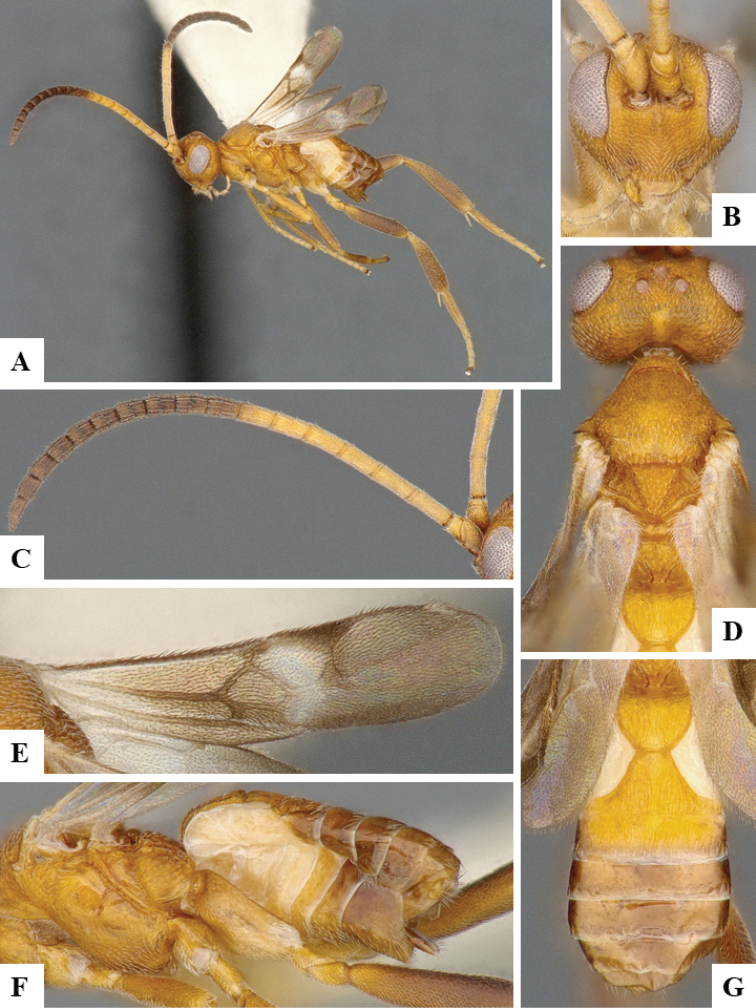
*Keylimepiepeckorum* female CNC483615 **A** Habitus, lateral **B** Head, frontal **C** Antenna **D** Head and mesosoma, dorsal **E** Fore wing **F** Mesosoma and metasoma, lateral **G** Metasoma, dorsal.

**Figure 136.**
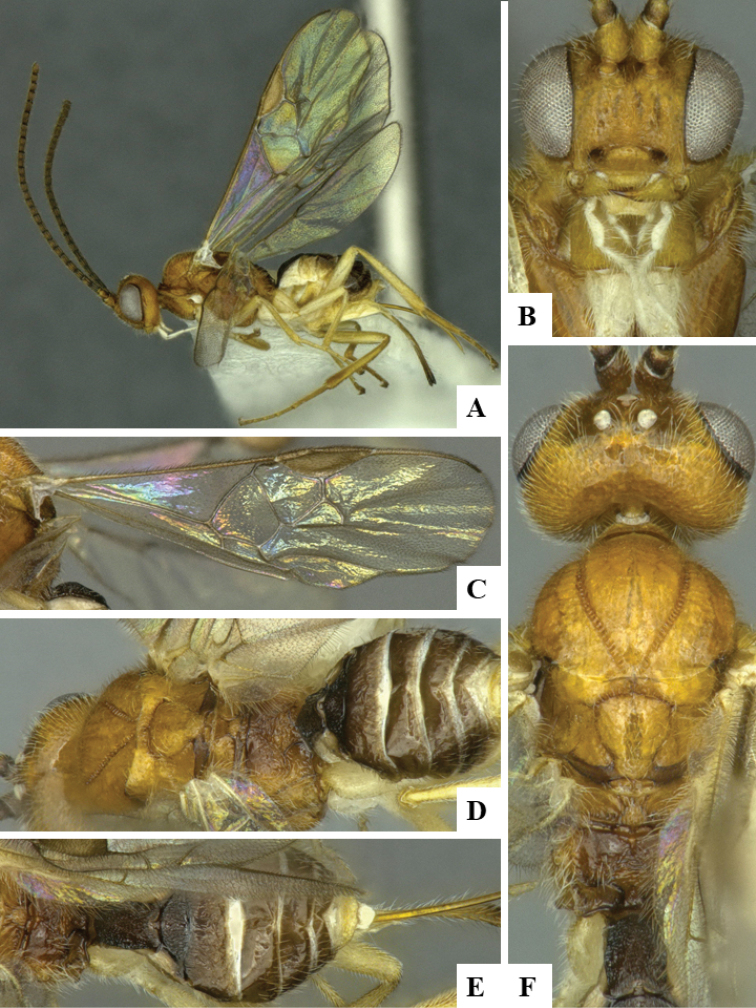
*Kiwigastervariabilis* female AMNZ71859 **A** Habitus, lateral **B** Head, frontal **C** Fore wing **D** Propodeum, dorsal **E** Metasoma, dorsal **F** Head and mesosoma, dorsal.

**Figure 137.**
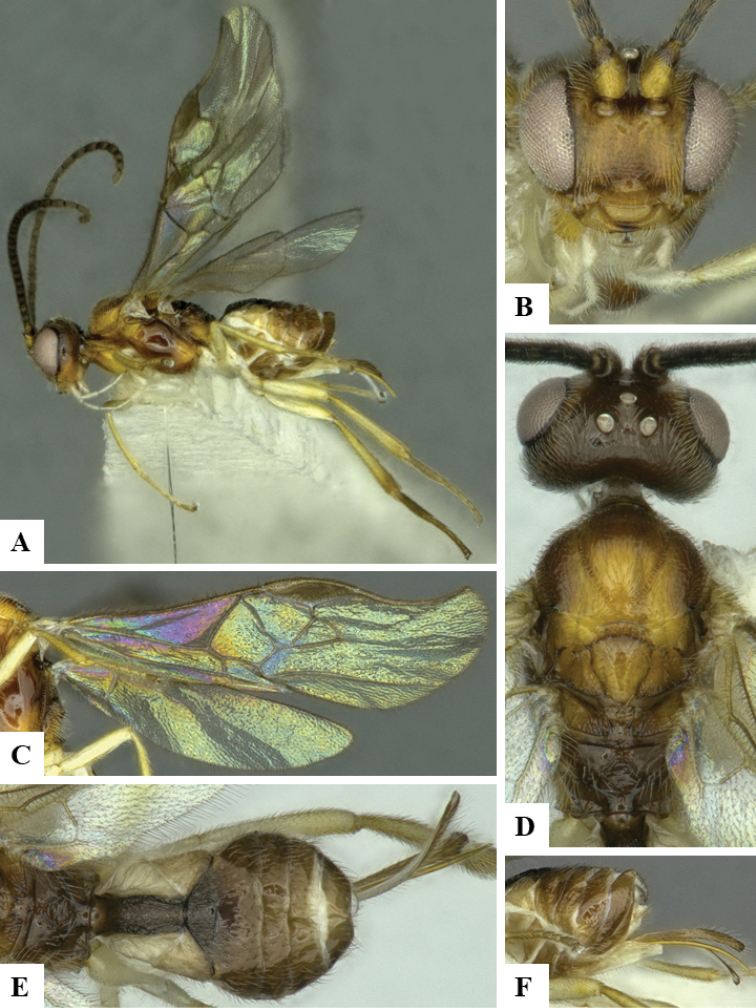
*Kiwigastervariabilis* female AMNZ71861 **A** Habitus, lateral **B** Head, frontal **C** Fore wing and hind wing **D** Head and mesosoma, dorsal **E** Propodeum and metasoma, dorsal **F** Ovipositor and ovipositor sheaths.

**Figure 138.**
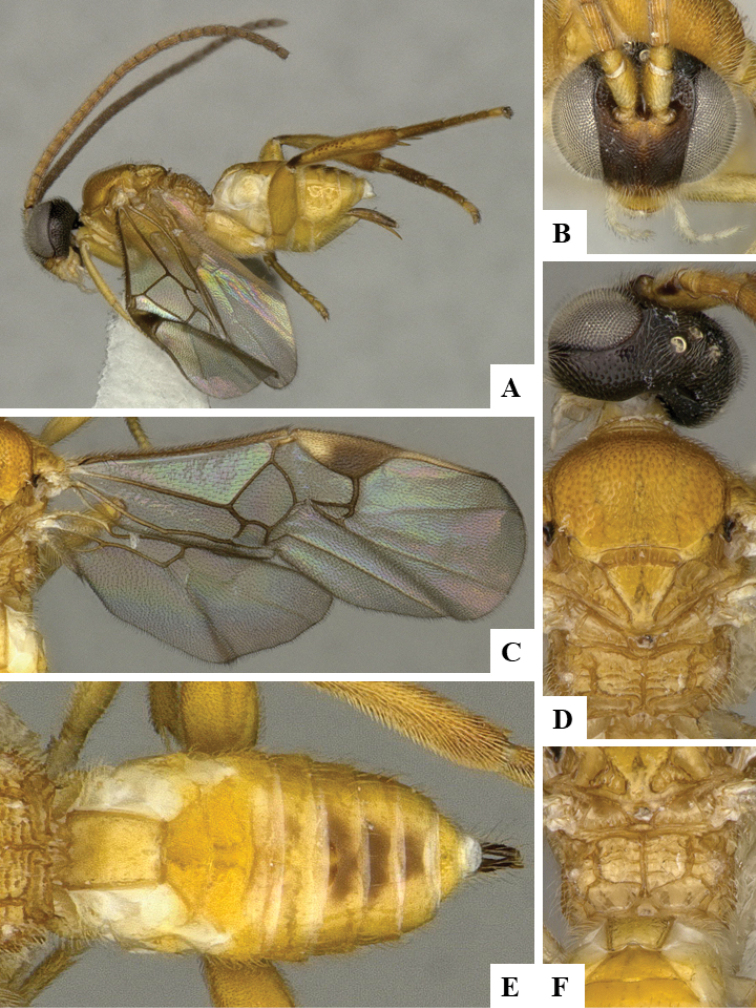
*Kotenkosiustricarinatus* female holotype **A** Habitus, lateral **B** Head, frontal **C** Fore wing and hind wing **D** Mesosoma, dorsal **E** Metasoma, dorsal **F** Propodeum, dorsal.

**Figure 139.**
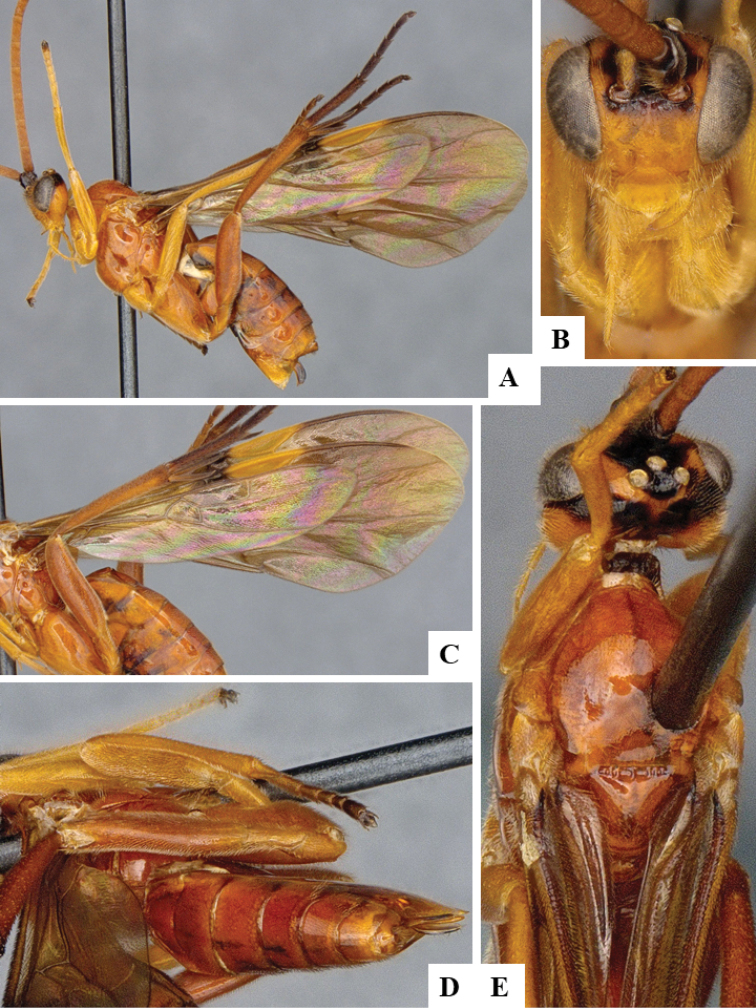
*Larissimuscassander* female CNC281020 **A** Habitus, lateral **B** Head, frontal **C** Fore wing and hind wing **D** Metasoma, laterodorsal **E** Head and mesosoma, dorsal.

**Figure 140.**
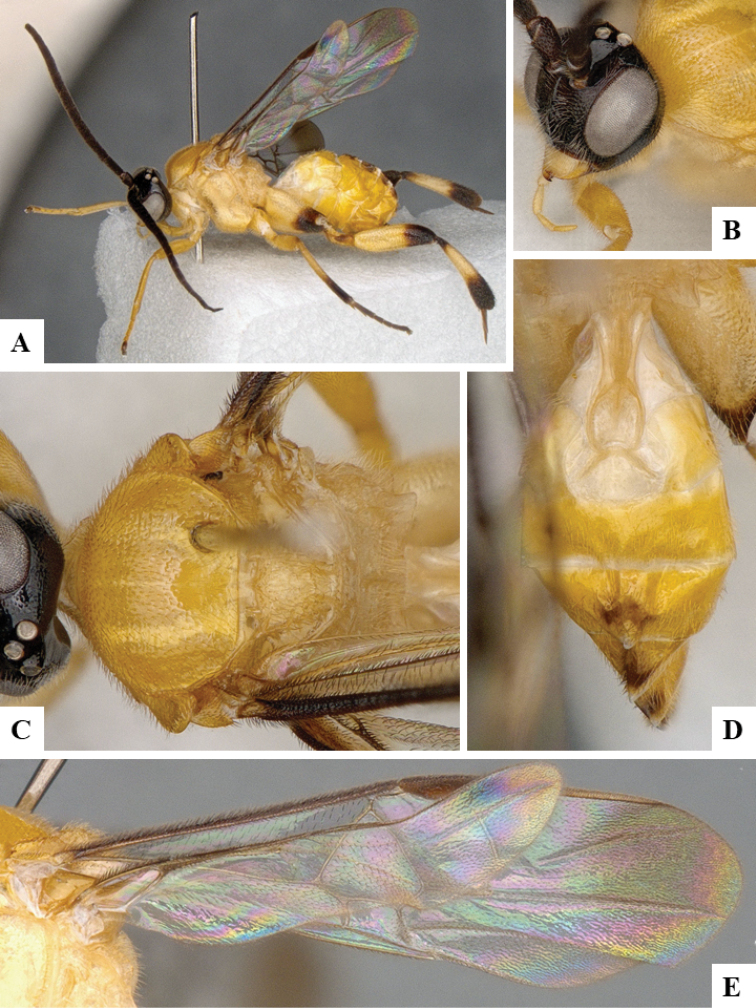
*Larissimus* sp. female CNC666286 **A** Habitus, lateral **B** Head, frontolateral **C** Mesosoma, dorsal **D** Metasoma, dorsal **E** Fore wing and hind wing.

**Figure 141.**
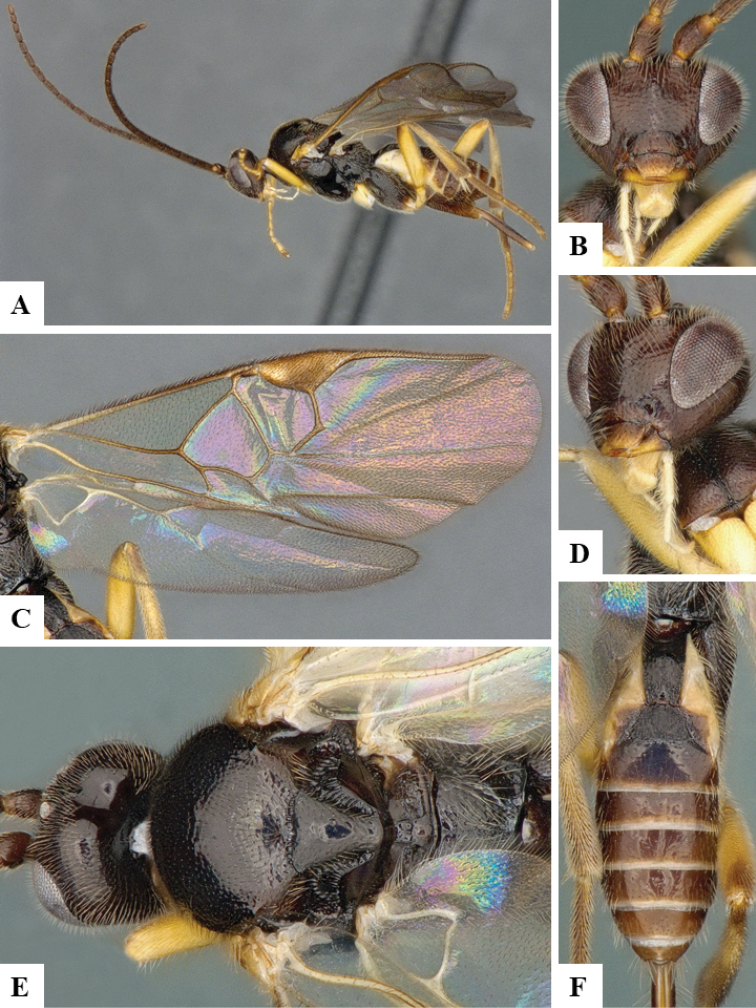
*Lathrapantelesampyx* female paratype CNCHYM01560 **A** Habitus, lateral **B** Head, frontal **C** Fore wing and hind wing **D** Head, laterofrontal **E** Mesosoma, dorsal **F** Metasoma, dorsal.

**Figure 142.**
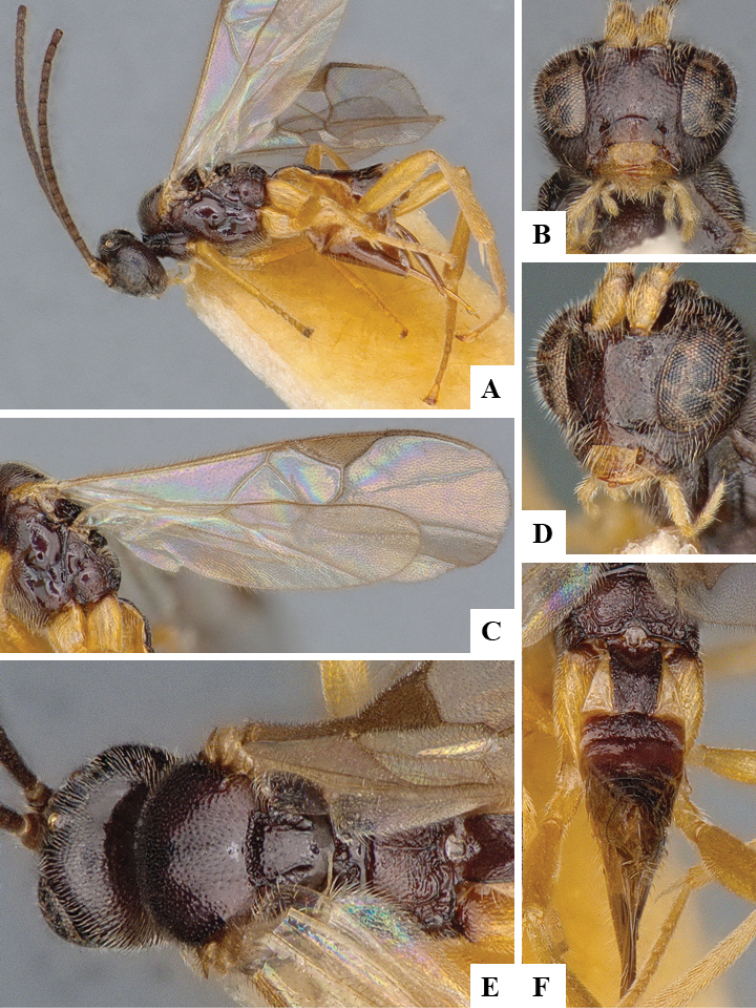
*Lathrapantelespapaipemae* female CNC807785 **A** Habitus, lateral **B** Head, frontal **C** Fore wing and hind wing **D** Head, frontolateral **E** Mesosoma, dorsal **F** Metasoma, dorsal.

**Figure 143.**
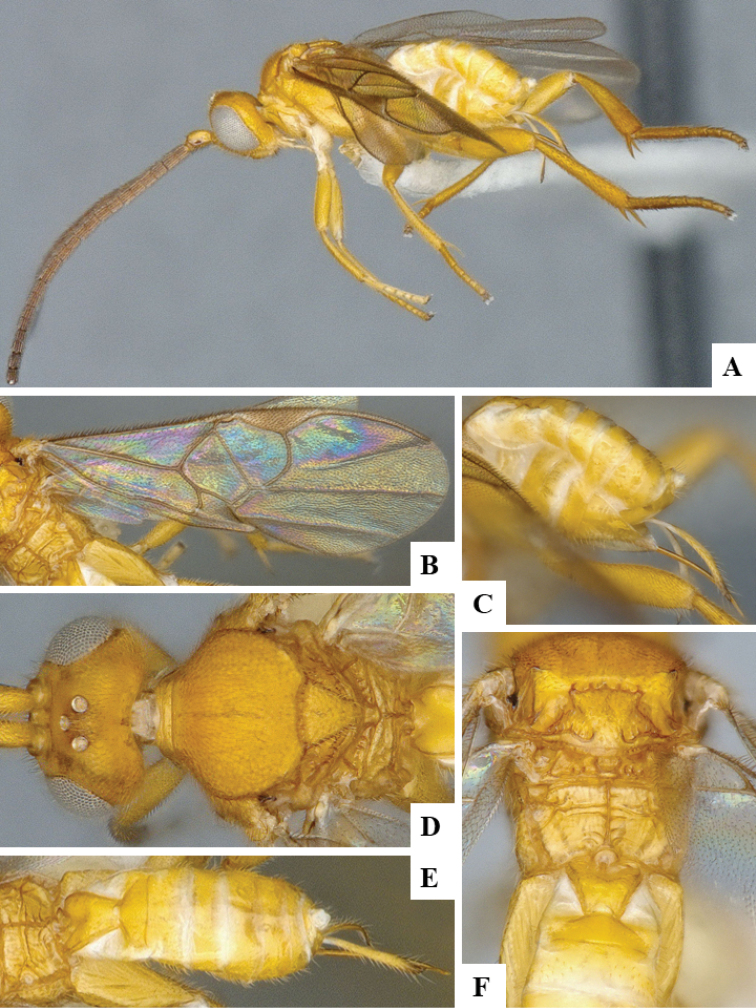
*Mariapantelesdapkeyae* female holotype **A** Habitus, lateral **B** Fore wing **C** Ovipositor and ovipositor sheaths **D** Head and mesosoma, dorsal **E** Propodeum and metasoma, dorsal **F** Propodeum and tergites 1–3, dorsal.

**Figure 144.**
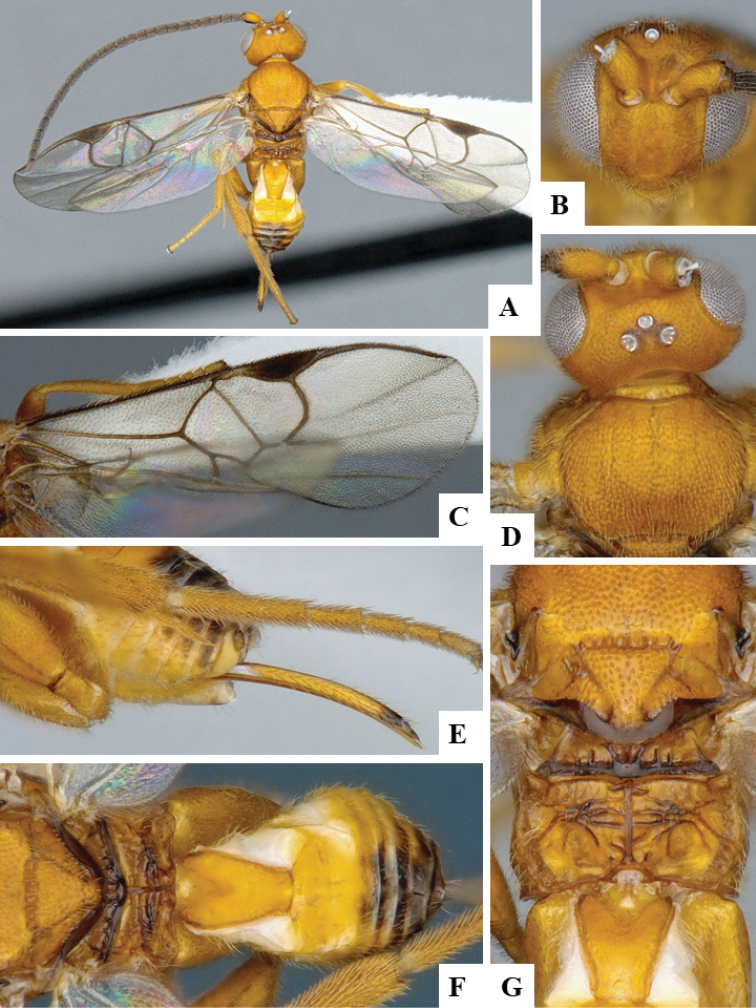
*Mariapantelesfelipei* female holotype **A** Habitus, dorsal **B** Head, frontal **C** Fore wing **D** Head and mesosoma, dorsal **E** Ovipositor and ovipositor sheaths **F** Metasoma, dorsal **G** Propodeum and tergite 1, dorsal.

**Figure 145.**
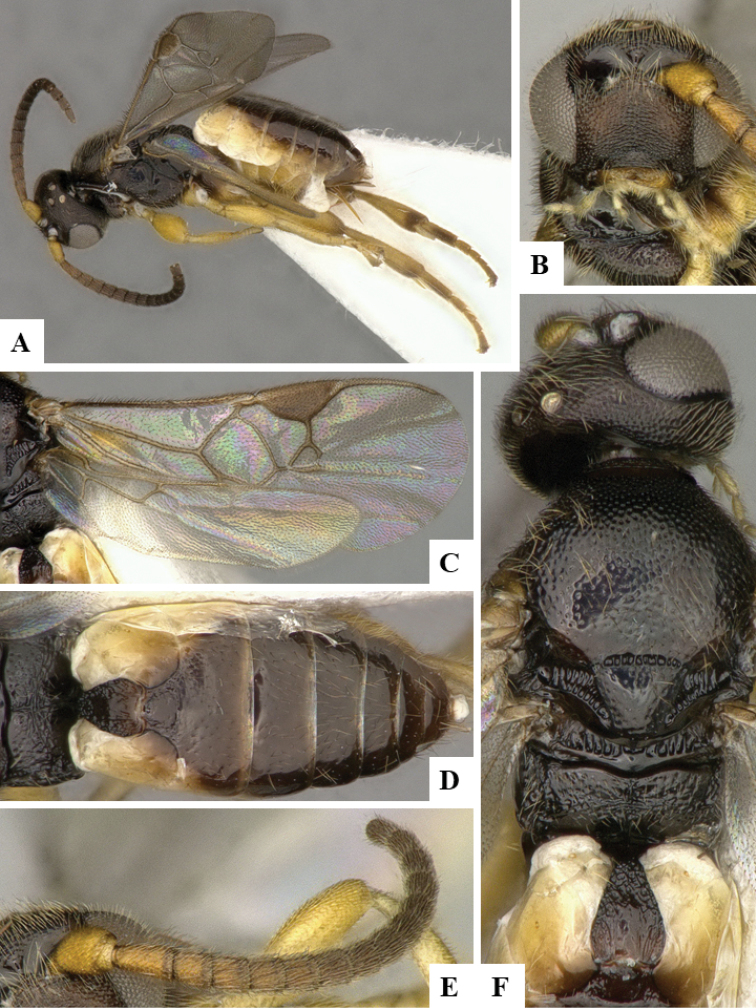
*Markshawiuserucidoctus* female holotype **A** Habitus, lateral **B** Head, frontal **C** Fore wing and hind wing **D** Metasoma, dorsal **E** Antenna **F** Mesosoma and tergite 1, dorsal.

**Figure 146.**
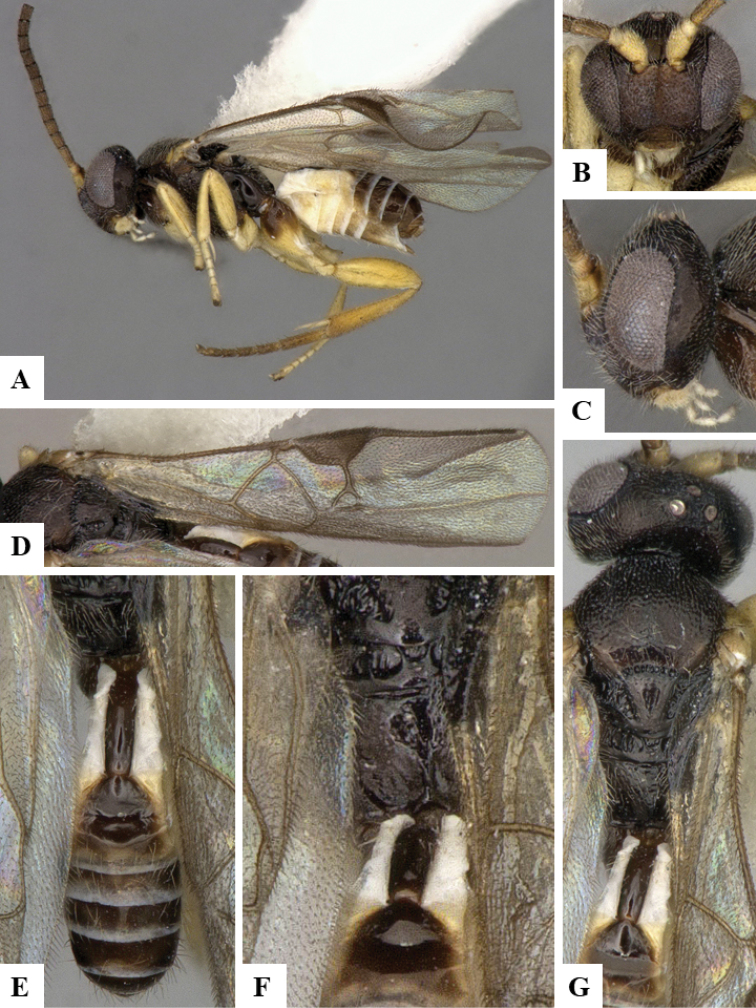
*Markshawiusfrancescae* female holotype **A** Habitus, lateral **B** Head, frontal **C** Head, lateral **D** Fore wing **E** Metasoma, dorsal **F** Propodeum and tergites 1–3, dorsal **G** Mesosoma and tergites 1–2, dorsal.

**Figure 147.**
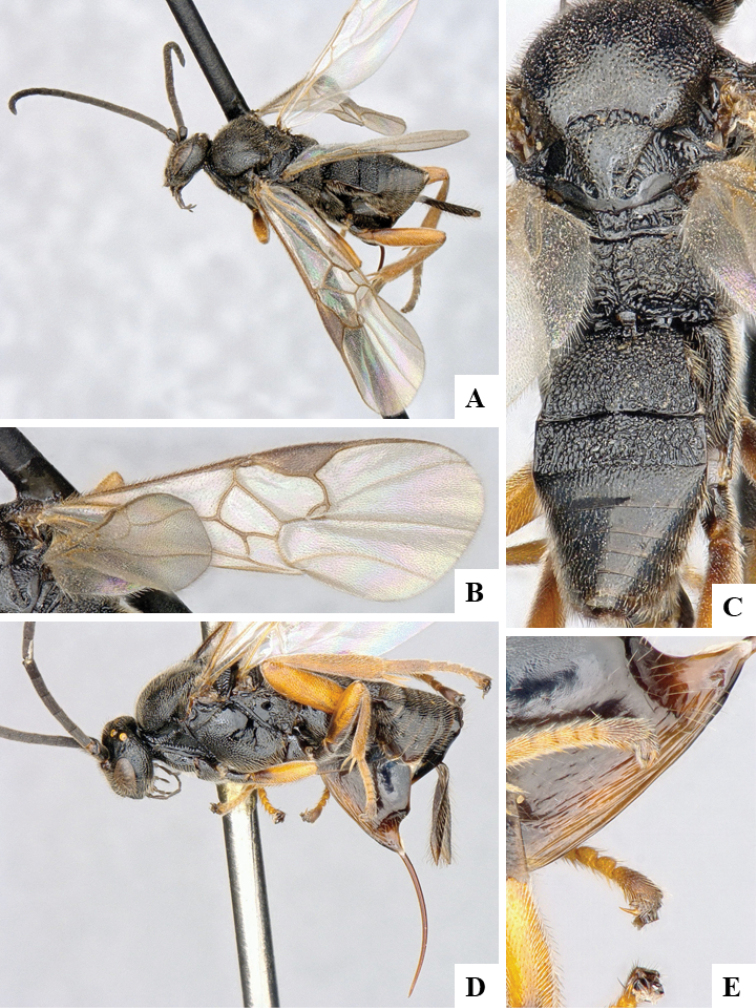
*Microgasterdeductor* female CNC752604 (for images A, B and C) and female CNC752606 (for images D and E) **A** Habitus, lateral **B** Fore wing **C** Mesosoma and metasoma, dorsal **D** Habitus, lateral **E** Hypopygium, ventrolateral.

**Figure 148.**
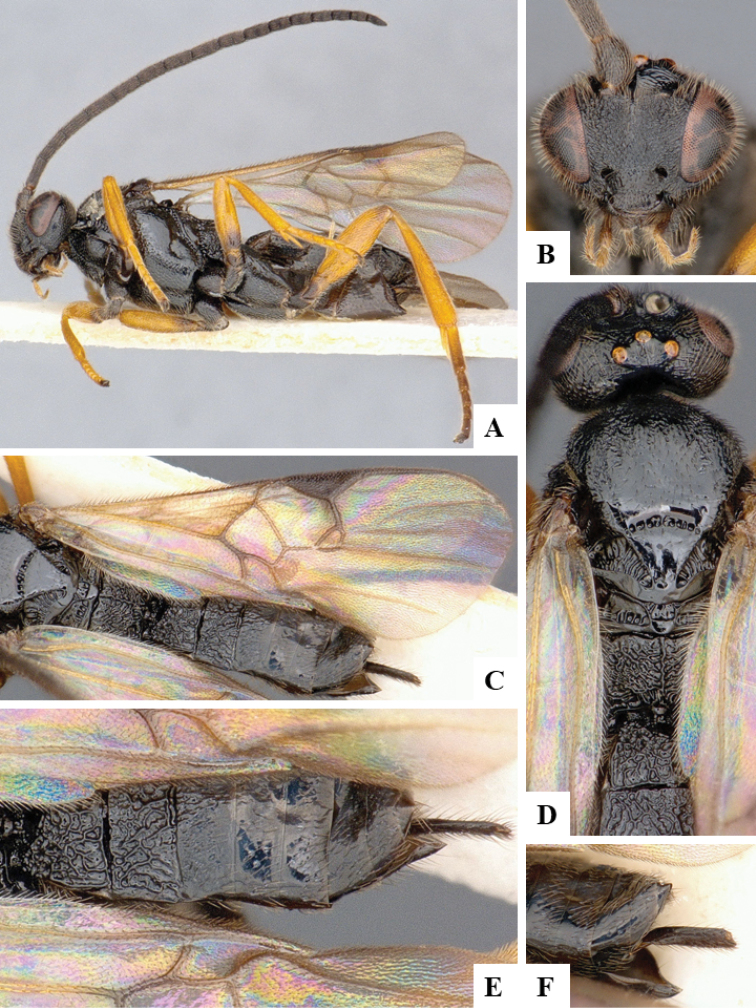
*Microgastermeridiana* female CNC474707 **A** Habitus, lateral **B** Head, frontal **C** Fore wing **D** Head and mesosoma, dorsal **E** Metasoma, dorsal **F** Hypopygium and ovipositor sheaths.

**Figure 149.**
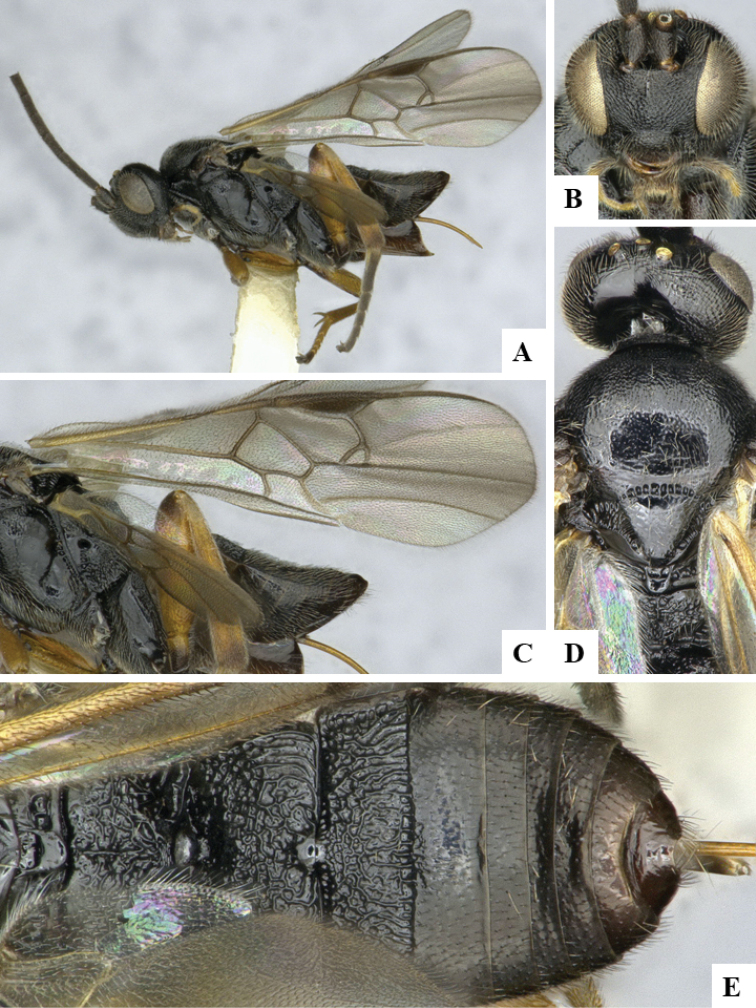
*Microgastermessoria* female CNCHYM01635 **A** Habitus, lateral **B** Head, frontal **C** Fore wing **D** Mesosoma, dorsal **E** Propodeum and metasoma, dorsal.

**Figure 150.**
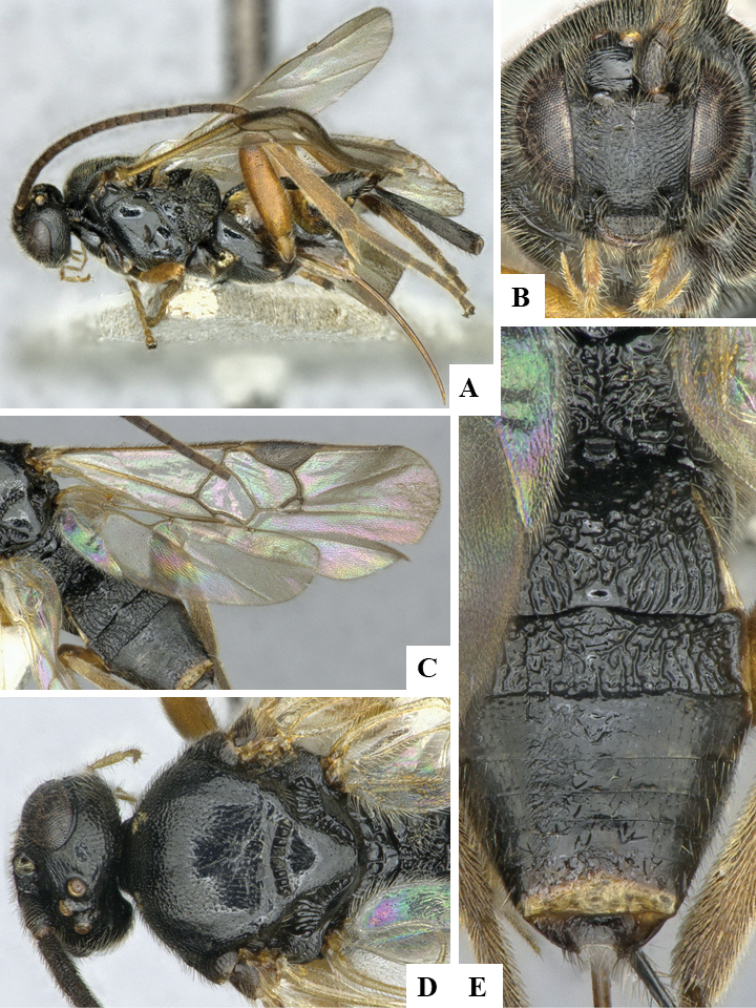
*Microgastersubcompleta* female CNCHYM01657 **A** Habitus, lateral **B** Head, frontal **C** Fore wing and hind wing **D** Head and mesosoma, dorsal **E** Propodeum and metasoma, dorsal.

**Figure 151.**
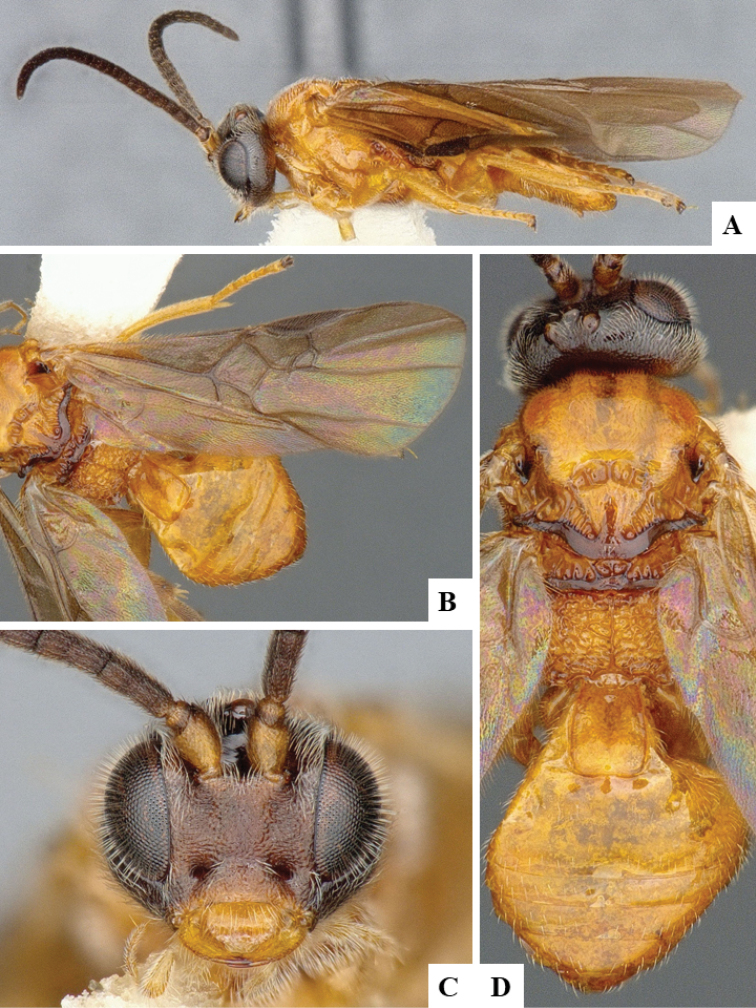
*Microplitischacoensis* female CNCHYM01728 **A** Habitus, lateral **B** Fore wing **C** Head, frontal **D** Habitus, dorsal.

**Figure 152.**
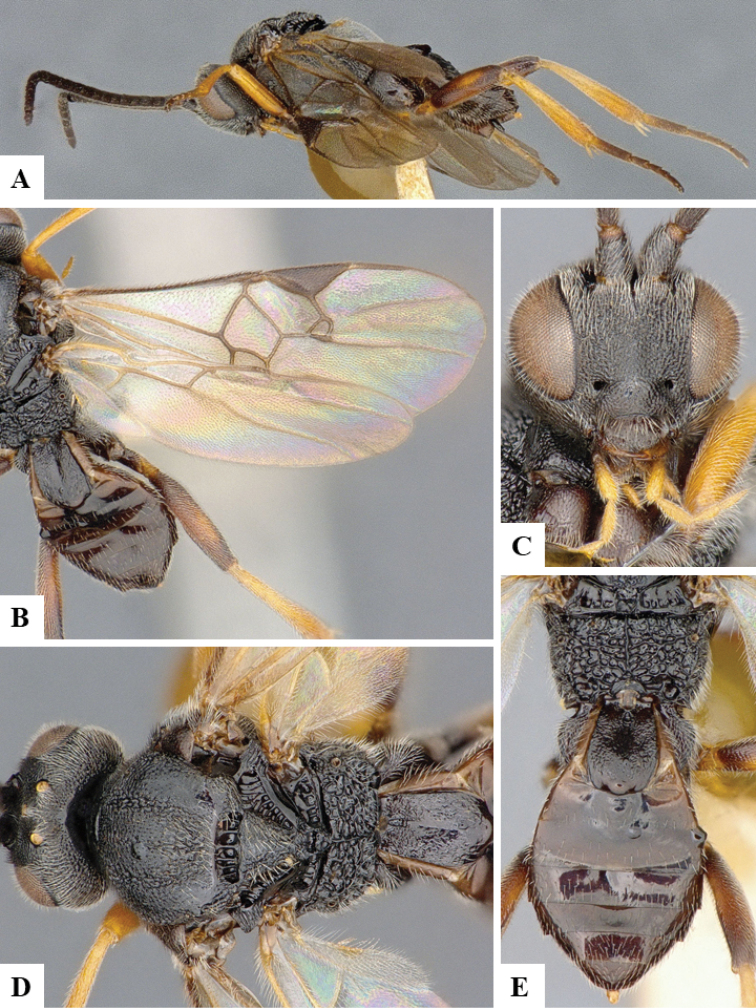
*Microplitisflavipalpis* female CNCHYM01748 **A** Habitus, lateral **B** Fore wing and hind wing **C** Head, frontal **D** Head and mesosoma, dorsal **E** Propodeum and metasoma, dorsal.

**Figure 153.**
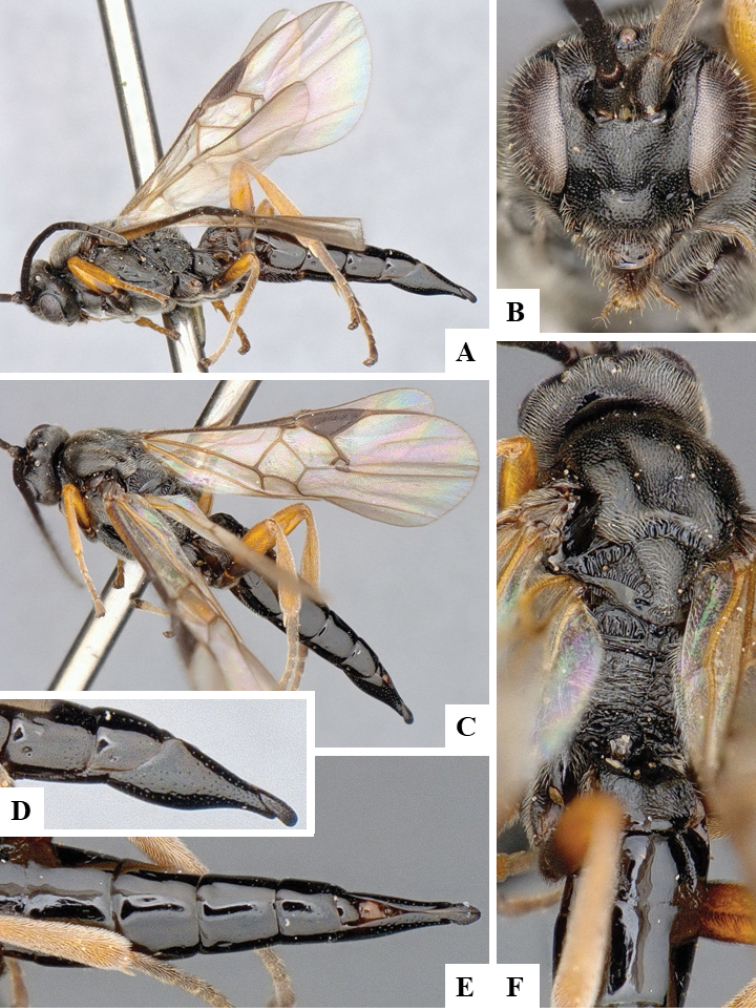
*Microplitisjuanmanueli* female holotype **A** Habitus, lateral **B** Head, frontal **C** Fore wing **D** Apex of metasoma, lateral **E** Metasoma, dorsal **F** Mesosoma and tergites 1–3, laterodorsal.

**Figure 154.**
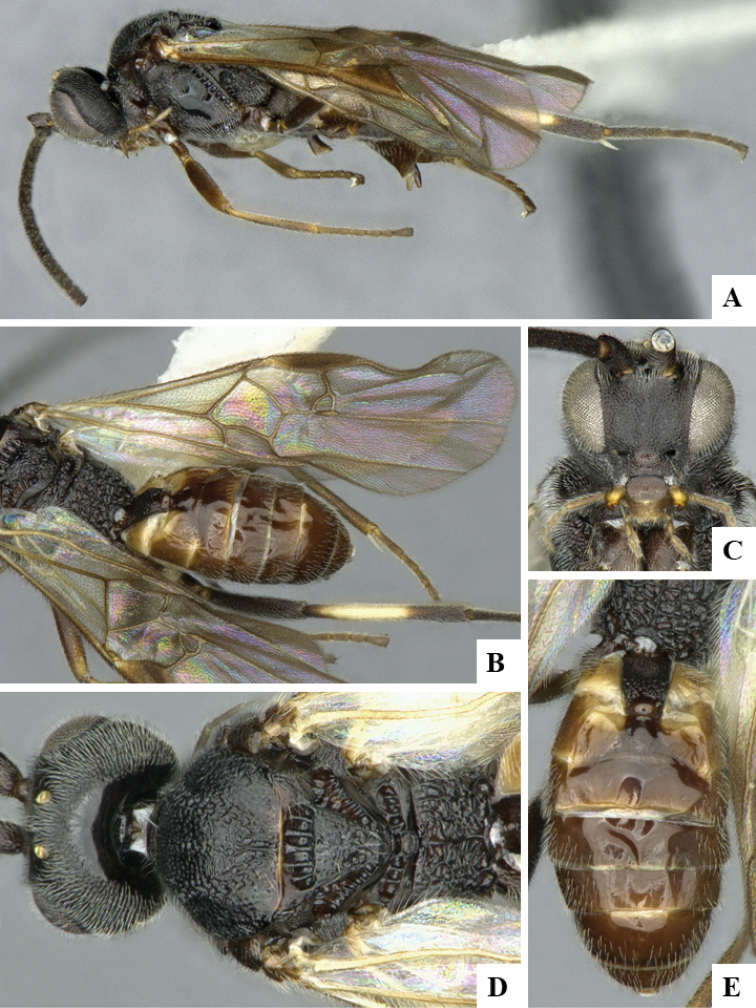
*Microplitismanilae* female CNC776776 **A** Habitus, lateral **B** Fore wing **C** Head, frontal **D** Head and mesosoma, dorsal **E** Metasoma, dorsal.

**Figure 155.**
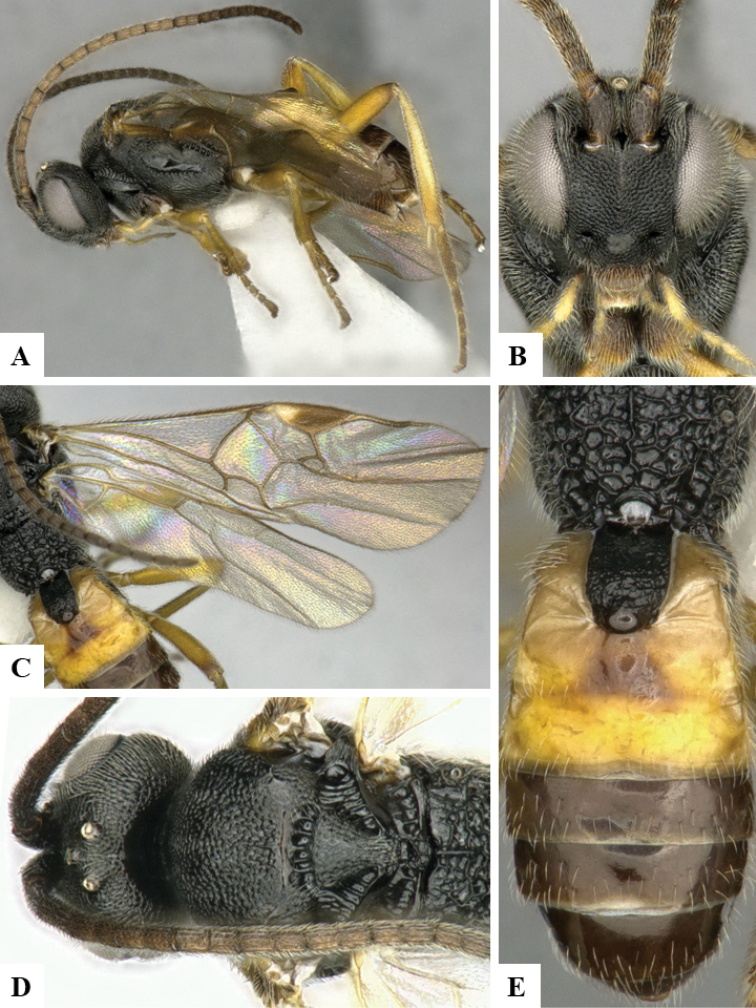
*Microplitismediator* female CNC677799 **A** Habitus, lateral **B** Head, frontal **C** Fore wing and hind wing **D** Head and mesosoma, dorsal **E** Propodeum and metasoma, dorsal.

**Figure 156.**
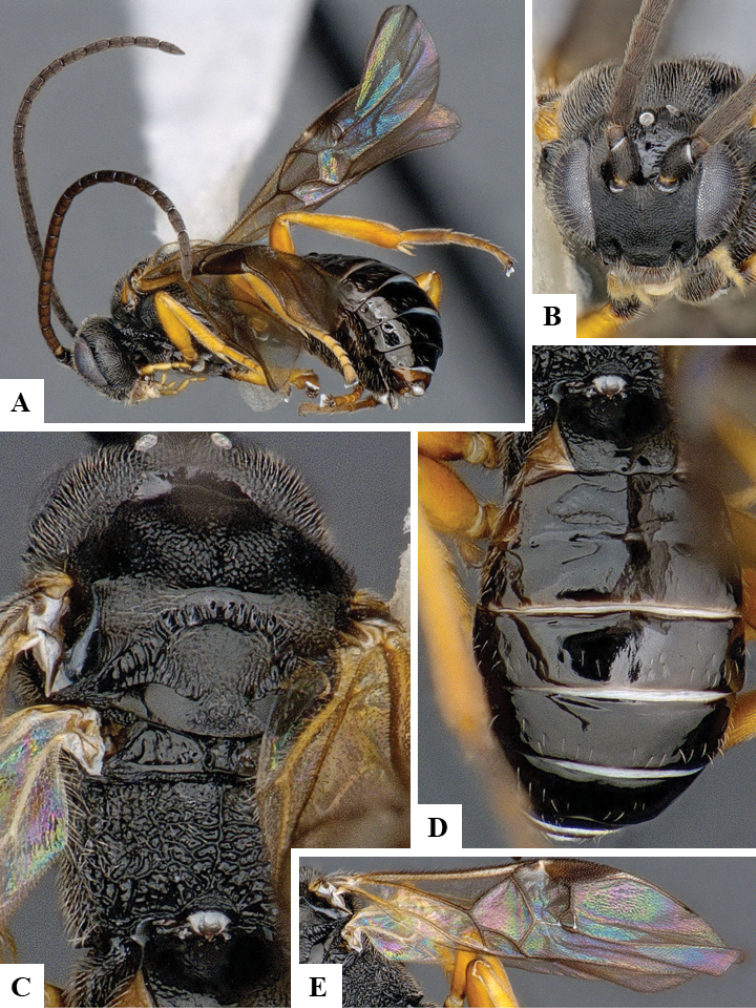
*Microplitisocellatae* female MRSJFT0076 **A** Habitus, lateral **B** Head, frontal **C** Mesosoma, dorsal **D** Metasoma, dorsal **E** Fore wing.

**Figure 157.**
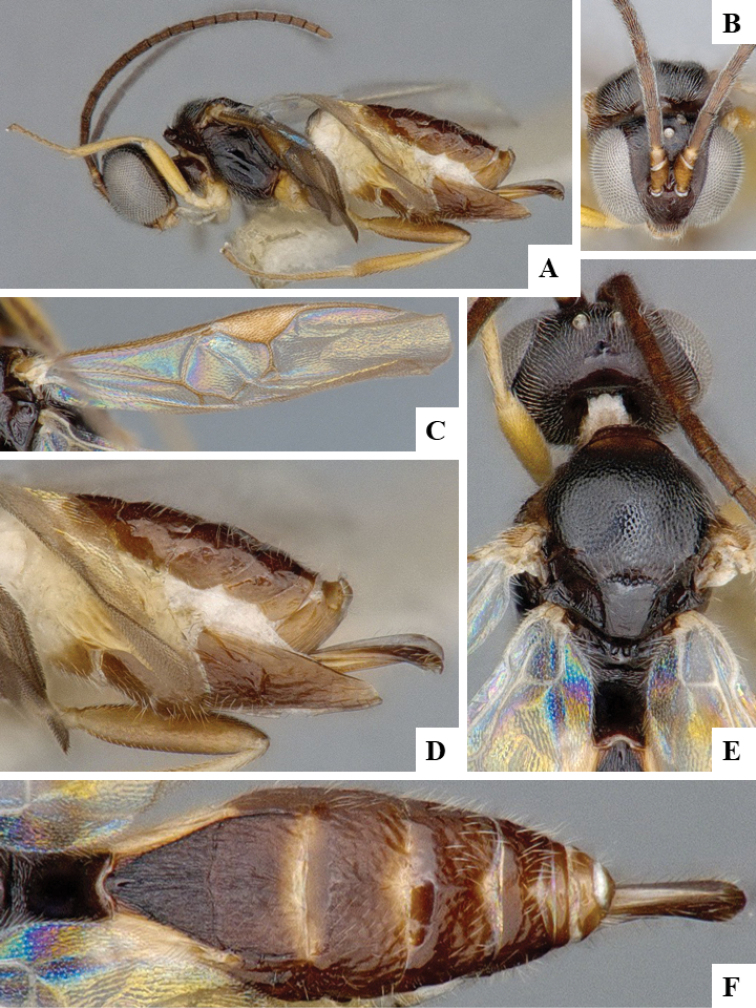
*Miropotesaustini* female holotype **A** Habitus, lateral **B** Head, frontal **C** Fore wing **D** Metasoma, lateral **E** Head and mesosoma, dorsal **F** Metasoma, dorsal.

**Figure 158.**
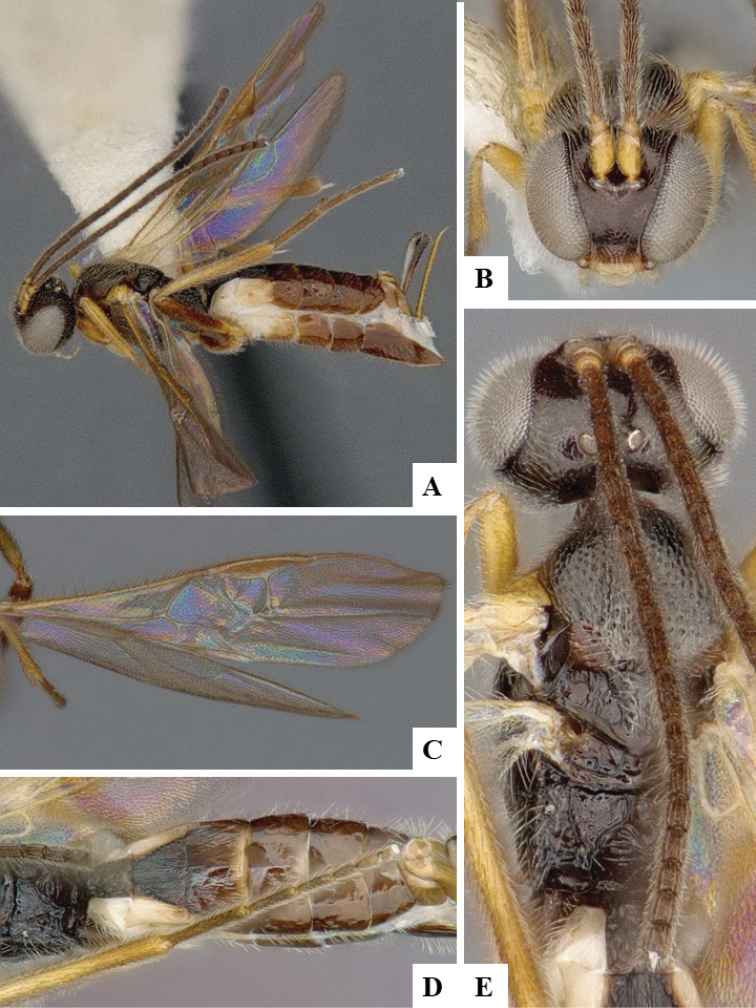
*Miropoteslordhowensis* female holotype **A** Habitus, lateral **B** Head, frontal **C** Fore wing and hind wing **D** Metasoma, dorsal **E** Head and mesosoma, dorsal.

**Figure 159.**
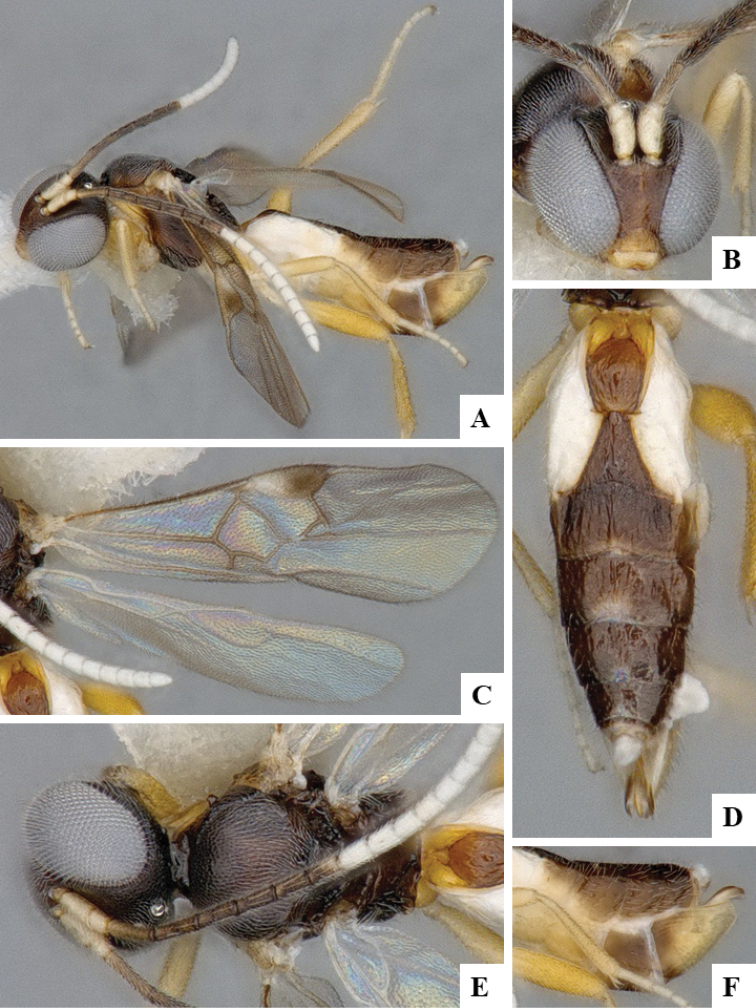
*Miropotesorientalis* female paratype CNCH2114 **A** Habitus, lateral **B** Head, frontal **C** Fore wing and hind wing **D** Metasoma, dorsal **E** Mesosoma, dorsal **F** Apex of metasoma, lateral.

**Figure 160.**
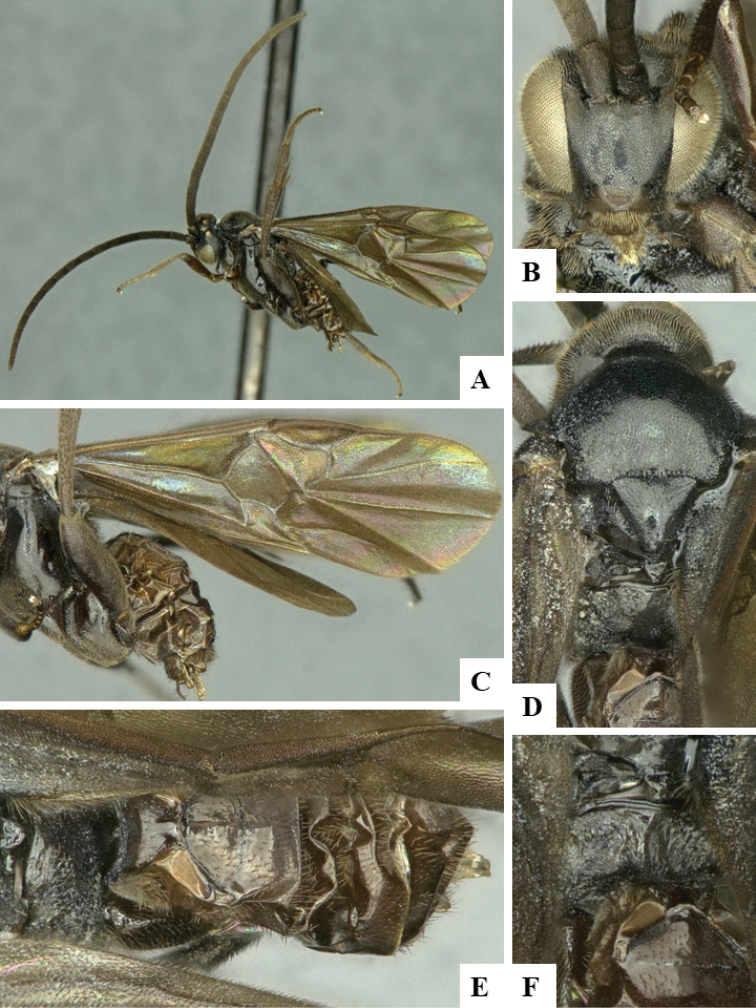
*Napamus* sp. male CNCHYM01899 **A** Habitus, lateral **B** Head, frontal **C** Fore wing **D** Mesosoma, dorsal **E** Metasoma, dorsal **F** Propodeum, dorsal.

**Figure 161.**
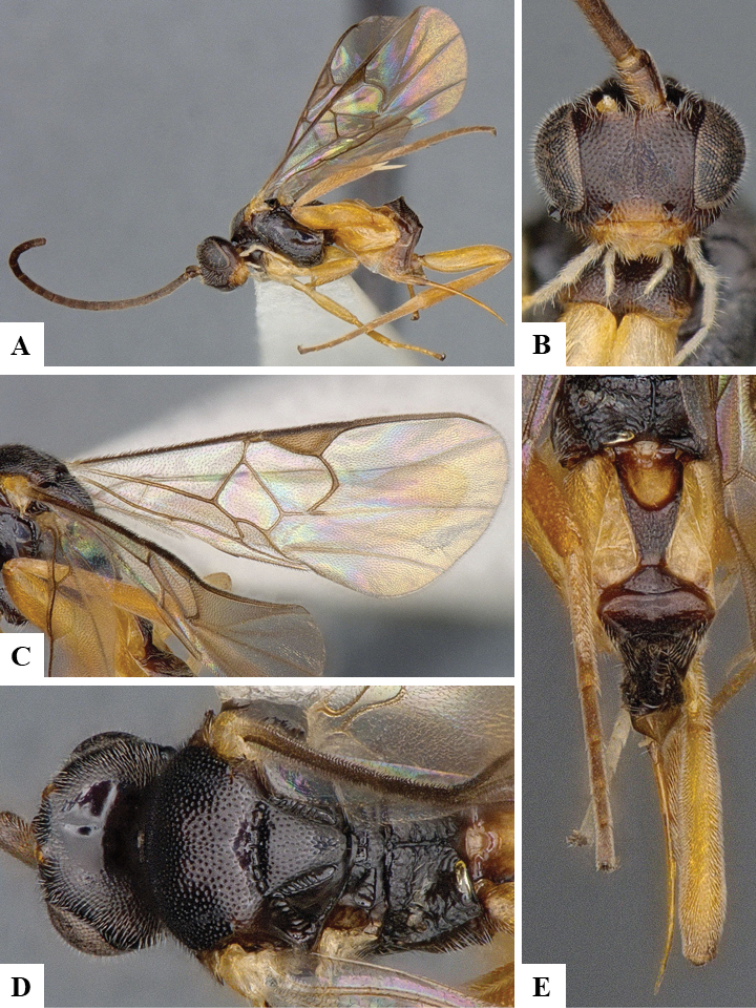
*Neoclarkinellaariadne* female CNCHYM01447 **A** Habitus, lateral **B** Head, frontal **C** Fore wing **D** Mesosoma, dorsal **E** Metasoma, dorsal.

**Figure 162.**
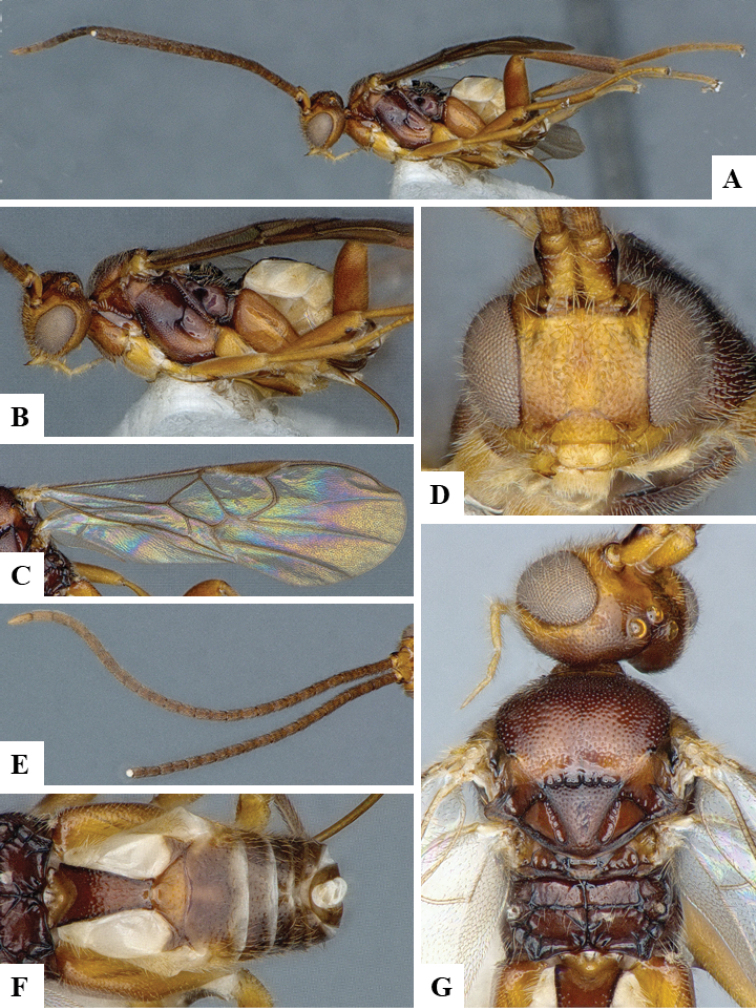
*Neoclarkinella* sp. female CNC924600 **A** Habitus, lateral **B** Habitus magnified, lateral **C** Fore wing **D** Head, frontal **E** Antennae **F** Metasoma, dorsal **G** Mesosoma, dorsal.

**Figure 163.**
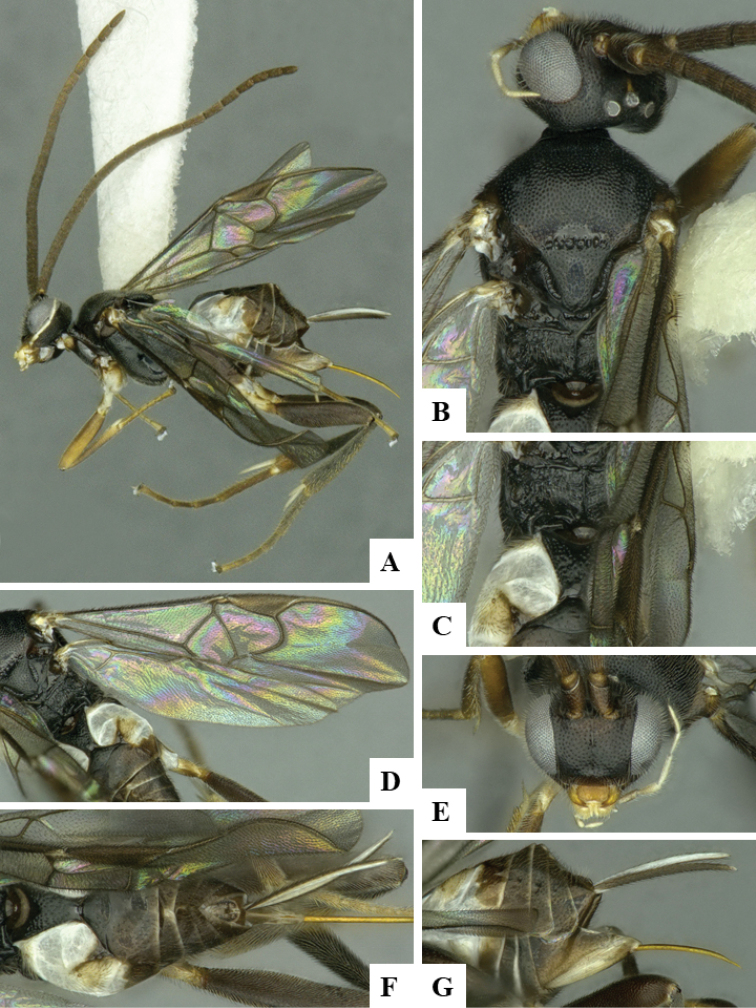
*Neoclarkinella* sp. female CNCH1454 **A** Habitus, lateral **B** Mesosoma, dorsal **C** Propodeum, dorsal **D** Fore wing and hind wing **E** Head, frontal **F** Metasoma, dorsal **G** Ovipositor and ovipositor sheaths.

**Figure 164.**
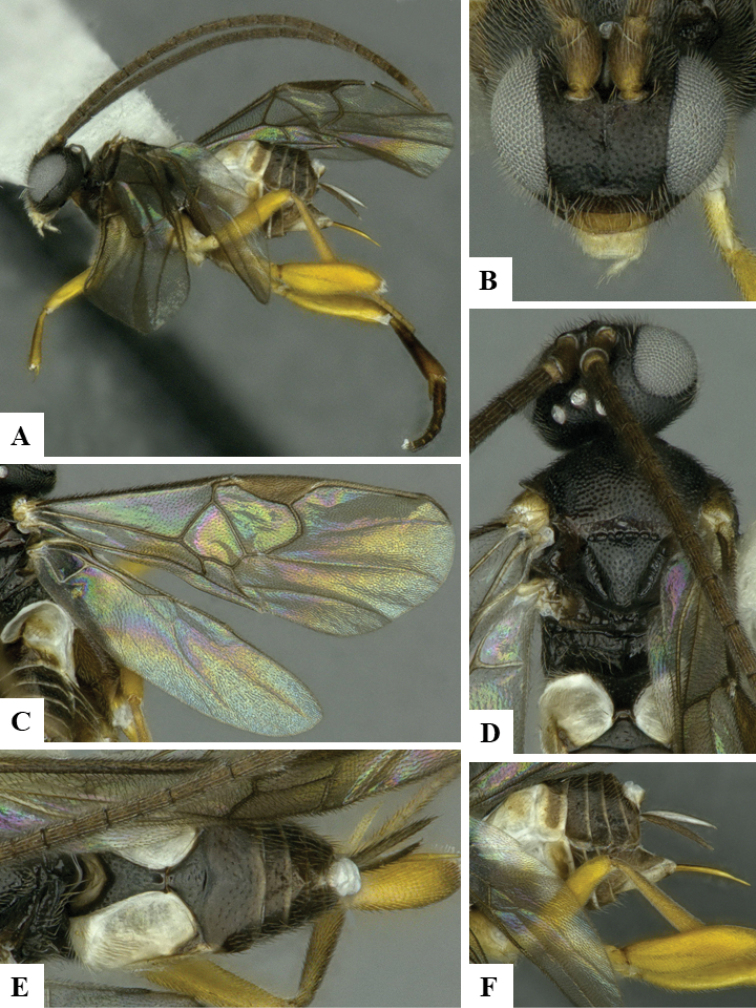
*Neoclarkinella* sp. female CNCH2005 **A** Habitus, lateral **B** Head, frontal **C** Fore wing and hind wing **D** Mesosoma, dorsal **E** Metasoma, dorsal **F** Ovipositor and ovipositor sheaths.

**Figure 165.**
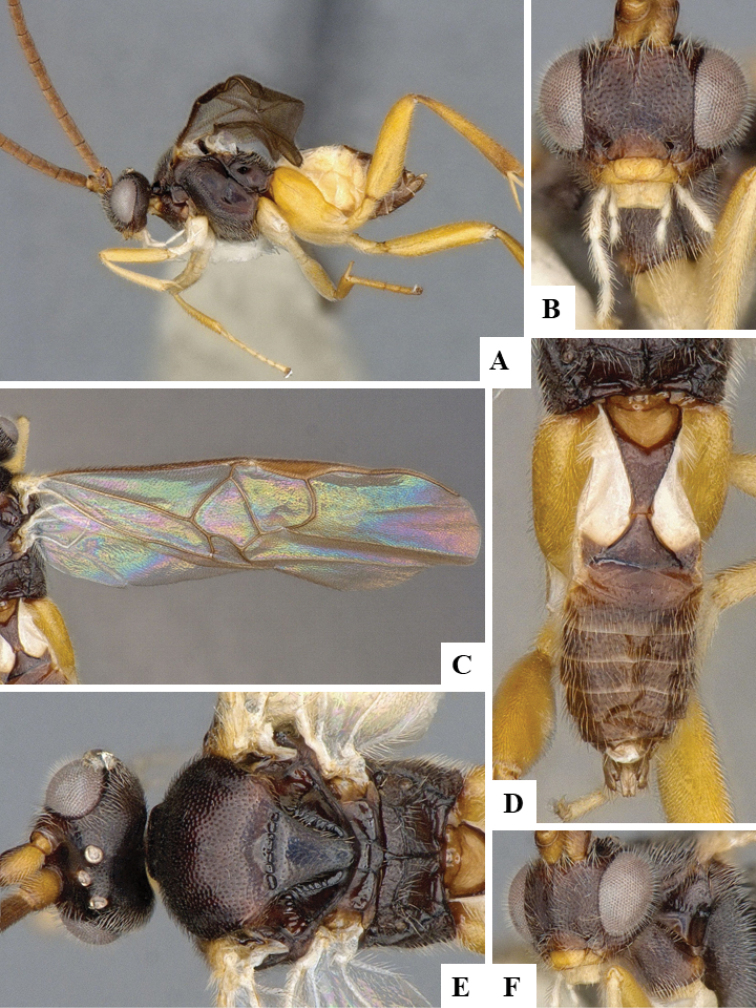
*Neoclarkinellasundanus* male CNCHYM01464 **A** Habitus, lateral **B** Head, frontal **C** Fore wing **D** Metasoma, dorsal **E** Head and mesosoma, dorsal **F** Head, frontolateral.

**Figure 166.**
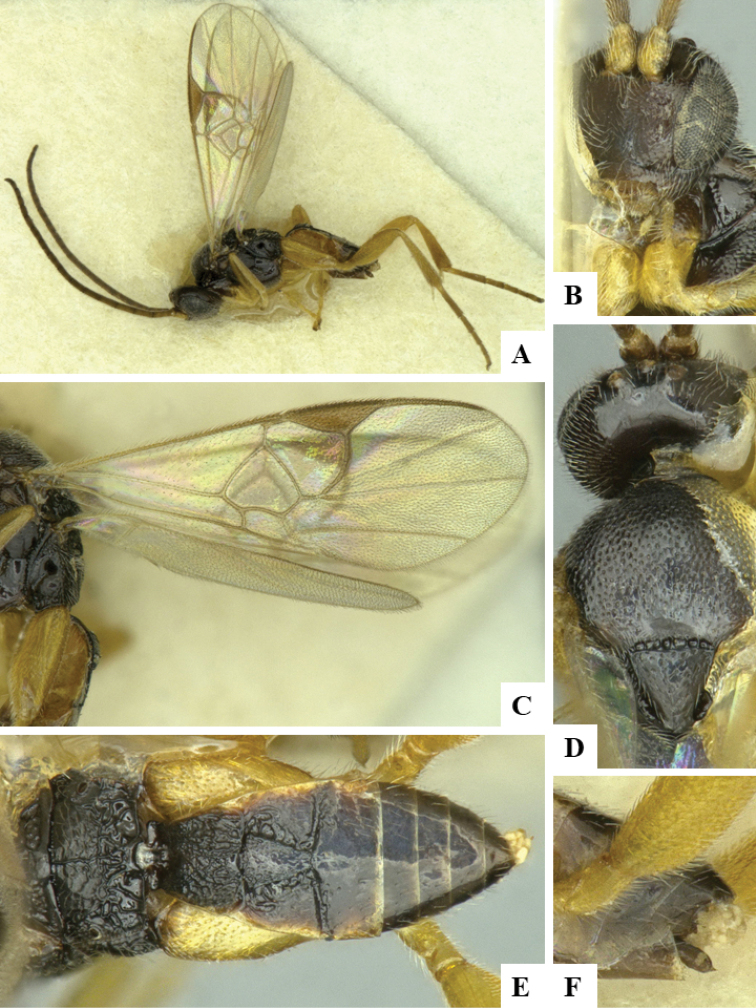
*Nyereriamlanje* female CNCHYM01901 **A** Habitus, lateral **B** Head, frontolateral **C** Fore wing and hind wing **D** Mesosoma, dorsal **E** Propodeum and metasoma, dorsal **F** Ovipositor sheaths.

**Figure 167.**
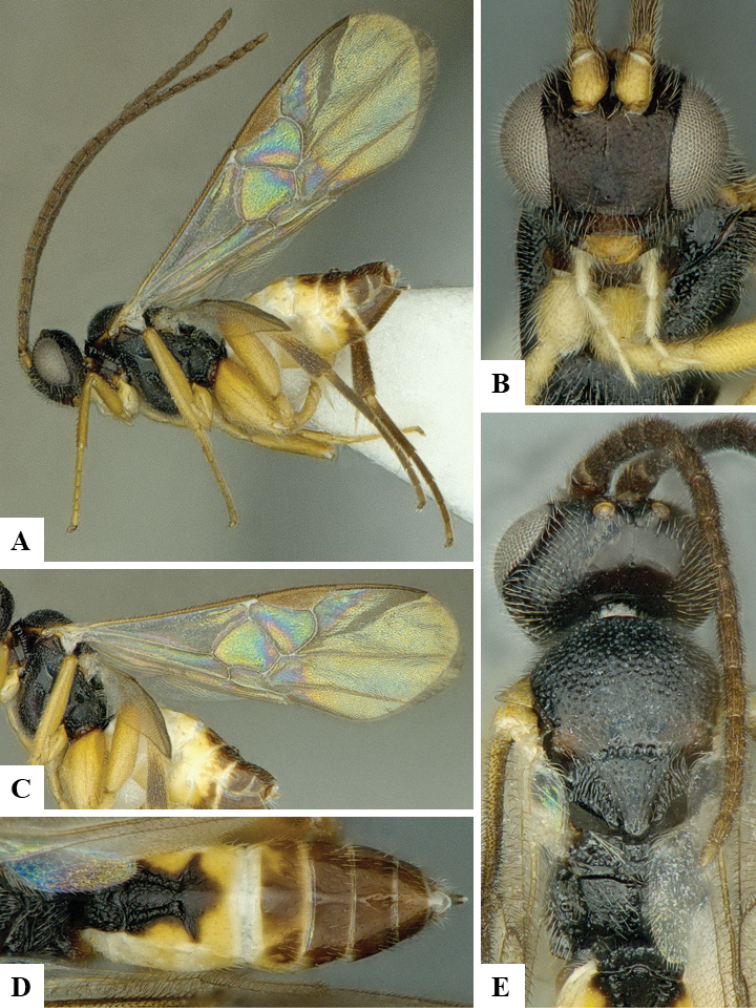
*Nyereria* sp. female CNCH0835 **A** Habitus, lateral **B** Head, frontal **C** Fore wing **D** Metasoma, dorsal **E** Head and mesosoma, dorsal.

**Figure 168.**
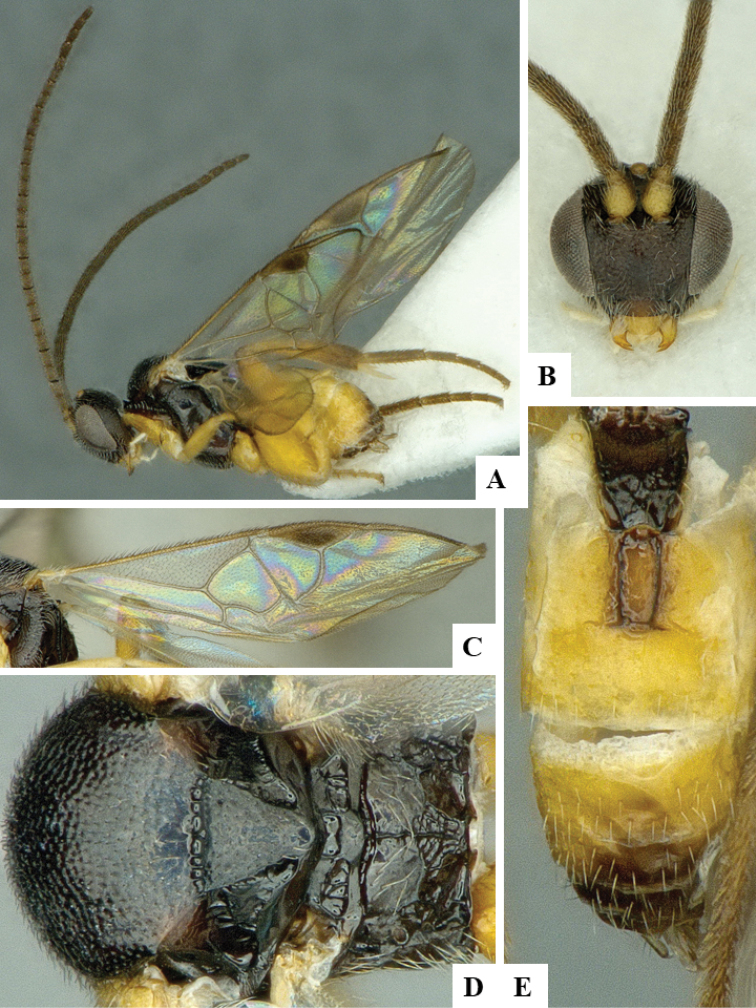
*Nyereria* sp. male CNCH0837 **A** Habitus, lateral **B** Head, frontal **C** Fore wing **D** Mesosoma, dorsal **E** Metasoma, dorsal.

**Figure 169.**
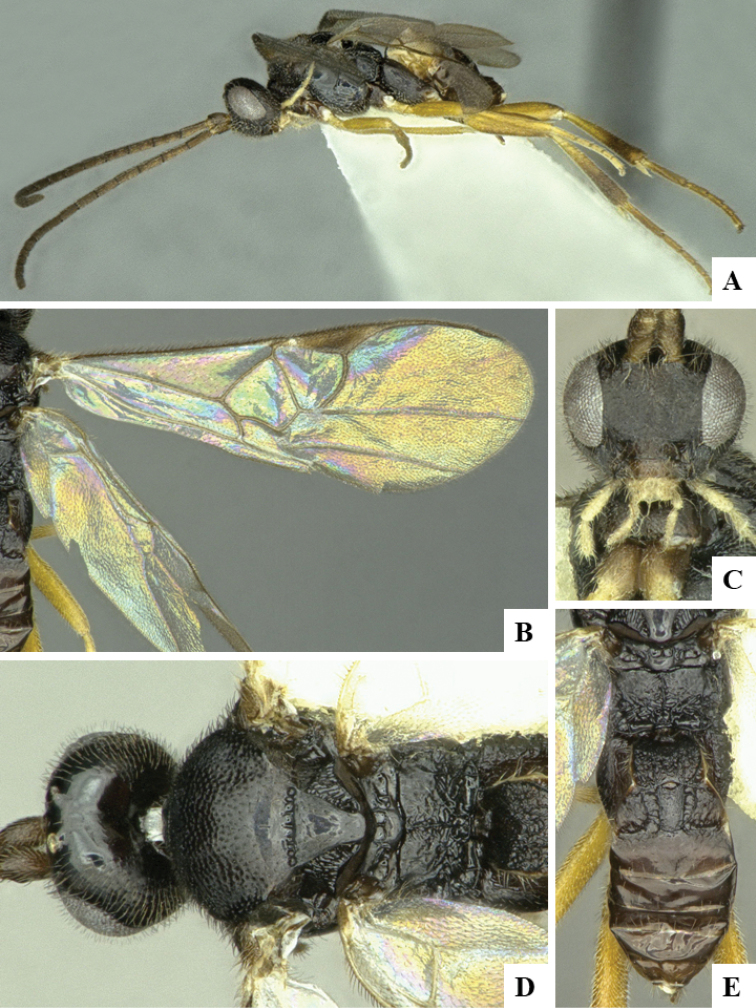
*Nyereria* sp. female CNCHYM01906 **A** Habitus, lateral **B** Fore wing **C** Head, frontal **D** Head and mesosoma, dorsal **E** Metasoma, dorsal.

**Figure 170.**
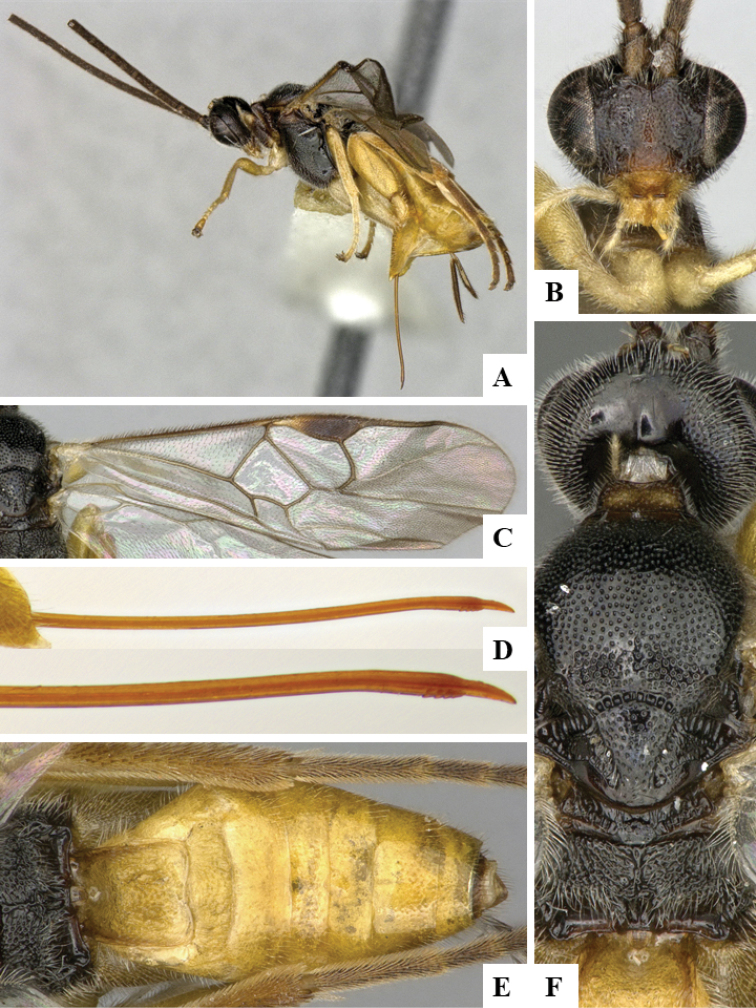
*Ohenrigouletarum* female holotype **A** Habitus, lateral **B** Head, frontal **C** Fore wing **D** Ovipositor and apex of ovipositor **E** Propodeum and metasoma, dorsal **F** Head and mesosoma, dorsal.

**Figure 171.**
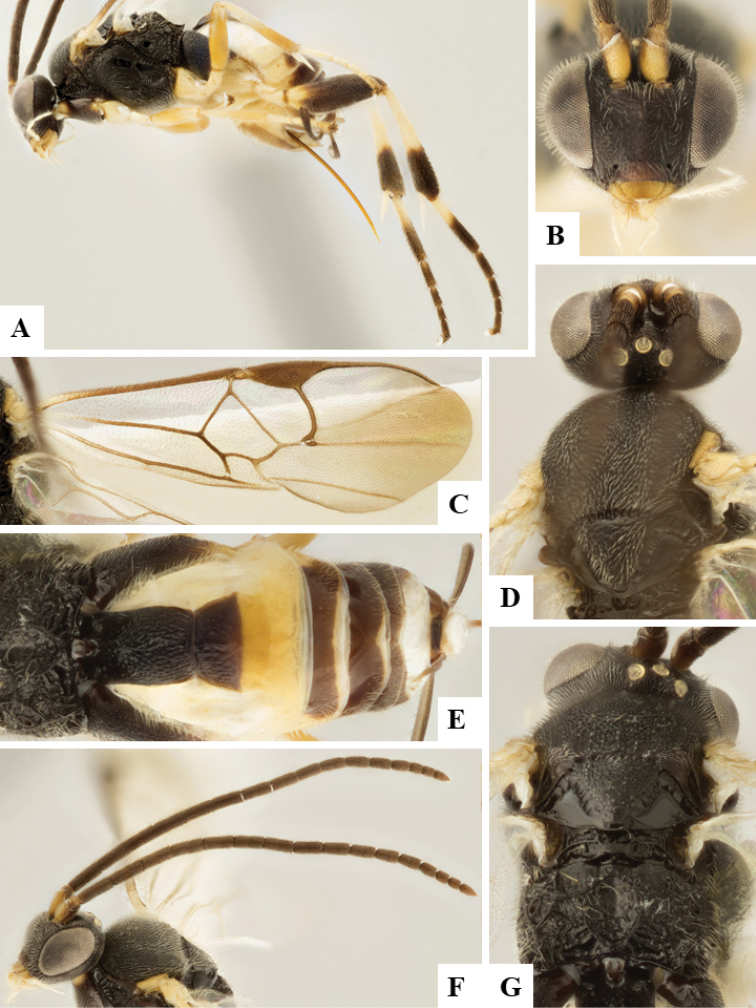
*Papantelespeckorum* female holotype **A** Habitus, lateral **B** Head, frontal **C** Fore wing **D** Head and mesosoma, dorsal **E** Metasoma, dorsal **F** Antennae **G** Propodeum, dorsal.

**Figure 172.**
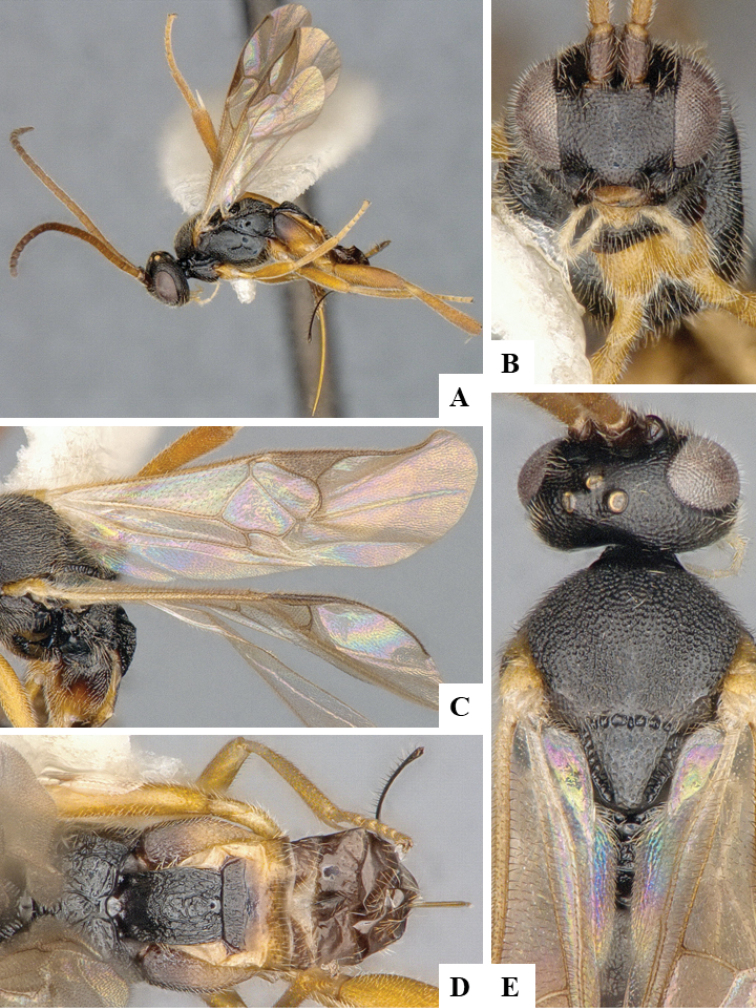
*Parapantelesaletiae* female CNCHYM01930 **A** Habitus, lateral **B** Head, frontal **C** Fore wing **D** Propodeum and metasoma, dorsal **E** Mesosoma, dorsal.

**Figure 173.**
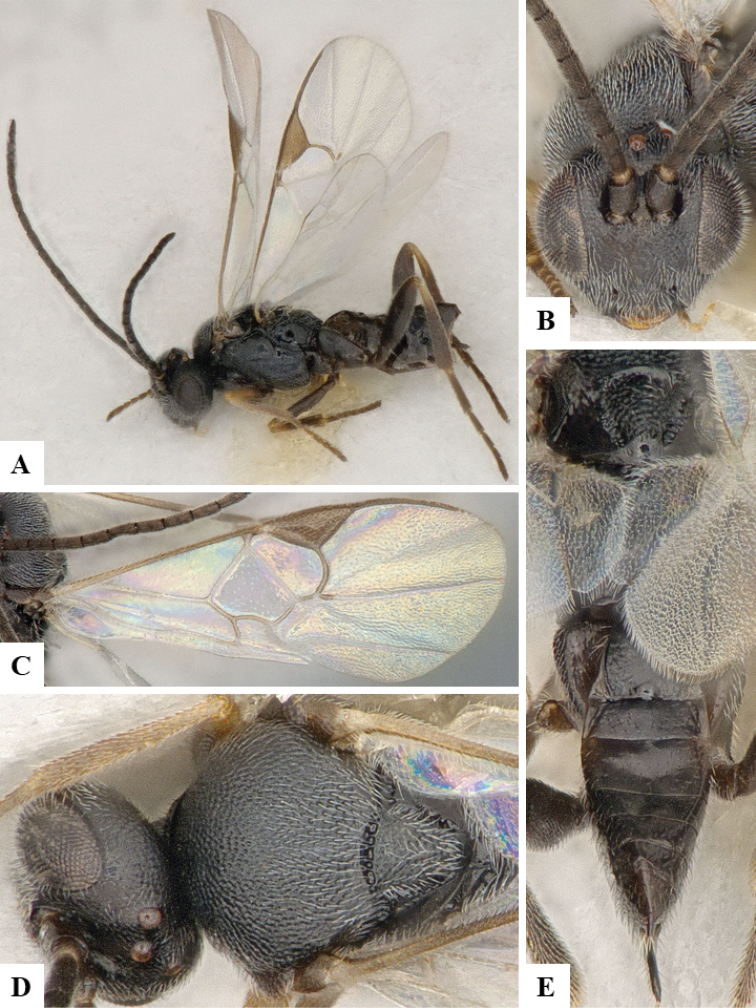
*Parapantelesgerontogeae* female CNC309845 **A** Habitus, lateral **B** Head, frontal **C** Fore wing **D** Mesosoma, dorsal **E** Metasoma, dorsal.

**Figure 174.**
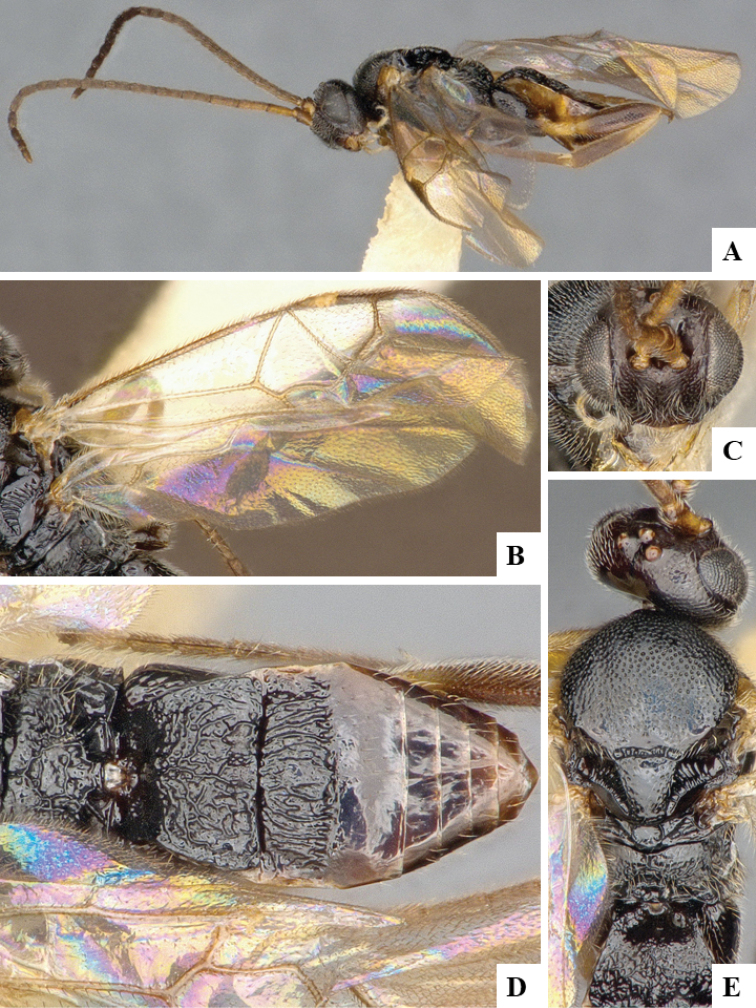
*Parapantelesparadoxus* female CNCHYM01936 **A** Habitus, lateral **B** Fore wing and hind wing **C** Head, frontal **D** Propodeum and metasoma, dorsal **E** Mesosoma, dorsal.

**Figure 175.**
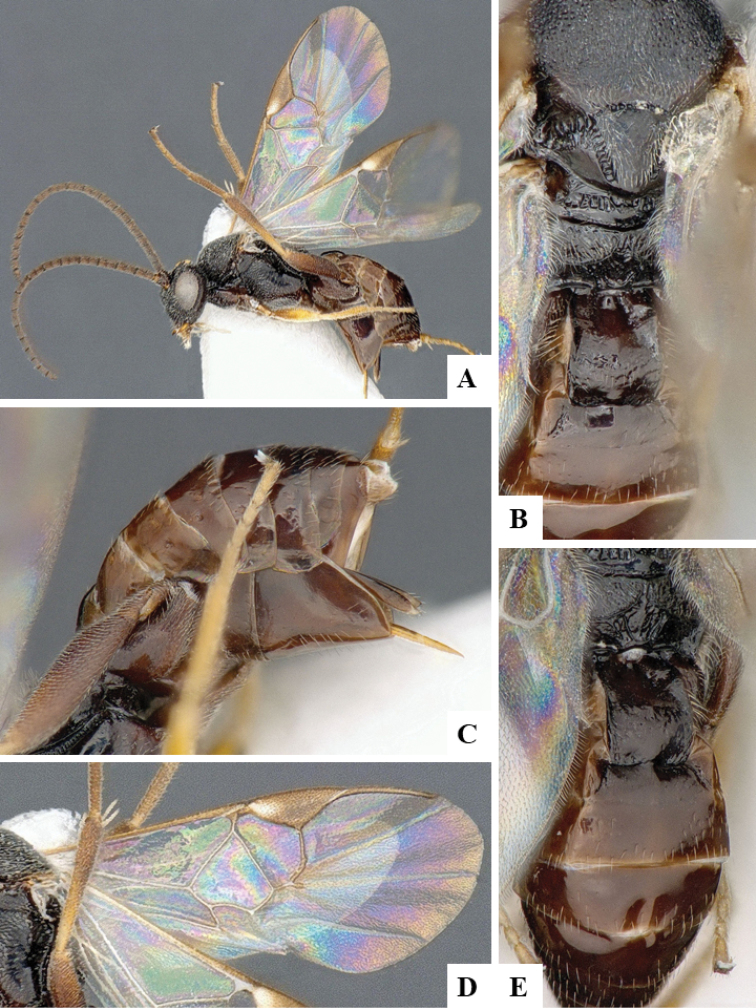
*Parapanteles* sp. female WAM 0186 **A** Habitus, lateral **B** Mesosoma and tergites 1–3, dorsal **C** Metasoma, lateral **D** Fore wing **E** Propodeum and metasoma, laterodorsal.

**Figure 176.**
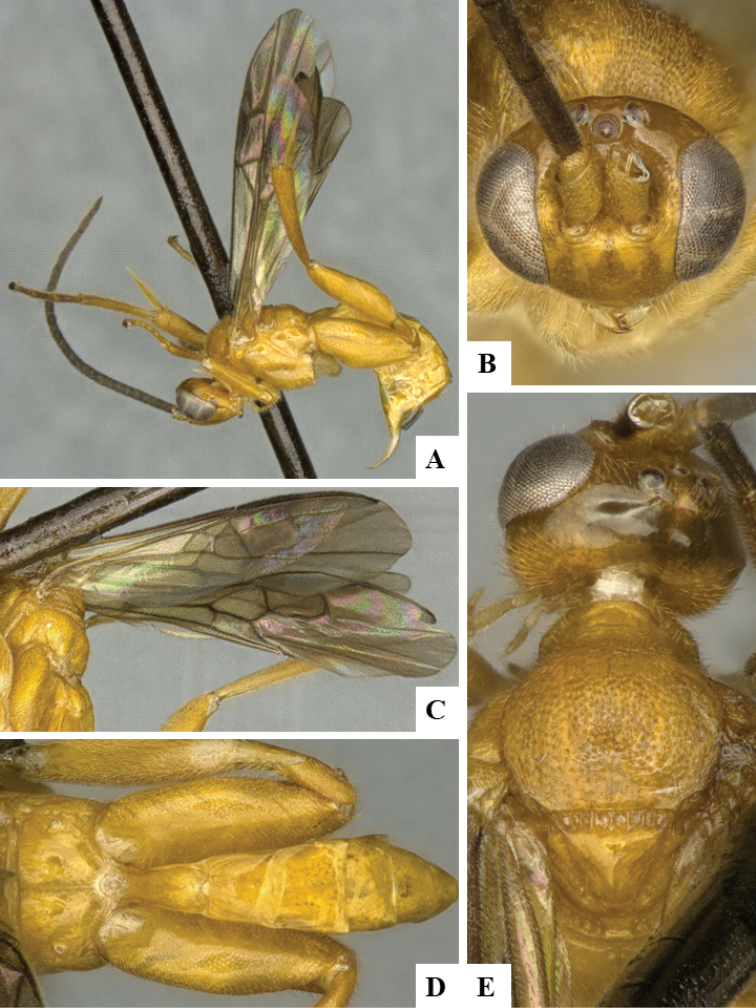
*Parenionkokodana* female CNCHYM01939 **A** Habitus, lateral **B** Head, frontal **C** Fore wing **D** Propodeum and metasoma, dorsal **E** Head and mesosoma, dorsal.

**Figure 177.**
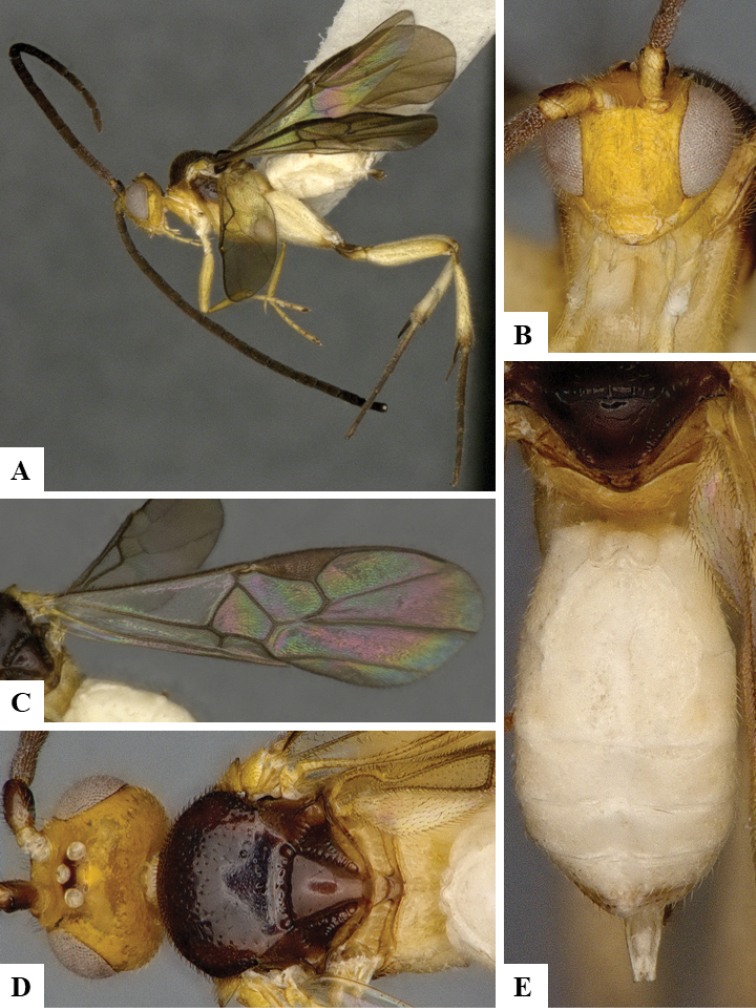
*Parenion* sp. male CNCHYM01945 **A** Habitus, lateral **B** Head, frontal **C** Fore wing **D** Head and mesosoma, dorsal **E** Metasoma, dorsal.

**Figure 178.**
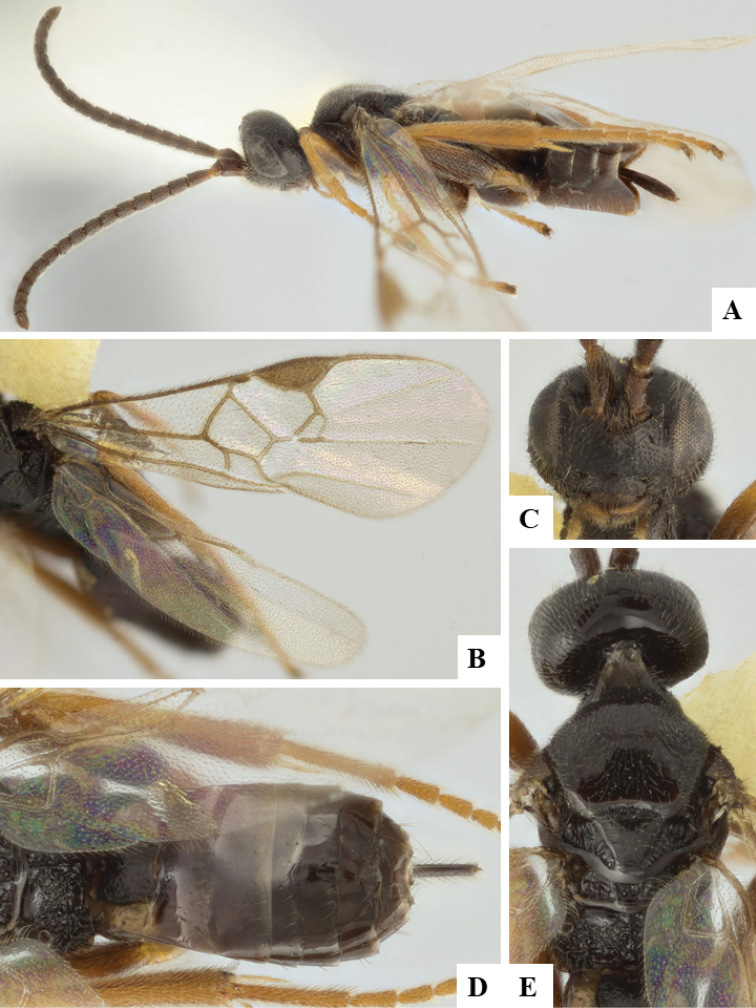
*Paroplitisberingianus* female holotype **A** Habitus, lateral **B** Fore wing **C** Head, frontal **D** Metasoma, dorsal **E** Head and mesosoma, dorsal.

**Figure 179.**
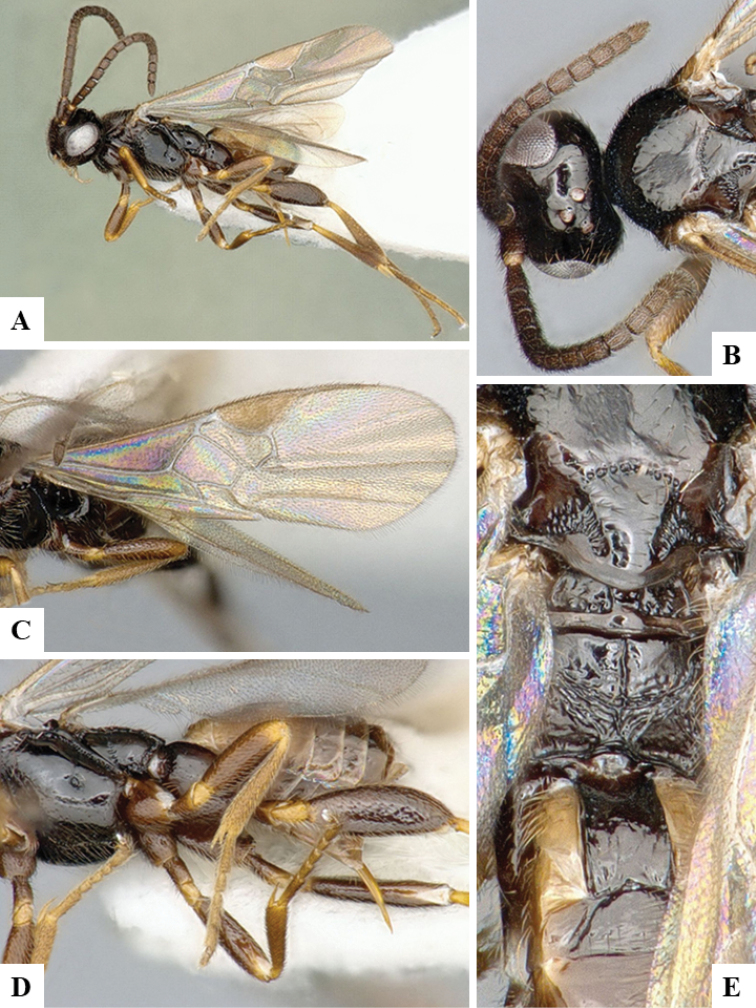
*Paroplitisvietnamensis* female holotype **A** Habitus, lateral **B** Head and mesosoma, dorsal **C** Fore wing **D** Mesosoma and metasoma, ventrolateral **E** Mesosoma and tergites 1–3, dorsal.

**Figure 180.**
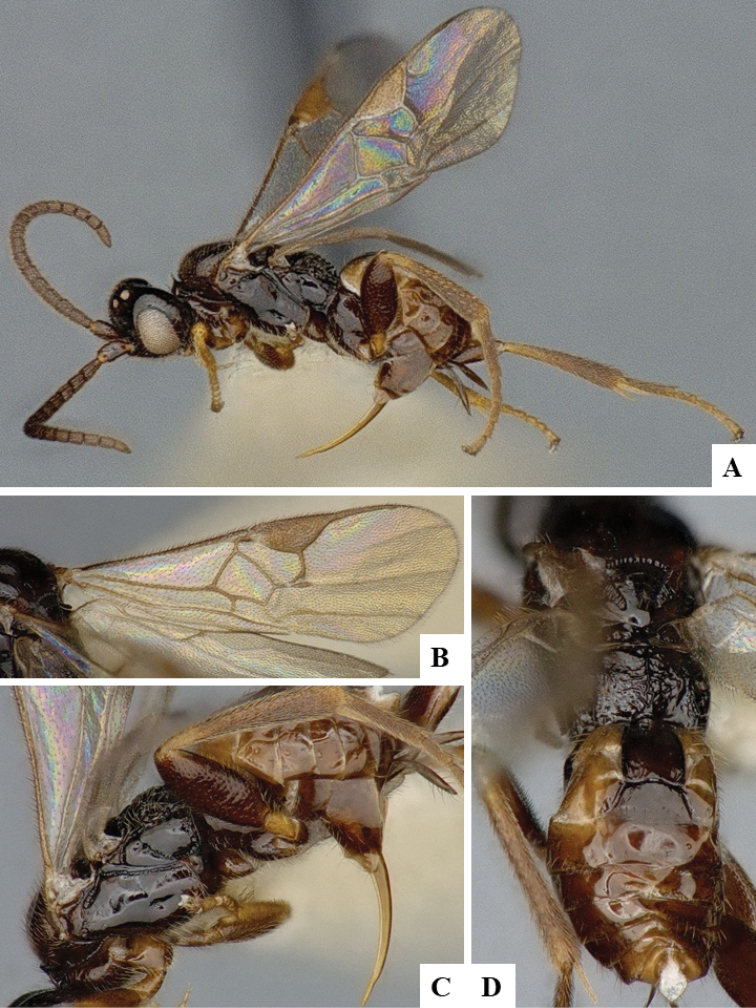
*Paroplitiswesmaeli* female CNCHYM01946 **A** Habitus, lateral **B** Fore wing **C** Mesosoma and metasoma, lateral **D** propodeum and metasoma, dorsal.

**Figure 181.**
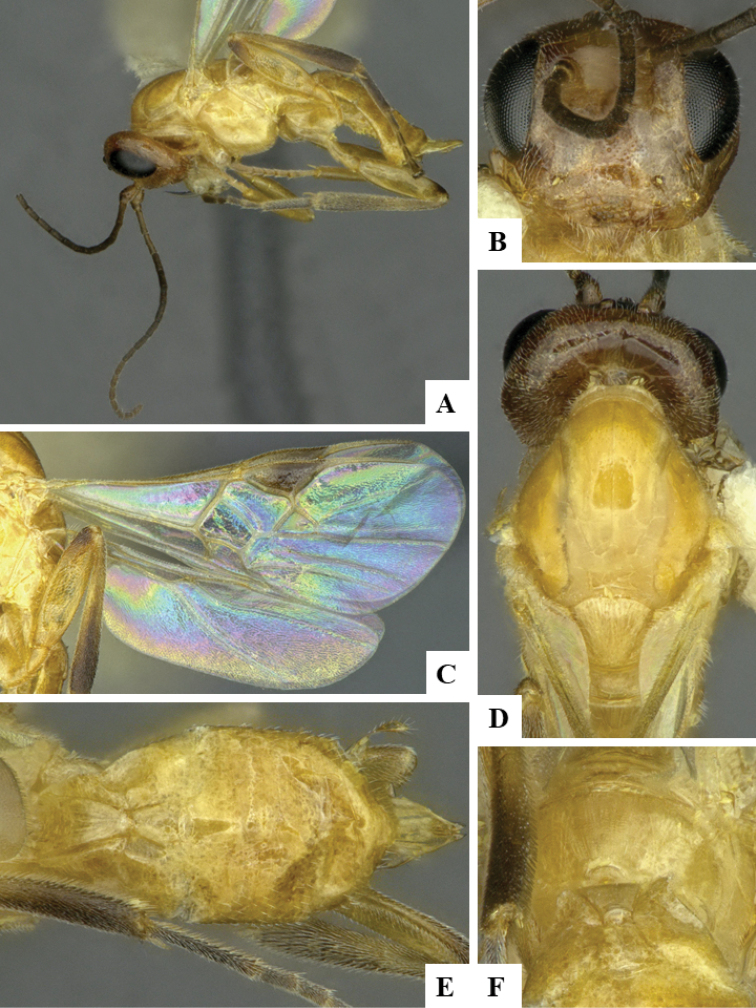
*Pelicopeyuccamica* male CNC309859 **A** Habitus, lateral **B** Head, dorsal **C** Fore wing and hind wing **D** Mesosoma, dorsal **E** Metasoma, dorsal **F** Propodeum and tergites 1–2, dorsal.

**Figure 182.**
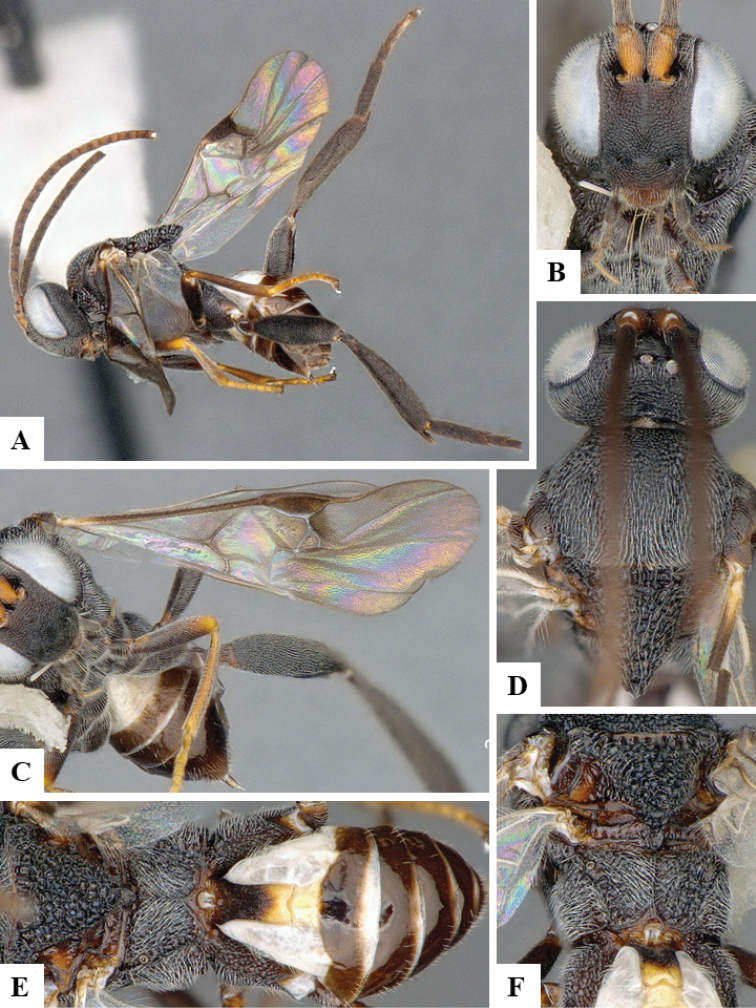
*Philoplitispunctatus* female CNC309861 **A** Habitus, lateral **B** Head, frontal **C** Fore wing **D** Head and mesosoma, dorsal **E** Propodeum and metasoma, dorsal **F** Propodeum and tergites 1–2, dorsal.

**Figure 183.**
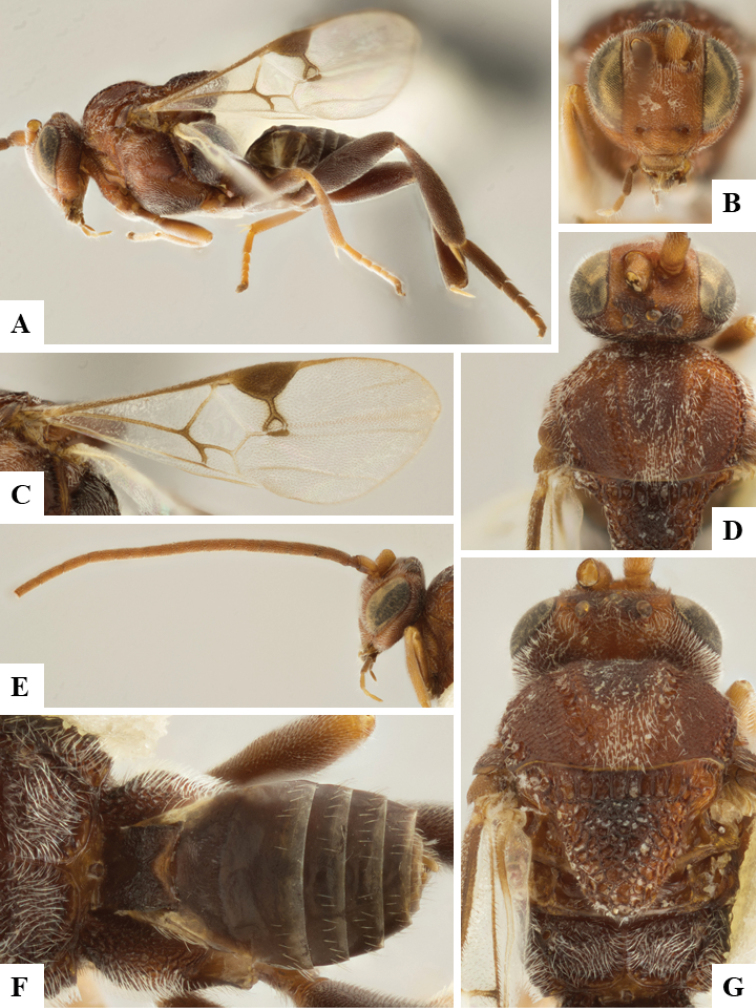
*Philoplitisstriatus* male holotype **A** Habitus, lateral **B** Head, frontal **C** Fore wing **D** Head and mesosoma, dorsal **E** Antenna **F** Metasoma, dorsal **G** Mesosoma, dorsal.

**Figure 184.**
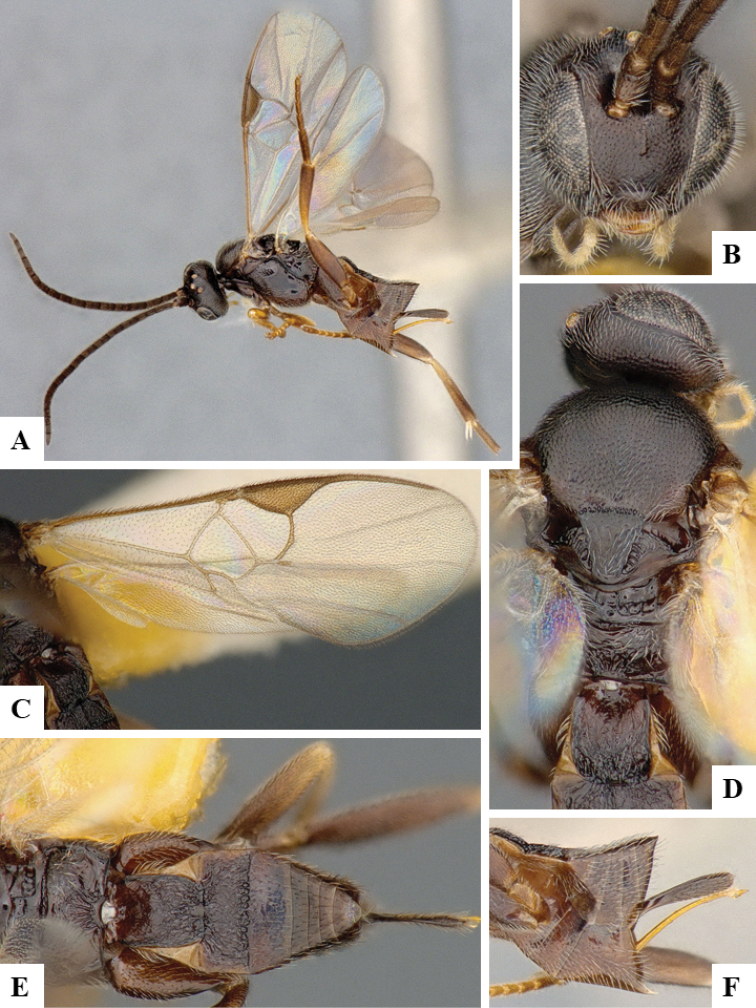
*Pholetesorbedelliae* female CNCHYM03137 **A** Habitus, lateral **B** Head, frontal **C** Fore wing **D** Mesosoma, dorsal **E** Propodeum and metasoma, dorsal **F** Ovipositor and ovipositor sheaths.

**Figure 185.**
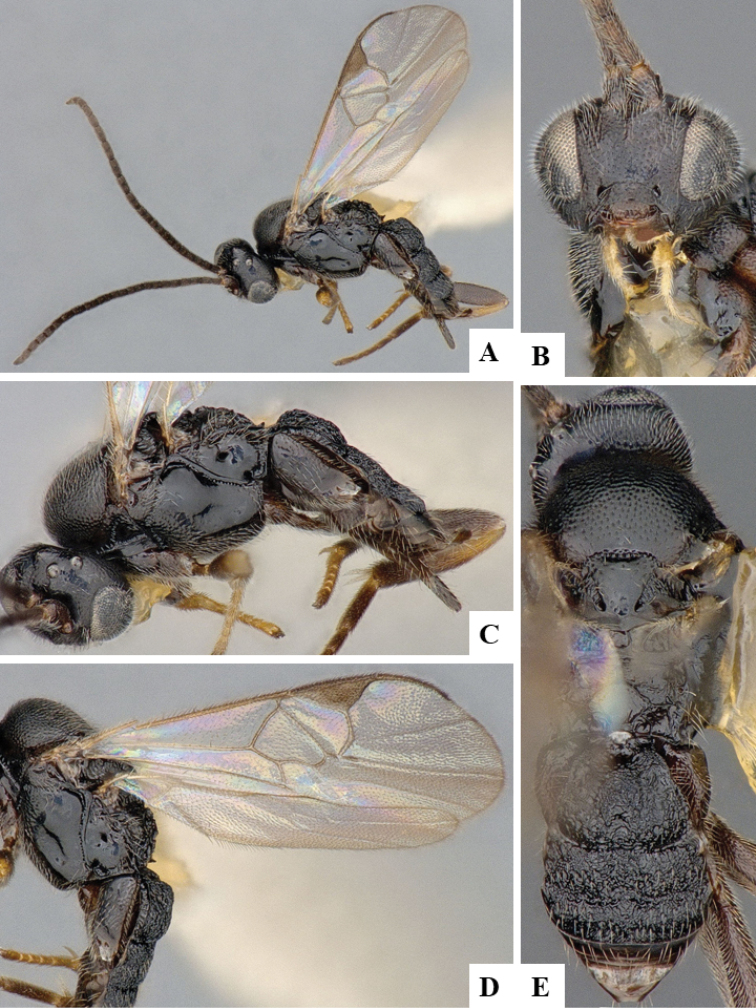
*Pholetesorbucculatricis* female CNCHYM03147 **A** Habitus, lateral **B** Head, frontal **C** Mesosoma and metasoma, lateral **D** Fore wing and hind wing **E** Mesosoma and metasoma, dorsal.

**Figure 186.**
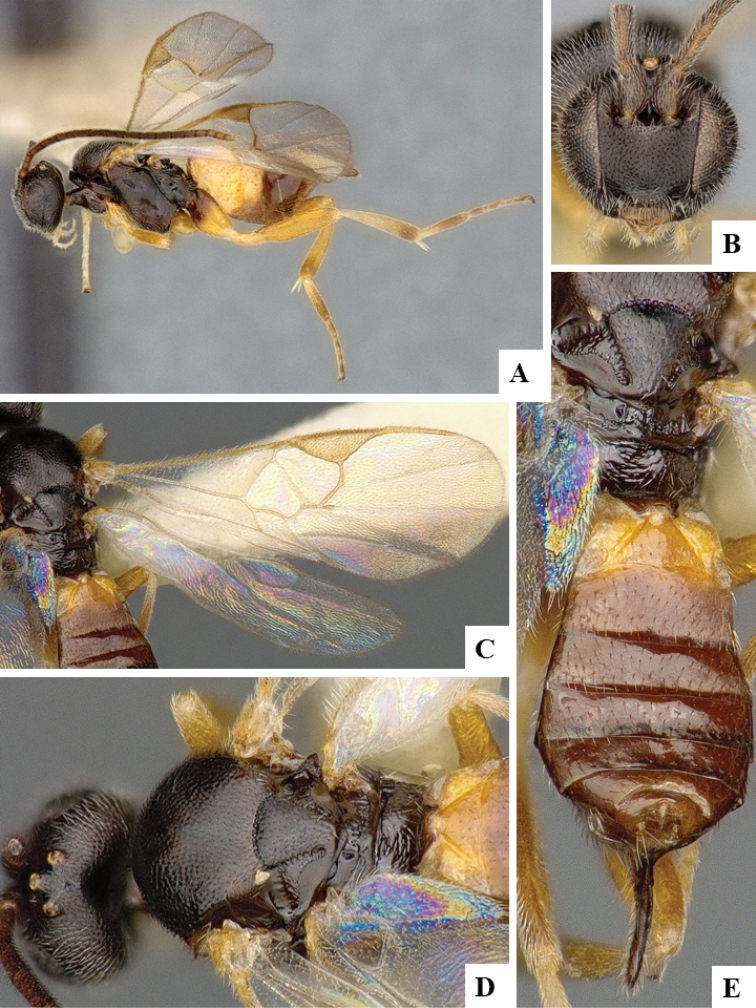
*Pholetesorexiguous* female CNCHYM03168 **A** Habitus, lateral **B** Head, frontal **C** Fore wing and hind wing **D** Head and mesosoma, dorsal **E** Propodeum and metasoma, dorsal.

**Figure 187.**
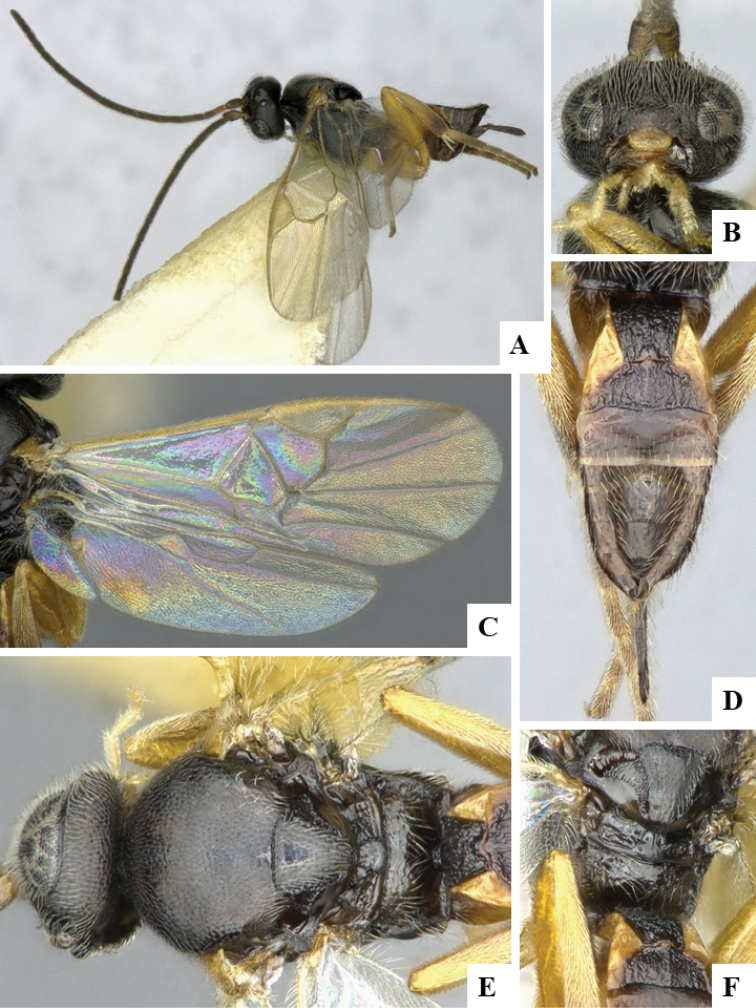
*Pholetesorlaetus* female CNCHYM03170 **A** Habitus, lateral **B** Head, frontoventral **C** Fore wing and hind wing **D** Metasoma, dorsal **E** Mesosoma, dorsal **F** Propodeum and tergites 1–2, dorsolateral.

**Figure 188.**
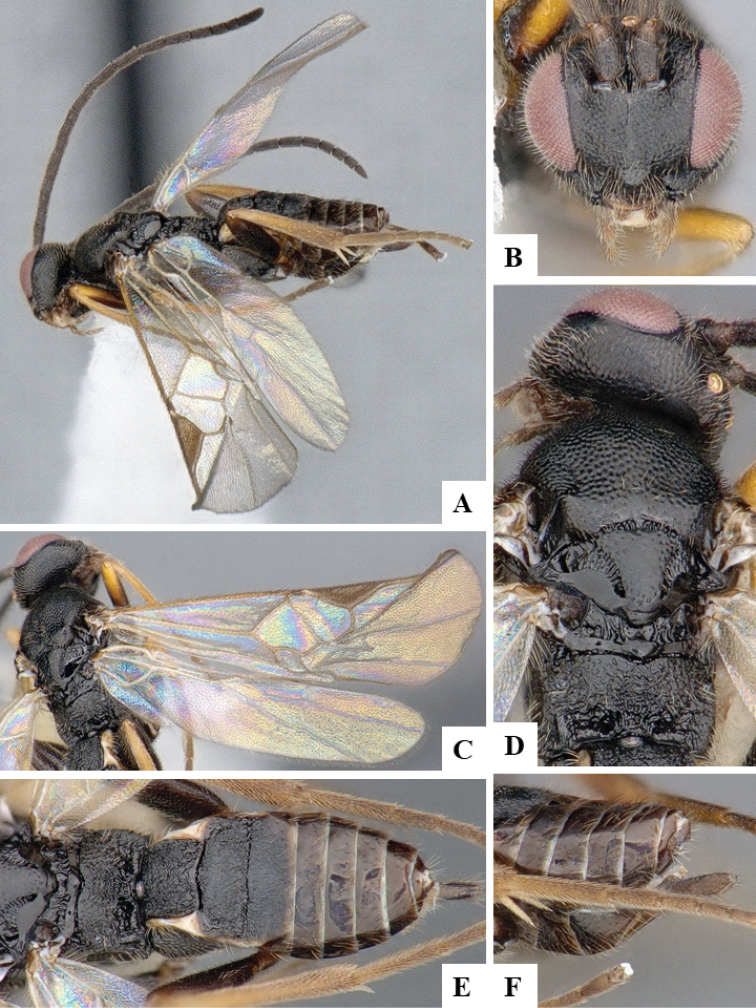
*Pholetesormaritimus* female MRSJFT0464 **A** Habitus, lateral **B** Head, frontal **C** Fore wing and hind wing **D** Mesosoma, dorsal **E** Propodeum and metasoma, dorsal **F** Apex of metasoma, lateral.

**Figure 189.**
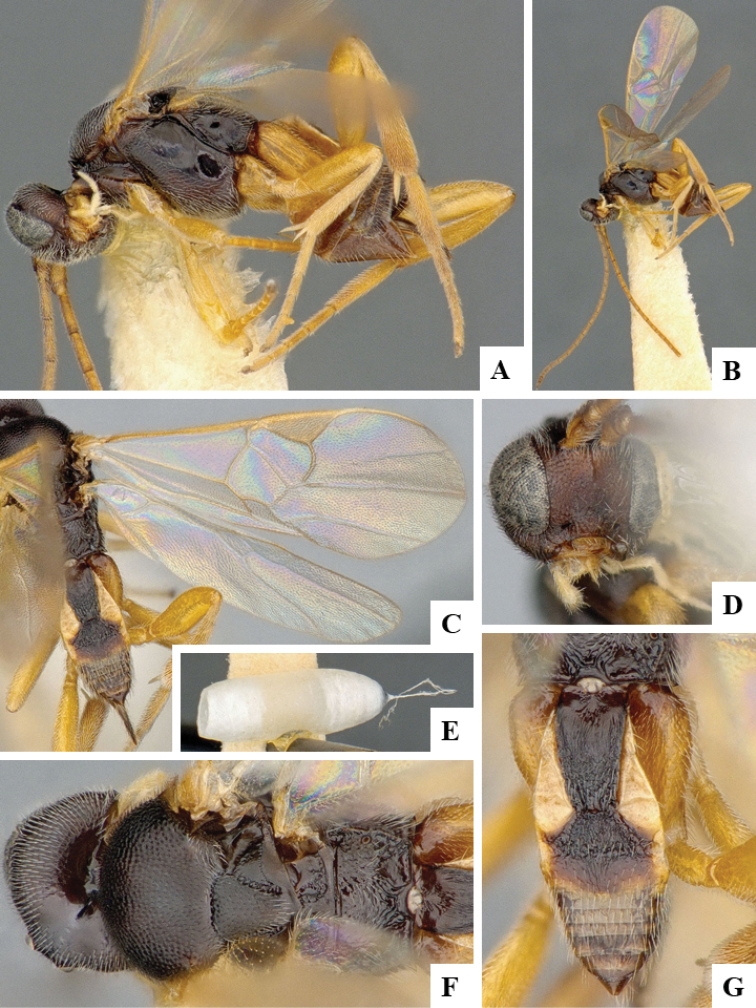
*Pholetesorsalalicus* female CNC483619 **A** Habitus magnified, lateral **B** Habitus, lateral **C** Fore wing and hind wing **D** Head, frontal **E** Cocoon **F** Mesosoma, dorsal **G** Metasoma, dorsal.

**Figure 190.**
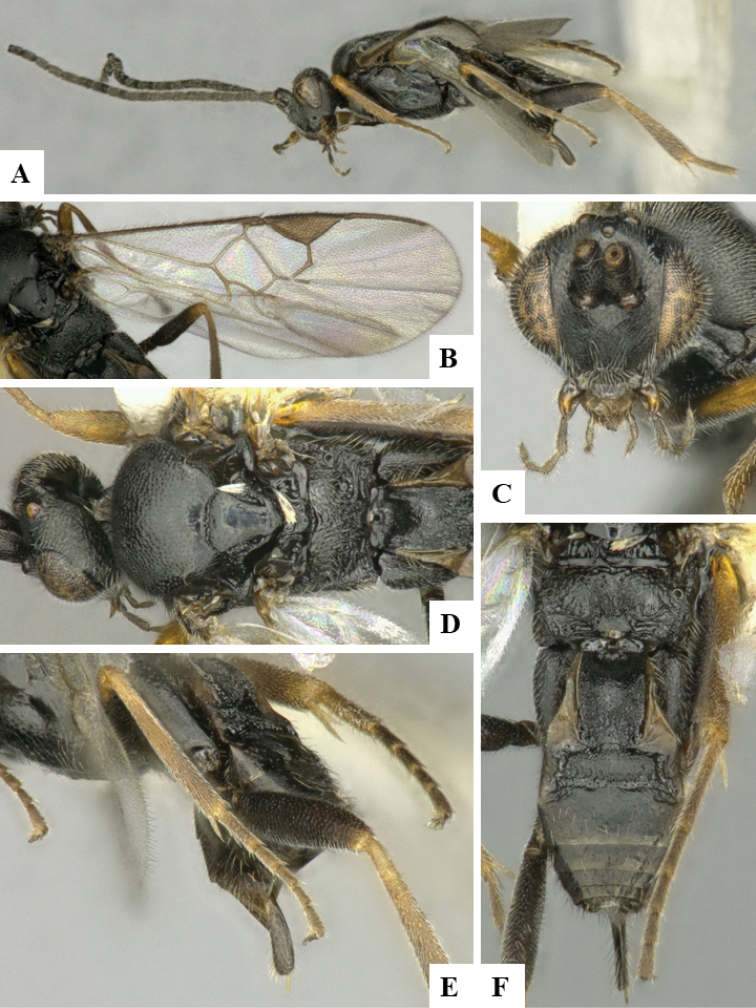
*Pholetesorviminetorum* female CNC678004 **A** Habitus, lateral **B** Fore wing **C** Head, frontal **D** Mesosoma, dorsal **E** Ovipositor sheaths **F** Propodeum and metasoma, dorsal.

**Figure 191.**
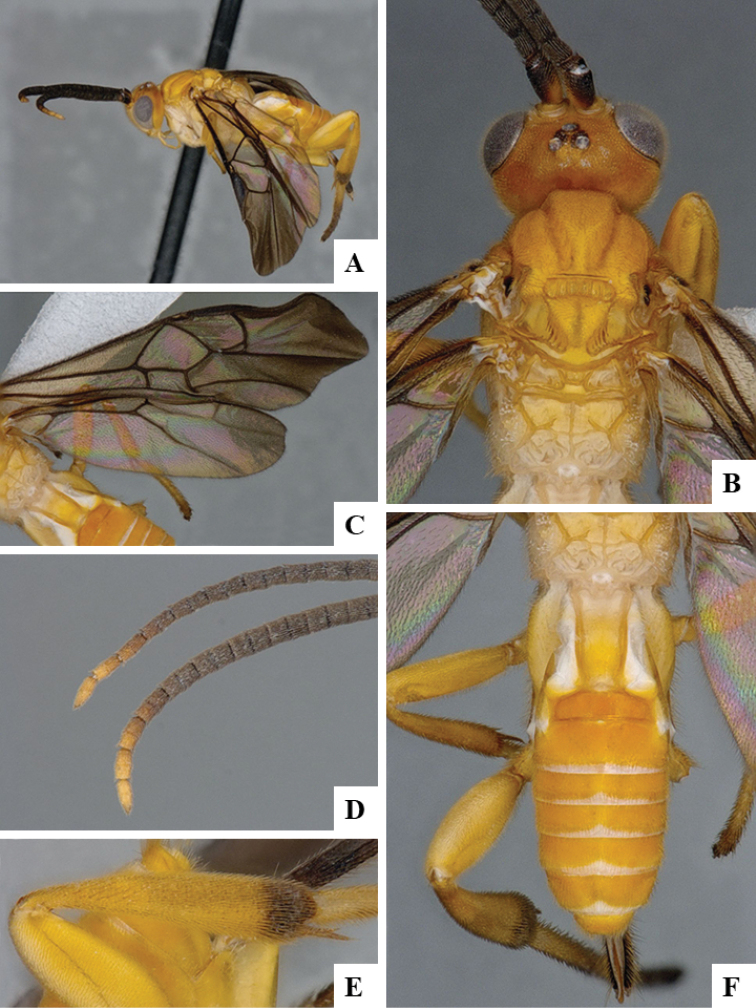
*Prasmodonalmasolisae* female holotype **A** Habitus, lateral **B** Head and mesosoma, dorsal **C** Fore wing and hind wing **D** Apex of antennae **E** Hind tibia, lateral **F** Metasoma, dorsal.

**Figure 192.**
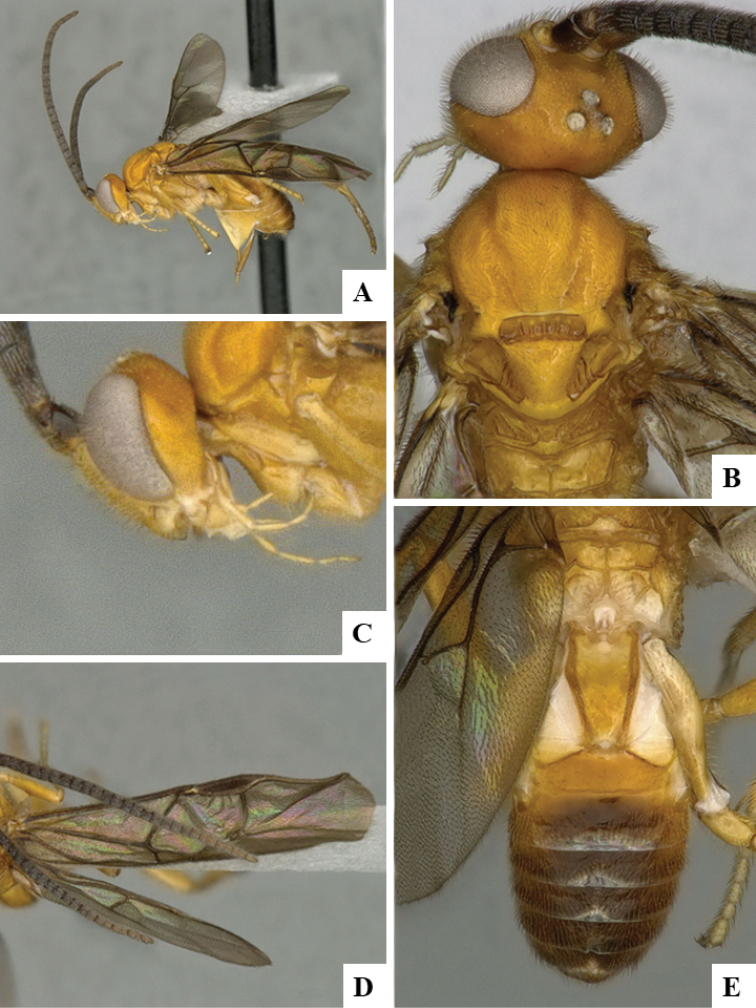
*Prasmodonbobrobbinsi* female holotype **A** Habitus, lateral **B** Head and mesosoma, dorsal **C** Head, lateral **D** Fore wing and hind wing **E** Propodeum and metasoma, dorsal.

**Figure 193.**
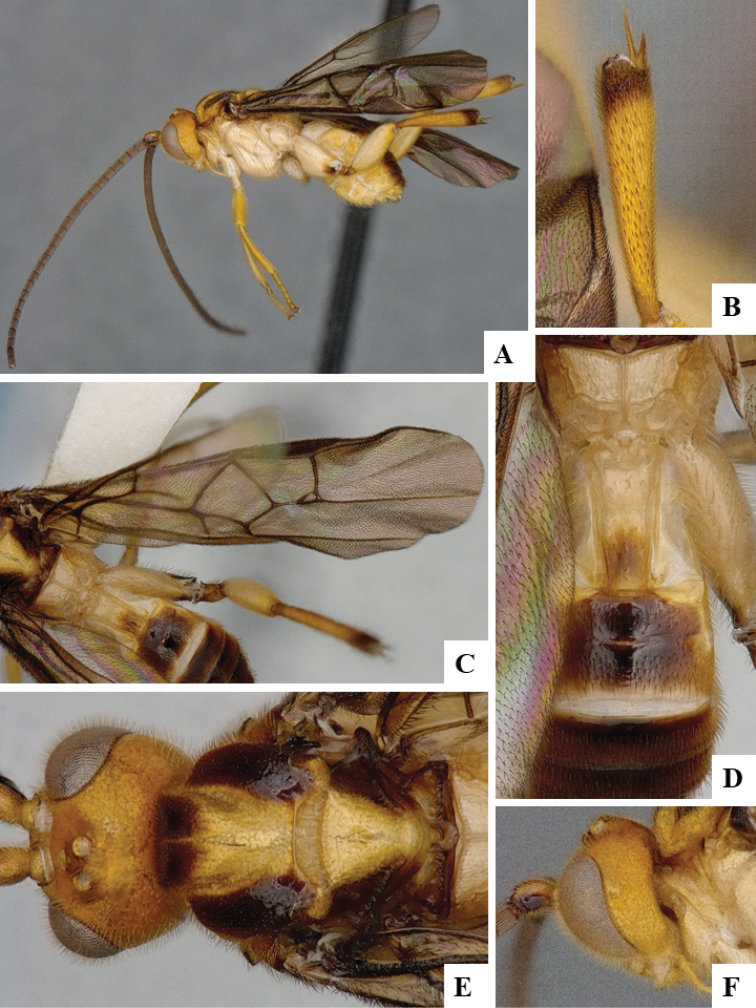
*Prasmodonsubfuscus* male holotype **A** Habitus, lateral **B** Hind tibia, lateral **C** Fore wing **D** Propodeum and metasoma, dorsal **E** Head and mesosoma, dorsal **F** Head, lateral.

**Figure 194.**
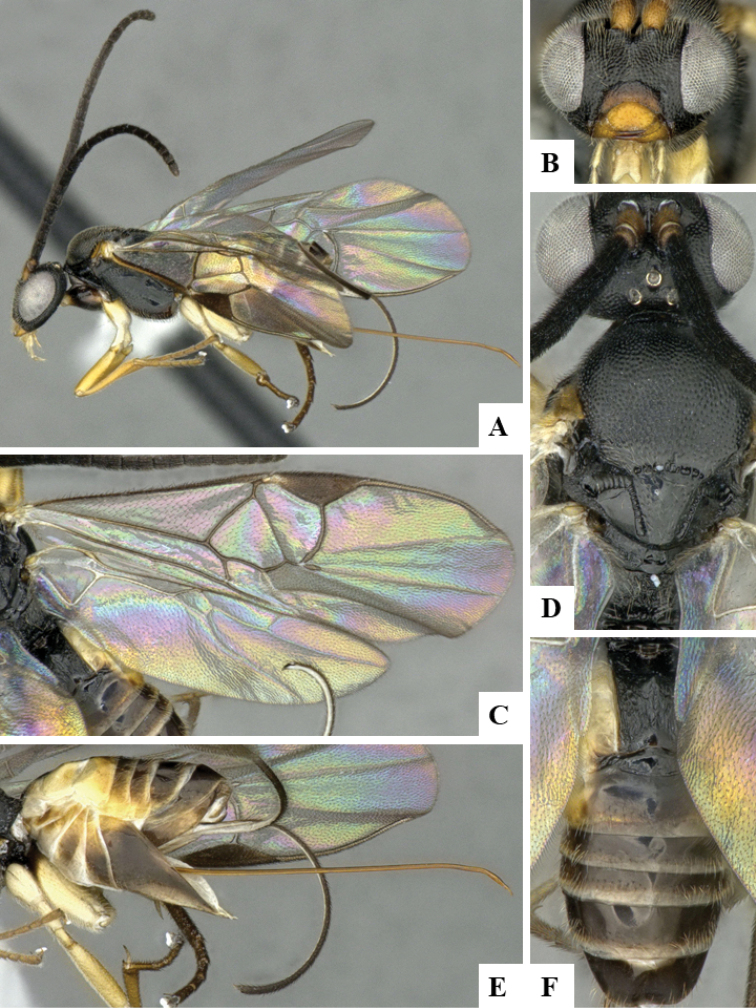
*Promicrogasterbrandondinartei* female DHJPAR0031326 **A** Habitus, lateral **B** Head, frontal **C** Fore wing and hind wing **D** Head and mesosoma, dorsal **E** Metasoma, lateral **F** Metasoma, dorsal.

**Figure 195.**
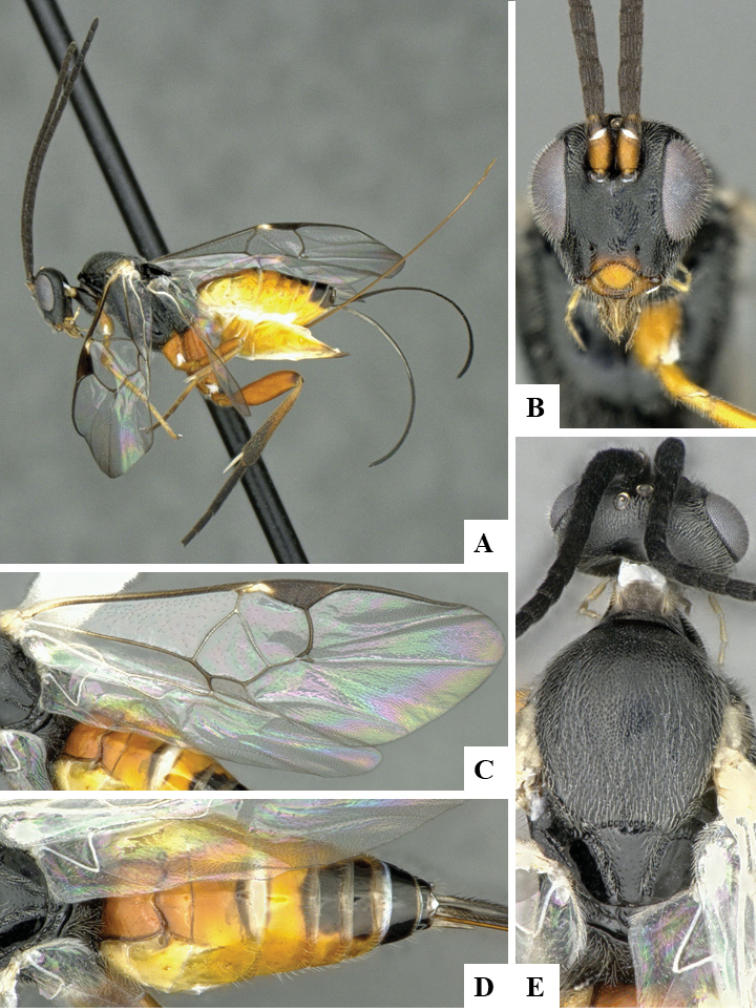
*Promicrogasterfabriciocambroneroi* female DHJPAR0012588 **A** Habitus, lateral **B** Head, frontal **C** Fore wing and hind wing **D** Metasoma, dorsal **E** Mesosoma, dorsal.

**Figure 196.**
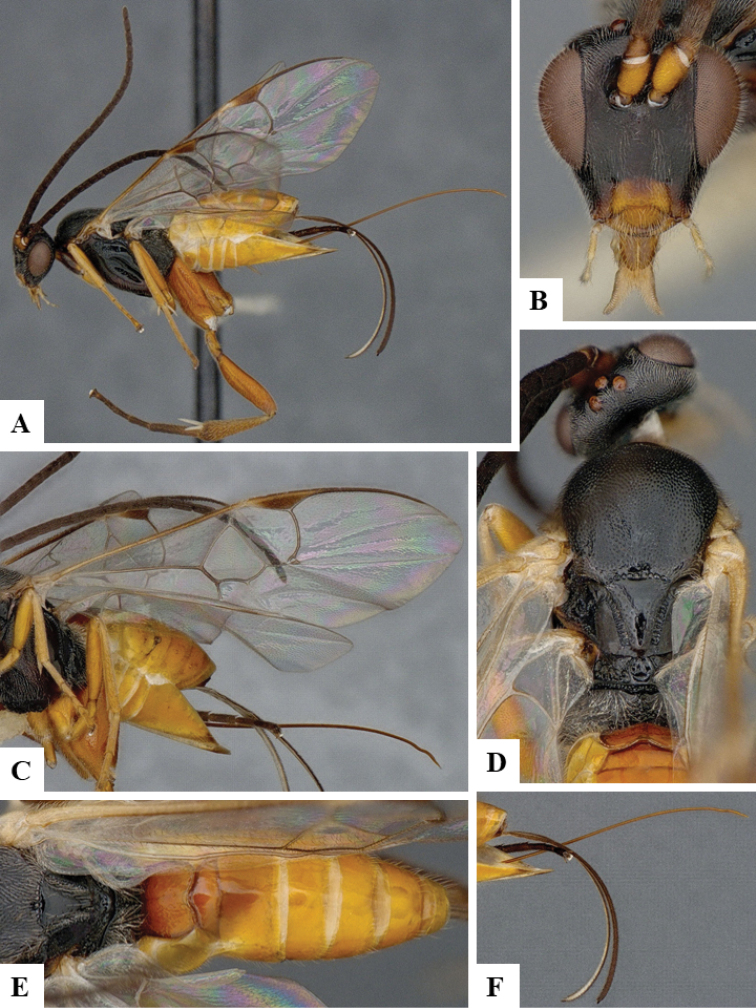
*Promicrogastermiranda* female CNCHYM01980 **A** Habitus, lateral **B** Head, frontal **C** Fore wing **D** Head and mesosoma, dorsal **E** Metasoma, dorsal **F** Ovipositor and ovipositor sheaths.

**Figure 197.**
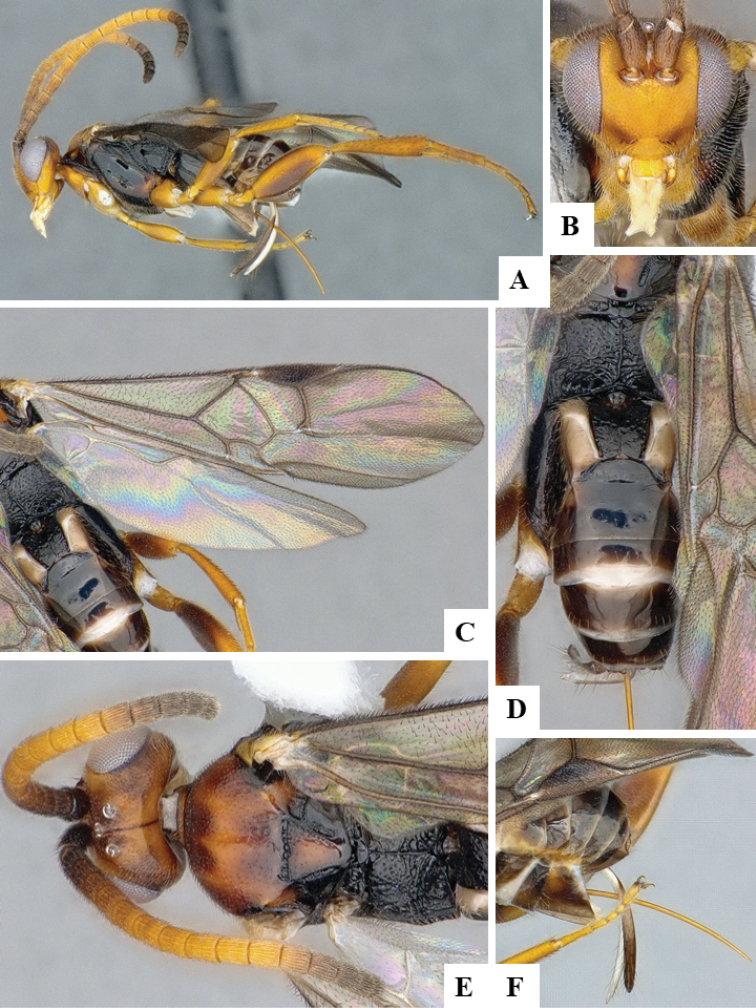
*Promicrogasterpablouzagai* female DHJPAR0025926 **A** Habitus, lateral **B** Head, frontal **C** Fore wing and hind wing **D** Propodeum and metasoma, dorsal **E** Head and mesosoma, dorsal **F** Ovipositor and ovipositor sheaths.

**Figure 198.**
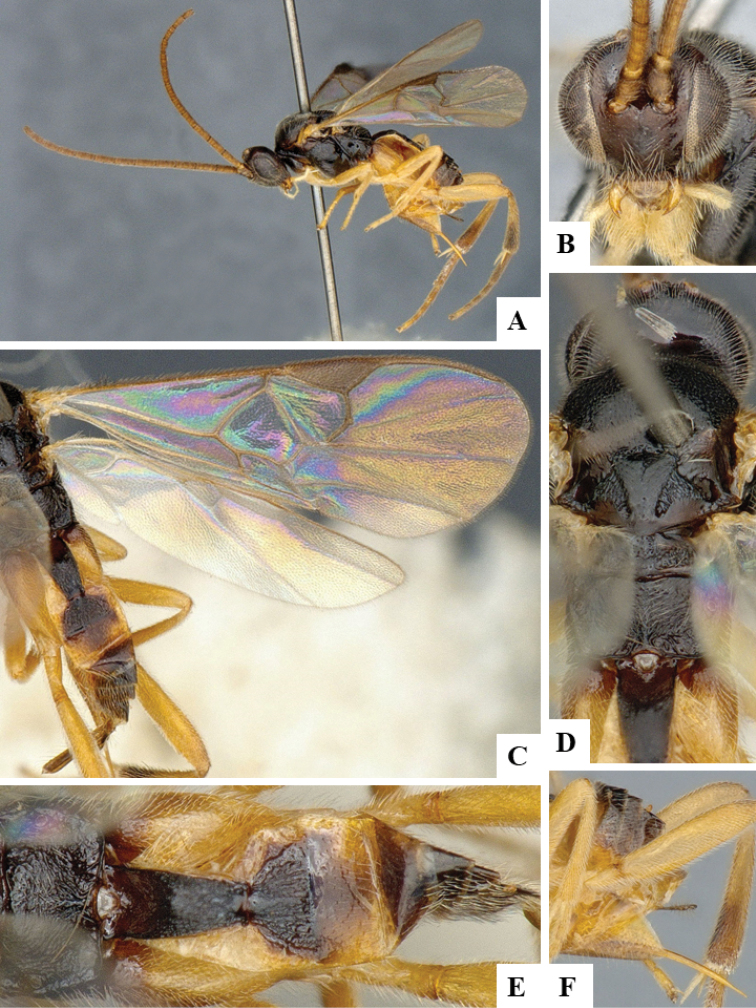
*Sathonfausta* female CNC474693 **A** Habitus, lateral **B** Head, frontal **C** Fore wing and hind wing **D** Mesosoma, dorsal **E** Propodeum and metasoma, dorsal **F** Ovipositor and ovipositor sheaths.

**Figure 199.**
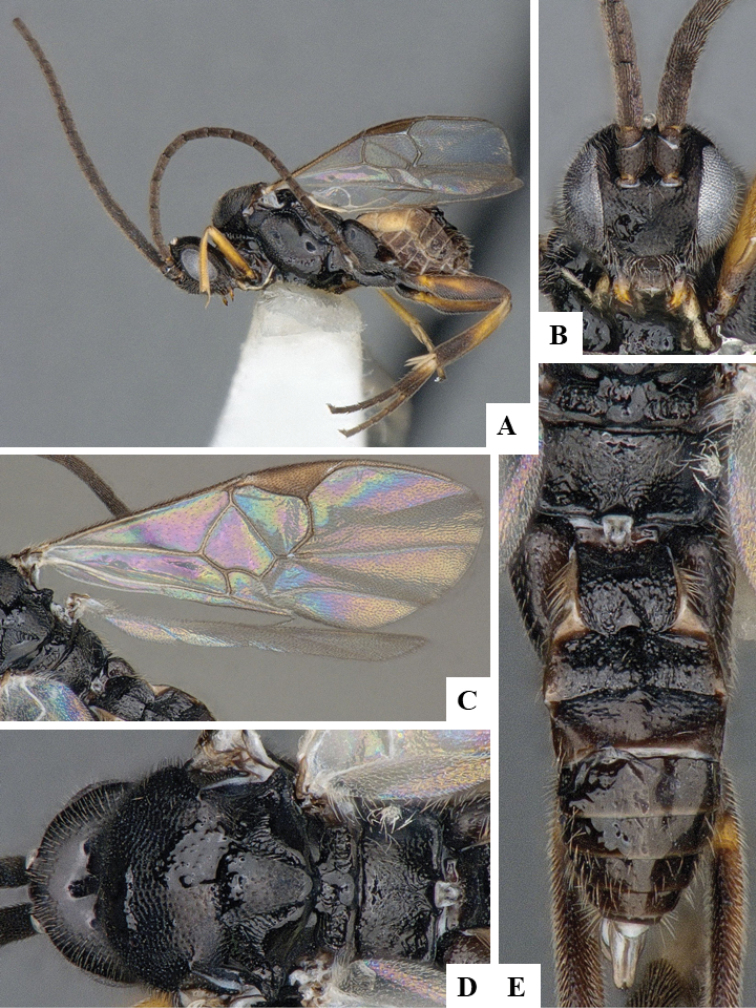
*Protapantelesimmunis* male CNC841408 **A** Habitus, lateral **B** Head, frontal **C** Fore wing **D** Mesosoma, dorsal **E** Propodeum and metasoma, dorsal.

**Figure 200.**
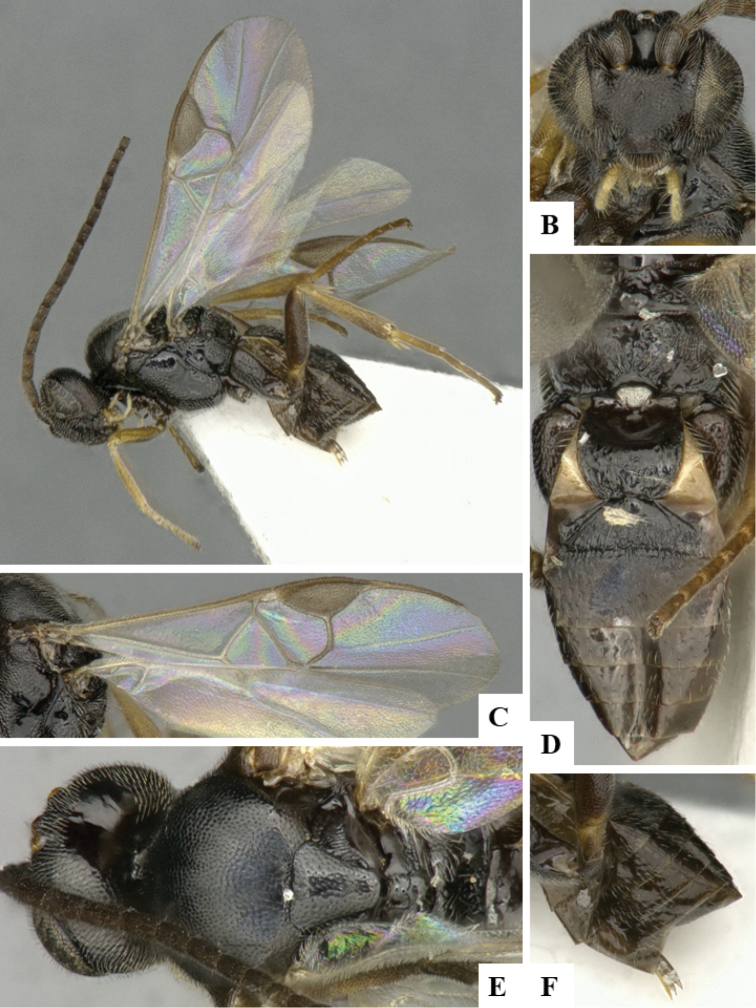
*Protapantelespopularis* female CNC309903 **A** Habitus, lateral **B** Head, frontal **C** Fore wing and hind wing **D** Propodeum and metasoma, dorsal **E** Mesosoma, dorsal **F** Metasoma, lateral.

**Figure 201.**
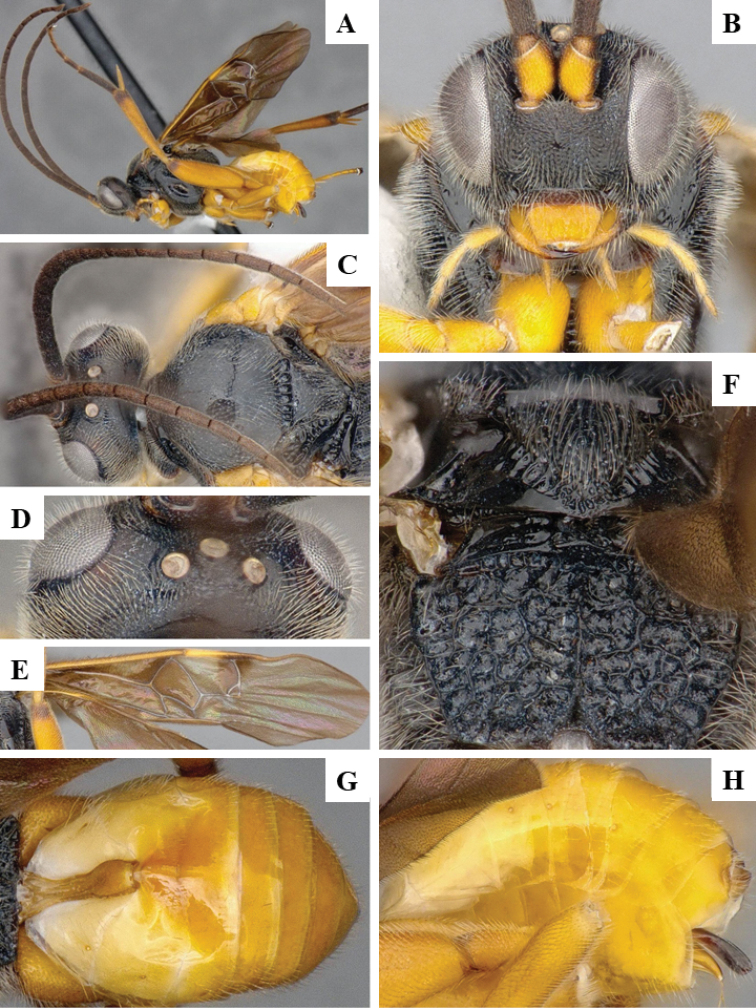
*Protomicroplitiscalliptera* female CNCH1333 **A** Habitus, lateral **B** Head, frontal **C** Head and mesosoma, dorsal **D** Head, dorsal **E** Fore wing **F** Propodeum, dorsal **G** Metasoma, dorsal **H** Metasoma, lateral.

**Figure 202.**
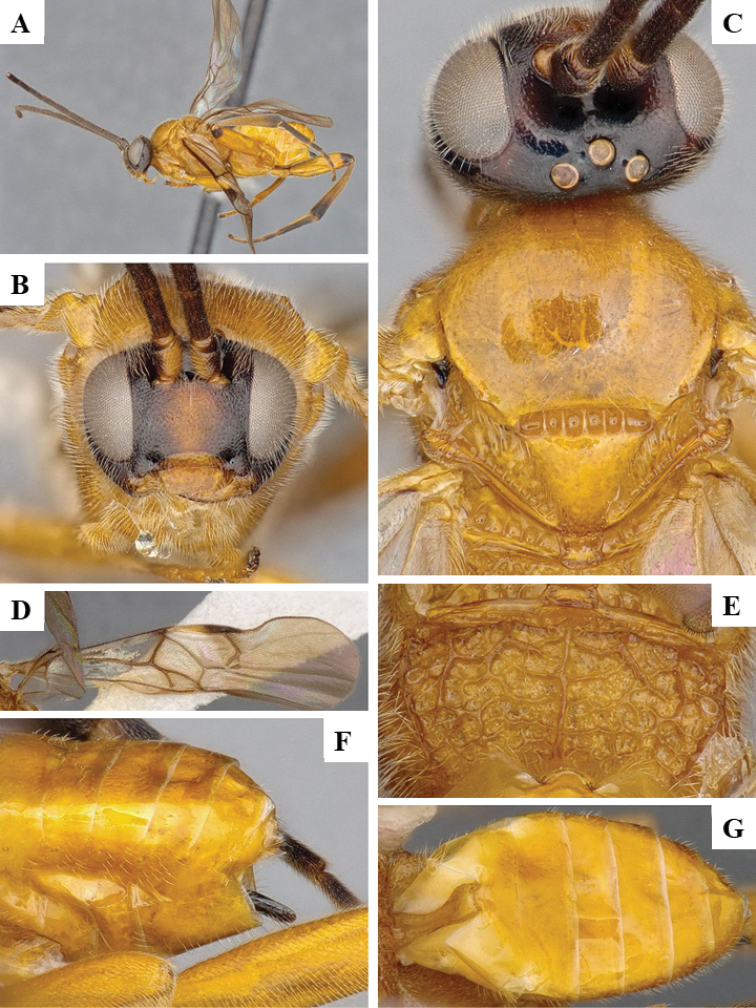
*Protomicroplitiscentroamericanus* female holotype **A** Habitus, lateral **B** Head, frontal **C** Head and mesosoma, dorsal **D** Fore wing **E** Propodeum, dorsal **F** Apex of metasoma, lateral **G** Metasoma, dorsal.

**Figure 203.**
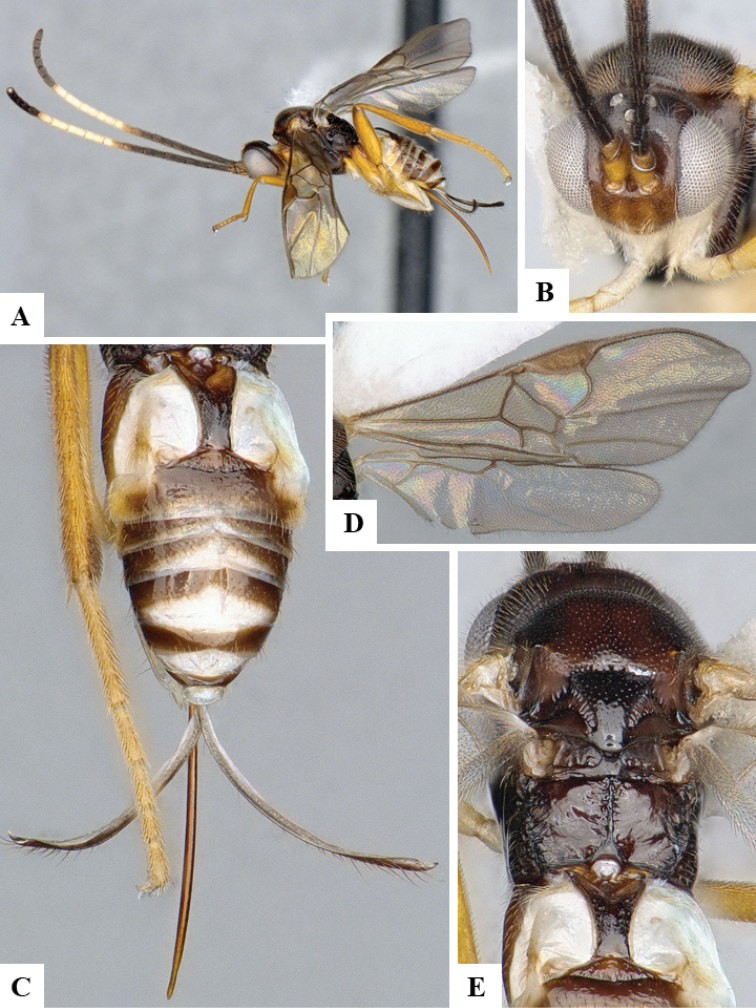
*Pseudapanteleschristinafigueresae* female holotype **A** Habitus, lateral **B** Head, frontal **C** Metasoma, dorsal **D** Fore wing and hind wing **E** Mesosoma and tergites 1–2, dorsal.

**Figure 204.**
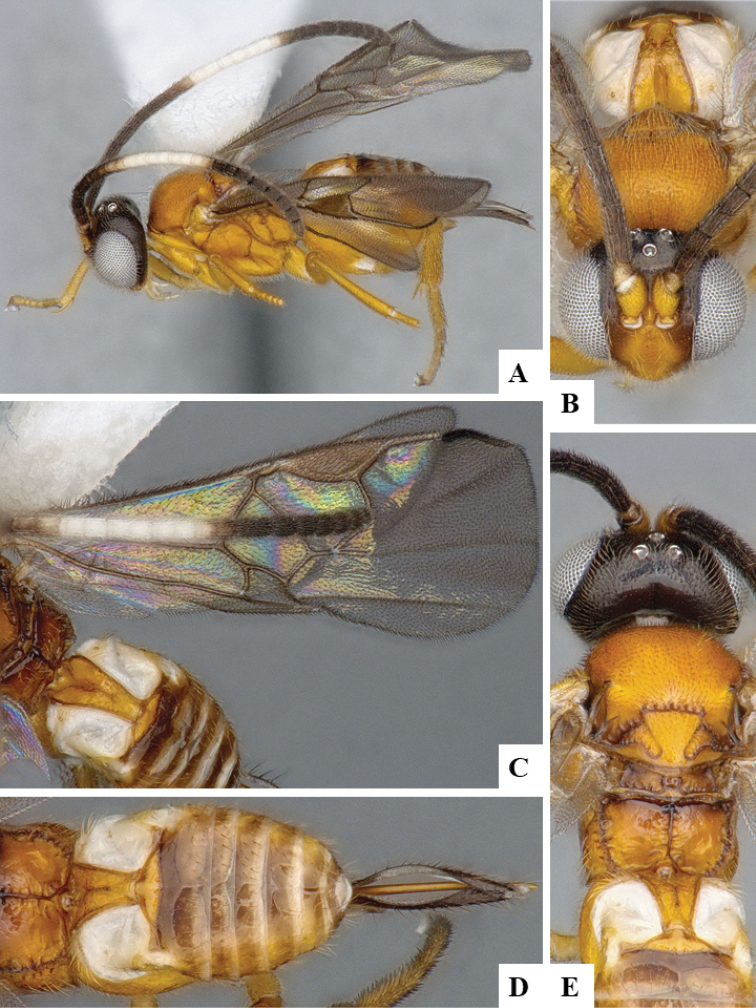
*Pseudapantelesmargaritapenonae* female holotype **A** Habitus, lateral **B** Head, frontal **C** Fore wing **D** Metasoma, dorsal **E** Head and mesosoma, dorsal.

**Figure 205.**
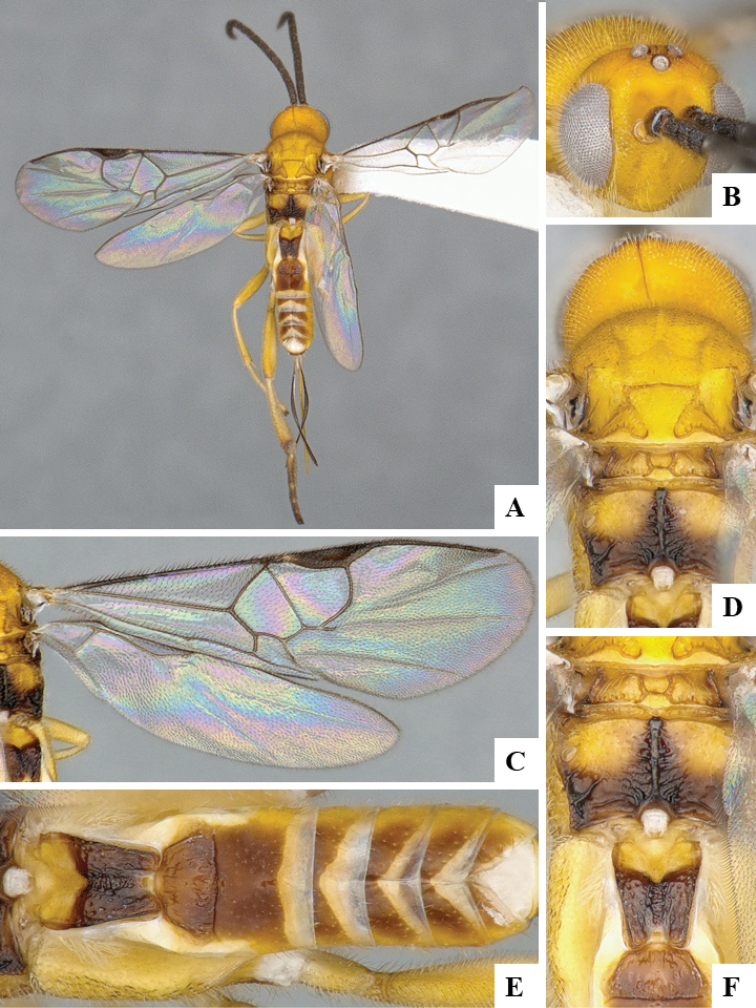
*Pseudapantelesmariocarvajali* female holotype **A** Habitus, dorsal **B** Head, frontal **C** Fore wing and hind wing **D** Mesosoma, dorsal **E** Metasoma, dorsal **F** Propodeum and tergites 1–2, dorsal.

**Figure 206.**
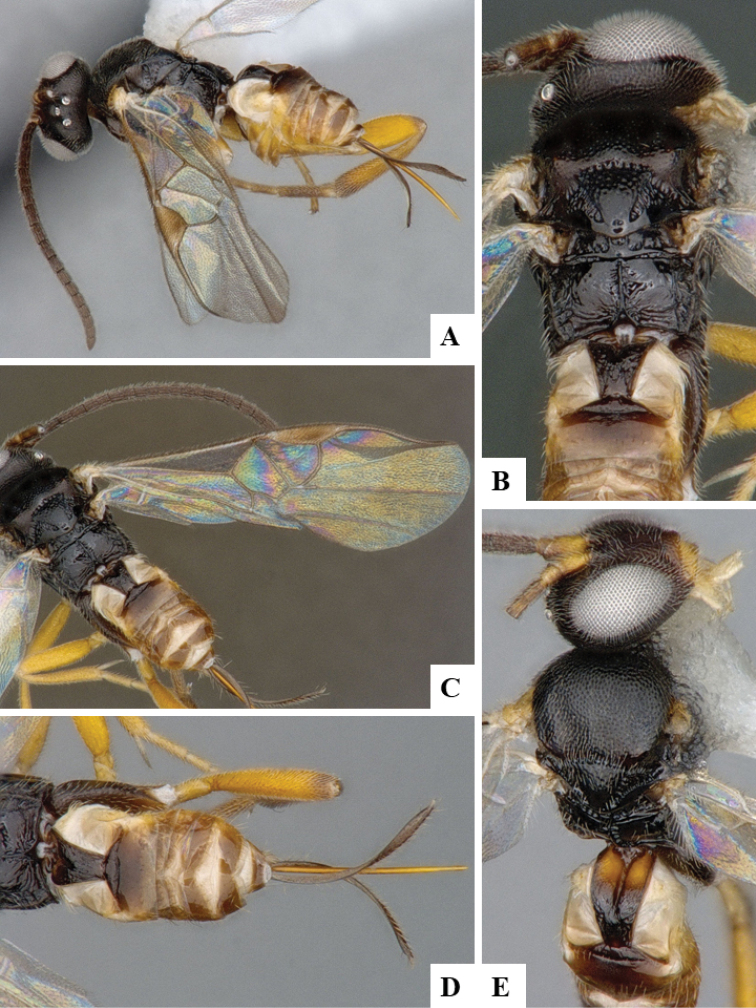
*Pseudapantelesrenecastroi* female holotype **A** Habitus, lateral **B** Mesosoma and tergites 1–3, dorsal **C** Fore wing **D** Metasoma, dorsal **E** Mesosoma and tergites 1–3, laterodorsal.

**Figure 207.**
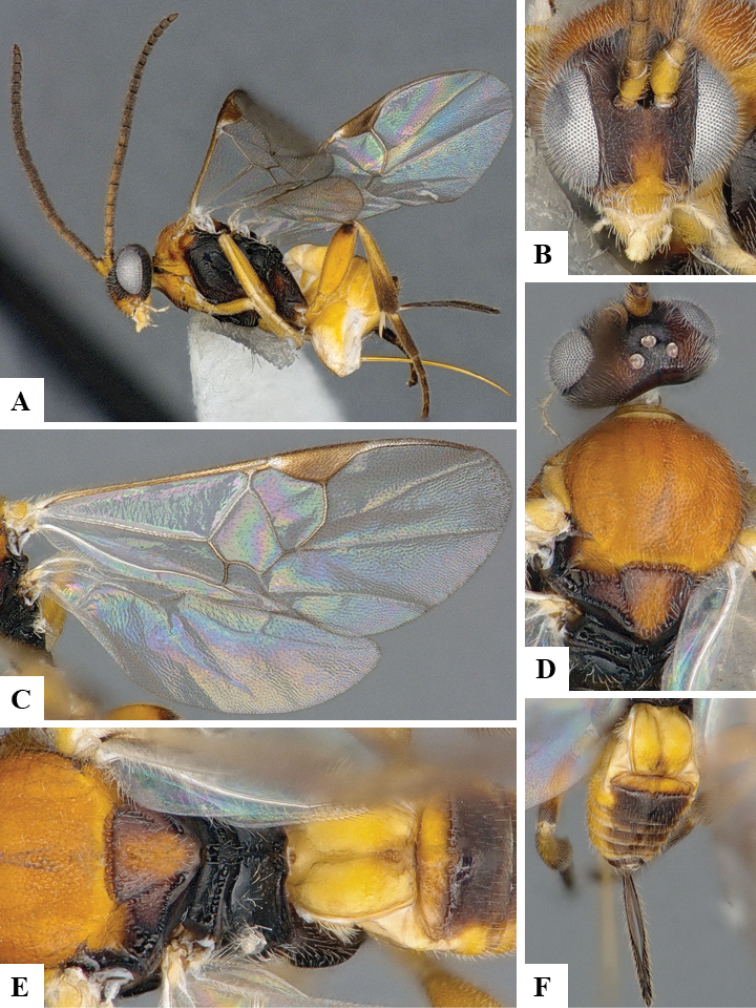
*Pseudapantelesteofilodelatorrei* female holotype **A** Habitus, lateral **B** Head, frontal **C** Fore wing and hind wing **D** Head and mesosoma, dorsal **E** Tergites 1–2, dorsal **F** Metasoma, dorsolateral.

**Figure 208.**
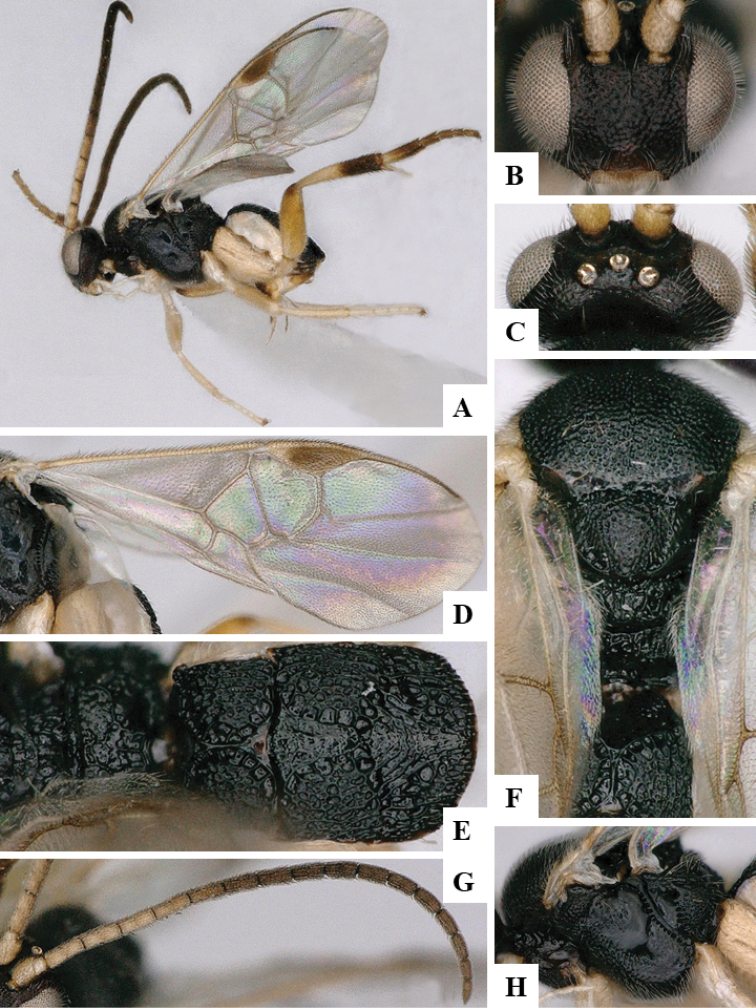
*Pseudofornicianigrisoma* female holotype based on modified images from the original descriptions of the species (van Achterberg et al. 2015) **A** Habitus, lateral **B** Head, frontal **C** Head, dorsal **D** Fore wing **E** Propodeum and metasoma, dorsal **F** Mesosoma, dorsal **G** Antenna **H** Mesosoma, lateral.

**Figure 209.**
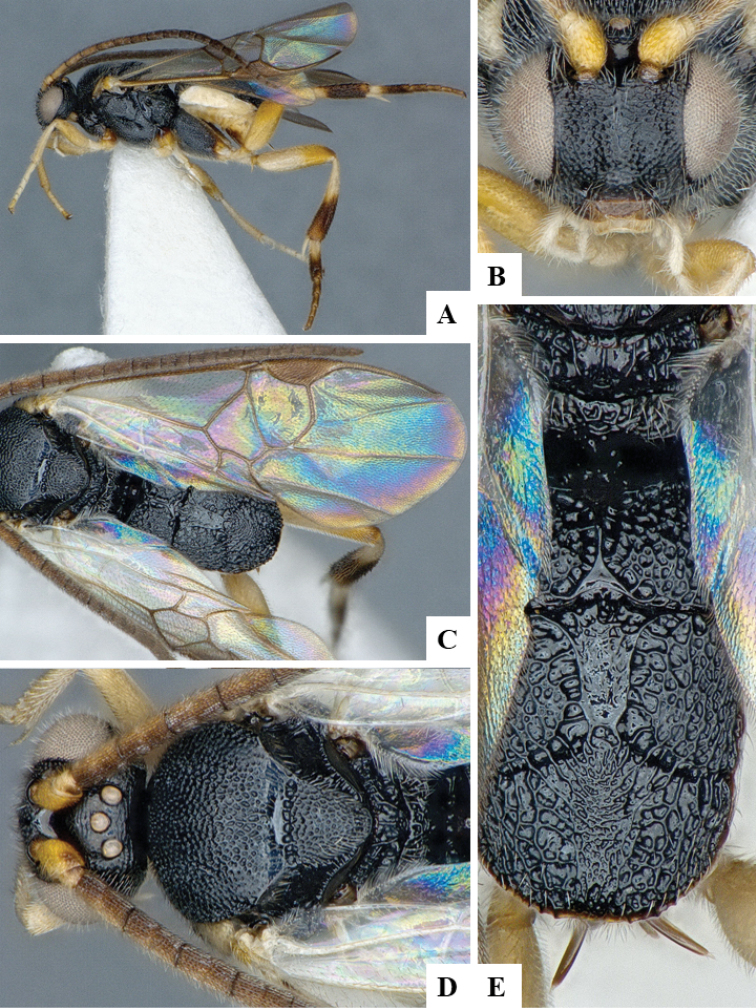
*Pseudofornicia* sp. female CNC92461 **A** Habitus, lateral **B** Head, frontal **C** Fore wing **D** Head and mesosoma, dorsal **E** Metasoma, dorsal.

**Figure 210.**
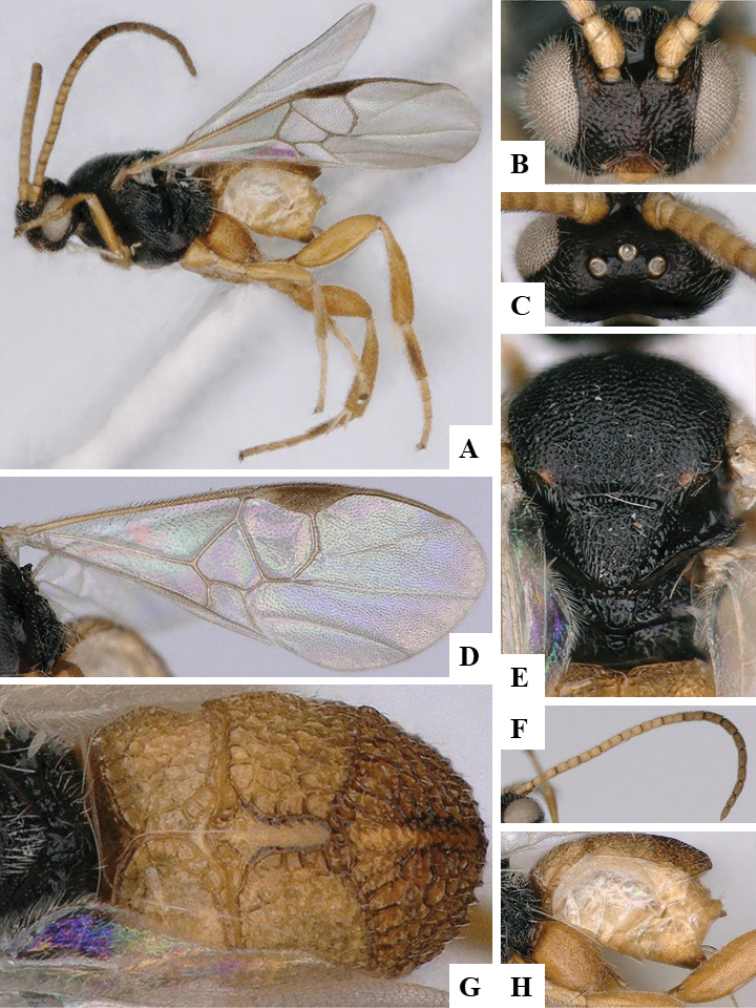
*Pseudoforniciavanachterbergi* female holotype based on modified images from van Achterberg et al. (2015)**A** Habitus, lateral **B** Head, frontal **C** Head, dorsal **D** Fore wing **E** Mesosoma, dorsal **F** Antenna **G** Metasoma, dorsal **H** Metasoma, lateral.

**Figure 211.**
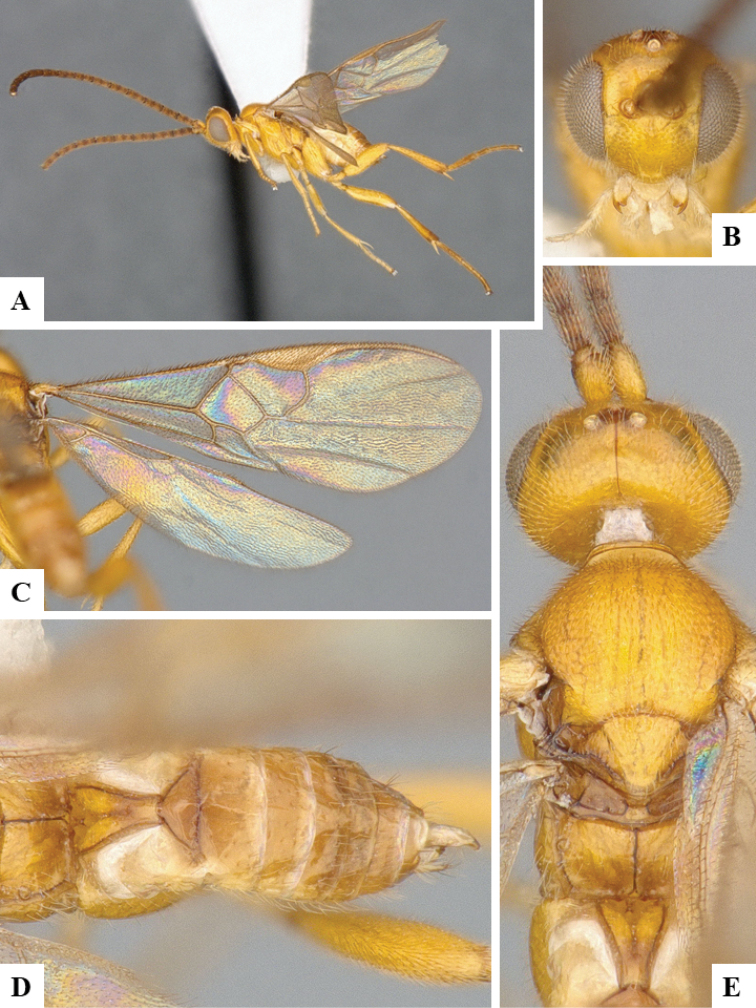
*Pseudovenanides* sp. male CNC661265 **A** Habitus, lateral **B** Head, frontal **C** Fore wing and hind wing **D** Metasoma, dorsal **E** Head and mesosoma, dorsal.

**Figure 212.**
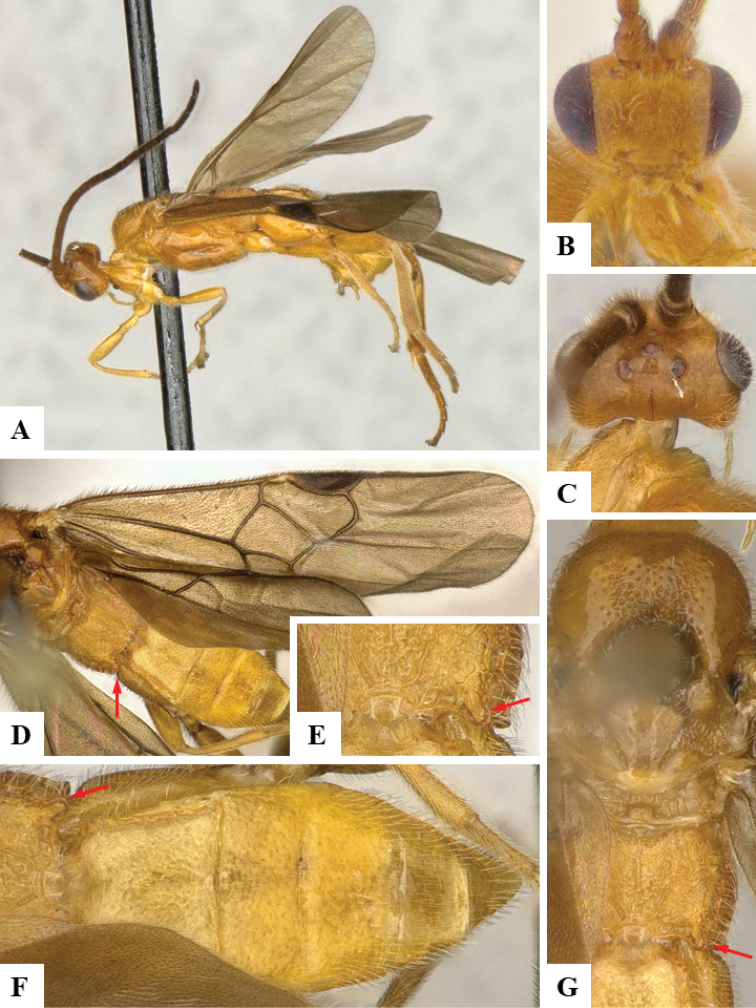
*Qrocodiledundeeoutbackense* male holotype **A** Habitus, lateral **B** Head, frontal **C** Head, dorsal **D** Fore wing **E** Propodeum, dorsal **F** Metasoma, dorsal **G** Mesosoma, dorsal. Red arrow shows the propodeal aphophysis.

**Figure 213.**
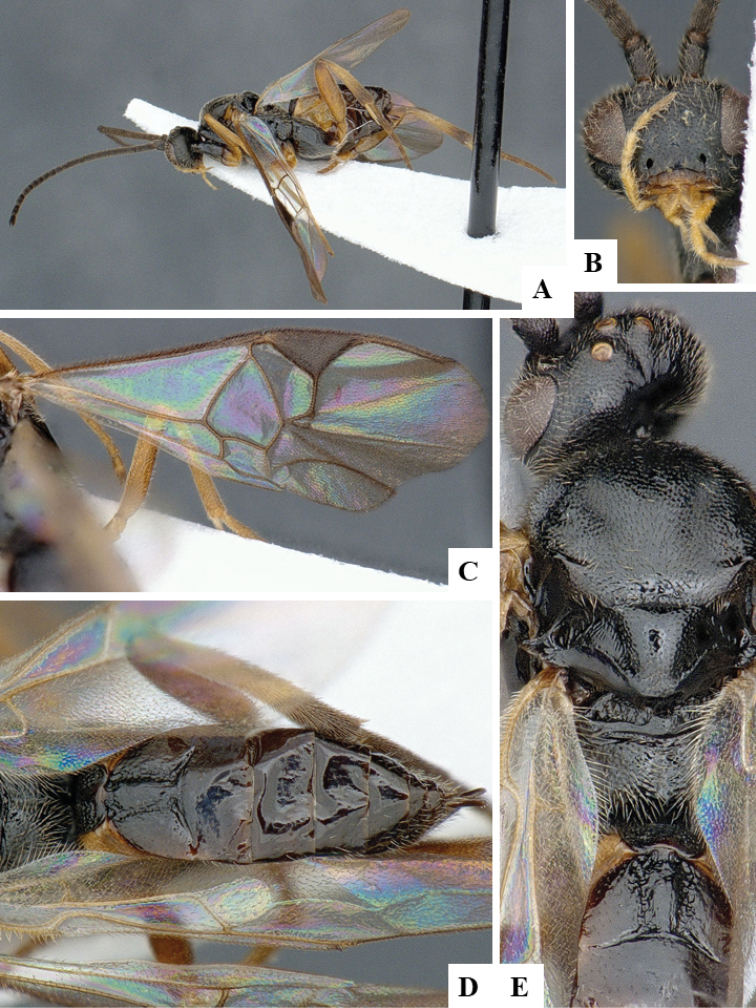
*Rasivalvacalceata* female CNC474694 **A** Habitus, lateral **B** Head, frontal **C** Fore wing **D** Metasoma, dorsal **E** Mesosoma and tergites 1–2, dorsal.

**Figure 214.**
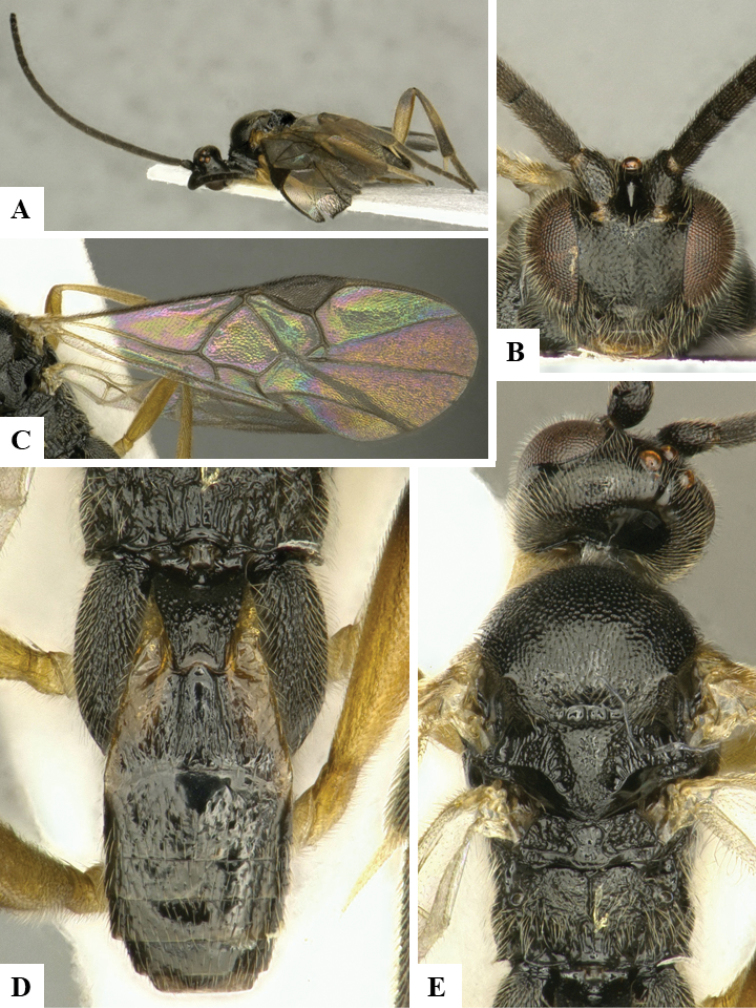
*Rasivalvamarginata* male CNC638380 **A** Habitus, lateral **B** Head, frontal **C** Fore wing **D** Metasoma, dorsal **E** Mesosoma, dorsal.

**Figure 215.**
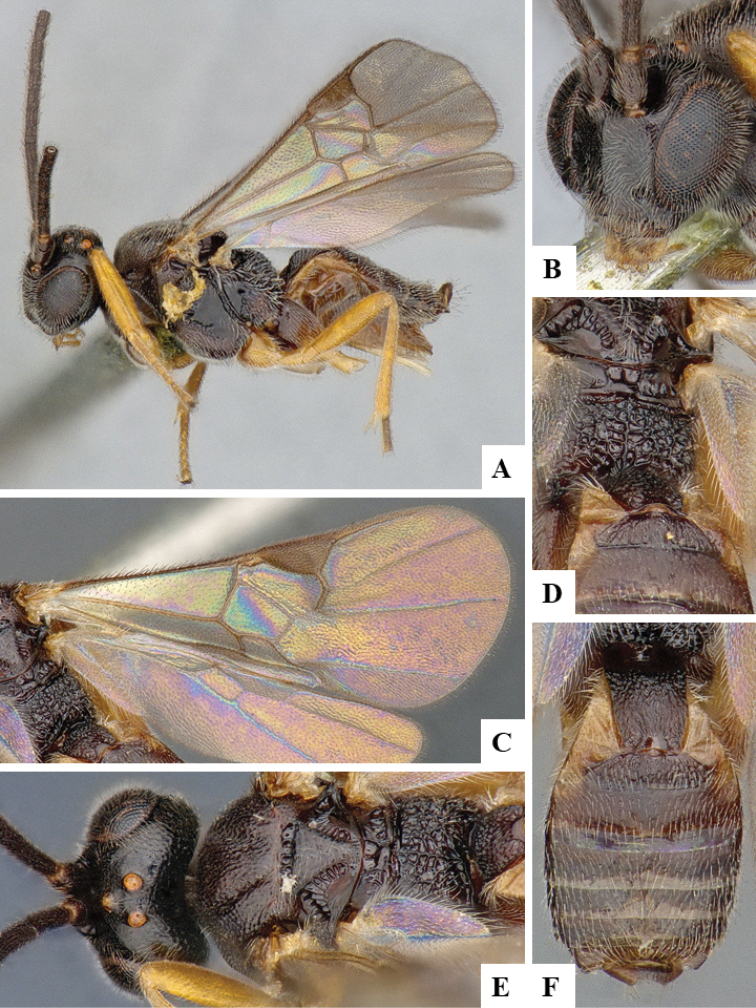
*Rhygoplitisaciculatus* female. The specimen photographed is the type of *Pseudapantelessancti-vincenti* (Ashmead, 1900), which is currently a synonym of *Rhygoplitisaciculatus* (see comments under that species in this paper, as well as details in Fernandez-Triana et al. 2014e) **A** Habitus, lateral **B** Head, frontolateral **C** Fore wing and hind wing **D** Propodeum and tergites 1–3, dorsal **E** Head and mesosoma, dorsal **F** Metasoma, dorsal.

**Figure 216.**
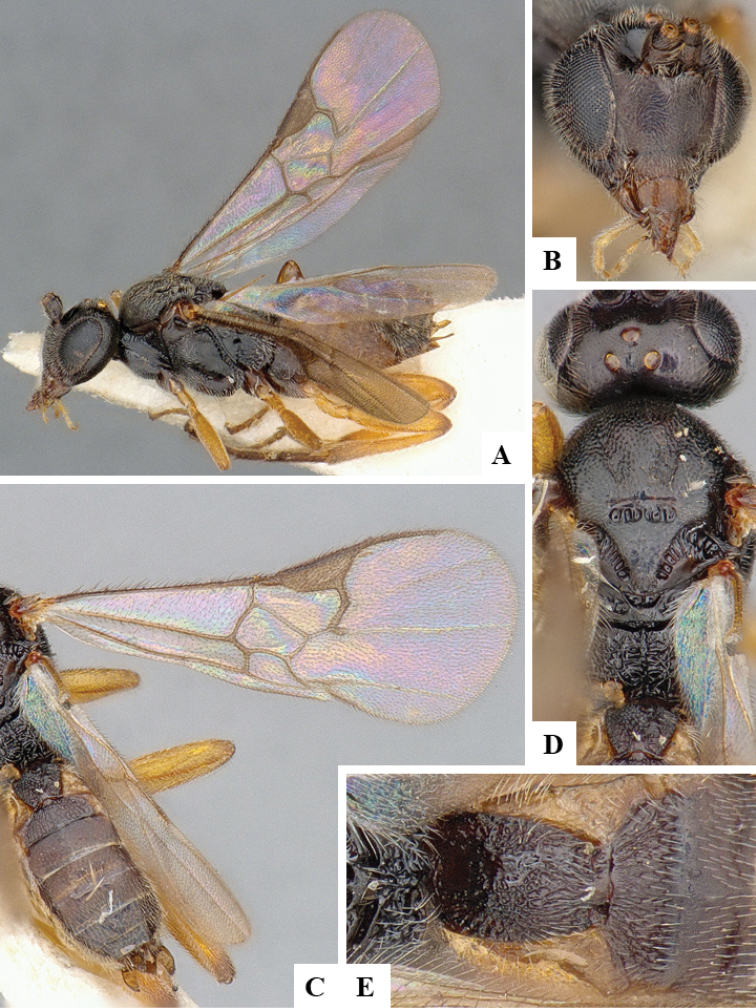
*Rhygoplitisaciculatus* male holotype **A** Habitus, lateral **B** Head, frontal **C** Fore wing **D** Head and mesosoma, dorsal **E** Tergites 1–3, dorsal.

**Figure 217.**
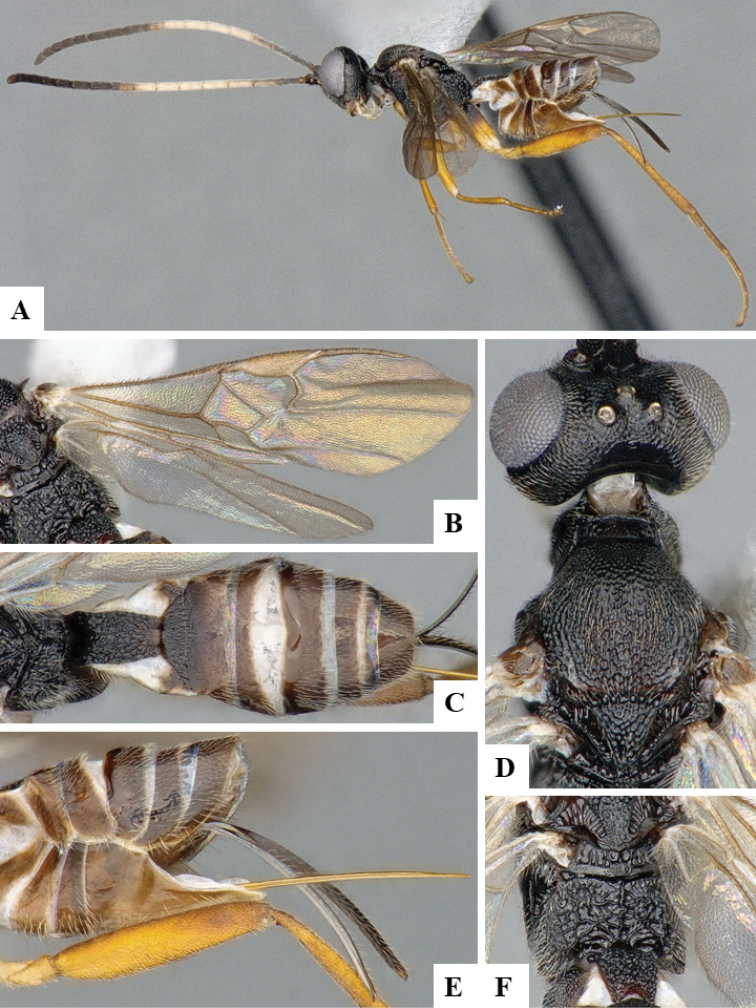
*Rhygoplitis* sp. female DHJPAR0012570 **A** Habitus, lateral **B** Fore wing and hind wing **C** Metasoma, dorsal **D** Head and mesosoma, dorsal **E** Ovipositor and ovipositor sheaths **F** Propodeum, dorsal.

**Figure 218.**
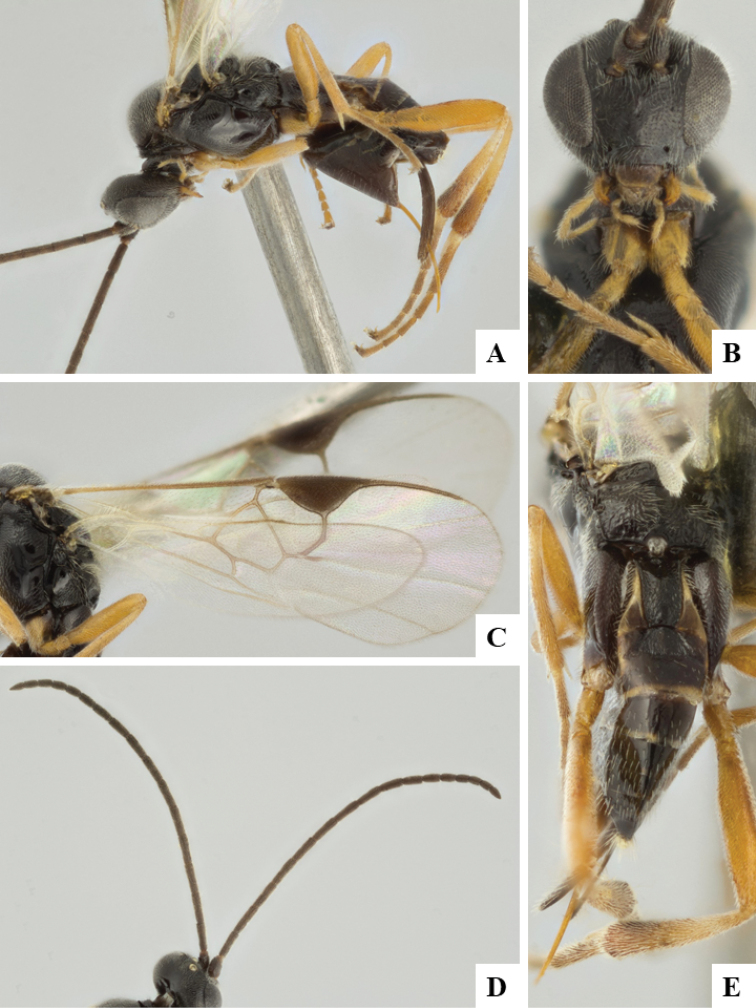
*Sathoncircumflexus* female holotype **A** Habitus, lateral **B** Head, frontal **C** Fore wing **D** Antennae **E** Propodeum and metasoma, dorsal.

**Figure 219.**
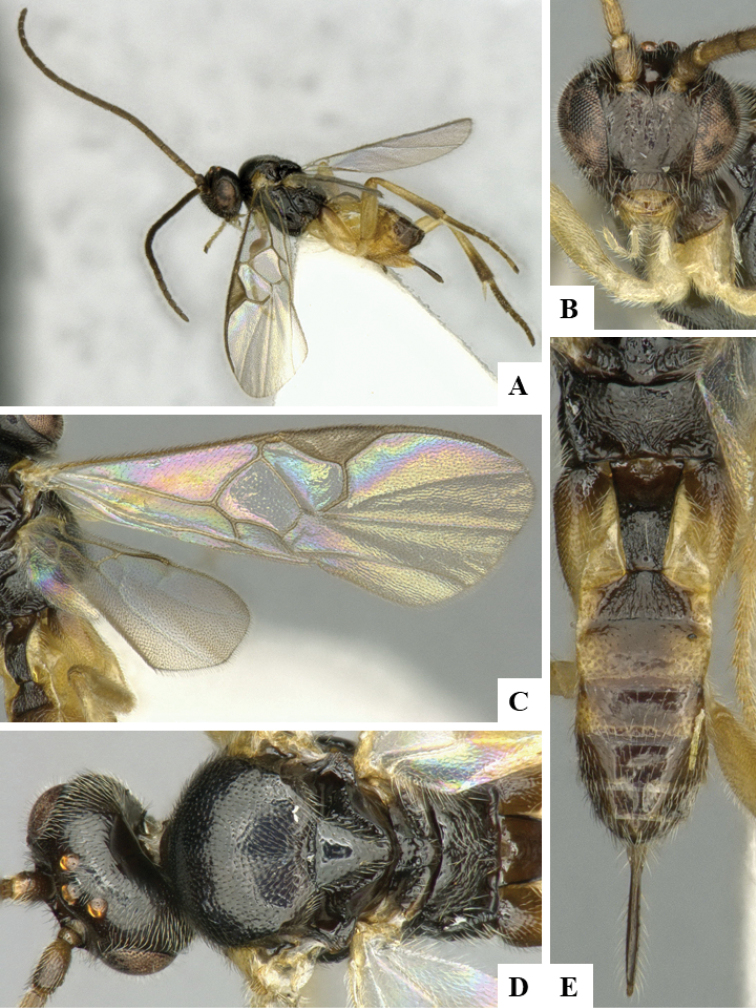
*Sathoneugeni* female CNCHYM01255 **A** Habitus, lateral **B** Head, frontal **C** Fore wing **D** Head and mesosoma, dorsal **E** Propodeum and metasoma, dorsal.

**Figure 220.**
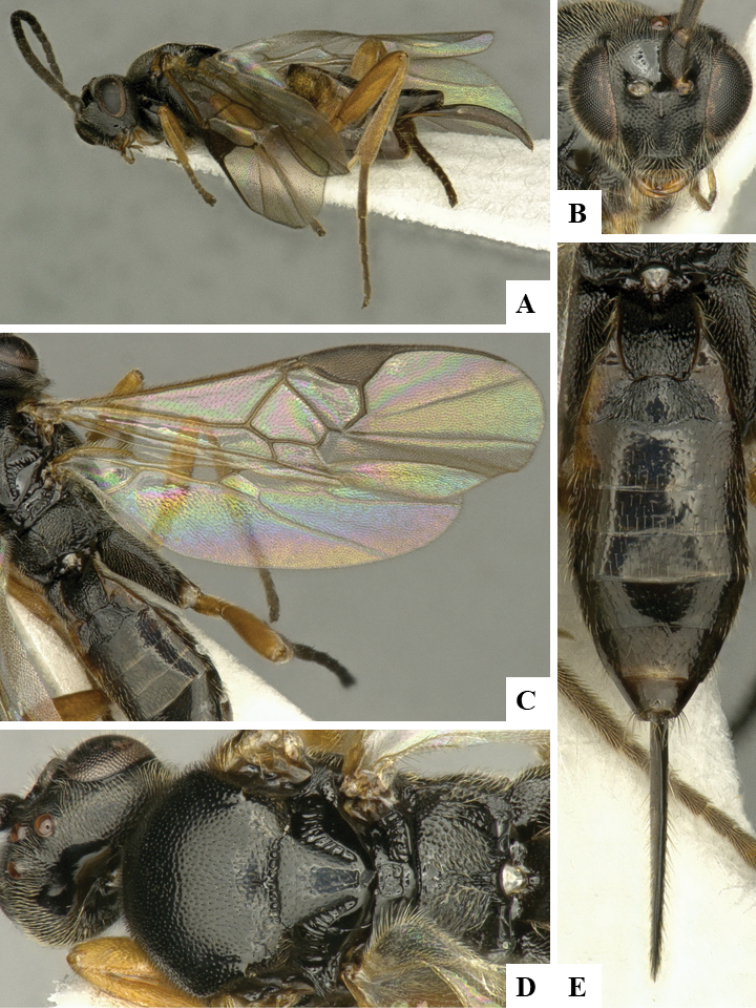
*Sathonfalcatus* female CNC638313 **A** Habitus, lateral **B** Head, frontal **C** Fore wing and hind wing **D** Mesosoma, dorsal **E** Metasoma, dorsal.

**Figure 221.**
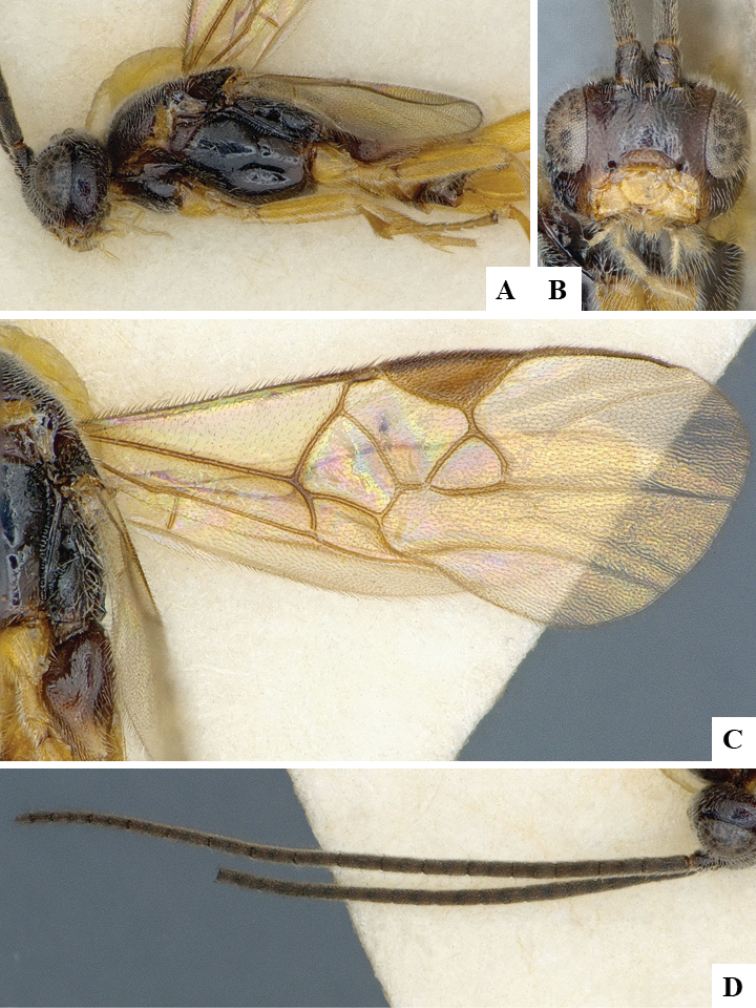
*Semionisrarus* male holotype **A** Habitus, lateral **B** Head, frontal **C** Fore wing **D** Antennae.

**Figure 222.**
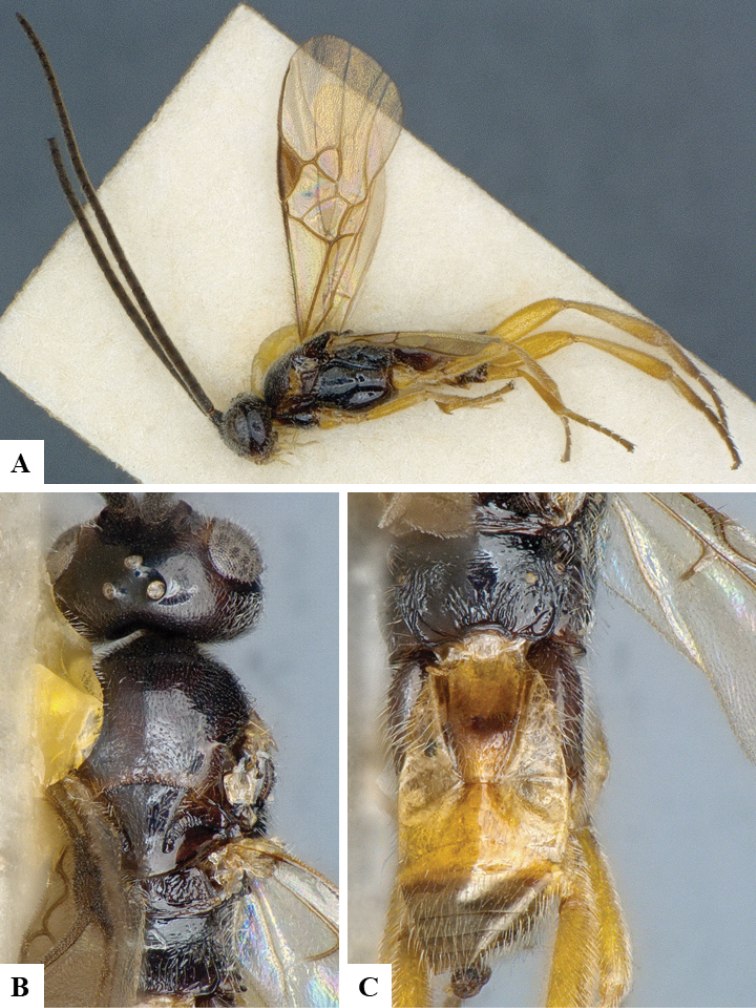
*Semionisrarus* male holotype **A** Habitus, lateral **B** Head and mesosoma, dorsal **C** Propodeum and metasoma, dorsal.

**Figure 223.**
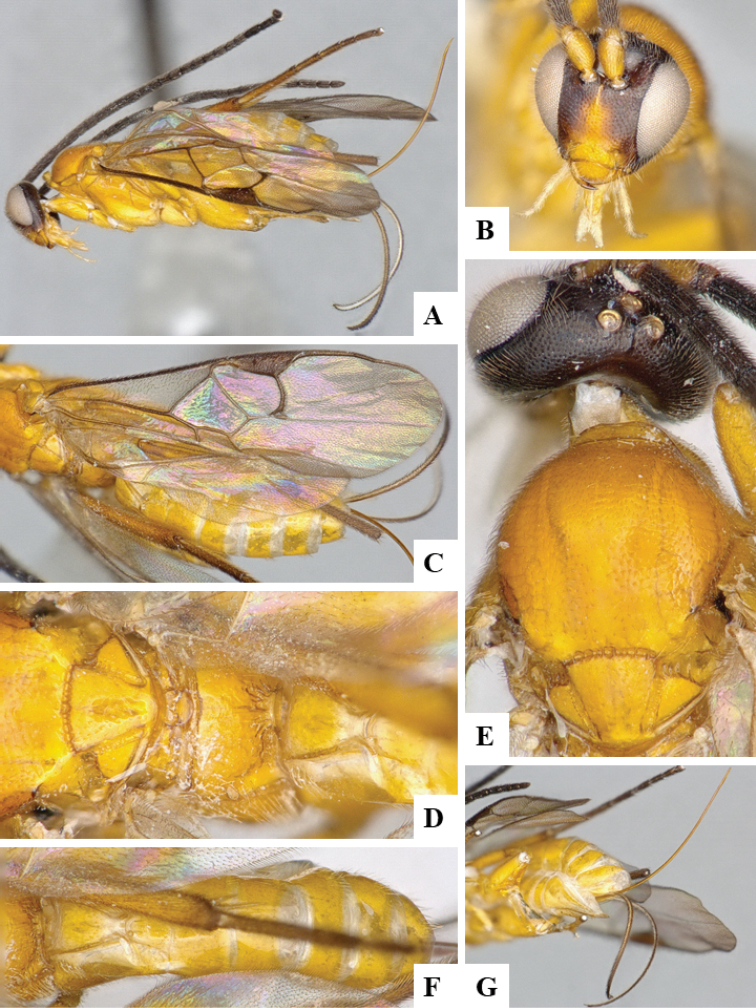
*Sendaphneanitae* female holotype **A** Habitus, lateral **B** Head, frontolateral **C** Fore wing **D** Propodeum and tergites 1–2, dorsal **E** Head and mesosoma, dorsal **F** Metasoma, dorsal **G** Ovipositor and ovipositor sheaths.

**Figure 224.**
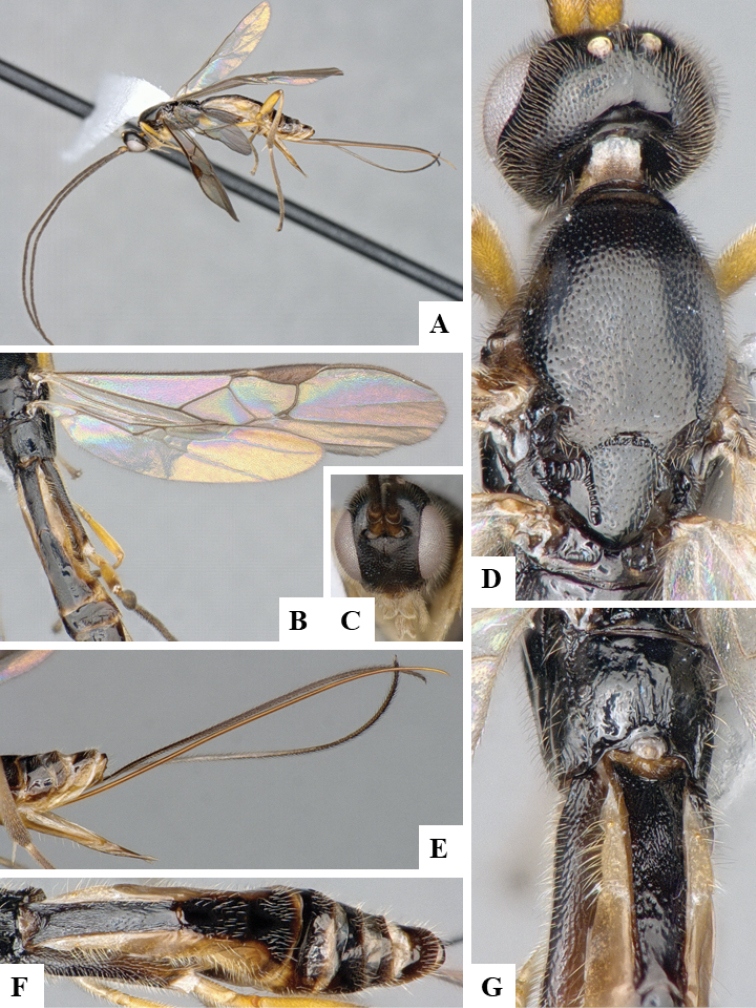
*Sendaphnerogerblancoi* female holotype **A** Habitus, lateral **B** Fore wing and hind wing **C** Head, frontal **D** Head and mesosoma, dorsal **E** Ovipositor and ovipositor sheaths **F** Metasoma, dorsal **G** Propodeum and tergite 1, dorsal.

**Figure 225.**
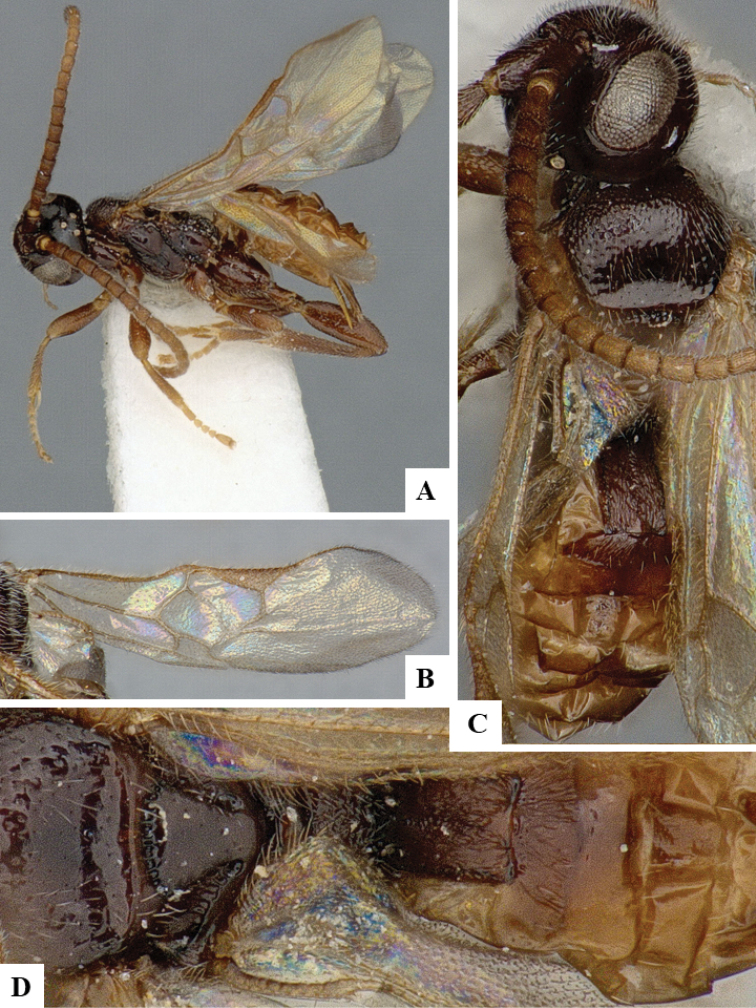
*Shireplitismeriadoci* female holotype **A** Habitus, lateral **B** Fore wing **C** Mesosoma and metasoma, dorsal **D** Scutellar disc, propodeum (partially), and mediotergites 1–5, dorsal.

**Figure 226.**
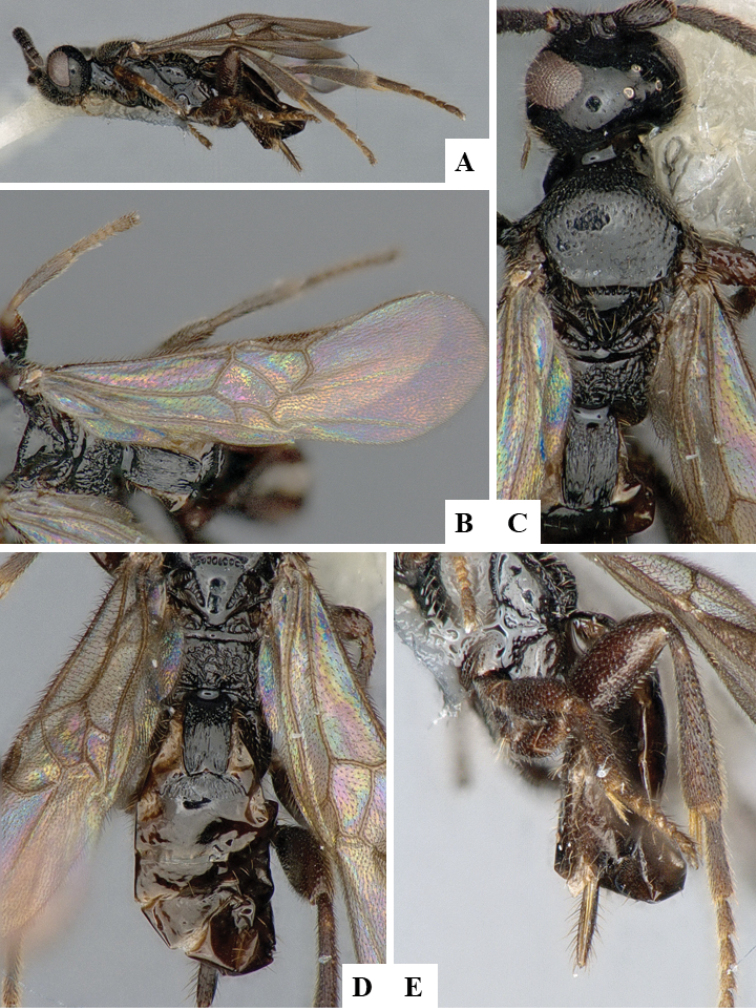
*Shireplitistolkieni* female holotype **A** Habitus, lateral **B** Fore wing **C** Head and mesosoma, dorsal **D** Propodeum and metasoma, dorsal **E** Metasoma, lateral.

**Figure 227.**
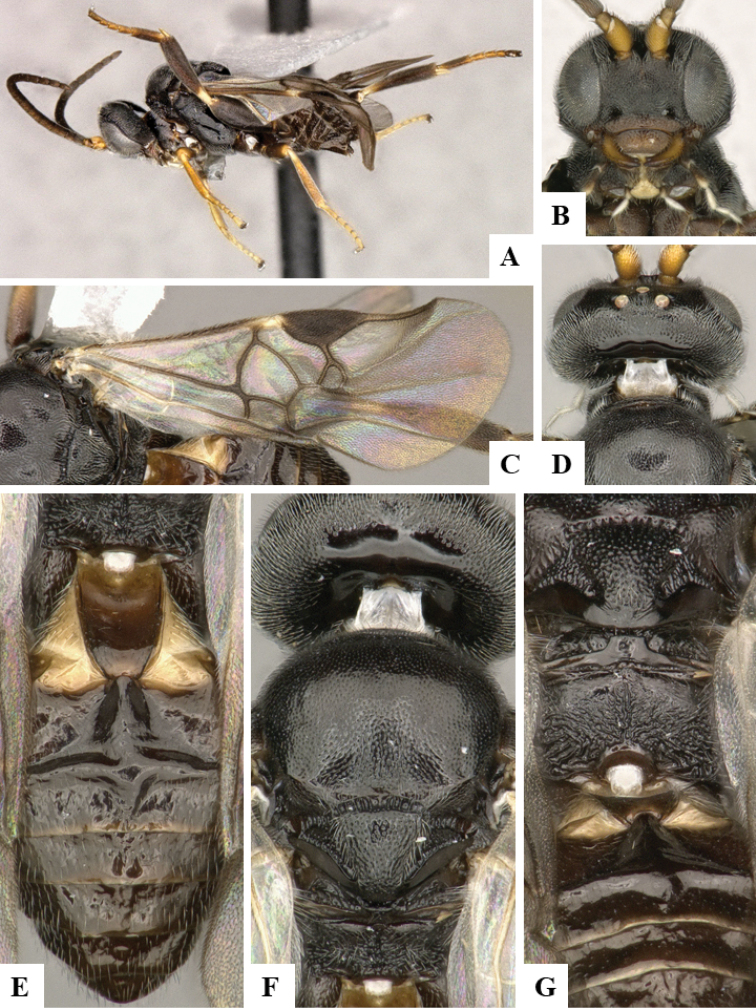
*Silvaspinosusvespa* female holotype **A** Habitus, lateral **B** Head, frontal **C** Fore wing **D** Head, dorsal **E** Metasoma, dorsal **F** Mesosoma, dorsal **G** Propodeum and tergites 1–4, dorsal.

**Figure 228.**
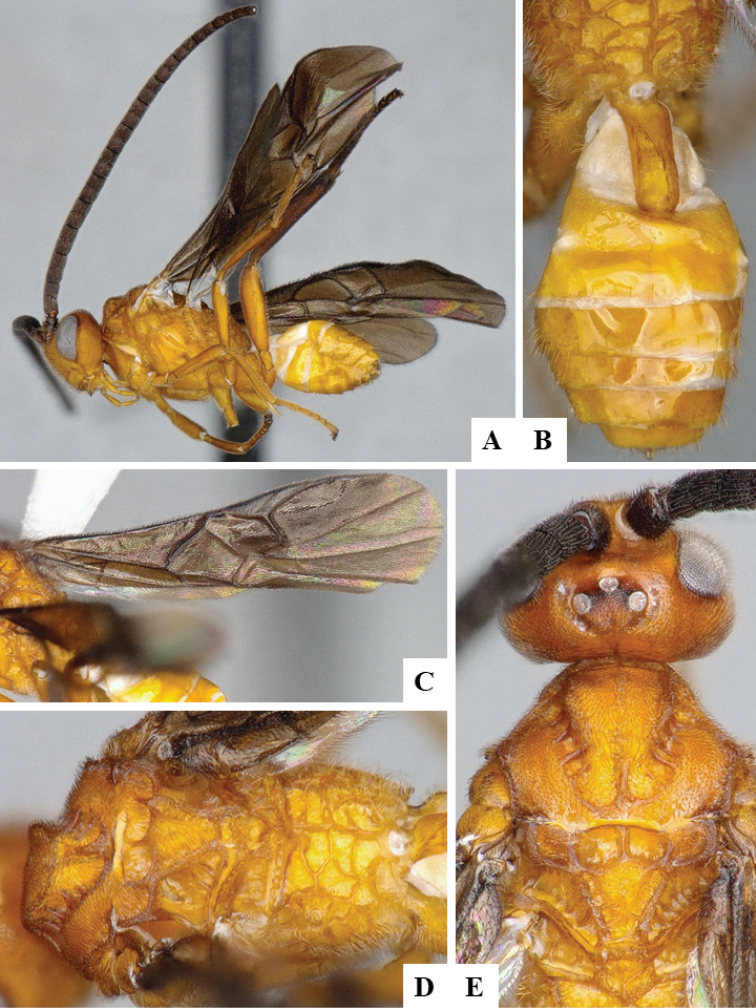
*Snelleniusisidrochaconi* male holotype **A** Habitus, lateral **B** Metasoma, dorsal **C** Fore wing **D** Mesosoma, dorsal **E** Head and mesosoma, dorsal.

**Figure 229.**
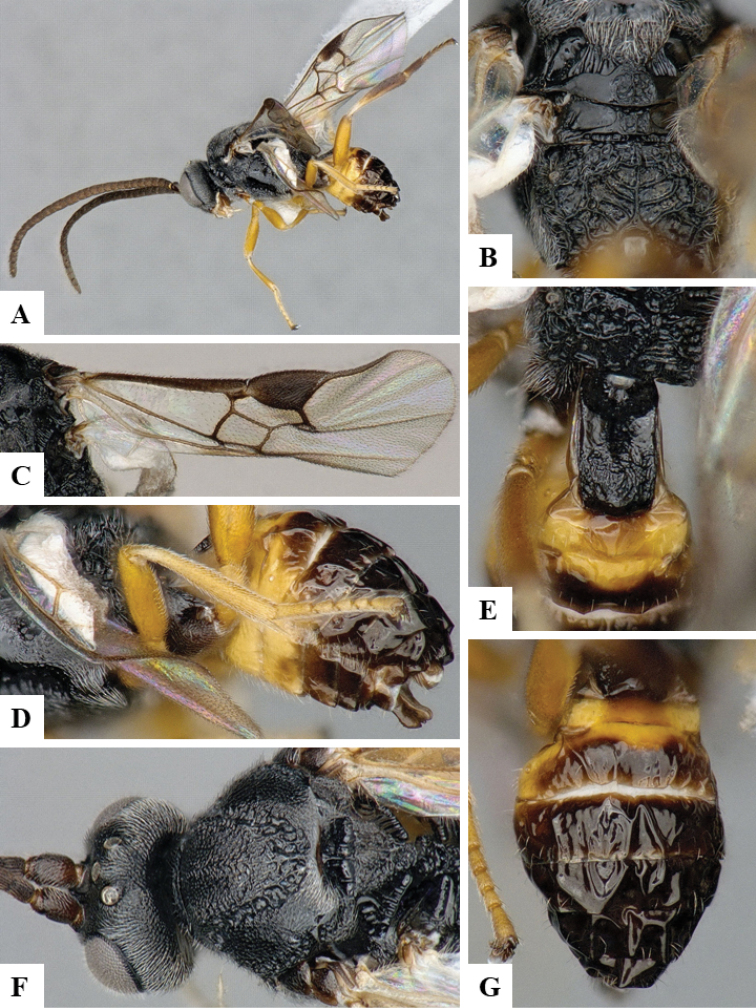
*Snelleniusmariakuzminae* male holotype **A** Habitus, lateral **B** Propodeum, dorsal **C** Fore wing **D** Metasoma, lateral **E** Tergites 1–4, dorsal **F** Head and mesosoma, dorsal **G** Metasoma, dorsal.

**Figure 230.**
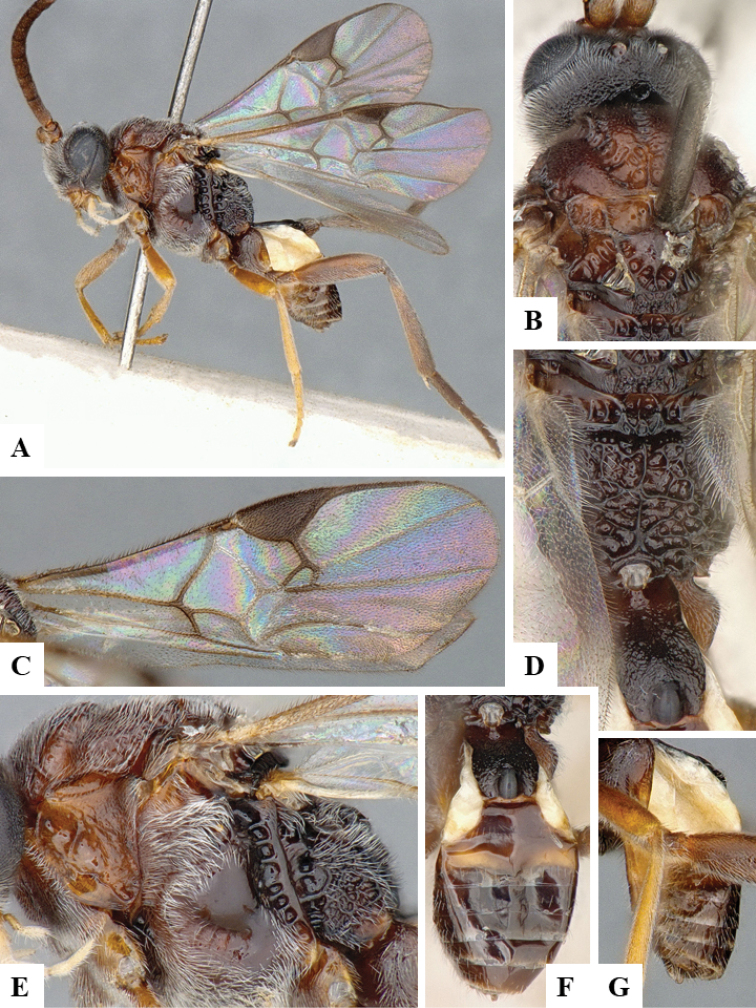
*Snelleniusrobertoespinozai* male holotype **A** Habitus, lateral **B** Mesosoma, dorsal **C** Fore wing **D** Propodeum and tergite 1, dorsal **E** Mesosoma, lateral **F** Metasoma, dorsal **G** Metasoma, lateral.

**Figure 231.**
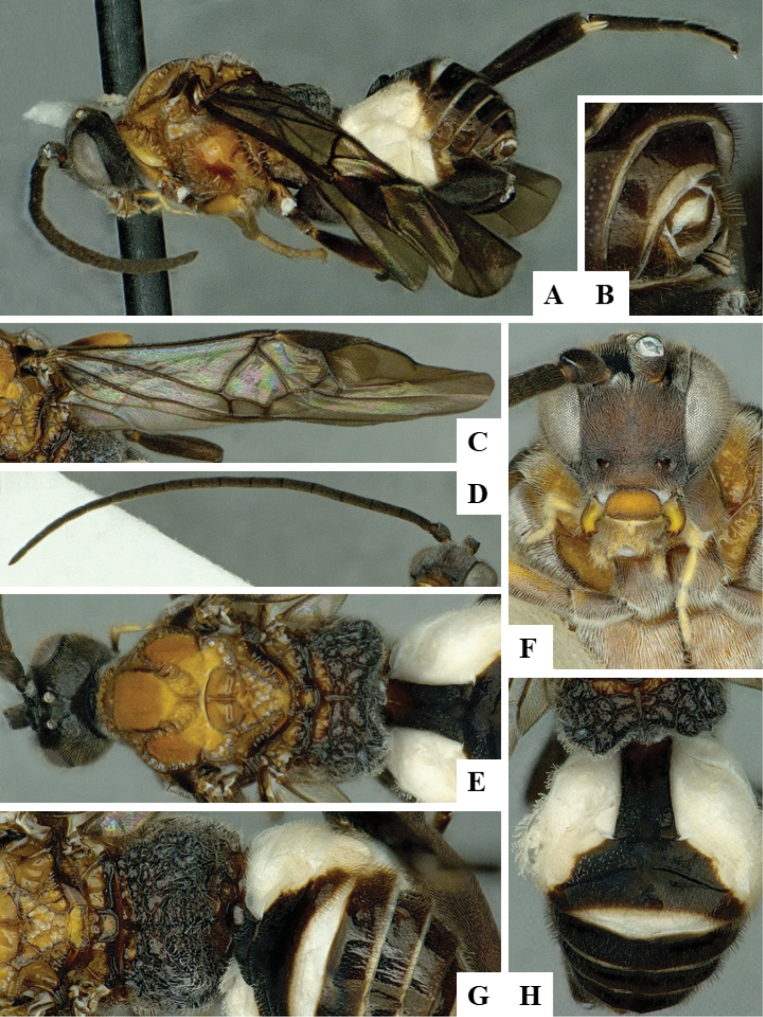
*Snellenius* sp. female CNCH1580 **A** Habitus, lateral **B** Apex of metasoma, dorsolateral **C** Fore wing **D** Antenna **E** Mesosoma, dorsal **F** Head, frontal **G** Propodeum and part of metasoma, dorsal **H** Metasoma, dorsal.

**Figure 232.**
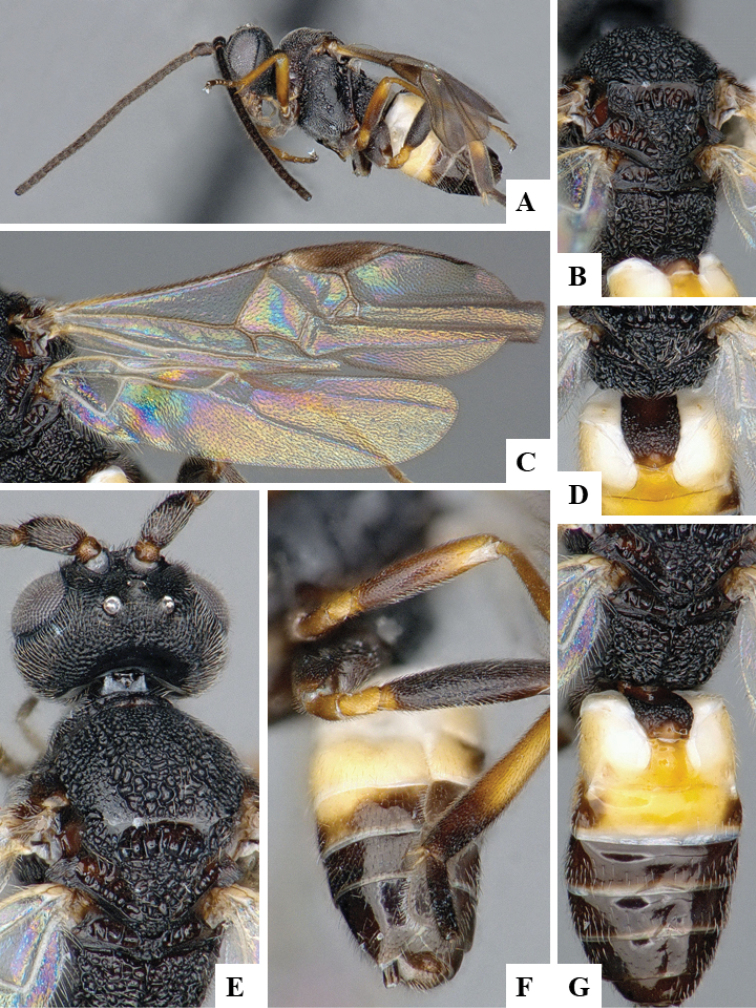
*Snelleniusvickifunkae* female holotype **A** Habitus, lateral **B** Mesosoma, dorsolateral **C** Fore wing and hind wing **D** Propodeum and tergites 1–2, dorsal **E** Head and mesosoma, dorsal **F** Metasoma, lateral **G** Propodeum and metasoma, dorsal.

**Figure 233.**
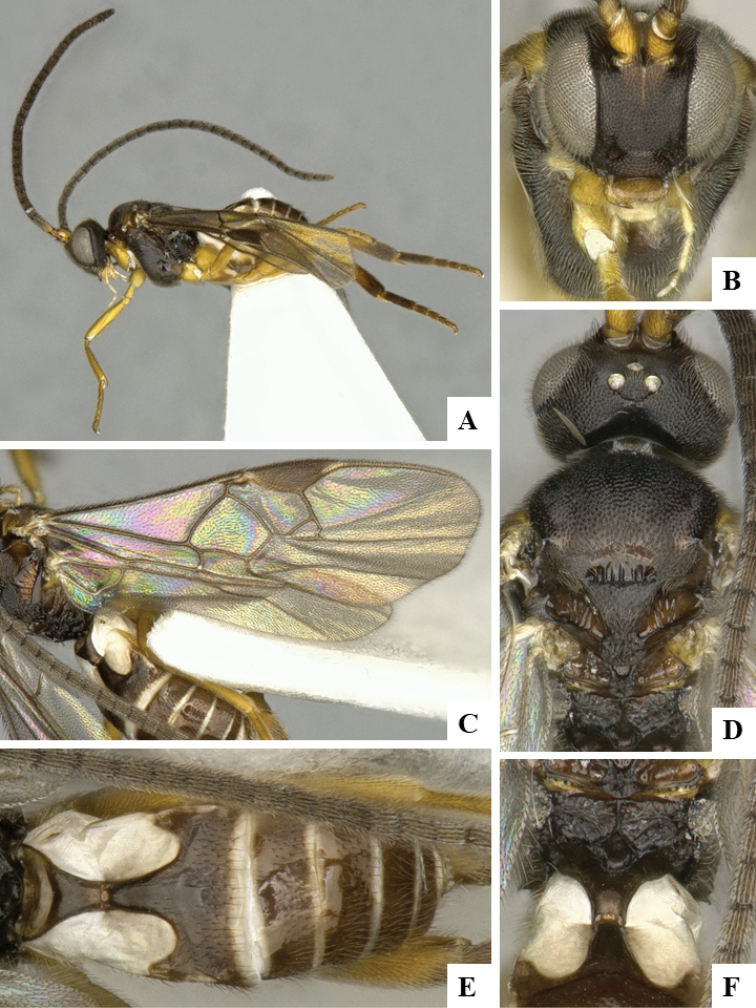
*Tobleroniusorientalis* male holotype **A** Habitus, lateral **B** Head, frontal **C** Fore wing and hind wing **D** Head and mesosoma, dorsal **E** Metasoma, dorsal **F** Propodeum and tergites 1–2, dorsal.

**Figure 234.**
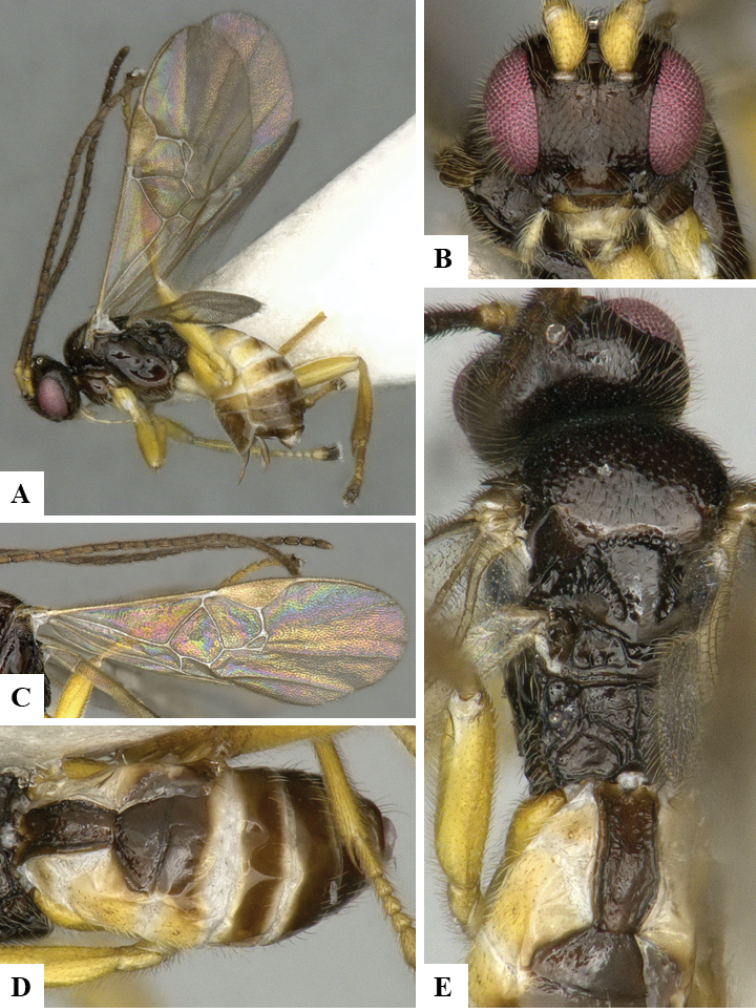
*Ungunicusvietnamensis* female holotype **A** Habitus, lateral **B** Head, frontal **C** Fore wing **D** Metasoma, dorsal **E** Head and mesosoma, dorsal.

**Figure 235.**
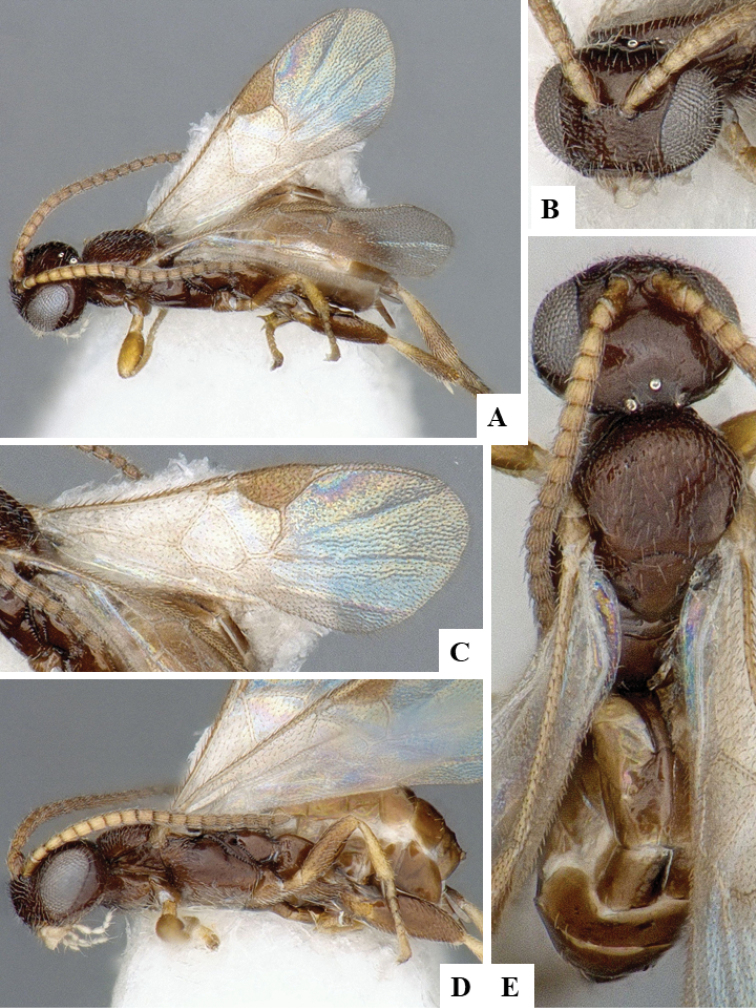
*Venanidessupracompressus* female holotype **A** Habitus, lateral **B** Head, frontal **C** Fore wing **D** Head, mesosoma and metasoma, lateral **E** Head, mesosoma and metasoma, dorsal.

**Figure 236.**
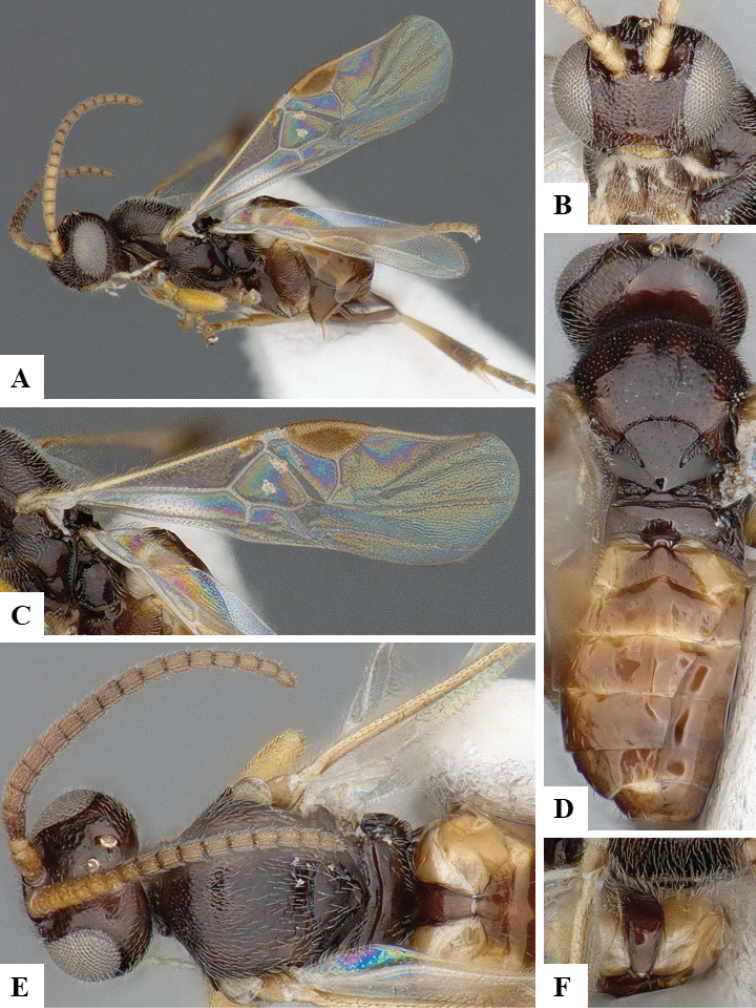
*Venanidestenuitergus* female paratype WAM 0128 **A** Habitus, lateral **B** Head, frontal **C** Fore wing **D** Head, mesosoma and metasoma, dorsal **E** Head and mesosoma, dorsal **F** Tergite 1, dorsal.

**Figure 237.**
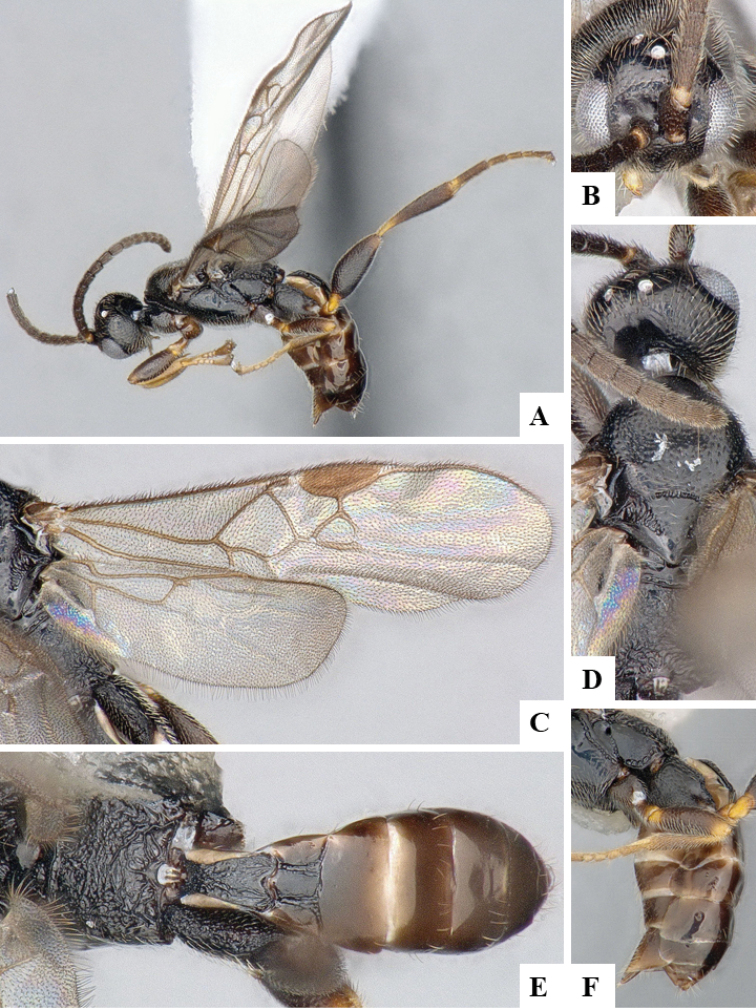
*Venanusjohnnyrosalesi* female holotype **A** Habitus, lateral **B** Head, frontal **C** Fore wing and hind wing **D** Mesosoma, dorsal **E** Propodeum and metasoma, dorsal **F** Metasoma, lateral.

**Figure 238.**
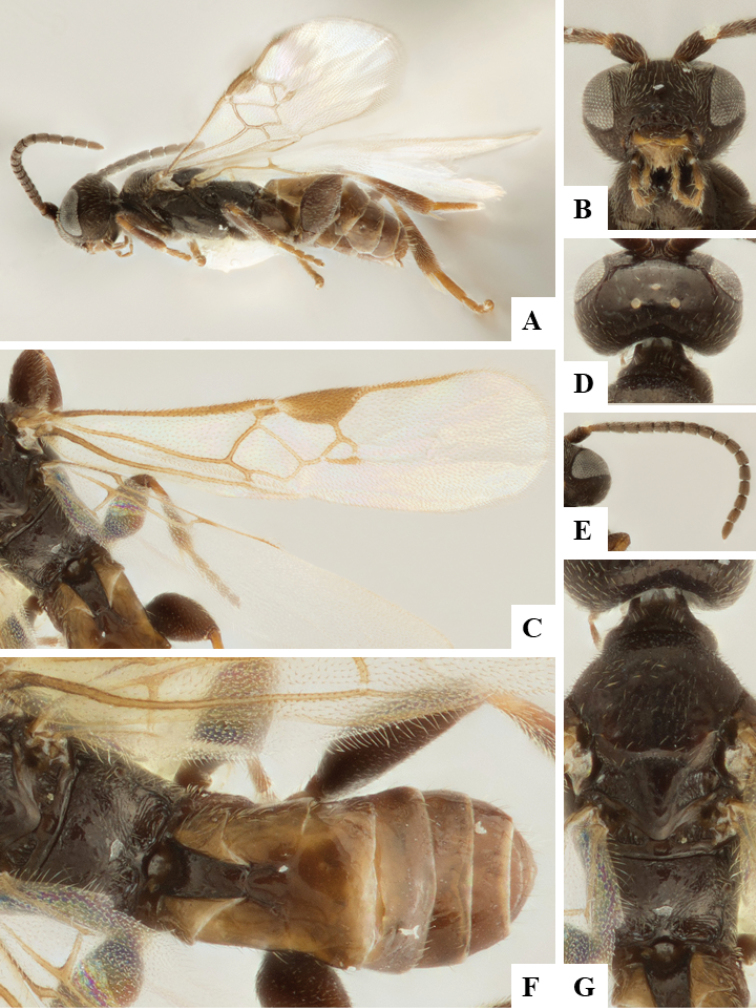
*Venanusperuensis* female holotype **A** Habitus, lateral **B** Head frontal **C** Fore wing and hind wing **D** Head dorsal **E** Antenna **F** Propodeum and metasoma, dorsal **G** Mesosoma dorsal.

**Figure 239.**
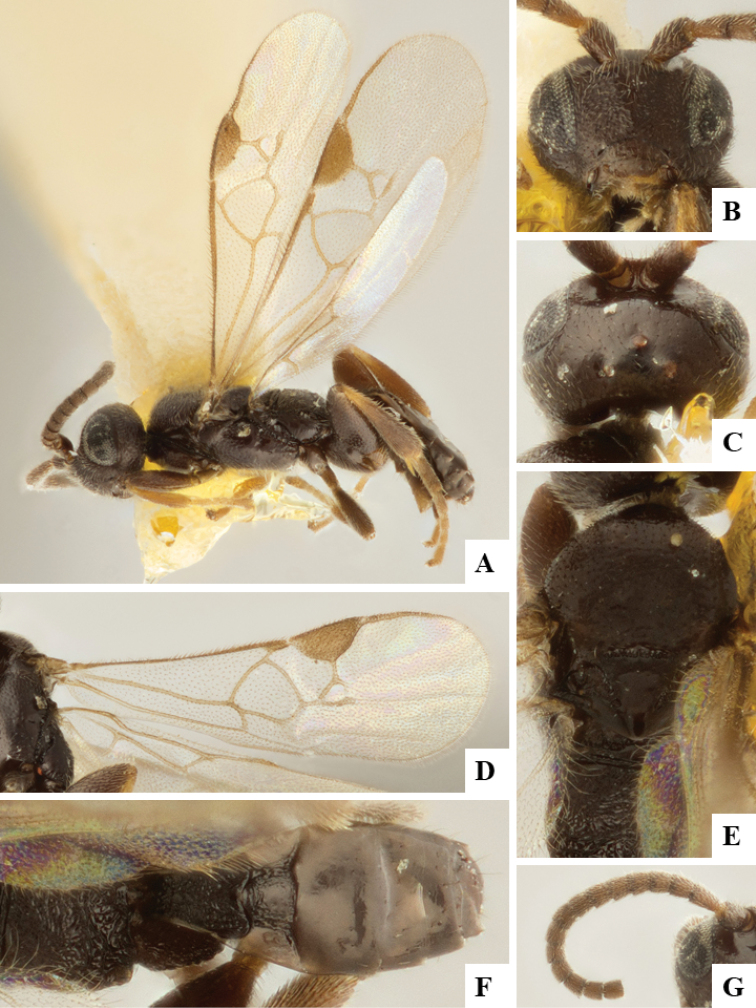
*Venanuspinicola* female holotype **A** Habitus, lateral **B** Head, frontal **C** Head, dorsal **D** Fore wing **E** Mesosoma, dorsal **F** Propodeum and metasoma, dorsal **G** Antenna.

**Figure 240.**
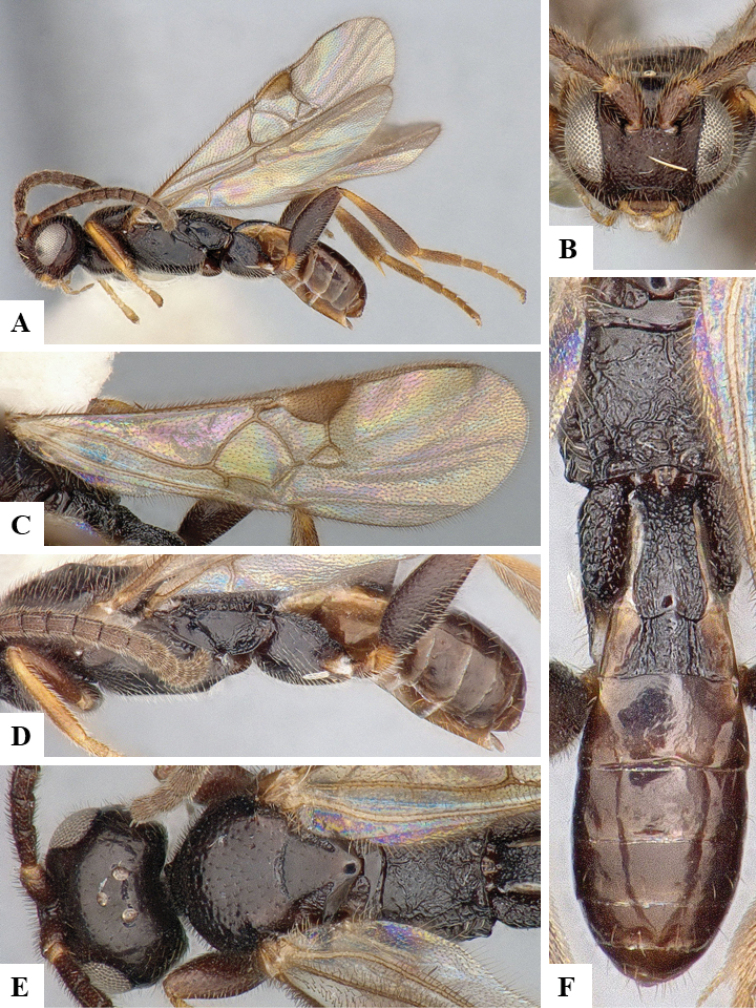
*Venanusrandallgarciai* female holotype **A** Habitus, lateral **B** Head, frontal **C** Fore wing **D** Mesosoma and metasoma, lateral **E** Head and mesosoma, dorsal **F** Propodeum and metasoma, dorsal.

**Figure 241.**
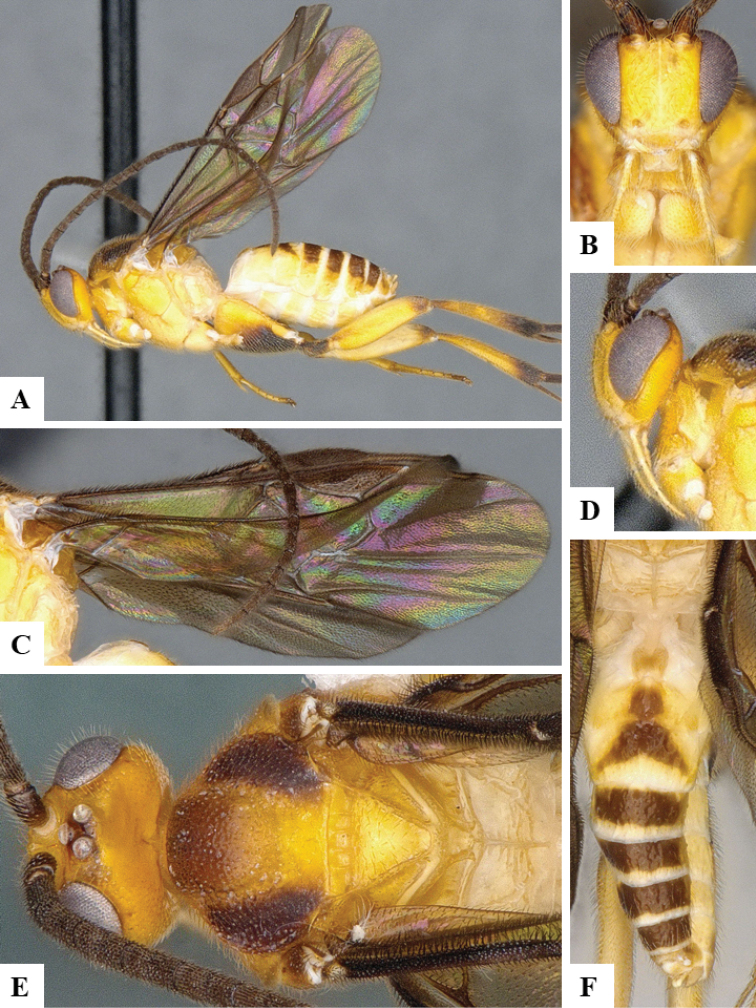
*Wilkinsonellusalexsmithi* male DHJPAR0047147 **A** Habitus, lateral **B** Head, frontal **C** Fore wing **D** Head, lateral **E** Head and mesosoma, dorsal **F** Propodeum and metasoma, dorsal.

**Figure 242.**
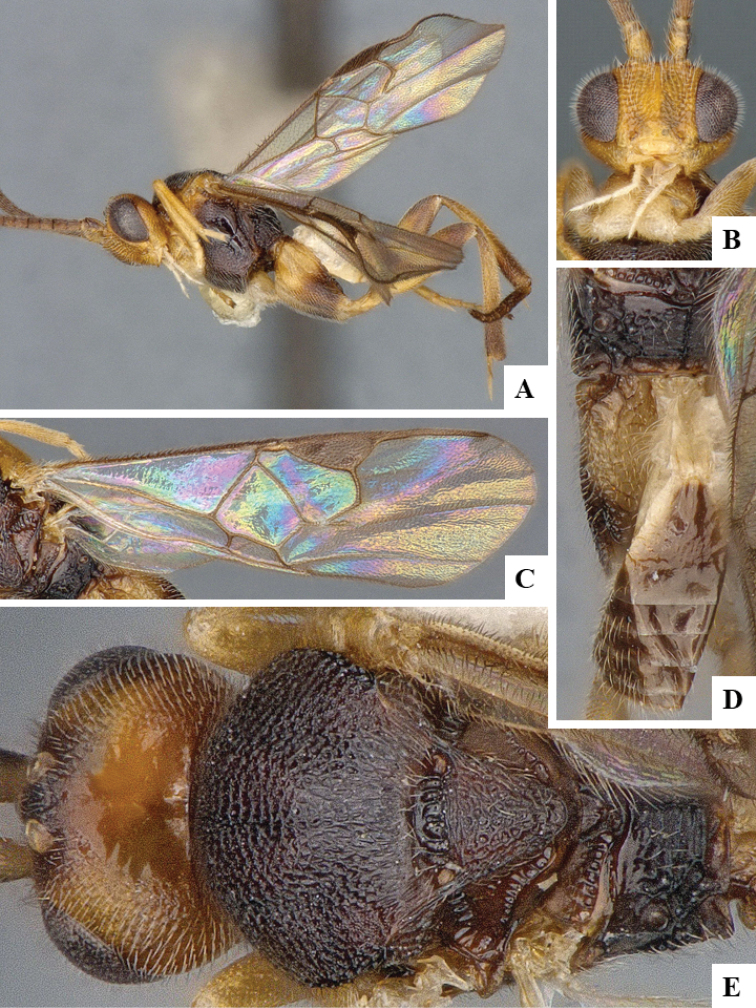
*Wilkinsonellushenicopus* male CNCHYM03452 **A** Habitus, lateral **B** Head, frontal **C** Fore wing **D** Propodeum and metasoma, dorsolateral **E** Mesosoma, dorsal.

**Figure 243.**
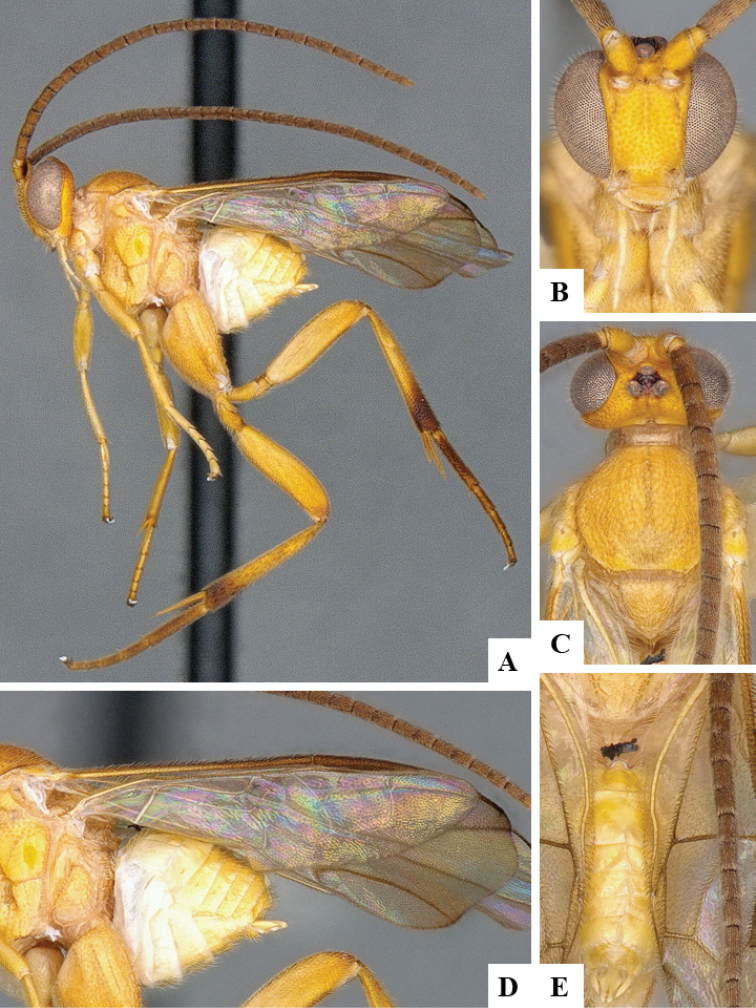
*Wilkinsonellusstriatus* male CNCH2428 **A** Habitus, lateral **B** Head, frontal **C** Head and mesosoma, dorsal **D** Fore wing and metasoma, lateral **E** Metasoma, dorsal.

**Figure 244.**
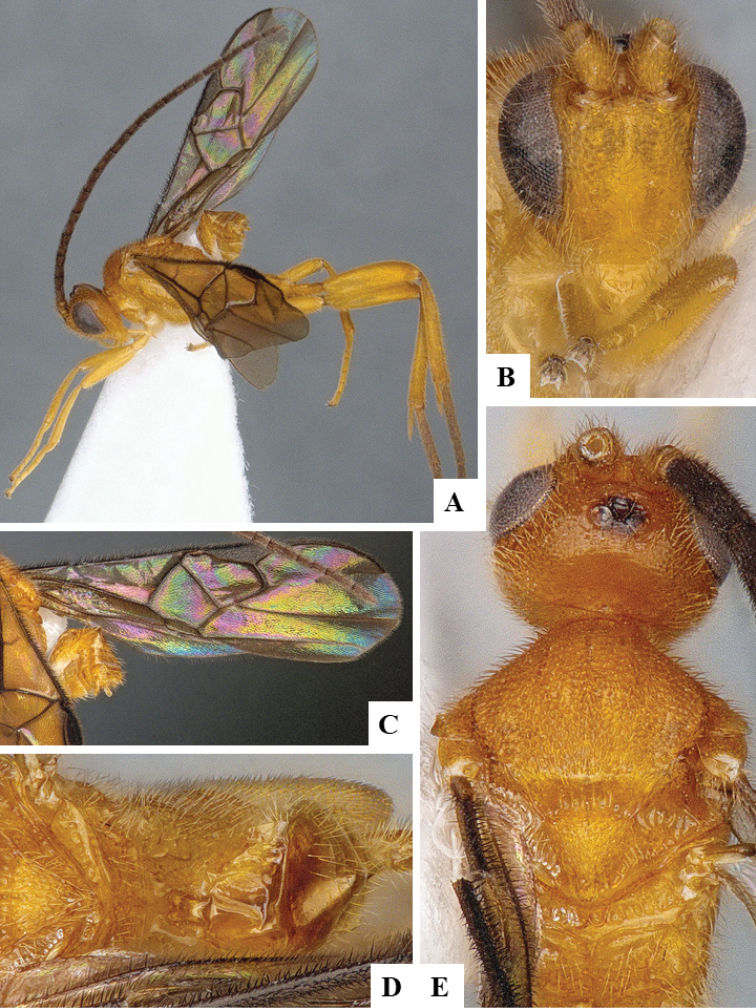
*Wilkinsonellustomi* male CNC309943 **A** Habitus, lateral **B** Head, frontal **C** Fore wing **D** Metasoma, dorsal **E** Head and mesosoma, dorsal.

**Figure 245.**
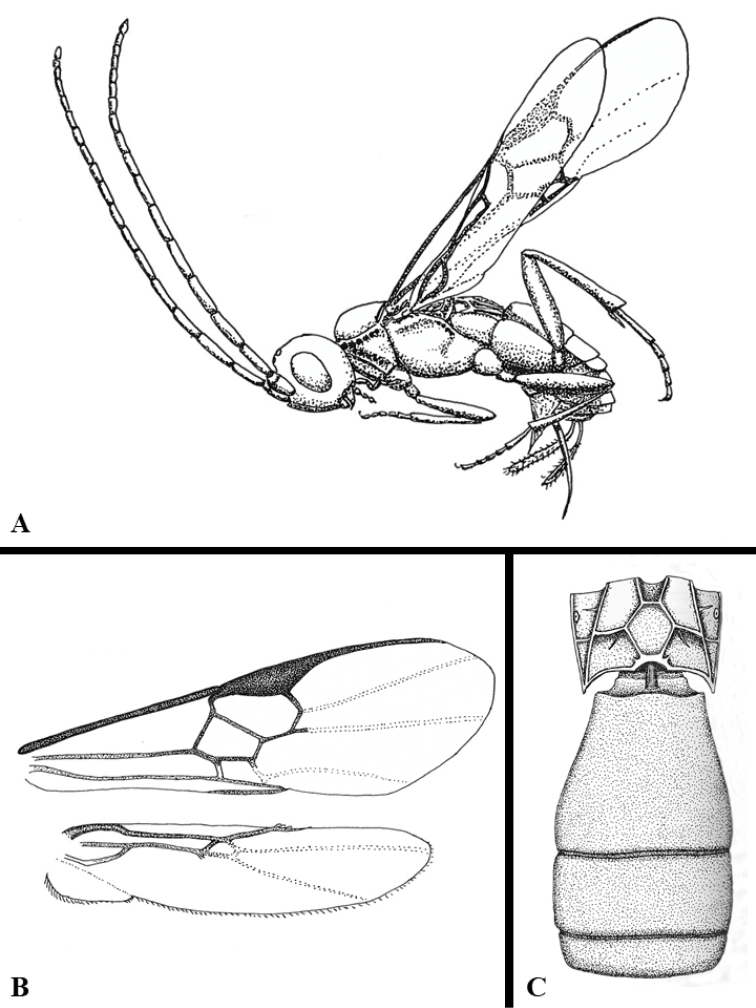
*Xanthapantelescameronae* female holotype based on modified drawings from the original descriptions of the species (Whitfield 1995) **A** Habitus, lateral **B** Fore wing and hind wing **C** Propodeum and metasoma, dorsal.

**Figure 246.**
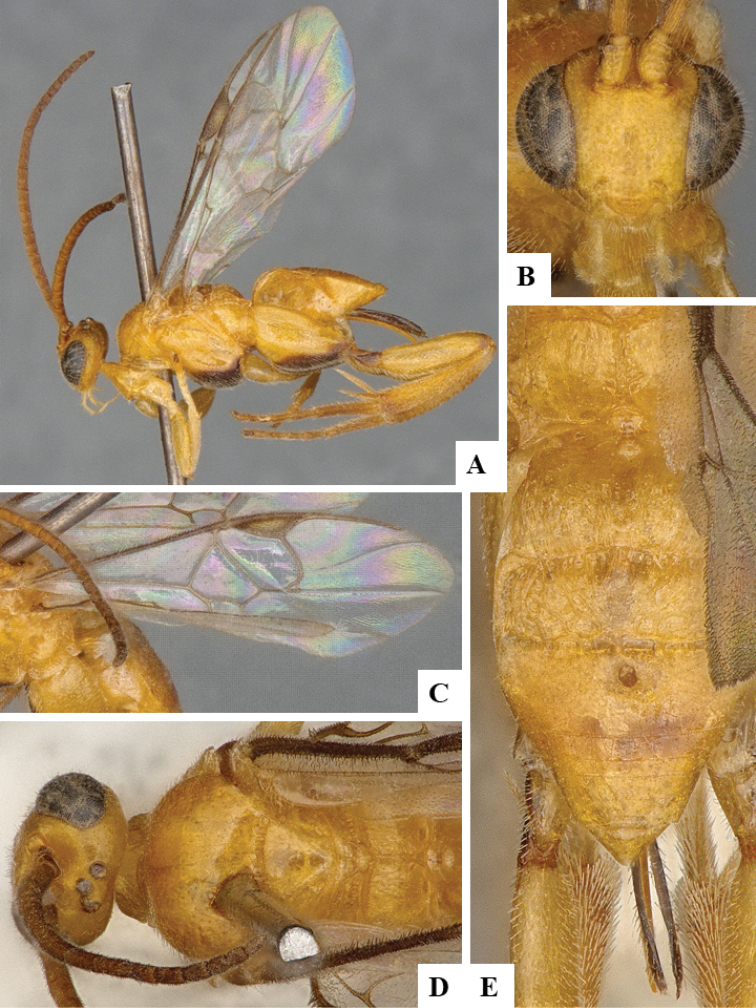
*Xanthomicrogasterfortipes* female CNCHYM07148 **A** Habitus, lateral **B** Head, frontal **C** Fore wing **D** Head and mesosoma, dorsal **E** Propodeum and metasoma, dorsal.

**Figure 247.**
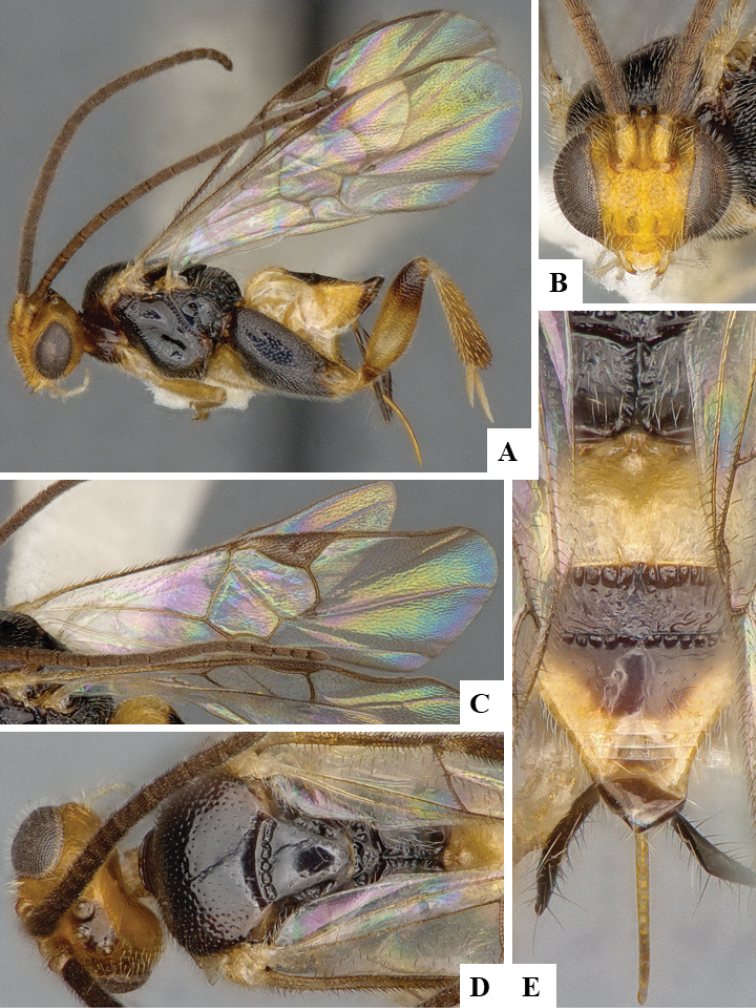
*Xanthomicrogasterpelides* female CNCHYM07146 **A** Habitus, lateral **B** Head frontal **C** Fore wing **D** Head and mesosoma, dorsal **E** Propodeum and metasoma, dorsal.

**Figure 248.**
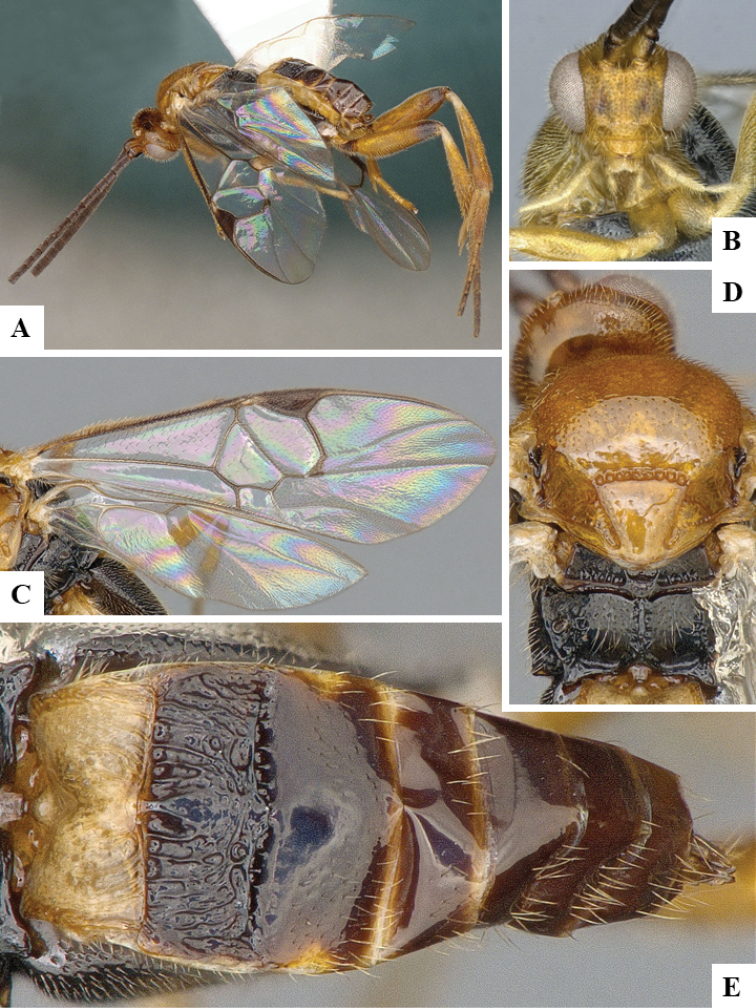
*Xanthomicrogaster* sp. male CNC492878 **A** Habitus, lateral **B** Head, frontal **C** Fore wing and hind wing **D** Mesosoma, dorsal **E** Metasoma, dorsal.

**Figure 249.**
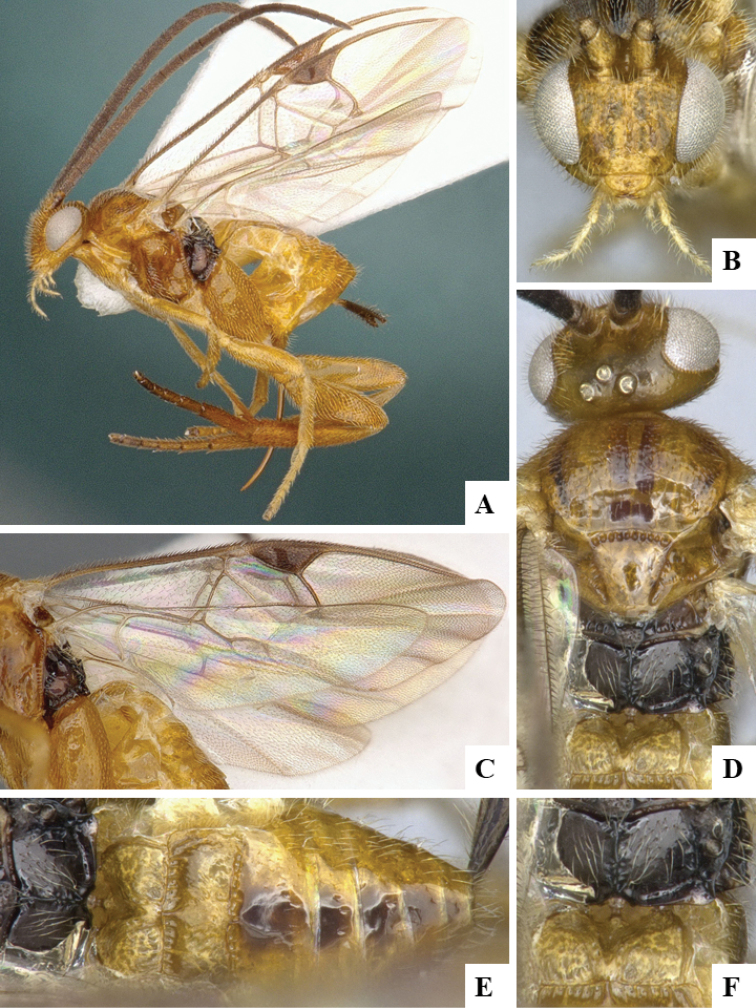
*Xanthomicrogaster* sp. female CNC492880 **A** Habitus, lateral **B** Head, frontal **C** Fore wing and hind wing **D** Head and mesosoma, dorsal **E** Propodeum and metasoma, dorsal **F** Propodeum and tergite 1, dorsal.

**Figure 250.**
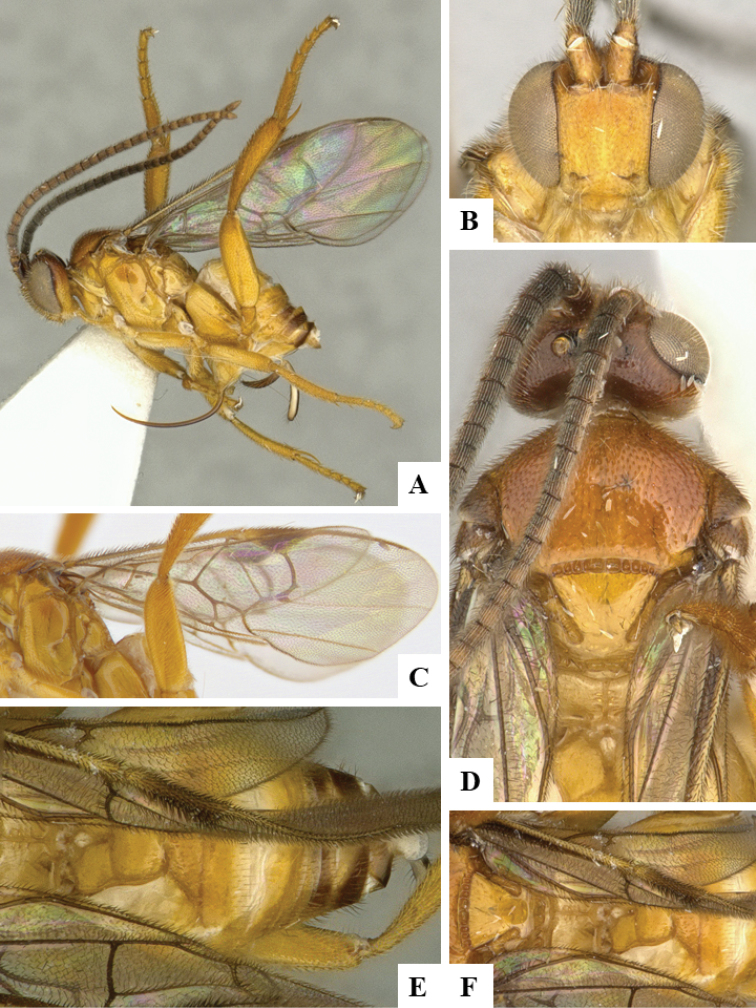
*Ypsilonigasternaturalis* female holotype **A** Habitus, lateral **B** Head, frontal **C** Fore wing **D** Head and mesosoma, dorsal **E** Metasoma, dorsal **F** Propodeum, dorsal.

**Figure 251.**
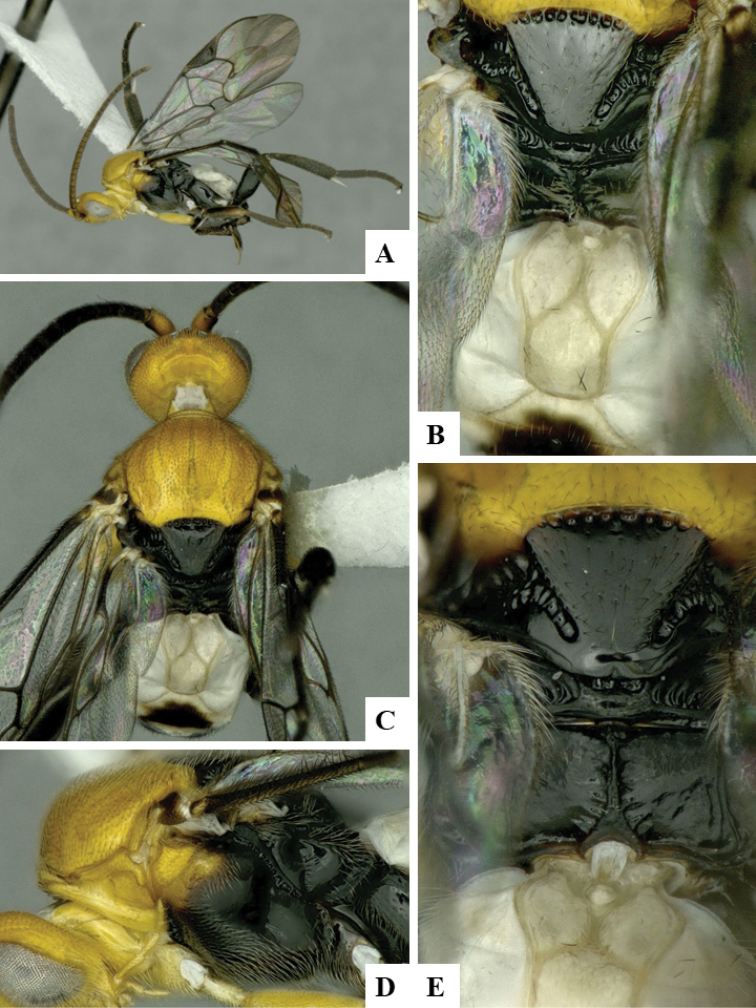
*Ypsilonigastertiger* female holotype **A** Habitus, lateral **B** Tergite 1, dorsal **C** Mesosoma, dorsal **D** Mesosoma, lateral **E** Propodeum, dorsal.

**Figure 252.**
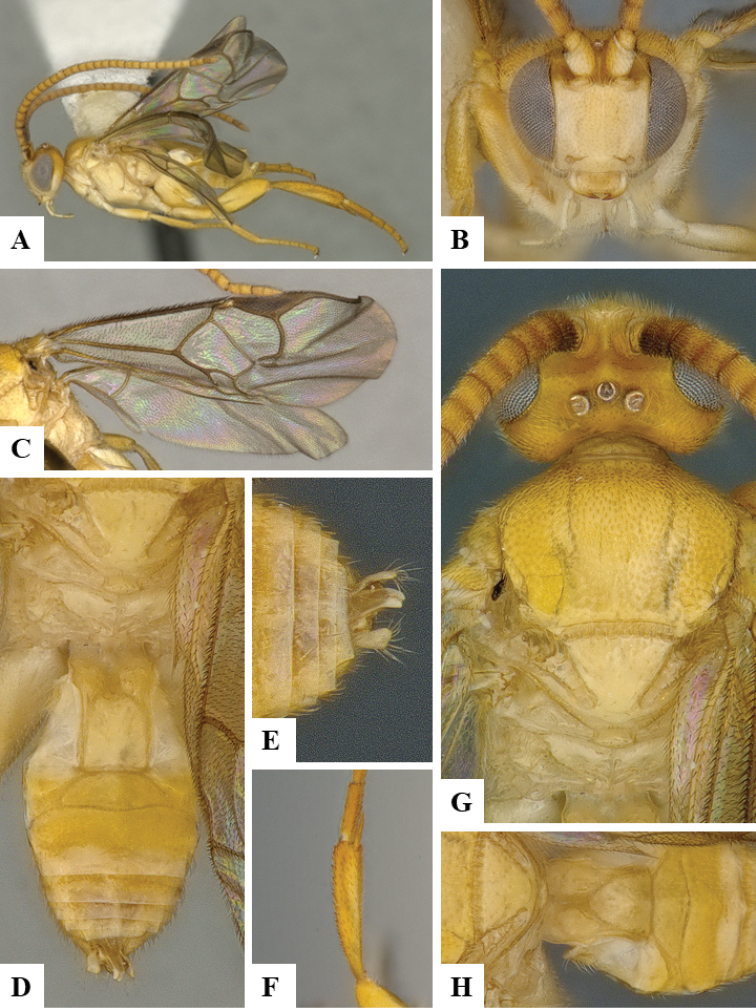
*Ypsilonigasterzuparkoi* male holotype **A** Habitus, lateral **B** Head, frontal **C** Fore wing and hind wing **D** Metasoma, dorsal **E** Genitalia **F** Metatibia, lateral **G** Head and mesosoma, dorsal **H** Tergites 1–2, dorsal.

**Figure 253.**
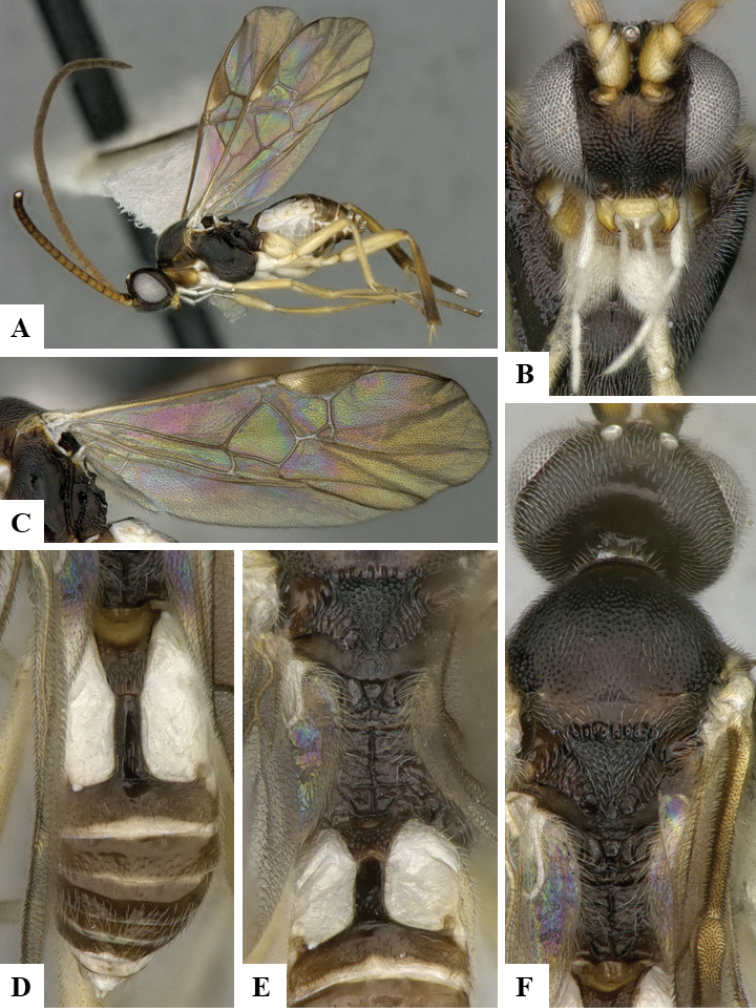
*Zachterbergiustenuitergum* male holotype **A** Habitus, lateral **B** Head, frontal **C** Fore wing **D** Metasoma, dorsal **E** Propodeum **F** Head and mesosoma, dorsal.

**Figure 254.**
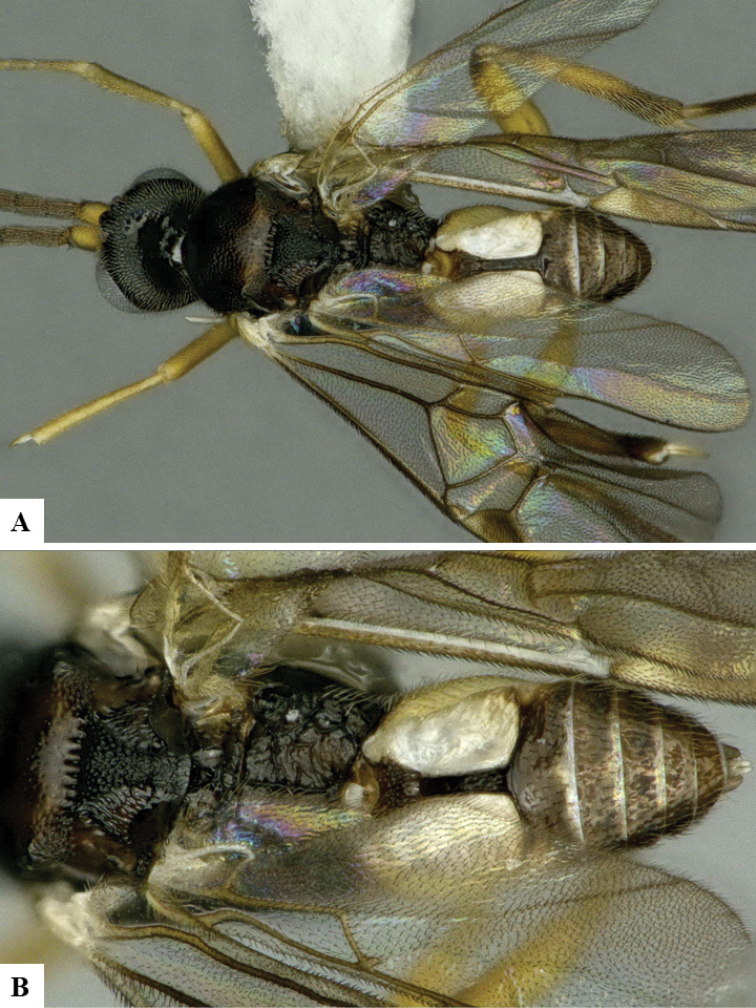
*Zachterbergiustenuitergum* male paratype JMIC 0538 **A** Habitus, dorsal **B** Mesosoma and metasoma, dorsal.

## Supplementary materials

Supplementary material 1 Supplementary table 1 Data type: species data https://binary.pensoft.net/file/392173This dataset is made available under the Open Database License (http://opendatacommons.org/licenses/odbl/1.0/). The Open Database License (ODbL) is a license agreement intended to allow users to freely share, modify, and use this Dataset while maintaining this same freedom for others, provided that the original source and author(s) are credited.Jose Fernandez-Triana, Mark R. Shaw, Caroline Boudreault, Melanie Beaudin, Gavin R. Broad

Supplementary material 2 Supplementary table 2 Data type: species data https://binary.pensoft.net/file/392174This dataset is made available under the Open Database License (http://opendatacommons.org/licenses/odbl/1.0/). The Open Database License (ODbL) is a license agreement intended to allow users to freely share, modify, and use this Dataset while maintaining this same freedom for others, provided that the original source and author(s) are credited.Jose Fernandez-Triana, Mark R. Shaw, Caroline Boudreault, Melanie Beaudin, Gavin R. Broad
